# Supplementary material for: Bioinformatic Identification and Expression Analyses of the MAPK–MAP4K Gene Family Reveal a Putative Functional MAP4K10-MAP3K7/8-MAP2K1/11-MAPK3/6 Cascade in Wheat (Triticum aestivum L.)
Source: Plants (Basel). 2024 Mar 24;13(7):941. doi: 10.3390/plants13070941 (PMC11013086; doi:10.3390/plants13070941)
Supplement: Supplementary file 1 [file plants-13-00941-s001.zip › plants-2867660-supplementary/Supplementary Figure S1-S10 and Supplementary table S1-S7/Supplementary Figure S1-S10/Supplemental Figure S1-S10.pdf]

Bioinformatic Identification and Expression Analyses of the MAPK–MAP4K Gene Family Reveal a Putative Functional MAP4K10-MAP3K7/8-MAP2K1/11-MAPK3/6 Cascade in Wheat (*Triticum aestivum* L.)

**Yongliang Li[1,2 †], You Li[1 †], Xiaoxiao Zou[1 †], Shuai Jiang[1], Miyuan Cao[1], Fenglin Chen[1], Yan Yin[1], Wenjun Xiao[1,2 *],
Shucan Liu[1,2 *] and Xinhong Guo[1,2 *]**

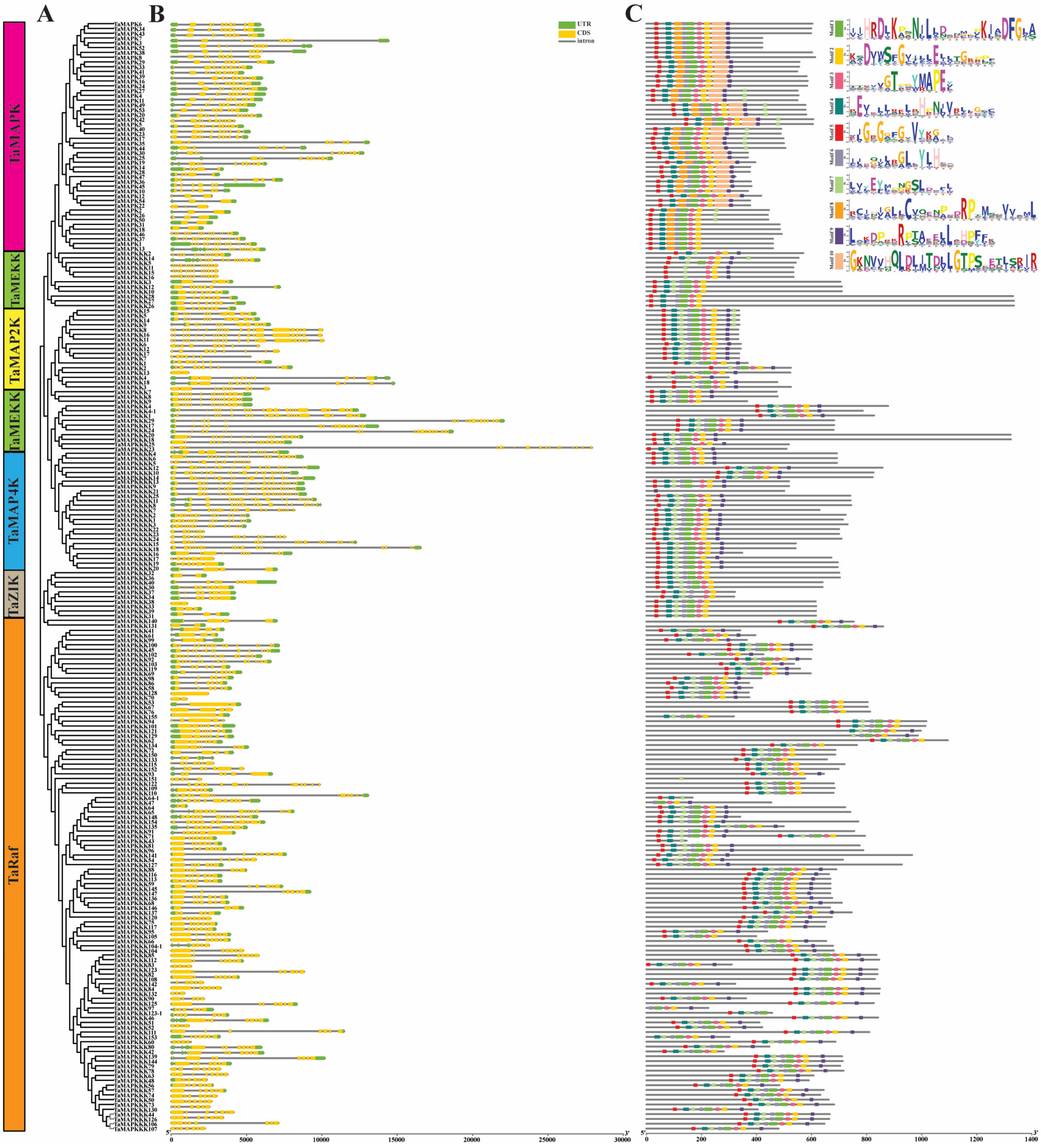

Supplemental Figure S1. The gene structures and protein structures of *TaMAPK-TaMAP4K* cascade genes in wheat. (A) Phylogenetic relationships; (B) protein structures; (C) gene structures.

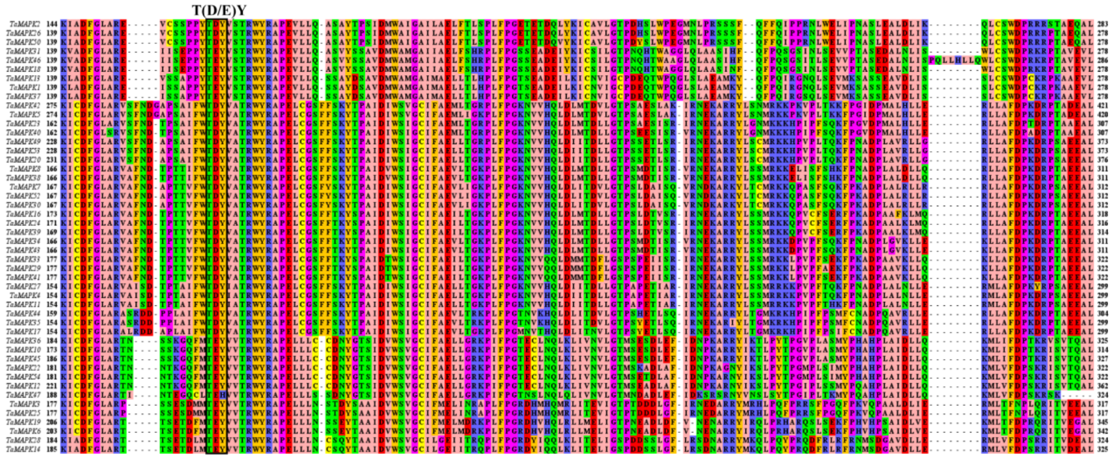

Supplemental Figure S2. Alignment of TaMAPK family. The highlighted part shows the conserved motif.

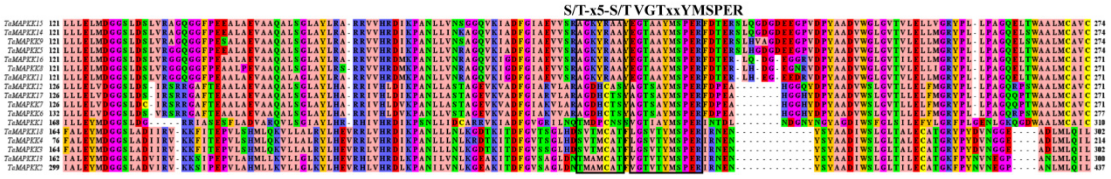

Supplemental Figure S3. Alignment of MAP2K family from wheat. The highlighted part shows the conserved motif.

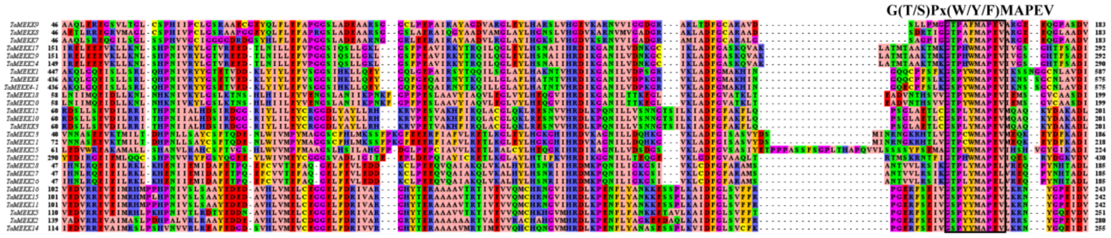

Supplemental Figure S4. Alignment of MEKK substype from wheat MAP3K. The highlighted part shows the conserved motif.

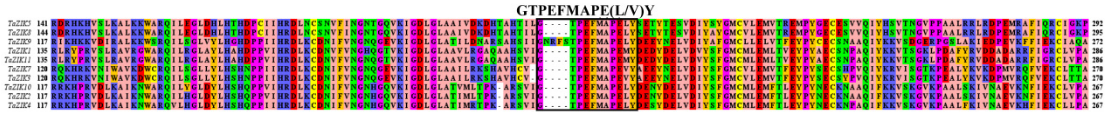

Supplemental Figure S5. Alignment of ZIK substype from wheat MAP3K. The highlighted part shows the conserved motif.

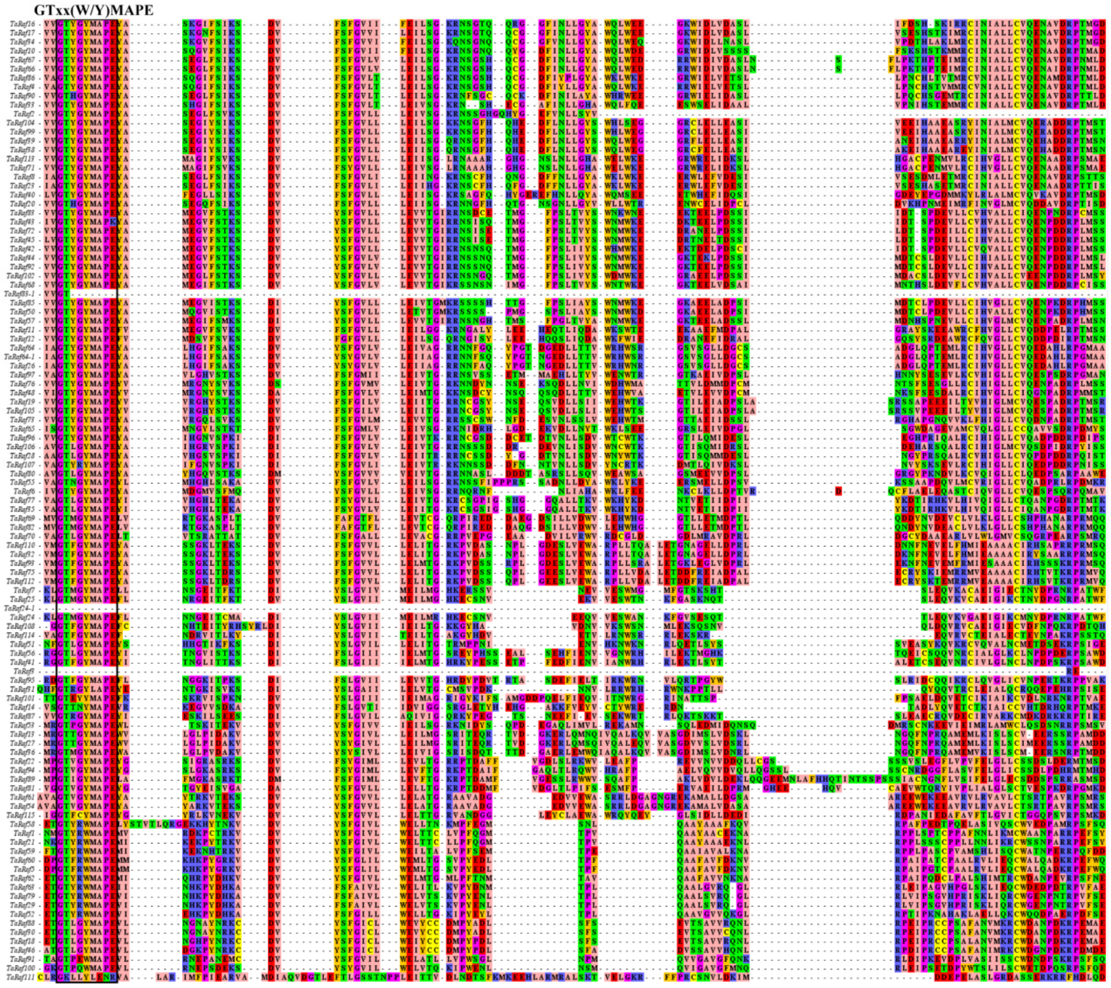

Supplemental Figure S6. Alignment of Raf substype from wheat MAP3K. The highlighted part shows the conserved motif.

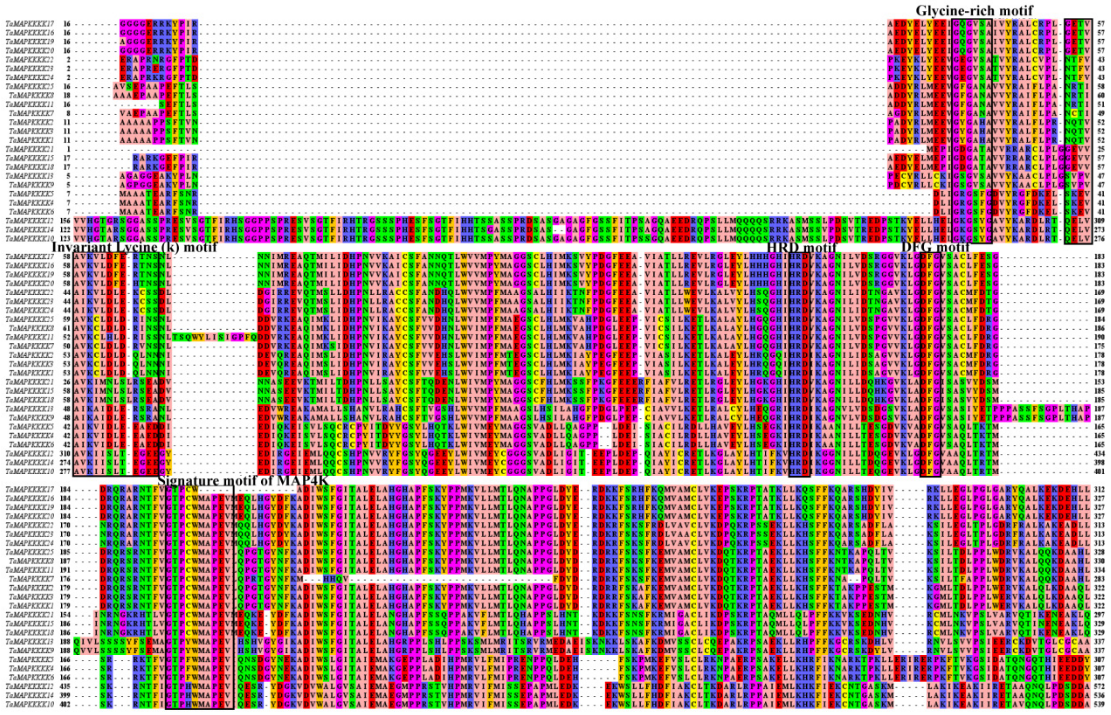

Supplemental Figure S7. Alignment of TaMAP4K family. The highlighted part shows the conserved motif.

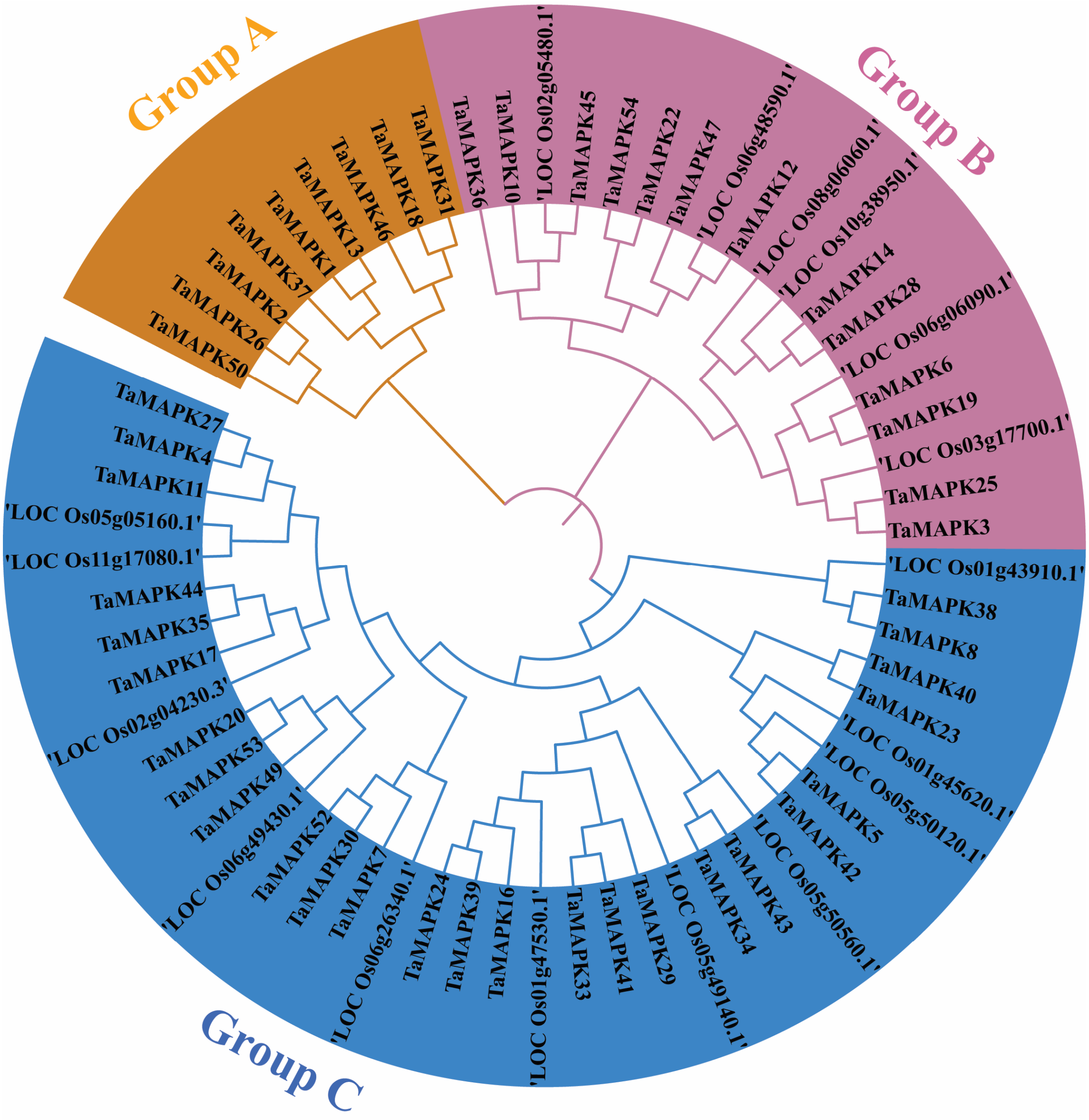

Supplementary Figure S8. Phylogenetic tree analysis of *MAPK* genes from wheat and rice.

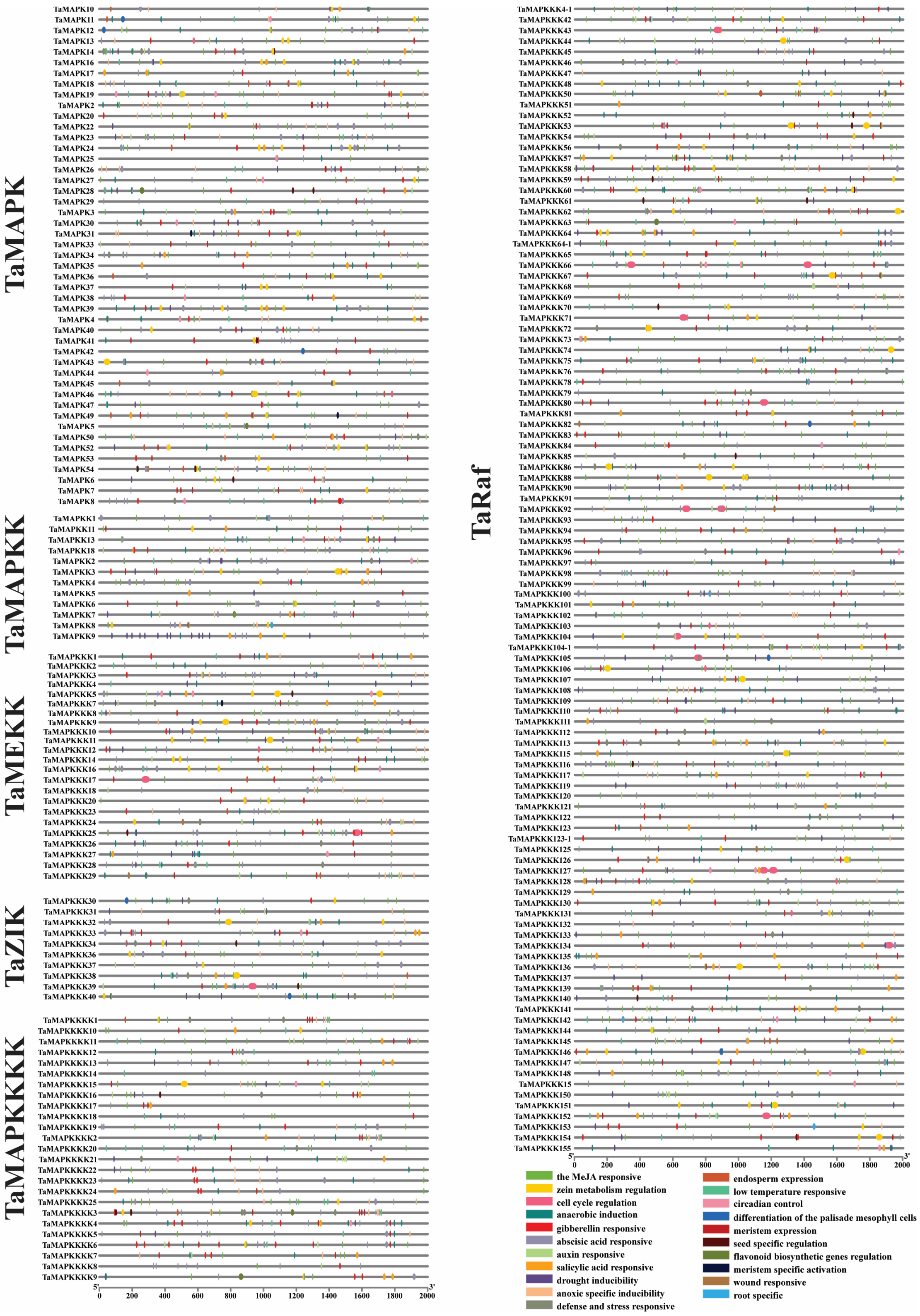

Supplementary Figure S9. Predicted cis-regulatory elements in the 1500 bp upstream promoter regions of *TaMAPK-TaMAP4K* cascade genes.

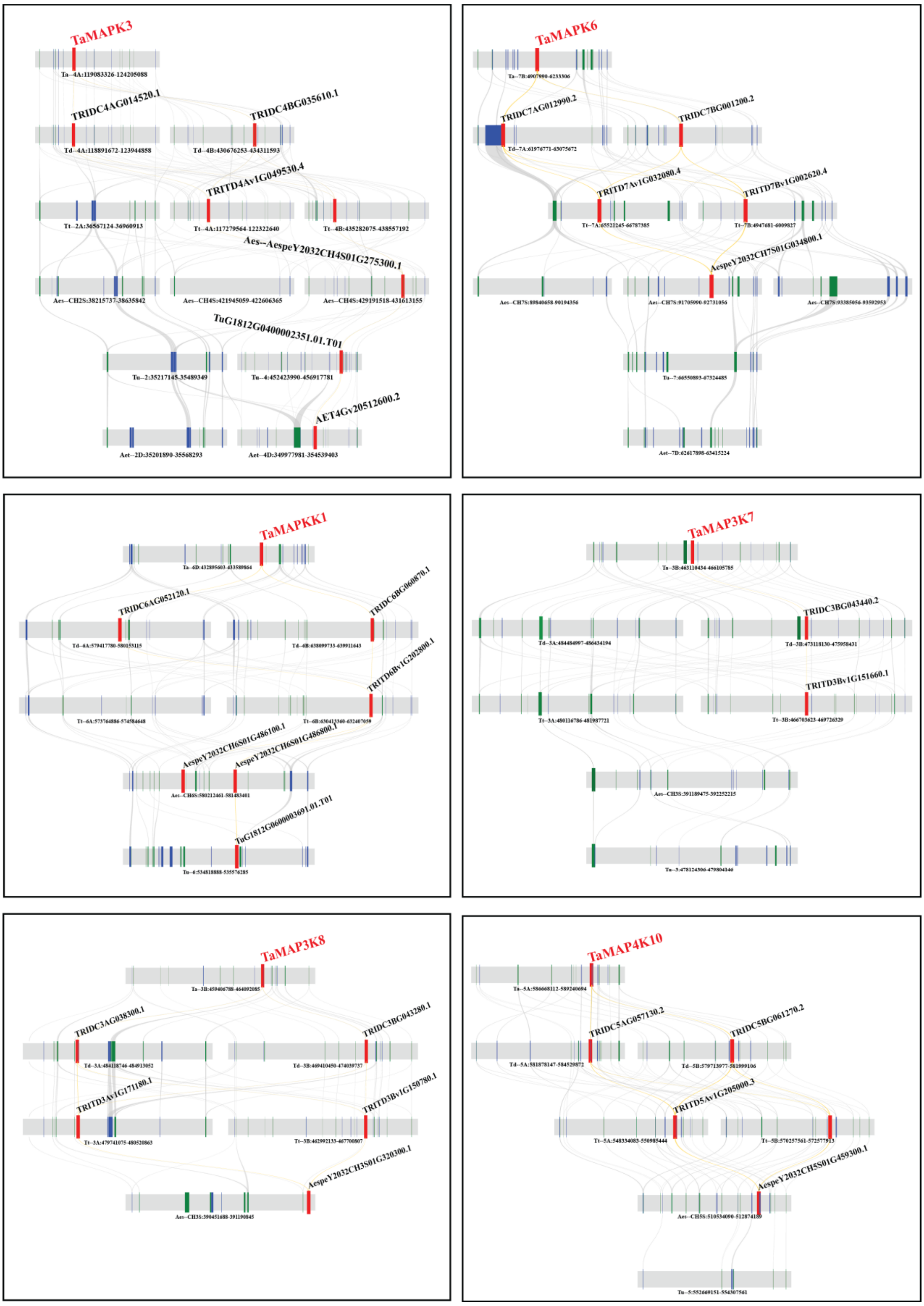

Supplementary Figure S10. The synteny analysis of *TaMAPK3* and *6*, *TaMAP2K1*, *TaMAP3K7* and *8*, and *TaMAP4K10* were conducted across 5 phylogenetically related species.
