# Supplementary material for: Bioinformatic Identification and Expression Analyses of the MAPK–MAP4K Gene Family Reveal a Putative Functional MAP4K10-MAP3K7/8-MAP2K1/11-MAPK3/6 Cascade in Wheat (Triticum aestivum L.)
Source: Plants (Basel). 2024 Mar 24;13(7):941. doi: 10.3390/plants13070941 (PMC11013086; doi:10.3390/plants13070941)
Supplement: Supplementary file 1 [file plants-13-00941-s001.zip › plants-2867660-supplementary/Supplementary Figure S1-S10 and Supplementary table S1-S7/Supplementary table S1-S7/Supplementary table 1.pdf]

Table S1. Physicochemical property aspects of TaMAPK-TaMAP4K											
**Gene Name**			**Gene ID**	**Chr start**	**Chr end**	**Length (aa)**	**MW (kDa)**	**pI**	**Subcellular Localizatio**	**GRAGY**	**Transmem brane**
**TaMAPK1**			TraesCS6B 02G296700. 2	532338556	532341647	460	51.60	9.71	Nucleus	-0.388	none
**TaMAPK2**			TraesCS4A 02G336800. 2	619184426	619188858	445	50.90	6.14	Nucleus	-0.345	none
**TaMAPK3**			TraesCS4A	120604688	120608133	369	42.83	5.46	Nucleus	-0.298	none
**TaMAPK4**			TraesCS1D 02G088000. 2	73674523	73680096	553	62.75	8.85	Nucleus	-0.513	none
**TaMAPK5**			TraesCS1D 02G422800. 1	478142725	478147790	605	68.33	7.71	Nucleus	-0.561	none
**TaMAPK6**			TraesCS7B	5513957	5521157	393	44.24	5.50	Nucleus	-0.238	none
**TaMAPK7**			TraesCS7D 02G342800. 3	439463570	439478002	421	48.54	8.91	Nucleus	-0.426	none
**TaMAPK8**			TraesCS3D 02G225600. 1	307226730	307233549	613	69.88	9.04	Nucleus	-0.554	none
**TaMAPK10**			TraesCS6B 02G146300. 1	146837429	146841338	369	42.36	6.67	Nucleus	-0.187	none
**TaMAPK11**			TraesCS1A 02G086500. 1	73295476	73300550	549	62.31	8.74	Nucleus	-0.492	none
**TaMAPK12**			TraesCS7A 02G422500. 1	612990989	612994043	824	47.32	8.24	Nucleus	-0.195	none
**TaMAPK13**			TraesCS6D 02G245500. 3	347684447	347687540	460	51.56	9.71	Nucleus	-0.406	none

**TaMAPK14**	TraesCS1A02G184500.1	334446534	334452739	377	42.87	5.75	Nucleus	-0.328	none
**TaMAPK16**	TraesCS3B02G270200.1	434980667	434986997	586	67.17	9.32	Nucleus	-0.551	none
**TaMAPK17**	TraesCS6B02G127800.1	123702609	123715378	598	56.88	6.43	Nucleus	-0.374	none
**TaMAPK18**	TraesCS7D02G044100.1	22581173	22585094	485	55.11	9.49	Nucleus	-0.487	none
**TaMAPK19**	TraesCS7A02G111300.1	68086983	68094361	396	44.44	5.50	Nucleus	-0.223	none
**TaMAPK20**	TraesCS7D02G403700.1	520827434	520832219	581	65.47	7.32	Nucleus	-0.482	none
**TaMAPK22**	TraesCS7D02G414900.1	533432518	533434635	377	43.02	8.04	Nucleus	-0.157	none
**TaMAPK23**	TraesCS3D02G221700.1	301965173	301974102	488	55.77	6.82	Nucleus	-0.425	none
**TaMAPK24**	TraesCS3D02G242200.2	337303815	337310057	584	66.87	9.32	Nucleus	-0.539	none
**TaMAPK25**	TraesCS4D02G198600.1	344940376	344943564	369	42.82	5.46	Nucleus	-0.312	none
**TaMAPK26**	TraesCS5D02G534000.2	548349748	548354652	441	50.24	5.74	Nucleus	-0.322	none
**TaMAPK27**	TraesCS1B02G104900.1	115953191	115959231	549	62.38	8.92	Nucleus	-0.492	none

**TaMAPK28**	TraesCS1B02G192600.3	344838254	344842121	376	42.86	5.75	Nucleus	-0.329	none
**TaMAPK29**	TraesCS1B02G431400.2	655781234	655786592	556	63.02	9.04	Nucleus	-0.513	none
**TaMAPK30**	TraesCS7A02G335300.2	490831796	490841117	421	48.45	8.83	Nucleus	-0.414	none
**TaMAPK31**	TraesCS4A02G434800.1	705502495	705508717	484	54.84	9.50	Nucleus	-0.476	none
**TaMAPK33**	TraesCS1D02G410100.1	471618853	471623653	555	62.75	9.33	Nucleus	-0.523	none
**TaMAPK34**	TraesCS1D02G428900.1	480888268	480894402	599	66.65	9.22	Nucleus	-0.416	none
**TaMAPK35**	TraesCS6A02G099600.1	67126390	67137086	500	57.25	6.36	Nucleus	-0.429	none
**TaMAPK36**	TraesCS6A02G118100.1	88854990	88859282	380	43.50	7.20	Nucleus	-0.181	none
**TaMAPK37**	TraesCS6A02G269400.1	495685844	495688911	460	51.54	9.71	Nucleus	-0.4	none
**TaMAPK38**	TraesCS3B02G256700.1	414020367	414026235	602	68.57	8.99	Nucleus	-0.534	none
**TaMAPK39**	TraesCS3A02G242100.1	454510686	454516596	582	66.51	9.25	Nucleus	-0.522	none
**TaMAPK40**	TraesCS3A02G231700.1	432855223	432868359	490	56.03	6.17	Nucleus	-0.429	none

**TaMAPK41**	TraesCS1A02G402400.2	566009663	566015714	548	62.14	9.06	Nucleus	-0.49	none
**TaMAPK42**	TraesCS1A02G415300.1	574351875	574357108	606	68.45	7.71	Nucleus	-0.581	none
**TaMAPK43**	TraesCS1A02G421000.1	577468378	577474524	598	66.51	9.27	Nucleus	-0.409	none
**TaMAPK44**	TraesCS6D02G082900.2	47937796	47944111	505	58.04	6.48	Nucleus	-0.437	none
**TaMAPK45**	TraesCS6D02G108100.1	72229215	72231632	382	43.82	7.63	Nucleus	-0.205	none
**TaMAPK46**	TraesCS7A02G049000.1	22930536	22936407	492	56.02	9.46	Nucleus	-0.457	none
**TaMAPK47**	TraesCS7A02G029700.1	12115497	12118204	324	37.21	9.16	Nucleus	-0.237	none
**TaMAPK49**	TraesCS7A02G410700.2	598216576	598222568	578	65.15	6.85	Nucleus	-0.496	none
**TaMAPK50**	TraesCS5B02G536500.1	692559588	692565223	443	50.42	6.04	Nucleus	-0.351	none
**TaMAPK52**	TraesCS7B02G246900.3	457243859	457252780	421	48.49	8.91	Nucleus	-0.418	none
**TaMAPK53**	TraesCS7B02G309900.1	553883880	553888100	578	65.21	6.85	Nucleus	-0.485	none
**TaMAPK54**	TraesCS7B02G322900.1	573261339	573264067	377	43.19	7.20	Nucleus	-0.188	none

**TaMAPKK1**	TraesCS6D02G328800.1	433408096	433409202	368	39.62	9.30	Cytoplasm. Nucleus	-0.323	none
**TaMAPKK2**	TraesCS5B02G565100.3	710226104	710231942	525	58.65	7.89	Nucleus	-0.329	none
**TaMAPKK3**	TraesCS5D02G130900.2	207244486	207252504	525	58.60	5.34	Nucleus	-0.242	none
**TaMAPKK4**	TraesCS5A02G122700.4	267940928	267946212	299	33.60	5.89	Nucleus	-0.039	none
**TaMAPKK5**	TraesCS4B02G049000.1	37296414	37297430	338	36.34	8.23	Nucleus	-0.117	none
**TaMAPKK6**	TraesCS4B02G048100.1	36012946	36013977	343	36.19	8.95	Nucleus	0.076	none
**TaMAPKK7**	TraesCS4B02G048600.1	37084618	37085631	337	35.63	8.81	Nucleus	0.09	none
**TaMAPKK8**	TraesCS4B02G048900.1	37173459	37174463	334	35.92	8.62	Nucleus	-0.106	none
**TaMAPKK9**	TraesCS3B02G066300.1	39545184	39546200	338	36.26	7.72	Nucleus	-0.052	none
**TaMAPKK11**	TraesCS4D02G048800.1	25193488	25194492	334	35.95	7.76	Nucleus	-0.096	none
**TaMAPKK12**	TraesCS4D02G048500.1	25159546	25160577	343	36.26	8.76	Nucleus	0.077	none
**TaMAPKK13**	TraesCS5D02G549600.1	556549608	556556744	523	58.42	5.71	Nucleus	-0.228	none

**TaMAPKK14**			TraesCS4A02G265900.1	578007015	578008028	337	36.11	7.15	Nucleus	-0.061	none
**TaMAPKK15**			TraesCS4A02G266000.1	578175110	578176265	337	36.26	8.21	Nucleus	-0.07	none
**TaMAPKK16**			TraesCS4A02G266100.1	578233482	578234994	334	35.77	8.84	Nucleus	-0.082	none
**TaMAPKK17**			TraesCS4A02G266200.1	578382450	578383463	337	35.60	8.88	Nucleus	0.098	none
**TaMAPKK18**			TraesCS5B02G122600.1	220811093	220817726	476	53.82	5.74	Nucleus	-0.311	none
**TaMAPKK**	**MEKK**	TaMEKK1	TraesCS2A	663319194	663325688	827	89.98	9.73	Nucleus	-0.671	none
**TaMAPKKK2**		TaMEKK2	TraesCS4D02G027600.1	12408039	12412107	570	63.22	8.89	Nucleus	-0.375	none
**TaMAPKKK3**		TaMEKK3	TraesCS4B02G210600.2	449128767	449134380	705	77.22	6.53	Nucleus	-0.286	none
**TaMAPKKK4**		TaMEKK4	TraesCS6A02G245000.3	456595800	456610297	878	94.93	9.94	Nucleus	-0.624	none
**TaMAPKKK4-1**		TaMEKK4-1	TraesCS6B02G279300.1	504591712	504606525	786	84.73	9.83	Nucleus	-0.606	none
**TaMAPKKK5**		TaMEKK5	TraesCS2A02G199700.1	172214508	172218294	543	60.97	6.59	Nucleus	-0.452	none
**TaMAPKKK7**		TaMEKK7	TraesCS3B02G289500.1	464615550	464617173	473	49.83	4.64	Nucleus	-0.147	none
**TaMAPKKK8**		TaMEKK8	TraesCS3B02G288100.1	462866244	462867785	477	51.60	5.26	Nucleus	-0.138	none

**TaMAPKKK9**	TaMEKK9	TraesCS3B02G288300.1	462890522	462891675	366	38.24	4.55	Nucleus	-0.172	none
**TaMAPKKK10**	TaMEKK10	TraesCS4D02G211300.2	363679689	363686267	710	77.70	6.55	Nucleus	-0.295	none
**TaMAPKKK11**	TaMEKK11	TraesCS5D02G475900.1	515114772	515119147	534	59.81	6.43	Nucleus	-0.45	none
**TaMAPKKK12**	TaMEKK12	TraesCS4A02G093800.2	101741028	101746878	710	77.72	6.55	Nucleus	-0.306	none
**TaMAPKKK14**	TaMEKK14	TraesCS5A02G118200.1	242531195	242538426	553	62.10	6.37	Nucleus	-0.344	none
**TaMAPKKK15**	TaMEKK15	TraesCS5A02G463100.2	643328443	643333346	534	59.83	6.43	Nucleus	-0.448	none
**TaMAPKKK16**	TaMEKK16	TraesCS5B02G474500.1	647940529	647944797	535	59.93	6.31	Nucleus	-0.464	none
**TaMAPKKK17**	TaMEKK17	TraesCS5A02G200800.1	406667546	406672895	682	74.39	6.29	Nucleus	-0.559	none
**TaMAPKKK18**	TaMEKK18	TraesCS2B02G526200.3	720473250	720486136	1324	146.41	5.83	Nucleus	-0.298	none
**TaMAPKKK20**	TaMEKK20	TraesCS2A02G498000.3	728546777	728559173	1323	146.44	5.86	Nucleus	-0.301	none
**TaMAPKKK21**	TaMEKK21	TraesCS6A02G149900.1	133414193	133419353	543	61.18	6.83	Nucleus	-0.552	none
**TaMAPKKK22**	TaMEKK22	TraesCS5A02G392500.1	588731002	588738966	825	89.69	5.31	Nucleus	-0.504	none

**TaMAPKKK23**		TaMEKK23	TraesCS6D02G139200.1	108447173	108452128	509	57.60	6.71	Nucleus	-0.504	none
**TaMAPKKK24**		TaMEKK24	TraesCS5B02G199400.1	359309183	359314533	680	74.21	6.29	Nucleus	-0.554	none
**TaMAPKKK25**		TaMEKK25	TraesCS5B02G196400.1	354227906	354229839	518	56.47	6.01	Nucleus	-0.16	none
**TaMAPKKK26**		TaMEKK26	TraesCS2D02G093700.1	45528501	45538616	1335	146.06	8.01	Nucleus	-0.051	none
**TaMAPKKK27**		TaMEKK27	TraesCS2B02G110500.1	72570247	72580245	1335	146.06	8.01	Nucleus	-0.049	none
**TaMAPKKK28**		TaMEKK28	TraesCS2A02G095300.1	48855363	48865404	1332	145.87	8.09	Nucleus	-0.042	none
**TaMAPKKK29**		TaMEKK29	TraesCS5D02G206500.1	312603930	312609217	682	74.38	6.29	Nucleus	-0.547	none
**TaMAPKKK30**	**ZIK**	TaZIK1	TraesCS5D02G145100.1	231743581	231750603	640	70.63	5.71	Nucleus	-0.606	none
**TaMAPKKK31**		TaZIK2	TraesCS6D02G236400.1	332952908	332957161	616	68.85	4.86	Nucleus	-0.325	none
**TaMAPKKK32**		TaZIK3	TraesCS2A02G195900.2	165847896	165850719	701	78.40	5.53	Nucleus	-0.506	none
**TaMAPKKK33**		TaZIK4	TraesCS6B02G270400.1	487199066	487203196	617	69.03	4.89	Nucleus	-0.367	none
**TaMAPKKK34**		TaZIK5	TraesCS2D02G197600.1	145361837	145364153	319	35.94	6.46	Nucleus	-0.443	none

**TaMAPKKK36**		TaZIK7	TraesCS2B02G223600.1	213252738	213256205	703	78.63	5.61	Cell membrane. Nucleus	-0.529	none
**TaMAPKKK37**		TaZIK8	TraesCS2B02G216800.1	202919276	202921526	322	36.36	6.41	Nucleus	-0.466	none
**TaMAPKKK38**		TaZIK9	TraesCS1D02G026200.2	10715309	10722269	615	68.76	5.04	Nucleus	-0.631	none
**TaMAPKKK39**		TaZIK10	TraesCS6A02G255100.2	472517606	472521858	616	68.94	4.82	Nucleus	-0.35	none
**TaMAPKKK40**		TaZIK11	TraesCS5B02G146100.1	273714479	273721500	640	70.50	5.55	Nucleus	-0.6	none
**TaMAPKKK41**	**Raf**	**TaRaf1**	TraesCS6A02G172600.1	183765974	183767047	341	38.49	8.87	Nucleus	-0.263	none
**TaMAPKKK42**		**TaRaf2**	TraesCSU02G203100.1	302673848	302675171	281	31.63	8.28	Nucleus	-0.288	none
**TaMAPKKK43**		**TaRaf3**	TraesCS3B02G110300.1	76121219	76122267	146	16.52	6.20	Nucleus	-0.304	none
**TaMAPKKK44**		**TaRaf4**	TraesCS2D02G003900.1	2448415	2452565	666	73.48	6.20	Nucleus	-0.047	yes
**TaMAPKKK45**		**TaRaf5**	TraesCS3D02G273200.1	378922472	378925538	601	67.50	9.28	Nucleus	-0.586	none
**TaMAPKKK46**		**TaRaf6**	TraesCS2D02G050700.1	18755065	18763417	842	93.01	6.62	Chloroplast. Nucleus.	-0.078	yes
**TaMAPKKK47**		**TaRaf7**	TraesCS7D02G022200.1	10480193	10483005	454	50.76	5.30	Cell membrane. Cytoplasm. Nucleus.	-0.418	none

**TaMAPKKK48**	**TaRaf8**	TraesCS7D02G079100.1	46798827	46802577	590	66.29	5.16	Nucleus	-0.153	yes
**TaMAPKKK50**	**TaRaf10**	TraesCS7D02G099200.2	59413722	59416749	662	73.14	5.78	Cell membrane. Nucleus.	-0.05	yes
**TaMAPKKK51**	**TaRaf11**	TraesCS7D02G230200.1	191491327	191494118	411	45.94	7.61	Cell membrane. Nucleus.	-0.084	yes
**TaMAPKKK52**	**TaRaf12**	TraesCS7D02G230500.1	191554886	191558690	421	46.94	8.84	Cell membrane. Nucleus.	-0.037	yes
**TaMAPKKK53**	**TaRaf13**	TraesCS1B02G372400.1	603091105	603093899	804	88.12	5.43	Nucleus	-0.064	yes
**TaMAPKKK54**	**TaRaf14**	TraesCS7D02G503600.1	608808978	608814007	714	79.66	6.28	Chloroplast. Cytoplasm.	-0.306	yes
**TaMAPKKK56**	**TaRaf16**	TraesCS3A02G039100.1	20834670	20837048	483	54.10	7.10	Cell membrane. Nucleus.	-0.083	yes
**TaMAPKK**	**TaRaf17**	TraesCS3D	15371189	15373968	644	70.45	7.43	Cell	0.016	yes
**TaMAPKKK58**	**TaRaf18**	TraesCS3B02G259800.1	417712407	417716508	385	42.62	7.57	Nucleus	-0.375	none
**TaMAPKKK59**	**TaRaf19**	TraesCS2A02G216900.1	203718668	203726292	669	72.89	6.26	Nucleus	-0.092	yes
**TaMAPKKK60**	**TaRaf20**	TraesCS2A02G217000.1	203756927	203768418	687	76.38	5.89	Nucleus	-0.231	yes
**TaMAPKK**	**TaRaf21**	TraesCS7D	498527161	498529159	396	44.67	9.19	Nucleus	-0.357	none
**TaMAPKKK62**	**TaRaf22**	TraesCS4A02G313900.1	604208153	604212003	1095	118.89	6.14	Cell membrane.	0.239	yes

**TaMAPKKK63**	**TaRaf23**	TraesCS4A02G383000.1	660923030	660926301	607	67.95	6.00	Nucleus	0.011	yes
**TaMAPKKK64**	**TaRaf24**	TraesCS4A02G465900.2	729138021	729142814	722	80.84	5.28	Nucleus	-0.2	none
**TaMAPKK**	**TaRaf24-1**	TraesCS4A	728455965	728458756	168	18.72	5.85	Nucleus	-0.07	none
**TaMAPKKK65**	**TaRaf25**	TraesCS4A02G465000.1	728517691	728524395	741	82.77	5.79	Nucleus	-0.107	none
**TaMAPKKK66**	**TaRaf26**	TraesCS1D02G273800.2	368278445	368281075	421	46.94	8.84	Cell membrane. Nucleus.	-0.037	yes
**TaMAPKKK67**	**TaRaf27**	TraesCS1D02G360600.1	443716098	443718946	804	88.21	5.44	Nucleus	-0.071	yes
**TaMAPKKK68**	**TaRaf28**	TraesCS1D02G431400.1	481556383	481559743	710	76.79	6.00	Nucleus	-0.097	yes
**TaMAPKKK69**	**TaRaf29**	TraesCS2D02G588200.1	645705545	645712161	597	66.95	5.48	Nucleus	-0.416	none
**TaMAPKKK70**	**TaRaf30**	TraesCS1D02G423800.1	478411677	478415662	374	41.10	7.05	Cytoplasm. Nucleus.	-0.299	none
**TaMAPKKK71**	**TaRaf31**	TraesCS6A02G004500.1	1877233	1883101	794	90.75	5.59	Nucleus	-0.357	none
**TaMAPKKK72**	**TaRaf32**	TraesCS3A02G003900.1	1924585	1928794	687	72.42	6.26	Nucleus	-0.429	yes
**TaMAPKKK73**	**TaRaf33**	TraesCS3A02G039200.1	20841799	20844493	682	73.97	7.78	Cell membrane. Nucleus.	0.019	yes
**TaMAPKKK74**	**TaRaf34**	TraesCS3A02G039400.1	20931615	20935238	632	69.75	8.19	Chloroplast.	-0.007	yes

**TaMAPKKK75**	**TaRaf35**	TraesCS3A02G096500.1	61445227	61448967	653	69.90	8.98	Chloroplast.	0.034	yes
**TaMAPKKK76**	**TaRaf36**	TraesCS3A02G246100.1	461839239	461841696	813	89.58	6.40	Chloroplast.	-0.155	yes
**TaMAPKKK78**	**TaRaf38**	TraesCS3D02G501400.1	590305938	590309917	716	79.31	5.85	Cell membrane. Nucleus.	-0.111	yes
**TaMAPKKK79**	**TaRaf39**	TraesCS3A02G493900.1	720213465	720223668	704	78.64	5.75	Cell membrane.	-0.082	yes
**TaMAPKKK80**	**TaRaf40**	TraesCS2A02G217600.1	204885066	204888307	477	50.96	8.00	Nucleus	-0.255	yes
**TaMAPKKK81**	**TaRaf41**	TraesCS5A02G292500.1	502584783	502592915	775	88.02	6.28	Nucleus	-0.284	none
**TaMAPKKK82**	**TaRaf42**	TraesCSU02G072500.1	63290513	63296324	840	92.42	5.99	Cell membrane. Nucleus.	-0.135	yes
**TaMAPKKK83**	**TaRaf43**	TraesCS5A02G351500.1	554181335	554183861	310	34.93	7.61	Nucleus	-0.235	none
**TaMAPKKK84**	**TaRaf44**	TraesCS5A02G351000.1	553490210	553499037	848	93.63	7.05	Cell membrane. Nucleus.	-0.176	yes
**TaMAPKKK85**	**TaRaf45**	TraesCS5A02G352000.1	554234499	554238433	836	92.61	7.09	Nucleus	-0.204	yes
**TaMAPKKK86**	**TaRaf46**	TraesCS5D02G386800.1	456607644	456612314	373	41.35	8.53	Nucleus	-0.355	none
**TaMAPKKK88**	**TaRaf48**	TraesCS1A02G422800.1	578196408	578199395	690	75.15	5.95	Nucleus	-0.066	yes

**TaMAPKKK90**	**TaRaf50**	TraesCS7A02G152100.1	105511739	105513870	362	40.41	7.04	Nucleus	-0.274	none
**TaMAPKKK91**	**TaRaf51**	TraesCS4D02G089300.1	64706830	64719914	755	85.85	6.45	Nucleus	-0.263	none
**TaMAPKKK92**	**TaRaf52**	TraesCS5B02G337300.1	520868787	520875955	598	65.62	5.78	Nucleus	-0.393	none
**TaMAPKKK93**	**TaRaf53**	TraesCS5D02G018800.1	11301422	11304114	645	72.62	6.00	Nucleus	-0.296	yes
**TaMAPKKK94**	**TaRaf54**	TraesCS5B02G012000.1	11764765	11774531	1017	106.30	4.67	Cell membrane	0.122	yes
**TaMAPKKK95**	**TaRaf55**	TraesCS5B02G204900.1	373215862	373220653	439	48.20	9.72	Nucleus	-0.515	none
**TaMAPKKK96**	**TaRaf56**	TraesCS5B02G292000.2	477512642	477518372	788	88.84	8.46	Nucleus	-0.263	none
**TaMAPKKK97**	**TaRaf57**	TraesCS5B02G353800.1	533375142	533376026	225	25.29	6.52	Nucleus	-0.145	none
**TaMAPKKK98**	**TaRaf58**	TraesCS5D02G482000.1	519649924	519653802	418	45.49	8.00	Nucleus	-0.276	none
**TaMAPKKK99**	**TaRaf59**	TraesCS3A02G001500.1	1028128	1031945	365	40.72	9.31	Nucleus	-0.247	none
**TaMAPKKK100**	**TaRaf60**	TraesCS3A02G274000.1	502835637	502839127	601	67.35	9.31	Nucleus	-0.593	none
**TaMAPKKK101**	**TaRaf61**	TraesCS5D02G019400.1	11949643	11952687	1014	105.97	6.33	Cell membrane	0.123	yes

**TaMAPKK K102**	**TaRaf62**	TraesCS7D02G474700.1	587069445	587072872	425	47.20	6.43	Nucleus	-0.185	none
**TaMAPKK K103**	**TaRaf63**	TraesCS7A02G326700.1	474729873	474737049	536	60.15	5.48	Nucleus	-0.332	none
**TaMAPKK K104**	**TaRaf64**	TraesCS1D02G273600.1	367915349	367918308	681	73.38	6.00	Nucleus	-0.117	none
**TaMAPKK**	**TaRaf64-1**	TraesCS1B	492327989	492331022	678	73.33	6.38	Nucleus	-0.141	yes
**TaMAPKK K105**	**TaRaf65**	TraesCS3A02G045200.1	24205959	24209204	399	43.36	6.72	Nucleus	-0.193	none
**TaMAPKK K106**	**TaRaf66**	TraesCS4A02G456900.1	722287491	722294625	647	71.63	6.20	Cell membrane. Nucleus	-0.133	yes
**TaMAPKK K107**	**TaRaf67**	TraesCS7A02G032700.1	13832914	13835159	480	54.35	8.59	Cell membrane. Nucleus	-0.128	yes
**TaMAPKK K108**	**TaRaf68**	TraesCS3D02G023600.1	7496834	7501605	830	91.83	7.19	Chloroplast. Nucleus	-0.208	yes
**TaMAPKK K109**	**TaRaf69**	TraesCS2D02G219800.1	186995581	186997635	684	74.88	6.00	Cell membrane. Cytoplasm. Nucleus	-0.064	yes
**TaMAPKK K110**	**TaRaf70**	TraesCS4B02G289100.1	574169282	574171714	680	72.78	6.51	Nucleus	0.061	yes
**TaMAPKK K111**	**TaRaf71**	TraesCS3A02G315100.1	556354721	556361160	810	89.49	6.45	Nucleus	-0.054	yes
**TaMAPKK K112**	**TaRaf72**	TraesCS5D02G359500.1	438720779	438724678	846	93.64	6.60	Nucleus	-0.159	yes

**TaMAPKKK113**	**TaRaf73**	TraesCS3D02G108500.1	61696216	61699845	669	72.21	8.21	Nucleus	-0.039	yes
**TaMAPKKK115**	**TaRaf75**	TraesCS3A02G229800.1	429615380	429618754	720	74.97	8.84	Nucleus	-0.402	yes
**TaMAPKKK116**	**TaRaf76**	TraesCS1B02G454000.2	670025362	670028705	663	72.92	7.09	Nucleus	-0.105	yes
**TaMAPKKK117**	**TaRaf77**	TraesCS3D02G097000.1	48958881	48962702	648	69.64	8.85	Chloroplast. Nucleus	0.04	yes
**TaMAPKKK119**	**TaRaf79**	TraesCS2A02G577000.1	771236940	771242936	558	62.29	5.51	Nucleus	-0.427	none
**TaMAPKKK120**	**TaRaf80**	TraesCS4A02G317600.1	606796989	606806238	674	73.40	8.38	Chloroplast. Nucleus	-0.119	yes
**TaMAPKKK121**	**TaRaf81**	TraesCS2D02G066900.1	27998176	28002775	997	108.03	6.01	Cell membrane	0.119	yes
**TaMAPKKK122**	**TaRaf82**	TraesCS2A02G214000.1	199713870	199715915	681	74.62	6.07	Cytoplasm. Extracell. Nucleus	-0.088	yes
**TaMAPKKK123**	**TaRaf83**	TraesCS5D02G358700.1	438557604	438562390	839	92.71	6.63	Nucleus	-0.161	yes
**TaMAPKK**	**TaRaf83-1**	TraesCS5D	438454390	438456577	457	50.56	8.22	Nucleus	-0.216	yes
**TaMAPKKK125**	**TaRaf85**	TraesCS7D02G153800.1	101736527	101739826	826	90.93	6.86	Nucleus	-0.133	yes
**TaMAPKKK126**	**TaRaf86**	TraesCSU02G011500.1	15720917	15724380	644	72.97	6.33	Nucleus	0.023	yes
**TaMAPKKK127**	**TaRaf87**	TraesCS6D02G339600.1	438942402	438946634	928	104.30	5.77	Chloroplast. Cytoplasm. Nucleus	-0.412	none

**TaMAPKKK128**	**TaRaf88**	TraesCS1B02G446500.1	666535726	666539402	372	40.91	7.54	Nucleus	-0.281	none
**TaMAPKKK129**	**TaRaf89**	TraesCS6B02G320800.1	567847786	567851826	986	108.30	6.21	Cell membrane	0.086	yes
**TaMAPKKK130**	**TaRaf90**	TraesCS7A02G044600.1	20510405	20512958	405	46.16	6.25	Nucleus	-0.169	yes
**TaMAPKKK131**	**TaRaf91**	TraesCS6B02G215100.2	290710332	290718338	860	93.43	5.41	Nucleus	-0.228	none
**TaMAPKKK132**	**TaRaf92**	TraesCS5B02G353600.1	533033821	533038318	846	93.47	5.85	Nucleus	-0.136	yes
**TaMAPKKK133**	**TaRaf93**	TraesCS1D02G004300.1	2108471	2112601	656	68.93	9.64	Nucleus	-0.394	yes
**TaMAPKKK134**	**TaRaf94**	TraesCS5D02G547500.1	555555819	555559333	765	83.36	6.02	Cell membrane	0.191	yes
**TaMAPKKK135**	**TaRaf95**	TraesCS2D02G598800.1	650601306	650604027	499	55.24	5.84	Nucleus	-0.286	none
**TaMAPKKK136**	**TaRaf96**	TraesCS2B02G241600.1	245686727	245691716	675	72.70	5.94	Nucleus	0.015	yes
**TaMAPKKK137**	**TaRaf97**	TraesCS3D02G472000.1	574118271	574125655	746	80.84	5.82	Nucleus	-0.239	yes
**TaMAPKKK139**	**TaRaf99**	TraesCS3A02G493500.1	719931689	719937693	711	79.40	6.02	Cell membrane	-0.137	yes
**TaMAPKKK140**	**TaRaf100**	TraesCS5D02G097900.1	108527463	108535238	753	83.11	7.94	Nucleus	-0.646	none

**TaMAPKKK141**		**TaRaf101**	TraesCS7D02G000800.1	981342	987541	965	107.83	6.39	Cytoplasm. Nucleus	-0.301	yes
**TaMAPKKK142**		**TaRaf102**	TraesCS2A02G032200.1	14729078	14730418	323	36.46	5.40	Nucleus	-0.275	none
**TaMAPKKK144**		**TaRaf104**	TraesCS3D02G501100.1	590199543	590205678	713	79.61	5.82	Cell membrane. Nucleus	-0.134	yes
**TaMAPKKK145**		**TaRaf105**	TraesCS2B02G242300.1	246113761	246119395	669	72.81	6.05	Cell membrane. Nucleus	-0.096	yes
**TaMAPKKK146**		**TaRaf106**	TraesCS2B02G241400.1	245317184	245320539	667	72.23	6.12	Chloroplast. Nucleus	0.058	yes
**TaMAPKKK147**		**TaRaf107**	TraesCS2A02G216600.1	203325595	203329022	668	73.47	8.53	Nucleus	-0.128	yes
**TaMAPKKK148**		**TaRaf108**	TraesCS1A02G003900.1	2391500	2393497	341	39.38	6.23	Nucleus	-0.331	none
**TaMAPKKK150**		**TaRaf110**	TraesCS3B02G008600.2	4323030	4327039	686	71.89	6.26	Nucleus	-0.438	yes
**TaMAPKKK151**		**TaRaf111**	TraesCS3B02G123800.1	96812311	96816428	577	63.83	5.79	Nucleus	-0.202	none
**TaMAPKKK152**		**TaRaf112**	TraesCS3B02G259100.1	416805836	416810951	698	72.98	5.89	Nucleus	-0.449	yes
**TaMAPKKK153**		**TaRaf113**	TraesCS3B02G351800.1	561590530	561591713	302	33.43	6.25	Nucleus	0.002	none
**TaMAPKKK154**		**TaRaf114**	TraesCS3B02G478400.1	726770459	726780334	770	87.44	5.95	Nucleus	-0.318	none

**TaMAPKKK155**		**TaRaf115**	TraesCS6B02G217100.1	298820612	298821648	318	36.07	5.20	Nucleus	-0.025	none
**TaMAPKKK1**			TraesCS1A02G181900.1	328244901	328253761	714	79.25	6.11	Nucleus	-0.543	none
**TaMAPKKK2**			TraesCS1B02G199100.2	357209635	357218446	724	80.56	6.27	Nucleus	-0.537	none
**TaMAPKKK3**			TraesCS1D02G185000.2	255824893	255833850	731	81.45	6.30	Nucleus	-0.555	none
**TaMAPKKK4**			TraesCS2A02G233400.1	278445853	278467939	692	76.02	6.97	Nucleus	-0.665	none
**TaMAPKKK5**			TraesCS2B02G249900.1	259009416	259028116	692	76.08	7.00	Nucleus	-0.672	none
**TaMAPKKK6**			TraesCS2D02G232200.1	204990636	205004380	692	76.09	7.00	Nucleus	-0.654	none
**TaMAPKKK7**			TraesCS4B02G395600.1	670201548	670229484	629	69.54	9.51	Nucleus	-0.468	none
**TaMAPKKK8**			TraesCS4B02G398400.3	672296531	672306045	744	82.04	6.52	Nucleus	-0.471	none
**TaMAPKKK9**			TraesCS5A02G187400.1	389131980	389133955	518	56.50	5.80	Nucleus	-0.191	none
**TaMAPKKK10**			TraesCS5A02G392500.1	588731002	588738966	825	89.69	5.31	Nucleus	-0.504	none
**TaMAPKKK11**			TraesCS5A02G556400.5	707802354	707810757	742	82.08	6.89	Nucleus	-0.448	none

**TaMAPKKK12**	TraesCS5B02G397300.1	574952105	574960822	858	93.17	5.31	Nucleus	-0.522	none
**TaMAPKKK13**	TraesCS5D02G203600.1	308863403	308865114	518	56.49	5.75	Nucleus	-0.201	none
**TaMAPKKK14**	TraesCS5D02G402300.1	468166467	468175209	822	89.42	5.24	Nucleus	-0.519	none
**TaMAPKKK15**	TraesCS6A02G149900.1	133414193	133419353	543	61.18	6.83	Nucleus	-0.552	none
**TaMAPKKK16**	TraesCS6A02G353400.1	585174093	585176268	348	38.89	8.97	Nucleus	-0.25	none
**TaMAPKKK17**	TraesCS6A02G353500.1	585187497	585195069	672	74.09	6.39	Nucleus	-0.444	none
**TaMAPKKK18**	TraesCS6B02G177800.1	195805196	195810462	542	61.16	7.02	Nucleus	-0.579	none
**TaMAPKKK19**	TraesCS6B02G386100.1	660664103	660676384	695	76.96	6.31	Nucleus	-0.415	none
**TaMAPKKK20**	TraesCS6D02G335800.1	436870500	436887069	695	76.92	6.26	Nucleus	-0.413	none
**TaMAPKKK21**	TraesCS6D02G139200.1	108447173	108452128	509	57.60	6.71	Nucleus	-0.504	none
**TaMAPKKK22**	TraesCS7A02G232300.1	203511879	203521492	700	77.96	6.18	Nucleus	-0.559	none
**TaMAPKKK23**	TraesCS7B02G130700.1	157674223	157684150	700	77.91	6.11	Nucleus	-0.578	none

**TaMAPKK KK24**	TraesCS7D02G232400.1	193922055	193930244	709	78.76	5.99	Nucleus	-0.417	none
**TaMAPKK KK25**	TraesCSU02G115300.1	99866931	99876736	741	81.70	6.34	Nucleus	-0.486	none

1(A,B,D)chromosomes	**TaMAPK41**			TraesCS1A02G402400.2
	**TaMAPK42**			TraesCS1A02G415300.1
	**TaMAPK43**			TraesCS1A02G421000.1
	**TaMAPK11**			TraesCS1A02G086500.1
	**TaMAPK14**			TraesCS1A02G184500.1
	**TaMAPK27**			TraesCS1B02G104900.1
	**TaMAPK28**			TraesCS1B02G192600.3
	**TaMAPK29**			TraesCS1B02G431400.2
	**TaMAPK4**			TraesCS1D02G088000.2
	**TaMAPK5**			TraesCS1D02G422800.1
	**TaMAPK33**			TraesCS1D02G410100.1
	**TaMAPK34**			TraesCS1D02G428900.1

3(A,B,D)chromosomes	**TaMAPK39**			TraesCS3A02G242100.1
	**TaMAPK40**			TraesCS3A02G231700.1
	**TaMAPK16**			TraesCS3B02G270200.1
	**TaMAPK38**			TraesCS3B02G256700.1
	**TaMAPK8**			TraesCS3D02G225600.1
	**TaMAPK23**			TraesCS3D02G221700.1
	**TaMAPK24**			TraesCS3D02G242200.2

4(A,B,D)chromosomes	**TaMAPK31**			TraesCS4A02G434800.1
	**TaMAPK2**			TraesCS4A02G336800.2
	**TaMAPK3**			TraesCS4A02G106400.1
	**TaMAPK25**			TraesCS4D02G198600.1

5(A,B,D)chromosomes	**TaMAPK50**			TraesCS5B02G536500.1
	**TaMAPK26**			TraesCS5D02G534000.2

6(A,B,D)chromosomes	**TaMAPK35**			TraesCS6A02G099600.1
	**TaMAPK36**			TraesCS6A02G118100.1
	**TaMAPK37**			TraesCS6A02G269400.1
	**TaMAPK17**			TraesCS6B02G127800.1

6(A,B,D)chromosomes	**TaMAPK1**			TraesCS6B02G296700.2
	**TaMAPK10**			TraesCS6B02G146300.1
	**TaMAPK13**			TraesCS6D02G245500.3
	**TaMAPK44**			TraesCS6D02G082900.2

7(A,B,D)chromosomes	**TaMAPK19**			TraesCS7A02G111300.1
	**TaMAPK12**			TraesCS7A02G422500.1
	**TaMAPK30**			TraesCS7A02G335300.2
	**TaMAPK46**			TraesCS7A02G049000.1
	**TaMAPK47**			TraesCS7A02G029700.1
	**TaMAPK49**			TraesCS7A02G410700.2
	**TaMAPK52**			TraesCS7B02G246900.3
	**TaMAPK53**			TraesCS7B02G309900.1
	**TaMAPK54**			TraesCS7B02G322900.1
	**TaMAPK6**			TraesCS7B02G009200.1
	**TaMAPK7**			TraesCS7D02G342800.3
	**TaMAPK18**			TraesCS7D02G044100.1
	**TaMAPK20**			TraesCS7D02G403700.1
	**TaMAPK22**			TraesCS7D02G414900.1

3(A,B,D)chromosomes	**TaMAPKK9**			TraesCS3B02G066300.1

4(A,B,D)chromosomes	**TaMAPKK14**			TraesCS4A02G265900.1
	**TaMAPKK15**			TraesCS4A02G266000.1
	**TaMAPKK16**			TraesCS4A02G266100.1
	**TaMAPKK17**			TraesCS4A02G266200.1
	**TaMAPKK5**			TraesCS4B02G049000.1
	**TaMAPKK6**			TraesCS4B02G048100.1
	**TaMAPKK7**			TraesCS4B02G048600.1
	**TaMAPKK8**			TraesCS4B02G048900.1
	**TaMAPKK11**			TraesCS4D02G048800.1
	**TaMAPKK12**			TraesCS4D02G048500.1

5(A,B,D)chromosomes	**TaMAPKK4**			TraesCS5A02G122700.4
	**TaMAPKK2**			TraesCS5B02G565100.3
	**TaMAPKK18**			TraesCS5B02G122600.1
	**TaMAPKK3**			TraesCS5D02G130900.2
	**TaMAPKK13**			TraesCS5D02G549600.1
6(A,B,D)chromosomes	**TaMAPKK1**			TraesCS6D02G328800.1
2(A,B,D)chromosomes	**TaMAPKKK5**	**MEKK**	TaMEKK5	TraesCS2A02G199700.1
	**TaMAPKKK1**		TaMEKK1	TraesCS2A02G407600.1
	**TaMAPKKK28**		TaMEKK28	TraesCS2A02G095300.1
	**TaMAPKKK20**		TaMEKK20	TraesCS2A02G498000.3
	**TaMAPKKK27**		TaMEKK27	TraesCS2B02G110500.1
	**TaMAPKKK18**		TaMEKK18	TraesCS2B02G526200.3
	**TaMAPKKK26**		TaMEKK26	TraesCS2D02G093700.1
3(A,B,D)chromosomes	**TaMAPKKK7**		TaMEKK7	TraesCS3B02G289500.1
	**TaMAPKKK8**		TaMEKK8	TraesCS3B02G288100.1
	**TaMAPKKK9**		TaMEKK9	TraesCS3B02G288300.1
4(A,B,D)chromosomes	**TaMAPKKK12**		TaMEKK12	TraesCS4A02G093800.2
	**TaMAPKKK3**		TaMEKK3	TraesCS4B02G210600.2
	**TaMAPKKK2**		TaMEKK2	TraesCS4D02G027600.1
	**TaMAPKKK10**		TaMEKK10	TraesCS4D02G211300.2
5(A,B,D)chromosomes	**TaMAPKKK17**		TaMEKK17	TraesCS5A02G200800.1
	**TaMAPKKK22**		TaMEKK22	TraesCS5A02G392500.1
	**TaMAPKKK14**		TaMEKK14	TraesCS5A02G118200.1
	**TaMAPKKK15**		TaMEKK15	TraesCS5A02G463100.2
	**TaMAPKKK16**		TaMEKK16	TraesCS5B02G474500.1
	**TaMAPKKK24**		TaMEKK24	TraesCS5B02G199400.1

	**TaMAPKKK25**		TaMEKK25	TraesCS5B02G196400.1
	**TaMAPKKK11**		TaMEKK11	TraesCS5D02G475900.1
	**TaMAPKKK29**		TaMEKK29	TraesCS5D02G206500.1
6(A,B,D)chromosomes	**TaMAPKKK21**		TaMEKK21	TraesCS6A02G149900.1
	**TaMAPKKK4**		TaMEKK4	TraesCS6A02G245000.3
	**TaMAPKKK4-1**		TaMEKK4-1	TraesCS6B02G279300.1
	**TaMAPKKK23**		TaMEKK23	TraesCS6D02G139200.1
1(A,B,D)chromosomes	**TaMAPKKK38**	**ZIK**	TaZIK9	TraesCS1D02G026200.2
2(A,B,D)chromosomes	**TaMAPKKK32**		TaZIK3	TraesCS2A02G195900.2
	**TaMAPKKK36**		TaZIK7	TraesCS2B02G223600.1
	**TaMAPKKK37**		TaZIK8	TraesCS2B02G216800.1
	**TaMAPKKK34**		TaZIK5	TraesCS2D02G197600.1
5(A,B,D)chromosomes	**TaMAPKKK40**		TaZIK11	TraesCS5B02G146100.1
	**TaMAPKKK30**		TaZIK1	TraesCS5D02G145100.1
6(A,B,D)chromosomes	**TaMAPKKK39**		TaZIK10	TraesCS6A02G255100.2
	**TaMAPKKK33**		TaZIK4	TraesCS6B02G270400.1
	**TaMAPKKK31**		TaZIK2	TraesCS6D02G236400.1
1(A,B,D)chromosomes	**TaMAPKKK148**	**Raf**	**TaRaf108**	TraesCS1A02G003900.1
	**TaMAPKKK88**		**TaRaf48**	TraesCS1A02G422800.1
	**TaMAPKKK53**		**TaRaf13**	TraesCS1B02G372400.1
	**TaMAPKKK104-1**		**TaRaf64-1**	TraesCS1B02G283400.1
	**TaMAPKKK128**		**TaRaf88**	TraesCS1B02G446500.1
	**TaMAPKKK116**		**TaRaf76**	TraesCS1B02G454000.2
	**TaMAPKKK104**		**TaRaf64**	TraesCS1D02G273600.1
	**TaMAPKKK66**		**TaRaf26**	TraesCS1D02G273800.2
	**TaMAPKKK67**		**TaRaf27**	TraesCS1D02G360600.1
	**TaMAPKKK68**		**TaRaf28**	TraesCS1D02G431400.1

	**TaMAPKKK70**	**TaRaf30**	TraesCS1D02G423800.1
	**TaMAPKKK133**	**TaRaf93**	TraesCS1D02G004300.1

2(A,B,D)chromosomes	**TaMAPKKK119**	**TaRaf79**	TraesCS2A02G577000.1
	**TaMAPKKK80**	**TaRaf40**	TraesCS2A02G217600.1
	**TaMAPKKK59**	**TaRaf19**	TraesCS2A02G216900.1
	**TaMAPKKK60**	**TaRaf20**	TraesCS2A02G217000.1
	**TaMAPKKK147**	**TaRaf107**	TraesCS2A02G216600.1
	**TaMAPKKK142**	**TaRaf102**	TraesCS2A02G032200.1
	**TaMAPKKK122**	**TaRaf82**	TraesCS2A02G214000.1
	**TaMAPKKK145**	**TaRaf105**	TraesCS2B02G242300.1
	**TaMAPKKK146**	**TaRaf106**	TraesCS2B02G241400.1
	**TaMAPKKK136**	**TaRaf96**	TraesCS2B02G241600.1
	**TaMAPKKK69**	**TaRaf29**	TraesCS2D02G588200.1
	**TaMAPKKK135**	**TaRaf95**	TraesCS2D02G598800.1
	**TaMAPKKK44**	**TaRaf4**	TraesCS2D02G003900.1
	**TaMAPKKK46**	**TaRaf6**	TraesCS2D02G050700.1
	**TaMAPKKK109**	**TaRaf69**	TraesCS2D02G219800.1
	**TaMAPKKK121**	**TaRaf81**	TraesCS2D02G066900.1

	**TaMAPKKK111**	**TaRaf71**	TraesCS3A02G315100.1
	**TaMAPKKK105**	**TaRaf65**	TraesCS3A02G045200.1
	**TaMAPKKK99**	**TaRaf59**	TraesCS3A02G001500.1
	**TaMAPKKK100**	**TaRaf60**	TraesCS3A02G274000.1
	**TaMAPKKK115**	**TaRaf75**	TraesCS3A02G229800.1
	**TaMAPKKK79**	**TaRaf39**	TraesCS3A02G493900.1
	**TaMAPKKK72**	**TaRaf32**	TraesCS3A02G003900.1
	**TaMAPKKK73**	**TaRaf33**	TraesCS3A02G039200.1
	**TaMAPKKK74**	**TaRaf34**	TraesCS3A02G039400.1
	**TaMAPKKK75**	**TaRaf35**	TraesCS3A02G096500.1
	**TaMAPKKK76**	**TaRaf36**	TraesCS3A02G246100.1
	**TaMAPKKK56**	**TaRaf16**	TraesCS3A02G039100.1
	**TaMAPKKK139**	**TaRaf99**	TraesCS3A02G493500.1

3(A,B,D)chromosomes	**TaMAPKKK150**	**TaRaf110**	TraesCS3B02G008600.2
	**TaMAPKKK151**	**TaRaf111**	TraesCS3B02G123800.1
	**TaMAPKKK152**	**TaRaf112**	TraesCS3B02G259100.1
	**TaMAPKKK153**	**TaRaf113**	TraesCS3B02G351800.1
	**TaMAPKKK154**	**TaRaf114**	TraesCS3B02G478400.1
	**TaMAPKKK43**	**TaRaf3**	TraesCS3B02G110300.1
	**TaMAPKKK58**	**TaRaf18**	TraesCS3B02G259800.1
	**TaMAPKKK144**	**TaRaf104**	TraesCS3D02G501100.1
	**TaMAPKKK137**	**TaRaf97**	TraesCS3D02G472000.1
	**TaMAPKKK45**	**TaRaf5**	TraesCS3D02G273200.1
	**TaMAPKKK57**	**TaRaf17**	TraesCS3D02G040600.1
	**TaMAPKKK78**	**TaRaf38**	TraesCS3D02G501400.1
	**TaMAPKKK113**	**TaRaf73**	TraesCS3D02G108500.1
	**TaMAPKKK117**	**TaRaf77**	TraesCS3D02G097000.1
	**TaMAPKKK108**	**TaRaf68**	TraesCS3D02G023600.1
4(A,B,D)chromosomes	**TaMAPKKK106**	**TaRaf66**	TraesCS4A02G456900.1
	**TaMAPKKK62**	**TaRaf22**	TraesCS4A02G313900.1
	**TaMAPKKK63**	**TaRaf23**	TraesCS4A02G383000.1
	**TaMAPKKK64**	**TaRaf24**	TraesCS4A02G465900.2
	**TaMAPKKK64-1**	**TaRaf24-1**	TraesCS4A02G464700.1
	**TaMAPKKK65**	**TaRaf25**	TraesCS4A02G465000.1
	**TaMAPKKK91**	**TaRaf51**	TraesCS4D02G089300.1
5(A,B,D)chromosomes	**TaMAPKKK81**	**TaRaf41**	TraesCS5A02G292500.1
	**TaMAPKKK83**	**TaRaf43**	TraesCS5A02G351500.1
	**TaMAPKKK84**	**TaRaf44**	TraesCS5A02G351000.1
	**TaMAPKKK85**	**TaRaf45**	TraesCS5A02G352000.1
	**TaMAPKKK92**	**TaRaf52**	TraesCS5B02G337300.1
	**TaMAPKKK94**	**TaRaf54**	TraesCS5B02G012000.1
	**TaMAPKKK95**	**TaRaf55**	TraesCS5B02G204900.1
	**TaMAPKKK96**	**TaRaf56**	TraesCS5B02G292000.2
	**TaMAPKKK97**	**TaRaf57**	TraesCS5B02G353800.1

5(A,B,D)chromosomes	**TaMAPKKK132**	**TaRaf92**	TraesCS5B02G353600.1
	**TaMAPKKK98**	**TaRaf58**	TraesCS5D02G482000.1
	**TaMAPKKK101**	**TaRaf61**	TraesCS5D02G019400.1
	**TaMAPKKK93**	**TaRaf53**	TraesCS5D02G018800.1
	**TaMAPKKK86**	**TaRaf46**	TraesCS5D02G386800.1
	**TaMAPKKK123**	**TaRaf83**	TraesCS5D02G358700.1
	**TaMAPKKK123-1**	**TaRaf83-1**	TraesCS5D02G358200.1
	**TaMAPKKK134**	**TaRaf94**	TraesCS5D02G547500.1
	**TaMAPKKK140**	**TaRaf100**	TraesCS5D02G097900.1
6(A,B,D)chromosomes	**TaMAPKKK71**	**TaRaf31**	TraesCS6A02G004500.1
	**TaMAPKKK41**	**TaRaf1**	TraesCS6A02G172600.1
	**TaMAPKKK155**	**TaRaf115**	TraesCS6B02G217100.1
	**TaMAPKKK131**	**TaRaf91**	TraesCS6B02G215100.2
	**TaMAPKKK129**	**TaRaf89**	TraesCS6B02G320800.1
	**TaMAPKKK127**	**TaRaf87**	TraesCS6D02G339600.1
7(A,B,D)chromosomes	**TaMAPKKK90**	**TaRaf50**	TraesCS7A02G152100.1
	**TaMAPKKK130**	**TaRaf90**	TraesCS7A02G044600.1
	**TaMAPKKK103**	**TaRaf63**	TraesCS7A02G326700.1
	**TaMAPKKK107**	**TaRaf67**	TraesCS7A02G032700.1
	**TaMAPKKK102**	**TaRaf62**	TraesCS7D02G474700.1
	**TaMAPKKK47**	**TaRaf7**	TraesCS7D02G022200.1
	**TaMAPKKK48**	**TaRaf8**	TraesCS7D02G079100.1
	**TaMAPKKK50**	**TaRaf10**	TraesCS7D02G099200.2
	**TaMAPKKK51**	**TaRaf11**	TraesCS7D02G230200.1
	**TaMAPKKK52**	**TaRaf12**	TraesCS7D02G230500.1
	**TaMAPKKK61**	**TaRaf21**	TraesCS7D02G384700.1
	**TaMAPKKK125**	**TaRaf85**	TraesCS7D02G153800.1
	**TaMAPKKK54**	**TaRaf14**	TraesCS7D02G503600.1
	**TaMAPKKK141**	**TaRaf101**	TraesCS7D02G000800.1
	**TaMAPKKK82**	**TaRaf42**	TraesCSU02G072500.1

Un	**TaMAPKKK42**		**TaRaf2**	TraesCSU02G203100.1
	**TaMAPKKK126**		**TaRaf86**	TraesCSU02G011500.1

1(A,B,D)chromosomes	**TaMAPKKKK1**			TraesCS1A02G181900.1
	**TaMAPKKKK2**			TraesCS1B02G199100.2
	**TaMAPKKKK3**			TraesCS1D02G185000.2

2(A,B,D)chromosomes	**TaMAPKKKK4**			TraesCS2A02G233400.1
	**TaMAPKKKK5**			TraesCS2B02G249900.1
	**TaMAPKKKK6**			TraesCS2D02G232200.1

4(A,B,D)chromosomes	**TaMAPKKKK7**			TraesCS4B02G395600.1
	**TaMAPKKKK8**			TraesCS4B02G398400.3

5(A,B,D)chromosomes	**TaMAPKKKK9**			TraesCS5A02G187400.1
	**TaMAPKKKK10**			TraesCS5A02G392500.1
	**TaMAPKKKK11**			TraesCS5A02G556400.5
	**TaMAPKKKK12**			TraesCS5B02G397300.1
	**TaMAPKKKK13**			TraesCS5D02G203600.1
	**TaMAPKKKK14**			TraesCS5D02G402300.1

6(A,B,D)chromosomes	**TaMAPKKKK15**			TraesCS6A02G149900.1
	**TaMAPKKKK16**			TraesCS6A02G353400.1
	**TaMAPKKKK17**			TraesCS6A02G353500.1
	**TaMAPKKKK18**			TraesCS6B02G177800.1
	**TaMAPKKKK19**			TraesCS6B02G386100.1
	**TaMAPKKKK20**			TraesCS6D02G335800.1
	**TaMAPKKKK21**			TraesCS6D02G139200.1

7(A,B,D)chromosomes	**TaMAPKKKK22**			TraesCS7A02G232300.1
	**TaMAPKKKK23**			TraesCS7B02G130700.1
	**TaMAPKKKK24**			TraesCS7D02G232400.1
	**TaMAPKKKK25**			TraesCSU02G115300.1

RefSeq version			IWGSC version		TGACv1 version	
**TaMAPK1**		TraesCS6B02G296700.2	**TaMAPK1**	Traes_6BL_BF59BFB93.2		
**TaMAPK2**		TraesCS4A02G336800.2	**TaMAPK2**	Traes_4AL_6F3D0ACCA.1		
**TaMAPK3**		TraesCS4A02G106400.1	**TaMAPK3**	Traes_7AL_F5620757F.2	**TaMAPK3**	TRIAE_CS42_4AS_TGACv1_306486_AA1008960/4BL_TGACv1_320270_AA1033300/4DL_TGACv1_344691_AA1148960
**TaMAPK4**		TraesCS1D02G088000.2	**TaMAPK4**	Traes_1DS_66BD773BF.2	**TaMAPK4**	TRIAE_CS42_1AL_TGACv1_000118_AA0003960/1BL_TGACv1_030274_AA0084790/1DL_TGACv1_061983_AA0206840
**TaMAPK5**		TraesCS1D02G422800.1	**TaMAPK5**	Traes_1DL_52D511CDD.1		
**TaMAPK6**		TraesCS7B02G009200.1	**TaMAPK6**	Traes_1BL_7FA80EF00.2	**TaMAPK6**	TRIAE_CS42_7AS_TGACv1_570369_AA1834630/7BS_TGACv1_593766_AA1954300/7DS_TGACv1_622461_AA2040040
**TaMAPK7**		TraesCS7D02G342800.3	**TaMAPK7**	Traes_7DL_FE0ECD387.2	**TaMAPK7**	TRIAE_CS42_7AL_TGACv1_558385_AA1792950/7BL_TGACv1_576902_AA1859240/7DL_TGACv1_603035_AA1974260/7DL_TGACv1_603254_AA1979200
**TaMAPK8**		TraesCS3D02G225600.1	**TaMAPK8**	Traes_3DL_5D82311EA.1		
**TaMAPK10**		TraesCS6B02G146300.1	**TaMAPK10**	Traes_6BS_17C1E5829.1		
**TaMAPK11**		TraesCS1A02G086500.1	**TaMAPK11**	Traes_1AS_C9CFD7AC8.1	**TaMAPK11**	TRIAE_CS42_7AL_TGACv1_555982_AA1751700/7BL_TGACv1_580661_AA1914980/7DL_TGACv1_603600_AA1986180
**TaMAPK12**		TraesCS7A02G422500.1	**TaMAPK12**	Traes_7AL_4AF13CC8B.2		
**TaMAPK13**		TraesCS6D02G245500.3	**TaMAPK13**	Traes_6DL_F48A5E31E.2		
**TaMAPK14**		TraesCS1A02G184500.1	**TaMAPK14**	Traes_1AL_89DDB4243.1	**TaMAPK14**	TRIAE_CS42_6AS_TGACv1_487866_AA1573320/6BS_TGACv1_514340_AA1658600/6DS_TGACv1_544171_AA1747030
**TaMAPK16**		TraesCS3B02G270200.1	**TaMAPK16**	TRAES3BF058500020CFD	**TaMAPK16**	TRIAE_CS42_1AS_TGACv1_020601_AA0078640/1BS_TGACv1_049733_AA0160590/U_TGACv1_642759_AA2122920

**TaMAPK17**		TraesCS6B02G127800.1	**TaMAPK17**	Traes_6BS_2D0054D1F.2	**TaMAPK17**	TRIAE_CS42_7AL_TGACv1_556562_AA1765430/U_TGACv1_641980_AA2108810/7DL_TGACv1_604680_AA2000630
**TaMAPK18**		TraesCS7D02G044100.1	**TaMAPK18**	Traes_7DS_C268073F4.3		
**TaMAPK19**		TraesCS7A02G111300.1	**TaMAPK19**	Traes_7DS_1D8A8BFA2.1		
**TaMAPK20**		TraesCS7D02G403700.1	**TaMAPK20**	Traes_7DL_FB75EA9C3.2	**TaMAPK20**	TaMAPK20-1: TRIAE_CS42_3AL_TGACv1_194002_AA0624350/3B_TGACv1_224428_AA0796670/3DL_TGACv1_253300_AA0894090. TaMAPK20-2: TRIAE_CS42_1AL_TGACv1_002534_AA0042880/1BL_TGACv1_030959_AA0104540/1DL_TGACv1_063491_AA0227870. TaMAPK20-3: TRIAE_CS42_7AL_TGACv1_557823_AA1786610/7BL_TGACv1_577050_AA1864020/7DL_TGACv1_604962_AA2003380. TaMAPK20-4: TRIAE_CS42_3AL_TGACv1_195023_AA0643580/3B_TGACv1_225288_AA0806810/3DL_TGACv1_250331_AA0866300. TaMAPK20-5: TRIAE_CS42_1AL_TGACv1_001596_AA0032670/1BL_TGACv1_032512_AA0130820/U_TGACv1_643598_AA2133390
**TaMAPK22**		TraesCS7D02G414900.1	**TaMAPK22**	Traes_7DL_73DF29BF0.1		
**TaMAPK23**		TraesCS3D02G221700.1	**TaMAPK23**	Traes_3DL_281B6BCBF.2		
**TaMAPK24**		TraesCS3D02G242200.2	**TaMAPK24**	Traes_3DL_38B762939.1	**TaMAPK24**	TRIAE_CS42_U_TGACv1_640751_AA2072490 & AA2072480/BS_TGACv1_513631_AA1646180/6DS_TGACv1_543835_AA1744530
**TaMAPK25**		TraesCS4D02G198600.1	**TaMAPK25**	Traes_4DL_15045954F.1	**TaMAPK25**	TRIAE_CS42_5AL_TGACv1_375843_AA1227850/5BL_TGACv1_405765_AA1334990
**TaMAPK26**		TraesCS5D02G534000.2	**TaMAPK26**	Traes_5DL_8DC610F26.1		

**TaMAPK27**		TraesCS1B02G104900.1	**TaMAPK27**	Traes_1BS_26C55B2B1.1		
**TaMAPK28**		TraesCS1B02G192600.3	**TaMAPK28**	Traes_1BL_C5CD09285.1		
**TaMAPK29**		TraesCS1B02G431400.2	**TaMAPK29**	Traes_1BL_9360D8CEC.1		
**TaMAPK30**		TraesCS7A02G335300.2	**TaMAPK30**	Traes_4AS_5015DF7A2.1		
**TaMAPK31**		TraesCS4A02G434800.1	**TaMAPK31**	Traes_4AL_E432524A0.2		
**TaMAPK33**		TraesCS1D02G410100.1	**TaMAPK33**	Traes_1DL_8C6B737E9.1		
**TaMAPK34**		TraesCS1D02G428900.1	**TaMAPK34**	Traes_1DL_73AAC8631.1		
**TaMAPK35**		TraesCS6A02G099600.1	**TaMAPK35**	Traes_6AS_50BE5D59F.1		
**TaMAPK36**		TraesCS6A02G118100.1	**TaMAPK36**	Traes_6AS_8225741A6.2		
**TaMAPK37**		TraesCS6A02G269400.1	**TaMAPK37**	Traes_6AL_BE97161B9.2		
**TaMAPK38**		TraesCS3B02G256700.1	**TaMAPK38**	Traes_3AL_F88B0A8E6.1		
**TaMAPK39**		TraesCS3A02G242100.1	**TaMAPK39**	Traes_3AL_669FE0293.1		
**TaMAPK40**		TraesCS3A02G231700.1	**TaMAPK40**	Traes_3AL_532CA9EE7.1		
**TaMAPK41**		TraesCS1A02G402400.2	**TaMAPK41**	Traes_1AL_30C4B017F.1		
**TaMAPK42**		TraesCS1A02G415300.1	**TaMAPK42**	Traes_1AL_14DCD6020.1		
**TaMAPK43**		TraesCS1A02G421000.1	**TaMAPK43**	Traes_1AL_A7173BBAE.1		
**TaMAPK44**		TraesCS6D02G082900.2	**TaMAPK44**	Traes_6DS_6424C38F2.2		

**TaMAPK45**		TraesCS6D02G108100.1	**TaMAPK45**	Traes_6DS_A61864DB2.2		
**TaMAPK46**		TraesCS7A02G049000.1	**TaMAPK46**	Traes_7AS_E1135D559.1		
**TaMAPK47**		TraesCS7A02G029700.1	**TaMAPK47**	Traes_7AS_71B4C13A6.1		
**TaMAPK49**		TraesCS7A02G410700.2	**TaMAPK49**	Traes_7AL_81A545A54.2		
**TaMAPK50**		TraesCS5B02G536500.1	**TaMAPK50**	Traes_5BL_A39EC2FCD.1		
**TaMAPK52**		TraesCS7B02G246900.3	**TaMAPK52**	Traes_7BL_44A22C7FC.2		
**TaMAPK53**		TraesCS7B02G309900.1	**TaMAPK53**	Traes_7BL_992E1F443.4		
**TaMAPK54**		TraesCS7B02G322900.1	**TaMAPK54**	Traes_7BL_95B1705A2.2		
**TaMAPKK1**		TraesCS6D02G328800.1	**TaMAPKK1**	Traes_6DL_8D2D914D7.2	**TaMKK1**	TaMKK1-1: TRIAE_CS42_4AL_TGACv1_290148_AA0982230/7AS_TGACv1_570427_AA1835590/7DS_TGACv1_622118_AA2033130. TaMKK1-2: TRIAE_CS42_6AS_TGACv1_485693_AA1550190/6BS_TGACv1_513382_AA1639520/U_TGACv1_644387_AA2139180. TaMKK1-3: TRIAE_CS42_7DS_TGACv1_623511_AA2053970
**TaMAPKK2**		TraesCS5B02G565100.3	**TaMAPKK2**	Traes_5BL_BB574E77D.2		
**TaMAPKK3**		TraesCS5D02G130900.2	**TaMAPKK3**	Traes_5DL_0886CB561.2	**TaMKK3**	TaMKK3-1: TRIAE_CS42_3B_TGACv1_228257_AA0826360. TaMKK3-2: TRIAE_CS42_5BL_TGACv1_405566_AA1330480/5DL_TGACv1_432926_AA1394740. TaMKK3-3: TRIAE_CS42_5AL_TGACv1_373997_AA1187200/5BL_TGACv1_405070_AA1319320/5DL_TGACv1_435057_AA1445420
**TaMAPKK4**		TraesCS5A02G122700.4	**TaMAPKK4**	Traes_5AL_5EFCEDAFB.2	**TaMKK4**	TRIAE_CS42_6AL_TGACv1_472053_AA1517260/6BL_TGACv1_502626_AA1625820/6DL_TGACv1_528809_AA1716310

**TaMAPKK5**		TraesCS4B02G049000.1	**TaMAPKK5**	Traes_4BS_EE048CC01.1	**TaMKK5**	TRIAE_CS42_7AS_TGACv1_572257_AA1851770/7BS_TGACv1_592906_AA1946090/7DS_TGACv1_623332_AA2052160
**TaMAPKK6**		TraesCS4B02G048100.1	**TaMAPKK6**	Traes_4BS_59F3F68D1.1	**TaMKK6**	TRIAE_CS42_4AL_TGACv1_290485_AA0985860/4BL_TGACv1_320624_AA1044950/4DL_TGACv1_342786_AA1122060
**TaMAPKK7**		TraesCS4B02G048600.1	**TaMAPKK7**	Traes_4BS_84D913A2E.1		
**TaMAPKK8**		TraesCS4B02G048900.1	**TaMAPKK8**	Traes_4BS_BDB56BC90.1		
**TaMAPKK9**		TraesCS3B02G066300.1	**TaMAPKK9**	TRAES3BF024700110CFD		
**TaMAPKK11**		TraesCS4D02G048800.1	**TaMAPKK11**	Traes_4DS_7F2F2671B.2		
**TaMAPKK12**		TraesCS4D02G048500.1	**TaMAPKK12**	Traes_4DS_7A016C2E4.1		
**TaMAPKK13**		TraesCS5D02G549600.1	**TaMAPKK13**	Traes_5DL_F89F21E65.1		
**TaMAPKK14**		TraesCS4A02G265900.1	**TaMAPKK14**	Traes_4AL_75E7BE9EE.1		
**TaMAPKK15**		TraesCS4A02G266000.1	**TaMAPKK15**	Traes_4AL_429683D72.1		
**TaMAPKK16**		TraesCS4A02G266100.1	**TaMAPKK16**	Traes_4AL_B7C432896.2		
**TaMAPKK17**		TraesCS4A02G266200.1	**TaMAPKK17**	Traes_4AL_84DFF6A541.1		
**TaMAPKK18**		TraesCS5B02G122600.1	**TaMAPKK18**	Traes_5BL_EC9896AB3.2		
**TaMAPKKK1**	TaMEKK1	TraesCS2A02G407600.1	TaMEKK1	Traes_2BL_23D01E7F4		
**TaMAPKKK2**	TaMEKK2	TraesCS4D02G027600.1	TaMEKK2	Traes_4DS_63F7CF3CE		
**TaMAPKKK3**	TaMEKK3	TraesCS4B02G210600.2	TaMEKK3	Traes_4BL_A7AE389EE		
**TaMAPKKK4**	TaMEKK4	TraesCS6A02G245000.3	TaMEKK4	Traes_6BL_93505FEAF		

**TaMAPKKK4-1**	TaMEKK4-1	TraesCS6B02G279300.1				
**TaMAPKKK5**	TaMEKK5	TraesCS2A02G199700.1	TaMEKK5	Traes_2AS_6DA49285E		
**TaMAPKKK7**	TaMEKK7	TraesCS3B02G289500.1	TaMEKK7	TRAES3BF169900020CFD_g		
**TaMAPKKK8**	TaMEKK8	TraesCS3B02G288100.1	TaMEKK8	TRAES3BF036800120CFD_g		
**TaMAPKKK9**	TaMEKK9	TraesCS3B02G288300.1	TaMEKK9	TRAES3BF036800100CFD_g		
**TaMAPKKK10**	TaMEKK10	TraesCS4D02G211300.2	TaMEKK10	Traes_4DL_94E10E6EB		
**TaMAPKKK11**	TaMEKK11	TraesCS5D02G475900.1	TaMEKK11	Traes_5DL_ADFFAE33D		
**TaMAPKKK12**	TaMEKK12	TraesCS4A02G093800.2	TaMEKK12	Traes_4AS_DF85CBD39		
**TaMAPKKK14**	TaMEKK14	TraesCS5A02G118200.1	TaMEKK14	Traes_5AS_9A8A9187C		
**TaMAPKKK15**	TaMEKK15	TraesCS5A02G463100.2	TaMEKK15	Traes_5AL_DEDF36AD2		
**TaMAPKKK16**	TaMEKK16	TraesCS5B02G474500.1	TaMEKK16	Traes_5BL_35A6B4387		
**TaMAPKKK17**	TaMEKK17	TraesCS5A02G200800.1	TaMEKK17	Traes_5AL_4D0919BA1		
**TaMAPKKK18**	TaMEKK18	TraesCS2B02G526200.3	TaMEKK18	Traes_2BL_84B12F4F8		
**TaMAPKKK20**	TaMEKK20	TraesCS2A02G498000.3	TaMEKK20	Traes_2AL_66079157A		
**TaMAPKKK21**	TaMEKK21	TraesCS6A02G149900.1	TaMEKK21	Traes_6AS_E690A27CA		
**TaMAPKKK22**	TaMEKK22	TraesCS5A02G392500.1	TaMEKK22	Traes_5AL_F9C2BEAF3		
**TaMAPKKK23**	TaMEKK23	TraesCS6D02G139200.1	TaMEKK23	Traes_6DS_185723D1E		

**TaMAPKKK24**	TaMEKK24	TraesCS5B02G199400.1	TaMEKK24	Traes_5BL_3EFFD8013		
**TaMAPKKK25**	TaMEKK25	TraesCS5B02G196400.1	TaMEKK25	Traes_5BL_38DB82ACF		
**TaMAPKKK26**	TaMEKK26	TraesCS2D02G093700.1	TaMEKK26	Traes_2DS_122AEE879		
**TaMAPKKK27**	TaMEKK27	TraesCS2B02G110500.1	TaMEKK27	Traes_2BS_8506C57C5		
**TaMAPKKK28**	TaMEKK28	TraesCS2A02G095300.1	TaMEKK28	Traes_2AS_F0521C4F2		
**TaMAPKKK29**	TaMEKK29	TraesCS5D02G206500.1	TaMEKK29	Traes_5DL_243735D6C		
**TaMAPKKK30**	TaZIK1	TraesCS5D02G145100.1	TaZIK1	Traes_5DL_9824E97A8		
**TaMAPKKK31**	TaZIK2	TraesCS6D02G236400.1	TaZIK2	Traes_6DL_F70F83614		
**TaMAPKKK32**	TaZIK3	TraesCS2A02G195900.2	TaZIK3	Traes_2AS_2B84A0A98		
**TaMAPKKK33**	TaZIK4	TraesCS6B02G270400.1	TaZIK4	Traes_6BL_4A17F7221		
**TaMAPKKK34**	TaZIK5	TraesCS2D02G197600.1	TaZIK5	Traes_2DS_AA3E486F3		
**TaMAPKKK36**	TaZIK7	TraesCS2B02G223600.1	TaZIK7	Traes_2BS_18264AA5C		
**TaMAPKKK37**	TaZIK8	TraesCS2B02G216800.1	TaZIK8	Traes_2BS_1E887CFE5		
**TaMAPKKK38**	TaZIK9	TraesCS1D02G026200.2	TaZIK9	Traes_1DS_34EFDA767		
**TaMAPKKK39**	TaZIK10	TraesCS6A02G255100.2	TaZIK10	Traes_6AL_48165ABE5		
**TaMAPKKK40**	TaZIK11	TraesCS5B02G146100.1	TaZIK11	Traes_5BL_4002B5518		
**TaMAPKKK41**	**TaRaf1**	TraesCS6A02G172600.1	TaRaf1	Traes_6DS_D8750EB5A		

**TaMAPKKK42**	**TaRaf2**	TraesCSU02 G203100.1	TaRaf2	Traes_2BL_4C AF2C184		
**TaMAPKKK43**	**TaRaf3**	TraesCS3B02 G110300.1	TaRaf3	Traes_6BL_01 E6CE316		
**TaMAPKKK44**	**TaRaf4**	TraesCS2D02 G003900.1	TaRaf4	Traes_2DS_DF E006BB6		
**TaMAPKKK45**	**TaRaf5**	TraesCS3D02 G273200.1	TaRaf5	Traes_3DL_CF CA7AA6B		
**TaMAPKKK46**	**TaRaf6**	TraesCS2D02 G050700.1	TaRaf6	Traes_2DS_0B FF3B23D		
**TaMAPKKK47**	**TaRaf7**	TraesCS7D02 G022200.1	TaRaf7	Traes_7DS_36 1EC0618		
**TaMAPKKK48**	**TaRaf8**	TraesCS7D02 G079100.1	TaRaf8	Traes_7DS_A3 EB5BFEB		
**TaMAPKKK50**	**TaRaf10**	TraesCS7D02 G099200.2	TaRaf10	Traes_7DS_D5 6FBFFD4		
**TaMAPKKK51**	**TaRaf11**	TraesCS7D02 G230200.1	TaRaf11	Traes_7DS_5A 97B2141		
**TaMAPKKK52**	**TaRaf12**	TraesCS7D02 G230500.1	TaRaf12	Traes_7DS_34 2F25C32		
**TaMAPKKK53**	**TaRaf13**	TraesCS1B02 G372400.1	TaRaf13	Traes_1BL_C9 B36DE76		
**TaMAPKKK54**	**TaRaf14**	TraesCS7D02 G503600.1	TaRaf14	Traes_7DL_F0 110933B		
**TaMAPKKK56**	**TaRaf16**	TraesCS3A02 G039100.1	TaRaf16	Traes_3DS_4E 61EE6EA		
**TaMAPKKK57**	**TaRaf17**	TraesCS3D02 G040600.1	TaRaf17	Traes_3DS_68 01BD0D2		
**TaMAPKKK58**	**TaRaf18**	TraesCS3B02 G259800.1	TaRaf18	Traes_3DL_B2 8036C5B		
**TaMAPKKK59**	**TaRaf19**	TraesCS2A02 G216900.1	TaRaf19	Traes_2AS_92 19695D6		
**TaMAPKKK60**	**TaRaf20**	TraesCS2A02 G217000.1	TaRaf20	Traes_2AS_79 A94F84A		

**TaMAPKKK61**	**TaRaf21**	TraesCS7D02 G384700.1	TaRaf21	Traes_7DL_70 5BA7CDD		
**TaMAPKKK62**	**TaRaf22**	TraesCS4A02 G313900.1	TaRaf22	Traes_4AL_1C 557F688		
**TaMAPKKK63**	**TaRaf23**	TraesCS4A02 G383000.1	TaRaf23	Traes_4AL_06 A8F8B8F		
**TaMAPKKK64**	**TaRaf24**	TraesCS4A02 G465900.2	TaRaf24	Traes_4AL_FE FC21AAB		
**TaMAPKKK64-1**	**TaRaf24-1**	TraesCS4A02 G464700.1				
**TaMAPKKK65**	**TaRaf25**	TraesCS4A02 G465000.1	TaRaf25	Traes_4AL_C2 17A20A1		
**TaMAPKKK66**	**TaRaf26**	TraesCS1D02 G273800.2	TaRaf26	Traes_1DL_FB 90601E7		
**TaMAPKKK67**	**TaRaf27**	TraesCS1D02 G360600.1	TaRaf27	Traes_1DL_F4 9D0E56A		
**TaMAPKKK68**	**TaRaf28**	TraesCS1D02 G431400.1	TaRaf28	Traes_1DL_A0 FB3E1D3		
**TaMAPKKK69**	**TaRaf29**	TraesCS2D02 G588200.1	TaRaf29	Traes_2DL_C5 A0BDC60		
**TaMAPKKK70**	**TaRaf30**	TraesCS1D02 G423800.1	TaRaf30	Traes_1DL_56 B195A26		
**TaMAPKKK71**	**TaRaf31**	TraesCS6A02 G004500.1	TaRaf31	Traes_6AS_00 6C344A3		
**TaMAPKKK72**	**TaRaf32**	TraesCS3A02 G003900.1	TaRaf32	Traes_3AS_A2 CECBF17		
**TaMAPKKK73**	**TaRaf33**	TraesCS3A02 G039200.1	TaRaf33	Traes_3AS_76 9E90DDD		
**TaMAPKKK74**	**TaRaf34**	TraesCS3A02 G039400.1	TaRaf34	Traes_3AS_5A F26B2FC		
**TaMAPKKK75**	**TaRaf35**	TraesCS3A02 G096500.1	TaRaf35	Traes_3AS_A5 42EC6F6		
**TaMAPKKK76**	**TaRaf36**	TraesCS3A02 G246100.1	TaRaf36	Traes_3AL_7F 6E774BB		

**TaMAPKKK78**	**TaRaf38**	TraesCS3D02G501400.1	TaRaf38	Traes_3AL_60BB7086F		
**TaMAPKKK79**	**TaRaf39**	TraesCS3A02G493900.1	TaRaf39	Traes_3AL_F384515F5		
**TaMAPKKK80**	**TaRaf40**	TraesCS2A02G217600.1	TaRaf40	Traes_2AS_0C8932B8E		
**TaMAPKKK81**	**TaRaf41**	TraesCS5A02G292500.1	TaRaf41	Traes_5AL_3FE725FD4		
**TaMAPKKK82**	**TaRaf42**	TraesCSU02G072500.1	TaRaf42	Traes_5AL_A236B0387		
**TaMAPKKK83**	**TaRaf43**	TraesCS5A02G351500.1	TaRaf43	Traes_5AL_CDD4A02E7		
**TaMAPKKK84**	**TaRaf44**	TraesCS5A02G351000.1	TaRaf44	Traes_5AL_13784C39B		
**TaMAPKKK85**	**TaRaf45**	TraesCS5A02G352000.1	TaRaf45	Traes_5AL_68C659562		
**TaMAPKKK86**	**TaRaf46**	TraesCS5D02G386800.1	TaRaf46	Traes_5AL_7B1C0342F		
**TaMAPKKK88**	**TaRaf48**	TraesCS1A02G422800.1	TaRaf48	Traes_1AL_C21696173		
**TaMAPKKK90**	**TaRaf50**	TraesCS7A02G152100.1	TaRaf50	Traes_7AS_81545C211		
**TaMAPKKK91**	**TaRaf51**	TraesCS4D02G089300.1	TaRaf51	Traes_4DS_7D8A5F90B		
**TaMAPKKK92**	**TaRaf52**	TraesCS5B02G337300.1	TaRaf52	Traes_5DL_3191490FE		
**TaMAPKKK93**	**TaRaf53**	TraesCS5D02G018800.1	TaRaf53	Traes_5BS_0B466F42F		
**TaMAPKKK94**	**TaRaf54**	TraesCS5B02G012000.1	TaRaf54	Traes_5BS_43731B6AC		
**TaMAPKKK95**	**TaRaf55**	TraesCS5B02G204900.1	TaRaf55	Traes_5BL_E44E042FD		
**TaMAPKKK96**	**TaRaf56**	TraesCS5B02G292000.2	TaRaf56	Traes_5BL_2DA8896EE		

**TaMAPKKK97**	**TaRaf57**	TraesCS5B02G353800.1	TaRaf57	Traes_5BL_11A7A1F5C		
**TaMAPKKK98**	**TaRaf58**	TraesCS5D02G482000.1	TaRaf58	Traes_5DL_294C4EDB3		
**TaMAPKKK99**	**TaRaf59**	TraesCS3A02G001500.1	TaRaf59	Traes_3AS_2A0765E10		
**TaMAPKKK100**	**TaRaf60**	TraesCS3A02G274000.1	TaRaf60	Traes_3AL_82306B917		
**TaMAPKKK101**	**TaRaf61**	TraesCS5D02G019400.1	TaRaf61	Traes_5DS_53F8C78FA		
**TaMAPKKK102**	**TaRaf62**	TraesCS7D02G474700.1	TaRaf62	Traes_7BL_46880A4FE		
**TaMAPKKK103**	**TaRaf63**	TraesCS7A02G326700.1	TaRaf63	Traes_7AL_9AD23808D		
**TaMAPKKK104**	**TaRaf64**	TraesCS1D02G273600.1	TaRaf64	Traes_1DL_0162A6BAC		
**TaMAPKKK104-1**	**TaRaf64-1**	TraesCS1B02G283400.1				
**TaMAPKKK105**	**TaRaf65**	TraesCS3A02G045200.1	TaRaf65	Traes_3AS_A0EA6D12C		
**TaMAPKKK106**	**TaRaf66**	TraesCS4A02G456900.1	TaRaf66	Traes_4AL_48E7FB1C6		
**TaMAPKKK107**	**TaRaf67**	TraesCS7A02G032700.1	TaRaf67	Traes_4AL_83D9333FE		
**TaMAPKKK108**	**TaRaf68**	TraesCS3D02G023600.1	TaRaf68	Traes_5DL_62B6846F6		
**TaMAPKKK109**	**TaRaf69**	TraesCS2D02G219800.1	TaRaf69	Traes_2DS_42A9CC22D		
**TaMAPKKK110**	**TaRaf70**	TraesCS4B02G289100.1	TaRaf70	Traes_4BL_3626CDB73		
**TaMAPKKK111**	**TaRaf71**	TraesCS3A02G315100.1	TaRaf71	Traes_3AL_5DC02A5FC		
**TaMAPKKK112**	**TaRaf72**	TraesCS5D02G359500.1	TaRaf72	Traes_5DL_0A74AE348		

**TaMAPKKK113**	**TaRaf73**	TraesCS3D02G108500.1	TaRaf73	Traes_3AS_C492FCE9A		
**TaMAPKKK115**	**TaRaf75**	TraesCS3A02G229800.1	TaRaf75	Traes_3AL_0187ECBAC		
**TaMAPKKK116**	**TaRaf76**	TraesCS1B02G454000.2	TaRaf76	Traes_1BL_1E2841006		
**TaMAPKKK117**	**TaRaf77**	TraesCS3D02G097000.1	TaRaf77	Traes_3DS_0B1914F50		
**TaMAPKKK119**	**TaRaf79**	TraesCS2A02G577000.1	TaRaf79	Traes_2AL_0E43EBBB6		
**TaMAPKKK120**	**TaRaf80**	TraesCS4A02G317600.1	TaRaf80	Traes_4AL_9601B9873		
**TaMAPKKK121**	**TaRaf81**	TraesCS2D02G066900.1	TaRaf81	Traes_2DS_964FA3D25		
**TaMAPKKK122**	**TaRaf82**	TraesCS2A02G214000.1	TaRaf82	Traes_2AS_DCD2F10331		
**TaMAPKKK123**	**TaRaf83**	TraesCS5D02G358700.1	TaRaf83	Traes_5DL_A367964F5		
**TaMAPKKK123-1**	**TaRaf83-1**	TraesCS5D02G358200.1				
**TaMAPKKK125**	**TaRaf85**	TraesCS7D02G153800.1	TaRaf85	Traes_7DS_81C827CE6		
**TaMAPKKK126**	**TaRaf86**	TraesCSU02G011500.1	TaRaf86	Traes_6BS_511AB47D71		
**TaMAPKKK127**	**TaRaf87**	TraesCS6D02G339600.1	TaRaf87	Traes_6DL_7662129AC		
**TaMAPKKK128**	**TaRaf88**	TraesCS1B02G446500.1	TaRaf88	Traes_1BL_CDC566E72		
**TaMAPKKK129**	**TaRaf89**	TraesCS6B02G320800.1	TaRaf89	Traes_6BL_658AE8589		
**TaMAPKKK130**	**TaRaf90**	TraesCS7A02G044600.1	TaRaf90	Traes_7AS_0BE0D89AC		
**TaMAPKKK131**	**TaRaf91**	TraesCS6B02G215100.2	TaRaf91	Traes_6BS_EAABDE59A		

**TaMAPKKK132**	**TaRaf92**	TraesCS5B02G353600.1	TaRaf92	Traes_5BL_17A56822E		
**TaMAPKKK133**	**TaRaf93**	TraesCS1D02G004300.1	TaRaf93	Traes_1BS_EA26D2661		
**TaMAPKKK134**	**TaRaf94**	TraesCS5D02G547500.1	TaRaf94	Traes_5DL_383D5A71F		
**TaMAPKKK135**	**TaRaf95**	TraesCS2D02G598800.1	TaRaf95	Traes_2DL_77990F25A		
**TaMAPKKK136**	**TaRaf96**	TraesCS2B02G241600.1	TaRaf96	Traes_2BS_C0AED9734		
**TaMAPKKK137**	**TaRaf97**	TraesCS3D02G472000.1	TaRaf97	Traes_3DL_73ACAB95C		
**TaMAPKKK139**	**TaRaf99**	TraesCS3A02G493500.1	TaRaf99	Traes_3AL_AB54706CA		
**TaMAPKKK140**	**TaRaf100**	TraesCS5D02G097900.1	TaRaf100	Traes_5BS_F1687AA56		
**TaMAPKKK141**	**TaRaf101**	TraesCS7D02G000800.1	TaRaf101	Traes_7DS_A46AFAE10		
**TaMAPKKK142**	**TaRaf102**	TraesCS2A02G032200.1	TaRaf102	Traes_2AS_CC27D1C41		
**TaMAPKKK144**	**TaRaf104**	TraesCS3D02G501100.1	TaRaf104	Traes_3DL_3D1CAD68F		
**TaMAPKKK145**	**TaRaf105**	TraesCS2B02G242300.1	TaRaf105	Traes_2BS_5C64FC44A		
**TaMAPKKK146**	**TaRaf106**	TraesCS2B02G241400.1	TaRaf106	Traes_4BS_C5AB35B0C		
**TaMAPKKK147**	**TaRaf107**	TraesCS2A02G216600.1	TaRaf107	Traes_2AS_E5AB3458C		
**TaMAPKKK148**	**TaRaf108**	TraesCS1A02G003900.1	TaRaf108	Traes_1BS_41E5F1990		
**TaMAPKKK150**	**TaRaf110**	TraesCS3B02G008600.2	TaRaf110	TRAES3BF061500080CFD_t1		
**TaMAPKKK151**	**TaRaf111**	TraesCS3B02G123800.1	TaRaf111	TRAES3BF104900080CFD_t1		

**TaMAPKKK152**	**TaRaf112**	TraesCS3B02G259100.1	TaRaf112	TRAES3BF026200090CFD_t1		
**TaMAPKKK153**	**TaRaf113**	TraesCS3B02G351800.1	TaRaf113	TRAES3BF086600060CFD_t1		
**TaMAPKKK154**	**TaRaf114**	TraesCS3B02G478400.1	TaRaf114	TRAES3BF078400040CFD_t1		
**TaMAPKKK155**	**TaRaf115**	TraesCS6B02G217100.1	TaRaf115	Traes_6BS_5BFDC774A		
