# Supplementary material for: Bioinformatic Identification and Expression Analyses of the MAPK–MAP4K Gene Family Reveal a Putative Functional MAP4K10-MAP3K7/8-MAP2K1/11-MAPK3/6 Cascade in Wheat (Triticum aestivum L.)
Source: Plants (Basel). 2024 Mar 24;13(7):941. doi: 10.3390/plants13070941 (PMC11013086; doi:10.3390/plants13070941)
Supplement: Supplementary file 1 [file plants-13-00941-s001.zip › plants-2867660-supplementary/Supplementary Figure S1-S10 and Supplementary table S1-S7/Supplementary table S1-S7/Supplementary table 2.pdf]

TaMAPK-TaMAP4K Gene ID	Gene Name	*TaMAPK-TaMAP4K* Gene ID	Gene Name	Ka	Ks	Ka/Ks	EffectiveLen	AverageS-sites	AverageN-sites	Selection pressure	Mya
TraesCS1A02G003900	TaMAPKKK148	TraesCS1B02G004000	/	0.21	0.65	0.32	996.00	219.17	776.83	Purifying selection	49.63
TraesCS1A02G003900	TaMAPKKK148	TraesCS1B02G003200	/	0.21	0.46	0.44	978.00	217.83	760.17	Purifying selection	35.67
TraesCS1A02G025900	/	TraesCS1D02G026200	TaMAPKKK38	0.01	0.06	0.09	1845.00	421.33	1423.67	Purifying selection	4.75
TraesCS1A02G086500	TaMAPK11	TraesCS1B02G104900	TaMAPK27	0.01	0.10	0.06	1647.00	387.50	1259.50	Purifying selection	7.63
TraesCS1A02G086500	TaMAPK11	TraesCS1D02G088000	TaMAPK4	0.01	0.10	0.06	1647.00	386.67	1260.33	Purifying selection	7.65
TraesCS1A02G181900	TaMAPKKK1	TraesCS1B02G199100	TaMAPKKK2	0.00	0.05	0.08	2142.00	492.83	1649.17	Purifying selection	3.87
TraesCS1A02G181900	TaMAPKKK1	TraesCS1D02G185000	TaMAPKKK3	0.00	0.04	0.05	2142.00	493.08	1648.92	Purifying selection	2.71
TraesCS1A02G184500	TaMAPK14	TraesCS1B02G192600	TaMAPK28	0.00	0.07	0.03	1128.00	267.83	860.17	Purifying selection	5.42
TraesCS1A02G184500	TaMAPK14	TraesCS1D02G192200	/	0.00	0.03	0.03	1128.00	267.83	860.17	Purifying selection	2.64
TraesCS1A02G273600	/	TraesCS1D02G273600	TaMAPKKK104	0.08	0.15	0.53	2019.00	514.67	1504.33	Purifying selection	11.64
TraesCS1A02G273600	/	TraesCS1B02G283400	TaMAPKKK104-1	0.08	0.17	0.47	2025.00	516.33	1508.67	Purifying selection	12.70
TraesCS1A02G339600	/	TraesCS3A02G274000	TaMAPKKK100	0.33	1.29	0.26	1734.00	411.00	1323.00	Purifying selection	99.09
TraesCS1A02G339600	/	TraesCS3D02G273200	TaMAPKKK45	0.33	1.32	0.25	1740.00	412.83	1327.17	Purifying selection	101.42
TraesCS1A02G356300	/	TraesCS1B02G372400	TaMAPKKK53	0.03	0.14	0.23	2400.00	592.92	1807.08	Purifying selection	10.92
TraesCS1A02G366900	/	TraesCS3B02G288100	TaMAPKKK8	0.31	0.73	0.43	1338.00	337.83	1000.17	Purifying selection	56.52
TraesCS1A02G402400	TaMAPK41	TraesCS1B02G431400	TaMAPK29	0.02	0.15	0.11	1644.00	385.17	1258.83	Purifying selection	11.45

Table S2 The Ka/Ks ratio of MAPK-MAP4K gene pairs in wheat

TraesCS1A02G402400	TaMAPK41	TraesCS1D02G410100	TaMAPK33	0.01	0.14	0.10	1644.00	385.33	1258.67	Purifying selection	10.48
TraesCS1A02G402400	TaMAPK41	TraesCS3A02G242100	TaMAPK39	0.12	1.02	0.12	1608.00	369.00	1239.00	Purifying selection	78.42
TraesCS1A02G402400	TaMAPK41	TraesCS3B02G270200	TaMAPK16	0.13	1.05	0.12	1620.00	371.17	1248.83	Purifying selection	80.72
TraesCS1A02G402400	TaMAPK41	TraesCS3D02G242200	TaMAPK24	0.13	1.03	0.12	1614.00	370.25	1243.75	Purifying selection	78.94
TraesCS1A02G415300	TaMAPK42	TraesCS1B02G445300	/	0.01	0.04	0.38	1812.00	424.33	1387.67	Purifying selection	2.79
TraesCS1A02G415300	TaMAPK42	TraesCS1D02G422800	TaMAPK5	0.01	0.03	0.38	1815.00	423.67	1391.33	Purifying selection	2.03
TraesCS1A02G415300	TaMAPK42	TraesCS3A02G231700	TaMAPK40	0.13	1.22	0.11	1440.00	327.83	1112.17	Purifying selection	93.60
TraesCS1A02G415300	TaMAPK42	TraesCS3B02G260900	/	0.12	1.28	0.09	1440.00	329.92	1110.08	Purifying selection	98.85
TraesCS1A02G415300	TaMAPK42	TraesCS3D02G221700	TaMAPK23	0.12	1.32	0.09	1425.00	326.25	1098.75	Purifying selection	101.76
TraesCS1A02G416200	/	TraesCS1B02G446500	TaMAPKKK128	0.01	0.16	0.05	1113.00	265.00	848.00	Purifying selection	12.06
TraesCS1A02G416200	/	TraesCS3B02G259800	TaMAPKKK58	0.10	0.70	0.15	1113.00	265.75	847.25	Purifying selection	53.77
TraesCS1A02G416200	/	TraesCS1D02G423800	TaMAPKKK70	0.01	0.15	0.05	1119.00	266.00	853.00	Purifying selection	11.83
TraesCS1A02G421000	TaMAPK43	TraesCS1B02G452200	TaMAPK6	0.01	0.13	0.08	1794.00	434.42	1359.58	Purifying selection	10.03
TraesCS1A02G421000	TaMAPK43	TraesCS1D02G428900	TaMAPK34	0.01	0.10	0.08	1791.00	431.25	1359.75	Purifying selection	7.62
TraesCS1A02G421000	TaMAPK43	TraesCS3A02G228000	/	0.15	1.00	0.15	1767.00	419.92	1347.08	Purifying selection	76.63
TraesCS1A02G421000	TaMAPK43	TraesCS3B02G256700	TaMAPK38	0.15	1.01	0.15	1761.00	419.08	1341.92	Purifying selection	77.77
TraesCS1A02G421000	TaMAPK43	TraesCS3D02G225600	TaMAPK8	0.15	1.03	0.15	1767.00	420.25	1346.75	Purifying selection	78.97
TraesCS1B02G001500	/	TraesCS1D02G004300	TaMAPKKK133	0.01	0.31	0.02	1077.00	254.50	822.50	Purifying selection	23.56

TraesCS1B02G032500	/	TraesCS1D02G026200	TaMAPKKK38	0.02	0.09	0.19	1845.00	423.58	1421.42	Purifying selection	7.25
TraesCS1B02G104900	TaMAPK27	TraesCS1D02G088000	TaMAPK4	0.00	0.11	0.04	1647.00	387.83	1259.17	Purifying selection	8.31
TraesCS1B02G192600	TaMAPK28	TraesCS1D02G192200	/	0.00	0.07	0.02	1128.00	268.17	859.83	Purifying selection	5.41
TraesCS1B02G199100	TaMAPKKKK2	TraesCS1D02G185000	TaMAPKKKK3	0.00	0.04	0.09	2142.00	492.42	1649.58	Purifying selection	3.05
TraesCS1B02G283400	TaMAPKKK104-1	TraesCS1D02G273600	TaMAPKKK104	0.07	0.16	0.42	2028.00	513.83	1514.17	Purifying selection	12.21
TraesCS1B02G351900	/	TraesCS3A02G274000	TaMAPKKK100	0.33	1.29	0.26	1734.00	408.58	1325.42	Purifying selection	99.25
TraesCS1B02G351900	/	TraesCS3D02G273200	TaMAPKKK45	0.33	1.31	0.25	1740.00	410.75	1329.25	Purifying selection	100.57
TraesCS1B02G372400	TaMAPKKK53	TraesCS1D02G360300	/	0.13	0.22	0.61	1800.00	453.25	1346.75	Purifying selection	16.64
TraesCS1B02G372400	TaMAPKKK53	TraesCS3A02G246100	TaMAPKKK76	0.12	0.34	0.35	2391.00	591.00	1800.00	Purifying selection	26.40
TraesCS1B02G372400	TaMAPKKK53	TraesCS3B02G273200	/	0.11	0.35	0.32	2406.00	596.25	1809.75	Purifying selection	26.92
TraesCS1B02G372400	TaMAPKKK53	TraesCS3D02G245300	/	0.12	0.34	0.34	2406.00	594.75	1811.25	Purifying selection	26.15
TraesCS1B02G384700	/	TraesCS3B02G288100	TaMAPKKK8	0.34	0.66	0.51	1314.00	330.58	983.42	Purifying selection	50.53
TraesCS1B02G431400	TaMAPK29	TraesCS1D02G410100	TaMAPK33	0.01	0.11	0.09	1665.00	391.33	1273.67	Purifying selection	8.45
TraesCS1B02G431400	TaMAPK29	TraesCS3A02G242100	TaMAPK39	0.12	1.19	0.10	1632.00	376.00	1256.00	Purifying selection	91.16
TraesCS1B02G431400	TaMAPK29	TraesCS3B02G270200	TaMAPK16	0.13	1.22	0.11	1644.00	378.17	1265.83	Purifying selection	93.90
TraesCS1B02G431400	TaMAPK29	TraesCS3D02G242200	TaMAPK24	0.13	1.22	0.11	1638.00	377.25	1260.75	Purifying selection	93.78
TraesCS1B02G445300	/	TraesCS3D02G221700	TaMAPK23	0.12	1.29	0.09	1425.00	326.25	1098.75	Purifying selection	98.85
TraesCS1B02G445300	/	TraesCS1D02G422800	TaMAPK5	0.01	0.04	0.28	1812.00	424.00	1388.00	Purifying selection	3.36

TraesCS1B02G445300	/	TraesCS3A02G231700	TaMAPK40	0.13	1.28	0.10	1440.00	327.50	1112.50	Purifying selection	98.29
TraesCS1B02G446500	TaMAPKKK128	TraesCS1D02G423800	TaMAPKKK70	0.01	0.14	0.05	1113.00	264.67	848.33	Purifying selection	10.84
TraesCS1B02G446500	TaMAPKKK128	TraesCS3A02G230500	/	0.10	0.68	0.15	1107.00	263.50	843.50	Purifying selection	52.57
TraesCS1B02G446500	TaMAPKKK128	TraesCS3B02G259800	TaMAPKKK58	0.10	0.70	0.14	1107.00	263.25	843.75	Purifying selection	53.75
TraesCS1B02G446500	TaMAPKKK128	TraesCS3D02G222800	/	0.10	0.69	0.15	1107.00	263.58	843.42	Purifying selection	52.90
TraesCS1B02G452200	TaMAPK6	TraesCS1D02G428900	TaMAPK34	0.01	0.10	0.06	1797.00	434.50	1362.50	Purifying selection	7.96
TraesCS1B02G452200	TaMAPK6	TraesCS3A02G228000	/	0.15	1.08	0.14	1770.00	423.08	1346.92	Purifying selection	82.85
TraesCS1B02G452200	TaMAPK6	TraesCS3B02G256700	TaMAPK38	0.15	1.11	0.13	1764.00	422.25	1341.75	Purifying selection	85.71
TraesCS1B02G452200	TaMAPK6	TraesCS3D02G225600	TaMAPK8	0.15	1.11	0.13	1770.00	423.42	1346.58	Purifying selection	85.43
TraesCS1B02G454000	TaMAPKKK116	TraesCS1D02G430800	/	0.21	0.60	0.34	1932.00	473.17	1458.83	Purifying selection	45.88
TraesCS1D02G341600	/	TraesCS3A02G274000	TaMAPKKK100	0.33	1.31	0.25	1725.00	407.25	1317.75	Purifying selection	100.66
TraesCS1D02G341600	/	TraesCS3D02G273200	TaMAPKKK45	0.33	1.29	0.26	1731.00	409.08	1321.92	Purifying selection	98.93
TraesCS1D02G360400	/	TraesCS3A02G246100	TaMAPKKK76	0.10	0.33	0.31	2373.00	590.67	1782.33	Purifying selection	25.33
TraesCS1D02G372900	/	TraesCS3B02G288100	TaMAPKKK8	0.37	0.79	0.47	1308.00	329.58	978.42	Purifying selection	60.51
TraesCS1D02G410100	TaMAPK33	TraesCS3A02G242100	TaMAPK39	0.12	1.22	0.10	1632.00	375.83	1256.17	Purifying selection	93.80
TraesCS1D02G410100	TaMAPK33	TraesCS3B02G270200	TaMAPK16	0.13	1.27	0.10	1644.00	378.00	1266.00	Purifying selection	97.75
TraesCS1D02G410100	TaMAPK33	TraesCS3D02G242200	TaMAPK24	0.13	1.23	0.10	1638.00	377.08	1260.92	Purifying selection	94.40
TraesCS1D02G422800	TaMAPK5	TraesCS3A02G231700	TaMAPK40	0.13	1.25	0.11	1440.00	327.50	1112.50	Purifying selection	96.28

TraesCS1D02G422800	TaMAPK5	TraesCS3B02G260900	/	0.12	1.32	0.09	1440.00	329.58	1110.42	Purifying selection	101.77
TraesCS1D02G422800	TaMAPK5	TraesCS3D02G221700	TaMAPK23	0.12	1.36	0.09	1425.00	325.92	1099.08	Purifying selection	104.88
TraesCS1D02G423800	TaMAPKKK70	TraesCS3A02G230500	/	0.10	0.69	0.15	1107.00	264.00	843.00	Purifying selection	53.25
TraesCS1D02G423800	TaMAPKKK70	TraesCS3B02G259800	TaMAPKKK58	0.10	0.71	0.14	1113.00	264.58	848.42	Purifying selection	54.54
TraesCS1D02G423800	TaMAPKKK70	TraesCS3D02G222800	/	0.10	0.72	0.14	1113.00	264.92	848.08	Purifying selection	55.17
TraesCS1D02G428900	TaMAPK34	TraesCS3A02G228000	/	0.16	1.01	0.15	1770.00	420.33	1349.67	Purifying selection	77.75
TraesCS1D02G428900	TaMAPK34	TraesCS3B02G256700	TaMAPK38	0.15	1.02	0.15	1764.00	419.67	1344.33	Purifying selection	78.13
TraesCS1D02G428900	TaMAPK34	TraesCS3D02G225600	TaMAPK8	0.16	1.04	0.15	1770.00	420.67	1349.33	Purifying selection	80.13
TraesCS2A02G051000	/	TraesCS2D02G050700	TaMAPKKK46	0.15	0.48	0.32	315.00	75.92	239.08	Purifying selection	36.77
TraesCS2A02G095300	TaMAPKKK28	TraesCS2B02G110500	TaMAPKKK27	0.01	0.07	0.11	3996.00	966.50	3029.50	Purifying selection	5.60
TraesCS2A02G095300	TaMAPKKK28	TraesCS2D02G093700	TaMAPKKK26	0.01	0.07	0.10	3996.00	965.42	3030.58	Purifying selection	5.69
TraesCS2A02G190200	/	TraesCS2D02G197600	TaMAPKKK34	0.00	0.04	0.09	957.00	232.67	724.33	Purifying selection	3.40
TraesCS2A02G190200	/	TraesCS2B02G216800	TaMAPKKK37	0.01	0.08	0.14	966.00	234.42	731.58	Purifying selection	6.23
TraesCS2A02G195900	TaMAPKKK32	TraesCS2B02G223600	TaMAPKKK36	0.01	0.08	0.09	2103.00	484.75	1618.25	Purifying selection	6.02
TraesCS2A02G195900	TaMAPKKK32	TraesCS2D02G203900	/	0.01	0.08	0.07	2103.00	485.83	1617.17	Purifying selection	6.53
TraesCS2A02G199700	TaMAPKKK5	TraesCS2B02G227000	/	0.00	0.06	0.06	1629.00	374.83	1254.17	Purifying selection	4.26
TraesCS2A02G199700	TaMAPKKK5	TraesCS2D02G207400	/	0.00	0.06	0.07	1629.00	374.67	1254.33	Purifying selection	4.48
TraesCS2A02G214000	TaMAPKKK122	TraesCS2D02G219800	TaMAPKKK109	0.02	0.10	0.22	2043.00	510.92	1532.08	Purifying selection	7.72

TraesCS2A02G216300	/	TraesCS2B02G241400	TaMAPKKK146	0.04	0.10	0.36	2001.00	489.92	1511.08	Purifying selection	7.81
TraesCS2A02G217000	TaMAPKKK60	TraesCS2B02G242400	/	0.07	0.15	0.49	1128.00	275.17	852.83	Purifying selection	11.68
TraesCS2A02G217600	TaMAPKKK80	TraesCS2B02G243100	/	0.01	0.09	0.09	1017.00	231.25	785.75	Purifying selection	6.69
TraesCS2A02G233400	TaMAPKKKK4	TraesCS2B02G249900	TaMAPKKKK5	0.00	0.05	0.08	2076.00	496.17	1579.83	Purifying selection	3.52
TraesCS2A02G233400	TaMAPKKKK4	TraesCS2D02G232200	TaMAPKKKK6	0.00	0.03	0.15	2076.00	495.92	1580.08	Purifying selection	2.13
TraesCS2A02G407600	TaMAPKKK1	TraesCS2B02G425500	/	0.02	0.08	0.31	2460.00	586.50	1873.50	Purifying selection	6.08
TraesCS2A02G407600	TaMAPKKK1	TraesCS2D02G404700	/	0.02	0.06	0.41	2481.00	591.58	1889.42	Purifying selection	4.48
TraesCS2A02G407600	TaMAPKKK1	TraesCS6A02G245000	TaMAPKKK4	0.15	0.91	0.17	2427.00	580.08	1846.92	Purifying selection	69.77
TraesCS2A02G407600	TaMAPKKK1	TraesCS6B02G279300	TaMAPKKK4-1	0.13	0.85	0.16	2343.00	561.42	1781.58	Purifying selection	65.46
TraesCS2A02G407600	TaMAPKKK1	TraesCS6D02G227300	/	0.15	0.88	0.17	2427.00	581.92	1845.08	Purifying selection	67.69
TraesCS2A02G498000	TaMAPKKK20	TraesCS2B02G526200	TaMAPKKK18	0.01	0.08	0.10	3969.00	917.92	3051.08	Purifying selection	6.28
TraesCS2A02G498000	TaMAPKKK20	TraesCS2D02G498100	/	0.01	0.07	0.10	3861.00	895.75	2965.25	Purifying selection	5.49
TraesCS2A02G578900	/	TraesCS2D02G598800	TaMAPKKK135	0.36	1.10	0.33	1290.00	301.00	989.00	Purifying selection	84.89
TraesCS2B02G082200	/	TraesCS2D02G066900	TaMAPKKK121	0.20	0.64	0.30	795.00	189.42	605.58	Purifying selection	49.51
TraesCS2B02G110500	TaMAPKKK27	TraesCS2D02G093700	TaMAPKKK26	0.00	0.04	0.11	4005.00	967.92	3037.08	Purifying selection	3.44
TraesCS2B02G216800	TaMAPKKK37	TraesCS2D02G197600	TaMAPKKK34	0.01	0.04	0.19	957.00	232.92	724.08	Purifying selection	3.40
TraesCS2B02G223600	TaMAPKKK36	TraesCS2D02G203900	/	0.00	0.07	0.06	2109.00	486.75	1622.25	Purifying selection	5.64
TraesCS2B02G241400	TaMAPKKK146	TraesCS2D02G221900	/	0.03	0.10	0.36	2001.00	491.33	1509.67	Purifying selection	7.34

TraesCS2B02G249900	TaMAPKKKK5	TraesCS2D02G232200	TaMAPKKKK6	0.01	0.05	0.09	2076.00	495.42	1580.58	Purifying selection	4.19
TraesCS2B02G425500	/	TraesCS6B02G279300	TaMAPKKK4-1	0.14	0.85	0.16	2355.00	566.33	1788.67	Purifying selection	65.10
TraesCS2B02G425500	/	TraesCS6A02G245000	TaMAPKKK4	0.16	0.86	0.18	2616.00	628.58	1987.42	Purifying selection	66.22
TraesCS2B02G526200	TaMAPKKK18	TraesCS2D02G498100	/	0.01	0.05	0.10	3864.00	897.67	2966.33	Purifying selection	3.90
TraesCS2B02G606100	/	TraesCS2D02G598800	TaMAPKKK135	0.25	1.21	0.21	1035.00	244.08	790.92	Purifying selection	92.80
TraesCS2B02G610200	/	TraesCS2D02G588200	TaMAPKKK69	0.02	0.11	0.19	1638.00	389.92	1248.08	Purifying selection	8.30
TraesCS2D02G404700	/	TraesCS6A02G245000	TaMAPKKK4	0.15	0.83	0.18	2616.00	627.92	1988.08	Purifying selection	64.01
TraesCS2D02G404700	/	TraesCS6B02G279300	TaMAPKKK4-1	0.13	0.82	0.16	2355.00	566.00	1789.00	Purifying selection	62.71
TraesCS3A02G001500	TaMAPKKK99	TraesCS3B02G000400	/	0.02	0.16	0.10	1095.00	268.17	826.83	Purifying selection	12.26
TraesCS3A02G001500	TaMAPKKK99	TraesCS3D02G010000	/	0.01	0.17	0.07	1092.00	268.67	823.33	Purifying selection	12.77
TraesCS3A02G003900	TaMAPKKK72	TraesCS3B02G008600	TaMAPKKK150	0.03	0.16	0.20	2043.00	532.83	1510.17	Purifying selection	12.53
TraesCS3A02G003900	TaMAPKKK72	TraesCS3D02G005400	/	0.02	0.14	0.18	1203.00	285.67	917.33	Purifying selection	10.61
TraesCS3A02G039100	TaMAPKKK56	TraesCS3B02G043700	/	0.11	0.37	0.30	1440.00	339.33	1100.67	Purifying selection	28.76
TraesCS3A02G039100	TaMAPKKK56	TraesCS3D02G040300	/	0.08	0.37	0.23	1284.00	297.75	986.25	Purifying selection	28.55
TraesCS3A02G039200	TaMAPKKK73	TraesCS3B02G043600	/	0.12	0.34	0.36	2031.00	490.42	1540.58	Purifying selection	26.43
TraesCS3A02G039200	TaMAPKKK73	TraesCS3D02G040400	/	0.02	0.14	0.14	540.00	119.67	420.33	Purifying selection	10.55
TraesCS3A02G045200	TaMAPKKK105	TraesCS3B02G038900	/	0.01	0.13	0.12	1185.00	298.75	886.25	Purifying selection	9.80
TraesCS3A02G045200	TaMAPKKK105	TraesCS3D02G036000	/	0.01	0.10	0.07	1197.00	302.25	894.75	Purifying selection	7.32

TraesCS3A02G094800	/	TraesCS3B02G110300	TaMAPKKK43	0.04	0.11	0.36	435.00	92.58	342.42	Purifying selection	8.57
TraesCS3A02G096500	TaMAPKKK75	TraesCS3B02G112300	/	0.01	0.11	0.11	1941.00	488.92	1452.08	Purifying selection	8.09
TraesCS3A02G096500	TaMAPKKK75	TraesCS3D02G097000	TaMAPKKK117	0.01	0.06	0.22	1944.00	488.92	1455.08	Purifying selection	4.75
TraesCS3A02G105400	/	TraesCS3B02G123800	TaMAPKKK151	0.02	0.09	0.26	1731.00	425.25	1305.75	Purifying selection	6.57
TraesCS3A02G228000	/	TraesCS3D02G225600	TaMAPK8	0.00	0.05	0.02	1839.00	433.67	1405.33	Purifying selection	3.47
TraesCS3A02G228000	/	TraesCS3B02G256700	TaMAPK38	0.00	0.05	0.03	1806.00	425.67	1380.33	Purifying selection	3.54
TraesCS3A02G229800	TaMAPKKK115	TraesCS3B02G259100	TaMAPKKK152	0.04	0.10	0.41	2094.00	567.58	1526.42	Purifying selection	7.59
TraesCS3A02G230500	/	TraesCS3B02G259800	TaMAPKKK58	0.00	0.08	0.04	1155.00	276.58	878.42	Purifying selection	6.47
TraesCS3A02G231700	TaMAPK40	TraesCS3B02G260900	/	0.02	0.08	0.25	1470.00	337.75	1132.25	Purifying selection	5.99
TraesCS3A02G231700	TaMAPK40	TraesCS3D02G221700	TaMAPK23	0.02	0.08	0.29	1464.00	336.58	1127.42	Purifying selection	6.02
TraesCS3A02G242100	TaMAPK39	TraesCS3B02G270200	TaMAPK16	0.00	0.03	0.09	1746.00	395.00	1351.00	Purifying selection	2.59
TraesCS3A02G242100	TaMAPK39	TraesCS3B02G256700	TaMAPK38	0.19	2.64	0.07	1692.00	389.83	1302.17	Purifying selection	202.72
TraesCS3A02G242100	TaMAPK39	TraesCS3D02G242200	TaMAPK24	0.00	0.02	0.11	1746.00	395.75	1350.25	Purifying selection	1.58
TraesCS3A02G242100	TaMAPK39	TraesCS3D02G225600	TaMAPK8	0.19	2.61	0.07	1722.00	397.08	1324.92	Purifying selection	200.95
TraesCS3A02G246100	TaMAPKKK76	TraesCS3B02G273200	/	0.03	0.12	0.22	2439.00	603.00	1836.00	Purifying selection	9.25
TraesCS3A02G246100	TaMAPKKK76	TraesCS3D02G245300	/	0.03	0.12	0.23	2439.00	601.50	1837.50	Purifying selection	9.05
TraesCS3A02G255600	/	TraesCS3B02G288100	TaMAPKKK8	0.03	0.13	0.27	1383.00	343.58	1039.42	Purifying selection	10.00
TraesCS3A02G274000	TaMAPKKK100	TraesCS3B02G307700	/	0.02	0.07	0.33	1800.00	417.00	1383.00	Purifying selection	5.01

TraesCS3A02G274000	TaMAPKKK100	TraesCS3D02G273200	TaMAPKKK45	0.02	0.09	0.20	1800.00	417.92	1382.08	Purifying selection	6.83
TraesCS3A02G315100	TaMAPKKK111	TraesCS3B02G351800	TaMAPKKK153	0.02	0.11	0.14	906.00	213.08	692.92	Purifying selection	8.54
TraesCS3A02G315100	TaMAPKKK111	TraesCS3D02G316400	/	0.04	0.09	0.47	2418.00	553.00	1865.00	Purifying selection	6.55
TraesCS3A02G477300	/	TraesCS3D02G472000	TaMAPKKK137	0.03	0.12	0.23	2235.00	537.83	1697.17	Purifying selection	8.96
TraesCS3A02G493500	TaMAPKKK139	TraesCS3B02G555500	/	0.03	0.09	0.34	2121.00	492.83	1628.17	Purifying selection	6.87
TraesCS3A02G493800	/	TraesCS3D02G501100	TaMAPKKK144	0.10	0.23	0.42	2106.00	489.75	1616.25	Purifying selection	17.69
TraesCS3B02G008600	TaMAPKKK150	TraesCS3D02G005400	/	0.03	0.20	0.13	1197.00	284.92	912.08	Purifying selection	15.67
TraesCS3B02G022400	/	TraesCS3D02G023600	TaMAPKKK108	0.03	0.10	0.26	2484.00	601.83	1882.17	Purifying selection	8.06
TraesCS3B02G110300	TaMAPKKK43	TraesCS3D02G095000	/	0.04	0.11	0.36	435.00	92.75	342.25	Purifying selection	8.55
TraesCS3B02G112300	/	TraesCS3D02G097000	TaMAPKKK117	0.01	0.10	0.11	1941.00	489.33	1451.67	Purifying selection	7.73
TraesCS3B02G123800	TaMAPKKK151	TraesCS3D02G107500	/	0.02	0.05	0.32	1731.00	426.17	1304.83	Purifying selection	4.02
TraesCS3B02G256700	TaMAPK38	TraesCS3B02G270200	TaMAPK16	0.19	2.55	0.07	1692.00	389.17	1302.83	Purifying selection	196.35
TraesCS3B02G256700	TaMAPK38	TraesCS3D02G225600	TaMAPK8	0.00	0.03	0.05	1806.00	426.00	1380.00	Purifying selection	2.21
TraesCS3B02G256700	TaMAPK38	TraesCS3D02G242200	TaMAPK24	0.19	2.97	0.06	1692.00	389.92	1302.08	Purifying selection	228.16
TraesCS3B02G259800	TaMAPKKK58	TraesCS3D02G222800	/	0.00	0.04	0.08	1155.00	276.67	878.33	Purifying selection	3.44
TraesCS3B02G260900	/	TraesCS3D02G221700	TaMAPK23	0.01	0.06	0.14	1464.00	338.67	1125.33	Purifying selection	4.49
TraesCS3B02G270200	TaMAPK16	TraesCS3D02G242200	TaMAPK24	0.00	0.05	0.03	1752.00	395.92	1356.08	Purifying selection	3.61
TraesCS3B02G270200	TaMAPK16	TraesCS3D02G225600	TaMAPK8	0.20	2.46	0.08	1722.00	396.42	1325.58	Purifying selection	189.45

TraesCS3B02G288100	TaMAPKKK8	TraesCS3D02G256400	/	0.30	0.67	0.45	1329.00	331.25	997.75	Purifying selection	51.63
TraesCS3B02G307700	/	TraesCS3D02G273200	TaMAPKKK45	0.01	0.07	0.19	1803.00	418.08	1384.92	Purifying selection	5.40
TraesCS3B02G351800	TaMAPKKK153	TraesCS3D02G316400	/	0.01	0.12	0.09	906.00	212.50	693.50	Purifying selection	9.42
TraesCS3B02G555800	/	TraesCS3D02G501100	TaMAPKKK144	0.09	0.22	0.41	2103.00	487.67	1615.33	Purifying selection	17.03
TraesCS3D02G225600	TaMAPK8	TraesCS3D02G242200	TaMAPK24	0.20	2.92	0.07	1722.00	397.17	1324.83	Purifying selection	224.88
TraesCS4A02G093800	TaMAPKKK12	TraesCS4B02G210600	TaMAPKKK3	0.01	0.07	0.11	2115.00	513.00	1602.00	Purifying selection	5.50
TraesCS4A02G093800	TaMAPKKK12	TraesCS4D02G211300	TaMAPKKK10	0.01	0.06	0.11	2130.00	516.33	1613.67	Purifying selection	4.65
TraesCS4A02G106400	TaMAPK30	TraesCS4B02G197800	/	0.00	0.08	0.00	1107.00	258.33	848.67	Purifying selection	6.29
TraesCS4A02G106400	TaMAPK30	TraesCS4D02G198600	TaMAPK25	0.00	0.06	0.02	1107.00	259.00	848.00	Purifying selection	4.64
TraesCS4A02G265900	TaMAPKK14	TraesCS4B02G048600	TaMAPKK7	0.23	0.62	0.38	975.00	265.42	709.58	Purifying selection	47.93
TraesCS4A02G265900	TaMAPKK14	TraesCS4D02G048800	TaMAPKK11	0.06	0.35	0.18	1002.00	271.92	730.08	Purifying selection	27.01
TraesCS4A02G266000	TaMAPKK15	TraesCS4B02G048100	TaMAPKK6	0.24	0.54	0.44	975.00	267.92	707.08	Purifying selection	41.27
TraesCS4A02G266000	TaMAPKK15	TraesCS4D02G047900	/	0.23	0.51	0.45	975.00	268.92	706.08	Purifying selection	38.90
TraesCS4A02G283400	/	TraesCS4D02G027600	TaMAPKKK2	0.01	0.14	0.07	1704.00	431.00	1273.00	Purifying selection	10.76
TraesCS4A02G313900	TaMAPKKK62	TraesCS5D02G547600	/	0.30	1.34	0.23	3252.00	779.92	2472.08	Purifying selection	102.76
TraesCS4A02G336800	TaMAPK2	TraesCS5B02G536500	TaMAPK50	0.02	0.08	0.24	1320.00	312.08	1007.92	Purifying selection	5.79
TraesCS4A02G336800	TaMAPK2	TraesCS5D02G534000	TaMAPK26	0.01	0.05	0.23	1320.00	311.17	1008.83	Purifying selection	4.19
TraesCS4A02G383000	TaMAPKKK63	TraesCS7D02G079100	TaMAPKKK48	0.13	0.35	0.36	1653.00	373.08	1279.92	Purifying selection	27.28

TraesCS4A02G434800	TaMAPK31	TraesCS7A02G049000	TaMAPK46	0.02	0.10	0.18	1449.00	344.75	1104.25	Purifying selection	7.62
TraesCS4A02G434800	TaMAPK31	TraesCS7D02G044100	TaMAPK18	0.01	0.08	0.16	1449.00	343.50	1105.50	Purifying selection	6.51
TraesCS4A02G456900	TaMAPKKK106	TraesCS7A02G032700	TaMAPKKK107	0.02	0.10	0.23	1440.00	332.92	1107.08	Purifying selection	7.65
TraesCS4A02G456900	TaMAPKKK106	TraesCS7D02G029300	/	0.02	0.05	0.31	1437.00	332.42	1104.58	Purifying selection	3.83
TraesCS4A02G464700	TaMAPKKK64-1	TraesCS7D02G020600	/	0.11	0.60	0.19	504.00	116.67	387.33	Purifying selection	45.88
TraesCS4A02G464700	TaMAPKKK64-1	TraesCS7D02G022000	/	0.13	0.60	0.21	504.00	117.33	386.67	Purifying selection	45.97
TraesCS4A02G465000	TaMAPKKK65	TraesCS7D02G022200	TaMAPKKK47	0.44	1.04	0.43	1239.00	283.33	955.67	Purifying selection	80.30
TraesCS4A02G465900	TaMAPKKK64	TraesCS7A02G023400	/	0.08	0.28	0.31	792.00	176.75	615.25	Purifying selection	21.24
TraesCS4B02G030100	/	TraesCS4D02G027600	TaMAPKKK2	0.01	0.15	0.09	1704.00	430.83	1273.17	Purifying selection	11.41
TraesCS4B02G048100	TaMAPKK6	TraesCS4D02G047900	/	0.04	0.13	0.31	1029.00	284.50	744.50	Purifying selection	9.69
TraesCS4B02G048900	TaMAPKK8	TraesCS4D02G048800	TaMAPKK11	0.02	0.15	0.17	1002.00	273.50	728.50	Purifying selection	11.30
TraesCS4B02G210600	TaMAPKKK3	TraesCS4D02G211300	TaMAPKKK10	0.01	0.05	0.16	2115.00	513.67	1601.33	Purifying selection	4.19
TraesCS4B02G289100	TaMAPKKK110	TraesCS4D02G288200	/	0.02	0.11	0.20	2004.00	526.50	1477.50	Purifying selection	8.12
TraesCS4B02G395600	TaMAPKKKK7	TraesCS5A02G556400	TaMAPKKKK11	0.11	0.18	0.58	1779.00	419.42	1359.58	Purifying selection	14.03
TraesCS4B02G398400	TaMAPKKKK8	TraesCS5A02G556400	TaMAPKKKK11	0.01	0.08	0.16	2187.00	511.25	1675.75	Purifying selection	5.94
TraesCS4B02G398400	TaMAPKKKK8	TraesCSU02G115300	TaMAPKKKK25	0.01	0.08	0.17	2223.00	519.08	1703.92	Purifying selection	6.25
TraesCS5A02G085900	/	TraesCS5D02G097900	TaMAPKKK140	0.00	0.03	0.13	2259.00	531.83	1727.17	Purifying selection	2.66
TraesCS5A02G118200	TaMAPKKK14	TraesCS5B02G112900	/	0.01	0.04	0.24	1638.00	383.33	1254.67	Purifying selection	3.09

TraesCS5A02G122700	TaMAPKK4	TraesCS5B02G122600	TaMAPKK18	0.02	0.06	0.36	897.00	213.17	683.83	Purifying selection	4.44
TraesCS5A02G122700	TaMAPKK4	TraesCS5D02G130900	TaMAPKK3	0.02	0.07	0.25	897.00	212.42	684.58	Purifying selection	5.64
TraesCS5A02G147500	/	TraesCS5D02G145100	TaMAPKKK30	0.01	0.05	0.22	1908.00	478.83	1429.17	Purifying selection	3.48
TraesCS5A02G147500	/	TraesCS5B02G146100	TaMAPKKK40	0.01	0.09	0.10	1908.00	477.75	1430.25	Purifying selection	6.65
TraesCS5A02G187400	TaMAPKKKK9	TraesCS5B02G196400	TaMAPKKK25	0.02	0.09	0.17	1554.00	383.08	1170.92	Purifying selection	7.27
TraesCS5A02G187400	TaMAPKKKK9	TraesCS5D02G203600	TaMAPKKKK13	0.02	0.09	0.17	1554.00	380.67	1173.33	Purifying selection	7.09
TraesCS5A02G200800	TaMAPKKK17	TraesCS5B02G199400	TaMAPKKK24	0.00	0.06	0.02	2040.00	474.92	1565.08	Purifying selection	4.37
TraesCS5A02G200800	TaMAPKKK17	TraesCS5D02G206500	TaMAPKKK29	0.00	0.03	0.04	2046.00	477.50	1568.50	Purifying selection	2.47
TraesCS5A02G206700	/	TraesCS5B02G204900	TaMAPKKK95	0.01	0.07	0.20	1317.00	349.25	967.75	Purifying selection	5.54
TraesCS5A02G292500	TaMAPKKK81	TraesCS5D02G299800	/	0.03	0.06	0.51	1047.00	228.58	818.42	Purifying selection	4.55
TraesCS5A02G338800	/	TraesCS5B02G337300	TaMAPKKK92	0.00	0.08	0.05	1785.00	416.67	1368.33	Purifying selection	6.44
TraesCS5A02G351100	/	TraesCS5D02G358200	TaMAPKKK123-1	0.10	0.30	0.34	1350.00	308.42	1041.58	Purifying selection	23.17
TraesCS5A02G392500	TaMAPKKK22	TraesCS5B02G397300	TaMAPKKKK12	0.01	0.06	0.10	2475.00	596.33	1878.67	Purifying selection	4.28
TraesCS5A02G392500	TaMAPKKK22	TraesCS5D02G402300	TaMAPKKKK14	0.01	0.05	0.11	2466.00	593.58	1872.42	Purifying selection	3.75
TraesCS5A02G463100	TaMAPKKK15	TraesCS5B02G474500	TaMAPKKK16	0.00	0.10	0.01	1602.00	371.42	1230.58	Purifying selection	7.75
TraesCS5A02G463100	TaMAPKKK15	TraesCS5D02G475900	TaMAPKKK11	0.00	0.07	0.06	1602.00	371.67	1230.33	Purifying selection	5.65
TraesCS5A02G469200	/	TraesCS5D02G482000	TaMAPKKK98	0.01	0.05	0.14	1242.00	296.83	945.17	Purifying selection	4.02
TraesCS5A02G556400	TaMAPKKKK11	TraesCSU02G115300	TaMAPKKKK25	0.01	0.05	0.14	2187.00	508.50	1678.50	Purifying selection	4.15

TraesCS5B02G012000	TaMAPKKK94	TraesCS5D02G019400	TaMAPKKK101	0.03	0.12	0.25	3039.00	812.67	2226.33	Purifying selection	9.00
TraesCS5B02G091700	/	TraesCS5D02G097900	TaMAPKKK140	0.01	0.07	0.08	2259.00	532.00	1727.00	Purifying selection	5.30
TraesCS5B02G122600	TaMAPKK18	TraesCS5D02G130900	TaMAPKK3	0.03	0.07	0.39	1386.00	322.33	1063.67	Purifying selection	5.03
TraesCS5B02G146100	TaMAPKKK40	TraesCS5D02G145100	TaMAPKKK30	0.01	0.08	0.13	1920.00	481.08	1438.92	Purifying selection	5.89
TraesCS5B02G196400	TaMAPKKK25	TraesCS5D02G203600	TaMAPKKK13	0.01	0.07	0.20	1554.00	382.92	1171.08	Purifying selection	5.70
TraesCS5B02G199400	TaMAPKKK24	TraesCS5D02G206500	TaMAPKKK29	0.00	0.07	0.02	2040.00	475.58	1564.42	Purifying selection	5.25
TraesCS5B02G204900	TaMAPKKK95	TraesCS5D02G212900	/	0.01	0.05	0.19	1317.00	349.17	967.83	Purifying selection	3.87
TraesCS5B02G292000	TaMAPKKK96	TraesCS5D02G299800	/	0.12	0.35	0.35	1047.00	230.50	816.50	Purifying selection	26.85
TraesCS5B02G337300	TaMAPKKK92	TraesCS5D02G343000	/	0.00	0.05	0.03	1788.00	416.83	1371.17	Purifying selection	4.21
TraesCS5B02G353600	TaMAPKKK132	TraesCS5D02G357900	/	0.17	0.65	0.26	861.00	221.25	639.75	Purifying selection	50.33
TraesCS5B02G353800	TaMAPKKK97	TraesCS5D02G358600	/	0.13	0.54	0.24	672.00	156.25	515.75	Purifying selection	41.39
TraesCS5B02G354400	/	TraesCS5D02G358200	TaMAPKKK123-1	0.12	0.43	0.28	1362.00	310.58	1051.42	Purifying selection	32.94
TraesCS5B02G380400	/	TraesCS5D02G386800	TaMAPKKK86	0.00	0.10	0.01	1119.00	263.50	855.50	Purifying selection	7.47
TraesCS5B02G397300	TaMAPKKKK12	TraesCS5D02G402300	TaMAPKKKK14	0.00	0.03	0.09	2466.00	593.42	1872.58	Purifying selection	2.58
TraesCS5B02G474500	TaMAPKKK16	TraesCS5D02G475900	TaMAPKKK11	0.00	0.08	0.04	1602.00	371.92	1230.08	Purifying selection	6.33
TraesCS5B02G481700	/	TraesCS5D02G482000	TaMAPKKK98	0.01	0.07	0.10	1245.00	299.00	946.00	Purifying selection	5.67
TraesCS5B02G536500	TaMAPK50	TraesCS5D02G534000	TaMAPK26	0.01	0.06	0.17	1320.00	312.08	1007.92	Purifying selection	4.88
TraesCS5B02G565100	TaMAPKK2	TraesCS5D02G549600	TaMAPKK13	0.02	0.06	0.37	1164.00	271.42	892.58	Purifying selection	4.34

TraesCS6A02G004500	TaMAPKKK71	TraesCS6B02G013900	/	0.20	0.26	0.74	420.00	93.50	326.50	Purifying selection	20.24
TraesCS6A02G099600	TaMAPK35	TraesCS6B02G127800	TaMAPK17	0.03	0.08	0.36	1494.00	350.42	1143.58	Purifying selection	6.25
TraesCS6A02G099600	TaMAPK35	TraesCS6D02G082900	TaMAPK44	0.01	0.06	0.19	1500.00	349.33	1150.67	Purifying selection	4.58
TraesCS6A02G118100	TaMAPK36	TraesCS6B02G146300	TaMAPK10	0.00	0.10	0.01	1107.00	254.83	852.17	Purifying selection	7.40
TraesCS6A02G118100	TaMAPK36	TraesCS6D02G108100	TaMAPK45	0.01	0.11	0.08	1140.00	263.17	876.83	Purifying selection	8.15
TraesCS6A02G118100	TaMAPK36	TraesCS7A02G422500	TaMAPK12	0.05	1.03	0.04	1107.00	254.92	852.08	Purifying selection	79.31
TraesCS6A02G118100	TaMAPK36	TraesCS7B02G322900	TaMAPK54	0.05	1.14	0.04	1107.00	254.58	852.42	Purifying selection	87.91
TraesCS6A02G118100	TaMAPK36	TraesCS7D02G414700	/	0.05	1.05	0.04	1107.00	254.92	852.08	Purifying selection	81.13
TraesCS6A02G149900	TaMAPKKK21	TraesCS6B02G177800	TaMAPKKK18	0.01	0.12	0.10	1626.00	362.17	1263.83	Purifying selection	9.57
TraesCS6A02G149900	TaMAPKKK21	TraesCS6D02G139200	TaMAPKKK23	0.00	0.11	0.04	1527.00	338.33	1188.67	Purifying selection	8.56
TraesCS6A02G155200	/	TraesCS7D02G474700	TaMAPKKK102	0.09	0.77	0.11	1254.00	292.42	961.58	Purifying selection	59.41
TraesCS6A02G172600	TaMAPKKK41	TraesCS7A02G388800	/	0.19	0.60	0.32	1014.00	243.33	770.67	Purifying selection	46.04
TraesCS6A02G172600	TaMAPKKK41	TraesCS7B02G290700	/	0.19	0.59	0.32	1014.00	243.67	770.33	Purifying selection	45.25
TraesCS6A02G172600	TaMAPKKK41	TraesCS7D02G384700	TaMAPKKK61	0.19	0.59	0.32	1014.00	243.67	770.33	Purifying selection	45.25
TraesCS6A02G186100	/	TraesCS6B02G215100	TaMAPKKK131	0.01	0.03	0.28	2580.00	621.58	1958.42	Purifying selection	2.66
TraesCS6A02G187400	/	TraesCS6B02G217100	TaMAPKKK155	0.07	0.09	0.72	909.00	217.67	691.33	Purifying selection	7.14
TraesCS6A02G245000	TaMAPKKK4	TraesCS6B02G279300	TaMAPKKK4-1	0.01	0.04	0.17	2358.00	568.58	1789.42	Purifying selection	3.06
TraesCS6A02G245000	TaMAPKKK4	TraesCS6D02G227300	/	0.01	0.04	0.20	2634.00	635.83	1998.17	Purifying selection	2.72

TraesCS6A02G255100	TaMAPKKK39	TraesCS6B02G270400	TaMAPKKK33	0.03	0.08	0.39	1848.00	419.25	1428.75	Purifying selection	5.99
TraesCS6A02G255100	TaMAPKKK39	TraesCS6D02G236400	TaMAPKKK31	0.01	0.04	0.19	1845.00	418.83	1426.17	Purifying selection	3.40
TraesCS6A02G269400	TaMAPK37	TraesCS6B02G296700	TaMAPK1	0.01	0.05	0.13	1380.00	326.00	1054.00	Purifying selection	3.90
TraesCS6A02G269400	TaMAPK37	TraesCS6D02G245500	TaMAPK13	0.00	0.04	0.12	1380.00	326.17	1053.83	Purifying selection	3.15
TraesCS6A02G276300	/	TraesCS7D02G230200	TaMAPKKK51	0.18	0.36	0.50	1206.00	295.58	910.42	Purifying selection	27.65
TraesCS6A02G290500	/	TraesCS6B02G320800	TaMAPKKK129	0.08	0.12	0.61	2223.00	518.83	1704.17	Purifying selection	9.57
TraesCS6A02G353400	TaMAPKKKK16	TraesCS6B02G386100	TaMAPKKKK19	0.01	0.06	0.20	1044.00	243.17	800.83	Purifying selection	4.89
TraesCS6A02G353400	TaMAPKKKK16	TraesCS6D02G335800	TaMAPKKKK20	0.01	0.07	0.19	1044.00	242.83	801.17	Purifying selection	5.25
TraesCS6B02G127800	TaMAPK17	TraesCS6D02G082900	TaMAPK44	0.03	0.09	0.31	1494.00	349.50	1144.50	Purifying selection	6.76
TraesCS6B02G146300	TaMAPK10	TraesCS6D02G108100	TaMAPK45	0.00	0.07	0.02	1107.00	254.67	852.33	Purifying selection	5.05
TraesCS6B02G146300	TaMAPK10	TraesCS7A02G422500	TaMAPK12	0.04	0.98	0.05	1107.00	254.42	852.58	Purifying selection	75.03
TraesCS6B02G146300	TaMAPK10	TraesCS7B02G322900	TaMAPK54	0.05	1.03	0.04	1107.00	254.08	852.92	Purifying selection	79.27
TraesCS6B02G146300	TaMAPK10	TraesCS7D02G414700	/	0.05	1.03	0.04	1107.00	254.42	852.58	Purifying selection	79.05
TraesCS6B02G177800	TaMAPKKKK18	TraesCS6D02G139200	TaMAPKKK23	0.01	0.09	0.13	1527.00	339.00	1188.00	Purifying selection	7.12
TraesCS6B02G183300	/	TraesCS7D02G474700	TaMAPKKK102	0.09	0.80	0.11	1254.00	292.25	961.75	Purifying selection	61.35
TraesCS6B02G200100	/	TraesCS7D02G384700	TaMAPKKK61	0.19	0.59	0.31	1014.00	243.50	770.50	Purifying selection	45.64
TraesCS6B02G215100	TaMAPKKK131	TraesCS6D02G173100	/	0.01	0.04	0.28	2577.00	620.17	1956.83	Purifying selection	2.80
TraesCS6B02G217100	TaMAPKKK155	TraesCS6D02G174300	/	0.09	0.13	0.67	948.00	225.58	722.42	Purifying selection	9.79

TraesCS6B02G217100	TaMAPKKK155	TraesCS7B02G270900	/	0.26	3.26	0.08	879.00	213.33	665.67	Purifying selection	250.45
TraesCS6B02G270400	TaMAPKKK33	TraesCS6D02G236400	TaMAPKKK31	0.03	0.07	0.41	1848.00	418.75	1429.25	Purifying selection	5.19
TraesCS6B02G279300	TaMAPKKK4-1	TraesCS6D02G227300	/	0.01	0.02	0.27	2358.00	570.67	1787.33	Purifying selection	1.78
TraesCS6B02G296700	TaMAPK1	TraesCS6D02G245500	TaMAPK13	0.00	0.03	0.14	1380.00	326.17	1053.83	Purifying selection	2.16
TraesCS6B02G303800	/	TraesCS7D02G230200	TaMAPKKK51	0.17	0.43	0.39	1218.00	297.92	920.08	Purifying selection	32.85
TraesCS6B02G320800	TaMAPKKK129	TraesCS6D02G271500	/	0.07	0.15	0.49	2952.00	706.42	2245.58	Purifying selection	11.39
TraesCS6B02G379500	/	TraesCS6D02G328800	TaMAPKK1	0.01	0.10	0.07	1104.00	300.08	803.92	Purifying selection	7.81
TraesCS6B02G386100	TaMAPKKK19	TraesCS6D02G335800	TaMAPKKK20	0.00	0.04	0.09	2085.00	477.42	1607.58	Purifying selection	3.32
TraesCS6D02G108100	TaMAPK45	TraesCS7A02G422500	TaMAPK12	0.05	1.00	0.05	1107.00	254.75	852.25	Purifying selection	76.70
TraesCS6D02G108100	TaMAPK45	TraesCS7B02G322900	TaMAPK54	0.05	1.07	0.04	1107.00	254.42	852.58	Purifying selection	82.31
TraesCS6D02G108100	TaMAPK45	TraesCS7D02G414700	/	0.05	1.02	0.05	1107.00	254.75	852.25	Purifying selection	78.43
TraesCS6D02G145100	/	TraesCS7D02G474700	TaMAPKKK102	0.09	0.77	0.11	1254.00	292.75	961.25	Purifying selection	59.29
TraesCS6D02G161600	/	TraesCS7D02G384700	TaMAPKKK61	0.21	0.58	0.37	1098.00	267.67	830.33	Purifying selection	44.75
TraesCS6D02G256700	/	TraesCS7D02G230200	TaMAPKKK51	0.18	0.36	0.49	1215.00	297.58	917.42	Purifying selection	27.76
TraesCS6D02G328800	TaMAPKK1	TraesCS7D02G155100	/	0.12	0.42	0.29	1035.00	278.50	756.50	Purifying selection	31.96
TraesCS7A02G032700	TaMAPKKK107	TraesCS7D02G029300	/	0.01	0.06	0.22	1440.00	336.25	1103.75	Purifying selection	4.52
TraesCS7A02G049000	TaMAPK46	TraesCS7D02G044100	TaMAPK18	0.01	0.09	0.15	1452.00	345.42	1106.58	Purifying selection	6.85
TraesCS7A02G111300	TaMAPK19	TraesCS7B02G009200	/	0.00	0.09	0.04	1179.00	272.42	906.58	Purifying selection	7.21

TraesCS7A02G111300	TaMAPK19	TraesCS7D02G106400	/	0.00	0.06	0.05	1179.00	272.50	906.50	Purifying selection	4.70
TraesCS7A02G229600	/	TraesCS7D02G230200	TaMAPKKK51	0.00	0.03	0.13	1233.00	304.50	928.50	Purifying selection	2.58
TraesCS7A02G232300	TaMAPKKKK22	TraesCS7B02G130700	TaMAPKKKK23	0.01	0.06	0.12	2100.00	482.33	1617.67	Purifying selection	4.48
TraesCS7A02G232300	TaMAPKKKK22	TraesCS7D02G232400	TaMAPKKKK24	0.02	0.05	0.37	2067.00	476.33	1590.67	Purifying selection	3.55
TraesCS7A02G326700	TaMAPKKK103	TraesCS7B02G227300	/	0.56	1.01	0.55	537.00	127.08	409.92	Purifying selection	77.84
TraesCS7A02G326700	TaMAPKKK103	TraesCS7D02G323500	/	0.02	0.06	0.28	1602.00	374.58	1227.42	Purifying selection	4.48
TraesCS7A02G335300	TaMAPK3	TraesCS7B02G246900	TaMAPK52	0.00	0.04	0.05	1263.00	287.50	975.50	Purifying selection	3.30
TraesCS7A02G335300	TaMAPK3	TraesCS7D02G342800	TaMAPK7	0.01	0.04	0.14	1263.00	287.67	975.33	Purifying selection	2.74
TraesCS7A02G388800	/	TraesCS7D02G384700	TaMAPKKK61	0.00	0.03	0.03	1188.00	286.33	901.67	Purifying selection	2.47
TraesCS7A02G410700	TaMAPK49	TraesCS7B02G309900	TaMAPK53	0.00	0.10	0.03	1734.00	409.08	1324.92	Purifying selection	8.06
TraesCS7A02G410700	TaMAPK49	TraesCS7D02G403700	TaMAPK20	0.00	0.07	0.05	1734.00	409.83	1324.17	Purifying selection	5.72
TraesCS7A02G422500	TaMAPK12	TraesCS7B02G322900	TaMAPK54	0.01	0.12	0.10	1131.00	260.33	870.67	Purifying selection	8.93
TraesCS7A02G422500	TaMAPK12	TraesCS7D02G414700	/	0.00	0.08	0.04	1107.00	254.50	852.50	Purifying selection	5.88
TraesCS7A02G488200	/	TraesCS7D02G474700	TaMAPKKK102	0.00	0.16	0.01	1275.00	299.08	975.92	Purifying selection	12.28
TraesCS7B02G130700	TaMAPKKKK23	TraesCS7D02G232400	TaMAPKKKK24	0.02	0.09	0.21	2067.00	476.50	1590.50	Purifying selection	6.55
TraesCS7B02G195500	/	TraesCS7D02G230200	TaMAPKKK51	0.00	0.07	0.02	1233.00	303.83	929.17	Purifying selection	5.02
TraesCS7B02G246900	TaMAPK52	TraesCS7D02G342800	TaMAPK7	0.00	0.03	0.11	1263.00	287.67	975.33	Purifying selection	2.18
TraesCS7B02G290700	/	TraesCS7D02G384700	TaMAPKKK61	0.00	0.05	0.05	1188.00	286.67	901.33	Purifying selection	3.60

TraesCS7B02G309900	TaMAPK53	TraesCS7D02G403700	TaMAPK20	0.01	0.09	0.06	1734.00	409.92	1324.08	Purifying selection	6.97
TraesCS7B02G322900	TaMAPK54	TraesCS7D02G414700	/	0.00	0.10	0.04	1107.00	254.17	852.83	Purifying selection	7.42
TraesCS7B02G391300	/	TraesCS7D02G474700	TaMAPKKK102	0.00	0.09	0.02	1275.00	299.42	975.58	Purifying selection	6.81
TraesCS7B02G424500	/	TraesCS7D02G503600	TaMAPKKK54	0.14	0.35	0.41	1350.00	304.25	1045.75	Purifying selection	26.78
TraesCS7B02G425200	/	TraesCS7D02G503600	TaMAPKKK54	0.13	0.46	0.28	1269.00	295.83	973.17	Purifying selection	35.76
TraesCS7B02G427800	/	TraesCS7D02G503600	TaMAPKKK54	0.14	0.42	0.34	1974.00	459.42	1514.58	Purifying selection	32.09
TraesCS7B02G430300	/	TraesCS7D02G503600	TaMAPKKK54	0.14	0.40	0.34	2136.00	493.08	1642.92	Purifying selection	30.81
