# Supplementary material for: Bioinformatic Identification and Expression Analyses of the MAPK–MAP4K Gene Family Reveal a Putative Functional MAP4K10-MAP3K7/8-MAP2K1/11-MAPK3/6 Cascade in Wheat (Triticum aestivum L.)
Source: Plants (Basel). 2024 Mar 24;13(7):941. doi: 10.3390/plants13070941 (PMC11013086; doi:10.3390/plants13070941)
Supplement: Supplementary file 1 [file plants-13-00941-s001.zip › plants-2867660-supplementary/Supplementary Figure S1-S10 and Supplementary table S1-S7/Supplementary table S1-S7/Supplementary table 3.pdf]

The Ka/Ks ratio and divergence times between MAPK-MAP4K genes in wheat and *T.dicoccoides*															
**Gene Name**	**Gene Name**	**Gene ID**	**Gene ID**	**Ka**	**Ks**	**Ka/Ks**	**EffectiveLen**	**AverageS-sites**	**Average N-sites**	**cN**	**cS**	**pN**	**pS**	**Divergence times (Mya)**
TaMAPK1		Ta--TraesCS6B02G296700.2	Td--TRIDC6AG041340.3	0.00742	0.05016795112393 05	0.14799	1236	288.5	947.5	7	14	0.007	0.049	3.85907316
TaMAPK1		Ta--TraesCS6B02G296700.2	Td--TRIDC6BG048300.1	0.00104	0		1254	292.67	961.333	1	0	0.001	0	0
TaMAPK2		Ta--TraesCS4A02G336800.2	Td--TRIDC5BG079810.1	0.01875	0.07521017361124 73	0.249335237585 456	1320	312.08	1007.92	18.67	22.33	0.019	0.072	5.78539797
TaMAPK3		Ta--TraesCS4A02G106400.1	Td--TRIDC4AG014520.1	0	0.00391901610199 883	0	1098	255.83	842.167	0	1	0	0.004	0.30146278
TaMAPK3		Ta--TraesCS4A02G106400.1	Td--TRIDC4BG035610.1	0	0.08171338784141 59	0	1107	258.33	848.667	0	20	0	0.077	6.28564522
TaMAPK4		Ta--TraesCS1D02G088000.2	Td--TRIDC1AG012310.2	0.00637	0.10236857568974 9	0.062270480206 5927	1647	386.67	1260.33	8	37	0.006	0.096	7.87450582
TaMAPK4		Ta--TraesCS1D02G088000.2	Td--TRIDC1BG015140.6	0.00398	0.10499874460090 9	0.037918828505 5864	1647	387.83	1259.17	5	38	0.004	0.098	8.07682651

TaMAPK5	Ta--TraesCS1D02G422800.1	Td--TRIDC1AG061100.1	0.05941	0.153051965259095	0.388161796728085	1686	402.08	1283.92	73.33	55.67	0.057	0.138	11.7732281
TaMAPK5	Ta--TraesCS1D02G422800.1	Td--TRIDC1BG070050.1	0.06018	0.157857730345453	0.381246126850021	1680	400.42	1279.58	74	57	0.058	0.142	12.1429023
TaMAPK5	Ta--TraesCS1D02G422800.1	Td--TRIDC3AG034650.7	0.14228	1.25243872352443	0.113600673522565	1470	337	1133	146.8	205.2	0.13	0.609	96.3414403
TaMAPK5	Ta--TraesCS1D02G422800.1	Td--TRIDC3BG039070.12	0.13336	1.3757077464143	0.0969425812673929	1482	344.33	1137.67	139	217	0.122	0.63	105.823673
TaMAPK6	Ta--TraesCS7B02G009200.1	Td--TRIDC7AG012990.2	0.12506	0.306027017934581	0.408641154673493	1032	243.42	788.583	90.83	61.17	0.115	0.251	23.5405398
TaMAPK6	Ta--TraesCS7B02G009200.1	Td--TRIDC7BG001200.2	0	0	NaN	1077	246.33	830.667	0	0	0	0	0
TaMAPK7	Ta--TraesCS7D02G342800.3	Td--TRIDC7AG047060.4	0.00514	0.0392479554227689	0.131065504737014	1263	287.67	975.333	5	11	0.005	0.038	3.01907349
TaMAPK7	Ta--TraesCS7D02G342800.3	Td--TRIDC7BG040680.1	0.00308	0.0283386721709847	0.108762898278818	1263	287.67	975.333	3	8	0.003	0.028	2.17989786

TaMAPK8	Ta--TraesCS3D02G225600.1	Td--TRIDC1AG061940.2	0.14901	1.03601193131669	0.143829560602657	1767	420.25	1346.75	182	236	0.135	0.562	79.6932255
TaMAPK8	Ta--TraesCS3D02G225600.1	Td--TRIDC1BG071030.1	0.14681	1.10004784487189	0.133456406945343	1770	423.75	1346.25	179.5	244.5	0.133	0.577	84.619065
TaMAPK8	Ta--TraesCS3D02G225600.1	Td--TRIDC3AG036190.14	0.19656	2.89929980159109	0.067795825020655	1707	395.17	1311.83	226.8	290.2	0.173	0.734	223.023062
TaMAPK8	Ta--TraesCS3D02G225600.1	Td--TRIDC3AG034020.1	0.00142	0.0451532097387966	0.0315463134457483	1839	433.58	1405.42	2	19	0.001	0.044	3.47332383
TaMAPK8	Ta--TraesCS3D02G225600.1	Td--TRIDC3BG040600.18	0.2013	2.44373001620979	0.0823738956703154	1722	396.58	1325.42	234	286	0.177	0.721	187.979232
TaMAPK8	Ta--TraesCS3D02G225600.1	Td--TRIDC3BG038510.2	0.00357	0.0281173106948522	0.126943531733047	1839	434.83	1404.17	5	12	0.004	0.028	2.16287005
TaMAPK10	Ta--TraesCS6B02G146300.1	Td--TRIDC6AG015690.2	0	0.096232086482528	0	1107	254.67	852.333	0	23	0	0.09	7.40246819
TaMAPK10	Ta--TraesCS6B02G146300.1	Td--TRIDC6BG021470.2	0	0.0118814365992434	0	1107	254.5	852.5	0	3	0	0.012	0.91395666

TaMAPK10	Ta--TraesCS6B02G146300.1	Td--TRIDC7AG058780.3	0.04491	0.98990566347023 4	0.045369209752 9397	1107	254.42	852.583	37.17	139.8	0.044	0.55	76.1465895
TaMAPK10	Ta--TraesCS6B02G146300.1	Td--TRIDC7BG051510.2	0.04552	1.00003826810639	0.045514051473 1277	1107	254.08	852.917	37.67	140.3	0.044	0.552	76.9260206
TaMAPK11	Ta--TraesCS1A02G086500.1	Td--TRIDC1AG012310.2	0.00079	0.00259291528789 334	0.306084379777 707	1647	386.33	1260.67	1	1	0.001	0.003	0.19945502
TaMAPK11	Ta--TraesCS1A02G086500.1	Td--TRIDC1BG015140.6	0.00478	0.09624168715841 6	0.049656119211 2174	1647	387.5	1259.5	6	35	0.005	0.09	7.4032067
TaMAPK12	Ta--TraesCS7A02G422500.1	Td--TRIDC6AG015690.2	0.05572	1.05263760077497	0.052936062441 8997	1134	261.92	872.083	46.83	148.2	0.054	0.566	80.9721231
TaMAPK12	Ta--TraesCS7A02G422500.1	Td--TRIDC6BG021470.2	0.06406	0.99781042704407 4	0.064200307641 729	1161	270.67	890.333	54.67	149.3	0.061	0.552	76.7546482
TaMAPK12	Ta--TraesCS7A02G422500.1	Td--TRIDC7AG058780.3	0.02452	0.02921313302862 25	0.839272417346 312	1152	267.58	884.417	21.33	7.667	0.024	0.029	2.24716408
TaMAPK12	Ta--TraesCS7A02G422500.1	Td--TRIDC7BG051510.2	0.00963	0.08987399964540 92	0.107122380206 223	1236	295.17	940.833	9	25	0.01	0.085	6.91338459

TaMAPK13	Ta--TraesCS6D02G245500.3	Td--TRIDC6AG041340.3	0.0053	0.0427669289697659	0.123848784815936	1236	288.67	947.333	5	12	0.005	0.042	3.28976377
TaMAPK13	Ta--TraesCS6D02G245500.3	Td--TRIDC6BG048300.1	0.00313	0.0278292795404714	0.11238952696524	1254	292.83	961.167	3	8	0.003	0.027	2.14071381
TaMAPK14	Ta--TraesCS1A02G184500.1	Td--TRIDC1AG027930.1	0	0	NaN	1044	244.5	799.5	0	0	0	0	0
TaMAPK14	Ta--TraesCS1A02G184500.1	Td--TRIDC1BG032170.1	0.00233	0.0704099735380411	0.0330740418354893	1128	267.83	860.167	2	18	0.002	0.067	5.41615181
TaMAPK16	Ta--TraesCS3B02G270200.1	Td--TRIDC1AG059290.6	0.11603	1.06436257300333	0.10900910751906	1587	365.25	1221.75	131.3	207.7	0.107	0.569	81.8740441
TaMAPK16	Ta--TraesCS3B02G270200.1	Td--TRIDC1BG068060.2	0.13001	1.2134906196101	0.107138444752693	1647	378.5	1268.5	151.4	227.6	0.119	0.601	93.3454323
TaMAPK16	Ta--TraesCS3B02G270200.1	Td--TRIDC3AG036190.14	0.01956	0.0544862269973564	0.3589096029114	1752	396.42	1355.58	26.17	20.83	0.019	0.053	4.19124823
TaMAPK16	Ta--TraesCS3B02G270200.1	Td--TRIDC3AG034020.1	0.19677	2.75912183869536	0.0713173284307232	1722	396	1326	229.5	289.5	0.173	0.731	212.240141

TaMAPK16	Ta--TraesCS3B02G270200.1	Td--TRIDC3BG040600.18	0.01727	0.0149152642820377	1.15782553175512	1752	395	1357	23.17	5.833	0.017	0.015	1.14732802
TaMAPK16	Ta--TraesCS3B02G270200.1	Td--TRIDC3BG038510.2	0.19226	2.43413962795926	0.0789831845357364	1722	397.25	1324.75	224.7	286.3	0.17	0.721	187.24151
TaMAPK17	Ta--TraesCS6B02G127800.1	Td--TRIDC6BG018000.3	0.02338	0.0348020073141141	0.671757693922535	1446	338.17	1107.83	25.5	11.5	0.023	0.034	2.67707749
TaMAPK18	Ta--TraesCS7D02G044100.1	Td--TRIDC4AG065120.1	0.01415	0.0814097621275007	0.173855279698641	1449	343.5	1105.5	15.5	26.5	0.014	0.077	6.26228939
TaMAPK18	Ta--TraesCS7D02G044100.1	Td--TRIDC7AG005070.14	0.0146	0.0890387506427807	0.163974799304442	1452	345.42	1106.58	16	29	0.014	0.084	6.84913466
TaMAPK19	Ta--TraesCS7A02G111300.1	Td--TRIDC7AG012990.2	0.12238	0.20642145853537	0.592883157990451	1032	243.83	788.167	89	44	0.113	0.18	15.8785737
TaMAPK19	Ta--TraesCS7A02G111300.1	Td--TRIDC7BG001200.2	0.0012	0.0951942122214532	0.0126526258242493	1077	246.08	830.917	1	22	0.001	0.089	7.32263171
TaMAPK20	Ta--TraesCS7D02G403700.1	Td--TRIDC7AG057270.3	0.00454	0.0716351513763928	0.0634450338639796	1734	409.83	1324.17	6	28	0.005	0.068	5.51039626

TaMAPK20	Ta--TraesCS7D02G403700.1	Td--TRIDC7BG049670.7	0.00531	0.09064693841771 07	0.058528142988 5363	1734	409.92	1324.08	7	35	0.005	0.085	6.97284142
TaMAPK23	Ta--TraesCS3D02G221700.1	Td--TRIDC1AG061100.1	0.12144	1.29162944784723	0.094023009198 9617	1428	327.25	1100.75	123.4	201.6	0.112	0.616	99.3561114
TaMAPK23	Ta--TraesCS3D02G221700.1	Td--TRIDC1BG070050.1	0.12329	1.27516804392155	0.096682257209 2444	1428	327.75	1100.25	125.1	200.9	0.114	0.613	98.0898495
TaMAPK23	Ta--TraesCS3D02G221700.1	Td--TRIDC3AG034650.7	0.02343	0.07484782846617 32	0.313043225405 584	1464	336.92	1127.08	26	24	0.023	0.071	5.75752527
TaMAPK23	Ta--TraesCS3D02G221700.1	Td--TRIDC3BG039070.12	0.02064	0.07621556842761 55	0.270859093568 191	1446	336.92	1109.08	22.58	24.42	0.02	0.072	5.86273603
TaMAPK24	Ta--TraesCS3D02G242200.2	Td--TRIDC1AG059290.6	0.11534	1.0223955958083	0.112815898147 014	1584	364.92	1219.08	130.3	203.7	0.107	0.558	78.6458151
TaMAPK24	Ta--TraesCS3D02G242200.2	Td--TRIDC1BG068060.2	0.13027	1.21194965965697	0.107487091284 717	1641	377.58	1263.42	151.1	226.9	0.12	0.601	93.2268969
TaMAPK24	Ta--TraesCS3D02G242200.2	Td--TRIDC3AG036190.14	0.00264	0.02201280150770 55	0.120014005113 195	1719	391.83	1327.17	3.5	8.5	0.003	0.022	1.69329242

TaMAPK24	Ta--TraesCS3D02G242200.2	Td--TRIDC3AG034020.1	0.19625	3.61641987712469	0.0542654779116772	1722	396.75	1325.25	228.8	295.2	0.173	0.744	278.186144
TaMAPK24	Ta--TraesCS3D02G242200.2	Td--TRIDC3BG040600.18	0.01158	0.0511673778753061	0.226337143047591	1743	394.25	1348.75	15.5	19.5	0.011	0.049	3.93595214
TaMAPK24	Ta--TraesCS3D02G242200.2	Td--TRIDC3BG038510.2	0.19173	2.87022581694835	0.0667995423121429	1722	398	1324	224	292	0.169	0.734	220.786601
TaMAPK25	Ta--TraesCS4D02G198600.1	Td--TRIDC4AG014520.1	0.00119	0.0609678778859364	0.0194992073648951	1098	256.17	841.833	1	15	0.001	0.059	4.68983676
TaMAPK25	Ta--TraesCS4D02G198600.1	Td--TRIDC4BG035610.1	0.00118	0.0687813198671241	0.0171515936394262	1107	258.67	848.333	1	17	0.001	0.066	5.29087076
TaMAPK26	Ta--TraesCS5D02G534000.2	Td--TRIDC5BG079810.1	0.012	0.0634946176153795	0.189012180999085	1320	312.08	1007.92	12	19	0.012	0.061	4.88420136
TaMAPK27	Ta--TraesCS1B02G104900.1	Td--TRIDC1AG012310.2	0.00638	0.102132733646731	0.0624557452302269	1647	387.5	1259.5	8	37	0.006	0.095	7.85636413
TaMAPK27	Ta--TraesCS1B02G104900.1	Td--TRIDC1BG015140.6	0.0008	0.0025773221239359	0.308507544009172	1647	388.67	1258.33	1	1	0.001	0.003	0.19825555

TaMAPK28	Ta--TraesCS1B02G192600.3	Td--TRIDC1AG027930.1	0	0.0728623919963763	0	1044	244.83	799.167	0	17	0	0.069	5.60479938
TaMAPK28	Ta--TraesCS1B02G192600.3	Td--TRIDC1BG032170.1	0	0	NaN	1128	268.17	859.833	0	0	0	0	0
TaMAPK30	Ta--TraesCS7A02G335300.2	Td--TRIDC7AG047060.4	0	0.00348635142598846	0	1263	287.5	975.5	0	1	0	0.003	0.26818088
TaMAPK30	Ta--TraesCS7A02G335300.2	Td--TRIDC7BG040680.1	0.00205	0.0429455406475612	0.0478056169421855	1263	287.5	975.5	2	12	0.002	0.042	3.30350313
TaMAPK31	Ta--TraesCS4A02G434800.1	Td--TRIDC4AG065120.1	0.0009	0.00291121180663198	0.310251040394682	1452	344.17	1107.83	1	1	0.001	0.003	0.22393937
TaMAPK31	Ta--TraesCS4A02G434800.1	Td--TRIDC7AG005070.14	0.01833	0.0990874493794773	0.185029679252049	1449	344.75	1104.25	20	32	0.018	0.093	7.62211149
TaMAPK33	Ta--TraesCS1D02G410100.1	Td--TRIDC1BG068060.2	0.00948	0.112849792914552	0.0840169518000014	1665	391.33	1273.67	12	41	0.009	0.105	8.6807533
TaMAPK33	Ta--TraesCS1D02G410100.1	Td--TRIDC3AG036190.14	0.12441	1.23950861579893	0.100372044934739	1632	378.08	1253.92	143.8	229.2	0.115	0.606	95.3468166

TaMAPK33	Ta--TraesCS1D02G410100.1	Td--TRIDC3BG040600.18	0.12406	1.27739892174097	0.0971176718441689	1629	376.17	1252.83	143.3	230.7	0.114	0.613	98.2614555
TaMAPK34	Ta--TraesCS1D02G428900.1	Td--TRIDC1AG061940.2	0.00813	0.0990106916882497	0.0821493881080352	1791	431.25	1359.75	11	40	0.008	0.093	7.61620705
TaMAPK34	Ta--TraesCS1D02G428900.1	Td--TRIDC1BG071030.1	0.00664	0.100764797139255	0.0658602602559447	1797	434.83	1362.17	9	41	0.007	0.094	7.75113824
TaMAPK34	Ta--TraesCS1D02G428900.1	Td--TRIDC3AG034020.1	0.15651	1.01117136838978	0.154783225167804	1770	420.25	1349.75	190.7	233.3	0.141	0.555	77.782413
TaMAPK34	Ta--TraesCS1D02G428900.1	Td--TRIDC3BG038510.2	0.15439	1.00940089350214	0.152954661439063	1770	421.5	1348.5	188.2	233.8	0.14	0.555	77.6462226
TaMAPK35	Ta--TraesCS6A02G099600.1	Td--TRIDC6BG018000.3	0.02935	0.0617791840474554	0.47508437752357	1449	337.25	1111.75	32	20	0.029	0.059	4.75224493
TaMAPK36	Ta--TraesCS6A02G118100.1	Td--TRIDC6AG015690.2	0.00117	0.00787925828566064	0.149107725072188	1107	255.17	851.833	1	2	0.001	0.008	0.60609679
TaMAPK36	Ta--TraesCS6A02G118100.1	Td--TRIDC6BG021470.2	0.00117	0.0872351457577674	0.0134650806662304	1107	255	852	1	21	0.001	0.082	6.71039583

TaMAPK36	Ta--TraesCS6A02G118100.1	Td--TRIDC7AG058780.3	0.04619	1.04669985672484	0.044124870900 0351	1107	254.92	852.083	38.17	143.8	0.045	0.564	80.5153736
TaMAPK36	Ta--TraesCS6A02G118100.1	Td--TRIDC7BG051510.2	0.04679	1.10759507837085	0.0422455256133219	1107	254.58	852.417	38.67	147.3	0.045	0.579	85.1996214
TaMAPK37	Ta--TraesCS6A02G269400.1	Td--TRIDC6AG041340.3	0	0	NaN	1236	288.5	947.5	0	0	0	0	0
TaMAPK37	Ta--TraesCS6A02G269400.1	Td--TRIDC6BG048300.1	0.00627	0.0494296489342025	0.126795275271909	1254	292.67	961.333	6	14	0.006	0.048	3.80228069
TaMAPK38	Ta--TraesCS3B02G256700.1	Td--TRIDC1AG061940.2	0.14718	1.02024043069682	0.144261217630693	1761	419.08	1341.92	179.3	233.7	0.134	0.558	78.4800331
TaMAPK38	Ta--TraesCS3B02G256700.1	Td--TRIDC1BG071030.1	0.14498	1.10354201974222	0.131374418531363	1764	422.58	1341.42	176.8	244.2	0.132	0.578	84.8878477
TaMAPK38	Ta--TraesCS3B02G256700.1	Td--TRIDC3AG034020.1	0.00218	0.0460285687371147	0.0472839308150044	1806	425.58	1380.42	3	19	0.002	0.045	3.54065913
TaMAPK38	Ta--TraesCS3B02G256700.1	Td--TRIDC3AG036190.14	0.19047	2.94021897914399	0.0647806723151071	1677	387.92	1289.08	216.8	285.2	0.168	0.735	226.170691

TaMAPK38	Ta--TraesCS3B02G256700.1	Td--TRIDC3BG038510.2	0.00145	0		1806	426.83	1379.17	2	0	0.001	0	0
TaMAPK38	Ta--TraesCS3B02G256700.1	Td--TRIDC3BG040600.18	0.19532	2.5306265319557	0.0771809137839172	1692	389.33	1302.67	224	282	0.172	0.724	194.663579
TaMAPK39	Ta--TraesCS3A02G242100.1	Td--TRIDC1AG059290.6	0.12871	1.02630359727683	0.125412317214093	1608	369.33	1238.67	146.5	206.5	0.118	0.559	78.9464306
TaMAPK39	Ta--TraesCS3A02G242100.1	Td--TRIDC3AG036190.14	0.00264	0.00641166546451419	0.411959428703249	1719	391.58	1327.42	3.5	2.5	0.003	0.006	0.49320504
TaMAPK39	Ta--TraesCS3A02G242100.1	Td--TRIDC3AG034020.1	0.19346	2.99299959956051	0.0646376983010901	1722	396.67	1325.33	226	292	0.171	0.736	230.230738
TaMAPK39	Ta--TraesCS3A02G242100.1	Td--TRIDC3BG040600.18	0.00415	0.0327134279906905	0.126904346259797	1719	390.5	1328.5	5.5	12.5	0.004	0.032	2.51641754
TaMAPK39	Ta--TraesCS3A02G242100.1	Td--TRIDC3BG038510.2	0.18896	2.57727262393975	0.073317037835 7298	1722	397.92	1324.08	221.2	288.8	0.167	0.726	198.25174
TaMAPK40	Ta--TraesCS3A02G231700.1	Td--TRIDC1AG061100.1	0.13542	1.23525197428024	0.109628540014771	1443	328.5	1114.5	138.1	198.9	0.124	0.606	95.0193826

TaMAPK40	Ta--TraesCS3A02G231700.1	Td--TRIDC1BG070050.1	0.13728	1.26799751426752	0.108265165489214	1443	329	1114	139.8	201.3	0.125	0.612	97.5382703
TaMAPK40	Ta--TraesCS3A02G231700.1	Td--TRIDC3AG034650.7	0.00088	0.00298211128472955	0.2958819972499 1	1470	336	1134	1	1	0.001	0.003	0.22939318
TaMAPK40	Ta--TraesCS3A02G231700.1	Td--TRIDC3BG039070.12	0.03908	0.0972947069609999	0.401692319318875	1449	335.17	1113.83	42.42	30.58	0.038	0.091	7.48420823
TaMAPK42	Ta--TraesCS1A02G415300.1	Td--TRIDC1AG061100.1	0.05393	0.13957788828226	0.386410524665208	1686	401.75	1284.25	66.83	51.17	0.052	0.127	10.7367606
TaMAPK42	Ta--TraesCS1A02G415300.1	Td--TRIDC1BG070050.1	0.06061	0.153177502270747	0.395713402283722	1680	400.58	1279.42	74.5	55.5	0.058	0.139	11.7828848
TaMAPK42	Ta--TraesCS1A02G415300.1	Td--TRIDC3AG034650.7	0.14524	1.27658932962714	0.113775712787159	1473	337.67	1135.33	149.9	207.1	0.132	0.613	98.1991792
TaMAPK42	Ta--TraesCS1A02G415300.1	Td--TRIDC3BG039070.12	0.13028	1.33481156665734	0.0976045111485081	1482	344.83	1137.17	136	215	0.12	0.623	102.677813
TaMAPK43	Ta--TraesCS1A02G421000.1	Td--TRIDC1AG061940.2	0	0.00463143115923732	0	1794	433.17	1360.83	0	2	0	0.005	0.35626394

TaMAPK43	Ta--TraesCS1A02G421000.1	Td--TRIDC1BG071030.1	0.01037	0.127568052158476	0.0812991626219065	1794	434.75	1359.25	14	51	0.01	0.117	9.81292709
TaMAPK43	Ta--TraesCS1A02G421000.1	Td--TRIDC3AG034020.1	0.15032	0.9966204228277430	0.1508272020576 2	1767	419.83	1347.17	183.5	231.5	0.136	0.551	76.6631094
TaMAPK43	Ta--TraesCS1A02G421000.1	Td--TRIDC3BG038510.2	0.14905	0.9973970366294370	0.149438936255203	1767	420.58	1346.42	182	232	0.135	0.552	76.722849
TaMAPK44	Ta--TraesCS6D02G082900.2	Td--TRIDC6BG018000.3	0.02699	0.0733432163085657	0.368034708880667	1449	336.33	1112.67	29.5	23.5	0.027	0.07	5.64178587
TaMAPK45	Ta--TraesCS6D02G108100.1	Td--TRIDC6AG015690.2	0.00117	0.105066594878011	0.0111798452773931	1107	255	852	1	25	0.001	0.098	8.08204576
TaMAPK45	Ta--TraesCS6D02G108100.1	Td--TRIDC6BG021470.2	0.00117	0.0528315849197334	0.0222290957711989	1107	254.83	852.167	1	13	0.001	0.051	4.06396807
TaMAPK45	Ta--TraesCS6D02G108100.1	Td--TRIDC7AG058780.3	0.04659	1.01202457519198	0.0460386591681408	1107	254.75	852.25	38.5	141.5	0.045	0.555	77.8480442
TaMAPK45	Ta--TraesCS6D02G108100.1	Td--TRIDC7BG051510.2	0.0472	1.03800311918125	0.0454697531536604	1107	254.42	852.583	39	143	0.046	0.562	79.8463938

TaMAPK46	Ta--TraesCS7A02G049000.1	Td--TRIDC4AG065120.1	0.01833	0.0990874493794773	0.185029679252049	1449	344.75	1104.25	20	32	0.018	0.093	7.62211149
TaMAPK46	Ta--TraesCS7A02G049000.1	Td--TRIDC7AG005070.14	0.00181	0.00289017698699247	0.626811123282763	1452	346.67	1105.33	2	1	0.002	0.003	0.22232131
TaMAPK47	Ta--TraesCS7A02G029700.1	Td--TRIDC7AG002740.1	0.02167	0.0320781997245268	0.675520783767096	972	222.92	749.083	16	7	0.021	0.031	2.46755382
TaMAPK49	Ta--TraesCS7A02G410700.2	Td--TRIDC7AG057270.3	0.00076	0.00244898176780432	0.308330987928357	1734	409	1325	1	1	0.001	0.002	0.18838321
TaMAPK49	Ta--TraesCS7A02G410700.2	Td--TRIDC7BG049670.7	0.00303	0.104767985475068	0.0288747618679432	1734	409.08	1324.92	4	40	0.003	0.098	8.05907581
TaMAPK50	Ta--TraesCS5B02G536500.1	Td--TRIDC5BG079810.1	0.00099	0.00955426933307228	0.103390121521531	1329	316	1013	1	3	0.001	0.009	0.73494379
TaMAPK52	Ta--TraesCS7B02G246900.3	Td--TRIDC7AG047060.4	0.00205	0.046637855797338	0.0440208502400651	1263	287.5	975.5	2	13	0.002	0.045	3.58752737
TaMAPK52	Ta--TraesCS7B02G246900.3	Td--TRIDC7BG040680.1	0	0.00698898476240196	0	1263	287.5	975.5	0	2	0	0.007	0.53761421

TaMAPK53	Ta--TraesCS7B02G309900.1	Td--TRIDC7AG057270.3	0.00378	0.1075842291033144	0.035166387714355 1	1734	409.08	1324.92	5	41	0.004	0.1	8.27570993
TaMAPK53	Ta--TraesCS7B02G309900.1	Td--TRIDC7BG049670.7	0	0	NaN	1734	409.17	1324.83	0	0	0	0	0
TaMAPK54	Ta--TraesCS7B02G322900.1	Td--TRIDC6AG015690.2	0.05496	1.11459355060578	0.049313869016 0926	1131	259.92	871.083	46.17	150.8	0.053	0.58	85.7379654
TaMAPK54	Ta--TraesCS7B02G322900.1	Td--TRIDC6BG021470.2	0.05436	1.08793777907221	0.049964545929 9791	1131	260.08	870.917	45.67	149.3	0.052	0.574	83.6875215
TaMAPK54	Ta--TraesCS7B02G322900.1	Td--TRIDC7AG058780.3	0.00701	0.10028824829668	0.069865297675 1843	1116	255.67	860.333	6	24	0.007	0.094	7.71448064
TaMAPK54	Ta--TraesCS7B02G322900.1	Td--TRIDC7BG051510.2	0.00578	0.0235313419258153	0.245456626690 206	1128	259	869	5	6	0.006	0.023	1.81010323
TaMAPKK1	Ta--TraesCS6D02G328800.1	Td--TRIDC6AG052120.1	0.00499	0.0736788296875599	0.067686082730 6238	1104	299.25	804.75	4	21	0.005	0.07	5.66760228
TaMAPKK1	Ta--TraesCS6D02G328800.1	Td--TRIDC6BG060870.1	0.00687	0.1053735899849 9 4	0.065224126188 2785	1104	300.08	803.917	5.5	29.5	0.007	0.098	8.10566077

TaMAPKK 2	Ta--TraesCS5B02G565100.3	Td--TRIDC4AG047480.2	0.06775	0.2100996055787 62	0.322489104402 995	1296	304.25	991.75	64.25	55.75	0.065	0.183	16.1615081
TaMAPKK 2	Ta--TraesCS5B02G565100.3	Td--TRIDC5BG083460.4	0.04287	0.101395307637	0.422827068605 33	1239	289.08	949.917	39.58	27.42	0.042	0.095	7.79963905
TaMAPKK 3	Ta--TraesCS5D02G130900.2	Td--TRIDC5AG020820.4	0.01676	0.0737858123438149	0.227084085437 421	885	211.08	673.917	11.17	14.83	0.017	0.07	5.67583172
TaMAPKK 3	Ta--TraesCS5D02G130900.2	Td--TRIDC5BG021620.3	0.04206	0.11534673911063	0.364649363323 799	1224	286.83	937.167	38.33	30.67	0.041	0.107	8.87282609
TaMAPKK 4	Ta--TraesCS5A02G122700.4	Td--TRIDC5AG020820.4	0.0542	0.086588072808598	0.625979776360 957	843	200.75	642.25	33.58	16.42	0.052	0.082	6.66062099
TaMAPKK 4	Ta--TraesCS5A02G122700.4	Td--TRIDC5BG021620.3	0.02157	0.057212478562223	0.376955266216 645	816	196.67	619.333	13.17	10.83	0.021	0.055	4.40095989
TaMAPKK 6	Ta--TraesCS4B02G048100.1	Td--TRIDC4AG041540.1	0.23616	0.512803675557325	0.460526017572 017	984	270.33	713.667	144.6	100.4	0.203	0.371	39.4464366
TaMAPKK 6	Ta--TraesCS4B02G048100.1	Td--TRIDC4BG007520.1	0.00134	0.00353774240860418	0.379417742290 736	1029	283.33	745.667	1	1	0.001	0.004	0.27213403

TaMAPKK9		Ta--TraesCS3B02G066300.1	Td--TRIDC3BG008530.2	0.03565	0.0358722624266235	0.993698297982508	975	268.83	706.167	24.58	9.417	0.035	0.035	2.7594048
TaMAPKK13		Ta--TraesCS5D02G549600.1	Td--TRIDC4AG047480.2	0.01255	0.161924562122982	0.077515842976551	1569	363.92	1205.08	15	53	0.012	0.146	12.4557355
TaMAPKK13		Ta--TraesCS5D02G549600.1	Td--TRIDC5BG083460.4	0.00835	0.06875039139436 79	0.121516475656622	1569	365.33	1203.67	10	24	0.008	0.066	5.28849165
TaMAPKK14		Ta--TraesCS4A02G265900.1	Td--TRIDC4AG041540.1	0.03051	0.135016924595119	0.225974124026006	1011	275.17	735.833	22	34	0.03	0.124	10.3859173
TaMAPKK14		Ta--TraesCS4A02G265900.1	Td--TRIDC4BG007520.1	0.23736	0.532852466532255	0.445459242121528	984	268.5	715.5	145.6	102.4	0.203	0.381	40.9886513
TaMAPKK18		Ta--TraesCS5B02G122600.1	Td--TRIDC5AG020820.4	0.01677	0.0632731980111069	0.264978963642157	885	211.5	673.5	11.17	12.83	0.017	0.061	4.86716908
TaMAPKK18		Ta--TraesCS5B02G122600.1	Td--TRIDC5BG021620.3	0.01353	0.0452310953758704	0.299033136571675	1266	296.17	969.833	13	13	0.013	0.044	3.47931503
TaMAPKKK1	TaMEKK1	Ta--TraesCS2A02G407600.1	Td--TRIDC2AG058830.1	0	0.00679506346830184	0	2481	591.33	1889.67	0	4	0	0.007	0.52269719

TaMAPKKK1	TaMEKK1	Ta--TraesCS2A02G407600.1	Td--TRIDC2BG061830.1	0.02386	0.0771288346688374	0.309393814125996	2460	586.67	1873.33	44	43	0.023	0.073	5.93298728
TaMAPKKK1	TaMEKK1	Ta--TraesCS2A02G407600.1	Td--TRIDC6AG037840.4	0.15108	0.907555974135041	0.1664715349863	2427	579.92	1847.08	252.8	305.3	0.137	0.526	69.811998
TaMAPKKK1	TaMEKK1	Ta--TraesCS2A02G407600.1	Td--TRIDC6BG044800.1	0.13714	0.873582894625792	0.156991213467956	2364	567.5	1796.5	225.2	292.8	0.125	0.516	67.1986842
TaMAPKKK2	TaMEKK2	Ta--TraesCS4D02G027600.1	Td--TRIDC4AG043860.6	0.01028	0.142620354827718	0.0721048219900967	1704	431.17	1272.83	13	56	0.01	0.13	10.9707965
TaMAPKKK2	TaMEKK2	Ta--TraesCS4D02G027600.1	Td--TRIDC4BG004990.4	0.01347	0.142772317164011	0.0943595615042622	1704	430.75	1273.25	17	56	0.013	0.13	10.9824859
TaMAPKKK3	TaMEKK3	Ta--TraesCS4B02G210600.2	Td--TRIDC4AG012750.6	0.00816	0.0715313693800487	0.114062911879357	2115	513	1602	13	35	0.008	0.068	5.50241303
TaMAPKKK3	TaMEKK3	Ta--TraesCS4B02G210600.2	Td--TRIDC4BG037560.5	0.00065	0.00413651513929987	0.157031115488524	2025	484.83	1540.17	1	2	0.001	0.004	0.31819347
TaMAPKKK4	TaMEKK4	Ta--TraesCS6A02G245000.3	Td--TRIDC2AG058830.1	0.1511	0.907048947969418	0.166581241928488	2427	580.08	1846.92	252.8	305.3	0.137	0.526	69.772996

TaMAPKKK4	TaMEKK4	Ta--TraesCS6A02G245000.3	Td--TRIDC2BG061830.1	0.15803	0.8604176568012 11	0.183667254687451	2616	628.75	1987.25	283.2	321.8	0.142	0.512	66.1859736
TaMAPKKK4	TaMEKK4	Ta--TraesCS6A02G245000.3	Td--TRIDC6AG037840.4	0.0005	0		2634	634	2000	1	0	0.001	0	0
TaMAPKKK4	TaMEKK4	Ta--TraesCS6A02G245000.3	Td--TRIDC6BG044800.1	0.00704	0.0371043401542991	0.189670133469085	2634	635.33	1998.67	14	23	0.007	0.036	2.85418001
TaMAPKKK4-1	TaMEKK4-1	Ta--TraesCS6B02G279300.1	Td--TRIDC2AG058830.1	0.13381	0.850934018800818	0.157247342927983	2343	561.42	1781.58	218.3	285.7	0.123	0.509	65.456463
TaMAPKKK4-1	TaMEKK4-1	Ta--TraesCS6B02G279300.1	Td--TRIDC2BG061830.1	0.1348	0.845901079883277	0.159357535114766	2355	566.5	1788.5	220.7	287.3	0.123	0.507	65.0693138
TaMAPKKK4-1	TaMEKK4-1	Ta--TraesCS6B02G279300.1	Td--TRIDC6AG037840.4	0.00617	0.0397384137080505	0.155315924428594	2358	568.42	1789.58	11	22	0.006	0.039	3.05680105
TaMAPKKK4-1	TaMEKK4-1	Ta--TraesCS6B02G279300.1	Td--TRIDC6BG044800.1	0.00056	0		2358	570.33	1787.67	1	0	0.001	0	0
TaMAPKKK5	TaMEKK5	Ta--TraesCS2A02G199700.1	Td--TRIDC2AG025680.4	0.0032	0.00267022978989298	1.19728308453262	1629	375.17	1253.83	4	1	0.003	0.003	0.20540229

TaMAPKKK5	TaMEKK5	Ta--TraesCS2A02G199700.1	Td--TRIDC2BG030100.5	0.0064	0.0582817382454873	0.109885336644952	1629	374.5	1254.5	8	21	0.006	0.056	4.48321063
TaMAPKKK7	TaMEKK7	Ta--TraesCS3B02G289500.1	Td--TRIDC1BG061690.1	0.23262	0.607610853588378	0.382837460611696	1047	266.17	780.833	156.2	110.8	0.2	0.416	46.7392964
TaMAPKKK7	TaMEKK7	Ta--TraesCS3B02G289500.1	Td--TRIDC3BG043440.2	0.00515	0		1305	331.08	973.917	5	0	0.005	0	0
TaMAPKKK8	TaMEKK8	Ta--TraesCS3B02G288100.1	Td--TRIDC1AG053880.1	0.28819	0.664517088821144	0.433689228405294	1251	315.92	935.083	223.7	139.3	0.239	0.441	51.1166991
TaMAPKKK8	TaMEKK8	Ta--TraesCS3B02G288100.1	Td--TRIDC3AG038300.1	0.02495	0.124458436060348	0.200507404436402	1251	313.92	937.083	23	36	0.025	0.115	9.57372585
TaMAPKKK8	TaMEKK8	Ta--TraesCS3B02G288100.1	Td--TRIDC3BG043280.1	0.00104	0.00628275925353592	0.166201630629482	1278	319.67	958.333	1	2	0.001	0.006	0.48328917
TaMAPKKK10	TaMEKK10	Ta--TraesCS4D02G211300.2	Td--TRIDC4AG012750.6	0.00685	0.060475996865251	0.113234038018108	2130	516.33	1613.67	11	30	0.007	0.058	4.65199976
TaMAPKKK10	TaMEKK10	Ta--TraesCS4D02G211300.2	Td--TRIDC4BG037560.5	0.00981	0.0599891356194491	0.163501857133416	2025	485.67	1539.33	15	28	0.01	0.058	4.61454889

TaMAPKKK11	TaMEKK11	Ta--TraesCS5D02G475900.1	Td--TRIDC5AG066400.8	0.00489	0.07357449819145 21	0.066463149503 7367	1602	371	1231	6	26	0.005	0.07	5.65957678
TaMAPKKK11	TaMEKK11	Ta--TraesCS5D02G475900.1	Td--TRIDC5BG071320.5	0.04094	0.12571155705319 4	0.325667883327 722	1548	360	1188	47.33	41.67	0.04	0.116	9.67011977
TaMAPKKK12	TaMEKK12	Ta--TraesCS4A02G093800.2	Td--TRIDC4AG012750.6	0	0	NaN	2130	515.67	1614.33	0	0	0	0	0
TaMAPKKK12	TaMEKK12	Ta--TraesCS4A02G093800.2	Td--TRIDC4BG037560.5	0.00783	0.07132801161797 8	0.109816174503 288	2025	485	1540	12	33	0.008	0.068	5.48677012
TaMAPKKK14	TaMEKK14	Ta--TraesCS5A02G118200.1	Td--TRIDC5AG020280.1	0.00162	0		1611	376	1235	2	0	0.002	0	0
TaMAPKKK15	TaMEKK15	Ta--TraesCS5A02G463100.2	Td--TRIDC5AG066400.8	0.00244	0.00541763078059 518	0.450384774775 975	1602	370.5	1231.5	3	2	0.002	0.005	0.41674083
TaMAPKKK15	TaMEKK15	Ta--TraesCS5A02G463100.2	Td--TRIDC5BG071320.5	0.03826	0.13583767862923 4	0.281670712740 502	1548	359.5	1188.5	44.33	44.67	0.037	0.124	10.4490522
TaMAPKKK16	TaMEKK16	Ta--TraesCS5B02G474500.1	Td--TRIDC5AG066400.8	0.00163	0.10089547248772 1	0.016116947849 7392	1602	370.75	1231.25	2	35	0.002	0.094	7.76119019

TaMAPKKK16	TaMEKK16	Ta--TraesCS5B02G474500.1	Td--TRIDC5BG071320.5	0.0373	0.03602872963299 93	1.035252136515 78	1551	360.08	1190.92	43.33	12.67	0.036	0.035	2.77144074
TaMAPKKK17	TaMEKK17	Ta--TraesCS5A02G200800.1	Td--TRIDC5AG032410.5	0.00064	0.00212766100139 979	0.301927010830 03	2028	470.67	1557.33	1	1	0.001	0.002	0.16366623
TaMAPKKK17	TaMEKK17	Ta--TraesCS5A02G200800.1	Td--TRIDC5BG034210.7	0.00192	0.05686818508009 71	0.033746107538 3591	2040	474.75	1565.25	3	26	0.002	0.055	4.37447578
TaMAPKKK18	TaMEKK18	Ta--TraesCS2B02G526200.3	Td--TRIDC2AG070140.10	0.00891	0.08165067844261 2	0.109126597570 206	3966	917.75	3048.25	27	71	0.009	0.077	6.28082142
TaMAPKKK18	TaMEKK18	Ta--TraesCS2B02G526200.3	Td--TRIDC2BG075830.1	0	0.00108833684978 57	0	3969	919.5	3049.5	0	1	0	0.001	0.08371822
TaMAPKKK20	TaMEKK20	Ta--TraesCS2A02G498000.3	Td--TRIDC2AG070140.10	0.00098	0		3966	916.83	3049.17	3	0	0.001	0	0
TaMAPKKK20	TaMEKK20	Ta--TraesCS2A02G498000.3	Td--TRIDC2BG075830.1	0.00858	0.08045214028031 23	0.106620963632 334	3966	917.58	3048.42	26	70	0.009	0.076	6.18862618
TaMAPKKK21/TaMAPKKKK15	TaMEKK21	Ta--TraesCS6A02G149900.1	Td--TRIDC6AG020750.1	0.00079	0		1626	361.33	1264.67	1	0	0.001	0	0

TaMAPKKK21/TaMAPKKKK15	TaMEKK21	Ta--TraesCS6A02G149900.1	Td--TRIDC6BG026600.3	0.01237	0.121067579367253	0.102145258798394	1626	362.25	1263.75	15.5	40.5	0.012	0.112	9.31289072
TaMAPKKK22/TaMAPKKKK10	TaMEKK22	Ta--TraesCS5A02G392500.1	Td--TRIDC5AG057130.2	0.00348	0.00421349422816744	0.825101821093778	2469	595	1874	6.5	2.5	0.003	0.004	0.32411494
TaMAPKKK22/TaMAPKKKK10	TaMEKK22	Ta--TraesCS5A02G392500.1	Td--TRIDC5BG061270.2	0.00683	0.05965876642654	0.114547461718917	2322	558	1764	12	32	0.007	0.057	4.58913588
TaMAPKKK23/TaMAPKKKK21	TaMEKK23	Ta--TraesCS6D02G139200.1	Td--TRIDC6AG020750.1	0.00506	0.111315003838704	0.0454991230793017	1527	338.33	1188.67	6	35	0.005	0.103	8.5626926
TaMAPKKK23/TaMAPKKKK21	TaMEKK23	Ta--TraesCS6D02G139200.1	Td--TRIDC6BG026600.3	0.01231	0.0891441126405373	0.138053566865557	1527	339.08	1187.92	14.5	28.5	0.012	0.084	6.85723943
TaMAPKKK24	TaMEKK24	Ta--TraesCS5B02G199400.1	Td--TRIDC5AG032410.5	0.00193	0.0619707176641425	0.0311268203039521	2028	470.75	1557.25	3	28	0.002	0.059	4.76697828
TaMAPKKK24	TaMEKK24	Ta--TraesCS5B02G199400.1	Td--TRIDC5BG034210.7	0.00064	0		2040	474.83	1565.17	1	0	0.001	0	0
TaMAPKKK25	TaMEKK25	Ta--TraesCS5B02G196400.1	Td--TRIDC5AG032000.2	0.02019	0.0936472148657488	0.215545948366101	1554	382.42	1171.58	23.33	33.67	0.02	0.088	7.20363191

TaMAPKKK25	TaMEKK25	Ta--TraesCS5B02G196400.1	Td--TRIDC5BG033660.2	0.00086	0		1554	385.33	1168.67	1	0	0.001	0	0
TaMAPKKK26	TaMEKK26	Ta--TraesCS2D02G093700.1	Td--TRIDC2AG010980.4	0.00663	0.07743886598097 46	0.085585968921 0269	3996	965	3031	20	71	0.007	0.074	5.95683584
TaMAPKKK26	TaMEKK26	Ta--TraesCS2D02G093700.1	Td--TRIDC2BG013430.2	0.00529	0.04467822018399 91	0.118346929228 289	4005	968.33	3036.67	16	42	0.005	0.043	3.43678617
TaMAPKKK27	TaMEKK27	Ta--TraesCS2B02G110500.1	Td--TRIDC2AG010980.4	0.0073	0.07620071317294 13	0.095751093825 3888	3996	966.08	3029.92	22	70	0.007	0.072	5.86159332
TaMAPKKK27	TaMEKK27	Ta--TraesCS2B02G110500.1	Td--TRIDC2BG013430.2	0.00231	0.03591919564766 26	0.064298047411 3478	4005	969.42	3035.58	7	34	0.002	0.035	2.76301505
TaMAPKKK28	TaMEKK28	Ta--TraesCS2A02G095300.1	Td--TRIDC2AG010980.4	0.00066	0.00519437623026 219	0.127125652210 642	3996	965.92	3030.08	2	5	0.001	0.005	0.3995674
TaMAPKKK28	TaMEKK28	Ta--TraesCS2A02G095300.1	Td--TRIDC2BG013430.2	0.00797	0.07613159092122 91	0.104625916491 222	3996	966.92	3029.08	24	70	0.008	0.072	5.85627622
TaMAPKKK29	TaMEKK29	Ta--TraesCS5D02G206500.1	Td--TRIDC5AG032410.5	0.00193	0.03473848126803 23	0.055548633491 0182	2028	471.33	1556.67	3	16	0.002	0.034	2.67219087

TaMAPKKK29	TaMEKK29	Ta--TraesCS5D02G206500.1	Td--TRIDC5BG034210.7	0.00192	0.06821631341323 87	0.028144274555 5598	2040	475.42	1564.58	3	31	0.002	0.065	5.24740872
TaMAPKKK30	TaZIK1	Ta--TraesCS5D02G145100.1	Td--TRIDC5AG024780.6	0.01057	0.04739552240345 18	0.223037936651 595	1908	479	1429	15	22	0.01	0.046	3.64580942
TaMAPKKK30	TaZIK1	Ta--TraesCS5D02G145100.1	Td--TRIDC5BG025890.5	0.00979	0.07883275503936 9	0.124227580090 338	1920	481.08	1438.92	14	36	0.01	0.075	6.06405808
TaMAPKKK31	TaZIK2	Ta--TraesCS6D02G236400.1	Td--TRIDC6AG039240.3	0.03393	0.05555069505076 19	0.610712438933 908	1164	264.58	899.417	29.83	14.17	0.033	0.054	4.27313039
TaMAPKKK31	TaZIK2	Ta--TraesCS6D02G236400.1	Td--TRIDC6BG046090.3	0.0278	0.06741903645111 25	0.412284227224 11	1848	418.75	1429.25	39	27	0.027	0.064	5.18607973
TaMAPKKK32	TaZIK3	Ta--TraesCS2A02G195900.2	Td--TRIDC2AG025210.3	0	0.00417682945176 646	0	2079	480.17	1598.83	0	2	0	0.004	0.32129457
TaMAPKKK32	TaZIK3	Ta--TraesCS2A02G195900.2	Td--TRIDC2BG029650.2	0.00683	0.07820425883144 67	0.087315673044 2644	2103	484.75	1618.25	11	36	0.007	0.074	6.01571222
TaMAPKKK33	TaZIK4	Ta--TraesCS6B02G270400.1	Td--TRIDC6AG039240.3	0.06121	0.07946559163798 32	0.770213064745 879	1167	265.25	901.75	53	20	0.059	0.075	6.11273782

TaMAPKKK33	TaZIK4	Ta--TraesCS6B02G270400.1	Td--TRIDC6BG046090.3	0	0	NaN	1851	419.17	1431.83	0	0	0	0	0
TaMAPKKK34	TaZIK5	Ta--TraesCS2D02G197600.1	Td--TRIDC2AG024350.1	0.00415	0.0442606285777913	0.0938354909455968	957	232.67	724.333	3	10	0.004	0.043	3.40466374
TaMAPKKK34	TaZIK5	Ta--TraesCS2D02G197600.1	Td--TRIDC2BG028790.2	0.00974	0.03952616023343305	0.246457692889926	957	233.75	723.25	7	9	0.01	0.039	3.04047386
TaMAPKKK36	TaZIK7	Ta--TraesCS2B02G223600.1	Td--TRIDC2AG025210.3	0.00691	0.0790638133957663	0.0873972304159756	2079	479.75	1599.25	11	36	0.007	0.075	6.0818318
TaMAPKKK36	TaZIK7	Ta--TraesCS2B02G223600.1	Td--TRIDC2BG029650.2	0	0.00412939790589186	0	2109	485.67	1623.33	0	2	0	0.004	0.31764599
TaMAPKKK37	TaZIK8	Ta--TraesCS2B02G216800.1	Td--TRIDC2AG025210.3	0.44259	0.740476325638006	0.59771193205696	864	206.67	657.333	219.8	97.25	0.334	0.471	56.9597174
TaMAPKKK37	TaZIK8	Ta--TraesCS2B02G216800.1	Td--TRIDC2AG024350.1	0.01102	0.0810078500852973	0.135982976313737	966	234.42	731.583	8	18	0.011	0.077	6.23137308
TaMAPKKK37	TaZIK8	Ta--TraesCS2B02G216800.1	Td--TRIDC2BG028790.2	0.00274	0.00426743683325892	0.642301403218437	966	235	731	2	1	0.003	0.004	0.32826437

TaMAPKKK38	TaZIK9	Ta--TraesCS1D02G026200.2	Td--TRIDC1AG002650.1	0.00573	0.06564321859055 35	0.087300516796 3942	1815	413.67	1401.33	8	26	0.006	0.063	5.04947835
TaMAPKKK39	TaZIK10	Ta--TraesCS6A02G255100.2	Td--TRIDC6AG039240.3	0.02347	0.01971705665024 3	1.190499992113 12	1167	265.5	901.5	20.83	5.167	0.023	0.019	1.51669667
TaMAPKKK39	TaZIK10	Ta--TraesCS6A02G255100.2	Td--TRIDC6BG046090.3	0.03072	0.07784526595656 14	0.394587263577 064	1848	419.25	1428.75	43	31	0.03	0.074	5.98809738
TaMAPKKK40	TaZIK11	Ta--TraesCS5B02G146100.1	Td--TRIDC5AG024780.6	0.00915	0.08874606292998 47	0.103057175454 398	1908	477.92	1430.08	13	40	0.009	0.084	6.82662023
TaMAPKKK40	TaZIK11	Ta--TraesCS5B02G146100.1	Td--TRIDC5BG025890.5	0	0.00208623222140 76	0	1920	480	1440	0	1	0	0.002	0.1604794
TaMAPKKK41	TaRaf1	Ta--TraesCS6A02G172600.1	Td--TRIDC6AG024780.3	0.102	0.19390753156804 2	0.526022867649 668	729	178.5	550.5	52.5	30.5	0.095	0.171	14.915964
TaMAPKKK41	TaRaf1	Ta--TraesCS6A02G172600.1	Td--TRIDC7AG054160.3	0.18886	0.59850035115593 1	0.315549772086 887	1014	243.33	770.667	128.7	100.3	0.167	0.412	46.0384886
TaMAPKKK41	TaRaf1	Ta--TraesCS6A02G172600.1	Td--TRIDC7BG046810.3	0.19239	0.57807313086824 4	0.332816160737 427	1014	244	770	130.7	98.33	0.17	0.403	44.4671639

TaMAPKKK45	TaRaf5	Ta--TraesCS3D02G273200.1	Td--TRIDC1AG050190.1	0.33168	1.31851456219555	0.251554379526124	1740	412.83	1327.17	355.7	256.3	0.268	0.621	101.424197
TaMAPKKK45	TaRaf5	Ta--TraesCS3D02G273200.1	Td--TRIDC1BG057000.1	0.32772	1.29084951575606	0.2538822442057 3	1740	411.08	1328.92	352.8	253.2	0.266	0.616	99.2961166
TaMAPKKK45	TaRaf5	Ta--TraesCS3D02G273200.1	Td--TRIDC3AG040710.1	0.01757	0.0887671886532731	0.197949512506304	1800	418.08	1381.92	24	35	0.017	0.084	6.82824528
TaMAPKKK45	TaRaf5	Ta--TraesCS3D02G273200.1	Td--TRIDC3BG045870.3	0.01238	0.0701533371907363	0.176423091941957	1803	418.08	1384.92	17	28	0.012	0.067	5.39641055
TaMAPKKK46	TaRaf6	Ta--TraesCS2D02G050700.1	Td--TRIDC2AG005450.2	0.03582	0.0801537454781221	0.446920837241676	2454	588.67	1865.33	65.25	44.75	0.035	0.076	6.16567273
TaMAPKKK48	TaRaf8	Ta--TraesCS7D02G079100.1	Td--TRIDC7AG009510.2	0.12735	0.296344597734739	0.429729677562347	1596	360.83	1235.17	144.7	88.33	0.117	0.245	22.7957383
TaMAPKKK50	TaRaf10	Ta--TraesCS7D02G099200.2	Td--TRIDC7AG012240.3	0.03	0.0730025354605975	0.410906376592397	1974	472	1502	44.17	32.83	0.029	0.07	5.61557965
TaMAPKKK51	TaRaf11	Ta--TraesCS7D02G230200.1	Td--TRIDC6AG042270.3	0.23143	0.524580545441436	0.441171081324421	1074	270.08	803.917	160.1	101.9	0.199	0.377	40.3523496

TaMAPKKK51	TaRaf11	Ta--TraesCS7D02G230200.1	Td--TRIDC6BG049330.2	0.16598	0.43299307974882 6	0.383339541419 749	1218	297.92	920.083	137	98	0.149	0.329	33.30716
TaMAPKKK51	TaRaf11	Ta--TraesCS7D02G230200.1	Td--TRIDC7AG029400.6	0.00432	0.03360024356652 47	0.128560567134 661	1233	304.33	928.667	4	10	0.004	0.033	2.58463412
TaMAPKKK51	TaRaf11	Ta--TraesCS7D02G230200.1	Td--TRIDC7BG020300.3	0	0.07081606689350 29	0	1143	281.17	861.833	0	19	0	0.068	5.44738976
TaMAPKKK53	TaRaf13	Ta--TraesCS1B02G372400.1	Td--TRIDC1AG052430.1	0.03779	0.14906312327034	0.253545912196 605	2403	594.25	1808.75	66.67	80.33	0.037	0.135	11.4663941
TaMAPKKK53	TaRaf13	Ta--TraesCS1B02G372400.1	Td--TRIDC1BG059980.1	0.00804	0.01780247470129 26	0.451873127713 191	2409	596.83	1812.17	14.5	10.5	0.008	0.018	1.36942113
TaMAPKKK53	TaRaf13	Ta--TraesCS1B02G372400.1	Td--TRIDC3AG036880.3	0.22015	0.58390009461945 2	0.377032129826 563	1986	489.08	1496.92	285.6	198.4	0.191	0.406	44.9153919
TaMAPKKK53	TaRaf13	Ta--TraesCS1B02G372400.1	Td--TRIDC3BG040940.1	0.35817	2.53907974502931	0.141063724130 798	2340	556.17	1783.83	508	403	0.285	0.725	195.313827
TaMAPKKK54	TaRaf14	Ta--TraesCS7D02G503600.1	Td--TRIDC7BG067700.1	0.1817	0.64627690619231 4	0.281143112833 595	585	134.67	450.333	72.67	58.33	0.161	0.433	49.7136082

TaMAPKKK58	TaRaf18	Ta--TraesCS3B02G259800.1	Td--TRIDC1AG061210.1	0.06104	0.79841495180714 1	0.076451825058 3799	894	211.67	682.333	40	104	0.059	0.491	61.4165348
TaMAPKKK58	TaRaf18	Ta--TraesCS3B02G259800.1	Td--TRIDC1BG070170.4	0.06253	0.72169844838785 1	0.086640679127 3554	918	217.92	700.083	42	101	0.06	0.463	55.5152653
TaMAPKKK58	TaRaf18	Ta--TraesCS3B02G259800.1	Td--TRIDC3AG034530.2	0	0.08071021096555 15	0	945	222.17	722.833	0	17	0	0.077	6.20847777
TaMAPKKK58	TaRaf18	Ta--TraesCS3B02G259800.1	Td--TRIDC3BG038930.3	0	0	NaN	942	221	721	0	0	0	0	0
TaMAPKKK60	TaRaf20	Ta--TraesCS2A02G217000.1	Td--TRIDC2AG028890.5	0.4403	2.60060537632376	0.169306611165 478	1944	463.92	1480.08	492.9	337.1	0.333	0.727	200.046567
TaMAPKKK60	TaRaf20	Ta--TraesCS2A02G217000.1	Td--TRIDC2BG032550.5	0.45131	2.98999431599426	0.150939077570 784	1947	465.42	1481.58	502.4	342.6	0.339	0.736	229.999563
TaMAPKKK61	TaRaf21	Ta--TraesCS7D02G384700.1	Td--TRIDC6AG024780.3	0.31839	0.91348602561009 3	0.348546700934 176	846	210.33	635.667	164.9	111.1	0.259	0.528	70.2681558
TaMAPKKK61	TaRaf21	Ta--TraesCS7D02G384700.1	Td--TRIDC6BG030200.2	0.20273	0.58316855242967 4	0.347632136087 723	1098	267.67	830.333	147.5	108.5	0.178	0.405	44.8591194

TaMAPKKK61	TaRaf21	Ta--TraesCS7D02G384700.1	Td--TRIDC7AG054160.3	0.00111	0.0321095409803891	0.0345653684882206	1188	286.33	901.667	1	9	0.001	0.031	2.46996469
TaMAPKKK61	TaRaf21	Ta--TraesCS7D02G384700.1	Td--TRIDC7BG046810.3	0.00445	0.0430225461599401	0.103496971777634	1188	287	901	4	12	0.004	0.042	3.30942663
TaMAPKKK62	TaRaf22	Ta--TraesCS4A02G313900.1	Td--TRIDC4AG047400.2	0.00122	0.00256465306544853	0.473806433284811	3252	781.17	2470.83	3	2	0.001	0.003	0.19728101
TaMAPKKK62	TaRaf22	Ta--TraesCS4A02G313900.1	Td--TRIDC5BG083500.1	0.14962	0.401914481131308	0.372262343345239	2946	714.58	2231.42	302.7	222.3	0.136	0.311	30.9164985
TaMAPKKK64-1	TaRaf24-1	Ta--TraesCS4A02G464700.1	Td--TRIDC7AG002140.2	0.11648	0.515682101632867	0.225875712229118	504	119.33	384.667	41.5	44.5	0.108	0.373	39.667854
TaMAPKKK69	TaRaf29	Ta--TraesCS2D02G588200.1	Td--TRIDC2AG080090.1	0.09901	0.222188173755199	0.445607455410613	1635	385.25	1249.75	115.9	74.08	0.093	0.192	17.091398
TaMAPKKK69	TaRaf29	Ta--TraesCS2D02G588200.1	Td--TRIDC2BG088230.2	0.01956	0.105255714213313	0.185793037493819	1479	347.92	1131.08	21.83	34.17	0.019	0.098	8.0965934
TaMAPKKK70	TaRaf30	Ta--TraesCS1D02G423800.1	Td--TRIDC1AG061210.1	0.00438	0.167065208812425	0.0262402989589348	900	213.67	686.333	3	32	0.004	0.15	12.8511699

TaMAPKKK70	TaRaf30	Ta--TraesCS1D02G423800.1	Td--TRIDC1BG070170.4	0.00858	0.1281271780739 33	0.066970301078 2587	924	220.75	703.25	6	26	0.009	0.118	9.85593677
TaMAPKKK70	TaRaf30	Ta--TraesCS1D02G423800.1	Td--TRIDC3AG034530.2	0.06587	0.709065631146232	0.0929011816907382	945	223.5	721.5	45.5	102.5	0.063	0.459	54.5435101
TaMAPKKK70	TaRaf30	Ta--TraesCS1D02G423800.1	Td--TRIDC3BG038930.3	0.06603	0.72797166817751	0.0907074718093406	942	222.17	719.833	45.5	103.5	0.063	0.466	55.9978206
TaMAPKKK71	TaRaf31	Ta--TraesCS6A02G004500.1	Td--TRIDC6AG000550.4	0.00799	0.0108003830071921	0.739939016430358	2337	512.92	1824.08	14.5	5.5	0.008	0.011	0.83079869
TaMAPKKK72	TaRaf32	Ta--TraesCS3A02G003900.1	Td--TRIDC3BG001050.1	0.0296	0.182536360166401	0.16217798200581	1554	382.67	1171.33	34	62	0.029	0.162	14.0412585
TaMAPKKK73	TaRaf33	Ta--TraesCS3A02G039200.1	Td--TRIDC3AG004290.4	0.0425	0.191460863811363	0.221985064171511	2034	493.17	1540.83	63.67	83.33	0.041	0.169	14.7277588
TaMAPKKK73	TaRaf33	Ta--TraesCS3A02G039200.1	Td--TRIDC3BG006020.1	0.0403	0.194784686359404	0.206875464695253	2019	489.67	1529.33	60	84	0.039	0.172	14.9834374
TaMAPKKK75	TaRaf35	Ta--TraesCS3A02G096500.1	Td--TRIDC3AG011590.6	0.00138	0.00205198486415606	0.672802347368806	1938	488	1450	2	1	0.001	0.002	0.15784499

TaMAPKKK75	TaRaf35	Ta--TraesCS3A02G096500.1	Td--TRIDC3BG015640.4	0.0111	0.1028745229051	0.107902258986652	1941	488.92	1452.08	16	47	0.011	0.096	7.91342484
TaMAPKKK76	TaRaf36	Ta--TraesCS3A02G246100.1	Td--TRIDC1AG052430.1	0.11719	0.358375135872824	0.326997444928042	2388	589.08	1798.92	195.2	167.8	0.108	0.285	27.5673181
TaMAPKKK76	TaRaf36	Ta--TraesCS3A02G246100.1	Td--TRIDC1BG059980.1	0.12166	0.335788354858775	0.362325434285263	2391	590.17	1800.83	202.3	159.8	0.112	0.271	25.8298735
TaMAPKKK76	TaRaf36	Ta--TraesCS3A02G246100.1	Td--TRIDC3AG036880.3	0.12118	0.184831431086476	0.655614425450289	2037	502.08	1534.92	171.8	82.25	0.112	0.164	14.2178024
TaMAPKKK76	TaRaf36	Ta--TraesCS3A02G246100.1	Td--TRIDC3BG040940.1	0.33313	1.90622751011775	0.174757289806033	2355	560.58	1794.42	482.7	387.3	0.269	0.691	146.632885
TaMAPKKK81	TaRaf41	Ta--TraesCS5A02G292500.1	Td--TRIDC5BG047840.2	0.12347	0.315380609777428	0.391485496445416	1884	421.42	1462.58	166.5	108.5	0.114	0.257	24.2600469
TaMAPKKK86	TaRaf46	Ta--TraesCS5D02G386800.1	Td--TRIDC5AG055160.3	0.00117	0.105974544191404	0.0110333005290468	1119	263.08	855.917	1	26	0.001	0.099	8.15188801
TaMAPKKK86	TaRaf46	Ta--TraesCS5D02G386800.1	Td--TRIDC5AG056200.2	0.35279	1.69527549905343	0.208100281006245	1056	250.58	805.417	226.7	168.3	0.281	0.672	130.405808

TaMAPKKK86	TaRaf46	Ta--TraesCS5D02G386800.1	Td--TRIDC5BG059170.3	0.00117	0.09703959165214 61	0.012057419237 4066	1119	263.67	855.333	1	24	0.001	0.091	7.46458397
TaMAPKKK92	TaRaf52	Ta--TraesCS5B02G337300.1	Td--TRIDC5AG049770.1	0.00525	0.08510623350483 43	0.061649329090 7322	1749	410.17	1338.83	7	33	0.005	0.08	6.54663335
TaMAPKKK92	TaRaf52	Ta--TraesCS5B02G337300.1	Td--TRIDC5BG053430.2	0	0.00250836354213 543	0	1719	399.33	1319.67	0	1	0	0.003	0.19295104
TaMAPKKK93	TaRaf53	Ta--TraesCS5D02G018800.1	Td--TRIDC5BG001980.1	0.04126	0.16050134351708 6	0.257066562002 931	939	204.17	734.833	29.5	29.5	0.04	0.144	12.3462572
TaMAPKKK95	TaRaf55	Ta--TraesCS5B02G204900.1	Td--TRIDC5AG033270.1	0.01936	0.07049902815488 56	0.274675491516 263	1317	349.25	967.75	18.5	23.5	0.019	0.067	5.42300217
TaMAPKKK95	TaRaf55	Ta--TraesCS5B02G204900.1	Td--TRIDC5BG035150.3	0	0	NaN	1317	348.33	968.667	0	0	0	0	0
TaMAPKKK96	TaRaf56	Ta--TraesCS5B02G292000.2	Td--TRIDC5BG047840.2	0.01238	0.06549613768639 25	0.189082635126 752	1896	430.5	1465.5	18	27	0.012	0.063	5.03816444
TaMAPKKK97	TaRaf57	Ta--TraesCS5B02G353800.1	Td--TRIDC5AG051290.3	0.14812	0.65233720638802 1	0.227054775682 187	672	154.92	517.083	69.5	67.5	0.134	0.436	50.1797851

TaMAPKKK98	TaRaf58	Ta--TraesCS5D02G482000.1	Td--TRIDC5AG067100.1	0.00892	0.0599891356194489	0.148743367833738	1032	242.83	789.167	7	14	0.009	0.058	4.61454889
TaMAPKKK98	TaRaf58	Ta--TraesCS5D02G482000.1	Td--TRIDC5BG072290.4	0.05698	0.144037958994054	0.395616243807195	1014	241	773	42.42	31.58	0.055	0.131	11.079843
TaMAPKKK99	TaRaf59	Ta--TraesCS3A02G001500.1	Td--TRIDC3AG003160.1	0	0.00534285429588826	0	777	187.83	589.167	0	1	0	0.005	0.41098879
TaMAPKKK99	TaRaf59	Ta--TraesCS3A02G001500.1	Td--TRIDC3BG000190.2	0.01651	0.150192333936826	0.109910384499504	1095	268.17	826.833	13.5	36.5	0.016	0.136	11.5532565
TaMAPKKK100	TaRaf60	Ta--TraesCS3A02G274000.1	Td--TRIDC1AG050190.1	0.33457	1.28815173176702	0.25972541647255	1734	411	1323	357.1	252.9	0.27	0.615	99.0885948
TaMAPKKK100	TaRaf60	Ta--TraesCS3A02G274000.1	Td--TRIDC1BG057000.1	0.3343	1.27396442395981	0.262405958611797	1734	408.92	1325.08	357.4	250.6	0.27	0.613	97.9972634
TaMAPKKK100	TaRaf60	Ta--TraesCS3A02G274000.1	Td--TRIDC3AG040710.1	0.00145	0		1803	418.5	1384.5	2	0	0.001	0	0
TaMAPKKK100	TaRaf60	Ta--TraesCS3A02G274000.1	Td--TRIDC3BG045870.3	0.02052	0.06509504500315 55	0.315294729559683	1800	417	1383	28	26	0.02	0.062	5.00731115

TaMAPKKK101	TaRaf61	Ta--TraesCS5D02G019400.1	Td--TRIDC5BG002040.2	0.02745	0.114000683918073	0.240810397495702	3039	813.17	2225.83	60	86	0.027	0.106	8.76928338
TaMAPKKK102	TaRaf62	Ta--TraesCS7D02G474700.1	Td--TRIDC6AG021710.1	0.08935	0.762826430901621	0.117134827726129	1254	292.42	961.583	81	140	0.084	0.479	58.6789562
TaMAPKKK102	TaRaf62	Ta--TraesCS7D02G474700.1	Td--TRIDC6BG027410.1	0.08758	0.78764160407195	0.111195495151347	1254	292.25	961.75	79.5	142.5	0.083	0.488	60.5878157
TaMAPKKK102	TaRaf62	Ta--TraesCS7D02G474700.1	Td--TRIDC7AG068050.1	0.00205	0.167932556374681	0.012220145795 3167	1275	299.08	975.917	2	45	0.002	0.15	12.917889
TaMAPKKK102	TaRaf62	Ta--TraesCS7D02G474700.1	Td--TRIDC7BG061870.1	0.00308	0.0884676775076339	0.0348368165719975	1275	299.58	975.417	3	25	0.003	0.083	6.80520596
TaMAPKKK103	TaRaf63	Ta--TraesCS7A02G326700.1	Td--TRIDC7AG045740.2	0.00081	0.00266016683228101	0.305436773783536	1608	376.58	1231.42	1	1	0.001	0.003	0.20462822
TaMAPKKK103	TaRaf63	Ta--TraesCS7A02G326700.1	Td--TRIDC7BG037680.2	0.01874	0.0455223847945452	0.411643577964265	1551	362.25	1188.75	22	16	0.019	0.044	3.50172191
TaMAPKKK104	TaRaf64	Ta--TraesCS1D02G273600.1	Td--TRIDC1AG040730.2	0.0795	0.148992338401155	0.533588031702845	2019	514.33	1504.67	113.5	69.5	0.075	0.135	11.4609491

TaMAPKKK104	TaRaf64	Ta--TraesCS1D02G273600.1	Td--TRIDC1BG046350.4	0.06725	0.157674609086785	0.426532760271581	2010	509.83	1500.17	96.5	72.5	0.064	0.142	12.1288161
TaMAPKKK104-1	TaRaf64-1	Ta--TraesCS1B02G283400.1	Td--TRIDC1AG040730.2	0.07779	0.162769016101706	0.477904584580205	2025	516	1509	111.5	75.5	0.074	0.146	12.5206935
TaMAPKKK104-1	TaRaf64-1	Ta--TraesCS1B02G283400.1	Td--TRIDC1BG046350.4	0	0.00195694827271029	0	2016	511.67	1504.33	0	1	0	0.002	0.15053448
TaMAPKKK105	TaRaf65	Ta--TraesCS3A02G045200.1	Td--TRIDC3AG005110.2	0.00112	0.00663721145773687	0.168592739268112	1197	302.67	894.333	1	2	0.001	0.007	0.51055473
TaMAPKKK105	TaRaf65	Ta--TraesCS3A02G045200.1	Td--TRIDC3BG005340.1	0.01481	0.127385536926584	0.116291770564942	1185	298.75	886.25	13	35	0.015	0.117	9.79888746
TaMAPKKK106	TaRaf66	Ta--TraesCS4A02G456900.1	Td--TRIDC4AG067540.2	0.3227	0.984266095198941	0.32785369434115	1899	447.75	1451.25	380.6	245.4	0.262	0.548	75.7127766
TaMAPKKK106	TaRaf66	Ta--TraesCS4A02G456900.1	Td--TRIDC7AG003050.2	0.02652	0.105767110789391	0.250770718092588	1851	437.58	1413.42	36.83	43.17	0.026	0.099	8.1359316
TaMAPKKK107	TaRaf67	Ta--TraesCS7A02G032700.1	Td--TRIDC4AG067560.1	0.1037	0.490098949476573	0.2115845316896	1428	333.5	1094.5	106	120	0.097	0.36	37.6999192

TaMAPKKK107	TaRaf67	Ta--TraesCS7A02G032700.1	Td--TRIDC7AG003050.2	0.01065	0.0096386868776853	1.1054194717482	1353	313.25	1039.75	11	3	0.011	0.01	0.74143745
TaMAPKKK108	TaRaf68	Ta--TraesCS3D02G023600.1	Td--TRIDC3BG003740.4	0.03168	0.115473669414864	0.274380277132693	1266	299	967	30	32	0.031	0.107	8.88258995
TaMAPKKK109	TaRaf69	Ta--TraesCS2D02G219800.1	Td--TRIDC2AG028370.1	0.02252	0.09817575500704 07	0.229430005811456	2043	510.75	1532.25	34	47	0.022	0.092	7.55198115
TaMAPKKK110	TaRaf70	Ta--TraesCS4B02G289100.1	Td--TRIDC4AG002010.1	0.15163	0.271007590476187	0.559498637007512	1080	285.42	794.583	109.1	64.92	0.137	0.227	20.8467377
TaMAPKKK110	TaRaf70	Ta--TraesCS4B02G289100.1	Td--TRIDC4BG049660.1	0.00067	0.00374065613327928	0.177826694026476	2040	536	1504	1	2	0.001	0.004	0.28774278
TaMAPKKK111	TaRaf71	Ta--TraesCS3A02G315100.1	Td--TRIDC3AG046380.3	0.00268	0		2430	558.58	1871.42	5	0	0.003	0	0
TaMAPKKK111	TaRaf71	Ta--TraesCS3A02G315100.1	Td--TRIDC3BG052620.2	0.04005	0.0488763000332292	0.819436304664022	1320	302.92	1017.08	39.67	14.33	0.039	0.047	3.75971539
TaMAPKKK111	TaRaf71	Ta--TraesCS3A02G315100.1	Td--TRIDC3BG052880.9	0.50524	NaN	NaN	2292	544.5	1747.5	642.4	464.6	0.368	0.853	High Sequence Divergence Value (pS>=0.75)

TaMAPKKK115	TaRaf75	Ta--TraesCS3A02G229800.1	Td--TRIDC3AG034390.3	0	0.01214355612280 19	0	1587	415.08	1171.92	0	5	0	0.012	0.9341197
TaMAPKKK115	TaRaf75	Ta--TraesCS3A02G229800.1	Td--TRIDC3BG038810.4	0.03875	0.103856701377086	0.373075336316 23	1596	417.58	1178.42	44.5	40.5	0.038	0.097	7.98897703
TaMAPKKK116	TaRaf76	Ta--TraesCS1B02G454000.2	Td--TRIDC1BG071250.6	0.20881	0.702612082577192	0.297191749689 681	1848	449.83	1398.17	254.8	205.2	0.182	0.456	54.0470833
TaMAPKKK117	TaRaf77	Ta--TraesCS3D02G097000.1	Td--TRIDC3AG011590.6	0.01815	0.061923825435111 9	0.293066611084 727	1938	487.92	1450.08	26	29	0.018	0.059	4.76337119
TaMAPKKK117	TaRaf77	Ta--TraesCS3D02G097000.1	Td--TRIDC3BG015640.4	0.0111	0.098107726882621 8	0.113177662699 222	1941	489.33	1451.67	16	45	0.011	0.092	7.54674822
TaMAPKKK119	TaRaf79	Ta--TraesCS2A02G577000.1	Td--TRIDC2BG088230.2	0.01001	0.100726559810219	0.099373767582 8018	1512	355.42	1156.58	11.5	33.5	0.01	0.094	7.74819691
TaMAPKKK121	TaRaf81	Ta--TraesCS2D02G066900.1	Td--TRIDC2AG007500.5	0.33726	1.32954169740596	0.253664636552 142	2886	715.42	2170.58	589.6	445.4	0.272	0.623	102.272438
TaMAPKKK122	TaRaf82	Ta--TraesCS2A02G214000.1	Td--TRIDC2AG028370.1	0.00131	0.00195758675574 277	0.667683048829 233	2043	511.5	1531.5	2	1	0.001	0.002	0.1505836

TaMAPKKK123-1	TaRaf83-1	Ta--TraesCS5D02G358200.1	Td--TRIDC5AG051310.6	0.18928	0.7391252290792470	0.256088825761523	1371	315.92	1055.08	176.5	148.5	0.167	0.47	56.8557869
TaMAPKKK125	TaRaf85	Ta--TraesCS7D02G153800.1	Td--TRIDC7AG018660.25	0.04654	0.165799485838111	0.280682683058412	1353	337.25	1015.75	45.83	50.17	0.045	0.149	12.7538066
TaMAPKKK128	TaRaf88	Ta--TraesCS1B02G446500.1	Td--TRIDC1AG061210.1	0.00292	0.160959796072858	0.0181482163088325	900	214	686	2	31	0.003	0.145	12.3815228
TaMAPKKK128	TaRaf88	Ta--TraesCS1B02G446500.1	Td--TRIDC1BG070170.4	0.00285	0.0811278807381575	0.0351383305338462	924	221.08	702.917	2	17	0.003	0.077	6.24060621
TaMAPKKK128	TaRaf88	Ta--TraesCS1B02G446500.1	Td--TRIDC3AG034530.2	0.06439	0.718870721894625	0.089574355804219	945	223.83	721.167	44.5	103.5	0.062	0.462	55.2977478
TaMAPKKK128	TaRaf88	Ta--TraesCS1B02G446500.1	Td--TRIDC3BG038930.3	0.06455	0.714389752627958	0.0903542474016787	942	222.5	719.5	44.5	102.5	0.062	0.461	54.9530579
TaMAPKKK129	TaRaf89	Ta--TraesCS6B02G320800.1	Td--TRIDC6BG052000.1	0.10106	0.12669376669724	0.797651372832766	1269	318.83	950.167	89.83	37.17	0.095	0.117	9.74567436
TaMAPKKK131	TaRaf91	Ta--TraesCS6B02G215100.2	Td--TRIDC6AG027120.2	0.01183	0.0271742485110101	0.435227614815954	2229	524.58	1704.42	20	14	0.012	0.027	2.09032681

TaMAPKKK131	TaRaf91	Ta--TraesCS6B02G215100.2	Td--TRIDC6BG033330.6	0.00073	0.00681048947680986	0.106567559573051	1821	442.5	1378.5	1	3	0.001	0.007	0.52388381
TaMAPKKK133	TaRaf93	Ta--TraesCS1D02G004300.1	Td--TRIDC1BG000450.3	0.01755	0.239527146517152	0.073287882963 5221	1620	409.67	1210.33	21	84	0.017	0.205	18.4251651
TaMAPKKK134	TaRaf94	Ta--TraesCS5D02G547500.1	Td--TRIDC4AG047400.2	0.31104	1.47185660992628	0.211326242963726	2178	514	1664	423.7	331.3	0.255	0.645	113.219739
TaMAPKKK137	TaRaf97	Ta--TraesCS3D02G472000.1	Td--TRIDC3AG067170.2	0.04134	0.143042227087951	0.289015156943727	870	211.17	658.833	26.5	27.5	0.04	0.13	11.0032482
TaMAPKKK140	TaRaf100	Ta--TraesCS5D02G097900.1	Td--TRIDC5AG013160.4	0.00581	0.0405178085329921	0.14349898268011	2259	532.42	1726.58	10	21	0.006	0.039	3.1167545
TaMAPKKK140	TaRaf100	Ta--TraesCS5D02G097900.1	Td--TRIDC5BG015110.1	0.00669	0.0729987511184199	0.091652243583 3342	1947	445.67	1501.33	10	31	0.007	0.07	5.61528855
TaMAPKKK146	TaRaf106	Ta--TraesCS2B02G241400.1	Td--TRIDC2AG028730.4	0.03695	0.0992190295197042	0.372454155456731	2001	489.58	1511.42	54.5	45.5	0.036	0.093	7.63223304
TaMAPKKK146	TaRaf106	Ta--TraesCS2B02G241400.1	Td--TRIDC2BG032450.3	0.00199	0		2001	490.83	1510.17	3	0	0.002	0	0

TaMAPKKK148	TaRaf108	Ta--TraesCS1A02G003900.1	Td--TRIDC1BG000340.1	0.11217	0.39633677302590 5	0.283008185313 808	1023	227.92	795.083	82.83	70.17	0.104	0.308	30.4874441
TaMAPKKK150	TaRaf110	Ta--TraesCS3B02G008600.2	Td--TRIDC3AG002440.3	0.02705	0.15867555612490 8	0.170472108279 048	2037	531.42	1505.58	40	76	0.027	0.143	12.205812
TaMAPKKK150	TaRaf110	Ta--TraesCS3B02G008600.2	Td--TRIDC3BG001050.1	0	0	NaN	1560	385	1175	0	0	0	0	0
TaMAPKKK151	TaRaf111	Ta--TraesCS3B02G123800.1	Td--TRIDC3AG012960.1	0.02228	0.08542187463344 56	0.260845510697 612	1731	425.25	1305.75	28.67	34.33	0.022	0.081	6.57091343
TaMAPKKK151	TaRaf111	Ta--TraesCS3B02G123800.1	Td--TRIDC3BG017420.1	0.00153	0		1731	425.67	1305.33	2	0	0.002	0	0
TaMAPKKK152	TaRaf112	Ta--TraesCS3B02G259100.1	Td--TRIDC3AG034390.3	0.04165	0.10468576338775 9	0.397828011104 016	1587	414.5	1172.5	47.5	40.5	0.041	0.098	8.05275103
TaMAPKKK152	TaRaf112	Ta--TraesCS3B02G259100.1	Td--TRIDC3BG038810.4	0.00212	0.00120048044838 44	1.768578298172 42	1596	416.83	1179.17	2.5	0.5	0.002	0.001	0.09234465
TaMAPKKK153	TaRaf113	Ta--TraesCS3B02G351800.1	Td--TRIDC3AG046380.3	0.01605	0.11108039055768	0.144448022233 575	906	213.08	692.917	11	22	0.016	0.103	8.54464543

TaMAPKKK154	TaRaf114	Ta--TraesCS3B02G478400.1	Td--TRIDC3BG070490.1	0.00323	0		792	172.33	619.667	2	0	0.003	0	0
TaMAPKKK155	TaRaf115	Ta--TraesCS6B02G217100.1	Td--TRIDC7AG049870.2	0.26127	2.59651603904645	0.100624156919516	867	209	658	145.2	151.8	0.221	0.726	199.732003
TaMAPKKK155	TaRaf115	Ta--TraesCS6B02G217100.1	Td--TRIDC7BG043940.1	0.2597	3.21278750775947	0.0808341227119502	879	213.5	665.5	146.1	157.9	0.22	0.74	247.137501
TaMAPKKKK1		Ta--TraesCS1A02G181900.1	Td--TRIDC1AG027610.11	0	0.00211491151775357	0	2067	473.5	1593.5	0	1	0	0.002	0.1626855
TaMAPKKKK1		Ta--TraesCS1A02G181900.1	Td--TRIDC1BG033320.4	0.00475	0.0496439547286897	0.0955855024765835	1917	437.17	1479.83	7	21	0.005	0.048	3.81876575
TaMAPKKKK2		Ta--TraesCS1B02G199100.2	Td--TRIDC1AG027610.11	0.00377	0.0457658297442697	0.0824543745325147	2067	473	1594	6	21	0.004	0.044	3.52044844
TaMAPKKKK2		Ta--TraesCS1B02G199100.2	Td--TRIDC1BG033320.4	0.00068	0		1917	436.67	1480.33	1	0	0.001	0	0
TaMAPKKKK3		Ta--TraesCS1D02G185000.2	Td--TRIDC1AG027610.11	0.00188	0.0346069679327911	0.0544549800224439	2067	473.08	1593.92	3	16	0.002	0.034	2.66207446

TaMAPKKKK3	Ta--TraesCS1D02G185000.2	Td--TRIDC1BG033320.4	0.00406	0.0399702803998843	0.101684605405365	1917	436.75	1480.25	6	17	0.004	0.039	3.07463695
TaMAPKKKK4	Ta--TraesCS2A02G233400.1	Td--TRIDC2AG030300.1	0.00063	0.00201748608540676	0.313911587860861	2076	496.33	1579.67	1	1	0.001	0.002	0.15519124
TaMAPKKKK4	Ta--TraesCS2A02G233400.1	Td--TRIDC2BG033980.3	0.00444	0.0478498463029498	0.0928735937118374	2076	496.17	1579.83	7	23	0.004	0.046	3.68075741
TaMAPKKKK5	Ta--TraesCS2B02G249900.1	Td--TRIDC2AG030300.1	0.00444	0.0435958062133858	0.101914549522575	2076	495.83	1580.17	7	21	0.004	0.042	3.35352355
TaMAPKKKK5	Ta--TraesCS2B02G249900.1	Td--TRIDC2BG033980.3	0.00063	0.00202020324166721	0.313357077504967	2076	495.67	1580.33	1	1	0.001	0.002	0.15540025
TaMAPKKKK6	Ta--TraesCS2D02G232200.1	Td--TRIDC2AG030300.1	0.00476	0.0256566833991391	0.185552483236487	2076	495.58	1580.42	7.5	12.5	0.005	0.025	1.97359103
TaMAPKKKK6	Ta--TraesCS2D02G232200.1	Td--TRIDC2BG033980.3	0.00572	0.0565811929013991	0.101019883145075	2076	495.42	1580.58	9	27	0.006	0.054	4.35239945
TaMAPKKKK7	Ta--TraesCS4B02G395600.1	Td--TRIDC1AG027610.11	0.2646	1.03634927928802	0.255316613349468	1719	400.75	1318.25	293.9	225.1	0.223	0.562	79.7191753

TaMAPKKKK8	Ta--TraesCS4B02G398400.3	Td--TRIDC1AG027610.11	0.18079	0.99534553783817 9	0.181639346371077	2028	466.67	1561.33	250.8	257.2	0.161	0.551	76.5650414
TaMAPKKKK8	Ta--TraesCS4B02G398400.3	Td--TRIDC1BG033320.4	0.18405	1.08413721642466	0.169762417820955	1878	430.42	1447.58	236.3	246.8	0.163	0.573	83.3951705
TaMAPKKKK8	Ta--TraesCS4B02G398400.3	Td--TRIDC5AG078780.1	0.02808	0.0983929473184089	0.285369651533873	2061	482.58	1578.42	43.5	44.5	0.028	0.092	7.56868826
TaMAPKKKK9	Ta--TraesCS5A02G187400.1	Td--TRIDC5AG032000.2	0.0037	0.00970744541864889	0.38122531414588	1554	380.17	1173.83	4.333	3.667	0.004	0.01	0.74672657
TaMAPKKKK9	Ta--TraesCS5A02G187400.1	Td--TRIDC5BG033660.2	0.01728	0.0944599268834398	0.182915004632196	1554	383.08	1170.92	20	34	0.017	0.089	7.26614822
TaMAPKKKK11	Ta--TraesCS5A02G556400.5	Td--TRIDC1AG027610.11	0.17268	0.924049900272294	0.186867878381902	2013	462.92	1550.08	239.1	245.9	0.154	0.531	71.0807616
TaMAPKKKK11	Ta--TraesCS5A02G556400.5	Td--TRIDC5AG078780.1	0.01354	0.0256755327798694	0.527492781220761	2040	475.42	1564.58	21	12	0.013	0.025	1.97504098
TaMAPKKKK12	Ta--TraesCS5B02G397300.1	Td--TRIDC5AG057130.2	0.00886	0.060364830835347	0.146707831087657	2469	594.83	1874.17	16.5	34.5	0.009	0.058	4.64344853

TaMAPKKKK12	Ta--TraesCS5B02G397300.1	Td--TRIDC5BG061270.2	0.00113	0.00901724117215036	0.125818684235447	2322	557.83	1764.17	2	5	0.001	0.009	0.69363394
TaMAPKKKK13	Ta--TraesCS5D02G203600.1	Td--TRIDC5AG032000.2	0.01927	0.0913036616244838	0.211039705262639	1554	380	1174	22.33	32.67	0.019	0.086	7.02335859
TaMAPKKKK13	Ta--TraesCS5D02G203600.1	Td--TRIDC5BG033660.2	0.01379	0.0740495906961091	0.186206824911701	1554	382.92	1171.08	16	27	0.014	0.071	5.69612236
TaMAPKKKK14	Ta--TraesCS5D02G402300.1	Td--TRIDC5AG057130.2	0.00889	0.0533674998893562	0.1665021141103	2460	592.08	1867.92	16.5	30.5	0.009	0.052	4.1051923
TaMAPKKKK14	Ta--TraesCS5D02G402300.1	Td--TRIDC5BG061270.2	0.0037	0.0377785985155642	0.0980233215300578	2316	556.42	1759.58	6.5	20.5	0.004	0.037	2.90604604
TaMAPKKKK16	Ta--TraesCS6A02G353400.1	Td--TRIDC6AG053160.4	0.01192	0.0292393982367445	0.407668280835926	894	203.42	690.583	8.167	5.833	0.012	0.029	2.24918448
TaMAPKKKK16	Ta--TraesCS6A02G353400.1	Td--TRIDC6BG061710.3	0.01028	0.0636394776412797	0.161597211809352	912	210.33	701.667	7.167	12.83	0.01	0.061	4.89534443
TaMAPKKKK18	Ta--TraesCS6B02G177800.1	Td--TRIDC6AG020750.1	0.01317	0.124349849267513	0.105914773423427	1626	362.17	1263.83	16.5	41.5	0.013	0.115	9.56537302

TaMAPKKKK18	Ta--TraesCS6B02G177800.1	Td--TRIDC6BG026600.3	0.00159	0.00275925811338641	0.574542138041081	1626	363.08	1262.92	2	1	0.002	0.003	0.21225062
TaMAPKKKK19	Ta--TraesCS6B02G386100.1	Td--TRIDC6AG053160.4	0.00216	0.0325254218363474	0.0665200615485585	1797	408.42	1388.58	3	13	0.002	0.032	2.50195553
TaMAPKKKK19	Ta--TraesCS6B02G386100.1	Td--TRIDC6BG061710.3	0.00066	0.00450451804515894	0.147312108258894	1953	445.33	1507.67	1	2	0.001	0.004	0.34650139
TaMAPKKKK20	Ta--TraesCS6D02G335800.1	Td--TRIDC6AG053160.4	0.00289	0.0376963760856662	0.0765408462740315	1797	408	1389	4	15	0.003	0.037	2.89972124
TaMAPKKKK20	Ta--TraesCS6D02G335800.1	Td--TRIDC6BG061710.3	0.00399	0.0392170822682073	0.101719705964875	1953	444.92	1508.08	6	17	0.004	0.038	3.01669864
TaMAPKKKK22	Ta--TraesCS7A02G232300.1	Td--TRIDC7AG029890.1	0.00062	0		2100	482.33	1617.67	1	0	0.001	0	0
TaMAPKKKK22	Ta--TraesCS7A02G232300.1	Td--TRIDC7BG020630.1	0.00683	0.0604208668478506	0.113055827432374	2100	482.33	1617.67	11	28	0.007	0.058	4.64775899
TaMAPKKKK23	Ta--TraesCS7B02G130700.1	Td--TRIDC7AG029890.1	0.00621	0.0581561403468654	0.106747151690557	2100	482.5	1617.5	10	27	0.006	0.056	4.47354926

TaMAPKKKK23	Ta--TraesCS7B02G130700.1	Td--TRIDC7BG020630.1	0.00124	0.0020754077581204	0.596266641452943	2100	482.5	1617.5	2	1	0.001	0.002	0.15964675
TaMAPKKKK24	Ta--TraesCS7D02G232400.1	Td--TRIDC7AG029890.1	0.01631	0.0461628678233344	0.353392829344622	2067	476.5	1590.5	25.67	21.33	0.016	0.045	3.55098983
TaMAPKKKK24	Ta--TraesCS7D02G232400.1	Td--TRIDC7BG020630.1	0.0176	0.0874524368939637	0.201250496811154	2067	476.5	1590.5	27.67	39.33	0.017	0.083	6.72711053
TaMAPKKKK25	Ta--TraesCSU02G115300.1	Td--TRIDC5AG078780.1	0.02771	0.0743642698449401	0.372596261921256	2061	480.25	1580.75	43	34	0.027	0.071	5.72032845
													Average=27.6055561113674

The Ka/Ks ratio and divergence times between the genes of *T.dicoccoides* and *T.turgidum*

Gene ID	Gene ID	Ka	Ks	Ka/Ks	EffectiveLen	AverageS-sites	AverageN-sites	cN	cS	pN	pS	Divergence times (Mya)
Td--TRIDC6AG041340.3	Tt--TRITD6Av1G170870.1	0.1065743	0.208721734135453	0.510604793066788	1131	261.16667	869.83333	86.42	47.58	0.099	0.182	16.055518
Td--TRIDC6AG041340.3	Tt--TRITD6Bv1G157460.4	0.0074245	0.0501679511239305	0.147992700003166	1236	288.5	947.5	7	14	0.007	0.049	3.85907316
Td--TRIDC6BG048300.1	Tt--TRITD6Av1G170870.1	0.1057604	0.2417510902655	0.437476287221586	1149	265.33333	883.66667	87.17	54.83	0.099	0.207	18.5962377
Td--TRIDC6BG048300.1	Tt--TRITD6Bv1G157460.4	0.0010409	0		1254	292.66667	961.33333	1	0	0.001	0	0
Td--TRIDC5BG079810.1	Tt--TRITD4Av1G214900.3	0.014998	0.0703247651031883	0.213267689904842	1323	312.83333	1010.1667	15	21	0.015	0.067	5.40959732
Td--TRIDC5BG079810.1	Tt--TRITD5Bv1G245660.3	0.0019773	0.00635260547548787	0.311252232754272	1329	316.16667	1012.8333	2	2	0.002	0.006	0.48866196
Td--TRIDC4AG014520.1	Tt--TRITD4Av1G049530.4	0.0236666	0.0284498004157667	0.831872335058616	984	232.83333	751.16667	17.5	6.5	0.023	0.028	2.18844619
Td--TRIDC4AG014520.1	Tt--TRITD4Bv1G124550.2	0	0.0870541284395653	0	1098	255.5	842.5	0	21	0	0.082	6.69647142
Td--TRIDC4BG035610.1	Tt--TRITD4Av1G049530.4	0.0250362	0.103533165597153	0.241817720611919	984	232.66667	751.33333	18.5	22.5	0.025	0.097	7.96408966
Td--TRIDC4BG035610.1	Tt--TRITD4Bv1G124550.2	0	0	NaN	1107	258	849	0	0	0	0	0

Td--TRIDC1AG012310.2	Tt--TRITD1Av1G032550.8	0.0007937	0.00259291528789334	0.306084379777707	1647	386.33333	1260.6667	1	1	0.001	0.003	0.19945502
Td--TRIDC1AG012310.2	Tt--TRITD1Bv1G041580.2	0.0043428	0.0962278867899456	0.0451304008214176	1509	354.33333	1154.6667	5	32	0.004	0.09	7.40214514
Td--TRIDC1BG015140.6	Tt--TRITD1Av1G032550.8	0.004779	0.096241687158416	0.0496561192112174	1647	387.5	1259.5	6	35	0.005	0.09	7.4032067
Td--TRIDC1BG015140.6	Tt--TRITD1Bv1G041580.2	0.0092335	0.0171118876470109	0.539596130336913	1545	364.5	1180.5	10.83	6.167	0.009	0.017	1.31629905
Td--TRIDC1AG061100.1	Tt--TRITD1Av1G220750.2	0.0539344	0.145598490894623	0.370432170769167	1686	401.75	1284.25	66.83	53.17	0.052	0.132	11.1998839
Td--TRIDC1AG061100.1	Tt--TRITD1Bv1G218290.1	0.0376254	0.111714311714502	0.336800571574661	1602	375.75	1226.25	45	39	0.037	0.104	8.59340859
Td--TRIDC1AG061100.1	Tt--TRITD3Av1G155110.1	0.1354189	1.23525197428024	0.109628540014771	1443	328.5	1114.5	138.1	198.9	0.124	0.606	95.0193826
Td--TRIDC1AG061100.1	Tt--TRITD3Bv1G135620.1	0.1265958	1.27125495123956	0.0995833668165323	1443	330.58333	1112.4167	129.6	202.4	0.116	0.612	97.7888424
Td--TRIDC1BG070050.1	Tt--TRITD1Av1G220750.2	0.0606144	0.159326652781303	0.380440996649074	1680	400.58333	1279.4167	74.5	57.5	0.058	0.144	12.2558964
Td--TRIDC1BG070050.1	Tt--TRITD1Bv1G218290.1	0.0624474	0.138259104546158	0.451669352754335	1689	403.91667	1285.0833	77	51	0.06	0.126	10.6353157
Td--TRIDC1BG070050.1	Tt--TRITD3Av1G155110.1	0.13728	1.26799751426752	0.108265165489214	1443	329	1114	139.8	201.3	0.125	0.612	97.5382703

Td--TRIDC1BG070050.1	Tt--TRITD3Av1G155110.1	0.13728	1.26799751426752	0.108265165489214	1443	329	1114	139.8	201.3	0.125	0.612	97.5382703
Td--TRIDC1BG070050.1	Tt--TRITD3Bv1G135620.1	0.1284346	1.28834650155023	0.0996895185728013	1443	331.08333	1111.9167	131.3	203.8	0.118	0.615	99.103577
Td--TRIDC3AG034650.7	Tt--TRITD1Av1G220750.2	0.1452449	1.29301334360786	0.112330519737356	1473	337.66667	1135.3333	149.9	208.1	0.132	0.616	99.4625649
Td--TRIDC3AG034650.7	Tt--TRITD1Bv1G218290.1	0.134263	1.2333260658534	0.10886253079586	1464	336	1128	138.7	203.3	0.123	0.605	94.8712358
Td--TRIDC3AG034650.7	Tt--TRITD3Av1G155110.1	0.0008824	0		1470	336	1134	1	0	0.001	0	0
Td--TRIDC3AG034650.7	Tt--TRITD3Bv1G135620.1	0.0205998	0.074576263040085	0.276225252490908	1470	338.08333	1131.9167	23	24	0.02	0.071	5.73663562
Td--TRIDC3BG039070.12	Tt--TRITD1Av1G220750.2	0.1302836	1.35220365034652	0.0963491190144152	1482	344.83333	1137.1667	136	216	0.12	0.626	104.015665
Td--TRIDC3BG039070.12	Tt--TRITD1Bv1G218290.1	0.1303671	1.29446660129644	0.100711056079871	1482	345.5	1136.5	136	213	0.12	0.616	99.5743539
Td--TRIDC3BG039070.12	Tt--TRITD3Av1G155110.1	0.0390825	0.0939055089510034	0.416190029032335	1449	335.16667	1113.8333	42.42	29.58	0.038	0.088	7.22350069
Td--TRIDC3BG039070.12	Tt--TRITD3Bv1G135620.1	0.018593	0.02282975827682	0.814420121405706	1449	337.25	1111.75	20.42	7.583	0.018	0.022	1.75613525
Td--TRIDC7AG012990.2	Tt--TRITD7Av1G032080.4	0.1223838	0.19569741523526	0.625372624703907	1032	243.83333	788.16667	89	42	0.113	0.172	15.0536473

Td--TRIDC7AG012990.2	Tt--TRITD7Bv1G002620.4	0.1114694	0.288879048267642	0.385868852164262	1017	239.83333	777.16667	80.5	57.5	0.104	0.24	22.2214653
Td--TRIDC7BG001200.2	Tt--TRITD7Av1G032080.4	0.0012045	0.0905947344103324	0.0132949972833615	1077	246.08333	830.91667	1	21	0.001	0.085	6.96882572
Td--TRIDC7BG001200.2	Tt--TRITD7Bv1G002620.4	0	0	NaN	1002	227.5	774.5	0	0	0	0	0
Td--TRIDC7AG047060.4	Tt--TRITD7Av1G178880.7	0.001084	0.00370142639004825	0.292863051858581	1194	270.83333	923.16667	1	1	0.001	0.004	0.28472511
Td--TRIDC7AG047060.4	Tt--TRITD7Bv1G140030.6	0.002053	0.046637855797338	0.0440208502400651	1263	287.5	975.5	2	13	0.002	0.045	3.58752737
Td--TRIDC7BG040680.1	Tt--TRITD7Av1G178880.7	0.0010838	0.0417829552605042	0.0259391709072096	1194	270.66667	923.33333	1	11	0.001	0.041	3.21407348
Td--TRIDC7BG040680.1	Tt--TRITD7Bv1G140030.6	0	0.00698898476240196	0	1263	287.5	975.5	0	2	0	0.007	0.53761421
Td--TRIDC1AG061940.2	Tt--TRITD1Av1G222580.3	0	0	NaN	1794	433.16667	1360.8333	0	0	0	0	0
Td--TRIDC1AG061940.2	Tt--TRITD1Bv1G220000.2	0.0103686	0.130408872715655	0.0795085120382178	1794	434.41667	1359.5833	14	52	0.01	0.12	10.0314517
Td--TRIDC1AG061940.2	Tt--TRITD3Av1G151000.4	0.1574447	1.01236019368328	0.155522381456451	1767	420.91667	1346.0833	191.2	233.8	0.142	0.556	77.8738611
Td--TRIDC1AG061940.2	Tt--TRITD3Bv1G133770.2	0.1570731	1.01354713755119	0.154973678433744	1767	421.58333	1345.4167	190.7	234.3	0.142	0.556	77.9651644

Td--TRIDC1BG071030.1	Tt--TRITD1Av1G222580.3	0.0103712	0.127568052158476	0.0812991626219065	1794	434.75	1359.25	14	51	0.01	0.117	9.81292709
Td--TRIDC1BG071030.1	Tt--TRITD1Bv1G220000.2	0.0014634	0.00227100854823082	0.644389955989513	1809	441	1368	2	1	0.001	0.002	0.17469297
Td--TRIDC1BG071030.1	Tt--TRITD3Av1G151000.4	0.1547643	1.07938269349323	0.143382194654205	1770	424.41667	1345.5833	188.2	242.8	0.14	0.572	83.029438
Td--TRIDC1BG071030.1	Tt--TRITD3Bv1G133770.2	0.1562218	1.09056278359719	0.143248794480683	1770	425.08333	1344.9167	189.7	244.3	0.141	0.575	83.8894449
Td--TRIDC3AG036190.14	Tt--TRITD1Bv1G214990.2	0.1277327	1.20443932889738	0.106051564562706	1632	378.25	1253.75	147.3	226.7	0.117	0.599	92.6491791
Td--TRIDC3AG036190.14	Tt--TRITD3Av1G162410.11	0.0018861	0.00383796146691783	0.491427997516935	1719	391.83333	1327.1667	2.5	1.5	0.002	0.004	0.29522781
Td--TRIDC3AG036190.14	Tt--TRITD3Av1G151000.4	0.1966788	3.51600464367557	0.0559381427433389	1722	398.33333	1323.6667	229	296	0.173	0.743	270.461896
Td--TRIDC3AG036190.14	Tt--TRITD3Bv1G140820.6	0.0195556	0.0544862269973564	0.35890960291144	1752	396.41667	1355.5833	26.17	20.83	0.019	0.053	4.19124823
Td--TRIDC3AG036190.14	Tt--TRITD3Bv1G133770.2	0.193118	2.84012649172185	0.0679962580274387	1722	399.5	1322.5	225.2	292.8	0.17	0.733	218.471269
Td--TRIDC3AG034020.1	Tt--TRITD1Av1G222580.3	0.1503175	1.00567030638758	0.149469929592073	1767	419.83333	1347.1667	183.5	232.5	0.136	0.554	77.3592543
Td--TRIDC3AG034020.1	Tt--TRITD1Bv1G220000.2	0.1482243	1.07749635951193	0.137563641918064	1770	423	1347	181.2	241.8	0.134	0.572	82.8843353

Td--TRIDC3AG034020.1	Tt--TRITD3Av1G151000.4	0.0108752	0.0065532058424920 2	1.65951877570267	1839	434.25	1404.75	15.17	2.833	0.011	0.007	0.50409276
Td--TRIDC3AG034020.1	Tt--TRITD3Av1G162410.11	0.1925261	3.29332166767146	0.058459543209101	1722	396.91667	1325.0833	225	294	0.17	0.741	253.332436
Td--TRIDC3AG034020.1	Tt--TRITD3Bv1G133770.2	0.0137814	0.0518984629605249	0.265544858451341	1839	435.41667	1403.5833	19.17	21.83	0.014	0.05	3.99218946
Td--TRIDC3AG034020.1	Tt--TRITD3Bv1G140820.6	0.1967732	2.75912183869536	0.0713173284307232	1722	396	1326	229.5	289.5	0.173	0.731	212.240141
Td--TRIDC3BG040600.18	Tt--TRITD1Av1G209190.3	0.1327002	1.05675798332382	0.125572912115734	1626	373.5	1252.5	152.3	211.7	0.122	0.567	81.2890756
Td--TRIDC3BG040600.18	Tt--TRITD1Bv1G214990.2	0.1273799	1.22659501334797	0.103848399585952	1629	376.33333	1252.6667	146.8	227.2	0.117	0.604	94.3534626
Td--TRIDC3BG040600.18	Tt--TRITD3Av1G162410.11	0.0033956	0.0353700117425536	0.0960019907100345	1719	390.75	1328.25	4.5	13.5	0.003	0.035	2.72077013
Td--TRIDC3BG040600.18	Tt--TRITD3Av1G151000.4	0.1970924	2.88520013464622	0.0683115275885759	1722	396.91667	1325.0833	229.7	291.3	0.173	0.734	221.938472
Td--TRIDC3BG040600.18	Tt--TRITD3Bv1G140820.6	0.0172693	0.0149152642820377	1.15782553175512	1752	395	1357	23.17	5.833	0.017	0.015	1.14732802
Td--TRIDC3BG040600.18	Tt--TRITD3Bv1G133770.2	0.1935338	2.51818065472993	0.0768546286579587	1722	398.08333	1323.9167	225.8	288.2	0.171	0.724	193.706204
Td--TRIDC3BG038510.2	Tt--TRITD1Av1G222580.3	0.14905	1.00644010021721	0.148096197822231	1767	420.58333	1346.4167	182	233	0.135	0.554	77.4184692

Td--TRIDC3BG038510.2	Tt--TRITD1Bv1G220000.2	0.1487684	1.08820660651699	0.136709683435741	1770	423.75	1346.25	181.7	243.3	0.135	0.574	83.7082005
Td--TRIDC3BG038510.2	Tt--TRITD3Av1G151000.4	0.0130568	0.0518881805508732	0.251633954457995	1839	435.5	1403.5	18.17	21.83	0.013	0.05	3.9913985
Td--TRIDC3BG038510.2	Tt--TRITD3Av1G162410.11	0.1880273	2.73460036435454	0.068758616061087	1722	398.16667	1323.8333	220.2	290.8	0.166	0.73	210.353874
Td--TRIDC3BG038510.2	Tt--TRITD3Bv1G133770.2	0.0101709	0.00651678007566091	1.56071926804005	1839	436.66667	1402.3333	14.17	2.833	0.01	0.006	0.50129078
Td--TRIDC3BG038510.2	Tt--TRITD3Bv1G140820.6	0.1922561	2.43413962795926	0.0789831845357364	1722	397.25	1324.75	224.7	286.3	0.17	0.721	187.24151
Td--TRIDC6AG015690.2	Tt--TRITD6Av1G037430.3	0.0011749	0.00787925828566064	0.149107725072188	1107	255.16667	851.83333	1	2	0.001	0.008	0.60609679
Td--TRIDC6AG015690.2	Tt--TRITD6Bv1G050580.1	0	0.0917810348426671	0	1107	254.66667	852.33333	0	22	0	0.086	7.0600796
Td--TRIDC6AG015690.2	Tt--TRITD7Av1G227590.3	0.0557225	1.05263760077497	0.0529360624418997	1134	261.91667	872.08333	46.83	148.2	0.054	0.566	80.9721231
Td--TRIDC6BG021470.2	Tt--TRITD6Av1G037430.3	0.0011746	0.0872351457577674	0.0134650806662304	1107	255	852	1	21	0.001	0.082	6.71039583
Td--TRIDC6BG021470.2	Tt--TRITD6Bv1G050580.1	0	0.00790000720941963	0	1107	254.5	852.5	0	2	0	0.008	0.60769286
Td--TRIDC6BG021470.2	Tt--TRITD7Av1G227590.3	0.0640597	0.997810427044074	0.064200307641729	1161	270.66667	890.33333	54.67	149.3	0.061	0.552	76.7546482

Td--TRIDC7AG058780.3	Tt--TRITD6Av1G037430.3	0.0461855	1.04669985672484	0.0441248709000351	1107	254.91667	852.08333	38.17	143.8	0.045	0.564	80.5153736
Td--TRIDC7AG058780.3	Tt--TRITD6Bv1G050580.1	0.0449112	1.00476376392246	0.044698305506437	1107	254.41667	852.58333	37.17	140.8	0.044	0.554	77.2895203
Td--TRIDC7AG058780.3	Tt--TRITD7Av1G227590.3	0.0245178	0.0292131330286225	0.839272417346312	1152	267.58333	884.41667	21.33	7.667	0.024	0.029	2.24716408
Td--TRIDC7BG051510.2	Tt--TRITD6Av1G037430.3	0.0467909	1.10759507837085	0.0422455256133219	1107	254.58333	852.41667	38.67	147.3	0.045	0.579	85.1996214
Td--TRIDC7BG051510.2	Tt--TRITD6Bv1G050580.1	0.0455158	1.01512046885208	0.044837824284207	1107	254.08333	852.91667	37.67	141.3	0.044	0.556	78.0861899
Td--TRIDC7BG051510.2	Tt--TRITD7Av1G227590.3	0.0096275	0.0898739996454092	0.107122380206223	1236	295.16667	940.83333	9	25	0.01	0.085	6.91338459
Td--TRIDC1AG027930.1	Tt--TRITD1Bv1G113560.1	0	0.0728623919963763	0	1044	244.83333	799.16667	0	17	0	0.069	5.60479938
Td--TRIDC1BG032170.1	Tt--TRITD1Av1G120860.1	0.0023287	0.0704099735380411	0.0330740418354893	1128	267.83333	860.16667	2	18	0.002	0.067	5.41615181
Td--TRIDC1BG032170.1	Tt--TRITD1Bv1G113560.1	0	0	NaN	1128	268.16667	859.83333	0	0	0	0	0
Td--TRIDC1AG059290.6	Tt--TRITD1Av1G209190.3	0.0032584	0.005355970095081	0.608375263720713	1605	374.75	1230.25	4	2	0.003	0.005	0.4119977
Td--TRIDC1AG059290.6	Tt--TRITD3Av1G162410.11	0.1153425	1.0117602624987	0.114001786468474	1584	364.91667	1219.0833	130.3	202.7	0.107	0.555	77.8277125

Td--TRIDC1AG059290.6	Tt--TRITD3Bv1G140820.6	0.1160252	1.06436257300333	0.10900910751906	1587	365.25	1221.75	131.3	207.7	0.107	0.569	81.8740441
Td--TRIDC1BG068060.2	Tt--TRITD1Bv1G214990.2	0.0015692	0.00255428088523709	0.61435820229795	1668	392.16667	1275.8333	2	1	0.002	0.003	0.19648315
Td--TRIDC1BG068060.2	Tt--TRITD3Av1G162410.11	0.1231475	1.16995122268611	0.1052586386829	1635	376.58333	1258.4167	142.9	223.1	0.114	0.592	89.9962479
Td--TRIDC1BG068060.2	Tt--TRITD3Bv1G140820.6	0.1300115	1.2134906196101	0.107138444752693	1647	378.5	1268.5	151.4	227.6	0.119	0.601	93.3454323
Td--TRIDC6BG018000.3	Tt--TRITD6Av1G027670.1	0.0302909	0.0617473768900052	0.490562113552078	1449	337.41667	1111.5833	33	20	0.03	0.059	4.74979822
Td--TRIDC6BG018000.3	Tt--TRITD6Bv1G041120.1	0.0233785	0.0348020073141141	0.671757693922535	1446	338.16667	1107.8333	25.5	11.5	0.023	0.034	2.67707749
Td--TRIDC4AG065120.1	Tt--TRITD4Av1G251160.7	0.0043789	0.00338928917659708	1.29198571027517	1452	345	1107	4.833	1.167	0.004	0.003	0.26071455
Td--TRIDC4AG065120.1	Tt--TRITD7Av1G011740.1	0.0251763	0.117991980172379	0.213373233276526	1449	345	1104	27.33	37.67	0.025	0.109	9.07630617
Td--TRIDC7AG005070.14	Tt--TRITD4Av1G251160.7	0.0219167	0.10269342006903	0.213418820044597	1449	345.58333	1103.4167	23.83	33.17	0.022	0.096	7.89949385
Td--TRIDC7AG005070.14	Tt--TRITD7Av1G011740.1	0.0158514	0.0194673918947138	0.814255430943479	1452	346.91667	1105.0833	17.33	6.667	0.016	0.019	1.49749168
Td--TRIDC7AG057270.3	Tt--TRITD7Av1G221430.1	0.0117685	0.0216651642448627	0.543197301074161	1719	405.83333	1313.1667	15.33	8.667	0.012	0.021	1.6665511

Td-- TRIDC7AG057 270.3	Tt-- TRITD7Bv1G17 0250.1	0.0204151	0.1253922 17062182	0.1628096 68231129	1734	409.91667	1324.0833	26.67	47.33	0.02	0.115	9.64555516
Td-- TRIDC7BG0496 70.7	Tt-- TRITD7Av1G22 1430.1	0.0140936	0.1189994 8460855	0.1184344 92438824	1719	405.91667	1313.0833	18.33	44.67	0.014	0.11	9.15380651
Td-- TRIDC7BG0496 70.7	Tt-- TRITD7Bv1G17 0250.1	0.0163527	0.0237459 614298487	0.6886524 9879315	1734	410	1324	21.42	9.583	0.016	0.023	1.82661242
Td-- TRIDC6AG052 120.1	Tt-- TRITD6Bv1G20 2800.1	0.0068126	0.0997973 386553715	0.0682643 299265672	1116	305	811	5.5	28.5	0.007	0.093	7.67671836
Td-- TRIDC6AG052 120.1	Tt-- TRITD7Av1G04 8910.1	0.12237	0.4533652 48935098	0.2699148 18893932	1035	277.75	757.25	85.5	94.5	0.113	0.34	34.8742499
Td-- TRIDC6AG052 120.1	Tt-- TRITD7Bv1G02 2360.1	0.1100307	0.4080214 22500717	0.2696689 13604755	1017	272.75	744.25	76.17	85.83	0.102	0.315	31.3862633
Td-- TRIDC6BG0608 70.1	Tt-- TRITD6Bv1G20 2800.1	0	0.0032769 030829979 1	0	1116	305.83333	810.16667	0	1	0	0.003	0.25206947
Td-- TRIDC6BG0608 70.1	Tt-- TRITD7Av1G04 8910.1	0.1194667	0.4187600 99697434	0.2852867 6932556	1035	278.91667	756.08333	83.5	89.5	0.11	0.321	32.2123154
Td-- TRIDC6BG0608 70.1	Tt-- TRITD7Bv1G02 2360.1	0.1071057	0.3994710 13689992	0.2681187 96897122	1017	273.91667	743.08333	74.17	84.83	0.1	0.31	30.7285395
Td-- TRIDC4AG047 480.2	Tt-- TRITD4Av1G20 6810.2	0	0	NaN	1569	361.16667	1207.8333	0	0	0	0	0
Td-- TRIDC4AG047 480.2	Tt-- TRITD5Bv1G25 2270.3	0.0677548	0.2100996 05578762	0.3224891 04402995	1296	304.25	991.75	64.25	55.75	0.065	0.183	16.1615081

Td--TRIDC5BG083460.4	Tt--TRITD4Av1G206810.2	0.0125377	0.16947222514417	0.0739810322579846	1569	362.58333	1206.4167	15	55	0.012	0.152	13.036325
Td--TRIDC5BG083460.4	Tt--TRITD5Bv1G252270.3	0.0428727	0.101395307637	0.42282706860533	1239	289.08333	949.91667	39.58	27.42	0.042	0.095	7.79963905
Td--TRIDC5AG020820.4	Tt--TRITD5Av1G091640.3	0.0542024	0.086588072808598	0.625979776360957	843	200.75	642.25	33.58	16.42	0.052	0.082	6.66062099
Td--TRIDC5AG020820.4	Tt--TRITD5Bv1G075270.1	0.0167661	0.0684352487877035	0.244991678006738	885	211.5	673.5	11.17	13.83	0.017	0.065	5.26424991
Td--TRIDC5BG021620.3	Tt--TRITD5Av1G091640.3	0.0215665	0.057212478562223	0.376955266216645	816	196.66667	619.33333	13.17	10.83	0.021	0.055	4.40095989
Td--TRIDC5BG021620.3	Tt--TRITD5Bv1G075270.1	0.0135256	0.0488260693050803	0.277015875193783	1266	296.16667	969.83333	13	14	0.013	0.047	3.75585149
Td--TRIDC4AG041540.1	Tt--TRITD4Av1G192120.1	0.0305103	0.135016924595119	0.225974124026006	1011	275.16667	735.83333	22	34	0.03	0.124	10.3859173
Td--TRIDC4AG041540.1	Tt--TRITD4Bv1G014100.1	0.2379509	0.506401622956579	0.469885710906565	984	270	714	145.6	99.42	0.204	0.368	38.953971
Td--TRIDC4BG007520.1	Tt--TRITD4Av1G192120.1	0.2392845	0.525311556754041	0.45550961817642	984	268.5	715.5	146.6	101.4	0.205	0.378	40.4085813
Td--TRIDC4BG007520.1	Tt--TRITD4Bv1G014100.1	0.0013417	0.0142691581083784	0.0940267058401689	1029	283	746	1	4	0.001	0.014	1.09762755
Td--TRIDC3BG008530.2	Tt--TRITD3Bv1G017840.1	0.0326584	0.0281619247560474	1.15966450543059	975	268.33333	706.66667	22.58	7.417	0.032	0.028	2.1663019

Td--TRIDC2AG058830.1	Tt--TRITD2Bv1G201730.10	0.0244122	0.0790440680872091	0.308843474581227	2460	586.5	1873.5	45	44	0.024	0.075	6.08031293
Td--TRIDC2AG058830.1	Tt--TRITD6Av1G156460.10	0.1517447	0.907555974135041	0.167201525921507	2427	579.91667	1847.0833	253.8	305.3	0.137	0.526	69.811998
Td--TRIDC2AG058830.1	Tt--TRITD6Bv1G142910.11	0.1371448	0.873582894625792	0.156991213467956	2364	567.5	1796.5	225.2	292.8	0.125	0.516	67.1986842
Td--TRIDC2BG061830.1	Tt--TRITD2Bv1G201730.10	0.0004933	0.00156658020404368	0.314863428603852	2667	639	2028	1	1	5E-04	0.002	0.12050617
Td--TRIDC2BG061830.1	Tt--TRITD6Av1G156460.10	0.1586372	0.860845215308567	0.184280777719342	2616	628.58333	1987.4167	284.2	321.8	0.143	0.512	66.2188627
Td--TRIDC2BG061830.1	Tt--TRITD6Bv1G142910.11	0.1587558	0.832942555202414	0.190596379634392	2616	629.91667	1986.0833	284.2	316.8	0.143	0.503	64.0725042
Td--TRIDC6AG037840.4	Tt--TRITD2Bv1G201730.10	0.1586224	0.8612732446822	0.18417199541233	2616	628.41667	1987.5833	284.2	321.8	0.143	0.512	66.2517881
Td--TRIDC6AG037840.4	Tt--TRITD6Av1G156460.10	0.0005001	0		2634	633.83333	2000.1667	1	0	5E-04	0	0
Td--TRIDC6AG037840.4	Tt--TRITD6Bv1G142910.11	0.0065322	0.0371143211932183	0.17600099691641	2634	635.16667	1998.8333	13	23	0.007	0.036	2.85494778
Td--TRIDC6BG044800.1	Tt--TRITD2Bv1G201730.10	0.1593634	0.833346822418081	0.191232979049828	2616	629.75	1986.25	285.2	316.8	0.144	0.503	64.1036017
Td--TRIDC6BG044800.1	Tt--TRITD6Av1G156460.10	0.007037	0.0371143211932183	0.189603240847122	2634	635.16667	1998.8333	14	23	0.007	0.036	2.85494778

Td--TRIDC6BG044800.1	Tt--TRITD6Bv1G142910.11	0	0	NaN	2634	636.5	1997.5	0	0	0	0	0
Td--TRIDC4AG043860.6	Tt--TRITD4Av1G198600.3	0.0007756	0.00692845573870227	0.111943371064782	1725	435	1290	1	3	0.001	0.007	0.53295813
Td--TRIDC4BG004990.4	Tt--TRITD4Av1G198600.3	0.0203814	0.178250233899183	0.114341632462028	1728	434.91667	1293.0833	26	69	0.02	0.159	13.7115565
Td--TRIDC4AG012750.6	Tt--TRITD4Av1G042360.2	0	0	NaN	2130	515.66667	1614.3333	0	0	0	0	0
Td--TRIDC4AG012750.6	Tt--TRITD4Bv1G128900.2	0.0081591	0.0715313693800487	0.114062911879357	2115	513	1602	13	35	0.008	0.068	5.50241303
Td--TRIDC4BG037560.5	Tt--TRITD4Av1G042360.2	0.007833	0.071328011617978	0.109816174503288	2025	485	1540	12	33	0.008	0.068	5.48677012
Td--TRIDC4BG037560.5	Tt--TRITD4Bv1G128900.2	0.0006496	0.00413651513929987	0.157031115488524	2025	484.83333	1540.1667	1	2	0.001	0.004	0.31819347
Td--TRIDC2AG025680.4	Tt--TRITD2Av1G072210.1	0.0380104	0.0606793079162398	0.626413831984514	1527	348.83333	1178.1667	43.67	20.33	0.037	0.058	4.66763907
Td--TRIDC2AG025680.4	Tt--TRITD2Bv1G081010.1	0.0104356	0.0640611878648543	0.162901272124366	1629	374.58333	1254.4167	13	23	0.01	0.061	4.92778368
Td--TRIDC2BG030100.5	Tt--TRITD2Av1G072210.1	0.0397752	0.11236887939933	0.353969672394324	1527	348.16667	1178.8333	45.67	36.33	0.039	0.104	8.64375995
Td--TRIDC2BG030100.5	Tt--TRITD2Bv1G081010.1	0.0072053	0.00806640085646106	0.893253313547796	1629	373.91667	1255.0833	9	3	0.007	0.008	0.62049237

Td--TRIDC1BG061690.1	Tt--TRITD3Av1G171650.1	0.2284842	0.578995916315227	0.394621362950363	1035	267.08333	767.91667	151.2	107.8	0.197	0.403	44.5381474
Td--TRIDC1BG061690.1	Tt--TRITD3Bv1G151660.1	0.2292926	0.58678582296574	0.39076036136575	1044	266.16667	777.83333	153.7	108.3	0.198	0.407	45.137371
Td--TRIDC3BG043440.2	Tt--TRITD3Bv1G151660.1	0.0051516	0		1305	331.08333	973.91667	5	0	0.005	0	0
Td--TRIDC1AG053880.1	Tt--TRITD1Av1G205950.1	0.0021284	0		609	138.5	470.5	1	0	0.002	0	0
Td--TRIDC1AG053880.1	Tt--TRITD1Bv1G200860.1	0.0284177	0.100148544468392	0.283755478883934	1485	373.33333	1111.6667	31	35	0.028	0.094	7.70373419
Td--TRIDC1AG053880.1	Tt--TRITD3Av1G171180.1	0.2861648	0.56346660719439	0.507864782488311	1092	279.33333	812.66667	193.3	110.7	0.238	0.396	43.3435852
Td--TRIDC1AG053880.1	Tt--TRITD3Bv1G150780.1	0.3071565	0.576841599716673	0.532479769666476	1035	266.91667	768.08333	193.6	107.4	0.252	0.402	44.3724307
Td--TRIDC3AG038300.1	Tt--TRITD1Bv1G200860.1	0.3239761	0.54031312010137	0.599608128188567	1239	311.83333	927.16667	243.9	120.1	0.263	0.385	41.5625477
Td--TRIDC3AG038300.1	Tt--TRITD3Av1G171180.1	0.2227947	0.537274102996463	0.414676108156998	1098	279.58333	818.41667	157.8	107.3	0.193	0.384	41.3287772
Td--TRIDC3AG038300.1	Tt--TRITD3Bv1G150780.1	0.2326162	0.52818246389706	0.440408783191285	1041	266.83333	774.16667	154.8	101.2	0.2	0.379	40.6294203
Td--TRIDC3BG043280.1	Tt--TRITD1Bv1G200860.1	0.277579	0.622344559877688	0.446021476202718	1167	299.91667	867.08333	201.2	126.8	0.232	0.423	47.8726585

Td--TRIDC3BG043280.1	Tt--TRITD3Av1G171180.1	0.2402904	0.573503774226506	0.418986562156567	1113	282.5	830.5	170.8	113.3	0.206	0.401	44.1156749
Td--TRIDC3BG043280.1	Tt--TRITD3Bv1G150780.1	0.2475903	0.606865769753381	0.407981946869876	1056	269.58333	786.41667	165.8	112.2	0.211	0.416	46.6819823
Td--TRIDC5AG066400.8	Tt--TRITD5Av1G228010.3	0.0031244	0.00525281201683735	0.594804130834112	1665	382.08333	1282.9167	4	2	0.003	0.005	0.40406246
Td--TRIDC5AG066400.8	Tt--TRITD5Bv1G226960.4	0.0016261	0.100895472487721	0.0161169478497392	1602	370.75	1231.25	2	35	0.002	0.094	7.76119019
Td--TRIDC5BG071320.5	Tt--TRITD5Av1G228010.3	0.0503831	0.179806254805906	0.280207954752309	1620	378.41667	1241.5833	60.5	60.5	0.049	0.16	13.8312504
Td--TRIDC5BG071320.5	Tt--TRITD5Bv1G226960.4	0.0372988	0.0418790883787865	0.890631118594394	1551	360.08333	1190.9167	43.33	14.67	0.036	0.041	3.22146834
Td--TRIDC5AG020280.1	Tt--TRITD5Av1G086470.1	0.0495012	0.0652585327422225	0.758539647030267	1596	380	1216	58.25	23.75	0.048	0.063	5.01988713
Td--TRIDC5AG032410.5	Tt--TRITD5Bv1G121250.4	0.0019256	0.0619250067196664	0.0310964782888502	2031	471.08333	1559.9167	3	28	0.002	0.059	4.76346206
Td--TRIDC5AG032410.5	Tt--TRITD0Uv1G042030.1	0.0245754	0.0289733090894047	0.848208089307106	1632	363.58333	1268.4167	30.67	10.33	0.024	0.028	2.22871608
Td--TRIDC5BG034210.7	Tt--TRITD5Bv1G121250.4	0.0006381	0		2043	475.16667	1567.8333	1	0	0.001	0	0
Td--TRIDC5BG034210.7	Tt--TRITD0Uv1G042030.1	0.026203	0.0884877733817011	0.29611949321391	1632	363.41667	1268.5833	32.67	30.33	0.026	0.083	6.8067518

Td--TRIDC2AG070140.10	Tt--TRITD2Av1G270510.6	0.0013128	0		3966	916.5	3049.5	4	0	0.001	0	0
Td--TRIDC2AG070140.10	Tt--TRITD2Bv1G233690.10	0.0082466	0.0828666073744607	0.0995165506588824	3966	917.75	3048.25	25	72	0.008	0.078	6.37435441
Td--TRIDC2BG075830.1	Tt--TRITD2Av1G270510.6	0.0089088	0.0804830033446936	0.110691578926508	3966	917.25	3048.75	27	70	0.009	0.076	6.19100026
Td--TRIDC2BG075830.1	Tt--TRITD2Bv1G233690.10	0.0006561	0.00217825529772948	0.301219119629607	3969	919.5	3049.5	2	2	0.001	0.002	0.1675581
Td--TRIDC6AG020750.1	Tt--TRITD6Av1G054950.1	0.0007911	0		1626	361.33333	1264.6667	1	0	0.001	0	0
Td--TRIDC6AG020750.1	Tt--TRITD6Bv1G065890.1	0.013174	0.120976973211965	0.108896685544764	1626	362.5	1263.5	16.5	40.5	0.013	0.112	9.30592102
Td--TRIDC6BG026600.3	Tt--TRITD6Av1G054950.1	0.0123665	0.121067579367253	0.102145258798394	1626	362.25	1263.75	15.5	40.5	0.012	0.112	9.31289072
Td--TRIDC6BG026600.3	Tt--TRITD6Bv1G065890.1	0.0015857	0		1626	363.41667	1262.5833	2	0	0.002	0	0
Td--TRIDC5AG057130.2	Tt--TRITD5Av1G205000.3	0.0022727	0.00734399992846323	0.309467461059725	2310	547.33333	1762.6667	4	4	0.002	0.007	0.56492307
Td--TRIDC5AG057130.2	Tt--TRITD5Bv1G200940.1	0.008856	0.060364830835347	0.146707831087657	2469	594.83333	1874.1667	16.5	34.5	0.009	0.058	4.64344853
Td--TRIDC5BG061270.2	Tt--TRITD5Av1G205000.3	0.0072934	0.0634324681209124	0.114979168161857	2163	509.66667	1653.3333	12	31	0.007	0.061	4.87942062

Td--TRIDC5BG061270.2	Tt--TRITD5Bv1G200940.1	0.0011345	0.00901724117215036	0.125818684235447	2322	557.83333	1764.1667	2	5	0.001	0.009	0.69363394
Td--TRIDC5AG032000.2	Tt--TRITD5Bv1G119650.1	0.0201853	0.0936472148657488	0.215545948366101	1554	382.41667	1171.5833	23.33	33.67	0.02	0.088	7.20363191
Td--TRIDC5AG032000.2	Tt--TRITD0Uv1G047610.1	0.0037007	0.00970744541864889	0.38122531414588	1554	380.16667	1173.8333	4.333	3.667	0.004	0.01	0.74672657
Td--TRIDC5BG033660.2	Tt--TRITD5Bv1G119650.1	0.0008562	0		1554	385.33333	1168.6667	1	0	0.001	0	0
Td--TRIDC5BG033660.2	Tt--TRITD0Uv1G047610.1	0.0172781	0.0944599268834398	0.182915004632196	1554	383.08333	1170.9167	20	34	0.017	0.089	7.26614822
Td--TRIDC2AG010980.4	Tt--TRITD2Av1G024070.6	0	0.00207451078759352	0	3996	965.41667	3030.5833	0	2	0	0.002	0.15957775
Td--TRIDC2AG010980.4	Tt--TRITD2Bv1G031320.6	0.0072973	0.0796098003311754	0.0916634636871207	3996	966.5	3029.5	22	73	0.007	0.076	6.12383079
Td--TRIDC2BG013430.2	Tt--TRITD2Av1G024070.6	0.0072971	0.0796170426266173	0.0916525921802457	3996	966.41667	3029.5833	22	73	0.007	0.076	6.12438789
Td--TRIDC2BG013430.2	Tt--TRITD2Bv1G031320.6	0	0	NaN	4005	969.83333	3035.1667	0	0	0	0	0
Td--TRIDC5AG024780.6	Tt--TRITD5Av1G112160.1	0.0006988	0.0062849529800631	0.111188114269111	1911	479.33333	1431.6667	1	3	0.001	0.006	0.48345792
Td--TRIDC5AG024780.6	Tt--TRITD5Bv1G092620.2	0.0091459	0.0887460629299847	0.103057175454398	1908	477.91667	1430.0833	13	40	0.009	0.084	6.82662023

Td--TRIDC5BG025890.5	Tt--TRITD5Av1G112160.1	0.0084374	0.0887789295178338	0.0950385889968959	1908	477.75	1430.25	12	40	0.008	0.084	6.82914842
Td--TRIDC5BG025890.5	Tt--TRITD5Bv1G092620.2	0	0.0020862322214076	0	1920	480	1440	0	1	0	0.002	0.1604794
Td--TRIDC6AG039240.3	Tt--TRITD6Av1G162640.1	0.0234732	0.019717056650243	1.19049999211312	1167	265.5	901.5	20.83	5.167	0.023	0.019	1.51669667
Td--TRIDC6AG039240.3	Tt--TRITD6Bv1G148270.1	0.0623976	0.0795182990242753	0.784695177566379	1167	265.08333	901.91667	54	20	0.06	0.075	6.11679223
Td--TRIDC6BG046090.3	Tt--TRITD6Av1G162640.1	0.0307168	0.0778452659565614	0.394587263577064	1848	419.25	1428.75	43	31	0.03	0.074	5.98809738
Td--TRIDC6BG046090.3	Tt--TRITD6Bv1G148270.1	0.0006986	0		1851	419	1432	1	0	0.001	0	0
Td--TRIDC2AG025210.3	Tt--TRITD2Av1G069790.2	0	0	NaN	2079	480.16667	1598.8333	0	0	0	0	0
Td--TRIDC2AG025210.3	Tt--TRITD2Bv1G079430.3	0.0091225	0.0825309635481236	0.11053400565534	2079	479.83333	1599.1667	14.5	37.5	0.009	0.078	6.34853566
Td--TRIDC2BG029650.2	Tt--TRITD2Av1G069790.2	0.0068285	0.0782042588314467	0.0873156730442644	2103	484.75	1618.25	11	36	0.007	0.074	6.01571222
Td--TRIDC2BG029650.2	Tt--TRITD2Bv1G079430.3	0.0021593	0.0072401872384622	0.298234484444953	2109	485.75	1623.25	3.5	3.5	0.002	0.007	0.55693748
Td--TRIDC2AG024350.1	Tt--TRITD2Av1G066770.1	0.0869896	0.11193563419003	0.777139866011752	972	236.41667	735.58333	60.42	24.58	0.082	0.104	8.6104334

Td--TRIDC2AG024350.1	Tt--TRITD2Bv1G076660.1	0.0109879	0.075872891291839	0.144820496226415	969	235.58333	733.41667	8	17	0.011	0.072	5.83637625
Td--TRIDC2BG028790.2	Tt--TRITD2Av1G069790.2	0.4536053	0.741593361128086	0.611663123071611	888	212.5	675.5	229.9	100.1	0.34	0.471	57.0456432
Td--TRIDC2BG028790.2	Tt--TRITD2Av1G066770.1	0.0775346	0.197701006191836	0.392181160108862	969	235.91667	733.08333	54	41	0.074	0.174	15.2077697
Td--TRIDC2BG028790.2	Tt--TRITD2Bv1G076660.1	0.0013667	0		969	236.66667	732.33333	1	0	0.001	0	0
Td--TRIDC1AG002650.1	Tt--TRITD0Uv1G000510.7	0.0014288	0		1815	413.91667	1401.0833	2	0	0.001	0	0
Td--TRIDC1AG002650.1	Tt--TRITD0Uv1G004130.5	0.0184536	0.118196062931122	0.156126715834684	1815	416.08333	1398.9167	25.5	45.5	0.018	0.109	9.09200484
Td--TRIDC6AG024780.3	Tt--TRITD6Av1G073760.2	0.1832943	0.27129041935898	0.675638645537406	822	205	617	100.3	46.67	0.163	0.228	20.8684938
Td--TRIDC6AG024780.3	Tt--TRITD7Av1G209280.1	0.3155857	0.934037145898307	0.337872772954532	846	210	636	163.8	112.2	0.258	0.534	71.8490112
Td--TRIDC6AG024780.3	Tt--TRITD7Bv1G162110.1	0.3153921	0.950547545282606	0.331800484979582	846	210.33333	635.66667	163.7	113.3	0.257	0.539	73.1190419
Td--TRIDC7AG054160.3	Tt--TRITD6Av1G073760.2	0.2072379	0.585928508332545	0.353691419239156	1098	266.83333	831.16667	150.5	108.5	0.181	0.407	45.0714237
Td--TRIDC7AG054160.3	Tt--TRITD6Bv1G077930.1	0.2046463	0.590843810669946	0.346362784436033	1098	267	831	148.8	109.2	0.179	0.409	45.4495239

Td--TRIDC7AG054160.3	Tt--TRITD7Av1G209280.1	0	0.00702581250270012	0	1188	286	902	0	2	0	0.007	0.54044712
Td--TRIDC7AG054160.3	Tt--TRITD7Bv1G162110.1	0.0011099	0.0505606560281201	0.0219514184182557	1188	286.33333	901.66667	1	14	0.001	0.049	3.88928123
Td--TRIDC7BG046810.3	Tt--TRITD6Av1G073760.2	0.2106117	0.567610355823165	0.371049794291614	1098	267.5	830.5	152.5	106.5	0.184	0.398	43.6623351
Td--TRIDC7BG046810.3	Tt--TRITD6Bv1G077930.1	0.2080071	0.57240603590732	0.363390740687512	1098	267.66667	830.33333	150.8	107.2	0.182	0.4	44.0312335
Td--TRIDC7BG046810.3	Tt--TRITD7Av1G209280.1	0.0033358	0.0617782480748455	0.0539965101411712	1188	286.66667	901.33333	3	17	0.003	0.059	4.75217293
Td--TRIDC7BG046810.3	Tt--TRITD7Bv1G162110.1	0.002223	0.00349243937246322	0.63653136473622	1188	287	901	2	1	0.002	0.003	0.26864918
Td--TRIDC1AG050190.1	Tt--TRITD1Av1G197990.2	0.0014657	0.00230680689353918	0.635397282851082	1800	434.16667	1365.8333	2	1	0.001	0.002	0.17744668
Td--TRIDC1AG050190.1	Tt--TRITD1Bv1G187410.2	0.007386	0.134508289498696	0.0549114011556151	1791	430.41667	1360.5833	10	53	0.007	0.123	10.3467915
Td--TRIDC1AG050190.1	Tt--TRITD3Av1G181840.2	0.3345657	1.28815173176702	0.25972541647255	1734	411	1323	357.1	252.9	0.27	0.615	99.0885948
Td--TRIDC1AG050190.1	Tt--TRITD3Bv1G162740.2	0.3259125	1.25624537336748	0.259433814973163	1731	409.75	1321.25	349.3	249.7	0.264	0.61	96.6342595
Td--TRIDC1BG057000.1	Tt--TRITD1Av1G197990.2	0.0059023	0.129023890418143	0.0457455942832789	1791	430.25	1360.75	8	51	0.006	0.119	9.92491465

Td--TRIDC1BG057000.1	Tt--TRITD1Bv1G187410.2	0	0.00232829057765231	0	1794	430.16667	1363.8333	0	1	0	0.002	0.17909928
Td--TRIDC1BG057000.1	Tt--TRITD3Av1G181840.2	0.3342959	1.27396442395981	0.262405958611797	1734	408.91667	1325.0833	357.4	250.6	0.27	0.613	97.9972634
Td--TRIDC1BG057000.1	Tt--TRITD3Bv1G162740.2	0.3230436	1.24413075712993	0.259654043033045	1731	408	1323	347.2	247.7	0.262	0.607	95.7023659
Td--TRIDC3AG040710.1	Tt--TRITD1Av1G197990.2	0.3313186	1.30007197761386	0.254846344381073	1734	410.66667	1323.3333	354.4	253.6	0.268	0.617	100.005537
Td--TRIDC3AG040710.1	Tt--TRITD1Bv1G187410.2	0.3168141	1.30084734326506	0.243544394626008	1704	402.75	1301.25	336.2	248.8	0.258	0.618	100.06518
Td--TRIDC3AG040710.1	Tt--TRITD3Av1G181840.2	0.001446	0		1803	418.5	1384.5	2	0	0.001	0	0
Td--TRIDC3AG040710.1	Tt--TRITD3Bv1G162740.2	0.0208983	0.0663762067337091	0.314846759708261	1800	417.16667	1382.8333	28.5	26.5	0.021	0.064	5.10586206
Td--TRIDC3BG045870.3	Tt--TRITD1Av1G197990.2	0.322262	1.24723019157164	0.258382101941531	1731	409.25	1321.75	346.3	248.7	0.262	0.608	95.940784
Td--TRIDC3BG045870.3	Tt--TRITD1Bv1G187410.2	0.3212048	1.26479921517767	0.253957108814932	1731	408	1323	345.7	249.3	0.261	0.611	97.2922473
Td--TRIDC3BG045870.3	Tt--TRITD3Av1G181840.2	0.0205241	0.0650950450031555	0.315294729559683	1800	417	1383	28	26	0.02	0.062	5.00731115
Td--TRIDC3BG045870.3	Tt--TRITD3Bv1G162740.2	0.0007219	0		1803	417.16667	1385.8333	1	0	0.001	0	0

Td--TRIDC7AG009510.2	Tt--TRITD7Av1G024300.1	0.0051209	0.0113746256035582	0.450206490956674	765	177.16667	587.83333	3	2	0.005	0.011	0.8749712
Td--TRIDC7AG012240.3	Tt--TRITD7Av1G030560.1	0.0157936	0.0297212119320387	0.531392181055493	1962	469	1493	23.33	13.67	0.016	0.029	2.28624707
Td--TRIDC6AG042270.3	Tt--TRITD6Av1G173820.1	0.1118106	0.178767375370161	0.62545314169559	1182	293.91667	888.08333	92.25	46.75	0.104	0.159	13.7513366
Td--TRIDC6AG042270.3	Tt--TRITD6Bv1G161420.1	0.1472336	0.281974552836387	0.522152146382099	1188	297.83333	890.16667	119	70	0.134	0.235	21.6903502
Td--TRIDC6AG042270.3	Tt--TRITD7Av1G083120.2	0.2577164	0.545085748949433	0.472799677421988	1146	289.75	856.25	186.8	112.3	0.218	0.387	41.929673
Td--TRIDC6AG042270.3	Tt--TRITD7Bv1G058080.2	0.4579181	1.22730677190666	0.373108094901961	591	158.66667	432.33333	148.2	95.83	0.343	0.604	94.4082132
Td--TRIDC6BG049330.2	Tt--TRITD6Av1G173820.1	0.0202785	0.086438668040675	0.234600233027205	1287	312.33333	974.66667	19.5	25.5	0.02	0.082	6.64912831
Td--TRIDC6BG049330.2	Tt--TRITD6Bv1G161420.1	0.0010471	0.00326087470207754	0.321116474752615	1263	307.33333	955.66667	1	1	0.001	0.003	0.25083652
Td--TRIDC6BG049330.2	Tt--TRITD7Av1G083120.2	0.1867691	0.448504124127258	0.416426642189408	1281	314.75	966.25	159.8	106.3	0.165	0.338	34.5003172
Td--TRIDC6BG049330.2	Tt--TRITD7Bv1G058080.2	0.4300403	1.33915637446288	0.32112774504063	597	160.33333	436.66667	142.9	100.1	0.327	0.624	103.012029
Td--TRIDC7AG029400.6	Tt--TRITD6Av1G173820.1	0.1931971	0.365466659887451	0.528631273689721	1257	310.25	946.75	161.3	89.75	0.17	0.289	28.11282

Td--TRIDC7AG029400.6	Tt--TRITD6Bv1G161420.1	0.1677721	0.426692427367973	0.393192004991309	1251	308.08333	942.91667	141.8	100.3	0.15	0.325	32.8224944
Td--TRIDC7AG029400.6	Tt--TRITD7Av1G083120.2	0.0010373	0		1284	319.33333	964.66667	1	0	0.001	0	0
Td--TRIDC7AG029400.6	Tt--TRITD7Bv1G058080.2	0.3318493	0.707801022400452	0.468845411243417	663	180.08333	482.91667	129.5	82.5	0.268	0.458	54.4462325
Td--TRIDC7BG020300.3	Tt--TRITD6Av1G173820.1	0.157325	0.334822578745814	0.469875631139311	1143	279.25	863.75	122.6	75.42	0.142	0.27	25.755583
Td--TRIDC7BG020300.3	Tt--TRITD6Bv1G161420.1	0.1559019	0.376109821010814	0.414511533793417	1143	278.08333	864.91667	121.8	82.25	0.141	0.296	28.9315247
Td--TRIDC7BG020300.3	Tt--TRITD7Av1G083120.2	0.0011612	0.0669174226371055	0.0173529641708794	1143	281.16667	861.83333	1	18	0.001	0.064	5.14749405
Td--TRIDC7BG020300.3	Tt--TRITD7Bv1G058080.2	0.381206	0.821231940768545	0.464187926158886	507	137.25	369.75	110.5	68.5	0.299	0.499	63.1716878
Td--TRIDC1AG052430.1	Tt--TRITD1Av1G203640.1	0.0211809	0.0373429983772616	0.567199481468634	2343	583.33333	1759.6667	36.75	21.25	0.021	0.036	2.87253834
Td--TRIDC1AG052430.1	Tt--TRITD3Av1G164820.1	0.1147778	0.395707852589574	0.290056801465112	1602	380.5	1221.5	130	117	0.106	0.307	30.4390656
Td--TRIDC1AG052430.1	Tt--TRITD3Bv1G142220.1	0.3514902	2.11776390663665	0.165972335986967	2346	557.08333	1788.9167	502	393	0.281	0.705	162.904916
Td--TRIDC1BG059980.1	Tt--TRITD1Av1G203640.1	0.0545668	0.143480067903826	0.380309408704363	2331	581.33333	1749.6667	92.08	75.92	0.053	0.131	11.0369283

Td--TRIDC1BG059980.1	Tt--TRITD1Av1G201620.1	0.0487389	0.176322278082073	0.276419691227632	372	85.916667	286.08333	13.5	13.5	0.047	0.157	13.5632522
Td--TRIDC1BG059980.1	Tt--TRITD1Bv1G199190.1	0.1210778	0.317467594792552	0.381386267631744	2376	587.25	1788.75	200	152	0.112	0.259	24.4205842
Td--TRIDC1BG059980.1	Tt--TRITD3Av1G164820.1	0.1203193	0.357928291418113	0.33615475446977	1605	383.83333	1221.1667	135.8	109.3	0.111	0.285	27.5329455
Td--TRIDC3AG036880.3	Tt--TRITD1Av1G203640.1	0.2588967	0.648928981782857	0.398959982918882	1953	489.5	1463.5	320.4	212.6	0.219	0.434	49.917614
Td--TRIDC3AG036880.3	Tt--TRITD1Bv1G199190.1	0.2108178	0.600137735044423	0.351282334416608	2019	501.33333	1517.6667	278.9	207.1	0.184	0.413	46.1644412
Td--TRIDC3AG036880.3	Tt--TRITD3Av1G164820.1	0.006696	0.00424299271808696	1.57813720236106	1644	393.91667	1250.0833	8.333	1.667	0.007	0.004	0.32638406
Td--TRIDC3AG036880.3	Tt--TRITD3Bv1G142350.1	0.1260918	0.301569733812641	0.418118256727455	2043	505.75	1537.25	178.4	125.6	0.116	0.248	23.1976718
Td--TRIDC3BG040940.1	Tt--TRITD1Bv1G199190.1	0.3541204	1.80737790825242	0.195930450882675	2349	556.91667	1792.0833	505.8	380.2	0.282	0.683	139.02907
Td--TRIDC3BG040940.1	Tt--TRITD3Av1G164850.1	0.2528121	1.54787213105319	0.163328814856083	2337	534.91667	1802.0833	386.8	350.3	0.215	0.655	119.067087
Td--TRIDC3BG040940.1	Tt--TRITD3Bv1G142220.1	0	0.00179910131248118	0	2433	556.5	1876.5	0	1	0	0.002	0.13839241
Td--TRIDC7BG067700.1	Tt--TRITD7Bv1G217320.3	0.168896	0.589916692738967	0.286304884632633	585	134.25	450.75	68.17	54.83	0.151	0.408	45.3782071

Td--TRIDC7BG067700.1	Tt--TRITD7Bv1G218360.3	0.1743293	0.592152417662069	0.294399335647513	585	133.91667	451.08333	70.17	54.83	0.156	0.409	45.550186
Td--TRIDC1AG061210.1	Tt--TRITD1Bv1G218630.1	0.0019934	0.154389040653111	0.012911257474677	660	157.66667	502.33333	1	22	0.002	0.14	11.8760801
Td--TRIDC1AG061210.1	Tt--TRITD3Av1G155690.2	0.0610403	0.798414951807141	0.0764518250583799	894	211.66667	682.33333	40	104	0.059	0.491	61.4165348
Td--TRIDC1AG061210.1	Tt--TRITD3Bv1G135290.1	0.0610403	0.798414951807141	0.0764518250583799	894	211.66667	682.33333	40	104	0.059	0.491	61.4165348
Td--TRIDC1BG070170.4	Tt--TRITD1Bv1G218630.1	0	0.117143011731485	0	660	156.75	503.25	0	17	0	0.108	9.0110009
Td--TRIDC1BG070170.4	Tt--TRITD3Av1G155690.2	0.0625284	0.733807692263891	0.0852109406220229	918	217.91667	700.08333	42	102	0.06	0.468	56.4467456
Td--TRIDC1BG070170.4	Tt--TRITD3Bv1G135290.1	0.0625284	0.721698448387851	0.0866406791273554	918	217.91667	700.08333	42	101	0.06	0.463	55.5152653
Td--TRIDC3AG034530.2	Tt--TRITD1Bv1G218630.1	0.0648148	0.709558818050817	0.0913452342297723	654	154.75	499.25	31	71	0.062	0.459	54.5814475
Td--TRIDC3AG034530.2	Tt--TRITD3Av1G155690.2	0	0	NaN	945	222.33333	722.66667	0	0	0	0	0
Td--TRIDC3AG034530.2	Tt--TRITD3Bv1G135290.1	0	0.0760143706952155	0	933	221.33333	711.66667	0	16	0	0.072	5.84725928
Td--TRIDC3BG038930.3	Tt--TRITD1Bv1G218630.1	0.0648148	0.693097251872527	0.0935147502887126	654	154.75	499.25	31	70	0.062	0.452	53.3151732

Td--TRIDC3BG038930.3	Tt--TRITD3Av1G155690.2	0	0.0761350440528097	0	942	221	721	0	16	0	0.072	5.85654185
Td--TRIDC3BG038930.3	Tt--TRITD3Bv1G135290.1	0.0097903	0		942	222.33333	719.66667	7	0	0.01	0	0
Td--TRIDC2AG028890.5	Tt--TRITD2Av1G084730.2	0.4427342	2.60060537632376	0.170242735787871	1944	463.91667	1480.0833	494.9	337.1	0.334	0.727	200.046567
Td--TRIDC2BG032550.5	Tt--TRITD2Av1G084730.2	0.453775	2.98999431599426	0.151764509822024	1947	465.41667	1481.5833	504.4	342.6	0.34	0.736	229.999563
Td--TRIDC2BG032550.5	Tt--TRITD2Bv1G090370.1	0	0	NaN	2100	518.33333	1581.6667	0	0	0	0	0
Td--TRIDC6BG030200.2	Tt--TRITD6Bv1G077930.1	0.001183	0.00359282124197445	0.32925811368404	1125	279	846	1	1	0.001	0.004	0.27637086
Td--TRIDC6BG030200.2	Tt--TRITD7Av1G209280.1	0.2010591	0.609133407760861	0.330073993985945	1098	267.33333	830.66667	146.5	111.5	0.176	0.417	46.856416
Td--TRIDC6BG030200.2	Tt--TRITD7Bv1G162110.1	0.2011517	0.583168552429674	0.344928871658064	1098	267.66667	830.33333	146.5	108.5	0.176	0.405	44.8591194
Td--TRIDC4AG047400.2	Tt--TRITD4Av1G206190.2	0.0008098	0.0025652013035985	0.315696848032724	3252	781	2471	2	2	0.001	0.003	0.19732318
Td--TRIDC5BG083500.1	Tt--TRITD4Av1G206190.2	0.1490584	0.402038536940989	0.370756551794264	2946	714.41667	2231.5833	301.7	222.3	0.135	0.311	30.9260413
Td--TRIDC7AG002140.2	Tt--TRITD7Av1G004690.5	0.0179356	0.0364042045856986	0.492680379335556	1008	232.16667	775.83333	13.75	8.25	0.018	0.036	2.80032343

Td--TRIDC2AG080090.1	Tt--TRITD2Av1G291610.2	0.0566158	0.104705627478431	0.540714145124144	1287	296.75	990.25	54	29	0.055	0.098	8.05427904
Td--TRIDC2AG080090.1	Tt--TRITD2Bv1G261380.1	0.0677454	0.163713170069208	0.41380539240602	588	132.58333	455.41667	29.5	19.5	0.065	0.147	12.5933208
Td--TRIDC2BG088230.2	Tt--TRITD2Av1G291610.2	0.059962	0.161909614151363	0.370342612796858	1293	301	992	57.17	43.83	0.058	0.146	12.4545857
Td--TRIDC6AG000550.4	Tt--TRITD6Av1G000920.4	0.0038486	0.0137573460987946	0.279750856630974	2337	513.5	1823.5	7	7	0.004	0.014	1.05825739
Td--TRIDC3BG001050.1	Tt--TRITD3Bv1G001440.2	0	0	NaN	1560	385	1175	0	0	0	0	0
Td--TRIDC3AG004290.4	Tt--TRITD3Av1G008980.2	0.0417496	0.19105651781488	0.218519471248368	1737	417.5	1319.5	53.58	70.42	0.041	0.169	14.6966552
Td--TRIDC3AG004290.4	Tt--TRITD3Bv1G011530.1	0.0993272	0.329132910186198	0.301784428714638	2001	483.58333	1517.4167	141.2	128.8	0.093	0.266	25.3179162
Td--TRIDC3BG006020.1	Tt--TRITD3Av1G008980.2	0.042369	0.179891216186298	0.235525567137798	1722	413.16667	1308.8333	53.92	66.08	0.041	0.16	13.8377859
Td--TRIDC3BG006020.1	Tt--TRITD3Bv1G011530.1	0.0957248	0.325156319881328	0.294396100619572	1986	480.08333	1505.9167	135.3	126.7	0.09	0.264	25.0120246
Td--TRIDC3AG011590.6	Tt--TRITD3Av1G028450.2	0.0013806	0.00205198486415606	0.672802347368806	1938	488	1450	2	1	0.001	0.002	0.15784499
Td--TRIDC3BG015640.4	Tt--TRITD3Av1G028450.2	0.0111004	0.1028745229051	0.107902258986652	1941	488.91667	1452.0833	16	47	0.011	0.096	7.91342484

Td--TRIDC5BG047840.2	Tt--TRITD5Av1G170840.1	0.1234669	0.315380609777428	0.391485496445416	1884	421.41667	1462.5833	166.5	108.5	0.114	0.257	24.2600469
Td--TRIDC5BG047840.2	Tt--TRITD5Bv1G162020.4	0.0123813	0.0604922649238134	0.204676461677485	1896	430.16667	1465.8333	18	25	0.012	0.058	4.65325115
Td--TRIDC5AG055160.3	Tt--TRITD5Av1G199510.5	0	0.0076531276306813	0	1119	262.66667	856.33333	0	2	0	0.008	0.58870213
Td--TRIDC5AG055160.3	Tt--TRITD5Av1G202570.2	0.4379037	NaN	NaN	1065	249.25	815.75	270.6	194.4	0.332	0.78	High Sequence Divergence Value (pS>=0.75)
Td--TRIDC5AG055160.3	Tt--TRITD5Bv1G194390.4	0.0023403	0.123694083778229	0.0189202556965995	1119	263.08333	855.91667	2	30	0.002	0.114	9.51492952
Td--TRIDC5AG055160.3	Tt--TRITD5Bv1G198000.2	0.353001	1.7928682933406	0.196891739015922	1074	254.16667	819.83333	230.8	173.2	0.282	0.681	137.912946
Td--TRIDC5AG056200.2	Tt--TRITD5Av1G202570.2	0.1453941	0.238307937901449	0.610110041530824	1032	244.5	787.5	104.1	49.92	0.132	0.204	18.3313798
Td--TRIDC5AG056200.2	Tt--TRITD5Av1G199510.5	0.3518928	1.57557323336148	0.223342735258737	1056	250.16667	805.83333	226.3	164.7	0.281	0.658	121.197941
Td--TRIDC5AG056200.2	Tt--TRITD5Bv1G198000.2	0.0048241	0.0843697968021209	0.0571784858824653	1095	263.16667	831.83333	4	21	0.005	0.08	6.48998437
Td--TRIDC5AG056200.2	Tt--TRITD5Bv1G194390.4	0.3509878	1.64991377837595	0.212731023459429	1056	250.91667	805.08333	225.7	167.3	0.28	0.667	126.916444

Td--TRIDC5BG059170.3	Tt--TRITD5Av1G199510.5	0.0023408	0.123608948443107	0.0189369801974374	1119	263.25	855.75	2	30	0.002	0.114	9.50838065
Td--TRIDC5BG059170.3	Tt--TRITD5Bv1G194390.4	0	0.00762395383263804	0	1119	263.66667	855.33333	0	2	0	0.008	0.58645799
Td--TRIDC5AG049770.1	Tt--TRITD5Av1G187760.2	0	0.0024174074111765	0	1761	414.33333	1346.6667	0	1	0	0.002	0.18595442
Td--TRIDC5AG049770.1	Tt--TRITD5Bv1G177650.5	0.006	0.0878043629503894	0.0683340986563195	1749	410.33333	1338.6667	8	34	0.006	0.083	6.75418177
Td--TRIDC5BG053430.2	Tt--TRITD5Av1G187760.2	0.0065987	0.074437593881568	0.0886480588887442	1719	399.83333	1319.1667	8.667	28.33	0.007	0.071	5.72596876
Td--TRIDC5BG053430.2	Tt--TRITD5Bv1G177650.5	0.0007582	0.00502304096064115	0.150953574727698	1719	399.5	1319.5	1	2	0.001	0.005	0.38638777
Td--TRIDC5AG033270.1	Tt--TRITD5Bv1G125410.2	0.167217	0.225227150593817	0.742437287929162	1167	315.25	851.75	127.7	61.33	0.15	0.195	17.3251654
Td--TRIDC5AG033270.1	Tt--TRITD0Uv1G046260.2	0.147273	0.198073199569571	0.743528128034025	1155	311.16667	843.83333	112.8	54.17	0.134	0.174	15.2364
Td--TRIDC5BG035150.3	Tt--TRITD5Bv1G125410.2	0.1547054	0.208702678702075	0.741271683100083	1164	313.33333	850.66667	118.9	57.08	0.14	0.182	16.0540522
Td--TRIDC5BG035150.3	Tt--TRITD0Uv1G046260.2	0.1619175	0.245864643775762	0.65856342272815	1167	313.25	853.75	124.3	65.67	0.146	0.21	18.9126649
Td--TRIDC5AG051290.3	Tt--TRITD5Bv1G183720.1	0.1219701	0.674239178141445	0.180900353429972	510	116.16667	393.83333	44.33	51.67	0.113	0.445	51.8645522

Td--TRIDC5AG067100.1	Tt--TRITD5Av1G229390.2	0	0.00413508973058175	0	1032	242.5	789.5	0	1	0	0.004	0.31808383
Td--TRIDC5AG067100.1	Tt--TRITD5Bv1G228940.2	0.0063668	0.0870541284395653	0.0731356640725363	1032	243.33333	788.66667	5	20	0.006	0.082	6.69647142
Td--TRIDC5BG072290.4	Tt--TRITD5Av1G229390.2	0.0551004	0.140907844978917	0.391038298255491	1014	240.66667	773.33333	41.08	30.92	0.053	0.128	10.839065
Td--TRIDC5BG072290.4	Tt--TRITD5Bv1G228940.2	0.0337764	0.0792939168477378	0.425964515464878	999	237	762	25.17	17.83	0.033	0.075	6.09953207
Td--TRIDC3AG003160.1	Tt--TRITD3Av1G003600.2	0	0	NaN	777	187.83333	589.16667	0	0	0	0	0
Td--TRIDC3BG000190.2	Tt--TRITD3Av1G003600.2	0.0256962	0.15185405301196	0.169216236315174	1059	260.66667	798.33333	20.17	35.83	0.025	0.137	11.681081
Td--TRIDC3BG000190.2	Tt--TRITD0Uv1G022400.1	0.0095351	0.0128400311303118	0.742609152080375	1071	261.83333	809.16667	7.667	3.333	0.009	0.013	0.9876947
Td--TRIDC6AG021710.1	Tt--TRITD6Av1G059560.3	0.001039	0.00690851171492774	0.150388571081626	1254	290.83333	963.16667	1	2	0.001	0.007	0.53142398
Td--TRIDC6AG021710.1	Tt--TRITD6Bv1G068340.3	0.0031207	0.0913536554131526	0.0341603215575783	1254	290.66667	963.33333	3	25	0.003	0.086	7.02720426
Td--TRIDC6AG021710.1	Tt--TRITD7Av1G255570.2	0.0911213	0.730086859285578	0.124808846421006	1254	292.5	961.5	82.5	136.5	0.086	0.467	56.1605276
Td--TRIDC6AG021710.1	Tt--TRITD7Bv1G205360.3	0.0917426	0.82995924850139	0.110538631359594	1254	292.83333	961.16667	83	147	0.086	0.502	63.8430191

Td--TRIDC6BG027410.1	Tt--TRITD6Av1G059560.3	0.002079	0.0759685005383254	0.02736665059236	1254	290.66667	963.33333	2	21	0.002	0.072	5.84373081
Td--TRIDC6BG027410.1	Tt--TRITD6Bv1G068340.3	0	0.0138973287208706	0	1254	290.5	963.5	0	4	0	0.014	1.06902529
Td--TRIDC6BG027410.1	Tt--TRITD7Av1G255570.2	0.0893453	0.735336931205108	0.12150255993614	1254	292.33333	961.66667	81	137	0.084	0.469	56.5643793
Td--TRIDC6BG027410.1	Tt--TRITD7Bv1G205360.3	0.0899644	0.825669593525126	0.108959307579007	1254	292.66667	961.33333	81.5	146.5	0.085	0.501	63.5130457
Td--TRIDC7AG068050.1	Tt--TRITD6Av1G059560.3	0.0899478	0.739191675198536	0.121684026087941	1254	292.5	961.5	81.5	137.5	0.085	0.47	56.8608981
Td--TRIDC7AG068050.1	Tt--TRITD6Bv1G068340.3	0.0893453	0.744511357531834	0.120005314429586	1254	292.33333	961.66667	81	138	0.084	0.472	57.2701044
Td--TRIDC7AG068050.1	Tt--TRITD7Av1G255570.2	0	0.00667041639863754	0	1281	301.16667	979.83333	0	2	0	0.007	0.51310895
Td--TRIDC7AG068050.1	Tt--TRITD7Bv1G205360.3	0.0030817	0.163506964677769	0.018847322132892	1275	299.5	975.5	3	44	0.003	0.147	12.5774588
Td--TRIDC7BG061870.1	Tt--TRITD6Bv1G068340.3	0.089981	0.856231636998301	0.105089507948703	1254	292.83333	961.16667	81.5	149.5	0.085	0.511	65.8639721
Td--TRIDC7BG061870.1	Tt--TRITD7Av1G255570.2	0.0025676	0.157206976847221	0.016332712390595	1275	299.66667	975.33333	2.5	42.5	0.003	0.142	12.0928444
Td--TRIDC7BG061870.1	Tt--TRITD7Bv1G205360.3	0.0020541	0		1275	300	975	2	0	0.002	0	0

Td--TRIDC7AG045740.2	Tt--TRITD7Av1G173340.3	0.0008125	0.00266016683228101	0.305436773783536	1608	376.58333	1231.4167	1	1	0.001	0.003	0.20462822
Td--TRIDC7AG045740.2	Tt--TRITD7Bv1G130380.4	0.0190882	0.0470634079226066	0.405584042148974	1593	372.66667	1220.3333	23	17	0.019	0.046	3.62026215
Td--TRIDC7BG037680.2	Tt--TRITD7Av1G173340.3	0.018739	0.0455223847945452	0.411643577964265	1551	362.25	1188.75	22	16	0.019	0.044	3.50172191
Td--TRIDC7BG037680.2	Tt--TRITD7Bv1G130380.4	0	0.00276498009016413	0	1551	362.33333	1188.6667	0	1	0	0.003	0.21269078
Td--TRIDC1AG040730.2	Tt--TRITD1Av1G170240.2	0.0013263	0.00191938684411613	0.690981235295084	2031	521.66667	1509.3333	2	1	0.001	0.002	0.14764514
Td--TRIDC1AG040730.2	Tt--TRITD1Bv1G156400.1	0.0777881	0.162769016101706	0.477904584580205	2025	516	1509	111.5	75.5	0.074	0.146	12.5206935
Td--TRIDC1BG046350.4	Tt--TRITD1Av1G170240.2	0.0785745	0.16164589643209	0.486090217715244	2007	512.33333	1494.6667	111.5	74.5	0.075	0.145	12.4342997
Td--TRIDC1BG046350.4	Tt--TRITD1Bv1G156400.1	0	0.00195694827271029	0	2016	511.66667	1504.3333	0	1	0	0.002	0.15053448
Td--TRIDC3AG005110.2	Tt--TRITD3Av1G011010.3	0.001119	0.00663721145773687	0.168592739268112	1197	302.66667	894.33333	1	2	0.001	0.007	0.51055473
Td--TRIDC3AG005110.2	Tt--TRITD3Bv1G009870.4	0.0170996	0.119928508409559	0.14258185550982	1185	297.75	887.25	15	33	0.017	0.111	9.22526988
Td--TRIDC3BG005340.1	Tt--TRITD3Av1G011010.3	0.0148139	0.127385536926584	0.116291770564942	1185	298.75	886.25	13	35	0.015	0.117	9.79888746

Td--TRIDC3BG005340.1	Tt--TRITD3Bv1G009870.4	0.0136497	0.0521344743064754	0.261816504845455	1185	297.83333	887.16667	12	15	0.014	0.05	4.01034418
Td--TRIDC4AG067540.2	Tt--TRITD4Av1G255960.1	0.0330435	0.0841249275951196	0.392790723555208	1959	458.66667	1500.3333	48.5	36.5	0.032	0.08	6.47114828
Td--TRIDC4AG067540.2	Tt--TRITD7Av1G006960.1	0.033066	0.0790808194415211	0.418129493874075	1959	459.66667	1499.3333	48.5	34.5	0.032	0.075	6.08313996
Td--TRIDC3BG003740.4	Tt--TRITD3Av1G001260.2	0.0454819	0.124556559659091	0.36515085286017	1266	299.16667	966.83333	42.67	34.33	0.044	0.115	9.58127382
Td--TRIDC2AG028370.1	Tt--TRITD2Av1G083210.1	0.0006532	0.00195758675574277	0.333696075523793	2043	511.5	1531.5	1	1	0.001	0.002	0.1505836
Td--TRIDC4AG002010.1	Tt--TRITD4Bv1G170030.2	0.1516284	0.271007590476187	0.559498637007512	1080	285.41667	794.58333	109.1	64.92	0.137	0.227	20.8467377
Td--TRIDC4BG049660.1	Tt--TRITD4Av1G004390.1	0.0315283	0.10623788779727	0.296770630047815	795	212	583	18	21	0.031	0.099	8.17214522
Td--TRIDC4BG049660.1	Tt--TRITD4Bv1G170030.2	0.0006652	0.00374065613327928	0.177826694026476	2040	536	1504	1	2	0.001	0.004	0.28774278
Td--TRIDC3AG046380.3	Tt--TRITD3Av1G202040.8	0.0010527	0		2466	564.83333	1901.1667	2	0	0.001	0	0
Td--TRIDC3AG046380.3	Tt--TRITD3Bv1G178340.3	0.0497061	0.0673883262055209	0.73760654264633	1623	363.33333	1259.6667	60.58	23.42	0.048	0.064	5.1837174
Td--TRIDC3BG052620.2	Tt--TRITD3Av1G202040.8	0.0389654	0.049085600209437	0.793826287436215	1320	301.66667	1018.3333	38.67	14.33	0.038	0.048	3.7758154

Td--TRIDC3BG052620.2	Tt--TRITD3Bv1G178340.3	0.0350502	0.0370966312101704	0.944834650270738	1326	303.91667	1022.0833	35	11	0.034	0.036	2.85358702
Td--TRIDC5AG051310.6	Tt--TRITD5Bv1G183000.5	0.2351317	0.724758240067772	0.324427785588307	1803	445.5	1357.5	274	207	0.202	0.465	55.7506339
Td--TRIDC7AG018660.25	Tt--TRITD7Bv1G021510.1	0.1975801	0.381903658401193	0.517355829158243	180	45.666667	134.33333	23.33	13.67	0.174	0.299	29.3772045
Td--TRIDC6BG052000.1	Tt--TRITD6Av1G181680.1	0.4503656	0.669904210998038	0.672283602392527	381	95.75	285.25	96.58	42.42	0.339	0.443	51.5310932
Td--TRIDC6BG052000.1	Tt--TRITD6Bv1G171100.1	0.1320585	0.187122184471385	0.705734226482892	795	196.25	598.75	72.5	32.5	0.121	0.166	14.3940142
Td--TRIDC6AG027120.2	Tt--TRITD6Av1G085990.3	0.0011735	0.00383142595704184	0.306277511004383	2229	523.33333	1705.6667	2	2	0.001	0.004	0.29472507
Td--TRIDC6AG027120.2	Tt--TRITD6Bv1G090340.3	0.011827	0.0271742485110101	0.435227614815954	2229	524.58333	1704.4167	20	14	0.012	0.027	2.09032681
Td--TRIDC6BG033330.6	Tt--TRITD6Av1G085990.3	0.0139003	0.0419282461401825	0.331525773699893	1821	441.41667	1379.5833	19	18	0.014	0.041	3.2252497
Td--TRIDC6BG033330.6	Tt--TRITD6Bv1G090340.3	0.0007258	0.00681048947680986	0.106567559573051	1821	442.5	1378.5	1	3	0.001	0.007	0.52388381
Td--TRIDC1BG000450.3	Tt--TRITD1Av1G000530.1	0.0097931	0.282694718241685	0.0346418204517112	1077	254.75	822.25	8	60	0.01	0.236	21.7457476
Td--TRIDC3AG067170.2	Tt--TRITD3Av1G263280.2	0	0.0156527420441852	0	795	193.66667	601.33333	0	3	0	0.015	1.20405708

Td--TRIDC5AG013160.4	Tt--TRITD5Av1G045620.11	0.0197938	0.0276829253871922	0.715019975198079	2202	521.25	1680.75	32.83	14.17	0.02	0.027	2.1294558
Td--TRIDC5AG013160.4	Tt--TRITD5Bv1G043010.13	0.0381645	0.120502436525644	0.316711530575384	2247	531.5	1715.5	63.83	59.17	0.037	0.111	9.26941819
Td--TRIDC5BG015110.1	Tt--TRITD4Av1G134780.6	0.2192454	0.97357992212686	0.225195039032581	1923	440.5	1482.5	281.8	240.2	0.19	0.545	74.8907632
Td--TRIDC5BG015110.1	Tt--TRITD5Av1G045620.11	0.0294258	0.11412809800106	0.257831692528109	1890	434.5	1455.5	42	46	0.029	0.106	8.77908446
Td--TRIDC5BG015110.1	Tt--TRITD5Bv1G043010.13	0.0277551	0.0359738765094755	0.77153450662917	1893	434.16667	1458.8333	39.75	15.25	0.027	0.035	2.76722127
Td--TRIDC2AG028730.4	Tt--TRITD2Av1G084500.2	0.0013233	0.00205058227535112	0.645346038019313	2001	488.33333	1512.6667	2	1	0.001	0.002	0.1577371
Td--TRIDC2AG028730.4	Tt--TRITD2Bv1G089940.2	0.0369545	0.0992190295197042	0.372454155456731	2001	489.58333	1511.4167	54.5	45.5	0.036	0.093	7.63223304
Td--TRIDC2BG032450.3	Tt--TRITD2Av1G084500.2	0.0376499	0.101554116052796	0.370737409304238	2001	489.58333	1511.4167	55.5	46.5	0.037	0.095	7.81185508
Td--TRIDC2BG032450.3	Tt--TRITD2Bv1G089940.2	0.0019892	0		2001	490.83333	1510.1667	3	0	0.002	0	0
Td--TRIDC3AG002440.3	Tt--TRITD3Bv1G001440.2	0.0270498	0.158675556124908	0.170472108279048	2037	531.41667	1505.5833	40	76	0.027	0.143	12.205812
Td--TRIDC3AG012960.1	Tt--TRITD3Av1G032230.2	0.0023021	0		1731	425.83333	1305.1667	3	0	0.002	0	0

Td--TRIDC3AG012960.1	Tt--TRITD3Bv1G039450.3	0.0222819	0.0854218746334456	0.260845510697612	1731	425.25	1305.75	28.67	34.33	0.022	0.081	6.57091343
Td--TRIDC3BG017420.1	Tt--TRITD3Av1G032230.2	0.0199343	0.0852096429892616	0.233944158885565	1731	426.25	1304.75	25.67	34.33	0.02	0.081	6.55458792
Td--TRIDC3BG017420.1	Tt--TRITD3Bv1G039450.3	0.0015337	0		1731	425.66667	1305.3333	2	0	0.002	0	0
Td--TRIDC7AG049870.2	Tt--TRITD7Av1G192070.4	0.000429	0.00391858951726676	0.109490022899238	3099	767.58333	2331.4167	1	3	4E-04	0.004	0.30142996
Td--TRIDC7AG049870.2	Tt--TRITD7Bv1G152370.2	0.0497018	0.121284736946398	0.409794251879709	3084	762	2322	111.7	85.33	0.048	0.112	9.32959515
Td--TRIDC7BG043940.1	Tt--TRITD7Av1G192070.4	0.0485464	0.12520568586254	0.387732826915449	2898	714	2184	102.7	82.33	0.047	0.115	9.6312066
Td--TRIDC7BG043940.1	Tt--TRITD7Bv1G152370.2	0.0009139	0.0028057082054386	0.325717427557193	2904	714.16667	2189.8333	2	2	0.001	0.003	0.21582371
Td--TRIDC1AG027610.11	Tt--TRITD1Av1G118910.11	0	0	NaN	2067	473.5	1593.5	0	0	0	0	0
Td--TRIDC1AG027610.11	Tt--TRITD1Bv1G117690.3	0.0044044	0.0457658297442697	0.0962371948287374	2067	473	1594	7	21	0.004	0.044	3.52044844
Td--TRIDC1BG033320.4	Tt--TRITD1Av1G118910.11	0.0047452	0.0472039363097519	0.100526412173093	1917	437.16667	1479.8333	7	20	0.005	0.046	3.63107202
Td--TRIDC1BG033320.4	Tt--TRITD1Bv1G117690.3	0.0013523	0		1917	436.66667	1480.3333	2	0	0.001	0	0

Td--TRIDC2AG030300.1	Tt--TRITD2Av1G095350.5	0.0098801	0.0131983101377534	0.74858796567626		2076	496.83333	1579.1667	15.5	6.5	0.01	0.013	1.01525463
Td--TRIDC2AG030300.1	Tt--TRITD2Bv1G096060.3	0.004443	0.0435958062133858	0.101914549522575		2076	495.83333	1580.1667	7	21	0.004	0.042	3.35352355
Td--TRIDC2BG033980.3	Tt--TRITD2Av1G095350.5	0.0130938	0.0596965353346621	0.219339303481989		2076	496.66667	1579.3333	20.5	28.5	0.013	0.057	4.59204118
Td--TRIDC2BG033980.3	Tt--TRITD2Bv1G096060.3	0.000633	0.00202020324166721	0.313357077504967		2076	495.66667	1580.3333	1	1	0.001	0.002	0.15540025
Td--TRIDC5AG078780.1	Tt--TRITD4Bv1G207930.11	0.0280784	0.0983929473184089	0.285369651533873		2061	482.58333	1578.4167	43.5	44.5	0.028	0.092	7.56868826
Td--TRIDC5AG078780.1	Tt--TRITD5Av1G258690.15	0.0135437	0.0256755327798694	0.527492781220761		2040	475.41667	1564.5833	21	12	0.013	0.025	1.97504098
Td--TRIDC6AG053160.4	Tt--TRITD6Av1G209030.4	0	0	NaN		1806	410.16667	1395.8333	0	0	0	0	0
Td--TRIDC6AG053160.4	Tt--TRITD6Bv1G205270.3	0.0021636	0.0325254218363474	0.0665200615485585		1797	408.41667	1388.5833	3	13	0.002	0.032	2.50195553
Td--TRIDC6BG061710.3	Tt--TRITD6Av1G209030.4	0.002658	0.0391718894193417	0.0678536905882627		1953	445.41667	1507.5833	4	17	0.003	0.038	3.01322226
Td--TRIDC6BG061710.3	Tt--TRITD6Bv1G205270.3	0.0006636	0.00224887724719241	0.295067261119309		1953	445.33333	1507.6667	1	1	0.001	0.002	0.17299056
Td--TRIDC7AG029890.1	Tt--TRITD7Av1G084620.26	0.0118291	0.0198475846758864	0.595999400914857		2067	476.5	1590.5	18.67	9.333	0.012	0.02	1.52673728

Td--TRIDC7AG029890.1	Tt--TRITD7Bv1G059260.26	0.0175961	0.0804704712156252	0.218665489880116	2067	476.16667	1590.8333	27.67	36.33	0.017	0.076	6.19003625
Td--TRIDC7BG020630.1	Tt--TRITD7Av1G084620.26	0.0182438	0.082750841243805	0.220466452986428	2067	476.5	1590.5	28.67	37.33	0.018	0.078	6.36544933
Td--TRIDC7BG020630.1	Tt--TRITD7Bv1G059260.26	0.0124655	0.0220212338645908	0.566067945203166	2067	476.16667	1590.8333	19.67	10.33	0.012	0.022	1.69394107
												Average=27.6340178125438

The Ka/Ks ratio and divergence times between the genes of *T.turgidum* and *A.speltoides*												
**Gene ID**	**Gene ID**	**Ka**	**Ks**	**Ka/Ks**	**EffectiveLen**	**Average S-sites**	**Average N-sites**	**cN**	**cS**	**pN**	**pS**	**Divergence times (Mya)**
Tt--TRITD6Av1G170870.1	Aes-CH6S01G388700.1	0.105272	0.250568389028305	0.42013262960469	1140	270.333	869.667	85.4167	57.5833	0.098	0.21	19.2744915
Tt--TRITD6Bv1G157460.4	Aes-CH6S01G388700.1	0.009032	0.036058446260535	0.250489244720249	1245	298.25	946.75	8.5	10.5	0.009	0.04	2.77372664
Tt--TRITD4Av1G214900.3	Aes-CH5S01G635300.1	0.009957	0.0462701767540049	0.215192605160078	1323	312	1011	10	14	0.01	0.04	3.55924437
Tt--TRITD5Bv1G245660.3	Aes-CH5S01G635300.1	0.008967	0.0359545967168393	0.249404198620978	1323	313.333	1009.67	9	11	0.009	0.04	2.76573821
Tt--TRITD4Av1G049530.4	Aes-CH4S01G275300.1	0.025036	0.103533165597153	0.241817720611919	984	232.667	751.333	18.5	22.5	0.025	0.1	7.96408966
Tt--TRITD4Bv1G124550.2	Aes-CH4S01G275300.1	0	0.0236240002945282	0	1107	258	849	0	6	0	0.02	1.81723079
Tt--TRITD1Av1G032550.8	Aes-CH1S01G121300.1	0.004774	0.0762734268698046	0.0625853913690585	1647	386.083	1260.92	6	28	0.005	0.07	5.86718668
Tt--TRITD1Bv1G041580.2	Aes-CH1S01G121300.1	0.000867	0.0616936821058823	0.0140490721543162	1509	354.583	1154.42	1	21	0.001	0.06	4.74566785
Tt--TRITD1Av1G220750.2	Aes-CH1S01G422500.1	0.010845	0.0386403355785428	0.280661875092429	1818	424.833	1393.17	15	16	0.011	0.04	2.97233351
Tt--TRITD1Av1G220750.2	Aes-CH3S01G287600.1	0.116746	1.37796524082199	0.0847232693703563	1185	267.75	917.25	99.1667	168.833	0.108	0.63	105.997326

Tt--TRITD1Bv1G218290.1	Aes-CH1S01G422500.1	0.005792	0.0311920202169871	0.185696001956616	1812	425.5	1386.5	8	13	0.006	0.03	2.39938617
Tt--TRITD1Bv1G218290.1	Aes-CH3S01G287600.1	0.120198	1.33524278151607	0.0900193726488985	1185	268.083	916.917	101.833	167.167	0.111	0.62	102.710983
Tt--TRITD3Av1G155110.1	Aes-CH1S01G422500.1	0.131744	1.30052768422985	0.1013001229066	1440	328.167	1111.83	134.333	202.667	0.121	0.62	100.040591
Tt--TRITD3Av1G155110.1	Aes-CH3S01G287600.1	0.016172	0.0646583257422691	0.250109791081995	1212	274.417	937.583	15	17	0.016	0.06	4.97371736
Tt--TRITD3Bv1G135620.1	Aes-CH1S01G422500.1	0.122936	1.32163028928653	0.0930182171527697	1440	330.25	1109.75	125.833	205.167	0.113	0.62	101.663868
Tt--TRITD3Bv1G135620.1	Aes-CH3S01G287600.1	0.003213	0.029515028952746	0.108874360126327	1212	276.417	935.583	3	8	0.003	0.03	2.27038684
Tt--TRITD7Av1G032080.4	Aes-CH7S01G034800.1	0.004416	0.118550524752603	0.0372519852884539	1182	273.583	908.417	4	30	0.004	0.11	9.11927113
Tt--TRITD7Av1G032080.4	Aes-CH7S01G040500.1	0.014617	0.120863906464681	0.12093589077302	174	35.8333	138.167	2	4	0.014	0.11	9.29722357
Tt--TRITD7Bv1G002620.4	Aes-CH7S01G034800.1	0.003538	0.0615098637456262	0.057515042192818	1104	254	850	3	15	0.004	0.06	4.73152798
Tt--TRITD7Bv1G002620.4	Aes-CH7S01G040500.1	0.014617	0.0579999025523778	0.25201394394436	174	35.8333	138.167	2	2	0.014	0.06	4.46153097
Tt--TRITD7Av1G178880.7	Aes-CH7S01G479200.1	0	0.0526531940049364	0	435	98.3333	336.667	0	5	0	0.05	4.05024569

Tt--TRITD7Av1G178880.7	Aes-CH7S01G477100.1	0.001084	0.0378868426026762	0.0286066386918701		1194	270.667	923.333	1	10	0.001	0.04	2.91437251
Tt--TRITD7Bv1G140030.6	Aes-CH7S01G479100.1	0.012498	0.0444574574471552	0.281129130147292		675	150.583	524.417	6.5	6.5	0.012	0.04	3.41980442
Tt--TRITD7Bv1G140030.6	Aes-CH7S01G477100.1	0	0.0247518043527187	0		1263	287.5	975.5	0	7	0	0.02	1.90398495
Tt--TRITD1Av1G222580.3	Aes-CH1S01G431400.1	0.008873	0.0958357091220637	0.0925809593007284		1794	433.5	1360.5	12	39	0.009	0.09	7.37197762
Tt--TRITD1Av1G222580.3	Aes-CH3S01G281200.1	0.157947	1.03385847950683	0.152773865744122		1767	421.25	1345.75	191.667	236.333	0.142	0.56	79.5275753
Tt--TRITD1Bv1G220000.2	Aes-CH1S01G431400.1	0.002927	0.0892438790498591	0.0327978959523383		1809	439.75	1369.25	4	37	0.003	0.08	6.86491377
Tt--TRITD1Bv1G220000.2	Aes-CH3S01G281200.1	0.155374	1.13434289729373	0.136972255185271		1770	424.417	1345.58	188.833	248.167	0.14	0.58	87.2571459
Tt--TRITD3Av1G151000.4	Aes-CH1S01G431400.1	0.155093	1.02255020345943	0.15167283869453		1770	423.417	1346.58	188.667	236.333	0.14	0.56	78.657708
Tt--TRITD3Av1G151000.4	Aes-CH3S01G281200.1	0.000713	0.0572159775533474	0.0124602924012231		1839	435.667	1403.33	1	24	0.001	0.06	4.40122904
Tt--TRITD3Av1G151000.4	Aes-CH3S01G300500.1	0.195384	2.73473987977138	0.0714452200488085		1701	392.917	1308.08	225	287	0.172	0.73	210.364606
Tt--TRITD3Bv1G133770.2	Aes-CH1S01G431400.1	0.15655	1.00548754279242	0.15569596695384		1770	424.083	1345.92	190.167	234.833	0.141	0.55	77.3451956

Tt--TRITD3Bv1G133770.2	Aes-CH3S01G281200.1	0.003574	0.0161980842889023	0.220668935044077	1839	436.833	1402.17	5	7	0.004	0.02	1.24600648
Tt--TRITD3Bv1G133770.2	Aes-CH3S01G300300.1	0.132592	2.44057814362181	0.0543282886721166	1473	340.25	1132.75	137.667	245.333	0.122	0.72	187.73678
Tt--TRITD1Bv1G214990.2	Aes-CH1S01G408400.1	0.010244	0.0754167015730403	0.135828976729899	1668	390.25	1277.75	13	28	0.01	0.07	5.80128474
Tt--TRITD1Bv1G214990.2	Aes-CH3S01G300300.1	0.126641	1.1634683506924	0.108848219217405	1494	343.333	1150.67	134.083	202.917	0.117	0.59	89.4975654
Tt--TRITD3Av1G162410.11	Aes-CH1S01G408400.1	0.135474	1.25740218445295	0.107740977267222	1638	376.667	1261.33	156.333	229.667	0.124	0.61	96.723245
Tt--TRITD3Av1G162410.11	Aes-CH3S01G300300.1	0.007797	0.0358544744535693	0.217471914046156	1503	342.75	1160.25	9	12	0.008	0.04	2.7580365
Tt--TRITD3Bv1G140820.6	Aes-CH1S01G408400.1	0.135671	1.30910986782328	0.10363574291475	1650	378.25	1271.75	157.833	234.167	0.124	0.62	100.700759
Tt--TRITD3Bv1G140820.6	Aes-CH3S01G300300.1	0.008584	0.0450135072946896	0.190693531792783	1515	343.333	1171.67	10	15	0.009	0.04	3.46257748
Tt--TRITD1Av1G209190.3	Aes-CH3S01G300300.1	0.128141	1.05006576243917	0.122031403208924	1491	340.667	1150.33	135.5	192.5	0.118	0.57	80.7742894
Tt--TRITD6Av1G037430.3	Aes-CH6S01G223700.1	0.003205	0.114947230865338	0.0278835171487635	813	187.667	625.333	2	20	0.003	0.11	8.84209468
Tt--TRITD6Av1G037430.3	Aes-CH7S01G615700.1	0.046758	1.11343770157145	0.0419941943560718	1107	254	853	38.6667	147.333	0.045	0.58	85.649054

Tt--TRITD6Av1G037430.3	Aes-CH7S01G592100.1	0.04681	1.07084141346941	0.0437131245361176	1107	254.917	852.083	38.6667	145.333	0.045	0.57	82.3724164
Tt--TRITD6Bv1G050580.1	Aes-CH6S01G223700.1	0.0016	0.0440089568527774	0.0363465568784514	813	187.167	625.833	1	8	0.002	0.04	3.38530437
Tt--TRITD6Bv1G050580.1	Aes-CH7S01G615700.1	0.045484	1.02009166299726	0.0445878777141053	1107	253.5	853.5	37.6667	141.333	0.044	0.56	78.4685895
Tt--TRITD6Bv1G050580.1	Aes-CH7S01G592100.1	0.045534	0.997297920439844	0.0456575099732291	1107	254.417	852.583	37.6667	140.333	0.044	0.55	76.7152246
Tt--TRITD7Av1G227590.3	Aes-CH6S01G223700.1	0.051366	1.01719459446228	0.050497539500752	813	188.583	624.417	31	105	0.05	0.56	78.245738
Tt--TRITD7Av1G227590.3	Aes-CH7S01G615700.1	0.046856	0.235309888194804	0.199126310713873	1248	295.833	952.167	43.25	59.75	0.045	0.2	18.1007606
Tt--TRITD7Av1G227590.3	Aes-CH7S01G592100.1	0.04804	0.185187135365258	0.259414860232998	1248	297.083	950.917	44.25	48.75	0.047	0.16	14.2451643
Tt--TRITD6Av1G027670.1	Aes-CH6S01G197100.1	0.109455	0.180511336279558	0.606358526767644	1086	262.833	823.167	83.8333	42.1667	0.102	0.16	13.8854874
Tt--TRITD6Av1G027670.1	Aes-CH6S01G225500.1	0.029791	0.0548121425538575	0.543502191735965	1497	350	1147	33.5	18.5	0.029	0.05	4.21631866
Tt--TRITD6Bv1G041120.1	Aes-CH6S01G197100.1	0.113074	0.232361987661441	0.486629162493911	1101	268.167	832.833	87.4167	53.5833	0.105	0.2	17.8739991
Tt--TRITD6Bv1G041120.1	Aes-CH6S01G225500.1	0.019046	0.039511696280512	0.482030188890218	1494	350.75	1143.25	21.5	13.5	0.019	0.04	3.03936125

Tt--TRITD4Av1G251160.7	Aes-CH7S01G875500.1	0.011221	0.0531062273628451	0.211284965323988	1452	344.583	1107.42	12.3333	17.6667	0.011	0.05	4.08509441
Tt--TRITD7Av1G011740.1	Aes-CH7S01G875500.1	0.021414	0.11505765997465	0.186116987396476	1449	343.75	1105.25	23.3333	36.6667	0.021	0.11	8.85058923
Tt--TRITD7Av1G221430.1	Aes-CH7S01G583200.1	0.013574	0.115236495246923	0.117791436367129	1719	405.667	1313.33	17.6667	43.3333	0.013	0.11	8.86434579
Tt--TRITD7Av1G221430.1	Aes-CH7S01G560300.1	0.012542	0.110412056839335	0.113596464923078	1719	405.833	1313.17	16.3333	41.6667	0.012	0.1	8.49323514
Tt--TRITD7Bv1G170250.1	Aes-CH7S01G560300.1	0.017897	0.0731871521317476	0.244537983139636	1734	409.917	1324.08	23.4167	28.5833	0.018	0.07	5.62978093
Tt--TRITD7Bv1G170250.1	Aes-CH7S01G583200.1	0.015835	0.0669660604809241	0.236466746132782	1734	409.75	1324.25	20.75	26.25	0.016	0.06	5.15123542
Tt--TRITD6Bv1G202800.1	Aes-CH6S01G486800.1	0.15913	0.256252859532278	0.620987834911066	1059	287.167	771.833	110.667	62.3333	0.143	0.22	19.7117584
Tt--TRITD6Bv1G202800.1	Aes-CH6S01G486100.1	0.004331	0.107105405370392	0.0404391215116197	1116	305.583	810.417	3.5	30.5	0.004	0.1	8.23887734
Tt--TRITD6Bv1G202800.1	Aes-CH7S01G128000.1	0.109122	0.400651023322969	0.272360697175106	1020	274.917	745.083	75.6667	85.3333	0.102	0.31	30.8193095
Tt--TRITD7Av1G048910.1	Aes-CH7S01G128000.1	0.002633	0.0961622513292521	0.0273780649076557	1038	277	761	2	25	0.003	0.09	7.39709626
Tt--TRITD7Bv1G022360.1	Aes-CH6S01G486800.1	0.266712	0.502971237768445	0.530271943876969	1002	268.333	733.667	164.667	98.3333	0.224	0.37	38.6900952

Tt--TRITD7Bv1G022360.1	Aes-CH7S01G128000.1	0.001319	0.0409531005261388	0.0321998002948671		1035	276	759	1	11	0.001	0.04	3.1502385
Tt--TRITD4Av1G206810.2	Aes-CH5S01G665200.1	0.011695	0.187009442781175	0.0625343561241292		1569	362.5	1206.5	14	60	0.012	0.17	14.3853418
Tt--TRITD5Bv1G252270.3	Aes-CH5S01G665200.1	0.019564	0.036904322238023	0.530140549196177		1164	270.75	893.25	17.25	9.75	0.019	0.04	2.83879402
Tt--TRITD5Av1G091640.3	Aes-CH5S01G146600.1	0.020066	0.0696419467394346	0.288124314098006		873	208	665	13.1667	13.8333	0.02	0.07	5.35707283
Tt--TRITD5Bv1G075270.1	Aes-CH5S01G146600.1	0.018257	0.0326895821136056	0.558500104085056		1362	317.833	1044.17	18.8333	10.1667	0.018	0.03	2.51458324
Tt--TRITD4Av1G192120.1	Aes-CH4S01G066000.1	0.240159	0.538902310923573	0.445643859839499		987	269.25	717.75	147.5	103.5	0.206	0.38	41.4540239
Tt--TRITD4Bv1G014100.1	Aes-CH4S01G066000.1	0.020384	0.114252748719646	0.178411438827204		1029	283.083	745.917	15	30	0.02	0.11	8.78867298
Tt--TRITD2Bv1G201730.10	Aes-CH2S01G410800.1	0.005443	0.0319792646706658	0.170214134658332		2667	638.833	2028.17	11	20	0.005	0.03	2.45994344
Tt--TRITD2Bv1G201730.10	Aes-CH6S01G361000.1	0.158178	0.808067960217736	0.195748662274665		2616	630.417	1985.58	283.167	311.833	0.143	0.49	62.1590739
Tt--TRITD6Av1G156460.10	Aes-CH2S01G410800.1	0.154284	0.871375824822425	0.177057507565776		2616	628.417	1987.58	277.167	323.833	0.139	0.52	67.0289096
Tt--TRITD6Av1G156460.10	Aes-CH6S01G361000.1	0.007039	0.0453824621193559	0.15511177933772		2634	635.833	1998.17	14	28	0.007	0.04	3.49095862

Tt--TRITD6Bv1G142910.11	Aes-CH2S01G410800.1	0.154399	0.828538471502112	0.18635058692384	2616	629.75	1986.25	277.167	315.833	0.14	0.5	63.7337286
Tt--TRITD6Bv1G142910.11	Aes-CH6S01G361000.1	0.003011	0.014259737710894	0.211139416707664	2634	637.167	1996.83	6	9	0.003	0.01	1.0969029
Tt--TRITD4Av1G198600.3	Aes-CH4S01G045900.1	0.012248	0.141219140034339	0.0867332536499366	1707	431.167	1275.83	15.5	55.5	0.012	0.13	10.8630108
Tt--TRITD4Av1G042360.2	Aes-CH4S01G288500.1	0.062643	0.126900351623443	0.493638236498916	549	141.333	407.667	24.5	16.5	0.06	0.12	9.76156551
Tt--TRITD4Bv1G128900.2	Aes-CH4S01G288500.1	0.060058	0.109906222020403	0.546444172010317	549	141.833	407.167	23.5	14.5	0.058	0.1	8.45432477
Tt--TRITD2Av1G072210.1	Aes-CH2S01G206600.1	0.0362	0.122509241501212	0.295491537153017	1527	348	1179	41.6667	39.3333	0.035	0.11	9.42378781
Tt--TRITD2Bv1G081010.1	Aes-CH2S01G206600.1	0.003994	0.044079702765306	0.0906059987749453	1629	373.75	1255.25	5	16	0.004	0.04	3.39074637
Tt--TRITD3Av1G171650.1	Aes-CH3S01G320300.1	0.266838	0.638027132594916	0.418223555174867	1191	305.083	885.917	198.917	131.083	0.225	0.43	49.0790102
Tt--TRITD1Bv1G200860.1	Aes-CH1S01G358800.1	0.023772	0.0916866770674624	0.25927505800895	1575	399.75	1175.25	27.5	34.5	0.023	0.09	7.05282131
Tt--TRITD3Av1G171180.1	Aes-CH1S01G358800.1	0.304656	0.652118830121486	0.46717818717868	1134	291.917	842.083	210.833	127.167	0.25	0.44	50.1629869
Tt--TRITD3Av1G171180.1	Aes-CH3S01G320300.1	0.022482	0.125522378871155	0.179111140417861	1152	294.167	857.833	19	34	0.022	0.12	9.65556761

Tt--TRITD3Bv1G150780.1	Aes-CH1S01G358800.1	0.325805	0.647798321944976	0.502941885672488	1077	279.5	797.5	210.75	121.25	0.264	0.43	49.8306401
Tt--TRITD3Bv1G150780.1	Aes-CH3S01G320300.1	0.044364	0.139667222025121	0.317637244003039	1095	282.5	812.5	35	36	0.043	0.13	10.7436325
Tt--TRITD5Av1G228010.3	Aes-CH5S01G571400.1	0.001627	0.0854407908989227	0.0190438228057703	1602	371.5	1230.5	2	30	0.002	0.08	6.57236853
Tt--TRITD5Bv1G226960.4	Aes-CH5S01G571400.1	0.000813	0.0587302022696126	0.0138477934849731	1602	371.75	1230.25	1	21	0.001	0.06	4.51770787
Tt--TRITD5Av1G086470.1	Aes-CH5S01G135500.1	0.033137	0.070830453628411	0.467833323532586	1569	373.583	1195.42	38.75	25.25	0.032	0.07	5.44849643
Tt--TRITD5Bv1G121250.4	Aes-CH5S01G228100.1	0.00192	0.0388366462279456	0.0494405063438468	2040	475.583	1564.42	3	18	0.002	0.04	2.98743433
Tt--TRITD0Uv1G042030.1	Aes-CH5S01G228100.1	0.02851	0.0740121482122232	0.38521164647193	1629	363	1266	35.4167	25.5833	0.028	0.07	5.69324217
Tt--TRITD2Av1G270510.6	Aes-CH2S01G510600.1	0.00827	0.0746742164637182	0.110754028654497	3954	914.5	3039.5	25	65	0.008	0.07	5.7441705
Tt--TRITD2Bv1G233690.10	Aes-CH2S01G510600.1	0.001976	0.0277783810997533	0.0711388830397603	3957	916.75	3040.25	6	25	0.002	0.03	2.13679855
Tt--TRITD6Av1G054950.1	Aes-CH6S01G239300.1	0.01196	0.109961698922761	0.108761430418351	1626	361.75	1264.25	15	37	0.012	0.1	8.45859222
Tt--TRITD6Bv1G065890.1	Aes-CH6S01G239300.1	0.006361	0.0167176645225422	0.380471962132943	1626	362.917	1263.08	8	6	0.006	0.02	1.28597419

Tt--TRITD5Av1G205000.3	Aes-CH5S01G459300.1	0.006821	0.0548102751379005	0.1244440469502	2316	548.667	1767.33	12	29	0.007	0.05	4.21617501
Tt--TRITD5Bv1G200940.1	Aes-CH5S01G459300.1	0.00213	0.0289891158257094	0.0734928971921728	2478	597.833	1880.17	4	17	0.002	0.03	2.22993199
Tt--TRITD5Bv1G119650.1	Aes-CH5S01G223800.1	0.00719	0.0482869864807181	0.148901177759489	1299	320.75	978.25	7	15	0.007	0.05	3.71438358
Tt--TRITD0Uv1G047610.1	Aes-CH5S01G223800.1	0.013374	0.0725242061553695	0.184403168413933	1299	318.25	980.75	13	22	0.013	0.07	5.57878509
Tt--TRITD5Av1G112160.1	Aes-CH5S01G171800.1	0.007828	0.0641866136241277	0.121954707796117	1884	471.417	1412.58	11	29	0.008	0.06	4.93743182
Tt--TRITD5Bv1G092620.2	Aes-CH5S01G171800.1	0.003524	0.0367773749447345	0.0958093029027097	1896	473.667	1422.33	5	17	0.004	0.04	2.82902884
Tt--TRITD6Av1G162640.1	Aes-CH6S01G373100.1	0.059463	0.0946611667118609	0.628168446467165	1536	352.333	1183.67	67.6667	31.3333	0.057	0.09	7.28162821
Tt--TRITD6Bv1G148270.1	Aes-CH6S01G373100.1	0.023727	0.0510208930518217	0.465044724753242	1536	351.417	1184.58	27.6667	17.3333	0.023	0.05	3.92468408
Tt--TRITD2Av1G069790.2	Aes-CH2S01G202900.1	0.0064	0.102940134689408	0.0621724350359807	2037	467.833	1569.17	10	45	0.006	0.1	7.9184719
Tt--TRITD2Bv1G079430.3	Aes-CH2S01G202900.1	0.005101	0.0714959540977767	0.0713425693667414	2043	469.25	1573.75	8	32	0.005	0.07	5.49968878
Tt--TRITD2Av1G066770.1	Aes-CH2S01G196500.1	0.079502	0.181517481848183	0.437983440544963	966	234.667	731.333	55.1667	37.8333	0.075	0.16	13.9628832

Tt--TRITD2Bv1G076660.1	Aes-CH2S01G196500.1	0.001369	0.0216153289016188	0.0633168028439896	966	234.667	731.333	1	5	0.001	0.02	1.66271761
Tt--TRITD0Uv1G000510.7	Aes-CH1S01G030400.1	0.016327	0.0961742563439188	0.16976658836637	1761	398.833	1362.17	22	36	0.016	0.09	7.39801972
Tt--TRITD0Uv1G004130.5	Aes-CH1S01G030400.1	0.010736	0.0774966998216712	0.138530061495741	1761	400.667	1360.33	14.5	29.5	0.011	0.07	5.9612846
Tt--TRITD6Av1G073760.2	Aes-CH6S01G267500.1	0.011514	0.0495755651565165	0.232258187977808	1167	291.833	875.167	10	14	0.011	0.05	3.81350501
Tt--TRITD6Av1G073760.2	Aes-CH7S01G572000.1	0.207406	0.567874847945159	0.365231085699519	1098	267.417	830.583	150.5	106.5	0.181	0.4	43.6826806
Tt--TRITD6Av1G073760.2	Aes-CH7S01G534900.1	0.207334	0.593029256307023	0.34961792639015	1098	267.167	830.833	150.5	109.5	0.181	0.41	45.6176351
Tt--TRITD7Av1G209280.1	Aes-CH6S01G267500.1	0.206134	0.591968280834634	0.348217465262988	1098	266.667	831.333	149.833	109.167	0.18	0.41	45.5360216
Tt--TRITD7Av1G209280.1	Aes-CH7S01G534900.1	0	0.0543059821634854	0	1188	286.333	901.667	0	15	0	0.05	4.17738324
Tt--TRITD7Av1G209280.1	Aes-CH7S01G572000.1	0	0.054256852862061	0	1188	286.583	901.417	0	15	0	0.05	4.17360407
Tt--TRITD7Bv1G162110.1	Aes-CH6S01G267500.1	0.206229	0.566539253690104	0.364015138196783	1098	267	831	149.833	106.167	0.18	0.4	43.5799426
Tt--TRITD7Bv1G162110.1	Aes-CH7S01G534900.1	0.00111	0.0393885257125847	0.028188129861152	1188	286.667	901.333	1	11	0.001	0.04	3.02988659

Tt--TRITD7Bv1G162110.1	Aes-CH7S01G572000.1	0.001111	0.0541914852970241	0.020493941856555	1188	286.917	901.083	1	15	0.001	0.05	4.16857579
Tt--TRITD6Bv1G077930.1	Aes-CH6S01G267500.1	0.003413	0.0033975143038125	1.0045506413814	1176	295	881	3	1	0.003	0	0.26134725
Tt--TRITD6Bv1G077930.1	Aes-CH7S01G572000.1	0.204812	0.572673557981767	0.357641109038273	1098	267.583	830.417	148.833	107.167	0.179	0.4	44.0518122
Tt--TRITD6Bv1G077930.1	Aes-CH7S01G534900.1	0.204741	0.597980785901273	0.342386805593968	1098	267.333	830.667	148.833	110.167	0.179	0.41	45.998522
Tt--TRITD1Av1G197990.2	Aes-CH1S01G324900.1	0.010316	0.125211941650912	0.082391539189843	1800	433.583	1366.42	14	50	0.01	0.12	9.63168782
Tt--TRITD1Av1G197990.2	Aes-CH3S01G359100.1	0.274845	1.2543756472696	0.219109387999411	1293	307.25	985.75	226.833	187.167	0.23	0.61	96.4904344
Tt--TRITD1Bv1G187410.2	Aes-CH1S01G324900.1	0.005151	0.109876198997481	0.0468845690315337	1794	430.5	1363.5	7	44	0.005	0.1	8.45201531
Tt--TRITD1Bv1G187410.2	Aes-CH3S01G359100.1	0.272404	1.32648478026529	0.205357453951548	1293	306.5	986.5	225.333	190.667	0.228	0.62	102.037291
Tt--TRITD3Av1G181840.2	Aes-CH1S01G324900.1	0.321732	1.34918169196563	0.238464178165405	1731	409.417	1321.58	345.75	256.25	0.262	0.63	103.783207
Tt--TRITD3Av1G181840.2	Aes-CH3S01G359100.1	0.015966	0.0716861350579924	0.222721049163425	1320	307.167	1012.83	16	21	0.016	0.07	5.51431808
Tt--TRITD3Bv1G162740.2	Aes-CH1S01G324900.1	0.303096	1.29630321663795	0.233815614882939	1713	405.167	1307.83	326.083	249.917	0.249	0.62	99.715632

Tt--TRITD3Bv1G162740.2	Aes-CH3S01G359100.1	0.003951	0.0164210172052585	0.240583538110935	1323	307.833	1015.17	4	5	0.004	0.02	1.26315517
Tt--TRITD6Av1G173820.1	Aes-CH6S01G396400.1	0.053081	0.0845966206090083	0.627465016964092	324	83.3333	240.667	12.3333	6.66667	0.051	0.08	6.50743235
Tt--TRITD6Av1G173820.1	Aes-CH7S01G293100.1	0.221359	0.448766688942792	0.493260373832198	1356	336.083	1019.92	195.5	113.5	0.192	0.34	34.5205145
Tt--TRITD6Av1G173820.1	Aes-CH7S01G292300.1	0.234012	0.489995908361605	0.4775785084182	1386	342.583	1043.42	209.75	123.25	0.201	0.36	37.691993
Tt--TRITD6Bv1G161420.1	Aes-CH6S01G396400.1	0.053081	0.0981512064037792	0.540812710616793	324	83.3333	240.667	12.3333	7.66667	0.051	0.09	7.5500928
Tt--TRITD6Bv1G161420.1	Aes-CH7S01G293100.1	0.169421	0.421975101311121	0.4014962583378	1254	308.833	945.167	143.333	99.6667	0.152	0.32	32.4596232
Tt--TRITD6Bv1G161420.1	Aes-CH7S01G292300.1	0.169388	0.463200269427842	0.365690444618567	1254	308.667	945.333	143.333	106.667	0.152	0.35	35.63079
Tt--TRITD7Av1G083120.2	Aes-CH6S01G396400.1	0.168767	0.37502085796009	0.450020656336791	324	83.5833	240.417	36.3333	24.6667	0.151	0.3	28.8477583
Tt--TRITD7Av1G083120.2	Aes-CH7S01G293100.1	0.033803	0.0867283306824468	0.389758427116124	1413	354.083	1058.92	35	29	0.033	0.08	6.67141005
Tt--TRITD7Av1G083120.2	Aes-CH7S01G292300.1	0.032124	0.0699041085036541	0.45954824254818	1416	353.333	1062.67	33.4167	23.5833	0.031	0.07	5.37723912
Tt--TRITD1Av1G203640.1	Aes-CH1S01G345800.1	0.052485	0.158936909681011	0.33022642471187	2331	581.833	1749.17	88.6667	83.3333	0.051	0.14	12.2259161

Tt--TRITD3Av1G164820.1	Aes-CH1S01G345800.1	0.119795	0.336132169650911	0.356391718216885	1605	382.667	1222.33	135.333	103.667	0.111	0.27	25.8563207
Tt--TRITD3Av1G164820.1	Aes-CH3S01G303500.1	0.030853	0.172646032765129	0.178706943220297	1578	381.5	1196.5	36.1667	58.8333	0.03	0.15	13.2804641
Tt--TRITD3Bv1G142220.1	Aes-CH3S01G303200.1	0.010199	0.0500804044045429	0.203647747629072	2433	557.333	1875.67	19	27	0.01	0.05	3.8523388
Tt--TRITD1Bv1G199190.1	Aes-CH1S01G345800.1	0.122627	0.302985638665569	0.404729441850311	2385	589.083	1795.92	203.167	146.833	0.113	0.25	23.3065876
Tt--TRITD3Av1G164850.1	Aes-CH3S01G303200.1	0.250197	1.56164432419734	0.160213528491737	2337	535.917	1801.08	383.167	351.833	0.213	0.66	120.126486
Tt--TRITD1Bv1G218630.1	Aes-CH1S01G423700.1	0.003988	0.116806960772631	0.0341421886387693	660	157.167	502.833	2	17	0.004	0.11	8.98515083
Tt--TRITD1Bv1G218630.1	Aes-CH3S01G286400.1	0.064815	0.676989250164609	0.095739801509245	654	154.75	499.25	31	69	0.062	0.45	52.0760962
Tt--TRITD3Av1G155690.2	Aes-CH1S01G423700.1	0.067549	0.789155341376171	0.0855969547681668	699	164.917	534.083	34.5	80.5	0.065	0.49	60.704257
Tt--TRITD3Av1G155690.2	Aes-CH3S01G286400.1	0.023523	0.10274396690584	0.228951969644575	990	234.333	755.667	17.5	22.5	0.023	0.1	7.90338207
Tt--TRITD3Bv1G135290.1	Aes-CH1S01G423700.1	0.067549	0.789155341376171	0.0855969547681668	699	164.917	534.083	34.5	80.5	0.065	0.49	60.704257
Tt--TRITD3Bv1G135290.1	Aes-CH3S01G286400.1	0	0.0303948974249952	0	984	235	749	0	7	0	0.03	2.33806903

Tt--TRITD2Av1G084730.2	Aes-CH2S01G225300.1	0.437188	3.00372926185952	0.145548428193269		1908	446.583	1461.42	484.167	328.833	0.331	0.74	231.056097
Tt--TRITD2Bv1G090370.1	Aes-CH2S01G225200.1	0.01405	0.0795852977293504	0.176545642529286		2097	516.5	1580.5	22	39	0.014	0.08	6.12194598
Tt--TRITD7Av1G004690.5	Aes-CH7S01G846700.1	0.077608	0.196655836641592	0.39463647419282		1005	230.75	774.25	57.0833	39.9167	0.074	0.17	15.127372
Tt--TRITD7Av1G004690.5	Aes-CH7S01G847900.1	0.114489	0.356430553297247	0.321211134083509		981	224.417	756.583	80.3333	63.6667	0.106	0.28	27.4177349
Tt--TRITD2Av1G291610.2	Aes-CH2S01G633500.1	0.058331	0.159801748239495	0.365020782994529		1293	301.083	991.917	55.6667	43.3333	0.056	0.14	12.2924422
Tt--TRITD6Av1G000920.4	Aes-CH6S01G007900.1	0.099679	0.181289952665359	0.549832888941937		489	108.667	380.333	35.5	17.5	0.093	0.16	13.945381
Tt--TRITD3Bv1G011530.1	Aes-CH3S01G029900.1	0.077347	0.369654123214705	0.209241298364992		1242	287.25	954.75	70.1667	83.8333	0.073	0.29	28.4349326
Tt--TRITD3Av1G028450.2	Aes-CH3S01G103500.1	0.013197	0.107660269622227	0.122580744872265		1941	488.583	1452.42	19	49	0.013	0.1	8.2815592
Tt--TRITD5Av1G170840.1	Aes-CH5S01G330400.1	0.04464	0.0888767065112392	0.502268851571693		1308	304.25	1003.75	43.5	25.5	0.043	0.08	6.83666973
Tt--TRITD5Av1G199510.5	Aes-CH5S01G438200.1	0.002342	0.110139826373957	0.0212610992027364		1119	263.583	855.417	2	27	0.002	0.1	8.47229434
Tt--TRITD5Av1G202570.2	Aes-CH5S01G446700.1	0.14269	0.292256479781646	0.488235034561334		1053	251.333	801.667	104.167	60.8333	0.13	0.24	22.4812677

Tt--TRITD5Bv1G194390.4	Aes-CH5S01G438200.1	0.002343	0.0969087986100044	0.0241756865100649	1119	264	855	2	24	0.002	0.09	7.45452297
Tt--TRITD5Bv1G198000.2	Aes-CH5S01G446700.1	0.003468	0.0570979485169519	0.0607297852177565	1140	272.833	867.167	3	15	0.003	0.05	4.39214989
Tt--TRITD5Av1G187760.2	Aes-CH5S01G383000.1	0.005869	0.122268780825224	0.0480014342340689	1785	416.583	1368.42	8	47	0.006	0.11	9.40529083
Tt--TRITD5Bv1G177650.5	Aes-CH5S01G383000.1	0.002185	0.0779105392690779	0.0280432572423906	1794	418.917	1375.08	3	31	0.002	0.07	5.99311841
Tt--TRITD5Bv1G125410.2	Aes-CH5S01G234600.1	0.165494	0.213148885020312	0.776423733139837	1167	315.75	851.25	126.417	58.5833	0.149	0.19	16.3960681
Tt--TRITD0Uv1G046260.2	Aes-CH5S01G234600.1	0.162903	0.237969956799587	0.684553246209904	1155	311	844	123.583	63.4167	0.146	0.2	18.3053813
Tt--TRITD5Av1G229390.2	Aes-CH5S01G579200.1	0.004247	0.0704329495874262	0.0602991539710432	1242	297.5	944.5	4	20	0.004	0.07	5.4179192
Tt--TRITD5Bv1G228940.2	Aes-CH5S01G579200.1	0.004243	0.058989929712129	0.0719324944206817	1245	299.667	945.333	4	17	0.004	0.06	4.5376869
Tt--TRITD6Av1G059560.3	Aes-CH6S01G246400.1	0.002079	0.0875309117576116	0.0237475337883332	1254	290.5	963.5	2	24	0.002	0.08	6.73314706
Tt--TRITD6Bv1G068340.3	Aes-CH6S01G246400.1	0	0.0316576890012354	0	1254	290.333	963.667	0	9	0	0.03	2.43520685
Tt--TRITD7Av1G255570.2	Aes-CH6S01G246400.1	0.089329	0.726972633199035	0.122877910390838	1254	292.167	961.833	81	136	0.084	0.47	55.9209718

Tt--TRITD7Bv1G205360.3	Aes-CH6S01G246400.1	0.089948	0.85805599705865	0.104827446457087	1254	292.5	961.5	81.5	149.5	0.085	0.51	66.0043075
Tt--TRITD7Av1G173340.3	Aes-CH7S01G417300.1	0.016078	0.0314755425123037	0.510795489868722	1599	373.083	1225.92	19.5	11.5	0.016	0.03	2.42119558
Tt--TRITD7Bv1G130380.4	Aes-CH7S01G417300.1	0.00782	0.0232153263596053	0.336849608321048	1593	371.833	1221.17	9.5	8.5	0.008	0.02	1.78579434
Tt--TRITD1Av1G170240.2	Aes-CH1S01G249300.1	0.079011	0.164270367467962	0.480982484841285	2022	515.167	1506.83	113	76	0.075	0.15	12.6361821
Tt--TRITD1Bv1G156400.1	Aes-CH1S01G249300.1	0.066631	0.136692609763071	0.487454073676192	2031	514.833	1516.17	96.6667	64.3333	0.064	0.12	10.5148161
Tt--TRITD3Av1G011010.3	Aes-CH3S01G024500.1	0.020725	0.112336048214884	0.184494265450723	1155	290.75	864.25	17.6667	30.3333	0.02	0.1	8.64123448
Tt--TRITD3Bv1G009870.4	Aes-CH3S01G024500.1	0.022885	0.117412175920595	0.194908431144324	1155	289.833	865.167	19.5	31.5	0.023	0.11	9.03170584
Tt--TRITD7Av1G006960.1	Aes-CH7S01G856400.1	0.015368	0.0511688378479657	0.300339457224188	1977	465	1512	23	23	0.015	0.05	3.93606445
Tt--TRITD3Av1G001260.2	Aes-CH3S01G011500.1	0.048228	0.130521723808777	0.369503022911355	2301	554.417	1746.58	81.5833	66.4167	0.047	0.12	10.0401326
Tt--TRITD2Av1G083210.1	Aes-CH2S01G221400.1	0.224394	1.89681134612709	0.118300459412427	552	136.917	415.083	80.5	94.5	0.194	0.69	145.908565
Tt--TRITD4Bv1G170030.2	Aes-CH4S01G415500.1	0.013459	0.0746811837582654	0.180213488120449	2034	534.583	1499.42	20	38	0.013	0.07	5.74470644

Tt--TRITD4Bv1G170030.2	Aes-CH4S01G404900.1	0.015802	0.0919972787562797	0.171761161384436	2040	537.083	1502.92	23.5	46.5	0.016	0.09	7.07671375
Tt--TRITD3Av1G202040.8	Aes-CH3S01G393100.1	0.017683	0.0926017809171765	0.190962140179784	2424	554.833	1869.17	32.6667	48.3333	0.017	0.09	7.12321392
Tt--TRITD3Bv1G178340.3	Aes-CH3S01G393100.1	0.052979	0.106234123237836	0.498699954773756	1617	360.917	1256.08	64.25	35.75	0.051	0.1	8.17185563
Tt--TRITD6Av1G085990.3	Aes-CH6S01G288200.1	0.012091	0.0473776098543071	0.25521192018948	2580	620.75	1959.25	23.5	28.5	0.012	0.05	3.64443153
Tt--TRITD6Bv1G090340.3	Aes-CH6S01G288200.1	0.008732	0.032872969220032	0.265635503836382	2580	621.833	1958.17	17	20	0.009	0.03	2.52868994
Tt--TRITD1Av1G000530.1	Aes-CH1S01G005100.1	0.011445	0.179100270476067	0.0639013751181213	1077	255.25	821.75	9.33333	40.6667	0.011	0.16	13.7769439
Tt--TRITD5Av1G045620.11	Aes-CH5S01G108100.1	0.025412	0.109302673120492	0.232492578738636	2202	521.083	1680.92	42	53	0.025	0.1	8.40789793
Tt--TRITD5Bv1G043010.13	Aes-CH5S01G108100.1	0.026749	0.0868843829832222	0.307873770895023	2205	521.083	1683.92	44.25	42.75	0.026	0.08	6.68341408
Tt--TRITD4Av1G134780.6	Aes-CH4S01G202500.1	0.01012	0.0379196153814435	0.266885754058882	2205	513.833	1691.17	17	19	0.01	0.04	2.91689349
Tt--TRITD4Av1G134780.6	Aes-CH5S01G108100.1	0.244969	0.968594800616026	0.252911662025259	2163	504.583	1658.42	346.583	274.417	0.209	0.54	74.5072924
Tt--TRITD2Av1G084500.2	Aes-CH2S01G224000.1	0.034953	0.106149161901035	0.329283519483	1998	490	1508	51.5	48.5	0.034	0.1	8.16532015

Tt--TRITD2Bv1G089940.2	Aes-CH2S01G224000.1	0.010022	0.0206375875560002	0.485612129089382	1998	491.25	1506.75	15	10	0.01	0.02	1.58750674
Tt--TRITD3Av1G032230.2	Aes-CH3S01G115500.1	0.017055	0.0948894656543	0.179735425310561	1731	426.333	1304.67	22	38	0.017	0.09	7.29918967
Tt--TRITD3Bv1G039450.3	Aes-CH3S01G115500.1	0.00925	0.0688302547185429	0.134395180687933	1731	425.75	1305.25	12	28	0.009	0.07	5.29463498
Tt--TRITD7Av1G192070.4	Aes-CH7S01G509000.1	0.046324	0.105046765345887	0.440987838379926	3066	758.333	2307.67	103.667	74.3333	0.045	0.1	8.08052041
Tt--TRITD7Av1G192070.4	Aes-CH7S01G458800.1	0.039171	0.109192605714890	0.358733572120284	2649	644.583	2004.42	76.5	65.5	0.038	0.1	8.39943121
Tt--TRITD7Bv1G152370.2	Aes-CH7S01G509000.1	0.012666	0.0491252062355354	0.257838329358305	3066	757.083	2308.92	29	36	0.013	0.05	3.77886202
Tt--TRITD7Bv1G152370.2	Aes-CH7S01G458800.1	0.009518	0.0446346831567349	0.213250557218117	2655	646.167	2008.83	19	28	0.009	0.04	3.43343717
Tt--TRITD2Av1G095350.5	Aes-CH2S01G234400.1	0.094022	0.182411273964383	0.515441047626232	558	131.75	426.25	37.6667	21.3333	0.088	0.16	14.0316365
Tt--TRITD2Bv1G096060.3	Aes-CH2S01G234400.1	0.094022	0.14465756334155	0.649964343233419	558	131.75	426.25	37.6667	17.3333	0.088	0.13	11.1275049
Tt--TRITD4Bv1G207930.11	Aes-CH4S01G568500.1	0.007702	0.0683432012778051	0.112699578510867	2217	520.5	1696.5	13	34	0.008	0.07	5.25716933
Tt--TRITD5Av1G258690.15	Aes-CH4S01G568500.1	0.009345	0.0455528752698572	0.205156001408206	2178	509.083	1668.92	15.5	22.5	0.009	0.04	3.50406733

Tt--TRITD6Av1G209030.4	Aes-CH6S01G495000.1	0.003211	0.0420541646048345	0.0763571660392743	2025	464.583	1560.42	5	19	0.003	0.04	3.23493574
Tt--TRITD6Bv1G205270.3	Aes-CH6S01G495000.1	0.001283	0.0240633108479577	0.0533067467454556	2025	464.5	1560.5	2	11	0.001	0.02	1.85102391
Tt--TRITD7Av1G084620.26	Aes-CH7S01G296700.1	0.02818	0.0986309890251347	0.285709848995147	2067	476.083	1590.92	44	44	0.028	0.09	7.58699916
Tt--TRITD7Av1G084620.26	Aes-CH7S01G268600.1	0.03355	0.0943993052449784	0.355401616577939	2121	490.417	1630.58	53.5	43.5	0.033	0.09	7.26148502
Tt--TRITD7Bv1G059260.26	Aes-CH7S01G296700.1	0.022324	0.0868116356111995	0.257157498518642	2067	475.75	1591.25	35	39	0.022	0.08	6.67781812
Tt--TRITD7Bv1G059260.26	Aes-CH7S01G268600.1	0.029068	0.0829842524189104	0.350279129930066	2121	490.083	1630.92	46.5	38.5	0.029	0.08	6.38340403
												Average=24.4088924598461

The Ka/Ks ratio and divergence times between the genes of *A.speltoides* and *T.urartu*													
**Gene ID**	**Gene ID**	**Ka**	**Ks**	**Ka/Ks**	**EffectiveLen**	**AverageS-sites**	**AverageN-sites**	**cN**	**cS**	**pN**	**pS**	**Divergence times (Mya)**
Aes-CH4S01G275300.1	Tu--TuG1812G0400002351.01.T01	0.0011783	0.0819367870481388	0.0143808320594586	1107	257.66667	849.33333	1	20	0.001	0.078	6.30282977
Aes-CH1S01G121300.1	Tu--TuG1812G0100001066.01.T01	0.0039759	0.0762734268698046	0.0521267909096672	1647	386.08333	1260.9167	5	28	0.004	0.073	5.86718668
Aes-CH1S01G422500.1	Tu--TuG1812G0100004520.01.T02	0.0169465	0.0452053417446256	0.374877251480609	1818	425.5	1392.5	23.33	18.67	0.017	0.044	3.47733398
Aes-CH1S01G422500.1	Tu--TuG1812G0300002745.01.T01	0.1306509	1.30230703060867	0.100322621026937	1440	328	1112	133.3	202.7	0.12	0.618	100.177464
Aes-CH3S01G287600.1	Tu--TuG1812G0300002745.01.T01	0.0150799	0.0646993644273757	0.233077163374837	1212	274.25	937.75	14	17	0.015	0.062	4.97687419
Aes-CH7S01G477100.1	Tu--TuG1812G0700003597.01.T01	0.0041544	0.0469750134988024	0.0884388468198459	1251	285.5	965.5	4	13	0.004	0.046	3.61346258
Aes-CH1S01G431400.1	Tu--TuG1812G0100004629.01.T01	0.0081272	0.0985425040468546	0.0824736244558751	1794	433.16667	1360.8333	11	40	0.008	0.092	7.58019262

Aes-CH3S01G281200.1	Tu--TuG1812G0100004629.01.T01	0.1574447	1.0309130593167	0.152723517071063		1767	420.91667	1346.0833	191.2	235.8	0.142	0.56	79.3010046
Aes-CH3S01G281200.1	Tu--TuG1812G0300002551.01.T01	0.0108758	0.0619691682854432	0.17550369512793		1839	434.33333	1404.6667	15.17	25.83	0.011	0.059	4.7668591
Aes-CH7S01G615700.1	Tu--TuG1812G0700004566.01.T01	0.0468564	0.230698036614541	0.203107016419973		1248	295.83333	952.16667	43.25	58.75	0.045	0.199	17.7460028
Aes-CH7S01G592100.1	Tu--TuG1812G0700004566.01.T01	0.0480403	0.180890668191215	0.265576413189793		1248	297.08333	950.91667	44.25	47.75	0.047	0.161	13.9146668
Aes-CH6S01G225500.1	Tu--TuG1812G0600001016.01.T02	0.0297773	0.0487616299489188	0.610670272798829		1497	349.5	1147.5	33.5	16.5	0.029	0.047	3.75089461
Aes-CH7S01G583200.1	Tu--TuG1812G0700004443.01.T01	0.0053	0.0992967804246585	0.0533757586321119		1734	408.58333	1325.4167	7	38	0.005	0.093	7.63821388
Aes-CH7S01G560300.1	Tu--TuG1812G0700004443.01.T01	0.0030244	0.0936888934181125	0.0322811817364989		1734	408.75	1325.25	4	36	0.003	0.088	7.20683796
Aes-CH6S01G486800.1	Tu--TuG1812G0600003691.01.T01	0.1621645	0.257053184937537	0.63085988150873		1059	286.41667	772.58333	112.7	62.33	0.146	0.218	19.7733219

Aes-CH6S01G486800.1	Tu--TuG1812G0700001616.01.T01	0.2789343	0.545030532789184	0.511777409949768		1002	269.33333	732.66667	170.7	104.3	0.233	0.387	41.9254256
Aes-CH7S01G128000.1	Tu--TuG1812G0700001616.01.T01	0.0013149	0.0880551203114362	0.0149329729380583		1038	276.83333	761.16667	1	23	0.001	0.083	6.77347079
Aes-CH4S01G066000.1	Tu--TuG1812G0400000463.01.T01	0.2343334	0.537286601069677	0.436142323385515		984	268.5	715.5	144	103	0.201	0.384	41.3297385
Aes-CH2S01G410800.1	Tu--TuG1812G0600002720.01.T01	0.1537231	0.854579852023839	0.17988146200477		2616	629.08333	1986.9167	276.2	320.8	0.139	0.51	65.7369117
Aes-CH6S01G361000.1	Tu--TuG1812G0600002720.01.T01	0.0065365	0.0436663323580672	0.149692828654531		2634	636.5	1997.5	13	27	0.007	0.042	3.35894864
Aes-CH3S01G320300.1	Tu--TuG1812G0100004071.01.T01	0.29772	0.6120213011709	0.486453640276547		1191	303.16667	887.83333	218.2	126.8	0.246	0.418	47.0785616
Aes-CH3S01G320300.1	Tu--TuG1812G0300003030.01.T01	0.311075	0.678576320758312	0.458422961761321		1185	298.41667	886.58333	225.8	133.3	0.255	0.447	52.1981785
Aes-CH1S01G358800.1	Tu--TuG1812G0100004071.01.T01	0.0409964	0.119134409751092	0.344118762660927		1458	367.66667	1090.3333	43.5	40.5	0.04	0.11	9.16418537

Aes-CH1S01G358800.1	Tu--TuG1812G0300003030.01.T01	0.3148171	0.78074362539595	0.403227278288915	1260	320.33333	939.66667	241.6	155.4	0.257	0.485	60.057202
Aes-CH5S01G571400.1	Tu--TuG1812G0500004830.01.T01	0.000813	0.0824693581318243	0.00985830650451029	1602	371.33333	1230.6667	1	29	0.001	0.078	6.34379678
Aes-CH5S01G228100.1	Tu--TuG1812G0500002311.01.T01	0.0038351	0.0364748313303763	0.105143719656632	2046	477.5	1568.5	6	17	0.004	0.036	2.80575626
Aes-CH2S01G510600.1	Tu--TuG1812G0200005431.01.T03	0.0072812	0.0698311478482388	0.104268784888706	3951	914.83333	3036.1667	22	61	0.007	0.067	5.37162676
Aes-CH5S01G223800.1	Tu--TuG1812G0500002254.01.T01	0.0123619	0.0658497822801345	0.187728121938305	1296	317.25	978.75	12	20	0.012	0.063	5.06536787
Aes-CH5S01G171800.1	Tu--TuG1812G0500001767.01.T01	0.0063985	0.0618793631984733	0.103403167077156	1884	471.41667	1412.5833	9	28	0.006	0.059	4.75995102
Aes-CH2S01G202900.1	Tu--TuG1812G0200002096.01.T01	0.4406804	0.831022624873256	0.530286859009922	822	195.58333	626.41667	208.8	98.25	0.333	0.502	63.9248173
Aes-CH2S01G196500.1	Tu--TuG1812G0200002096.01.T01	0.0130932	0.0693230556785387	0.188872895819973	966	234.08333	731.91667	9.5	15.5	0.013	0.066	5.33254274

Aes-CH6S01G267500.1	Tu--TuG1812G0600001715.01.T01	0.0092008	0.0495170953733576	0.185810685625442	1167	292.16667	874.83333	8	14	0.009	0.048	3.80900734
Aes-CH6S01G267500.1	Tu--TuG1812G0700004249.01.T01	0.2061337	0.583756519967381	0.353115874885377	1098	266.66667	831.33333	149.8	108.2	0.18	0.406	44.9043477
Aes-CH7S01G572000.1	Tu--TuG1812G0700004249.01.T01	0	0.0467917834558258	0	1188	286.58333	901.41667	0	13	0	0.045	3.59936796
Aes-CH1S01G324900.1	Tu--TuG1812G0100003823.01.T01	0.0159659	0.129501396370546	0.123287855603543	1794	433	1361	21.5	51.5	0.016	0.119	9.96164587
Aes-CH3S01G359100.1	Tu--TuG1812G0100003823.01.T01	0.277234	1.3653680454514	0.203047109000297	1284	306	978	226.7	192.3	0.232	0.629	105.028311
Aes-CH1S01G345800.1	Tu--TuG1812G0100003984.01.T01	0.0721759	0.278953321268972	0.25873844532606	2385	590.25	1794.75	123.5	137.5	0.069	0.233	21.4579478
Aes-CH1S01G345800.1	Tu--TuG1812G0100003933.01.T01	0.0353006	0.122096350832699	0.289121115436606	303	71	232	8	8	0.034	0.113	9.39202699
Aes-CH3S01G303200.1	Tu--TuG1812G0300002902.01.T01	0.251813	1.53109020096301	0.164466490426571	2337	535.91667	1801.0833	385.3	349.8	0.214	0.653	117.776169

Aes-CH1S01G423700.1	Tu--TuG1812G0100004544.01.T01	0.0018634	0.166240206114792	0.0112088106679786	705	167.66667	537.33333	1	25	0.002	0.149	12.7877082
Aes-CH1S01G423700.1	Tu--TuG1812G0300002735.01.T01	0.0675493	0.789155341376171	0.0855969547681668	699	164.91667	534.08333	34.5	80.5	0.065	0.488	60.704257
Aes-CH2S01G225200.1	Tu--TuG1812G0200002657.01.T01	0.4053403	0.912481096235994	0.444217731974035	1929	465.58333	1463.4167	458.3	245.8	0.313	0.528	70.1908536
Aes-CH2S01G225200.1	Tu--TuG1812G0200002474.01.T01	0.3549384	NaN	NaN	1407	327.5	1079.5	305.3	270.7	0.283	0.827	High Sequence Divergence Value (pS>=0.75)
Aes-CH7S01G846700.1	Tu--TuG1812G0700000286.01.T01	0.1121885	0.413743832750576	0.271154608185072	462	106.91667	355.08333	37	34	0.104	0.318	31.8264487
Aes-CH7S01G847900.1	Tu--TuG1812G0700000286.01.T01	0.1326304	0.489010644309415	0.271221954960689	465	107.16667	357.83333	43.5	38.5	0.122	0.359	37.6162034
Aes-CH2S01G633500.1	Tu--TuG1812G0200006101.01.T01	0.0086957	0.0941251378043485	0.0923849865034429	1668	395.66667	1272.3333	11	35	0.009	0.088	7.24039522
Aes-CH3S01G029900.1	Tu--TuG1812G0300000417.01.T01	0.0242467	0.173851210542993	0.139467912019651	1254	290	964	23	45	0.024	0.155	13.37317

Aes-CH3S01G103500.1	Tu--TuG1812G0300001074.01.T02	0.0082367	0.104157408587433	0.0790793319101409	1785	442.16667	1342.8333	11	43	0.008	0.097	8.01210835
Aes-CH5S01G330400.1	Tu--TuG1812G0500003267.01.T02	0.0303455	0.0730665847556012	0.415312680741179	696	158	538	16	11	0.03	0.07	5.62050652
Aes-CH5S01G383000.1	Tu--TuG1812G0500003673.01.T01	0.0058691	0.122268780825224	0.0480014342340689	1785	416.58333	1368.4167	8	47	0.006	0.113	9.40529083
Aes-CH5S01G234600.1	Tu--TuG1812G0500002335.01.T01	0.0135373	0.0779922851430357	0.1735727300046	1320	351	969	13	26	0.013	0.074	5.99940655
Aes-CH5S01G579200.1	Tu--TuG1812G0500004860.01.T01	0.0073762	0.0697366111169322	0.105772918938188	1254	300.33333	953.66667	7	20	0.007	0.067	5.3643547
Aes-CH6S01G246400.1	Tu--TuG1812G0600001625.01.T01	0.0010386	0.0836723823466833	0.0124127159646731	1254	290.5	963.5	1	23	0.001	0.079	6.4363371
Aes-CH7S01G856400.1	Tu--TuG1812G0700000347.01.T01	0.0169365	0.0583471668574607	0.29027076423613	1956	463.16667	1492.8333	25	26	0.017	0.056	4.4882436
Aes-CH1S01G005100.1	Tu--TuG1812G0100000037.01.T01	0.0323787	0.182739482090037	0.177184994745305	1911	501.5	1409.5	44.67	81.33	0.032	0.162	14.0568832

Aes-CH5S01G108100.1	Tu--TuG1812G0500000997.01.T01	0.0069832	0.0896439612271905	0.0778995355442498	2259	532.58333	1726.4167	12	45	0.007	0.084	6.89568933
Aes-CH2S01G224000.1	Tu--TuG1812G0200002486.01.T01	0.0342704	0.103688266402051	0.330513303772615	1998	490.5	1507.5	50.5	47.5	0.033	0.097	7.97602049
Aes-CH3S01G115500.1	Tu--TuG1812G0300001179.01.T01	0.0135383	0.0988104531232662	0.137012566041847	1731	426.66667	1304.3333	17.5	39.5	0.013	0.093	7.60080409
Aes-CH7S01G509000.1	Tu--TuG1812G0700003889.01.T01	0.0458635	0.0990034222564522	0.463252067661587	3066	758.33333	2307.6667	102.7	70.33	0.044	0.093	7.61564787
Aes-CH7S01G458800.1	Tu--TuG1812G0700003889.01.T01	0.0386456	0.102048631473971	0.378697782157535	2649	644.58333	2004.4167	75.5	61.5	0.038	0.095	7.84989473
Aes-CH2S01G234400.1	Tu--TuG1812G0200002891.01.T01	0.0962172	0.164552423773864	0.584720380915915	327	77.833333	249.16667	22.5	11.5	0.09	0.148	12.6578788
Aes-CH4S01G568500.1	Tu--TuG1812G0500005695.01.T01	0.0095136	0.0480644914227494	0.197933132886887	2208	515.5	1692.5	16	24	0.009	0.047	3.69726857
Aes-CH6S01G495000.1	Tu--TuG1812G0600003785.01.T06	0.0025682	0.0420309009556518	0.0611031586212621	2025	464.83333	1560.1667	4	19	0.003	0.041	3.23314623
												Average=22.1596285155407

The Ka/Ks ratio and divergence times between the genes of *T.urartu* and *A.tauschii*												
**Gene ID**	**Gene ID**	**Ka**	**Ks**	**Ka/Ks**	**Effective Len**	**AverageS-sites**	**Average N-sites**	**cN**	**cS**	**pN**	**pS**	**Divergence times (Mya)**
Tu--TuG1812G0400002351.01.T01	Aet-:AET4Gv20512600.2	0	0.0689208432992557	0	1107	258.16667	848.833	0	17	0	0.0658	5.30160333
Tu--TuG1812G0100001066.01.T01	Aet-:AET1Gv20214200.10	0.0048	0.093527608875718	0.0510631972220011	1647	386.66667	1260.33	6	34	0.0048	0.0879	7.19443145
Tu--TuG1812G0100004520.01.T02	Aet-:AET1Gv20987400.1	0.017	0.0377546106718633	0.450964061209805	1725	398.33333	1326.67	22.333	14.667	0.0168	0.0368	2.90420082
Tu--TuG1812G0100004520.01.T02	Aet-:AET3Gv20567900.18	0.1255	1.26283408254075	0.0993922257548571	1440	330.33333	1109.67	128.25	201.75	0.1156	0.6107	97.1410833
Tu--TuG1812G0300002745.01.T01	Aet-:AET1Gv20987400.1	0.1309	1.25327918270647	0.104463539926869	1440	327.33333	1112.67	133.67	199.33	0.1201	0.609	96.406091
Tu--TuG1812G0300002745.01.T01	Aet-:AET3Gv20567900.18	0.0363	0.0858754406979184	0.422546403567249	1458	335.83333	1122.17	39.75	27.25	0.0354	0.0811	6.60580313
Tu--TuG1812G0700003597.01.T01	Aet-:AET7Gv20853400.1	0.0073	0.043229251154466	0.168558641312925	1251	285.66667	965.333	7	12	0.0073	0.042	3.32532701
Tu--TuG1812G0100004629.01.T01	Aet-:AET1Gv20998400.2	0.0089	0.126082259559629	0.0703885638903304	1791	430.83333	1360.17	12	50	0.0088	0.1161	9.69863535
Tu--TuG1812G0100004629.01.T01	Aet-:AET3Gv20558100.1	0.1485	1.01440376366681	0.146406949757957	1767	419.91667	1347.08	181.5	233.5	0.1347	0.5561	78.0310587
Tu--TuG1812G0300002551.01.T01	Aet-:AET3Gv20558100.1	0.0014	0.0427330760569053	0.0333269650444869	1839	433.33333	1405.67	2	18	0.0014	0.0415	3.2871597

Tu--TuG1812G0700004566.01.T01	Aet-:AET7Gv21020800.4	0.0029	0.0721214136778505	0.0407410143095772	1107	254.5	852.5	2.5	17.5	0.0029	0.0688	5.54780105
Tu--TuG1812G0600001016.01.T02	Aet-:AET6Gv20256900.11	0.0114	0.0534880057367498	0.21270402880801	1500	348.66667	1151.33	13	18	0.0113	0.0516	4.11446198
Tu--TuG1812G0700004443.01.T01	Aet-:AET7Gv20996900.4	0.0045	0.0689994663193708	0.0658560759696207	1734	409.58333	1324.42	6	27	0.0045	0.0659	5.30765126
Tu--TuG1812G0600003691.01.T01	Aet-:AET6Gv20859000.1	0.0096	0.0812125508979431	0.117994177104405	1008	272.83333	735.167	7	21	0.0095	0.077	6.2471193
Tu--TuG1812G0700001616.01.T01	Aet-:AET7Gv20388900.1	0.0229	0.144541788542599	0.158770153659166	1026	273.83333	752.167	17	36	0.0226	0.1315	11.1185991
Tu--TuG1812G0400000463.01.T01	Aet-:AET4Gv20097400.1	0.2413	0.531471610109033	0.45393406287235	984	269.41667	714.583	147.42	102.58	0.2063	0.3808	40.8824315
Tu--TuG1812G0600002720.01.T01	Aet-:AET2Gv20903900.5	0.1538	0.81663532615612	0.188295525832228	2616	628.41667	1987.58	276.33	312.67	0.139	0.4975	62.818102
Tu--TuG1812G0600002720.01.T01	Aet-:AET6Gv20637800.1	0.0065	0.0337496682818668	0.193660839041328	2634	636.33333	1997.67	13	21	0.0065	0.033	2.59612833
Tu--TuG1812G0100004071.01.T01	Aet-:AET1Gv20883200.1	0.027	0.117426795518655	0.230042598326359	1461	368	1093	29	40	0.0265	0.1087	9.03283042
Tu--TuG1812G0100004071.01.T01	Aet-:AET3Gv20622200.1	0.3013	0.650759760758272	0.462975095537248	1260	317.58333	942.417	233.83	138.17	0.2481	0.4351	50.0584431
Tu--TuG1812G0300003030.01.T01	Aet-:AET1Gv20883200.1	0.3035	0.71738402605807	0.422999337790689	1230	311.08333	918.917	229.33	143.67	0.2496	0.4618	55.1833866

Tu--TuG1812G0300003030.01.T01	Aet-:AET3Gv20622200.1	0.2948	0.688156918212449	0.428428371489293	1194	299.75	894.25	218	135	0.2438	0.4504	52.9351476
Tu--TuG1812G0500004830.01.T01	Aet-:AET5Gv21075800.4	0.0033	0.0705074951700408	0.0462046801684237	1602	371.5	1230.5	4	25	0.0033	0.0673	5.42365347
Tu--TuG1812G0500002311.01.T01	Aet-:AET5Gv20490900.10	0.0026	0.0301873302205371	0.085203762352627	2031	473.16667	1557.83	4	14	0.0026	0.0296	2.32210232
Tu--TuG1812G0200005431.01.T03	Aet-:AET2Gv21094700.6	0.0119	0.073152065664957	0.162756801087456	3966	918.25	3047.75	36	64	0.0118	0.0697	5.62708197
Tu--TuG1812G0500002254.01.T01	Aet-:AET5Gv20485300.2	0.0147	0.086446553978471	0.169534100202832	1551	379.66667	1171.33	17	31	0.0145	0.0817	6.64973492
Tu--TuG1812G0500001767.01.T01	Aet-:AET5Gv20380700.1	0.0092	0.04295322878838	0.213102296440828	1908	479.08333	1428.92	13	20	0.0091	0.0417	3.30409452
Tu--TuG1812G0200002096.01.T01	Aet-:AET2Gv20411300.2	0.4684	0.776603063734958	0.603115062118616	945	227.58333	717.417	249.92	110.08	0.3484	0.4837	59.7386972
Tu--TuG1812G0200002096.01.T01	Aet-:AET2Gv20397600.3	0.0109	0.0625277380608518	0.173582970034577	879	213.91667	665.083	7.1667	12.833	0.0108	0.06	4.809826
Tu--TuG1812G0600001715.01.T01	Aet-:AET6Gv20435900.1	0.0506	0.11468206723452	0.441185946972528	1107	275.83333	831.167	40.667	29.333	0.0489	0.1063	8.82169748
Tu--TuG1812G0700004249.01.T01	Aet-:AET6Gv20435900.1	0.228	0.719696062505103	0.316868129660233	1116	272.66667	843.333	165.83	126.17	0.1966	0.4627	55.3612356
Tu--TuG1812G0100003823.01.T01	Aet-:AET1Gv20814900.1	0.0126	0.12030733072903	0.104608084987805	1794	431.83333	1362.17	17	48	0.0125	0.1112	9.25441006

Tu--TuG1812G0100003823.01.T01	Aet-:AET3Gv20654200.3	0.3323	1.32564508688524	0.250669932744008	1731	411.08333	1319.92	354.33	255.67	0.2685	0.6219	101.972699
Tu--TuG1812G0100003984.01.T01	Aet-:AET1Gv20861500.1	0.2889	0.520595450298151	0.555010591037392	1179	311.91667	867.083	207.92	117.08	0.2398	0.3754	40.0458039
Tu--TuG1812G0300002902.01.T01	Aet-:AET3Gv20596900.1	0.2492	1.424770224899	0.174885440119388	2337	536.75	1800.25	381.67	342.33	0.212	0.6378	109.59771
Tu--TuG1812G0100004544.01.T01	Aet-:AET1Gv20988700.4	0.0035	0.157766368269016	0.022373363685757	1119	267.08333	851.917	3	38	0.0035	0.1423	12.1358745
Tu--TuG1812G0100004544.01.T01	Aet-:AET3Gv20565300.1	0.1068	0.758017187858094	0.140858427551491	1119	266.58333	852.417	84.833	127.17	0.0995	0.477	58.3090145
Tu--TuG1812G0300002735.01.T01	Aet-:AET1Gv20988700.4	0.0957	0.673627687173771	0.142036339268602	1113	265.08333	847.917	76.167	117.83	0.0898	0.4445	51.8175144
Tu--TuG1812G0200002657.01.T01	Aet-:AET2Gv20463700.1	0.2872	NaN	NaN	990	226.25	763.75	182.25	189.75	0.2386	0.8387	High Sequence Divergence Value (pS>=0.75)
Tu--TuG1812G0200002657.01.T01	Aet-:AET2Gv20462800.7	0.4162	0.953097440882319	0.436717095386415	1941	467.83333	1473.17	470.58	252.42	0.3194	0.5395	73.3151878
Tu--TuG1812G0200002474.01.T01	Aet-:AET2Gv20462900.1	0.3861	NaN	NaN	1443	331.91667	1111.08	335.33	260.67	0.3018	0.7853	High Sequence Divergence Value (pS>=0.75)
Tu--TuG1812G0200006101.01.T01	Aet-:AET2Gv21265400.2	0.0714	0.149238265816286	0.478733567325328	1536	362.08333	1173.92	80	49	0.0681	0.1353	11.4798666

Tu--TuG1812G0300001074.01.T02	Aet-:AET3Gv20208200.4	0.0105	0.0515929085464107	0.203354434926737	1785	441.25	1343.75	14	22	0.0104	0.0499	3.96868527
Tu--TuG1812G0500003267.01.T02	Aet-:AET5Gv20680900.1	0.0437	0.134231405074957	0.325265902887181	198	44.75	153.25	6.5	5.5	0.0424	0.1229	10.3254927
Tu--TuG1812G0500003673.01.T01	Aet-:AET5Gv20774000.3	0.0044	0.0758220876535267	0.0579727769805434	1785	416	1369	6	30	0.0044	0.0721	5.83246828
Tu--TuG1812G0500002335.01.T01	Aet-:AET5Gv20505600.1	0.0104	0.0561700471395463	0.185018110220011	1320	351.08333	968.917	10	19	0.0103	0.0541	4.32077286
Tu--TuG1812G0500004860.01.T01	Aet-:AET5Gv21088300.4	0.0128	0.0560026828092803	0.228566026851822	1242	296.5	945.5	12	16	0.0127	0.054	4.30789868
Tu--TuG1812G0600001625.01.T01	Aet-:AET6Gv20396400.1	0.001	0.0645173681433777	0.0161091683246037	1254	291.16667	962.833	1	18	0.001	0.0618	4.96287447
Tu--TuG1812G0600001625.01.T01	Aet-:AET7Gv21189400.1	0.0882	0.77234281551536	0.114175885447156	1254	292.41667	961.583	80	141	0.0832	0.4822	59.4109858
Tu--TuG1812G0700000347.01.T01	Aet-:AET7Gv20063100.2	0.0128	0.0652830325380597	0.196720184341483	1956	463.83333	1492.17	19	29	0.0127	0.0625	5.02177173
Tu--TuG1812G0100000037.01.T01	Aet-:AET1Gv20001400.6	0.0199	0.221015774276258	0.0898804075221016	1185	283.83333	901.167	17.667	54.333	0.0196	0.1914	17.0012134
Tu--TuG1812G0500000997.01.T01	Aet-:AET5Gv20234400.6	0.0058	0.0366043750210879	0.158786837991951	2259	531.83333	1727.17	10	19	0.0058	0.0357	2.81572116
Tu--TuG1812G0300001179.01.T01	Aet-:AET3Gv20232400.2	0.0198	0.077639544852257	0.255230186457891	1731	427.08333	1303.92	25.5	31.5	0.0196	0.0738	5.97227268

Tu--TuG1812G0700003889.01.T01	Aet-:AET7Gv20906800.1	0.022	0.0660140212071353	0.332941355640537	3099	767.5	2331.5	50.5	48.5	0.0217	0.0632	5.07800163
Tu--TuG1812G0500005695.01.T01	Aet-:AET4Gv20885100.12	0.0053	0.0481851575604212	0.110081205880307	2217	514.25	1702.75	9	24	0.0053	0.0467	3.70655058
Tu--TuG1812G0600003785.01.T06	Aet-:AET6Gv20872100.4	0.0019	0.0378208023040004	0.0508769919960789	2022	460.91667	1561.08	3	17	0.0019	0.0369	2.90929248
												Average=25.3955129918934
