# Supplementary material for: Bioinformatic Identification and Expression Analyses of the MAPK–MAP4K Gene Family Reveal a Putative Functional MAP4K10-MAP3K7/8-MAP2K1/11-MAPK3/6 Cascade in Wheat (Triticum aestivum L.)
Source: Plants (Basel). 2024 Mar 24;13(7):941. doi: 10.3390/plants13070941 (PMC11013086; doi:10.3390/plants13070941)
Supplement: Supplementary file 1 [file plants-13-00941-s001.zip › plants-2867660-supplementary/Supplementary Figure S1-S10 and Supplementary table S1-S7/Supplementary table S1-S7/Supplementary table 4.pdf]

Table S3 The cis-regulatory element of the MAPK-MAP4K cascade gene promoter region in wheat

	Name						Function
TaMAPK1	CGTCA-motif	CGTCA	894	5	+	Hordeum vulgare	the MeJA responsive
TaMAPK1	CGTCA-motif	CGTCA	1816	5	-	Hordeum vulgare	the MeJA responsive
TaMAPK1	TGACG-motif	TGACG	894	5	-	Hordeum vulgare	the MeJA responsive
TaMAPK1	TGACG-motif	TGACG	1816	5	+	Hordeum vulgare	the MeJA responsive
TaMAPK1	O2-site	GATGACATGG	1018	10	-	Zea mays	zein metabolism regulation
TaMAPK1	O2-site	GATGATGTGG	1052	9	-	Zea mays	zein metabolism regulation
TaMAPK1	MSA-like	TCAAACGGT	450	9	-	Catharanthus roseus	cell cycle regulation
TaMAPK1	ARE	AAACCA	587	6	-	Zea mays	anaerobic induction
TaMAPK1	P-box	CCTTTTG	180	7	+	Oryza sativa	gibberellin responsive
TaMAPK1	ABRE	CACGTG	745	6	+	Arabidopsis thaliana	abscisic acid responsive
TaMAPK1	ABRE	ACGTG	746	5	+	Arabidopsis thaliana	abscisic acid responsive
TaMAPK1	TGA-element	AACGAC	1270	6	+	Brassica oleracea	auxin responsive
TaMAPK10	TCA-element	TCAGAAGAGG	1467	9	-	Brassica oleracea	salicylic acid responsive
TaMAPK10	MBS	CAACTG	1408	6	+	Arabidopsis thaliana	drought inducibility
TaMAPK10	ABRE	CGCACGTGTC	253	9	+	Hordeum vulgare	abscisic acid responsive
TaMAPK10	ABRE	CACGTG	255	6	+	Arabidopsis thaliana	abscisic acid responsive
TaMAPK10	ABRE	ACGTG	256	5	+	Arabidopsis thaliana	abscisic acid responsive
TaMAPK10	ABRE	ACGTG	976	5	+	Arabidopsis thaliana	abscisic acid responsive

TaMAPK10	GC-motif	CCCCCG	302	6 +	Zea mays	anoxic specific inducibility
TaMAPK10	GC-motif	CCCCCG	1080	6 -	Zea mays	anoxic specific inducibility
TaMAPK10	TC-rich repeats	ATTCTCTAAC	1646	9 -	Nicotiana tabacum	defense and stress responsive
TaMAPK10	GCN4_motif	TGAGTCA	72	7 -	Oryza sativa	endosperm expression
TaMAPK10	O2-site	GATGACATGG	1420	9 +	Zea mays	zein metabolism regulation
TaMAPK10	ARE	AAACCA	1639	6 -	Zea mays	anaerobic induction
TaMAPK11	ABRE	ACGTG	618	5 -	Arabidopsis thaliana	abscisic acid responsive
TaMAPK11	ABRE	CGTACGTGCA	1401	9 -	Hordeum vulgare	abscisic acid responsive
TaMAPK11	ABRE	CACGTG	1698	6 -	Arabidopsis thaliana	abscisic acid responsive
TaMAPK11	ABRE	ACGTG	1699	5 +	Arabidopsis thaliana	abscisic acid responsive
TaMAPK11	GC-motif	CCCCCG	1035	6 -	Zea mays	anoxic specific inducibility
TaMAPK11	CGTCA-motif	CGTCA	1427	5 -	Hordeum vulgare	the MeJA responsive
TaMAPK11	CGTCA-motif	CGTCA	1456	5 +	Hordeum vulgare	the MeJA responsive
TaMAPK11	CGTCA-motif	CGTCA	1760	5 -	Hordeum vulgare	the MeJA responsive
TaMAPK11	CGTCA-motif	CGTCA	1948	5 +	Hordeum vulgare	the MeJA responsive
TaMAPK11	ARE	AAACCA	81	6 -	Zea mays	anaerobic induction
TaMAPK11	ARE	AAACCA	379	6 +	Zea mays	anaerobic induction
TaMAPK11	ARE	AAACCA	1441	6 -	Zea mays	anaerobic induction
TaMAPK11	TGACG-motif	TGACG	1427	5 +	Hordeum vulgare	the MeJA responsive

TaMAPK11	TGACG-motif	TGACG	1456	5 -	Hordeum vulgare	the MeJA responsive
TaMAPK11	TGACG-motif	TGACG	1760	5 +	Hordeum vulgare	the MeJA responsive
TaMAPK11	TGACG-motif	TGACG	1948	5 -	Hordeum vulgare	the MeJA responsive
TaMAPK11	O2-site	GATGACATGG	1923	9 -	Zea mays	zein metabolism regulation
TaMAPK11	GCN4_motif	TGAGTCA	49	7 -	Oryza sativa	endosperm expression
TaMAPK11	LTR	CCGAAA	824	6 +	Hordeum vulgare	low temperature responsive
TaMAPK11	circadian	CAAAGATATC	1045	9 +	Lycopersicon esculentum	circadian control
TaMAPK11	HD-Zip 1	CAAT(A/T)ATT G	146	8 +	Arabidopsis thaliana	differentiation of the palisade mesophyll cells
TaMAPK12	GC-motif	CCCCCG	1889	6 +	Zea mays	anoxic specific inducibility
TaMAPK12	O2-site	GTTGACGTGA	1047	9 +	Zea mays	zein metabolism regulation
TaMAPK12	ARE	AAACCA	495	6 -	Zea mays	anaerobic induction
TaMAPK12	ABRE	ACGTG	1051	5 +	Arabidopsis thaliana	abscisic acid responsive
TaMAPK12	ABRE	GCCGCGTGGC	1543	9 -	Oryza sativa	abscisic acid responsive
TaMAPK12	ABRE	CACGTG	1606	6 -	Arabidopsis thaliana	abscisic acid responsive
TaMAPK12	ABRE	ACGTG	1607	5 +	Arabidopsis thaliana	abscisic acid responsive
TaMAPK12	ABRE	GACACGTGGC	1702	9 -	Triticum aestivum	abscisic acid responsive
TaMAPK12	TGA-element	AACGAC	1480	6 +	Brassica oleracea	auxin responsive
TaMAPK12	HD-Zip 1	CAAT(A/T)ATT G	30	8 +	Arabidopsis thaliana	differentiation of the palisade mesophyll cells

TaMAPK12	CGTCA-motif	CGTCA	170	5 +	Hordeum vulgare	the MeJA responsive
TaMAPK12	CGTCA-motif	CGTCA	1049	5 -	Hordeum vulgare	the MeJA responsive
TaMAPK12	CGTCA-motif	CGTCA	1381	5 +	Hordeum vulgare	the MeJA responsive
TaMAPK12	CGTCA-motif	CGTCA	1663	5 -	Hordeum vulgare	the MeJA responsive
TaMAPK12	TGACG-motif	TGACG	170	5 -	Hordeum vulgare	the MeJA responsive
TaMAPK12	TGACG-motif	TGACG	1049	5 +	Hordeum vulgare	the MeJA responsive
TaMAPK12	TGACG-motif	TGACG	1381	5 -	Hordeum vulgare	the MeJA responsive
TaMAPK12	TGACG-motif	TGACG	1663	5 +	Hordeum vulgare	the MeJA responsive
TaMAPK12	LTR	CCGAAA	1973	6 +	Hordeum vulgare	low temperature responsive
TaMAPK12	AuxRR-core	GGTCCAT	139	7 +	Nicotiana tabacum	auxin responsive
TaMAPK12	CAT-box	GCCACT	1698	6 -	Arabidopsis thaliana	meristem expression
TaMAPK13	TATC-box	TATCCCA	1921	7 +	Oryza sativa	gibberellin responsive
TaMAPK13	P-box	CCTTTTG	315	7 +	Oryza sativa	gibberellin responsive
TaMAPK13	ABRE	CACGTG	15	6 +	Arabidopsis thaliana	abscisic acid responsive
TaMAPK13	ABRE	ACGTG	16	5 +	Arabidopsis thaliana	abscisic acid responsive
TaMAPK13	ABRE	CACGTG	873	6 +	Arabidopsis thaliana	abscisic acid responsive
TaMAPK13	ABRE	ACGTG	874	5 +	Arabidopsis thaliana	abscisic acid responsive
TaMAPK13	GC-motif	CCCCCG	97	6 +	Zea mays	anoxic specific inducibility
TaMAPK13	TGA-element	AACGAC	674	6 -	Brassica oleracea	auxin responsive
TaMAPK13	TGA-element	AACGAC	1376	6 +	Brassica oleracea	auxin responsive

TaMAPK13	TGACG-motif	TGACG	1022	5 -	Hordeum vulgare	the MeJA responsive
TaMAPK13	TGACG-motif	TGACG	1227	5 -	Hordeum vulgare	the MeJA responsive
TaMAPK13	CGTCA-motif	CGTCA	1022	5 +	Hordeum vulgare	the MeJA responsive
TaMAPK13	CGTCA-motif	CGTCA	1227	5 +	Hordeum vulgare	the MeJA responsive
TaMAPK13	ARE	AAACCA	715	6 -	Zea mays	anaerobic induction
TaMAPK13	O2-site	GATGACATGG	1120	9 -	Zea mays	zein metabolism regulation
TaMAPK13	O2-site	GATGATGTGG	1154	9 -	Zea mays	zein metabolism regulation
TaMAPK13	MSA-like	TCAAACGGT	578	9 -	Catharanthus roseus	cell cycle regulation
TaMAPK14	TC-rich repeats	ATTCTCTAAC	29	9 +	Nicotiana tabacum	defense and stress responsive
TaMAPK14	TC-rich repeats	GTTTTCTTAC	260	9 +	Nicotiana tabacum	defense and stress responsive
TaMAPK14	RY-element	CATGCATG	1068	8 -	Helianthus annuus	seed specific regulation
TaMAPK14	ABRE	ACGTG	38	5 -	Arabidopsis thaliana	abscisic acid responsive
TaMAPK14	ABRE	ACGTG	68	5 +	Arabidopsis thaliana	abscisic acid responsive
TaMAPK14	ABRE	ACGTG	206	5 -	Arabidopsis thaliana	abscisic acid responsive
TaMAPK14	ABRE	GCAACGTGTC	304	9 -	Hordeum vulgare	abscisic acid responsive
TaMAPK14	ABRE	ACGTG	764	5 +	Arabidopsis thaliana	abscisic acid responsive
TaMAPK14	ABRE	GACACGTGGC	880	9 -	Triticum aestivum	abscisic acid responsive
TaMAPK14	ABRE	CACGTG	882	6 +	Arabidopsis thaliana	abscisic acid responsive
TaMAPK14	ABRE	ACGTG	883	5 +	Arabidopsis thaliana	abscisic acid responsive

TaMAPK14	LTR	CCGAAA	47	6 +	Hordeum vulgare	low temperature responsive
TaMAPK14	GARE-motif	TCTGTTG	828	7 -	Brassica oleracea	gibberellin responsive
TaMAPK14	TGACG-motif	TGACG	40	5 -	Hordeum vulgare	the MeJA responsive
TaMAPK14	TGACG-motif	TGACG	208	5 -	Hordeum vulgare	the MeJA responsive
TaMAPK14	TGACG-motif	TGACG	638	5 -	Hordeum vulgare	the MeJA responsive
TaMAPK14	MBS	CAACTG	117	6 -	Arabidopsis thaliana	drought inducibility
TaMAPK14	CGTCA-motif	CGTCA	40	5 +	Hordeum vulgare	the MeJA responsive
TaMAPK14	CGTCA-motif	CGTCA	208	5 +	Hordeum vulgare	the MeJA responsive
TaMAPK14	CGTCA-motif	CGTCA	638	5 +	Hordeum vulgare	the MeJA responsive
TaMAPK14	TGA-element	AACGAC	278	6 -	Brassica oleracea	auxin responsive
TaMAPK14	TGA-element	AACGAC	1637	6 -	Brassica oleracea	auxin responsive
TaMAPK14	TGA-element	AACGAC	1908	6 +	Brassica oleracea	auxin responsive
TaMAPK14	P-box	CCTTTTG	712	7 -	Oryza sativa	gibberellin responsive
TaMAPK14	O2-site	GATGACATGG	1059	9 -	Zea mays	zein metabolism regulation
TaMAPK14	TCA-element	CCATCTTTTT	1864	9 -	Nicotiana tabacum	salicylic acid responsive
TaMAPK14	ARE	AAACCA	84	6 -	Zea mays	anaerobic induction
TaMAPK14	ARE	AAACCA	202	6 +	Zea mays	anaerobic induction
TaMAPK16	ARE	AAACCA	1438	6 -	Zea mays	anaerobic induction
TaMAPK16	ARE	AAACCA	1470	6 +	Zea mays	anaerobic induction
TaMAPK16	ARE	AAACCA	1767	6 +	Zea mays	anaerobic induction

TaMAPK16	ABRE	ACGTG	325	5 -	Arabidopsis thaliana	abscisic acid responsive
TaMAPK16	ABRE	AACCCGG	892	7 -	Arabidopsis thaliana	abscisic acid responsive
TaMAPK16	ABRE	GCCGCGTGGC	1500	9 -	Oryza sativa	abscisic acid responsive
TaMAPK16	ABRE	GCCGCGTGGC	1580	9 -	Oryza sativa	abscisic acid responsive
TaMAPK16	ABRE	ACGTG	1734	5 -	Arabidopsis thaliana	abscisic acid responsive
TaMAPK16	ABRE	CGCACGTGTC	1837	9 -	Hordeum vulgare	abscisic acid responsive
TaMAPK16	CGTCA-motif	CGTCA	247	5 +	Hordeum vulgare	the MeJA responsive
TaMAPK16	CGTCA-motif	CGTCA	1076	5 +	Hordeum vulgare	the MeJA responsive
TaMAPK16	CGTCA-motif	CGTCA	1557	5 +	Hordeum vulgare	the MeJA responsive
TaMAPK16	LTR	CCGAAA	124	6 -	Hordeum vulgare	low temperature responsive
TaMAPK16	TCA-element	CCATCTTTTT	1021	9 -	Nicotiana tabacum	salicylic acid responsive
TaMAPK16	O2-site	GATGATGTGG	378	9 +	Zea mays	zein metabolism regulation
TaMAPK16	O2-site	GATGACATGG	991	9 +	Zea mays	zein metabolism regulation
TaMAPK16	O2-site	GATGACATGG	1129	9 +	Zea mays	zein metabolism regulation
TaMAPK16	O2-site	GATGATGTGG	1551	9 -	Zea mays	zein metabolism regulation
TaMAPK16	GC-motif	CCCCCG	58	6 -	Zea mays	anoxic specific inducibility
TaMAPK16	MBS	CAACTG	331	6 -	Arabidopsis thaliana	drought inducibility
TaMAPK16	TGACG-motif	TGACG	247	5 -	Hordeum vulgare	the MeJA responsive
TaMAPK16	TGACG-motif	TGACG	1076	5 -	Hordeum vulgare	the MeJA responsive

TaMAPK16	TGACG-motif	TGACG	1557	5 -	Hordeum vulgare	the MeJA responsive
TaMAPK17	TATC-box	TATCCCA	875	7 +	Oryza sativa	gibberellin responsive
TaMAPK17	TCA-element	TCAGAAGAGG	34	9 +	Brassica oleracea	salicylic acid responsive
TaMAPK17	TCA-element	CCATCTTTTT	291	9 +	Nicotiana tabacum	salicylic acid responsive
TaMAPK17	TCA-element	TCAGAAGAGG	1518	9 +	Brassica oleracea	salicylic acid responsive
TaMAPK17	TGA-element	AACGAC	1940	6 -	Brassica oleracea	auxin responsive
TaMAPK17	ABRE	ACGTG	240	5 +	Arabidopsis thaliana	abscisic acid responsive
TaMAPK17	ABRE	ACGTG	821	5 +	Arabidopsis thaliana	abscisic acid responsive
TaMAPK17	ABRE	ACGTG	1198	5 -	Arabidopsis thaliana	abscisic acid responsive
TaMAPK17	TGACG-motif	TGACG	304	5 -	Hordeum vulgare	the MeJA responsive
TaMAPK17	TGACG-motif	TGACG	1946	5 -	Hordeum vulgare	the MeJA responsive
TaMAPK17	CGTCA-motif	CGTCA	304	5 +	Hordeum vulgare	the MeJA responsive
TaMAPK17	CGTCA-motif	CGTCA	1946	5 +	Hordeum vulgare	the MeJA responsive
TaMAPK17	ARE	AAACCA	1434	6 -	Zea mays	anaerobic induction
TaMAPK17	ARE	AAACCA	1550	6 -	Zea mays	anaerobic induction
TaMAPK18	TGACG-motif	TGACG	792	5 -	Hordeum vulgare	the MeJA responsive
TaMAPK18	TGACG-motif	TGACG	1230	5 -	Hordeum vulgare	the MeJA responsive
TaMAPK18	CGTCA-motif	CGTCA	792	5 +	Hordeum vulgare	the MeJA responsive
TaMAPK18	CGTCA-motif	CGTCA	1230	5 +	Hordeum vulgare	the MeJA responsive

TaMAPK18	TATC-box	TATCCCA	1154	7 +	Oryza sativa	gibberellin responsive
TaMAPK18	ARE	AAACCA	371	6 -	Zea mays	anaerobic induction
TaMAPK18	ARE	AAACCA	469	6 -	Zea mays	anaerobic induction
TaMAPK18	ARE	AAACCA	1570	6 -	Zea mays	anaerobic induction
TaMAPK18	ABRE	ACGTG	69	5 +	Arabidopsis thaliana	abscisic acid responsive
TaMAPK18	ABRE	ACGTG	156	5 +	Arabidopsis thaliana	abscisic acid responsive
TaMAPK18	ABRE	ACGTG	1023	5 -	Arabidopsis thaliana	abscisic acid responsive
TaMAPK18	ABRE	ACGTG	1159	5 -	Arabidopsis thaliana	abscisic acid responsive
TaMAPK18	ABRE	ACGTG	1694	5 +	Arabidopsis thaliana	abscisic acid responsive
TaMAPK18	GC-motif	CCCCCG	40	6 -	Zea mays	anoxic specific inducibility
TaMAPK18	GC-motif	CCCCCG	92	6 -	Zea mays	anoxic specific inducibility
TaMAPK18	O2-site	GATGACATGG	1215	9 -	Zea mays	zein metabolism regulation
TaMAPK18	GCN4_motif	TGAGTCA	912	7 +	Oryza sativa	endosperm expression
TaMAPK18	GCN4_motif	TGAGTCA	1739	7 -	Oryza sativa	endosperm expression
TaMAPK18	GARE-motif	TCTGTTG	1041	7 +	Brassica oleracea	gibberellin responsive
TaMAPK18	LTR	CCGAAA	472	6 -	Hordeum vulgare	low temperature responsive
TaMAPK19	ABRE	CGTACGTGCA	348	9 +	Hordeum vulgare	abscisic acid responsive
TaMAPK19	ABRE	ACGTG	640	5 +	Arabidopsis thaliana	abscisic acid responsive
TaMAPK19	ABRE	ACGTG	1437	5 +	Arabidopsis thaliana	abscisic acid responsive

TaMAPK19	circadian	CAAAGATATC	105	9 -	Lycopersicon esculentum	circadian control
TaMAPK19	circadian	CAAAGATATC	711	9 -	Lycopersicon esculentum	circadian control
TaMAPK19	GC-motif	CCCCCG	407	6 +	Zea mays	anoxic specific inducibility
TaMAPK19	GC-motif	CCCCCG	1094	6 -	Zea mays	anoxic specific inducibility
TaMAPK19	GC-motif	CCCCCG	1975	6 +	Zea mays	anoxic specific inducibility
TaMAPK19	O2-site	GATGA(C/T)(A/G)TG(A/G)	507	8 +	Zea mays	zein metabolism regulation
TaMAPK19	O2-site	GATGACATGG	1841	9 -	Zea mays	zein metabolism regulation
TaMAPK19	CGTCA-motif	CGTCA	859	5 -	Hordeum vulgare	the MeJA responsive
TaMAPK19	CGTCA-motif	CGTCA	881	5 -	Hordeum vulgare	the MeJA responsive
TaMAPK19	CGTCA-motif	CGTCA	1003	5 -	Hordeum vulgare	the MeJA responsive
TaMAPK19	CGTCA-motif	CGTCA	1435	5 -	Hordeum vulgare	the MeJA responsive
TaMAPK19	TGACG-motif	TGACG	859	5 +	Hordeum vulgare	the MeJA responsive
TaMAPK19	TGACG-motif	TGACG	881	5 +	Hordeum vulgare	the MeJA responsive
TaMAPK19	TGACG-motif	TGACG	1003	5 +	Hordeum vulgare	the MeJA responsive
TaMAPK19	TGACG-motif	TGACG	1435	5 +	Hordeum vulgare	the MeJA responsive
TaMAPK19	AuxRR-core	GGTCCAT	499	7 -	Nicotiana tabacum	auxin responsive
TaMAPK19	LTR	CCGAAA	180	6 +	Hordeum vulgare	low temperature responsive
TaMAPK19	LTR	CCGAAA	387	6 +	Hordeum vulgare	low temperature responsive
TaMAPK19	MBS	CAACTG	433	6 +	Arabidopsis thaliana	drought inducibility

TaMAPK19	CAT-box	GCCACT	301	6 -	Arabidopsis thaliana	meristem expression
TaMAPK19	CAT-box	GCCACT	1768	6 +	Arabidopsis thaliana	meristem expression
TaMAPK19	CAT-box	GCCACT	1781	6 +	Arabidopsis thaliana	meristem expression
TaMAPK2	ABRE	CACGTG	1334	6 -	Arabidopsis thaliana	abscisic acid responsive
TaMAPK2	ABRE	ACGTG	1335	5 +	Arabidopsis thaliana	abscisic acid responsive
TaMAPK2	TGA-element	AACGAC	1929	6 +	Brassica oleracea	auxin responsive
TaMAPK2	CGTCA-motif	CGTCA	126	5 +	Hordeum vulgare	the MeJA responsive
TaMAPK2	CGTCA-motif	CGTCA	359	5 +	Hordeum vulgare	the MeJA responsive
TaMAPK2	CGTCA-motif	CGTCA	1839	5 +	Hordeum vulgare	the MeJA responsive
TaMAPK2	LTR	CCGAAA	766	6 -	Hordeum vulgare	low temperature responsive
TaMAPK2	LTR	CCGAAA	902	6 -	Hordeum vulgare	low temperature responsive
TaMAPK2	MBS	CAACTG	1357	6 -	Arabidopsis thaliana	drought inducibility
TaMAPK2	MBS	CAACTG	1742	6 -	Arabidopsis thaliana	drought inducibility
TaMAPK2	GARE-motif	TCTGTTG	1393	7 +	Brassica oleracea	gibberellin responsive
TaMAPK2	TGACG-motif	TGACG	126	5 -	Hordeum vulgare	the MeJA responsive
TaMAPK2	TGACG-motif	TGACG	359	5 -	Hordeum vulgare	the MeJA responsive
TaMAPK2	TGACG-motif	TGACG	1839	5 -	Hordeum vulgare	the MeJA responsive
TaMAPK2	TC-rich repeats	GTTTTCTTAC	1880	9 +	Nicotiana tabacum	defense and stress responsive
TaMAPK2	GC-motif	CCCCCG	270	6 +	Zea mays	anoxic specific inducibility

TaMAPK2	GC-motif	CCCCCG	299	6 +	Zea mays	anoxic specific inducibility
TaMAPK2	GC-motif	CCCCCG	378	6 +	Zea mays	anoxic specific inducibility
TaMAPK2	GC-motif	CCCCCG	545	6 +	Zea mays	anoxic specific inducibility
TaMAPK2	GC-motif	CCCCCG	629	6 +	Zea mays	anoxic specific inducibility
TaMAPK2	GC-motif	CCCCCG	1784	6 +	Zea mays	anoxic specific inducibility
TaMAPK2	ARE	AAACCA	25	6 +	Zea mays	anaerobic induction
TaMAPK2	ARE	AAACCA	115	6 +	Zea mays	anaerobic induction
TaMAPK2	CAT-box	GCCACT	1296	6 -	Arabidopsis thaliana	meristem expression
TaMAPK2	CAT-box	GCCACT	1300	6 +	Arabidopsis thaliana	meristem expression
TaMAPK20	CAT-box	GCCACT	1885	6 -	Arabidopsis thaliana	meristem expression
TaMAPK20	LTR	CCGAAA	1720	6 +	Hordeum vulgare	low temperature responsive
TaMAPK20	GARE-motif	TCTGTTG	29	7 +	Brassica oleracea	gibberellin responsive
TaMAPK20	GCN4_motif	TGAGTCA	750	7 +	Oryza sativa	endosperm expression
TaMAPK20	TATC-box	TATCCCA	704	7 -	Oryza sativa	gibberellin responsive
TaMAPK20	CGTCA-motif	CGTCA	449	5 -	Hordeum vulgare	the MeJA responsive
TaMAPK20	CGTCA-motif	CGTCA	565	5 -	Hordeum vulgare	the MeJA responsive
TaMAPK20	CGTCA-motif	CGTCA	1634	5 +	Hordeum vulgare	the MeJA responsive
TaMAPK20	TGACG-motif	TGACG	449	5 +	Hordeum vulgare	the MeJA responsive
TaMAPK20	TGACG-motif	TGACG	565	5 +	Hordeum vulgare	the MeJA responsive

TaMAPK20	TGACG-motif	TGACG	1634	5 -	Hordeum vulgare	the MeJA responsive
TaMAPK20	O2-site	GATGACATGG	771	9 +	Zea mays	zein metabolism regulation
TaMAPK20	GC-motif	CCCCCG	234	6 -	Zea mays	anoxic specific inducibility
TaMAPK20	TGA-element	AACGAC	322	6 -	Brassica oleracea	auxin responsive
TaMAPK20	TGA-element	AACGAC	380	6 -	Brassica oleracea	auxin responsive
TaMAPK22	LTR	CCGAAA	1787	6 +	Hordeum vulgare	low temperature responsive
TaMAPK22	GARE-motif	TCTGTTG	959	7 -	Brassica oleracea	gibberellin responsive
TaMAPK22	MBS	CAACTG	84	6 -	Arabidopsis thaliana	drought inducibility
TaMAPK22	CAT-box	GCCACT	1550	6 -	Arabidopsis thaliana	meristem expression
TaMAPK22	TGA-element	AACGAC	1322	6 +	Brassica oleracea	auxin responsive
TaMAPK22	ABRE	ACGTG	554	5 +	Arabidopsis thaliana	abscisic acid responsive
TaMAPK22	ABRE	ACGTG	980	5 +	Arabidopsis thaliana	abscisic acid responsive
TaMAPK22	ABRE	ACGTG	1091	5 -	Arabidopsis thaliana	abscisic acid responsive
TaMAPK22	ABRE	ACGTG	1150	5 +	Arabidopsis thaliana	abscisic acid responsive
TaMAPK22	ABRE	ACGTG	1171	5 +	Arabidopsis thaliana	abscisic acid responsive
TaMAPK22	ABRE	CGTACGTGCA	1455	9 -	Hordeum vulgare	abscisic acid responsive
TaMAPK22	ABRE	ACGTG	1457	5 -	Arabidopsis thaliana	abscisic acid responsive
TaMAPK22	ABRE	GACACGTGGC	1554	9 -	Triticum aestivum	abscisic acid responsive
TaMAPK22	ABRE	ACGTG	1744	5 +	Arabidopsis thaliana	abscisic acid responsive
TaMAPK22	O2-site	GTTGACGTGA	550	9 +	Zea mays	zein metabolism regulation

TaMAPK22	GC-motif	CCCCCG	1263	6 -	Zea mays	anoxic specific inducibility
TaMAPK22	GC-motif	CCCCCG	1393	6 +	Zea mays	anoxic specific inducibility
TaMAPK22	CGTCA-motif	CGTCA	552	5 -	Hordeum vulgare	the MeJA responsive
TaMAPK22	CGTCA-motif	CGTCA	943	5 -	Hordeum vulgare	the MeJA responsive
TaMAPK22	CGTCA-motif	CGTCA	1148	5 -	Hordeum vulgare	the MeJA responsive
TaMAPK22	TGACG-motif	TGACG	552	5 +	Hordeum vulgare	the MeJA responsive
TaMAPK22	TGACG-motif	TGACG	943	5 +	Hordeum vulgare	the MeJA responsive
TaMAPK22	TGACG-motif	TGACG	1148	5 +	Hordeum vulgare	the MeJA responsive
TaMAPK23	CAT-box	GCCACT	522	6 -	Arabidopsis thaliana	meristem expression
TaMAPK23	MBS	CAACTG	101	6 -	Arabidopsis thaliana	drought inducibility
TaMAPK23	MBS	CAACTG	1244	6 -	Arabidopsis thaliana	drought inducibility
TaMAPK23	TGACG-motif	TGACG	283	5 +	Hordeum vulgare	the MeJA responsive
TaMAPK23	TGACG-motif	TGACG	337	5 +	Hordeum vulgare	the MeJA responsive
TaMAPK23	TGACG-motif	TGACG	455	5 +	Hordeum vulgare	the MeJA responsive
TaMAPK23	TGACG-motif	TGACG	942	5 -	Hordeum vulgare	the MeJA responsive
TaMAPK23	TGACG-motif	TGACG	1337	5 -	Hordeum vulgare	the MeJA responsive
TaMAPK23	TGACG-motif	TGACG	1467	5 -	Hordeum vulgare	the MeJA responsive
TaMAPK23	ARE	AAACCA	1577	6 -	Zea mays	anaerobic induction
TaMAPK23	GC-motif	CCCCCG	140	6 -	Zea mays	anoxic specific inducibility

TaMAPK23	GC-motif	CCCCCG	736	6 -	Zea mays	anoxic specific inducibility
TaMAPK23	GC-motif	CCCCCG	1630	6 +	Zea mays	anoxic specific inducibility
TaMAPK23	ABRE	GCCGCGTGGC	298	9 +	Oryza sativa	abscisic acid responsive
TaMAPK23	ABRE	GCCGCGTGGC	1109	9 +	Oryza sativa	abscisic acid responsive
TaMAPK23	ABRE	CACGTG	1543	6 -	Arabidopsis thaliana	abscisic acid responsive
TaMAPK23	ABRE	ACGTG	1544	5 +	Arabidopsis thaliana	abscisic acid responsive
TaMAPK23	ABRE	CACGTG	1668	6 -	Arabidopsis thaliana	abscisic acid responsive
TaMAPK23	ABRE	ACGTG	1669	5 +	Arabidopsis thaliana	abscisic acid responsive
TaMAPK23	CGTCA-motif	CGTCA	283	5 -	Hordeum vulgare	the MeJA responsive
TaMAPK23	CGTCA-motif	CGTCA	337	5 -	Hordeum vulgare	the MeJA responsive
TaMAPK23	CGTCA-motif	CGTCA	455	5 -	Hordeum vulgare	the MeJA responsive
TaMAPK23	CGTCA-motif	CGTCA	942	5 +	Hordeum vulgare	the MeJA responsive
TaMAPK23	CGTCA-motif	CGTCA	1337	5 +	Hordeum vulgare	the MeJA responsive
TaMAPK23	CGTCA-motif	CGTCA	1467	5 +	Hordeum vulgare	the MeJA responsive
TaMAPK23	LTR	CCGAAA	1207	6 -	Hordeum vulgare	low temperature responsive
TaMAPK23	LTR	CCGAAA	1488	6 +	Hordeum vulgare	low temperature responsive
TaMAPK23	TGA-element	AACGAC	195	6 -	Brassica oleracea	auxin responsive
TaMAPK24	TATC-box	TATCCCA	1248	7 -	Oryza sativa	gibberellin responsive
TaMAPK24	TCA-element	CCATCTTTTT	1007	9 -	Nicotiana tabacum	salicylic acid responsive

TaMAPK24	GCN4_motif	TGAGTCA	443	7 +	Oryza sativa	endosperm expression
TaMAPK24	ABRE	CGTACGTGCA	136	10 +	Hordeum vulgare	abscisic acid responsive
TaMAPK24	ABRE	ACGTG	139	5 +	Arabidopsis thaliana	abscisic acid responsive
TaMAPK24	ABRE	ACGTG	248	5 -	Arabidopsis thaliana	abscisic acid responsive
TaMAPK24	ABRE	GCCGCGTGGC	1478	9 -	Oryza sativa	abscisic acid responsive
TaMAPK24	ABRE	GCCGCGTGGC	1558	9 -	Oryza sativa	abscisic acid responsive
TaMAPK24	ABRE	CGCACGTGTC	1829	9 -	Hordeum vulgare	abscisic acid responsive
TaMAPK24	CGTCA-motif	CGTCA	174	5 +	Hordeum vulgare	the MeJA responsive
TaMAPK24	CGTCA-motif	CGTCA	1062	5 +	Hordeum vulgare	the MeJA responsive
TaMAPK24	CGTCA-motif	CGTCA	1535	5 +	Hordeum vulgare	the MeJA responsive
TaMAPK24	CGTCA-motif	CGTCA	1618	5 +	Hordeum vulgare	the MeJA responsive
TaMAPK24	LTR	CCGAAA	150	6 -	Hordeum vulgare	low temperature responsive
TaMAPK24	P-box	CCTTTTG	841	7 -	Oryza sativa	gibberellin responsive
TaMAPK24	ARE	AAACCA	1416	6 -	Zea mays	anaerobic induction
TaMAPK24	ARE	AAACCA	1759	6 +	Zea mays	anaerobic induction
TaMAPK24	TC-rich repeats	GTTTTCTTAC	1576	9 -	Nicotiana tabacum	defense and stress responsive
TaMAPK24	TGACG-motif	TGACG	174	5 -	Hordeum vulgare	the MeJA responsive
TaMAPK24	TGACG-motif	TGACG	1062	5 -	Hordeum vulgare	the MeJA responsive
TaMAPK24	TGACG-motif	TGACG	1535	5 -	Hordeum vulgare	the MeJA responsive

TaMAPK24	TGACG-motif	TGACG	1618	5 -	Hordeum vulgare	the MeJA responsive
TaMAPK24	O2-site	GATGACATGG	977	9 +	Zea mays	zein metabolism regulation
TaMAPK24	O2-site	GATGACATGG	1115	9 +	Zea mays	zein metabolism regulation
TaMAPK24	O2-site	GATGATGTGG	1529	9 -	Zea mays	zein metabolism regulation
TaMAPK25	TC-rich repeats	GTTTTCTTAC	1086	9 +	Nicotiana tabacum	defense and stress responsive
TaMAPK25	TGACG-motif	TGACG	1534	5 -	Hordeum vulgare	the MeJA responsive
TaMAPK25	ARE	AAACCA	1357	6 +	Zea mays	anaerobic induction
TaMAPK25	ABRE	ACGTG	1245	5 -	Arabidopsis thaliana	abscisic acid responsive
TaMAPK25	CGTCA-motif	CGTCA	1534	5 +	Hordeum vulgare	the MeJA responsive
TaMAPK25	circadian	CAAAGATATC	1082	9 +	Lycopersicon esculentum	circadian control
TaMAPK26	TC-rich repeats	GTTTTCTTAC	1945	9 +	Nicotiana tabacum	defense and stress responsive
TaMAPK26	LTR	CCGAAA	830	6 -	Hordeum vulgare	low temperature responsive
TaMAPK26	LTR	CCGAAA	875	6 -	Hordeum vulgare	low temperature responsive
TaMAPK26	CAT-box	GCCACT	1379	6 -	Arabidopsis thaliana	meristem expression
TaMAPK26	CAT-box	GCCACT	1383	6 +	Arabidopsis thaliana	meristem expression
TaMAPK26	GARE-motif	TCTGTTG	1475	7 +	Brassica oleracea	gibberellin responsive
TaMAPK26	MBS	CAACTG	1088	6 +	Arabidopsis thaliana	drought inducibility
TaMAPK26	MBS	CAACTG	1439	6 -	Arabidopsis thaliana	drought inducibility
TaMAPK26	MBS	CAACTG	1807	6 -	Arabidopsis thaliana	drought inducibility

TaMAPK26	ABRE	GCCGCGTGGC	140	9 +	Oryza sativa	abscisic acid responsive
TaMAPK26	ABRE	CACGTG	1416	6 -	Arabidopsis thaliana	abscisic acid responsive
TaMAPK26	ABRE	ACGTG	1417	5 +	Arabidopsis thaliana	abscisic acid responsive
TaMAPK26	ABRE	ACGTG	1712	5 +	Arabidopsis thaliana	abscisic acid responsive
TaMAPK26	GC-motif	CCCCCG	17	6 +	Zea mays	anoxic specific inducibility
TaMAPK26	GC-motif	CCCCCG	46	6 +	Zea mays	anoxic specific inducibility
TaMAPK26	GC-motif	CCCCCG	125	6 +	Zea mays	anoxic specific inducibility
TaMAPK26	GC-motif	CCCCCG	588	6 +	Zea mays	anoxic specific inducibility
TaMAPK26	GC-motif	CCCCCG	1849	6 +	Zea mays	anoxic specific inducibility
TaMAPK26	TGA-element	AACGAC	1994	6 +	Brassica oleracea	auxin responsive
TaMAPK27	ARE	AAACCA	99	6 +	Zea mays	anaerobic induction
TaMAPK27	TGACG-motif	TGACG	11	5 -	Hordeum vulgare	the MeJA responsive
TaMAPK27	TGACG-motif	TGACG	1443	5 +	Hordeum vulgare	the MeJA responsive
TaMAPK27	TGACG-motif	TGACG	1948	5 -	Hordeum vulgare	the MeJA responsive
TaMAPK27	GCN4_motif	TGAGTCA	1507	7 +	Oryza sativa	endosperm expression
TaMAPK27	O2-site	GATGACATGG	1923	9 -	Zea mays	zein metabolism regulation
TaMAPK27	LTR	CCGAAA	778	6 +	Hordeum vulgare	low temperature responsive
TaMAPK27	circadian	CAAAGATATC	999	9 +	Lycopersicon esculentum	circadian control
TaMAPK27	P-box	CCTTTTG	1853	7 +	Oryza sativa	gibberellin responsive

TaMAPK27	ABRE	ACGTG	339	5 +	Arabidopsis thaliana	abscisic acid responsive
TaMAPK27	ABRE	ACGTG	567	5 -	Arabidopsis thaliana	abscisic acid responsive
TaMAPK27	GC-motif	CCCCCG	961	6 +	Zea mays	anoxic specific inducibility
TaMAPK27	TGA-element	AACGAC	1556	6 -	Brassica oleracea	auxin responsive
TaMAPK27	CGTCA-motif	CGTCA	11	5 +	Hordeum vulgare	the MeJA responsive
TaMAPK27	CGTCA-motif	CGTCA	1443	5 -	Hordeum vulgare	the MeJA responsive
TaMAPK27	CGTCA-motif	CGTCA	1948	5 +	Hordeum vulgare	the MeJA responsive
TaMAPK28	TATC-box	TATCCCA	1737	7 -	Oryza sativa	gibberellin responsive
TaMAPK28	AuxRR-core	GGTCCAT	1640	7 -	Nicotiana tabacum	auxin responsive
TaMAPK28	LTR	CCGAAA	41	6 +	Hordeum vulgare	low temperature responsive
TaMAPK28	MBSI	aaaAaaC(G/C)GTTA	262	10.5 -	Petunia hybrida	flavonoid biosynthetic genes regulation
TaMAPK28	TGACG-motif	TGACG	34	5 -	Hordeum vulgare	the MeJA responsive
TaMAPK28	TGACG-motif	TGACG	205	5 -	Hordeum vulgare	the MeJA responsive
TaMAPK28	TGACG-motif	TGACG	637	5 -	Hordeum vulgare	the MeJA responsive
TaMAPK28	TCA-element	CCATCTTTTT	1865	9 -	Nicotiana tabacum	salicylic acid responsive
TaMAPK28	ARE	AAACCA	78	6 -	Zea mays	anaerobic induction
TaMAPK28	ARE	AAACCA	199	6 +	Zea mays	anaerobic induction
TaMAPK28	RY-element	CATGCATG	1182	8 -	Helianthus annuus	seed specific regulation
TaMAPK28	RY-element	CATGCATG	1307	8 -	Helianthus annuus	seed specific regulation

TaMAPK28	MBS	CAACTG	111	6 -	Arabidopsis thaliana	drought inducibility
TaMAPK28	TGA-element	AACGAC	1908	6 +	Brassica oleracea	auxin responsive
TaMAPK28	CGTCA-motif	CGTCA	34	5 +	Hordeum vulgare	the MeJA responsive
TaMAPK28	CGTCA-motif	CGTCA	205	5 +	Hordeum vulgare	the MeJA responsive
TaMAPK28	CGTCA-motif	CGTCA	637	5 +	Hordeum vulgare	the MeJA responsive
TaMAPK28	P-box	CCTTTTG	805	7 -	Oryza sativa	gibberellin responsive
TaMAPK28	ABRE	ACGTG	32	5 -	Arabidopsis thaliana	abscisic acid responsive
TaMAPK28	ABRE	ACGTG	203	5 -	Arabidopsis thaliana	abscisic acid responsive
TaMAPK28	ABRE	ACGTG	343	5 -	Arabidopsis thaliana	abscisic acid responsive
TaMAPK29	GC-motif	CCCCCG	1348	6 +	Zea mays	anoxic specific inducibility
TaMAPK29	ABRE	ACGTG	30	5 +	Arabidopsis thaliana	abscisic acid responsive
TaMAPK29	ABRE	ACGTG	244	5 -	Arabidopsis thaliana	abscisic acid responsive
TaMAPK29	ABRE	ACGTG	1457	5 -	Arabidopsis thaliana	abscisic acid responsive
TaMAPK29	ABRE	CACGTG	1616	6 -	Arabidopsis thaliana	abscisic acid responsive
TaMAPK29	ABRE	ACGTG	1617	5 +	Arabidopsis thaliana	abscisic acid responsive
TaMAPK29	ABRE	ACGTG	1663	5 -	Arabidopsis thaliana	abscisic acid responsive
TaMAPK29	CGTCA-motif	CGTCA	741	5 -	Hordeum vulgare	the MeJA responsive
TaMAPK29	CGTCA-motif	CGTCA	1255	5 -	Hordeum vulgare	the MeJA responsive
TaMAPK29	CAT-box	GCCACT	1568	6 -	Arabidopsis thaliana	meristem expression

TaMAPK29	TGACG-motif	TGACG	741	5 +	Hordeum vulgare	the MeJA responsive
TaMAPK29	TGACG-motif	TGACG	1255	5 +	Hordeum vulgare	the MeJA responsive
TaMAPK29	LTR	CCGAAA	899	6 -	Hordeum vulgare	low temperature responsive
TaMAPK3	AuxRR-core	GGTCCAT	1153	7 +	Nicotiana tabacum	auxin responsive
TaMAPK3	AuxRR-core	GGTCCAT	1165	7 -	Nicotiana tabacum	auxin responsive
TaMAPK3	CAT-box	GCCACT	1066	6 +	Arabidopsis thaliana	meristem expression
TaMAPK3	GC-motif	CCCCCG	999	6 +	Zea mays	anoxic specific inducibility
TaMAPK3	TCA-element	CCATCTTTTT	828	9 -	Nicotiana tabacum	salicylic acid responsive
TaMAPK3	ARE	AAACCA	273	6 +	Zea mays	anaerobic induction
TaMAPK3	ARE	AAACCA	1335	6 +	Zea mays	anaerobic induction
TaMAPK3	ARE	AAACCA	1389	6 -	Zea mays	anaerobic induction
TaMAPK3	TGA-element	AACGAC	411	6 +	Brassica oleracea	auxin responsive
TaMAPK3	TGA-element	AACGAC	590	6 +	Brassica oleracea	auxin responsive
TaMAPK3	TGA-element	AACGAC	1840	6 +	Brassica oleracea	auxin responsive
TaMAPK3	ABRE	GACACGTGGC	809	9 +	Triticum aestivum	abscisic acid responsive
TaMAPK3	ABRE	AACCCGG	1774	7 +	Arabidopsis thaliana	abscisic acid responsive
TaMAPK3	P-box	CCTTTTG	1047	7 -	Oryza sativa	gibberellin responsive
TaMAPK3	CGTCA-motif	CGTCA	38	5 +	Hordeum vulgare	the MeJA responsive
TaMAPK3	CGTCA-motif	CGTCA	773	5 -	Hordeum vulgare	the MeJA responsive
TaMAPK3	CGTCA-motif	CGTCA	1635	5 +	Hordeum vulgare	the MeJA responsive

TaMAPK3	TGACG-motif	TGACG	38	5 -	Hordeum vulgare	the MeJA responsive
TaMAPK3	TGACG-motif	TGACG	773	5 +	Hordeum vulgare	the MeJA responsive
TaMAPK3	TGACG-motif	TGACG	1635	5 -	Hordeum vulgare	the MeJA responsive
TaMAPK30	ABRE	ACGTG	111	5 +	Arabidopsis thaliana	abscisic acid responsive
TaMAPK30	ABRE	ACGTG	965	5 -	Arabidopsis thaliana	abscisic acid responsive
TaMAPK30	ABRE	GCCGCGTGGC	1546	9 -	Oryza sativa	abscisic acid responsive
TaMAPK30	ABRE	GCCGCGTGGC	1665	9 -	Oryza sativa	abscisic acid responsive
TaMAPK30	TGA-element	AACGAC	931	6 +	Brassica oleracea	auxin responsive
TaMAPK30	circadian	CAAAGATATC	474	9 +	Lycopersicon esculentum	circadian control
TaMAPK30	TATC-box	TATCCCA	377	7 +	Oryza sativa	gibberellin responsive
TaMAPK30	MBS	CAACTG	702	6 -	Arabidopsis thaliana	drought inducibility
TaMAPK30	MBS	CAACTG	847	6 +	Arabidopsis thaliana	drought inducibility
TaMAPK30	TC-rich repeats	ATTCTCTAAC	255	9 +	Nicotiana tabacum	defense and stress responsive
TaMAPK30	ARE	AAACCA	233	6 +	Zea mays	anaerobic induction
TaMAPK30	ARE	AAACCA	1357	6 +	Zea mays	anaerobic induction
TaMAPK30	GC-motif	CCCCCG	575	6 +	Zea mays	anoxic specific inducibility
TaMAPK30	CAT-box	GCCACT	788	6 +	Arabidopsis thaliana	meristem expression
TaMAPK30	CAT-box	GCCACT	976	6 +	Arabidopsis thaliana	meristem expression
TaMAPK31	MBS	CAACTG	700	6 -	Arabidopsis thaliana	drought inducibility

TaMAPK31	GARE-motif	TCTGTTG	1039	7 +	Brassica oleracea	gibberellin responsive
TaMAPK31	O2-site	GATGACATGG	1211	9 -	Zea mays	zein metabolism regulation
TaMAPK31	TGACG-motif	TGACG	747	5 +	Hordeum vulgare	the MeJA responsive
TaMAPK31	TGACG-motif	TGACG	1226	5 -	Hordeum vulgare	the MeJA responsive
TaMAPK31	GC-motif	CCCCCG	104	6 -	Zea mays	anoxic specific inducibility
TaMAPK31	GC-motif	CCCCCG	119	6 -	Zea mays	anoxic specific inducibility
TaMAPK31	GC-motif	CCCCCG	613	6 -	Zea mays	anoxic specific inducibility
TaMAPK31	ARE	AAACCA	581	6 -	Zea mays	anaerobic induction
TaMAPK31	ARE	AAACCA	1568	6 -	Zea mays	anaerobic induction
TaMAPK31	NON-box	AGATCGACG	562	9 +	Arabidopsis thaliana	meristem specific activation
TaMAPK31	ABRE	ACGTG	294	5 +	Arabidopsis thaliana	abscisic acid responsive
TaMAPK31	ABRE	ACGTG	777	5 +	Arabidopsis thaliana	abscisic acid responsive
TaMAPK31	ABRE	ACGTG	1155	5 -	Arabidopsis thaliana	abscisic acid responsive
TaMAPK31	ABRE	ACGTG	1692	5 +	Arabidopsis thaliana	abscisic acid responsive
TaMAPK31	CGTCA-motif	CGTCA	747	5 -	Hordeum vulgare	the MeJA responsive
TaMAPK31	CGTCA-motif	CGTCA	1226	5 +	Hordeum vulgare	the MeJA responsive
TaMAPK31	TATC-box	TATCCCA	1150	7 +	Oryza sativa	gibberellin responsive
TaMAPK31	GCN4_motif	TGAGTCA	850	7 +	Oryza sativa	endosperm expression
TaMAPK31	GCN4_motif	TGAGTCA	1738	7 -	Oryza sativa	endosperm expression

TaMAPK33	CGTCA-motif	CGTCA	956	5 -	Hordeum vulgare	the MeJA responsive
TaMAPK33	CGTCA-motif	CGTCA	1313	5 -	Hordeum vulgare	the MeJA responsive
TaMAPK33	CGTCA-motif	CGTCA	1414	5 +	Hordeum vulgare	the MeJA responsive
TaMAPK33	CGTCA-motif	CGTCA	1916	5 -	Hordeum vulgare	the MeJA responsive
TaMAPK33	CAT-box	GCCACT	438	6 -	Arabidopsis thaliana	meristem expression
TaMAPK33	CAT-box	GCCACT	661	6 +	Arabidopsis thaliana	meristem expression
TaMAPK33	CAT-box	GCCACT	1766	6 +	Arabidopsis thaliana	meristem expression
TaMAPK33	TGA-element	AACGAC	9	6 +	Brassica oleracea	auxin responsive
TaMAPK33	GC-motif	CCCCCG	186	6 +	Zea mays	anoxic specific inducibility
TaMAPK33	GC-motif	CCCCCG	1973	6 +	Zea mays	anoxic specific inducibility
TaMAPK33	ABRE	ACGTG	532	5 -	Arabidopsis thaliana	abscisic acid responsive
TaMAPK33	ABRE	CGCACGTGTC	1176	10 +	Hordeum vulgare	abscisic acid responsive
TaMAPK33	ABRE	CACGTG	1178	6 -	Arabidopsis thaliana	abscisic acid responsive
TaMAPK33	ABRE	ACGTG	1179	5 +	Arabidopsis thaliana	abscisic acid responsive
TaMAPK33	TGACG-motif	TGACG	956	5 +	Hordeum vulgare	the MeJA responsive
TaMAPK33	TGACG-motif	TGACG	1313	5 +	Hordeum vulgare	the MeJA responsive
TaMAPK33	TGACG-motif	TGACG	1414	5 -	Hordeum vulgare	the MeJA responsive
TaMAPK33	TGACG-motif	TGACG	1916	5 +	Hordeum vulgare	the MeJA responsive
TaMAPK33	LTR	CCGAAA	1708	6 +	Hordeum vulgare	low temperature responsive

TaMAPK33	GCN4_motif	TGAGTCA	928	7 +	Oryza sativa	endosperm expression
TaMAPK34	GC-motif	CCCCCG	1294	6 +	Zea mays	anoxic specific inducibility
TaMAPK34	GC-motif	CCCCCG	1839	6 +	Zea mays	anoxic specific inducibility
TaMAPK34	ABRE	CACGTG	32	6 +	Arabidopsis thaliana	abscisic acid responsive
TaMAPK34	ABRE	ACGTG	33	5 +	Arabidopsis thaliana	abscisic acid responsive
TaMAPK34	ABRE	CACGTG	147	6 +	Arabidopsis thaliana	abscisic acid responsive
TaMAPK34	ABRE	ACGTG	148	5 +	Arabidopsis thaliana	abscisic acid responsive
TaMAPK34	ABRE	ACGTG	170	5 +	Arabidopsis thaliana	abscisic acid responsive
TaMAPK34	ABRE	ACGTG	718	5 +	Arabidopsis thaliana	abscisic acid responsive
TaMAPK34	ABRE	CACGTG	1161	6 -	Arabidopsis thaliana	abscisic acid responsive
TaMAPK34	ABRE	ACGTG	1162	5 +	Arabidopsis thaliana	abscisic acid responsive
TaMAPK34	ABRE	ACGTG	1881	5 +	Arabidopsis thaliana	abscisic acid responsive
TaMAPK34	TC-rich repeats	ATTCTCTAAC	62	9 -	Nicotiana tabacum	defense and stress responsive
TaMAPK34	TGACG-motif	TGACG	151	5 +	Hordeum vulgare	the MeJA responsive
TaMAPK34	TGACG-motif	TGACG	336	5 -	Hordeum vulgare	the MeJA responsive
TaMAPK34	TGACG-motif	TGACG	422	5 -	Hordeum vulgare	the MeJA responsive
TaMAPK34	AuxRR-core	GGTCCAT	1049	7 -	Nicotiana tabacum	auxin responsive
TaMAPK34	TGA-element	AACGAC	1302	6 +	Brassica oleracea	auxin responsive
TaMAPK34	CGTCA-motif	CGTCA	151	5 -	Hordeum vulgare	the MeJA responsive

TaMAPK34	CGTCA-motif	CGTCA	336	5 +	Hordeum vulgare	the MeJA responsive
TaMAPK34	CGTCA-motif	CGTCA	422	5 +	Hordeum vulgare	the MeJA responsive
TaMAPK34	CAT-box	GCCACT	828	6 -	Arabidopsis thaliana	meristem expression
TaMAPK34	MBS	CAACTG	372	6 +	Arabidopsis thaliana	drought inducibility
TaMAPK34	MBS	CAACTG	1503	6 -	Arabidopsis thaliana	drought inducibility
TaMAPK34	ARE	AAACCA	730	6 -	Zea mays	anaerobic induction
TaMAPK34	ARE	AAACCA	1675	6 +	Zea mays	anaerobic induction
TaMAPK34	TCA-element	CCATCTTTTT	400	9 -	Nicotiana tabacum	salicylic acid responsive
TaMAPK34	TCA-element	CCATCTTTTT	904	9 +	Nicotiana tabacum	salicylic acid responsive
TaMAPK35	GARE-motif	TCTGTTG	1629	7 +	Brassica oleracea	gibberellin responsive
TaMAPK35	TCA-element	CCATCTTTTT	262	9 +	Nicotiana tabacum	salicylic acid responsive
TaMAPK35	TCA-element	TCAGAAGAGG	1514	9 +	Brassica oleracea	salicylic acid responsive
TaMAPK35	TATC-box	TATCCCA	879	7 +	Oryza sativa	gibberellin responsive
TaMAPK35	TGA-element	AACGAC	1940	6 -	Brassica oleracea	auxin responsive
TaMAPK35	TGACG-motif	TGACG	1946	5 -	Hordeum vulgare	the MeJA responsive
TaMAPK35	CGTCA-motif	CGTCA	1946	5 +	Hordeum vulgare	the MeJA responsive
TaMAPK35	ARE	AAACCA	1430	6 -	Zea mays	anaerobic induction
TaMAPK35	ARE	AAACCA	1546	6 -	Zea mays	anaerobic induction
TaMAPK35	ARE	AAACCA	1783	6 -	Zea mays	anaerobic induction

TaMAPK36	MBS	CAACTG	1412	6 +	Arabidopsis thaliana	drought inducibility
TaMAPK36	TGACG-motif	TGACG	1004	5 +	Hordeum vulgare	the MeJA responsive
TaMAPK36	TGACG-motif	TGACG	1380	5 -	Hordeum vulgare	the MeJA responsive
TaMAPK36	ARE	AAACCA	1162	6 -	Zea mays	anaerobic induction
TaMAPK36	ARE	AAACCA	1636	6 -	Zea mays	anaerobic induction
TaMAPK36	ABRE	CGCACGTGTC	292	9 +	Hordeum vulgare	abscisic acid responsive
TaMAPK36	ABRE	CACGTG	294	6 +	Arabidopsis thaliana	abscisic acid responsive
TaMAPK36	ABRE	ACGTG	295	5 +	Arabidopsis thaliana	abscisic acid responsive
TaMAPK36	GC-motif	CCCCCG	1077	6 -	Zea mays	anoxic specific inducibility
TaMAPK36	CGTCA-motif	CGTCA	1004	5 -	Hordeum vulgare	the MeJA responsive
TaMAPK36	CGTCA-motif	CGTCA	1380	5 +	Hordeum vulgare	the MeJA responsive
TaMAPK36	GCN4_motif	TGAGTCA	86	7 -	Oryza sativa	endosperm expression
TaMAPK36	CAT-box	GCCACT	1264	6 -	Arabidopsis thaliana	meristem expression
TaMAPK36	O2-site	GATGACATGG	1424	9 +	Zea mays	zein metabolism regulation
TaMAPK36	O2-site	GTTGACGTGA	1718	9 -	Zea mays	zein metabolism regulation
TaMAPK37	ARE	AAACCA	874	6 -	Zea mays	anaerobic induction
TaMAPK37	ARE	AAACCA	1757	6 +	Zea mays	anaerobic induction
TaMAPK37	O2-site	GATGACATGG	989	10 -	Zea mays	zein metabolism regulation
TaMAPK37	O2-site	GATGATGTGG	1023	9 -	Zea mays	zein metabolism regulation

TaMAPK37	TGACG-motif	TGACG	891	5 -	Hordeum vulgare	the MeJA responsive
TaMAPK37	TGACG-motif	TGACG	1783	5 +	Hordeum vulgare	the MeJA responsive
TaMAPK37	CGTCA-motif	CGTCA	891	5 +	Hordeum vulgare	the MeJA responsive
TaMAPK37	CGTCA-motif	CGTCA	1783	5 -	Hordeum vulgare	the MeJA responsive
TaMAPK37	TGA-element	AACGAC	528	6 -	Brassica oleracea	auxin responsive
TaMAPK37	TGA-element	AACGAC	1241	6 +	Brassica oleracea	auxin responsive
TaMAPK37	ABRE	CACGTG	730	6 +	Arabidopsis thaliana	abscisic acid responsive
TaMAPK37	ABRE	ACGTG	731	5 +	Arabidopsis thaliana	abscisic acid responsive
TaMAPK37	MBS	CAACTG	894	6 -	Arabidopsis thaliana	drought inducibility
TaMAPK37	TATC-box	TATCCCA	449	7 -	Oryza sativa	gibberellin responsive
TaMAPK38	TGA-element	AACGAC	1930	6 -	Brassica oleracea	auxin responsive
TaMAPK38	ABRE	ACGTG	31	5 -	Arabidopsis thaliana	abscisic acid responsive
TaMAPK38	ABRE	ACGTG	76	5 -	Arabidopsis thaliana	abscisic acid responsive
TaMAPK38	ABRE	ACGTG	92	5 -	Arabidopsis thaliana	abscisic acid responsive
TaMAPK38	ARE	AAACCA	1274	6 +	Zea mays	anaerobic induction
TaMAPK38	circadian	CAAAGATATC	524	9 +	Lycopersicon esculentum	circadian control
TaMAPK38	GC-motif	CCCCCG	1956	6 -	Zea mays	anoxic specific inducibility
TaMAPK38	TCA-element	TCAGAAGAGG	1430	9 +	Brassica oleracea	salicylic acid responsive
TaMAPK38	P-box	CCTTTTG	158	7 -	Oryza sativa	gibberellin responsive
TaMAPK38	P-box	CCTTTTG	1301	7 -	Oryza sativa	gibberellin responsive

TaMAPK38	LTR	CCGAAA	878	6 +	Hordeum vulgare	low temperature responsive
TaMAPK39	TGACG-motif	TGACG	622	5 -	Hordeum vulgare	the MeJA responsive
TaMAPK39	TGACG-motif	TGACG	1077	5 -	Hordeum vulgare	the MeJA responsive
TaMAPK39	TGACG-motif	TGACG	1640	5 -	Hordeum vulgare	the MeJA responsive
TaMAPK39	ARE	AAACCA	107	6 +	Zea mays	anaerobic induction
TaMAPK39	ARE	AAACCA	215	6 +	Zea mays	anaerobic induction
TaMAPK39	ARE	AAACCA	318	6 +	Zea mays	anaerobic induction
TaMAPK39	ARE	AAACCA	1449	6 -	Zea mays	anaerobic induction
TaMAPK39	ARE	AAACCA	1759	6 +	Zea mays	anaerobic induction
TaMAPK39	ABRE	GACACGTGGC	514	9 +	Triticum aestivum	abscisic acid responsive
TaMAPK39	ABRE	CACGTG	516	6 +	Arabidopsis thaliana	abscisic acid responsive
TaMAPK39	ABRE	ACGTG	517	5 +	Arabidopsis thaliana	abscisic acid responsive
TaMAPK39	ABRE	ACGTG	699	5 -	Arabidopsis thaliana	abscisic acid responsive
TaMAPK39	ABRE	AACCCGG	785	7 -	Arabidopsis thaliana	abscisic acid responsive
TaMAPK39	ABRE	AACCCGG	897	7 -	Arabidopsis thaliana	abscisic acid responsive
TaMAPK39	ABRE	GCCGCGTGGC	1512	9 -	Oryza sativa	abscisic acid responsive
TaMAPK39	ABRE	GCCGCGTGGC	1592	9 -	Oryza sativa	abscisic acid responsive
TaMAPK39	CAT-box	GCCACT	285	6 +	Arabidopsis thaliana	meristem expression
TaMAPK39	LTR	CCGAAA	891	6 -	Hordeum vulgare	low temperature responsive

TaMAPK39	CGTCA-motif	CGTCA	622	5 +	Hordeum vulgare	the MeJA responsive
TaMAPK39	CGTCA-motif	CGTCA	1077	5 +	Hordeum vulgare	the MeJA responsive
TaMAPK39	CGTCA-motif	CGTCA	1640	5 +	Hordeum vulgare	the MeJA responsive
TaMAPK39	MBS	CAACTG	435	6 -	Arabidopsis thaliana	drought inducibility
TaMAPK39	TCA-element	CCATCTTTTT	1022	9 -	Nicotiana tabacum	salicylic acid responsive
TaMAPK39	O2-site	GATGATGTGG	377	9 -	Zea mays	zein metabolism regulation
TaMAPK39	O2-site	GATGATGTGG	752	9 +	Zea mays	zein metabolism regulation
TaMAPK39	O2-site	GATGACATGG	992	9 +	Zea mays	zein metabolism regulation
TaMAPK39	O2-site	GATGACATGG	1126	9 +	Zea mays	zein metabolism regulation
TaMAPK39	GC-motif	CCCCCG	1912	6 -	Zea mays	anoxic specific inducibility
TaMAPK4	CGTCA-motif	CGTCA	581	5 +	Hordeum vulgare	the MeJA responsive
TaMAPK4	CGTCA-motif	CGTCA	1760	5 -	Hordeum vulgare	the MeJA responsive
TaMAPK4	ABRE	ACGTG	579	5 -	Arabidopsis thaliana	abscisic acid responsive
TaMAPK4	ABRE	CACGTG	1698	6 -	Arabidopsis thaliana	abscisic acid responsive
TaMAPK4	ABRE	ACGTG	1699	5 +	Arabidopsis thaliana	abscisic acid responsive
TaMAPK4	GC-motif	CCCCCG	614	6 +	Zea mays	anoxic specific inducibility
TaMAPK4	GC-motif	CCCCCG	994	6 +	Zea mays	anoxic specific inducibility
TaMAPK4	GC-motif	CCCCCG	1033	6 -	Zea mays	anoxic specific inducibility
TaMAPK4	TC-rich repeats	GTTTTCTTAC	52	9 +	Nicotiana tabacum	defense and stress responsive

TaMAPK4	TGACG-motif	TGACG	581	5 -	Hordeum vulgare	the MeJA responsive
TaMAPK4	TGACG-motif	TGACG	1760	5 +	Hordeum vulgare	the MeJA responsive
TaMAPK4	ARE	AAACCA	50	6 -	Zea mays	anaerobic induction
TaMAPK4	ARE	AAACCA	257	6 -	Zea mays	anaerobic induction
TaMAPK4	ARE	AAACCA	1420	6 -	Zea mays	anaerobic induction
TaMAPK4	circadian	CAAAGATATC	494	9 +	Lycopersicon esculentum	circadian control
TaMAPK4	GARE-motif	TCTGTTG	548	7 -	Brassica oleracea	gibberellin responsive
TaMAPK4	GCN4_motif	TGAGTCA	1963	7 -	Oryza sativa	endosperm expression
TaMAPK4	O2-site	GATGACATGG	1923	9 -	Zea mays	zein metabolism regulation
TaMAPK4	LTR	CCGAAA	792	6 +	Hordeum vulgare	low temperature responsive
TaMAPK40	O2-site	GATGATGTGG	320	9 -	Zea mays	zein metabolism regulation
TaMAPK40	GC-motif	CCCCCG	1304	6 +	Zea mays	anoxic specific inducibility
TaMAPK40	MBS	CAACTG	870	6 -	Arabidopsis thaliana	drought inducibility
TaMAPK40	CGTCA-motif	CGTCA	134	5 -	Hordeum vulgare	the MeJA responsive
TaMAPK40	CGTCA-motif	CGTCA	1144	5 +	Hordeum vulgare	the MeJA responsive
TaMAPK40	LTR	CCGAAA	1165	6 +	Hordeum vulgare	low temperature responsive
TaMAPK40	ABRE	CACGTG	256	6 +	Arabidopsis thaliana	abscisic acid responsive
TaMAPK40	ABRE	ACGTG	257	5 +	Arabidopsis thaliana	abscisic acid responsive
TaMAPK40	ABRE	GCCGCGTGGC	741	9 +	Oryza sativa	abscisic acid responsive

TaMAPK40	ABRE	ACGTG	990	5 +	Arabidopsis thaliana	abscisic acid responsive
TaMAPK40	ABRE	GACACGTACG T	1086	10 -	Oryza sativa	abscisic acid responsive
TaMAPK40	ABRE	TACGTGTC	1089	8 +	Oryza sativa	abscisic acid responsive
TaMAPK40	ABRE	ACGTG	1090	5 +	Arabidopsis thaliana	abscisic acid responsive
TaMAPK40	ABRE	CACGTG	1218	6 -	Arabidopsis thaliana	abscisic acid responsive
TaMAPK40	ABRE	ACGTG	1219	5 +	Arabidopsis thaliana	abscisic acid responsive
TaMAPK40	ABRE	CACGTG	1340	6 -	Arabidopsis thaliana	abscisic acid responsive
TaMAPK40	ABRE	ACGTG	1341	5 +	Arabidopsis thaliana	abscisic acid responsive
TaMAPK40	GARE-motif	TCTGTTG	835	7 +	Brassica oleracea	gibberellin responsive
TaMAPK40	GARE-motif	TCTGTTG	1123	7 +	Brassica oleracea	gibberellin responsive
TaMAPK40	TGACG-motif	TGACG	134	5 +	Hordeum vulgare	the MeJA responsive
TaMAPK40	TGACG-motif	TGACG	1144	5 -	Hordeum vulgare	the MeJA responsive
TaMAPK41	P-box	CCTTTTG	971	7 -	Oryza sativa	gibberellin responsive
TaMAPK41	ABRE	ACGTG	1114	5 -	Arabidopsis thaliana	abscisic acid responsive
TaMAPK41	ABRE	ACGTG	1130	5 -	Arabidopsis thaliana	abscisic acid responsive
TaMAPK41	ABRE	ACGTG	1169	5 +	Arabidopsis thaliana	abscisic acid responsive
TaMAPK41	ABRE	ACGTG	1454	5 -	Arabidopsis thaliana	abscisic acid responsive
TaMAPK41	CAT-box	GCCACT	195	6 -	Arabidopsis thaliana	meristem expression
TaMAPK41	CAT-box	GCCACT	1563	6 -	Arabidopsis thaliana	meristem expression

TaMAPK41	RY-element	CATGCATG	962	8 +	Helianthus annuus	seed specific regulation
TaMAPK41	ARE	AAACCA	42	6 -	Zea mays	anaerobic induction
TaMAPK41	O2-site	GATGATGTGG	946	9 -	Zea mays	zein metabolism regulation
TaMAPK41	GARE-motif	TCTGTTG	580	7 +	Brassica oleracea	gibberellin responsive
TaMAPK42	TATC-box	TATCCCA	1446	7 -	Oryza sativa	gibberellin responsive
TaMAPK42	HD-Zip 1	CAAT(A/T)ATTG	1243	8 -	Arabidopsis thaliana	differentiation of the palisade mesophyll cells
TaMAPK42	TGACG-motif	TGACG	1757	5 -	Hordeum vulgare	the MeJA responsive
TaMAPK42	CGTCA-motif	CGTCA	1757	5 +	Hordeum vulgare	the MeJA responsive
TaMAPK42	P-box	CCTTTTG	1652	7 +	Oryza sativa	gibberellin responsive
TaMAPK42	ABRE	ACGTG	1737	5 +	Arabidopsis thaliana	abscisic acid responsive
TaMAPK42	ABRE	ACGTG	1809	5 -	Arabidopsis thaliana	abscisic acid responsive
TaMAPK43	GC-motif	CCCCCG	1106	6 -	Zea mays	anoxic specific inducibility
TaMAPK43	GC-motif	CCCCCG	1917	6 -	Zea mays	anoxic specific inducibility
TaMAPK43	ABRE	CGTACGTGCA	153	9 -	Hordeum vulgare	abscisic acid responsive
TaMAPK43	ABRE	ACGTG	155	5 -	Arabidopsis thaliana	abscisic acid responsive
TaMAPK43	ABRE	AACCCGG	956	7 +	Arabidopsis thaliana	abscisic acid responsive
TaMAPK43	ABRE	AACCCGG	1003	7 +	Arabidopsis thaliana	abscisic acid responsive
TaMAPK43	ABRE	ACGTG	1875	5 +	Arabidopsis thaliana	abscisic acid responsive

TaMAPK43	TC-rich repeats	GTTTTCTTAC	923	9 +	Nicotiana tabacum	defense and stress responsive
TaMAPK43	TGACG-motif	TGACG	394	5 -	Hordeum vulgare	the MeJA responsive
TaMAPK43	TGACG-motif	TGACG	778	5 +	Hordeum vulgare	the MeJA responsive
TaMAPK43	TGA-element	AACGAC	1387	6 +	Brassica oleracea	auxin responsive
TaMAPK43	CGTCA-motif	CGTCA	394	5 +	Hordeum vulgare	the MeJA responsive
TaMAPK43	CGTCA-motif	CGTCA	778	5 -	Hordeum vulgare	the MeJA responsive
TaMAPK43	P-box	CCTTTTG	657	7 -	Oryza sativa	gibberellin responsive
TaMAPK43	ARE	AAACCA	161	6 -	Zea mays	anaerobic induction
TaMAPK43	ARE	AAACCA	1068	6 +	Zea mays	anaerobic induction
TaMAPK43	ARE	AAACCA	1708	6 +	Zea mays	anaerobic induction
TaMAPK43	TATC-box	TATCCCA	994	7 -	Oryza sativa	gibberellin responsive
TaMAPK43	O2-site	GATGA(C/T)(A/G)TG(A/G)	49	8 +	Zea mays	zein metabolism regulation
TaMAPK44	CGTCA-motif	CGTCA	756	5 +	Hordeum vulgare	the MeJA responsive
TaMAPK44	CGTCA-motif	CGTCA	1698	5 +	Hordeum vulgare	the MeJA responsive
TaMAPK44	TGACG-motif	TGACG	756	5 -	Hordeum vulgare	the MeJA responsive
TaMAPK44	TGACG-motif	TGACG	1698	5 -	Hordeum vulgare	the MeJA responsive
TaMAPK44	LTR	CCGAAA	1898	6 +	Hordeum vulgare	low temperature responsive
TaMAPK44	TATC-box	TATCCCA	1372	7 +	Oryza sativa	gibberellin responsive
TaMAPK44	circadian	CAAAGATATC	295	9 -	Lycopersicon esculentum	circadian control

TaMAPK44	TCA-element	CCATCTTTTT	744	9 +	Nicotiana tabacum	salicylic acid responsive
TaMAPK44	P-box	CCTTTTG	1529	7 +	Oryza sativa	gibberellin responsive
TaMAPK44	ABRE	ACGTG	693	5 +	Arabidopsis thaliana	abscisic acid responsive
TaMAPK44	ABRE	ACGTG	1318	5 +	Arabidopsis thaliana	abscisic acid responsive
TaMAPK44	ABRE	ACGTG	1696	5 -	Arabidopsis thaliana	abscisic acid responsive
TaMAPK45	O2-site	GATGACATGG	1432	9 +	Zea mays	zein metabolism regulation
TaMAPK45	GC-motif	CCCCCG	1091	6 -	Zea mays	anoxic specific inducibility
TaMAPK45	ABRE	CGCACGTGTC	307	9 +	Hordeum vulgare	abscisic acid responsive
TaMAPK45	ABRE	CACGTG	309	6 +	Arabidopsis thaliana	abscisic acid responsive
TaMAPK45	ABRE	ACGTG	310	5 +	Arabidopsis thaliana	abscisic acid responsive
TaMAPK45	ARE	AAACCA	1176	6 -	Zea mays	anaerobic induction
TaMAPK45	MBS	CAACTG	1420	6 +	Arabidopsis thaliana	drought inducibility
TaMAPK45	GCN4_motif	TGAGTCA	126	7 -	Oryza sativa	endosperm expression
TaMAPK46	TC-rich repeats	GTTTTCTTAC	935	9 +	Nicotiana tabacum	defense and stress responsive
TaMAPK46	GCN4_motif	TGAGTCA	1739	7 -	Oryza sativa	endosperm expression
TaMAPK46	LTR	CCGAAA	41	6 +	Hordeum vulgare	low temperature responsive
TaMAPK46	MSA-like	TCCAACGGT	1786	9 +	Catharanthus roseus	cell cycle regulation
TaMAPK46	ARE	AAACCA	73	6 +	Zea mays	anaerobic induction
TaMAPK46	ARE	AAACCA	737	6 -	Zea mays	anaerobic induction

TaMAPK46	ARE	AAACCA	1570	6 -	Zea mays	anaerobic induction
TaMAPK46	ABRE	ACGTG	1	5 +	Arabidopsis thaliana	abscisic acid responsive
TaMAPK46	ABRE	ACGTG	116	5 -	Arabidopsis thaliana	abscisic acid responsive
TaMAPK46	ABRE	ACGTG	729	5 +	Arabidopsis thaliana	abscisic acid responsive
TaMAPK46	ABRE	ACGTG	1159	5 -	Arabidopsis thaliana	abscisic acid responsive
TaMAPK46	ABRE	ACGTG	1694	5 +	Arabidopsis thaliana	abscisic acid responsive
TaMAPK46	GC-motif	CCCCCG	433	6 -	Zea mays	anoxic specific inducibility
TaMAPK46	GC-motif	CCCCCG	534	6 -	Zea mays	anoxic specific inducibility
TaMAPK46	GC-motif	CCCCCG	616	6 -	Zea mays	anoxic specific inducibility
TaMAPK46	O2-site	GATGA(C/T)(A/G)TG(A/G)	949	8 +	Zea mays	zein metabolism regulation
TaMAPK46	O2-site	GATGACATGG	1215	9 -	Zea mays	zein metabolism regulation
TaMAPK46	TATC-box	TATCCCA	1154	7 +	Oryza sativa	gibberellin responsive
TaMAPK46	CGTCA-motif	CGTCA	1018	5 +	Hordeum vulgare	the MeJA responsive
TaMAPK46	CGTCA-motif	CGTCA	1230	5 +	Hordeum vulgare	the MeJA responsive
TaMAPK46	TGACG-motif	TGACG	1018	5 -	Hordeum vulgare	the MeJA responsive
TaMAPK46	TGACG-motif	TGACG	1230	5 -	Hordeum vulgare	the MeJA responsive
TaMAPK46	P-box	CCTTTTG	810	7 -	Oryza sativa	gibberellin responsive
TaMAPK47	LTR	CCGAAA	47	6 +	Hordeum vulgare	low temperature responsive
TaMAPK47	MBS	CAACTG	221	6 +	Arabidopsis thaliana	drought inducibility

TaMAPK47	ABRE	ACGTG	999	5 -	Arabidopsis thaliana	abscisic acid responsive
TaMAPK47	ABRE	GACACGTGGC	1950	9 -	Triticum aestivum	abscisic acid responsive
TaMAPK47	ABRE	CACGTG	1952	6 -	Arabidopsis thaliana	abscisic acid responsive
TaMAPK47	ABRE	ACGTG	1953	5 +	Arabidopsis thaliana	abscisic acid responsive
TaMAPK47	ARE	AAACCA	51	6 +	Zea mays	anaerobic induction
TaMAPK47	ARE	AAACCA	1207	6 +	Zea mays	anaerobic induction
TaMAPK47	TGACG-motif	TGACG	255	5 -	Hordeum vulgare	the MeJA responsive
TaMAPK47	TGACG-motif	TGACG	1031	5 +	Hordeum vulgare	the MeJA responsive
TaMAPK47	TGACG-motif	TGACG	1716	5 -	Hordeum vulgare	the MeJA responsive
TaMAPK47	TGACG-motif	TGACG	1766	5 -	Hordeum vulgare	the MeJA responsive
TaMAPK47	CGTCA-motif	CGTCA	255	5 +	Hordeum vulgare	the MeJA responsive
TaMAPK47	CGTCA-motif	CGTCA	1031	5 -	Hordeum vulgare	the MeJA responsive
TaMAPK47	CGTCA-motif	CGTCA	1716	5 +	Hordeum vulgare	the MeJA responsive
TaMAPK47	CGTCA-motif	CGTCA	1766	5 +	Hordeum vulgare	the MeJA responsive
TaMAPK47	TATC-box	TATCCCA	992	7 -	Oryza sativa	gibberellin responsive
TaMAPK49	GCN4_motif	TGAGTCA	73	7 +	Oryza sativa	endosperm expression
TaMAPK49	LTR	CCGAAA	562	6 +	Hordeum vulgare	low temperature responsive
TaMAPK49	LTR	CCGAAA	1030	6 +	Hordeum vulgare	low temperature responsive
TaMAPK49	NON-box	AGATCGACG	1455	9 +	Arabidopsis thaliana	meristem specific activation

TaMAPK49	CAT-box	GCCACT	1881	6 -	Arabidopsis thaliana	meristem expression
TaMAPK49	TCA-element	CCATCTTTTT	194	9 -	Nicotiana tabacum	salicylic acid responsive
TaMAPK49	TCA-element	CCATCTTTTT	777	9 -	Nicotiana tabacum	salicylic acid responsive
TaMAPK49	GC-motif	CCCCCG	1567	6 +	Zea mays	anoxic specific inducibility
TaMAPK49	O2-site	GATGACATGG	1021	9 -	Zea mays	zein metabolism regulation
TaMAPK49	ABRE	ACGTG	299	5 -	Arabidopsis thaliana	abscisic acid responsive
TaMAPK49	ABRE	ACGTG	638	5 -	Arabidopsis thaliana	abscisic acid responsive
TaMAPK49	P-box	CCTTTTG	253	7 -	Oryza sativa	gibberellin responsive
TaMAPK49	P-box	CCTTTTG	933	7 +	Oryza sativa	gibberellin responsive
TaMAPK49	TGACG-motif	TGACG	474	5 -	Hordeum vulgare	the MeJA responsive
TaMAPK49	TGACG-motif	TGACG	640	5 -	Hordeum vulgare	the MeJA responsive
TaMAPK49	TGACG-motif	TGACG	942	5 +	Hordeum vulgare	the MeJA responsive
TaMAPK49	TGACG-motif	TGACG	1297	5 +	Hordeum vulgare	the MeJA responsive
TaMAPK49	TGACG-motif	TGACG	1315	5 -	Hordeum vulgare	the MeJA responsive
TaMAPK49	TGACG-motif	TGACG	1629	5 -	Hordeum vulgare	the MeJA responsive
TaMAPK49	CGTCA-motif	CGTCA	474	5 +	Hordeum vulgare	the MeJA responsive
TaMAPK49	CGTCA-motif	CGTCA	640	5 +	Hordeum vulgare	the MeJA responsive
TaMAPK49	CGTCA-motif	CGTCA	942	5 -	Hordeum vulgare	the MeJA responsive
TaMAPK49	CGTCA-motif	CGTCA	1297	5 -	Hordeum vulgare	the MeJA responsive

TaMAPK49	CGTCA-motif	CGTCA	1315	5 +	Hordeum vulgare	the MeJA responsive
TaMAPK49	CGTCA-motif	CGTCA	1629	5 +	Hordeum vulgare	the MeJA responsive
TaMAPK5	LTR	CCGAAA	655	6 -	Hordeum vulgare	low temperature responsive
TaMAPK5	LTR	CCGAAA	737	6 -	Hordeum vulgare	low temperature responsive
TaMAPK5	LTR	CCGAAA	904	6 -	Hordeum vulgare	low temperature responsive
TaMAPK5	ARE	AAACCA	1031	6 +	Zea mays	anaerobic induction
TaMAPK5	ARE	AAACCA	1255	6 -	Zea mays	anaerobic induction
TaMAPK5	TGACG-motif	TGACG	797	5 +	Hordeum vulgare	the MeJA responsive
TaMAPK5	TGACG-motif	TGACG	1520	5 -	Hordeum vulgare	the MeJA responsive
TaMAPK5	MBSI	TTTTTACGGTTA	901	11 +	Petunia hybrida	flavonoid biosynthetic genes regulation
TaMAPK5	TGA-box	TGACGTAA	797	8 +	Glycine max	auxin responsive
TaMAPK5	ABRE	ACGTG	515	5 -	Arabidopsis thaliana	abscisic acid responsive
TaMAPK5	ABRE	ACGTG	691	5 -	Arabidopsis thaliana	abscisic acid responsive
TaMAPK5	ABRE	ACGTG	886	5 -	Arabidopsis thaliana	abscisic acid responsive
TaMAPK5	ABRE	CACGTG	1286	6 -	Arabidopsis thaliana	abscisic acid responsive
TaMAPK5	ABRE	ACGTG	1287	5 +	Arabidopsis thaliana	abscisic acid responsive
TaMAPK5	ABRE	ACGTG	1742	5 +	Arabidopsis thaliana	abscisic acid responsive
TaMAPK5	ABRE	ACGTG	1804	5 -	Arabidopsis thaliana	abscisic acid responsive
TaMAPK5	GC-motif	CCCCCG	1856	6 +	Zea mays	anoxic specific inducibility

TaMAPK5	CGTCA-motif	CGTCA	797	5 -	Hordeum vulgare	the MeJA responsive
TaMAPK5	CGTCA-motif	CGTCA	1520	5 +	Hordeum vulgare	the MeJA responsive
TaMAPK5	TGA-element	AACGAC	694	6 -	Brassica oleracea	auxin responsive
TaMAPK50	TC-rich repeats	GTTTTCTTAC	1945	9 +	Nicotiana tabacum	defense and stress responsive
TaMAPK50	TGACG-motif	TGACG	41	5 -	Hordeum vulgare	the MeJA responsive
TaMAPK50	TGACG-motif	TGACG	1904	5 -	Hordeum vulgare	the MeJA responsive
TaMAPK50	CGTCA-motif	CGTCA	41	5 +	Hordeum vulgare	the MeJA responsive
TaMAPK50	CGTCA-motif	CGTCA	1904	5 +	Hordeum vulgare	the MeJA responsive
TaMAPK50	LTR	CCGAAA	197	6 +	Hordeum vulgare	low temperature responsive
TaMAPK50	LTR	CCGAAA	844	6 -	Hordeum vulgare	low temperature responsive
TaMAPK50	LTR	CCGAAA	997	6 -	Hordeum vulgare	low temperature responsive
TaMAPK50	CAT-box	GCCACT	1415	6 +	Arabidopsis thaliana	meristem expression
TaMAPK50	GARE-motif	TCTGTTG	1494	7 +	Brassica oleracea	gibberellin responsive
TaMAPK50	MBS	CAACTG	1807	6 -	Arabidopsis thaliana	drought inducibility
TaMAPK50	TCA-element	TCAGAAGAGG	1062	9 -	Brassica oleracea	salicylic acid responsive
TaMAPK50	TCA-element	CCATCTTTTT	1424	9 +	Nicotiana tabacum	salicylic acid responsive
TaMAPK50	ABRE	CACGTG	1450	6 -	Arabidopsis thaliana	abscisic acid responsive
TaMAPK50	ABRE	ACGTG	1451	5 +	Arabidopsis thaliana	abscisic acid responsive
TaMAPK50	ABRE	ACGTG	1712	5 +	Arabidopsis thaliana	abscisic acid responsive
TaMAPK50	TGA-element	AACGAC	1264	6 +	Brassica oleracea	auxin responsive

TaMAPK50	TGA-element	AACGAC	1994	6 +	Brassica oleracea	auxin responsive
TaMAPK50	GC-motif	CCCCCG	214	6 +	Zea mays	anoxic specific inducibility
TaMAPK50	GC-motif	CCCCCG	768	6 -	Zea mays	anoxic specific inducibility
TaMAPK50	GC-motif	CCCCCG	1849	6 +	Zea mays	anoxic specific inducibility
TaMAPK52	LTR	CCGAAA	1147	6 +	Hordeum vulgare	low temperature responsive
TaMAPK52	GCN4_motif	TGAGTCA	276	7 +	Oryza sativa	endosperm expression
TaMAPK52	MBS	CAACTG	95	6 +	Arabidopsis thaliana	drought inducibility
TaMAPK52	ABRE	TACGGTC	1238	7 +	Arabidopsis thaliana	abscisic acid responsive
TaMAPK52	ABRE	ACGTG	1437	5 +	Arabidopsis thaliana	abscisic acid responsive
TaMAPK52	ABRE	AACCCGG	1767	7 +	Arabidopsis thaliana	abscisic acid responsive
TaMAPK52	ABRE	ACGTG	1796	5 -	Arabidopsis thaliana	abscisic acid responsive
TaMAPK52	ARE	AAACCA	657	6 -	Zea mays	anaerobic induction
TaMAPK52	O2-site	GATGACATGG	428	9 -	Zea mays	zein metabolism regulation
TaMAPK52	O2-site	GATGACATGG	1460	9 -	Zea mays	zein metabolism regulation
TaMAPK52	O2-site	GATGATGTGG	1630	9 -	Zea mays	zein metabolism regulation
TaMAPK52	CGTCA-motif	CGTCA	1428	5 +	Hordeum vulgare	the MeJA responsive
TaMAPK52	CGTCA-motif	CGTCA	1636	5 +	Hordeum vulgare	the MeJA responsive
TaMAPK52	GARE-motif	TCTGTTG	1166	7 -	Brassica oleracea	gibberellin responsive
TaMAPK52	CAT-box	GCCACT	360	6 +	Arabidopsis thaliana	meristem expression
TaMAPK52	TGA-element	AACGAC	1833	6 +	Brassica oleracea	auxin responsive

TaMAPK52	GC-motif	CCCCCG	415	6 +	Zea mays	anoxic specific inducibility
TaMAPK52	GC-motif	CCCCCG	1386	6 -	Zea mays	anoxic specific inducibility
TaMAPK52	TGACG-motif	TGACG	1428	5 -	Hordeum vulgare	the MeJA responsive
TaMAPK52	TGACG-motif	TGACG	1636	5 -	Hordeum vulgare	the MeJA responsive
TaMAPK53	GCN4_motif	TGAGTCA	768	7 +	Oryza sativa	endosperm expression
TaMAPK53	LTR	CCGAAA	1715	6 +	Hordeum vulgare	low temperature responsive
TaMAPK53	TC-rich repeats	GTTTTCTTAC	745	9 -	Nicotiana tabacum	defense and stress responsive
TaMAPK53	CAT-box	GCCACT	323	6 +	Arabidopsis thaliana	meristem expression
TaMAPK53	CAT-box	GCCACT	1881	6 -	Arabidopsis thaliana	meristem expression
TaMAPK53	O2-site	GATGATGTGG	974	10 +	Zea mays	zein metabolism regulation
TaMAPK53	ABRE	ACGTG	955	5 -	Arabidopsis thaliana	abscisic acid responsive
TaMAPK53	P-box	CCTTTTG	226	7 -	Oryza sativa	gibberellin responsive
TaMAPK53	CGTCA-motif	CGTCA	1629	5 +	Hordeum vulgare	the MeJA responsive
TaMAPK53	TGACG-motif	TGACG	1629	5 -	Hordeum vulgare	the MeJA responsive
TaMAPK54	GC-motif	CCCCCG	827	6 -	Zea mays	anoxic specific inducibility
TaMAPK54	GC-motif	CCCCCG	1281	6 +	Zea mays	anoxic specific inducibility
TaMAPK54	GC-motif	CCCCCG	1378	6 -	Zea mays	anoxic specific inducibility
TaMAPK54	O2-site	GATGACATGG	596	9 -	Zea mays	zein metabolism regulation
TaMAPK54	O2-site	GATGATGTGG	1032	9 -	Zea mays	zein metabolism regulation

TaMAPK54	ARE	AAACCA	232	6 +	Zea mays	anaerobic induction
TaMAPK54	ARE	AAACCA	584	6 +	Zea mays	anaerobic induction
TaMAPK54	TGA-element	AACGAC	713	6 +	Brassica oleracea	auxin responsive
TaMAPK54	ABRE	CGCACGTGTC	287	9 -	Hordeum vulgare	abscisic acid responsive
TaMAPK54	ABRE	ACGTG	430	5 +	Arabidopsis thaliana	abscisic acid responsive
TaMAPK54	ABRE	ACGTG	618	5 -	Arabidopsis thaliana	abscisic acid responsive
TaMAPK54	ABRE	CACGTG	847	6 +	Arabidopsis thaliana	abscisic acid responsive
TaMAPK54	ABRE	ACGTG	848	5 +	Arabidopsis thaliana	abscisic acid responsive
TaMAPK54	ABRE	GACACGTGGC	963	9 -	Triticum aestivum	abscisic acid responsive
TaMAPK54	ABRE	ACGTG	1154	5 +	Arabidopsis thaliana	abscisic acid responsive
TaMAPK54	ABRE	ACGTG	1294	5 +	Arabidopsis thaliana	abscisic acid responsive
TaMAPK54	RY-element	CATGCATG	236	8 +	Helianthus annuus	seed specific regulation
TaMAPK54	RY-element	CATGCATG	588	8 +	Helianthus annuus	seed specific regulation
TaMAPK54	TGACG-motif	TGACG	611	5 -	Hordeum vulgare	the MeJA responsive
TaMAPK54	TGACG-motif	TGACG	1124	5 -	Hordeum vulgare	the MeJA responsive
TaMAPK54	CGTCA-motif	CGTCA	611	5 +	Hordeum vulgare	the MeJA responsive
TaMAPK54	CGTCA-motif	CGTCA	1124	5 +	Hordeum vulgare	the MeJA responsive
TaMAPK54	GCN4_motif	TGAGTCA	514	7 -	Oryza sativa	endosperm expression
TaMAPK54	LTR	CCGAAA	1193	6 +	Hordeum vulgare	low temperature responsive

TaMAPK54	WUN-motif	AAATTTCCT	299	9 -	Brassica oleracea	wound responsive
TaMAPK6	GARE-motif	TCTGTTG	1314	7 -	Brassica oleracea	gibberellin responsive
TaMAPK6	O2-site	GATGACATGG	707	9 +	Zea mays	zein metabolism regulation
TaMAPK6	TGACG-motif	TGACG	456	5 -	Hordeum vulgare	the MeJA responsive
TaMAPK6	TGACG-motif	TGACG	653	5 +	Hordeum vulgare	the MeJA responsive
TaMAPK6	TGACG-motif	TGACG	736	5 -	Hordeum vulgare	the MeJA responsive
TaMAPK6	TGACG-motif	TGACG	1875	5 -	Hordeum vulgare	the MeJA responsive
TaMAPK6	ARE	AAACCA	1669	6 +	Zea mays	anaerobic induction
TaMAPK6	RY-element	CATGCATG	819	8 +	Helianthus annuus	seed specific regulation
TaMAPK6	MBS	CAACTG	199	6 +	Arabidopsis thaliana	drought inducibility
TaMAPK6	CGTCA-motif	CGTCA	456	5 +	Hordeum vulgare	the MeJA responsive
TaMAPK6	CGTCA-motif	CGTCA	653	5 -	Hordeum vulgare	the MeJA responsive
TaMAPK6	CGTCA-motif	CGTCA	736	5 +	Hordeum vulgare	the MeJA responsive
TaMAPK6	CGTCA-motif	CGTCA	1875	5 +	Hordeum vulgare	the MeJA responsive
TaMAPK6	TGA-element	AACGAC	1370	6 +	Brassica oleracea	auxin responsive
TaMAPK6	ABRE	TACGGTC	1373	7 -	Arabidopsis thaliana	abscisic acid responsive
TaMAPK6	GC-motif	CCCCCG	1361	6 +	Zea mays	anoxic specific inducibility
TaMAPK7	CAT-box	GCCACT	476	6 -	Arabidopsis thaliana	meristem expression
TaMAPK7	CAT-box	GCCACT	499	6 -	Arabidopsis thaliana	meristem expression

TaMAPK7	CAT-box	GCCACT	570	6 -	Arabidopsis thaliana	meristem expression
TaMAPK7	CAT-box	GCCACT	1094	6 +	Arabidopsis thaliana	meristem expression
TaMAPK7	AuxRR-core	GGTCCAT	748	7 +	Nicotiana tabacum	auxin responsive
TaMAPK7	LTR	CCGAAA	971	6 +	Hordeum vulgare	low temperature responsive
TaMAPK7	TGACG-motif	TGACG	1638	5 -	Hordeum vulgare	the MeJA responsive
TaMAPK7	CGTCA-motif	CGTCA	1638	5 +	Hordeum vulgare	the MeJA responsive
TaMAPK7	TGA-element	AACGAC	1068	6 +	Brassica oleracea	auxin responsive
TaMAPK7	TGA-element	AACGAC	1835	6 +	Brassica oleracea	auxin responsive
TaMAPK7	ABRE	ACGTG	195	5 +	Arabidopsis thaliana	abscisic acid responsive
TaMAPK7	ABRE	AACCCGG	1769	7 +	Arabidopsis thaliana	abscisic acid responsive
TaMAPK7	ABRE	ACGTG	1798	5 -	Arabidopsis thaliana	abscisic acid responsive
TaMAPK7	ARE	AAACCA	1350	6 +	Zea mays	anaerobic induction
TaMAPK7	ARE	AAACCA	1404	6 -	Zea mays	anaerobic induction
TaMAPK7	O2-site	GATGATGTGG	1632	9 -	Zea mays	zein metabolism regulation
TaMAPK7	GC-motif	CCCCCG	1228	6 +	Zea mays	anoxic specific inducibility
TaMAPK7	GC-motif	CCCCCG	1239	6 +	Zea mays	anoxic specific inducibility
TaMAPK8	TGACG-motif	TGACG	1359	5 -	Hordeum vulgare	the MeJA responsive
TaMAPK8	circadian	CAAAGATATC	522	9 +	Lycopersicon esculentum	circadian control
TaMAPK8	ARE	AAACCA	1289	6 +	Zea mays	anaerobic induction
TaMAPK8	ABRE	ACGTG	65	5 +	Arabidopsis thaliana	abscisic acid responsive

TaMAPK8	ABRE	ACGTG	107	5 -	Arabidopsis thaliana	abscisic acid responsive
TaMAPK8	ABRE	ACGTG	156	5 -	Arabidopsis thaliana	abscisic acid responsive
TaMAPK8	ABRE	ACGTG	172	5 -	Arabidopsis thaliana	abscisic acid responsive
TaMAPK8	ABRE	CACGTG	885	6 +	Arabidopsis thaliana	abscisic acid responsive
TaMAPK8	ABRE	ACGTG	886	5 +	Arabidopsis thaliana	abscisic acid responsive
TaMAPK8	ABRE	GCCGCGTGGC	1485	9 -	Oryza sativa	abscisic acid responsive
TaMAPK8	CGTCA-motif	CGTCA	1359	5 +	Hordeum vulgare	the MeJA responsive
TaMAPK8	LTR	CCGAAA	798	6 -	Hordeum vulgare	low temperature responsive
TaMAPK8	LTR	CCGAAA	1005	6 -	Hordeum vulgare	low temperature responsive
TaMAPK8	LTR	CCGAAA	1850	6 -	Hordeum vulgare	low temperature responsive
TaMAPK8	P-box	CCTTTTG	238	7 -	Oryza sativa	gibberellin responsive
TaMAPK8	P-box	CCTTTTG	1316	7 -	Oryza sativa	gibberellin responsive
TaMAPK8	P-box	TTCCAACAAA CCCC	1468	13 +	Petroselinum crispum	gibberellin responsive
TaMAPK8	GC-motif	CCCCCG	1770	6 +	Zea mays	anoxic specific inducibility
TaMAPK8	GC-motif	CCCCCG	1956	6 -	Zea mays	anoxic specific inducibility
TaMAPK8	TC-rich repeats	GTTTTCTTAC	462	9 +	Nicotiana tabacum	defense and stress responsive
TaMAPKK1	LTR	CCGAAA	1978	6 -	Hordeum vulgare	low temperature responsive
TaMAPKK1	ABRE	GACACGTACG T	14	10 -	Oryza sativa	abscisic acid responsive
TaMAPKK1	ABRE	CACGTG	400	6 +	Arabidopsis thaliana	abscisic acid responsive

TaMAPKK1	ABRE	ACGTG	401	5 +	Arabidopsis thaliana	abscisic acid responsive
TaMAPKK1	ABRE	CGCACGTGTC	679	9 +	Hordeum vulgare	abscisic acid responsive
TaMAPKK1	ABRE	ACGTG	681	5 -	Arabidopsis thaliana	abscisic acid responsive
TaMAPKK1	ABRE	CGCACGTGTC	1037	9 -	Hordeum vulgare	abscisic acid responsive
TaMAPKK1	ABRE	CACGTG	1039	6 -	Arabidopsis thaliana	abscisic acid responsive
TaMAPKK1	ABRE	ACGTG	1040	5 +	Arabidopsis thaliana	abscisic acid responsive
TaMAPKK1	ABRE	ACGTG	1160	5 +	Arabidopsis thaliana	abscisic acid responsive
TaMAPKK1	ABRE	CACGTG	1486	6 -	Arabidopsis thaliana	abscisic acid responsive
TaMAPKK1	ABRE	ACGTG	1487	5 +	Arabidopsis thaliana	abscisic acid responsive
TaMAPKK1	TGA-element	AACGAC	959	6 +	Brassica oleracea	auxin responsive
TaMAPKK1	ARE	AAACCA	1024	6 -	Zea mays	anaerobic induction
TaMAPKK1	GC-motif	CCCCCG	1665	6 -	Zea mays	anoxic specific inducibility
TaMAPKK1	GC-motif	CCCCCG	1728	6 +	Zea mays	anoxic specific inducibility
TaMAPKK1	CGTCA-motif	CGTCA	228	5 +	Hordeum vulgare	the MeJA responsive
TaMAPKK1	CGTCA-motif	CGTCA	492	5 -	Hordeum vulgare	the MeJA responsive
TaMAPKK1	CGTCA-motif	CGTCA	1172	5 +	Hordeum vulgare	the MeJA responsive
TaMAPKK1	CGTCA-motif	CGTCA	1278	5 +	Hordeum vulgare	the MeJA responsive
TaMAPKK1	CGTCA-motif	CGTCA	1735	5 +	Hordeum vulgare	the MeJA responsive
TaMAPKK1	TGACG-motif	TGACG	228	5 -	Hordeum vulgare	the MeJA responsive

TaMAPKK1	TGACG-motif	TGACG	492	5 +	Hordeum vulgare	the MeJA responsive
TaMAPKK1	TGACG-motif	TGACG	1172	5 -	Hordeum vulgare	the MeJA responsive
TaMAPKK1	TGACG-motif	TGACG	1278	5 -	Hordeum vulgare	the MeJA responsive
TaMAPKK1	TGACG-motif	TGACG	1735	5 -	Hordeum vulgare	the MeJA responsive
TaMAPKK11	CAT-box	GCCACT	42	6 -	Arabidopsis thaliana	meristem expression
TaMAPKK11	O2-site	GATGACATGG	570	10 -	Zea mays	zein metabolism regulation
TaMAPKK11	TGACG-motif	TGACG	26	5 -	Hordeum vulgare	the MeJA responsive
TaMAPKK11	TGACG-motif	TGACG	423	5 -	Hordeum vulgare	the MeJA responsive
TaMAPKK11	TGACG-motif	TGACG	1271	5 +	Hordeum vulgare	the MeJA responsive
TaMAPKK11	TGACG-motif	TGACG	1274	5 -	Hordeum vulgare	the MeJA responsive
TaMAPKK11	TGACG-motif	TGACG	1325	5 +	Hordeum vulgare	the MeJA responsive
TaMAPKK11	TGACG-motif	TGACG	1827	5 -	Hordeum vulgare	the MeJA responsive
TaMAPKK11	GC-motif	CCCCCG	1901	6 +	Zea mays	anoxic specific inducibility
TaMAPKK11	ARE	AAACCA	1084	6 +	Zea mays	anaerobic induction
TaMAPKK11	ARE	AAACCA	1639	6 -	Zea mays	anaerobic induction
TaMAPKK11	TATC-box	TATCCCA	1473	7 +	Oryza sativa	gibberellin responsive
TaMAPKK11	TCA-element	TCAGAAGAGG	774	9 -	Brassica oleracea	salicylic acid responsive
TaMAPKK11	LTR	CCGAAA	1904	6 +	Hordeum vulgare	low temperature responsive
TaMAPKK11	CGTCA-motif	CGTCA	26	5 +	Hordeum vulgare	the MeJA responsive

TaMAPKK11	CGTCA-motif	CGTCA	423	5 +	Hordeum vulgare	the MeJA responsive
TaMAPKK11	CGTCA-motif	CGTCA	1271	5 -	Hordeum vulgare	the MeJA responsive
TaMAPKK11	CGTCA-motif	CGTCA	1274	5 +	Hordeum vulgare	the MeJA responsive
TaMAPKK11	CGTCA-motif	CGTCA	1325	5 -	Hordeum vulgare	the MeJA responsive
TaMAPKK11	CGTCA-motif	CGTCA	1827	5 +	Hordeum vulgare	the MeJA responsive
TaMAPKK12	ARE	AAACCA	77	6 -	Zea mays	anaerobic induction
TaMAPKK12	TGACG-motif	TGACG	194	5 +	Hordeum vulgare	the MeJA responsive
TaMAPKK12	TGACG-motif	TGACG	1186	5 -	Hordeum vulgare	the MeJA responsive
TaMAPKK12	TGACG-motif	TGACG	1234	5 -	Hordeum vulgare	the MeJA responsive
TaMAPKK12	TGACG-motif	TGACG	1548	5 +	Hordeum vulgare	the MeJA responsive
TaMAPKK12	GARE-motif	TCTGTTG	1344	7 -	Brassica oleracea	gibberellin responsive
TaMAPKK12	LTR	CCGAAA	1291	6 -	Hordeum vulgare	low temperature responsive
TaMAPKK12	CGTCA-motif	CGTCA	194	5 -	Hordeum vulgare	the MeJA responsive
TaMAPKK12	CGTCA-motif	CGTCA	1186	5 +	Hordeum vulgare	the MeJA responsive
TaMAPKK12	CGTCA-motif	CGTCA	1234	5 +	Hordeum vulgare	the MeJA responsive
TaMAPKK12	CGTCA-motif	CGTCA	1548	5 -	Hordeum vulgare	the MeJA responsive
TaMAPKK12	AuxRR-core	GGTCCAT	261	7 -	Nicotiana tabacum	auxin responsive
TaMAPKK12	AuxRR-core	GGTCCAT	819	7 -	Nicotiana tabacum	auxin responsive
TaMAPKK12	ABRE	ACGTG	292	5 +	Arabidopsis thaliana	abscisic acid responsive

TaMAPKK12	ABRE	ACGTG	316	5 +	Arabidopsis thaliana	abscisic acid responsive
TaMAPKK12	ABRE	ACGTG	641	5 +	Arabidopsis thaliana	abscisic acid responsive
TaMAPKK12	ABRE	ACGTG	1472	5 -	Arabidopsis thaliana	abscisic acid responsive
TaMAPKK12	ABRE	ACGTG	1484	5 -	Arabidopsis thaliana	abscisic acid responsive
TaMAPKK12	ABRE	ACGTG	1626	5 -	Arabidopsis thaliana	abscisic acid responsive
TaMAPKK12	ABRE	ACGTG	1905	5 -	Arabidopsis thaliana	abscisic acid responsive
TaMAPKK13	MBS	CAACTG	1643	6 -	Arabidopsis thaliana	drought inducibility
TaMAPKK13	MBS	CAACTG	1804	6 +	Arabidopsis thaliana	drought inducibility
TaMAPKK13	ARE	AAACCA	846	6 +	Zea mays	anaerobic induction
TaMAPKK13	ARE	AAACCA	873	6 +	Zea mays	anaerobic induction
TaMAPKK13	ARE	AAACCA	1179	6 -	Zea mays	anaerobic induction
TaMAPKK13	TGACG-motif	TGACG	138	5 -	Hordeum vulgare	the MeJA responsive
TaMAPKK13	TGACG-motif	TGACG	1037	5 -	Hordeum vulgare	the MeJA responsive
TaMAPKK13	LTR	CCGAAA	767	6 +	Hordeum vulgare	low temperature responsive
TaMAPKK13	LTR	CCGAAA	900	6 -	Hordeum vulgare	low temperature responsive
TaMAPKK13	ABRE	AACCCGG	145	7 -	Arabidopsis thaliana	abscisic acid responsive
TaMAPKK13	ABRE	CACGTG	877	6 +	Arabidopsis thaliana	abscisic acid responsive
TaMAPKK13	ABRE	ACGTG	878	5 +	Arabidopsis thaliana	abscisic acid responsive
TaMAPKK13	ABRE	GCCGCGTGGC	1470	9 -	Oryza sativa	abscisic acid responsive

TaMAPKK13	GC-motif	CCCCCG	1400	6 +	Zea mays	anoxic specific inducibility
TaMAPKK13	GC-motif	CCCCCG	1437	6 +	Zea mays	anoxic specific inducibility
TaMAPKK13	GARE-motif	TCTGTTG	1709	7 +	Brassica oleracea	gibberellin responsive
TaMAPKK13	circadian	CAAAGATATC	1248	10 -	Lycopersicon esculentum	circadian control
TaMAPKK13	CAT-box	GCCACT	1374	6 -	Arabidopsis thaliana	meristem expression
TaMAPKK13	O2-site	GATGACATGG	1634	9 -	Zea mays	zein metabolism regulation
TaMAPKK13	TC-rich repeats	ATTCTCTAAC	1675	10 -	Nicotiana tabacum	defense and stress responsive
TaMAPKK13	CGTCA-motif	CGTCA	138	5 +	Hordeum vulgare	the MeJA responsive
TaMAPKK13	CGTCA-motif	CGTCA	1037	5 +	Hordeum vulgare	the MeJA responsive
TaMAPKK14	TCA-element	TCAGAAGAGG	271	9 +	Brassica oleracea	salicylic acid responsive
TaMAPKK14	TCA-element	CCATCTTTTT	552	9 +	Nicotiana tabacum	salicylic acid responsive
TaMAPKK14	LTR	CCGAAA	118	6 +	Hordeum vulgare	low temperature responsive
TaMAPKK14	CGTCA-motif	CGTCA	1349	5 -	Hordeum vulgare	the MeJA responsive
TaMAPKK14	ABRE	ACGTG	196	5 -	Arabidopsis thaliana	abscisic acid responsive
TaMAPKK14	ARE	AAACCA	181	6 +	Zea mays	anaerobic induction
TaMAPKK14	ARE	AAACCA	1452	6 +	Zea mays	anaerobic induction
TaMAPKK14	TGACG-motif	TGACG	1349	5 +	Hordeum vulgare	the MeJA responsive
TaMAPKK14	O2-site	GATGATGTGG	99	9 +	Zea mays	zein metabolism regulation
TaMAPKK14	O2-site	GATGATGTGG	698	9 -	Zea mays	zein metabolism regulation

TaMAPKK15	ABRE	ACGTG	170	5 -	Arabidopsis thaliana	abscisic acid responsive
TaMAPKK15	LTR	CCGAAA	92	6 +	Hordeum vulgare	low temperature responsive
TaMAPKK15	CGTCA-motif	CGTCA	1342	5 -	Hordeum vulgare	the MeJA responsive
TaMAPKK15	TCA-element	TCAGAAGAGG	245	9 +	Brassica oleracea	salicylic acid responsive
TaMAPKK15	TCA-element	CCATCTTTTT	539	9 +	Nicotiana tabacum	salicylic acid responsive
TaMAPKK15	GC-motif	CCCCCG	1679	6 -	Zea mays	anoxic specific inducibility
TaMAPKK15	ARE	AAACCA	155	6 +	Zea mays	anaerobic induction
TaMAPKK15	ARE	AAACCA	1443	6 +	Zea mays	anaerobic induction
TaMAPKK15	MBS	CAACTG	1186	6 -	Arabidopsis thaliana	drought inducibility
TaMAPKK15	O2-site	GATGATGTGG	685	9 -	Zea mays	zein metabolism regulation
TaMAPKK15	TGACG-motif	TGACG	1342	5 +	Hordeum vulgare	the MeJA responsive
TaMAPKK16	GC-motif	CCCCCG	1899	6 +	Zea mays	anoxic specific inducibility
TaMAPKK16	ARE	AAACCA	1401	6 -	Zea mays	anaerobic induction
TaMAPKK16	TGACG-motif	TGACG	839	5 -	Hordeum vulgare	the MeJA responsive
TaMAPKK16	TGACG-motif	TGACG	1835	5 -	Hordeum vulgare	the MeJA responsive
TaMAPKK16	O2-site	GATGACATGG	1022	9 -	Zea mays	zein metabolism regulation
TaMAPKK16	P-box	CCTTTTG	65	7 +	Oryza sativa	gibberellin responsive
TaMAPKK16	P-box	CCTTTTG	459	7 +	Oryza sativa	gibberellin responsive
TaMAPKK16	CAT-box	GCCACT	1149	6 +	Arabidopsis thaliana	meristem expression

TaMAPKK16	ABRE	AACCCGG	1477	7 -	Arabidopsis thaliana	abscisic acid responsive
TaMAPKK16	CGTCA-motif	CGTCA	839	5 +	Hordeum vulgare	the MeJA responsive
TaMAPKK16	CGTCA-motif	CGTCA	1835	5 +	Hordeum vulgare	the MeJA responsive
TaMAPKK16	LTR	CCGAAA	123	6 +	Hordeum vulgare	low temperature responsive
TaMAPKK16	LTR	CCGAAA	690	6 +	Hordeum vulgare	low temperature responsive
TaMAPKK16	LTR	CCGAAA	888	6 +	Hordeum vulgare	low temperature responsive
TaMAPKK16	LTR	CCGAAA	1135	6 +	Hordeum vulgare	low temperature responsive
TaMAPKK16	LTR	CCGAAA	1902	6 +	Hordeum vulgare	low temperature responsive
TaMAPKK16	TATC-box	TATCCCA	1539	7 +	Oryza sativa	gibberellin responsive
TaMAPKK16	circadian	CAAAGATATC	1592	9 +	Lycopersicon esculentum	circadian control
TaMAPKK17	circadian	CAAAGATATC	1814	9 -	Lycopersicon esculentum	circadian control
TaMAPKK17	TGA-element	AACGAC	1405	6 +	Brassica oleracea	auxin responsive
TaMAPKK17	AuxRR-core	GGTCCAT	168	7 -	Nicotiana tabacum	auxin responsive
TaMAPKK17	CGTCA-motif	CGTCA	101	5 -	Hordeum vulgare	the MeJA responsive
TaMAPKK17	CGTCA-motif	CGTCA	1463	5 -	Hordeum vulgare	the MeJA responsive
TaMAPKK17	LTR	CCGAAA	1289	6 -	Hordeum vulgare	low temperature responsive
TaMAPKK17	ABRE	ACGTG	199	5 +	Arabidopsis thaliana	abscisic acid responsive
TaMAPKK17	ABRE	ACGTG	223	5 +	Arabidopsis thaliana	abscisic acid responsive
TaMAPKK17	ABRE	GCAACGTGTC	1469	9 -	Hordeum vulgare	abscisic acid responsive

TaMAPKK17	ABRE	ACGTG	1471	5 -	Arabidopsis thaliana	abscisic acid responsive
TaMAPKK17	P-box	CCTTTTG	563	7 +	Oryza sativa	gibberellin responsive
TaMAPKK17	P-box	CCTTTTG	1333	7 +	Oryza sativa	gibberellin responsive
TaMAPKK17	TGACG-motif	TGACG	101	5 +	Hordeum vulgare	the MeJA responsive
TaMAPKK17	TGACG-motif	TGACG	1463	5 +	Hordeum vulgare	the MeJA responsive
TaMAPKK17	TC-rich repeats	GTTTTCTTAC	1157	9 +	Nicotiana tabacum	defense and stress responsive
TaMAPKK17	GARE-motif	TCTGTTG	1343	7 -	Brassica oleracea	gibberellin responsive
TaMAPKK17	ARE	AAACCA	551	6 -	Zea mays	anaerobic induction
TaMAPKK17	ARE	AAACCA	926	6 -	Zea mays	anaerobic induction
TaMAPKK17	ARE	AAACCA	1001	6 -	Zea mays	anaerobic induction
TaMAPKK17	ARE	AAACCA	1083	6 -	Zea mays	anaerobic induction
TaMAPKK17	ARE	AAACCA	1118	6 -	Zea mays	anaerobic induction
TaMAPKK17	ARE	AAACCA	1179	6 -	Zea mays	anaerobic induction
TaMAPKK18	TGACG-motif	TGACG	674	5 -	Hordeum vulgare	the MeJA responsive
TaMAPKK18	TGACG-motif	TGACG	923	5 -	Hordeum vulgare	the MeJA responsive
TaMAPKK18	TGACG-motif	TGACG	1090	5 +	Hordeum vulgare	the MeJA responsive
TaMAPKK18	TGACG-motif	TGACG	1378	5 -	Hordeum vulgare	the MeJA responsive
TaMAPKK18	TGACG-motif	TGACG	1603	5 +	Hordeum vulgare	the MeJA responsive
TaMAPKK18	CGTCA-motif	CGTCA	674	5 +	Hordeum vulgare	the MeJA responsive

TaMAPKK18	CGTCA-motif	CGTCA	923	5 +	Hordeum vulgare	the MeJA responsive
TaMAPKK18	CGTCA-motif	CGTCA	1090	5 -	Hordeum vulgare	the MeJA responsive
TaMAPKK18	CGTCA-motif	CGTCA	1378	5 +	Hordeum vulgare	the MeJA responsive
TaMAPKK18	CGTCA-motif	CGTCA	1603	5 -	Hordeum vulgare	the MeJA responsive
TaMAPKK18	LTR	CCGAAA	1703	6 -	Hordeum vulgare	low temperature responsive
TaMAPKK18	LTR	CCGAAA	1755	6 -	Hordeum vulgare	low temperature responsive
TaMAPKK18	CAT-box	GCCACT	297	6 -	Arabidopsis thaliana	meristem expression
TaMAPKK18	ARE	AAACCA	528	6 +	Zea mays	anaerobic induction
TaMAPKK18	TCA-element	CCATCTTTTT	212	9 +	Nicotiana tabacum	salicylic acid responsive
TaMAPKK18	P-box	CCTTTTG	216	7 +	Oryza sativa	gibberellin responsive
TaMAPKK18	ABRE	ACGTG	1254	5 +	Arabidopsis thaliana	abscisic acid responsive
TaMAPKK18	ABRE	CGCACGTGTC	1406	9 +	Hordeum vulgare	abscisic acid responsive
TaMAPKK18	ABRE	AACCCGG	1673	7 -	Arabidopsis thaliana	abscisic acid responsive
TaMAPKK18	GC-motif	CCCCCG	700	6 +	Zea mays	anoxic specific inducibility
TaMAPKK18	GC-motif	CCCCCG	737	6 +	Zea mays	anoxic specific inducibility
TaMAPKK18	GC-motif	CCCCCG	767	6 -	Zea mays	anoxic specific inducibility
TaMAPKK18	GC-motif	CCCCCG	1133	6 +	Zea mays	anoxic specific inducibility
TaMAPKK18	GC-motif	CCCCCG	1652	6 +	Zea mays	anoxic specific inducibility
TaMAPKK18	TGA-element	AACGAC	27	6 -	Brassica oleracea	auxin responsive
TaMAPKK18	TGA-element	AACGAC	1333	6 +	Brassica oleracea	auxin responsive

TaMAPKK2	TGA-element	AACGAC	1942	6 -	Brassica oleracea	auxin responsive
TaMAPKK2	ABRE	ACGTG	153	5 -	Arabidopsis thaliana	abscisic acid responsive
TaMAPKK2	ABRE	GACACGTGGC	618	9 -	Triticum aestivum	abscisic acid responsive
TaMAPKK2	ABRE	CACGTG	620	6 +	Arabidopsis thaliana	abscisic acid responsive
TaMAPKK2	ABRE	ACGTG	621	5 +	Arabidopsis thaliana	abscisic acid responsive
TaMAPKK2	ABRE	ACGTG	665	5 -	Arabidopsis thaliana	abscisic acid responsive
TaMAPKK2	ABRE	CACGTG	927	6 +	Arabidopsis thaliana	abscisic acid responsive
TaMAPKK2	ABRE	ACGTG	928	5 +	Arabidopsis thaliana	abscisic acid responsive
TaMAPKK2	ABRE	ACGTG	982	5 +	Arabidopsis thaliana	abscisic acid responsive
TaMAPKK2	ABRE	CACGTG	993	6 +	Arabidopsis thaliana	abscisic acid responsive
TaMAPKK2	ABRE	ACGTG	994	5 +	Arabidopsis thaliana	abscisic acid responsive
TaMAPKK2	ABRE	CACGTG	1021	6 -	Arabidopsis thaliana	abscisic acid responsive
TaMAPKK2	ABRE	ACGTG	1022	5 +	Arabidopsis thaliana	abscisic acid responsive
TaMAPKK2	MBS	CAACTG	675	6 +	Arabidopsis thaliana	drought inducibility
TaMAPKK2	MBS	CAACTG	743	6 +	Arabidopsis thaliana	drought inducibility
TaMAPKK2	MBS	CAACTG	749	6 -	Arabidopsis thaliana	drought inducibility
TaMAPKK2	MBS	CAACTG	1710	6 +	Arabidopsis thaliana	drought inducibility
TaMAPKK2	circadian	CAAAGATATC	1905	9 -	Lycopersicon esculentum	circadian control
TaMAPKK2	ARE	AAACCA	842	6 -	Zea mays	anaerobic induction

TaMAPKK2	ARE	AAACCA	1600	6 +	Zea mays	anaerobic induction
TaMAPKK2	LTR	CCGAAA	334	6 +	Hordeum vulgare	low temperature responsive
TaMAPKK2	TGACG-motif	TGACG	155	5 -	Hordeum vulgare	the MeJA responsive
TaMAPKK2	TGACG-motif	TGACG	444	5 +	Hordeum vulgare	the MeJA responsive
TaMAPKK2	TC-rich repeats	GTTTTCTTAC	1661	9 -	Nicotiana tabacum	defense and stress responsive
TaMAPKK2	CGTCA-motif	CGTCA	155	5 +	Hordeum vulgare	the MeJA responsive
TaMAPKK2	CGTCA-motif	CGTCA	444	5 -	Hordeum vulgare	the MeJA responsive
TaMAPKK3	P-box	CCTTTTG	1640	7 +	Oryza sativa	gibberellin responsive
TaMAPKK3	TCA-element	CCATCTTTTT	1476	9 +	Nicotiana tabacum	salicylic acid responsive
TaMAPKK3	TCA-element	CCATCTTTTT	1636	9 +	Nicotiana tabacum	salicylic acid responsive
TaMAPKK3	GARE-motif	TCTGTTG	73	7 +	Brassica oleracea	gibberellin responsive
TaMAPKK3	MBS	CAACTG	221	6 +	Arabidopsis thaliana	drought inducibility
TaMAPKK3	ABRE	ACGTG	339	5 -	Arabidopsis thaliana	abscisic acid responsive
TaMAPKK3	ABRE	GCCGCGTGGC	1088	9 -	Oryza sativa	abscisic acid responsive
TaMAPKK3	TGA-element	AACGAC	581	6 +	Brassica oleracea	auxin responsive
TaMAPKK3	GC-motif	CCCCCG	635	6 +	Zea mays	anoxic specific inducibility
TaMAPKK3	GC-motif	CCCCCG	1270	6 +	Zea mays	anoxic specific inducibility
TaMAPKK3	CGTCA-motif	CGTCA	822	5 -	Hordeum vulgare	the MeJA responsive
TaMAPKK3	CGTCA-motif	CGTCA	853	5 +	Hordeum vulgare	the MeJA responsive

TaMAPKK3	TGACG-motif	TGACG	822	5 +	Hordeum vulgare	the MeJA responsive
TaMAPKK3	TGACG-motif	TGACG	853	5 -	Hordeum vulgare	the MeJA responsive
TaMAPKK3	O2-site	GATGACATGG	744	9 -	Zea mays	zein metabolism regulation
TaMAPKK3	O2-site	GATGA(C/T)(A/G)TG(A/G)	1456	8.5 -	Zea mays	zein metabolism regulation
TaMAPKK3	O2-site	GATGACATGG	1508	9 +	Zea mays	zein metabolism regulation
TaMAPKK3	CAT-box	GCCACT	162	6 +	Arabidopsis thaliana	meristem expression
TaMAPKK3	CAT-box	GCCACT	351	6 +	Arabidopsis thaliana	meristem expression
TaMAPKK3	CAT-box	GCCACT	1721	6 -	Arabidopsis thaliana	meristem expression
TaMAPKK4	CGTCA-motif	CGTCA	104	5 -	Hordeum vulgare	the MeJA responsive
TaMAPKK4	CGTCA-motif	CGTCA	132	5 +	Hordeum vulgare	the MeJA responsive
TaMAPKK4	CGTCA-motif	CGTCA	228	5 +	Hordeum vulgare	the MeJA responsive
TaMAPKK4	CGTCA-motif	CGTCA	399	5 -	Hordeum vulgare	the MeJA responsive
TaMAPKK4	CGTCA-motif	CGTCA	471	5 +	Hordeum vulgare	the MeJA responsive
TaMAPKK4	CGTCA-motif	CGTCA	486	5 +	Hordeum vulgare	the MeJA responsive
TaMAPKK4	CGTCA-motif	CGTCA	1678	5 -	Hordeum vulgare	the MeJA responsive
TaMAPKK4	ABRE	GCCGCGTGGC	187	9 +	Oryza sativa	abscisic acid responsive
TaMAPKK4	ABRE	AACCCGG	275	7 -	Arabidopsis thaliana	abscisic acid responsive
TaMAPKK4	ABRE	ACGTG	527	5 +	Arabidopsis thaliana	abscisic acid responsive
TaMAPKK4	ABRE	GCAACGTGTC	555	9 -	Hordeum vulgare	abscisic acid responsive

TaMAPKK4	ABRE	ACGTG	1154	5 +	Arabidopsis thaliana	abscisic acid responsive
TaMAPKK4	TCA-element	CCATCTTTTT	1605	9 +	Nicotiana tabacum	salicylic acid responsive
TaMAPKK4	GC-motif	CCCCCG	1641	6 +	Zea mays	anoxic specific inducibility
TaMAPKK4	ARE	AAACCA	1233	6 -	Zea mays	anaerobic induction
TaMAPKK4	TGACG-motif	TGACG	104	5 +	Hordeum vulgare	the MeJA responsive
TaMAPKK4	TGACG-motif	TGACG	132	5 -	Hordeum vulgare	the MeJA responsive
TaMAPKK4	TGACG-motif	TGACG	228	5 -	Hordeum vulgare	the MeJA responsive
TaMAPKK4	TGACG-motif	TGACG	399	5 +	Hordeum vulgare	the MeJA responsive
TaMAPKK4	TGACG-motif	TGACG	471	5 -	Hordeum vulgare	the MeJA responsive
TaMAPKK4	TGACG-motif	TGACG	486	5 -	Hordeum vulgare	the MeJA responsive
TaMAPKK4	TGACG-motif	TGACG	1678	5 +	Hordeum vulgare	the MeJA responsive
TaMAPKK4	O2-site	GTTGACGTGA	985	9 +	Zea mays	zein metabolism regulation
TaMAPKK4	GARE-motif	TCTGTTG	1171	7 +	Brassica oleracea	gibberellin responsive
TaMAPKK5	TCA-element	CCATCTTTTT	551	9 +	Nicotiana tabacum	salicylic acid responsive
TaMAPKK5	ABRE	ACGTG	675	5 -	Arabidopsis thaliana	abscisic acid responsive
TaMAPKK5	CGTCA-motif	CGTCA	677	5 +	Hordeum vulgare	the MeJA responsive
TaMAPKK5	CGTCA-motif	CGTCA	1326	5 -	Hordeum vulgare	the MeJA responsive
TaMAPKK5	CAT-box	GCCACT	1852	6 +	Arabidopsis thaliana	meristem expression
TaMAPKK5	MBS	CAACTG	945	6 +	Arabidopsis thaliana	drought inducibility

TaMAPKK5	TGACG-motif	TGACG	677	5 -	Hordeum vulgare	the MeJA responsive
TaMAPKK5	TGACG-motif	TGACG	1326	5 +	Hordeum vulgare	the MeJA responsive
TaMAPKK6	TGA-box	TGACGTAA	1186	8 -	Glycine max	auxin responsive
TaMAPKK6	ABRE	TACGGTC	958	7 -	Arabidopsis thaliana	abscisic acid responsive
TaMAPKK6	ABRE	CGCACGTGTC	971	9 +	Hordeum vulgare	abscisic acid responsive
TaMAPKK6	ABRE	ACGTG	1550	5 -	Arabidopsis thaliana	abscisic acid responsive
TaMAPKK6	ABRE	ACGTG	1695	5 -	Arabidopsis thaliana	abscisic acid responsive
TaMAPKK6	ABRE	GACACGTACG T	1696	10 -	Oryza sativa	abscisic acid responsive
TaMAPKK6	ABRE	ACGTG	1700	5 +	Arabidopsis thaliana	abscisic acid responsive
TaMAPKK6	ABRE	GACACGTGGC	1704	9 +	Triticum aestivum	abscisic acid responsive
TaMAPKK6	ABRE	ACGTG	1868	5 -	Arabidopsis thaliana	abscisic acid responsive
TaMAPKK6	CGTCA-motif	CGTCA	355	5 -	Hordeum vulgare	the MeJA responsive
TaMAPKK6	CGTCA-motif	CGTCA	784	5 -	Hordeum vulgare	the MeJA responsive
TaMAPKK6	CGTCA-motif	CGTCA	799	5 -	Hordeum vulgare	the MeJA responsive
TaMAPKK6	CGTCA-motif	CGTCA	1127	5 -	Hordeum vulgare	the MeJA responsive
TaMAPKK6	CGTCA-motif	CGTCA	1189	5 +	Hordeum vulgare	the MeJA responsive
TaMAPKK6	CGTCA-motif	CGTCA	1552	5 +	Hordeum vulgare	the MeJA responsive
TaMAPKK6	CGTCA-motif	CGTCA	1962	5 -	Hordeum vulgare	the MeJA responsive
TaMAPKK6	LTR	CCGAAA	1438	6 -	Hordeum vulgare	low temperature responsive
TaMAPKK6	TGA-element	AACGAC	955	6 +	Brassica oleracea	auxin responsive

TaMAPKK6	CAT-box	GCCACT	475	6 -	Arabidopsis thaliana	meristem expression
TaMAPKK6	GARE-motif	TCTGTTG	175	7 +	Brassica oleracea	gibberellin responsive
TaMAPKK6	MBS	CAACTG	1555	6 -	Arabidopsis thaliana	drought inducibility
TaMAPKK6	O2-site	GATGATGTGG	1199	9 -	Zea mays	zein metabolism regulation
TaMAPKK6	TGACG-motif	TGACG	355	5 +	Hordeum vulgare	the MeJA responsive
TaMAPKK6	TGACG-motif	TGACG	784	5 +	Hordeum vulgare	the MeJA responsive
TaMAPKK6	TGACG-motif	TGACG	799	5 +	Hordeum vulgare	the MeJA responsive
TaMAPKK6	TGACG-motif	TGACG	1127	5 +	Hordeum vulgare	the MeJA responsive
TaMAPKK6	TGACG-motif	TGACG	1189	5 -	Hordeum vulgare	the MeJA responsive
TaMAPKK6	TGACG-motif	TGACG	1552	5 -	Hordeum vulgare	the MeJA responsive
TaMAPKK6	TGACG-motif	TGACG	1962	5 +	Hordeum vulgare	the MeJA responsive
TaMAPKK7	MBS	CAACTG	53	6 -	Arabidopsis thaliana	drought inducibility
TaMAPKK7	MBSI	TTTTTACGGTT A	825	11 +	Petunia hybrida	flavonoid biosynthetic genes regulation
TaMAPKK7	ABRE	AACCCGG	725	7 -	Arabidopsis thaliana	abscisic acid responsive
TaMAPKK7	ABRE	CACGTG	1104	6 -	Arabidopsis thaliana	abscisic acid responsive
TaMAPKK7	ABRE	ACGTG	1105	5 +	Arabidopsis thaliana	abscisic acid responsive
TaMAPKK7	ABRE	GCAACGTGTC	1434	9 -	Hordeum vulgare	abscisic acid responsive
TaMAPKK7	ABRE	ACGTG	1436	5 -	Arabidopsis thaliana	abscisic acid responsive

TaMAPKK7	ABRE	ACGTG	1449	5 -	Arabidopsis thaliana	abscisic acid responsive
TaMAPKK7	ABRE	ACGTG	1532	5 -	Arabidopsis thaliana	abscisic acid responsive
TaMAPKK7	ABRE	ACGTG	1886	5 -	Arabidopsis thaliana	abscisic acid responsive
TaMAPKK7	AuxRR-core	GGTCCAT	370	7 -	Nicotiana tabacum	auxin responsive
TaMAPKK7	TCA-element	CCATCTTTTT	1165	9 +	Nicotiana tabacum	salicylic acid responsive
TaMAPKK7	TGACG-motif	TGACG	828	5 +	Hordeum vulgare	the MeJA responsive
TaMAPKK7	TGACG-motif	TGACG	1995	5 +	Hordeum vulgare	the MeJA responsive
TaMAPKK7	GC-motif	CCCCCG	704	6 -	Zea mays	anoxic specific inducibility
TaMAPKK7	ARE	AAACCA	319	6 +	Zea mays	anaerobic induction
TaMAPKK7	CAT-box	GCCACT	1190	6 -	Arabidopsis thaliana	meristem expression
TaMAPKK7	CAT-box	GCCACT	1550	6 +	Arabidopsis thaliana	meristem expression
TaMAPKK7	LTR	CCGAAA	1250	6 -	Hordeum vulgare	low temperature responsive
TaMAPKK7	CGTCA-motif	CGTCA	828	5 -	Hordeum vulgare	the MeJA responsive
TaMAPKK7	CGTCA-motif	CGTCA	1995	5 -	Hordeum vulgare	the MeJA responsive
TaMAPKK8	motif I	gGTACGTGGCG	1053	10 -	Oryza sativa	root specific
TaMAPKK8	CAT-box	GCCACT	543	6 +	Arabidopsis thaliana	meristem expression
TaMAPKK8	GC-motif	CCCCCG	493	6 +	Zea mays	anoxic specific inducibility
TaMAPKK8	GC-motif	CCCCCG	497	6 -	Zea mays	anoxic specific inducibility
TaMAPKK8	GC-motif	CCCCCG	963	6 +	Zea mays	anoxic specific inducibility

TaMAPKK8	TGACG-motif	TGACG	55	5 +	Hordeum vulgare	the MeJA responsive
TaMAPKK8	TGACG-motif	TGACG	549	5 -	Hordeum vulgare	the MeJA responsive
TaMAPKK8	TGACG-motif	TGACG	1476	5 +	Hordeum vulgare	the MeJA responsive
TaMAPKK8	TGACG-motif	TGACG	1835	5 -	Hordeum vulgare	the MeJA responsive
TaMAPKK8	O2-site	GATGACATGG	79	9 +	Zea mays	zein metabolism regulation
TaMAPKK8	O2-site	GATGACATGG	1030	9 +	Zea mays	zein metabolism regulation
TaMAPKK8	ABRE	ACGTG	57	5 +	Arabidopsis thaliana	abscisic acid responsive
TaMAPKK8	ABRE	ACGTG	435	5 -	Arabidopsis thaliana	abscisic acid responsive
TaMAPKK8	ABRE	TACGGTC	463	7 +	Arabidopsis thaliana	abscisic acid responsive
TaMAPKK8	CGTCA-motif	CGTCA	55	5 -	Hordeum vulgare	the MeJA responsive
TaMAPKK8	CGTCA-motif	CGTCA	549	5 +	Hordeum vulgare	the MeJA responsive
TaMAPKK8	CGTCA-motif	CGTCA	1476	5 -	Hordeum vulgare	the MeJA responsive
TaMAPKK8	CGTCA-motif	CGTCA	1835	5 +	Hordeum vulgare	the MeJA responsive
TaMAPKK8	TGA-element	AACGAC	944	6 +	Brassica oleracea	auxin responsive
TaMAPKK9	ABRE	ACGTG	832	5 -	Arabidopsis thaliana	abscisic acid responsive
TaMAPKK9	ABRE	ACGTG	873	5 +	Arabidopsis thaliana	abscisic acid responsive
TaMAPKK9	ABRE	CACGTG	899	6 +	Arabidopsis thaliana	abscisic acid responsive
TaMAPKK9	ABRE	ACGTG	900	5 +	Arabidopsis thaliana	abscisic acid responsive
TaMAPKK9	ABRE	ACGTG	1864	5 -	Arabidopsis thaliana	abscisic acid responsive

TaMAPKK9	ABRE	ACGTG	1977	5 -	Arabidopsis thaliana	abscisic acid responsive
TaMAPKK9	ARE	AAACCA	496	6 +	Zea mays	anaerobic induction
TaMAPKK9	TGACG-motif	TGACG	1534	5 -	Hordeum vulgare	the MeJA responsive
TaMAPKK9	TCA-element	CCATCTTTTT	797	9 -	Nicotiana tabacum	salicylic acid responsive
TaMAPKK9	TCA-element	CCATCTTTTT	984	9 +	Nicotiana tabacum	salicylic acid responsive
TaMAPKK9	O2-site	GATGATGTGG	1129	9 -	Zea mays	zein metabolism regulation
TaMAPKK9	GCN4_motif	TGAGTCA	1	7 -	Oryza sativa	endosperm expression
TaMAPKK9	MBS	CAACTG	79	6 -	Arabidopsis thaliana	drought inducibility
TaMAPKK9	MBS	CAACTG	171	6 -	Arabidopsis thaliana	drought inducibility
TaMAPKK9	MBS	CAACTG	201	6 +	Arabidopsis thaliana	drought inducibility
TaMAPKK9	MBS	CAACTG	250	6 -	Arabidopsis thaliana	drought inducibility
TaMAPKK9	MBS	CAACTG	280	6 +	Arabidopsis thaliana	drought inducibility
TaMAPKK9	MBS	CAACTG	359	6 +	Arabidopsis thaliana	drought inducibility
TaMAPKK9	MBS	CAACTG	416	6 -	Arabidopsis thaliana	drought inducibility
TaMAPKK9	MBS	CAACTG	436	6 +	Arabidopsis thaliana	drought inducibility
TaMAPKK9	MBS	CAACTG	565	6 +	Arabidopsis thaliana	drought inducibility
TaMAPKK9	MBS	CAACTG	610	6 +	Arabidopsis thaliana	drought inducibility
TaMAPKK9	MBS	CAACTG	632	6 +	Arabidopsis thaliana	drought inducibility
TaMAPKK9	MBS	CAACTG	674	6 +	Arabidopsis thaliana	drought inducibility

TaMAPKK9	MBS	CAACTG	1285	6 -	Arabidopsis thaliana	drought inducibility
TaMAPKK9	CGTCA-motif	CGTCA	1534	5 +	Hordeum vulgare	the MeJA responsive
TaMAPKKK1	TCA-element	CCATCTTTTT	1024	9 +	Nicotiana tabacum	salicylic acid responsive
TaMAPKKK1	TCA-element	TCAGAAGAGG	1902	9 -	Brassica oleracea	salicylic acid responsive
TaMAPKKK1	ARE	AAACCA	143	6 +	Zea mays	anaerobic induction
TaMAPKKK1	ARE	AAACCA	976	6 +	Zea mays	anaerobic induction
TaMAPKKK1	ARE	AAACCA	1727	6 -	Zea mays	anaerobic induction
TaMAPKKK1	ARE	AAACCA	1818	6 -	Zea mays	anaerobic induction
TaMAPKKK1	LTR	CCGAAA	714	6 +	Hordeum vulgare	low temperature responsive
TaMAPKKK1	CAT-box	GCCACT	859	6 -	Arabidopsis thaliana	meristem expression
TaMAPKKK1	P-box	CCTTTTG	318	7 +	Oryza sativa	gibberellin responsive
TaMAPKKK1	P-box	CCTTTTG	1669	7 +	Oryza sativa	gibberellin responsive
TaMAPKKK1	ABRE	ACGTG	1582	5 -	Arabidopsis thaliana	abscisic acid responsive
TaMAPKKK1	GC-motif	CCCCCG	1103	6 +	Zea mays	anoxic specific inducibility
TaMAPKKK1	GC-motif	CCCCCG	1587	6 -	Zea mays	anoxic specific inducibility
TaMAPKKK1	MBS	CAACTG	947	6 -	Arabidopsis thaliana	drought inducibility
TaMAPKKK10	TC-rich repeats	ATTCTCTAAC	1619	9 -	Nicotiana tabacum	defense and stress responsive
TaMAPKKK10	TGACG-motif	TGACG	1155	5 -	Hordeum vulgare	the MeJA responsive
TaMAPKKK10	TGACG-motif	TGACG	1400	5 +	Hordeum vulgare	the MeJA responsive

TaMAPKKK10	TGACG-motif	TGACG	1661	5 +	Hordeum vulgare	the MeJA responsive
TaMAPKKK10	O2-site	GTTGACGTGA	1398	9 +	Zea mays	zein metabolism regulation
TaMAPKKK10	GARE-motif	TCTGTTG	408	7 -	Brassica oleracea	gibberellin responsive
TaMAPKKK10	MBS	CAACTG	429	6 +	Arabidopsis thaliana	drought inducibility
TaMAPKKK10	MBS	CAACTG	508	6 +	Arabidopsis thaliana	drought inducibility
TaMAPKKK10	MBS	CAACTG	935	6 -	Arabidopsis thaliana	drought inducibility
TaMAPKKK10	ARE	AAACCA	1632	6 +	Zea mays	anaerobic induction
TaMAPKKK10	ARE	AAACCA	1772	6 +	Zea mays	anaerobic induction
TaMAPKKK10	GC-motif	CCCCCG	1439	6 +	Zea mays	anoxic specific inducibility
TaMAPKKK10	GC-motif	CCCCCG	1731	6 -	Zea mays	anoxic specific inducibility
TaMAPKKK10	GC-motif	CCCCCG	1931	6 +	Zea mays	anoxic specific inducibility
TaMAPKKK10	GC-motif	CCCCCG	1983	6 +	Zea mays	anoxic specific inducibility
TaMAPKKK10	CAT-box	GCCACT	1316	6 +	Arabidopsis thaliana	meristem expression
TaMAPKKK10	P-box	CCTTTTG	68	7 -	Oryza sativa	gibberellin responsive
TaMAPKKK10	CGTCA-motif	CGTCA	1155	5 +	Hordeum vulgare	the MeJA responsive
TaMAPKKK10	CGTCA-motif	CGTCA	1400	5 -	Hordeum vulgare	the MeJA responsive
TaMAPKKK10	CGTCA-motif	CGTCA	1661	5 -	Hordeum vulgare	the MeJA responsive
TaMAPKKK10	ABRE	AACCCGG	713	7 +	Arabidopsis thaliana	abscisic acid responsive
TaMAPKKK10	ABRE	GCCGCGTGGC	1348	9 +	Oryza sativa	abscisic acid responsive

TaMAPKKK10	ABRE	ACGTG	1360	5 +	Arabidopsis thaliana	abscisic acid responsive
TaMAPKKK10	ABRE	ACGTG	1402	5 +	Arabidopsis thaliana	abscisic acid responsive
TaMAPKKK10	TCA-element	TCAGAAGAGG	566	9 +	Brassica oleracea	salicylic acid responsive
TaMAPKKK100	MBS	CAACTG	789	6 +	Arabidopsis thaliana	drought inducibility
TaMAPKKK100	P-box	CCTTTTG	1625	7 +	Oryza sativa	gibberellin responsive
TaMAPKKK100	motif I	gGTACGTGGCG	824	10 -	Oryza sativa	root specific
TaMAPKKK100	GC-motif	CCCCCG	1312	6 +	Zea mays	anoxic specific inducibility
TaMAPKKK100	GC-motif	CCCCCG	1662	6 -	Zea mays	anoxic specific inducibility
TaMAPKKK100	CGTCA-motif	CGTCA	943	5 +	Hordeum vulgare	the MeJA responsive
TaMAPKKK100	AuxRR-core	GGTCCAT	1981	7 +	Nicotiana tabacum	auxin responsive
TaMAPKKK100	GARE-motif	TCTGTTG	692	7 +	Brassica oleracea	gibberellin responsive
TaMAPKKK100	ABRE	GCAACGTGTC	24	9 +	Hordeum vulgare	abscisic acid responsive
TaMAPKKK100	ABRE	ACGTG	27	5 +	Arabidopsis thaliana	abscisic acid responsive
TaMAPKKK100	ABRE	CACGTG	64	6 +	Arabidopsis thaliana	abscisic acid responsive
TaMAPKKK100	ABRE	ACGTG	65	5 +	Arabidopsis thaliana	abscisic acid responsive
TaMAPKKK100	ABRE	TACGTGTC	768	8 -	Oryza sativa	abscisic acid responsive
TaMAPKKK100	ABRE	ACGTG	770	5 -	Arabidopsis thaliana	abscisic acid responsive
TaMAPKKK100	ABRE	ACGTG	827	5 -	Arabidopsis thaliana	abscisic acid responsive
TaMAPKKK100	ABRE	CACGTG	875	6 +	Arabidopsis thaliana	abscisic acid responsive

TaMAPKKK100	ABRE	ACGTG	876	5 +	Arabidopsis thaliana	abscisic acid responsive
TaMAPKKK100	ABRE	ACGTG	1392	5 -	Arabidopsis thaliana	abscisic acid responsive
TaMAPKKK100	ABRE	ACGTG	1460	5 -	Arabidopsis thaliana	abscisic acid responsive
TaMAPKKK100	ABRE	ACGTG	1492	5 -	Arabidopsis thaliana	abscisic acid responsive
TaMAPKKK100	ABRE	AACCCGG	1885	7 -	Arabidopsis thaliana	abscisic acid responsive
TaMAPKKK100	TGACG-motif	TGACG	943	5 -	Hordeum vulgare	the MeJA responsive
TaMAPKKK101	ABRE	ACGTG	1144	5 -	Arabidopsis thaliana	abscisic acid responsive
TaMAPKKK101	TGA-element	AACGAC	1139	6 +	Brassica oleracea	auxin responsive
TaMAPKKK101	TATC-box	TATCCCA	293	7 -	Oryza sativa	gibberellin responsive
TaMAPKKK101	TCA-element	CCATCTTTTT	356	9 +	Nicotiana tabacum	salicylic acid responsive
TaMAPKKK101	O2-site	GATGATGTGG	100	9 -	Zea mays	zein metabolism regulation
TaMAPKKK101	CAT-box	GCCACT	1580	6 -	Arabidopsis thaliana	meristem expression
TaMAPKKK101	ARE	AAACCA	568	6 -	Zea mays	anaerobic induction
TaMAPKKK101	ARE	AAACCA	1710	6 -	Zea mays	anaerobic induction
TaMAPKKK101	CGTCA-motif	CGTCA	516	5 +	Hordeum vulgare	the MeJA responsive
TaMAPKKK101	CGTCA-motif	CGTCA	609	5 -	Hordeum vulgare	the MeJA responsive
TaMAPKKK101	TGACG-motif	TGACG	516	5 -	Hordeum vulgare	the MeJA responsive
TaMAPKKK101	TGACG-motif	TGACG	609	5 +	Hordeum vulgare	the MeJA responsive
TaMAPKKK102	CAT-box	GCCACT	1559	6 +	Arabidopsis thaliana	meristem expression

TaMAPKKK102	GC-motif	CCCCCG	1007	6 +	Zea mays	anoxic specific inducibility
TaMAPKKK102	GC-motif	CCCCCG	1329	6 +	Zea mays	anoxic specific inducibility
TaMAPKKK102	GC-motif	CCCCCG	1340	6 +	Zea mays	anoxic specific inducibility
TaMAPKKK102	GC-motif	CCCCCG	1357	6 +	Zea mays	anoxic specific inducibility
TaMAPKKK102	TGA-element	AACGAC	135	6 -	Brassica oleracea	auxin responsive
TaMAPKKK102	TGACG-motif	TGACG	284	5 +	Hordeum vulgare	the MeJA responsive
TaMAPKKK102	LTR	CCGAAA	241	6 +	Hordeum vulgare	low temperature responsive
TaMAPKKK102	LTR	CCGAAA	412	6 -	Hordeum vulgare	low temperature responsive
TaMAPKKK102	MBS	CAACTG	130	6 -	Arabidopsis thaliana	drought inducibility
TaMAPKKK102	ARE	AAACCA	1675	6 -	Zea mays	anaerobic induction
TaMAPKKK102	ABRE	ACGTG	772	5 -	Arabidopsis thaliana	abscisic acid responsive
TaMAPKKK102	ABRE	ACGTG	932	5 -	Arabidopsis thaliana	abscisic acid responsive
TaMAPKKK102	CGTCA-motif	CGTCA	284	5 -	Hordeum vulgare	the MeJA responsive
TaMAPKKK103	LTR	CCGAAA	772	6 +	Hordeum vulgare	low temperature responsive
TaMAPKKK103	TC-rich repeats	ATTCTCTAAC	512	9 -	Nicotiana tabacum	defense and stress responsive
TaMAPKKK103	MBS	CAACTG	643	6 +	Arabidopsis thaliana	drought inducibility
TaMAPKKK103	MSA-like	TCCAACGGT	822	9 +	Catharanthus roseus	cell cycle regulation
TaMAPKKK103	CAT-box	GCCACT	709	6 +	Arabidopsis thaliana	meristem expression
TaMAPKKK103	ABRE	ACGTG	968	5 +	Arabidopsis thaliana	abscisic acid responsive

TaMAPKKK103	ABRE	ACGTG	1581	5 -	Arabidopsis thaliana	abscisic acid responsive
TaMAPKKK103	ABRE	GACACGTGGC	1780	9 -	Triticum aestivum	abscisic acid responsive
TaMAPKKK103	GC-motif	CCCCCG	862	6 +	Zea mays	anoxic specific inducibility
TaMAPKKK103	TGACG-motif	TGACG	943	5 -	Hordeum vulgare	the MeJA responsive
TaMAPKKK103	TGACG-motif	TGACG	1524	5 +	Hordeum vulgare	the MeJA responsive
TaMAPKKK103	TGACG-motif	TGACG	1727	5 -	Hordeum vulgare	the MeJA responsive
TaMAPKKK103	CGTCA-motif	CGTCA	943	5 +	Hordeum vulgare	the MeJA responsive
TaMAPKKK103	CGTCA-motif	CGTCA	1524	5 -	Hordeum vulgare	the MeJA responsive
TaMAPKKK103	CGTCA-motif	CGTCA	1727	5 +	Hordeum vulgare	the MeJA responsive
TaMAPKKK104	MBS	CAACTG	113	6 -	Arabidopsis thaliana	drought inducibility
TaMAPKKK104	MBS	CAACTG	913	6 -	Arabidopsis thaliana	drought inducibility
TaMAPKKK104	P-box	CCTTTTG	1892	7 +	Oryza sativa	gibberellin responsive
TaMAPKKK104	GC-motif	CCCCCG	1148	6 -	Zea mays	anoxic specific inducibility
TaMAPKKK104	ABRE	ACGTG	1808	5 -	Arabidopsis thaliana	abscisic acid responsive
TaMAPKKK104	MSA-like	(T/C)C(T/C)AAC GG(T/C)(T/C)A	626	9 +	Catharanthus roseus	cell cycle regulation
TaMAPKKK104	CGTCA-motif	CGTCA	501	5 +	Hordeum vulgare	the MeJA responsive
TaMAPKKK104	CGTCA-motif	CGTCA	944	5 +	Hordeum vulgare	the MeJA responsive
TaMAPKKK104	CGTCA-motif	CGTCA	1762	5 -	Hordeum vulgare	the MeJA responsive

TaMAPKKK104	AuxRR-core	GGTCCAT	614	7 +	Nicotiana tabacum	auxin responsive
TaMAPKKK104	O2-site	GATGATGTGG	294	9 -	Zea mays	zein metabolism regulation
TaMAPKKK104	O2-site	GATGACATGG	799	9 +	Zea mays	zein metabolism regulation
TaMAPKKK104	O2-site	GATGATGTGG	994	9 +	Zea mays	zein metabolism regulation
TaMAPKKK104	GCN4_motif	TGAGTCA	1483	7 -	Oryza sativa	endosperm expression
TaMAPKKK104	TGACG-motif	TGACG	501	5 -	Hordeum vulgare	the MeJA responsive
TaMAPKKK104	TGACG-motif	TGACG	944	5 -	Hordeum vulgare	the MeJA responsive
TaMAPKKK104	TGACG-motif	TGACG	1762	5 +	Hordeum vulgare	the MeJA responsive
TaMAPKKK104-1	MBS	CAACTG	1187	6 +	Arabidopsis thaliana	drought inducibility
TaMAPKKK104-1	MBS	CAACTG	1650	6 -	Arabidopsis thaliana	drought inducibility
TaMAPKKK104-1	TGA-element	AACGAC	1192	6 -	Brassica oleracea	auxin responsive
TaMAPKKK104-1	TGA-element	AACGAC	1383	6 +	Brassica oleracea	auxin responsive
TaMAPKKK104-1	TGA-element	AACGAC	1475	6 +	Brassica oleracea	auxin responsive
TaMAPKKK104-1	CAT-box	GCCACT	483	6 +	Arabidopsis thaliana	meristem expression
TaMAPKKK104-1	CAT-box	GCCACT	734	6 +	Arabidopsis thaliana	meristem expression
TaMAPKKK104-1	CAT-box	GCCACT	1294	6 +	Arabidopsis thaliana	meristem expression
TaMAPKKK104-1	CGTCA-motif	CGTCA	958	5 -	Hordeum vulgare	the MeJA responsive
TaMAPKKK104-1	CGTCA-motif	CGTCA	1055	5 -	Hordeum vulgare	the MeJA responsive
TaMAPKKK104-1	CGTCA-motif	CGTCA	1197	5 -	Hordeum vulgare	the MeJA responsive

TaMAPKKK104-1	CGTCA-motif	CGTCA	1208	5 +	Hordeum vulgare	the MeJA responsive
TaMAPKKK104-1	CGTCA-motif	CGTCA	1492	5 +	Hordeum vulgare	the MeJA responsive
TaMAPKKK104-1	CGTCA-motif	CGTCA	1541	5 +	Hordeum vulgare	the MeJA responsive
TaMAPKKK104-1	P-box	CCTTTTG	1901	7 +	Oryza sativa	gibberellin responsive
TaMAPKKK104-1	ABRE	CACGTG	138	6 +	Arabidopsis thaliana	abscisic acid responsive
TaMAPKKK104-1	ABRE	ACGTG	139	5 +	Arabidopsis thaliana	abscisic acid responsive
TaMAPKKK104-1	ABRE	ACGTG	1206	5 -	Arabidopsis thaliana	abscisic acid responsive
TaMAPKKK104-1	ABRE	CGTACGTGCA	1982	9 -	Hordeum vulgare	abscisic acid responsive
TaMAPKKK104-1	GC-motif	CCCCCG	1456	6 -	Zea mays	anoxic specific inducibility
TaMAPKKK104-1	GARE-motif	TCTGTTG	908	7 -	Brassica oleracea	gibberellin responsive
TaMAPKKK104-1	O2-site	GATGATGTGG	1749	9 +	Zea mays	zein metabolism regulation
TaMAPKKK104-1	TGACG-motif	TGACG	958	5 +	Hordeum vulgare	the MeJA responsive
TaMAPKKK104-1	TGACG-motif	TGACG	1055	5 +	Hordeum vulgare	the MeJA responsive
TaMAPKKK104-1	TGACG-motif	TGACG	1197	5 +	Hordeum vulgare	the MeJA responsive
TaMAPKKK104-1	TGACG-motif	TGACG	1208	5 -	Hordeum vulgare	the MeJA responsive
TaMAPKKK104-1	TGACG-motif	TGACG	1492	5 -	Hordeum vulgare	the MeJA responsive
TaMAPKKK104-1	TGACG-motif	TGACG	1541	5 -	Hordeum vulgare	the MeJA responsive
TaMAPKKK104-1	ARE	AAACCA	1973	6 -	Zea mays	anaerobic induction
TaMAPKKK105	ABRE	ACGTG	183	5 -	Arabidopsis thaliana	abscisic acid responsive

TaMAPKKK105	ABRE	GCCGCGTGGC	590	9	+	Oryza sativa	abscisic acid responsive
TaMAPKKK105	ABRE	ACGTG	1808	5	-	Arabidopsis thaliana	abscisic acid responsive
TaMAPKKK105	ABRE	ACGTG	1813	5	-	Arabidopsis thaliana	abscisic acid responsive
TaMAPKKK105	MSA-like	(T/C)C(T/C)AACGG(T/C)(T/C)A	753	8.5	-	Catharanthus roseus	cell cycle regulation
TaMAPKKK105	TGA-element	AACGAC	1320	6	-	Brassica oleracea	auxin responsive
TaMAPKKK105	TGACG-motif	TGACG	1692	5	+	Hordeum vulgare	the MeJA responsive
TaMAPKKK105	TGACG-motif	TGACG	1810	5	-	Hordeum vulgare	the MeJA responsive
TaMAPKKK105	MBS	CAACTG	308	6	+	Arabidopsis thaliana	drought inducibility
TaMAPKKK105	MBS	CAACTG	1102	6	+	Arabidopsis thaliana	drought inducibility
TaMAPKKK105	GC-motif	CCCCCG	1503	6	-	Zea mays	anoxic specific inducibility
TaMAPKKK105	TC-rich repeats	ATTCTCTAAC	758	9	-	Nicotiana tabacum	defense and stress responsive
TaMAPKKK105	TC-rich repeats	ATTCTCTAAC	929	9	+	Nicotiana tabacum	defense and stress responsive
TaMAPKKK105	LTR	CCGAAA	554	6	-	Hordeum vulgare	low temperature responsive
TaMAPKKK105	CGTCA-motif	CGTCA	1692	5	-	Hordeum vulgare	the MeJA responsive
TaMAPKKK105	CGTCA-motif	CGTCA	1810	5	+	Hordeum vulgare	the MeJA responsive
TaMAPKKK105	HD-Zip 1	CAAT(A/T)ATTG	1182	8	-	Arabidopsis thaliana	differentiation of the palisade mesophyll cells
TaMAPKKK106	TGA-box	TGACGTAA	955	8	+	Glycine max	auxin responsive
TaMAPKKK106	ABRE	ACGTG	63	5	+	Arabidopsis thaliana	abscisic acid responsive
TaMAPKKK106	ABRE	ACGTG	197	5	+	Arabidopsis thaliana	abscisic acid responsive

TaMAPKKK106	ABRE	ACGTG	1288	5 -	Arabidopsis thaliana	abscisic acid responsive
TaMAPKKK106	LTR	CCGAAA	177	6 +	Hordeum vulgare	low temperature responsive
TaMAPKKK106	CGTCA-motif	CGTCA	99	5 +	Hordeum vulgare	the MeJA responsive
TaMAPKKK106	CGTCA-motif	CGTCA	450	5 -	Hordeum vulgare	the MeJA responsive
TaMAPKKK106	CGTCA-motif	CGTCA	776	5 +	Hordeum vulgare	the MeJA responsive
TaMAPKKK106	CGTCA-motif	CGTCA	955	5 -	Hordeum vulgare	the MeJA responsive
TaMAPKKK106	CGTCA-motif	CGTCA	1801	5 -	Hordeum vulgare	the MeJA responsive
TaMAPKKK106	TGA-element	AACGAC	857	6 +	Brassica oleracea	auxin responsive
TaMAPKKK106	TGA-element	AACGAC	920	6 +	Brassica oleracea	auxin responsive
TaMAPKKK106	MSA-like	TCCAACGGT	797	9 -	Catharanthus roseus	cell cycle regulation
TaMAPKKK106	GARE-motif	TCTGTTG	159	7 -	Brassica oleracea	gibberellin responsive
TaMAPKKK106	MBS	CAACTG	537	6 +	Arabidopsis thaliana	drought inducibility
TaMAPKKK106	MBS	CAACTG	1888	6 +	Arabidopsis thaliana	drought inducibility
TaMAPKKK106	O2-site	GATGA(C/T)(A/G)TG(A/G)	201	8 +	Zea mays	zein metabolism regulation
TaMAPKKK106	TGACG-motif	TGACG	99	5 -	Hordeum vulgare	the MeJA responsive
TaMAPKKK106	TGACG-motif	TGACG	450	5 +	Hordeum vulgare	the MeJA responsive
TaMAPKKK106	TGACG-motif	TGACG	776	5 -	Hordeum vulgare	the MeJA responsive
TaMAPKKK106	TGACG-motif	TGACG	955	5 +	Hordeum vulgare	the MeJA responsive
TaMAPKKK106	TGACG-motif	TGACG	1801	5 +	Hordeum vulgare	the MeJA responsive
TaMAPKKK106	ARE	AAACCA	1248	6 -	Zea mays	anaerobic induction

TaMAPKKK107	TGA-element	AACGAC	1677	6 +	Brassica oleracea	auxin responsive
TaMAPKKK107	TGA-element	AACGAC	1740	6 +	Brassica oleracea	auxin responsive
TaMAPKKK107	ABRE	ACGTG	885	5 +	Arabidopsis thaliana	abscisic acid responsive
TaMAPKKK107	ABRE	GCCGCGTGGC	1837	9 -	Oryza sativa	abscisic acid responsive
TaMAPKKK107	ARE	AAACCA	523	6 -	Zea mays	anaerobic induction
TaMAPKKK107	O2-site	GATGACATGG	915	9 -	Zea mays	zein metabolism regulation
TaMAPKKK107	O2-site	GATGA(C/T)(A/G)TG(A/G)	1023	8 +	Zea mays	zein metabolism regulation
TaMAPKKK107	TGACG-motif	TGACG	1176	5 +	Hordeum vulgare	the MeJA responsive
TaMAPKKK107	TGACG-motif	TGACG	1271	5 +	Hordeum vulgare	the MeJA responsive
TaMAPKKK107	TGACG-motif	TGACG	1772	5 +	Hordeum vulgare	the MeJA responsive
TaMAPKKK107	CGTCA-motif	CGTCA	1176	5 -	Hordeum vulgare	the MeJA responsive
TaMAPKKK107	CGTCA-motif	CGTCA	1271	5 -	Hordeum vulgare	the MeJA responsive
TaMAPKKK107	CGTCA-motif	CGTCA	1772	5 -	Hordeum vulgare	the MeJA responsive
TaMAPKKK107	LTR	CCGAAA	999	6 +	Hordeum vulgare	low temperature responsive
TaMAPKKK107	GARE-motif	TCTGTTG	981	7 -	Brassica oleracea	gibberellin responsive
TaMAPKKK107	MBS	CAACTG	474	6 -	Arabidopsis thaliana	drought inducibility
TaMAPKKK107	MBS	CAACTG	1357	6 +	Arabidopsis thaliana	drought inducibility
TaMAPKKK108	TGACG-motif	TGACG	238	5 -	Hordeum vulgare	the MeJA responsive
TaMAPKKK108	TGACG-motif	TGACG	625	5 -	Hordeum vulgare	the MeJA responsive
TaMAPKKK108	AuxRR-core	GGTCCAT	572	7 +	Nicotiana tabacum	auxin responsive

TaMAPKKK108	LTR	CCGAAA	781	6 +	Hordeum vulgare	low temperature responsive
TaMAPKKK108	LTR	CCGAAA	856	6 -	Hordeum vulgare	low temperature responsive
TaMAPKKK108	LTR	CCGAAA	1620	6 +	Hordeum vulgare	low temperature responsive
TaMAPKKK108	CGTCA-motif	CGTCA	238	5 +	Hordeum vulgare	the MeJA responsive
TaMAPKKK108	CGTCA-motif	CGTCA	625	5 +	Hordeum vulgare	the MeJA responsive
TaMAPKKK108	GC-motif	CCCCCG	621	6 +	Zea mays	anoxic specific inducibility
TaMAPKKK108	GC-motif	CCCCCG	664	6 +	Zea mays	anoxic specific inducibility
TaMAPKKK108	GC-motif	CCCCCG	694	6 +	Zea mays	anoxic specific inducibility
TaMAPKKK108	MBS	CAACTG	23	6 -	Arabidopsis thaliana	drought inducibility
TaMAPKKK108	MBS	CAACTG	769	6 -	Arabidopsis thaliana	drought inducibility
TaMAPKKK108	ARE	AAACCA	1067	6 +	Zea mays	anaerobic induction
TaMAPKKK108	ARE	AAACCA	1161	6 -	Zea mays	anaerobic induction
TaMAPKKK108	TGA-element	AACGAC	1763	6 -	Brassica oleracea	auxin responsive
TaMAPKKK108	ABRE	ACGTG	47	5 -	Arabidopsis thaliana	abscisic acid responsive
TaMAPKKK108	ABRE	ACGTG	383	5 -	Arabidopsis thaliana	abscisic acid responsive
TaMAPKKK108	ABRE	ACGTG	1822	5 +	Arabidopsis thaliana	abscisic acid responsive
TaMAPKKK108	ABRE	GCAACGTGTC	1833	9 +	Hordeum vulgare	abscisic acid responsive
TaMAPKKK108	ABRE	ACGTG	1836	5 +	Arabidopsis thaliana	abscisic acid responsive
TaMAPKKK108	ABRE	ACGTG	1915	5 -	Arabidopsis thaliana	abscisic acid responsive

TaMAPKKK108	CAT-box	GCCACT	733	6 -	Arabidopsis thaliana	meristem expression
TaMAPKKK109	CGTCA-motif	CGTCA	345	5 +	Hordeum vulgare	the MeJA responsive
TaMAPKKK109	CGTCA-motif	CGTCA	479	5 -	Hordeum vulgare	the MeJA responsive
TaMAPKKK109	CGTCA-motif	CGTCA	712	5 +	Hordeum vulgare	the MeJA responsive
TaMAPKKK109	CGTCA-motif	CGTCA	860	5 +	Hordeum vulgare	the MeJA responsive
TaMAPKKK109	CGTCA-motif	CGTCA	1680	5 +	Hordeum vulgare	the MeJA responsive
TaMAPKKK109	GC-motif	CCCCCG	509	6 +	Zea mays	anoxic specific inducibility
TaMAPKKK109	GC-motif	CCCCCG	1056	6 +	Zea mays	anoxic specific inducibility
TaMAPKKK109	P-box	CCTTTTG	1209	7 +	Oryza sativa	gibberellin responsive
TaMAPKKK109	MBS	CAACTG	674	6 +	Arabidopsis thaliana	drought inducibility
TaMAPKKK109	MBS	CAACTG	680	6 -	Arabidopsis thaliana	drought inducibility
TaMAPKKK109	MBS	CAACTG	1937	6 +	Arabidopsis thaliana	drought inducibility
TaMAPKKK109	ARE	AAACCA	1019	6 -	Zea mays	anaerobic induction
TaMAPKKK109	ARE	AAACCA	1281	6 +	Zea mays	anaerobic induction
TaMAPKKK109	ARE	AAACCA	1383	6 -	Zea mays	anaerobic induction
TaMAPKKK109	ARE	AAACCA	1630	6 -	Zea mays	anaerobic induction
TaMAPKKK109	TGACG-motif	TGACG	345	5 -	Hordeum vulgare	the MeJA responsive
TaMAPKKK109	TGACG-motif	TGACG	479	5 +	Hordeum vulgare	the MeJA responsive
TaMAPKKK109	TGACG-motif	TGACG	712	5 -	Hordeum vulgare	the MeJA responsive

TaMAPKKK109	TGACG-motif	TGACG	860	5 -	Hordeum vulgare	the MeJA responsive
TaMAPKKK109	TGACG-motif	TGACG	1680	5 -	Hordeum vulgare	the MeJA responsive
TaMAPKKK109	ABRE	ACGTG	291	5 +	Arabidopsis thaliana	abscisic acid responsive
TaMAPKKK109	ABRE	ACGTG	1102	5 -	Arabidopsis thaliana	abscisic acid responsive
TaMAPKKK109	ABRE	GCCGCGTGGC	1854	9 -	Oryza sativa	abscisic acid responsive
TaMAPKKK109	CAT-box	GCCACT	565	6 -	Arabidopsis thaliana	meristem expression
TaMAPKKK11	LTR	CCGAAA	1495	6 +	Hordeum vulgare	low temperature responsive
TaMAPKKK11	TGACG-motif	TGACG	900	5 -	Hordeum vulgare	the MeJA responsive
TaMAPKKK11	ARE	AAACCA	727	6 +	Zea mays	anaerobic induction
TaMAPKKK11	ARE	AAACCA	1415	6 +	Zea mays	anaerobic induction
TaMAPKKK11	MBS	CAACTG	1012	6 +	Arabidopsis thaliana	drought inducibility
TaMAPKKK11	MBS	CAACTG	1571	6 +	Arabidopsis thaliana	drought inducibility
TaMAPKKK11	CGTCA-motif	CGTCA	900	5 +	Hordeum vulgare	the MeJA responsive
TaMAPKKK11	CAT-box	GCCACT	1119	6 +	Arabidopsis thaliana	meristem expression
TaMAPKKK11	O2-site	GATGATGTGG	444	10 -	Zea mays	zein metabolism regulation
TaMAPKKK11	O2-site	GATGATGTGG	644	9 -	Zea mays	zein metabolism regulation
TaMAPKKK11	O2-site	GATGA(C/T)(A/G)TG(A/G)	1040	8 -	Zea mays	zein metabolism regulation
TaMAPKKK11	O2-site	GATGATGTGG	1577	9 -	Zea mays	zein metabolism regulation
TaMAPKKK11	circadian	CAAAGATATC	1700	9 +	Lycopersicon esculentum	circadian control

TaMAPKKK11	P-box	CCTTTTG	1343	7 -	Oryza sativa	gibberellin responsive
TaMAPKKK11	ABRE	CACGTG	556	6 +	Arabidopsis thaliana	abscisic acid responsive
TaMAPKKK11	ABRE	ACGTG	557	5 +	Arabidopsis thaliana	abscisic acid responsive
TaMAPKKK11	ABRE	GACACGTGGC	1119	9 -	Triticum aestivum	abscisic acid responsive
TaMAPKKK110	GCN4_motif	TGAGTCA	262	7 -	Oryza sativa	endosperm expression
TaMAPKKK110	ABRE	AACCCGG	89	7 -	Arabidopsis thaliana	abscisic acid responsive
TaMAPKKK110	ABRE	ACGTG	195	5 -	Arabidopsis thaliana	abscisic acid responsive
TaMAPKKK110	ABRE	CACGTG	610	6 +	Arabidopsis thaliana	abscisic acid responsive
TaMAPKKK110	ABRE	ACGTG	611	5 +	Arabidopsis thaliana	abscisic acid responsive
TaMAPKKK110	TGA-element	AACGAC	219	6 -	Brassica oleracea	auxin responsive
TaMAPKKK110	CGTCA-motif	CGTCA	1523	5 -	Hordeum vulgare	the MeJA responsive
TaMAPKKK110	CGTCA-motif	CGTCA	1641	5 -	Hordeum vulgare	the MeJA responsive
TaMAPKKK110	P-box	CCTTTTG	156	7 +	Oryza sativa	gibberellin responsive
TaMAPKKK110	P-box	CCTTTTG	622	7 +	Oryza sativa	gibberellin responsive
TaMAPKKK110	MBS	CAACTG	915	6 -	Arabidopsis thaliana	drought inducibility
TaMAPKKK110	MBS	CAACTG	978	6 -	Arabidopsis thaliana	drought inducibility
TaMAPKKK110	GARE-motif	TCTGTTG	1543	7 +	Brassica oleracea	gibberellin responsive
TaMAPKKK110	TGACG-motif	TGACG	1523	5 +	Hordeum vulgare	the MeJA responsive
TaMAPKKK110	TGACG-motif	TGACG	1641	5 +	Hordeum vulgare	the MeJA responsive

TaMAPKKK110	ARE	AAACCA	903	6 -	Zea mays	anaerobic induction
TaMAPKKK110	ARE	AAACCA	1733	6 +	Zea mays	anaerobic induction
TaMAPKKK110	ARE	AAACCA	1958	6 +	Zea mays	anaerobic induction
TaMAPKKK110	ARE	AAACCA	1963	6 +	Zea mays	anaerobic induction
TaMAPKKK111	TC-rich repeats	ATTCTCTAAC	1199	9 -	Nicotiana tabacum	defense and stress responsive
TaMAPKKK111	TCA-element	CCATCTTTTT	79	9 +	Nicotiana tabacum	salicylic acid responsive
TaMAPKKK111	CGTCA-motif	CGTCA	1098	5 +	Hordeum vulgare	the MeJA responsive
TaMAPKKK111	ABRE	AACCCGG	115	7 +	Arabidopsis thaliana	abscisic acid responsive
TaMAPKKK111	ABRE	CACGTG	399	6 +	Arabidopsis thaliana	abscisic acid responsive
TaMAPKKK111	ABRE	ACGTG	400	5 +	Arabidopsis thaliana	abscisic acid responsive
TaMAPKKK111	ABRE	ACGTG	852	5 +	Arabidopsis thaliana	abscisic acid responsive
TaMAPKKK111	TGACG-motif	TGACG	1098	5 -	Hordeum vulgare	the MeJA responsive
TaMAPKKK111	AuxRE	TGTCTCAATA AG	797	11 -	Glycine max	auxin responsive
TaMAPKKK112	CAT-box	GCCACT	799	6 +	Arabidopsis thaliana	meristem expression
TaMAPKKK112	ARE	AAACCA	849	6 +	Zea mays	anaerobic induction
TaMAPKKK112	TC-rich repeats	ATTCTCTAAC	671	9 -	Nicotiana tabacum	defense and stress responsive
TaMAPKKK112	TGACG-motif	TGACG	190	5 +	Hordeum vulgare	the MeJA responsive
TaMAPKKK112	TGACG-motif	TGACG	1749	5 +	Hordeum vulgare	the MeJA responsive
TaMAPKKK112	TGACG-motif	TGACG	1844	5 -	Hordeum vulgare	the MeJA responsive

TaMAPKKK112	CGTCA-motif	CGTCA	190	5 -	Hordeum vulgare	the MeJA responsive
TaMAPKKK112	CGTCA-motif	CGTCA	1749	5 -	Hordeum vulgare	the MeJA responsive
TaMAPKKK112	CGTCA-motif	CGTCA	1844	5 +	Hordeum vulgare	the MeJA responsive
TaMAPKKK112	MBS	CAACTG	563	6 -	Arabidopsis thaliana	drought inducibility
TaMAPKKK112	MBS	CAACTG	1490	6 -	Arabidopsis thaliana	drought inducibility
TaMAPKKK112	P-box	CCTTTTG	495	7 +	Oryza sativa	gibberellin responsive
TaMAPKKK112	TCA-element	CCATCTTTTT	1516	9 -	Nicotiana tabacum	salicylic acid responsive
TaMAPKKK113	TGACG-motif	TGACG	861	5 +	Hordeum vulgare	the MeJA responsive
TaMAPKKK113	TGACG-motif	TGACG	1238	5 +	Hordeum vulgare	the MeJA responsive
TaMAPKKK113	TGACG-motif	TGACG	1636	5 -	Hordeum vulgare	the MeJA responsive
TaMAPKKK113	ARE	AAACCA	1177	6 -	Zea mays	anaerobic induction
TaMAPKKK113	ARE	AAACCA	1376	6 +	Zea mays	anaerobic induction
TaMAPKKK113	ABRE	ACGTG	318	5 -	Arabidopsis thaliana	abscisic acid responsive
TaMAPKKK113	ABRE	ACGTG	919	5 -	Arabidopsis thaliana	abscisic acid responsive
TaMAPKKK113	ABRE	ACGTG	1044	5 -	Arabidopsis thaliana	abscisic acid responsive
TaMAPKKK113	ABRE	ACGTG	1240	5 +	Arabidopsis thaliana	abscisic acid responsive
TaMAPKKK113	ABRE	CACGTG	1626	6 -	Arabidopsis thaliana	abscisic acid responsive
TaMAPKKK113	ABRE	ACGTG	1627	5 +	Arabidopsis thaliana	abscisic acid responsive
TaMAPKKK113	ABRE	ACGTG	1775	5 -	Arabidopsis thaliana	abscisic acid responsive

TaMAPKKK113	TGA-element	AACGAC	1013	6 +	Brassica oleracea	auxin responsive
TaMAPKKK113	CGTCA-motif	CGTCA	861	5 -	Hordeum vulgare	the MeJA responsive
TaMAPKKK113	CGTCA-motif	CGTCA	1238	5 -	Hordeum vulgare	the MeJA responsive
TaMAPKKK113	CGTCA-motif	CGTCA	1636	5 +	Hordeum vulgare	the MeJA responsive
TaMAPKKK113	P-box	CCTTTTG	148	7 -	Oryza sativa	gibberellin responsive
TaMAPKKK113	P-box	CCTTTTG	298	7 -	Oryza sativa	gibberellin responsive
TaMAPKKK113	MBS	CAACTG	1222	6 +	Arabidopsis thaliana	drought inducibility
TaMAPKKK113	TC-rich repeats	GTTTTCTTAC	196	9 +	Nicotiana tabacum	defense and stress responsive
TaMAPKKK113	TC-rich repeats	GTTTTCTTAC	402	9 +	Nicotiana tabacum	defense and stress responsive
TaMAPKKK113	TC-rich repeats	ATTCTCTAAC	634	9 +	Nicotiana tabacum	defense and stress responsive
TaMAPKKK113	TCA-element	CCATCTTTTT	846	9 -	Nicotiana tabacum	salicylic acid responsive
TaMAPKKK113	TCA-element	CCATCTTTTT	1049	9 +	Nicotiana tabacum	salicylic acid responsive
TaMAPKKK113	O2-site	GATGATGTGG	1891	9 +	Zea mays	zein metabolism regulation
TaMAPKKK113	GC-motif	CCCCCG	1581	6 -	Zea mays	anoxic specific inducibility
TaMAPKKK115	ABRE	ACGTG	319	5 -	Arabidopsis thaliana	abscisic acid responsive
TaMAPKKK115	TGA-element	AACGAC	1482	6 +	Brassica oleracea	auxin responsive
TaMAPKKK115	TGA-element	AACGAC	1771	6 +	Brassica oleracea	auxin responsive
TaMAPKKK115	TGA-element	AACGAC	1972	6 +	Brassica oleracea	auxin responsive
TaMAPKKK115	TGACG-motif	TGACG	39	5 +	Hordeum vulgare	the MeJA responsive
TaMAPKKK115	ARE	AAACCA	219	6 -	Zea mays	anaerobic induction
TaMAPKKK115	O2-site	GATGA(C/T)(A/G)TG(A/G)	1290	8 +	Zea mays	zein metabolism regulation

TaMAPKKK115	TCA-element	CCATCTTTTT	139	9 +	Nicotiana tabacum	salicylic acid responsive
TaMAPKKK115	LTR	CCGAAA	1213	6 -	Hordeum vulgare	low temperature responsive
TaMAPKKK115	LTR	CCGAAA	1310	6 -	Hordeum vulgare	low temperature responsive
TaMAPKKK115	CGTCA-motif	CGTCA	39	5 -	Hordeum vulgare	the MeJA responsive
TaMAPKKK116	ABRE	CACGTG	68	6 +	Arabidopsis thaliana	abscisic acid responsive
TaMAPKKK116	ABRE	ACGTG	69	5 +	Arabidopsis thaliana	abscisic acid responsive
TaMAPKKK116	ABRE	CACGTG	99	6 +	Arabidopsis thaliana	abscisic acid responsive
TaMAPKKK116	ABRE	ACGTG	100	5 +	Arabidopsis thaliana	abscisic acid responsive
TaMAPKKK116	ABRE	GCAACGTGTC	191	9 +	Hordeum vulgare	abscisic acid responsive
TaMAPKKK116	ABRE	CACGTG	193	6 +	Arabidopsis thaliana	abscisic acid responsive
TaMAPKKK116	ABRE	ACGTG	194	5 +	Arabidopsis thaliana	abscisic acid responsive
TaMAPKKK116	ABRE	ACGTG	389	5 -	Arabidopsis thaliana	abscisic acid responsive
TaMAPKKK116	ABRE	ACGTG	723	5 +	Arabidopsis thaliana	abscisic acid responsive
TaMAPKKK116	ABRE	GACACGTGGC	935	9 -	Triticum aestivum	abscisic acid responsive
TaMAPKKK116	ABRE	CACGTG	937	6 +	Arabidopsis thaliana	abscisic acid responsive
TaMAPKKK116	ABRE	ACGTG	938	5 +	Arabidopsis thaliana	abscisic acid responsive
TaMAPKKK116	ABRE	ACGTG	1184	5 +	Arabidopsis thaliana	abscisic acid responsive
TaMAPKKK116	ABRE	ACGTG	1580	5 +	Arabidopsis thaliana	abscisic acid responsive
TaMAPKKK116	ABRE	ACGTG	1651	5 +	Arabidopsis thaliana	abscisic acid responsive

TaMAPKKK116	GC-motif	CCCCCG	485	6 +	Zea mays	anoxic specific inducibility
TaMAPKKK116	GC-motif	CCCCCG	1172	6 -	Zea mays	anoxic specific inducibility
TaMAPKKK116	CAT-box	GCCACT	682	6 -	Arabidopsis thaliana	meristem expression
TaMAPKKK116	CAT-box	GCCACT	852	6 +	Arabidopsis thaliana	meristem expression
TaMAPKKK116	CGTCA-motif	CGTCA	210	5 -	Hordeum vulgare	the MeJA responsive
TaMAPKKK116	CGTCA-motif	CGTCA	227	5 -	Hordeum vulgare	the MeJA responsive
TaMAPKKK116	CGTCA-motif	CGTCA	242	5 +	Hordeum vulgare	the MeJA responsive
TaMAPKKK116	CGTCA-motif	CGTCA	341	5 +	Hordeum vulgare	the MeJA responsive
TaMAPKKK116	CGTCA-motif	CGTCA	505	5 -	Hordeum vulgare	the MeJA responsive
TaMAPKKK116	CGTCA-motif	CGTCA	721	5 -	Hordeum vulgare	the MeJA responsive
TaMAPKKK116	CGTCA-motif	CGTCA	969	5 +	Hordeum vulgare	the MeJA responsive
TaMAPKKK116	MBS	CAACTG	1227	6 -	Arabidopsis thaliana	drought inducibility
TaMAPKKK116	RY-element	CATGCATG	354	8 +	Helianthus annuus	seed specific regulation
TaMAPKKK116	ARE	AAACCA	1483	6 -	Zea mays	anaerobic induction
TaMAPKKK116	TGACG-motif	TGACG	210	5 +	Hordeum vulgare	the MeJA responsive
TaMAPKKK116	TGACG-motif	TGACG	227	5 +	Hordeum vulgare	the MeJA responsive
TaMAPKKK116	TGACG-motif	TGACG	242	5 -	Hordeum vulgare	the MeJA responsive
TaMAPKKK116	TGACG-motif	TGACG	341	5 -	Hordeum vulgare	the MeJA responsive
TaMAPKKK116	TGACG-motif	TGACG	505	5 +	Hordeum vulgare	the MeJA responsive

TaMAPKKK116	TGACG-motif	TGACG	721	5 +	Hordeum vulgare	the MeJA responsive
TaMAPKKK116	TGACG-motif	TGACG	969	5 -	Hordeum vulgare	the MeJA responsive
TaMAPKKK116	LTR	CCGAAA	1101	6 -	Hordeum vulgare	low temperature responsive
TaMAPKKK116	LTR	CCGAAA	1143	6 -	Hordeum vulgare	low temperature responsive
TaMAPKKK117	TGA-element	AACGAC	506	6 -	Brassica oleracea	auxin responsive
TaMAPKKK117	GARE-motif	TCTGTTG	1743	7 +	Brassica oleracea	gibberellin responsive
TaMAPKKK117	ABRE	ACGTG	265	5 +	Arabidopsis thaliana	abscisic acid responsive
TaMAPKKK117	ABRE	ACGTG	476	5 -	Arabidopsis thaliana	abscisic acid responsive
TaMAPKKK117	ABRE	ACGTG	688	5 +	Arabidopsis thaliana	abscisic acid responsive
TaMAPKKK117	ABRE	ACGTG	1244	5 -	Arabidopsis thaliana	abscisic acid responsive
TaMAPKKK117	ABRE	ACGTG	1648	5 -	Arabidopsis thaliana	abscisic acid responsive
TaMAPKKK117	ABRE	CACGTG	1762	6 -	Arabidopsis thaliana	abscisic acid responsive
TaMAPKKK117	ABRE	ACGTG	1763	5 +	Arabidopsis thaliana	abscisic acid responsive
TaMAPKKK117	ABRE	ACGTG	1768	5 +	Arabidopsis thaliana	abscisic acid responsive
TaMAPKKK117	ARE	AAACCA	764	6 -	Zea mays	anaerobic induction
TaMAPKKK117	TGACG-motif	TGACG	680	5 -	Hordeum vulgare	the MeJA responsive
TaMAPKKK117	TGACG-motif	TGACG	833	5 +	Hordeum vulgare	the MeJA responsive
TaMAPKKK117	TGACG-motif	TGACG	1077	5 +	Hordeum vulgare	the MeJA responsive
TaMAPKKK117	TGACG-motif	TGACG	1766	5 +	Hordeum vulgare	the MeJA responsive

TaMAPKKK117	GC-motif	CCCCCG	445	6 +	Zea mays	anoxic specific inducibility
TaMAPKKK117	O2-site	GATGATGTGG	1419	10 -	Zea mays	zein metabolism regulation
TaMAPKKK117	TCA-element	CCATCTTTTT	864	9 -	Nicotiana tabacum	salicylic acid responsive
TaMAPKKK117	P-box	CCTTTTG	1869	7 +	Oryza sativa	gibberellin responsive
TaMAPKKK117	AuxRR-core	GGTCCAT	51	7 -	Nicotiana tabacum	auxin responsive
TaMAPKKK117	CGTCA-motif	CGTCA	680	5 +	Hordeum vulgare	the MeJA responsive
TaMAPKKK117	CGTCA-motif	CGTCA	833	5 -	Hordeum vulgare	the MeJA responsive
TaMAPKKK117	CGTCA-motif	CGTCA	1077	5 -	Hordeum vulgare	the MeJA responsive
TaMAPKKK117	CGTCA-motif	CGTCA	1766	5 -	Hordeum vulgare	the MeJA responsive
TaMAPKKK119	TGACG-motif	TGACG	1892	5 -	Hordeum vulgare	the MeJA responsive
TaMAPKKK119	TGACG-motif	TGACG	1900	5 -	Hordeum vulgare	the MeJA responsive
TaMAPKKK119	LTR	CCGAAA	1244	6 -	Hordeum vulgare	low temperature responsive
TaMAPKKK119	CAT-box	GCCACT	979	6 -	Arabidopsis thaliana	meristem expression
TaMAPKKK119	CGTCA-motif	CGTCA	1892	5 +	Hordeum vulgare	the MeJA responsive
TaMAPKKK119	CGTCA-motif	CGTCA	1900	5 +	Hordeum vulgare	the MeJA responsive
TaMAPKKK119	ABRE	ACGTG	1131	5 +	Arabidopsis thaliana	abscisic acid responsive
TaMAPKKK119	ABRE	CACGTG	1228	6 -	Arabidopsis thaliana	abscisic acid responsive
TaMAPKKK119	ABRE	ACGTG	1229	5 +	Arabidopsis thaliana	abscisic acid responsive
TaMAPKKK119	ABRE	ACGTG	1365	5 +	Arabidopsis thaliana	abscisic acid responsive

TaMAPKKK119	ABRE	ACGTG	1382	5 -	Arabidopsis thaliana	abscisic acid responsive
TaMAPKKK119	ABRE	ACGTG	1399	5 +	Arabidopsis thaliana	abscisic acid responsive
TaMAPKKK119	ABRE	GCCGCGTGGC	1599	9 -	Oryza sativa	abscisic acid responsive
TaMAPKKK119	GC-motif	CCCCCG	1351	6 -	Zea mays	anoxic specific inducibility
TaMAPKKK119	GC-motif	CCCCCG	1840	6 +	Zea mays	anoxic specific inducibility
TaMAPKKK119	MBS	CAACTG	251	6 +	Arabidopsis thaliana	drought inducibility
TaMAPKKK119	MBS	CAACTG	312	6 -	Arabidopsis thaliana	drought inducibility
TaMAPKKK119	MBS	CAACTG	927	6 +	Arabidopsis thaliana	drought inducibility
TaMAPKKK119	MBS	CAACTG	1289	6 -	Arabidopsis thaliana	drought inducibility
TaMAPKKK12	O2-site	GTTGACGTGA	1377	9 +	Zea mays	zein metabolism regulation
TaMAPKKK12	TGACG-motif	TGACG	1142	5 -	Hordeum vulgare	the MeJA responsive
TaMAPKKK12	TGACG-motif	TGACG	1379	5 +	Hordeum vulgare	the MeJA responsive
TaMAPKKK12	TGACG-motif	TGACG	1688	5 +	Hordeum vulgare	the MeJA responsive
TaMAPKKK12	MBS	CAACTG	64	6 +	Arabidopsis thaliana	drought inducibility
TaMAPKKK12	MBS	CAACTG	143	6 +	Arabidopsis thaliana	drought inducibility
TaMAPKKK12	GARE-motif	TCTGTTG	43	7 -	Brassica oleracea	gibberellin responsive
TaMAPKKK12	GC-motif	CCCCCG	1418	6 +	Zea mays	anoxic specific inducibility
TaMAPKKK12	GC-motif	CCCCCG	1931	6 +	Zea mays	anoxic specific inducibility
TaMAPKKK12	GC-motif	CCCCCG	1983	6 +	Zea mays	anoxic specific inducibility

TaMAPKKK12	ARE	AAACCA	1780	6 +	Zea mays	anaerobic induction
TaMAPKKK12	CAT-box	GCCACT	1302	6 +	Arabidopsis thaliana	meristem expression
TaMAPKKK12	MSA-like	TCAAACGGT	972	9 -	Catharanthus roseus	cell cycle regulation
TaMAPKKK12	CGTCA-motif	CGTCA	1142	5 +	Hordeum vulgare	the MeJA responsive
TaMAPKKK12	CGTCA-motif	CGTCA	1379	5 -	Hordeum vulgare	the MeJA responsive
TaMAPKKK12	CGTCA-motif	CGTCA	1688	5 -	Hordeum vulgare	the MeJA responsive
TaMAPKKK12	LTR	CCGAAA	283	6 +	Hordeum vulgare	low temperature responsive
TaMAPKKK12	ABRE	AACCCGG	348	7 +	Arabidopsis thaliana	abscisic acid responsive
TaMAPKKK12	ABRE	AACCCGG	409	7 -	Arabidopsis thaliana	abscisic acid responsive
TaMAPKKK12	ABRE	ACGTG	1176	5 +	Arabidopsis thaliana	abscisic acid responsive
TaMAPKKK12	ABRE	ACGTG	1343	5 +	Arabidopsis thaliana	abscisic acid responsive
TaMAPKKK12	ABRE	ACGTG	1381	5 +	Arabidopsis thaliana	abscisic acid responsive
TaMAPKKK12	ABRE	ACGTG	1514	5 +	Arabidopsis thaliana	abscisic acid responsive
TaMAPKKK120	CGTCA-motif	CGTCA	156	5 -	Hordeum vulgare	the MeJA responsive
TaMAPKKK120	CGTCA-motif	CGTCA	816	5 -	Hordeum vulgare	the MeJA responsive
TaMAPKKK120	CGTCA-motif	CGTCA	1469	5 -	Hordeum vulgare	the MeJA responsive
TaMAPKKK120	TGA-element	AACGAC	286	6 +	Brassica oleracea	auxin responsive
TaMAPKKK120	TGA-element	AACGAC	744	6 -	Brassica oleracea	auxin responsive
TaMAPKKK120	ABRE	ACGTG	378	5 -	Arabidopsis thaliana	abscisic acid responsive
TaMAPKKK120	ABRE	ACGTG	843	5 -	Arabidopsis thaliana	abscisic acid responsive

TaMAPKKK120	ABRE	ACGTG	1069	5 -	Arabidopsis thaliana	abscisic acid responsive
TaMAPKKK120	ABRE	GCCGCGTGGC	1831	9 -	Oryza sativa	abscisic acid responsive
TaMAPKKK120	TGACG-motif	TGACG	156	5 +	Hordeum vulgare	the MeJA responsive
TaMAPKKK120	TGACG-motif	TGACG	816	5 +	Hordeum vulgare	the MeJA responsive
TaMAPKKK120	TGACG-motif	TGACG	1469	5 +	Hordeum vulgare	the MeJA responsive
TaMAPKKK120	ARE	AAACCA	1714	6 +	Zea mays	anaerobic induction
TaMAPKKK120	ARE	AAACCA	1739	6 -	Zea mays	anaerobic induction
TaMAPKKK120	GC-motif	CCCCCG	1619	6 -	Zea mays	anoxic specific inducibility
TaMAPKKK121	TGACG-motif	TGACG	24	5 +	Hordeum vulgare	the MeJA responsive
TaMAPKKK121	TGACG-motif	TGACG	1067	5 +	Hordeum vulgare	the MeJA responsive
TaMAPKKK121	TGACG-motif	TGACG	1597	5 +	Hordeum vulgare	the MeJA responsive
TaMAPKKK121	TGACG-motif	TGACG	1812	5 +	Hordeum vulgare	the MeJA responsive
TaMAPKKK121	ARE	AAACCA	532	6 -	Zea mays	anaerobic induction
TaMAPKKK121	ABRE	ACGTG	402	5 -	Arabidopsis thaliana	abscisic acid responsive
TaMAPKKK121	ABRE	ACGTG	549	5 +	Arabidopsis thaliana	abscisic acid responsive
TaMAPKKK121	ABRE	AACCCGG	1096	7 -	Arabidopsis thaliana	abscisic acid responsive
TaMAPKKK121	ABRE	GCAACGTGTC	1346	9 -	Hordeum vulgare	abscisic acid responsive
TaMAPKKK121	ABRE	ACGTG	1348	5 -	Arabidopsis thaliana	abscisic acid responsive
TaMAPKKK121	TGA-element	AACGAC	1343	6 +	Brassica oleracea	auxin responsive

TaMAPKKK121	LTR	CCGAAA	1589	6 +	Hordeum vulgare	low temperature responsive
TaMAPKKK121	CGTCA-motif	CGTCA	24	5 -	Hordeum vulgare	the MeJA responsive
TaMAPKKK121	CGTCA-motif	CGTCA	1067	5 -	Hordeum vulgare	the MeJA responsive
TaMAPKKK121	CGTCA-motif	CGTCA	1597	5 -	Hordeum vulgare	the MeJA responsive
TaMAPKKK121	CGTCA-motif	CGTCA	1812	5 -	Hordeum vulgare	the MeJA responsive
TaMAPKKK121	MBS	CAACTG	1235	6 +	Arabidopsis thaliana	drought inducibility
TaMAPKKK121	GCN4_motif	TGAGTCA	426	7 +	Oryza sativa	endosperm expression
TaMAPKKK121	TCA-element	CCATCTTTTT	1529	9 +	Nicotiana tabacum	salicylic acid responsive
TaMAPKKK122	CAT-box	GCCACT	520	6 -	Arabidopsis thaliana	meristem expression
TaMAPKKK122	CGTCA-motif	CGTCA	973	5 +	Hordeum vulgare	the MeJA responsive
TaMAPKKK122	CGTCA-motif	CGTCA	1541	5 +	Hordeum vulgare	the MeJA responsive
TaMAPKKK122	ABRE	ACGTG	879	5 +	Arabidopsis thaliana	abscisic acid responsive
TaMAPKKK122	ABRE	GCCGCGTGGC	1738	9 -	Oryza sativa	abscisic acid responsive
TaMAPKKK122	TC-rich repeats	GTTTTCTTAC	1495	9 +	Nicotiana tabacum	defense and stress responsive
TaMAPKKK122	MBS	CAACTG	1940	6 +	Arabidopsis thaliana	drought inducibility
TaMAPKKK122	TGACG-motif	TGACG	973	5 -	Hordeum vulgare	the MeJA responsive
TaMAPKKK122	TGACG-motif	TGACG	1541	5 -	Hordeum vulgare	the MeJA responsive
TaMAPKKK122	ARE	AAACCA	1433	6 -	Zea mays	anaerobic induction
TaMAPKKK122	GARE-motif	TCTGTTG	425	7 -	Brassica oleracea	gibberellin responsive

TaMAPKKK123	MBS	CAACTG	1105	6 +	Arabidopsis thaliana	drought inducibility
TaMAPKKK123	TCA-element	CCATCTTTTT	696	9 +	Nicotiana tabacum	salicylic acid responsive
TaMAPKKK123	P-box	CCTTTTG	249	7 +	Oryza sativa	gibberellin responsive
TaMAPKKK123	ARE	AAACCA	270	6 +	Zea mays	anaerobic induction
TaMAPKKK123	ARE	AAACCA	1080	6 +	Zea mays	anaerobic induction
TaMAPKKK123	ARE	AAACCA	1878	6 +	Zea mays	anaerobic induction
TaMAPKKK123	GCN4_motif	TGAGTCA	402	7 -	Oryza sativa	endosperm expression
TaMAPKKK123	TC-rich repeats	ATTCTCTAAC	1426	9 -	Nicotiana tabacum	defense and stress responsive
TaMAPKKK123	TGACG-motif	TGACG	567	5 -	Hordeum vulgare	the MeJA responsive
TaMAPKKK123	TGACG-motif	TGACG	1629	5 +	Hordeum vulgare	the MeJA responsive
TaMAPKKK123	TGACG-motif	TGACG	1692	5 +	Hordeum vulgare	the MeJA responsive
TaMAPKKK123	TGACG-motif	TGACG	1874	5 -	Hordeum vulgare	the MeJA responsive
TaMAPKKK123	TGACG-motif	TGACG	1991	5 +	Hordeum vulgare	the MeJA responsive
TaMAPKKK123	CGTCA-motif	CGTCA	567	5 +	Hordeum vulgare	the MeJA responsive
TaMAPKKK123	CGTCA-motif	CGTCA	1629	5 -	Hordeum vulgare	the MeJA responsive
TaMAPKKK123	CGTCA-motif	CGTCA	1692	5 -	Hordeum vulgare	the MeJA responsive
TaMAPKKK123	CGTCA-motif	CGTCA	1874	5 +	Hordeum vulgare	the MeJA responsive
TaMAPKKK123	CGTCA-motif	CGTCA	1991	5 -	Hordeum vulgare	the MeJA responsive
TaMAPKKK123-1	P-box	CCTTTTG	919	7 +	Oryza sativa	gibberellin responsive

TaMAPKKK123-1	TATC-box	TATCCCA	52	7 -	Oryza sativa	gibberellin responsive
TaMAPKKK123-1	MBS	CAACTG	1506	6 -	Arabidopsis thaliana	drought inducibility
TaMAPKKK123-1	GC-motif	CCCCCG	1922	6 -	Zea mays	anoxic specific inducibility
TaMAPKKK123-1	TGA-element	AACGAC	1355	6 +	Brassica oleracea	auxin responsive
TaMAPKKK123-1	ABRE	ACGTG	454	5 +	Arabidopsis thaliana	abscisic acid responsive
TaMAPKKK123-1	ABRE	ACGTG	1644	5 +	Arabidopsis thaliana	abscisic acid responsive
TaMAPKKK123-1	LTR	CCGAAA	771	6 -	Hordeum vulgare	low temperature responsive
TaMAPKKK123-1	CGTCA-motif	CGTCA	432	5 -	Hordeum vulgare	the MeJA responsive
TaMAPKKK123-1	CGTCA-motif	CGTCA	1408	5 -	Hordeum vulgare	the MeJA responsive
TaMAPKKK123-1	CGTCA-motif	CGTCA	1708	5 +	Hordeum vulgare	the MeJA responsive
TaMAPKKK123-1	TGACG-motif	TGACG	432	5 +	Hordeum vulgare	the MeJA responsive
TaMAPKKK123-1	TGACG-motif	TGACG	1408	5 +	Hordeum vulgare	the MeJA responsive
TaMAPKKK123-1	TGACG-motif	TGACG	1708	5 -	Hordeum vulgare	the MeJA responsive
TaMAPKKK125	ARE	AAACCA	229	6 +	Zea mays	anaerobic induction
TaMAPKKK125	ARE	AAACCA	773	6 +	Zea mays	anaerobic induction
TaMAPKKK125	ARE	AAACCA	1018	6 +	Zea mays	anaerobic induction
TaMAPKKK125	ABRE	ACGTG	27	5 +	Arabidopsis thaliana	abscisic acid responsive
TaMAPKKK125	ABRE	ACGTG	1595	5 -	Arabidopsis thaliana	abscisic acid responsive
TaMAPKKK125	ABRE	ACGTG	1638	5 -	Arabidopsis thaliana	abscisic acid responsive

TaMAPKKK125	O2-site	GTTGACGTGA	891	9 -	Zea mays	zein metabolism regulation
TaMAPKKK125	CGTCA-motif	CGTCA	1158	5 +	Hordeum vulgare	the MeJA responsive
TaMAPKKK125	TGACG-motif	TGACG	1158	5 -	Hordeum vulgare	the MeJA responsive
TaMAPKKK125	LTR	CCGAAA	1201	6 +	Hordeum vulgare	low temperature responsive
TaMAPKKK125	LTR	CCGAAA	1816	6 -	Hordeum vulgare	low temperature responsive
TaMAPKKK125	MBS	CAACTG	511	6 +	Arabidopsis thaliana	drought inducibility
TaMAPKKK125	WUN-motif	AAATTTCCT	1109	9 +	Brassica oleracea	wound responsive
TaMAPKKK126	TGACG-motif	TGACG	447	5 -	Hordeum vulgare	the MeJA responsive
TaMAPKKK126	TGACG-motif	TGACG	1852	5 +	Hordeum vulgare	the MeJA responsive
TaMAPKKK126	TGA-element	AACGAC	1585	6 +	Brassica oleracea	auxin responsive
TaMAPKKK126	CAT-box	GCCACT	451	6 -	Arabidopsis thaliana	meristem expression
TaMAPKKK126	GARE-motif	TCTGTTG	1621	7 +	Brassica oleracea	gibberellin responsive
TaMAPKKK126	CGTCA-motif	CGTCA	447	5 +	Hordeum vulgare	the MeJA responsive
TaMAPKKK126	CGTCA-motif	CGTCA	1852	5 -	Hordeum vulgare	the MeJA responsive
TaMAPKKK126	P-box	CCTTTTG	265	7 -	Oryza sativa	gibberellin responsive
TaMAPKKK126	ABRE	ACGTG	445	5 -	Arabidopsis thaliana	abscisic acid responsive
TaMAPKKK126	ABRE	CACGTG	1453	6 -	Arabidopsis thaliana	abscisic acid responsive
TaMAPKKK126	ABRE	ACGTG	1454	5 +	Arabidopsis thaliana	abscisic acid responsive
TaMAPKKK126	ARE	AAACCA	760	6 +	Zea mays	anaerobic induction

TaMAPKKK126	ARE	AAACCA	1319	6 +	Zea mays	anaerobic induction
TaMAPKKK126	TCA-element	TCAGAAGAGG	501	9 +	Brassica oleracea	salicylic acid responsive
TaMAPKKK126	O2-site	GATGACATGG	1654	9 -	Zea mays	zein metabolism regulation
TaMAPKKK126	O2-site	GATGA(C/T)(A/G)TG(A/G)	1659	8 -	Zea mays	zein metabolism regulation
TaMAPKKK126	MBS	CAACTG	727	6 +	Arabidopsis thaliana	drought inducibility
TaMAPKKK126	MBS	CAACTG	998	6 -	Arabidopsis thaliana	drought inducibility
TaMAPKKK126	LTR	CCGAAA	1678	6 +	Hordeum vulgare	low temperature responsive
TaMAPKKK127	MSA-like	(T/C)C(T/C)AACGG(T/C)(T/C)A	1152	8.5 +	Catharanthus roseus	cell cycle regulation
TaMAPKKK127	MSA-like	(T/C)C(T/C)AACGG(T/C)(T/C)A	1210	8.5 +	Catharanthus roseus	cell cycle regulation
TaMAPKKK127	ARE	AAACCA	212	6 -	Zea mays	anaerobic induction
TaMAPKKK127	CGTCA-motif	CGTCA	54	5 +	Hordeum vulgare	the MeJA responsive
TaMAPKKK127	CGTCA-motif	CGTCA	553	5 -	Hordeum vulgare	the MeJA responsive
TaMAPKKK127	CGTCA-motif	CGTCA	976	5 -	Hordeum vulgare	the MeJA responsive
TaMAPKKK127	CGTCA-motif	CGTCA	1376	5 +	Hordeum vulgare	the MeJA responsive
TaMAPKKK127	TC-rich repeats	GTTTTCTTAC	214	9 +	Nicotiana tabacum	defense and stress responsive
TaMAPKKK127	TGACG-motif	TGACG	54	5 -	Hordeum vulgare	the MeJA responsive
TaMAPKKK127	TGACG-motif	TGACG	553	5 +	Hordeum vulgare	the MeJA responsive
TaMAPKKK127	TGACG-motif	TGACG	976	5 +	Hordeum vulgare	the MeJA responsive

TaMAPKKK127	TGACG-motif	TGACG	1376	5 -	Hordeum vulgare	the MeJA responsive
TaMAPKKK127	TGA-element	AACGAC	1868	6 -	Brassica oleracea	auxin responsive
TaMAPKKK127	ABRE	ACGTG	599	5 -	Arabidopsis thaliana	abscisic acid responsive
TaMAPKKK127	TCA-element	TCAGAAGAGG	1124	9 +	Brassica oleracea	salicylic acid responsive
TaMAPKKK127	circadian	CAAAGATATC	827	9 +	Lycopersicon esculentum	circadian control
TaMAPKKK127	circadian	CAAAGATATC	1102	9 +	Lycopersicon esculentum	circadian control
TaMAPKKK127	MBS	CAACTG	564	6 +	Arabidopsis thaliana	drought inducibility
TaMAPKKK127	GARE-motif	TCTGTTG	415	7 -	Brassica oleracea	gibberellin responsive
TaMAPKKK128	MBS	CAACTG	70	6 +	Arabidopsis thaliana	drought inducibility
TaMAPKKK128	MBS	CAACTG	133	6 -	Arabidopsis thaliana	drought inducibility
TaMAPKKK128	CAT-box	GCCACT	165	6 -	Arabidopsis thaliana	meristem expression
TaMAPKKK128	CAT-box	GCCACT	251	6 -	Arabidopsis thaliana	meristem expression
TaMAPKKK128	CGTCA-motif	CGTCA	1175	5 -	Hordeum vulgare	the MeJA responsive
TaMAPKKK128	CGTCA-motif	CGTCA	1613	5 -	Hordeum vulgare	the MeJA responsive
TaMAPKKK128	CGTCA-motif	CGTCA	1659	5 +	Hordeum vulgare	the MeJA responsive
TaMAPKKK128	MSA-like	TCAAACGGT	1596	9 +	Catharanthus roseus	cell cycle regulation
TaMAPKKK128	ABRE	ACGTG	312	5 +	Arabidopsis thaliana	abscisic acid responsive
TaMAPKKK128	ABRE	ACGTG	356	5 +	Arabidopsis thaliana	abscisic acid responsive
TaMAPKKK128	ABRE	CACGTG	438	6 +	Arabidopsis thaliana	abscisic acid responsive

TaMAPKKK128	ABRE	ACGTG	439	5 +	Arabidopsis thaliana	abscisic acid responsive
TaMAPKKK128	ABRE	GACACGTACGT	1177	10 -	Oryza sativa	abscisic acid responsive
TaMAPKKK128	ABRE	CGCACGTGTC	1178	9 +	Hordeum vulgare	abscisic acid responsive
TaMAPKKK128	ABRE	ACGTG	1181	5 +	Arabidopsis thaliana	abscisic acid responsive
TaMAPKKK128	ABRE	CGTACGTGCA	1287	9 +	Hordeum vulgare	abscisic acid responsive
TaMAPKKK128	ABRE	ACGTG	1380	5 -	Arabidopsis thaliana	abscisic acid responsive
TaMAPKKK128	GC-motif	CCCCCG	1842	6 +	Zea mays	anoxic specific inducibility
TaMAPKKK128	P-box	CCTTTTG	393	7 -	Oryza sativa	gibberellin responsive
TaMAPKKK128	LTR	CCGAAA	459	6 +	Hordeum vulgare	low temperature responsive
TaMAPKKK128	LTR	CCGAAA	605	6 -	Hordeum vulgare	low temperature responsive
TaMAPKKK128	O2-site	GATGATGTGG	715	9 +	Zea mays	zein metabolism regulation
TaMAPKKK128	TGACG-motif	TGACG	1175	5 +	Hordeum vulgare	the MeJA responsive
TaMAPKKK128	TGACG-motif	TGACG	1613	5 +	Hordeum vulgare	the MeJA responsive
TaMAPKKK128	TGACG-motif	TGACG	1659	5 -	Hordeum vulgare	the MeJA responsive
TaMAPKKK128	TCA-element	CCATCTTTTT	60	9 -	Nicotiana tabacum	salicylic acid responsive
TaMAPKKK129	ARE	AAACCA	668	6 -	Zea mays	anaerobic induction
TaMAPKKK129	ARE	AAACCA	701	6 +	Zea mays	anaerobic induction
TaMAPKKK129	LTR	CCGAAA	1384	6 -	Hordeum vulgare	low temperature responsive
TaMAPKKK129	LTR	CCGAAA	1965	6 +	Hordeum vulgare	low temperature responsive

TaMAPKKK129	CGTCA-motif	CGTCA	1095	5 +	Hordeum vulgare	the MeJA responsive
TaMAPKKK129	CGTCA-motif	CGTCA	1574	5 -	Hordeum vulgare	the MeJA responsive
TaMAPKKK129	TGACG-motif	TGACG	1095	5 -	Hordeum vulgare	the MeJA responsive
TaMAPKKK129	TGACG-motif	TGACG	1574	5 +	Hordeum vulgare	the MeJA responsive
TaMAPKKK129	TC-rich repeats	ATTCTCTAAC	1302	9 +	Nicotiana tabacum	defense and stress responsive
TaMAPKKK129	ABRE	ACGTG	545	5 +	Arabidopsis thaliana	abscisic acid responsive
TaMAPKKK129	ABRE	ACGTG	853	5 -	Arabidopsis thaliana	abscisic acid responsive
TaMAPKKK129	ABRE	ACGTG	1253	5 +	Arabidopsis thaliana	abscisic acid responsive
TaMAPKKK129	TCA-element	CCATCTTTTT	110	9 -	Nicotiana tabacum	salicylic acid responsive
TaMAPKKK130	MBS	CAACTG	644	6 -	Arabidopsis thaliana	drought inducibility
TaMAPKKK130	CGTCA-motif	CGTCA	468	5 -	Hordeum vulgare	the MeJA responsive
TaMAPKKK130	CGTCA-motif	CGTCA	1109	5 -	Hordeum vulgare	the MeJA responsive
TaMAPKKK130	CGTCA-motif	CGTCA	1715	5 +	Hordeum vulgare	the MeJA responsive
TaMAPKKK130	CGTCA-motif	CGTCA	1757	5 -	Hordeum vulgare	the MeJA responsive
TaMAPKKK130	O2-site	GATGATGTGG	479	9 -	Zea mays	zein metabolism regulation
TaMAPKKK130	ABRE	ACGTG	1056	5 +	Arabidopsis thaliana	abscisic acid responsive
TaMAPKKK130	ABRE	ACGTG	1118	5 +	Arabidopsis thaliana	abscisic acid responsive
TaMAPKKK130	ABRE	ACGTG	1480	5 +	Arabidopsis thaliana	abscisic acid responsive
TaMAPKKK130	ABRE	ACGTG	1560	5 +	Arabidopsis thaliana	abscisic acid responsive

TaMAPKKK130	ABRE	ACGTG	1916	5 -	Arabidopsis thaliana	abscisic acid responsive
TaMAPKKK130	ARE	AAACCA	1128	6 -	Zea mays	anaerobic induction
TaMAPKKK130	CAT-box	GCCACT	19	6 +	Arabidopsis thaliana	meristem expression
TaMAPKKK130	CAT-box	GCCACT	1183	6 +	Arabidopsis thaliana	meristem expression
TaMAPKKK130	GARE-motif	TCTGTTG	856	7 +	Brassica oleracea	gibberellin responsive
TaMAPKKK130	SARE	TTCGACCATCTT	518	11 -	Nicotiana tabacum	salicylic acid responsive
TaMAPKKK130	AuxRR-core	GGTCCAT	1797	7 -	Nicotiana tabacum	auxin responsive
TaMAPKKK130	TGACG-motif	TGACG	468	5 +	Hordeum vulgare	the MeJA responsive
TaMAPKKK130	TGACG-motif	TGACG	1109	5 +	Hordeum vulgare	the MeJA responsive
TaMAPKKK130	TGACG-motif	TGACG	1715	5 -	Hordeum vulgare	the MeJA responsive
TaMAPKKK130	TGACG-motif	TGACG	1757	5 +	Hordeum vulgare	the MeJA responsive
TaMAPKKK130	GC-motif	CCCCCG	1474	6 +	Zea mays	anoxic specific inducibility
TaMAPKKK130	TGA-element	AACGAC	1362	6 +	Brassica oleracea	auxin responsive
TaMAPKKK130	TGA-element	AACGAC	1973	6 -	Brassica oleracea	auxin responsive
TaMAPKKK131	P-box	CCTTTTG	1279	7 -	Oryza sativa	gibberellin responsive
TaMAPKKK131	MBS	CAACTG	1795	6 +	Arabidopsis thaliana	drought inducibility
TaMAPKKK131	ABRE	ACGTG	309	5 -	Arabidopsis thaliana	abscisic acid responsive
TaMAPKKK131	ABRE	ACGTG	1167	5 +	Arabidopsis thaliana	abscisic acid responsive
TaMAPKKK131	ABRE	GCCGCGTGGC	1505	9 -	Oryza sativa	abscisic acid responsive
TaMAPKKK131	ABRE	ACGTG	1553	5 -	Arabidopsis thaliana	abscisic acid responsive

TaMAPKKK131	ABRE	ACGTG	1625	5 -	Arabidopsis thaliana	abscisic acid responsive
TaMAPKKK131	GC-motif	CCCCCG	1818	6 -	Zea mays	anoxic specific inducibility
TaMAPKKK131	CGTCA-motif	CGTCA	1165	5 -	Hordeum vulgare	the MeJA responsive
TaMAPKKK131	CGTCA-motif	CGTCA	1555	5 +	Hordeum vulgare	the MeJA responsive
TaMAPKKK131	CGTCA-motif	CGTCA	1571	5 +	Hordeum vulgare	the MeJA responsive
TaMAPKKK131	TGACG-motif	TGACG	1165	5 +	Hordeum vulgare	the MeJA responsive
TaMAPKKK131	TGACG-motif	TGACG	1555	5 -	Hordeum vulgare	the MeJA responsive
TaMAPKKK131	TGACG-motif	TGACG	1571	5 -	Hordeum vulgare	the MeJA responsive
TaMAPKKK131	LTR	CCGAAA	346	6 +	Hordeum vulgare	low temperature responsive
TaMAPKKK131	LTR	CCGAAA	1815	6 -	Hordeum vulgare	low temperature responsive
TaMAPKKK131	O2-site	GTTGACGTGA	1552	9 -	Zea mays	zein metabolism regulation
TaMAPKKK131	MSA-like	TCAAACGGT	1322	9 -	Catharanthus roseus	cell cycle regulation
TaMAPKKK131	CAT-box	GCCACT	473	6 -	Arabidopsis thaliana	meristem expression
TaMAPKKK132	ABRE	ACGTG	274	5 -	Arabidopsis thaliana	abscisic acid responsive
TaMAPKKK132	ABRE	AACCCGG	605	7 +	Arabidopsis thaliana	abscisic acid responsive
TaMAPKKK132	ABRE	GCCGCGTGGC	721	9 -	Oryza sativa	abscisic acid responsive
TaMAPKKK132	ABRE	ACGTG	1787	5 +	Arabidopsis thaliana	abscisic acid responsive
TaMAPKKK132	CGTCA-motif	CGTCA	1765	5 -	Hordeum vulgare	the MeJA responsive
TaMAPKKK132	TGACG-motif	TGACG	1765	5 +	Hordeum vulgare	the MeJA responsive

TaMAPKKK132	ARE	AAACCA	1047	6 +	Zea mays	anaerobic induction
TaMAPKKK132	GC-motif	CCCCCG	357	6 -	Zea mays	anoxic specific inducibility
TaMAPKKK132	GC-motif	CCCCCG	950	6 +	Zea mays	anoxic specific inducibility
TaMAPKKK133	GC-motif	CCCCCG	1947	6 +	Zea mays	anoxic specific inducibility
TaMAPKKK133	ABRE	ACGTG	792	5 +	Arabidopsis thaliana	abscisic acid responsive
TaMAPKKK133	CGTCA-motif	CGTCA	165	5 +	Hordeum vulgare	the MeJA responsive
TaMAPKKK133	CGTCA-motif	CGTCA	390	5 +	Hordeum vulgare	the MeJA responsive
TaMAPKKK133	CGTCA-motif	CGTCA	1788	5 -	Hordeum vulgare	the MeJA responsive
TaMAPKKK133	CAT-box	GCCACT	1163	6 -	Arabidopsis thaliana	meristem expression
TaMAPKKK133	MBS	CAACTG	9	6 -	Arabidopsis thaliana	drought inducibility
TaMAPKKK133	MBS	CAACTG	1267	6 -	Arabidopsis thaliana	drought inducibility
TaMAPKKK133	TC-rich repeats	GTTTTCTTAC	1206	9 +	Nicotiana tabacum	defense and stress responsive
TaMAPKKK133	TCA-element	CCATCTTTTT	283	9 +	Nicotiana tabacum	salicylic acid responsive
TaMAPKKK133	TGACG-motif	TGACG	165	5 -	Hordeum vulgare	the MeJA responsive
TaMAPKKK133	TGACG-motif	TGACG	390	5 -	Hordeum vulgare	the MeJA responsive
TaMAPKKK133	TGACG-motif	TGACG	1788	5 +	Hordeum vulgare	the MeJA responsive
TaMAPKKK133	LTR	CCGAAA	591	6 -	Hordeum vulgare	low temperature responsive
TaMAPKKK134	MSA-like	(T/C)C(T/C)AACGG(T/C)(T/C)A	1916	8.5 -	Catharanthus roseus	cell cycle regulation

TaMAPKKK134	TGACG-motif	TGACG	1670	5 -	Hordeum vulgare	the MeJA responsive
TaMAPKKK134	MBS	CAACTG	1892	6 +	Arabidopsis thaliana	drought inducibility
TaMAPKKK134	CAT-box	GCCACT	587	6 -	Arabidopsis thaliana	meristem expression
TaMAPKKK134	CGTCA-motif	CGTCA	1670	5 +	Hordeum vulgare	the MeJA responsive
TaMAPKKK134	GC-motif	CCCCCG	1107	6 -	Zea mays	anoxic specific inducibility
TaMAPKKK134	ABRE	CACGTG	448	6 +	Arabidopsis thaliana	abscisic acid responsive
TaMAPKKK134	ABRE	ACGTG	449	5 +	Arabidopsis thaliana	abscisic acid responsive
TaMAPKKK134	ABRE	ACGTG	608	5 -	Arabidopsis thaliana	abscisic acid responsive
TaMAPKKK134	ABRE	ACGTG	1229	5 -	Arabidopsis thaliana	abscisic acid responsive
TaMAPKKK134	ABRE	CACGTG	1292	6 -	Arabidopsis thaliana	abscisic acid responsive
TaMAPKKK134	ABRE	ACGTG	1293	5 +	Arabidopsis thaliana	abscisic acid responsive
TaMAPKKK134	ABRE	GACACGTGGC	1445	9 -	Triticum aestivum	abscisic acid responsive
TaMAPKKK134	ABRE	ACGTG	1907	5 -	Arabidopsis thaliana	abscisic acid responsive
TaMAPKKK134	ABRE	GCAACGTGTC	1952	10 +	Hordeum vulgare	abscisic acid responsive
TaMAPKKK134	ABRE	ACGTG	1955	5 +	Arabidopsis thaliana	abscisic acid responsive
TaMAPKKK134	P-box	CCTTTTG	416	7 -	Oryza sativa	gibberellin responsive
TaMAPKKK134	P-box	CCTTTTG	1011	7 -	Oryza sativa	gibberellin responsive
TaMAPKKK134	TCA-element	CCATCTTTTT	1753	9 +	Nicotiana tabacum	salicylic acid responsive
TaMAPKKK135	GCN4_motif	TGAGTCA	1839	7 -	Oryza sativa	endosperm expression

TaMAPKKK135	TCA-element	CCATCTTTTT	134	9 +	Nicotiana tabacum	salicylic acid responsive
TaMAPKKK135	TCA-element	CCATCTTTTT	769	9 -	Nicotiana tabacum	salicylic acid responsive
TaMAPKKK135	TCA-element	CCATCTTTTT	1218	10 -	Nicotiana tabacum	salicylic acid responsive
TaMAPKKK135	AuxRR-core	GGTCCAT	698	7 +	Nicotiana tabacum	auxin responsive
TaMAPKKK135	CGTCA-motif	CGTCA	70	5 +	Hordeum vulgare	the MeJA responsive
TaMAPKKK135	CGTCA-motif	CGTCA	103	5 +	Hordeum vulgare	the MeJA responsive
TaMAPKKK135	ABRE	AACCCGG	1654	7 -	Arabidopsis thaliana	abscisic acid responsive
TaMAPKKK135	CAT-box	GCCACT	1967	6 -	Arabidopsis thaliana	meristem expression
TaMAPKKK135	ARE	AAACCA	10	6 +	Zea mays	anaerobic induction
TaMAPKKK135	ARE	AAACCA	26	6 +	Zea mays	anaerobic induction
TaMAPKKK135	TGACG-motif	TGACG	70	5 -	Hordeum vulgare	the MeJA responsive
TaMAPKKK135	TGACG-motif	TGACG	103	5 -	Hordeum vulgare	the MeJA responsive
TaMAPKKK136	TGA-element	AACGAC	703	6 +	Brassica oleracea	auxin responsive
TaMAPKKK136	TGA-element	AACGAC	1821	6 +	Brassica oleracea	auxin responsive
TaMAPKKK136	CAT-box	GCCACT	801	6 +	Arabidopsis thaliana	meristem expression
TaMAPKKK136	CAT-box	GCCACT	1848	6 +	Arabidopsis thaliana	meristem expression
TaMAPKKK136	CGTCA-motif	CGTCA	795	5 -	Hordeum vulgare	the MeJA responsive
TaMAPKKK136	CGTCA-motif	CGTCA	1540	5 +	Hordeum vulgare	the MeJA responsive
TaMAPKKK136	CGTCA-motif	CGTCA	1800	5 -	Hordeum vulgare	the MeJA responsive
TaMAPKKK136	ABRE	GCCGCGTGGC	121	9 -	Oryza sativa	abscisic acid responsive

TaMAPKKK136	ABRE	ACGTG	289	5 +	Arabidopsis thaliana	abscisic acid responsive
TaMAPKKK136	ABRE	CGTACGTGCA	1424	9 -	Hordeum vulgare	abscisic acid responsive
TaMAPKKK136	ABRE	ACGTG	1426	5 -	Arabidopsis thaliana	abscisic acid responsive
TaMAPKKK136	ABRE	ACGTG	1682	5 +	Arabidopsis thaliana	abscisic acid responsive
TaMAPKKK136	GC-motif	CCCCCG	206	6 -	Zea mays	anoxic specific inducibility
TaMAPKKK136	GC-motif	CCCCCG	374	6 +	Zea mays	anoxic specific inducibility
TaMAPKKK136	GC-motif	CCCCCG	881	6 +	Zea mays	anoxic specific inducibility
TaMAPKKK136	TGACG-motif	TGACG	795	5 +	Hordeum vulgare	the MeJA responsive
TaMAPKKK136	TGACG-motif	TGACG	1540	5 -	Hordeum vulgare	the MeJA responsive
TaMAPKKK136	TGACG-motif	TGACG	1800	5 +	Hordeum vulgare	the MeJA responsive
TaMAPKKK136	TCA-element	CCATCTTTTT	846	9 +	Nicotiana tabacum	salicylic acid responsive
TaMAPKKK136	ARE	AAACCA	1446	6 +	Zea mays	anaerobic induction
TaMAPKKK136	ARE	AAACCA	1631	6 -	Zea mays	anaerobic induction
TaMAPKKK136	O2-site	GATGA(C/T)(A/G)TG(A/G)	1005	8 -	Zea mays	zein metabolism regulation
TaMAPKKK136	O2-site	GATGACATGG	1246	9 +	Zea mays	zein metabolism regulation
TaMAPKKK136	LTR	CCGAAA	1768	6 +	Hordeum vulgare	low temperature responsive
TaMAPKKK137	CGTCA-motif	CGTCA	1385	5 -	Hordeum vulgare	the MeJA responsive
TaMAPKKK137	MBS	CAACTG	240	6 +	Arabidopsis thaliana	drought inducibility
TaMAPKKK137	MBS	CAACTG	410	6 -	Arabidopsis thaliana	drought inducibility

TaMAPKKK137	TCA-element	CCATCTTTTT	1959	9 +	Nicotiana tabacum	salicylic acid responsive
TaMAPKKK137	TGACG-motif	TGACG	1385	5 +	Hordeum vulgare	the MeJA responsive
TaMAPKKK137	CAT-box	GCCACT	942	6 -	Arabidopsis thaliana	meristem expression
TaMAPKKK137	CAT-box	GCCACT	1284	6 -	Arabidopsis thaliana	meristem expression
TaMAPKKK137	ABRE	ACGTG	1726	5 -	Arabidopsis thaliana	abscisic acid responsive
TaMAPKKK137	ABRE	ACGTG	1884	5 -	Arabidopsis thaliana	abscisic acid responsive
TaMAPKKK139	CGTCA-motif	CGTCA	157	5 +	Hordeum vulgare	the MeJA responsive
TaMAPKKK139	CGTCA-motif	CGTCA	477	5 -	Hordeum vulgare	the MeJA responsive
TaMAPKKK139	CGTCA-motif	CGTCA	519	5 -	Hordeum vulgare	the MeJA responsive
TaMAPKKK139	CGTCA-motif	CGTCA	1685	5 -	Hordeum vulgare	the MeJA responsive
TaMAPKKK139	TC-rich repeats	GTTTTCTTAC	1437	9 -	Nicotiana tabacum	defense and stress responsive
TaMAPKKK139	TCA-element	CCATCTTTTT	464	9 +	Nicotiana tabacum	salicylic acid responsive
TaMAPKKK139	TCA-element	TCAGAAGAGG	1914	9 -	Brassica oleracea	salicylic acid responsive
TaMAPKKK139	TGACG-motif	TGACG	157	5 -	Hordeum vulgare	the MeJA responsive
TaMAPKKK139	TGACG-motif	TGACG	477	5 +	Hordeum vulgare	the MeJA responsive
TaMAPKKK139	TGACG-motif	TGACG	519	5 +	Hordeum vulgare	the MeJA responsive
TaMAPKKK139	TGACG-motif	TGACG	1685	5 +	Hordeum vulgare	the MeJA responsive
TaMAPKKK139	ARE	AAACCA	932	6 +	Zea mays	anaerobic induction
TaMAPKKK139	ABRE	TACGGTC	166	7 -	Arabidopsis thaliana	abscisic acid responsive

TaMAPKKK139	ABRE	ACGTG	172	5 +	Arabidopsis thaliana	abscisic acid responsive
TaMAPKKK139	ABRE	ACGTG	231	5 -	Arabidopsis thaliana	abscisic acid responsive
TaMAPKKK139	WUN-motif	AAATTTCCT	356	9 -	Brassica oleracea	wound responsive
TaMAPKKK139	GARE-motif	TCTGTTG	386	7 -	Brassica oleracea	gibberellin responsive
TaMAPKKK139	CAT-box	GCCACT	710	6 -	Arabidopsis thaliana	meristem expression
TaMAPKKK14	ABRE	ACGTG	108	5 +	Arabidopsis thaliana	abscisic acid responsive
TaMAPKKK14	ABRE	ACGTG	538	5 -	Arabidopsis thaliana	abscisic acid responsive
TaMAPKKK14	ABRE	ACGTG	1416	5 +	Arabidopsis thaliana	abscisic acid responsive
TaMAPKKK14	LTR	CCGAAA	806	6 -	Hordeum vulgare	low temperature responsive
TaMAPKKK14	CGTCA-motif	CGTCA	1982	5 -	Hordeum vulgare	the MeJA responsive
TaMAPKKK14	TGA-element	AACGAC	323	6 +	Brassica oleracea	auxin responsive
TaMAPKKK14	TGA-element	AACGAC	1968	6 +	Brassica oleracea	auxin responsive
TaMAPKKK14	ARE	AAACCA	1753	6 +	Zea mays	anaerobic induction
TaMAPKKK14	ARE	AAACCA	1801	6 +	Zea mays	anaerobic induction
TaMAPKKK14	O2-site	GATGATGTGG	454	9 +	Zea mays	zein metabolism regulation
TaMAPKKK14	O2-site	GATGACATGG	495	9 -	Zea mays	zein metabolism regulation
TaMAPKKK14	TGACG-motif	TGACG	1982	5 +	Hordeum vulgare	the MeJA responsive
TaMAPKKK14	P-box	CCTTTTG	970	7 +	Oryza sativa	gibberellin responsive
TaMAPKKK14	CAT-box	GCCACT	1583	6 -	Arabidopsis thaliana	meristem expression
TaMAPKKK14	CAT-box	GCCACT	1623	6 -	Arabidopsis thaliana	meristem expression

TaMAPKKK140	RY-element	CATGCATG	383	8 +	Helianthus annuus	seed specific regulation
TaMAPKKK140	ABRE	GCAACGTGTC	192	9 +	Hordeum vulgare	abscisic acid responsive
TaMAPKKK140	ABRE	ACGTG	569	5 -	Arabidopsis thaliana	abscisic acid responsive
TaMAPKKK140	MBS	CAACTG	13	6 +	Arabidopsis thaliana	drought inducibility
TaMAPKKK140	ARE	AAACCA	927	6 -	Zea mays	anaerobic induction
TaMAPKKK140	ARE	AAACCA	1596	6 -	Zea mays	anaerobic induction
TaMAPKKK140	GCN4_motif	TGAGTCA	594	7 +	Oryza sativa	endosperm expression
TaMAPKKK140	LTR	CCGAAA	540	6 -	Hordeum vulgare	low temperature responsive
TaMAPKKK140	TGACG-motif	TGACG	1228	5 +	Hordeum vulgare	the MeJA responsive
TaMAPKKK140	CGTCA-motif	CGTCA	1228	5 -	Hordeum vulgare	the MeJA responsive
TaMAPKKK140	AuxRR-core	GGTCCAT	1182	7 -	Nicotiana tabacum	auxin responsive
TaMAPKKK141	TC-rich repeats	GTTTTCTTAC	718	9 +	Nicotiana tabacum	defense and stress responsive
TaMAPKKK141	TC-rich repeats	ATTCTCTAAC	1735	9 +	Nicotiana tabacum	defense and stress responsive
TaMAPKKK141	O2-site	GATGATGTGG	1370	9 +	Zea mays	zein metabolism regulation
TaMAPKKK141	TCA-element	CCATCTTTTT	1590	9 +	Nicotiana tabacum	salicylic acid responsive
TaMAPKKK141	ABRE	ACGTG	203	5 -	Arabidopsis thaliana	abscisic acid responsive
TaMAPKKK141	TGA-element	AACGAC	441	6 +	Brassica oleracea	auxin responsive
TaMAPKKK141	TGA-element	AACGAC	820	6 -	Brassica oleracea	auxin responsive
TaMAPKKK141	ARE	AAACCA	988	6 -	Zea mays	anaerobic induction
TaMAPKKK141	ARE	AAACCA	1130	6 -	Zea mays	anaerobic induction

TaMAPKKK141	ARE	AAACCA	1877	6 -	Zea mays	anaerobic induction
TaMAPKKK141	P-box	CCTTTTG	839	7 -	Oryza sativa	gibberellin responsive
TaMAPKKK141	P-box	CCTTTTG	1340	7 +	Oryza sativa	gibberellin responsive
TaMAPKKK141	TGACG-motif	TGACG	290	5 -	Hordeum vulgare	the MeJA responsive
TaMAPKKK141	TGACG-motif	TGACG	355	5 -	Hordeum vulgare	the MeJA responsive
TaMAPKKK141	TGACG-motif	TGACG	460	5 -	Hordeum vulgare	the MeJA responsive
TaMAPKKK141	TGACG-motif	TGACG	502	5 +	Hordeum vulgare	the MeJA responsive
TaMAPKKK141	TGACG-motif	TGACG	898	5 +	Hordeum vulgare	the MeJA responsive
TaMAPKKK141	TGACG-motif	TGACG	958	5 +	Hordeum vulgare	the MeJA responsive
TaMAPKKK141	TGACG-motif	TGACG	1410	5 -	Hordeum vulgare	the MeJA responsive
TaMAPKKK141	CGTCA-motif	CGTCA	290	5 +	Hordeum vulgare	the MeJA responsive
TaMAPKKK141	CGTCA-motif	CGTCA	355	5 +	Hordeum vulgare	the MeJA responsive
TaMAPKKK141	CGTCA-motif	CGTCA	460	5 +	Hordeum vulgare	the MeJA responsive
TaMAPKKK141	CGTCA-motif	CGTCA	502	5 -	Hordeum vulgare	the MeJA responsive
TaMAPKKK141	CGTCA-motif	CGTCA	898	5 -	Hordeum vulgare	the MeJA responsive
TaMAPKKK141	CGTCA-motif	CGTCA	958	5 -	Hordeum vulgare	the MeJA responsive
TaMAPKKK141	CGTCA-motif	CGTCA	1410	5 +	Hordeum vulgare	the MeJA responsive
TaMAPKKK142	TCA-element	TCAGAAGAGG	1933	9 -	Brassica oleracea	salicylic acid responsive
TaMAPKKK142	ARE	AAACCA	470	6 -	Zea mays	anaerobic induction

TaMAPKKK142	ARE	AAACCA	560	6 -	Zea mays	anaerobic induction
TaMAPKKK142	ARE	AAACCA	733	6 -	Zea mays	anaerobic induction
TaMAPKKK142	ARE	AAACCA	1347	6 -	Zea mays	anaerobic induction
TaMAPKKK142	MBS	CAACTG	1523	6 -	Arabidopsis thaliana	drought inducibility
TaMAPKKK142	CGTCA-motif	CGTCA	169	5 -	Hordeum vulgare	the MeJA responsive
TaMAPKKK142	CGTCA-motif	CGTCA	212	5 +	Hordeum vulgare	the MeJA responsive
TaMAPKKK142	CGTCA-motif	CGTCA	1959	5 +	Hordeum vulgare	the MeJA responsive
TaMAPKKK142	CAT-box	GCCACT	373	6 -	Arabidopsis thaliana	meristem expression
TaMAPKKK142	CAT-box	GCCACT	699	6 -	Arabidopsis thaliana	meristem expression
TaMAPKKK142	TGA-element	AACGAC	1715	6 +	Brassica oleracea	auxin responsive
TaMAPKKK142	P-box	CCTTTTG	1799	7 -	Oryza sativa	gibberellin responsive
TaMAPKKK142	circadian	CAAAGATATC	1234	9 -	Lycopersicon esculentum	circadian control
TaMAPKKK142	LTR	CCGAAA	77	6 -	Hordeum vulgare	low temperature responsive
TaMAPKKK142	AuxRR-core	GGTCCAT	411	7 +	Nicotiana tabacum	auxin responsive
TaMAPKKK142	TGACG-motif	TGACG	169	5 +	Hordeum vulgare	the MeJA responsive
TaMAPKKK142	TGACG-motif	TGACG	212	5 -	Hordeum vulgare	the MeJA responsive
TaMAPKKK142	TGACG-motif	TGACG	1959	5 -	Hordeum vulgare	the MeJA responsive
TaMAPKKK142	motif I	gGTACGTGGCG	295	10 +	Oryza sativa	root specific
TaMAPKKK142	GC-motif	CCCCCG	614	6 -	Zea mays	anoxic specific inducibility

TaMAPKKK142	GC-motif	CCCCCG	1138	6 +	Zea mays	anoxic specific inducibility
TaMAPKKK142	ABRE	ACGTG	298	5 +	Arabidopsis thaliana	abscisic acid responsive
TaMAPKKK142	ABRE	ACGTG	453	5 +	Arabidopsis thaliana	abscisic acid responsive
TaMAPKKK142	ABRE	ACGTG	718	5 +	Arabidopsis thaliana	abscisic acid responsive
TaMAPKKK142	ABRE	ACGTG	1211	5 -	Arabidopsis thaliana	abscisic acid responsive
TaMAPKKK144	TGA-element	AACGAC	1769	6 -	Brassica oleracea	auxin responsive
TaMAPKKK144	CAT-box	GCCACT	878	6 -	Arabidopsis thaliana	meristem expression
TaMAPKKK144	ABRE	ACGTG	372	5 -	Arabidopsis thaliana	abscisic acid responsive
TaMAPKKK144	ABRE	AACCCGG	1063	7 +	Arabidopsis thaliana	abscisic acid responsive
TaMAPKKK144	ARE	AAACCA	193	6 +	Zea mays	anaerobic induction
TaMAPKKK144	ARE	AAACCA	1106	6 -	Zea mays	anaerobic induction
TaMAPKKK144	TGACG-motif	TGACG	482	5 +	Hordeum vulgare	the MeJA responsive
TaMAPKKK144	TGACG-motif	TGACG	575	5 -	Hordeum vulgare	the MeJA responsive
TaMAPKKK144	TGACG-motif	TGACG	695	5 +	Hordeum vulgare	the MeJA responsive
TaMAPKKK144	TGACG-motif	TGACG	1681	5 +	Hordeum vulgare	the MeJA responsive
TaMAPKKK144	O2-site	GATGACATGG	470	9 -	Zea mays	zein metabolism regulation
TaMAPKKK144	TATC-box	TATCCCA	1190	7 +	Oryza sativa	gibberellin responsive
TaMAPKKK144	CGTCA-motif	CGTCA	482	5 -	Hordeum vulgare	the MeJA responsive
TaMAPKKK144	CGTCA-motif	CGTCA	575	5 +	Hordeum vulgare	the MeJA responsive

TaMAPKKK144	CGTCA-motif	CGTCA	695	5 -	Hordeum vulgare	the MeJA responsive
TaMAPKKK144	CGTCA-motif	CGTCA	1681	5 -	Hordeum vulgare	the MeJA responsive
TaMAPKKK145	MBS	CAACTG	1673	6 +	Arabidopsis thaliana	drought inducibility
TaMAPKKK145	P-box	CCTTTTG	770	7 +	Oryza sativa	gibberellin responsive
TaMAPKKK145	GC-motif	CCCCCG	615	6 -	Zea mays	anoxic specific inducibility
TaMAPKKK145	ABRE	ACGTG	493	5 -	Arabidopsis thaliana	abscisic acid responsive
TaMAPKKK145	ABRE	ACGTG	1045	5 +	Arabidopsis thaliana	abscisic acid responsive
TaMAPKKK145	TGA-element	AACGAC	551	6 +	Brassica oleracea	auxin responsive
TaMAPKKK145	CGTCA-motif	CGTCA	505	5 +	Hordeum vulgare	the MeJA responsive
TaMAPKKK145	CGTCA-motif	CGTCA	1858	5 +	Hordeum vulgare	the MeJA responsive
TaMAPKKK145	O2-site	GATGATGTGG	1933	9 -	Zea mays	zein metabolism regulation
TaMAPKKK145	GCN4_motif	TGAGTCA	1157	7 -	Oryza sativa	endosperm expression
TaMAPKKK145	GCN4_motif	TGAGTCA	1190	7 -	Oryza sativa	endosperm expression
TaMAPKKK145	GCN4_motif	TGAGTCA	1234	7 +	Oryza sativa	endosperm expression
TaMAPKKK145	LTR	CCGAAA	284	6 -	Hordeum vulgare	low temperature responsive
TaMAPKKK145	LTR	CCGAAA	649	6 -	Hordeum vulgare	low temperature responsive
TaMAPKKK145	TCA-element	CCATCTTTTT	1056	9 +	Nicotiana tabacum	salicylic acid responsive
TaMAPKKK145	TGACG-motif	TGACG	505	5 -	Hordeum vulgare	the MeJA responsive
TaMAPKKK145	TGACG-motif	TGACG	1858	5 -	Hordeum vulgare	the MeJA responsive

TaMAPKKK146	TCA-element	CCATCTTTTT	76	9 -	Nicotiana tabacum	salicylic acid responsive
TaMAPKKK146	TCA-element	CCATCTTTTT	990	9 +	Nicotiana tabacum	salicylic acid responsive
TaMAPKKK146	TGACG-motif	TGACG	1198	5 -	Hordeum vulgare	the MeJA responsive
TaMAPKKK146	TGACG-motif	TGACG	1889	5 +	Hordeum vulgare	the MeJA responsive
TaMAPKKK146	TGACG-motif	TGACG	1928	5 -	Hordeum vulgare	the MeJA responsive
TaMAPKKK146	ARE	AAACCA	192	6 +	Zea mays	anaerobic induction
TaMAPKKK146	ARE	AAACCA	374	6 +	Zea mays	anaerobic induction
TaMAPKKK146	ARE	AAACCA	469	6 +	Zea mays	anaerobic induction
TaMAPKKK146	ARE	AAACCA	900	6 -	Zea mays	anaerobic induction
TaMAPKKK146	HD-Zip 1	CAAT(A/T)ATTG	893	8 +	Arabidopsis thaliana	differentiation of the palisade mesophyll cells
TaMAPKKK146	LTR	CCGAAA	1683	6 +	Hordeum vulgare	low temperature responsive
TaMAPKKK146	O2-site	GATGACATGG	195	9 -	Zea mays	zein metabolism regulation
TaMAPKKK146	O2-site	GATGA(C/T)(A/G)TG(A/G)	1755	8 -	Zea mays	zein metabolism regulation
TaMAPKKK146	CGTCA-motif	CGTCA	1198	5 +	Hordeum vulgare	the MeJA responsive
TaMAPKKK146	CGTCA-motif	CGTCA	1889	5 -	Hordeum vulgare	the MeJA responsive
TaMAPKKK146	CGTCA-motif	CGTCA	1928	5 +	Hordeum vulgare	the MeJA responsive
TaMAPKKK146	CAT-box	GCCACT	1731	6 -	Arabidopsis thaliana	meristem expression
TaMAPKKK146	TGA-element	AACGAC	1352	6 -	Brassica oleracea	auxin responsive
TaMAPKKK146	GC-motif	CCCCCG	1984	6 +	Zea mays	anoxic specific inducibility

TaMAPKKK146	ABRE	GCCGCGTGGC	1735	9	-	Oryza sativa	abscisic acid responsive
TaMAPKKK146	P-box	CCTTTTG	10	7	-	Oryza sativa	gibberellin responsive
TaMAPKKK146	TC-rich repeats	GTTTTCTTAC	1403	9	+	Nicotiana tabacum	defense and stress responsive
TaMAPKKK146	MBS	CAACTG	716	6	+	Arabidopsis thaliana	drought inducibility
TaMAPKKK147	ABRE	ACGTG	483	5	+	Arabidopsis thaliana	abscisic acid responsive
TaMAPKKK147	ABRE	ACGTG	1135	5	-	Arabidopsis thaliana	abscisic acid responsive
TaMAPKKK147	ABRE	AACCCGG	1426	7	-	Arabidopsis thaliana	abscisic acid responsive
TaMAPKKK147	ABRE	GCAACGTGTC	1890	9	-	Hordeum vulgare	abscisic acid responsive
TaMAPKKK147	ABRE	ACGTG	1892	5	-	Arabidopsis thaliana	abscisic acid responsive
TaMAPKKK147	GC-motif	CCCCCG	134	6	-	Zea mays	anoxic specific inducibility
TaMAPKKK147	GC-motif	CCCCCG	291	6	+	Zea mays	anoxic specific inducibility
TaMAPKKK147	CAT-box	GCCACT	536	6	+	Arabidopsis thaliana	meristem expression
TaMAPKKK147	CAT-box	GCCACT	952	6	-	Arabidopsis thaliana	meristem expression
TaMAPKKK147	CGTCA-motif	CGTCA	407	5	-	Hordeum vulgare	the MeJA responsive
TaMAPKKK147	CGTCA-motif	CGTCA	456	5	-	Hordeum vulgare	the MeJA responsive
TaMAPKKK147	CGTCA-motif	CGTCA	481	5	-	Hordeum vulgare	the MeJA responsive
TaMAPKKK147	CGTCA-motif	CGTCA	1313	5	+	Hordeum vulgare	the MeJA responsive
TaMAPKKK147	CGTCA-motif	CGTCA	1855	5	+	Hordeum vulgare	the MeJA responsive
TaMAPKKK147	CGTCA-motif	CGTCA	1947	5	+	Hordeum vulgare	the MeJA responsive

TaMAPKKK147	ARE	AAACCA	1280	6 +	Zea mays	anaerobic induction
TaMAPKKK147	ARE	AAACCA	1905	6 +	Zea mays	anaerobic induction
TaMAPKKK147	TGACG-motif	TGACG	407	5 +	Hordeum vulgare	the MeJA responsive
TaMAPKKK147	TGACG-motif	TGACG	456	5 +	Hordeum vulgare	the MeJA responsive
TaMAPKKK147	TGACG-motif	TGACG	481	5 +	Hordeum vulgare	the MeJA responsive
TaMAPKKK147	TGACG-motif	TGACG	1313	5 -	Hordeum vulgare	the MeJA responsive
TaMAPKKK147	TGACG-motif	TGACG	1855	5 -	Hordeum vulgare	the MeJA responsive
TaMAPKKK147	TGACG-motif	TGACG	1947	5 -	Hordeum vulgare	the MeJA responsive
TaMAPKKK147	O2-site	GATGACATGG	878	9 -	Zea mays	zein metabolism regulation
TaMAPKKK147	GCN4_motif	TGAGTCA	1090	7 +	Oryza sativa	endosperm expression
TaMAPKKK147	AuxRR-core	GGTCCAT	32	7 -	Nicotiana tabacum	auxin responsive
TaMAPKKK147	LTR	CCGAAA	1712	6 -	Hordeum vulgare	low temperature responsive
TaMAPKKK148	CGTCA-motif	CGTCA	621	5 -	Hordeum vulgare	the MeJA responsive
TaMAPKKK148	CGTCA-motif	CGTCA	653	5 -	Hordeum vulgare	the MeJA responsive
TaMAPKKK148	CGTCA-motif	CGTCA	663	5 -	Hordeum vulgare	the MeJA responsive
TaMAPKKK148	CGTCA-motif	CGTCA	772	5 -	Hordeum vulgare	the MeJA responsive
TaMAPKKK148	TGA-element	AACGAC	935	6 +	Brassica oleracea	auxin responsive
TaMAPKKK148	GC-motif	CCCCCG	1236	6 +	Zea mays	anoxic specific inducibility
TaMAPKKK148	ABRE	CACGTG	473	6 +	Arabidopsis thaliana	abscisic acid responsive

TaMAPKKK148	ABRE	ACGTG	474	5 +	Arabidopsis thaliana	abscisic acid responsive
TaMAPKKK148	ABRE	CGCACGTGTC	877	9 +	Hordeum vulgare	abscisic acid responsive
TaMAPKKK148	ABRE	CACGTG	879	6 +	Arabidopsis thaliana	abscisic acid responsive
TaMAPKKK148	ABRE	ACGTG	880	5 +	Arabidopsis thaliana	abscisic acid responsive
TaMAPKKK148	ABRE	AACCCGG	1044	7 +	Arabidopsis thaliana	abscisic acid responsive
TaMAPKKK148	ABRE	ACGTG	1123	5 -	Arabidopsis thaliana	abscisic acid responsive
TaMAPKKK148	ABRE	CACGTG	1486	6 -	Arabidopsis thaliana	abscisic acid responsive
TaMAPKKK148	ABRE	ACGTG	1487	5 +	Arabidopsis thaliana	abscisic acid responsive
TaMAPKKK148	TGA-box	TGACGTAA	653	8 +	Glycine max	auxin responsive
TaMAPKKK148	MBS	CAACTG	149	6 +	Arabidopsis thaliana	drought inducibility
TaMAPKKK148	MBS	CAACTG	1725	6 -	Arabidopsis thaliana	drought inducibility
TaMAPKKK148	TCA-element	TCAGAAGAGG	230	9 -	Brassica oleracea	salicylic acid responsive
TaMAPKKK148	TGACG-motif	TGACG	621	5 +	Hordeum vulgare	the MeJA responsive
TaMAPKKK148	TGACG-motif	TGACG	653	5 +	Hordeum vulgare	the MeJA responsive
TaMAPKKK148	TGACG-motif	TGACG	663	5 +	Hordeum vulgare	the MeJA responsive
TaMAPKKK148	TGACG-motif	TGACG	772	5 +	Hordeum vulgare	the MeJA responsive
TaMAPKKK148	ARE	AAACCA	1864	6 -	Zea mays	anaerobic induction
TaMAPKKK148	circadian	CAAAGATATC	1557	9 +	Lycopersicon esculentum	circadian control
TaMAPKKK148	O2-site	GTTGACGTGA	1483	9 +	Zea mays	zein metabolism regulation

TaMAPKKK15	CGTCA-motif	CGTCA	303	5 +	Hordeum vulgare	the MeJA responsive
TaMAPKKK15	CGTCA-motif	CGTCA	690	5 -	Hordeum vulgare	the MeJA responsive
TaMAPKKK15	ABRE	ACGTG	85	5 -	Arabidopsis thaliana	abscisic acid responsive
TaMAPKKK15	circadian	CAAAGATATC	1706	9 +	Lycopersicon esculentum	circadian control
TaMAPKKK15	GC-motif	CCCCCG	1962	6 +	Zea mays	anoxic specific inducibility
TaMAPKKK15	ARE	AAACCA	1027	6 +	Zea mays	anaerobic induction
TaMAPKKK15	TGACG-motif	TGACG	303	5 -	Hordeum vulgare	the MeJA responsive
TaMAPKKK15	TGACG-motif	TGACG	690	5 +	Hordeum vulgare	the MeJA responsive
TaMAPKKK15	MBS	CAACTG	1182	6 +	Arabidopsis thaliana	drought inducibility
TaMAPKKK150	CGTCA-motif	CGTCA	538	5 +	Hordeum vulgare	the MeJA responsive
TaMAPKKK150	CGTCA-motif	CGTCA	616	5 -	Hordeum vulgare	the MeJA responsive
TaMAPKKK150	AuxRR-core	GGTCCAT	575	7 -	Nicotiana tabacum	auxin responsive
TaMAPKKK150	MBS	CAACTG	231	6 +	Arabidopsis thaliana	drought inducibility
TaMAPKKK150	TC-rich repeats	ATTCTCTAAC	1325	9 -	Nicotiana tabacum	defense and stress responsive
TaMAPKKK150	TGACG-motif	TGACG	538	5 -	Hordeum vulgare	the MeJA responsive
TaMAPKKK150	TGACG-motif	TGACG	616	5 +	Hordeum vulgare	the MeJA responsive
TaMAPKKK150	ARE	AAACCA	1103	6 -	Zea mays	anaerobic induction
TaMAPKKK150	ARE	AAACCA	1490	6 -	Zea mays	anaerobic induction
TaMAPKKK150	ABRE	ACGTG	509	5 -	Arabidopsis thaliana	abscisic acid responsive

TaMAPKKK150	ABRE	GCCGCGTGGC	1236	9 +	Oryza sativa	abscisic acid responsive
TaMAPKKK150	TGA-element	AACGAC	560	6 +	Brassica oleracea	auxin responsive
TaMAPKKK151	LTR	CCGAAA	1603	6 -	Hordeum vulgare	low temperature responsive
TaMAPKKK151	CGTCA-motif	CGTCA	785	5 +	Hordeum vulgare	the MeJA responsive
TaMAPKKK151	O2-site	GATGATGTGG	637	9 +	Zea mays	zein metabolism regulation
TaMAPKKK151	O2-site	GATGATGTGG	1064	9 +	Zea mays	zein metabolism regulation
TaMAPKKK151	O2-site	GATGA(C/T)(A/G)TG(A/G)	1219	8 -	Zea mays	zein metabolism regulation
TaMAPKKK151	GC-motif	CCCCCG	1610	6 -	Zea mays	anoxic specific inducibility
TaMAPKKK151	P-box	CCTTTTG	1139	7 +	Oryza sativa	gibberellin responsive
TaMAPKKK151	MBS	CAACTG	331	6 +	Arabidopsis thaliana	drought inducibility
TaMAPKKK151	MBS	CAACTG	1944	6 +	Arabidopsis thaliana	drought inducibility
TaMAPKKK151	ARE	AAACCA	838	6 -	Zea mays	anaerobic induction
TaMAPKKK151	ARE	AAACCA	1199	6 +	Zea mays	anaerobic induction
TaMAPKKK151	ARE	AAACCA	1647	6 -	Zea mays	anaerobic induction
TaMAPKKK151	TGACG-motif	TGACG	785	5 -	Hordeum vulgare	the MeJA responsive
TaMAPKKK152	TGA-element	AACGAC	823	6 +	Brassica oleracea	auxin responsive
TaMAPKKK152	TGA-element	AACGAC	862	6 +	Brassica oleracea	auxin responsive
TaMAPKKK152	TGA-element	AACGAC	1425	6 +	Brassica oleracea	auxin responsive
TaMAPKKK152	TGA-element	AACGAC	1772	6 +	Brassica oleracea	auxin responsive
TaMAPKKK152	ABRE	ACGTG	474	5 -	Arabidopsis thaliana	abscisic acid responsive
TaMAPKKK152	ABRE	ACGTG	562	5 +	Arabidopsis thaliana	abscisic acid responsive

TaMAPKKK152	ABRE	ACGTG	1272	5 -	Arabidopsis thaliana	abscisic acid responsive
TaMAPKKK152	CAT-box	GCCACT	171	6 -	Arabidopsis thaliana	meristem expression
TaMAPKKK152	CAT-box	GCCACT	739	6 +	Arabidopsis thaliana	meristem expression
TaMAPKKK152	CAT-box	GCCACT	1254	6 -	Arabidopsis thaliana	meristem expression
TaMAPKKK152	ARE	AAACCA	635	6 -	Zea mays	anaerobic induction
TaMAPKKK152	ARE	AAACCA	1587	6 +	Zea mays	anaerobic induction
TaMAPKKK152	TCA-element	CCATCTTTTT	142	9 +	Nicotiana tabacum	salicylic acid responsive
TaMAPKKK152	TCA-element	TCAGAAGAGG	383	9 +	Brassica oleracea	salicylic acid responsive
TaMAPKKK152	TCA-element	CCATCTTTTT	1308	9 +	Nicotiana tabacum	salicylic acid responsive
TaMAPKKK152	CGTCA-motif	CGTCA	420	5 +	Hordeum vulgare	the MeJA responsive
TaMAPKKK152	LTR	CCGAAA	93	6 +	Hordeum vulgare	low temperature responsive
TaMAPKKK152	LTR	CCGAAA	872	6 +	Hordeum vulgare	low temperature responsive
TaMAPKKK152	MSA-like	(T/C)C(T/C)AAC GG(T/C)(T/C)A	1168	8.5 -	Catharanthus roseus	cell cycle regulation
TaMAPKKK152	TGACG-motif	TGACG	420	5 -	Hordeum vulgare	the MeJA responsive
TaMAPKKK153	GARE-motif	TCTGTTG	271	7 -	Brassica oleracea	gibberellin responsive
TaMAPKKK153	TGACG-motif	TGACG	676	5 +	Hordeum vulgare	the MeJA responsive
TaMAPKKK153	TGACG-motif	TGACG	1065	5 -	Hordeum vulgare	the MeJA responsive
TaMAPKKK153	motif I	gGTACGTGGCG	1459	10 +	Oryza sativa	root specific

TaMAPKKK153	O2-site	GATGATGTGG	1756	9 -	Zea mays	zein metabolism regulation
TaMAPKKK153	GC-motif	CCCCCG	1206	6 -	Zea mays	anoxic specific inducibility
TaMAPKKK153	GC-motif	CCCCCG	1872	6 -	Zea mays	anoxic specific inducibility
TaMAPKKK153	P-box	CCTTTTG	624	7 -	Oryza sativa	gibberellin responsive
TaMAPKKK153	MBS	CAACTG	126	6 +	Arabidopsis thaliana	drought inducibility
TaMAPKKK153	TATC-box	TATCCCA	205	7 -	Oryza sativa	gibberellin responsive
TaMAPKKK153	CGTCA-motif	CGTCA	676	5 -	Hordeum vulgare	the MeJA responsive
TaMAPKKK153	CGTCA-motif	CGTCA	1065	5 +	Hordeum vulgare	the MeJA responsive
TaMAPKKK154	CAT-box	GCCACT	1939	6 -	Arabidopsis thaliana	meristem expression
TaMAPKKK154	ABRE	ACGTG	722	5 -	Arabidopsis thaliana	abscisic acid responsive
TaMAPKKK154	ABRE	ACGTG	833	5 -	Arabidopsis thaliana	abscisic acid responsive
TaMAPKKK154	ABRE	ACGTG	1983	5 -	Arabidopsis thaliana	abscisic acid responsive
TaMAPKKK154	TGACG-motif	TGACG	308	5 -	Hordeum vulgare	the MeJA responsive
TaMAPKKK154	O2-site	GATGATGTGG	1734	10 +	Zea mays	zein metabolism regulation
TaMAPKKK154	O2-site	GATGA(C/T)(A/G)TG(A/G)	1856	8 +	Zea mays	zein metabolism regulation
TaMAPKKK154	RY-element	CATGCATG	1352	8 -	Helianthus annuus	seed specific regulation
TaMAPKKK154	MBS	CAACTG	287	6 -	Arabidopsis thaliana	drought inducibility
TaMAPKKK154	P-box	CCTTTTG	50	7 +	Oryza sativa	gibberellin responsive
TaMAPKKK154	P-box	CCTTTTG	1136	7 +	Oryza sativa	gibberellin responsive

TaMAPKKK154	P-box	CCTTTTG	1361	7 -	Oryza sativa	gibberellin responsive
TaMAPKKK154	CGTCA-motif	CGTCA	308	5 +	Hordeum vulgare	the MeJA responsive
TaMAPKKK154	LTR	CCGAAA	530	6 +	Hordeum vulgare	low temperature responsive
TaMAPKKK155	GC-motif	CCCCCG	1728	6 -	Zea mays	anoxic specific inducibility
TaMAPKKK155	circadian	CAAAGATATC	1858	9 +	Lycopersicon esculentum	circadian control
TaMAPKKK155	TCA-element	CCATCTTTTT	1884	9 +	Nicotiana tabacum	salicylic acid responsive
TaMAPKKK155	MBS	CAACTG	244	6 -	Arabidopsis thaliana	drought inducibility
TaMAPKKK155	MBS	CAACTG	1926	6 -	Arabidopsis thaliana	drought inducibility
TaMAPKKK155	ARE	AAACCA	108	6 -	Zea mays	anaerobic induction
TaMAPKKK155	ARE	AAACCA	1182	6 -	Zea mays	anaerobic induction
TaMAPKKK155	ARE	AAACCA	1576	6 +	Zea mays	anaerobic induction
TaMAPKKK155	ARE	AAACCA	1933	6 -	Zea mays	anaerobic induction
TaMAPKKK155	LTR	CCGAAA	1185	6 -	Hordeum vulgare	low temperature responsive
TaMAPKKK16	ARE	AAACCA	1409	6 +	Zea mays	anaerobic induction
TaMAPKKK16	TGACG-motif	TGACG	61	5 +	Hordeum vulgare	the MeJA responsive
TaMAPKKK16	TGACG-motif	TGACG	121	5 -	Hordeum vulgare	the MeJA responsive
TaMAPKKK16	TGACG-motif	TGACG	996	5 -	Hordeum vulgare	the MeJA responsive
TaMAPKKK16	TGACG-motif	TGACG	1110	5 +	Hordeum vulgare	the MeJA responsive
TaMAPKKK16	LTR	CCGAAA	271	6 -	Hordeum vulgare	low temperature responsive

TaMAPKKK16	MBS	CAACTG	1556	6 +	Arabidopsis thaliana	drought inducibility
TaMAPKKK16	CAT-box	GCCACT	1306	6 +	Arabidopsis thaliana	meristem expression
TaMAPKKK16	O2-site	GATGATGTGG	547	9 -	Zea mays	zein metabolism regulation
TaMAPKKK16	O2-site	GATGATGTGG	728	9 -	Zea mays	zein metabolism regulation
TaMAPKKK16	O2-site	GATGATGTGG	1566	9 -	Zea mays	zein metabolism regulation
TaMAPKKK16	CGTCA-motif	CGTCA	61	5 -	Hordeum vulgare	the MeJA responsive
TaMAPKKK16	CGTCA-motif	CGTCA	121	5 +	Hordeum vulgare	the MeJA responsive
TaMAPKKK16	CGTCA-motif	CGTCA	996	5 +	Hordeum vulgare	the MeJA responsive
TaMAPKKK16	CGTCA-motif	CGTCA	1110	5 -	Hordeum vulgare	the MeJA responsive
TaMAPKKK16	ABRE	GCCGCGTGGC	99	9 +	Oryza sativa	abscisic acid responsive
TaMAPKKK16	ABRE	CGCACGTGTC	137	9 +	Hordeum vulgare	abscisic acid responsive
TaMAPKKK16	ABRE	ACGTG	263	5 +	Arabidopsis thaliana	abscisic acid responsive
TaMAPKKK16	ABRE	ACGTG	290	5 -	Arabidopsis thaliana	abscisic acid responsive
TaMAPKKK16	ABRE	CGTACGTGCA	351	10 +	Hordeum vulgare	abscisic acid responsive
TaMAPKKK16	ABRE	ACGTG	354	5 +	Arabidopsis thaliana	abscisic acid responsive
TaMAPKKK16	ABRE	CACGTG	648	6 +	Arabidopsis thaliana	abscisic acid responsive
TaMAPKKK16	ABRE	ACGTG	649	5 +	Arabidopsis thaliana	abscisic acid responsive
TaMAPKKK16	TCA-element	TCAGAAGAGG	1024	9 +	Brassica oleracea	salicylic acid responsive
TaMAPKKK16	circadian	CAAAGATATC	1710	9 +	Lycopersicon esculentum	circadian control

TaMAPKKK17	TGACG-motif	TGACG	397	5 +	Hordeum vulgare	the MeJA responsive
TaMAPKKK17	TGACG-motif	TGACG	1301	5 +	Hordeum vulgare	the MeJA responsive
TaMAPKKK17	GC-motif	CCCCCG	1333	6 +	Zea mays	anoxic specific inducibility
TaMAPKKK17	GC-motif	CCCCCG	1434	6 +	Zea mays	anoxic specific inducibility
TaMAPKKK17	CAT-box	GCCACT	88	6 +	Arabidopsis thaliana	meristem expression
TaMAPKKK17	MSA-like	(T/C)C(T/C)AACGG(T/C)(T/C)A	282	8.5 -	Catharanthus roseus	cell cycle regulation
TaMAPKKK17	ABRE	ACGTG	399	5 +	Arabidopsis thaliana	abscisic acid responsive
TaMAPKKK17	ABRE	ACGTG	1303	5 +	Arabidopsis thaliana	abscisic acid responsive
TaMAPKKK17	ABRE	GACACGTACGT	1354	10 +	Oryza sativa	abscisic acid responsive
TaMAPKKK17	ABRE	ACGTG	1356	5 -	Arabidopsis thaliana	abscisic acid responsive
TaMAPKKK17	AuxRR-core	GGTCCAT	1421	7 +	Nicotiana tabacum	auxin responsive
TaMAPKKK17	CGTCA-motif	CGTCA	397	5 -	Hordeum vulgare	the MeJA responsive
TaMAPKKK17	CGTCA-motif	CGTCA	1301	5 -	Hordeum vulgare	the MeJA responsive
TaMAPKKK17	TATC-box	TATCCCA	1066	7 +	Oryza sativa	gibberellin responsive
TaMAPKKK17	GCN4_motif	TGAGTCA	901	7 +	Oryza sativa	endosperm expression
TaMAPKKK18	CGTCA-motif	CGTCA	1329	5 -	Hordeum vulgare	the MeJA responsive
TaMAPKKK18	GC-motif	CCCCCG	1312	6 +	Zea mays	anoxic specific inducibility
TaMAPKKK18	GC-motif	CCCCCG	1484	6 +	Zea mays	anoxic specific inducibility

TaMAPKKK18	P-box	CCTTTTG	805	7 -	Oryza sativa	gibberellin responsive
TaMAPKKK18	TGACG-motif	TGACG	1329	5 +	Hordeum vulgare	the MeJA responsive
TaMAPKKK18	TGA-element	AACGAC	53	6 -	Brassica oleracea	auxin responsive
TaMAPKKK18	ABRE	ACGTG	259	5 +	Arabidopsis thaliana	abscisic acid responsive
TaMAPKKK18	ABRE	ACGTG	1095	5 +	Arabidopsis thaliana	abscisic acid responsive
TaMAPKKK18	ABRE	GCAACGTGTC	1269	9 -	Hordeum vulgare	abscisic acid responsive
TaMAPKKK18	ABRE	CACGTG	1271	6 -	Arabidopsis thaliana	abscisic acid responsive
TaMAPKKK18	ABRE	ACGTG	1272	5 +	Arabidopsis thaliana	abscisic acid responsive
TaMAPKKK2	ABRE	ACGTG	157	5 +	Arabidopsis thaliana	abscisic acid responsive
TaMAPKKK2	ABRE	ACGTG	350	5 +	Arabidopsis thaliana	abscisic acid responsive
TaMAPKKK2	CGTCA-motif	CGTCA	348	5 -	Hordeum vulgare	the MeJA responsive
TaMAPKKK2	CGTCA-motif	CGTCA	1288	5 +	Hordeum vulgare	the MeJA responsive
TaMAPKKK2	CGTCA-motif	CGTCA	1364	5 -	Hordeum vulgare	the MeJA responsive
TaMAPKKK2	LTR	CCGAAA	89	6 -	Hordeum vulgare	low temperature responsive
TaMAPKKK2	AuxRR-core	GGTCCAT	1745	7 -	Nicotiana tabacum	auxin responsive
TaMAPKKK2	TGACG-motif	TGACG	348	5 +	Hordeum vulgare	the MeJA responsive
TaMAPKKK2	TGACG-motif	TGACG	1288	5 -	Hordeum vulgare	the MeJA responsive
TaMAPKKK2	TGACG-motif	TGACG	1364	5 +	Hordeum vulgare	the MeJA responsive
TaMAPKKK2	ARE	AAACCA	455	6 -	Zea mays	anaerobic induction

TaMAPKKK2	ARE	AAACCA	524	6 -	Zea mays	anaerobic induction
TaMAPKKK2	ARE	AAACCA	650	6 -	Zea mays	anaerobic induction
TaMAPKKK2	GC-motif	CCCCCG	1611	6 +	Zea mays	anoxic specific inducibility
TaMAPKKK2	GC-motif	CCCCCG	1705	6 +	Zea mays	anoxic specific inducibility
TaMAPKKK20	CGTCA-motif	CGTCA	10	5 +	Hordeum vulgare	the MeJA responsive
TaMAPKKK20	CGTCA-motif	CGTCA	889	5 +	Hordeum vulgare	the MeJA responsive
TaMAPKKK20	CGTCA-motif	CGTCA	984	5 -	Hordeum vulgare	the MeJA responsive
TaMAPKKK20	CGTCA-motif	CGTCA	1130	5 +	Hordeum vulgare	the MeJA responsive
TaMAPKKK20	CGTCA-motif	CGTCA	1573	5 -	Hordeum vulgare	the MeJA responsive
TaMAPKKK20	GC-motif	CCCCCG	1556	6 +	Zea mays	anoxic specific inducibility
TaMAPKKK20	GC-motif	CCCCCG	1626	6 +	Zea mays	anoxic specific inducibility
TaMAPKKK20	GC-motif	CCCCCG	1728	6 +	Zea mays	anoxic specific inducibility
TaMAPKKK20	ABRE	ACGTG	887	5 -	Arabidopsis thaliana	abscisic acid responsive
TaMAPKKK20	P-box	CCTTTTG	739	7 +	Oryza sativa	gibberellin responsive
TaMAPKKK20	P-box	CCTTTTG	1247	7 -	Oryza sativa	gibberellin responsive
TaMAPKKK20	TGACG-motif	TGACG	10	5 -	Hordeum vulgare	the MeJA responsive
TaMAPKKK20	TGACG-motif	TGACG	889	5 -	Hordeum vulgare	the MeJA responsive
TaMAPKKK20	TGACG-motif	TGACG	984	5 +	Hordeum vulgare	the MeJA responsive
TaMAPKKK20	TGACG-motif	TGACG	1130	5 -	Hordeum vulgare	the MeJA responsive

TaMAPKKK20	TGACG-motif	TGACG	1573	5 +	Hordeum vulgare	the MeJA responsive
TaMAPKKK20	circadian	CAAAGATATC	892	9 +	Lycopersicon esculentum	circadian control
TaMAPKKK20	O2-site	GTTGACGTGA	886	9 -	Zea mays	zein metabolism regulation
TaMAPKKK20	O2-site	GATGATGTGG	1031	9 +	Zea mays	zein metabolism regulation
TaMAPKKK23	ARE	AAACCA	856	6 +	Zea mays	anaerobic induction
TaMAPKKK23	GCN4_motif	TGAGTCA	780	7 +	Oryza sativa	endosperm expression
TaMAPKKK23	TGA-element	AACGAC	1051	6 +	Brassica oleracea	auxin responsive
TaMAPKKK23	TGA-element	AACGAC	1096	6 +	Brassica oleracea	auxin responsive
TaMAPKKK23	GC-motif	CCCCCG	325	6 +	Zea mays	anoxic specific inducibility
TaMAPKKK23	ABRE	CACGTG	487	6 +	Arabidopsis thaliana	abscisic acid responsive
TaMAPKKK23	ABRE	ACGTG	488	5 +	Arabidopsis thaliana	abscisic acid responsive
TaMAPKKK23	ABRE	ACGTG	943	5 +	Arabidopsis thaliana	abscisic acid responsive
TaMAPKKK23	ABRE	CACGTG	970	6 +	Arabidopsis thaliana	abscisic acid responsive
TaMAPKKK23	ABRE	ACGTG	971	5 +	Arabidopsis thaliana	abscisic acid responsive
TaMAPKKK23	ABRE	ACGTG	1010	5 +	Arabidopsis thaliana	abscisic acid responsive
TaMAPKKK23	P-box	CCTTTTG	165	7 -	Oryza sativa	gibberellin responsive
TaMAPKKK24	ARE	AAACCA	364	6 -	Zea mays	anaerobic induction
TaMAPKKK24	ARE	AAACCA	612	6 -	Zea mays	anaerobic induction
TaMAPKKK24	ARE	AAACCA	940	6 +	Zea mays	anaerobic induction
TaMAPKKK24	GC-motif	CCCCCG	1750	6 +	Zea mays	anoxic specific inducibility

TaMAPKKK24	GC-motif	CCCCCG	1851	6 +	Zea mays	anoxic specific inducibility
TaMAPKKK24	GC-motif	CCCCCG	1917	6 +	Zea mays	anoxic specific inducibility
TaMAPKKK24	TC-rich repeats	GTTTTCTTAC	614	9 +	Nicotiana tabacum	defense and stress responsive
TaMAPKKK24	O2-site	GATGACATGG	215	9 +	Zea mays	zein metabolism regulation
TaMAPKKK24	TGACG-motif	TGACG	1300	5 +	Hordeum vulgare	the MeJA responsive
TaMAPKKK24	TGACG-motif	TGACG	1718	5 +	Hordeum vulgare	the MeJA responsive
TaMAPKKK24	ABRE	ACGTG	1524	5 +	Arabidopsis thaliana	abscisic acid responsive
TaMAPKKK24	ABRE	ACGTG	1720	5 +	Arabidopsis thaliana	abscisic acid responsive
TaMAPKKK24	ABRE	GACACGTACG T	1771	10 +	Oryza sativa	abscisic acid responsive
TaMAPKKK24	ABRE	ACGTG	1773	5 -	Arabidopsis thaliana	abscisic acid responsive
TaMAPKKK24	CGTCA-motif	CGTCA	1300	5 -	Hordeum vulgare	the MeJA responsive
TaMAPKKK24	CGTCA-motif	CGTCA	1718	5 -	Hordeum vulgare	the MeJA responsive
TaMAPKKK24	AuxRR-core	GGTCCAT	869	7 -	Nicotiana tabacum	auxin responsive
TaMAPKKK24	AuxRR-core	GGTCCAT	1838	7 +	Nicotiana tabacum	auxin responsive
TaMAPKKK24	WUN-motif	AAATTTCCT	509	9 +	Brassica oleracea	wound responsive
TaMAPKKK24	TATC-box	TATCCCA	1329	7 -	Oryza sativa	gibberellin responsive
TaMAPKKK24	GCN4_motif	TGAGTCA	1352	7 +	Oryza sativa	endosperm expression
TaMAPKKK25	CGTCA-motif	CGTCA	52	5 -	Hordeum vulgare	the MeJA responsive
TaMAPKKK25	CGTCA-motif	CGTCA	1413	5 -	Hordeum vulgare	the MeJA responsive

TaMAPKKK25	CGTCA-motif	CGTCA	1484	5 +	Hordeum vulgare	the MeJA responsive
TaMAPKKK25	TCA-element	CCATCTTTTT	1782	9 +	Nicotiana tabacum	salicylic acid responsive
TaMAPKKK25	P-box	CCTTTTG	1240	7 +	Oryza sativa	gibberellin responsive
TaMAPKKK25	GARE-motif	TCTGTTG	1598	7 +	Brassica oleracea	gibberellin responsive
TaMAPKKK25	ABRE	ACGTG	54	5 +	Arabidopsis thaliana	abscisic acid responsive
TaMAPKKK25	ABRE	ACGTG	572	5 -	Arabidopsis thaliana	abscisic acid responsive
TaMAPKKK25	ABRE	GACACGTGGC	596	9 -	Triticum aestivum	abscisic acid responsive
TaMAPKKK25	ABRE	CACGTG	598	6 +	Arabidopsis thaliana	abscisic acid responsive
TaMAPKKK25	ABRE	ACGTG	599	5 +	Arabidopsis thaliana	abscisic acid responsive
TaMAPKKK25	ABRE	ACGTG	837	5 -	Arabidopsis thaliana	abscisic acid responsive
TaMAPKKK25	ABRE	ACGTG	1040	5 +	Arabidopsis thaliana	abscisic acid responsive
TaMAPKKK25	ABRE	CACGTG	1360	6 -	Arabidopsis thaliana	abscisic acid responsive
TaMAPKKK25	ABRE	ACGTG	1361	5 +	Arabidopsis thaliana	abscisic acid responsive
TaMAPKKK25	ABRE	CGCACGTGTC	1504	9 -	Hordeum vulgare	abscisic acid responsive
TaMAPKKK25	ABRE	CACGTG	1506	6 -	Arabidopsis thaliana	abscisic acid responsive
TaMAPKKK25	ABRE	ACGTG	1507	5 +	Arabidopsis thaliana	abscisic acid responsive
TaMAPKKK25	AuxRR-core	GGTCCAT	1340	7 -	Nicotiana tabacum	auxin responsive
TaMAPKKK25	TGACG-motif	TGACG	52	5 +	Hordeum vulgare	the MeJA responsive
TaMAPKKK25	TGACG-motif	TGACG	1413	5 +	Hordeum vulgare	the MeJA responsive

TaMAPKKK25	TGACG-motif	TGACG	1484	5 -	Hordeum vulgare	the MeJA responsive
TaMAPKKK25	LTR	CCGAAA	1433	6 -	Hordeum vulgare	low temperature responsive
TaMAPKKK25	MSA-like	(T/C)C(T/C)AAC GG(T/C)(T/C)A	1568	8.5 -	Catharanthus roseus	cell cycle regulation
TaMAPKKK25	ARE	AAACCA	222	6 +	Zea mays	anaerobic induction
TaMAPKKK25	ARE	AAACCA	1130	6 -	Zea mays	anaerobic induction
TaMAPKKK25	TATC-box	TATCCCA	1552	7 -	Oryza sativa	gibberellin responsive
TaMAPKKK25	MBS	CAACTG	226	6 +	Arabidopsis thaliana	drought inducibility
TaMAPKKK25	RY-element	CATGCATG	171	8 +	Helianthus annuus	seed specific regulation
TaMAPKKK26	LTR	CCGAAA	503	6 +	Hordeum vulgare	low temperature responsive
TaMAPKKK26	LTR	CCGAAA	628	6 -	Hordeum vulgare	low temperature responsive
TaMAPKKK26	CGTCA-motif	CGTCA	535	5 +	Hordeum vulgare	the MeJA responsive
TaMAPKKK26	CGTCA-motif	CGTCA	841	5 +	Hordeum vulgare	the MeJA responsive
TaMAPKKK26	TC-rich repeats	GTTTTCTTAC	1352	9 -	Nicotiana tabacum	defense and stress responsive
TaMAPKKK26	P-box	CCTTTTG	791	7 -	Oryza sativa	gibberellin responsive
TaMAPKKK26	MBS	CAACTG	217	6 +	Arabidopsis thaliana	drought inducibility
TaMAPKKK26	MBS	CAACTG	468	6 -	Arabidopsis thaliana	drought inducibility
TaMAPKKK26	MBS	CAACTG	597	6 -	Arabidopsis thaliana	drought inducibility
TaMAPKKK26	ARE	AAACCA	99	6 +	Zea mays	anaerobic induction

TaMAPKKK26	ARE	AAACCA	270	6 -	Zea mays	anaerobic induction
TaMAPKKK26	TGACG-motif	TGACG	535	5 -	Hordeum vulgare	the MeJA responsive
TaMAPKKK26	TGACG-motif	TGACG	841	5 -	Hordeum vulgare	the MeJA responsive
TaMAPKKK26	ABRE	ACGTG	282	5 -	Arabidopsis thaliana	abscisic acid responsive
TaMAPKKK26	ABRE	ACGTG	953	5 -	Arabidopsis thaliana	abscisic acid responsive
TaMAPKKK26	ABRE	ACGTG	982	5 -	Arabidopsis thaliana	abscisic acid responsive
TaMAPKKK26	ABRE	ACGTG	1780	5 -	Arabidopsis thaliana	abscisic acid responsive
TaMAPKKK26	CAT-box	GCCACT	1547	6 +	Arabidopsis thaliana	meristem expression
TaMAPKKK27	TGACG-motif	TGACG	818	5 -	Hordeum vulgare	the MeJA responsive
TaMAPKKK27	TGACG-motif	TGACG	1783	5 -	Hordeum vulgare	the MeJA responsive
TaMAPKKK27	TCA-element	CCATCTTTTT	82	9 -	Nicotiana tabacum	salicylic acid responsive
TaMAPKKK27	ARE	AAACCA	67	6 +	Zea mays	anaerobic induction
TaMAPKKK27	ARE	AAACCA	581	6 -	Zea mays	anaerobic induction
TaMAPKKK27	ARE	AAACCA	602	6 -	Zea mays	anaerobic induction
TaMAPKKK27	ARE	AAACCA	611	6 -	Zea mays	anaerobic induction
TaMAPKKK27	circadian	CAAAGATATC	1390	9 +	Lycopersicon esculentum	circadian control
TaMAPKKK27	CAT-box	GCCACT	1555	6 +	Arabidopsis thaliana	meristem expression
TaMAPKKK27	CGTCA-motif	CGTCA	818	5 +	Hordeum vulgare	the MeJA responsive
TaMAPKKK27	CGTCA-motif	CGTCA	1783	5 +	Hordeum vulgare	the MeJA responsive

TaMAPKKK27	ABRE	ACGTG	233	5 -	Arabidopsis thaliana	abscisic acid responsive
TaMAPKKK27	ABRE	ACGTG	1778	5 -	Arabidopsis thaliana	abscisic acid responsive
TaMAPKKK27	MBS	CAACTG	440	6 -	Arabidopsis thaliana	drought inducibility
TaMAPKKK27	MBS	CAACTG	569	6 -	Arabidopsis thaliana	drought inducibility
TaMAPKKK28	TC-rich repeats	GTTTTCTTAC	1361	9 -	Nicotiana tabacum	defense and stress responsive
TaMAPKKK28	MBS	CAACTG	223	6 -	Arabidopsis thaliana	drought inducibility
TaMAPKKK28	CGTCA-motif	CGTCA	590	5 +	Hordeum vulgare	the MeJA responsive
TaMAPKKK28	CGTCA-motif	CGTCA	853	5 -	Hordeum vulgare	the MeJA responsive
TaMAPKKK28	ABRE	ACGTG	44	5 -	Arabidopsis thaliana	abscisic acid responsive
TaMAPKKK28	ABRE	CACGTG	202	6 +	Arabidopsis thaliana	abscisic acid responsive
TaMAPKKK28	ABRE	ACGTG	203	5 +	Arabidopsis thaliana	abscisic acid responsive
TaMAPKKK28	ABRE	GCAACGTGTC	584	9 +	Hordeum vulgare	abscisic acid responsive
TaMAPKKK28	ABRE	ACGTG	873	5 -	Arabidopsis thaliana	abscisic acid responsive
TaMAPKKK28	ABRE	ACGTG	1778	5 -	Arabidopsis thaliana	abscisic acid responsive
TaMAPKKK28	P-box	CCTTTTG	540	7 -	Oryza sativa	gibberellin responsive
TaMAPKKK28	LTR	CCGAAA	258	6 +	Hordeum vulgare	low temperature responsive
TaMAPKKK28	LTR	CCGAAA	369	6 -	Hordeum vulgare	low temperature responsive
TaMAPKKK28	LTR	CCGAAA	656	6 -	Hordeum vulgare	low temperature responsive
TaMAPKKK28	TGACG-motif	TGACG	590	5 -	Hordeum vulgare	the MeJA responsive

TaMAPKKK28	TGACG-motif	TGACG	853	5 +	Hordeum vulgare	the MeJA responsive
TaMAPKKK28	ARE	AAACCA	381	6 -	Zea mays	anaerobic induction
TaMAPKKK29	CGTCA-motif	CGTCA	1141	5 -	Hordeum vulgare	the MeJA responsive
TaMAPKKK29	CGTCA-motif	CGTCA	1704	5 -	Hordeum vulgare	the MeJA responsive
TaMAPKKK29	AuxRR-core	GGTCCAT	855	7 -	Nicotiana tabacum	auxin responsive
TaMAPKKK29	AuxRR-core	GGTCCAT	1828	7 +	Nicotiana tabacum	auxin responsive
TaMAPKKK29	TGACG-motif	TGACG	1141	5 +	Hordeum vulgare	the MeJA responsive
TaMAPKKK29	TGACG-motif	TGACG	1704	5 +	Hordeum vulgare	the MeJA responsive
TaMAPKKK29	TC-rich repeats	GTTTTCTTAC	286	9 +	Nicotiana tabacum	defense and stress responsive
TaMAPKKK29	LTR	CCGAAA	1781	6 +	Hordeum vulgare	low temperature responsive
TaMAPKKK29	GCN4_motif	TGAGTCA	1347	7 +	Oryza sativa	endosperm expression
TaMAPKKK29	ARE	AAACCA	34	6 -	Zea mays	anaerobic induction
TaMAPKKK29	ARE	AAACCA	284	6 -	Zea mays	anaerobic induction
TaMAPKKK29	ARE	AAACCA	926	6 +	Zea mays	anaerobic induction
TaMAPKKK29	CAT-box	GCCACT	457	6 -	Arabidopsis thaliana	meristem expression
TaMAPKKK29	TATC-box	TATCCCA	1324	7 -	Oryza sativa	gibberellin responsive
TaMAPKKK29	WUN-motif	AAATTTCCT	180	9 +	Brassica oleracea	wound responsive
TaMAPKKK29	ABRE	ACGTG	1517	5 +	Arabidopsis thaliana	abscisic acid responsive
TaMAPKKK29	ABRE	ACGTG	1706	5 +	Arabidopsis thaliana	abscisic acid responsive

TaMAPKKK29	ABRE	ACGTG	1766	5 -	Arabidopsis thaliana	abscisic acid responsive
TaMAPKKK29	GC-motif	CCCCCG	1743	6 +	Zea mays	anoxic specific inducibility
TaMAPKKK29	GC-motif	CCCCCG	1841	6 +	Zea mays	anoxic specific inducibility
TaMAPKKK29	GC-motif	CCCCCG	1918	6 +	Zea mays	anoxic specific inducibility
TaMAPKKK3	CAT-box	GCCACT	554	6 +	Arabidopsis thaliana	meristem expression
TaMAPKKK3	GC-motif	CCCCCG	673	6 +	Zea mays	anoxic specific inducibility
TaMAPKKK3	GC-motif	CCCCCG	1931	6 +	Zea mays	anoxic specific inducibility
TaMAPKKK3	GC-motif	CCCCCG	1983	6 +	Zea mays	anoxic specific inducibility
TaMAPKKK3	ARE	AAACCA	1327	6 -	Zea mays	anaerobic induction
TaMAPKKK3	ARE	AAACCA	1784	6 +	Zea mays	anaerobic induction
TaMAPKKK3	O2-site	GTTGACGTGA	632	9 +	Zea mays	zein metabolism regulation
TaMAPKKK3	TGACG-motif	TGACG	634	5 +	Hordeum vulgare	the MeJA responsive
TaMAPKKK3	TGACG-motif	TGACG	895	5 +	Hordeum vulgare	the MeJA responsive
TaMAPKKK3	GARE-motif	TCTGTTG	170	7 +	Brassica oleracea	gibberellin responsive
TaMAPKKK3	CGTCA-motif	CGTCA	634	5 -	Hordeum vulgare	the MeJA responsive
TaMAPKKK3	CGTCA-motif	CGTCA	895	5 -	Hordeum vulgare	the MeJA responsive
TaMAPKKK3	MBS	CAACTG	1191	6 -	Arabidopsis thaliana	drought inducibility
TaMAPKKK3	ABRE	ACGTG	596	5 +	Arabidopsis thaliana	abscisic acid responsive
TaMAPKKK3	ABRE	ACGTG	636	5 +	Arabidopsis thaliana	abscisic acid responsive

TaMAPKKK3	ABRE	TACGTGTC	750	8 -	Oryza sativa	abscisic acid responsive
TaMAPKKK3	ABRE	ACGTG	752	5 -	Arabidopsis thaliana	abscisic acid responsive
TaMAPKKK3	ABRE	ACGTG	770	5 -	Arabidopsis thaliana	abscisic acid responsive
TaMAPKKK3	ABRE	ACGTG	935	5 +	Arabidopsis thaliana	abscisic acid responsive
TaMAPKKK3	ABRE	CACGTG	950	6 +	Arabidopsis thaliana	abscisic acid responsive
TaMAPKKK3	ABRE	ACGTG	951	5 +	Arabidopsis thaliana	abscisic acid responsive
TaMAPKKK3	ABRE	ACGTG	983	5 -	Arabidopsis thaliana	abscisic acid responsive
TaMAPKKK3	ABRE	GCCGCGTGGC	1264	9 +	Oryza sativa	abscisic acid responsive
TaMAPKKK3	ABRE	CGTACGTGCA	1313	9 +	Hordeum vulgare	abscisic acid responsive
TaMAPKKK30	CGTCA-motif	CGTCA	223	5 -	Hordeum vulgare	the MeJA responsive
TaMAPKKK30	CGTCA-motif	CGTCA	426	5 +	Hordeum vulgare	the MeJA responsive
TaMAPKKK30	CGTCA-motif	CGTCA	1614	5 -	Hordeum vulgare	the MeJA responsive
TaMAPKKK30	CGTCA-motif	CGTCA	1759	5 +	Hordeum vulgare	the MeJA responsive
TaMAPKKK30	CGTCA-motif	CGTCA	1811	5 +	Hordeum vulgare	the MeJA responsive
TaMAPKKK30	TGACG-motif	TGACG	223	5 +	Hordeum vulgare	the MeJA responsive
TaMAPKKK30	TGACG-motif	TGACG	426	5 -	Hordeum vulgare	the MeJA responsive
TaMAPKKK30	TGACG-motif	TGACG	1614	5 +	Hordeum vulgare	the MeJA responsive
TaMAPKKK30	TGACG-motif	TGACG	1759	5 -	Hordeum vulgare	the MeJA responsive
TaMAPKKK30	TGACG-motif	TGACG	1811	5 -	Hordeum vulgare	the MeJA responsive

TaMAPKKK30	TC-rich repeats	ATTCTCTAAC	545	9 -	Nicotiana tabacum	defense and stress responsive
TaMAPKKK30	O2-site	GATGATGTGG	1436	9 -	Zea mays	zein metabolism regulation
TaMAPKKK30	ARE	AAACCA	323	6 +	Zea mays	anaerobic induction
TaMAPKKK30	ARE	AAACCA	412	6 +	Zea mays	anaerobic induction
TaMAPKKK30	ARE	AAACCA	719	6 +	Zea mays	anaerobic induction
TaMAPKKK30	HD-Zip 1	CAAT(A/T)ATT G	167	8.5 +	Arabidopsis thaliana	differentiation of the palisade mesophyll cells
TaMAPKKK30	GARE-motif	TCTGTTG	1291	7 -	Brassica oleracea	gibberellin responsive
TaMAPKKK30	MBS	CAACTG	507	6 -	Arabidopsis thaliana	drought inducibility
TaMAPKKK30	ABRE	CACGTG	998	6 +	Arabidopsis thaliana	abscisic acid responsive
TaMAPKKK30	ABRE	ACGTG	999	5 +	Arabidopsis thaliana	abscisic acid responsive
TaMAPKKK31	TGACG-motif	TGACG	883	5 -	Hordeum vulgare	the MeJA responsive
TaMAPKKK31	TGACG-motif	TGACG	1856	5 -	Hordeum vulgare	the MeJA responsive
TaMAPKKK31	CGTCA-motif	CGTCA	883	5 +	Hordeum vulgare	the MeJA responsive
TaMAPKKK31	CGTCA-motif	CGTCA	1856	5 +	Hordeum vulgare	the MeJA responsive
TaMAPKKK31	GC-motif	CCCCCG	257	6 +	Zea mays	anoxic specific inducibility
TaMAPKKK31	GC-motif	CCCCCG	1008	6 +	Zea mays	anoxic specific inducibility
TaMAPKKK31	GC-motif	CCCCCG	1785	6 +	Zea mays	anoxic specific inducibility
TaMAPKKK31	TGA-element	AACGAC	861	6 -	Brassica oleracea	auxin responsive
TaMAPKKK31	ABRE	AACCCGG	706	7 +	Arabidopsis thaliana	abscisic acid responsive

TaMAPKKK31	ARE	AAACCA	1019	6 -	Zea mays	anaerobic induction
TaMAPKKK31	CAT-box	GCCACT	731	6 -	Arabidopsis thaliana	meristem expression
TaMAPKKK31	MBS	CAACTG	62	6 -	Arabidopsis thaliana	drought inducibility
TaMAPKKK32	ARE	AAACCA	1317	6 +	Zea mays	anaerobic induction
TaMAPKKK32	ARE	AAACCA	1461	6 -	Zea mays	anaerobic induction
TaMAPKKK32	TGACG-motif	TGACG	731	5 +	Hordeum vulgare	the MeJA responsive
TaMAPKKK32	TCA-element	TCAGAAGAGG	1350	9 -	Brassica oleracea	salicylic acid responsive
TaMAPKKK32	LTR	CCGAAA	1284	6 -	Hordeum vulgare	low temperature responsive
TaMAPKKK32	O2-site	GATGA(C/T)(A/G)TG(A/G)	787	8 -	Zea mays	zein metabolism regulation
TaMAPKKK32	O2-site	GATGATGTGG	1729	9 -	Zea mays	zein metabolism regulation
TaMAPKKK32	ABRE	ACGTG	33	5 +	Arabidopsis thaliana	abscisic acid responsive
TaMAPKKK32	ABRE	GCCGCGTGGC	65	9 -	Oryza sativa	abscisic acid responsive
TaMAPKKK32	ABRE	ACGTG	1324	5 +	Arabidopsis thaliana	abscisic acid responsive
TaMAPKKK32	GC-motif	CCCCCG	963	6 -	Zea mays	anoxic specific inducibility
TaMAPKKK32	GC-motif	CCCCCG	1861	6 +	Zea mays	anoxic specific inducibility
TaMAPKKK32	CAT-box	GCCACT	420	6 +	Arabidopsis thaliana	meristem expression
TaMAPKKK32	CGTCA-motif	CGTCA	731	5 -	Hordeum vulgare	the MeJA responsive
TaMAPKKK32	TGA-element	AACGAC	1279	6 -	Brassica oleracea	auxin responsive
TaMAPKKK33	GC-motif	CCCCCG	1797	6 +	Zea mays	anoxic specific inducibility
TaMAPKKK33	TGA-element	AACGAC	934	6 +	Brassica oleracea	auxin responsive

TaMAPKKK33	ABRE	GCCGCGTGGC	36	9 -	Oryza sativa	abscisic acid responsive
TaMAPKKK33	ABRE	GACACGTGGC	205	9 +	Triticum aestivum	abscisic acid responsive
TaMAPKKK33	ABRE	ACGTG	219	5 -	Arabidopsis thaliana	abscisic acid responsive
TaMAPKKK33	ABRE	ACGTG	858	5 -	Arabidopsis thaliana	abscisic acid responsive
TaMAPKKK33	P-box	CCTTTTG	195	7 +	Oryza sativa	gibberellin responsive
TaMAPKKK33	TCA-element	CCATCTTTTT	1926	9 +	Nicotiana tabacum	salicylic acid responsive
TaMAPKKK33	TCA-element	CCATCTTTTT	1950	9 +	Nicotiana tabacum	salicylic acid responsive
TaMAPKKK33	circadian	CAAAGATATC	1230	9 -	Lycopersicon esculentum	circadian control
TaMAPKKK33	MBS	CAACTG	131	6 +	Arabidopsis thaliana	drought inducibility
TaMAPKKK33	MBS	CAACTG	1070	6 -	Arabidopsis thaliana	drought inducibility
TaMAPKKK33	GARE-motif	TCTGTTG	1099	7 +	Brassica oleracea	gibberellin responsive
TaMAPKKK33	GARE-motif	TCTGTTG	1265	7 +	Brassica oleracea	gibberellin responsive
TaMAPKKK33	CAT-box	GCCACT	257	6 -	Arabidopsis thaliana	meristem expression
TaMAPKKK33	CGTCA-motif	CGTCA	1868	5 +	Hordeum vulgare	the MeJA responsive
TaMAPKKK33	TGACG-motif	TGACG	1868	5 -	Hordeum vulgare	the MeJA responsive
TaMAPKKK34	TGACG-motif	TGACG	1101	5 +	Hordeum vulgare	the MeJA responsive
TaMAPKKK34	O2-site	GATGACATGG	388	9 -	Zea mays	zein metabolism regulation
TaMAPKKK34	TC-rich repeats	GTTTTCTTAC	220	9 +	Nicotiana tabacum	defense and stress responsive
TaMAPKKK34	RY-element	CATGCATG	835	8 +	Helianthus annuus	seed specific regulation

TaMAPKKK34	MBS	CAACTG	408	6 +	Arabidopsis thaliana	drought inducibility
TaMAPKKK34	MBS	CAACTG	1364	6 -	Arabidopsis thaliana	drought inducibility
TaMAPKKK34	LTR	CCGAAA	606	6 -	Hordeum vulgare	low temperature responsive
TaMAPKKK34	CGTCA-motif	CGTCA	1101	5 -	Hordeum vulgare	the MeJA responsive
TaMAPKKK34	TGA-element	AACGAC	1245	6 +	Brassica oleracea	auxin responsive
TaMAPKKK34	CAT-box	GCCACT	167	6 +	Arabidopsis thaliana	meristem expression
TaMAPKKK34	ABRE	ACGTG	163	5 +	Arabidopsis thaliana	abscisic acid responsive
TaMAPKKK34	ABRE	CACGTG	1182	6 -	Arabidopsis thaliana	abscisic acid responsive
TaMAPKKK34	ABRE	ACGTG	1183	5 +	Arabidopsis thaliana	abscisic acid responsive
TaMAPKKK34	ABRE	ACGTG	1576	5 +	Arabidopsis thaliana	abscisic acid responsive
TaMAPKKK34	ABRE	GCCGCGTGGC	1688	9 -	Oryza sativa	abscisic acid responsive
TaMAPKKK34	ABRE	ACGTG	1765	5 +	Arabidopsis thaliana	abscisic acid responsive
TaMAPKKK34	ABRE	CGCACGTGTC	1836	9 +	Hordeum vulgare	abscisic acid responsive
TaMAPKKK34	ABRE	CACGTG	1838	6 -	Arabidopsis thaliana	abscisic acid responsive
TaMAPKKK34	ABRE	ACGTG	1839	5 +	Arabidopsis thaliana	abscisic acid responsive
TaMAPKKK34	GARE-motif	TCTGTTG	313	7 -	Brassica oleracea	gibberellin responsive
TaMAPKKK34	ARE	AAACCA	972	6 +	Zea mays	anaerobic induction
TaMAPKKK34	P-box	CCTTTTG	501	7 -	Oryza sativa	gibberellin responsive
TaMAPKKK36	GC-motif	CCCCCG	980	6 -	Zea mays	anoxic specific inducibility

TaMAPKKK36	GC-motif	CCCCCG	1233	6 +	Zea mays	anoxic specific inducibility
TaMAPKKK36	O2-site	GATGACATGG	186	9 -	Zea mays	zein metabolism regulation
TaMAPKKK36	O2-site	GATGATGTGG	1721	9 -	Zea mays	zein metabolism regulation
TaMAPKKK36	CGTCA-motif	CGTCA	581	5 -	Hordeum vulgare	the MeJA responsive
TaMAPKKK36	CGTCA-motif	CGTCA	740	5 +	Hordeum vulgare	the MeJA responsive
TaMAPKKK36	CGTCA-motif	CGTCA	760	5 -	Hordeum vulgare	the MeJA responsive
TaMAPKKK36	LTR	CCGAAA	1299	6 -	Hordeum vulgare	low temperature responsive
TaMAPKKK36	TGA-element	AACGAC	215	6 +	Brassica oleracea	auxin responsive
TaMAPKKK36	TGA-element	AACGAC	1294	6 -	Brassica oleracea	auxin responsive
TaMAPKKK36	TGA-element	AACGAC	1567	6 +	Brassica oleracea	auxin responsive
TaMAPKKK36	ABRE	GCCGCGTGGC	267	9 +	Oryza sativa	abscisic acid responsive
TaMAPKKK36	ABRE	GCAACGTGTC	386	10 -	Hordeum vulgare	abscisic acid responsive
TaMAPKKK36	ABRE	ACGTG	388	5 -	Arabidopsis thaliana	abscisic acid responsive
TaMAPKKK36	CAT-box	GCCACT	527	6 -	Arabidopsis thaliana	meristem expression
TaMAPKKK36	ARE	AAACCA	1332	6 +	Zea mays	anaerobic induction
TaMAPKKK36	TGACG-motif	TGACG	581	5 +	Hordeum vulgare	the MeJA responsive
TaMAPKKK36	TGACG-motif	TGACG	740	5 -	Hordeum vulgare	the MeJA responsive
TaMAPKKK36	TGACG-motif	TGACG	760	5 +	Hordeum vulgare	the MeJA responsive
TaMAPKKK37	ABRE	AACCCGG	594	7 +	Arabidopsis thaliana	abscisic acid responsive
TaMAPKKK37	ABRE	ACGTG	824	5 -	Arabidopsis thaliana	abscisic acid responsive

TaMAPKKK37	ABRE	AACCCGG	1397	7 -	Arabidopsis thaliana	abscisic acid responsive
TaMAPKKK37	ABRE	GCCGCGTGGC	1697	9 -	Oryza sativa	abscisic acid responsive
TaMAPKKK37	ABRE	CGCACGTGTC	1840	9 +	Hordeum vulgare	abscisic acid responsive
TaMAPKKK37	ABRE	CACGTG	1842	6 -	Arabidopsis thaliana	abscisic acid responsive
TaMAPKKK37	ABRE	ACGTG	1843	5 +	Arabidopsis thaliana	abscisic acid responsive
TaMAPKKK37	TGA-element	AACGAC	98	6 -	Brassica oleracea	auxin responsive
TaMAPKKK37	TGA-element	AACGAC	215	6 -	Brassica oleracea	auxin responsive
TaMAPKKK37	CGTCA-motif	CGTCA	934	5 -	Hordeum vulgare	the MeJA responsive
TaMAPKKK37	CGTCA-motif	CGTCA	1300	5 +	Hordeum vulgare	the MeJA responsive
TaMAPKKK37	CGTCA-motif	CGTCA	1619	5 +	Hordeum vulgare	the MeJA responsive
TaMAPKKK37	O2-site	GATGATGTGG	632	9 +	Zea mays	zein metabolism regulation
TaMAPKKK37	TGACG-motif	TGACG	934	5 +	Hordeum vulgare	the MeJA responsive
TaMAPKKK37	TGACG-motif	TGACG	1300	5 -	Hordeum vulgare	the MeJA responsive
TaMAPKKK37	TGACG-motif	TGACG	1619	5 -	Hordeum vulgare	the MeJA responsive
TaMAPKKK38	GC-motif	CCCCCG	1483	6 -	Zea mays	anoxic specific inducibility
TaMAPKKK38	ABRE	AACCCGG	473	7 -	Arabidopsis thaliana	abscisic acid responsive
TaMAPKKK38	ABRE	ACGTG	623	5 -	Arabidopsis thaliana	abscisic acid responsive
TaMAPKKK38	ABRE	ACGTG	761	5 +	Arabidopsis thaliana	abscisic acid responsive
TaMAPKKK38	ABRE	ACGTG	842	5 +	Arabidopsis thaliana	abscisic acid responsive
TaMAPKKK38	ABRE	ACGTG	1630	5 +	Arabidopsis thaliana	abscisic acid responsive

TaMAPKKK38	ABRE	ACGTG	1695	5 -	Arabidopsis thaliana	abscisic acid responsive
TaMAPKKK38	CGTCA-motif	CGTCA	817	5 -	Hordeum vulgare	the MeJA responsive
TaMAPKKK38	CGTCA-motif	CGTCA	826	5 +	Hordeum vulgare	the MeJA responsive
TaMAPKKK38	CGTCA-motif	CGTCA	840	5 -	Hordeum vulgare	the MeJA responsive
TaMAPKKK38	TC-rich repeats	ATTCTCTAAC	540	9 -	Nicotiana tabacum	defense and stress responsive
TaMAPKKK38	ARE	AAACCA	379	6 -	Zea mays	anaerobic induction
TaMAPKKK38	ARE	AAACCA	448	6 -	Zea mays	anaerobic induction
TaMAPKKK38	ARE	AAACCA	1244	6 -	Zea mays	anaerobic induction
TaMAPKKK38	ARE	AAACCA	1260	6 +	Zea mays	anaerobic induction
TaMAPKKK38	TCA-element	CCATCTTTTT	711	9 +	Nicotiana tabacum	salicylic acid responsive
TaMAPKKK38	TGACG-motif	TGACG	817	5 +	Hordeum vulgare	the MeJA responsive
TaMAPKKK38	TGACG-motif	TGACG	826	5 -	Hordeum vulgare	the MeJA responsive
TaMAPKKK38	TGACG-motif	TGACG	840	5 +	Hordeum vulgare	the MeJA responsive
TaMAPKKK38	LTR	CCGAAA	438	6 +	Hordeum vulgare	low temperature responsive
TaMAPKKK38	GCN4_motif	TGAGTCA	1877	7 -	Oryza sativa	endosperm expression
TaMAPKKK38	O2-site	GATGA(C/T)(A/G)TG(A/G)	838	8 +	Zea mays	zein metabolism regulation
TaMAPKKK39	TCA-element	TCAGAAGAGG	771	9 +	Brassica oleracea	salicylic acid responsive
TaMAPKKK39	GC-motif	CCCCCG	1797	6 +	Zea mays	anoxic specific inducibility
TaMAPKKK39	ABRE	ACGTG	815	5 -	Arabidopsis thaliana	abscisic acid responsive

TaMAPKKK39	ABRE	ACGTG	877	5 +	Arabidopsis thaliana	abscisic acid responsive
TaMAPKKK39	ABRE	CACGTG	1026	6 -	Arabidopsis thaliana	abscisic acid responsive
TaMAPKKK39	ABRE	ACGTG	1027	5 +	Arabidopsis thaliana	abscisic acid responsive
TaMAPKKK39	ABRE	GCAACGTGTC	1058	9 +	Hordeum vulgare	abscisic acid responsive
TaMAPKKK39	RY-element	CATGCATG	1212	8 -	Helianthus annuus	seed specific regulation
TaMAPKKK39	LTR	CCGAAA	715	6 -	Hordeum vulgare	low temperature responsive
TaMAPKKK39	AuxRR-core	GGTCCAT	24	7 +	Nicotiana tabacum	auxin responsive
TaMAPKKK39	CGTCA-motif	CGTCA	690	5 -	Hordeum vulgare	the MeJA responsive
TaMAPKKK39	CGTCA-motif	CGTCA	1230	5 +	Hordeum vulgare	the MeJA responsive
TaMAPKKK39	CGTCA-motif	CGTCA	1868	5 +	Hordeum vulgare	the MeJA responsive
TaMAPKKK39	TGACG-motif	TGACG	690	5 +	Hordeum vulgare	the MeJA responsive
TaMAPKKK39	TGACG-motif	TGACG	1230	5 -	Hordeum vulgare	the MeJA responsive
TaMAPKKK39	TGACG-motif	TGACG	1868	5 -	Hordeum vulgare	the MeJA responsive
TaMAPKKK39	MSA-like	(T/C)C(T/C)AAC GG(T/C)(T/C)A	934	8.5 -	Catharanthus roseus	cell cycle regulation
TaMAPKKK39	MSA-like	TCAAACGGT	936	9 -	Catharanthus roseus	cell cycle regulation
TaMAPKKK39	CAT-box	GCCACT	626	6 +	Arabidopsis thaliana	meristem expression
TaMAPKKK39	ARE	AAACCA	335	6 -	Zea mays	anaerobic induction
TaMAPKKK39	ARE	AAACCA	827	6 -	Zea mays	anaerobic induction

TaMAPKKK39	ARE	AAACCA	1079	6 -	Zea mays	anaerobic induction
TaMAPKKK4	TGA-element	AACGAC	941	6 -	Brassica oleracea	auxin responsive
TaMAPKKK4	MBS	CAACTG	538	6 -	Arabidopsis thaliana	drought inducibility
TaMAPKKK4	MBS	CAACTG	1687	6 -	Arabidopsis thaliana	drought inducibility
TaMAPKKK4	MBS	CAACTG	1904	6 +	Arabidopsis thaliana	drought inducibility
TaMAPKKK4	ARE	AAACCA	595	6 -	Zea mays	anaerobic induction
TaMAPKKK4	ARE	AAACCA	1398	6 +	Zea mays	anaerobic induction
TaMAPKKK4	LTR	CCGAAA	73	6 -	Hordeum vulgare	low temperature responsive
TaMAPKKK4	TGACG-motif	TGACG	1164	5 +	Hordeum vulgare	the MeJA responsive
TaMAPKKK4	CGTCA-motif	CGTCA	1164	5 -	Hordeum vulgare	the MeJA responsive
TaMAPKKK40	TC-rich repeats	ATTCTCTAAC	1557	9 -	Nicotiana tabacum	defense and stress responsive
TaMAPKKK40	TGACG-motif	TGACG	74	5 +	Hordeum vulgare	the MeJA responsive
TaMAPKKK40	TGACG-motif	TGACG	1245	5 +	Hordeum vulgare	the MeJA responsive
TaMAPKKK40	TGACG-motif	TGACG	1437	5 -	Hordeum vulgare	the MeJA responsive
TaMAPKKK40	TGACG-motif	TGACG	1742	5 -	Hordeum vulgare	the MeJA responsive
TaMAPKKK40	TGACG-motif	TGACG	1794	5 -	Hordeum vulgare	the MeJA responsive
TaMAPKKK40	O2-site	GATGATGTGG	29	9 -	Zea mays	zein metabolism regulation
TaMAPKKK40	MBS	CAACTG	70	6 -	Arabidopsis thaliana	drought inducibility
TaMAPKKK40	MBS	CAACTG	531	6 +	Arabidopsis thaliana	drought inducibility

TaMAPKKK40	MBS	CAACTG	609	6 -	Arabidopsis thaliana	drought inducibility
TaMAPKKK40	MBS	CAACTG	744	6 +	Arabidopsis thaliana	drought inducibility
TaMAPKKK40	MBS	CAACTG	1518	6 -	Arabidopsis thaliana	drought inducibility
TaMAPKKK40	ARE	AAACCA	1261	6 -	Zea mays	anaerobic induction
TaMAPKKK40	ARE	AAACCA	1328	6 +	Zea mays	anaerobic induction
TaMAPKKK40	ARE	AAACCA	1423	6 +	Zea mays	anaerobic induction
TaMAPKKK40	GC-motif	CCCCCG	1788	6 -	Zea mays	anoxic specific inducibility
TaMAPKKK40	HD-Zip 1	CAAT(A/T)ATTG	1160	8.5 -	Arabidopsis thaliana	differentiation of the palisade mesophyll cells
TaMAPKKK40	CGTCA-motif	CGTCA	74	5 -	Hordeum vulgare	the MeJA responsive
TaMAPKKK40	CGTCA-motif	CGTCA	1245	5 -	Hordeum vulgare	the MeJA responsive
TaMAPKKK40	CGTCA-motif	CGTCA	1437	5 +	Hordeum vulgare	the MeJA responsive
TaMAPKKK40	CGTCA-motif	CGTCA	1742	5 +	Hordeum vulgare	the MeJA responsive
TaMAPKKK40	CGTCA-motif	CGTCA	1794	5 +	Hordeum vulgare	the MeJA responsive
TaMAPKKK40	TGA-element	AACGAC	971	6 +	Brassica oleracea	auxin responsive
TaMAPKKK40	ABRE	ACGTG	1135	5 +	Arabidopsis thaliana	abscisic acid responsive
TaMAPKKK41	ARE	AAACCA	84	6 +	Zea mays	anaerobic induction
TaMAPKKK41	CGTCA-motif	CGTCA	733	5 -	Hordeum vulgare	the MeJA responsive
TaMAPKKK41	CGTCA-motif	CGTCA	1877	5 +	Hordeum vulgare	the MeJA responsive
TaMAPKKK41	TGACG-motif	TGACG	733	5 +	Hordeum vulgare	the MeJA responsive

TaMAPKKK41	TGACG-motif	TGACG	1877	5 -	Hordeum vulgare	the MeJA responsive
TaMAPKKK41	ABRE	CGTACGTGCA	1754	9 +	Hordeum vulgare	abscisic acid responsive
TaMAPKKK41	RY-element	CATGCATG	1124	8 -	Helianthus annuus	seed specific regulation
TaMAPKKK41	GC-motif	CCCCCG	111	6 +	Zea mays	anoxic specific inducibility
TaMAPKKK41	TGA-element	AACGAC	1323	6 -	Brassica oleracea	auxin responsive
TaMAPKKK41	TCA-element	CCATCTTTTT	959	9 -	Nicotiana tabacum	salicylic acid responsive
TaMAPKKK4-1	ARE	AAACCA	120	6 +	Zea mays	anaerobic induction
TaMAPKKK4-1	ARE	AAACCA	619	6 -	Zea mays	anaerobic induction
TaMAPKKK4-1	ARE	AAACCA	1422	6 +	Zea mays	anaerobic induction
TaMAPKKK4-1	LTR	CCGAAA	392	6 -	Hordeum vulgare	low temperature responsive
TaMAPKKK4-1	LTR	CCGAAA	734	6 +	Hordeum vulgare	low temperature responsive
TaMAPKKK4-1	CGTCA-motif	CGTCA	243	5 -	Hordeum vulgare	the MeJA responsive
TaMAPKKK4-1	CGTCA-motif	CGTCA	296	5 -	Hordeum vulgare	the MeJA responsive
TaMAPKKK4-1	CGTCA-motif	CGTCA	604	5 +	Hordeum vulgare	the MeJA responsive
TaMAPKKK4-1	CGTCA-motif	CGTCA	840	5 +	Hordeum vulgare	the MeJA responsive
TaMAPKKK4-1	CGTCA-motif	CGTCA	1193	5 -	Hordeum vulgare	the MeJA responsive
TaMAPKKK4-1	CGTCA-motif	CGTCA	1652	5 +	Hordeum vulgare	the MeJA responsive
TaMAPKKK4-1	TGACG-motif	TGACG	243	5 +	Hordeum vulgare	the MeJA responsive
TaMAPKKK4-1	TGACG-motif	TGACG	296	5 +	Hordeum vulgare	the MeJA responsive

TaMAPKKK4-1	TGACG-motif	TGACG	604	5 -	Hordeum vulgare	the MeJA responsive
TaMAPKKK4-1	TGACG-motif	TGACG	840	5 -	Hordeum vulgare	the MeJA responsive
TaMAPKKK4-1	TGACG-motif	TGACG	1193	5 +	Hordeum vulgare	the MeJA responsive
TaMAPKKK4-1	TGACG-motif	TGACG	1652	5 -	Hordeum vulgare	the MeJA responsive
TaMAPKKK4-1	TGA-element	AACGAC	973	6 -	Brassica oleracea	auxin responsive
TaMAPKKK4-1	GC-motif	CCCCCG	358	6 -	Zea mays	anoxic specific inducibility
TaMAPKKK4-1	GC-motif	CCCCCG	1028	6 +	Zea mays	anoxic specific inducibility
TaMAPKKK4-1	GARE-motif	TCTGTTG	1853	7 +	Brassica oleracea	gibberellin responsive
TaMAPKKK4-1	MBS	CAACTG	565	6 -	Arabidopsis thaliana	drought inducibility
TaMAPKKK4-1	MBS	CAACTG	1711	6 -	Arabidopsis thaliana	drought inducibility
TaMAPKKK4-1	MBS	CAACTG	1910	6 +	Arabidopsis thaliana	drought inducibility
TaMAPKKK42	LTR	CCGAAA	857	6 -	Hordeum vulgare	low temperature responsive
TaMAPKKK42	GARE-motif	TCTGTTG	433	7 -	Brassica oleracea	gibberellin responsive
TaMAPKKK42	GARE-motif	TCTGTTG	966	7 +	Brassica oleracea	gibberellin responsive
TaMAPKKK42	GARE-motif	TCTGTTG	1761	7 -	Brassica oleracea	gibberellin responsive
TaMAPKKK42	AuxRR-core	GGTCCAT	372	7 +	Nicotiana tabacum	auxin responsive
TaMAPKKK42	AuxRR-core	GGTCCAT	689	7 +	Nicotiana tabacum	auxin responsive
TaMAPKKK42	MBS	CAACTG	460	6 +	Arabidopsis thaliana	drought inducibility
TaMAPKKK42	O2-site	GATGACATGG	1268	9 +	Zea mays	zein metabolism regulation

TaMAPKKK42	ABRE	GCAACGTGTC	600	9 -	Hordeum vulgare	abscisic acid responsive
TaMAPKKK42	ABRE	ACGTG	1377	5 +	Arabidopsis thaliana	abscisic acid responsive
TaMAPKKK42	TGA-element	AACGAC	470	6 +	Brassica oleracea	auxin responsive
TaMAPKKK42	ARE	AAACCA	1188	6 +	Zea mays	anaerobic induction
TaMAPKKK42	ARE	AAACCA	1981	6 -	Zea mays	anaerobic induction
TaMAPKKK42	CGTCA-motif	CGTCA	70	5 -	Hordeum vulgare	the MeJA responsive
TaMAPKKK42	CGTCA-motif	CGTCA	222	5 -	Hordeum vulgare	the MeJA responsive
TaMAPKKK42	CGTCA-motif	CGTCA	356	5 -	Hordeum vulgare	the MeJA responsive
TaMAPKKK42	CGTCA-motif	CGTCA	1012	5 -	Hordeum vulgare	the MeJA responsive
TaMAPKKK42	CGTCA-motif	CGTCA	1018	5 -	Hordeum vulgare	the MeJA responsive
TaMAPKKK42	CGTCA-motif	CGTCA	1137	5 +	Hordeum vulgare	the MeJA responsive
TaMAPKKK42	CGTCA-motif	CGTCA	1528	5 +	Hordeum vulgare	the MeJA responsive
TaMAPKKK42	CGTCA-motif	CGTCA	1726	5 +	Hordeum vulgare	the MeJA responsive
TaMAPKKK42	TGACG-motif	TGACG	70	5 +	Hordeum vulgare	the MeJA responsive
TaMAPKKK42	TGACG-motif	TGACG	222	5 +	Hordeum vulgare	the MeJA responsive
TaMAPKKK42	TGACG-motif	TGACG	356	5 +	Hordeum vulgare	the MeJA responsive
TaMAPKKK42	TGACG-motif	TGACG	1012	5 +	Hordeum vulgare	the MeJA responsive
TaMAPKKK42	TGACG-motif	TGACG	1018	5 +	Hordeum vulgare	the MeJA responsive
TaMAPKKK42	TGACG-motif	TGACG	1137	5 -	Hordeum vulgare	the MeJA responsive

TaMAPKKK42	TGACG-motif	TGACG	1528	5 -	Hordeum vulgare	the MeJA responsive
TaMAPKKK42	TGACG-motif	TGACG	1726	5 -	Hordeum vulgare	the MeJA responsive
TaMAPKKK43	LTR	CCGAAA	829	6 +	Hordeum vulgare	low temperature responsive
TaMAPKKK43	LTR	CCGAAA	1353	6 -	Hordeum vulgare	low temperature responsive
TaMAPKKK43	LTR	CCGAAA	1932	6 -	Hordeum vulgare	low temperature responsive
TaMAPKKK43	MBS	CAACTG	1476	6 -	Arabidopsis thaliana	drought inducibility
TaMAPKKK43	O2-site	GATGATGTGG	890	9 +	Zea mays	zein metabolism regulation
TaMAPKKK43	GC-motif	CCCCCG	1489	6 -	Zea mays	anoxic specific inducibility
TaMAPKKK43	ABRE	CACGTG	1090	6 -	Arabidopsis thaliana	abscisic acid responsive
TaMAPKKK43	ABRE	ACGTG	1091	5 +	Arabidopsis thaliana	abscisic acid responsive
TaMAPKKK43	ABRE	AACCCGG	1270	7 +	Arabidopsis thaliana	abscisic acid responsive
TaMAPKKK43	CAT-box	GCCACT	1231	6 -	Arabidopsis thaliana	meristem expression
TaMAPKKK43	CAT-box	GCCACT	1463	6 -	Arabidopsis thaliana	meristem expression
TaMAPKKK43	TGA-element	AACGAC	1155	6 +	Brassica oleracea	auxin responsive
TaMAPKKK43	TGA-element	AACGAC	1385	6 +	Brassica oleracea	auxin responsive
TaMAPKKK43	MSA-like	(T/C)C(T/C)AAC GG(T/C)(T/C)A	872	8.5 -	Catharanthus roseus	cell cycle regulation
TaMAPKKK44	CAT-box	GCCACT	1858	6 +	Arabidopsis thaliana	meristem expression
TaMAPKKK44	ABRE	ACGTG	303	5 +	Arabidopsis thaliana	abscisic acid responsive
TaMAPKKK44	ABRE	ACGTG	840	5 +	Arabidopsis thaliana	abscisic acid responsive

TaMAPKKK44	ABRE	ACGTG	1299	5 -	Arabidopsis thaliana	abscisic acid responsive
TaMAPKKK44	ABRE	CGTACGTGCA	1652	9 -	Hordeum vulgare	abscisic acid responsive
TaMAPKKK44	TGA-element	AACGAC	276	6 -	Brassica oleracea	auxin responsive
TaMAPKKK44	TGA-element	AACGAC	1036	6 +	Brassica oleracea	auxin responsive
TaMAPKKK44	TGACG-motif	TGACG	736	5 +	Hordeum vulgare	the MeJA responsive
TaMAPKKK44	TGACG-motif	TGACG	1141	5 +	Hordeum vulgare	the MeJA responsive
TaMAPKKK44	TGACG-motif	TGACG	1814	5 +	Hordeum vulgare	the MeJA responsive
TaMAPKKK44	ARE	AAACCA	89	6 +	Zea mays	anaerobic induction
TaMAPKKK44	O2-site	GATGA(C/T)(A/G)TG(A/G)	1272	8 -	Zea mays	zein metabolism regulation
TaMAPKKK44	CGTCA-motif	CGTCA	736	5 -	Hordeum vulgare	the MeJA responsive
TaMAPKKK44	CGTCA-motif	CGTCA	1141	5 -	Hordeum vulgare	the MeJA responsive
TaMAPKKK44	CGTCA-motif	CGTCA	1814	5 -	Hordeum vulgare	the MeJA responsive
TaMAPKKK44	LTR	CCGAAA	1308	6 +	Hordeum vulgare	low temperature responsive
TaMAPKKK44	AuxRR-core	GGTCCAT	488	7 +	Nicotiana tabacum	auxin responsive
TaMAPKKK45	ARE	AAACCA	491	6 -	Zea mays	anaerobic induction
TaMAPKKK45	ARE	AAACCA	1431	6 -	Zea mays	anaerobic induction
TaMAPKKK45	ABRE	TACGTGTC	737	8 -	Oryza sativa	abscisic acid responsive
TaMAPKKK45	ABRE	ACGTG	739	5 -	Arabidopsis thaliana	abscisic acid responsive
TaMAPKKK45	ABRE	CACGTG	781	6 +	Arabidopsis thaliana	abscisic acid responsive
TaMAPKKK45	ABRE	ACGTG	782	5 +	Arabidopsis thaliana	abscisic acid responsive

TaMAPKKK45	ABRE	ACGTG	1229	5 -	Arabidopsis thaliana	abscisic acid responsive
TaMAPKKK45	ABRE	ACGTG	1306	5 -	Arabidopsis thaliana	abscisic acid responsive
TaMAPKKK45	ABRE	ACGTG	1326	5 -	Arabidopsis thaliana	abscisic acid responsive
TaMAPKKK45	ABRE	AACCCGG	1885	7 -	Arabidopsis thaliana	abscisic acid responsive
TaMAPKKK45	ABRE	ACGTG	1923	5 -	Arabidopsis thaliana	abscisic acid responsive
TaMAPKKK45	P-box	CCTTTTG	1456	7 +	Oryza sativa	gibberellin responsive
TaMAPKKK45	TGACG-motif	TGACG	839	5 -	Hordeum vulgare	the MeJA responsive
TaMAPKKK45	TGACG-motif	TGACG	1925	5 -	Hordeum vulgare	the MeJA responsive
TaMAPKKK45	CGTCA-motif	CGTCA	839	5 +	Hordeum vulgare	the MeJA responsive
TaMAPKKK45	CGTCA-motif	CGTCA	1925	5 +	Hordeum vulgare	the MeJA responsive
TaMAPKKK45	MBS	CAACTG	758	6 +	Arabidopsis thaliana	drought inducibility
TaMAPKKK45	MBS	CAACTG	1119	6 +	Arabidopsis thaliana	drought inducibility
TaMAPKKK45	GC-motif	CCCCCG	1161	6 +	Zea mays	anoxic specific inducibility
TaMAPKKK45	GC-motif	CCCCCG	1280	6 +	Zea mays	anoxic specific inducibility
TaMAPKKK46	TC-rich repeats	GTTTTCTTAC	295	9 +	Nicotiana tabacum	defense and stress responsive
TaMAPKKK46	MBS	CAACTG	1644	6 -	Arabidopsis thaliana	drought inducibility
TaMAPKKK46	CGTCA-motif	CGTCA	39	5 -	Hordeum vulgare	the MeJA responsive
TaMAPKKK46	TGA-element	AACGAC	427	6 +	Brassica oleracea	auxin responsive
TaMAPKKK46	TGA-element	AACGAC	497	6 -	Brassica oleracea	auxin responsive
TaMAPKKK46	CAT-box	GCCACT	1413	6 -	Arabidopsis thaliana	meristem expression

TaMAPKKK46	ABRE	ACGTG	41	5 +	Arabidopsis thaliana	abscisic acid responsive
TaMAPKKK46	ABRE	AACCCGG	369	7 -	Arabidopsis thaliana	abscisic acid responsive
TaMAPKKK46	ABRE	TACGTGTC	430	8 -	Oryza sativa	abscisic acid responsive
TaMAPKKK46	ABRE	ACGTG	432	5 -	Arabidopsis thaliana	abscisic acid responsive
TaMAPKKK46	ABRE	ACGTG	1274	5 +	Arabidopsis thaliana	abscisic acid responsive
TaMAPKKK46	ABRE	CGTACGTGCA	1746	9 -	Hordeum vulgare	abscisic acid responsive
TaMAPKKK46	ABRE	CACGTG	1830	6 -	Arabidopsis thaliana	abscisic acid responsive
TaMAPKKK46	ABRE	ACGTG	1831	5 +	Arabidopsis thaliana	abscisic acid responsive
TaMAPKKK46	ARE	AAACCA	568	6 -	Zea mays	anaerobic induction
TaMAPKKK46	circadian	CAAAGATATC	621	9 -	Lycopersicon esculentum	circadian control
TaMAPKKK46	TGACG-motif	TGACG	39	5 +	Hordeum vulgare	the MeJA responsive
TaMAPKKK47	TGA-element	AACGAC	500	6 -	Brassica oleracea	auxin responsive
TaMAPKKK47	ARE	AAACCA	755	6 -	Zea mays	anaerobic induction
TaMAPKKK47	ARE	AAACCA	1388	6 -	Zea mays	anaerobic induction
TaMAPKKK47	GC-motif	CCCCCG	48	6 +	Zea mays	anoxic specific inducibility
TaMAPKKK47	GC-motif	CCCCCG	1874	6 -	Zea mays	anoxic specific inducibility
TaMAPKKK47	CGTCA-motif	CGTCA	926	5 +	Hordeum vulgare	the MeJA responsive
TaMAPKKK47	CGTCA-motif	CGTCA	967	5 -	Hordeum vulgare	the MeJA responsive
TaMAPKKK47	TGACG-motif	TGACG	926	5 -	Hordeum vulgare	the MeJA responsive

TaMAPKKK47	TGACG-motif	TGACG	967	5 +	Hordeum vulgare	the MeJA responsive
TaMAPKKK47	MBS	CAACTG	1427	6 +	Arabidopsis thaliana	drought inducibility
TaMAPKKK47	MBS	CAACTG	1527	6 +	Arabidopsis thaliana	drought inducibility
TaMAPKKK47	CAT-box	GCCACT	766	6 +	Arabidopsis thaliana	meristem expression
TaMAPKKK48	ARE	AAACCA	450	6 +	Zea mays	anaerobic induction
TaMAPKKK48	ARE	AAACCA	517	6 +	Zea mays	anaerobic induction
TaMAPKKK48	ARE	AAACCA	635	6 +	Zea mays	anaerobic induction
TaMAPKKK48	ARE	AAACCA	786	6 +	Zea mays	anaerobic induction
TaMAPKKK48	ARE	AAACCA	1472	6 -	Zea mays	anaerobic induction
TaMAPKKK48	ARE	AAACCA	1915	6 +	Zea mays	anaerobic induction
TaMAPKKK48	ABRE	ACGTG	703	5 -	Arabidopsis thaliana	abscisic acid responsive
TaMAPKKK48	O2-site	GATGATGTGG	166	10 +	Zea mays	zein metabolism regulation
TaMAPKKK48	O2-site	GATGATGTGG	1370	9 -	Zea mays	zein metabolism regulation
TaMAPKKK48	TGACG-motif	TGACG	371	5 -	Hordeum vulgare	the MeJA responsive
TaMAPKKK48	TGACG-motif	TGACG	705	5 -	Hordeum vulgare	the MeJA responsive
TaMAPKKK48	CGTCA-motif	CGTCA	371	5 +	Hordeum vulgare	the MeJA responsive
TaMAPKKK48	CGTCA-motif	CGTCA	705	5 +	Hordeum vulgare	the MeJA responsive
TaMAPKKK48	P-box	CCTTTTG	1987	7 +	Oryza sativa	gibberellin responsive
TaMAPKKK48	LTR	CCGAAA	1587	6 -	Hordeum vulgare	low temperature responsive

TaMAPKKK48	TC-rich repeats	GTTTTCTTAC	1055	9 +	Nicotiana tabacum	defense and stress responsive
TaMAPKKK5	circadian	CAAAGATATC	382	9 -	Lycopersicon esculentum	circadian control
TaMAPKKK5	circadian	CAAAGATATC	572	9 -	Lycopersicon esculentum	circadian control
TaMAPKKK5	circadian	CAAAGATATC	1660	9 -	Lycopersicon esculentum	circadian control
TaMAPKKK5	O2-site	GATGATGTGG	935	9 -	Zea mays	zein metabolism regulation
TaMAPKKK5	O2-site	GATGA(C/T)(A/G)TG(A/G)	1088	8 +	Zea mays	zein metabolism regulation
TaMAPKKK5	O2-site	GATGACATGG	1093	9 +	Zea mays	zein metabolism regulation
TaMAPKKK5	O2-site	GATGA(C/T)(A/G)TG(A/G)	1710	8 -	Zea mays	zein metabolism regulation
TaMAPKKK5	TGACG-motif	TGACG	49	5 -	Hordeum vulgare	the MeJA responsive
TaMAPKKK5	TCA-element	CCATCTTTTT	531	9 +	Nicotiana tabacum	salicylic acid responsive
TaMAPKKK5	ARE	AAACCA	433	6 +	Zea mays	anaerobic induction
TaMAPKKK5	RY-element	CATGCATG	1178	8 -	Helianthus annuus	seed specific regulation
TaMAPKKK5	TGA-element	AACGAC	290	6 +	Brassica oleracea	auxin responsive
TaMAPKKK5	CGTCA-motif	CGTCA	49	5 +	Hordeum vulgare	the MeJA responsive
TaMAPKKK5	ABRE	CGTACGTGCA	30	9 -	Hordeum vulgare	abscisic acid responsive
TaMAPKKK5	ABRE	ACGTG	32	5 -	Arabidopsis thaliana	abscisic acid responsive
TaMAPKKK5	ABRE	ACGTG	359	5 -	Arabidopsis thaliana	abscisic acid responsive
TaMAPKKK5	GC-motif	CCCCCG	1824	6 +	Zea mays	anoxic specific inducibility
TaMAPKKK50	TCA-element	CCATCTTTTT	1889	9 -	Nicotiana tabacum	salicylic acid responsive

TaMAPKKK50	O2-site	GATGACATGG	919	9 -	Zea mays	zein metabolism regulation
TaMAPKKK50	O2-site	GATGACATGG	1564	9 +	Zea mays	zein metabolism regulation
TaMAPKKK50	GC-motif	CCCCCG	877	6 +	Zea mays	anoxic specific inducibility
TaMAPKKK50	ABRE	ACGTG	309	5 -	Arabidopsis thaliana	abscisic acid responsive
TaMAPKKK50	ABRE	ACGTG	1627	5 -	Arabidopsis thaliana	abscisic acid responsive
TaMAPKKK50	ARE	AAACCA	354	6 +	Zea mays	anaerobic induction
TaMAPKKK50	ARE	AAACCA	1896	6 -	Zea mays	anaerobic induction
TaMAPKKK50	CGTCA-motif	CGTCA	235	5 +	Hordeum vulgare	the MeJA responsive
TaMAPKKK50	CGTCA-motif	CGTCA	247	5 +	Hordeum vulgare	the MeJA responsive
TaMAPKKK50	CGTCA-motif	CGTCA	1348	5 +	Hordeum vulgare	the MeJA responsive
TaMAPKKK50	CGTCA-motif	CGTCA	1375	5 +	Hordeum vulgare	the MeJA responsive
TaMAPKKK50	TGACG-motif	TGACG	235	5 -	Hordeum vulgare	the MeJA responsive
TaMAPKKK50	TGACG-motif	TGACG	247	5 -	Hordeum vulgare	the MeJA responsive
TaMAPKKK50	TGACG-motif	TGACG	1348	5 -	Hordeum vulgare	the MeJA responsive
TaMAPKKK50	TGACG-motif	TGACG	1375	5 -	Hordeum vulgare	the MeJA responsive
TaMAPKKK50	LTR	CCGAAA	1521	6 -	Hordeum vulgare	low temperature responsive
TaMAPKKK50	GCN4_motif	TGAGTCA	1365	7 +	Oryza sativa	endosperm expression
TaMAPKKK50	AACA_motif	TAACAAACTC CA	1134	11 +	Oryza sativa	endosperm expression
TaMAPKKK50	MBS	CAACTG	1857	6 +	Arabidopsis thaliana	drought inducibility

TaMAPKKK51	ABRE	ACGTG	1875	5 -	Arabidopsis thaliana	abscisic acid responsive
TaMAPKKK51	ABRE	ACGTG	1911	5 -	Arabidopsis thaliana	abscisic acid responsive
TaMAPKKK51	ARE	AAACCA	269	6 +	Zea mays	anaerobic induction
TaMAPKKK51	ARE	AAACCA	1272	6 +	Zea mays	anaerobic induction
TaMAPKKK51	ARE	AAACCA	1620	6 -	Zea mays	anaerobic induction
TaMAPKKK51	TCA-element	CCATCTTTTT	272	9 +	Nicotiana tabacum	salicylic acid responsive
TaMAPKKK51	CGTCA-motif	CGTCA	668	5 +	Hordeum vulgare	the MeJA responsive
TaMAPKKK51	CGTCA-motif	CGTCA	1913	5 +	Hordeum vulgare	the MeJA responsive
TaMAPKKK51	CGTCA-motif	CGTCA	1923	5 +	Hordeum vulgare	the MeJA responsive
TaMAPKKK51	TGACG-motif	TGACG	668	5 -	Hordeum vulgare	the MeJA responsive
TaMAPKKK51	TGACG-motif	TGACG	1913	5 -	Hordeum vulgare	the MeJA responsive
TaMAPKKK51	TGACG-motif	TGACG	1923	5 -	Hordeum vulgare	the MeJA responsive
TaMAPKKK51	MBS	CAACTG	1789	6 +	Arabidopsis thaliana	drought inducibility
TaMAPKKK51	CAT-box	GCCACT	1486	6 -	Arabidopsis thaliana	meristem expression
TaMAPKKK52	MSA-like	(T/C)C(T/C)AAC GG(T/C)(T/C)A	17	8.5 -	Catharanthus roseus	cell cycle regulation
TaMAPKKK52	CGTCA-motif	CGTCA	1539	5 +	Hordeum vulgare	the MeJA responsive
TaMAPKKK52	CGTCA-motif	CGTCA	1674	5 -	Hordeum vulgare	the MeJA responsive
TaMAPKKK52	TGACG-motif	TGACG	1539	5 -	Hordeum vulgare	the MeJA responsive

TaMAPKKK52	TGACG-motif	TGACG	1674	5 +	Hordeum vulgare	the MeJA responsive
TaMAPKKK52	RY-element	CATGCATG	1698	8 -	Helianthus annuus	seed specific regulation
TaMAPKKK52	TGA-element	AACGAC	1878	6 -	Brassica oleracea	auxin responsive
TaMAPKKK52	ABRE	TACGGTC	915	7 +	Arabidopsis thaliana	abscisic acid responsive
TaMAPKKK52	ARE	AAACCA	179	6 +	Zea mays	anaerobic induction
TaMAPKKK52	ARE	AAACCA	253	6 +	Zea mays	anaerobic induction
TaMAPKKK52	ARE	AAACCA	1629	6 +	Zea mays	anaerobic induction
TaMAPKKK52	TCA-element	CCATCTTTTT	1801	9 -	Nicotiana tabacum	salicylic acid responsive
TaMAPKKK53	O2-site	GATGA(C/T)(A/G)TG(A/G)	1318	8 +	Zea mays	zein metabolism regulation
TaMAPKKK53	O2-site	GATGA(C/T)(A/G)TG(A/G)	1778	8 -	Zea mays	zein metabolism regulation
TaMAPKKK53	GCN4_motif	TGAGTCA	1479	7 +	Oryza sativa	endosperm expression
TaMAPKKK53	TGACG-motif	TGACG	479	5 +	Hordeum vulgare	the MeJA responsive
TaMAPKKK53	TGACG-motif	TGACG	971	5 +	Hordeum vulgare	the MeJA responsive
TaMAPKKK53	TGACG-motif	TGACG	1870	5 +	Hordeum vulgare	the MeJA responsive
TaMAPKKK53	ARE	AAACCA	890	6 -	Zea mays	anaerobic induction
TaMAPKKK53	RY-element	CATGCATG	1690	8 -	Helianthus annuus	seed specific regulation
TaMAPKKK53	TGA-box	TGACGTAA	1870	8 +	Glycine max	auxin responsive
TaMAPKKK53	CGTCA-motif	CGTCA	479	5 -	Hordeum vulgare	the MeJA responsive
TaMAPKKK53	CGTCA-motif	CGTCA	971	5 -	Hordeum vulgare	the MeJA responsive
TaMAPKKK53	CGTCA-motif	CGTCA	1870	5 -	Hordeum vulgare	the MeJA responsive

TaMAPKKK53	CAT-box	GCCACT	564	6 +	Arabidopsis thaliana	meristem expression
TaMAPKKK53	CAT-box	GCCACT	1339	6 -	Arabidopsis thaliana	meristem expression
TaMAPKKK53	CAT-box	GCCACT	1863	6 -	Arabidopsis thaliana	meristem expression
TaMAPKKK53	P-box	CCTTTTG	533	7 -	Oryza sativa	gibberellin responsive
TaMAPKKK53	ABRE	GACACGTGGC	545	9 +	Triticum aestivum	abscisic acid responsive
TaMAPKKK53	ABRE	CACGTG	547	6 +	Arabidopsis thaliana	abscisic acid responsive
TaMAPKKK53	ABRE	ACGTG	548	5 +	Arabidopsis thaliana	abscisic acid responsive
TaMAPKKK53	ABRE	ACGTG	1369	5 +	Arabidopsis thaliana	abscisic acid responsive
TaMAPKKK54	MBS	CAACTG	556	6 -	Arabidopsis thaliana	drought inducibility
TaMAPKKK54	MBS	CAACTG	1159	6 -	Arabidopsis thaliana	drought inducibility
TaMAPKKK54	CAT-box	GCCACT	822	6 -	Arabidopsis thaliana	meristem expression
TaMAPKKK54	CAT-box	GCCACT	971	6 +	Arabidopsis thaliana	meristem expression
TaMAPKKK54	CAT-box	GCCACT	1539	6 +	Arabidopsis thaliana	meristem expression
TaMAPKKK54	GARE-motif	TCTGTTG	647	7 +	Brassica oleracea	gibberellin responsive
TaMAPKKK54	TC-rich repeats	ATTCTCTAAC	1052	9 -	Nicotiana tabacum	defense and stress responsive
TaMAPKKK54	TGACG-motif	TGACG	58	5 +	Hordeum vulgare	the MeJA responsive
TaMAPKKK54	TGACG-motif	TGACG	83	5 +	Hordeum vulgare	the MeJA responsive
TaMAPKKK54	CGTCA-motif	CGTCA	58	5 -	Hordeum vulgare	the MeJA responsive
TaMAPKKK54	CGTCA-motif	CGTCA	83	5 -	Hordeum vulgare	the MeJA responsive

TaMAPKKK54	TATC-box	TATCCCA	1819	7 +	Oryza sativa	gibberellin responsive
TaMAPKKK54	TGA-element	AACGAC	118	6 +	Brassica oleracea	auxin responsive
TaMAPKKK54	ABRE	ACGTG	1108	5 -	Arabidopsis thaliana	abscisic acid responsive
TaMAPKKK54	ABRE	ACGTG	1498	5 +	Arabidopsis thaliana	abscisic acid responsive
TaMAPKKK54	O2-site	GATGACATGG	1703	9 -	Zea mays	zein metabolism regulation
TaMAPKKK56	ARE	AAACCA	1088	6 -	Zea mays	anaerobic induction
TaMAPKKK56	ARE	AAACCA	1356	6 +	Zea mays	anaerobic induction
TaMAPKKK56	TGACG-motif	TGACG	1446	5 +	Hordeum vulgare	the MeJA responsive
TaMAPKKK56	TGACG-motif	TGACG	1602	5 -	Hordeum vulgare	the MeJA responsive
TaMAPKKK56	TGACG-motif	TGACG	1926	5 -	Hordeum vulgare	the MeJA responsive
TaMAPKKK56	TGACG-motif	TGACG	1934	5 -	Hordeum vulgare	the MeJA responsive
TaMAPKKK56	TGACG-motif	TGACG	1961	5 -	Hordeum vulgare	the MeJA responsive
TaMAPKKK56	ABRE	ACGTG	698	5 +	Arabidopsis thaliana	abscisic acid responsive
TaMAPKKK56	CGTCA-motif	CGTCA	1446	5 -	Hordeum vulgare	the MeJA responsive
TaMAPKKK56	CGTCA-motif	CGTCA	1602	5 +	Hordeum vulgare	the MeJA responsive
TaMAPKKK56	CGTCA-motif	CGTCA	1926	5 +	Hordeum vulgare	the MeJA responsive
TaMAPKKK56	CGTCA-motif	CGTCA	1934	5 +	Hordeum vulgare	the MeJA responsive
TaMAPKKK56	CGTCA-motif	CGTCA	1961	5 +	Hordeum vulgare	the MeJA responsive
TaMAPKKK56	TC-rich repeats	GTTTTCTTAC	279	9 -	Nicotiana tabacum	defense and stress responsive

TaMAPKKK56	TC-rich repeats	GTTTTCTTAC	1304	9 -	Nicotiana tabacum	defense and stress responsive
TaMAPKKK56	O2-site	GATGATGTGG	787	10 -	Zea mays	zein metabolism regulation
TaMAPKKK56	GC-motif	CCCCCG	1682	6 +	Zea mays	anoxic specific inducibility
TaMAPKKK56	TCA-element	CCATCTTTTT	635	9 -	Nicotiana tabacum	salicylic acid responsive
TaMAPKKK56	P-box	CCTTTTG	503	7 -	Oryza sativa	gibberellin responsive
TaMAPKKK56	MBS	CAACTG	148	6 +	Arabidopsis thaliana	drought inducibility
TaMAPKKK56	MBS	CAACTG	967	6 +	Arabidopsis thaliana	drought inducibility
TaMAPKKK56	MBS	CAACTG	1758	6 +	Arabidopsis thaliana	drought inducibility
TaMAPKKK57	CAT-box	GCCACT	620	6 +	Arabidopsis thaliana	meristem expression
TaMAPKKK57	CAT-box	GCCACT	670	6 -	Arabidopsis thaliana	meristem expression
TaMAPKKK57	CAT-box	GCCACT	1883	6 -	Arabidopsis thaliana	meristem expression
TaMAPKKK57	MBS	CAACTG	1633	6 +	Arabidopsis thaliana	drought inducibility
TaMAPKKK57	O2-site	GATGACATGG	225	9 +	Zea mays	zein metabolism regulation
TaMAPKKK57	TGACG-motif	TGACG	35	5 +	Hordeum vulgare	the MeJA responsive
TaMAPKKK57	TGACG-motif	TGACG	597	5 -	Hordeum vulgare	the MeJA responsive
TaMAPKKK57	TGACG-motif	TGACG	612	5 -	Hordeum vulgare	the MeJA responsive
TaMAPKKK57	TGACG-motif	TGACG	629	5 +	Hordeum vulgare	the MeJA responsive
TaMAPKKK57	TGACG-motif	TGACG	1042	5 +	Hordeum vulgare	the MeJA responsive
TaMAPKKK57	TGACG-motif	TGACG	1045	5 -	Hordeum vulgare	the MeJA responsive

TaMAPKKK57	TC-rich repeats	ATTCTCTAAC	1262	9 +	Nicotiana tabacum	defense and stress responsive
TaMAPKKK57	ARE	AAACCA	701	6 +	Zea mays	anaerobic induction
TaMAPKKK57	ARE	AAACCA	928	6 -	Zea mays	anaerobic induction
TaMAPKKK57	ARE	AAACCA	963	6 -	Zea mays	anaerobic induction
TaMAPKKK57	ARE	AAACCA	1344	6 -	Zea mays	anaerobic induction
TaMAPKKK57	TCA-element	TCAGAAGAGG	55	9 -	Brassica oleracea	salicylic acid responsive
TaMAPKKK57	TCA-element	CCATCTTTTT	949	9 -	Nicotiana tabacum	salicylic acid responsive
TaMAPKKK57	ABRE	AACCCGG	433	7 -	Arabidopsis thaliana	abscisic acid responsive
TaMAPKKK57	ABRE	ACGTG	1309	5 -	Arabidopsis thaliana	abscisic acid responsive
TaMAPKKK57	ABRE	CGCACGTGTC	1688	9 +	Hordeum vulgare	abscisic acid responsive
TaMAPKKK57	ABRE	CACGTG	1690	6 -	Arabidopsis thaliana	abscisic acid responsive
TaMAPKKK57	ABRE	ACGTG	1691	5 +	Arabidopsis thaliana	abscisic acid responsive
TaMAPKKK57	ABRE	ACGTG	1929	5 -	Arabidopsis thaliana	abscisic acid responsive
TaMAPKKK57	TGA-element	AACGAC	836	6 +	Brassica oleracea	auxin responsive
TaMAPKKK57	CGTCA-motif	CGTCA	35	5 -	Hordeum vulgare	the MeJA responsive
TaMAPKKK57	CGTCA-motif	CGTCA	597	5 +	Hordeum vulgare	the MeJA responsive
TaMAPKKK57	CGTCA-motif	CGTCA	612	5 +	Hordeum vulgare	the MeJA responsive
TaMAPKKK57	CGTCA-motif	CGTCA	629	5 -	Hordeum vulgare	the MeJA responsive
TaMAPKKK57	CGTCA-motif	CGTCA	1042	5 -	Hordeum vulgare	the MeJA responsive

TaMAPKKK57	CGTCA-motif	CGTCA	1045	5 +	Hordeum vulgare	the MeJA responsive
TaMAPKKK58	TGACG-motif	TGACG	7	5 -	Hordeum vulgare	the MeJA responsive
TaMAPKKK58	TGACG-motif	TGACG	59	5 +	Hordeum vulgare	the MeJA responsive
TaMAPKKK58	TGACG-motif	TGACG	563	5 -	Hordeum vulgare	the MeJA responsive
TaMAPKKK58	TGACG-motif	TGACG	726	5 +	Hordeum vulgare	the MeJA responsive
TaMAPKKK58	TGACG-motif	TGACG	733	5 +	Hordeum vulgare	the MeJA responsive
TaMAPKKK58	TGACG-motif	TGACG	1276	5 +	Hordeum vulgare	the MeJA responsive
TaMAPKKK58	GARE-motif	TCTGTTG	695	7 -	Brassica oleracea	gibberellin responsive
TaMAPKKK58	ABRE	GACACGTGGC	114	9 -	Triticum aestivum	abscisic acid responsive
TaMAPKKK58	ABRE	ACGTG	509	5 -	Arabidopsis thaliana	abscisic acid responsive
TaMAPKKK58	ABRE	GCCGCGTGGC	1967	9 +	Oryza sativa	abscisic acid responsive
TaMAPKKK58	CAT-box	GCCACT	114	6 +	Arabidopsis thaliana	meristem expression
TaMAPKKK58	CAT-box	GCCACT	672	6 -	Arabidopsis thaliana	meristem expression
TaMAPKKK58	CAT-box	GCCACT	853	6 -	Arabidopsis thaliana	meristem expression
TaMAPKKK58	TGA-element	AACGAC	994	6 -	Brassica oleracea	auxin responsive
TaMAPKKK58	CGTCA-motif	CGTCA	7	5 +	Hordeum vulgare	the MeJA responsive
TaMAPKKK58	CGTCA-motif	CGTCA	59	5 -	Hordeum vulgare	the MeJA responsive
TaMAPKKK58	CGTCA-motif	CGTCA	563	5 +	Hordeum vulgare	the MeJA responsive
TaMAPKKK58	CGTCA-motif	CGTCA	726	5 -	Hordeum vulgare	the MeJA responsive

TaMAPKKK58	CGTCA-motif	CGTCA	733	5 -	Hordeum vulgare	the MeJA responsive
TaMAPKKK58	CGTCA-motif	CGTCA	1276	5 -	Hordeum vulgare	the MeJA responsive
TaMAPKKK58	AuxRR-core	GGTCCAT	410	7 +	Nicotiana tabacum	auxin responsive
TaMAPKKK58	P-box	CCTTTTG	353	7 +	Oryza sativa	gibberellin responsive
TaMAPKKK58	MBS	CAACTG	12	6 -	Arabidopsis thaliana	drought inducibility
TaMAPKKK58	MBS	CAACTG	481	6 +	Arabidopsis thaliana	drought inducibility
TaMAPKKK58	GCN4_motif	TGAGTCA	1491	7 +	Oryza sativa	endosperm expression
TaMAPKKK58	GC-motif	CCCCCG	1341	6 +	Zea mays	anoxic specific inducibility
TaMAPKKK58	GC-motif	CCCCCG	1424	6 -	Zea mays	anoxic specific inducibility
TaMAPKKK58	GC-motif	CCCCCG	1793	6 +	Zea mays	anoxic specific inducibility
TaMAPKKK58	O2-site	GTTGACGTGA	508	9 -	Zea mays	zein metabolism regulation
TaMAPKKK58	O2-site	GATGATGTGG	848	9 +	Zea mays	zein metabolism regulation
TaMAPKKK59	TGACG-motif	TGACG	321	5 +	Hordeum vulgare	the MeJA responsive
TaMAPKKK59	TGACG-motif	TGACG	359	5 -	Hordeum vulgare	the MeJA responsive
TaMAPKKK59	TGACG-motif	TGACG	603	5 +	Hordeum vulgare	the MeJA responsive
TaMAPKKK59	TGACG-motif	TGACG	896	5 -	Hordeum vulgare	the MeJA responsive
TaMAPKKK59	TGACG-motif	TGACG	1088	5 -	Hordeum vulgare	the MeJA responsive
TaMAPKKK59	TGACG-motif	TGACG	1174	5 -	Hordeum vulgare	the MeJA responsive
TaMAPKKK59	O2-site	GATGATGTGG	1944	9 -	Zea mays	zein metabolism regulation

TaMAPKKK59	TATC-box	TATCCCA	1761	7 +	Oryza sativa	gibberellin responsive
TaMAPKKK59	ABRE	TACGGTC	212	7 -	Arabidopsis thaliana	abscisic acid responsive
TaMAPKKK59	ABRE	ACGTG	250	5 +	Arabidopsis thaliana	abscisic acid responsive
TaMAPKKK59	ABRE	ACGTG	513	5 -	Arabidopsis thaliana	abscisic acid responsive
TaMAPKKK59	ABRE	ACGTG	532	5 +	Arabidopsis thaliana	abscisic acid responsive
TaMAPKKK59	ABRE	ACGTG	1076	5 -	Arabidopsis thaliana	abscisic acid responsive
TaMAPKKK59	TGA-element	AACGAC	858	6 -	Brassica oleracea	auxin responsive
TaMAPKKK59	CAT-box	GCCACT	40	6 -	Arabidopsis thaliana	meristem expression
TaMAPKKK59	CAT-box	GCCACT	82	6 -	Arabidopsis thaliana	meristem expression
TaMAPKKK59	CAT-box	GCCACT	877	6 +	Arabidopsis thaliana	meristem expression
TaMAPKKK59	CGTCA-motif	CGTCA	321	5 -	Hordeum vulgare	the MeJA responsive
TaMAPKKK59	CGTCA-motif	CGTCA	359	5 +	Hordeum vulgare	the MeJA responsive
TaMAPKKK59	CGTCA-motif	CGTCA	603	5 -	Hordeum vulgare	the MeJA responsive
TaMAPKKK59	CGTCA-motif	CGTCA	896	5 +	Hordeum vulgare	the MeJA responsive
TaMAPKKK59	CGTCA-motif	CGTCA	1088	5 +	Hordeum vulgare	the MeJA responsive
TaMAPKKK59	CGTCA-motif	CGTCA	1174	5 +	Hordeum vulgare	the MeJA responsive
TaMAPKKK59	MBS	CAACTG	1680	6 +	Arabidopsis thaliana	drought inducibility
TaMAPKKK59	RY-element	CATGCATG	475	8 +	Helianthus annuus	seed specific regulation
TaMAPKKK60	MBS	CAACTG	1597	6 +	Arabidopsis thaliana	drought inducibility

TaMAPKKK60	RY-element	CATGCATG	1704	8 -	Helianthus annuus	seed specific regulation
TaMAPKKK60	TC-rich repeats	ATTCTCTAAC	1425	9 +	Nicotiana tabacum	defense and stress responsive
TaMAPKKK60	P-box	CCTTTTG	220	7 +	Oryza sativa	gibberellin responsive
TaMAPKKK60	ABRE	GACACGTGGC	51	9 +	Triticum aestivum	abscisic acid responsive
TaMAPKKK60	ABRE	CACGTG	53	6 +	Arabidopsis thaliana	abscisic acid responsive
TaMAPKKK60	ABRE	ACGTG	54	5 +	Arabidopsis thaliana	abscisic acid responsive
TaMAPKKK60	ABRE	GACACGTGGC	755	9 +	Triticum aestivum	abscisic acid responsive
TaMAPKKK60	ABRE	ACGTG	1009	5 +	Arabidopsis thaliana	abscisic acid responsive
TaMAPKKK60	ABRE	ACGTG	1715	5 +	Arabidopsis thaliana	abscisic acid responsive
TaMAPKKK60	TGA-element	AACGAC	1432	6 +	Brassica oleracea	auxin responsive
TaMAPKKK60	CGTCA-motif	CGTCA	434	5 -	Hordeum vulgare	the MeJA responsive
TaMAPKKK60	CGTCA-motif	CGTCA	726	5 -	Hordeum vulgare	the MeJA responsive
TaMAPKKK60	CGTCA-motif	CGTCA	735	5 +	Hordeum vulgare	the MeJA responsive
TaMAPKKK60	CGTCA-motif	CGTCA	1245	5 -	Hordeum vulgare	the MeJA responsive
TaMAPKKK60	CGTCA-motif	CGTCA	1286	5 +	Hordeum vulgare	the MeJA responsive
TaMAPKKK60	CAT-box	GCCACT	237	6 -	Arabidopsis thaliana	meristem expression
TaMAPKKK60	O2-site	GATGACATGG	376	9 -	Zea mays	zein metabolism regulation
TaMAPKKK60	O2-site	GATGACATGG	1694	9 -	Zea mays	zein metabolism regulation
TaMAPKKK60	LTR	CCGAAA	121	6 -	Hordeum vulgare	low temperature responsive

TaMAPKKK60	LTR	CCGAAA	947	6 +	Hordeum vulgare	low temperature responsive
TaMAPKKK60	LTR	CCGAAA	1442	6 -	Hordeum vulgare	low temperature responsive
TaMAPKKK60	LTR	CCGAAA	1655	6 +	Hordeum vulgare	low temperature responsive
TaMAPKKK60	circadian	CAAAGATATC	766	9 -	Lycopersicon esculentum	circadian control
TaMAPKKK60	ARE	AAACCA	538	6 +	Zea mays	anaerobic induction
TaMAPKKK60	ARE	AAACCA	548	6 +	Zea mays	anaerobic induction
TaMAPKKK60	TCA-element	CCATCTTTTT	1526	9 -	Nicotiana tabacum	salicylic acid responsive
TaMAPKKK60	TGACG-motif	TGACG	434	5 +	Hordeum vulgare	the MeJA responsive
TaMAPKKK60	TGACG-motif	TGACG	726	5 +	Hordeum vulgare	the MeJA responsive
TaMAPKKK60	TGACG-motif	TGACG	735	5 -	Hordeum vulgare	the MeJA responsive
TaMAPKKK60	TGACG-motif	TGACG	1245	5 +	Hordeum vulgare	the MeJA responsive
TaMAPKKK60	TGACG-motif	TGACG	1286	5 -	Hordeum vulgare	the MeJA responsive
TaMAPKKK61	LTR	CCGAAA	660	6 +	Hordeum vulgare	low temperature responsive
TaMAPKKK61	MBS	CAACTG	1115	6 +	Arabidopsis thaliana	drought inducibility
TaMAPKKK61	MBS	CAACTG	1456	6 +	Arabidopsis thaliana	drought inducibility
TaMAPKKK61	CAT-box	GCCACT	602	6 +	Arabidopsis thaliana	meristem expression
TaMAPKKK61	TGA-element	AACGAC	614	6 +	Brassica oleracea	auxin responsive
TaMAPKKK61	O2-site	GTTGACGTGA	1109	9 -	Zea mays	zein metabolism regulation
TaMAPKKK61	CGTCA-motif	CGTCA	1112	5 +	Hordeum vulgare	the MeJA responsive

TaMAPKKK61	CGTCA-motif	CGTCA	1137	5 -	Hordeum vulgare	the MeJA responsive
TaMAPKKK61	TGACG-motif	TGACG	1112	5 -	Hordeum vulgare	the MeJA responsive
TaMAPKKK61	TGACG-motif	TGACG	1137	5 +	Hordeum vulgare	the MeJA responsive
TaMAPKKK61	RY-element	CATGCATG	419	8 +	Helianthus annuus	seed specific regulation
TaMAPKKK61	RY-element	CATGCATG	1416	8 -	Helianthus annuus	seed specific regulation
TaMAPKKK61	P-box	CCTTTTG	695	7 +	Oryza sativa	gibberellin responsive
TaMAPKKK62	ABRE	ACGTG	56	5 -	Arabidopsis thaliana	abscisic acid responsive
TaMAPKKK62	ABRE	ACGTG	85	5 +	Arabidopsis thaliana	abscisic acid responsive
TaMAPKKK62	ABRE	ACGTG	431	5 +	Arabidopsis thaliana	abscisic acid responsive
TaMAPKKK62	ABRE	ACGTG	643	5 -	Arabidopsis thaliana	abscisic acid responsive
TaMAPKKK62	ABRE	CACGTG	801	6 +	Arabidopsis thaliana	abscisic acid responsive
TaMAPKKK62	ABRE	ACGTG	802	5 +	Arabidopsis thaliana	abscisic acid responsive
TaMAPKKK62	ABRE	ACGTG	1576	5 +	Arabidopsis thaliana	abscisic acid responsive
TaMAPKKK62	ABRE	ACGTG	1740	5 -	Arabidopsis thaliana	abscisic acid responsive
TaMAPKKK62	TC-rich repeats	GTTTTCTTAC	443	9 -	Nicotiana tabacum	defense and stress responsive
TaMAPKKK62	MBS	CAACTG	834	6 +	Arabidopsis thaliana	drought inducibility
TaMAPKKK62	CGTCA-motif	CGTCA	83	5 -	Hordeum vulgare	the MeJA responsive
TaMAPKKK62	CGTCA-motif	CGTCA	1554	5 -	Hordeum vulgare	the MeJA responsive
TaMAPKKK62	TGACG-motif	TGACG	83	5 +	Hordeum vulgare	the MeJA responsive

TaMAPKKK62	TGACG-motif	TGACG	1554	5 +	Hordeum vulgare	the MeJA responsive
TaMAPKKK62	O2-site	GATGATGTGG	602	9 +	Zea mays	zein metabolism regulation
TaMAPKKK62	O2-site	GATGA(C/T)(A/G)TG(A/G)	1970	8 -	Zea mays	zein metabolism regulation
TaMAPKKK62	GC-motif	CCCCCG	465	6 -	Zea mays	anoxic specific inducibility
TaMAPKKK62	GC-motif	CCCCCG	1152	6 +	Zea mays	anoxic specific inducibility
TaMAPKKK62	GC-motif	CCCCCG	1484	6 +	Zea mays	anoxic specific inducibility
TaMAPKKK62	GC-motif	CCCCCG	1681	6 -	Zea mays	anoxic specific inducibility
TaMAPKKK62	CAT-box	GCCACT	1729	6 -	Arabidopsis thaliana	meristem expression
TaMAPKKK62	CAT-box	GCCACT	1783	6 -	Arabidopsis thaliana	meristem expression
TaMAPKKK62	P-box	CCTTTTG	1549	7 +	Oryza sativa	gibberellin responsive
TaMAPKKK63	ARE	AAACCA	1153	6 +	Zea mays	anaerobic induction
TaMAPKKK63	TGACG-motif	TGACG	70	5 -	Hordeum vulgare	the MeJA responsive
TaMAPKKK63	TGACG-motif	TGACG	1221	5 -	Hordeum vulgare	the MeJA responsive
TaMAPKKK63	TGACG-motif	TGACG	1337	5 -	Hordeum vulgare	the MeJA responsive
TaMAPKKK63	CAT-box	GCCACT	85	6 +	Arabidopsis thaliana	meristem expression
TaMAPKKK63	CAT-box	GCCACT	1352	6 +	Arabidopsis thaliana	meristem expression
TaMAPKKK63	MBSI	aaaAaaC(G/C)GTTA	499	10.5 +	Petunia hybrida	flavonoid biosynthetic genes regulation
TaMAPKKK63	AuxRR-core	GGTCCAT	669	7 +	Nicotiana tabacum	auxin responsive

TaMAPKKK63	CGTCA-motif	CGTCA	70	5 +	Hordeum vulgare	the MeJA responsive
TaMAPKKK63	CGTCA-motif	CGTCA	1221	5 +	Hordeum vulgare	the MeJA responsive
TaMAPKKK63	CGTCA-motif	CGTCA	1337	5 +	Hordeum vulgare	the MeJA responsive
TaMAPKKK63	LTR	CCGAAA	374	6 +	Hordeum vulgare	low temperature responsive
TaMAPKKK63	LTR	CCGAAA	1589	6 -	Hordeum vulgare	low temperature responsive
TaMAPKKK63	circadian	CAAAGATATC	976	9 +	Lycopersicon esculentum	circadian control
TaMAPKKK64	TCA-element	TCAGAAGAGG	415	9 -	Brassica oleracea	salicylic acid responsive
TaMAPKKK64	TCA-element	CCATCTTTTT	489	9 -	Nicotiana tabacum	salicylic acid responsive
TaMAPKKK64	TCA-element	CCATCTTTTT	725	9 -	Nicotiana tabacum	salicylic acid responsive
TaMAPKKK64	TCA-element	CCATCTTTTT	1624	9 +	Nicotiana tabacum	salicylic acid responsive
TaMAPKKK64	CGTCA-motif	CGTCA	422	5 -	Hordeum vulgare	the MeJA responsive
TaMAPKKK64	CGTCA-motif	CGTCA	430	5 +	Hordeum vulgare	the MeJA responsive
TaMAPKKK64	CGTCA-motif	CGTCA	1301	5 -	Hordeum vulgare	the MeJA responsive
TaMAPKKK64	ABRE	CGTACGTGCA	1929	9 -	Hordeum vulgare	abscisic acid responsive
TaMAPKKK64	TGACG-motif	TGACG	422	5 +	Hordeum vulgare	the MeJA responsive
TaMAPKKK64	TGACG-motif	TGACG	430	5 -	Hordeum vulgare	the MeJA responsive
TaMAPKKK64	TGACG-motif	TGACG	1301	5 +	Hordeum vulgare	the MeJA responsive
TaMAPKKK64	O2-site	GATGACATGG	158	9 -	Zea mays	zein metabolism regulation
TaMAPKKK64	O2-site	GATGACATGG	201	9 +	Zea mays	zein metabolism regulation

TaMAPKKK64	GARE-motif	TCTGTTG	136	7 -	Brassica oleracea	gibberellin responsive
TaMAPKKK64	MBS	CAACTG	19	6 -	Arabidopsis thaliana	drought inducibility
TaMAPKKK64	MBS	CAACTG	501	6 +	Arabidopsis thaliana	drought inducibility
TaMAPKKK64	MBS	CAACTG	532	6 -	Arabidopsis thaliana	drought inducibility
TaMAPKKK64	MBS	CAACTG	1892	6 +	Arabidopsis thaliana	drought inducibility
TaMAPKKK64	ARE	AAACCA	1067	6 -	Zea mays	anaerobic induction
TaMAPKKK64	ARE	AAACCA	1853	6 -	Zea mays	anaerobic induction
TaMAPKKK64-1	ABRE	TACGGTC	428	7 +	Arabidopsis thaliana	abscisic acid responsive
TaMAPKKK64-1	ABRE	CGTACGTGCA	1929	9 -	Hordeum vulgare	abscisic acid responsive
TaMAPKKK64-1	CGTCA-motif	CGTCA	157	5 -	Hordeum vulgare	the MeJA responsive
TaMAPKKK64-1	CGTCA-motif	CGTCA	1096	5 -	Hordeum vulgare	the MeJA responsive
TaMAPKKK64-1	CGTCA-motif	CGTCA	1394	5 +	Hordeum vulgare	the MeJA responsive
TaMAPKKK64-1	CGTCA-motif	CGTCA	1435	5 -	Hordeum vulgare	the MeJA responsive
TaMAPKKK64-1	LTR	CCGAAA	173	6 -	Hordeum vulgare	low temperature responsive
TaMAPKKK64-1	P-box	CCTTTTG	864	7 -	Oryza sativa	gibberellin responsive
TaMAPKKK64-1	P-box	CCTTTTG	1866	7 +	Oryza sativa	gibberellin responsive
TaMAPKKK64-1	CAT-box	GCCACT	193	6 -	Arabidopsis thaliana	meristem expression
TaMAPKKK64-1	ARE	AAACCA	580	6 -	Zea mays	anaerobic induction
TaMAPKKK64-1	ARE	AAACCA	1221	6 -	Zea mays	anaerobic induction

TaMAPKKK64-1	ARE	AAACCA	1532	6 -	Zea mays	anaerobic induction
TaMAPKKK64-1	ARE	AAACCA	1853	6 -	Zea mays	anaerobic induction
TaMAPKKK64-1	MBS	CAACTG	4	6 +	Arabidopsis thaliana	drought inducibility
TaMAPKKK64-1	MBS	CAACTG	35	6 -	Arabidopsis thaliana	drought inducibility
TaMAPKKK64-1	MBS	CAACTG	1892	6 +	Arabidopsis thaliana	drought inducibility
TaMAPKKK64-1	TGACG-motif	TGACG	157	5 +	Hordeum vulgare	the MeJA responsive
TaMAPKKK64-1	TGACG-motif	TGACG	1096	5 +	Hordeum vulgare	the MeJA responsive
TaMAPKKK64-1	TGACG-motif	TGACG	1394	5 -	Hordeum vulgare	the MeJA responsive
TaMAPKKK64-1	TGACG-motif	TGACG	1435	5 +	Hordeum vulgare	the MeJA responsive
TaMAPKKK64-1	O2-site	GTTGACGTGA	977	9 -	Zea mays	zein metabolism regulation
TaMAPKKK65	ABRE	ACGTG	219	5 -	Arabidopsis thaliana	abscisic acid responsive
TaMAPKKK65	ABRE	ACGTG	1234	5 -	Arabidopsis thaliana	abscisic acid responsive
TaMAPKKK65	WUN-motif	AAATTTCCT	5	9 +	Brassica oleracea	wound responsive
TaMAPKKK65	TGA-element	AACGAC	945	6 +	Brassica oleracea	auxin responsive
TaMAPKKK65	CGTCA-motif	CGTCA	1516	5 +	Hordeum vulgare	the MeJA responsive
TaMAPKKK65	CGTCA-motif	CGTCA	1667	5 -	Hordeum vulgare	the MeJA responsive
TaMAPKKK65	CGTCA-motif	CGTCA	1847	5 +	Hordeum vulgare	the MeJA responsive
TaMAPKKK65	LTR	CCGAAA	1865	6 +	Hordeum vulgare	low temperature responsive
TaMAPKKK65	ARE	AAACCA	235	6 -	Zea mays	anaerobic induction

TaMAPKKK65	ARE	AAACCA	312	6 -	Zea mays	anaerobic induction
TaMAPKKK65	ARE	AAACCA	1138	6 -	Zea mays	anaerobic induction
TaMAPKKK65	ARE	AAACCA	1823	6 -	Zea mays	anaerobic induction
TaMAPKKK65	GARE-motif	TCTGTTG	805	7 -	Brassica oleracea	gibberellin responsive
TaMAPKKK65	TGACG-motif	TGACG	1516	5 -	Hordeum vulgare	the MeJA responsive
TaMAPKKK65	TGACG-motif	TGACG	1667	5 +	Hordeum vulgare	the MeJA responsive
TaMAPKKK65	TGACG-motif	TGACG	1847	5 -	Hordeum vulgare	the MeJA responsive
TaMAPKKK65	O2-site	GATGACATGG	341	9 -	Zea mays	zein metabolism regulation
TaMAPKKK65	O2-site	GATGATGTGG	423	9 +	Zea mays	zein metabolism regulation
TaMAPKKK65	P-box	CCTTTTG	685	7 +	Oryza sativa	gibberellin responsive
TaMAPKKK65	CAT-box	GCCACT	798	6 +	Arabidopsis thaliana	meristem expression
TaMAPKKK65	CAT-box	GCCACT	1163	6 -	Arabidopsis thaliana	meristem expression
TaMAPKKK66	TGACG-motif	TGACG	313	5 +	Hordeum vulgare	the MeJA responsive
TaMAPKKK66	TGACG-motif	TGACG	766	5 +	Hordeum vulgare	the MeJA responsive
TaMAPKKK66	TGACG-motif	TGACG	1024	5 -	Hordeum vulgare	the MeJA responsive
TaMAPKKK66	TGACG-motif	TGACG	1697	5 +	Hordeum vulgare	the MeJA responsive
TaMAPKKK66	ARE	AAACCA	1896	6 -	Zea mays	anaerobic induction
TaMAPKKK66	circadian	CAAAGATATC	770	9 -	Lycopersicon esculentum	circadian control
TaMAPKKK66	circadian	CAAAGATATC	799	9 -	Lycopersicon esculentum	circadian control

TaMAPKKK66	circadian	CAAAGATATC	1015	9 +	Lycopersicon esculentum	circadian control
TaMAPKKK66	LTR	CCGAAA	110	6 +	Hordeum vulgare	low temperature responsive
TaMAPKKK66	LTR	CCGAAA	934	6 -	Hordeum vulgare	low temperature responsive
TaMAPKKK66	GCN4_motif	TGAGTCA	542	7 +	Oryza sativa	endosperm expression
TaMAPKKK66	CGTCA-motif	CGTCA	313	5 -	Hordeum vulgare	the MeJA responsive
TaMAPKKK66	CGTCA-motif	CGTCA	766	5 -	Hordeum vulgare	the MeJA responsive
TaMAPKKK66	CGTCA-motif	CGTCA	1024	5 +	Hordeum vulgare	the MeJA responsive
TaMAPKKK66	CGTCA-motif	CGTCA	1697	5 -	Hordeum vulgare	the MeJA responsive
TaMAPKKK66	MSA-like	(T/C)C(T/C)AAC GG(T/C)(T/C)A	345	8.5 -	Catharanthus roseus	cell cycle regulation
TaMAPKKK66	MSA-like	(T/C)C(T/C)AAC GG(T/C)(T/C)A	1420	9 -	Catharanthus roseus	cell cycle regulation
TaMAPKKK66	ABRE	GCAACGTGTC	257	9 -	Hordeum vulgare	abscisic acid responsive
TaMAPKKK66	ABRE	ACGTG	645	5 -	Arabidopsis thaliana	abscisic acid responsive
TaMAPKKK66	ABRE	CGTACGTGCA	1905	9 -	Hordeum vulgare	abscisic acid responsive
TaMAPKKK66	P-box	CCTTTTG	1824	7 +	Oryza sativa	gibberellin responsive
TaMAPKKK66	MBS	CAACTG	1527	6 +	Arabidopsis thaliana	drought inducibility
TaMAPKKK67	TGA-box	TGACGTAA	1868	8 +	Glycine max	auxin responsive
TaMAPKKK67	MBS	CAACTG	1452	6 +	Arabidopsis thaliana	drought inducibility
TaMAPKKK67	CGTCA-motif	CGTCA	1868	5 -	Hordeum vulgare	the MeJA responsive

TaMAPKKK67	CAT-box	GCCACT	1097	6 -	Arabidopsis thaliana	meristem expression
TaMAPKKK67	CAT-box	GCCACT	1590	6 -	Arabidopsis thaliana	meristem expression
TaMAPKKK67	CAT-box	GCCACT	1861	6 -	Arabidopsis thaliana	meristem expression
TaMAPKKK67	ABRE	GCAACGTGTC	619	9 -	Hordeum vulgare	abscisic acid responsive
TaMAPKKK67	ABRE	ACGTG	621	5 -	Arabidopsis thaliana	abscisic acid responsive
TaMAPKKK67	ABRE	ACGTG	1383	5 -	Arabidopsis thaliana	abscisic acid responsive
TaMAPKKK67	ABRE	ACGTG	1625	5 +	Arabidopsis thaliana	abscisic acid responsive
TaMAPKKK67	O2-site	GATGA(C/T)(A/G)TG(A/G)	1569	8 +	Zea mays	zein metabolism regulation
TaMAPKKK67	GCN4_motif	TGAGTCA	1749	7 +	Oryza sativa	endosperm expression
TaMAPKKK67	GARE-motif	TCTGTTG	78	7 +	Brassica oleracea	gibberellin responsive
TaMAPKKK67	LTR	CCGAAA	762	6 -	Hordeum vulgare	low temperature responsive
TaMAPKKK67	LTR	CCGAAA	1397	6 +	Hordeum vulgare	low temperature responsive
TaMAPKKK67	TGACG-motif	TGACG	1868	5 +	Hordeum vulgare	the MeJA responsive
TaMAPKKK67	ARE	AAACCA	543	6 -	Zea mays	anaerobic induction
TaMAPKKK67	ARE	AAACCA	927	6 +	Zea mays	anaerobic induction
TaMAPKKK67	ARE	AAACCA	1411	6 -	Zea mays	anaerobic induction
TaMAPKKK68	TGACG-motif	TGACG	85	5 -	Hordeum vulgare	the MeJA responsive
TaMAPKKK68	TGACG-motif	TGACG	337	5 -	Hordeum vulgare	the MeJA responsive
TaMAPKKK68	TGACG-motif	TGACG	728	5 -	Hordeum vulgare	the MeJA responsive

TaMAPKKK68	TGACG-motif	TGACG	1757	5 +	Hordeum vulgare	the MeJA responsive
TaMAPKKK68	LTR	CCGAAA	1158	6 -	Hordeum vulgare	low temperature responsive
TaMAPKKK68	AuxRE	TGTCTCAATAAG	1456	11 -	Glycine max	auxin responsive
TaMAPKKK68	TATC-box	TATCCCA	635	7 +	Oryza sativa	gibberellin responsive
TaMAPKKK68	ABRE	ACGTG	775	5 -	Arabidopsis thaliana	abscisic acid responsive
TaMAPKKK68	GC-motif	CCCCCG	1605	6 -	Zea mays	anoxic specific inducibility
TaMAPKKK68	CGTCA-motif	CGTCA	85	5 +	Hordeum vulgare	the MeJA responsive
TaMAPKKK68	CGTCA-motif	CGTCA	337	5 +	Hordeum vulgare	the MeJA responsive
TaMAPKKK68	CGTCA-motif	CGTCA	728	5 +	Hordeum vulgare	the MeJA responsive
TaMAPKKK68	CGTCA-motif	CGTCA	1757	5 -	Hordeum vulgare	the MeJA responsive
TaMAPKKK68	MBS	CAACTG	1669	6 +	Arabidopsis thaliana	drought inducibility
TaMAPKKK69	CGTCA-motif	CGTCA	328	5 +	Hordeum vulgare	the MeJA responsive
TaMAPKKK69	CGTCA-motif	CGTCA	722	5 -	Hordeum vulgare	the MeJA responsive
TaMAPKKK69	CGTCA-motif	CGTCA	1359	5 +	Hordeum vulgare	the MeJA responsive
TaMAPKKK69	CGTCA-motif	CGTCA	1883	5 +	Hordeum vulgare	the MeJA responsive
TaMAPKKK69	CGTCA-motif	CGTCA	1891	5 +	Hordeum vulgare	the MeJA responsive
TaMAPKKK69	GCN4_motif	TGAGTCA	271	7 +	Oryza sativa	endosperm expression
TaMAPKKK69	GC-motif	CCCCCG	1836	6 +	Zea mays	anoxic specific inducibility
TaMAPKKK69	GC-motif	CCCCCG	1980	6 +	Zea mays	anoxic specific inducibility

TaMAPKKK69	TGACG-motif	TGACG	328	5 -	Hordeum vulgare	the MeJA responsive
TaMAPKKK69	TGACG-motif	TGACG	722	5 +	Hordeum vulgare	the MeJA responsive
TaMAPKKK69	TGACG-motif	TGACG	1359	5 -	Hordeum vulgare	the MeJA responsive
TaMAPKKK69	TGACG-motif	TGACG	1883	5 -	Hordeum vulgare	the MeJA responsive
TaMAPKKK69	TGACG-motif	TGACG	1891	5 -	Hordeum vulgare	the MeJA responsive
TaMAPKKK69	ABRE	ACGTG	310	5 -	Arabidopsis thaliana	abscisic acid responsive
TaMAPKKK69	ABRE	ACGTG	1021	5 +	Arabidopsis thaliana	abscisic acid responsive
TaMAPKKK69	ABRE	ACGTG	1136	5 +	Arabidopsis thaliana	abscisic acid responsive
TaMAPKKK69	ABRE	ACGTG	1304	5 +	Arabidopsis thaliana	abscisic acid responsive
TaMAPKKK69	ABRE	ACGTG	1314	5 -	Arabidopsis thaliana	abscisic acid responsive
TaMAPKKK69	ABRE	ACGTG	1331	5 +	Arabidopsis thaliana	abscisic acid responsive
TaMAPKKK69	ABRE	CGCACGTGTC	1603	9 -	Hordeum vulgare	abscisic acid responsive
TaMAPKKK69	ABRE	CACGTG	1605	6 -	Arabidopsis thaliana	abscisic acid responsive
TaMAPKKK69	ABRE	ACGTG	1606	5 +	Arabidopsis thaliana	abscisic acid responsive
TaMAPKKK7	NON-box	AGATCGACG	750	9 -	Arabidopsis thaliana	meristem specific activation
TaMAPKKK7	ARE	AAACCA	721	6 +	Zea mays	anaerobic induction
TaMAPKKK7	TGACG-motif	TGACG	288	5 +	Hordeum vulgare	the MeJA responsive
TaMAPKKK7	TGACG-motif	TGACG	631	5 -	Hordeum vulgare	the MeJA responsive
TaMAPKKK7	TGACG-motif	TGACG	1076	5 -	Hordeum vulgare	the MeJA responsive

TaMAPKKK7	TGACG-motif	TGACG	1156	5 -	Hordeum vulgare	the MeJA responsive
TaMAPKKK7	TGACG-motif	TGACG	1212	5 +	Hordeum vulgare	the MeJA responsive
TaMAPKKK7	TGACG-motif	TGACG	1577	5 +	Hordeum vulgare	the MeJA responsive
TaMAPKKK7	TGACG-motif	TGACG	1607	5 -	Hordeum vulgare	the MeJA responsive
TaMAPKKK7	TGA-element	AACGAC	117	6 +	Brassica oleracea	auxin responsive
TaMAPKKK7	CAT-box	GCCACT	4	6 +	Arabidopsis thaliana	meristem expression
TaMAPKKK7	CAT-box	GCCACT	1106	6 -	Arabidopsis thaliana	meristem expression
TaMAPKKK7	ABRE	ACGTG	26	5 -	Arabidopsis thaliana	abscisic acid responsive
TaMAPKKK7	ABRE	ACGTG	1201	5 +	Arabidopsis thaliana	abscisic acid responsive
TaMAPKKK7	CGTCA-motif	CGTCA	288	5 -	Hordeum vulgare	the MeJA responsive
TaMAPKKK7	CGTCA-motif	CGTCA	631	5 +	Hordeum vulgare	the MeJA responsive
TaMAPKKK7	CGTCA-motif	CGTCA	1076	5 +	Hordeum vulgare	the MeJA responsive
TaMAPKKK7	CGTCA-motif	CGTCA	1156	5 +	Hordeum vulgare	the MeJA responsive
TaMAPKKK7	CGTCA-motif	CGTCA	1212	5 -	Hordeum vulgare	the MeJA responsive
TaMAPKKK7	CGTCA-motif	CGTCA	1577	5 -	Hordeum vulgare	the MeJA responsive
TaMAPKKK7	CGTCA-motif	CGTCA	1607	5 +	Hordeum vulgare	the MeJA responsive
TaMAPKKK7	TCA-element	CCATCTTTTT	1390	9 -	Nicotiana tabacum	salicylic acid responsive
TaMAPKKK7	TCA-element	CCATCTTTTT	1681	9 -	Nicotiana tabacum	salicylic acid responsive
TaMAPKKK7	GC-motif	CCCCCG	337	6 +	Zea mays	anoxic specific inducibility

TaMAPKKK7	TC-rich repeats	ATTCTCTAAC	342	9 -	Nicotiana tabacum	defense and stress responsive
TaMAPKKK7	MBS	CAACTG	101	6 +	Arabidopsis thaliana	drought inducibility
TaMAPKKK70	ARE	AAACCA	1765	6 +	Zea mays	anaerobic induction
TaMAPKKK70	TGACG-motif	TGACG	1452	5 +	Hordeum vulgare	the MeJA responsive
TaMAPKKK70	TGACG-motif	TGACG	1455	5 -	Hordeum vulgare	the MeJA responsive
TaMAPKKK70	TGACG-motif	TGACG	1908	5 -	Hordeum vulgare	the MeJA responsive
TaMAPKKK70	LTR	CCGAAA	89	6 +	Hordeum vulgare	low temperature responsive
TaMAPKKK70	LTR	CCGAAA	354	6 -	Hordeum vulgare	low temperature responsive
TaMAPKKK70	LTR	CCGAAA	824	6 -	Hordeum vulgare	low temperature responsive
TaMAPKKK70	O2-site	GATGACATGG	4	9 +	Zea mays	zein metabolism regulation
TaMAPKKK70	O2-site	GATGATGTGG	937	9 +	Zea mays	zein metabolism regulation
TaMAPKKK70	ABRE	ACGTG	184	5 +	Arabidopsis thaliana	abscisic acid responsive
TaMAPKKK70	GC-motif	CCCCCG	1703	6 +	Zea mays	anoxic specific inducibility
TaMAPKKK70	CGTCA-motif	CGTCA	1452	5 -	Hordeum vulgare	the MeJA responsive
TaMAPKKK70	CGTCA-motif	CGTCA	1455	5 +	Hordeum vulgare	the MeJA responsive
TaMAPKKK70	CGTCA-motif	CGTCA	1908	5 +	Hordeum vulgare	the MeJA responsive
TaMAPKKK70	TGA-element	AACGAC	916	6 -	Brassica oleracea	auxin responsive
TaMAPKKK70	RY-element	CATGCATG	510	8 +	Helianthus annuus	seed specific regulation
TaMAPKKK71	TATC-box	TATCCCA	815	7 +	Oryza sativa	gibberellin responsive

TaMAPKKK71	TCA-element	TCAGAAGAGG	1054	9 -	Brassica oleracea	salicylic acid responsive
TaMAPKKK71	MBS	CAACTG	1751	6 -	Arabidopsis thaliana	drought inducibility
TaMAPKKK71	TGA-element	AACGAC	1331	6 -	Brassica oleracea	auxin responsive
TaMAPKKK71	CGTCA-motif	CGTCA	687	5 -	Hordeum vulgare	the MeJA responsive
TaMAPKKK71	CGTCA-motif	CGTCA	966	5 -	Hordeum vulgare	the MeJA responsive
TaMAPKKK71	CGTCA-motif	CGTCA	1488	5 +	Hordeum vulgare	the MeJA responsive
TaMAPKKK71	CGTCA-motif	CGTCA	1606	5 -	Hordeum vulgare	the MeJA responsive
TaMAPKKK71	TGACG-motif	TGACG	687	5 +	Hordeum vulgare	the MeJA responsive
TaMAPKKK71	TGACG-motif	TGACG	966	5 +	Hordeum vulgare	the MeJA responsive
TaMAPKKK71	TGACG-motif	TGACG	1488	5 -	Hordeum vulgare	the MeJA responsive
TaMAPKKK71	TGACG-motif	TGACG	1606	5 +	Hordeum vulgare	the MeJA responsive
TaMAPKKK71	O2-site	GATGACATGG	1111	9 +	Zea mays	zein metabolism regulation
TaMAPKKK71	MSA-like	(T/C)C(T/C)AAC GG(T/C)(T/C)A	664	8.5 -	Catharanthus roseus	cell cycle regulation
TaMAPKKK72	CGTCA-motif	CGTCA	881	5 +	Hordeum vulgare	the MeJA responsive
TaMAPKKK72	P-box	CCTTTTG	439	7 -	Oryza sativa	gibberellin responsive
TaMAPKKK72	GC-motif	CCCCCG	1830	6 +	Zea mays	anoxic specific inducibility
TaMAPKKK72	O2-site	GATGA(C/T)(A/G)TG(A/G)	451	8 -	Zea mays	zein metabolism regulation
TaMAPKKK72	TC-rich repeats	GTTTTCTTAC	136	9 -	Nicotiana tabacum	defense and stress responsive
TaMAPKKK72	TC-rich repeats	ATTCTCTAAC	1415	9 -	Nicotiana tabacum	defense and stress responsive

TaMAPKKK72	TGACG-motif	TGACG	881	5 -	Hordeum vulgare	the MeJA responsive
TaMAPKKK72	ARE	AAACCA	930	6 +	Zea mays	anaerobic induction
TaMAPKKK72	ARE	AAACCA	1573	6 -	Zea mays	anaerobic induction
TaMAPKKK72	CAT-box	GCCACT	741	6 -	Arabidopsis thaliana	meristem expression
TaMAPKKK72	ABRE	GCAACGTGTC	1296	9 -	Hordeum vulgare	abscisic acid responsive
TaMAPKKK72	ABRE	GCCGCGTGGC	1340	9 +	Oryza sativa	abscisic acid responsive
TaMAPKKK72	ABRE	AACCCGG	1800	7 -	Arabidopsis thaliana	abscisic acid responsive
TaMAPKKK73	TGACG-motif	TGACG	45	5 -	Hordeum vulgare	the MeJA responsive
TaMAPKKK73	TGACG-motif	TGACG	608	5 +	Hordeum vulgare	the MeJA responsive
TaMAPKKK73	TGACG-motif	TGACG	1570	5 +	Hordeum vulgare	the MeJA responsive
TaMAPKKK73	TGACG-motif	TGACG	1838	5 +	Hordeum vulgare	the MeJA responsive
TaMAPKKK73	circadian	CAAAGATATC	36	9 +	Lycopersicon esculentum	circadian control
TaMAPKKK73	ARE	AAACCA	216	6 +	Zea mays	anaerobic induction
TaMAPKKK73	CAT-box	GCCACT	1512	6 -	Arabidopsis thaliana	meristem expression
TaMAPKKK73	ABRE	CACGTG	1632	6 -	Arabidopsis thaliana	abscisic acid responsive
TaMAPKKK73	ABRE	ACGTG	1633	5 +	Arabidopsis thaliana	abscisic acid responsive
TaMAPKKK73	ABRE	GACACGTACGT	1981	10 +	Oryza sativa	abscisic acid responsive
TaMAPKKK73	ABRE	ACGTG	1983	5 -	Arabidopsis thaliana	abscisic acid responsive
TaMAPKKK73	TGA-element	AACGAC	1886	6 +	Brassica oleracea	auxin responsive

TaMAPKKK73	CGTCA-motif	CGTCA	45	5 +	Hordeum vulgare	the MeJA responsive
TaMAPKKK73	CGTCA-motif	CGTCA	608	5 -	Hordeum vulgare	the MeJA responsive
TaMAPKKK73	CGTCA-motif	CGTCA	1570	5 -	Hordeum vulgare	the MeJA responsive
TaMAPKKK73	CGTCA-motif	CGTCA	1838	5 -	Hordeum vulgare	the MeJA responsive
TaMAPKKK73	LTR	CCGAAA	1462	6 -	Hordeum vulgare	low temperature responsive
TaMAPKKK73	TCA-element	CCATCTTTTT	66	9 -	Nicotiana tabacum	salicylic acid responsive
TaMAPKKK74	MBS	CAACTG	1258	6 +	Arabidopsis thaliana	drought inducibility
TaMAPKKK74	GC-motif	CCCCCG	1743	6 +	Zea mays	anoxic specific inducibility
TaMAPKKK74	O2-site	GATGA(C/T)(A/G)TG(A/G)	1928	8 -	Zea mays	zein metabolism regulation
TaMAPKKK74	TCA-element	CCATCTTTTT	1431	9 +	Nicotiana tabacum	salicylic acid responsive
TaMAPKKK74	CGTCA-motif	CGTCA	390	5 +	Hordeum vulgare	the MeJA responsive
TaMAPKKK74	LTR	CCGAAA	1795	6 -	Hordeum vulgare	low temperature responsive
TaMAPKKK74	ABRE	ACGTG	924	5 +	Arabidopsis thaliana	abscisic acid responsive
TaMAPKKK74	TGA-element	AACGAC	1822	6 -	Brassica oleracea	auxin responsive
TaMAPKKK74	TGACG-motif	TGACG	390	5 -	Hordeum vulgare	the MeJA responsive
TaMAPKKK74	ARE	AAACCA	1298	6 +	Zea mays	anaerobic induction
TaMAPKKK74	ARE	AAACCA	1439	6 -	Zea mays	anaerobic induction
TaMAPKKK74	ARE	AAACCA	1466	6 +	Zea mays	anaerobic induction
TaMAPKKK75	ARE	AAACCA	38	6 -	Zea mays	anaerobic induction

TaMAPKKK75	ARE	AAACCA	1390	6 -	Zea mays	anaerobic induction
TaMAPKKK75	ARE	AAACCA	1407	6 -	Zea mays	anaerobic induction
TaMAPKKK75	ARE	AAACCA	1938	6 +	Zea mays	anaerobic induction
TaMAPKKK75	TGA-element	AACGAC	1153	6 -	Brassica oleracea	auxin responsive
TaMAPKKK75	CAT-box	GCCACT	318	6 +	Arabidopsis thaliana	meristem expression
TaMAPKKK75	ABRE	GACACGTGGC	344	9 -	Triticum aestivum	abscisic acid responsive
TaMAPKKK75	ABRE	CACGTG	346	6 +	Arabidopsis thaliana	abscisic acid responsive
TaMAPKKK75	ABRE	ACGTG	347	5 +	Arabidopsis thaliana	abscisic acid responsive
TaMAPKKK75	ABRE	ACGTG	504	5 +	Arabidopsis thaliana	abscisic acid responsive
TaMAPKKK75	ABRE	ACGTG	1084	5 -	Arabidopsis thaliana	abscisic acid responsive
TaMAPKKK75	ABRE	ACGTG	1302	5 +	Arabidopsis thaliana	abscisic acid responsive
TaMAPKKK75	ABRE	CGTACGTGCA	1376	9 +	Hordeum vulgare	abscisic acid responsive
TaMAPKKK75	ABRE	CGTACGTGCA	1650	9 -	Hordeum vulgare	abscisic acid responsive
TaMAPKKK75	ABRE	ACGTG	1652	5 -	Arabidopsis thaliana	abscisic acid responsive
TaMAPKKK75	ABRE	ACGTG	1766	5 +	Arabidopsis thaliana	abscisic acid responsive
TaMAPKKK75	P-box	CCTTTTG	758	7 +	Oryza sativa	gibberellin responsive
TaMAPKKK75	TGACG-motif	TGACG	343	5 -	Hordeum vulgare	the MeJA responsive
TaMAPKKK75	TGACG-motif	TGACG	1515	5 +	Hordeum vulgare	the MeJA responsive
TaMAPKKK75	CGTCA-motif	CGTCA	343	5 +	Hordeum vulgare	the MeJA responsive

TaMAPKKK75	CGTCA-motif	CGTCA	1515	5 -	Hordeum vulgare	the MeJA responsive
TaMAPKKK75	LTR	CCGAAA	524	6 -	Hordeum vulgare	low temperature responsive
TaMAPKKK75	LTR	CCGAAA	1871	6 -	Hordeum vulgare	low temperature responsive
TaMAPKKK75	O2-site	GATGACATGG	1098	9 -	Zea mays	zein metabolism regulation
TaMAPKKK75	MBS	CAACTG	887	6 -	Arabidopsis thaliana	drought inducibility
TaMAPKKK76	LTR	CCGAAA	995	6 +	Hordeum vulgare	low temperature responsive
TaMAPKKK76	LTR	CCGAAA	1248	6 +	Hordeum vulgare	low temperature responsive
TaMAPKKK76	LTR	CCGAAA	1444	6 +	Hordeum vulgare	low temperature responsive
TaMAPKKK76	CGTCA-motif	CGTCA	765	5 -	Hordeum vulgare	the MeJA responsive
TaMAPKKK76	CGTCA-motif	CGTCA	1139	5 -	Hordeum vulgare	the MeJA responsive
TaMAPKKK76	CGTCA-motif	CGTCA	1314	5 -	Hordeum vulgare	the MeJA responsive
TaMAPKKK76	CGTCA-motif	CGTCA	1403	5 -	Hordeum vulgare	the MeJA responsive
TaMAPKKK76	CGTCA-motif	CGTCA	1460	5 +	Hordeum vulgare	the MeJA responsive
TaMAPKKK76	CGTCA-motif	CGTCA	1896	5 -	Hordeum vulgare	the MeJA responsive
TaMAPKKK76	MBS	CAACTG	1366	6 +	Arabidopsis thaliana	drought inducibility
TaMAPKKK76	MBS	CAACTG	1901	6 +	Arabidopsis thaliana	drought inducibility
TaMAPKKK76	P-box	CCTTTTG	1166	7 -	Oryza sativa	gibberellin responsive
TaMAPKKK76	ARE	AAACCA	527	6 +	Zea mays	anaerobic induction
TaMAPKKK76	TGACG-motif	TGACG	765	5 +	Hordeum vulgare	the MeJA responsive

TaMAPKKK76	TGACG-motif	TGACG	1139	5 +	Hordeum vulgare	the MeJA responsive
TaMAPKKK76	TGACG-motif	TGACG	1314	5 +	Hordeum vulgare	the MeJA responsive
TaMAPKKK76	TGACG-motif	TGACG	1403	5 +	Hordeum vulgare	the MeJA responsive
TaMAPKKK76	TGACG-motif	TGACG	1460	5 -	Hordeum vulgare	the MeJA responsive
TaMAPKKK76	TGACG-motif	TGACG	1896	5 +	Hordeum vulgare	the MeJA responsive
TaMAPKKK76	TGA-element	AACGAC	1273	6 -	Brassica oleracea	auxin responsive
TaMAPKKK76	GARE-motif	TCTGTTG	172	7 +	Brassica oleracea	gibberellin responsive
TaMAPKKK76	GARE-motif	TCTGTTG	1591	7 +	Brassica oleracea	gibberellin responsive
TaMAPKKK76	ABRE	ACGTG	254	5 -	Arabidopsis thaliana	abscisic acid responsive
TaMAPKKK76	ABRE	ACGTG	568	5 +	Arabidopsis thaliana	abscisic acid responsive
TaMAPKKK76	ABRE	ACGTG	1728	5 +	Arabidopsis thaliana	abscisic acid responsive
TaMAPKKK76	ABRE	TACGTGTC	1906	8 -	Oryza sativa	abscisic acid responsive
TaMAPKKK76	ABRE	ACGTG	1908	5 -	Arabidopsis thaliana	abscisic acid responsive
TaMAPKKK78	CGTCA-motif	CGTCA	317	5 -	Hordeum vulgare	the MeJA responsive
TaMAPKKK78	CGTCA-motif	CGTCA	399	5 +	Hordeum vulgare	the MeJA responsive
TaMAPKKK78	CGTCA-motif	CGTCA	1770	5 +	Hordeum vulgare	the MeJA responsive
TaMAPKKK78	CGTCA-motif	CGTCA	1996	5 -	Hordeum vulgare	the MeJA responsive
TaMAPKKK78	LTR	CCGAAA	14	6 -	Hordeum vulgare	low temperature responsive
TaMAPKKK78	TATC-box	TATCCCA	50	7 +	Oryza sativa	gibberellin responsive
TaMAPKKK78	TGA-element	AACGAC	1593	6 -	Brassica oleracea	auxin responsive

TaMAPKKK78	CAT-box	GCCACT	1689	6 +	Arabidopsis thaliana	meristem expression
TaMAPKKK78	ABRE	AACCCGG	153	7 +	Arabidopsis thaliana	abscisic acid responsive
TaMAPKKK78	ABRE	GCCGCGTGGC	1426	9 +	Oryza sativa	abscisic acid responsive
TaMAPKKK78	ARE	AAACCA	1414	6 +	Zea mays	anaerobic induction
TaMAPKKK78	TGACG-motif	TGACG	317	5 +	Hordeum vulgare	the MeJA responsive
TaMAPKKK78	TGACG-motif	TGACG	399	5 -	Hordeum vulgare	the MeJA responsive
TaMAPKKK78	TGACG-motif	TGACG	1770	5 -	Hordeum vulgare	the MeJA responsive
TaMAPKKK78	TGACG-motif	TGACG	1996	5 +	Hordeum vulgare	the MeJA responsive
TaMAPKKK79	CGTCA-motif	CGTCA	1042	5 +	Hordeum vulgare	the MeJA responsive
TaMAPKKK79	TC-rich repeats	GTTTTCTTAC	1074	9 -	Nicotiana tabacum	defense and stress responsive
TaMAPKKK79	ARE	AAACCA	535	6 +	Zea mays	anaerobic induction
TaMAPKKK79	TGACG-motif	TGACG	1042	5 -	Hordeum vulgare	the MeJA responsive
TaMAPKKK79	TGA-element	AACGAC	861	6 -	Brassica oleracea	auxin responsive
TaMAPKKK79	ABRE	ACGTG	23	5 +	Arabidopsis thaliana	abscisic acid responsive
TaMAPKKK79	ABRE	ACGTG	1554	5 -	Arabidopsis thaliana	abscisic acid responsive
TaMAPKKK79	CAT-box	GCCACT	978	6 +	Arabidopsis thaliana	meristem expression
TaMAPKKK8	LTR	CCGAAA	15	6 -	Hordeum vulgare	low temperature responsive
TaMAPKKK8	LTR	CCGAAA	1562	6 +	Hordeum vulgare	low temperature responsive
TaMAPKKK8	CGTCA-motif	CGTCA	193	5 +	Hordeum vulgare	the MeJA responsive

TaMAPKKK8	CGTCA-motif	CGTCA	945	5 +	Hordeum vulgare	the MeJA responsive
TaMAPKKK8	CGTCA-motif	CGTCA	1769	5 -	Hordeum vulgare	the MeJA responsive
TaMAPKKK8	GC-motif	CCCCCG	63	6 +	Zea mays	anoxic specific inducibility
TaMAPKKK8	GC-motif	CCCCCG	1772	6 -	Zea mays	anoxic specific inducibility
TaMAPKKK8	MBS	CAACTG	41	6 +	Arabidopsis thaliana	drought inducibility
TaMAPKKK8	TGACG-motif	TGACG	193	5 -	Hordeum vulgare	the MeJA responsive
TaMAPKKK8	TGACG-motif	TGACG	945	5 -	Hordeum vulgare	the MeJA responsive
TaMAPKKK8	TGACG-motif	TGACG	1769	5 +	Hordeum vulgare	the MeJA responsive
TaMAPKKK8	TGA-element	AACGAC	986	6 +	Brassica oleracea	auxin responsive
TaMAPKKK8	CAT-box	GCCACT	474	6 -	Arabidopsis thaliana	meristem expression
TaMAPKKK8	ABRE	ACGTG	1575	5 +	Arabidopsis thaliana	abscisic acid responsive
TaMAPKKK8	ABRE	CGCACGTGTC	1822	9 +	Hordeum vulgare	abscisic acid responsive
TaMAPKKK8	ABRE	ACGTG	1824	5 -	Arabidopsis thaliana	abscisic acid responsive
TaMAPKKK8	ABRE	ACGTG	1917	5 -	Arabidopsis thaliana	abscisic acid responsive
TaMAPKKK80	TGACG-motif	TGACG	893	5 -	Hordeum vulgare	the MeJA responsive
TaMAPKKK80	TGACG-motif	TGACG	963	5 +	Hordeum vulgare	the MeJA responsive
TaMAPKKK80	GARE-motif	TCTGTTG	869	7 -	Brassica oleracea	gibberellin responsive
TaMAPKKK80	LTR	CCGAAA	1325	6 -	Hordeum vulgare	low temperature responsive
TaMAPKKK80	TATC-box	TATCCCA	98	7 -	Oryza sativa	gibberellin responsive

TaMAPKKK80	TATC-box	TATCCCA	795	7 -	Oryza sativa	gibberellin responsive
TaMAPKKK80	P-box	CCTTTTG	1060	7 -	Oryza sativa	gibberellin responsive
TaMAPKKK80	ABRE	ACGTG	582	5 +	Arabidopsis thaliana	abscisic acid responsive
TaMAPKKK80	ABRE	CACGTG	1025	6 -	Arabidopsis thaliana	abscisic acid responsive
TaMAPKKK80	ABRE	ACGTG	1026	5 +	Arabidopsis thaliana	abscisic acid responsive
TaMAPKKK80	ABRE	TACGGTC	1279	7 +	Arabidopsis thaliana	abscisic acid responsive
TaMAPKKK80	ABRE	ACGTG	1726	5 +	Arabidopsis thaliana	abscisic acid responsive
TaMAPKKK80	GC-motif	CCCCCG	1583	6 -	Zea mays	anoxic specific inducibility
TaMAPKKK80	TGA-element	AACGAC	1476	6 +	Brassica oleracea	auxin responsive
TaMAPKKK80	MSA-like	(T/C)C(T/C)AAC GG(T/C)(T/C)A	1154	8.5 -	Catharanthus roseus	cell cycle regulation
TaMAPKKK80	CAT-box	GCCACT	49	6 +	Arabidopsis thaliana	meristem expression
TaMAPKKK80	CGTCA-motif	CGTCA	893	5 +	Hordeum vulgare	the MeJA responsive
TaMAPKKK80	CGTCA-motif	CGTCA	963	5 -	Hordeum vulgare	the MeJA responsive
TaMAPKKK80	MBS	CAACTG	206	6 -	Arabidopsis thaliana	drought inducibility
TaMAPKKK81	ABRE	ACGTG	1202	5 -	Arabidopsis thaliana	abscisic acid responsive
TaMAPKKK81	WUN-motif	AAATTTCCT	1692	9 +	Brassica oleracea	wound responsive
TaMAPKKK81	CGTCA-motif	CGTCA	639	5 +	Hordeum vulgare	the MeJA responsive
TaMAPKKK81	CGTCA-motif	CGTCA	1840	5 +	Hordeum vulgare	the MeJA responsive
TaMAPKKK81	CGTCA-motif	CGTCA	1962	5 +	Hordeum vulgare	the MeJA responsive

TaMAPKKK81	TATC-box	TATCCCA	1049	7 -	Oryza sativa	gibberellin responsive
TaMAPKKK81	TATC-box	TATCCCA	1876	7 +	Oryza sativa	gibberellin responsive
TaMAPKKK81	TCA-element	CCATCTTTTT	281	10 -	Nicotiana tabacum	salicylic acid responsive
TaMAPKKK81	O2-site	GATGACATGG	1207	9 -	Zea mays	zein metabolism regulation
TaMAPKKK81	TGACG-motif	TGACG	639	5 -	Hordeum vulgare	the MeJA responsive
TaMAPKKK81	TGACG-motif	TGACG	1840	5 -	Hordeum vulgare	the MeJA responsive
TaMAPKKK81	TGACG-motif	TGACG	1962	5 -	Hordeum vulgare	the MeJA responsive
TaMAPKKK81	CAT-box	GCCACT	985	6 +	Arabidopsis thaliana	meristem expression
TaMAPKKK82	WUN-motif	AAATTTCCT	29	9 -	Brassica oleracea	wound responsive
TaMAPKKK82	MBS	CAACTG	789	6 +	Arabidopsis thaliana	drought inducibility
TaMAPKKK82	GARE-motif	TCTGTTG	1286	7 +	Brassica oleracea	gibberellin responsive
TaMAPKKK82	HD-Zip 1	CAAT(A/T)ATT G	1433	8 -	Arabidopsis thaliana	differentiation of the palisade mesophyll cells
TaMAPKKK82	TGACG-motif	TGACG	716	5 -	Hordeum vulgare	the MeJA responsive
TaMAPKKK82	TGACG-motif	TGACG	1211	5 +	Hordeum vulgare	the MeJA responsive
TaMAPKKK82	CGTCA-motif	CGTCA	716	5 +	Hordeum vulgare	the MeJA responsive
TaMAPKKK82	CGTCA-motif	CGTCA	1211	5 -	Hordeum vulgare	the MeJA responsive
TaMAPKKK82	TCA-element	CCATCTTTTT	1706	9 -	Nicotiana tabacum	salicylic acid responsive
TaMAPKKK82	TGA-element	AACGAC	184	6 -	Brassica oleracea	auxin responsive
TaMAPKKK82	ABRE	ACGTG	853	5 +	Arabidopsis thaliana	abscisic acid responsive

TaMAPKKK82	ARE	AAACCA	660	6 -	Zea mays	anaerobic induction
TaMAPKKK82	ARE	AAACCA	869	6 +	Zea mays	anaerobic induction
TaMAPKKK82	ARE	AAACCA	1793	6 +	Zea mays	anaerobic induction
TaMAPKKK83	CAT-box	GCCACT	14	6 -	Arabidopsis thaliana	meristem expression
TaMAPKKK83	CAT-box	GCCACT	269	6 -	Arabidopsis thaliana	meristem expression
TaMAPKKK83	TGACG-motif	TGACG	287	5 +	Hordeum vulgare	the MeJA responsive
TaMAPKKK83	TGACG-motif	TGACG	485	5 +	Hordeum vulgare	the MeJA responsive
TaMAPKKK83	TGACG-motif	TGACG	620	5 +	Hordeum vulgare	the MeJA responsive
TaMAPKKK83	TGACG-motif	TGACG	784	5 +	Hordeum vulgare	the MeJA responsive
TaMAPKKK83	TGACG-motif	TGACG	1383	5 -	Hordeum vulgare	the MeJA responsive
TaMAPKKK83	TGACG-motif	TGACG	1901	5 +	Hordeum vulgare	the MeJA responsive
TaMAPKKK83	MBS	CAACTG	886	6 +	Arabidopsis thaliana	drought inducibility
TaMAPKKK83	GARE-motif	TCTGTTG	64	7 -	Brassica oleracea	gibberellin responsive
TaMAPKKK83	ARE	AAACCA	926	6 -	Zea mays	anaerobic induction
TaMAPKKK83	ARE	AAACCA	1661	6 -	Zea mays	anaerobic induction
TaMAPKKK83	AuxRR-core	GGTCCAT	1035	7 +	Nicotiana tabacum	auxin responsive
TaMAPKKK83	CGTCA-motif	CGTCA	287	5 -	Hordeum vulgare	the MeJA responsive
TaMAPKKK83	CGTCA-motif	CGTCA	485	5 -	Hordeum vulgare	the MeJA responsive
TaMAPKKK83	CGTCA-motif	CGTCA	620	5 -	Hordeum vulgare	the MeJA responsive

TaMAPKKK83	CGTCA-motif	CGTCA	784	5 -	Hordeum vulgare	the MeJA responsive
TaMAPKKK83	CGTCA-motif	CGTCA	1383	5 +	Hordeum vulgare	the MeJA responsive
TaMAPKKK83	CGTCA-motif	CGTCA	1901	5 -	Hordeum vulgare	the MeJA responsive
TaMAPKKK84	GC-motif	CCCCCG	549	6 -	Zea mays	anoxic specific inducibility
TaMAPKKK84	TGACG-motif	TGACG	430	5 -	Hordeum vulgare	the MeJA responsive
TaMAPKKK84	TGACG-motif	TGACG	911	5 +	Hordeum vulgare	the MeJA responsive
TaMAPKKK84	TGACG-motif	TGACG	1754	5 +	Hordeum vulgare	the MeJA responsive
TaMAPKKK84	TATC-box	TATCCCA	127	7 -	Oryza sativa	gibberellin responsive
TaMAPKKK84	TATC-box	TATCCCA	574	7 +	Oryza sativa	gibberellin responsive
TaMAPKKK84	circadian	CAAAGATATC	1506	9 -	Lycopersicon esculentum	circadian control
TaMAPKKK84	TGA-element	AACGAC	1895	6 -	Brassica oleracea	auxin responsive
TaMAPKKK84	CGTCA-motif	CGTCA	430	5 +	Hordeum vulgare	the MeJA responsive
TaMAPKKK84	CGTCA-motif	CGTCA	911	5 -	Hordeum vulgare	the MeJA responsive
TaMAPKKK84	CGTCA-motif	CGTCA	1754	5 -	Hordeum vulgare	the MeJA responsive
TaMAPKKK84	LTR	CCGAAA	487	6 -	Hordeum vulgare	low temperature responsive
TaMAPKKK84	ABRE	ACGTG	990	5 +	Arabidopsis thaliana	abscisic acid responsive
TaMAPKKK84	ABRE	ACGTG	1776	5 +	Arabidopsis thaliana	abscisic acid responsive
TaMAPKKK85	MBS	CAACTG	1457	6 -	Arabidopsis thaliana	drought inducibility
TaMAPKKK85	TGACG-motif	TGACG	1338	5 +	Hordeum vulgare	the MeJA responsive

TaMAPKKK85	TGACG-motif	TGACG	1713	5 +	Hordeum vulgare	the MeJA responsive
TaMAPKKK85	TGACG-motif	TGACG	1808	5 -	Hordeum vulgare	the MeJA responsive
TaMAPKKK85	TC-rich repeats	ATTCTCTAAC	668	9 -	Nicotiana tabacum	defense and stress responsive
TaMAPKKK85	ARE	AAACCA	855	6 +	Zea mays	anaerobic induction
TaMAPKKK85	ARE	AAACCA	1131	6 -	Zea mays	anaerobic induction
TaMAPKKK85	ABRE	CGTACGTGCA	244	9 -	Hordeum vulgare	abscisic acid responsive
TaMAPKKK85	ABRE	ACGTG	246	5 -	Arabidopsis thaliana	abscisic acid responsive
TaMAPKKK85	ABRE	ACGTG	1340	5 +	Arabidopsis thaliana	abscisic acid responsive
TaMAPKKK85	TGA-element	AACGAC	70	6 -	Brassica oleracea	auxin responsive
TaMAPKKK85	CGTCA-motif	CGTCA	1338	5 -	Hordeum vulgare	the MeJA responsive
TaMAPKKK85	CGTCA-motif	CGTCA	1713	5 -	Hordeum vulgare	the MeJA responsive
TaMAPKKK85	CGTCA-motif	CGTCA	1808	5 +	Hordeum vulgare	the MeJA responsive
TaMAPKKK85	RY-element	CATGCATG	981	8 +	Helianthus annuus	seed specific regulation
TaMAPKKK86	TGA-element	AACGAC	314	6 -	Brassica oleracea	auxin responsive
TaMAPKKK86	MBS	CAACTG	372	6 +	Arabidopsis thaliana	drought inducibility
TaMAPKKK86	ARE	AAACCA	230	6 -	Zea mays	anaerobic induction
TaMAPKKK86	TGACG-motif	TGACG	344	5 +	Hordeum vulgare	the MeJA responsive
TaMAPKKK86	TGACG-motif	TGACG	1431	5 -	Hordeum vulgare	the MeJA responsive
TaMAPKKK86	TGACG-motif	TGACG	1747	5 -	Hordeum vulgare	the MeJA responsive
TaMAPKKK86	TGACG-motif	TGACG	1834	5 -	Hordeum vulgare	the MeJA responsive

TaMAPKKK86	LTR	CCGAAA	1737	6 -	Hordeum vulgare	low temperature responsive
TaMAPKKK86	ABRE	ACGTG	42	5 +	Arabidopsis thaliana	abscisic acid responsive
TaMAPKKK86	ABRE	TACGGTC	796	7 +	Arabidopsis thaliana	abscisic acid responsive
TaMAPKKK86	ABRE	ACGTG	1179	5 +	Arabidopsis thaliana	abscisic acid responsive
TaMAPKKK86	ABRE	TACGGTC	1637	7 +	Arabidopsis thaliana	abscisic acid responsive
TaMAPKKK86	GC-motif	CCCCCG	1513	6 +	Zea mays	anoxic specific inducibility
TaMAPKKK86	P-box	CCTTTTG	1789	7 +	Oryza sativa	gibberellin responsive
TaMAPKKK86	TCA-element	CCATCTTTTT	763	9 -	Nicotiana tabacum	salicylic acid responsive
TaMAPKKK86	O2-site	GATGA(C/T)(A/G)TG(A/G)	208	8 +	Zea mays	zein metabolism regulation
TaMAPKKK86	O2-site	GATGATGTGG	967	9 -	Zea mays	zein metabolism regulation
TaMAPKKK86	TC-rich repeats	GTTTTCTTAC	123	9 -	Nicotiana tabacum	defense and stress responsive
TaMAPKKK86	CGTCA-motif	CGTCA	344	5 -	Hordeum vulgare	the MeJA responsive
TaMAPKKK86	CGTCA-motif	CGTCA	1431	5 +	Hordeum vulgare	the MeJA responsive
TaMAPKKK86	CGTCA-motif	CGTCA	1747	5 +	Hordeum vulgare	the MeJA responsive
TaMAPKKK86	CGTCA-motif	CGTCA	1834	5 +	Hordeum vulgare	the MeJA responsive
TaMAPKKK88	CGTCA-motif	CGTCA	68	5 -	Hordeum vulgare	the MeJA responsive
TaMAPKKK88	CGTCA-motif	CGTCA	325	5 -	Hordeum vulgare	the MeJA responsive
TaMAPKKK88	CGTCA-motif	CGTCA	1918	5 +	Hordeum vulgare	the MeJA responsive
TaMAPKKK88	CAT-box	GCCACT	507	6 -	Arabidopsis thaliana	meristem expression

TaMAPKKK88	GC-motif	CCCCCG	623	6 -	Zea mays	anoxic specific inducibility
TaMAPKKK88	ABRE	ACGTG	1043	5 -	Arabidopsis thaliana	abscisic acid responsive
TaMAPKKK88	ABRE	ACGTG	1190	5 -	Arabidopsis thaliana	abscisic acid responsive
TaMAPKKK88	TGACG-motif	TGACG	68	5 +	Hordeum vulgare	the MeJA responsive
TaMAPKKK88	TGACG-motif	TGACG	325	5 +	Hordeum vulgare	the MeJA responsive
TaMAPKKK88	TGACG-motif	TGACG	1918	5 -	Hordeum vulgare	the MeJA responsive
TaMAPKKK88	ARE	AAACCA	1055	6 -	Zea mays	anaerobic induction
TaMAPKKK88	ARE	AAACCA	1818	6 +	Zea mays	anaerobic induction
TaMAPKKK88	LTR	CCGAAA	525	6 -	Hordeum vulgare	low temperature responsive
TaMAPKKK88	O2-site	GATGA(C/T)(A/G)TG(A/G)	819	8 -	Zea mays	zein metabolism regulation
TaMAPKKK88	O2-site	GATGACATGG	1029	9 +	Zea mays	zein metabolism regulation
TaMAPKKK88	O2-site	GATGACATGG	1048	9 +	Zea mays	zein metabolism regulation
TaMAPKKK9	ARE	AAACCA	566	6 +	Zea mays	anaerobic induction
TaMAPKKK9	TGACG-motif	TGACG	772	5 +	Hordeum vulgare	the MeJA responsive
TaMAPKKK9	TGACG-motif	TGACG	1095	5 +	Hordeum vulgare	the MeJA responsive
TaMAPKKK9	TGACG-motif	TGACG	1150	5 -	Hordeum vulgare	the MeJA responsive
TaMAPKKK9	TGACG-motif	TGACG	1449	5 -	Hordeum vulgare	the MeJA responsive
TaMAPKKK9	TGACG-motif	TGACG	1621	5 +	Hordeum vulgare	the MeJA responsive
TaMAPKKK9	TGA-element	AACGAC	1703	6 +	Brassica oleracea	auxin responsive

TaMAPKKK9	GARE-motif	TCTGTTG	871	7	-	Brassica oleracea	gibberellin responsive
TaMAPKKK9	ABRE	AACCCGG	240	7	-	Arabidopsis thaliana	abscisic acid responsive
TaMAPKKK9	ABRE	ACGTG	774	5	+	Arabidopsis thaliana	abscisic acid responsive
TaMAPKKK9	ABRE	GACACGTGGC	1034	9	-	Triticum aestivum	abscisic acid responsive
TaMAPKKK9	ABRE	CACGTG	1036	6	-	Arabidopsis thaliana	abscisic acid responsive
TaMAPKKK9	ABRE	ACGTG	1037	5	+	Arabidopsis thaliana	abscisic acid responsive
TaMAPKKK9	ABRE	ACGTG	1245	5	+	Arabidopsis thaliana	abscisic acid responsive
TaMAPKKK9	ABRE	ACGTG	1285	5	+	Arabidopsis thaliana	abscisic acid responsive
TaMAPKKK9	ABRE	ACGTG	1327	5	-	Arabidopsis thaliana	abscisic acid responsive
TaMAPKKK9	ABRE	ACGTG	1447	5	-	Arabidopsis thaliana	abscisic acid responsive
TaMAPKKK9	ABRE	CACGTG	1452	6	-	Arabidopsis thaliana	abscisic acid responsive
TaMAPKKK9	ABRE	ACGTG	1453	5	+	Arabidopsis thaliana	abscisic acid responsive
TaMAPKKK9	ABRE	CACGTG	1617	6	-	Arabidopsis thaliana	abscisic acid responsive
TaMAPKKK9	ABRE	ACGTG	1618	5	+	Arabidopsis thaliana	abscisic acid responsive
TaMAPKKK9	ABRE	ACGTG	1752	5	-	Arabidopsis thaliana	abscisic acid responsive
TaMAPKKK9	ABRE	CGCACGTGTC	1845	9	+	Hordeum vulgare	abscisic acid responsive
TaMAPKKK9	ABRE	CACGTG	1847	6	-	Arabidopsis thaliana	abscisic acid responsive
TaMAPKKK9	ABRE	ACGTG	1848	5	+	Arabidopsis thaliana	abscisic acid responsive
TaMAPKKK9	CAT-box	GCCACT	1894	6	+	Arabidopsis thaliana	meristem expression

TaMAPKKK9	CGTCA-motif	CGTCA	772	5 -	Hordeum vulgare	the MeJA responsive
TaMAPKKK9	CGTCA-motif	CGTCA	1095	5 -	Hordeum vulgare	the MeJA responsive
TaMAPKKK9	CGTCA-motif	CGTCA	1150	5 +	Hordeum vulgare	the MeJA responsive
TaMAPKKK9	CGTCA-motif	CGTCA	1449	5 +	Hordeum vulgare	the MeJA responsive
TaMAPKKK9	CGTCA-motif	CGTCA	1621	5 -	Hordeum vulgare	the MeJA responsive
TaMAPKKK9	LTR	CCGAAA	1196	6 +	Hordeum vulgare	low temperature responsive
TaMAPKKK9	GC-motif	CCCCCG	359	6 -	Zea mays	anoxic specific inducibility
TaMAPKKK9	GC-motif	CCCCCG	1193	6 +	Zea mays	anoxic specific inducibility
TaMAPKKK9	O2-site	GATGACATGG	616	10 +	Zea mays	zein metabolism regulation
TaMAPKKK9	O2-site	GATGA(C/T)(A/G)TG(A/G)	770	8 +	Zea mays	zein metabolism regulation
TaMAPKKK9	TCA-element	TCAGAAGAGG	1309	9 -	Brassica oleracea	salicylic acid responsive
TaMAPKKK9	P-box	CCTTTTG	960	7 -	Oryza sativa	gibberellin responsive
TaMAPKKK9	MBS	CAACTG	570	6 +	Arabidopsis thaliana	drought inducibility
TaMAPKKK90	CGTCA-motif	CGTCA	309	5 +	Hordeum vulgare	the MeJA responsive
TaMAPKKK90	CGTCA-motif	CGTCA	1228	5 +	Hordeum vulgare	the MeJA responsive
TaMAPKKK90	CGTCA-motif	CGTCA	1405	5 -	Hordeum vulgare	the MeJA responsive
TaMAPKKK90	O2-site	GATGATGTGG	910	9 -	Zea mays	zein metabolism regulation
TaMAPKKK90	TCA-element	TCAGAAGAGG	654	9 -	Brassica oleracea	salicylic acid responsive
TaMAPKKK90	ABRE	ACGTG	208	5 -	Arabidopsis thaliana	abscisic acid responsive

TaMAPKKK90	ABRE	ACGTG	251	5 -	Arabidopsis thaliana	abscisic acid responsive
TaMAPKKK90	ABRE	GCAACGTGTC	968	9 -	Hordeum vulgare	abscisic acid responsive
TaMAPKKK90	ABRE	ACGTG	970	5 -	Arabidopsis thaliana	abscisic acid responsive
TaMAPKKK90	ABRE	CGCACGTGTC	1009	10 -	Hordeum vulgare	abscisic acid responsive
TaMAPKKK90	ABRE	CACGTG	1011	6 -	Arabidopsis thaliana	abscisic acid responsive
TaMAPKKK90	ABRE	ACGTG	1012	5 +	Arabidopsis thaliana	abscisic acid responsive
TaMAPKKK90	ABRE	ACGTG	1430	5 +	Arabidopsis thaliana	abscisic acid responsive
TaMAPKKK90	ABRE	GCAACGTGTC	1589	9 -	Hordeum vulgare	abscisic acid responsive
TaMAPKKK90	ABRE	ACGTG	1591	5 -	Arabidopsis thaliana	abscisic acid responsive
TaMAPKKK90	ARE	AAACCA	1447	6 -	Zea mays	anaerobic induction
TaMAPKKK90	ARE	AAACCA	1534	6 -	Zea mays	anaerobic induction
TaMAPKKK90	ARE	AAACCA	1559	6 +	Zea mays	anaerobic induction
TaMAPKKK90	MBS	CAACTG	218	6 -	Arabidopsis thaliana	drought inducibility
TaMAPKKK90	MBS	CAACTG	517	6 +	Arabidopsis thaliana	drought inducibility
TaMAPKKK90	MBS	CAACTG	1627	6 +	Arabidopsis thaliana	drought inducibility
TaMAPKKK90	TGACG-motif	TGACG	309	5 -	Hordeum vulgare	the MeJA responsive
TaMAPKKK90	TGACG-motif	TGACG	1228	5 -	Hordeum vulgare	the MeJA responsive
TaMAPKKK90	TGACG-motif	TGACG	1405	5 +	Hordeum vulgare	the MeJA responsive
TaMAPKKK90	TGA-box	TGACGTAA	306	8 -	Glycine max	auxin responsive

TaMAPKKK90	CAT-box	GCCACT	1368	6 -	Arabidopsis thaliana	meristem expression
TaMAPKKK90	CAT-box	GCCACT	1667	6 -	Arabidopsis thaliana	meristem expression
TaMAPKKK91	ABRE	ACGTG	598	5 -	Arabidopsis thaliana	abscisic acid responsive
TaMAPKKK91	ABRE	ACGTG	921	5 +	Arabidopsis thaliana	abscisic acid responsive
TaMAPKKK91	ABRE	ACGTG	1366	5 -	Arabidopsis thaliana	abscisic acid responsive
TaMAPKKK91	ABRE	CACGTG	1385	6 -	Arabidopsis thaliana	abscisic acid responsive
TaMAPKKK91	ABRE	ACGTG	1386	5 +	Arabidopsis thaliana	abscisic acid responsive
TaMAPKKK91	CGTCA-motif	CGTCA	405	5 -	Hordeum vulgare	the MeJA responsive
TaMAPKKK91	CGTCA-motif	CGTCA	501	5 -	Hordeum vulgare	the MeJA responsive
TaMAPKKK91	CGTCA-motif	CGTCA	1205	5 -	Hordeum vulgare	the MeJA responsive
TaMAPKKK91	CGTCA-motif	CGTCA	1208	5 +	Hordeum vulgare	the MeJA responsive
TaMAPKKK91	TGA-element	AACGAC	212	6 +	Brassica oleracea	auxin responsive
TaMAPKKK91	TGA-element	AACGAC	1164	6 +	Brassica oleracea	auxin responsive
TaMAPKKK91	ARE	AAACCA	332	6 +	Zea mays	anaerobic induction
TaMAPKKK91	ARE	AAACCA	1112	6 -	Zea mays	anaerobic induction
TaMAPKKK91	ARE	AAACCA	1989	6 -	Zea mays	anaerobic induction
TaMAPKKK91	TGACG-motif	TGACG	405	5 +	Hordeum vulgare	the MeJA responsive
TaMAPKKK91	TGACG-motif	TGACG	501	5 +	Hordeum vulgare	the MeJA responsive
TaMAPKKK91	TGACG-motif	TGACG	1205	5 +	Hordeum vulgare	the MeJA responsive
TaMAPKKK91	TGACG-motif	TGACG	1208	5 -	Hordeum vulgare	the MeJA responsive

TaMAPKKK91	CAT-box	GCCACT	1231	6 +	Arabidopsis thaliana	meristem expression
TaMAPKKK92	TGACG-motif	TGACG	425	5 +	Hordeum vulgare	the MeJA responsive
TaMAPKKK92	TGACG-motif	TGACG	924	5 -	Hordeum vulgare	the MeJA responsive
TaMAPKKK92	CGTCA-motif	CGTCA	425	5 -	Hordeum vulgare	the MeJA responsive
TaMAPKKK92	CGTCA-motif	CGTCA	924	5 +	Hordeum vulgare	the MeJA responsive
TaMAPKKK92	AuxRR-core	GGTCCAT	837	7 +	Nicotiana tabacum	auxin responsive
TaMAPKKK92	LTR	CCGAAA	1044	6 -	Hordeum vulgare	low temperature responsive
TaMAPKKK92	LTR	CCGAAA	1962	6 +	Hordeum vulgare	low temperature responsive
TaMAPKKK92	ARE	AAACCA	1820	6 +	Zea mays	anaerobic induction
TaMAPKKK92	MSA-like	(T/C)C(T/C)AAC GG(T/C)(T/C)A	680	8.5 +	Catharanthus roseus	cell cycle regulation
TaMAPKKK92	MSA-like	(T/C)C(T/C)AAC GG(T/C)(T/C)A	895	8.5 +	Catharanthus roseus	cell cycle regulation
TaMAPKKK92	WUN-motif	AAATTTCCT	1218	9 +	Brassica oleracea	wound responsive
TaMAPKKK92	ABRE	ACGTG	107	5 -	Arabidopsis thaliana	abscisic acid responsive
TaMAPKKK92	ABRE	ACGTG	439	5 -	Arabidopsis thaliana	abscisic acid responsive
TaMAPKKK92	ABRE	CACGTG	644	6 +	Arabidopsis thaliana	abscisic acid responsive
TaMAPKKK92	ABRE	ACGTG	645	5 +	Arabidopsis thaliana	abscisic acid responsive
TaMAPKKK92	ABRE	ACGTG	888	5 +	Arabidopsis thaliana	abscisic acid responsive
TaMAPKKK92	ABRE	ACGTG	1101	5 +	Arabidopsis thaliana	abscisic acid responsive

TaMAPKKK92	ABRE	CGTACGTGCA	1804	9 -	Hordeum vulgare	abscisic acid responsive
TaMAPKKK92	ABRE	ACGTG	1810	5 -	Arabidopsis thaliana	abscisic acid responsive
TaMAPKKK92	GC-motif	CCCCCG	179	6 -	Zea mays	anoxic specific inducibility
TaMAPKKK93	CGTCA-motif	CGTCA	242	5 -	Hordeum vulgare	the MeJA responsive
TaMAPKKK93	CGTCA-motif	CGTCA	286	5 +	Hordeum vulgare	the MeJA responsive
TaMAPKKK93	CGTCA-motif	CGTCA	420	5 +	Hordeum vulgare	the MeJA responsive
TaMAPKKK93	TGACG-motif	TGACG	242	5 +	Hordeum vulgare	the MeJA responsive
TaMAPKKK93	TGACG-motif	TGACG	286	5 -	Hordeum vulgare	the MeJA responsive
TaMAPKKK93	TGACG-motif	TGACG	420	5 -	Hordeum vulgare	the MeJA responsive
TaMAPKKK93	P-box	CCTTTTG	476	7 +	Oryza sativa	gibberellin responsive
TaMAPKKK93	MBS	CAACTG	1331	6 -	Arabidopsis thaliana	drought inducibility
TaMAPKKK93	MBS	CAACTG	1466	6 -	Arabidopsis thaliana	drought inducibility
TaMAPKKK93	TGA-element	AACGAC	1151	6 +	Brassica oleracea	auxin responsive
TaMAPKKK93	ABRE	TACGGTC	351	7 -	Arabidopsis thaliana	abscisic acid responsive
TaMAPKKK93	ABRE	ACGTG	385	5 +	Arabidopsis thaliana	abscisic acid responsive
TaMAPKKK93	ABRE	CACGTG	1028	6 -	Arabidopsis thaliana	abscisic acid responsive
TaMAPKKK93	ABRE	ACGTG	1029	5 +	Arabidopsis thaliana	abscisic acid responsive
TaMAPKKK93	ABRE	ACGTG	1155	5 +	Arabidopsis thaliana	abscisic acid responsive
TaMAPKKK94	TATC-box	TATCCCA	314	7 -	Oryza sativa	gibberellin responsive

TaMAPKKK94	TCA-element	TCAGAAGAGG	1044	9 +	Brassica oleracea	salicylic acid responsive
TaMAPKKK94	ABRE	ACGTG	289	5 -	Arabidopsis thaliana	abscisic acid responsive
TaMAPKKK94	ABRE	TACGGTC	1890	7 +	Arabidopsis thaliana	abscisic acid responsive
TaMAPKKK94	TGA-element	AACGAC	1143	6 +	Brassica oleracea	auxin responsive
TaMAPKKK94	CGTCA-motif	CGTCA	524	5 +	Hordeum vulgare	the MeJA responsive
TaMAPKKK94	CGTCA-motif	CGTCA	553	5 +	Hordeum vulgare	the MeJA responsive
TaMAPKKK94	CGTCA-motif	CGTCA	617	5 -	Hordeum vulgare	the MeJA responsive
TaMAPKKK94	CAT-box	GCCACT	971	6 +	Arabidopsis thaliana	meristem expression
TaMAPKKK94	CAT-box	GCCACT	1586	6 -	Arabidopsis thaliana	meristem expression
TaMAPKKK94	GARE-motif	TCTGTTG	769	7 -	Brassica oleracea	gibberellin responsive
TaMAPKKK94	TGACG-motif	TGACG	524	5 -	Hordeum vulgare	the MeJA responsive
TaMAPKKK94	TGACG-motif	TGACG	553	5 -	Hordeum vulgare	the MeJA responsive
TaMAPKKK94	TGACG-motif	TGACG	617	5 +	Hordeum vulgare	the MeJA responsive
TaMAPKKK94	ARE	AAACCA	834	6 +	Zea mays	anaerobic induction
TaMAPKKK94	ARE	AAACCA	1716	6 -	Zea mays	anaerobic induction
TaMAPKKK94	ARE	AAACCA	1760	6 +	Zea mays	anaerobic induction
TaMAPKKK95	ABRE	CGCACGTGTC	163	9 -	Hordeum vulgare	abscisic acid responsive
TaMAPKKK95	ABRE	ACGTG	1200	5 +	Arabidopsis thaliana	abscisic acid responsive
TaMAPKKK95	ABRE	CGTACGTGCA	1305	9 -	Hordeum vulgare	abscisic acid responsive

TaMAPKKK95	ABRE	ACGTG	1612	5 -	Arabidopsis thaliana	abscisic acid responsive
TaMAPKKK95	ABRE	CGTACGTGCA	1624	9 -	Hordeum vulgare	abscisic acid responsive
TaMAPKKK95	ABRE	CGTACGTGCA	1693	9 +	Hordeum vulgare	abscisic acid responsive
TaMAPKKK95	ABRE	CGCACGTGTC	1851	9 -	Hordeum vulgare	abscisic acid responsive
TaMAPKKK95	TGA-element	AACGAC	390	6 +	Brassica oleracea	auxin responsive
TaMAPKKK95	CGTCA-motif	CGTCA	522	5 +	Hordeum vulgare	the MeJA responsive
TaMAPKKK95	CGTCA-motif	CGTCA	622	5 +	Hordeum vulgare	the MeJA responsive
TaMAPKKK95	CGTCA-motif	CGTCA	895	5 -	Hordeum vulgare	the MeJA responsive
TaMAPKKK95	CGTCA-motif	CGTCA	1341	5 -	Hordeum vulgare	the MeJA responsive
TaMAPKKK95	CAT-box	GCCACT	1299	6 +	Arabidopsis thaliana	meristem expression
TaMAPKKK95	GC-motif	CCCCCG	1452	6 -	Zea mays	anoxic specific inducibility
TaMAPKKK95	ARE	AAACCA	279	6 +	Zea mays	anaerobic induction
TaMAPKKK95	GARE-motif	TCTGTTG	59	7 -	Brassica oleracea	gibberellin responsive
TaMAPKKK95	GARE-motif	TCTGTTG	1597	7 +	Brassica oleracea	gibberellin responsive
TaMAPKKK95	TGACG-motif	TGACG	522	5 -	Hordeum vulgare	the MeJA responsive
TaMAPKKK95	TGACG-motif	TGACG	622	5 -	Hordeum vulgare	the MeJA responsive
TaMAPKKK95	TGACG-motif	TGACG	895	5 +	Hordeum vulgare	the MeJA responsive
TaMAPKKK95	TGACG-motif	TGACG	1341	5 +	Hordeum vulgare	the MeJA responsive
TaMAPKKK96	TC-rich repeats	GTTTTCTTAC	797	9 +	Nicotiana tabacum	defense and stress responsive

TaMAPKKK96	ARE	AAACCA	959	6 +	Zea mays	anaerobic induction
TaMAPKKK96	ARE	AAACCA	1109	6 +	Zea mays	anaerobic induction
TaMAPKKK96	ARE	AAACCA	1285	6 -	Zea mays	anaerobic induction
TaMAPKKK96	CAT-box	GCCACT	147	6 +	Arabidopsis thaliana	meristem expression
TaMAPKKK96	CAT-box	GCCACT	917	6 +	Arabidopsis thaliana	meristem expression
TaMAPKKK96	TATC-box	TATCCCA	1871	7 +	Oryza sativa	gibberellin responsive
TaMAPKKK96	circadian	CAAAGATATC	1977	9 -	Lycopersicon esculentum	circadian control
TaMAPKKK96	TGA-element	AACGAC	1572	6 +	Brassica oleracea	auxin responsive
TaMAPKKK96	ABRE	ACGTG	395	5 -	Arabidopsis thaliana	abscisic acid responsive
TaMAPKKK96	ABRE	AACCCGG	404	7 +	Arabidopsis thaliana	abscisic acid responsive
TaMAPKKK96	ABRE	ACGTG	794	5 -	Arabidopsis thaliana	abscisic acid responsive
TaMAPKKK97	AuxRR-core	GGTCCAT	730	7 -	Nicotiana tabacum	auxin responsive
TaMAPKKK97	CGTCA-motif	CGTCA	129	5 -	Hordeum vulgare	the MeJA responsive
TaMAPKKK97	CGTCA-motif	CGTCA	1461	5 -	Hordeum vulgare	the MeJA responsive
TaMAPKKK97	TGACG-motif	TGACG	129	5 +	Hordeum vulgare	the MeJA responsive
TaMAPKKK97	TGACG-motif	TGACG	1461	5 +	Hordeum vulgare	the MeJA responsive
TaMAPKKK97	CAT-box	GCCACT	101	6 -	Arabidopsis thaliana	meristem expression
TaMAPKKK97	ARE	AAACCA	835	6 +	Zea mays	anaerobic induction
TaMAPKKK97	ARE	AAACCA	1269	6 -	Zea mays	anaerobic induction

TaMAPKKK97	ARE	AAACCA	1927	6 +	Zea mays	anaerobic induction
TaMAPKKK97	TATC-box	TATCCCA	1165	7 -	Oryza sativa	gibberellin responsive
TaMAPKKK97	MBS	CAACTG	1946	6 +	Arabidopsis thaliana	drought inducibility
TaMAPKKK97	ABRE	ACGTG	64	5 +	Arabidopsis thaliana	abscisic acid responsive
TaMAPKKK98	GC-motif	CCCCCG	1455	6 -	Zea mays	anoxic specific inducibility
TaMAPKKK98	GC-motif	CCCCCG	1491	6 +	Zea mays	anoxic specific inducibility
TaMAPKKK98	GC-motif	CCCCCG	1585	6 +	Zea mays	anoxic specific inducibility
TaMAPKKK98	GC-motif	CCCCCG	1689	6 +	Zea mays	anoxic specific inducibility
TaMAPKKK98	TGA-element	AACGAC	946	6 -	Brassica oleracea	auxin responsive
TaMAPKKK98	ABRE	GCCGCGTGGC	156	9 -	Oryza sativa	abscisic acid responsive
TaMAPKKK98	ABRE	ACGTG	289	5 -	Arabidopsis thaliana	abscisic acid responsive
TaMAPKKK98	ABRE	ACGTG	305	5 -	Arabidopsis thaliana	abscisic acid responsive
TaMAPKKK98	ABRE	CACGTG	344	6 +	Arabidopsis thaliana	abscisic acid responsive
TaMAPKKK98	ABRE	ACGTG	345	5 +	Arabidopsis thaliana	abscisic acid responsive
TaMAPKKK98	ABRE	ACGTG	513	5 -	Arabidopsis thaliana	abscisic acid responsive
TaMAPKKK98	ABRE	ACGTG	541	5 -	Arabidopsis thaliana	abscisic acid responsive
TaMAPKKK98	ABRE	ACGTG	902	5 -	Arabidopsis thaliana	abscisic acid responsive
TaMAPKKK98	ABRE	ACGTG	1021	5 -	Arabidopsis thaliana	abscisic acid responsive
TaMAPKKK98	ABRE	GCCGCGTGGC	1701	9 -	Oryza sativa	abscisic acid responsive

TaMAPKKK98	LTR	CCGAAA	319	6 +	Hordeum vulgare	low temperature responsive
TaMAPKKK98	TGACG-motif	TGACG	448	5 -	Hordeum vulgare	the MeJA responsive
TaMAPKKK98	TGACG-motif	TGACG	482	5 -	Hordeum vulgare	the MeJA responsive
TaMAPKKK98	TGACG-motif	TGACG	876	5 -	Hordeum vulgare	the MeJA responsive
TaMAPKKK98	TGACG-motif	TGACG	904	5 -	Hordeum vulgare	the MeJA responsive
TaMAPKKK98	TGACG-motif	TGACG	1023	5 -	Hordeum vulgare	the MeJA responsive
TaMAPKKK98	CGTCA-motif	CGTCA	448	5 +	Hordeum vulgare	the MeJA responsive
TaMAPKKK98	CGTCA-motif	CGTCA	482	5 +	Hordeum vulgare	the MeJA responsive
TaMAPKKK98	CGTCA-motif	CGTCA	876	5 +	Hordeum vulgare	the MeJA responsive
TaMAPKKK98	CGTCA-motif	CGTCA	904	5 +	Hordeum vulgare	the MeJA responsive
TaMAPKKK98	CGTCA-motif	CGTCA	1023	5 +	Hordeum vulgare	the MeJA responsive
TaMAPKKK98	CAT-box	GCCACT	562	6 -	Arabidopsis thaliana	meristem expression
TaMAPKKK99	ABRE	ACGTG	261	5 +	Arabidopsis thaliana	abscisic acid responsive
TaMAPKKK99	ABRE	ACGTG	373	5 -	Arabidopsis thaliana	abscisic acid responsive
TaMAPKKK99	ABRE	ACGTG	1098	5 +	Arabidopsis thaliana	abscisic acid responsive
TaMAPKKK99	GARE-motif	TCTGTTG	998	7 -	Brassica oleracea	gibberellin responsive
TaMAPKKK99	TGACG-motif	TGACG	1105	5 -	Hordeum vulgare	the MeJA responsive
TaMAPKKK99	TGACG-motif	TGACG	1129	5 +	Hordeum vulgare	the MeJA responsive
TaMAPKKK99	ARE	AAACCA	431	6 -	Zea mays	anaerobic induction

TaMAPKKK99	ARE	AAACCA	1575	6 +	Zea mays	anaerobic induction
TaMAPKKK99	ARE	AAACCA	1658	6 +	Zea mays	anaerobic induction
TaMAPKKK99	MBS	CAACTG	418	6 +	Arabidopsis thaliana	drought inducibility
TaMAPKKK99	MBS	CAACTG	1213	6 -	Arabidopsis thaliana	drought inducibility
TaMAPKKK99	GC-motif	CCCCCG	117	6 +	Zea mays	anoxic specific inducibility
TaMAPKKK99	GC-motif	CCCCCG	1955	6 +	Zea mays	anoxic specific inducibility
TaMAPKKK99	CGTCA-motif	CGTCA	1105	5 +	Hordeum vulgare	the MeJA responsive
TaMAPKKK99	CGTCA-motif	CGTCA	1129	5 -	Hordeum vulgare	the MeJA responsive
TaMAPKKKK1	TC-rich repeats	ATTCTCTAAC	553	9 -	Nicotiana tabacum	defense and stress responsive
TaMAPKKKK1	TC-rich repeats	GTTTTCTTAC	1124	9 +	Nicotiana tabacum	defense and stress responsive
TaMAPKKKK1	CGTCA-motif	CGTCA	367	5 +	Hordeum vulgare	the MeJA responsive
TaMAPKKKK1	CGTCA-motif	CGTCA	417	5 -	Hordeum vulgare	the MeJA responsive
TaMAPKKKK1	CGTCA-motif	CGTCA	1395	5 +	Hordeum vulgare	the MeJA responsive
TaMAPKKKK1	CAT-box	GCCACT	1270	6 +	Arabidopsis thaliana	meristem expression
TaMAPKKKK1	CAT-box	GCCACT	1284	6 +	Arabidopsis thaliana	meristem expression
TaMAPKKKK1	CAT-box	GCCACT	1299	6 +	Arabidopsis thaliana	meristem expression
TaMAPKKKK1	GC-motif	CCCCCG	1325	6 -	Zea mays	anoxic specific inducibility
TaMAPKKKK1	GC-motif	CCCCCG	1380	6 +	Zea mays	anoxic specific inducibility
TaMAPKKKK1	ABRE	ACGTG	365	5 -	Arabidopsis thaliana	abscisic acid responsive

TaMAPKKKK1	ABRE	TACGGTC	809	7 -	Arabidopsis thaliana	abscisic acid responsive
TaMAPKKKK1	ABRE	ACGTG	1036	5 +	Arabidopsis thaliana	abscisic acid responsive
TaMAPKKKK1	ABRE	ACGTG	1369	5 -	Arabidopsis thaliana	abscisic acid responsive
TaMAPKKKK1	ABRE	ACGTG	1393	5 -	Arabidopsis thaliana	abscisic acid responsive
TaMAPKKKK1	O2-site	GTTGACGTGA	364	9 -	Zea mays	zein metabolism regulation
TaMAPKKKK1	GCN4_motif	TGAGTCA	160	7 -	Oryza sativa	endosperm expression
TaMAPKKKK1	LTR	CCGAAA	1403	6 +	Hordeum vulgare	low temperature responsive
TaMAPKKKK1	TGACG-motif	TGACG	367	5 -	Hordeum vulgare	the MeJA responsive
TaMAPKKKK1	TGACG-motif	TGACG	417	5 +	Hordeum vulgare	the MeJA responsive
TaMAPKKKK1	TGACG-motif	TGACG	1395	5 -	Hordeum vulgare	the MeJA responsive
TaMAPKKKK10	O2-site	GATGACATGG	1228	9 +	Zea mays	zein metabolism regulation
TaMAPKKKK10	TGACG-motif	TGACG	43	5 -	Hordeum vulgare	the MeJA responsive
TaMAPKKKK10	TGACG-motif	TGACG	59	5 -	Hordeum vulgare	the MeJA responsive
TaMAPKKKK10	TGACG-motif	TGACG	985	5 -	Hordeum vulgare	the MeJA responsive
TaMAPKKKK10	CAT-box	GCCACT	486	6 +	Arabidopsis thaliana	meristem expression
TaMAPKKKK10	ABRE	ACGTG	41	5 -	Arabidopsis thaliana	abscisic acid responsive
TaMAPKKKK10	CGTCA-motif	CGTCA	43	5 +	Hordeum vulgare	the MeJA responsive
TaMAPKKKK10	CGTCA-motif	CGTCA	59	5 +	Hordeum vulgare	the MeJA responsive
TaMAPKKKK10	CGTCA-motif	CGTCA	985	5 +	Hordeum vulgare	the MeJA responsive

TaMAPKKKK10	LTR	CCGAAA	1138	6 +	Hordeum vulgare	low temperature responsive
TaMAPKKKK10	LTR	CCGAAA	1386	6 +	Hordeum vulgare	low temperature responsive
TaMAPKKKK10	LTR	CCGAAA	1605	6 -	Hordeum vulgare	low temperature responsive
TaMAPKKKK10	TGA-element	AACGAC	644	6 +	Brassica oleracea	auxin responsive
TaMAPKKKK10	TCA-element	CCATCTTTTT	830	9 -	Nicotiana tabacum	salicylic acid responsive
TaMAPKKKK11	TCA-element	TCAGAAGAGG	1725	9 +	Brassica oleracea	salicylic acid responsive
TaMAPKKKK11	CGTCA-motif	CGTCA	174	5 -	Hordeum vulgare	the MeJA responsive
TaMAPKKKK11	CGTCA-motif	CGTCA	397	5 +	Hordeum vulgare	the MeJA responsive
TaMAPKKKK11	CGTCA-motif	CGTCA	523	5 +	Hordeum vulgare	the MeJA responsive
TaMAPKKKK11	CGTCA-motif	CGTCA	649	5 +	Hordeum vulgare	the MeJA responsive
TaMAPKKKK11	CGTCA-motif	CGTCA	775	5 +	Hordeum vulgare	the MeJA responsive
TaMAPKKKK11	CGTCA-motif	CGTCA	901	5 +	Hordeum vulgare	the MeJA responsive
TaMAPKKKK11	CGTCA-motif	CGTCA	1244	5 -	Hordeum vulgare	the MeJA responsive
TaMAPKKKK11	CGTCA-motif	CGTCA	1551	5 +	Hordeum vulgare	the MeJA responsive
TaMAPKKKK11	CGTCA-motif	CGTCA	1656	5 +	Hordeum vulgare	the MeJA responsive
TaMAPKKKK11	LTR	CCGAAA	110	6 +	Hordeum vulgare	low temperature responsive
TaMAPKKKK11	LTR	CCGAAA	254	6 +	Hordeum vulgare	low temperature responsive
TaMAPKKKK11	TGA-element	AACGAC	1160	6 -	Brassica oleracea	auxin responsive
TaMAPKKKK11	TGA-element	AACGAC	1188	6 -	Brassica oleracea	auxin responsive

TaMAPKKKK11	TGA-element	AACGAC	1943	6 +	Brassica oleracea	auxin responsive
TaMAPKKKK11	ABRE	ACGTG	1387	5 -	Arabidopsis thaliana	abscisic acid responsive
TaMAPKKKK11	ABRE	ACGTG	1879	5 -	Arabidopsis thaliana	abscisic acid responsive
TaMAPKKKK11	CAT-box	GCCACT	1796	6 +	Arabidopsis thaliana	meristem expression
TaMAPKKKK11	TGACG-motif	TGACG	174	5 +	Hordeum vulgare	the MeJA responsive
TaMAPKKKK11	TGACG-motif	TGACG	397	5 -	Hordeum vulgare	the MeJA responsive
TaMAPKKKK11	TGACG-motif	TGACG	523	5 -	Hordeum vulgare	the MeJA responsive
TaMAPKKKK11	TGACG-motif	TGACG	649	5 -	Hordeum vulgare	the MeJA responsive
TaMAPKKKK11	TGACG-motif	TGACG	775	5 -	Hordeum vulgare	the MeJA responsive
TaMAPKKKK11	TGACG-motif	TGACG	901	5 -	Hordeum vulgare	the MeJA responsive
TaMAPKKKK11	TGACG-motif	TGACG	1244	5 +	Hordeum vulgare	the MeJA responsive
TaMAPKKKK11	TGACG-motif	TGACG	1551	5 -	Hordeum vulgare	the MeJA responsive
TaMAPKKKK11	TGACG-motif	TGACG	1656	5 -	Hordeum vulgare	the MeJA responsive
TaMAPKKKK11	GARE-motif	TCTGTTG	1893	7 +	Brassica oleracea	gibberellin responsive
TaMAPKKKK11	GC-motif	CCCCCG	1506	6 -	Zea mays	anoxic specific inducibility
TaMAPKKKK12	P-box	CCTTTTG	813	7 -	Oryza sativa	gibberellin responsive
TaMAPKKKK12	ABRE	ACGTG	836	5 -	Arabidopsis thaliana	abscisic acid responsive
TaMAPKKKK12	ABRE	GCAACGTGTC	876	9 -	Hordeum vulgare	abscisic acid responsive
TaMAPKKKK12	ABRE	ACGTG	878	5 -	Arabidopsis thaliana	abscisic acid responsive

TaMAPKKKK12	LTR	CCGAAA	944	6 +	Hordeum vulgare	low temperature responsive
TaMAPKKKK12	LTR	CCGAAA	1458	6 +	Hordeum vulgare	low temperature responsive
TaMAPKKKK12	TGACG-motif	TGACG	564	5 +	Hordeum vulgare	the MeJA responsive
TaMAPKKKK12	TGACG-motif	TGACG	854	5 -	Hordeum vulgare	the MeJA responsive
TaMAPKKKK12	TGACG-motif	TGACG	1587	5 -	Hordeum vulgare	the MeJA responsive
TaMAPKKKK12	CGTCA-motif	CGTCA	564	5 -	Hordeum vulgare	the MeJA responsive
TaMAPKKKK12	CGTCA-motif	CGTCA	854	5 +	Hordeum vulgare	the MeJA responsive
TaMAPKKKK12	CGTCA-motif	CGTCA	1587	5 +	Hordeum vulgare	the MeJA responsive
TaMAPKKKK12	ARE	AAACCA	344	6 -	Zea mays	anaerobic induction
TaMAPKKKK13	CAT-box	GCCACT	676	6 +	Arabidopsis thaliana	meristem expression
TaMAPKKKK13	CAT-box	GCCACT	1273	6 -	Arabidopsis thaliana	meristem expression
TaMAPKKKK13	TGACG-motif	TGACG	51	5 +	Hordeum vulgare	the MeJA responsive
TaMAPKKKK13	TGACG-motif	TGACG	871	5 -	Hordeum vulgare	the MeJA responsive
TaMAPKKKK13	TGACG-motif	TGACG	1384	5 +	Hordeum vulgare	the MeJA responsive
TaMAPKKKK13	CGTCA-motif	CGTCA	51	5 -	Hordeum vulgare	the MeJA responsive
TaMAPKKKK13	CGTCA-motif	CGTCA	871	5 +	Hordeum vulgare	the MeJA responsive
TaMAPKKKK13	CGTCA-motif	CGTCA	1384	5 -	Hordeum vulgare	the MeJA responsive
TaMAPKKKK13	LTR	CCGAAA	1407	6 -	Hordeum vulgare	low temperature responsive
TaMAPKKKK13	ABRE	ACGTG	53	5 +	Arabidopsis thaliana	abscisic acid responsive

TaMAPKKKK13	ABRE	ACGTG	481	5 +	Arabidopsis thaliana	abscisic acid responsive
TaMAPKKKK13	ABRE	ACGTG	563	5 -	Arabidopsis thaliana	abscisic acid responsive
TaMAPKKKK13	ABRE	ACGTG	726	5 -	Arabidopsis thaliana	abscisic acid responsive
TaMAPKKKK13	ABRE	ACGTG	1034	5 +	Arabidopsis thaliana	abscisic acid responsive
TaMAPKKKK13	ABRE	CACGTG	1332	6 -	Arabidopsis thaliana	abscisic acid responsive
TaMAPKKKK13	ABRE	ACGTG	1333	5 +	Arabidopsis thaliana	abscisic acid responsive
TaMAPKKKK13	ABRE	ACGTG	1504	5 +	Arabidopsis thaliana	abscisic acid responsive
TaMAPKKKK13	TGA-element	AACGAC	896	6 -	Brassica oleracea	auxin responsive
TaMAPKKKK13	MBS	CAACTG	338	6 -	Arabidopsis thaliana	drought inducibility
TaMAPKKKK13	GARE-motif	TCTGTTG	1594	7 +	Brassica oleracea	gibberellin responsive
TaMAPKKKK13	TCA-element	CCATCTTTTT	1732	9 +	Nicotiana tabacum	salicylic acid responsive
TaMAPKKKK13	TCA-element	CCATCTTTTT	1788	9 +	Nicotiana tabacum	salicylic acid responsive
TaMAPKKKK14	LTR	CCGAAA	154	6 +	Hordeum vulgare	low temperature responsive
TaMAPKKKK14	LTR	CCGAAA	1664	6 -	Hordeum vulgare	low temperature responsive
TaMAPKKKK14	LTR	CCGAAA	1678	6 -	Hordeum vulgare	low temperature responsive
TaMAPKKKK14	ABRE	TACGGTC	648	7 +	Arabidopsis thaliana	abscisic acid responsive
TaMAPKKKK14	ABRE	ACGTG	1588	5 +	Arabidopsis thaliana	abscisic acid responsive
TaMAPKKKK15	AuxRR-core	GGTCCAT	1100	7 -	Nicotiana tabacum	auxin responsive
TaMAPKKKK15	CGTCA-motif	CGTCA	113	5 -	Hordeum vulgare	the MeJA responsive

TaMAPKKKK15	CGTCA-motif	CGTCA	907	5 -	Hordeum vulgare	the MeJA responsive
TaMAPKKKK15	CGTCA-motif	CGTCA	951	5 -	Hordeum vulgare	the MeJA responsive
TaMAPKKKK15	TGACG-motif	TGACG	113	5 +	Hordeum vulgare	the MeJA responsive
TaMAPKKKK15	TGACG-motif	TGACG	907	5 +	Hordeum vulgare	the MeJA responsive
TaMAPKKKK15	TGACG-motif	TGACG	951	5 +	Hordeum vulgare	the MeJA responsive
TaMAPKKKK15	LTR	CCGAAA	1405	6 +	Hordeum vulgare	low temperature responsive
TaMAPKKKK15	O2-site	GATGA(C/T)(A/G)TG(A/G)	520	8 -	Zea mays	zein metabolism regulation
TaMAPKKKK15	O2-site	GATGACATGG	1361	9 -	Zea mays	zein metabolism regulation
TaMAPKKKK15	CAT-box	GCCACT	75	6 +	Arabidopsis thaliana	meristem expression
TaMAPKKKK15	circadian	CAAAGATATC	1200	9 +	Lycopersicon esculentum	circadian control
TaMAPKKKK15	MBS	CAACTG	1018	6 -	Arabidopsis thaliana	drought inducibility
TaMAPKKKK15	ABRE	CACGTG	409	6 +	Arabidopsis thaliana	abscisic acid responsive
TaMAPKKKK15	ABRE	ACGTG	410	5 +	Arabidopsis thaliana	abscisic acid responsive
TaMAPKKKK15	GC-motif	CCCCCG	862	6 -	Zea mays	anoxic specific inducibility
TaMAPKKKK15	TGA-element	AACGAC	935	6 -	Brassica oleracea	auxin responsive
TaMAPKKKK15	TGA-element	AACGAC	1598	6 +	Brassica oleracea	auxin responsive
TaMAPKKKK15	TGA-element	AACGAC	1641	6 +	Brassica oleracea	auxin responsive
TaMAPKKKK16	CAT-box	GCCACT	1548	6 +	Arabidopsis thaliana	meristem expression
TaMAPKKKK16	CAT-box	GCCACT	1574	6 +	Arabidopsis thaliana	meristem expression

TaMAPKKKK16	CGTCA-motif	CGTCA	168	5 -	Hordeum vulgare	the MeJA responsive
TaMAPKKKK16	CGTCA-motif	CGTCA	194	5 -	Hordeum vulgare	the MeJA responsive
TaMAPKKKK16	CGTCA-motif	CGTCA	762	5 +	Hordeum vulgare	the MeJA responsive
TaMAPKKKK16	CGTCA-motif	CGTCA	1447	5 -	Hordeum vulgare	the MeJA responsive
TaMAPKKKK16	CGTCA-motif	CGTCA	1891	5 +	Hordeum vulgare	the MeJA responsive
TaMAPKKKK16	TGACG-motif	TGACG	168	5 +	Hordeum vulgare	the MeJA responsive
TaMAPKKKK16	TGACG-motif	TGACG	194	5 +	Hordeum vulgare	the MeJA responsive
TaMAPKKKK16	TGACG-motif	TGACG	762	5 -	Hordeum vulgare	the MeJA responsive
TaMAPKKKK16	TGACG-motif	TGACG	1447	5 +	Hordeum vulgare	the MeJA responsive
TaMAPKKKK16	TGACG-motif	TGACG	1891	5 -	Hordeum vulgare	the MeJA responsive
TaMAPKKKK16	TC-rich repeats	GTTTTCTTAC	990	9 -	Nicotiana tabacum	defense and stress responsive
TaMAPKKKK16	TGA-element	AACGAC	81	6 -	Brassica oleracea	auxin responsive
TaMAPKKKK16	RY-element	CATGCATG	372	8 +	Helianthus annuus	seed specific regulation
TaMAPKKKK16	ABRE	CACGTG	266	6 +	Arabidopsis thaliana	abscisic acid responsive
TaMAPKKKK16	ABRE	ACGTG	267	5 +	Arabidopsis thaliana	abscisic acid responsive
TaMAPKKKK16	ABRE	ACGTG	1347	5 +	Arabidopsis thaliana	abscisic acid responsive
TaMAPKKKK16	TCA-element	TCAGAAGAGG	1590	9 -	Brassica oleracea	salicylic acid responsive
TaMAPKKKK16	MBS	CAACTG	216	6 -	Arabidopsis thaliana	drought inducibility
TaMAPKKKK17	ABRE	ACGTG	71	5 +	Arabidopsis thaliana	abscisic acid responsive

TaMAPKKKK17	CAT-box	GCCACT	272	6 +	Arabidopsis thaliana	meristem expression
TaMAPKKKK17	CAT-box	GCCACT	298	6 +	Arabidopsis thaliana	meristem expression
TaMAPKKKK17	CGTCA-motif	CGTCA	171	5 -	Hordeum vulgare	the MeJA responsive
TaMAPKKKK17	CGTCA-motif	CGTCA	1891	5 +	Hordeum vulgare	the MeJA responsive
TaMAPKKKK17	TCA-element	TCAGAAGAGG	314	9 -	Brassica oleracea	salicylic acid responsive
TaMAPKKKK17	TGACG-motif	TGACG	171	5 +	Hordeum vulgare	the MeJA responsive
TaMAPKKKK17	TGACG-motif	TGACG	1891	5 -	Hordeum vulgare	the MeJA responsive
TaMAPKKKK18	ARE	AAACCA	376	6 -	Zea mays	anaerobic induction
TaMAPKKKK18	TGACG-motif	TGACG	1053	5 -	Hordeum vulgare	the MeJA responsive
TaMAPKKKK18	TGACG-motif	TGACG	1071	5 -	Hordeum vulgare	the MeJA responsive
TaMAPKKKK18	CGTCA-motif	CGTCA	1053	5 +	Hordeum vulgare	the MeJA responsive
TaMAPKKKK18	CGTCA-motif	CGTCA	1071	5 +	Hordeum vulgare	the MeJA responsive
TaMAPKKKK18	LTR	CCGAAA	276	6 +	Hordeum vulgare	low temperature responsive
TaMAPKKKK18	ABRE	ACGTG	480	5 +	Arabidopsis thaliana	abscisic acid responsive
TaMAPKKKK18	ABRE	ACGTG	520	5 +	Arabidopsis thaliana	abscisic acid responsive
TaMAPKKKK18	ABRE	CACGTG	882	6 +	Arabidopsis thaliana	abscisic acid responsive
TaMAPKKKK18	ABRE	ACGTG	883	5 +	Arabidopsis thaliana	abscisic acid responsive
TaMAPKKKK18	GARE-motif	TCTGTTG	1916	7 -	Brassica oleracea	gibberellin responsive
TaMAPKKKK19	ARE	AAACCA	980	6 -	Zea mays	anaerobic induction

TaMAPKKKK19	CAT-box	GCCACT	1699	6 -	Arabidopsis thaliana	meristem expression
TaMAPKKKK19	TGACG-motif	TGACG	218	5 +	Hordeum vulgare	the MeJA responsive
TaMAPKKKK19	TGACG-motif	TGACG	1869	5 -	Hordeum vulgare	the MeJA responsive
TaMAPKKKK19	CGTCA-motif	CGTCA	218	5 -	Hordeum vulgare	the MeJA responsive
TaMAPKKKK19	CGTCA-motif	CGTCA	1869	5 +	Hordeum vulgare	the MeJA responsive
TaMAPKKKK19	LTR	CCGAAA	461	6 -	Hordeum vulgare	low temperature responsive
TaMAPKKKK19	LTR	CCGAAA	1562	6 -	Hordeum vulgare	low temperature responsive
TaMAPKKKK19	ABRE	TACGGTC	17	7 +	Arabidopsis thaliana	abscisic acid responsive
TaMAPKKKK19	ABRE	CACGTG	29	6 +	Arabidopsis thaliana	abscisic acid responsive
TaMAPKKKK19	ABRE	ACGTG	30	5 +	Arabidopsis thaliana	abscisic acid responsive
TaMAPKKKK19	ABRE	ACGTG	429	5 +	Arabidopsis thaliana	abscisic acid responsive
TaMAPKKKK19	ABRE	AACCCGG	1626	7 -	Arabidopsis thaliana	abscisic acid responsive
TaMAPKKKK19	ABRE	GCCGCGTGGC	1695	9 +	Oryza sativa	abscisic acid responsive
TaMAPKKKK19	GC-motif	CCCCCG	1448	6 +	Zea mays	anoxic specific inducibility
TaMAPKKKK19	P-box	CCTTTTG	727	7 +	Oryza sativa	gibberellin responsive
TaMAPKKKK19	P-box	CCTTTTG	1134	7 -	Oryza sativa	gibberellin responsive
TaMAPKKKK2	LTR	CCGAAA	555	6 -	Hordeum vulgare	low temperature responsive
TaMAPKKKK2	LTR	CCGAAA	1721	6 +	Hordeum vulgare	low temperature responsive
TaMAPKKKK2	TGACG-motif	TGACG	1629	5 +	Hordeum vulgare	the MeJA responsive

TaMAPKKKK2	TGACG-motif	TGACG	1713	5 -	Hordeum vulgare	the MeJA responsive
TaMAPKKKK2	TCA-element	CCATCTTTTT	1017	9 +	Nicotiana tabacum	salicylic acid responsive
TaMAPKKKK2	ARE	AAACCA	608	6 +	Zea mays	anaerobic induction
TaMAPKKKK2	ARE	AAACCA	708	6 -	Zea mays	anaerobic induction
TaMAPKKKK2	ARE	AAACCA	1441	6 +	Zea mays	anaerobic induction
TaMAPKKKK2	CAT-box	GCCACT	1589	6 +	Arabidopsis thaliana	meristem expression
TaMAPKKKK2	CAT-box	GCCACT	1603	6 +	Arabidopsis thaliana	meristem expression
TaMAPKKKK2	CGTCA-motif	CGTCA	1629	5 -	Hordeum vulgare	the MeJA responsive
TaMAPKKKK2	CGTCA-motif	CGTCA	1713	5 +	Hordeum vulgare	the MeJA responsive
TaMAPKKKK2	TGA-element	AACGAC	696	6 -	Brassica oleracea	auxin responsive
TaMAPKKKK2	ABRE	GACACGTGGC	619	9 +	Triticum aestivum	abscisic acid responsive
TaMAPKKKK2	ABRE	ACGTG	1312	5 +	Arabidopsis thaliana	abscisic acid responsive
TaMAPKKKK2	ABRE	ACGTG	1689	5 -	Arabidopsis thaliana	abscisic acid responsive
TaMAPKKKK2	ABRE	ACGTG	1711	5 -	Arabidopsis thaliana	abscisic acid responsive
TaMAPKKKK2	GC-motif	CCCCCG	1218	6 +	Zea mays	anoxic specific inducibility
TaMAPKKKK2	GC-motif	CCCCCG	1645	6 -	Zea mays	anoxic specific inducibility
TaMAPKKKK2	GC-motif	CCCCCG	1704	6 +	Zea mays	anoxic specific inducibility
TaMAPKKKK20	LTR	CCGAAA	44	6 +	Hordeum vulgare	low temperature responsive
TaMAPKKKK20	LTR	CCGAAA	163	6 +	Hordeum vulgare	low temperature responsive

TaMAPKKKK20	LTR	CCGAAA	264	6 -	Hordeum vulgare	low temperature responsive
TaMAPKKKK20	LTR	CCGAAA	395	6 +	Hordeum vulgare	low temperature responsive
TaMAPKKKK20	LTR	CCGAAA	1128	6 -	Hordeum vulgare	low temperature responsive
TaMAPKKKK20	TC-rich repeats	GTTTTCTTAC	1205	9 +	Nicotiana tabacum	defense and stress responsive
TaMAPKKKK20	CAT-box	GCCACT	806	6 +	Arabidopsis thaliana	meristem expression
TaMAPKKKK20	CAT-box	GCCACT	1661	6 -	Arabidopsis thaliana	meristem expression
TaMAPKKKK20	ABRE	ACGTG	580	5 +	Arabidopsis thaliana	abscisic acid responsive
TaMAPKKKK20	ABRE	CACGTG	1294	6 -	Arabidopsis thaliana	abscisic acid responsive
TaMAPKKKK20	ABRE	ACGTG	1295	5 +	Arabidopsis thaliana	abscisic acid responsive
TaMAPKKKK20	ABRE	GCCGCGTGGC	1657	9 +	Oryza sativa	abscisic acid responsive
TaMAPKKKK20	ARE	AAACCA	541	6 -	Zea mays	anaerobic induction
TaMAPKKKK20	TGACG-motif	TGACG	1277	5 +	Hordeum vulgare	the MeJA responsive
TaMAPKKKK20	TGACG-motif	TGACG	1871	5 -	Hordeum vulgare	the MeJA responsive
TaMAPKKKK20	CGTCA-motif	CGTCA	1277	5 -	Hordeum vulgare	the MeJA responsive
TaMAPKKKK20	CGTCA-motif	CGTCA	1871	5 +	Hordeum vulgare	the MeJA responsive
TaMAPKKKK21	CAT-box	GCCACT	799	6 -	Arabidopsis thaliana	meristem expression
TaMAPKKKK21	MBS	CAACTG	91	6 -	Arabidopsis thaliana	drought inducibility
TaMAPKKKK21	MBS	CAACTG	1024	6 -	Arabidopsis thaliana	drought inducibility
TaMAPKKKK21	TC-rich repeats	GTTTTCTTAC	258	9 +	Nicotiana tabacum	defense and stress responsive

TaMAPKKKK21	CGTCA-motif	CGTCA	923	5 -	Hordeum vulgare	the MeJA responsive
TaMAPKKKK21	TGACG-motif	TGACG	923	5 +	Hordeum vulgare	the MeJA responsive
TaMAPKKKK21	TCA-element	CCATCTTTTT	1737	9 +	Nicotiana tabacum	salicylic acid responsive
TaMAPKKKK21	circadian	CAAAGATATC	480	9 +	Lycopersicon esculentum	circadian control
TaMAPKKKK21	TGA-element	AACGAC	1450	6 -	Brassica oleracea	auxin responsive
TaMAPKKKK21	TGA-element	AACGAC	1483	6 -	Brassica oleracea	auxin responsive
TaMAPKKKK21	TGA-element	AACGAC	1498	6 -	Brassica oleracea	auxin responsive
TaMAPKKKK21	TGA-element	AACGAC	1507	6 -	Brassica oleracea	auxin responsive
TaMAPKKKK21	ARE	AAACCA	1329	6 -	Zea mays	anaerobic induction
TaMAPKKKK22	MBS	CAACTG	94	6 +	Arabidopsis thaliana	drought inducibility
TaMAPKKKK22	P-box	CCTTTTG	572	7 +	Oryza sativa	gibberellin responsive
TaMAPKKKK22	P-box	CCTTTTG	589	7 +	Oryza sativa	gibberellin responsive
TaMAPKKKK22	TGACG-motif	TGACG	196	5 -	Hordeum vulgare	the MeJA responsive
TaMAPKKKK22	TGACG-motif	TGACG	1979	5 +	Hordeum vulgare	the MeJA responsive
TaMAPKKKK22	CGTCA-motif	CGTCA	196	5 +	Hordeum vulgare	the MeJA responsive
TaMAPKKKK22	CGTCA-motif	CGTCA	1979	5 -	Hordeum vulgare	the MeJA responsive
TaMAPKKKK22	GC-motif	CCCCCG	828	6 +	Zea mays	anoxic specific inducibility
TaMAPKKKK22	GC-motif	CCCCCG	977	6 +	Zea mays	anoxic specific inducibility
TaMAPKKKK22	ABRE	ACGTG	130	5 +	Arabidopsis thaliana	abscisic acid responsive

TaMAPKKKK22	ABRE	ACGTG	283	5 -	Arabidopsis thaliana	abscisic acid responsive
TaMAPKKKK22	ABRE	AACCCGG	1083	7 -	Arabidopsis thaliana	abscisic acid responsive
TaMAPKKKK22	TGA-element	AACGAC	308	6 -	Brassica oleracea	auxin responsive
TaMAPKKKK22	ARE	AAACCA	694	6 +	Zea mays	anaerobic induction
TaMAPKKKK22	ARE	AAACCA	1888	6 -	Zea mays	anaerobic induction
TaMAPKKKK23	GC-motif	CCCCCG	828	6 +	Zea mays	anoxic specific inducibility
TaMAPKKKK23	GC-motif	CCCCCG	980	6 +	Zea mays	anoxic specific inducibility
TaMAPKKKK23	ARE	AAACCA	181	6 -	Zea mays	anaerobic induction
TaMAPKKKK23	ARE	AAACCA	702	6 +	Zea mays	anaerobic induction
TaMAPKKKK23	ARE	AAACCA	1549	6 -	Zea mays	anaerobic induction
TaMAPKKKK23	ABRE	ACGTG	17	5 +	Arabidopsis thaliana	abscisic acid responsive
TaMAPKKKK23	ABRE	AACCCGG	1085	7 -	Arabidopsis thaliana	abscisic acid responsive
TaMAPKKKK23	ABRE	CGCACGTGTC	1374	9 +	Hordeum vulgare	abscisic acid responsive
TaMAPKKKK23	ABRE	ACGTG	1675	5 +	Arabidopsis thaliana	abscisic acid responsive
TaMAPKKKK23	P-box	CCTTTTG	580	7 +	Oryza sativa	gibberellin responsive
TaMAPKKKK23	P-box	CCTTTTG	597	7 +	Oryza sativa	gibberellin responsive
TaMAPKKKK23	TGACG-motif	TGACG	204	5 -	Hordeum vulgare	the MeJA responsive
TaMAPKKKK23	TGACG-motif	TGACG	1979	5 +	Hordeum vulgare	the MeJA responsive
TaMAPKKKK23	CGTCA-motif	CGTCA	204	5 +	Hordeum vulgare	the MeJA responsive

TaMAPKKKK23	CGTCA-motif	CGTCA	1979	5 -	Hordeum vulgare	the MeJA responsive
TaMAPKKKK24	CAT-box	GCCACT	961	6 +	Arabidopsis thaliana	meristem expression
TaMAPKKKK24	GC-motif	CCCCCG	852	6 +	Zea mays	anoxic specific inducibility
TaMAPKKKK24	GC-motif	CCCCCG	997	6 +	Zea mays	anoxic specific inducibility
TaMAPKKKK24	TCA-element	CCATCTTTTT	99	9 -	Nicotiana tabacum	salicylic acid responsive
TaMAPKKKK24	TGA-element	AACGAC	413	6 -	Brassica oleracea	auxin responsive
TaMAPKKKK24	ABRE	ACGTG	299	5 -	Arabidopsis thaliana	abscisic acid responsive
TaMAPKKKK24	ABRE	ACGTG	388	5 -	Arabidopsis thaliana	abscisic acid responsive
TaMAPKKKK24	ABRE	AACCCGG	1102	7 -	Arabidopsis thaliana	abscisic acid responsive
TaMAPKKKK24	ARE	AAACCA	726	6 +	Zea mays	anaerobic induction
TaMAPKKKK24	P-box	CCTTTTG	604	7 +	Oryza sativa	gibberellin responsive
TaMAPKKKK24	P-box	CCTTTTG	621	7 +	Oryza sativa	gibberellin responsive
TaMAPKKKK24	TGACG-motif	TGACG	1979	5 +	Hordeum vulgare	the MeJA responsive
TaMAPKKKK24	CGTCA-motif	CGTCA	1979	5 -	Hordeum vulgare	the MeJA responsive
TaMAPKKKK25	LTR	CCGAAA	1203	6 +	Hordeum vulgare	low temperature responsive
TaMAPKKKK25	LTR	CCGAAA	1372	6 -	Hordeum vulgare	low temperature responsive
TaMAPKKKK25	LTR	CCGAAA	1394	6 -	Hordeum vulgare	low temperature responsive
TaMAPKKKK25	O2-site	GATGATGTGG	1457	9 +	Zea mays	zein metabolism regulation
TaMAPKKKK25	GC-motif	CCCCCG	1005	6 +	Zea mays	anoxic specific inducibility

TaMAPKKKK25	GC-motif	CCCCCG	1646	6 -	Zea mays	anoxic specific inducibility
TaMAPKKKK25	ABRE	AACCCGG	220	7 +	Arabidopsis thaliana	abscisic acid responsive
TaMAPKKKK25	ABRE	ACGTG	691	5 +	Arabidopsis thaliana	abscisic acid responsive
TaMAPKKKK25	ABRE	GCCGCGTGGC	1685	9 -	Oryza sativa	abscisic acid responsive
TaMAPKKKK25	ABRE	ACGTG	1796	5 -	Arabidopsis thaliana	abscisic acid responsive
TaMAPKKKK25	TGA-element	AACGAC	1933	6 +	Brassica oleracea	auxin responsive
TaMAPKKKK25	ARE	AAACCA	147	6 +	Zea mays	anaerobic induction
TaMAPKKKK25	TGACG-motif	TGACG	198	5 -	Hordeum vulgare	the MeJA responsive
TaMAPKKKK25	TGACG-motif	TGACG	259	5 -	Hordeum vulgare	the MeJA responsive
TaMAPKKKK25	TGACG-motif	TGACG	306	5 -	Hordeum vulgare	the MeJA responsive
TaMAPKKKK25	TGACG-motif	TGACG	449	5 +	Hordeum vulgare	the MeJA responsive
TaMAPKKKK25	TGACG-motif	TGACG	1048	5 -	Hordeum vulgare	the MeJA responsive
TaMAPKKKK25	TGACG-motif	TGACG	1163	5 -	Hordeum vulgare	the MeJA responsive
TaMAPKKKK25	TGACG-motif	TGACG	1217	5 -	Hordeum vulgare	the MeJA responsive
TaMAPKKKK25	CGTCA-motif	CGTCA	198	5 +	Hordeum vulgare	the MeJA responsive
TaMAPKKKK25	CGTCA-motif	CGTCA	259	5 +	Hordeum vulgare	the MeJA responsive
TaMAPKKKK25	CGTCA-motif	CGTCA	306	5 +	Hordeum vulgare	the MeJA responsive
TaMAPKKKK25	CGTCA-motif	CGTCA	449	5 -	Hordeum vulgare	the MeJA responsive
TaMAPKKKK25	CGTCA-motif	CGTCA	1048	5 +	Hordeum vulgare	the MeJA responsive

TaMAPKKKK25	CGTCA-motif	CGTCA	1163	5 +	Hordeum vulgare	the MeJA responsive
TaMAPKKKK25	CGTCA-motif	CGTCA	1217	5 +	Hordeum vulgare	the MeJA responsive
TaMAPKKKK3	TATC-box	TATCCCA	95	7 +	Oryza sativa	gibberellin responsive
TaMAPKKKK3	LTR	CCGAAA	1720	6 +	Hordeum vulgare	low temperature responsive
TaMAPKKKK3	GCN4_motif	TGAGTCA	479	7 -	Oryza sativa	endosperm expression
TaMAPKKKK3	O2-site	GTTGACGTGA	145	9 -	Zea mays	zein metabolism regulation
TaMAPKKKK3	O2-site	GTTGACGTGA	683	9 -	Zea mays	zein metabolism regulation
TaMAPKKKK3	TGACG-motif	TGACG	686	5 -	Hordeum vulgare	the MeJA responsive
TaMAPKKKK3	TGACG-motif	TGACG	736	5 +	Hordeum vulgare	the MeJA responsive
TaMAPKKKK3	TGACG-motif	TGACG	1112	5 +	Hordeum vulgare	the MeJA responsive
TaMAPKKKK3	TGACG-motif	TGACG	1712	5 -	Hordeum vulgare	the MeJA responsive
TaMAPKKKK3	MBSI	TTTTTACGGTTA	1178	11 +	Petunia hybrida	flavonoid biosynthetic genes regulation
TaMAPKKKK3	ARE	AAACCA	192	6 +	Zea mays	anaerobic induction
TaMAPKKKK3	TC-rich repeats	ATTCTCTAAC	871	9 -	Nicotiana tabacum	defense and stress responsive
TaMAPKKKK3	TC-rich repeats	ATTCTCTAAC	886	9 +	Nicotiana tabacum	defense and stress responsive
TaMAPKKKK3	TC-rich repeats	GTTTTCTTAC	1439	9 +	Nicotiana tabacum	defense and stress responsive
TaMAPKKKK3	RY-element	CATGCATG	103	8 +	Helianthus annuus	seed specific regulation
TaMAPKKKK3	RY-element	CATGCATG	196	8 +	Helianthus annuus	seed specific regulation

TaMAPKKKK3	CGTCA-motif	CGTCA	686	5 +	Hordeum vulgare	the MeJA responsive
TaMAPKKKK3	CGTCA-motif	CGTCA	736	5 -	Hordeum vulgare	the MeJA responsive
TaMAPKKKK3	CGTCA-motif	CGTCA	1112	5 -	Hordeum vulgare	the MeJA responsive
TaMAPKKKK3	CGTCA-motif	CGTCA	1712	5 +	Hordeum vulgare	the MeJA responsive
TaMAPKKKK3	CAT-box	GCCACT	936	6 -	Arabidopsis thaliana	meristem expression
TaMAPKKKK3	CAT-box	GCCACT	1587	6 +	Arabidopsis thaliana	meristem expression
TaMAPKKKK3	CAT-box	GCCACT	1601	6 +	Arabidopsis thaliana	meristem expression
TaMAPKKKK3	CAT-box	GCCACT	1617	6 +	Arabidopsis thaliana	meristem expression
TaMAPKKKK3	GC-motif	CCCCCG	1643	6 -	Zea mays	anoxic specific inducibility
TaMAPKKKK3	GC-motif	CCCCCG	1697	6 +	Zea mays	anoxic specific inducibility
TaMAPKKKK3	GC-motif	CCCCCG	1703	6 +	Zea mays	anoxic specific inducibility
TaMAPKKKK3	ABRE	CACGTG	351	6 +	Arabidopsis thaliana	abscisic acid responsive
TaMAPKKKK3	ABRE	ACGTG	352	5 +	Arabidopsis thaliana	abscisic acid responsive
TaMAPKKKK3	ABRE	ACGTG	501	5 +	Arabidopsis thaliana	abscisic acid responsive
TaMAPKKKK3	ABRE	ACGTG	684	5 -	Arabidopsis thaliana	abscisic acid responsive
TaMAPKKKK3	ABRE	CACGTG	1084	6 -	Arabidopsis thaliana	abscisic acid responsive
TaMAPKKKK3	ABRE	ACGTG	1085	5 +	Arabidopsis thaliana	abscisic acid responsive
TaMAPKKKK3	ABRE	ACGTG	1109	5 +	Arabidopsis thaliana	abscisic acid responsive
TaMAPKKKK3	ABRE	ACGTG	1351	5 +	Arabidopsis thaliana	abscisic acid responsive

TaMAPKKKK3	ABRE	ACGTG	1687	5 -	Arabidopsis thaliana	abscisic acid responsive
TaMAPKKKK3	ABRE	ACGTG	1710	5 -	Arabidopsis thaliana	abscisic acid responsive
TaMAPKKKK4	P-box	CCTTTTG	458	7 -	Oryza sativa	gibberellin responsive
TaMAPKKKK4	P-box	CCTTTTG	506	7 -	Oryza sativa	gibberellin responsive
TaMAPKKKK4	P-box	CCTTTTG	1106	7 -	Oryza sativa	gibberellin responsive
TaMAPKKKK4	ABRE	ACGTG	153	5 +	Arabidopsis thaliana	abscisic acid responsive
TaMAPKKKK4	ABRE	ACGTG	601	5 -	Arabidopsis thaliana	abscisic acid responsive
TaMAPKKKK4	ABRE	ACGTG	661	5 -	Arabidopsis thaliana	abscisic acid responsive
TaMAPKKKK4	ABRE	CACGTG	996	6 +	Arabidopsis thaliana	abscisic acid responsive
TaMAPKKKK4	ABRE	ACGTG	997	5 +	Arabidopsis thaliana	abscisic acid responsive
TaMAPKKKK4	ABRE	ACGTG	1276	5 +	Arabidopsis thaliana	abscisic acid responsive
TaMAPKKKK4	ABRE	ACGTG	1589	5 +	Arabidopsis thaliana	abscisic acid responsive
TaMAPKKKK4	ABRE	GCCGCGTGGC	1752	9 -	Oryza sativa	abscisic acid responsive
TaMAPKKKK4	CAT-box	GCCACT	1336	6 -	Arabidopsis thaliana	meristem expression
TaMAPKKKK4	CAT-box	GCCACT	1774	6 +	Arabidopsis thaliana	meristem expression
TaMAPKKKK4	CGTCA-motif	CGTCA	151	5 -	Hordeum vulgare	the MeJA responsive
TaMAPKKKK4	CGTCA-motif	CGTCA	169	5 +	Hordeum vulgare	the MeJA responsive
TaMAPKKKK4	MBS	CAACTG	1561	6 -	Arabidopsis thaliana	drought inducibility
TaMAPKKKK4	ARE	AAACCA	419	6 -	Zea mays	anaerobic induction

TaMAPKKKK4	ARE	AAACCA	898	6 +	Zea mays	anaerobic induction
TaMAPKKKK4	ARE	AAACCA	1518	6 -	Zea mays	anaerobic induction
TaMAPKKKK4	ARE	AAACCA	1799	6 +	Zea mays	anaerobic induction
TaMAPKKKK4	ARE	AAACCA	1844	6 +	Zea mays	anaerobic induction
TaMAPKKKK4	TGACG-motif	TGACG	151	5 +	Hordeum vulgare	the MeJA responsive
TaMAPKKKK4	TGACG-motif	TGACG	169	5 -	Hordeum vulgare	the MeJA responsive
TaMAPKKKK4	TCA-element	CCATCTTTTT	1350	9 -	Nicotiana tabacum	salicylic acid responsive
TaMAPKKKK4	O2-site	GTTGACGTGA	923	9 +	Zea mays	zein metabolism regulation
TaMAPKKKK4	LTR	CCGAAA	1053	6 -	Hordeum vulgare	low temperature responsive
TaMAPKKKK4	circadian	CAAAGATATC	1533	9 -	Lycopersicon esculentum	circadian control
TaMAPKKKK5	LTR	CCGAAA	1488	6 -	Hordeum vulgare	low temperature responsive
TaMAPKKKK5	TGACG-motif	TGACG	477	5 +	Hordeum vulgare	the MeJA responsive
TaMAPKKKK5	TGACG-motif	TGACG	675	5 -	Hordeum vulgare	the MeJA responsive
TaMAPKKKK5	ARE	AAACCA	1799	6 +	Zea mays	anaerobic induction
TaMAPKKKK5	MBS	CAACTG	343	6 +	Arabidopsis thaliana	drought inducibility
TaMAPKKKK5	CGTCA-motif	CGTCA	477	5 -	Hordeum vulgare	the MeJA responsive
TaMAPKKKK5	CGTCA-motif	CGTCA	675	5 +	Hordeum vulgare	the MeJA responsive
TaMAPKKKK5	CAT-box	GCCACT	252	6 +	Arabidopsis thaliana	meristem expression
TaMAPKKKK5	CAT-box	GCCACT	1774	6 +	Arabidopsis thaliana	meristem expression

TaMAPKKKK5	GC-motif	CCCCCG	1894	6 +	Zea mays	anoxic specific inducibility
TaMAPKKKK5	ABRE	ACGTG	18	5 -	Arabidopsis thaliana	abscisic acid responsive
TaMAPKKKK5	ABRE	ACGTG	624	5 +	Arabidopsis thaliana	abscisic acid responsive
TaMAPKKKK5	ABRE	AACCCGG	659	7 +	Arabidopsis thaliana	abscisic acid responsive
TaMAPKKKK5	ABRE	ACGTG	1070	5 -	Arabidopsis thaliana	abscisic acid responsive
TaMAPKKKK5	ABRE	GCCGCGTGGC	1752	9 -	Oryza sativa	abscisic acid responsive
TaMAPKKKK6	O2-site	GTTGACGTGA	893	9 +	Zea mays	zein metabolism regulation
TaMAPKKKK6	P-box	CCTTTTG	229	7 -	Oryza sativa	gibberellin responsive
TaMAPKKKK6	P-box	CCTTTTG	277	7 -	Oryza sativa	gibberellin responsive
TaMAPKKKK6	P-box	CCTTTTG	1076	7 -	Oryza sativa	gibberellin responsive
TaMAPKKKK6	LTR	CCGAAA	1023	6 -	Hordeum vulgare	low temperature responsive
TaMAPKKKK6	CGTCA-motif	CGTCA	900	5 -	Hordeum vulgare	the MeJA responsive
TaMAPKKKK6	CGTCA-motif	CGTCA	1483	5 +	Hordeum vulgare	the MeJA responsive
TaMAPKKKK6	CGTCA-motif	CGTCA	1881	5 -	Hordeum vulgare	the MeJA responsive
TaMAPKKKK6	TGA-element	AACGAC	401	6 +	Brassica oleracea	auxin responsive
TaMAPKKKK6	TGA-element	AACGAC	1333	6 +	Brassica oleracea	auxin responsive
TaMAPKKKK6	ABRE	CACGTG	966	6 +	Arabidopsis thaliana	abscisic acid responsive
TaMAPKKKK6	ABRE	ACGTG	967	5 +	Arabidopsis thaliana	abscisic acid responsive
TaMAPKKKK6	ABRE	CACGTG	1006	6 -	Arabidopsis thaliana	abscisic acid responsive
TaMAPKKKK6	ABRE	ACGTG	1007	5 +	Arabidopsis thaliana	abscisic acid responsive

TaMAPKKKK6	ABRE	ACGTG	1635	5 +	Arabidopsis thaliana	abscisic acid responsive
TaMAPKKKK6	ABRE	GCCGCGTGGC	1752	9 -	Oryza sativa	abscisic acid responsive
TaMAPKKKK6	CAT-box	GCCACT	426	6 -	Arabidopsis thaliana	meristem expression
TaMAPKKKK6	CAT-box	GCCACT	608	6 +	Arabidopsis thaliana	meristem expression
TaMAPKKKK6	CAT-box	GCCACT	731	6 +	Arabidopsis thaliana	meristem expression
TaMAPKKKK6	CAT-box	GCCACT	1305	6 -	Arabidopsis thaliana	meristem expression
TaMAPKKKK6	CAT-box	GCCACT	1774	6 +	Arabidopsis thaliana	meristem expression
TaMAPKKKK6	ARE	AAACCA	514	6 +	Zea mays	anaerobic induction
TaMAPKKKK6	ARE	AAACCA	1799	6 +	Zea mays	anaerobic induction
TaMAPKKKK6	TGACG-motif	TGACG	900	5 +	Hordeum vulgare	the MeJA responsive
TaMAPKKKK6	TGACG-motif	TGACG	1483	5 -	Hordeum vulgare	the MeJA responsive
TaMAPKKKK6	TGACG-motif	TGACG	1881	5 +	Hordeum vulgare	the MeJA responsive
TaMAPKKKK7	TGACG-motif	TGACG	753	5 +	Hordeum vulgare	the MeJA responsive
TaMAPKKKK7	TGACG-motif	TGACG	1472	5 -	Hordeum vulgare	the MeJA responsive
TaMAPKKKK7	TGACG-motif	TGACG	1496	5 -	Hordeum vulgare	the MeJA responsive
TaMAPKKKK7	GARE-motif	TCTGTTG	684	7 +	Brassica oleracea	gibberellin responsive
TaMAPKKKK7	ARE	AAACCA	554	6 -	Zea mays	anaerobic induction
TaMAPKKKK7	ARE	AAACCA	1192	6 +	Zea mays	anaerobic induction
TaMAPKKKK7	GC-motif	CCCCCG	247	6 +	Zea mays	anoxic specific inducibility

TaMAPKKKK7	GC-motif	CCCCCG	1362	6 +	Zea mays	anoxic specific inducibility
TaMAPKKKK7	CAT-box	GCCACT	366	6 -	Arabidopsis thaliana	meristem expression
TaMAPKKKK7	CAT-box	GCCACT	1440	6 -	Arabidopsis thaliana	meristem expression
TaMAPKKKK7	P-box	CCTTTTG	648	7 +	Oryza sativa	gibberellin responsive
TaMAPKKKK7	CGTCA-motif	CGTCA	753	5 -	Hordeum vulgare	the MeJA responsive
TaMAPKKKK7	CGTCA-motif	CGTCA	1472	5 +	Hordeum vulgare	the MeJA responsive
TaMAPKKKK7	CGTCA-motif	CGTCA	1496	5 +	Hordeum vulgare	the MeJA responsive
TaMAPKKKK7	TGA-element	AACGAC	565	6 -	Brassica oleracea	auxin responsive
TaMAPKKKK7	TCA-element	CCATCTTTTT	1573	9 -	Nicotiana tabacum	salicylic acid responsive
TaMAPKKKK8	TGACG-motif	TGACG	963	5 -	Hordeum vulgare	the MeJA responsive
TaMAPKKKK8	TGA-element	AACGAC	1355	6 +	Brassica oleracea	auxin responsive
TaMAPKKKK8	CGTCA-motif	CGTCA	963	5 +	Hordeum vulgare	the MeJA responsive
TaMAPKKKK8	ABRE	ACGTG	332	5 -	Arabidopsis thaliana	abscisic acid responsive
TaMAPKKKK8	ABRE	ACGTG	348	5 -	Arabidopsis thaliana	abscisic acid responsive
TaMAPKKKK8	ABRE	ACGTG	481	5 +	Arabidopsis thaliana	abscisic acid responsive
TaMAPKKKK8	ABRE	ACGTG	535	5 -	Arabidopsis thaliana	abscisic acid responsive
TaMAPKKKK8	ABRE	CACGTG	599	6 +	Arabidopsis thaliana	abscisic acid responsive
TaMAPKKKK8	ABRE	ACGTG	600	5 +	Arabidopsis thaliana	abscisic acid responsive
TaMAPKKKK8	ABRE	ACGTG	1103	5 -	Arabidopsis thaliana	abscisic acid responsive
TaMAPKKKK8	ABRE	ACGTG	1594	5 -	Arabidopsis thaliana	abscisic acid responsive

TaMAPKKKK8	TATC-box	TATCCCA	1468	7 -	Oryza sativa	gibberellin responsive
TaMAPKKKK9	MBSI	aaaAaaC(G/C)GTTA	865	10.5 +	Petunia hybrida	flavonoid biosynthetic genes regulation
TaMAPKKKK9	MBS	CAACTG	39	6 -	Arabidopsis thaliana	drought inducibility
TaMAPKKKK9	GARE-motif	TCTGTTG	1604	7 +	Brassica oleracea	gibberellin responsive
TaMAPKKKK9	O2-site	GTTGACGTGA	1248	9 +	Zea mays	zein metabolism regulation
TaMAPKKKK9	TGACG-motif	TGACG	564	5 -	Hordeum vulgare	the MeJA responsive
TaMAPKKKK9	TGACG-motif	TGACG	1250	5 +	Hordeum vulgare	the MeJA responsive
TaMAPKKKK9	TGACG-motif	TGACG	1302	5 +	Hordeum vulgare	the MeJA responsive
TaMAPKKKK9	ARE	AAACCA	43	6 -	Zea mays	anaerobic induction
TaMAPKKKK9	TATC-box	TATCCCA	1558	7 -	Oryza sativa	gibberellin responsive
TaMAPKKKK9	TCA-element	CCATCTTTTT	1738	9 +	Nicotiana tabacum	salicylic acid responsive
TaMAPKKKK9	TCA-element	CCATCTTTTT	1789	9 +	Nicotiana tabacum	salicylic acid responsive
TaMAPKKKK9	ABRE	ACGTG	729	5 +	Arabidopsis thaliana	abscisic acid responsive
TaMAPKKKK9	ABRE	ACGTG	1252	5 +	Arabidopsis thaliana	abscisic acid responsive
TaMAPKKKK9	ABRE	GACACGTACGT	1920	10 +	Oryza sativa	abscisic acid responsive
TaMAPKKKK9	CGTCA-motif	CGTCA	564	5 +	Hordeum vulgare	the MeJA responsive
TaMAPKKKK9	CGTCA-motif	CGTCA	1250	5 -	Hordeum vulgare	the MeJA responsive
TaMAPKKKK9	CGTCA-motif	CGTCA	1302	5 -	Hordeum vulgare	the MeJA responsive
