# Supplementary material for: Bioinformatic Identification and Expression Analyses of the MAPK–MAP4K Gene Family Reveal a Putative Functional MAP4K10-MAP3K7/8-MAP2K1/11-MAPK3/6 Cascade in Wheat (Triticum aestivum L.)
Source: Plants (Basel). 2024 Mar 24;13(7):941. doi: 10.3390/plants13070941 (PMC11013086; doi:10.3390/plants13070941)
Supplement: Supplementary file 1 [file plants-13-00941-s001.zip › plants-2867660-supplementary/Supplementary Figure S1-S10 and Supplementary table S1-S7/Supplementary table S1-S7/Supplementary table 5.pdf]

Table S4 Parameters for MAPK-MAP4K protein interaction analysis in wheat			
degree.layo	name	selected	shared name
75	TaMAPKK11	FALSE	TaMAPKK11
74	TaMAPKK16	FALSE	TaMAPKK16
73	TaMAPKK15	FALSE	TaMAPKK15
73	TaMAPKK14	FALSE	TaMAPKK14
72	TaMAPKK5	FALSE	TaMAPKK5
71	TaMAPKK12	FALSE	TaMAPKK12
70	TaMAPKK8	FALSE	TaMAPKK8
69	TaMAPKK9	FALSE	TaMAPKK9
67	TaMAPKK17	FALSE	TaMAPKK17
67	TaMAPKK7	FALSE	TaMAPKK7
66	TaMAPKK6	FALSE	TaMAPKK6
61	TaMAPKK1	FALSE	TaMAPKK1
58	TaMEKK29	FALSE	TaMEKK29
58	TaMEKK17	FALSE	TaMEKK17
55	TaMEKK24	FALSE	TaMEKK24
54	TaMAPKK18	FALSE	TaMAPKK18
54	TaMAPKK2	FALSE	TaMAPKK2
54	TaMAPKK3	FALSE	TaMAPKK3
53	TaMAPKK4	FALSE	TaMAPKK4
53	TaMAPKK13	FALSE	TaMAPKK13
39	TaMEKK16	FALSE	TaMEKK16
39	TaRaf87	FALSE	TaRaf87
38	TaMEKK5	FALSE	TaMEKK5
38	TaMEKK14	FALSE	TaMEKK14
38	TaMEKK15	FALSE	TaMEKK15
38	TaMEKK11	FALSE	TaMEKK11
37	TaMEKK2	FALSE	TaMEKK2
35	TaMEKK1	FALSE	TaMEKK1
34	TaMEKK4	FALSE	TaMEKK4
34	TaMEKK4-1	FALSE	TaMEKK4-1
33	TaMAPK47	FALSE	TaMAPK47
33	TaMAPK25	FALSE	TaMAPK25
33	TaMAPK3	FALSE	TaMAPK3
33	TaMAPKKKK4	FALSE	TaMAPKKKK4
33	TaMAPKKKK5	FALSE	TaMAPKKKK5
33	TaMAPKKKK6	FALSE	TaMAPKKKK6
32	TaMAPK12	FALSE	TaMAPK12
31	TaMAPK35	FALSE	TaMAPK35
30	TaMAPK17	FALSE	TaMAPK17
30	TaMAPK34	FALSE	TaMAPK34
30	TaMAPK44	FALSE	TaMAPK44
29	TaMAPK19	FALSE	TaMAPK19
29	TaMAPK6	FALSE	TaMAPK6
29	TaMAPK20	FALSE	TaMAPK20
29	TaMAPK29	FALSE	TaMAPK29
29	TaMAPK33	FALSE	TaMAPK33
29	TaMAPK39	FALSE	TaMAPK39
29	TaMAPK40	FALSE	TaMAPK40
29	TaMAPK41	FALSE	TaMAPK41
29	TaMAPK49	FALSE	TaMAPK49
29	TaMAPK53	FALSE	TaMAPK53
28	TaMAPK10	FALSE	TaMAPK10
28	TaMAPK14	FALSE	TaMAPK14
28	TaMAPK28	FALSE	TaMAPK28

28	TaMAPK24	FALSE	TaMAPK24
28	TaMAPK43	FALSE	TaMAPK43
28	TaMAPKKKK10	FALSE	TaMAPKKKK10
28	TaMAPKKKK12	FALSE	TaMAPKKKK12
28	TaMAPKKKK14	FALSE	TaMAPKKKK14
27	TaMAPK22	FALSE	TaMAPK22
27	TaMAPK54	FALSE	TaMAPK54
27	TaMAPK11	FALSE	TaMAPK11
27	TaMAPK16	FALSE	TaMAPK16
27	TaRaf30	FALSE	TaRaf30
27	TaMAPK23	FALSE	TaMAPK23
27	TaMAPK27	FALSE	TaMAPK27
27	TaMAPK30	FALSE	TaMAPK30
27	TaMAPK38	FALSE	TaMAPK38
27	TaMAPK4	FALSE	TaMAPK4
27	TaMAPK42	FALSE	TaMAPK42
27	TaMAPK5	FALSE	TaMAPK5
27	TaMAPK52	FALSE	TaMAPK52
27	TaMAPK7	FALSE	TaMAPK7
27	TaMAPK8	FALSE	TaMAPK8
25	TaRaf88	FALSE	TaRaf88
24	TaMEKK9	FALSE	TaMEKK9
23	TaMAPK36	FALSE	TaMAPK36
23	TaMEKK7	FALSE	TaMEKK7
23	TaMEKK20	FALSE	TaMEKK20
23	TaMEKK18	FALSE	TaMEKK18
23	TaMEKK8	FALSE	TaMEKK8
22	TaMAPK45	FALSE	TaMAPK45
22	TaMAPKKKK16	FALSE	TaMAPKKKK16
19	TaZIK1	FALSE	TaZIK1
19	TaZIK11	FALSE	TaZIK11
19	TaZIK8	FALSE	TaZIK8
19	TaZIK5	FALSE	TaZIK5
19	TaZIK10	FALSE	TaZIK10
19	TaZIK2	FALSE	TaZIK2
19	TaZIK9	FALSE	TaZIK9
19	TaZIK7	FALSE	TaZIK7
19	TaZIK4	FALSE	TaZIK4
19	TaZIK3	FALSE	TaZIK3
18	TaRaf91	FALSE	TaRaf91
18	TaRaf62	FALSE	TaRaf62
18	TaMEKK12	FALSE	TaMEKK12
18	TaMEKK3	FALSE	TaMEKK3
18	TaMEKK10	FALSE	TaMEKK10
17	TaRaf18	FALSE	TaRaf18
17	TaRaf60	FALSE	TaRaf60
17	TaRaf5	FALSE	TaRaf5
16	TaRaf41	FALSE	TaRaf41
15	TaRaf46	FALSE	TaRaf46
13	TaRaf7	FALSE	TaRaf7
12	TaRaf56	FALSE	TaRaf56
11	TaMAPKKKK1	FALSE	TaMAPKKKK1
11	TaMAPKKKK11	FALSE	TaMAPKKKK11
11	TaMAPKKKK13	FALSE	TaMAPKKKK13
11	TaMAPKKKK15	FALSE	TaMAPKKKK15
11	TaMAPKKKK2	FALSE	TaMAPKKKK2

11	TaMAPKKKK3	FALSE	TaMAPKKKK3
11	TaMAPKKKK7	FALSE	TaMAPKKKK7
11	TaMAPKKKK8	FALSE	TaMAPKKKK8
11	TaMAPKKKK9	FALSE	TaMAPKKKK9
11	TaMEKK25	FALSE	TaMEKK25
11	TaMAPKKKK20	FALSE	TaMAPKKKK20
11	TaMAPKKKK19	FALSE	TaMAPKKKK19
11	TaMAPKKKK17	FALSE	TaMAPKKKK17
11	TaMAPKKKK23	FALSE	TaMAPKKKK23
11	TaMAPKKKK24	FALSE	TaMAPKKKK24
11	TaMAPKKKK22	FALSE	TaMAPKKKK22
11	TaMAPKKKK25	FALSE	TaMAPKKKK25
11	TaMAPKKKK18	FALSE	TaMAPKKKK18
11	TaMAPKKKK21	FALSE	TaMAPKKKK21
10	TaRaf21	FALSE	TaRaf21
10	TaRaf95	FALSE	TaRaf95
10	TaRaf1	FALSE	TaRaf1
9	TaRaf59	FALSE	TaRaf59
9	TaRaf52	FALSE	TaRaf52
7	TaRaf111	FALSE	TaRaf111
7	TaRaf102	FALSE	TaRaf102
7	TaRaf42	FALSE	TaRaf42
7	TaRaf50	FALSE	TaRaf50
7	TaRaf43	FALSE	TaRaf43
7	TaRaf29	FALSE	TaRaf29
7	TaRaf79	FALSE	TaRaf79
7	TaRaf58	FALSE	TaRaf58
6	TaRaf105	FALSE	TaRaf105
6	TaRaf100	FALSE	TaRaf100
6	TaRaf63	FALSE	TaRaf63
5	TaRaf19	FALSE	TaRaf19
4	TaRaf73	FALSE	TaRaf73
3	TaRaf45	FALSE	TaRaf45
3	TaRaf72	FALSE	TaRaf72
3	TaRaf22	FALSE	TaRaf22
3	TaRaf81	FALSE	TaRaf81
3	TaRaf94	FALSE	TaRaf94
3	TaRaf89	FALSE	TaRaf89
2	TaRaf14	FALSE	TaRaf14
2	TaRaf83	FALSE	TaRaf83
2	TaRaf44	FALSE	TaRaf44
1	TaRaf113	FALSE	TaRaf113
1	TaRaf71	FALSE	TaRaf71

Table S4 Parameters for MAPK-MAP4K protein interaction analysis in wheat												
**node1**	**node2**	**node1_string_id**	**node2_string_id**	**neighborhood_on_chromosome**	**gene_fusion**	**phylogenetic_cooccurrence**	**homology**	**coexpression**	**experimentally_determined_interaction**	**database_annotated**	**automated_textmining**	**combined_score**
TaMAPK10	TaMAPKK9	4565.A0A3B6PGY7	4565.A0A077RVQ4	0	0	0.108	0.667	0	0.705	0.583	0.146	0.893
TaMAPK10	TaMEKK5	4565.A0A3B6PGY7	4565.A0A3B6AWC1	0	0	0.124	0.636	0	0.222	0.186	0.065	0.412
TaMAPK10	TaMAPKK15	4565.A0A3B6PGY7	4565.A0A3B6HW51	0	0	0.112	0.66	0	0.705	0.583	0.146	0.894
TaMAPK10	TaMAPKK14	4565.A0A3B6PGY7	4565.A0A3B6HY95	0	0	0.117	0.652	0	0.705	0.583	0.146	0.894
TaMAPK10	TaMAPKK16	4565.A0A3B6PGY7	4565.A0A3B6HZP7	0	0	0.115	0.666	0	0.705	0.583	0.146	0.894
TaMAPK10	TaMAPKK17	4565.A0A3B6PGY7	4565.A0A3B6I0M7	0	0	0.117	0.636	0	0.705	0.583	0.146	0.894
TaMAPK10	TaMAPKK8	4565.A0A3B6PGY7	4565.A0A3B6IK39	0	0	0.112	0.656	0	0.705	0.583	0.146	0.894
TaMAPK10	TaMAPKK6	4565.A0A3B6PGY7	4565.A0A3B6ILF0	0	0	0.114	0.635	0	0.705	0.583	0.146	0.894
TaMAPK10	TaMAPKK5	4565.A0A3B6PGY7	4565.A0A3B6IMW7	0	0	0.117	0.643	0	0.705	0.583	0.146	0.894
TaMAPK10	TaMAPKK7	4565.A0A3B6PGY7	4565.A0A3B6INV0	0	0	0.118	0.635	0	0.705	0.583	0.146	0.894
TaMAPK10	TaMEKK2	4565.A0A3B6PGY7	4565.A0A3B6JCC4	0	0	0.111	0.608	0	0.222	0.186	0.065	0.403
TaMAPK10	TaMAPKK11	4565.A0A3B6PGY7	4565.A0A3B6JEH0	0	0	0.119	0.656	0	0.705	0.583	0.146	0.895
TaMAPK10	TaMAPKK12	4565.A0A3B6PGY7	4565.A0A3B6JG06	0	0	0.119	0.63	0	0.705	0.583	0.146	0.895
TaMAPK10	TaMEKK14	4565.A0A3B6PGY7	4565.A0A3B6KF43	0	0	0.123	0.622	0	0.222	0.186	0.065	0.411
TaMAPK10	TaMAPKK4	4565.A0A3B6PGY7	4565.A0A3B6KFB5	0	0	0	0.613	0	0.705	0.825	0.195	0.954
TaMAPK10	TaMEKK15	4565.A0A3B6PGY7	4565.A0A3B6KPK7	0	0	0.112	0.634	0	0.222	0.186	0.065	0.403

TaMAPK10	TaMAPKK18	4565.A0A3B6PGY7	4565.A0A3B6LJ27	0	0	0	0.596	0	0.705	0.825	0.195	0.954
TaMAPK10	TaMEKK16	4565.A0A3B6PGY7	4565.A0A3B6LW00	0	0	0.11	0.634	0	0.222	0.186	0.065	0.402
TaMAPK10	TaMAPKK2	4565.A0A3B6PGY7	4565.A0A3B6LYW0	0	0	0	0.606	0	0.705	0.825	0.195	0.954
TaMAPK10	TaMAPKK3	4565.A0A3B6PGY7	4565.A0A3B6MNP8	0	0	0	0.595	0	0.705	0.825	0.195	0.954
TaMAPK10	TaMEKK11	4565.A0A3B6PGY7	4565.A0A3B6N0D8	0	0	0.11	0.633	0	0.222	0.186	0.065	0.402
TaMAPK10	TaMAPKK13	4565.A0A3B6PGY7	4565.A0A3B6N2X8	0	0	0	0.603	0	0.705	0.825	0.195	0.954
TaMAPK10	TaRaf87	4565.A0A3B6PGY7	4565.A0A3B6QMZ9	0	0	0	0.584	0.16	0.134	0.389	0.088	0.54
TaMAPK10	TaMAPK22	4565.A0A3B6PGY7	4565.A0A3B6TPT1	0	0	0.048	0.984	0	0	0.793	0	0.794
TaMAPK10	TaMAPK12	4565.A0A3B6PGY7	4565.A0A3B6RP80	0	0	0.048	0.985	0	0	0.793	0	0.794
TaMAPK10	TaMAPK54	4565.A0A3B6PGY7	4565.A0A3B6SKC9	0	0	0.048	0.985	0	0	0.793	0	0.794
TaMAPK10	TaMAPK47	4565.A0A3B6PGY7	4565.A0A3B6RAZ7	0	0	0.053	0.975	0	0	0.793	0	0.795
TaMAPK10	TaMAPKK1	4565.A0A3B6PGY7	4565.A0A3B6QJ87	0	0	0.131	0.609	0	0.705	0.583	0.146	0.896
TaMAPK11	TaMAPKK9	4565.A0A3B5XVG6	4565.A0A077RVQ4	0	0	0.126	0.623	0	0.705	0.583	0.146	0.896
TaMAPK11	TaMEKK24	4565.A0A3B5XVG6	4565.A0A3B6LLV5	0	0	0.177	0.59	0	0.27	0.063	0.088	0.417
TaMAPK11	TaMEKK29	4565.A0A3B5XVG6	4565.A0A3B6MSP6	0	0	0.177	0.59	0	0.27	0.063	0.088	0.418
TaMAPK11	TaMEKK17	4565.A0A3B5XVG6	4565.A0A3B6KFL8	0	0	0.177	0.59	0	0.27	0.063	0.088	0.418
TaMAPK11	TaMEKK2	4565.A0A3B5XVG6	4565.A0A3B6JCC4	0	0	0.137	0.568	0	0.222	0.186	0.065	0.42
TaMAPK11	TaMEKK11	4565.A0A3B5XVG6	4565.A0A3B6N0D8	0	0	0.149	0.57	0	0.222	0.186	0.065	0.428

TaMAPK11	TaMEKK15	4565.A0A3B5XVG6	4565.A0A3B6KPK7	0	0	0.152	0.573	0	0.222	0.186	0.065	0.43
TaMAPK11	TaMEKK16	4565.A0A3B5XVG6	4565.A0A3B6LW00	0	0	0.15	0.572	0	0.222	0.186	0.065	0.43
TaMAPK11	TaMEKK14	4565.A0A3B5XVG6	4565.A0A3B6KF43	0	0	0.153	0.58	0	0.222	0.186	0.065	0.431
TaMAPK11	TaMEKK5	4565.A0A3B5XVG6	4565.A0A3B6AWC1	0	0	0.159	0.574	0	0.222	0.186	0.065	0.435
TaMAPK11	TaRaf87	4565.A0A3B5XVG6	4565.A0A3B6QMZ9	0	0	0	0.565	0.3	0.134	0.389	0.088	0.617
TaMAPK11	TaMAPKK8	4565.A0A3B5XVG6	4565.A0A3B6IK39	0	0	0.123	0.616	0	0.705	0.583	0.146	0.895
TaMAPK11	TaMAPKK15	4565.A0A3B5XVG6	4565.A0A3B6HW51	0	0	0.134	0.615	0	0.705	0.583	0.146	0.896
TaMAPK11	TaMAPKK16	4565.A0A3B5XVG6	4565.A0A3B6HZP7	0	0	0.132	0.621	0	0.705	0.583	0.146	0.896
TaMAPK11	TaMAPKK5	4565.A0A3B5XVG6	4565.A0A3B6IMW7	0	0	0.128	0.609	0	0.705	0.583	0.146	0.896
TaMAPK11	TaMAPKK14	4565.A0A3B5XVG6	4565.A0A3B6HY95	0	0	0.133	0.611	0	0.705	0.583	0.146	0.896
TaMAPK11	TaMAPKK11	4565.A0A3B5XVG6	4565.A0A3B6JEH0	0	0	0.127	0.621	0	0.705	0.583	0.146	0.896
TaMAPK11	TaMAPKK7	4565.A0A3B5XVG6	4565.A0A3B6INV0	0	0	0.139	0.611	0	0.705	0.583	0.146	0.897
TaMAPK11	TaMAPKK17	4565.A0A3B5XVG6	4565.A0A3B6I0M7	0	0	0.137	0.613	0	0.705	0.583	0.146	0.897
TaMAPK11	TaMAPKK6	4565.A0A3B5XVG6	4565.A0A3B6ILF0	0	0	0.14	0.607	0	0.705	0.583	0.146	0.897
TaMAPK11	TaMAPKK1	4565.A0A3B5XVG6	4565.A0A3B6QJ87	0	0	0.136	0.615	0	0.705	0.583	0.146	0.897
TaMAPK11	TaMAPKK12	4565.A0A3B5XVG6	4565.A0A3B6JG06	0	0	0.146	0.608	0	0.705	0.583	0.146	0.898
TaMAPK11	TaMAPKK13	4565.A0A3B5XVG6	4565.A0A3B6N2X8	0	0	0.108	0.58	0	0.705	0.612	0.146	0.901
TaMAPK11	TaMAPKK2	4565.A0A3B5XVG6	4565.A0A3B6LYW0	0	0	0.123	0.581	0	0.705	0.612	0.146	0.902

TaMAPK11	TaMAPKK3	4565.A0A3B5XVG6	4565.A0A3B6MNP8	0	0	0.131	0.577	0	0.705	0.612	0.146	0.903
TaMAPK11	TaMAPKK4	4565.A0A3B5XVG6	4565.A0A3B6KFB5	0	0	0.13	0.63	0	0.705	0.612	0.146	0.903
TaMAPK11	TaMAPKK18	4565.A0A3B5XVG6	4565.A0A3B6LJ27	0	0	0.137	0.583	0	0.705	0.612	0.146	0.904
TaMAPK12	TaMAPKK9	4565.A0A3B6RP80	4565.A0A077RVQ4	0	0	0.123	0.65	0	0.705	0.583	0.146	0.895
TaMAPK12	TaMEKK5	4565.A0A3B6RP80	4565.A0A3B6AWC1	0	0	0.132	0.623	0	0.222	0.186	0.065	0.417
TaMAPK12	TaMEKK1	4565.A0A3B6RP80	4565.A0A3B6B3I4	0	0	0.152	0.613	0	0.27	0.063	0.088	0.4
TaMAPK12	TaMAPKK15	4565.A0A3B6RP80	4565.A0A3B6HW51	0	0	0.127	0.643	0	0.705	0.583	0.146	0.896
TaMAPK12	TaMAPKK14	4565.A0A3B6RP80	4565.A0A3B6HY95	0	0	0.132	0.633	0	0.705	0.583	0.146	0.896
TaMAPK12	TaMAPKK16	4565.A0A3B6RP80	4565.A0A3B6HZP7	0	0	0.129	0.648	0	0.705	0.583	0.146	0.896
TaMAPK12	TaMAPKK17	4565.A0A3B6RP80	4565.A0A3B6I0M7	0	0	0.131	0.623	0	0.705	0.583	0.146	0.896
TaMAPK12	TaMAPKK8	4565.A0A3B6RP80	4565.A0A3B6IK39	0	0	0.125	0.638	0	0.705	0.583	0.146	0.895
TaMAPK12	TaMAPKK6	4565.A0A3B6RP80	4565.A0A3B6ILF0	0	0	0.129	0.625	0	0.705	0.583	0.146	0.896
TaMAPK12	TaMAPKK5	4565.A0A3B6RP80	4565.A0A3B6IMW7	0	0	0.131	0.626	0	0.705	0.583	0.146	0.896
TaMAPK12	TaMAPKK7	4565.A0A3B6RP80	4565.A0A3B6INV0	0	0	0.13	0.622	0	0.705	0.583	0.146	0.896
TaMAPK12	TaMEKK2	4565.A0A3B6RP80	4565.A0A3B6JCC4	0	0	0.115	0.604	0	0.222	0.186	0.065	0.406
TaMAPK12	TaMAPKK11	4565.A0A3B6RP80	4565.A0A3B6JEH0	0	0	0.133	0.641	0	0.705	0.583	0.146	0.896
TaMAPK12	TaMAPKK12	4565.A0A3B6RP80	4565.A0A3B6JG06	0	0	0.133	0.615	0	0.705	0.583	0.146	0.896
TaMAPK12	TaMEKK14	4565.A0A3B6RP80	4565.A0A3B6KF43	0	0	0.131	0.611	0	0.222	0.186	0.065	0.416

TaMAPK12	TaMAPKK 4	4565.A0A3 B6RP80	4565.A0A3 B6KFB5	0	0	0.104	0.611	0	0.705	0.739	0.195	0.937
TaMAPK12	TaMEKK1 7	4565.A0A3 B6RP80	4565.A0A3 B6KFL8	0	0	0.153	0.619	0	0.27	0.063	0.088	0.4
TaMAPK12	TaMEKK1 5	4565.A0A3 B6RP80	4565.A0A3 B6KPK7	0	0	0.122	0.617	0	0.222	0.186	0.065	0.41
TaMAPK12	TaMAPKK 18	4565.A0A3 B6RP80	4565.A0A3 B6LJ27	0	0	0	0.582	0	0.705	0.739	0.195	0.932
TaMAPK12	TaMEKK1 6	4565.A0A3 B6RP80	4565.A0A3 B6LW00	0	0	0.12	0.616	0	0.222	0.186	0.065	0.409
TaMAPK12	TaMAPKK 2	4565.A0A3 B6RP80	4565.A0A3 B6LYW0	0	0	0	0.593	0	0.705	0.886	0.195	0.97
TaMAPK12	TaMAPKK 3	4565.A0A3 B6RP80	4565.A0A3 B6MNP8	0	0	0	0.581	0	0.705	0.739	0.195	0.932
TaMAPK12	TaMEKK2 9	4565.A0A3 B6RP80	4565.A0A3 B6MSP6	0	0	0.153	0.62	0	0.27	0.063	0.088	0.401
TaMAPK12	TaMEKK1 1	4565.A0A3 B6RP80	4565.A0A3 B6N0D8	0	0	0.12	0.616	0	0.222	0.186	0.065	0.409
TaMAPK12	TaMAPKK 13	4565.A0A3 B6RP80	4565.A0A3 B6N2X8	0	0	0	0.588	0	0.705	0.886	0.195	0.97
TaMAPK12	TaMAPK3 6	4565.A0A3 B6RP80	4565.A0A3 B6NN33	0	0	0.048	0.984	0	0	0.793	0	0.794
TaMAPK12	TaMEKK4	4565.A0A3 B6RP80	4565.A0A3 B6NRN9	0	0	0.155	0.608	0	0.27	0.063	0.088	0.402
TaMAPK12	TaMEKK4-1	4565.A0A3 B6RP80	4565.A0A3 B6PNI6	0	0	0.154	0.61	0	0.27	0.063	0.088	0.402
TaMAPK12	TaMAPK4 5	4565.A0A3 B6RP80	4565.A0A3 B6QBD2	0	0	0.049	0.983	0	0	0.793	0	0.794
TaMAPK12	TaMAPKK 1	4565.A0A3 B6RP80	4565.A0A3 B6QJ87	0	0	0.142	0.611	0	0.705	0.583	0.146	0.897
TaMAPK12	TaRaf87	4565.A0A3 B6RP80	4565.A0A3 B6QMZ9	0	0	0	0.573	0.16	0.134	0.389	0.088	0.54
TaMAPK14	TaMAPKK 9	4565.A0A3 B5Y080	4565.A0A0 77RVQ4	0	0	0.137	0.61	0	0.705	0.583	0.146	0.897
TaMAPK14	TaMEKK1 7	4565.A0A3 B5Y080	4565.A0A3 B6KFL8	0	0	0.152	0.64	0	0.27	0.063	0.088	0.4

TaMAPK14	TaMEKK29	4565.A0A3B5Y080	4565.A0A3B6MSP6	0	0	0.152	0.64	0	0.27	0.063	0.088	0.4
TaMAPK14	TaMEKK2	4565.A0A3B5Y080	4565.A0A3B6JCC4	0	0	0.108	0.616	0	0.222	0.186	0.065	0.401
TaMAPK14	TaMEKK16	4565.A0A3B5Y080	4565.A0A3B6LW00	0	0	0.113	0.622	0	0.222	0.186	0.065	0.404
TaMAPK14	TaMEKK11	4565.A0A3B5Y080	4565.A0A3B6N0D8	0	0	0.113	0.621	0	0.222	0.186	0.065	0.404
TaMAPK14	TaMEKK15	4565.A0A3B5Y080	4565.A0A3B6KPK7	0	0	0.113	0.622	0	0.222	0.186	0.065	0.405
TaMAPK14	TaMEKK5	4565.A0A3B5Y080	4565.A0A3B6AWC1	0	0	0.12	0.634	0	0.222	0.186	0.065	0.409
TaMAPK14	TaMEKK14	4565.A0A3B5Y080	4565.A0A3B6KF43	0	0	0.12	0.628	0	0.222	0.186	0.065	0.409
TaMAPK14	TaRaf87	4565.A0A3B5Y080	4565.A0A3B6QMZ9	0	0	0	0.604	0.16	0.134	0.389	0.088	0.54
TaMAPK14	TaMAPK19	4565.A0A3B5Y080	4565.A0A3B6RDB9	0	0	0.054	0.973	0	0.139	0.841	0	0.859
TaMAPK14	TaMAPK6	4565.A0A3B5Y080	4565.A0A3B6SCW0	0	0	0.054	0.972	0	0.139	0.841	0	0.859
TaMAPK14	TaMAPKK13	4565.A0A3B5Y080	4565.A0A3B6N2X8	0	0	0	0.61	0	0.705	0.583	0.146	0.885
TaMAPK14	TaMAPKK2	4565.A0A3B5Y080	4565.A0A3B6LYW0	0	0	0.111	0.61	0	0.705	0.583	0.146	0.894
TaMAPK14	TaMAPKK3	4565.A0A3B5Y080	4565.A0A3B6MNP8	0	0	0.111	0.606	0	0.705	0.583	0.146	0.894
TaMAPK14	TaMAPKK4	4565.A0A3B5Y080	4565.A0A3B6KFB5	0	0	0.125	0.629	0	0.705	0.583	0.146	0.895
TaMAPK14	TaMAPKK18	4565.A0A3B5Y080	4565.A0A3B6LJ27	0	0	0.119	0.604	0	0.705	0.583	0.146	0.895
TaMAPK14	TaMAPKK1	4565.A0A3B5Y080	4565.A0A3B6QJ87	0	0	0.141	0.617	0	0.705	0.583	0.146	0.897
TaMAPK14	TaMAPKK14	4565.A0A3B5Y080	4565.A0A3B6HY95	0	0	0.143	0.607	0	0.705	0.583	0.146	0.897
TaMAPK14	TaMAPKK11	4565.A0A3B5Y080	4565.A0A3B6JEH0	0	0	0.139	0.618	0	0.705	0.583	0.146	0.897

TaMAPK14	TaMAPKK12	4565.A0A3B5Y080	4565.A0A3B6JG06	0	0	0.139	0.615	0	0.705	0.583	0.146	0.897
TaMAPK14	TaMAPKK7	4565.A0A3B5Y080	4565.A0A3B6INV0	0	0	0.138	0.615	0	0.705	0.583	0.146	0.897
TaMAPK14	TaMAPKK15	4565.A0A3B5Y080	4565.A0A3B6HW51	0	0	0.14	0.612	0	0.705	0.583	0.146	0.897
TaMAPK14	TaMAPKK6	4565.A0A3B5Y080	4565.A0A3B6ILF0	0	0	0.135	0.613	0	0.705	0.583	0.146	0.897
TaMAPK14	TaMAPKK8	4565.A0A3B5Y080	4565.A0A3B6IK39	0	0	0.137	0.607	0	0.705	0.583	0.146	0.897
TaMAPK14	TaMAPKK5	4565.A0A3B5Y080	4565.A0A3B6IMW7	0	0	0.141	0.6	0	0.705	0.583	0.146	0.897
TaMAPK14	TaMAPKK17	4565.A0A3B5Y080	4565.A0A3B6I0M7	0	0	0.136	0.617	0	0.705	0.583	0.146	0.897
TaMAPK14	TaMAPKK16	4565.A0A3B5Y080	4565.A0A3B6HZP7	0	0	0.147	0.608	0	0.705	0.583	0.146	0.898
TaMAPK16	TaMEKK2	4565.A0A077RTL3	4565.A0A3B6JCC4	0	0	0.127	0.578	0	0.222	0.186	0.065	0.414
TaMAPK16	TaMEKK16	4565.A0A077RTL3	4565.A0A3B6LW00	0	0	0.133	0.574	0	0.222	0.186	0.065	0.417
TaMAPK16	TaMEKK11	4565.A0A077RTL3	4565.A0A3B6N0D8	0	0	0.132	0.572	0	0.222	0.186	0.065	0.417
TaMAPK16	TaMEKK15	4565.A0A077RTL3	4565.A0A3B6KPK7	0	0	0.134	0.575	0	0.222	0.186	0.065	0.418
TaMAPK16	TaMEKK5	4565.A0A077RTL3	4565.A0A3B6AWC1	0	0	0.144	0.579	0	0.222	0.186	0.065	0.425
TaMAPK16	TaMEKK17	4565.A0A077RTL3	4565.A0A3B6KFL8	0	0	0.189	0.574	0	0.27	0.063	0.088	0.426
TaMAPK16	TaMEKK24	4565.A0A077RTL3	4565.A0A3B6LLV5	0	0	0.189	0.574	0	0.27	0.063	0.088	0.426
TaMAPK16	TaMEKK14	4565.A0A077RTL3	4565.A0A3B6KF43	0	0	0.145	0.586	0	0.222	0.186	0.065	0.426
TaMAPK16	TaMEKK29	4565.A0A077RTL3	4565.A0A3B6MSP6	0	0	0.189	0.574	0	0.27	0.063	0.088	0.426
TaMAPK16	TaRaf87	4565.A0A077RTL3	4565.A0A3B6QMZ9	0	0	0	0.564	0.277	0.134	0.389	0.088	0.604

TaMAPK16	TaMAPKK 4	4565.A0A077RTL3	4565.A0A3B6KFB5	0	0	0	0.639	0	0.705	0.583	0.146	0.885
TaMAPK16	TaMAPKK 18	4565.A0A077RTL3	4565.A0A3B6LJ27	0	0	0	0.586	0	0.705	0.583	0.146	0.885
TaMAPK16	TaMAPKK 13	4565.A0A077RTL3	4565.A0A3B6N2X8	0	0	0	0.583	0	0.705	0.583	0.146	0.885
TaMAPK16	TaMAPKK 2	4565.A0A077RTL3	4565.A0A3B6LYW0	0	0	0	0.584	0	0.705	0.583	0.146	0.885
TaMAPK16	TaMAPKK 3	4565.A0A077RTL3	4565.A0A3B6MNP8	0	0	0	0.581	0	0.705	0.583	0.146	0.885
TaMAPK16	TaMAPKK 14	4565.A0A077RTL3	4565.A0A3B6HY95	0	0	0.116	0.619	0	0.705	0.583	0.146	0.894
TaMAPK16	TaMAPKK 9	4565.A0A077RTL3	4565.A0A077RVQ4	0	0	0.115	0.622	0	0.705	0.583	0.146	0.894
TaMAPK16	TaMAPKK 8	4565.A0A077RTL3	4565.A0A3B6IK39	0	0	0.113	0.621	0	0.705	0.583	0.146	0.894
TaMAPK16	TaMAPKK 16	4565.A0A077RTL3	4565.A0A3B6HZP7	0	0	0.117	0.621	0	0.705	0.583	0.146	0.894
TaMAPK16	TaMAPKK 5	4565.A0A077RTL3	4565.A0A3B6IMW7	0	0	0.115	0.614	0	0.705	0.583	0.146	0.894
TaMAPK16	TaMAPKK 11	4565.A0A077RTL3	4565.A0A3B6JEH0	0	0	0.118	0.618	0	0.705	0.583	0.146	0.895
TaMAPK16	TaMAPKK 12	4565.A0A077RTL3	4565.A0A3B6JG06	0	0	0.125	0.61	0	0.705	0.583	0.146	0.895
TaMAPK16	TaMAPKK 7	4565.A0A077RTL3	4565.A0A3B6INV0	0	0	0.123	0.613	0	0.705	0.583	0.146	0.895
TaMAPK16	TaMAPKK 15	4565.A0A077RTL3	4565.A0A3B6HW51	0	0	0.118	0.621	0	0.705	0.583	0.146	0.895
TaMAPK16	TaMAPKK 6	4565.A0A077RTL3	4565.A0A3B6ILF0	0	0	0.122	0.611	0	0.705	0.583	0.146	0.895
TaMAPK16	TaMAPKK 17	4565.A0A077RTL3	4565.A0A3B6I0M7	0	0	0.121	0.615	0	0.705	0.583	0.146	0.895
TaMAPK16	TaMAPKK 1	4565.A0A077RTL3	4565.A0A3B6QJ87	0	0	0.128	0.619	0	0.705	0.583	0.146	0.896
TaMAPK17	TaMAPKK 9	4565.A0A3B6PHX1	4565.A0A077RVQ4	0	0	0.151	0.614	0	0.705	0.583	0.146	0.898

TaMAPK17	TaMEKK5	4565.A0A3 B6PHX1	4565.A0A3 B6AWC1	0	0	0.17	0.58	0	0.222	0.186	0.065	0.443
TaMAPK17	TaMEKK1	4565.A0A3 B6PHX1	4565.A0A3 B6B3I4	0	0	0.16	0.615	0	0.27	0.063	0.088	0.406
TaMAPK17	TaMAPKK 15	4565.A0A3 B6PHX1	4565.A0A3 B6HW51	0	0	0.157	0.608	0	0.705	0.583	0.146	0.899
TaMAPK17	TaMAPKK 14	4565.A0A3 B6PHX1	4565.A0A3 B6HY95	0	0	0.156	0.604	0	0.705	0.583	0.146	0.899
TaMAPK17	TaMAPKK 16	4565.A0A3 B6PHX1	4565.A0A3 B6HZP7	0	0	0.152	0.618	0	0.705	0.583	0.146	0.899
TaMAPK17	TaMAPKK 17	4565.A0A3 B6PHX1	4565.A0A3 B6I0M7	0	0	0.15	0.609	0	0.705	0.583	0.146	0.898
TaMAPK17	TaMAPKK 8	4565.A0A3 B6PHX1	4565.A0A3 B6IK39	0	0	0.146	0.609	0	0.705	0.583	0.146	0.898
TaMAPK17	TaMAPKK 6	4565.A0A3 B6PHX1	4565.A0A3 B6ILF0	0	0	0.149	0.606	0	0.705	0.583	0.146	0.898
TaMAPK17	TaMAPKK 5	4565.A0A3 B6PHX1	4565.A0A3 B6IMW7	0	0	0.151	0.601	0	0.705	0.583	0.146	0.898
TaMAPK17	TaMAPKK 7	4565.A0A3 B6PHX1	4565.A0A3 B6INV0	0	0	0.153	0.607	0	0.705	0.583	0.146	0.899
TaMAPK17	TaMEKK2	4565.A0A3 B6PHX1	4565.A0A3 B6JCC4	0	0	0.153	0.579	0	0.222	0.186	0.065	0.431
TaMAPK17	TaMAPKK 11	4565.A0A3 B6PHX1	4565.A0A3 B6JEH0	0	0	0.15	0.613	0	0.705	0.583	0.146	0.898
TaMAPK17	TaMAPKK 12	4565.A0A3 B6PHX1	4565.A0A3 B6JG06	0	0	0.154	0.605	0	0.705	0.583	0.146	0.899
TaMAPK17	TaMEKK1 4	4565.A0A3 B6PHX1	4565.A0A3 B6KF43	0	0	0.17	0.588	0	0.222	0.186	0.065	0.443
TaMAPK17	TaMAPKK 4	4565.A0A3 B6PHX1	4565.A0A3 B6KFB5	0	0	0.119	0.638	0	0.705	0.612	0.146	0.902
TaMAPK17	TaMEKK1 7	4565.A0A3 B6PHX1	4565.A0A3 B6KFL8	0	0	0.18	0.607	0	0.27	0.063	0.088	0.42
TaMAPK17	TaMEKK1 5	4565.A0A3 B6PHX1	4565.A0A3 B6KPK7	0	0	0.165	0.582	0	0.222	0.186	0.065	0.439
TaMAPK17	TaMAPKK 18	4565.A0A3 B6PHX1	4565.A0A3 B6LJ27	0	0	0.119	0.585	0	0.705	0.612	0.146	0.902

TaMAPK17	TaMEKK24	4565.A0A3B6PHX1	4565.A0A3B6LLV5	0	0	0.18	0.607	0	0.27	0.063	0.088	0.42
TaMAPK17	TaMEKK16	4565.A0A3B6PHX1	4565.A0A3B6LW00	0	0	0.164	0.582	0	0.222	0.186	0.065	0.438
TaMAPK17	TaMAPKK2	4565.A0A3B6PHX1	4565.A0A3B6LYW0	0	0	0.107	0.583	0	0.705	0.612	0.146	0.901
TaMAPK17	TaMAPKK3	4565.A0A3B6PHX1	4565.A0A3B6MNP8	0	0	0.109	0.582	0	0.705	0.612	0.146	0.901
TaMAPK17	TaMEKK29	4565.A0A3B6PHX1	4565.A0A3B6MSP6	0	0	0.181	0.607	0	0.27	0.063	0.088	0.421
TaMAPK17	TaMEKK11	4565.A0A3B6PHX1	4565.A0A3B6N0D8	0	0	0.162	0.58	0	0.222	0.186	0.065	0.437
TaMAPK17	TaMAPKK13	4565.A0A3B6PHX1	4565.A0A3B6N2X8	0	0	0	0.581	0	0.705	0.612	0.146	0.893
TaMAPK17	TaMEKK4	4565.A0A3B6PHX1	4565.A0A3B6NRN9	0	0	0.16	0.597	0	0.27	0.063	0.088	0.406
TaMAPK17	TaMEKK4-1	4565.A0A3B6PHX1	4565.A0A3B6PNI6	0	0	0.162	0.6	0	0.27	0.063	0.088	0.407
TaMAPK17	TaRaf87	4565.A0A3B6PHX1	4565.A0A3B6QMZ9	0	0	0	0.587	0.3	0.134	0.389	0.088	0.617
TaMAPK17	TaMAPKK1	4565.A0A3B6PHX1	4565.A0A3B6QJ87	0	0	0.156	0.607	0	0.705	0.583	0.146	0.899
TaMAPK19	TaMAPKK9	4565.A0A3B6RDB9	4565.A0A077RVQ4	0	0	0.13	0.633	0	0.705	0.763	0.146	0.941
TaMAPK19	TaMAPK28	4565.A0A3B6RDB9	4565.A0A3B5YXF4	0	0	0.054	0.973	0	0.139	0.841	0	0.859
TaMAPK19	TaMEKK5	4565.A0A3B6RDB9	4565.A0A3B6AWC1	0	0	0.126	0.634	0	0.222	0.186	0.065	0.413
TaMAPK19	TaRaf111	4565.A0A3B6RDB9	4565.A0A3B6FHS8	0	0	0	0	0.07	0.141	0.15	0.297	0.458
TaMAPK19	TaMAPKK15	4565.A0A3B6RDB9	4565.A0A3B6HW51	0	0	0.131	0.645	0	0.705	0.763	0.146	0.941
TaMAPK19	TaMAPKK14	4565.A0A3B6RDB9	4565.A0A3B6HY95	0	0	0.132	0.649	0	0.705	0.763	0.146	0.941
TaMAPK19	TaMAPKK16	4565.A0A3B6RDB9	4565.A0A3B6HZP7	0	0	0.136	0.652	0	0.705	0.763	0.146	0.941

TaMAPK19	TaMAPKK 17	4565.A0A3 B6RDB9	4565.A0A3 B6I0M7	0	0	0.13	0.639	0	0.705	0.763	0.146	0.941
TaMAPK19	TaMAPKK 8	4565.A0A3 B6RDB9	4565.A0A3 B6IK39	0	0	0.128	0.643	0	0.705	0.763	0.146	0.94
TaMAPK19	TaMAPKK 6	4565.A0A3 B6RDB9	4565.A0A3 B6ILF0	0	0	0.129	0.633	0	0.705	0.763	0.146	0.941
TaMAPK19	TaMAPKK 5	4565.A0A3 B6RDB9	4565.A0A3 B6IMW7	0	0	0.128	0.65	0	0.705	0.763	0.146	0.941
TaMAPK19	TaMAPKK 7	4565.A0A3 B6RDB9	4565.A0A3 B6INV0	0	0	0.131	0.638	0	0.705	0.763	0.146	0.941
TaMAPK19	TaMEKK2	4565.A0A3 B6RDB9	4565.A0A3 B6JCC4	0	0	0.108	0.623	0	0.222	0.186	0.065	0.401
TaMAPK19	TaMAPKK 11	4565.A0A3 B6RDB9	4565.A0A3 B6JEH0	0	0	0.134	0.645	0	0.705	0.763	0.146	0.941
TaMAPK19	TaMAPKK 12	4565.A0A3 B6RDB9	4565.A0A3 B6JG06	0	0	0.134	0.636	0	0.705	0.946	0.146	0.986
TaMAPK19	TaMAPK2 5	4565.A0A3 B6RDB9	4565.A0A3 B6JLL7	0	0	0.052	0.976	0	0	0.946	0.446	0.969
TaMAPK19	TaMEKK1 4	4565.A0A3 B6RDB9	4565.A0A3 B6KF43	0	0	0.124	0.62	0	0.222	0.186	0.065	0.412
TaMAPK19	TaMAPKK 4	4565.A0A3 B6RDB9	4565.A0A3 B6KFB5	0	0	0.101	0.636	0	0.705	0.825	0.209	0.958
TaMAPK19	TaMEKK1 5	4565.A0A3 B6RDB9	4565.A0A3 B6KPK7	0	0	0.117	0.62	0	0.222	0.186	0.065	0.407
TaMAPK19	TaMAPKK 18	4565.A0A3 B6RDB9	4565.A0A3 B6LJ27	0	0	0	0.602	0	0.705	0.825	0.209	0.955
TaMAPK19	TaMEKK1 6	4565.A0A3 B6RDB9	4565.A0A3 B6LW00	0	0	0.115	0.621	0	0.222	0.186	0.065	0.406
TaMAPK19	TaMAPKK 2	4565.A0A3 B6RDB9	4565.A0A3 B6LYW0	0	0	0	0.611	0	0.705	0.927	0.209	0.981
TaMAPK19	TaMAPKK 3	4565.A0A3 B6RDB9	4565.A0A3 B6MNP8	0	0	0	0.604	0	0.705	0.825	0.209	0.955
TaMAPK19	TaMEKK1 1	4565.A0A3 B6RDB9	4565.A0A3 B6N0D8	0	0	0.115	0.619	0	0.222	0.186	0.065	0.406
TaMAPK19	TaMAPKK 13	4565.A0A3 B6RDB9	4565.A0A3 B6N2X8	0	0	0	0.615	0	0.705	0.927	0.209	0.981

TaMAPK19	TaMAPKK 1	4565.A0A3 B6RDB9	4565.A0A3 B6QJ87	0	0	0.148	0.625	0	0.705	0.883	0.146	0.971
TaMAPK19	TaRaf87	4565.A0A3 B6RDB9	4565.A0A3 B6QMZ9	0	0	0	0.598	0.16	0.134	0.389	0.088	0.54
TaMAPK19	TaMAPK3	4565.A0A3 B6RDB9	4565.A7L5 U5	0	0	0.052	0.976	0	0	0.946	0.446	0.969
TaMAPK20	TaMAPKK 9	4565.A0A3 B6TNR5	4565.A0A0 77RVQ4	0	0	0.12	0.616	0	0.705	0.583	0.146	0.895
TaMAPK20	TaRaf88	4565.A0A3 B6TNR5	4565.A0A3 B5Z5X1	0	0	0.285	0	0	0.15	0.177	0.05	0.461
TaMAPK20	TaRaf30	4565.A0A3 B6TNR5	4565.A0A3 B6A1Z4	0	0	0.282	0	0	0.15	0.177	0.05	0.458
TaMAPK20	TaMEKK5	4565.A0A3 B6TNR5	4565.A0A3 B6AWC1	0	0	0.164	0.575	0	0.222	0.186	0.065	0.438
TaMAPK20	TaMAPKK 15	4565.A0A3 B6TNR5	4565.A0A3 B6HW51	0	0	0.126	0.611	0	0.705	0.583	0.146	0.895
TaMAPK20	TaMAPKK 14	4565.A0A3 B6TNR5	4565.A0A3 B6HY95	0	0	0.125	0.607	0	0.705	0.583	0.146	0.895
TaMAPK20	TaMAPKK 16	4565.A0A3 B6TNR5	4565.A0A3 B6HZP7	0	0	0.124	0.616	0	0.705	0.583	0.146	0.895
TaMAPK20	TaMAPKK 17	4565.A0A3 B6TNR5	4565.A0A3 B6I0M7	0	0	0.12	0.624	0	0.705	0.583	0.146	0.895
TaMAPK20	TaMAPKK 8	4565.A0A3 B6TNR5	4565.A0A3 B6IK39	0	0	0.118	0.615	0	0.705	0.583	0.146	0.894
TaMAPK20	TaMAPKK 6	4565.A0A3 B6TNR5	4565.A0A3 B6ILF0	0	0	0.122	0.609	0	0.705	0.583	0.146	0.895
TaMAPK20	TaMAPKK 5	4565.A0A3 B6TNR5	4565.A0A3 B6IMW7	0	0	0.122	0.605	0	0.705	0.583	0.146	0.895
TaMAPK20	TaMAPKK 7	4565.A0A3 B6TNR5	4565.A0A3 B6INV0	0	0	0.122	0.626	0	0.705	0.583	0.146	0.895
TaMAPK20	TaMEKK2	4565.A0A3 B6TNR5	4565.A0A3 B6JCC4	0	0	0.144	0.572	0	0.222	0.186	0.065	0.425
TaMAPK20	TaMAPKK 11	4565.A0A3 B6TNR5	4565.A0A3 B6JEH0	0	0	0.122	0.615	0	0.705	0.583	0.146	0.895
TaMAPK20	TaMAPKK 12	4565.A0A3 B6TNR5	4565.A0A3 B6JG06	0	0	0.126	0.621	0	0.705	0.583	0.146	0.895

TaMAPK20	TaMEKK14	4565.A0A3B6TNR5	4565.A0A3B6KF43	0	0	0.155	0.583	0	0.222	0.186	0.065	0.433
TaMAPK20	TaMAPKK4	4565.A0A3B6TNR5	4565.A0A3B6KFB5	0	0	0.105	0.637	0	0.705	0.612	0.146	0.9
TaMAPK20	TaMEKK17	4565.A0A3B6TNR5	4565.A0A3B6KFL8	0	0	0.17	0.582	0	0.27	0.063	0.088	0.413
TaMAPK20	TaMEKK15	4565.A0A3B6TNR5	4565.A0A3B6KPK7	0	0	0.156	0.575	0	0.222	0.186	0.065	0.433
TaMAPK20	TaMAPKK18	4565.A0A3B6TNR5	4565.A0A3B6LJ27	0	0	0	0.585	0	0.705	0.612	0.146	0.893
TaMAPK20	TaMEKK24	4565.A0A3B6TNR5	4565.A0A3B6LLV5	0	0	0.169	0.582	0	0.27	0.063	0.088	0.412
TaMAPK20	TaMEKK16	4565.A0A3B6TNR5	4565.A0A3B6LW00	0	0	0.154	0.575	0	0.222	0.186	0.065	0.432
TaMAPK20	TaMAPKK2	4565.A0A3B6TNR5	4565.A0A3B6LYW0	0	0	0	0.575	0	0.705	0.612	0.205	0.901
TaMAPK20	TaMAPKK3	4565.A0A3B6TNR5	4565.A0A3B6MNP8	0	0	0	0.58	0	0.705	0.612	0.146	0.893
TaMAPK20	TaMEKK29	4565.A0A3B6TNR5	4565.A0A3B6MSP6	0	0	0.171	0.581	0	0.27	0.063	0.088	0.414
TaMAPK20	TaMEKK11	4565.A0A3B6TNR5	4565.A0A3B6N0D8	0	0	0.153	0.574	0	0.222	0.186	0.065	0.431
TaMAPK20	TaMAPKK13	4565.A0A3B6TNR5	4565.A0A3B6N2X8	0	0	0	0.576	0	0.705	0.612	0.205	0.901
TaMAPK20	TaMAPKK1	4565.A0A3B6TNR5	4565.A0A3B6QJ87	0	0	0.126	0.619	0	0.705	0.583	0.146	0.895
TaMAPK20	TaRaf87	4565.A0A3B6TNR5	4565.A0A3B6QMZ9	0	0	0	0.565	0.3	0.134	0.389	0.088	0.617
TaMAPK22	TaMAPKK9	4565.A0A3B6TPT1	4565.A0A077RVQ4	0	0	0.118	0.646	0	0.705	0.583	0.146	0.895
TaMAPK22	TaMEKK5	4565.A0A3B6TPT1	4565.A0A3B6AWC1	0	0	0.125	0.634	0	0.222	0.186	0.065	0.412
TaMAPK22	TaMAPKK15	4565.A0A3B6TPT1	4565.A0A3B6HW51	0	0	0.122	0.638	0	0.705	0.583	0.146	0.895
TaMAPK22	TaMAPKK14	4565.A0A3B6TPT1	4565.A0A3B6HY95	0	0	0.127	0.629	0	0.705	0.583	0.146	0.896

TaMAPK22	TaMAPKK16	4565.A0A3B6TPT1	4565.A0A3B6HZP7	0	0	0.126	0.644	0	0.705	0.583	0.146	0.895
TaMAPK22	TaMAPKK17	4565.A0A3B6TPT1	4565.A0A3B6I0M7	0	0	0.127	0.621	0	0.705	0.583	0.146	0.896
TaMAPK22	TaMAPKK8	4565.A0A3B6TPT1	4565.A0A3B6IK39	0	0	0.12	0.635	0	0.705	0.583	0.146	0.895
TaMAPK22	TaMAPKK6	4565.A0A3B6TPT1	4565.A0A3B6ILF0	0	0	0.126	0.622	0	0.705	0.583	0.146	0.895
TaMAPK22	TaMAPKK5	4565.A0A3B6TPT1	4565.A0A3B6IMW7	0	0	0.125	0.624	0	0.705	0.583	0.146	0.895
TaMAPK22	TaMAPKK7	4565.A0A3B6TPT1	4565.A0A3B6INV0	0	0	0.127	0.619	0	0.705	0.583	0.146	0.896
TaMAPK22	TaMEKK2	4565.A0A3B6TPT1	4565.A0A3B6JCC4	0	0	0.11	0.607	0	0.222	0.186	0.065	0.403
TaMAPK22	TaMAPKK11	4565.A0A3B6TPT1	4565.A0A3B6JEH0	0	0	0.131	0.632	0	0.705	0.583	0.146	0.896
TaMAPK22	TaMAPKK12	4565.A0A3B6TPT1	4565.A0A3B6JG06	0	0	0.129	0.615	0	0.705	0.583	0.146	0.896
TaMAPK22	TaMEKK14	4565.A0A3B6TPT1	4565.A0A3B6KF43	0	0	0.125	0.621	0	0.222	0.186	0.065	0.413
TaMAPK22	TaMAPKK4	4565.A0A3B6TPT1	4565.A0A3B6KFB5	0	0	0.103	0.612	0	0.705	0.739	0.195	0.937
TaMAPK22	TaMEKK15	4565.A0A3B6TPT1	4565.A0A3B6KPK7	0	0	0.115	0.63	0	0.222	0.186	0.065	0.406
TaMAPK22	TaMAPKK18	4565.A0A3B6TPT1	4565.A0A3B6LJ27	0	0	0	0.591	0	0.705	0.739	0.195	0.932
TaMAPK22	TaMEKK16	4565.A0A3B6TPT1	4565.A0A3B6LW00	0	0	0.113	0.63	0	0.222	0.186	0.065	0.405
TaMAPK22	TaMAPKK2	4565.A0A3B6TPT1	4565.A0A3B6LYW0	0	0	0	0.604	0	0.705	0.886	0.195	0.97
TaMAPK22	TaMAPKK3	4565.A0A3B6TPT1	4565.A0A3B6MNP8	0	0	0	0.591	0	0.705	0.739	0.195	0.932
TaMAPK22	TaMEKK11	4565.A0A3B6TPT1	4565.A0A3B6N0D8	0	0	0.114	0.629	0	0.222	0.186	0.065	0.405
TaMAPK22	TaMAPKK13	4565.A0A3B6TPT1	4565.A0A3B6N2X8	0	0	0	0.6	0	0.705	0.886	0.195	0.97

TaMAPK22	TaMAPK36	4565.A0A3B6TPT1	4565.A0A3B6NN33	0	0	0.049	0.983	0	0	0.793	0	0.794
TaMAPK22	TaMAPK45	4565.A0A3B6TPT1	4565.A0A3B6QBD2	0	0	0.049	0.983	0	0	0.793	0	0.794
TaMAPK22	TaMAPKK1	4565.A0A3B6TPT1	4565.A0A3B6QJ87	0	0	0.138	0.608	0	0.705	0.583	0.146	0.897
TaMAPK22	TaRaf87	4565.A0A3B6TPT1	4565.A0A3B6QMZ9	0	0	0	0.579	0.16	0.134	0.389	0.088	0.54
TaMAPK23	TaMAPKK9	4565.A0A3B6GWR5	4565.A0A077RVQ4	0	0	0.12	0.625	0	0.705	0.583	0.146	0.895
TaMAPK23	TaMEKK5	4565.A0A3B6GWR5	4565.A0A3B6AWC1	0	0	0.148	0.583	0	0.222	0.186	0.065	0.428
TaMAPK23	TaMEKK17	4565.A0A3B6GWR5	4565.A0A3B6KFL8	0	0	0.161	0.605	0	0.27	0.063	0.088	0.406
TaMAPK23	TaMEKK24	4565.A0A3B6GWR5	4565.A0A3B6LLV5	0	0	0.161	0.605	0	0.27	0.063	0.088	0.406
TaMAPK23	TaMEKK29	4565.A0A3B6GWR5	4565.A0A3B6MSP6	0	0	0.161	0.605	0	0.27	0.063	0.088	0.407
TaMAPK23	TaMEKK2	4565.A0A3B6GWR5	4565.A0A3B6JCC4	0	0	0.133	0.577	0	0.222	0.186	0.065	0.418
TaMAPK23	TaMEKK16	4565.A0A3B6GWR5	4565.A0A3B6LW00	0	0	0.143	0.58	0	0.222	0.186	0.065	0.424
TaMAPK23	TaMEKK11	4565.A0A3B6GWR5	4565.A0A3B6N0D8	0	0	0.142	0.578	0	0.222	0.186	0.065	0.424
TaMAPK23	TaMEKK15	4565.A0A3B6GWR5	4565.A0A3B6KPK7	0	0	0.144	0.581	0	0.222	0.186	0.065	0.425
TaMAPK23	TaMEKK14	4565.A0A3B6GWR5	4565.A0A3B6KF43	0	0	0.148	0.596	0	0.222	0.186	0.065	0.428
TaMAPK23	TaRaf87	4565.A0A3B6GWR5	4565.A0A3B6QMZ9	0	0	0	0.571	0.3	0.134	0.389	0.088	0.617
TaMAPK23	TaMAPKK13	4565.A0A3B6GWR5	4565.A0A3B6N2X8	0	0	0	0.579	0	0.705	0.612	0.146	0.893
TaMAPK23	TaMAPKK2	4565.A0A3B6GWR5	4565.A0A3B6LYW0	0	0	0	0.579	0	0.705	0.612	0.146	0.893
TaMAPK23	TaMAPKK3	4565.A0A3B6GWR5	4565.A0A3B6MNP8	0	0	0	0.577	0	0.705	0.612	0.146	0.893

TaMAPK23	TaMAPKK8	4565.A0A3B6GWR5	4565.A0A3B6IK39	0	0	0.117	0.616	0	0.705	0.583	0.146	0.894
TaMAPK23	TaMAPKK14	4565.A0A3B6GWR5	4565.A0A3B6HY95	0	0	0.125	0.613	0	0.705	0.583	0.146	0.895
TaMAPK23	TaMAPKK11	4565.A0A3B6GWR5	4565.A0A3B6JEH0	0	0	0.121	0.618	0	0.705	0.583	0.146	0.895
TaMAPK23	TaMAPKK7	4565.A0A3B6GWR5	4565.A0A3B6INV0	0	0	0.126	0.612	0	0.705	0.583	0.146	0.895
TaMAPK23	TaMAPKK15	4565.A0A3B6GWR5	4565.A0A3B6HW51	0	0	0.126	0.617	0	0.705	0.583	0.146	0.895
TaMAPK23	TaMAPKK17	4565.A0A3B6GWR5	4565.A0A3B6I0M7	0	0	0.125	0.613	0	0.705	0.583	0.146	0.895
TaMAPK23	TaMAPKK6	4565.A0A3B6GWR5	4565.A0A3B6ILF0	0	0	0.125	0.606	0	0.705	0.583	0.146	0.895
TaMAPK23	TaMAPKK16	4565.A0A3B6GWR5	4565.A0A3B6HZP7	0	0	0.123	0.621	0	0.705	0.583	0.146	0.895
TaMAPK23	TaMAPKK5	4565.A0A3B6GWR5	4565.A0A3B6IMW7	0	0	0.121	0.61	0	0.705	0.583	0.146	0.895
TaMAPK23	TaMAPKK1	4565.A0A3B6GWR5	4565.A0A3B6QJ87	0	0	0.133	0.613	0	0.705	0.583	0.146	0.896
TaMAPK23	TaMAPKK12	4565.A0A3B6GWR5	4565.A0A3B6JG06	0	0	0.131	0.608	0	0.705	0.583	0.146	0.896
TaMAPK23	TaMAPKK18	4565.A0A3B6GWR5	4565.A0A3B6LJ27	0	0	0.108	0.576	0	0.705	0.612	0.146	0.901
TaMAPK23	TaMAPKK4	4565.A0A3B6GWR5	4565.A0A3B6KFB5	0	0	0.111	0.62	0	0.705	0.612	0.146	0.901
TaMAPK24	TaMAPKK9	4565.A0A3B6GX04	4565.A0A077RVQ4	0	0	0.114	0.622	0	0.705	0.583	0.146	0.894
TaMAPK24	TaRaf30	4565.A0A3B6GX04	4565.A0A3B6A1Z4	0	0	0.228	0	0	0.15	0.177	0.05	0.418
TaMAPK24	TaMEKK5	4565.A0A3B6GX04	4565.A0A3B6AWC1	0	0	0.144	0.579	0	0.222	0.186	0.065	0.425
TaMAPK24	TaMEKK2	4565.A0A3B6GX04	4565.A0A3B6JCC4	0	0	0.126	0.578	0	0.222	0.186	0.065	0.413
TaMAPK24	TaMEKK11	4565.A0A3B6GX04	4565.A0A3B6N0D8	0	0	0.131	0.572	0	0.222	0.186	0.065	0.417

TaMAPK24	TaMEKK16	4565.A0A3B6GX04	4565.A0A3B6LW00	0	0	0.132	0.574	0	0.222	0.186	0.065	0.417
TaMAPK24	TaMEKK15	4565.A0A3B6GX04	4565.A0A3B6KPK7	0	0	0.134	0.575	0	0.222	0.186	0.065	0.418
TaMAPK24	TaMEKK29	4565.A0A3B6GX04	4565.A0A3B6MSP6	0	0	0.188	0.574	0	0.27	0.063	0.088	0.425
TaMAPK24	TaMEKK14	4565.A0A3B6GX04	4565.A0A3B6KF43	0	0	0.144	0.586	0	0.222	0.186	0.065	0.425
TaMAPK24	TaMEKK24	4565.A0A3B6GX04	4565.A0A3B6LLV5	0	0	0.188	0.573	0	0.27	0.063	0.088	0.425
TaMAPK24	TaMEKK17	4565.A0A3B6GX04	4565.A0A3B6KFL8	0	0	0.188	0.574	0	0.27	0.063	0.088	0.425
TaMAPK24	TaRaf87	4565.A0A3B6GX04	4565.A0A3B6QMZ9	0	0	0	0.564	0.277	0.134	0.389	0.088	0.604
TaMAPK24	TaMAPKK2	4565.A0A3B6GX04	4565.A0A3B6LYW0	0	0	0	0.584	0	0.705	0.583	0.146	0.885
TaMAPK24	TaMAPKK3	4565.A0A3B6GX04	4565.A0A3B6MNP8	0	0	0	0.581	0	0.705	0.583	0.146	0.885
TaMAPK24	TaMAPKK13	4565.A0A3B6GX04	4565.A0A3B6N2X8	0	0	0	0.583	0	0.705	0.583	0.146	0.885
TaMAPK24	TaMAPKK18	4565.A0A3B6GX04	4565.A0A3B6LJ27	0	0	0	0.587	0	0.705	0.583	0.146	0.885
TaMAPK24	TaMAPKK4	4565.A0A3B6GX04	4565.A0A3B6KFB5	0	0	0	0.639	0	0.705	0.583	0.146	0.885
TaMAPK24	TaMAPKK15	4565.A0A3B6GX04	4565.A0A3B6HW51	0	0	0.117	0.621	0	0.705	0.583	0.146	0.894
TaMAPK24	TaMAPKK5	4565.A0A3B6GX04	4565.A0A3B6IMW7	0	0	0.115	0.613	0	0.705	0.583	0.146	0.894
TaMAPK24	TaMAPKK16	4565.A0A3B6GX04	4565.A0A3B6HZP7	0	0	0.117	0.621	0	0.705	0.583	0.146	0.894
TaMAPK24	TaMAPKK8	4565.A0A3B6GX04	4565.A0A3B6IK39	0	0	0.113	0.621	0	0.705	0.583	0.146	0.894
TaMAPK24	TaMAPKK14	4565.A0A3B6GX04	4565.A0A3B6HY95	0	0	0.115	0.619	0	0.705	0.583	0.146	0.894
TaMAPK24	TaMAPKK11	4565.A0A3B6GX04	4565.A0A3B6JEH0	0	0	0.118	0.618	0	0.705	0.583	0.146	0.894

TaMAPK24	TaMAPKK7	4565.A0A3B6GX04	4565.A0A3B6INV0	0	0	0.123	0.613	0	0.705	0.583	0.146	0.895
TaMAPK24	TaMAPKK17	4565.A0A3B6GX04	4565.A0A3B6I0M7	0	0	0.12	0.615	0	0.705	0.583	0.146	0.895
TaMAPK24	TaMAPKK6	4565.A0A3B6GX04	4565.A0A3B6ILF0	0	0	0.121	0.611	0	0.705	0.583	0.146	0.895
TaMAPK24	TaMAPKK12	4565.A0A3B6GX04	4565.A0A3B6JG06	0	0	0.124	0.61	0	0.705	0.583	0.146	0.895
TaMAPK24	TaMAPKK1	4565.A0A3B6GX04	4565.A0A3B6QJ87	0	0	0.128	0.619	0	0.705	0.583	0.146	0.896
TaMAPK25	TaMAPKK9	4565.A0A3B6JLL7	4565.A0A077RVQ4	0	0	0.127	0.63	0	0.705	0.791	0.146	0.947
TaMAPK25	TaMEKK5	4565.A0A3B6JLL7	4565.A0A3B6AWC1	0	0	0.118	0.652	0	0.222	0.186	0.065	0.408
TaMAPK25	TaMEKK1	4565.A0A3B6JLL7	4565.A0A3B6B3I4	0	0	0.127	0.658	0	0.27	0.063	0.137	0.416
TaMAPK25	TaRaf111	4565.A0A3B6JLL7	4565.A0A3B6FHS8	0	0	0	0	0.051	0.141	0.15	0.403	0.53
TaMAPK25	TaMAPKK15	4565.A0A3B6JLL7	4565.A0A3B6HW51	0	0	0.123	0.649	0	0.705	0.791	0.146	0.947
TaMAPK25	TaMAPKK14	4565.A0A3B6JLL7	4565.A0A3B6HY95	0	0	0.128	0.645	0	0.705	0.791	0.146	0.947
TaMAPK25	TaMAPKK16	4565.A0A3B6JLL7	4565.A0A3B6HZP7	0	0	0.133	0.643	0	0.705	0.791	0.146	0.948
TaMAPK25	TaMAPKK17	4565.A0A3B6JLL7	4565.A0A3B6I0M7	0	0	0.125	0.635	0	0.705	0.791	0.146	0.947
TaMAPK25	TaMAPKK8	4565.A0A3B6JLL7	4565.A0A3B6IK39	0	0	0.127	0.644	0	0.705	0.791	0.146	0.947
TaMAPK25	TaMAPKK6	4565.A0A3B6JLL7	4565.A0A3B6ILF0	0	0	0.125	0.634	0	0.705	0.791	0.146	0.947
TaMAPK25	TaMAPKK5	4565.A0A3B6JLL7	4565.A0A3B6IMW7	0	0	0.126	0.639	0	0.705	0.791	0.146	0.947
TaMAPK25	TaMAPKK7	4565.A0A3B6JLL7	4565.A0A3B6INV0	0	0	0.127	0.634	0	0.705	0.791	0.146	0.947
TaMAPK25	TaMEKK2	4565.A0A3B6JLL7	4565.A0A3B6JCC4	0	0	0.106	0.624	0	0.222	0.186	0.074	0.406

TaMAPK25	TaMAPKK11	4565.A0A3B6JLL7	4565.A0A3B6JEH0	0	0	0.134	0.633	0	0.705	0.791	0.146	0.948
TaMAPK25	TaMAPKK12	4565.A0A3B6JLL7	4565.A0A3B6JG06	0	0	0.127	0.63	0	0.705	0.959	0.146	0.989
TaMAPK25	TaMEKK11	4565.A0A3B6JLL7	4565.A0A3B6N0D8	0	0	0.106	0.638	0	0.222	0.186	0.065	0.4
TaMAPK25	TaMEKK14	4565.A0A3B6JLL7	4565.A0A3B6KF43	0	0	0.116	0.654	0	0.222	0.186	0.065	0.406
TaMAPK25	TaMEKK4-1	4565.A0A3B6JLL7	4565.A0A3B6PNI6	0	0	0.125	0.666	0	0.27	0.063	0.137	0.414
TaMAPK25	TaMEKK4	4565.A0A3B6JLL7	4565.A0A3B6NRN9	0	0	0.126	0.661	0	0.27	0.063	0.137	0.415
TaMAPK25	TaMEKK24	4565.A0A3B6JLL7	4565.A0A3B6LLV5	0	0	0.142	0.636	0	0.27	0.063	0.136	0.425
TaMAPK25	TaMEKK29	4565.A0A3B6JLL7	4565.A0A3B6MSP6	0	0	0.143	0.636	0	0.27	0.063	0.136	0.425
TaMAPK25	TaMEKK17	4565.A0A3B6JLL7	4565.A0A3B6KFL8	0	0	0.142	0.637	0	0.27	0.063	0.136	0.425
TaMAPK25	TaMEKK15	4565.A0A3B6JLL7	4565.A0A3B6KPK7	0	0	0.107	0.639	0.111	0.222	0.186	0.065	0.444
TaMAPK25	TaMEKK16	4565.A0A3B6JLL7	4565.A0A3B6LW00	0	0	0.106	0.639	0.114	0.222	0.186	0.065	0.445
TaMAPK25	TaRaf87	4565.A0A3B6JLL7	4565.A0A3B6QMZ9	0	0	0	0.598	0.16	0.134	0.389	0.088	0.54
TaMAPK25	TaMAPKK2	4565.A0A3B6JLL7	4565.A0A3B6LYW0	0	0	0	0.621	0	0.705	0.583	0.149	0.886
TaMAPK25	TaMAPKK3	4565.A0A3B6JLL7	4565.A0A3B6MNP8	0	0	0	0.62	0	0.705	0.583	0.149	0.886
TaMAPK25	TaMAPKK18	4565.A0A3B6JLL7	4565.A0A3B6LJ27	0	0	0	0.618	0	0.705	0.583	0.149	0.886
TaMAPK25	TaMAPKK13	4565.A0A3B6JLL7	4565.A0A3B6N2X8	0	0	0	0.618	0	0.705	0.583	0.149	0.886
TaMAPK25	TaMAPKK4	4565.A0A3B6JLL7	4565.A0A3B6KFB5	0	0	0.102	0.645	0	0.705	0.583	0.149	0.893
TaMAPK25	TaMAPK6	4565.A0A3B6JLL7	4565.A0A3B6SCW0	0	0	0.052	0.976	0	0	0.946	0.446	0.969

TaMAPK25	TaMAPKK1	4565.A0A3B6JLL7	4565.A0A3B6QJ87	0	0	0.141	0.615	0	0.705	0.888	0.146	0.972
TaMAPK27	TaMAPKK9	4565.A9RAB0	4565.A0A077RVQ4	0	0	0.127	0.623	0	0.705	0.583	0.146	0.896
TaMAPK27	TaMEKK5	4565.A9RAB0	4565.A0A3B6AWC1	0	0	0.159	0.574	0	0.222	0.186	0.065	0.435
TaMAPK27	TaMAPKK15	4565.A9RAB0	4565.A0A3B6HW51	0	0	0.134	0.615	0	0.705	0.583	0.146	0.896
TaMAPK27	TaMAPKK14	4565.A9RAB0	4565.A0A3B6HY95	0	0	0.134	0.61	0	0.705	0.583	0.146	0.896
TaMAPK27	TaMAPKK16	4565.A9RAB0	4565.A0A3B6HZP7	0	0	0.133	0.621	0	0.705	0.583	0.146	0.896
TaMAPK27	TaMAPKK17	4565.A9RAB0	4565.A0A3B6I0M7	0	0	0.138	0.613	0	0.705	0.583	0.146	0.897
TaMAPK27	TaMAPKK8	4565.A9RAB0	4565.A0A3B6IK39	0	0	0.124	0.615	0	0.705	0.583	0.146	0.895
TaMAPK27	TaMAPKK6	4565.A9RAB0	4565.A0A3B6ILF0	0	0	0.14	0.607	0	0.705	0.583	0.146	0.897
TaMAPK27	TaMAPKK5	4565.A9RAB0	4565.A0A3B6IMW7	0	0	0.129	0.609	0	0.705	0.583	0.146	0.896
TaMAPK27	TaMAPKK7	4565.A9RAB0	4565.A0A3B6INV0	0	0	0.139	0.611	0	0.705	0.583	0.146	0.897
TaMAPK27	TaMEKK2	4565.A9RAB0	4565.A0A3B6JCC4	0	0	0.137	0.568	0	0.222	0.186	0.065	0.42
TaMAPK27	TaMAPKK11	4565.A9RAB0	4565.A0A3B6JEH0	0	0	0.128	0.621	0	0.705	0.583	0.146	0.896
TaMAPK27	TaMAPKK12	4565.A9RAB0	4565.A0A3B6JG06	0	0	0.146	0.608	0	0.705	0.583	0.146	0.898
TaMAPK27	TaMEKK14	4565.A9RAB0	4565.A0A3B6KF43	0	0	0.153	0.581	0	0.222	0.186	0.065	0.431
TaMAPK27	TaMAPKK4	4565.A9RAB0	4565.A0A3B6KFB5	0	0	0.131	0.629	0	0.705	0.612	0.146	0.903
TaMAPK27	TaMEKK17	4565.A9RAB0	4565.A0A3B6KFL8	0	0	0.178	0.59	0	0.27	0.063	0.088	0.418
TaMAPK27	TaMEKK15	4565.A9RAB0	4565.A0A3B6KPK7	0	0	0.152	0.573	0	0.222	0.186	0.065	0.431

TaMAPK27	TaMAPKK18	4565.A9RAB0	4565.A0A3B6LJ27	0	0	0.138	0.582	0	0.705	0.612	0.146	0.904
TaMAPK27	TaMEKK24	4565.A9RAB0	4565.A0A3B6LLV5	0	0	0.177	0.59	0	0.27	0.063	0.088	0.418
TaMAPK27	TaMEKK16	4565.A9RAB0	4565.A0A3B6LW00	0	0	0.151	0.572	0	0.222	0.186	0.065	0.43
TaMAPK27	TaMAPKK2	4565.A9RAB0	4565.A0A3B6LYW0	0	0	0.124	0.581	0	0.705	0.612	0.146	0.903
TaMAPK27	TaMAPKK3	4565.A9RAB0	4565.A0A3B6MNP8	0	0	0.131	0.577	0	0.705	0.612	0.146	0.903
TaMAPK27	TaMEKK29	4565.A9RAB0	4565.A0A3B6MSP6	0	0	0.178	0.59	0	0.27	0.063	0.088	0.418
TaMAPK27	TaMEKK11	4565.A9RAB0	4565.A0A3B6N0D8	0	0	0.149	0.57	0	0.222	0.186	0.065	0.428
TaMAPK27	TaMAPKK13	4565.A9RAB0	4565.A0A3B6N2X8	0	0	0.109	0.58	0	0.705	0.612	0.146	0.901
TaMAPK27	TaMAPKK1	4565.A9RAB0	4565.A0A3B6QJ87	0	0	0.137	0.614	0	0.705	0.583	0.146	0.897
TaMAPK27	TaRaf87	4565.A9RAB0	4565.A0A3B6QMZ9	0	0	0	0.565	0.3	0.134	0.389	0.088	0.617
TaMAPK28	TaMAPKK9	4565.A0A3B5YXF4	4565.A0A077RVQ4	0	0	0.136	0.61	0	0.705	0.583	0.146	0.897
TaMAPK28	TaMEKK17	4565.A0A3B5YXF4	4565.A0A3B6KFL8	0	0	0.152	0.641	0	0.27	0.063	0.088	0.4
TaMAPK28	TaMEKK29	4565.A0A3B5YXF4	4565.A0A3B6MSP6	0	0	0.152	0.641	0	0.27	0.063	0.088	0.4
TaMAPK28	TaMEKK2	4565.A0A3B5YXF4	4565.A0A3B6JCC4	0	0	0.108	0.612	0	0.222	0.186	0.065	0.401
TaMAPK28	TaMEKK16	4565.A0A3B5YXF4	4565.A0A3B6LW00	0	0	0.112	0.623	0	0.222	0.186	0.065	0.404
TaMAPK28	TaMEKK15	4565.A0A3B5YXF4	4565.A0A3B6KPK7	0	0	0.113	0.623	0	0.222	0.186	0.065	0.404
TaMAPK28	TaMEKK11	4565.A0A3B5YXF4	4565.A0A3B6N0D8	0	0	0.112	0.622	0	0.222	0.186	0.065	0.404
TaMAPK28	TaMEKK5	4565.A0A3B5YXF4	4565.A0A3B6AWC1	0	0	0.121	0.633	0	0.222	0.186	0.065	0.409

TaMAPK28	TaMEKK14	4565.A0A3B5YXF4	4565.A0A3B6KF43	0	0	0.12	0.629	0	0.222	0.186	0.065	0.409
TaMAPK28	TaRaf87	4565.A0A3B5YXF4	4565.A0A3B6QMZ9	0	0	0	0.604	0.16	0.134	0.389	0.088	0.54
TaMAPK28	TaMAPK6	4565.A0A3B5YXF4	4565.A0A3B6SCW0	0	0	0.054	0.973	0	0.139	0.841	0	0.859
TaMAPK28	TaMAPKK13	4565.A0A3B5YXF4	4565.A0A3B6N2X8	0	0	0	0.61	0	0.705	0.583	0.146	0.885
TaMAPK28	TaMAPKK3	4565.A0A3B5YXF4	4565.A0A3B6MNP8	0	0	0.11	0.606	0	0.705	0.583	0.146	0.894
TaMAPK28	TaMAPKK2	4565.A0A3B5YXF4	4565.A0A3B6LYW0	0	0	0.111	0.61	0	0.705	0.583	0.146	0.894
TaMAPK28	TaMAPKK18	4565.A0A3B5YXF4	4565.A0A3B6LJ27	0	0	0.118	0.604	0	0.705	0.583	0.146	0.895
TaMAPK28	TaMAPKK4	4565.A0A3B5YXF4	4565.A0A3B6KFB5	0	0	0.125	0.629	0	0.705	0.583	0.146	0.895
TaMAPK28	TaMAPKK11	4565.A0A3B5YXF4	4565.A0A3B6JEH0	0	0	0.139	0.618	0	0.705	0.583	0.146	0.897
TaMAPK28	TaMAPKK12	4565.A0A3B5YXF4	4565.A0A3B6JG06	0	0	0.139	0.615	0	0.705	0.583	0.146	0.897
TaMAPK28	TaMAPKK14	4565.A0A3B5YXF4	4565.A0A3B6HY95	0	0	0.143	0.607	0	0.705	0.583	0.146	0.897
TaMAPK28	TaMAPKK1	4565.A0A3B5YXF4	4565.A0A3B6QJ87	0	0	0.141	0.617	0	0.705	0.583	0.146	0.897
TaMAPK28	TaMAPKK5	4565.A0A3B5YXF4	4565.A0A3B6IMW7	0	0	0.14	0.6	0	0.705	0.583	0.146	0.897
TaMAPK28	TaMAPKK6	4565.A0A3B5YXF4	4565.A0A3B6ILF0	0	0	0.135	0.613	0	0.705	0.583	0.146	0.897
TaMAPK28	TaMAPKK8	4565.A0A3B5YXF4	4565.A0A3B6IK39	0	0	0.137	0.607	0	0.705	0.583	0.146	0.897
TaMAPK28	TaMAPKK17	4565.A0A3B5YXF4	4565.A0A3B6I0M7	0	0	0.135	0.617	0	0.705	0.583	0.146	0.897
TaMAPK28	TaMAPKK15	4565.A0A3B5YXF4	4565.A0A3B6HW51	0	0	0.14	0.612	0	0.705	0.583	0.146	0.897
TaMAPK28	TaMAPKK7	4565.A0A3B5YXF4	4565.A0A3B6INV0	0	0	0.138	0.615	0	0.705	0.583	0.146	0.897

TaMAPK28	TaMAPKK16	4565.A0A3B5YXF4	4565.A0A3B6HZP7	0	0	0.146	0.608	0	0.705	0.583	0.146	0.898
TaMAPK29	TaMAPKK9	4565.A0A3B5Z4C1	4565.A0A077RVQ4	0	0	0.117	0.617	0	0.705	0.583	0.146	0.894
TaMAPK29	TaMEKK2	4565.A0A3B5Z4C1	4565.A0A3B6JCC4	0	0	0.128	0.576	0	0.222	0.186	0.065	0.414
TaMAPK29	TaMEKK17	4565.A0A3B5Z4C1	4565.A0A3B6KFL8	0	0	0.175	0.581	0	0.27	0.063	0.088	0.416
TaMAPK29	TaMEKK24	4565.A0A3B5Z4C1	4565.A0A3B6LLV5	0	0	0.174	0.581	0	0.27	0.063	0.088	0.416
TaMAPK29	TaMEKK29	4565.A0A3B5Z4C1	4565.A0A3B6MSP6	0	0	0.175	0.581	0	0.27	0.063	0.088	0.417
TaMAPK29	TaMEKK11	4565.A0A3B5Z4C1	4565.A0A3B6N0D8	0	0	0.136	0.572	0	0.222	0.186	0.065	0.42
TaMAPK29	TaMEKK16	4565.A0A3B5Z4C1	4565.A0A3B6LW00	0	0	0.137	0.573	0	0.222	0.186	0.065	0.421
TaMAPK29	TaMEKK15	4565.A0A3B5Z4C1	4565.A0A3B6KPK7	0	0	0.139	0.574	0	0.222	0.186	0.065	0.422
TaMAPK29	TaMEKK5	4565.A0A3B5Z4C1	4565.A0A3B6AWC1	0	0	0.146	0.578	0	0.222	0.186	0.065	0.427
TaMAPK29	TaMEKK14	4565.A0A3B5Z4C1	4565.A0A3B6KF43	0	0	0.15	0.582	0	0.222	0.186	0.065	0.429
TaMAPK29	TaRaf88	4565.A0A3B5Z4C1	4565.A0A3B5Z5X1	0	0	0.254	0	0	0.15	0.177	0.05	0.437
TaMAPK29	TaRaf30	4565.A0A3B5Z4C1	4565.A0A3B6A1Z4	0	0	0.255	0	0	0.15	0.177	0.05	0.438
TaMAPK29	TaRaf87	4565.A0A3B5Z4C1	4565.A0A3B6QMZ9	0	0	0	0.569	0.277	0.134	0.389	0.088	0.604
TaMAPK29	TaMAPKK18	4565.A0A3B5Z4C1	4565.A0A3B6LJ27	0	0	0	0.583	0	0.705	0.583	0.146	0.885
TaMAPK29	TaMAPKK13	4565.A0A3B5Z4C1	4565.A0A3B6N2X8	0	0	0	0.58	0	0.705	0.583	0.146	0.885
TaMAPK29	TaMAPKK2	4565.A0A3B5Z4C1	4565.A0A3B6LYW0	0	0	0	0.581	0	0.705	0.583	0.146	0.885
TaMAPK29	TaMAPKK3	4565.A0A3B5Z4C1	4565.A0A3B6MNP8	0	0	0	0.577	0	0.705	0.583	0.146	0.885

TaMAPK29	TaMAPKK4	4565.A0A3B5Z4C1	4565.A0A3B6KFB5	0	0	0.101	0.634	0	0.705	0.583	0.146	0.893
TaMAPK29	TaMAPKK8	4565.A0A3B5Z4C1	4565.A0A3B6IK39	0	0	0.115	0.621	0	0.705	0.583	0.146	0.894
TaMAPK29	TaMAPKK5	4565.A0A3B5Z4C1	4565.A0A3B6IMW7	0	0	0.118	0.607	0	0.705	0.583	0.146	0.894
TaMAPK29	TaMAPKK14	4565.A0A3B5Z4C1	4565.A0A3B6HY95	0	0	0.119	0.616	0	0.705	0.583	0.146	0.895
TaMAPK29	TaMAPKK11	4565.A0A3B5Z4C1	4565.A0A3B6JEH0	0	0	0.119	0.619	0	0.705	0.583	0.146	0.895
TaMAPK29	TaMAPKK12	4565.A0A3B5Z4C1	4565.A0A3B6JG06	0	0	0.123	0.613	0	0.705	0.583	0.146	0.895
TaMAPK29	TaMAPKK7	4565.A0A3B5Z4C1	4565.A0A3B6INV0	0	0	0.12	0.618	0	0.705	0.583	0.146	0.895
TaMAPK29	TaMAPKK15	4565.A0A3B5Z4C1	4565.A0A3B6HW51	0	0	0.119	0.62	0	0.705	0.583	0.146	0.895
TaMAPK29	TaMAPKK6	4565.A0A3B5Z4C1	4565.A0A3B6ILF0	0	0	0.119	0.616	0	0.705	0.583	0.146	0.895
TaMAPK29	TaMAPKK16	4565.A0A3B5Z4C1	4565.A0A3B6HZP7	0	0	0.12	0.621	0	0.705	0.583	0.146	0.895
TaMAPK29	TaMAPKK17	4565.A0A3B5Z4C1	4565.A0A3B6I0M7	0	0	0.118	0.618	0	0.705	0.583	0.146	0.895
TaMAPK29	TaMAPKK1	4565.A0A3B5Z4C1	4565.A0A3B6QJ87	0	0	0.129	0.613	0	0.705	0.583	0.146	0.896
TaMAPK3	TaMAPKK9	4565.A7L5U5	4565.A0A077RVQ4	0	0	0.126	0.629	0	0.705	0.791	0.146	0.947
TaMAPK3	TaMEKK5	4565.A7L5U5	4565.A0A3B6AWC1	0	0	0.119	0.65	0	0.222	0.186	0.065	0.408
TaMAPK3	TaMEKK1	4565.A7L5U5	4565.A0A3B6B3I4	0	0	0.124	0.658	0	0.27	0.063	0.137	0.414
TaMAPK3	TaRaf111	4565.A7L5U5	4565.A0A3B6FHS8	0	0	0	0	0.051	0.141	0.15	0.403	0.53
TaMAPK3	TaMAPKK15	4565.A7L5U5	4565.A0A3B6HW51	0	0	0.122	0.647	0	0.705	0.791	0.146	0.947
TaMAPK3	TaMAPKK14	4565.A7L5U5	4565.A0A3B6HY95	0	0	0.128	0.643	0	0.705	0.791	0.146	0.947

TaMAPK3	TaMAPKK 16	4565.A7L5 U5	4565.A0A3 B6HZP7	0	0	0.134	0.64	0	0.705	0.791	0.146	0.948
TaMAPK3	TaMAPKK 17	4565.A7L5 U5	4565.A0A3 B6I0M7	0	0	0.124	0.635	0	0.705	0.791	0.146	0.947
TaMAPK3	TaMAPKK 8	4565.A7L5 U5	4565.A0A3 B6IK39	0	0	0.128	0.642	0	0.705	0.791	0.146	0.947
TaMAPK3	TaMAPKK 6	4565.A7L5 U5	4565.A0A3 B6ILF0	0	0	0.126	0.63	0	0.705	0.791	0.146	0.947
TaMAPK3	TaMAPKK 5	4565.A7L5 U5	4565.A0A3 B6IMW7	0	0	0.126	0.636	0	0.705	0.791	0.146	0.947
TaMAPK3	TaMAPKK 7	4565.A7L5 U5	4565.A0A3 B6INV0	0	0	0.127	0.634	0	0.705	0.791	0.146	0.947
TaMAPK3	TaMEKK2	4565.A7L5 U5	4565.A0A3 B6JCC4	0	0	0.107	0.624	0	0.222	0.186	0.074	0.406
TaMAPK3	TaMAPKK 11	4565.A7L5 U5	4565.A0A3 B6JEH0	0	0	0.135	0.629	0	0.705	0.791	0.146	0.948
TaMAPK3	TaMAPKK 12	4565.A7L5 U5	4565.A0A3 B6JG06	0	0	0.127	0.63	0	0.705	0.959	0.146	0.989
TaMAPK3	TaMEKK1 4	4565.A7L5 U5	4565.A0A3 B6KF43	0	0	0.117	0.654	0	0.222	0.186	0.065	0.407
TaMAPK3	TaMAPKK 4	4565.A7L5 U5	4565.A0A3 B6KFB5	0	0	0.103	0.64	0	0.705	0.583	0.149	0.893
TaMAPK3	TaMEKK1 7	4565.A7L5 U5	4565.A0A3 B6KFL8	0	0	0.142	0.636	0	0.27	0.063	0.136	0.425
TaMAPK3	TaMEKK1 5	4565.A7L5 U5	4565.A0A3 B6KPK7	0	0	0.108	0.638	0.111	0.222	0.186	0.065	0.445
TaMAPK3	TaMAPKK 18	4565.A7L5 U5	4565.A0A3 B6LJ27	0	0	0	0.614	0	0.705	0.583	0.149	0.886
TaMAPK3	TaMEKK2 4	4565.A7L5 U5	4565.A0A3 B6LLV5	0	0	0.141	0.636	0	0.27	0.063	0.136	0.424
TaMAPK3	TaMEKK1 6	4565.A7L5 U5	4565.A0A3 B6LW00	0	0	0.107	0.638	0.114	0.222	0.186	0.065	0.446
TaMAPK3	TaMAPKK 2	4565.A7L5 U5	4565.A0A3 B6LYW0	0	0	0	0.622	0	0.705	0.583	0.149	0.886
TaMAPK3	TaMAPKK 3	4565.A7L5 U5	4565.A0A3 B6MNP8	0	0	0	0.616	0	0.705	0.583	0.149	0.886

TaMAPK3	TaMEKK29	4565.A7L5U5	4565.A0A3B6MSP6	0	0	0.142	0.636	0	0.27	0.063	0.136	0.425
TaMAPK3	TaMEKK11	4565.A7L5U5	4565.A0A3B6N0D8	0	0	0.107	0.637	0	0.222	0.186	0.065	0.4
TaMAPK3	TaMAPKK13	4565.A7L5U5	4565.A0A3B6N2X8	0	0	0	0.618	0	0.705	0.583	0.149	0.886
TaMAPK3	TaMEKK4	4565.A7L5U5	4565.A0A3B6NRN9	0	0	0.123	0.66	0	0.27	0.063	0.137	0.413
TaMAPK3	TaMEKK4-1	4565.A7L5U5	4565.A0A3B6PNI6	0	0	0.122	0.666	0	0.27	0.063	0.137	0.412
TaMAPK3	TaMAPKK1	4565.A7L5U5	4565.A0A3B6QJ87	0	0	0.14	0.615	0	0.705	0.888	0.146	0.972
TaMAPK3	TaRaf87	4565.A7L5U5	4565.A0A3B6QMZ9	0	0	0	0.597	0.16	0.134	0.389	0.088	0.54
TaMAPK3	TaMAPK6	4565.A7L5U5	4565.A0A3B6SCW0	0	0	0.052	0.976	0	0	0.946	0.446	0.969
TaMAPK30	TaMAPKK9	4565.A0A3B6RL73	4565.A0A077RVQ4	0	0	0.117	0.625	0	0.705	0.583	0.146	0.894
TaMAPK30	TaMEKK5	4565.A0A3B6RL73	4565.A0A3B6AWC1	0	0	0.151	0.595	0	0.222	0.186	0.065	0.43
TaMAPK30	TaMAPKK15	4565.A0A3B6RL73	4565.A0A3B6HW51	0	0	0.122	0.623	0	0.705	0.583	0.146	0.895
TaMAPK30	TaMAPKK14	4565.A0A3B6RL73	4565.A0A3B6HY95	0	0	0.12	0.619	0	0.705	0.583	0.146	0.895
TaMAPK30	TaMAPKK16	4565.A0A3B6RL73	4565.A0A3B6HZP7	0	0	0.121	0.624	0	0.705	0.583	0.146	0.895
TaMAPK30	TaMAPKK17	4565.A0A3B6RL73	4565.A0A3B6I0M7	0	0	0.12	0.626	0	0.705	0.583	0.146	0.895
TaMAPK30	TaMAPKK8	4565.A0A3B6RL73	4565.A0A3B6IK39	0	0	0.113	0.624	0	0.705	0.583	0.146	0.894
TaMAPK30	TaMAPKK6	4565.A0A3B6RL73	4565.A0A3B6ILF0	0	0	0.12	0.619	0	0.705	0.583	0.146	0.895
TaMAPK30	TaMAPKK5	4565.A0A3B6RL73	4565.A0A3B6IMW7	0	0	0.116	0.616	0	0.705	0.583	0.146	0.894
TaMAPK30	TaMAPKK7	4565.A0A3B6RL73	4565.A0A3B6INV0	0	0	0.122	0.623	0	0.705	0.583	0.146	0.895

TaMAPK30	TaMEKK2	4565.A0A3B6RL73	4565.A0A3B6JCC4	0	0	0.131	0.596	0	0.222	0.186	0.065	0.417
TaMAPK30	TaMAPKK11	4565.A0A3B6RL73	4565.A0A3B6JEH0	0	0	0.118	0.624	0	0.705	0.583	0.146	0.895
TaMAPK30	TaMAPKK12	4565.A0A3B6RL73	4565.A0A3B6JG06	0	0	0.124	0.619	0	0.705	0.583	0.146	0.895
TaMAPK30	TaMEKK14	4565.A0A3B6RL73	4565.A0A3B6KF43	0	0	0.145	0.609	0	0.222	0.186	0.065	0.426
TaMAPK30	TaMAPKK4	4565.A0A3B6RL73	4565.A0A3B6KFB5	0	0	0.103	0.626	0	0.705	0.583	0.146	0.893
TaMAPK30	TaMEKK17	4565.A0A3B6RL73	4565.A0A3B6KFL8	0	0	0.16	0.609	0	0.27	0.063	0.088	0.406
TaMAPK30	TaMEKK15	4565.A0A3B6RL73	4565.A0A3B6KPK7	0	0	0.142	0.594	0	0.222	0.186	0.065	0.424
TaMAPK30	TaMAPKK18	4565.A0A3B6RL73	4565.A0A3B6LJ27	0	0	0	0.592	0	0.705	0.583	0.146	0.885
TaMAPK30	TaMEKK24	4565.A0A3B6RL73	4565.A0A3B6LLV5	0	0	0.159	0.609	0	0.27	0.063	0.088	0.405
TaMAPK30	TaMEKK16	4565.A0A3B6RL73	4565.A0A3B6LW00	0	0	0.141	0.593	0	0.222	0.186	0.065	0.423
TaMAPK30	TaMAPKK2	4565.A0A3B6RL73	4565.A0A3B6LYW0	0	0	0	0.594	0	0.705	0.583	0.146	0.885
TaMAPK30	TaMAPKK3	4565.A0A3B6RL73	4565.A0A3B6MNP8	0	0	0	0.593	0	0.705	0.583	0.146	0.885
TaMAPK30	TaMEKK29	4565.A0A3B6RL73	4565.A0A3B6MSP6	0	0	0.161	0.608	0	0.27	0.063	0.088	0.406
TaMAPK30	TaMEKK11	4565.A0A3B6RL73	4565.A0A3B6N0D8	0	0	0.141	0.591	0	0.222	0.186	0.065	0.423
TaMAPK30	TaMAPKK13	4565.A0A3B6RL73	4565.A0A3B6N2X8	0	0	0	0.594	0	0.705	0.583	0.146	0.885
TaMAPK30	TaMAPKK1	4565.A0A3B6RL73	4565.A0A3B6QJ87	0	0	0.132	0.625	0	0.705	0.583	0.146	0.896
TaMAPK30	TaRaf87	4565.A0A3B6RL73	4565.A0A3B6QMZ9	0	0	0	0.585	0.277	0.134	0.389	0.088	0.604
TaMAPK33	TaMAPKK9	4565.A0A3B6A1J3	4565.A0A077RVQ4	0	0	0.118	0.617	0	0.705	0.583	0.146	0.894

TaMAPK33	TaRaf88	4565.A0A3B6A1J3	4565.A0A3B5Z5X1	0	0	0.254	0	0	0.15	0.177	0.05	0.437
TaMAPK33	TaMEKK2	4565.A0A3B6A1J3	4565.A0A3B6JCC4	0	0	0.128	0.577	0	0.222	0.186	0.065	0.414
TaMAPK33	TaMEKK24	4565.A0A3B6A1J3	4565.A0A3B6LLV5	0	0	0.174	0.581	0	0.27	0.063	0.088	0.416
TaMAPK33	TaMEKK29	4565.A0A3B6A1J3	4565.A0A3B6MSP6	0	0	0.175	0.581	0	0.27	0.063	0.088	0.416
TaMAPK33	TaMEKK17	4565.A0A3B6A1J3	4565.A0A3B6KFL8	0	0	0.175	0.581	0	0.27	0.063	0.088	0.416
TaMAPK33	TaMEKK11	4565.A0A3B6A1J3	4565.A0A3B6N0D8	0	0	0.136	0.572	0	0.222	0.186	0.065	0.419
TaMAPK33	TaMEKK16	4565.A0A3B6A1J3	4565.A0A3B6LW00	0	0	0.136	0.574	0	0.222	0.186	0.065	0.42
TaMAPK33	TaMEKK15	4565.A0A3B6A1J3	4565.A0A3B6KPK7	0	0	0.138	0.574	0	0.222	0.186	0.065	0.421
TaMAPK33	TaMEKK5	4565.A0A3B6A1J3	4565.A0A3B6AWC1	0	0	0.147	0.579	0	0.222	0.186	0.065	0.427
TaMAPK33	TaMEKK14	4565.A0A3B6A1J3	4565.A0A3B6KF43	0	0	0.15	0.582	0	0.222	0.186	0.065	0.429
TaMAPK33	TaRaf30	4565.A0A3B6A1J3	4565.A0A3B6A1Z4	0	0	0.255	0	0	0.15	0.177	0.05	0.438
TaMAPK33	TaRaf87	4565.A0A3B6A1J3	4565.A0A3B6QMZ9	0	0	0	0.569	0.277	0.134	0.389	0.088	0.604
TaMAPK33	TaMAPKK2	4565.A0A3B6A1J3	4565.A0A3B6LYW0	0	0	0	0.583	0	0.705	0.583	0.146	0.885
TaMAPK33	TaMAPKK3	4565.A0A3B6A1J3	4565.A0A3B6MNP8	0	0	0	0.578	0	0.705	0.583	0.146	0.885
TaMAPK33	TaMAPKK18	4565.A0A3B6A1J3	4565.A0A3B6LJ27	0	0	0	0.583	0	0.705	0.583	0.146	0.885
TaMAPK33	TaMAPKK13	4565.A0A3B6A1J3	4565.A0A3B6N2X8	0	0	0	0.581	0	0.705	0.583	0.146	0.885
TaMAPK33	TaMAPKK4	4565.A0A3B6A1J3	4565.A0A3B6KFB5	0	0	0.102	0.634	0	0.705	0.583	0.146	0.893
TaMAPK33	TaMAPKK8	4565.A0A3B6A1J3	4565.A0A3B6IK39	0	0	0.115	0.621	0	0.705	0.583	0.146	0.894

TaMAPK33	TaMAPKK15	4565.A0A3B6A1J3	4565.A0A3B6HW51	0	0	0.12	0.62	0	0.705	0.583	0.146	0.895
TaMAPK33	TaMAPKK7	4565.A0A3B6A1J3	4565.A0A3B6INV0	0	0	0.121	0.617	0	0.705	0.583	0.146	0.895
TaMAPK33	TaMAPKK5	4565.A0A3B6A1J3	4565.A0A3B6IMW7	0	0	0.119	0.607	0	0.705	0.583	0.146	0.895
TaMAPK33	TaMAPKK16	4565.A0A3B6A1J3	4565.A0A3B6HZP7	0	0	0.12	0.621	0	0.705	0.583	0.146	0.895
TaMAPK33	TaMAPKK6	4565.A0A3B6A1J3	4565.A0A3B6ILF0	0	0	0.12	0.616	0	0.705	0.583	0.146	0.895
TaMAPK33	TaMAPKK17	4565.A0A3B6A1J3	4565.A0A3B6I0M7	0	0	0.119	0.618	0	0.705	0.583	0.146	0.895
TaMAPK33	TaMAPKK14	4565.A0A3B6A1J3	4565.A0A3B6HY95	0	0	0.12	0.616	0	0.705	0.583	0.146	0.895
TaMAPK33	TaMAPKK11	4565.A0A3B6A1J3	4565.A0A3B6JEH0	0	0	0.12	0.62	0	0.705	0.583	0.146	0.895
TaMAPK33	TaMAPKK12	4565.A0A3B6A1J3	4565.A0A3B6JG06	0	0	0.123	0.613	0	0.705	0.583	0.146	0.895
TaMAPK33	TaMAPKK1	4565.A0A3B6A1J3	4565.A0A3B6QJ87	0	0	0.129	0.612	0	0.705	0.583	0.146	0.896
TaMAPK34	TaMAPKK9	4565.A0A3B6A3E4	4565.A0A077RVQ4	0	0	0.117	0.619	0	0.705	0.583	0.146	0.894
TaMAPK34	TaMAPK43	4565.A0A3B6A3E4	4565.A0A3B5Y7D4	0	0	0.047	0.987	0	0	0.54	0	0.543
TaMAPK34	TaRaf30	4565.A0A3B6A3E4	4565.A0A3B6A1Z4	0	0	0.235	0	0	0.15	0.177	0.05	0.423
TaMAPK34	TaRaf14	4565.A0A3B6A3E4	4565.A0A3B6TYA5	0	0	0.213	0	0.041	0.15	0.177	0	0.401
TaMAPK34	TaMEKK2	4565.A0A3B6A3E4	4565.A0A3B6JCC4	0	0	0.128	0.574	0	0.222	0.186	0.065	0.414
TaMAPK34	TaMEKK11	4565.A0A3B6A3E4	4565.A0A3B6N0D8	0	0	0.132	0.572	0	0.222	0.186	0.065	0.417
TaMAPK34	TaMEKK16	4565.A0A3B6A3E4	4565.A0A3B6LW00	0	0	0.133	0.573	0	0.222	0.186	0.065	0.418
TaMAPK34	TaMEKK17	4565.A0A3B6A3E4	4565.A0A3B6KFL8	0	0	0.177	0.575	0	0.27	0.063	0.088	0.418

TaMAPK34	TaMEKK24	4565.A0A3B6A3E4	4565.A0A3B6LLV5	0	0	0.177	0.575	0	0.27	0.063	0.088	0.418
TaMAPK34	TaMEKK29	4565.A0A3B6A3E4	4565.A0A3B6MSP6	0	0	0.177	0.575	0	0.27	0.063	0.088	0.418
TaMAPK34	TaMEKK15	4565.A0A3B6A3E4	4565.A0A3B6KPK7	0	0	0.135	0.574	0	0.222	0.186	0.065	0.419
TaMAPK34	TaMEKK5	4565.A0A3B6A3E4	4565.A0A3B6AWC1	0	0	0.14	0.582	0	0.222	0.186	0.065	0.423
TaMAPK34	TaMEKK14	4565.A0A3B6A3E4	4565.A0A3B6KF43	0	0	0.142	0.582	0	0.222	0.186	0.065	0.424
TaMAPK34	TaRaf87	4565.A0A3B6A3E4	4565.A0A3B6QMZ9	0	0	0	0.56	0.277	0.134	0.389	0.088	0.604
TaMAPK34	TaMAPKK18	4565.A0A3B6A3E4	4565.A0A3B6LJ27	0	0	0	0.589	0	0.705	0.583	0.146	0.885
TaMAPK34	TaMAPKK13	4565.A0A3B6A3E4	4565.A0A3B6N2X8	0	0	0	0.583	0	0.705	0.583	0.146	0.885
TaMAPK34	TaMAPKK3	4565.A0A3B6A3E4	4565.A0A3B6MNP8	0	0	0	0.583	0	0.705	0.583	0.146	0.885
TaMAPK34	TaMAPKK2	4565.A0A3B6A3E4	4565.A0A3B6LYW0	0	0	0	0.585	0	0.705	0.583	0.146	0.885
TaMAPK34	TaMAPKK4	4565.A0A3B6A3E4	4565.A0A3B6KFB5	0	0	0.097	0.644	0	0.705	0.583	0.146	0.892
TaMAPK34	TaMAPKK11	4565.A0A3B6A3E4	4565.A0A3B6JEH0	0	0	0.118	0.615	0	0.705	0.583	0.146	0.894
TaMAPK34	TaMAPKK8	4565.A0A3B6A3E4	4565.A0A3B6IK39	0	0	0.117	0.612	0	0.705	0.583	0.146	0.894
TaMAPK34	TaMAPKK12	4565.A0A3B6A3E4	4565.A0A3B6JG06	0	0	0.122	0.607	0	0.705	0.583	0.146	0.895
TaMAPK34	TaMAPKK14	4565.A0A3B6A3E4	4565.A0A3B6HY95	0	0	0.119	0.612	0	0.705	0.583	0.146	0.895
TaMAPK34	TaMAPKK6	4565.A0A3B6A3E4	4565.A0A3B6ILF0	0	0	0.121	0.608	0	0.705	0.583	0.146	0.895
TaMAPK34	TaMAPKK16	4565.A0A3B6A3E4	4565.A0A3B6HZP7	0	0	0.12	0.616	0	0.705	0.583	0.146	0.895
TaMAPK34	TaMAPKK5	4565.A0A3B6A3E4	4565.A0A3B6IMW7	0	0	0.12	0.609	0	0.705	0.583	0.146	0.895

TaMAPK34	TaMAPKK17	4565.A0A3B6A3E4	4565.A0A3B6I0M7	0	0	0.12	0.612	0	0.705	0.583	0.146	0.895
TaMAPK34	TaMAPKK7	4565.A0A3B6A3E4	4565.A0A3B6INV0	0	0	0.123	0.61	0	0.705	0.583	0.146	0.895
TaMAPK34	TaMAPKK15	4565.A0A3B6A3E4	4565.A0A3B6HW51	0	0	0.121	0.617	0	0.705	0.583	0.146	0.895
TaMAPK34	TaMAPKK1	4565.A0A3B6A3E4	4565.A0A3B6QJ87	0	0	0.127	0.605	0	0.705	0.583	0.146	0.896
TaMAPK35	TaMAPKK9	4565.A0A3B6NJX2	4565.A0A077RVQ4	0	0	0.144	0.619	0	0.705	0.583	0.146	0.898
TaMAPK35	TaMEKK5	4565.A0A3B6NJX2	4565.A0A3B6AWC1	0	0	0.173	0.574	0	0.222	0.186	0.065	0.445
TaMAPK35	TaMEKK1	4565.A0A3B6NJX2	4565.A0A3B6B3I4	0	0	0.16	0.615	0	0.27	0.063	0.088	0.406
TaMAPK35	TaMAPKK15	4565.A0A3B6NJX2	4565.A0A3B6HW51	0	0	0.151	0.613	0	0.705	0.583	0.146	0.898
TaMAPK35	TaMAPKK14	4565.A0A3B6NJX2	4565.A0A3B6HY95	0	0	0.15	0.607	0	0.705	0.583	0.146	0.898
TaMAPK35	TaMAPKK16	4565.A0A3B6NJX2	4565.A0A3B6HZP7	0	0	0.146	0.62	0	0.705	0.583	0.146	0.898
TaMAPK35	TaMAPKK17	4565.A0A3B6NJX2	4565.A0A3B6I0M7	0	0	0.146	0.602	0	0.705	0.583	0.146	0.898
TaMAPK35	TaMAPKK8	4565.A0A3B6NJX2	4565.A0A3B6IK39	0	0	0.14	0.613	0	0.705	0.583	0.146	0.897
TaMAPK35	TaMAPKK6	4565.A0A3B6NJX2	4565.A0A3B6ILF0	0	0	0.141	0.612	0	0.705	0.583	0.146	0.897
TaMAPK35	TaMAPKK5	4565.A0A3B6NJX2	4565.A0A3B6IMW7	0	0	0.146	0.604	0	0.705	0.583	0.146	0.898
TaMAPK35	TaMAPKK7	4565.A0A3B6NJX2	4565.A0A3B6INV0	0	0	0.147	0.602	0	0.705	0.583	0.146	0.898
TaMAPK35	TaMEKK2	4565.A0A3B6NJX2	4565.A0A3B6JCC4	0	0	0.152	0.574	0	0.222	0.186	0.065	0.431
TaMAPK35	TaMAPKK11	4565.A0A3B6NJX2	4565.A0A3B6JEH0	0	0	0.144	0.618	0	0.705	0.583	0.146	0.898
TaMAPK35	TaMAPKK12	4565.A0A3B6NJX2	4565.A0A3B6JG06	0	0	0.149	0.6	0	0.705	0.583	0.146	0.898

TaMAPK35	TaMEKK14	4565.A0A3B6NJX2	4565.A0A3B6KF43	0	0	0.17	0.578	0	0.222	0.186	0.065	0.443
TaMAPK35	TaMAPKK4	4565.A0A3B6NJX2	4565.A0A3B6KFB5	0	0	0.117	0.628	0	0.705	0.612	0.146	0.902
TaMAPK35	TaMEKK17	4565.A0A3B6NJX2	4565.A0A3B6KFL8	0	0	0.178	0.607	0	0.27	0.063	0.088	0.419
TaMAPK35	TaMEKK15	4565.A0A3B6NJX2	4565.A0A3B6KPK7	0	0	0.165	0.575	0	0.222	0.186	0.065	0.44
TaMAPK35	TaMAPKK18	4565.A0A3B6NJX2	4565.A0A3B6LJ27	0	0	0.115	0.581	0	0.705	0.612	0.146	0.901
TaMAPK35	TaMEKK24	4565.A0A3B6NJX2	4565.A0A3B6LLV5	0	0	0.178	0.607	0	0.27	0.063	0.088	0.418
TaMAPK35	TaMEKK16	4565.A0A3B6NJX2	4565.A0A3B6LW00	0	0	0.165	0.575	0	0.222	0.186	0.065	0.439
TaMAPK35	TaMAPKK2	4565.A0A3B6NJX2	4565.A0A3B6LYW0	0	0	0	0.579	0	0.705	0.612	0.146	0.893
TaMAPK35	TaMAPKK3	4565.A0A3B6NJX2	4565.A0A3B6MNP8	0	0	0	0.578	0	0.705	0.612	0.146	0.893
TaMAPK35	TaMEKK29	4565.A0A3B6NJX2	4565.A0A3B6MSP6	0	0	0.179	0.607	0	0.27	0.063	0.088	0.419
TaMAPK35	TaMEKK11	4565.A0A3B6NJX2	4565.A0A3B6N0D8	0	0	0.163	0.573	0	0.222	0.186	0.065	0.438
TaMAPK35	TaMAPKK13	4565.A0A3B6NJX2	4565.A0A3B6N2X8	0	0	0	0.579	0	0.705	0.612	0.146	0.893
TaMAPK35	TaMEKK4	4565.A0A3B6NJX2	4565.A0A3B6NRN9	0	0	0.161	0.597	0	0.27	0.063	0.088	0.407
TaMAPK35	TaMEKK4-1	4565.A0A3B6NJX2	4565.A0A3B6PNI6	0	0	0.164	0.599	0	0.27	0.063	0.088	0.408
TaMAPK35	TaRaf21	4565.A0A3B6NJX2	4565.A0A3B6TVC5	0	0	0.229	0	0	0.15	0.177	0.05	0.419
TaMAPK35	TaRaf87	4565.A0A3B6NJX2	4565.A0A3B6QMZ9	0	0	0	0.578	0.3	0.134	0.389	0.088	0.617
TaMAPK35	TaMAPKK1	4565.A0A3B6NJX2	4565.A0A3B6QJ87	0	0	0.147	0.612	0	0.705	0.583	0.146	0.898
TaMAPK36	TaMAPKK9	4565.A0A3B6NN33	4565.A0A077RVQ4	0	0	0	0.661	0	0.705	0.583	0.146	0.885

TaMAPK36	TaMAPKK15	4565.A0A3B6NN33	4565.A0A3B6HW51	0	0	0	0.653	0	0.705	0.583	0.146	0.885
TaMAPK36	TaMAPKK14	4565.A0A3B6NN33	4565.A0A3B6HY95	0	0	0	0.644	0	0.705	0.583	0.146	0.885
TaMAPK36	TaMAPKK16	4565.A0A3B6NN33	4565.A0A3B6HZP7	0	0	0.095	0.661	0	0.705	0.583	0.146	0.892
TaMAPK36	TaMAPKK17	4565.A0A3B6NN33	4565.A0A3B6I0M7	0	0	0	0.633	0	0.705	0.583	0.146	0.885
TaMAPK36	TaMAPKK8	4565.A0A3B6NN33	4565.A0A3B6IK39	0	0	0.097	0.651	0	0.705	0.583	0.146	0.892
TaMAPK36	TaMAPKK6	4565.A0A3B6NN33	4565.A0A3B6ILF0	0	0	0	0.632	0	0.705	0.583	0.146	0.885
TaMAPK36	TaMAPKK5	4565.A0A3B6NN33	4565.A0A3B6IMW7	0	0	0	0.636	0	0.705	0.583	0.146	0.885
TaMAPK36	TaMAPKK7	4565.A0A3B6NN33	4565.A0A3B6INV0	0	0	0	0.634	0	0.705	0.583	0.146	0.885
TaMAPK36	TaMAPKK11	4565.A0A3B6NN33	4565.A0A3B6JEH0	0	0	0.102	0.654	0	0.705	0.583	0.146	0.893
TaMAPK36	TaMAPKK12	4565.A0A3B6NN33	4565.A0A3B6JG06	0	0	0	0.626	0	0.705	0.583	0.146	0.885
TaMAPK36	TaMEKK14	4565.A0A3B6NN33	4565.A0A3B6KF43	0	0	0.107	0.608	0	0.222	0.186	0.065	0.4
TaMAPK36	TaMAPKK4	4565.A0A3B6NN33	4565.A0A3B6KFB5	0	0	0	0.605	0	0.705	0.825	0.195	0.954
TaMAPK36	TaMAPKK18	4565.A0A3B6NN33	4565.A0A3B6LJ27	0	0	0	0.588	0	0.705	0.825	0.195	0.954
TaMAPK36	TaMAPKK2	4565.A0A3B6NN33	4565.A0A3B6LYW0	0	0	0	0.593	0	0.705	0.825	0.195	0.954
TaMAPK36	TaMAPKK3	4565.A0A3B6NN33	4565.A0A3B6MNP8	0	0	0	0.586	0	0.705	0.825	0.195	0.954
TaMAPK36	TaMAPKK13	4565.A0A3B6NN33	4565.A0A3B6N2X8	0	0	0	0.589	0	0.705	0.825	0.195	0.954
TaMAPK36	TaRaf87	4565.A0A3B6NN33	4565.A0A3B6QMZ9	0	0	0	0.58	0.16	0.134	0.389	0.088	0.54
TaMAPK36	TaMAPK54	4565.A0A3B6NN33	4565.A0A3B6SKC9	0	0	0.048	0.984	0	0	0.793	0	0.794

TaMAPK36	TaMAPK47	4565.A0A3B6NN33	4565.A0A3B6RAZ7	0	0	0.053	0.974	0	0	0.793	0	0.795
TaMAPK36	TaMAPKK1	4565.A0A3B6NN33	4565.A0A3B6QJ87	0	0	0.106	0.608	0	0.705	0.583	0.146	0.893
TaMAPK38	TaMAPKK9	4565.A0A3B6FMN5	4565.A0A077RVQ4	0	0	0.112	0.617	0	0.705	0.583	0.146	0.894
TaMAPK38	TaMEKK5	4565.A0A3B6FMN5	4565.A0A3B6AWC1	0	0	0.14	0.584	0	0.222	0.186	0.065	0.422
TaMAPK38	TaMEKK2	4565.A0A3B6FMN5	4565.A0A3B6JCC4	0	0	0.127	0.575	0	0.222	0.186	0.065	0.414
TaMAPK38	TaMEKK16	4565.A0A3B6FMN5	4565.A0A3B6LW00	0	0	0.129	0.578	0	0.222	0.186	0.065	0.415
TaMAPK38	TaMEKK11	4565.A0A3B6FMN5	4565.A0A3B6N0D8	0	0	0.129	0.576	0	0.222	0.186	0.065	0.415
TaMAPK38	TaMEKK15	4565.A0A3B6FMN5	4565.A0A3B6KPK7	0	0	0.13	0.579	0	0.222	0.186	0.065	0.416
TaMAPK38	TaMEKK24	4565.A0A3B6FMN5	4565.A0A3B6LLV5	0	0	0.178	0.579	0	0.27	0.063	0.088	0.418
TaMAPK38	TaMEKK17	4565.A0A3B6FMN5	4565.A0A3B6KFL8	0	0	0.179	0.579	0	0.27	0.063	0.088	0.419
TaMAPK38	TaMEKK29	4565.A0A3B6FMN5	4565.A0A3B6MSP6	0	0	0.179	0.579	0	0.27	0.063	0.088	0.419
TaMAPK38	TaMEKK14	4565.A0A3B6FMN5	4565.A0A3B6KF43	0	0	0.139	0.586	0	0.222	0.186	0.065	0.422
TaMAPK38	TaRaf87	4565.A0A3B6FMN5	4565.A0A3B6QMZ9	0	0	0	0.562	0.277	0.134	0.389	0.088	0.604
TaMAPK38	TaMAPKK4	4565.A0A3B6FMN5	4565.A0A3B6KFB5	0	0	0	0.647	0	0.705	0.583	0.146	0.885
TaMAPK38	TaMAPKK18	4565.A0A3B6FMN5	4565.A0A3B6LJ27	0	0	0	0.589	0	0.705	0.583	0.146	0.885
TaMAPK38	TaMAPKK13	4565.A0A3B6FMN5	4565.A0A3B6N2X8	0	0	0	0.581	0	0.705	0.583	0.146	0.885
TaMAPK38	TaMAPKK2	4565.A0A3B6FMN5	4565.A0A3B6LYW0	0	0	0	0.585	0	0.705	0.583	0.146	0.885
TaMAPK38	TaMAPKK3	4565.A0A3B6FMN5	4565.A0A3B6MNP8	0	0	0	0.582	0	0.705	0.583	0.146	0.885

TaMAPK38	TaMAPKK14	4565.A0A3B6FMN5	4565.A0A3B6HY95	0	0	0.115	0.607	0	0.705	0.583	0.146	0.894
TaMAPK38	TaMAPKK11	4565.A0A3B6FMN5	4565.A0A3B6JEH0	0	0	0.115	0.614	0	0.705	0.583	0.146	0.894
TaMAPK38	TaMAPKK15	4565.A0A3B6FMN5	4565.A0A3B6HW51	0	0	0.117	0.614	0	0.705	0.583	0.146	0.894
TaMAPK38	TaMAPKK5	4565.A0A3B6FMN5	4565.A0A3B6IMW7	0	0	0.115	0.606	0	0.705	0.583	0.146	0.894
TaMAPK38	TaMAPKK16	4565.A0A3B6FMN5	4565.A0A3B6HZP7	0	0	0.115	0.616	0	0.705	0.583	0.146	0.894
TaMAPK38	TaMAPKK8	4565.A0A3B6FMN5	4565.A0A3B6IK39	0	0	0.111	0.616	0	0.705	0.583	0.146	0.894
TaMAPK38	TaMAPKK6	4565.A0A3B6FMN5	4565.A0A3B6ILF0	0	0	0.118	0.607	0	0.705	0.583	0.146	0.894
TaMAPK38	TaMAPKK17	4565.A0A3B6FMN5	4565.A0A3B6I0M7	0	0	0.117	0.611	0	0.705	0.583	0.146	0.894
TaMAPK38	TaMAPKK12	4565.A0A3B6FMN5	4565.A0A3B6JG06	0	0	0.121	0.605	0	0.705	0.583	0.146	0.895
TaMAPK38	TaMAPKK7	4565.A0A3B6FMN5	4565.A0A3B6INV0	0	0	0.12	0.609	0	0.705	0.583	0.146	0.895
TaMAPK38	TaMAPKK1	4565.A0A3B6FMN5	4565.A0A3B6QJ87	0	0	0.129	0.611	0	0.705	0.583	0.146	0.896
TaMAPK39	TaMAPKK9	4565.A0A3B6EGM9	4565.A0A077RVQ4	0	0	0.115	0.619	0	0.705	0.583	0.146	0.894
TaMAPK39	TaRaf88	4565.A0A3B6EGM9	4565.A0A3B5Z5X1	0	0	0.231	0	0	0.15	0.177	0.05	0.42
TaMAPK39	TaRaf30	4565.A0A3B6EGM9	4565.A0A3B6A1Z4	0	0	0.232	0	0	0.15	0.177	0.05	0.421
TaMAPK39	TaMEKK5	4565.A0A3B6EGM9	4565.A0A3B6AWC1	0	0	0.149	0.576	0	0.222	0.186	0.065	0.429
TaMAPK39	TaMEKK2	4565.A0A3B6EGM9	4565.A0A3B6JCC4	0	0	0.131	0.579	0	0.222	0.186	0.065	0.416
TaMAPK39	TaMEKK16	4565.A0A3B6EGM9	4565.A0A3B6LW00	0	0	0.137	0.571	0	0.222	0.186	0.065	0.42
TaMAPK39	TaMEKK11	4565.A0A3B6EGM9	4565.A0A3B6N0D8	0	0	0.136	0.57	0	0.222	0.186	0.065	0.42

TaMAPK39	TaMEKK15	4565.A0A3B6EGM9	4565.A0A3B6KPK7	0	0	0.138	0.572	0	0.222	0.186	0.065	0.421
TaMAPK39	TaMEKK17	4565.A0A3B6EGM9	4565.A0A3B6KFL8	0	0	0.189	0.573	0	0.27	0.063	0.088	0.426
TaMAPK39	TaMEKK24	4565.A0A3B6EGM9	4565.A0A3B6LLV5	0	0	0.188	0.573	0	0.27	0.063	0.088	0.426
TaMAPK39	TaMEKK29	4565.A0A3B6EGM9	4565.A0A3B6MSP6	0	0	0.189	0.572	0	0.27	0.063	0.088	0.426
TaMAPK39	TaMEKK14	4565.A0A3B6EGM9	4565.A0A3B6KF43	0	0	0.151	0.583	0	0.222	0.186	0.065	0.43
TaMAPK39	TaRaf87	4565.A0A3B6EGM9	4565.A0A3B6QMZ9	0	0	0	0.565	0.277	0.134	0.389	0.088	0.604
TaMAPK39	TaMAPKK4	4565.A0A3B6EGM9	4565.A0A3B6KFB5	0	0	0	0.638	0	0.705	0.583	0.146	0.885
TaMAPK39	TaMAPKK18	4565.A0A3B6EGM9	4565.A0A3B6LJ27	0	0	0	0.586	0	0.705	0.583	0.146	0.885
TaMAPK39	TaMAPKK13	4565.A0A3B6EGM9	4565.A0A3B6N2X8	0	0	0	0.587	0	0.705	0.583	0.146	0.885
TaMAPK39	TaMAPKK2	4565.A0A3B6EGM9	4565.A0A3B6LYW0	0	0	0	0.587	0	0.705	0.583	0.146	0.885
TaMAPK39	TaMAPKK3	4565.A0A3B6EGM9	4565.A0A3B6MNP8	0	0	0	0.581	0	0.705	0.583	0.146	0.885
TaMAPK39	TaMAPKK14	4565.A0A3B6EGM9	4565.A0A3B6HY95	0	0	0.117	0.616	0	0.705	0.583	0.146	0.894
TaMAPK39	TaMAPKK8	4565.A0A3B6EGM9	4565.A0A3B6IK39	0	0	0.114	0.618	0	0.705	0.583	0.146	0.894
TaMAPK39	TaMAPKK16	4565.A0A3B6EGM9	4565.A0A3B6HZP7	0	0	0.118	0.618	0	0.705	0.583	0.146	0.894
TaMAPK39	TaMAPKK5	4565.A0A3B6EGM9	4565.A0A3B6IMW7	0	0	0.115	0.61	0	0.705	0.583	0.146	0.894
TaMAPK39	TaMAPKK11	4565.A0A3B6EGM9	4565.A0A3B6JEH0	0	0	0.119	0.615	0	0.705	0.583	0.146	0.895
TaMAPK39	TaMAPKK12	4565.A0A3B6EGM9	4565.A0A3B6JG06	0	0	0.125	0.612	0	0.705	0.583	0.146	0.895
TaMAPK39	TaMAPKK7	4565.A0A3B6EGM9	4565.A0A3B6INV0	0	0	0.123	0.615	0	0.705	0.583	0.146	0.895

TaMAPK39	TaMAPKK15	4565.A0A3B6EGM9	4565.A0A3B6HW51	0	0	0.118	0.618	0	0.705	0.583	0.146	0.895
TaMAPK39	TaMAPKK6	4565.A0A3B6EGM9	4565.A0A3B6ILF0	0	0	0.122	0.611	0	0.705	0.583	0.146	0.895
TaMAPK39	TaMAPKK17	4565.A0A3B6EGM9	4565.A0A3B6I0M7	0	0	0.122	0.616	0	0.705	0.583	0.146	0.895
TaMAPK39	TaMAPKK1	4565.A0A3B6EGM9	4565.A0A3B6QJ87	0	0	0.13	0.613	0	0.705	0.583	0.146	0.896
TaMAPK4	TaMAPKK9	4565.A0A3B5ZRD7	4565.A0A077RVQ4	0	0	0.126	0.622	0	0.705	0.583	0.146	0.895
TaMAPK4	TaMEKK29	4565.A0A3B5ZRD7	4565.A0A3B6MSP6	0	0	0.178	0.589	0	0.27	0.063	0.088	0.418
TaMAPK4	TaMEKK24	4565.A0A3B5ZRD7	4565.A0A3B6LLV5	0	0	0.177	0.589	0	0.27	0.063	0.088	0.418
TaMAPK4	TaMEKK17	4565.A0A3B5ZRD7	4565.A0A3B6KFL8	0	0	0.178	0.589	0	0.27	0.063	0.088	0.418
TaMAPK4	TaMEKK2	4565.A0A3B5ZRD7	4565.A0A3B6JCC4	0	0	0.135	0.568	0	0.222	0.186	0.065	0.419
TaMAPK4	TaMEKK11	4565.A0A3B5ZRD7	4565.A0A3B6N0D8	0	0	0.148	0.57	0	0.222	0.186	0.065	0.428
TaMAPK4	TaMEKK16	4565.A0A3B5ZRD7	4565.A0A3B6LW00	0	0	0.149	0.572	0	0.222	0.186	0.065	0.429
TaMAPK4	TaMEKK14	4565.A0A3B5ZRD7	4565.A0A3B6KF43	0	0	0.152	0.581	0	0.222	0.186	0.065	0.43
TaMAPK4	TaMEKK15	4565.A0A3B5ZRD7	4565.A0A3B6KPK7	0	0	0.151	0.573	0	0.222	0.186	0.065	0.43
TaMAPK4	TaMEKK5	4565.A0A3B5ZRD7	4565.A0A3B6AWC1	0	0	0.158	0.574	0	0.222	0.186	0.065	0.435
TaMAPK4	TaRaf87	4565.A0A3B5ZRD7	4565.A0A3B6QMZ9	0	0	0	0.565	0.3	0.134	0.389	0.088	0.617
TaMAPK4	TaMAPKK8	4565.A0A3B5ZRD7	4565.A0A3B6IK39	0	0	0.123	0.615	0	0.705	0.583	0.146	0.895
TaMAPK4	TaMAPKK15	4565.A0A3B5ZRD7	4565.A0A3B6HW51	0	0	0.133	0.615	0	0.705	0.583	0.146	0.896
TaMAPK4	TaMAPKK5	4565.A0A3B5ZRD7	4565.A0A3B6IMW7	0	0	0.127	0.609	0	0.705	0.583	0.146	0.896

TaMAPK4	TaMAPKK16	4565.A0A3B5ZRD7	4565.A0A3B6HZP7	0	0	0.131	0.621	0	0.705	0.583	0.146	0.896
TaMAPK4	TaMAPKK1	4565.A0A3B5ZRD7	4565.A0A3B6QJ87	0	0	0.134	0.615	0	0.705	0.583	0.146	0.896
TaMAPK4	TaMAPKK14	4565.A0A3B5ZRD7	4565.A0A3B6HY95	0	0	0.133	0.61	0	0.705	0.583	0.146	0.896
TaMAPK4	TaMAPKK11	4565.A0A3B5ZRD7	4565.A0A3B6JEH0	0	0	0.127	0.62	0	0.705	0.583	0.146	0.896
TaMAPK4	TaMAPKK7	4565.A0A3B5ZRD7	4565.A0A3B6INV0	0	0	0.138	0.611	0	0.705	0.583	0.146	0.897
TaMAPK4	TaMAPKK17	4565.A0A3B5ZRD7	4565.A0A3B6I0M7	0	0	0.136	0.613	0	0.705	0.583	0.146	0.897
TaMAPK4	TaMAPKK6	4565.A0A3B5ZRD7	4565.A0A3B6ILF0	0	0	0.138	0.607	0	0.705	0.583	0.146	0.897
TaMAPK4	TaMAPKK12	4565.A0A3B5ZRD7	4565.A0A3B6JG06	0	0	0.144	0.608	0	0.705	0.583	0.146	0.898
TaMAPK4	TaMAPKK13	4565.A0A3B5ZRD7	4565.A0A3B6N2X8	0	0	0.108	0.58	0	0.705	0.612	0.146	0.901
TaMAPK4	TaMAPKK2	4565.A0A3B5ZRD7	4565.A0A3B6LYW0	0	0	0.123	0.581	0	0.705	0.612	0.146	0.902
TaMAPK4	TaMAPKK3	4565.A0A3B5ZRD7	4565.A0A3B6MNP8	0	0	0.131	0.577	0	0.705	0.612	0.146	0.903
TaMAPK4	TaMAPKK4	4565.A0A3B5ZRD7	4565.A0A3B6KFB5	0	0	0.13	0.629	0	0.705	0.612	0.146	0.903
TaMAPK4	TaMAPKK18	4565.A0A3B5ZRD7	4565.A0A3B6LJ27	0	0	0.137	0.582	0	0.705	0.612	0.146	0.904
TaMAPK40	TaMAPKK9	4565.A0A3B6EH95	4565.A0A077RVQ4	0	0	0.114	0.622	0	0.705	0.583	0.146	0.894
TaMAPK40	TaRaf88	4565.A0A3B6EH95	4565.A0A3B5Z5X1	0	0	0.29	0	0	0.15	0.177	0.05	0.465
TaMAPK40	TaRaf30	4565.A0A3B6EH95	4565.A0A3B6A1Z4	0	0	0.292	0	0	0.15	0.177	0.05	0.466
TaMAPK40	TaMEKK5	4565.A0A3B6EH95	4565.A0A3B6AWC1	0	0	0.151	0.586	0	0.222	0.186	0.065	0.43
TaMAPK40	TaMEKK24	4565.A0A3B6EH95	4565.A0A3B6LLV5	0	0	0.156	0.609	0	0.27	0.063	0.088	0.403

TaMAPK40	TaMEKK29	4565.A0A3B6EH95	4565.A0A3B6MSP6	0	0	0.157	0.609	0	0.27	0.063	0.088	0.404
TaMAPK40	TaMEKK17	4565.A0A3B6EH95	4565.A0A3B6KFL8	0	0	0.157	0.609	0	0.27	0.063	0.088	0.404
TaMAPK40	TaMEKK2	4565.A0A3B6EH95	4565.A0A3B6JCC4	0	0	0.135	0.582	0	0.222	0.186	0.065	0.419
TaMAPK40	TaMEKK11	4565.A0A3B6EH95	4565.A0A3B6N0D8	0	0	0.144	0.583	0	0.222	0.186	0.065	0.425
TaMAPK40	TaMEKK15	4565.A0A3B6EH95	4565.A0A3B6KPK7	0	0	0.146	0.585	0	0.222	0.186	0.065	0.426
TaMAPK40	TaMEKK16	4565.A0A3B6EH95	4565.A0A3B6LW00	0	0	0.145	0.585	0	0.222	0.186	0.065	0.426
TaMAPK40	TaMEKK14	4565.A0A3B6EH95	4565.A0A3B6KF43	0	0	0.151	0.594	0	0.222	0.186	0.065	0.43
TaMAPK40	TaRaf87	4565.A0A3B6EH95	4565.A0A3B6QMZ9	0	0	0	0.572	0.3	0.134	0.389	0.088	0.617
TaMAPK40	TaMAPKK2	4565.A0A3B6EH95	4565.A0A3B6LYW0	0	0	0	0.582	0	0.705	0.612	0.146	0.893
TaMAPK40	TaMAPKK3	4565.A0A3B6EH95	4565.A0A3B6MNP8	0	0	0	0.58	0	0.705	0.612	0.146	0.893
TaMAPK40	TaMAPKK13	4565.A0A3B6EH95	4565.A0A3B6N2X8	0	0	0	0.582	0	0.705	0.612	0.146	0.893
TaMAPK40	TaMAPKK18	4565.A0A3B6EH95	4565.A0A3B6LJ27	0	0	0	0.579	0	0.705	0.612	0.146	0.893
TaMAPK40	TaMAPKK8	4565.A0A3B6EH95	4565.A0A3B6IK39	0	0	0.112	0.615	0	0.705	0.583	0.146	0.894
TaMAPK40	TaMAPKK5	4565.A0A3B6EH95	4565.A0A3B6IMW7	0	0	0.117	0.609	0	0.705	0.583	0.146	0.894
TaMAPK40	TaMAPKK11	4565.A0A3B6EH95	4565.A0A3B6JEH0	0	0	0.115	0.618	0	0.705	0.583	0.146	0.894
TaMAPK40	TaMAPKK7	4565.A0A3B6EH95	4565.A0A3B6INV0	0	0	0.124	0.611	0	0.705	0.583	0.146	0.895
TaMAPK40	TaMAPKK15	4565.A0A3B6EH95	4565.A0A3B6HW51	0	0	0.12	0.616	0	0.705	0.583	0.146	0.895
TaMAPK40	TaMAPKK17	4565.A0A3B6EH95	4565.A0A3B6I0M7	0	0	0.122	0.613	0	0.705	0.583	0.146	0.895

TaMAPK40	TaMAPKK6	4565.A0A3B6EH95	4565.A0A3B6ILF0	0	0	0.122	0.611	0	0.705	0.583	0.146	0.895
TaMAPK40	TaMAPKK16	4565.A0A3B6EH95	4565.A0A3B6HZP7	0	0	0.118	0.622	0	0.705	0.583	0.146	0.895
TaMAPK40	TaMAPKK14	4565.A0A3B6EH95	4565.A0A3B6HY95	0	0	0.121	0.612	0	0.705	0.583	0.146	0.895
TaMAPK40	TaMAPKK1	4565.A0A3B6EH95	4565.A0A3B6QJ87	0	0	0.128	0.616	0	0.705	0.583	0.146	0.896
TaMAPK40	TaMAPKK12	4565.A0A3B6EH95	4565.A0A3B6JG06	0	0	0.129	0.607	0	0.705	0.583	0.146	0.896
TaMAPK40	TaMAPKK4	4565.A0A3B6EH95	4565.A0A3B6KFB5	0	0	0.108	0.628	0	0.705	0.612	0.146	0.901
TaMAPK41	TaMAPKK9	4565.A0A3B5Y6A3	4565.A0A077RVQ4	0	0	0.119	0.619	0	0.705	0.583	0.146	0.895
TaMAPK41	TaMEKK24	4565.A0A3B5Y6A3	4565.A0A3B6LLV5	0	0	0.177	0.582	0	0.27	0.063	0.088	0.418
TaMAPK41	TaMEKK17	4565.A0A3B5Y6A3	4565.A0A3B6KFL8	0	0	0.178	0.582	0	0.27	0.063	0.088	0.418
TaMAPK41	TaMEKK2	4565.A0A3B5Y6A3	4565.A0A3B6JCC4	0	0	0.133	0.577	0	0.222	0.186	0.065	0.418
TaMAPK41	TaMEKK29	4565.A0A3B5Y6A3	4565.A0A3B6MSP6	0	0	0.178	0.582	0	0.27	0.063	0.088	0.419
TaMAPK41	TaMEKK11	4565.A0A3B5Y6A3	4565.A0A3B6N0D8	0	0	0.142	0.572	0	0.222	0.186	0.065	0.423
TaMAPK41	TaMEKK16	4565.A0A3B5Y6A3	4565.A0A3B6LW00	0	0	0.142	0.573	0	0.222	0.186	0.065	0.424
TaMAPK41	TaMEKK15	4565.A0A3B5Y6A3	4565.A0A3B6KPK7	0	0	0.144	0.574	0	0.222	0.186	0.065	0.425
TaMAPK41	TaMEKK5	4565.A0A3B5Y6A3	4565.A0A3B6AWC1	0	0	0.151	0.58	0	0.222	0.186	0.065	0.43
TaMAPK41	TaMEKK14	4565.A0A3B5Y6A3	4565.A0A3B6KF43	0	0	0.153	0.584	0	0.222	0.186	0.065	0.431
TaMAPK41	TaRaf30	4565.A0A3B5Y6A3	4565.A0A3B6A1Z4	0	0	0.267	0	0	0.15	0.177	0.05	0.447
TaMAPK41	TaRaf88	4565.A0A3B5Y6A3	4565.A0A3B5Z5X1	0	0	0.267	0	0	0.15	0.177	0.05	0.447

TaMAPK41	TaRaf87	4565.A0A3B5Y6A3	4565.A0A3B6QMZ9	0	0	0	0.57	0.277	0.134	0.389	0.088	0.604
TaMAPK41	TaMAPKK3	4565.A0A3B5Y6A3	4565.A0A3B6MNP8	0	0	0	0.579	0	0.705	0.583	0.146	0.885
TaMAPK41	TaMAPKK2	4565.A0A3B5Y6A3	4565.A0A3B6LYW0	0	0	0	0.583	0	0.705	0.583	0.146	0.885
TaMAPK41	TaMAPKK13	4565.A0A3B5Y6A3	4565.A0A3B6N2X8	0	0	0	0.582	0	0.705	0.583	0.146	0.885
TaMAPK41	TaMAPKK18	4565.A0A3B5Y6A3	4565.A0A3B6LJ27	0	0	0	0.585	0	0.705	0.583	0.146	0.885
TaMAPK41	TaMAPKK4	4565.A0A3B5Y6A3	4565.A0A3B6KFB5	0	0	0.102	0.638	0	0.705	0.583	0.146	0.893
TaMAPK41	TaMAPKK8	4565.A0A3B5Y6A3	4565.A0A3B6IK39	0	0	0.116	0.621	0	0.705	0.583	0.146	0.894
TaMAPK41	TaMAPKK17	4565.A0A3B5Y6A3	4565.A0A3B6I0M7	0	0	0.121	0.619	0	0.705	0.583	0.146	0.895
TaMAPK41	TaMAPKK6	4565.A0A3B5Y6A3	4565.A0A3B6ILF0	0	0	0.122	0.617	0	0.705	0.583	0.146	0.895
TaMAPK41	TaMAPKK16	4565.A0A3B5Y6A3	4565.A0A3B6HZP7	0	0	0.122	0.622	0	0.705	0.583	0.146	0.895
TaMAPK41	TaMAPKK5	4565.A0A3B5Y6A3	4565.A0A3B6IMW7	0	0	0.119	0.609	0	0.705	0.583	0.146	0.895
TaMAPK41	TaMAPKK7	4565.A0A3B5Y6A3	4565.A0A3B6INV0	0	0	0.124	0.618	0	0.705	0.583	0.146	0.895
TaMAPK41	TaMAPKK15	4565.A0A3B5Y6A3	4565.A0A3B6HW51	0	0	0.122	0.621	0	0.705	0.583	0.146	0.895
TaMAPK41	TaMAPKK11	4565.A0A3B5Y6A3	4565.A0A3B6JEH0	0	0	0.12	0.62	0	0.705	0.583	0.146	0.895
TaMAPK41	TaMAPKK14	4565.A0A3B5Y6A3	4565.A0A3B6HY95	0	0	0.122	0.618	0	0.705	0.583	0.146	0.895
TaMAPK41	TaMAPKK12	4565.A0A3B5Y6A3	4565.A0A3B6JG06	0	0	0.127	0.614	0	0.705	0.583	0.146	0.896
TaMAPK41	TaMAPKK1	4565.A0A3B5Y6A3	4565.A0A3B6QJ87	0	0	0.131	0.615	0	0.705	0.583	0.146	0.896
TaMAPK42	TaMAPKK9	4565.A0A3B5Y6P8	4565.A0A077RVQ4	0	0	0.115	0.63	0	0.705	0.583	0.146	0.894

TaMAPK42	TaMEKK24	4565.A0A3B5Y6P8	4565.A0A3B6LLV5	0	0	0.176	0.582	0	0.27	0.063	0.088	0.417
TaMAPK42	TaMEKK17	4565.A0A3B5Y6P8	4565.A0A3B6KFL8	0	0	0.176	0.582	0	0.27	0.063	0.088	0.417
TaMAPK42	TaMEKK29	4565.A0A3B5Y6P8	4565.A0A3B6MSP6	0	0	0.177	0.582	0	0.27	0.063	0.088	0.418
TaMAPK42	TaMEKK2	4565.A0A3B5Y6P8	4565.A0A3B6JCC4	0	0	0.138	0.576	0	0.222	0.186	0.065	0.421
TaMAPK42	TaMEKK11	4565.A0A3B5Y6P8	4565.A0A3B6N0D8	0	0	0.151	0.577	0	0.222	0.186	0.065	0.43
TaMAPK42	TaMEKK16	4565.A0A3B5Y6P8	4565.A0A3B6LW00	0	0	0.153	0.579	0	0.222	0.186	0.065	0.431
TaMAPK42	TaMEKK15	4565.A0A3B5Y6P8	4565.A0A3B6KPK7	0	0	0.154	0.579	0	0.222	0.186	0.065	0.432
TaMAPK42	TaMEKK5	4565.A0A3B5Y6P8	4565.A0A3B6AWC1	0	0	0.158	0.578	0	0.222	0.186	0.065	0.434
TaMAPK42	TaMEKK14	4565.A0A3B5Y6P8	4565.A0A3B6KF43	0	0	0.158	0.59	0	0.222	0.186	0.065	0.435
TaMAPK42	TaRaf87	4565.A0A3B5Y6P8	4565.A0A3B6QMZ9	0	0	0	0.564	0.3	0.134	0.389	0.088	0.617
TaMAPK42	TaMAPKK2	4565.A0A3B5Y6P8	4565.A0A3B6LYW0	0	0	0	0.578	0	0.705	0.612	0.146	0.893
TaMAPK42	TaMAPKK3	4565.A0A3B5Y6P8	4565.A0A3B6MNP8	0	0	0	0.577	0	0.705	0.612	0.146	0.893
TaMAPK42	TaMAPKK13	4565.A0A3B5Y6P8	4565.A0A3B6N2X8	0	0	0	0.578	0	0.705	0.612	0.146	0.893
TaMAPK42	TaMAPKK18	4565.A0A3B5Y6P8	4565.A0A3B6LJ27	0	0	0	0.583	0	0.705	0.612	0.146	0.893
TaMAPK42	TaMAPKK8	4565.A0A3B5Y6P8	4565.A0A3B6IK39	0	0	0.116	0.619	0	0.705	0.583	0.146	0.894
TaMAPK42	TaMAPKK7	4565.A0A3B5Y6P8	4565.A0A3B6INV0	0	0	0.125	0.615	0	0.705	0.583	0.146	0.895
TaMAPK42	TaMAPKK15	4565.A0A3B5Y6P8	4565.A0A3B6HW51	0	0	0.122	0.623	0	0.705	0.583	0.146	0.895
TaMAPK42	TaMAPKK17	4565.A0A3B5Y6P8	4565.A0A3B6I0M7	0	0	0.124	0.616	0	0.705	0.583	0.146	0.895

TaMAPK42	TaMAPKK6	4565.A0A3B5Y6P8	4565.A0A3B6ILF0	0	0	0.123	0.612	0	0.705	0.583	0.146	0.895
TaMAPK42	TaMAPKK16	4565.A0A3B5Y6P8	4565.A0A3B6HZP7	0	0	0.121	0.624	0	0.705	0.583	0.146	0.895
TaMAPK42	TaMAPKK5	4565.A0A3B5Y6P8	4565.A0A3B6IMW7	0	0	0.119	0.616	0	0.705	0.583	0.146	0.895
TaMAPK42	TaMAPKK14	4565.A0A3B5Y6P8	4565.A0A3B6HY95	0	0	0.123	0.616	0	0.705	0.583	0.146	0.895
TaMAPK42	TaMAPKK11	4565.A0A3B5Y6P8	4565.A0A3B6JEH0	0	0	0.118	0.626	0	0.705	0.583	0.146	0.895
TaMAPK42	TaMAPKK1	4565.A0A3B5Y6P8	4565.A0A3B6QJ87	0	0	0.135	0.617	0	0.705	0.583	0.146	0.896
TaMAPK42	TaMAPKK12	4565.A0A3B5Y6P8	4565.A0A3B6JG06	0	0	0.131	0.613	0	0.705	0.583	0.146	0.896
TaMAPK42	TaMAPKK4	4565.A0A3B5Y6P8	4565.A0A3B6KFB5	0	0	0.107	0.63	0	0.705	0.612	0.146	0.901
TaMAPK43	TaMAPKK9	4565.A0A3B5Y7D4	4565.A0A077RVQ4	0	0	0.117	0.618	0	0.705	0.583	0.146	0.894
TaMAPK43	TaMEKK2	4565.A0A3B5Y7D4	4565.A0A3B6JCC4	0	0	0.128	0.574	0	0.222	0.186	0.065	0.415
TaMAPK43	TaMEKK11	4565.A0A3B5Y7D4	4565.A0A3B6N0D8	0	0	0.133	0.572	0	0.222	0.186	0.065	0.417
TaMAPK43	TaMEKK17	4565.A0A3B5Y7D4	4565.A0A3B6KFL8	0	0	0.178	0.576	0	0.27	0.063	0.088	0.418
TaMAPK43	TaMEKK16	4565.A0A3B5Y7D4	4565.A0A3B6LW00	0	0	0.133	0.573	0	0.222	0.186	0.065	0.418
TaMAPK43	TaMEKK24	4565.A0A3B5Y7D4	4565.A0A3B6LLV5	0	0	0.177	0.576	0	0.27	0.063	0.088	0.418
TaMAPK43	TaMEKK29	4565.A0A3B5Y7D4	4565.A0A3B6MSP6	0	0	0.178	0.576	0	0.27	0.063	0.088	0.418
TaMAPK43	TaMEKK15	4565.A0A3B5Y7D4	4565.A0A3B6KPK7	0	0	0.135	0.574	0	0.222	0.186	0.065	0.419
TaMAPK43	TaMEKK5	4565.A0A3B5Y7D4	4565.A0A3B6AWC1	0	0	0.141	0.582	0	0.222	0.186	0.065	0.423
TaMAPK43	TaMEKK14	4565.A0A3B5Y7D4	4565.A0A3B6KF43	0	0	0.142	0.581	0	0.222	0.186	0.065	0.424

TaMAPK43	TaRaf87	4565.A0A3B5Y7D4	4565.A0A3B6QMZ9	0	0	0	0.56	0.277	0.134	0.389	0.088	0.604
TaMAPK43	TaMAPKK18	4565.A0A3B5Y7D4	4565.A0A3B6LJ27	0	0	0	0.589	0	0.705	0.583	0.146	0.885
TaMAPK43	TaMAPKK13	4565.A0A3B5Y7D4	4565.A0A3B6N2X8	0	0	0	0.583	0	0.705	0.583	0.146	0.885
TaMAPK43	TaMAPKK2	4565.A0A3B5Y7D4	4565.A0A3B6LYW0	0	0	0	0.585	0	0.705	0.583	0.146	0.885
TaMAPK43	TaMAPKK3	4565.A0A3B5Y7D4	4565.A0A3B6MNP8	0	0	0	0.583	0	0.705	0.583	0.146	0.885
TaMAPK43	TaMAPKK4	4565.A0A3B5Y7D4	4565.A0A3B6KFB5	0	0	0.097	0.644	0	0.705	0.583	0.146	0.892
TaMAPK43	TaMAPKK11	4565.A0A3B5Y7D4	4565.A0A3B6JEH0	0	0	0.118	0.614	0	0.705	0.583	0.146	0.894
TaMAPK43	TaMAPKK8	4565.A0A3B5Y7D4	4565.A0A3B6IK39	0	0	0.116	0.612	0	0.705	0.583	0.146	0.894
TaMAPK43	TaMAPKK14	4565.A0A3B5Y7D4	4565.A0A3B6HY95	0	0	0.119	0.611	0	0.705	0.583	0.146	0.895
TaMAPK43	TaMAPKK12	4565.A0A3B5Y7D4	4565.A0A3B6JG06	0	0	0.122	0.608	0	0.705	0.583	0.146	0.895
TaMAPK43	TaMAPKK15	4565.A0A3B5Y7D4	4565.A0A3B6HW51	0	0	0.121	0.617	0	0.705	0.583	0.146	0.895
TaMAPK43	TaMAPKK7	4565.A0A3B5Y7D4	4565.A0A3B6INV0	0	0	0.122	0.611	0	0.705	0.583	0.146	0.895
TaMAPK43	TaMAPKK5	4565.A0A3B5Y7D4	4565.A0A3B6IMW7	0	0	0.119	0.608	0	0.705	0.583	0.146	0.895
TaMAPK43	TaMAPKK16	4565.A0A3B5Y7D4	4565.A0A3B6HZP7	0	0	0.12	0.616	0	0.705	0.583	0.146	0.895
TaMAPK43	TaMAPKK6	4565.A0A3B5Y7D4	4565.A0A3B6ILF0	0	0	0.119	0.611	0	0.705	0.583	0.146	0.895
TaMAPK43	TaMAPKK17	4565.A0A3B5Y7D4	4565.A0A3B6I0M7	0	0	0.119	0.612	0	0.705	0.583	0.146	0.895
TaMAPK43	TaMAPKK1	4565.A0A3B5Y7D4	4565.A0A3B6QJ87	0	0	0.127	0.605	0	0.705	0.583	0.146	0.896
TaMAPK44	TaMAPKK9	4565.A0A3B6QDH5	4565.A0A077RVQ4	0	0	0.137	0.618	0	0.705	0.583	0.146	0.897

TaMAPK44	TaMEKK5	4565.A0A3B6QDH5	4565.A0A3B6AWC1	0	0	0.169	0.573	0	0.222	0.186	0.065	0.442
TaMAPK44	TaMEKK1	4565.A0A3B6QDH5	4565.A0A3B6B3I4	0	0	0.157	0.612	0	0.27	0.063	0.088	0.403
TaMAPK44	TaMAPKK15	4565.A0A3B6QDH5	4565.A0A3B6HW51	0	0	0.143	0.615	0	0.705	0.583	0.146	0.898
TaMAPK44	TaMAPKK14	4565.A0A3B6QDH5	4565.A0A3B6HY95	0	0	0.142	0.608	0	0.705	0.583	0.146	0.897
TaMAPK44	TaMAPKK16	4565.A0A3B6QDH5	4565.A0A3B6HZP7	0	0	0.138	0.622	0	0.705	0.583	0.146	0.897
TaMAPK44	TaMAPKK17	4565.A0A3B6QDH5	4565.A0A3B6I0M7	0	0	0.141	0.606	0	0.705	0.583	0.146	0.897
TaMAPK44	TaMAPKK8	4565.A0A3B6QDH5	4565.A0A3B6IK39	0	0	0.132	0.613	0	0.705	0.583	0.146	0.896
TaMAPK44	TaMAPKK6	4565.A0A3B6QDH5	4565.A0A3B6ILF0	0	0	0.135	0.613	0	0.705	0.583	0.146	0.897
TaMAPK44	TaMAPKK5	4565.A0A3B6QDH5	4565.A0A3B6IMW7	0	0	0.136	0.606	0	0.705	0.583	0.146	0.897
TaMAPK44	TaMAPKK7	4565.A0A3B6QDH5	4565.A0A3B6INV0	0	0	0.142	0.603	0	0.705	0.583	0.146	0.897
TaMAPK44	TaMEKK2	4565.A0A3B6QDH5	4565.A0A3B6JCC4	0	0	0.15	0.572	0	0.222	0.186	0.065	0.429
TaMAPK44	TaMAPKK11	4565.A0A3B6QDH5	4565.A0A3B6JEH0	0	0	0.137	0.618	0	0.705	0.583	0.146	0.897
TaMAPK44	TaMAPKK12	4565.A0A3B6QDH5	4565.A0A3B6JG06	0	0	0.145	0.603	0	0.705	0.583	0.146	0.898
TaMAPK44	TaMEKK14	4565.A0A3B6QDH5	4565.A0A3B6KF43	0	0	0.165	0.579	0	0.222	0.186	0.065	0.439
TaMAPK44	TaMAPKK4	4565.A0A3B6QDH5	4565.A0A3B6KFB5	0	0	0.113	0.629	0	0.705	0.612	0.146	0.901
TaMAPK44	TaMEKK17	4565.A0A3B6QDH5	4565.A0A3B6KFL8	0	0	0.176	0.601	0	0.27	0.063	0.088	0.417
TaMAPK44	TaMEKK15	4565.A0A3B6QDH5	4565.A0A3B6KPK7	0	0	0.163	0.572	0	0.222	0.186	0.065	0.438
TaMAPK44	TaMAPKK18	4565.A0A3B6QDH5	4565.A0A3B6LJ27	0	0	0.113	0.581	0	0.705	0.612	0.146	0.901

TaMAPK44	TaMEKK24	4565.A0A3B6QDH5	4565.A0A3B6LLV5	0	0	0.175	0.601	0	0.27	0.063	0.088	0.416
TaMAPK44	TaMEKK16	4565.A0A3B6QDH5	4565.A0A3B6LW00	0	0	0.162	0.572	0	0.222	0.186	0.065	0.437
TaMAPK44	TaMAPKK2	4565.A0A3B6QDH5	4565.A0A3B6LYW0	0	0	0	0.58	0	0.705	0.612	0.146	0.893
TaMAPK44	TaMAPKK3	4565.A0A3B6QDH5	4565.A0A3B6MNP8	0	0	0	0.578	0	0.705	0.612	0.146	0.893
TaMAPK44	TaMEKK29	4565.A0A3B6QDH5	4565.A0A3B6MSP6	0	0	0.176	0.601	0	0.27	0.063	0.088	0.417
TaMAPK44	TaMEKK11	4565.A0A3B6QDH5	4565.A0A3B6N0D8	0	0	0.161	0.57	0	0.222	0.186	0.065	0.437
TaMAPK44	TaMAPKK13	4565.A0A3B6QDH5	4565.A0A3B6N2X8	0	0	0	0.579	0	0.705	0.612	0.146	0.893
TaMAPK44	TaMEKK4	4565.A0A3B6QDH5	4565.A0A3B6NRN9	0	0	0.158	0.596	0	0.27	0.063	0.088	0.404
TaMAPK44	TaMEKK4-1	4565.A0A3B6QDH5	4565.A0A3B6PNI6	0	0	0.16	0.599	0	0.27	0.063	0.088	0.406
TaMAPK44	TaRaf87	4565.A0A3B6QDH5	4565.A0A3B6QMZ9	0	0	0	0.58	0.3	0.134	0.389	0.088	0.617
TaMAPK44	TaMAPKK1	4565.A0A3B6QDH5	4565.A0A3B6QJ87	0	0	0.145	0.605	0	0.705	0.583	0.146	0.898
TaMAPK45	TaMAPKK9	4565.A0A3B6QBD2	4565.A0A077RVQ4	0	0	0	0.651	0	0.705	0.583	0.146	0.885
TaMAPK45	TaMAPKK15	4565.A0A3B6QBD2	4565.A0A3B6HW51	0	0	0	0.643	0	0.705	0.583	0.146	0.885
TaMAPK45	TaMAPKK14	4565.A0A3B6QBD2	4565.A0A3B6HY95	0	0	0	0.636	0	0.705	0.583	0.146	0.885
TaMAPK45	TaMAPKK16	4565.A0A3B6QBD2	4565.A0A3B6HZP7	0	0	0	0.651	0	0.705	0.583	0.146	0.885
TaMAPK45	TaMAPKK17	4565.A0A3B6QBD2	4565.A0A3B6I0M7	0	0	0	0.623	0	0.705	0.583	0.146	0.885
TaMAPK45	TaMAPKK8	4565.A0A3B6QBD2	4565.A0A3B6IK39	0	0	0	0.642	0	0.705	0.583	0.146	0.885
TaMAPK45	TaMAPKK6	4565.A0A3B6QBD2	4565.A0A3B6ILF0	0	0	0	0.623	0	0.705	0.583	0.146	0.885

TaMAPK45	TaMAPKK5	4565.A0A3B6QBD2	4565.A0A3B6IMW7	0	0	0	0.627	0	0.705	0.583	0.146	0.885
TaMAPK45	TaMAPKK7	4565.A0A3B6QBD2	4565.A0A3B6INV0	0	0	0	0.623	0	0.705	0.583	0.146	0.885
TaMAPK45	TaMAPKK11	4565.A0A3B6QBD2	4565.A0A3B6JEH0	0	0	0.101	0.642	0	0.705	0.583	0.146	0.892
TaMAPK45	TaMAPKK12	4565.A0A3B6QBD2	4565.A0A3B6JG06	0	0	0	0.617	0	0.705	0.583	0.146	0.885
TaMAPK45	TaMAPKK4	4565.A0A3B6QBD2	4565.A0A3B6KFB5	0	0	0	0.606	0	0.705	0.825	0.195	0.954
TaMAPK45	TaMAPKK18	4565.A0A3B6QBD2	4565.A0A3B6LJ27	0	0	0	0.589	0	0.705	0.825	0.195	0.954
TaMAPK45	TaMAPKK2	4565.A0A3B6QBD2	4565.A0A3B6LYW0	0	0	0	0.592	0	0.705	0.825	0.195	0.954
TaMAPK45	TaMAPKK3	4565.A0A3B6QBD2	4565.A0A3B6MNP8	0	0	0	0.588	0	0.705	0.825	0.195	0.954
TaMAPK45	TaMAPKK13	4565.A0A3B6QBD2	4565.A0A3B6N2X8	0	0	0	0.588	0	0.705	0.825	0.195	0.954
TaMAPK45	TaRaf87	4565.A0A3B6QBD2	4565.A0A3B6QMZ9	0	0	0	0.578	0.16	0.134	0.389	0.088	0.54
TaMAPK45	TaMAPK54	4565.A0A3B6QBD2	4565.A0A3B6SKC9	0	0	0.048	0.984	0	0	0.793	0	0.794
TaMAPK45	TaMAPK47	4565.A0A3B6QBD2	4565.A0A3B6RAZ7	0	0	0.053	0.974	0	0	0.793	0	0.795
TaMAPK45	TaMAPKK1	4565.A0A3B6QBD2	4565.A0A3B6QJ87	0	0	0	0.602	0	0.705	0.583	0.146	0.885
TaMAPK47	TaMAPKK9	4565.A0A3B6RAZ7	4565.A0A077RVQ4	0	0	0.153	0.651	0	0.705	0.583	0.146	0.899
TaMAPK47	TaMEKK5	4565.A0A3B6RAZ7	4565.A0A3B6AWC1	0	0	0.137	0.649	0	0.222	0.186	0.065	0.42
TaMAPK47	TaMEKK1	4565.A0A3B6RAZ7	4565.A0A3B6B3I4	0	0	0.156	0.66	0	0.27	0.063	0.088	0.403
TaMAPK47	TaMAPKK15	4565.A0A3B6RAZ7	4565.A0A3B6HW51	0	0	0.161	0.646	0	0.705	0.583	0.146	0.9
TaMAPK47	TaMAPKK14	4565.A0A3B6RAZ7	4565.A0A3B6HY95	0	0	0.161	0.643	0	0.705	0.583	0.146	0.9

TaMAPK47	TaMAPKK16	4565.A0A3B6RAZ7	4565.A0A3B6HZP7	0	0	0.16	0.643	0	0.705	0.583	0.146	0.9
TaMAPK47	TaMAPKK17	4565.A0A3B6RAZ7	4565.A0A3B6I0M7	0	0	0.156	0.63	0	0.705	0.583	0.146	0.899
TaMAPK47	TaMAPKK8	4565.A0A3B6RAZ7	4565.A0A3B6IK39	0	0	0.155	0.642	0	0.705	0.583	0.146	0.899
TaMAPK47	TaMAPKK6	4565.A0A3B6RAZ7	4565.A0A3B6ILF0	0	0	0.155	0.628	0	0.705	0.583	0.146	0.899
TaMAPK47	TaMAPKK5	4565.A0A3B6RAZ7	4565.A0A3B6IMW7	0	0	0.159	0.639	0	0.705	0.583	0.146	0.899
TaMAPK47	TaMAPKK7	4565.A0A3B6RAZ7	4565.A0A3B6INV0	0	0	0.155	0.629	0	0.705	0.583	0.146	0.899
TaMAPK47	TaMEKK2	4565.A0A3B6RAZ7	4565.A0A3B6JCC4	0	0	0.123	0.618	0	0.222	0.186	0.065	0.411
TaMAPK47	TaMAPKK11	4565.A0A3B6RAZ7	4565.A0A3B6JEH0	0	0	0.164	0.639	0	0.705	0.583	0.146	0.9
TaMAPK47	TaMAPKK12	4565.A0A3B6RAZ7	4565.A0A3B6JG06	0	0	0.159	0.628	0	0.705	0.583	0.146	0.899
TaMAPK47	TaMEKK14	4565.A0A3B6RAZ7	4565.A0A3B6KF43	0	0	0.14	0.64	0	0.222	0.186	0.065	0.423
TaMAPK47	TaMAPKK4	4565.A0A3B6RAZ7	4565.A0A3B6KFB5	0	0	0.12	0.606	0	0.705	0.739	0.195	0.938
TaMAPK47	TaMEKK17	4565.A0A3B6RAZ7	4565.A0A3B6KFL8	0	0	0.169	0.646	0	0.27	0.063	0.088	0.412
TaMAPK47	TaMEKK15	4565.A0A3B6RAZ7	4565.A0A3B6KPK7	0	0	0.13	0.636	0	0.222	0.186	0.065	0.416
TaMAPK47	TaMAPKK18	4565.A0A3B6RAZ7	4565.A0A3B6LJ27	0	0	0	0.604	0	0.705	0.739	0.195	0.932
TaMAPK47	TaMEKK24	4565.A0A3B6RAZ7	4565.A0A3B6LLV5	0	0	0.168	0.646	0	0.27	0.063	0.088	0.412
TaMAPK47	TaMEKK16	4565.A0A3B6RAZ7	4565.A0A3B6LW00	0	0	0.129	0.636	0	0.222	0.186	0.065	0.415
TaMAPK47	TaMAPKK2	4565.A0A3B6RAZ7	4565.A0A3B6LYW0	0	0	0	0.622	0	0.705	0.886	0.195	0.97
TaMAPK47	TaMAPKK3	4565.A0A3B6RAZ7	4565.A0A3B6MNP8	0	0	0	0.603	0	0.705	0.739	0.195	0.932

TaMAPK47	TaMEKK29	4565.A0A3B6RAZ7	4565.A0A3B6MSP6	0	0	0.17	0.646	0	0.27	0.063	0.088	0.413
TaMAPK47	TaMEKK11	4565.A0A3B6RAZ7	4565.A0A3B6N0D8	0	0	0.129	0.635	0	0.222	0.186	0.065	0.415
TaMAPK47	TaMAPKK13	4565.A0A3B6RAZ7	4565.A0A3B6N2X8	0	0	0	0.621	0	0.705	0.886	0.195	0.97
TaMAPK47	TaMEKK4	4565.A0A3B6RAZ7	4565.A0A3B6NRN9	0	0	0.153	0.668	0	0.27	0.063	0.088	0.401
TaMAPK47	TaMEKK4-1	4565.A0A3B6RAZ7	4565.A0A3B6PNI6	0	0	0.155	0.668	0	0.27	0.063	0.088	0.402
TaMAPK47	TaMAPKK1	4565.A0A3B6RAZ7	4565.A0A3B6QJ87	0	0	0.161	0.617	0	0.705	0.583	0.146	0.9
TaMAPK47	TaRaf87	4565.A0A3B6RAZ7	4565.A0A3B6QMZ9	0	0	0	0.616	0.16	0.134	0.389	0.088	0.54
TaMAPK49	TaMAPKK9	4565.A9RAB3	4565.A0A077RVQ4	0	0	0.12	0.616	0	0.705	0.583	0.146	0.895
TaMAPK49	TaRaf88	4565.A9RAB3	4565.A0A3B5Z5X1	0	0	0.284	0	0	0.15	0.177	0.05	0.46
TaMAPK49	TaRaf30	4565.A9RAB3	4565.A0A3B6A1Z4	0	0	0.281	0	0	0.15	0.177	0.05	0.458
TaMAPK49	TaMEKK5	4565.A9RAB3	4565.A0A3B6AWC1	0	0	0.165	0.575	0	0.222	0.186	0.065	0.439
TaMAPK49	TaMAPKK15	4565.A9RAB3	4565.A0A3B6HW51	0	0	0.125	0.614	0	0.705	0.583	0.146	0.895
TaMAPK49	TaMAPKK14	4565.A9RAB3	4565.A0A3B6HY95	0	0	0.125	0.606	0	0.705	0.583	0.146	0.895
TaMAPK49	TaMAPKK16	4565.A9RAB3	4565.A0A3B6HZP7	0	0	0.124	0.616	0	0.705	0.583	0.146	0.895
TaMAPK49	TaMAPKK17	4565.A9RAB3	4565.A0A3B6I0M7	0	0	0.122	0.62	0	0.705	0.583	0.146	0.895
TaMAPK49	TaMAPKK8	4565.A9RAB3	4565.A0A3B6IK39	0	0	0.117	0.615	0	0.705	0.583	0.146	0.894
TaMAPK49	TaMAPKK6	4565.A9RAB3	4565.A0A3B6ILF0	0	0	0.123	0.611	0	0.705	0.583	0.146	0.895
TaMAPK49	TaMAPKK5	4565.A9RAB3	4565.A0A3B6IMW7	0	0	0.122	0.605	0	0.705	0.583	0.146	0.895

TaMAPK49	TaMAPKK7	4565.A9RAB3	4565.A0A3B6INV0	0	0	0.124	0.621	0	0.705	0.583	0.146	0.895
TaMAPK49	TaMEKK2	4565.A9RAB3	4565.A0A3B6JCC4	0	0	0.144	0.572	0	0.222	0.186	0.065	0.425
TaMAPK49	TaMAPKK11	4565.A9RAB3	4565.A0A3B6JEH0	0	0	0.121	0.615	0	0.705	0.583	0.146	0.895
TaMAPK49	TaMAPKK12	4565.A9RAB3	4565.A0A3B6JG06	0	0	0.128	0.616	0	0.705	0.583	0.146	0.896
TaMAPK49	TaMEKK14	4565.A9RAB3	4565.A0A3B6KF43	0	0	0.155	0.583	0	0.222	0.186	0.065	0.432
TaMAPK49	TaMAPKK4	4565.A9RAB3	4565.A0A3B6KFB5	0	0	0.105	0.637	0	0.705	0.612	0.146	0.9
TaMAPK49	TaMEKK17	4565.A9RAB3	4565.A0A3B6KFL8	0	0	0.171	0.577	0	0.27	0.063	0.088	0.414
TaMAPK49	TaMEKK15	4565.A9RAB3	4565.A0A3B6KPK7	0	0	0.156	0.576	0	0.222	0.186	0.065	0.433
TaMAPK49	TaMAPKK18	4565.A9RAB3	4565.A0A3B6LJ27	0	0	0	0.584	0	0.705	0.612	0.146	0.893
TaMAPK49	TaMEKK24	4565.A9RAB3	4565.A0A3B6LLV5	0	0	0.17	0.577	0	0.27	0.063	0.088	0.413
TaMAPK49	TaMEKK16	4565.A9RAB3	4565.A0A3B6LW00	0	0	0.155	0.576	0	0.222	0.186	0.065	0.433
TaMAPK49	TaMAPKK2	4565.A9RAB3	4565.A0A3B6LYW0	0	0	0	0.575	0	0.705	0.612	0.205	0.901
TaMAPK49	TaMAPKK3	4565.A9RAB3	4565.A0A3B6MNP8	0	0	0	0.58	0	0.705	0.612	0.146	0.893
TaMAPK49	TaMEKK29	4565.A9RAB3	4565.A0A3B6MSP6	0	0	0.172	0.577	0	0.27	0.063	0.088	0.414
TaMAPK49	TaMEKK11	4565.A9RAB3	4565.A0A3B6N0D8	0	0	0.154	0.573	0	0.222	0.186	0.065	0.432
TaMAPK49	TaMAPKK13	4565.A9RAB3	4565.A0A3B6N2X8	0	0	0	0.576	0	0.705	0.612	0.205	0.901
TaMAPK49	TaMAPKK1	4565.A9RAB3	4565.A0A3B6QJ87	0	0	0.127	0.619	0	0.705	0.583	0.146	0.896
TaMAPK49	TaRaf87	4565.A9RAB3	4565.A0A3B6QMZ9	0	0	0	0.565	0.3	0.134	0.389	0.088	0.617

TaMAPK5	TaMAPKK9	4565.A0A3B6A1I9	4565.A0A077RVQ4	0	0	0.109	0.629	0	0.705	0.583	0.146	0.893
TaMAPK5	TaMEKK24	4565.A0A3B6A1I9	4565.A0A3B6LLV5	0	0	0.168	0.583	0	0.27	0.063	0.088	0.411
TaMAPK5	TaMEKK29	4565.A0A3B6A1I9	4565.A0A3B6MSP6	0	0	0.169	0.583	0	0.27	0.063	0.088	0.412
TaMAPK5	TaMEKK17	4565.A0A3B6A1I9	4565.A0A3B6KFL8	0	0	0.169	0.583	0	0.27	0.063	0.088	0.412
TaMAPK5	TaMEKK2	4565.A0A3B6A1I9	4565.A0A3B6JCC4	0	0	0.139	0.575	0	0.222	0.186	0.065	0.422
TaMAPK5	TaMEKK11	4565.A0A3B6A1I9	4565.A0A3B6N0D8	0	0	0.148	0.576	0	0.222	0.186	0.065	0.428
TaMAPK5	TaMEKK16	4565.A0A3B6A1I9	4565.A0A3B6LW00	0	0	0.149	0.578	0	0.222	0.186	0.065	0.429
TaMAPK5	TaMEKK15	4565.A0A3B6A1I9	4565.A0A3B6KPK7	0	0	0.151	0.578	0	0.222	0.186	0.065	0.43
TaMAPK5	TaMEKK5	4565.A0A3B6A1I9	4565.A0A3B6AWC1	0	0	0.154	0.578	0	0.222	0.186	0.065	0.432
TaMAPK5	TaMEKK14	4565.A0A3B6A1I9	4565.A0A3B6KF43	0	0	0.155	0.591	0	0.222	0.186	0.065	0.433
TaMAPK5	TaRaf87	4565.A0A3B6A1I9	4565.A0A3B6QMZ9	0	0	0	0.565	0.3	0.134	0.389	0.088	0.617
TaMAPK5	TaMAPKK3	4565.A0A3B6A1I9	4565.A0A3B6MNP8	0	0	0	0.574	0	0.705	0.612	0.146	0.893
TaMAPK5	TaMAPKK8	4565.A0A3B6A1I9	4565.A0A3B6IK39	0	0	0.109	0.621	0	0.705	0.583	0.146	0.893
TaMAPK5	TaMAPKK2	4565.A0A3B6A1I9	4565.A0A3B6LYW0	0	0	0	0.577	0	0.705	0.612	0.146	0.893
TaMAPK5	TaMAPKK18	4565.A0A3B6A1I9	4565.A0A3B6LJ27	0	0	0	0.579	0	0.705	0.612	0.146	0.893
TaMAPK5	TaMAPKK13	4565.A0A3B6A1I9	4565.A0A3B6N2X8	0	0	0	0.577	0	0.705	0.612	0.146	0.893
TaMAPK5	TaMAPKK16	4565.A0A3B6A1I9	4565.A0A3B6HZP7	0	0	0.115	0.623	0	0.705	0.583	0.146	0.894
TaMAPK5	TaMAPKK5	4565.A0A3B6A1I9	4565.A0A3B6IMW7	0	0	0.112	0.616	0	0.705	0.583	0.146	0.894

TaMAPK5	TaMAPKK 6	4565.A0A3 B6A1I9	4565.A0A3 B6ILF0	0	0	0.118	0.612	0	0.705	0.583	0.146	0.894
TaMAPK5	TaMAPKK 15	4565.A0A3 B6A1I9	4565.A0A3 B6HW51	0	0	0.115	0.623	0	0.705	0.583	0.146	0.894
TaMAPK5	TaMAPKK 11	4565.A0A3 B6A1I9	4565.A0A3 B6JEH0	0	0	0.111	0.626	0	0.705	0.583	0.146	0.894
TaMAPK5	TaMAPKK 14	4565.A0A3 B6A1I9	4565.A0A3 B6HY95	0	0	0.116	0.615	0	0.705	0.583	0.146	0.894
TaMAPK5	TaMAPKK 17	4565.A0A3 B6A1I9	4565.A0A3 B6I0M7	0	0	0.118	0.614	0	0.705	0.583	0.146	0.895
TaMAPK5	TaMAPKK 7	4565.A0A3 B6A1I9	4565.A0A3 B6INV0	0	0	0.119	0.614	0	0.705	0.583	0.146	0.895
TaMAPK5	TaMAPKK 12	4565.A0A3 B6A1I9	4565.A0A3 B6JG06	0	0	0.124	0.612	0	0.705	0.583	0.146	0.895
TaMAPK5	TaMAPKK 1	4565.A0A3 B6A1I9	4565.A0A3 B6QJ87	0	0	0.125	0.617	0	0.705	0.583	0.146	0.895
TaMAPK5	TaMAPKK 4	4565.A0A3 B6A1I9	4565.A0A3 B6KFB5	0	0	0.104	0.622	0	0.705	0.612	0.146	0.9
TaMAPK5 2	TaMAPKK 9	4565.A0A3 B6SH79	4565.A0A0 77RVQ4	0	0	0.117	0.625	0	0.705	0.583	0.146	0.894
TaMAPK5 2	TaMEKK5	4565.A0A3 B6SH79	4565.A0A3 B6AWC1	0	0	0.151	0.595	0	0.222	0.186	0.065	0.43
TaMAPK5 2	TaMAPKK 15	4565.A0A3 B6SH79	4565.A0A3 B6HW51	0	0	0.122	0.624	0	0.705	0.583	0.146	0.895
TaMAPK5 2	TaMAPKK 14	4565.A0A3 B6SH79	4565.A0A3 B6HY95	0	0	0.12	0.619	0	0.705	0.583	0.146	0.895
TaMAPK5 2	TaMAPKK 16	4565.A0A3 B6SH79	4565.A0A3 B6HZP7	0	0	0.12	0.625	0	0.705	0.583	0.146	0.895
TaMAPK5 2	TaMAPKK 17	4565.A0A3 B6SH79	4565.A0A3 B6I0M7	0	0	0.12	0.626	0	0.705	0.583	0.146	0.895
TaMAPK5 2	TaMAPKK 8	4565.A0A3 B6SH79	4565.A0A3 B6IK39	0	0	0.113	0.624	0	0.705	0.583	0.146	0.894
TaMAPK5 2	TaMAPKK 6	4565.A0A3 B6SH79	4565.A0A3 B6ILF0	0	0	0.121	0.619	0	0.705	0.583	0.146	0.895
TaMAPK5 2	TaMAPKK 5	4565.A0A3 B6SH79	4565.A0A3 B6IMW7	0	0	0.116	0.616	0	0.705	0.583	0.146	0.894

TaMAPK52	TaMAPKK7	4565.A0A3B6SH79	4565.A0A3B6INV0	0	0	0.122	0.624	0	0.705	0.583	0.146	0.895
TaMAPK52	TaMEKK2	4565.A0A3B6SH79	4565.A0A3B6JCC4	0	0	0.132	0.597	0	0.222	0.186	0.065	0.417
TaMAPK52	TaMAPKK11	4565.A0A3B6SH79	4565.A0A3B6JEH0	0	0	0.118	0.624	0	0.705	0.583	0.146	0.895
TaMAPK52	TaMAPKK12	4565.A0A3B6SH79	4565.A0A3B6JG06	0	0	0.124	0.619	0	0.705	0.583	0.146	0.895
TaMAPK52	TaMEKK14	4565.A0A3B6SH79	4565.A0A3B6KF43	0	0	0.145	0.609	0	0.222	0.186	0.065	0.426
TaMAPK52	TaMAPKK4	4565.A0A3B6SH79	4565.A0A3B6KFB5	0	0	0.103	0.626	0	0.705	0.583	0.146	0.893
TaMAPK52	TaMEKK17	4565.A0A3B6SH79	4565.A0A3B6KFL8	0	0	0.161	0.609	0	0.27	0.063	0.088	0.406
TaMAPK52	TaMEKK15	4565.A0A3B6SH79	4565.A0A3B6KPK7	0	0	0.142	0.594	0	0.222	0.186	0.065	0.424
TaMAPK52	TaMAPKK18	4565.A0A3B6SH79	4565.A0A3B6LJ27	0	0	0	0.591	0	0.705	0.583	0.146	0.885
TaMAPK52	TaMEKK24	4565.A0A3B6SH79	4565.A0A3B6LLV5	0	0	0.16	0.609	0	0.27	0.063	0.088	0.406
TaMAPK52	TaMEKK16	4565.A0A3B6SH79	4565.A0A3B6LW00	0	0	0.141	0.594	0	0.222	0.186	0.065	0.423
TaMAPK52	TaMAPKK2	4565.A0A3B6SH79	4565.A0A3B6LYW0	0	0	0	0.595	0	0.705	0.583	0.146	0.885
TaMAPK52	TaMAPKK3	4565.A0A3B6SH79	4565.A0A3B6MNP8	0	0	0	0.593	0	0.705	0.583	0.146	0.885
TaMAPK52	TaMEKK29	4565.A0A3B6SH79	4565.A0A3B6MSP6	0	0	0.161	0.609	0	0.27	0.063	0.088	0.406
TaMAPK52	TaMEKK11	4565.A0A3B6SH79	4565.A0A3B6N0D8	0	0	0.142	0.591	0	0.222	0.186	0.065	0.423
TaMAPK52	TaMAPKK13	4565.A0A3B6SH79	4565.A0A3B6N2X8	0	0	0	0.594	0	0.705	0.583	0.146	0.885
TaMAPK52	TaMAPKK1	4565.A0A3B6SH79	4565.A0A3B6QJ87	0	0	0.132	0.625	0	0.705	0.583	0.146	0.896
TaMAPK52	TaRaf87	4565.A0A3B6SH79	4565.A0A3B6QMZ9	0	0	0	0.585	0.277	0.134	0.389	0.088	0.604

TaMAPK53	TaMAPKK9	4565.A0A3B6SPB2	4565.A0A077RVQ4	0	0	0.12	0.616	0	0.705	0.583	0.146	0.895
TaMAPK53	TaRaf88	4565.A0A3B6SPB2	4565.A0A3B5Z5X1	0	0	0.286	0	0	0.15	0.177	0.05	0.462
TaMAPK53	TaRaf30	4565.A0A3B6SPB2	4565.A0A3B6A1Z4	0	0	0.282	0	0	0.15	0.177	0.05	0.458
TaMAPK53	TaMEKK5	4565.A0A3B6SPB2	4565.A0A3B6AWC1	0	0	0.164	0.575	0	0.222	0.186	0.065	0.439
TaMAPK53	TaMAPKK15	4565.A0A3B6SPB2	4565.A0A3B6HW51	0	0	0.126	0.614	0	0.705	0.583	0.146	0.895
TaMAPK53	TaMAPKK14	4565.A0A3B6SPB2	4565.A0A3B6HY95	0	0	0.125	0.607	0	0.705	0.583	0.146	0.895
TaMAPK53	TaMAPKK16	4565.A0A3B6SPB2	4565.A0A3B6HZP7	0	0	0.124	0.617	0	0.705	0.583	0.146	0.895
TaMAPK53	TaMAPKK17	4565.A0A3B6SPB2	4565.A0A3B6I0M7	0	0	0.122	0.62	0	0.705	0.583	0.146	0.895
TaMAPK53	TaMAPKK8	4565.A0A3B6SPB2	4565.A0A3B6IK39	0	0	0.118	0.615	0	0.705	0.583	0.146	0.894
TaMAPK53	TaMAPKK6	4565.A0A3B6SPB2	4565.A0A3B6ILF0	0	0	0.123	0.61	0	0.705	0.583	0.146	0.895
TaMAPK53	TaMAPKK5	4565.A0A3B6SPB2	4565.A0A3B6IMW7	0	0	0.123	0.605	0	0.705	0.583	0.146	0.895
TaMAPK53	TaMAPKK7	4565.A0A3B6SPB2	4565.A0A3B6INV0	0	0	0.124	0.621	0	0.705	0.583	0.146	0.895
TaMAPK53	TaMEKK2	4565.A0A3B6SPB2	4565.A0A3B6JCC4	0	0	0.144	0.572	0	0.222	0.186	0.065	0.425
TaMAPK53	TaMAPKK11	4565.A0A3B6SPB2	4565.A0A3B6JEH0	0	0	0.122	0.615	0	0.705	0.583	0.146	0.895
TaMAPK53	TaMAPKK12	4565.A0A3B6SPB2	4565.A0A3B6JG06	0	0	0.128	0.616	0	0.705	0.583	0.146	0.896
TaMAPK53	TaMEKK14	4565.A0A3B6SPB2	4565.A0A3B6KF43	0	0	0.155	0.583	0	0.222	0.186	0.065	0.433
TaMAPK53	TaMAPKK4	4565.A0A3B6SPB2	4565.A0A3B6KFB5	0	0	0.106	0.637	0	0.705	0.583	0.146	0.893
TaMAPK53	TaMEKK17	4565.A0A3B6SPB2	4565.A0A3B6KFL8	0	0	0.172	0.577	0	0.27	0.063	0.088	0.414

TaMAPK53	TaMEKK15	4565.A0A3B6SPB2	4565.A0A3B6KPK7	0	0	0.156	0.576	0	0.222	0.186	0.065	0.433
TaMAPK53	TaMAPKK18	4565.A0A3B6SPB2	4565.A0A3B6LJ27	0	0	0	0.584	0	0.705	0.583	0.146	0.885
TaMAPK53	TaMEKK24	4565.A0A3B6SPB2	4565.A0A3B6LLV5	0	0	0.171	0.577	0	0.27	0.063	0.088	0.413
TaMAPK53	TaMEKK16	4565.A0A3B6SPB2	4565.A0A3B6LW00	0	0	0.155	0.575	0	0.222	0.186	0.065	0.433
TaMAPK53	TaMAPKK2	4565.A0A3B6SPB2	4565.A0A3B6LYW0	0	0	0	0.576	0	0.705	0.583	0.146	0.885
TaMAPK53	TaMAPKK3	4565.A0A3B6SPB2	4565.A0A3B6MNP8	0	0	0	0.58	0	0.705	0.583	0.146	0.885
TaMAPK53	TaMEKK29	4565.A0A3B6SPB2	4565.A0A3B6MSP6	0	0	0.172	0.577	0	0.27	0.063	0.088	0.415
TaMAPK53	TaMEKK11	4565.A0A3B6SPB2	4565.A0A3B6N0D8	0	0	0.154	0.573	0	0.222	0.186	0.065	0.432
TaMAPK53	TaMAPKK13	4565.A0A3B6SPB2	4565.A0A3B6N2X8	0	0	0	0.576	0	0.705	0.583	0.146	0.885
TaMAPK53	TaMAPKK1	4565.A0A3B6SPB2	4565.A0A3B6QJ87	0	0	0.127	0.619	0	0.705	0.583	0.146	0.896
TaMAPK53	TaRaf87	4565.A0A3B6SPB2	4565.A0A3B6QMZ9	0	0	0	0.565	0.275	0.134	0.389	0.088	0.603
TaMAPK54	TaMAPKK9	4565.A0A3B6SKC9	4565.A0A077RVQ4	0	0	0.121	0.647	0	0.705	0.583	0.146	0.895
TaMAPK54	TaMEKK5	4565.A0A3B6SKC9	4565.A0A3B6AWC1	0	0	0.128	0.636	0	0.222	0.186	0.065	0.415
TaMAPK54	TaMAPKK15	4565.A0A3B6SKC9	4565.A0A3B6HW51	0	0	0.126	0.639	0	0.705	0.583	0.146	0.895
TaMAPK54	TaMAPKK14	4565.A0A3B6SKC9	4565.A0A3B6HY95	0	0	0.13	0.63	0	0.705	0.583	0.146	0.896
TaMAPK54	TaMAPKK16	4565.A0A3B6SKC9	4565.A0A3B6HZP7	0	0	0.128	0.644	0	0.705	0.583	0.146	0.896
TaMAPK54	TaMAPKK17	4565.A0A3B6SKC9	4565.A0A3B6I0M7	0	0	0.13	0.621	0	0.705	0.583	0.146	0.896
TaMAPK54	TaMAPKK8	4565.A0A3B6SKC9	4565.A0A3B6IK39	0	0	0.123	0.635	0	0.705	0.583	0.146	0.895

TaMAPK54	TaMAPKK6	4565.A0A3B6SKC9	4565.A0A3B6ILF0	0	0	0.127	0.625	0	0.705	0.583	0.146	0.896
TaMAPK54	TaMAPKK5	4565.A0A3B6SKC9	4565.A0A3B6IMW7	0	0	0.128	0.625	0	0.705	0.583	0.146	0.896
TaMAPK54	TaMAPKK7	4565.A0A3B6SKC9	4565.A0A3B6INV0	0	0	0.129	0.62	0	0.705	0.583	0.146	0.896
TaMAPK54	TaMEKK2	4565.A0A3B6SKC9	4565.A0A3B6JCC4	0	0	0.114	0.607	0	0.222	0.186	0.065	0.405
TaMAPK54	TaMAPKK11	4565.A0A3B6SKC9	4565.A0A3B6JEH0	0	0	0.133	0.634	0	0.705	0.583	0.146	0.896
TaMAPK54	TaMAPKK12	4565.A0A3B6SKC9	4565.A0A3B6JG06	0	0	0.131	0.615	0	0.705	0.583	0.146	0.896
TaMAPK54	TaMEKK14	4565.A0A3B6SKC9	4565.A0A3B6KF43	0	0	0.128	0.624	0	0.222	0.186	0.065	0.414
TaMAPK54	TaMAPKK4	4565.A0A3B6SKC9	4565.A0A3B6KFB5	0	0	0.105	0.61	0	0.705	0.739	0.195	0.937
TaMAPK54	TaMEKK15	4565.A0A3B6SKC9	4565.A0A3B6KPK7	0	0	0.119	0.632	0	0.222	0.186	0.065	0.408
TaMAPK54	TaMAPKK18	4565.A0A3B6SKC9	4565.A0A3B6LJ27	0	0	0	0.59	0	0.705	0.739	0.195	0.932
TaMAPK54	TaMEKK16	4565.A0A3B6SKC9	4565.A0A3B6LW00	0	0	0.118	0.631	0	0.222	0.186	0.065	0.407
TaMAPK54	TaMAPKK2	4565.A0A3B6SKC9	4565.A0A3B6LYW0	0	0	0	0.601	0	0.705	0.886	0.195	0.97
TaMAPK54	TaMAPKK3	4565.A0A3B6SKC9	4565.A0A3B6MNP8	0	0	0	0.589	0	0.705	0.739	0.195	0.932
TaMAPK54	TaMEKK11	4565.A0A3B6SKC9	4565.A0A3B6N0D8	0	0	0.117	0.63	0	0.222	0.186	0.065	0.407
TaMAPK54	TaMAPKK13	4565.A0A3B6SKC9	4565.A0A3B6N2X8	0	0	0	0.597	0	0.705	0.886	0.195	0.97
TaMAPK54	TaMAPKK1	4565.A0A3B6SKC9	4565.A0A3B6QJ87	0	0	0.141	0.606	0	0.705	0.583	0.146	0.897
TaMAPK54	TaRaf87	4565.A0A3B6SKC9	4565.A0A3B6QMZ9	0	0	0	0.579	0.16	0.134	0.389	0.088	0.54
TaMAPK6	TaMAPKK9	4565.A0A3B6SCW0	4565.A0A077RVQ4	0	0	0.13	0.633	0	0.705	0.763	0.146	0.941

TaMAPK6	TaMEKK5	4565.A0A3 B6SCW0	4565.A0A3 B6AWC1	0	0	0.126	0.635	0	0.222	0.186	0.065	0.413
TaMAPK6	TaRaf111	4565.A0A3 B6SCW0	4565.A0A3 B6FHS8	0	0	0	0	0.07	0.141	0.15	0.297	0.458
TaMAPK6	TaMAPKK 15	4565.A0A3 B6SCW0	4565.A0A3 B6HW51	0	0	0.13	0.645	0	0.705	0.763	0.146	0.941
TaMAPK6	TaMAPKK 14	4565.A0A3 B6SCW0	4565.A0A3 B6HY95	0	0	0.132	0.649	0	0.705	0.763	0.146	0.941
TaMAPK6	TaMAPKK 16	4565.A0A3 B6SCW0	4565.A0A3 B6HZP7	0	0	0.136	0.652	0	0.705	0.763	0.146	0.941
TaMAPK6	TaMAPKK 17	4565.A0A3 B6SCW0	4565.A0A3 B6I0M7	0	0	0.129	0.639	0	0.705	0.763	0.146	0.941
TaMAPK6	TaMAPKK 8	4565.A0A3 B6SCW0	4565.A0A3 B6IK39	0	0	0.128	0.643	0	0.705	0.763	0.146	0.94
TaMAPK6	TaMAPKK 6	4565.A0A3 B6SCW0	4565.A0A3 B6ILF0	0	0	0.129	0.633	0	0.705	0.763	0.146	0.941
TaMAPK6	TaMAPKK 5	4565.A0A3 B6SCW0	4565.A0A3 B6IMW7	0	0	0.128	0.65	0	0.705	0.763	0.146	0.941
TaMAPK6	TaMAPKK 7	4565.A0A3 B6SCW0	4565.A0A3 B6INV0	0	0	0.131	0.637	0	0.705	0.763	0.146	0.941
TaMAPK6	TaMEKK2	4565.A0A3 B6SCW0	4565.A0A3 B6JCC4	0	0	0.107	0.625	0	0.222	0.186	0.065	0.4
TaMAPK6	TaMAPKK 11	4565.A0A3 B6SCW0	4565.A0A3 B6JEH0	0	0	0.134	0.644	0	0.705	0.763	0.146	0.941
TaMAPK6	TaMAPKK 12	4565.A0A3 B6SCW0	4565.A0A3 B6JG06	0	0	0.133	0.636	0	0.705	0.946	0.146	0.986
TaMAPK6	TaMEKK1 4	4565.A0A3 B6SCW0	4565.A0A3 B6KF43	0	0	0.124	0.622	0	0.222	0.186	0.065	0.411
TaMAPK6	TaMAPKK 4	4565.A0A3 B6SCW0	4565.A0A3 B6KFB5	0	0	0.101	0.636	0	0.705	0.825	0.209	0.958
TaMAPK6	TaMEKK1 5	4565.A0A3 B6SCW0	4565.A0A3 B6KPK7	0	0	0.116	0.622	0	0.222	0.186	0.065	0.406
TaMAPK6	TaMAPKK 18	4565.A0A3 B6SCW0	4565.A0A3 B6LJ27	0	0	0	0.602	0	0.705	0.825	0.209	0.955
TaMAPK6	TaMEKK1 6	4565.A0A3 B6SCW0	4565.A0A3 B6LW00	0	0	0.115	0.622	0	0.222	0.186	0.065	0.406

TaMAPK6	TaMAPKK 2	4565.A0A3 B6SCW0	4565.A0A3 B6LYW0	0	0	0	0.612	0	0.705	0.927	0.209	0.981
TaMAPK6	TaMAPKK 3	4565.A0A3 B6SCW0	4565.A0A3 B6MNP8	0	0	0	0.605	0	0.705	0.825	0.209	0.955
TaMAPK6	TaMEKK1 1	4565.A0A3 B6SCW0	4565.A0A3 B6N0D8	0	0	0.115	0.62	0	0.222	0.186	0.065	0.405
TaMAPK6	TaMAPKK 13	4565.A0A3 B6SCW0	4565.A0A3 B6N2X8	0	0	0	0.617	0	0.705	0.927	0.209	0.981
TaMAPK6	TaMAPKK 1	4565.A0A3 B6SCW0	4565.A0A3 B6QJ87	0	0	0.148	0.625	0	0.705	0.883	0.146	0.971
TaMAPK6	TaRaf87	4565.A0A3 B6SCW0	4565.A0A3 B6QMZ9	0	0	0	0.599	0.16	0.134	0.389	0.088	0.54
TaMAPK7	TaMAPKK 9	4565.A0A3 B6TM72	4565.A0A0 77RVQ4	0	0	0.117	0.625	0	0.705	0.583	0.146	0.894
TaMAPK7	TaMEKK5	4565.A0A3 B6TM72	4565.A0A3 B6AWC1	0	0	0.151	0.595	0	0.222	0.186	0.065	0.43
TaMAPK7	TaMAPKK 15	4565.A0A3 B6TM72	4565.A0A3 B6HW51	0	0	0.122	0.623	0	0.705	0.583	0.146	0.895
TaMAPK7	TaMAPKK 14	4565.A0A3 B6TM72	4565.A0A3 B6HY95	0	0	0.12	0.619	0	0.705	0.583	0.146	0.895
TaMAPK7	TaMAPKK 16	4565.A0A3 B6TM72	4565.A0A3 B6HZP7	0	0	0.121	0.625	0	0.705	0.583	0.146	0.895
TaMAPK7	TaMAPKK 17	4565.A0A3 B6TM72	4565.A0A3 B6I0M7	0	0	0.12	0.626	0	0.705	0.583	0.146	0.895
TaMAPK7	TaMAPKK 8	4565.A0A3 B6TM72	4565.A0A3 B6IK39	0	0	0.113	0.624	0	0.705	0.583	0.146	0.894
TaMAPK7	TaMAPKK 6	4565.A0A3 B6TM72	4565.A0A3 B6ILF0	0	0	0.121	0.619	0	0.705	0.583	0.146	0.895
TaMAPK7	TaMAPKK 5	4565.A0A3 B6TM72	4565.A0A3 B6IMW7	0	0	0.117	0.616	0	0.705	0.583	0.146	0.894
TaMAPK7	TaMAPKK 7	4565.A0A3 B6TM72	4565.A0A3 B6INV0	0	0	0.122	0.624	0	0.705	0.583	0.146	0.895
TaMAPK7	TaMEKK2	4565.A0A3 B6TM72	4565.A0A3 B6JCC4	0	0	0.131	0.597	0	0.222	0.186	0.065	0.417
TaMAPK7	TaMAPKK 11	4565.A0A3 B6TM72	4565.A0A3 B6JEH0	0	0	0.118	0.624	0	0.705	0.583	0.146	0.895

TaMAPK7	TaMAPKK 12	4565.A0A3 B6TM72	4565.A0A3 B6JG06	0	0	0.125	0.618	0	0.705	0.583	0.146	0.895
TaMAPK7	TaMEKK1 4	4565.A0A3 B6TM72	4565.A0A3 B6KF43	0	0	0.145	0.609	0	0.222	0.186	0.065	0.426
TaMAPK7	TaMAPKK 4	4565.A0A3 B6TM72	4565.A0A3 B6KFB5	0	0	0.103	0.626	0	0.705	0.583	0.146	0.893
TaMAPK7	TaMEKK1 7	4565.A0A3 B6TM72	4565.A0A3 B6KFL8	0	0	0.16	0.609	0	0.27	0.063	0.088	0.406
TaMAPK7	TaMEKK1 5	4565.A0A3 B6TM72	4565.A0A3 B6KPK7	0	0	0.142	0.594	0	0.222	0.186	0.065	0.424
TaMAPK7	TaMAPKK 18	4565.A0A3 B6TM72	4565.A0A3 B6LJ27	0	0	0	0.591	0	0.705	0.583	0.146	0.885
TaMAPK7	TaMEKK2 4	4565.A0A3 B6TM72	4565.A0A3 B6LLV5	0	0	0.159	0.609	0	0.27	0.063	0.088	0.405
TaMAPK7	TaMEKK1 6	4565.A0A3 B6TM72	4565.A0A3 B6LW00	0	0	0.141	0.594	0	0.222	0.186	0.065	0.423
TaMAPK7	TaMAPKK 2	4565.A0A3 B6TM72	4565.A0A3 B6LYW0	0	0	0	0.594	0	0.705	0.583	0.146	0.885
TaMAPK7	TaMAPKK 3	4565.A0A3 B6TM72	4565.A0A3 B6MNP8	0	0	0	0.593	0	0.705	0.583	0.146	0.885
TaMAPK7	TaMEKK2 9	4565.A0A3 B6TM72	4565.A0A3 B6MSP6	0	0	0.161	0.609	0	0.27	0.063	0.088	0.406
TaMAPK7	TaMEKK1 1	4565.A0A3 B6TM72	4565.A0A3 B6N0D8	0	0	0.141	0.591	0	0.222	0.186	0.065	0.423
TaMAPK7	TaMAPKK 13	4565.A0A3 B6TM72	4565.A0A3 B6N2X8	0	0	0	0.593	0	0.705	0.583	0.146	0.885
TaMAPK7	TaMAPKK 1	4565.A0A3 B6TM72	4565.A0A3 B6QJ87	0	0	0.132	0.625	0	0.705	0.583	0.146	0.896
TaMAPK7	TaRaf87	4565.A0A3 B6TM72	4565.A0A3 B6QMZ9	0	0	0	0.585	0.277	0.134	0.389	0.088	0.604
TaMAPK8	TaMAPKK 9	4565.A0A3 B6GTS3	4565.A0A0 77RVQ4	0	0	0.112	0.617	0	0.705	0.583	0.146	0.894
TaMAPK8	TaMEKK5	4565.A0A3 B6GTS3	4565.A0A3 B6AWC1	0	0	0.139	0.584	0	0.222	0.186	0.065	0.422
TaMAPK8	TaMEKK2	4565.A0A3 B6GTS3	4565.A0A3 B6JCC4	0	0	0.126	0.574	0	0.222	0.186	0.065	0.413

TaMAPK8	TaMEKK16	4565.A0A3B6GTS3	4565.A0A3B6LW00	0	0	0.128	0.578	0	0.222	0.186	0.065	0.414
TaMAPK8	TaMEKK11	4565.A0A3B6GTS3	4565.A0A3B6N0D8	0	0	0.127	0.576	0	0.222	0.186	0.065	0.414
TaMAPK8	TaMEKK15	4565.A0A3B6GTS3	4565.A0A3B6KPK7	0	0	0.129	0.578	0	0.222	0.186	0.065	0.415
TaMAPK8	TaMEKK17	4565.A0A3B6GTS3	4565.A0A3B6KFL8	0	0	0.179	0.577	0	0.27	0.063	0.088	0.419
TaMAPK8	TaMEKK24	4565.A0A3B6GTS3	4565.A0A3B6LLV5	0	0	0.178	0.577	0	0.27	0.063	0.088	0.419
TaMAPK8	TaMEKK29	4565.A0A3B6GTS3	4565.A0A3B6MSP6	0	0	0.179	0.577	0	0.27	0.063	0.088	0.419
TaMAPK8	TaMEKK14	4565.A0A3B6GTS3	4565.A0A3B6KF43	0	0	0.138	0.586	0	0.222	0.186	0.065	0.421
TaMAPK8	TaRaf87	4565.A0A3B6GTS3	4565.A0A3B6QMZ9	0	0	0	0.561	0.277	0.134	0.389	0.088	0.604
TaMAPK8	TaMAPKK18	4565.A0A3B6GTS3	4565.A0A3B6LJ27	0	0	0	0.59	0	0.705	0.583	0.146	0.885
TaMAPK8	TaMAPKK4	4565.A0A3B6GTS3	4565.A0A3B6KFB5	0	0	0	0.647	0	0.705	0.583	0.146	0.885
TaMAPK8	TaMAPKK13	4565.A0A3B6GTS3	4565.A0A3B6N2X8	0	0	0	0.581	0	0.705	0.583	0.146	0.885
TaMAPK8	TaMAPKK2	4565.A0A3B6GTS3	4565.A0A3B6LYW0	0	0	0	0.585	0	0.705	0.583	0.146	0.885
TaMAPK8	TaMAPKK3	4565.A0A3B6GTS3	4565.A0A3B6MNP8	0	0	0	0.582	0	0.705	0.583	0.146	0.885
TaMAPK8	TaMAPKK14	4565.A0A3B6GTS3	4565.A0A3B6HY95	0	0	0.114	0.607	0	0.705	0.583	0.146	0.894
TaMAPK8	TaMAPKK11	4565.A0A3B6GTS3	4565.A0A3B6JEH0	0	0	0.115	0.614	0	0.705	0.583	0.146	0.894
TaMAPK8	TaMAPKK15	4565.A0A3B6GTS3	4565.A0A3B6HW51	0	0	0.117	0.614	0	0.705	0.583	0.146	0.894
TaMAPK8	TaMAPKK8	4565.A0A3B6GTS3	4565.A0A3B6IK39	0	0	0.111	0.616	0	0.705	0.583	0.146	0.894
TaMAPK8	TaMAPKK6	4565.A0A3B6GTS3	4565.A0A3B6ILF0	0	0	0.118	0.607	0	0.705	0.583	0.146	0.894

TaMAPK8	TaMAPKK5	4565.A0A3B6GTS3	4565.A0A3B6IMW7	0	0	0.115	0.606	0	0.705	0.583	0.146	0.894
TaMAPK8	TaMAPKK16	4565.A0A3B6GTS3	4565.A0A3B6HZP7	0	0	0.115	0.616	0	0.705	0.583	0.146	0.894
TaMAPK8	TaMAPKK17	4565.A0A3B6GTS3	4565.A0A3B6I0M7	0	0	0.117	0.611	0	0.705	0.583	0.146	0.894
TaMAPK8	TaMAPKK12	4565.A0A3B6GTS3	4565.A0A3B6JG06	0	0	0.121	0.605	0	0.705	0.583	0.146	0.895
TaMAPK8	TaMAPKK7	4565.A0A3B6GTS3	4565.A0A3B6INV0	0	0	0.12	0.608	0	0.705	0.583	0.146	0.895
TaMAPK8	TaMAPKK1	4565.A0A3B6GTS3	4565.A0A3B6QJ87	0	0	0.13	0.611	0	0.705	0.583	0.146	0.896
TaMAPKK1	TaMEKK7	4565.A0A3B6QJ87	4565.A0A077RUI2	0	0	0.179	0.701	0.05	0.652	0.431	0.136	0.842
TaMAPKK1	TaRaf18	4565.A0A3B6QJ87	4565.A0A077RY41	0	0	0.191	0.657	0	0.172	0.176	0	0.4
TaMAPKK1	TaMEKK9	4565.A0A3B6QJ87	4565.A0A077S2G5	0	0	0.202	0.638	0.05	0.652	0.431	0.136	0.846
TaMAPKK1	TaMAPKKK4	4565.A0A3B6QJ87	4565.A0A3B6AY64	0	0	0.102	0.726	0	0.176	0.579	0.195	0.715
TaMAPKK1	TaMEKK1	4565.A0A3B6QJ87	4565.A0A3B6B3I4	0	0	0.154	0.745	0.05	0.652	0.883	0.136	0.966
TaMAPKK1	TaMEKK20	4565.A0A3B6QJ87	4565.A0A3B6B6T5	0	0	0	0.671	0.05	0.652	0.431	0.136	0.815
TaMAPKK1	TaMAPKKK5	4565.A0A3B6QJ87	4565.A0A3B6C4T6	0	0	0.101	0.726	0	0.176	0.579	0.195	0.715
TaMAPKK1	TaMEKK18	4565.A0A3B6QJ87	4565.A0A3B6CEA6	0	0	0	0.671	0.05	0.652	0.431	0.136	0.815
TaMAPKK1	TaMAPKKK6	4565.A0A3B6QJ87	4565.A0A3B6DEP0	0	0	0.102	0.726	0	0.176	0.579	0.195	0.715
TaMAPKK1	TaRaf60	4565.A0A3B6QJ87	4565.A0A3B6EHW0	0	0	0.207	0.623	0	0.172	0.176	0	0.412
TaMAPKK1	TaMEKK8	4565.A0A3B6QJ87	4565.A0A3B6FNU8	0	0	0.18	0.691	0.05	0.652	0.431	0.136	0.842
TaMAPKK1	TaRaf5	4565.A0A3B6QJ87	4565.A0A3B6GYQ1	0	0	0.206	0.625	0	0.172	0.176	0	0.411

TaMAPKK1	TaMEKK17	4565.A0A3B6QJ87	4565.A0A3B6KFL8	0	0	0.153	0.744	0.05	0.652	0.883	0.136	0.966
TaMAPKK1	TaRaf41	4565.A0A3B6QJ87	4565.A0A3B6KLD7	0	0	0.205	0.62	0	0.172	0.176	0	0.41
TaMAPKK1	TaMEKK24	4565.A0A3B6QJ87	4565.A0A3B6LLV5	0	0	0.153	0.744	0.05	0.652	0.883	0.136	0.966
TaMAPKK1	TaRaf56	4565.A0A3B6QJ87	4565.A0A3B6LPY3	0	0	0.217	0.593	0	0.172	0.176	0	0.419
TaMAPKK1	TaMEKK29	4565.A0A3B6QJ87	4565.A0A3B6MSP6	0	0	0.153	0.744	0.05	0.652	0.883	0.136	0.966
TaMAPKK1	TaMEKK4	4565.A0A3B6QJ87	4565.A0A3B6NRN9	0	0	0.157	0.73	0.05	0.652	0.883	0.136	0.966
TaMAPKK1	TaRaf91	4565.A0A3B6QJ87	4565.A0A3B6PMI5	0	0	0.125	0.644	0	0.172	0.489	0	0.597
TaMAPKK1	TaMEKK4-1	4565.A0A3B6QJ87	4565.A0A3B6PNI6	0	0	0.158	0.73	0.05	0.652	0.883	0.136	0.966
TaMAPKK1	TaRaf62	4565.A0A3B6QJ87	4565.A9RAA9	0	0	0.193	0.662	0	0.172	0.176	0	0.401
TaMAPKK1	TaRaf7	4565.A0A3B6QJ87	4565.A0A3B6TBH5	0	0	0.215	0.595	0	0.172	0.176	0	0.418
TaMAPKK11	TaMEKK7	4565.A0A3B6JEH0	4565.A0A077RUI2	0	0	0.176	0.695	0.05	0.652	0.431	0.136	0.841
TaMAPKK11	TaRaf18	4565.A0A3B6JEH0	4565.A0A077RY41	0	0	0.192	0.637	0	0.172	0.176	0	0.401
TaMAPKK11	TaMEKK9	4565.A0A3B6JEH0	4565.A0A077S2G5	0	0	0.19	0.652	0.05	0.652	0.431	0.136	0.844
TaMAPKK11	TaRaf95	4565.A0A3B6JEH0	4565.A0A1D5UN01	0	0	0.193	0.601	0	0.172	0.176	0	0.401
TaMAPKK11	TaRaf88	4565.A0A3B6JEH0	4565.A0A3B5Z5X1	0	0	0.197	0.618	0	0.172	0.176	0	0.404
TaMAPKK11	TaRaf30	4565.A0A3B6JEH0	4565.A0A3B6A1Z4	0	0	0.196	0.62	0	0.172	0.176	0	0.404
TaMAPKK11	TaRaf102	4565.A0A3B6JEH0	4565.A0A3B6AQT7	0	0	0.232	0	0	0.172	0.176	0	0.43
TaMAPKK11	TaMAPKKKK4	4565.A0A3B6JEH0	4565.A0A3B6AY64	0	0	0.109	0.682	0	0.176	0.579	0.195	0.718

TaMAPKK11	TaMEKK1	4565.A0A3B6JEH0	4565.A0A3B6B3I4	0	0	0.152	0.722	0.05	0.652	0.573	0.136	0.877
TaMAPKK11	TaMEKK20	4565.A0A3B6JEH0	4565.A0A3B6B6T5	0	0	0	0.702	0.05	0.652	0.431	0.136	0.815
TaMAPKK11	TaRaf105	4565.A0A3B6JEH0	4565.A0A3B6C472	0	0	0.254	0	0	0.172	0.176	0	0.446
TaMAPKK11	TaMAPKKK5	4565.A0A3B6JEH0	4565.A0A3B6C4T6	0	0	0.109	0.682	0	0.176	0.579	0.195	0.717
TaMAPKK11	TaMEKK18	4565.A0A3B6JEH0	4565.A0A3B6CEA6	0	0	0	0.703	0.05	0.652	0.431	0.136	0.815
TaMAPKK11	TaMAPKKK6	4565.A0A3B6JEH0	4565.A0A3B6DEP0	0	0	0.109	0.684	0	0.176	0.579	0.195	0.718
TaMAPKK11	TaRaf60	4565.A0A3B6JEH0	4565.A0A3B6EHW0	0	0	0.201	0.587	0	0.172	0.176	0	0.407
TaMAPKK11	TaMEKK8	4565.A0A3B6JEH0	4565.A0A3B6FNU8	0	0	0.168	0.71	0.05	0.652	0.431	0.136	0.84
TaMAPKK11	TaRaf73	4565.A0A3B6JEH0	4565.A0A3B6GRN8	0	0	0.244	0	0	0.172	0.176	0	0.439
TaMAPKK11	TaRaf5	4565.A0A3B6JEH0	4565.A0A3B6GYQ1	0	0	0.198	0.594	0	0.172	0.176	0	0.405
TaMAPKK11	TaRaf46	4565.A0A3B6JEH0	4565.A0A3B6MW69	0	0	0.194	0.628	0	0.172	0.176	0	0.402
TaMAPKK11	TaRaf83	4565.A0A3B6JEH0	4565.A0A3B6MWW7	0	0	0.204	0	0	0.172	0.176	0	0.409
TaMAPKK11	TaRaf44	4565.A0A3B6JEH0	4565.A0A3B6KNC2	0	0	0.205	0	0	0.172	0.176	0	0.41
TaMAPKK11	TaRaf62	4565.A0A3B6JEH0	4565.A9RAA9	0	0	0.206	0.598	0	0.172	0.176	0	0.411
TaMAPKK11	TaRaf41	4565.A0A3B6JEH0	4565.A0A3B6KLD7	0	0	0.216	0.586	0	0.172	0.176	0	0.418
TaMAPKK11	TaRaf56	4565.A0A3B6JEH0	4565.A0A3B6LPY3	0	0	0.216	0.584	0	0.172	0.176	0	0.418
TaMAPKK11	TaRaf7	4565.A0A3B6JEH0	4565.A0A3B6TBH5	0	0	0.216	0.593	0	0.172	0.176	0	0.418
TaMAPKK11	TaRaf42	4565.A0A3B6JEH0	4565.A0A3B6U554	0	0	0.223	0	0	0.172	0.176	0	0.423

TaMAPKK 11	TaRaf45	4565.A0A3 B6JEH0	4565.A0A3 B6KN82	0	0	0.235	0	0	0.172	0.176	0	0.432
TaMAPKK 11	TaRaf72	4565.A0A3 B6JEH0	4565.A0A3 B6MXW5	0	0	0.247	0	0	0.172	0.176	0	0.441
TaMAPKK 11	TaRaf50	4565.A0A3 B6JEH0	4565.A0A3 B6RET0	0	0	0.28	0	0	0.172	0.176	0	0.465
TaMAPKK 11	TaRaf43	4565.A0A3 B6JEH0	4565.A0A3 B6KNC6	0	0	0.292	0	0	0.172	0.176	0	0.474
TaMAPKK 11	TaRaf91	4565.A0A3 B6JEH0	4565.A0A3 B6PMI5	0	0	0.136	0.604	0	0.172	0.489	0	0.602
TaMAPKK 11	TaMEKK4-1	4565.A0A3 B6JEH0	4565.A0A3 B6PNI6	0	0	0.163	0.701	0.05	0.652	0.573	0.136	0.879
TaMAPKK 11	TaMEKK4	4565.A0A3 B6JEH0	4565.A0A3 B6NRN9	0	0	0.163	0.699	0.05	0.652	0.573	0.136	0.879
TaMAPKK 11	TaMEKK2 4	4565.A0A3 B6JEH0	4565.A0A3 B6LLV5	0	0	0.156	0.74	0.05	0.652	0.614	0.136	0.89
TaMAPKK 11	TaMEKK2 9	4565.A0A3 B6JEH0	4565.A0A3 B6MSP6	0	0	0.156	0.74	0.05	0.652	0.614	0.136	0.89
TaMAPKK 11	TaMEKK1 7	4565.A0A3 B6JEH0	4565.A0A3 B6KFL8	0	0	0.156	0.74	0.05	0.652	0.614	0.136	0.89
TaMAPKK 12	TaMEKK7	4565.A0A3 B6JG06	4565.A0A0 77RUI2	0	0	0.182	0.702	0.05	0.652	0.431	0.136	0.842
TaMAPKK 12	TaRaf18	4565.A0A3 B6JG06	4565.A0A0 77RY41	0	0	0.21	0.621	0	0.172	0.176	0	0.414
TaMAPKK 12	TaMEKK9	4565.A0A3 B6JG06	4565.A0A0 77S2G5	0	0	0.199	0.647	0.05	0.652	0.431	0.136	0.846
TaMAPKK 12	TaRaf29	4565.A0A3 B6JG06	4565.A0A1 D5UHD7	0	0	0.194	0.634	0	0.172	0.176	0	0.402
TaMAPKK 12	TaRaf95	4565.A0A3 B6JG06	4565.A0A1 D5UN01	0	0	0.192	0.603	0	0.172	0.176	0	0.401
TaMAPKK 12	TaRaf88	4565.A0A3 B6JG06	4565.A0A3 B5Z5X1	0	0	0.213	0.622	0	0.172	0.176	0	0.416
TaMAPKK 12	TaRaf30	4565.A0A3 B6JG06	4565.A0A3 B6A1Z4	0	0	0.211	0.621	0	0.172	0.176	0	0.415
TaMAPKK 12	TaMAPKK KK4	4565.A0A3 B6JG06	4565.A0A3 B6AY64	0	0	0.114	0.685	0	0.176	0.579	0.195	0.719

TaMAPKK12	TaMEKK1	4565.A0A3B6JG06	4565.A0A3B6B3I4	0	0	0.153	0.735	0.05	0.652	0.573	0.136	0.877
TaMAPKK12	TaMEKK20	4565.A0A3B6JG06	4565.A0A3B6B6T5	0	0	0	0.686	0.05	0.652	0.431	0.136	0.815
TaMAPKK12	TaRaf79	4565.A0A3B6JG06	4565.A0A3B6B9C7	0	0	0.194	0.63	0	0.172	0.176	0	0.402
TaMAPKK12	TaMAPKKK5	4565.A0A3B6JG06	4565.A0A3B6C4T6	0	0	0.113	0.684	0	0.176	0.579	0.195	0.719
TaMAPKK12	TaMEKK18	4565.A0A3B6JG06	4565.A0A3B6CEA6	0	0	0	0.686	0.05	0.652	0.431	0.136	0.815
TaMAPKK12	TaMAPKKK6	4565.A0A3B6JG06	4565.A0A3B6DEP0	0	0	0.114	0.684	0	0.176	0.579	0.195	0.719
TaMAPKK12	TaRaf59	4565.A0A3B6JG06	4565.A0A3B6E9E2	0	0	0.196	0.609	0	0.172	0.176	0	0.404
TaMAPKK12	TaRaf60	4565.A0A3B6JG06	4565.A0A3B6EHW0	0	0	0.218	0.591	0	0.172	0.176	0	0.419
TaMAPKK12	TaMEKK8	4565.A0A3B6JG06	4565.A0A3B6FNU8	0	0	0.181	0.695	0.05	0.652	0.431	0.136	0.842
TaMAPKK12	TaRaf5	4565.A0A3B6JG06	4565.A0A3B6GYQ1	0	0	0.218	0.595	0	0.172	0.176	0	0.42
TaMAPKK12	TaRaf58	4565.A0A3B6JG06	4565.A0A3B6N1Y2	0	0	0.195	0.589	0	0.172	0.176	0	0.403
TaMAPKK12	TaRaf46	4565.A0A3B6JG06	4565.A0A3B6MW69	0	0	0.198	0.647	0	0.172	0.176	0	0.405
TaMAPKK12	TaRaf21	4565.A0A3B6JG06	4565.A0A3B6TVC5	0	0	0.198	0.594	0	0.172	0.176	0	0.405
TaMAPKK12	TaRaf1	4565.A0A3B6JG06	4565.A0A3B6NP57	0	0	0.202	0.586	0	0.172	0.176	0	0.408
TaMAPKK12	TaRaf7	4565.A0A3B6JG06	4565.A0A3B6TBH5	0	0	0.211	0.601	0	0.172	0.176	0	0.414
TaMAPKK12	TaRaf41	4565.A0A3B6JG06	4565.A0A3B6KLD7	0	0	0.212	0.608	0	0.172	0.176	0	0.415
TaMAPKK12	TaRaf62	4565.A0A3B6JG06	4565.A9RAA9	0	0	0.213	0.615	0	0.172	0.176	0	0.416
TaMAPKK12	TaRaf56	4565.A0A3B6JG06	4565.A0A3B6LPY3	0	0	0.217	0.595	0	0.172	0.176	0	0.419

TaMAPKK 12	TaRaf91	4565.A0A3 B6JG06	4565.A0A3 B6PMI5	0	0	0.151	0.597	0	0.172	0.489	0	0.609
TaMAPKK 12	TaMEKK4	4565.A0A3 B6JG06	4565.A0A3 B6NRN9	0	0	0.165	0.708	0.05	0.652	0.573	0.136	0.879
TaMAPKK 12	TaMEKK4-1	4565.A0A3 B6JG06	4565.A0A3 B6PNI6	0	0	0.165	0.71	0.05	0.652	0.573	0.136	0.879
TaMAPKK 12	TaMEKK1 7	4565.A0A3 B6JG06	4565.A0A3 B6KFL8	0	0	0.166	0.729	0.05	0.652	0.614	0.136	0.891
TaMAPKK 12	TaMEKK2 9	4565.A0A3 B6JG06	4565.A0A3 B6MSP6	0	0	0.166	0.729	0.05	0.652	0.614	0.136	0.891
TaMAPKK 12	TaMEKK2 4	4565.A0A3 B6JG06	4565.A0A3 B6LLV5	0	0	0.166	0.729	0.05	0.652	0.614	0.136	0.891
TaMAPKK 13	TaMEKK7	4565.A0A3 B6N2X8	4565.A0A0 77RUI2	0	0	0.111	0.617	0.05	0.652	0.927	0.136	0.978
TaMAPKK 13	TaMEKK9	4565.A0A3 B6N2X8	4565.A0A0 77S2G5	0	0	0.109	0.654	0.05	0.652	0.791	0.136	0.937
TaMAPKK 13	TaMAPKK KK4	4565.A0A3 B6N2X8	4565.A0A3 B6AY64	0	0	0	0.654	0	0.176	0.579	0.195	0.696
TaMAPKK 13	TaMEKK1	4565.A0A3 B6N2X8	4565.A0A3 B6B3I4	0	0	0	0.631	0.05	0.652	0.431	0.136	0.815
TaMAPKK 13	TaMEKK2 0	4565.A0A3 B6N2X8	4565.A0A3 B6B6T5	0	0	0	0.631	0.05	0.652	0.431	0.136	0.815
TaMAPKK 13	TaMAPKK KK5	4565.A0A3 B6N2X8	4565.A0A3 B6C4T6	0	0	0	0.655	0	0.176	0.579	0.195	0.696
TaMAPKK 13	TaMEKK1 8	4565.A0A3 B6N2X8	4565.A0A3 B6CEA6	0	0	0	0.631	0.05	0.652	0.431	0.136	0.815
TaMAPKK 13	TaMAPKK KK6	4565.A0A3 B6N2X8	4565.A0A3 B6DEP0	0	0	0	0.655	0	0.176	0.579	0.195	0.696
TaMAPKK 13	TaMEKK8	4565.A0A3 B6N2X8	4565.A0A3 B6FNU8	0	0	0.107	0.628	0.05	0.652	0.946	0.136	0.983
TaMAPKK 13	TaMEKK1 7	4565.A0A3 B6N2X8	4565.A0A3 B6KFL8	0	0	0.138	0.644	0.05	0.652	0.431	0.136	0.834
TaMAPKK 13	TaMEKK2 4	4565.A0A3 B6N2X8	4565.A0A3 B6LLV5	0	0	0.14	0.643	0.05	0.652	0.431	0.136	0.834
TaMAPKK 13	TaMEKK2 9	4565.A0A3 B6N2X8	4565.A0A3 B6MSP6	0	0	0.138	0.644	0.05	0.652	0.431	0.136	0.834

TaMAPKK13	TaMEKK4-1	4565.A0A3B6N2X8	4565.A0A3B6PNI6	0	0	0	0.625	0.05	0.652	0.431	0.136	0.815
TaMAPKK13	TaMEKK4	4565.A0A3B6N2X8	4565.A0A3B6NRN9	0	0	0	0.623	0.05	0.652	0.431	0.136	0.815
TaMAPKK14	TaMEKK7	4565.A0A3B6HY95	4565.A0A077RUI2	0	0	0.175	0.705	0.05	0.652	0.431	0.136	0.841
TaMAPKK14	TaRaf18	4565.A0A3B6HY95	4565.A0A077RY41	0	0	0.202	0.623	0	0.172	0.176	0	0.408
TaMAPKK14	TaMEKK9	4565.A0A3B6HY95	4565.A0A077S2G5	0	0	0.189	0.662	0.05	0.652	0.431	0.136	0.844
TaMAPKK14	TaRaf95	4565.A0A3B6HY95	4565.A0A1D5UN01	0	0	0.196	0.594	0	0.172	0.176	0	0.403
TaMAPKK14	TaRaf88	4565.A0A3B6HY95	4565.A0A3B5Z5X1	0	0	0.207	0.612	0	0.172	0.176	0	0.412
TaMAPKK14	TaRaf30	4565.A0A3B6HY95	4565.A0A3B6A1Z4	0	0	0.205	0.613	0	0.172	0.176	0	0.41
TaMAPKK14	TaRaf102	4565.A0A3B6HY95	4565.A0A3B6AQT7	0	0	0.214	0	0	0.172	0.176	0	0.416
TaMAPKK14	TaRaf19	4565.A0A3B6HY95	4565.A0A3B6AWY0	0	0	0.243	0	0	0.172	0.176	0	0.438
TaMAPKK14	TaMAPKKKK4	4565.A0A3B6HY95	4565.A0A3B6AY64	0	0	0.109	0.69	0	0.176	0.579	0.195	0.718
TaMAPKK14	TaMEKK1	4565.A0A3B6HY95	4565.A0A3B6B3I4	0	0	0.158	0.728	0.05	0.652	0.573	0.136	0.878
TaMAPKK14	TaMEKK20	4565.A0A3B6HY95	4565.A0A3B6B6T5	0	0	0	0.699	0.05	0.652	0.431	0.136	0.815
TaMAPKK14	TaRaf105	4565.A0A3B6HY95	4565.A0A3B6C472	0	0	0.228	0	0	0.172	0.176	0	0.427
TaMAPKK14	TaMAPKKKK5	4565.A0A3B6HY95	4565.A0A3B6C4T6	0	0	0.109	0.69	0	0.176	0.579	0.195	0.717
TaMAPKK14	TaMEKK18	4565.A0A3B6HY95	4565.A0A3B6CEA6	0	0	0	0.699	0.05	0.652	0.431	0.136	0.815
TaMAPKK14	TaMAPKKKK6	4565.A0A3B6HY95	4565.A0A3B6DEP0	0	0	0.11	0.69	0	0.176	0.579	0.195	0.718
TaMAPKK14	TaRaf60	4565.A0A3B6HY95	4565.A0A3B6EHW0	0	0	0.209	0.585	0	0.172	0.176	0	0.413

TaMAPKK14	TaMEKK8	4565.A0A3B6HY95	4565.A0A3B6FNU8	0	0	0.177	0.695	0.05	0.652	0.431	0.136	0.841
TaMAPKK14	TaRaf73	4565.A0A3B6HY95	4565.A0A3B6GRN8	0	0	0.222	0	0	0.172	0.176	0	0.422
TaMAPKK14	TaRaf5	4565.A0A3B6HY95	4565.A0A3B6GYQ1	0	0	0.21	0.589	0	0.172	0.176	0	0.414
TaMAPKK14	TaRaf46	4565.A0A3B6HY95	4565.A0A3B6MW69	0	0	0.201	0.616	0	0.172	0.176	0	0.407
TaMAPKK14	TaRaf62	4565.A0A3B6HY95	4565.A9RAA9	0	0	0.212	0.601	0	0.172	0.176	0	0.415
TaMAPKK14	TaRaf42	4565.A0A3B6HY95	4565.A0A3B6U554	0	0	0.213	0	0	0.172	0.176	0	0.416
TaMAPKK14	TaRaf41	4565.A0A3B6HY95	4565.A0A3B6KLD7	0	0	0.213	0.592	0	0.172	0.176	0	0.416
TaMAPKK14	TaRaf7	4565.A0A3B6HY95	4565.A0A3B6TBH5	0	0	0.216	0.594	0	0.172	0.176	0	0.418
TaMAPKK14	TaRaf56	4565.A0A3B6HY95	4565.A0A3B6LPY3	0	0	0.217	0.586	0	0.172	0.176	0	0.419
TaMAPKK14	TaRaf72	4565.A0A3B6HY95	4565.A0A3B6MXW5	0	0	0.232	0	0	0.172	0.176	0	0.43
TaMAPKK14	TaRaf50	4565.A0A3B6HY95	4565.A0A3B6RET0	0	0	0.266	0	0	0.172	0.176	0	0.455
TaMAPKK14	TaRaf43	4565.A0A3B6HY95	4565.A0A3B6KNC6	0	0	0.281	0	0	0.172	0.176	0	0.466
TaMAPKK14	TaRaf91	4565.A0A3B6HY95	4565.A0A3B6PMI5	0	0	0.133	0.604	0	0.172	0.489	0	0.601
TaMAPKK14	TaMEKK4-1	4565.A0A3B6HY95	4565.A0A3B6PNI6	0	0	0.174	0.695	0.05	0.652	0.573	0.136	0.88
TaMAPKK14	TaMEKK4	4565.A0A3B6HY95	4565.A0A3B6NRN9	0	0	0.175	0.693	0.05	0.652	0.573	0.136	0.881
TaMAPKK14	TaMEKK29	4565.A0A3B6HY95	4565.A0A3B6MSP6	0	0	0.16	0.739	0.05	0.652	0.614	0.136	0.89
TaMAPKK14	TaMEKK24	4565.A0A3B6HY95	4565.A0A3B6LLV5	0	0	0.16	0.739	0.05	0.652	0.614	0.136	0.89
TaMAPKK14	TaMEKK17	4565.A0A3B6HY95	4565.A0A3B6KFL8	0	0	0.16	0.739	0.05	0.652	0.614	0.136	0.89

TaMAPKK15	TaMEKK7	4565.A0A3B6HW51	4565.A0A077RUI2	0	0	0.174	0.709	0.05	0.652	0.431	0.136	0.841
TaMAPKK15	TaRaf18	4565.A0A3B6HW51	4565.A0A077RY41	0	0	0.201	0.619	0	0.172	0.176	0	0.407
TaMAPKK15	TaMEKK9	4565.A0A3B6HW51	4565.A0A077S2G5	0	0	0.191	0.661	0.05	0.652	0.431	0.136	0.844
TaMAPKK15	TaRaf95	4565.A0A3B6HW51	4565.A0A1D5UN01	0	0	0.201	0.598	0	0.172	0.176	0	0.407
TaMAPKK15	TaRaf88	4565.A0A3B6HW51	4565.A0A3B5Z5X1	0	0	0.206	0.608	0	0.172	0.176	0	0.411
TaMAPKK15	TaRaf30	4565.A0A3B6HW51	4565.A0A3B6A1Z4	0	0	0.203	0.609	0	0.172	0.176	0	0.409
TaMAPKK15	TaRaf102	4565.A0A3B6HW51	4565.A0A3B6AQT7	0	0	0.214	0	0	0.172	0.176	0	0.416
TaMAPKK15	TaRaf19	4565.A0A3B6HW51	4565.A0A3B6AWY0	0	0	0.249	0	0	0.172	0.176	0	0.442
TaMAPKK15	TaMAPKKK4	4565.A0A3B6HW51	4565.A0A3B6AY64	0	0	0.111	0.683	0	0.176	0.579	0.195	0.718
TaMAPKK15	TaMEKK1	4565.A0A3B6HW51	4565.A0A3B6B3I4	0	0	0.159	0.726	0.05	0.652	0.573	0.136	0.878
TaMAPKK15	TaMEKK20	4565.A0A3B6HW51	4565.A0A3B6B6T5	0	0	0	0.699	0.05	0.652	0.431	0.136	0.815
TaMAPKK15	TaRaf105	4565.A0A3B6HW51	4565.A0A3B6C472	0	0	0.235	0	0	0.172	0.176	0	0.432
TaMAPKK15	TaMAPKKK5	4565.A0A3B6HW51	4565.A0A3B6C4T6	0	0	0.111	0.682	0	0.176	0.579	0.195	0.718
TaMAPKK15	TaMEKK18	4565.A0A3B6HW51	4565.A0A3B6CEA6	0	0	0	0.699	0.05	0.652	0.431	0.136	0.815
TaMAPKK15	TaMAPKKK6	4565.A0A3B6HW51	4565.A0A3B6DEP0	0	0	0.112	0.683	0	0.176	0.579	0.195	0.719
TaMAPKK15	TaRaf60	4565.A0A3B6HW51	4565.A0A3B6EHW0	0	0	0.204	0.592	0	0.172	0.176	0	0.409
TaMAPKK15	TaMEKK8	4565.A0A3B6HW51	4565.A0A3B6FNU8	0	0	0.172	0.703	0.05	0.652	0.431	0.136	0.84
TaMAPKK15	TaRaf73	4565.A0A3B6HW51	4565.A0A3B6GRN8	0	0	0.229	0	0	0.172	0.176	0	0.428

TaMAPKK 15	TaRaf5	4565.A0A3 B6HW51	4565.A0A3 B6GYQ1	0	0	0.204	0.596	0	0.172	0.176	0	0.409
TaMAPKK 15	TaRaf46	4565.A0A3 B6HW51	4565.A0A3 B6MW69	0	0	0.198	0.615	0	0.172	0.176	0	0.405
TaMAPKK 15	TaRaf62	4565.A0A3 B6HW51	4565.A9RA A9	0	0	0.207	0.608	0	0.172	0.176	0	0.412
TaMAPKK 15	TaRaf42	4565.A0A3 B6HW51	4565.A0A3 B6U554	0	0	0.211	0	0	0.172	0.176	0	0.414
TaMAPKK 15	TaRaf41	4565.A0A3 B6HW51	4565.A0A3 B6KLD7	0	0	0.214	0.593	0	0.172	0.176	0	0.417
TaMAPKK 15	TaRaf56	4565.A0A3 B6HW51	4565.A0A3 B6LPY3	0	0	0.218	0.587	0	0.172	0.176	0	0.42
TaMAPKK 15	TaRaf7	4565.A0A3 B6HW51	4565.A0A3 B6TBH5	0	0	0.22	0.591	0	0.172	0.176	0	0.421
TaMAPKK 15	TaRaf72	4565.A0A3 B6HW51	4565.A0A3 B6MXW5	0	0	0.233	0	0	0.172	0.176	0	0.431
TaMAPKK 15	TaRaf50	4565.A0A3 B6HW51	4565.A0A3 B6RET0	0	0	0.268	0	0	0.172	0.176	0	0.456
TaMAPKK 15	TaRaf43	4565.A0A3 B6HW51	4565.A0A3 B6KNC6	0	0	0.278	0	0	0.172	0.176	0	0.464
TaMAPKK 15	TaRaf91	4565.A0A3 B6HW51	4565.A0A3 B6PMI5	0	0	0.139	0.597	0	0.172	0.489	0	0.604
TaMAPKK 15	TaMEKK4-1	4565.A0A3 B6HW51	4565.A0A3 B6PNI6	0	0	0.173	0.702	0.05	0.652	0.573	0.136	0.88
TaMAPKK 15	TaMEKK4	4565.A0A3 B6HW51	4565.A0A3 B6NRN9	0	0	0.174	0.699	0.05	0.652	0.573	0.136	0.88
TaMAPKK 15	TaMEKK24	4565.A0A3 B6HW51	4565.A0A3 B6LLV5	0	0	0.159	0.742	0.05	0.652	0.614	0.136	0.89
TaMAPKK 15	TaMEKK29	4565.A0A3 B6HW51	4565.A0A3 B6MSP6	0	0	0.159	0.741	0.05	0.652	0.614	0.136	0.89
TaMAPKK 15	TaMEKK17	4565.A0A3 B6HW51	4565.A0A3 B6KFL8	0	0	0.159	0.741	0.05	0.652	0.614	0.136	0.89
TaMAPKK 16	TaMEKK7	4565.A0A3 B6HZP7	4565.A0A0 77RUI2	0	0	0.183	0.687	0.05	0.652	0.431	0.136	0.843
TaMAPKK 16	TaRaf18	4565.A0A3 B6HZP7	4565.A0A0 77RY41	0	0	0.199	0.627	0	0.172	0.176	0	0.406

TaMAPKK16	TaMEKK9	4565.A0A3B6HZP7	4565.A0A077S2G5	0	0	0.197	0.641	0.05	0.652	0.431	0.136	0.845
TaMAPKK16	TaRaf95	4565.A0A3B6HZP7	4565.A0A1D5UN01	0	0	0.191	0.609	0	0.172	0.176	0	0.4
TaMAPKK16	TaRaf88	4565.A0A3B6HZP7	4565.A0A3B5Z5X1	0	0	0.205	0.612	0	0.172	0.176	0	0.41
TaMAPKK16	TaRaf30	4565.A0A3B6HZP7	4565.A0A3B6A1Z4	0	0	0.203	0.612	0	0.172	0.176	0	0.409
TaMAPKK16	TaRaf102	4565.A0A3B6HZP7	4565.A0A3B6AQT7	0	0	0.232	0	0	0.172	0.176	0	0.43
TaMAPKK16	TaRaf19	4565.A0A3B6HZP7	4565.A0A3B6AWY0	0	0	0.269	0	0	0.172	0.176	0	0.457
TaMAPKK16	TaMAPKKK4	4565.A0A3B6HZP7	4565.A0A3B6AY64	0	0	0.114	0.682	0	0.176	0.579	0.195	0.719
TaMAPKK16	TaMEKK1	4565.A0A3B6HZP7	4565.A0A3B6B3I4	0	0	0.155	0.732	0.05	0.652	0.573	0.136	0.878
TaMAPKK16	TaMEKK20	4565.A0A3B6HZP7	4565.A0A3B6B6T5	0	0	0	0.701	0.05	0.652	0.431	0.136	0.815
TaMAPKK16	TaRaf105	4565.A0A3B6HZP7	4565.A0A3B6C472	0	0	0.251	0	0	0.172	0.176	0	0.444
TaMAPKK16	TaMAPKKK5	4565.A0A3B6HZP7	4565.A0A3B6C4T6	0	0	0.113	0.683	0	0.176	0.579	0.195	0.719
TaMAPKK16	TaMEKK18	4565.A0A3B6HZP7	4565.A0A3B6CEA6	0	0	0	0.702	0.05	0.652	0.431	0.136	0.815
TaMAPKK16	TaMAPKKK6	4565.A0A3B6HZP7	4565.A0A3B6DEP0	0	0	0.114	0.683	0	0.176	0.579	0.195	0.719
TaMAPKK16	TaRaf60	4565.A0A3B6HZP7	4565.A0A3B6EHW0	0	0	0.203	0.587	0	0.172	0.176	0	0.409
TaMAPKK16	TaMEKK8	4565.A0A3B6HZP7	4565.A0A3B6FNU8	0	0	0.179	0.688	0.05	0.652	0.431	0.136	0.842
TaMAPKK16	TaRaf5	4565.A0A3B6HZP7	4565.A0A3B6GYQ1	0	0	0.204	0.594	0	0.172	0.176	0	0.409
TaMAPKK16	TaRaf46	4565.A0A3B6HZP7	4565.A0A3B6MW69	0	0	0.201	0.612	0	0.172	0.176	0	0.407
TaMAPKK16	TaRaf83	4565.A0A3B6HZP7	4565.A0A3B6MWW7	0	0	0.208	0	0	0.172	0.176	0	0.412

TaMAPKK 16	TaRaf44	4565.A0A3 B6HZP7	4565.A0A3 B6KNC2	0	0	0.21	0	0	0.172	0.176	0	0.413
TaMAPKK 16	TaRaf62	4565.A0A3 B6HZP7	4565.A9RA A9	0	0	0.21	0.597	0	0.172	0.176	0	0.414
TaMAPKK 16	TaRaf41	4565.A0A3 B6HZP7	4565.A0A3 B6KLD7	0	0	0.217	0.589	0	0.172	0.176	0	0.419
TaMAPKK 16	TaRaf56	4565.A0A3 B6HZP7	4565.A0A3 B6LPY3	0	0	0.217	0.589	0	0.172	0.176	0	0.419
TaMAPKK 16	TaRaf7	4565.A0A3 B6HZP7	4565.A0A3 B6TBH5	0	0	0.218	0.592	0	0.172	0.176	0	0.42
TaMAPKK 16	TaRaf42	4565.A0A3 B6HZP7	4565.A0A3 B6U554	0	0	0.223	0	0	0.172	0.176	0	0.423
TaMAPKK 16	TaRaf45	4565.A0A3 B6HZP7	4565.A0A3 B6KN82	0	0	0.238	0	0	0.172	0.176	0	0.434
TaMAPKK 16	TaRaf50	4565.A0A3 B6HZP7	4565.A0A3 B6RET0	0	0	0.286	0	0	0.172	0.176	0	0.47
TaMAPKK 16	TaRaf43	4565.A0A3 B6HZP7	4565.A0A3 B6KNC6	0	0	0.291	0	0	0.172	0.176	0	0.474
TaMAPKK 16	TaRaf91	4565.A0A3 B6HZP7	4565.A0A3 B6PMI5	0	0	0.129	0.602	0	0.172	0.489	0	0.599
TaMAPKK 16	TaMEKK4-1	4565.A0A3 B6HZP7	4565.A0A3 B6PNI6	0	0	0.166	0.709	0.05	0.652	0.573	0.136	0.879
TaMAPKK 16	TaMEKK4	4565.A0A3 B6HZP7	4565.A0A3 B6NRN9	0	0	0.167	0.705	0.05	0.652	0.573	0.136	0.879
TaMAPKK 16	TaMEKK24	4565.A0A3 B6HZP7	4565.A0A3 B6LLV5	0	0	0.158	0.741	0.05	0.652	0.614	0.136	0.89
TaMAPKK 16	TaMEKK29	4565.A0A3 B6HZP7	4565.A0A3 B6MSP6	0	0	0.158	0.742	0.05	0.652	0.614	0.136	0.89
TaMAPKK 16	TaMEKK17	4565.A0A3 B6HZP7	4565.A0A3 B6KFL8	0	0	0.158	0.741	0.05	0.652	0.614	0.136	0.89
TaMAPKK 17	TaMEKK7	4565.A0A3 B6I0M7	4565.A0A0 77RUI2	0	0	0.178	0.702	0.05	0.652	0.431	0.136	0.842
TaMAPKK 17	TaRaf18	4565.A0A3 B6I0M7	4565.A0A0 77RY41	0	0	0.201	0.633	0	0.172	0.176	0	0.407
TaMAPKK 17	TaMEKK9	4565.A0A3 B6I0M7	4565.A0A0 77S2G5	0	0	0.195	0.65	0.05	0.652	0.431	0.136	0.845

TaMAPKK17	TaRaf95	4565.A0A3B6I0M7	4565.A0A1D5UN01	0	0	0.191	0.606	0	0.172	0.176	0	0.4
TaMAPKK17	TaRaf88	4565.A0A3B6I0M7	4565.A0A3B5Z5X1	0	0	0.206	0.628	0	0.172	0.176	0	0.411
TaMAPKK17	TaRaf30	4565.A0A3B6I0M7	4565.A0A3B6A1Z4	0	0	0.203	0.629	0	0.172	0.176	0	0.409
TaMAPKK17	TaMAPKKK4	4565.A0A3B6I0M7	4565.A0A3B6AY64	0	0	0.108	0.692	0	0.176	0.579	0.195	0.717
TaMAPKK17	TaMEKK1	4565.A0A3B6I0M7	4565.A0A3B6B3I4	0	0	0.148	0.742	0.05	0.652	0.573	0.136	0.877
TaMAPKK17	TaMEKK20	4565.A0A3B6I0M7	4565.A0A3B6B6T5	0	0	0	0.691	0.05	0.652	0.431	0.136	0.815
TaMAPKK17	TaMAPKKK5	4565.A0A3B6I0M7	4565.A0A3B6C4T6	0	0	0.108	0.691	0	0.176	0.579	0.195	0.717
TaMAPKK17	TaMEKK18	4565.A0A3B6I0M7	4565.A0A3B6CEA6	0	0	0	0.693	0.05	0.652	0.431	0.136	0.815
TaMAPKK17	TaMAPKKK6	4565.A0A3B6I0M7	4565.A0A3B6DEP0	0	0	0.109	0.692	0	0.176	0.579	0.195	0.718
TaMAPKK17	TaRaf59	4565.A0A3B6I0M7	4565.A0A3B6E9E2	0	0	0.192	0.609	0	0.172	0.176	0	0.4
TaMAPKK17	TaRaf60	4565.A0A3B6I0M7	4565.A0A3B6EHW0	0	0	0.212	0.589	0	0.172	0.176	0	0.415
TaMAPKK17	TaMEKK8	4565.A0A3B6I0M7	4565.A0A3B6FNU8	0	0	0.178	0.697	0.05	0.652	0.431	0.136	0.842
TaMAPKK17	TaRaf5	4565.A0A3B6I0M7	4565.A0A3B6GYQ1	0	0	0.212	0.593	0	0.172	0.176	0	0.415
TaMAPKK17	TaRaf21	4565.A0A3B6I0M7	4565.A0A3B6TVC5	0	0	0.192	0.592	0	0.172	0.176	0	0.401
TaMAPKK17	TaRaf1	4565.A0A3B6I0M7	4565.A0A3B6NP57	0	0	0.194	0.589	0	0.172	0.176	0	0.402
TaMAPKK17	TaRaf62	4565.A0A3B6I0M7	4565.A9RAA9	0	0	0.207	0.62	0	0.172	0.176	0	0.411
TaMAPKK17	TaRaf7	4565.A0A3B6I0M7	4565.A0A3B6TBH5	0	0	0.207	0.602	0	0.172	0.176	0	0.412
TaMAPKK17	TaRaf41	4565.A0A3B6I0M7	4565.A0A3B6KLD7	0	0	0.209	0.607	0	0.172	0.176	0	0.413

TaMAPKK17	TaRaf56	4565.A0A3B6I0M7	4565.A0A3B6LPY3	0	0	0.216	0.592	0	0.172	0.176	0	0.419
TaMAPKK17	TaRaf91	4565.A0A3B6I0M7	4565.A0A3B6PMI5	0	0	0.151	0.597	0	0.172	0.489	0	0.609
TaMAPKK17	TaMEKK4-1	4565.A0A3B6I0M7	4565.A0A3B6PNI6	0	0	0.161	0.716	0.05	0.652	0.573	0.136	0.879
TaMAPKK17	TaMEKK4	4565.A0A3B6I0M7	4565.A0A3B6NRN9	0	0	0.161	0.714	0.05	0.652	0.573	0.136	0.879
TaMAPKK17	TaMEKK24	4565.A0A3B6I0M7	4565.A0A3B6LLV5	0	0	0.162	0.732	0.05	0.652	0.614	0.136	0.89
TaMAPKK17	TaMEKK29	4565.A0A3B6I0M7	4565.A0A3B6MSP6	0	0	0.162	0.732	0.05	0.652	0.614	0.136	0.89
TaMAPKK17	TaMEKK17	4565.A0A3B6I0M7	4565.A0A3B6KFL8	0	0	0.162	0.732	0.05	0.652	0.614	0.136	0.89
TaMAPKK18	TaMEKK7	4565.A0A3B6LJ27	4565.A0A077RUI2	0	0	0.14	0.613	0.05	0.652	0.823	0.136	0.948
TaMAPKK18	TaMEKK9	4565.A0A3B6LJ27	4565.A0A077S2G5	0	0	0.139	0.633	0.05	0.652	0.791	0.136	0.939
TaMAPKK18	TaMAPKKK4	4565.A0A3B6LJ27	4565.A0A3B6AY64	0	0	0	0.673	0	0.176	0.579	0.195	0.696
TaMAPKK18	TaMEKK1	4565.A0A3B6LJ27	4565.A0A3B6B3I4	0	0	0.1	0.651	0.05	0.652	0.431	0.136	0.827
TaMAPKK18	TaMEKK20	4565.A0A3B6LJ27	4565.A0A3B6B6T5	0	0	0	0.634	0.05	0.652	0.431	0.136	0.815
TaMAPKK18	TaMAPKKK5	4565.A0A3B6LJ27	4565.A0A3B6C4T6	0	0	0	0.673	0	0.176	0.579	0.195	0.696
TaMAPKK18	TaMEKK18	4565.A0A3B6LJ27	4565.A0A3B6CEA6	0	0	0	0.633	0.05	0.652	0.431	0.136	0.815
TaMAPKK18	TaMAPKKK6	4565.A0A3B6LJ27	4565.A0A3B6DEP0	0	0	0	0.673	0	0.176	0.579	0.195	0.696
TaMAPKK18	TaMEKK8	4565.A0A3B6LJ27	4565.A0A3B6FNU8	0	0	0.139	0.602	0.05	0.652	0.791	0.136	0.939
TaMAPKK18	TaMEKK17	4565.A0A3B6LJ27	4565.A0A3B6KFL8	0	0	0.157	0.644	0.05	0.652	0.431	0.136	0.838
TaMAPKK18	TaRaf52	4565.A0A3B6LJ27	4565.A0A3B6LRR0	0	0	0.196	0.603	0	0.172	0.176	0	0.403

TaMAPKK18	TaMEKK4	4565.A0A3B6LJ27	4565.A0A3B6NRN9	0	0	0.107	0.645	0.05	0.652	0.431	0.136	0.828
TaMAPKK18	TaMEKK4-1	4565.A0A3B6LJ27	4565.A0A3B6PNI6	0	0	0.109	0.645	0.05	0.652	0.431	0.136	0.828
TaMAPKK18	TaMEKK24	4565.A0A3B6LJ27	4565.A0A3B6LLV5	0	0	0.157	0.644	0.05	0.652	0.431	0.136	0.838
TaMAPKK18	TaMEKK29	4565.A0A3B6LJ27	4565.A0A3B6MSP6	0	0	0.158	0.643	0.05	0.652	0.431	0.136	0.838
TaMAPKK2	TaMEKK7	4565.A0A3B6LYW0	4565.A0A077RUI2	0	0	0.154	0.622	0.05	0.652	0.927	0.136	0.979
TaMAPKK2	TaMEKK9	4565.A0A3B6LYW0	4565.A0A077S2G5	0	0	0.149	0.653	0.05	0.652	0.791	0.136	0.939
TaMAPKK2	TaMAPKKK4	4565.A0A3B6LYW0	4565.A0A3B6AY64	0	0	0	0.657	0	0.176	0.579	0.195	0.696
TaMAPKK2	TaMEKK1	4565.A0A3B6LYW0	4565.A0A3B6B3I4	0	0	0.109	0.639	0.05	0.652	0.431	0.136	0.828
TaMAPKK2	TaMEKK20	4565.A0A3B6LYW0	4565.A0A3B6B6T5	0	0	0	0.637	0.05	0.652	0.431	0.136	0.815
TaMAPKK2	TaMAPKKK5	4565.A0A3B6LYW0	4565.A0A3B6C4T6	0	0	0	0.657	0	0.176	0.579	0.195	0.696
TaMAPKK2	TaMEKK18	4565.A0A3B6LYW0	4565.A0A3B6CEA6	0	0	0	0.636	0.05	0.652	0.431	0.136	0.815
TaMAPKK2	TaMAPKKK6	4565.A0A3B6LYW0	4565.A0A3B6DEP0	0	0	0	0.657	0	0.176	0.579	0.195	0.696
TaMAPKK2	TaMEKK8	4565.A0A3B6LYW0	4565.A0A3B6FNU8	0	0	0.149	0.628	0.05	0.652	0.946	0.136	0.984
TaMAPKK2	TaMEKK17	4565.A0A3B6LYW0	4565.A0A3B6KFL8	0	0	0.166	0.648	0.05	0.652	0.431	0.136	0.839
TaMAPKK2	TaMEKK24	4565.A0A3B6LYW0	4565.A0A3B6LLV5	0	0	0.167	0.648	0.05	0.652	0.431	0.136	0.84
TaMAPKK2	TaRaf52	4565.A0A3B6LYW0	4565.A0A3B6LRR0	0	0	0.194	0.598	0	0.172	0.176	0	0.402
TaMAPKK2	TaMEKK4	4565.A0A3B6LYW0	4565.A0A3B6NRN9	0	0	0.114	0.632	0.05	0.652	0.431	0.136	0.829
TaMAPKK2	TaMEKK4-1	4565.A0A3B6LYW0	4565.A0A3B6PNI6	0	0	0.116	0.634	0.05	0.652	0.431	0.136	0.83

TaMAPKK2	TaMEKK29	4565.A0A3B6LYW0	4565.A0A3B6MSP6	0	0	0.166	0.648	0.05	0.652	0.431	0.136	0.839
TaMAPKK3	TaMEKK7	4565.A0A3B6MNP8	4565.A0A077RUI2	0	0	0.124	0.615	0.05	0.652	0.823	0.136	0.947
TaMAPKK3	TaMEKK9	4565.A0A3B6MNP8	4565.A0A077S2G5	0	0	0.124	0.627	0.05	0.652	0.791	0.136	0.938
TaMAPKK3	TaMAPKKK4	4565.A0A3B6MNP8	4565.A0A3B6AY64	0	0	0	0.653	0	0.176	0.579	0.195	0.696
TaMAPKK3	TaMEKK1	4565.A0A3B6MNP8	4565.A0A3B6B3I4	0	0	0	0.632	0.05	0.652	0.431	0.136	0.815
TaMAPKK3	TaMEKK20	4565.A0A3B6MNP8	4565.A0A3B6B6T5	0	0	0	0.62	0.05	0.652	0.431	0.136	0.815
TaMAPKK3	TaMAPKKK5	4565.A0A3B6MNP8	4565.A0A3B6C4T6	0	0	0	0.652	0	0.176	0.579	0.195	0.696
TaMAPKK3	TaMEKK18	4565.A0A3B6MNP8	4565.A0A3B6CEA6	0	0	0	0.62	0.05	0.652	0.431	0.136	0.815
TaMAPKK3	TaMAPKKK6	4565.A0A3B6MNP8	4565.A0A3B6DEP0	0	0	0	0.652	0	0.176	0.579	0.195	0.696
TaMAPKK3	TaMEKK8	4565.A0A3B6MNP8	4565.A0A3B6FNU8	0	0	0.12	0.602	0.05	0.652	0.791	0.136	0.937
TaMAPKK3	TaMEKK17	4565.A0A3B6MNP8	4565.A0A3B6KFL8	0	0	0.155	0.627	0.05	0.652	0.431	0.136	0.837
TaMAPKK3	TaMEKK24	4565.A0A3B6MNP8	4565.A0A3B6LLV5	0	0	0.155	0.627	0.05	0.652	0.431	0.136	0.837
TaMAPKK3	TaRaf52	4565.A0A3B6MNP8	4565.A0A3B6LRR0	0	0	0.2	0.59	0	0.172	0.176	0	0.406
TaMAPKK3	TaMEKK4-1	4565.A0A3B6MNP8	4565.A0A3B6PNI6	0	0	0.107	0.628	0.05	0.652	0.431	0.136	0.828
TaMAPKK3	TaMEKK4	4565.A0A3B6MNP8	4565.A0A3B6NRN9	0	0	0.105	0.627	0.05	0.652	0.431	0.136	0.828
TaMAPKK3	TaMEKK29	4565.A0A3B6MNP8	4565.A0A3B6MSP6	0	0	0.154	0.627	0.05	0.652	0.431	0.136	0.837
TaMAPKK4	TaMEKK7	4565.A0A3B6KFB5	4565.A0A077RUI2	0	0	0.135	0.689	0.05	0.652	0.823	0.136	0.948
TaMAPKK4	TaMEKK9	4565.A0A3B6KFB5	4565.A0A077S2G5	0	0	0.152	0.656	0.05	0.652	0.791	0.136	0.94

TaMAPKK4	TaMAPKKKK4	4565.A0A3B6KFB5	4565.A0A3B6AY64	0	0	0	0.776	0	0.176	0.579	0.195	0.696
TaMAPKK4	TaMEKK1	4565.A0A3B6KFB5	4565.A0A3B6B3I4	0	0	0.094	0.749	0.05	0.652	0.431	0.136	0.825
TaMAPKK4	TaMEKK20	4565.A0A3B6KFB5	4565.A0A3B6B6T5	0	0	0	0.724	0.05	0.652	0.431	0.136	0.815
TaMAPKK4	TaMAPKKK5	4565.A0A3B6KFB5	4565.A0A3B6C4T6	0	0	0	0.776	0	0.176	0.579	0.195	0.696
TaMAPKK4	TaMEKK18	4565.A0A3B6KFB5	4565.A0A3B6CEA6	0	0	0	0.723	0.05	0.652	0.431	0.136	0.815
TaMAPKK4	TaMAPKKK6	4565.A0A3B6KFB5	4565.A0A3B6DEP0	0	0	0	0.776	0	0.176	0.579	0.195	0.696
TaMAPKK4	TaMEKK8	4565.A0A3B6KFB5	4565.A0A3B6FNU8	0	0	0.14	0.66	0.05	0.652	0.791	0.136	0.939
TaMAPKK4	TaMEKK4-1	4565.A0A3B6KFB5	4565.A0A3B6PNI6	0	0	0.102	0.741	0.05	0.652	0.431	0.136	0.827
TaMAPKK4	TaMEKK4	4565.A0A3B6KFB5	4565.A0A3B6NRN9	0	0	0.1	0.74	0.05	0.652	0.431	0.136	0.827
TaMAPKK4	TaMEKK24	4565.A0A3B6KFB5	4565.A0A3B6LLV5	0	0	0.138	0.731	0.05	0.652	0.431	0.136	0.834
TaMAPKK4	TaMEKK29	4565.A0A3B6KFB5	4565.A0A3B6MSP6	0	0	0.138	0.73	0.05	0.652	0.431	0.136	0.834
TaMAPKK4	TaMEKK17	4565.A0A3B6KFB5	4565.A0A3B6KFL8	0	0	0.138	0.73	0.05	0.652	0.431	0.136	0.834
TaMAPKK5	TaMEKK7	4565.A0A3B6IMW7	4565.A0A077RUI2	0	0	0.181	0.689	0.05	0.652	0.431	0.136	0.842
TaMAPKK5	TaRaf18	4565.A0A3B6IMW7	4565.A0A077RY41	0	0	0.195	0.62	0	0.172	0.176	0	0.403
TaMAPKK5	TaMEKK9	4565.A0A3B6IMW7	4565.A0A077S2G5	0	0	0.192	0.652	0.05	0.652	0.431	0.136	0.844
TaMAPKK5	TaRaf95	4565.A0A3B6IMW7	4565.A0A1D5UN01	0	0	0.192	0.592	0	0.172	0.176	0	0.401
TaMAPKK5	TaRaf88	4565.A0A3B6IMW7	4565.A0A3B5Z5X1	0	0	0.201	0.607	0	0.172	0.176	0	0.407
TaMAPKK5	TaRaf30	4565.A0A3B6IMW7	4565.A0A3B6A1Z4	0	0	0.199	0.609	0	0.172	0.176	0	0.406

TaMAPKK5	TaRaf102	4565.A0A3B6IMW7	4565.A0A3B6AQT7	0	0	0.212	0	0	0.172	0.176	0	0.415
TaMAPKK5	TaRaf19	4565.A0A3B6IMW7	4565.A0A3B6AWY0	0	0	0.255	0	0	0.172	0.176	0	0.447
TaMAPKK5	TaMAPKKK4	4565.A0A3B6IMW7	4565.A0A3B6AY64	0	0	0.112	0.691	0	0.176	0.579	0.195	0.718
TaMAPKK5	TaMEKK1	4565.A0A3B6IMW7	4565.A0A3B6B3I4	0	0	0.16	0.72	0.05	0.652	0.573	0.136	0.878
TaMAPKK5	TaMEKK20	4565.A0A3B6IMW7	4565.A0A3B6B6T5	0	0	0	0.687	0.05	0.652	0.431	0.136	0.815
TaMAPKK5	TaRaf105	4565.A0A3B6IMW7	4565.A0A3B6C472	0	0	0.24	0	0	0.172	0.176	0	0.436
TaMAPKK5	TaMAPKKK5	4565.A0A3B6IMW7	4565.A0A3B6C4T6	0	0	0.111	0.691	0	0.176	0.579	0.195	0.718
TaMAPKK5	TaMEKK18	4565.A0A3B6IMW7	4565.A0A3B6CEA6	0	0	0	0.688	0.05	0.652	0.431	0.136	0.815
TaMAPKK5	TaMAPKKK6	4565.A0A3B6IMW7	4565.A0A3B6DEP0	0	0	0.113	0.692	0	0.176	0.579	0.195	0.719
TaMAPKK5	TaRaf60	4565.A0A3B6IMW7	4565.A0A3B6EHW0	0	0	0.201	0.588	0	0.172	0.176	0	0.407
TaMAPKK5	TaMEKK8	4565.A0A3B6IMW7	4565.A0A3B6FNU8	0	0	0.18	0.679	0.05	0.652	0.431	0.136	0.842
TaMAPKK5	TaRaf73	4565.A0A3B6IMW7	4565.A0A3B6GRN8	0	0	0.227	0	0	0.172	0.176	0	0.426
TaMAPKK5	TaRaf5	4565.A0A3B6IMW7	4565.A0A3B6GYQ1	0	0	0.201	0.594	0	0.172	0.176	0	0.407
TaMAPKK5	TaRaf46	4565.A0A3B6IMW7	4565.A0A3B6MW69	0	0	0.196	0.614	0	0.172	0.176	0	0.404
TaMAPKK5	TaRaf62	4565.A0A3B6IMW7	4565.A9RAA9	0	0	0.207	0.598	0	0.172	0.176	0	0.411
TaMAPKK5	TaRaf41	4565.A0A3B6IMW7	4565.A0A3B6KLD7	0	0	0.209	0.588	0	0.172	0.176	0	0.413
TaMAPKK5	TaRaf56	4565.A0A3B6IMW7	4565.A0A3B6LPY3	0	0	0.211	0.583	0	0.172	0.176	0	0.414
TaMAPKK5	TaRaf7	4565.A0A3B6IMW7	4565.A0A3B6TBH5	0	0	0.216	0.592	0	0.172	0.176	0	0.419

TaMAPKK5	TaRaf42	4565.A0A3B6IMW7	4565.A0A3B6U554	0	0	0.217	0	0	0.172	0.176	0	0.419
TaMAPKK5	TaRaf50	4565.A0A3B6IMW7	4565.A0A3B6RET0	0	0	0.261	0	0	0.172	0.176	0	0.451
TaMAPKK5	TaRaf43	4565.A0A3B6IMW7	4565.A0A3B6KNC6	0	0	0.277	0	0	0.172	0.176	0	0.463
TaMAPKK5	TaRaf91	4565.A0A3B6IMW7	4565.A0A3B6PMI5	0	0	0.13	0.595	0	0.172	0.489	0	0.599
TaMAPKK5	TaMEKK4-1	4565.A0A3B6IMW7	4565.A0A3B6PNI6	0	0	0.172	0.698	0.05	0.652	0.573	0.136	0.88
TaMAPKK5	TaMEKK4	4565.A0A3B6IMW7	4565.A0A3B6NRN9	0	0	0.173	0.695	0.05	0.652	0.573	0.136	0.88
TaMAPKK5	TaMEKK17	4565.A0A3B6IMW7	4565.A0A3B6KFL8	0	0	0.164	0.731	0.05	0.652	0.614	0.136	0.891
TaMAPKK5	TaMEKK24	4565.A0A3B6IMW7	4565.A0A3B6LLV5	0	0	0.164	0.731	0.05	0.652	0.614	0.136	0.891
TaMAPKK5	TaMEKK29	4565.A0A3B6IMW7	4565.A0A3B6MSP6	0	0	0.164	0.731	0.05	0.652	0.614	0.136	0.891
TaMAPKK6	TaMEKK7	4565.A0A3B6ILF0	4565.A0A077RUI2	0	0	0.181	0.697	0.05	0.652	0.431	0.136	0.842
TaMAPKK6	TaRaf18	4565.A0A3B6ILF0	4565.A0A077RY41	0	0	0.202	0.628	0	0.172	0.176	0	0.408
TaMAPKK6	TaMEKK9	4565.A0A3B6ILF0	4565.A0A077S2G5	0	0	0.196	0.649	0.05	0.652	0.431	0.136	0.845
TaMAPKK6	TaRaf95	4565.A0A3B6ILF0	4565.A0A1D5UN01	0	0	0.192	0.6	0	0.172	0.176	0	0.4
TaMAPKK6	TaRaf88	4565.A0A3B6ILF0	4565.A0A3B5Z5X1	0	0	0.205	0.627	0	0.172	0.176	0	0.41
TaMAPKK6	TaRaf30	4565.A0A3B6ILF0	4565.A0A3B6A1Z4	0	0	0.206	0.622	0	0.172	0.176	0	0.411
TaMAPKK6	TaMAPKKK4	4565.A0A3B6ILF0	4565.A0A3B6AY64	0	0	0.108	0.684	0	0.176	0.579	0.195	0.717
TaMAPKK6	TaMEKK1	4565.A0A3B6ILF0	4565.A0A3B6B3I4	0	0	0.151	0.735	0.05	0.652	0.573	0.136	0.877
TaMAPKK6	TaMEKK20	4565.A0A3B6ILF0	4565.A0A3B6B6T5	0	0	0	0.687	0.05	0.652	0.431	0.136	0.815

TaMAPKK6	TaMAPKKKK5	4565.A0A3B6ILF0	4565.A0A3B6C4T6	0	0	0.108	0.683	0	0.176	0.579	0.195	0.717
TaMAPKK6	TaMEKK18	4565.A0A3B6ILF0	4565.A0A3B6CEA6	0	0	0	0.684	0.05	0.652	0.431	0.136	0.815
TaMAPKK6	TaMAPKKKK6	4565.A0A3B6ILF0	4565.A0A3B6DEP0	0	0	0.109	0.684	0	0.176	0.579	0.195	0.717
TaMAPKK6	TaRaf60	4565.A0A3B6ILF0	4565.A0A3B6EHW0	0	0	0.209	0.589	0	0.172	0.176	0	0.413
TaMAPKK6	TaMEKK8	4565.A0A3B6ILF0	4565.A0A3B6FNU8	0	0	0.177	0.698	0.05	0.652	0.431	0.136	0.841
TaMAPKK6	TaRaf5	4565.A0A3B6ILF0	4565.A0A3B6GYQ1	0	0	0.21	0.593	0	0.172	0.176	0	0.414
TaMAPKK6	TaRaf1	4565.A0A3B6ILF0	4565.A0A3B6NP57	0	0	0.194	0.585	0	0.172	0.176	0	0.402
TaMAPKK6	TaRaf46	4565.A0A3B6ILF0	4565.A0A3B6MW69	0	0	0.194	0.642	0	0.172	0.176	0	0.402
TaMAPKK6	TaRaf41	4565.A0A3B6ILF0	4565.A0A3B6KLD7	0	0	0.208	0.607	0	0.172	0.176	0	0.413
TaMAPKK6	TaRaf62	4565.A0A3B6ILF0	4565.A9RAA9	0	0	0.209	0.609	0	0.172	0.176	0	0.413
TaMAPKK6	TaRaf7	4565.A0A3B6ILF0	4565.A0A3B6TBH5	0	0	0.212	0.596	0	0.172	0.176	0	0.415
TaMAPKK6	TaRaf56	4565.A0A3B6ILF0	4565.A0A3B6LPY3	0	0	0.214	0.591	0	0.172	0.176	0	0.416
TaMAPKK6	TaRaf91	4565.A0A3B6ILF0	4565.A0A3B6PMI5	0	0	0.147	0.596	0	0.172	0.489	0	0.607
TaMAPKK6	TaMEKK4	4565.A0A3B6ILF0	4565.A0A3B6NRN9	0	0	0.163	0.71	0.05	0.652	0.573	0.136	0.879
TaMAPKK6	TaMEKK4-1	4565.A0A3B6ILF0	4565.A0A3B6PNI6	0	0	0.163	0.711	0.05	0.652	0.573	0.136	0.879
TaMAPKK6	TaMEKK24	4565.A0A3B6ILF0	4565.A0A3B6LLV5	0	0	0.162	0.733	0.05	0.652	0.614	0.136	0.89
TaMAPKK6	TaMEKK29	4565.A0A3B6ILF0	4565.A0A3B6MSP6	0	0	0.162	0.733	0.05	0.652	0.614	0.136	0.89
TaMAPKK6	TaMEKK17	4565.A0A3B6ILF0	4565.A0A3B6KFL8	0	0	0.161	0.734	0.05	0.652	0.614	0.136	0.89

TaMAPKK7	TaMEKK7	4565.A0A3B6INV0	4565.A0A077RUI2	0	0	0.18	0.699	0.05	0.652	0.431	0.136	0.842
TaMAPKK7	TaRaf18	4565.A0A3B6INV0	4565.A0A077RY41	0	0	0.202	0.628	0	0.172	0.176	0	0.408
TaMAPKK7	TaMEKK9	4565.A0A3B6INV0	4565.A0A077S2G5	0	0	0.195	0.648	0.05	0.652	0.431	0.136	0.845
TaMAPKK7	TaRaf95	4565.A0A3B6INV0	4565.A0A1D5UN01	0	0	0.191	0.6	0	0.172	0.176	0	0.4
TaMAPKK7	TaRaf88	4565.A0A3B6INV0	4565.A0A3B5Z5X1	0	0	0.206	0.624	0	0.172	0.176	0	0.411
TaMAPKK7	TaRaf30	4565.A0A3B6INV0	4565.A0A3B6A1Z4	0	0	0.204	0.624	0	0.172	0.176	0	0.409
TaMAPKK7	TaMAPKKK4	4565.A0A3B6INV0	4565.A0A3B6AY64	0	0	0.11	0.689	0	0.176	0.579	0.195	0.718
TaMAPKK7	TaMEKK1	4565.A0A3B6INV0	4565.A0A3B6B3I4	0	0	0.148	0.741	0.05	0.652	0.573	0.136	0.877
TaMAPKK7	TaMEKK20	4565.A0A3B6INV0	4565.A0A3B6B6T5	0	0	0	0.693	0.05	0.652	0.431	0.136	0.815
TaMAPKK7	TaMAPKKK5	4565.A0A3B6INV0	4565.A0A3B6C4T6	0	0	0.11	0.688	0	0.176	0.579	0.195	0.718
TaMAPKK7	TaMEKK18	4565.A0A3B6INV0	4565.A0A3B6CEA6	0	0	0	0.692	0.05	0.652	0.431	0.136	0.815
TaMAPKK7	TaMAPKKK6	4565.A0A3B6INV0	4565.A0A3B6DEP0	0	0	0.111	0.69	0	0.176	0.579	0.195	0.718
TaMAPKK7	TaRaf59	4565.A0A3B6INV0	4565.A0A3B6E9E2	0	0	0.191	0.608	0	0.172	0.176	0	0.4
TaMAPKK7	TaRaf60	4565.A0A3B6INV0	4565.A0A3B6EHW0	0	0	0.211	0.588	0	0.172	0.176	0	0.415
TaMAPKK7	TaMEKK8	4565.A0A3B6INV0	4565.A0A3B6FNU8	0	0	0.177	0.698	0.05	0.652	0.431	0.136	0.842
TaMAPKK7	TaRaf5	4565.A0A3B6INV0	4565.A0A3B6GYQ1	0	0	0.212	0.591	0	0.172	0.176	0	0.415
TaMAPKK7	TaRaf21	4565.A0A3B6INV0	4565.A0A3B6TVC5	0	0	0.194	0.593	0	0.172	0.176	0	0.402
TaMAPKK7	TaRaf1	4565.A0A3B6INV0	4565.A0A3B6NP57	0	0	0.197	0.589	0	0.172	0.176	0	0.404

TaMAPKK7	TaRaf62	4565.A0A3B6INV0	4565.A9RAA9	0	0	0.206	0.616	0	0.172	0.176	0	0.411
TaMAPKK7	TaRaf7	4565.A0A3B6INV0	4565.A0A3B6TBH5	0	0	0.209	0.599	0	0.172	0.176	0	0.413
TaMAPKK7	TaRaf41	4565.A0A3B6INV0	4565.A0A3B6KLD7	0	0	0.208	0.605	0	0.172	0.176	0	0.413
TaMAPKK7	TaRaf56	4565.A0A3B6INV0	4565.A0A3B6LPY3	0	0	0.214	0.591	0	0.172	0.176	0	0.417
TaMAPKK7	TaRaf91	4565.A0A3B6INV0	4565.A0A3B6PMI5	0	0	0.149	0.596	0	0.172	0.489	0	0.608
TaMAPKK7	TaMEKK4	4565.A0A3B6INV0	4565.A0A3B6NRN9	0	0	0.161	0.715	0.05	0.652	0.573	0.136	0.879
TaMAPKK7	TaMEKK4-1	4565.A0A3B6INV0	4565.A0A3B6PNI6	0	0	0.161	0.716	0.05	0.652	0.573	0.136	0.879
TaMAPKK7	TaMEKK17	4565.A0A3B6INV0	4565.A0A3B6KFL8	0	0	0.163	0.731	0.05	0.652	0.614	0.136	0.89
TaMAPKK7	TaMEKK29	4565.A0A3B6INV0	4565.A0A3B6MSP6	0	0	0.163	0.731	0.05	0.652	0.614	0.136	0.89
TaMAPKK7	TaMEKK24	4565.A0A3B6INV0	4565.A0A3B6LLV5	0	0	0.163	0.731	0.05	0.652	0.614	0.136	0.89
TaMAPKK8	TaMEKK7	4565.A0A3B6IK39	4565.A0A077RUI2	0	0	0.178	0.694	0.05	0.652	0.431	0.136	0.842
TaMAPKK8	TaRaf113	4565.A0A3B6IK39	4565.A0A077RV42	0	0	0.304	0	0	0.172	0.176	0	0.483
TaMAPKK8	TaMEKK9	4565.A0A3B6IK39	4565.A0A077S2G5	0	0	0.196	0.642	0.05	0.652	0.431	0.136	0.845
TaMAPKK8	TaRaf88	4565.A0A3B6IK39	4565.A0A3B5Z5X1	0	0	0.193	0.617	0	0.172	0.176	0	0.401
TaMAPKK8	TaRaf30	4565.A0A3B6IK39	4565.A0A3B6A1Z4	0	0	0.193	0.617	0	0.172	0.176	0	0.401
TaMAPKK8	TaRaf102	4565.A0A3B6IK39	4565.A0A3B6AQT7	0	0	0.224	0	0	0.172	0.176	0	0.424
TaMAPKK8	TaRaf19	4565.A0A3B6IK39	4565.A0A3B6AWY0	0	0	0.265	0	0	0.172	0.176	0	0.454
TaMAPKK8	TaMAPKKKK4	4565.A0A3B6IK39	4565.A0A3B6AY64	0	0	0.109	0.691	0	0.176	0.579	0.195	0.717

TaMAPKK8	TaMEKK1	4565.A0A3B6IK39	4565.A0A3B6B3I4	0	0	0.151	0.735	0.05	0.652	0.573	0.136	0.877
TaMAPKK8	TaMEKK20	4565.A0A3B6IK39	4565.A0A3B6B6T5	0	0	0	0.705	0.05	0.652	0.431	0.136	0.815
TaMAPKK8	TaRaf105	4565.A0A3B6IK39	4565.A0A3B6C472	0	0	0.251	0	0	0.172	0.176	0	0.444
TaMAPKK8	TaMAPKKK5	4565.A0A3B6IK39	4565.A0A3B6C4T6	0	0	0.108	0.69	0	0.176	0.579	0.195	0.717
TaMAPKK8	TaMEKK18	4565.A0A3B6IK39	4565.A0A3B6CEA6	0	0	0	0.706	0.05	0.652	0.431	0.136	0.815
TaMAPKK8	TaMAPKKK6	4565.A0A3B6IK39	4565.A0A3B6DEP0	0	0	0.109	0.691	0	0.176	0.579	0.195	0.718
TaMAPKK8	TaRaf71	4565.A0A3B6IK39	4565.A0A3B6EJJ5	0	0	0.202	0	0	0.172	0.176	0	0.408
TaMAPKK8	TaMEKK8	4565.A0A3B6IK39	4565.A0A3B6FNU8	0	0	0.174	0.694	0.05	0.652	0.431	0.136	0.841
TaMAPKK8	TaRaf46	4565.A0A3B6IK39	4565.A0A3B6MW69	0	0	0.192	0.623	0	0.172	0.176	0	0.4
TaMAPKK8	TaRaf62	4565.A0A3B6IK39	4565.A9RAA9	0	0	0.2	0.597	0	0.172	0.176	0	0.406
TaMAPKK8	TaRaf56	4565.A0A3B6IK39	4565.A0A3B6LPY3	0	0	0.211	0.586	0	0.172	0.176	0	0.415
TaMAPKK8	TaRaf41	4565.A0A3B6IK39	4565.A0A3B6KLD7	0	0	0.212	0.591	0	0.172	0.176	0	0.415
TaMAPKK8	TaRaf42	4565.A0A3B6IK39	4565.A0A3B6U554	0	0	0.213	0	0	0.172	0.176	0	0.416
TaMAPKK8	TaRaf7	4565.A0A3B6IK39	4565.A0A3B6TBH5	0	0	0.216	0.59	0	0.172	0.176	0	0.419
TaMAPKK8	TaRaf45	4565.A0A3B6IK39	4565.A0A3B6KN82	0	0	0.224	0	0	0.172	0.176	0	0.424
TaMAPKK8	TaRaf50	4565.A0A3B6IK39	4565.A0A3B6RET0	0	0	0.27	0	0	0.172	0.176	0	0.458
TaMAPKK8	TaRaf43	4565.A0A3B6IK39	4565.A0A3B6KNC6	0	0	0.281	0	0	0.172	0.176	0	0.466
TaMAPKK8	TaRaf91	4565.A0A3B6IK39	4565.A0A3B6PMI5	0	0	0.125	0.606	0	0.172	0.489	0	0.597

TaMAPKK8	TaMEKK4-1	4565.A0A3B6IK39	4565.A0A3B6PNI6	0	0	0.164	0.709	0.05	0.652	0.573	0.136	0.879
TaMAPKK8	TaMEKK4	4565.A0A3B6IK39	4565.A0A3B6NRN9	0	0	0.165	0.706	0.05	0.652	0.573	0.136	0.879
TaMAPKK8	TaMEKK17	4565.A0A3B6IK39	4565.A0A3B6KFL8	0	0	0.156	0.743	0.05	0.652	0.614	0.136	0.889
TaMAPKK8	TaMEKK29	4565.A0A3B6IK39	4565.A0A3B6MSP6	0	0	0.155	0.744	0.05	0.652	0.614	0.136	0.889
TaMAPKK8	TaMEKK24	4565.A0A3B6IK39	4565.A0A3B6LLV5	0	0	0.156	0.743	0.05	0.652	0.614	0.136	0.89
TaMAPKK9	TaMEKK7	4565.A0A077RVQ4	4565.A0A077RUI2	0	0	0.176	0.704	0.05	0.652	0.431	0.136	0.841
TaMAPKK9	TaRaf46	4565.A0A077RVQ4	4565.A0A3B6MW69	0	0	0.195	0.622	0	0.172	0.176	0	0.403
TaMAPKK9	TaRaf95	4565.A0A077RVQ4	4565.A0A1D5UN01	0	0	0.197	0.602	0	0.172	0.176	0	0.404
TaMAPKK9	TaRaf30	4565.A0A077RVQ4	4565.A0A3B6A1Z4	0	0	0.198	0.619	0	0.172	0.176	0	0.405
TaMAPKK9	TaRaf18	4565.A0A077RVQ4	4565.A0A077RY41	0	0	0.199	0.622	0	0.172	0.176	0	0.406
TaMAPKK9	TaRaf88	4565.A0A077RVQ4	4565.A0A3B5Z5X1	0	0	0.2	0.618	0	0.172	0.176	0	0.407
TaMAPKK9	TaRaf5	4565.A0A077RVQ4	4565.A0A3B6GYQ1	0	0	0.202	0.592	0	0.172	0.176	0	0.408
TaMAPKK9	TaRaf60	4565.A0A077RVQ4	4565.A0A3B6EHW0	0	0	0.205	0.584	0	0.172	0.176	0	0.41
TaMAPKK9	TaRaf62	4565.A0A077RVQ4	4565.A9RAA9	0	0	0.204	0.608	0	0.172	0.176	0	0.41
TaMAPKK9	TaRaf42	4565.A0A077RVQ4	4565.A0A3B6U554	0	0	0.215	0	0	0.172	0.176	0	0.417
TaMAPKK9	TaRaf56	4565.A0A077RVQ4	4565.A0A3B6LPY3	0	0	0.216	0.585	0	0.172	0.176	0	0.418
TaMAPKK9	TaRaf41	4565.A0A077RVQ4	4565.A0A3B6KLD7	0	0	0.215	0.591	0	0.172	0.176	0	0.418
TaMAPKK9	TaRaf7	4565.A0A077RVQ4	4565.A0A3B6TBH5	0	0	0.218	0.594	0	0.172	0.176	0	0.419

TaMAPKK9	TaRaf102	4565.A0A077RVQ4	4565.A0A3B6AQT7	0	0	0.221	0	0	0.172	0.176	0	0.422
TaMAPKK9	TaRaf50	4565.A0A077RVQ4	4565.A0A3B6RET0	0	0	0.274	0	0	0.172	0.176	0	0.461
TaMAPKK9	TaRaf43	4565.A0A077RVQ4	4565.A0A3B6KNC6	0	0	0.285	0	0	0.172	0.176	0	0.469
TaMAPKK9	TaRaf91	4565.A0A077RVQ4	4565.A0A3B6PMI5	0	0	0.133	0.595	0	0.172	0.489	0	0.601
TaMAPKK9	TaMAPKKK4	4565.A0A077RVQ4	4565.A0A3B6AY64	0	0	0.11	0.689	0	0.176	0.579	0.195	0.718
TaMAPKK9	TaMAPKKK5	4565.A0A077RVQ4	4565.A0A3B6C4T6	0	0	0.11	0.689	0	0.176	0.579	0.195	0.718
TaMAPKK9	TaMAPKKK6	4565.A0A077RVQ4	4565.A0A3B6DEP0	0	0	0.111	0.689	0	0.176	0.579	0.195	0.718
TaMAPKK9	TaMEKK18	4565.A0A077RVQ4	4565.A0A3B6CEA6	0	0	0	0.702	0.05	0.652	0.431	0.136	0.815
TaMAPKK9	TaMEKK20	4565.A0A077RVQ4	4565.A0A3B6B6T5	0	0	0	0.702	0.05	0.652	0.431	0.136	0.815
TaMAPKK9	TaMEKK8	4565.A0A077RVQ4	4565.A0A3B6FNU8	0	0	0.174	0.699	0.05	0.652	0.431	0.136	0.841
TaMAPKK9	TaMEKK9	4565.A0A077RVQ4	4565.A0A077S2G5	0	0	0.19	0.658	0.05	0.652	0.431	0.136	0.844
TaMAPKK9	TaMEKK1	4565.A0A077RVQ4	4565.A0A3B6B3I4	0	0	0.156	0.729	0.05	0.652	0.573	0.136	0.878
TaMAPKK9	TaMEKK4	4565.A0A077RVQ4	4565.A0A3B6NRN9	0	0	0.169	0.703	0.05	0.652	0.573	0.136	0.88
TaMAPKK9	TaMEKK4-1	4565.A0A077RVQ4	4565.A0A3B6PNI6	0	0	0.168	0.705	0.05	0.652	0.573	0.136	0.88
TaMAPKK9	TaMEKK29	4565.A0A077RVQ4	4565.A0A3B6MSP6	0	0	0.159	0.738	0.05	0.652	0.614	0.136	0.89
TaMAPKK9	TaMEKK24	4565.A0A077RVQ4	4565.A0A3B6LLV5	0	0	0.159	0.738	0.05	0.652	0.614	0.136	0.89
TaMAPKK9	TaMEKK17	4565.A0A077RVQ4	4565.A0A3B6KFL8	0	0	0.159	0.739	0.05	0.652	0.614	0.136	0.89
TaMAPKKK1	TaMAPKKK16	4565.A0A3B5XZB3	4565.A0A3B6NTU1	0	0	0.058	0.964	0	0.231	0.43	0.043	0.552

TaMAPKKKK1	TaZIK1	4565.A0A3B5XZB3	4565.Q84XZ4	0	0	0.116	0.568	0	0.699	0	0.151	0.754
TaMAPKKKK1	TaZIK11	4565.A0A3B5XZB3	4565.A0A3B6LI77	0	0	0.117	0.568	0	0.699	0	0.151	0.754
TaMAPKKKK1	TaZIK8	4565.A0A3B5XZB3	4565.A0A3B6C5U6	0	0	0.126	0.616	0	0.699	0	0.151	0.757
TaMAPKKKK1	TaZIK5	4565.A0A3B5XZB3	4565.A0A3B6DA61	0	0	0.13	0.619	0	0.699	0	0.151	0.758
TaMAPKKKK1	TaZIK10	4565.A0A3B5XZB3	4565.A0A3B6NT46	0	0	0.134	0.57	0	0.699	0	0.151	0.759
TaMAPKKKK1	TaZIK2	4565.A0A3B5XZB3	4565.A0A3B6QG64	0	0	0.135	0.57	0	0.699	0	0.151	0.759
TaMAPKKKK1	TaZIK9	4565.A0A3B5XZB3	4565.A0A3B5ZP32	0	0	0.137	0.569	0	0.699	0	0.151	0.76
TaMAPKKKK1	TaZIK7	4565.A0A3B5XZB3	4565.A0A3B6C620	0	0	0.138	0.564	0	0.699	0	0.151	0.76
TaMAPKKKK1	TaZIK4	4565.A0A3B5XZB3	4565.A0A3B6PML2	0	0	0.136	0.572	0	0.699	0	0.151	0.76
TaMAPKKKK1	TaZIK3	4565.A0A3B5XZB3	4565.A0A3B6AUS6	0	0	0.139	0.564	0	0.699	0	0.151	0.761
TaMAPKKKK10	TaMEKK7	4565.A0A3B6KPR0	4565.A0A077RUI2	0	0	0.141	0.625	0.06	0.144	0.548	0	0.646
TaMAPKKKK10	TaRaf18	4565.A0A3B6KPR0	4565.A0A077RY41	0	0	0.11	0.61	0	0.122	0.493	0	0.569
TaMAPKKKK10	TaMEKK9	4565.A0A3B6KPR0	4565.A0A077S2G5	0	0	0.128	0.644	0.06	0.144	0.548	0	0.64
TaMAPKKKK10	TaRaf29	4565.A0A3B6KPR0	4565.A0A1D5UHD7	0	0	0.152	0.587	0	0.122	0.493	0	0.589
TaMAPKKKK10	TaRaf88	4565.A0A3B6KPR0	4565.A0A3B5Z5X1	0	0	0.114	0.616	0	0.122	0.493	0	0.571
TaMAPKKKK10	TaRaf30	4565.A0A3B6KPR0	4565.A0A3B6A1Z4	0	0	0.115	0.614	0	0.122	0.493	0	0.571
TaMAPKKKK10	TaMEKK1	4565.A0A3B6KPR0	4565.A0A3B6B3I4	0	0	0.121	0.617	0.06	0.144	0.548	0	0.637
TaMAPKKKK10	TaMEKK20	4565.A0A3B6KPR0	4565.A0A3B6B6T5	0	0	0.117	0.626	0.06	0.144	0.548	0	0.636

TaMAPKKKK10	TaRaf79	4565.A0A3B6KPR0	4565.A0A3B6B9C7	0	0	0.148	0.593	0	0.122	0.493	0	0.588
TaMAPKKKK10	TaMEKK18	4565.A0A3B6KPR0	4565.A0A3B6CEA6	0	0	0.117	0.627	0.06	0.144	0.548	0	0.636
TaMAPKKKK10	TaRaf59	4565.A0A3B6KPR0	4565.A0A3B6E9E2	0	0	0	0.638	0	0.122	0.493	0	0.535
TaMAPKKKK10	TaRaf60	4565.A0A3B6KPR0	4565.A0A3B6EHW0	0	0	0.142	0.572	0	0.122	0.493	0	0.584
TaMAPKKKK10	TaMEKK8	4565.A0A3B6KPR0	4565.A0A3B6FNU8	0	0	0.129	0.63	0.06	0.144	0.548	0	0.641
TaMAPKKKK10	TaRaf5	4565.A0A3B6KPR0	4565.A0A3B6GYQ1	0	0	0.146	0.576	0	0.122	0.493	0	0.586
TaMAPKKKK10	TaMEKK17	4565.A0A3B6KPR0	4565.A0A3B6KFL8	0	0	0.137	0.643	0.06	0.144	0.548	0	0.644
TaMAPKKKK10	TaRaf100	4565.A0A3B6KPR0	4565.A0A3B6ML06	0	0	0	0.58	0	0.122	0.493	0	0.535
TaMAPKKKK10	TaRaf52	4565.A0A3B6KPR0	4565.A0A3B6LRR0	0	0	0	0.594	0	0.122	0.493	0	0.535
TaMAPKKKK10	TaRaf1	4565.A0A3B6KPR0	4565.A0A3B6NP57	0	0	0.117	0.655	0	0.122	0.493	0	0.572
TaMAPKKKK10	TaRaf58	4565.A0A3B6KPR0	4565.A0A3B6N1Y2	0	0	0.118	0.602	0	0.122	0.493	0	0.573
TaMAPKKKK10	TaRaf46	4565.A0A3B6KPR0	4565.A0A3B6MW69	0	0	0.121	0.637	0	0.122	0.493	0	0.575
TaMAPKKKK10	TaRaf21	4565.A0A3B6KPR0	4565.A0A3B6TVC5	0	0	0.122	0.634	0	0.122	0.493	0	0.575
TaMAPKKKK10	TaRaf62	4565.A0A3B6KPR0	4565.A9RAA9	0	0	0.133	0.635	0	0.122	0.493	0	0.58
TaMAPKKKK10	TaRaf63	4565.A0A3B6KPR0	4565.A0A3B6RKW0	0	0	0.137	0.583	0	0.122	0.493	0	0.582
TaMAPKKKK10	TaRaf91	4565.A0A3B6KPR0	4565.A0A3B6PMI5	0	0	0.169	0.573	0	0.122	0.493	0	0.598
TaMAPKKKK10	TaMEKK4-1	4565.A0A3B6KPR0	4565.A0A3B6PNI6	0	0	0.114	0.617	0.06	0.144	0.548	0	0.635
TaMAPKKKK10	TaMEKK4	4565.A0A3B6KPR0	4565.A0A3B6NRN9	0	0	0.12	0.608	0.06	0.144	0.548	0	0.637

TaMAPKKKK10	TaMEKK29	4565.A0A3B6KPR0	4565.A0A3B6MSP6	0	0	0.137	0.643	0.06	0.144	0.548	0	0.644
TaMAPKKKK10	TaMEKK24	4565.A0A3B6KPR0	4565.A0A3B6LLV5	0	0	0.137	0.643	0.06	0.144	0.548	0	0.644
TaMAPKKKK11	TaZIK9	4565.A0A3B6KVE1	4565.A0A3B5ZP32	0	0	0.151	0.57	0	0.699	0	0.151	0.764
TaMAPKKKK11	TaZIK3	4565.A0A3B6KVE1	4565.A0A3B6AUS6	0	0	0.157	0.56	0	0.699	0	0.151	0.765
TaMAPKKKK11	TaZIK8	4565.A0A3B6KVE1	4565.A0A3B6C5U6	0	0	0.14	0.607	0	0.699	0	0.151	0.761
TaMAPKKKK11	TaZIK7	4565.A0A3B6KVE1	4565.A0A3B6C620	0	0	0.155	0.56	0	0.699	0	0.151	0.765
TaMAPKKKK11	TaZIK5	4565.A0A3B6KVE1	4565.A0A3B6DA61	0	0	0.143	0.61	0	0.699	0	0.151	0.762
TaMAPKKKK11	TaMAPKKKK16	4565.A0A3B6KVE1	4565.A0A3B6NTU1	0	0	0.06	0.96	0	0.231	0.43	0.043	0.553
TaMAPKKKK11	TaZIK11	4565.A0A3B6KVE1	4565.A0A3B6LI77	0	0	0.129	0.564	0	0.699	0	0.151	0.758
TaMAPKKKK11	TaZIK1	4565.A0A3B6KVE1	4565.Q84XZ4	0	0	0.13	0.563	0	0.699	0	0.151	0.758
TaMAPKKKK11	TaZIK2	4565.A0A3B6KVE1	4565.A0A3B6QG64	0	0	0.15	0.565	0	0.699	0	0.151	0.763
TaMAPKKKK11	TaZIK10	4565.A0A3B6KVE1	4565.A0A3B6NT46	0	0	0.15	0.564	0	0.699	0	0.151	0.763
TaMAPKKKK11	TaZIK4	4565.A0A3B6KVE1	4565.A0A3B6PML2	0	0	0.152	0.567	0	0.699	0	0.151	0.764
TaMAPKKKK12	TaMEKK7	4565.A0A3B6LTF3	4565.A0A077RUI2	0	0	0.14	0.625	0.06	0.144	0.548	0	0.645
TaMAPKKKK12	TaRaf18	4565.A0A3B6LTF3	4565.A0A077RY41	0	0	0.11	0.609	0	0.122	0.493	0	0.569
TaMAPKKKK12	TaMEKK9	4565.A0A3B6LTF3	4565.A0A077S2G5	0	0	0.127	0.645	0.06	0.144	0.548	0	0.64
TaMAPKKKK12	TaRaf29	4565.A0A3B6LTF3	4565.A0A1D5UHD7	0	0	0.151	0.589	0	0.122	0.493	0	0.589
TaMAPKKKK12	TaRaf88	4565.A0A3B6LTF3	4565.A0A3B5Z5X1	0	0	0.114	0.615	0	0.122	0.493	0	0.571

TaMAPKKKK12	TaRaf30	4565.A0A3B6LTF3	4565.A0A3B6A1Z4	0	0	0.115	0.613	0	0.122	0.493	0	0.571
TaMAPKKKK12	TaMEKK1	4565.A0A3B6LTF3	4565.A0A3B6B3I4	0	0	0.119	0.617	0.06	0.144	0.548	0	0.636
TaMAPKKKK12	TaMEKK20	4565.A0A3B6LTF3	4565.A0A3B6B6T5	0	0	0.118	0.62	0.06	0.144	0.548	0	0.636
TaMAPKKKK12	TaRaf79	4565.A0A3B6LTF3	4565.A0A3B6B9C7	0	0	0.147	0.595	0	0.122	0.493	0	0.587
TaMAPKKKK12	TaMEKK18	4565.A0A3B6LTF3	4565.A0A3B6CEA6	0	0	0.118	0.62	0.06	0.144	0.548	0	0.636
TaMAPKKKK12	TaRaf59	4565.A0A3B6LTF3	4565.A0A3B6E9E2	0	0	0	0.635	0	0.122	0.493	0	0.535
TaMAPKKKK12	TaRaf60	4565.A0A3B6LTF3	4565.A0A3B6EHW0	0	0	0.14	0.573	0	0.122	0.493	0	0.583
TaMAPKKKK12	TaMEKK8	4565.A0A3B6LTF3	4565.A0A3B6FNU8	0	0	0.128	0.629	0.06	0.144	0.548	0	0.64
TaMAPKKKK12	TaRaf5	4565.A0A3B6LTF3	4565.A0A3B6GYQ1	0	0	0.144	0.576	0	0.122	0.493	0	0.586
TaMAPKKKK12	TaMEKK17	4565.A0A3B6LTF3	4565.A0A3B6KFL8	0	0	0.136	0.644	0.06	0.144	0.548	0	0.643
TaMAPKKKK12	TaMEKK24	4565.A0A3B6LTF3	4565.A0A3B6LLV5	0	0	0.135	0.644	0.06	0.144	0.548	0	0.643
TaMAPKKKK12	TaRaf52	4565.A0A3B6LTF3	4565.A0A3B6LRR0	0	0	0	0.593	0	0.122	0.493	0	0.535
TaMAPKKKK12	TaRaf100	4565.A0A3B6LTF3	4565.A0A3B6ML06	0	0	0	0.578	0	0.122	0.493	0	0.535
TaMAPKKKK12	TaRaf1	4565.A0A3B6LTF3	4565.A0A3B6NP57	0	0	0.116	0.659	0	0.122	0.493	0	0.572
TaMAPKKKK12	TaRaf58	4565.A0A3B6LTF3	4565.A0A3B6N1Y2	0	0	0.118	0.603	0	0.122	0.493	0	0.573
TaMAPKKKK12	TaRaf21	4565.A0A3B6LTF3	4565.A0A3B6TVC5	0	0	0.121	0.637	0	0.122	0.493	0	0.574
TaMAPKKKK12	TaRaf46	4565.A0A3B6LTF3	4565.A0A3B6MW69	0	0	0.122	0.637	0	0.122	0.493	0	0.575
TaMAPKKKK12	TaRaf62	4565.A0A3B6LTF3	4565.A9RAA9	0	0	0.132	0.635	0	0.122	0.493	0	0.58

TaMAPKKKK12	TaRaf63	4565.A0A3B6LTF3	4565.A0A3B6RKW0	0	0	0.138	0.583	0	0.122	0.493	0	0.582
TaMAPKKKK12	TaRaf91	4565.A0A3B6LTF3	4565.A0A3B6PMI5	0	0	0.168	0.571	0	0.122	0.493	0	0.597
TaMAPKKKK12	TaMEKK4-1	4565.A0A3B6LTF3	4565.A0A3B6PNI6	0	0	0.113	0.617	0.06	0.144	0.548	0	0.634
TaMAPKKKK12	TaMEKK4	4565.A0A3B6LTF3	4565.A0A3B6NRN9	0	0	0.119	0.603	0.06	0.144	0.548	0	0.636
TaMAPKKKK12	TaMEKK29	4565.A0A3B6LTF3	4565.A0A3B6MSP6	0	0	0.136	0.644	0.06	0.144	0.548	0	0.643
TaMAPKKKK13	TaZIK9	4565.A0A3B6MRC5	4565.A0A3B5ZP32	0	0	0.109	0.572	0	0.699	0	0.151	0.752
TaMAPKKKK13	TaZIK3	4565.A0A3B6MRC5	4565.A0A3B6AUS6	0	0	0.118	0.571	0	0.699	0	0.151	0.754
TaMAPKKKK13	TaZIK8	4565.A0A3B6MRC5	4565.A0A3B6C5U6	0	0	0.111	0.596	0	0.699	0	0.151	0.753
TaMAPKKKK13	TaZIK7	4565.A0A3B6MRC5	4565.A0A3B6C620	0	0	0.117	0.571	0	0.699	0	0.151	0.754
TaMAPKKKK13	TaZIK5	4565.A0A3B6MRC5	4565.A0A3B6DA61	0	0	0.115	0.598	0	0.699	0	0.151	0.754
TaMAPKKKK13	TaZIK11	4565.A0A3B6MRC5	4565.A0A3B6LI77	0	0	0	0.565	0	0.699	0	0.151	0.733
TaMAPKKKK13	TaMAPKKKK16	4565.A0A3B6MRC5	4565.A0A3B6NTU1	0	0	0.079	0.916	0	0.231	0.43	0.043	0.562
TaMAPKKKK13	TaZIK1	4565.A0A3B6MRC5	4565.Q84XZ4	0	0	0	0.562	0	0.699	0	0.151	0.733
TaMAPKKKK13	TaZIK2	4565.A0A3B6MRC5	4565.A0A3B6QG64	0	0	0.109	0.57	0	0.699	0	0.151	0.752
TaMAPKKKK13	TaZIK4	4565.A0A3B6MRC5	4565.A0A3B6PML2	0	0	0.109	0.569	0	0.699	0	0.151	0.752
TaMAPKKKK13	TaZIK10	4565.A0A3B6MRC5	4565.A0A3B6NT46	0	0	0.109	0.569	0	0.699	0	0.151	0.752
TaMAPKKKK14	TaMEKK7	4565.A0A3B6MZG0	4565.A0A077RUI2	0	0	0.141	0.624	0.06	0.144	0.548	0	0.645
TaMAPKKKK14	TaRaf18	4565.A0A3B6MZG0	4565.A0A077RY41	0	0	0.11	0.609	0	0.122	0.493	0	0.569

TaMAPKKKK14	TaMEKK9	4565.A0A3B6MZG0	4565.A0A077S2G5	0	0	0.127	0.645	0.06	0.144	0.548	0	0.64
TaMAPKKKK14	TaRaf29	4565.A0A3B6MZG0	4565.A0A1D5UHD7	0	0	0.151	0.589	0	0.122	0.493	0	0.589
TaMAPKKKK14	TaRaf88	4565.A0A3B6MZG0	4565.A0A3B5Z5X1	0	0	0.114	0.615	0	0.122	0.493	0	0.571
TaMAPKKKK14	TaRaf30	4565.A0A3B6MZG0	4565.A0A3B6A1Z4	0	0	0.115	0.613	0	0.122	0.493	0	0.571
TaMAPKKKK14	TaMEKK1	4565.A0A3B6MZG0	4565.A0A3B6B3I4	0	0	0.119	0.617	0.06	0.144	0.548	0	0.637
TaMAPKKKK14	TaMEKK20	4565.A0A3B6MZG0	4565.A0A3B6B6T5	0	0	0.117	0.626	0.06	0.144	0.548	0	0.636
TaMAPKKKK14	TaRaf79	4565.A0A3B6MZG0	4565.A0A3B6B9C7	0	0	0.147	0.595	0	0.122	0.493	0	0.587
TaMAPKKKK14	TaMEKK18	4565.A0A3B6MZG0	4565.A0A3B6CEA6	0	0	0.117	0.627	0.06	0.144	0.548	0	0.636
TaMAPKKKK14	TaRaf59	4565.A0A3B6MZG0	4565.A0A3B6E9E2	0	0	0	0.635	0	0.122	0.493	0	0.535
TaMAPKKKK14	TaRaf60	4565.A0A3B6MZG0	4565.A0A3B6EHW0	0	0	0.139	0.574	0	0.122	0.493	0	0.583
TaMAPKKKK14	TaMEKK8	4565.A0A3B6MZG0	4565.A0A3B6FNU8	0	0	0.129	0.629	0.06	0.144	0.548	0	0.64
TaMAPKKKK14	TaRaf5	4565.A0A3B6MZG0	4565.A0A3B6GYQ1	0	0	0.144	0.576	0	0.122	0.493	0	0.585
TaMAPKKKK14	TaMEKK17	4565.A0A3B6MZG0	4565.A0A3B6KFL8	0	0	0.136	0.644	0.06	0.144	0.548	0	0.643
TaMAPKKKK14	TaMEKK24	4565.A0A3B6MZG0	4565.A0A3B6LLV5	0	0	0.135	0.644	0.06	0.144	0.548	0	0.643
TaMAPKKKK14	TaRaf52	4565.A0A3B6MZG0	4565.A0A3B6LRR0	0	0	0	0.593	0	0.122	0.493	0	0.535
TaMAPKKKK14	TaRaf100	4565.A0A3B6MZG0	4565.A0A3B6ML06	0	0	0	0.579	0	0.122	0.493	0	0.535
TaMAPKKKK14	TaMEKK29	4565.A0A3B6MZG0	4565.A0A3B6MSP6	0	0	0.136	0.644	0.06	0.144	0.548	0	0.643
TaMAPKKKK14	TaRaf46	4565.A0A3B6MZG0	4565.A0A3B6MW69	0	0	0.122	0.637	0	0.122	0.493	0	0.575

TaMAPKKKK14	TaRaf1	4565.A0A3B6MZG0	4565.A0A3B6NP57	0	0	0.116	0.659	0	0.122	0.493	0	0.572
TaMAPKKKK14	TaRaf58	4565.A0A3B6MZG0	4565.A0A3B6N1Y2	0	0	0.118	0.601	0	0.122	0.493	0	0.573
TaMAPKKKK14	TaRaf21	4565.A0A3B6MZG0	4565.A0A3B6TVC5	0	0	0.121	0.637	0	0.122	0.493	0	0.574
TaMAPKKKK14	TaRaf62	4565.A0A3B6MZG0	4565.A9RAA9	0	0	0.132	0.635	0	0.122	0.493	0	0.58
TaMAPKKKK14	TaRaf63	4565.A0A3B6MZG0	4565.A0A3B6RKW0	0	0	0.137	0.583	0	0.122	0.493	0	0.582
TaMAPKKKK14	TaRaf91	4565.A0A3B6MZG0	4565.A0A3B6PMI5	0	0	0.167	0.574	0	0.122	0.493	0	0.597
TaMAPKKKK14	TaMEKK4-1	4565.A0A3B6MZG0	4565.A0A3B6PNI6	0	0	0.113	0.617	0.06	0.144	0.548	0	0.634
TaMAPKKKK14	TaMEKK4	4565.A0A3B6MZG0	4565.A0A3B6NRN9	0	0	0.119	0.608	0.06	0.144	0.548	0	0.636
TaMAPKKKK15	TaZIK9	4565.A0A3B6NNC4	4565.A0A3B5ZP32	0	0	0.157	0.574	0	0.699	0	0.151	0.765
TaMAPKKKK15	TaZIK3	4565.A0A3B6NNC4	4565.A0A3B6AUS6	0	0	0.16	0.573	0	0.699	0	0.151	0.766
TaMAPKKKK15	TaZIK8	4565.A0A3B6NNC4	4565.A0A3B6C5U6	0	0	0.15	0.63	0	0.699	0	0.151	0.763
TaMAPKKKK15	TaZIK7	4565.A0A3B6NNC4	4565.A0A3B6C620	0	0	0.159	0.573	0	0.699	0	0.151	0.766
TaMAPKKKK15	TaZIK5	4565.A0A3B6NNC4	4565.A0A3B6DA61	0	0	0.155	0.634	0	0.699	0	0.151	0.765
TaMAPKKKK15	TaZIK11	4565.A0A3B6NNC4	4565.A0A3B6LI77	0	0	0.135	0.582	0	0.699	0	0.151	0.759
TaMAPKKKK15	TaMAPKKKK16	4565.A0A3B6NNC4	4565.A0A3B6NTU1	0	0	0.064	0.952	0	0.231	0.43	0.043	0.555
TaMAPKKKK15	TaZIK1	4565.A0A3B6NNC4	4565.Q84XZ4	0	0	0.135	0.581	0	0.699	0	0.151	0.759
TaMAPKKKK15	TaZIK10	4565.A0A3B6NNC4	4565.A0A3B6NT46	0	0	0.16	0.572	0	0.699	0	0.151	0.766
TaMAPKKKK15	TaZIK2	4565.A0A3B6NNC4	4565.A0A3B6QG64	0	0	0.16	0.573	0	0.699	0	0.151	0.766

TaMAPKKKK15	TaZIK4	4565.A0A3B6NNC4	4565.A0A3B6PML2	0	0	0.161	0.577	0	0.699	0	0.151	0.767
TaMAPKKKK16	TaMAPKKKK2	4565.A0A3B6NTU1	4565.A0A3B5YXW4	0	0	0.058	0.964	0	0.231	0.43	0.043	0.552
TaMAPKKKK16	TaMAPKKKK3	4565.A0A3B6NTU1	4565.A0A3B5ZTC4	0	0	0.059	0.963	0	0.231	0.43	0.043	0.552
TaMAPKKKK16	TaMEKK12	4565.A0A3B6NTU1	4565.A0A3B6HQC5	0	0	0.108	0.676	0	0.165	0.603	0.098	0.697
TaMAPKKKK16	TaMEKK3	4565.A0A3B6NTU1	4565.A0A3B6ISF1	0	0	0.109	0.671	0	0.165	0.603	0.098	0.698
TaMAPKKKK16	TaMAPKKKK7	4565.A0A3B6NTU1	4565.A0A3B6IX47	0	0	0.074	0.929	0	0.231	0.43	0.043	0.559
TaMAPKKKK16	TaMAPKKKK8	4565.A0A3B6NTU1	4565.A0A3B6J0T1	0	0	0.059	0.962	0	0.231	0.43	0.043	0.552
TaMAPKKKK16	TaMEKK10	4565.A0A3B6NTU1	4565.A0A3B6JM32	0	0	0.109	0.671	0	0.165	0.603	0.098	0.698
TaMAPKKKK16	TaMAPKKKK9	4565.A0A3B6NTU1	4565.A0A3B6KID5	0	0	0.079	0.916	0	0.231	0.43	0.043	0.562
TaMAPKKKK16	TaMEKK25	4565.A0A3B6NTU1	4565.A0A3B6LLP7	0	0	0.079	0.917	0	0.231	0.43	0.043	0.562
TaMAPKKKK16	TaMAPKKKK20	4565.A0A3B6NTU1	4565.A0A3B6QMW0	0	0	0.047	0.987	0	0.231	0.43	0.043	0.546
TaMAPKKKK16	TaMAPKKKK19	4565.A0A3B6NTU1	4565.A0A3B6PS74	0	0	0.047	0.987	0	0.231	0.43	0.043	0.546
TaMAPKKKK16	TaMAPKKKK17	4565.A0A3B6NTU1	4565.A0A3B6NVG6	0	0	0.048	0.985	0	0.231	0.43	0.043	0.547
TaMAPKKKK16	TaMAPKKKK23	4565.A0A3B6NTU1	4565.A0A3B6SHY6	0	0	0.055	0.97	0	0.231	0.43	0.043	0.55
TaMAPKKKK16	TaMAPKKKK24	4565.A0A3B6NTU1	4565.A0A3B6TDA8	0	0	0.055	0.97	0	0.231	0.43	0.043	0.55
TaMAPKKKK16	TaMAPKKKK22	4565.A0A3B6NTU1	4565.A0A3B6RD82	0	0	0.055	0.97	0	0.231	0.43	0.043	0.55
TaMAPKKKK16	TaMAPKKKK25	4565.A0A3B6NTU1	4565.A0A3B6UB28	0	0	0.059	0.963	0	0.231	0.43	0.043	0.552
TaMAPKKKK16	TaMAPKKKK18	4565.A0A3B6NTU1	4565.A0A3B6PL54	0	0	0.064	0.953	0	0.231	0.43	0.043	0.554

TaMAPKKKK16	TaMAPKKKK21	4565.A0A3B6NTU1	4565.A0A3B6QCF0	0	0	0.067	0.947	0	0.231	0.43	0.043	0.556
TaMAPKKKK17	TaZIK9	4565.A0A3B6NVG6	4565.A0A3B5ZP32	0	0	0.147	0.564	0	0.699	0	0.151	0.763
TaMAPKKKK17	TaZIK3	4565.A0A3B6NVG6	4565.A0A3B6AUS6	0	0	0.143	0.564	0	0.699	0	0.151	0.762
TaMAPKKKK17	TaZIK8	4565.A0A3B6NVG6	4565.A0A3B6C5U6	0	0	0.126	0.602	0	0.699	0	0.151	0.757
TaMAPKKKK17	TaZIK7	4565.A0A3B6NVG6	4565.A0A3B6C620	0	0	0.141	0.564	0	0.699	0	0.151	0.761
TaMAPKKKK17	TaZIK5	4565.A0A3B6NVG6	4565.A0A3B6DA61	0	0	0.13	0.605	0	0.699	0	0.151	0.758
TaMAPKKKK17	TaZIK11	4565.A0A3B6NVG6	4565.A0A3B6LI77	0	0	0.119	0.562	0	0.699	0	0.151	0.755
TaMAPKKKK17	TaZIK10	4565.A0A3B6NVG6	4565.A0A3B6NT46	0	0	0.141	0.564	0	0.699	0	0.151	0.761
TaMAPKKKK17	TaZIK1	4565.A0A3B6NVG6	4565.Q84XZ4	0	0	0.118	0.563	0	0.699	0	0.151	0.755
TaMAPKKKK17	TaZIK2	4565.A0A3B6NVG6	4565.A0A3B6QG64	0	0	0.141	0.565	0	0.699	0	0.151	0.761
TaMAPKKKK17	TaZIK4	4565.A0A3B6NVG6	4565.A0A3B6PML2	0	0	0.142	0.566	0	0.699	0	0.151	0.761
TaMAPKKKK18	TaZIK9	4565.A0A3B6PL54	4565.A0A3B5ZP32	0	0	0.156	0.574	0	0.699	0	0.151	0.765
TaMAPKKKK18	TaZIK3	4565.A0A3B6PL54	4565.A0A3B6AUS6	0	0	0.158	0.573	0	0.699	0	0.151	0.766
TaMAPKKKK18	TaZIK8	4565.A0A3B6PL54	4565.A0A3B6C5U6	0	0	0.149	0.63	0	0.699	0	0.151	0.763
TaMAPKKKK18	TaZIK7	4565.A0A3B6PL54	4565.A0A3B6C620	0	0	0.157	0.573	0	0.699	0	0.151	0.765
TaMAPKKKK18	TaZIK5	4565.A0A3B6PL54	4565.A0A3B6DA61	0	0	0.154	0.634	0	0.699	0	0.151	0.765
TaMAPKKKK18	TaZIK11	4565.A0A3B6PL54	4565.A0A3B6LI77	0	0	0.133	0.582	0	0.699	0	0.151	0.759
TaMAPKKKK18	TaZIK10	4565.A0A3B6PL54	4565.A0A3B6NT46	0	0	0.159	0.572	0	0.699	0	0.151	0.766

TaMAPKKKK18	TaZIK1	4565.A0A3B6PL54	4565.Q84XZ4	0	0	0.133	0.581	0	0.699	0	0.151	0.759
TaMAPKKKK18	TaZIK2	4565.A0A3B6PL54	4565.A0A3B6QG64	0	0	0.159	0.573	0	0.699	0	0.151	0.766
TaMAPKKKK18	TaZIK4	4565.A0A3B6PL54	4565.A0A3B6PML2	0	0	0.16	0.577	0	0.699	0	0.151	0.766
TaMAPKKKK19	TaZIK9	4565.A0A3B6PS74	4565.A0A3B5ZP32	0	0	0.142	0.573	0	0.699	0	0.151	0.761
TaMAPKKKK19	TaZIK3	4565.A0A3B6PS74	4565.A0A3B6AUS6	0	0	0.14	0.569	0	0.699	0	0.151	0.761
TaMAPKKKK19	TaZIK8	4565.A0A3B6PS74	4565.A0A3B6C5U6	0	0	0.126	0.629	0	0.699	0	0.151	0.757
TaMAPKKKK19	TaZIK7	4565.A0A3B6PS74	4565.A0A3B6C620	0	0	0.138	0.569	0	0.699	0	0.151	0.76
TaMAPKKKK19	TaZIK5	4565.A0A3B6PS74	4565.A0A3B6DA61	0	0	0.131	0.632	0	0.699	0	0.151	0.758
TaMAPKKKK19	TaZIK11	4565.A0A3B6PS74	4565.A0A3B6LI77	0	0	0.119	0.572	0	0.699	0	0.151	0.755
TaMAPKKKK19	TaZIK10	4565.A0A3B6PS74	4565.A0A3B6NT46	0	0	0.139	0.575	0	0.699	0	0.151	0.76
TaMAPKKKK19	TaZIK4	4565.A0A3B6PS74	4565.A0A3B6PML2	0	0	0.14	0.577	0	0.699	0	0.151	0.761
TaMAPKKKK19	TaZIK1	4565.A0A3B6PS74	4565.Q84XZ4	0	0	0.117	0.573	0	0.699	0	0.151	0.754
TaMAPKKKK19	TaZIK2	4565.A0A3B6PS74	4565.A0A3B6QG64	0	0	0.14	0.575	0	0.699	0	0.151	0.761
TaMAPKKKK2	TaZIK1	4565.A0A3B5YXW4	4565.Q84XZ4	0	0	0.116	0.568	0	0.699	0	0.151	0.754
TaMAPKKKK2	TaZIK11	4565.A0A3B5YXW4	4565.A0A3B6LI77	0	0	0.117	0.568	0	0.699	0	0.151	0.754
TaMAPKKKK2	TaZIK8	4565.A0A3B5YXW4	4565.A0A3B6C5U6	0	0	0.127	0.616	0	0.699	0	0.151	0.757
TaMAPKKKK2	TaZIK5	4565.A0A3B5YXW4	4565.A0A3B6DA61	0	0	0.131	0.618	0	0.699	0	0.151	0.758
TaMAPKKKK2	TaZIK2	4565.A0A3B5YXW4	4565.A0A3B6QG64	0	0	0.135	0.57	0	0.699	0	0.151	0.759

TaMAPKKKK2	TaZIK10	4565.A0A3B5YXW4	4565.A0A3B6NT46	0	0	0.134	0.57	0	0.699	0	0.151	0.759
TaMAPKKKK2	TaZIK7	4565.A0A3B5YXW4	4565.A0A3B6C620	0	0	0.138	0.564	0	0.699	0	0.151	0.76
TaMAPKKKK2	TaZIK9	4565.A0A3B5YXW4	4565.A0A3B5ZP32	0	0	0.137	0.569	0	0.699	0	0.151	0.76
TaMAPKKKK2	TaZIK4	4565.A0A3B5YXW4	4565.A0A3B6PML2	0	0	0.136	0.572	0	0.699	0	0.151	0.76
TaMAPKKKK2	TaZIK3	4565.A0A3B5YXW4	4565.A0A3B6AUS6	0	0	0.139	0.564	0	0.699	0	0.151	0.761
TaMAPKKKK20	TaZIK9	4565.A0A3B6QMW0	4565.A0A3B5ZP32	0	0	0.142	0.571	0	0.699	0	0.151	0.761
TaMAPKKKK20	TaZIK3	4565.A0A3B6QMW0	4565.A0A3B6AUS6	0	0	0.139	0.569	0	0.699	0	0.151	0.76
TaMAPKKKK20	TaZIK8	4565.A0A3B6QMW0	4565.A0A3B6C5U6	0	0	0.126	0.629	0	0.699	0	0.151	0.757
TaMAPKKKK20	TaZIK7	4565.A0A3B6QMW0	4565.A0A3B6C620	0	0	0.138	0.569	0	0.699	0	0.151	0.76
TaMAPKKKK20	TaZIK5	4565.A0A3B6QMW0	4565.A0A3B6DA61	0	0	0.131	0.632	0	0.699	0	0.151	0.758
TaMAPKKKK20	TaZIK11	4565.A0A3B6QMW0	4565.A0A3B6LI77	0	0	0.12	0.571	0	0.699	0	0.151	0.755
TaMAPKKKK20	TaZIK10	4565.A0A3B6QMW0	4565.A0A3B6NT46	0	0	0.139	0.575	0	0.699	0	0.151	0.76
TaMAPKKKK20	TaZIK4	4565.A0A3B6QMW0	4565.A0A3B6PML2	0	0	0.139	0.58	0	0.699	0	0.151	0.76
TaMAPKKKK20	TaZIK2	4565.A0A3B6QMW0	4565.A0A3B6QG64	0	0	0.14	0.575	0	0.699	0	0.151	0.761
TaMAPKKKK20	TaZIK1	4565.A0A3B6QMW0	4565.Q84XZ4	0	0	0.118	0.571	0	0.699	0	0.151	0.754
TaMAPKKKK21	TaZIK9	4565.A0A3B6QCF0	4565.A0A3B5ZP32	0	0	0.151	0.578	0	0.699	0	0.151	0.764
TaMAPKKKK21	TaZIK3	4565.A0A3B6QCF0	4565.A0A3B6AUS6	0	0	0.16	0.576	0	0.699	0	0.151	0.766
TaMAPKKKK21	TaZIK8	4565.A0A3B6QCF0	4565.A0A3B6C5U6	0	0	0.151	0.63	0	0.699	0	0.151	0.764

TaMAPKKKK21	TaZIK7	4565.A0A3B6QCF0	4565.A0A3B6C620	0	0	0.158	0.576	0	0.699	0	0.151	0.766
TaMAPKKKK21	TaZIK5	4565.A0A3B6QCF0	4565.A0A3B6DA61	0	0	0.156	0.633	0	0.699	0	0.151	0.765
TaMAPKKKK21	TaZIK11	4565.A0A3B6QCF0	4565.A0A3B6LI77	0	0	0.134	0.586	0	0.699	0	0.151	0.759
TaMAPKKKK21	TaZIK10	4565.A0A3B6QCF0	4565.A0A3B6NT46	0	0	0.16	0.575	0	0.699	0	0.151	0.766
TaMAPKKKK21	TaZIK4	4565.A0A3B6QCF0	4565.A0A3B6PML2	0	0	0.161	0.58	0	0.699	0	0.151	0.766
TaMAPKKKK21	TaZIK1	4565.A0A3B6QCF0	4565.Q84XZ4	0	0	0.133	0.586	0	0.699	0	0.151	0.759
TaMAPKKKK21	TaZIK2	4565.A0A3B6QCF0	4565.A0A3B6QG64	0	0	0.16	0.576	0	0.699	0	0.151	0.766
TaMAPKKKK22	TaZIK9	4565.A0A3B6RD82	4565.A0A3B5ZP32	0	0	0.136	0.572	0	0.699	0	0.151	0.76
TaMAPKKKK22	TaZIK3	4565.A0A3B6RD82	4565.A0A3B6AUS6	0	0	0.142	0.568	0	0.699	0	0.151	0.761
TaMAPKKKK22	TaZIK8	4565.A0A3B6RD82	4565.A0A3B6C5U6	0	0	0.13	0.619	0	0.699	0	0.151	0.758
TaMAPKKKK22	TaZIK7	4565.A0A3B6RD82	4565.A0A3B6C620	0	0	0.139	0.569	0	0.699	0	0.151	0.76
TaMAPKKKK22	TaZIK5	4565.A0A3B6RD82	4565.A0A3B6DA61	0	0	0.135	0.622	0	0.699	0	0.151	0.759
TaMAPKKKK22	TaZIK11	4565.A0A3B6RD82	4565.A0A3B6LI77	0	0	0.123	0.57	0	0.699	0	0.151	0.756
TaMAPKKKK22	TaZIK10	4565.A0A3B6RD82	4565.A0A3B6NT46	0	0	0.14	0.574	0	0.699	0	0.151	0.761
TaMAPKKKK22	TaZIK4	4565.A0A3B6RD82	4565.A0A3B6PML2	0	0	0.142	0.574	0	0.699	0	0.151	0.761
TaMAPKKKK22	TaZIK2	4565.A0A3B6RD82	4565.A0A3B6QG64	0	0	0.14	0.574	0	0.699	0	0.151	0.761
TaMAPKKKK22	TaZIK1	4565.A0A3B6RD82	4565.Q84XZ4	0	0	0.122	0.569	0	0.699	0	0.151	0.756
TaMAPKKKK23	TaZIK9	4565.A0A3B6SHY6	4565.A0A3B5ZP32	0	0	0.138	0.571	0	0.699	0	0.151	0.76

TaMAPKKKK23	TaZIK3	4565.A0A3B6SHY6	4565.A0A3B6AUS6	0	0	0.142	0.568	0	0.699	0	0.151	0.761
TaMAPKKKK23	TaZIK8	4565.A0A3B6SHY6	4565.A0A3B6C5U6	0	0	0.131	0.621	0	0.699	0	0.151	0.758
TaMAPKKKK23	TaZIK7	4565.A0A3B6SHY6	4565.A0A3B6C620	0	0	0.14	0.569	0	0.699	0	0.151	0.761
TaMAPKKKK23	TaZIK5	4565.A0A3B6SHY6	4565.A0A3B6DA61	0	0	0.135	0.623	0	0.699	0	0.151	0.759
TaMAPKKKK23	TaZIK11	4565.A0A3B6SHY6	4565.A0A3B6LI77	0	0	0.125	0.57	0	0.699	0	0.151	0.756
TaMAPKKKK23	TaZIK10	4565.A0A3B6SHY6	4565.A0A3B6NT46	0	0	0.141	0.574	0	0.699	0	0.151	0.761
TaMAPKKKK23	TaZIK4	4565.A0A3B6SHY6	4565.A0A3B6PML2	0	0	0.143	0.574	0	0.699	0	0.151	0.762
TaMAPKKKK23	TaZIK2	4565.A0A3B6SHY6	4565.A0A3B6QG64	0	0	0.141	0.574	0	0.699	0	0.151	0.761
TaMAPKKKK23	TaZIK1	4565.A0A3B6SHY6	4565.Q84XZ4	0	0	0.124	0.568	0	0.699	0	0.151	0.756
TaMAPKKKK24	TaZIK9	4565.A0A3B6TDA8	4565.A0A3B5ZP32	0	0	0.139	0.571	0	0.699	0	0.151	0.76
TaMAPKKKK24	TaZIK3	4565.A0A3B6TDA8	4565.A0A3B6AUS6	0	0	0.143	0.567	0	0.699	0	0.151	0.762
TaMAPKKKK24	TaZIK8	4565.A0A3B6TDA8	4565.A0A3B6C5U6	0	0	0.132	0.619	0	0.699	0	0.151	0.758
TaMAPKKKK24	TaZIK7	4565.A0A3B6TDA8	4565.A0A3B6C620	0	0	0.141	0.567	0	0.699	0	0.151	0.761
TaMAPKKKK24	TaZIK5	4565.A0A3B6TDA8	4565.A0A3B6DA61	0	0	0.137	0.622	0	0.699	0	0.151	0.76
TaMAPKKKK24	TaZIK11	4565.A0A3B6TDA8	4565.A0A3B6LI77	0	0	0.126	0.569	0	0.699	0	0.151	0.757
TaMAPKKKK24	TaZIK10	4565.A0A3B6TDA8	4565.A0A3B6NT46	0	0	0.142	0.573	0	0.699	0	0.151	0.761
TaMAPKKKK24	TaZIK4	4565.A0A3B6TDA8	4565.A0A3B6PML2	0	0	0.144	0.573	0	0.699	0	0.151	0.762
TaMAPKKKK24	TaZIK2	4565.A0A3B6TDA8	4565.A0A3B6QG64	0	0	0.142	0.574	0	0.699	0	0.151	0.761

TaMAPKKKK24	TaZIK1	4565.A0A3B6TDA8	4565.Q84XZ4	0	0	0.125	0.568	0	0.699	0	0.151	0.757
TaMAPKKKK25	TaZIK9	4565.A0A3B6UB28	4565.A0A3B5ZP32	0	0	0.145	0.577	0	0.699	0	0.151	0.762
TaMAPKKKK25	TaZIK3	4565.A0A3B6UB28	4565.A0A3B6AUS6	0	0	0.148	0.565	0	0.699	0	0.151	0.763
TaMAPKKKK25	TaZIK8	4565.A0A3B6UB28	4565.A0A3B6C5U6	0	0	0.131	0.615	0	0.699	0	0.151	0.758
TaMAPKKKK25	TaZIK7	4565.A0A3B6UB28	4565.A0A3B6C620	0	0	0.147	0.565	0	0.699	0	0.151	0.763
TaMAPKKKK25	TaZIK5	4565.A0A3B6UB28	4565.A0A3B6DA61	0	0	0.136	0.62	0	0.699	0	0.151	0.759
TaMAPKKKK25	TaZIK11	4565.A0A3B6UB28	4565.A0A3B6LI77	0	0	0.124	0.565	0	0.699	0	0.151	0.756
TaMAPKKKK25	TaZIK10	4565.A0A3B6UB28	4565.A0A3B6NT46	0	0	0.141	0.571	0	0.699	0	0.151	0.761
TaMAPKKKK25	TaZIK4	4565.A0A3B6UB28	4565.A0A3B6PML2	0	0	0.144	0.574	0	0.699	0	0.151	0.762
TaMAPKKKK25	TaZIK2	4565.A0A3B6UB28	4565.A0A3B6QG64	0	0	0.142	0.571	0	0.699	0	0.151	0.761
TaMAPKKKK25	TaZIK1	4565.A0A3B6UB28	4565.Q84XZ4	0	0	0.123	0.565	0	0.699	0	0.151	0.756
TaMAPKKKK3	TaZIK9	4565.A0A3B5ZTC4	4565.A0A3B5ZP32	0	0	0.138	0.569	0	0.699	0	0.151	0.76
TaMAPKKKK3	TaZIK1	4565.A0A3B5ZTC4	4565.Q84XZ4	0	0	0.116	0.568	0	0.699	0	0.151	0.754
TaMAPKKKK3	TaZIK11	4565.A0A3B5ZTC4	4565.A0A3B6LI77	0	0	0.117	0.568	0	0.699	0	0.151	0.754
TaMAPKKKK3	TaZIK8	4565.A0A3B5ZTC4	4565.A0A3B6C5U6	0	0	0.127	0.616	0	0.699	0	0.151	0.757
TaMAPKKKK3	TaZIK5	4565.A0A3B5ZTC4	4565.A0A3B6DA61	0	0	0.131	0.619	0	0.699	0	0.151	0.758
TaMAPKKKK3	TaZIK10	4565.A0A3B5ZTC4	4565.A0A3B6NT46	0	0	0.134	0.57	0	0.699	0	0.151	0.759
TaMAPKKKK3	TaZIK2	4565.A0A3B5ZTC4	4565.A0A3B6QG64	0	0	0.135	0.57	0	0.699	0	0.151	0.759

TaMAPKKKK3	TaZIK7	4565.A0A3B5ZTC4	4565.A0A3B6C620	0	0	0.138	0.564	0	0.699	0	0.151	0.76
TaMAPKKKK3	TaZIK4	4565.A0A3B5ZTC4	4565.A0A3B6PML2	0	0	0.136	0.572	0	0.699	0	0.151	0.76
TaMAPKKKK3	TaZIK3	4565.A0A3B5ZTC4	4565.A0A3B6AUS6	0	0	0.139	0.564	0	0.699	0	0.151	0.761
TaMAPKKKK4	TaMEKK7	4565.A0A3B6AY64	4565.A0A077RUI2	0	0	0.143	0.623	0	0.175	0.685	0.151	0.785
TaMAPKKKK4	TaMEKK9	4565.A0A3B6AY64	4565.A0A077S2G5	0	0	0.13	0.633	0	0.175	0.685	0.151	0.782
TaMAPKKKK4	TaRaf22	4565.A0A3B6AY64	4565.A0A3B6I1C9	0	0	0	0.562	0	0.384	0	0.097	0.419
TaMAPKKKK4	TaRaf81	4565.A0A3B6AY64	4565.A0A3B6D624	0	0	0	0.557	0	0.384	0	0.097	0.419
TaMAPKKKK4	TaRaf94	4565.A0A3B6AY64	4565.A0A3B6N1G5	0	0	0	0.562	0	0.384	0	0.097	0.419
TaMAPKKKK4	TaRaf89	4565.A0A3B6AY64	4565.A0A3B6PRF9	0	0	0	0.552	0	0.384	0	0.097	0.419
TaMAPKKKK4	TaRaf111	4565.A0A3B6AY64	4565.A0A3B6FHS8	0	0	0	0	0	0.148	0.509	0	0.563
TaMAPKKKK4	TaMEKK20	4565.A0A3B6AY64	4565.A0A3B6B6T5	0	0	0	0.664	0	0.175	0.685	0.151	0.76
TaMAPKKKK4	TaMEKK18	4565.A0A3B6AY64	4565.A0A3B6CEA6	0	0	0	0.664	0	0.175	0.685	0.151	0.76
TaMAPKKKK4	TaMEKK4	4565.A0A3B6AY64	4565.A0A3B6NRN9	0	0	0.12	0.669	0	0.175	0.685	0.151	0.779
TaMAPKKKK4	TaMEKK4-1	4565.A0A3B6AY64	4565.A0A3B6PNI6	0	0	0.119	0.671	0	0.175	0.685	0.151	0.779
TaMAPKKKK4	TaMEKK1	4565.A0A3B6AY64	4565.A0A3B6B3I4	0	0	0.123	0.671	0	0.175	0.685	0.151	0.78
TaMAPKKKK4	TaMEKK8	4565.A0A3B6AY64	4565.A0A3B6FNU8	0	0	0.136	0.62	0	0.175	0.685	0.151	0.783
TaMAPKKKK4	TaMEKK17	4565.A0A3B6AY64	4565.A0A3B6KFL8	0	0	0.145	0.655	0	0.175	0.685	0.151	0.786
TaMAPKKKK4	TaMEKK29	4565.A0A3B6AY64	4565.A0A3B6MSP6	0	0	0.146	0.654	0	0.175	0.685	0.151	0.786

TaMAPKKKK4	TaMEKK24	4565.A0A3B6AY64	4565.A0A3B6LLV5	0	0	0.145	0.655	0	0.175	0.685	0.151	0.786
TaMAPKKKK5	TaMEKK7	4565.A0A3B6C4T6	4565.A0A077RUI2	0	0	0.143	0.623	0	0.175	0.685	0.151	0.785
TaMAPKKKK5	TaMEKK9	4565.A0A3B6C4T6	4565.A0A077S2G5	0	0	0.13	0.632	0	0.175	0.685	0.151	0.782
TaMAPKKKK5	TaMEKK1	4565.A0A3B6C4T6	4565.A0A3B6B3I4	0	0	0.123	0.67	0	0.175	0.685	0.151	0.78
TaMAPKKKK5	TaMEKK20	4565.A0A3B6C4T6	4565.A0A3B6B6T5	0	0	0	0.664	0	0.175	0.685	0.151	0.76
TaMAPKKKK5	TaRaf89	4565.A0A3B6C4T6	4565.A0A3B6PRF9	0	0	0	0.551	0	0.384	0	0.097	0.419
TaMAPKKKK5	TaRaf94	4565.A0A3B6C4T6	4565.A0A3B6N1G5	0	0	0	0.562	0	0.384	0	0.097	0.419
TaMAPKKKK5	TaRaf81	4565.A0A3B6C4T6	4565.A0A3B6D624	0	0	0	0.557	0	0.384	0	0.097	0.419
TaMAPKKKK5	TaRaf22	4565.A0A3B6C4T6	4565.A0A3B6I1C9	0	0	0	0.562	0	0.384	0	0.097	0.419
TaMAPKKKK5	TaRaf111	4565.A0A3B6C4T6	4565.A0A3B6FHS8	0	0	0	0	0	0.148	0.509	0	0.563
TaMAPKKKK5	TaMEKK18	4565.A0A3B6C4T6	4565.A0A3B6CEA6	0	0	0	0.664	0	0.175	0.685	0.151	0.76
TaMAPKKKK5	TaMEKK4	4565.A0A3B6C4T6	4565.A0A3B6NRN9	0	0	0.12	0.669	0	0.175	0.685	0.151	0.779
TaMAPKKKK5	TaMEKK4-1	4565.A0A3B6C4T6	4565.A0A3B6PNI6	0	0	0.119	0.67	0	0.175	0.685	0.151	0.779
TaMAPKKKK5	TaMEKK8	4565.A0A3B6C4T6	4565.A0A3B6FNU8	0	0	0.136	0.62	0	0.175	0.685	0.151	0.783
TaMAPKKKK5	TaMEKK17	4565.A0A3B6C4T6	4565.A0A3B6KFL8	0	0	0.144	0.655	0	0.175	0.685	0.151	0.785
TaMAPKKKK5	TaMEKK29	4565.A0A3B6C4T6	4565.A0A3B6MSP6	0	0	0.145	0.654	0	0.175	0.685	0.151	0.786
TaMAPKKKK5	TaMEKK24	4565.A0A3B6C4T6	4565.A0A3B6LLV5	0	0	0.144	0.655	0	0.175	0.685	0.151	0.786
TaMAPKKKK6	TaMEKK7	4565.A0A3B6DEP0	4565.A0A077RUI2	0	0	0.144	0.624	0	0.175	0.685	0.151	0.785

TaMAPKKKK6	TaMEKK9	4565.A0A3B6DEP0	4565.A0A077S2G5	0	0	0.131	0.633	0	0.175	0.685	0.151	0.782
TaMAPKKKK6	TaMEKK1	4565.A0A3B6DEP0	4565.A0A3B6B3I4	0	0	0.124	0.671	0	0.175	0.685	0.151	0.78
TaMAPKKKK6	TaMEKK20	4565.A0A3B6DEP0	4565.A0A3B6B6T5	0	0	0	0.664	0	0.175	0.685	0.151	0.76
TaMAPKKKK6	TaMEKK18	4565.A0A3B6DEP0	4565.A0A3B6CEA6	0	0	0	0.665	0	0.175	0.685	0.151	0.76
TaMAPKKKK6	TaRaf81	4565.A0A3B6DEP0	4565.A0A3B6D624	0	0	0	0.557	0	0.384	0	0.097	0.419
TaMAPKKKK6	TaRaf22	4565.A0A3B6DEP0	4565.A0A3B6I1C9	0	0	0	0.562	0	0.384	0	0.097	0.419
TaMAPKKKK6	TaRaf89	4565.A0A3B6DEP0	4565.A0A3B6PRF9	0	0	0	0.551	0	0.384	0	0.097	0.419
TaMAPKKKK6	TaRaf94	4565.A0A3B6DEP0	4565.A0A3B6N1G5	0	0	0	0.562	0	0.384	0	0.097	0.419
TaMAPKKKK6	TaRaf111	4565.A0A3B6DEP0	4565.A0A3B6FHS8	0	0	0	0	0	0.148	0.509	0	0.563
TaMAPKKKK6	TaMEKK4-1	4565.A0A3B6DEP0	4565.A0A3B6PNI6	0	0	0.12	0.671	0	0.175	0.685	0.151	0.779
TaMAPKKKK6	TaMEKK4	4565.A0A3B6DEP0	4565.A0A3B6NRN9	0	0	0.12	0.669	0	0.175	0.685	0.151	0.78
TaMAPKKKK6	TaMEKK8	4565.A0A3B6DEP0	4565.A0A3B6FNU8	0	0	0.136	0.62	0	0.175	0.685	0.151	0.784
TaMAPKKKK6	TaMEKK17	4565.A0A3B6DEP0	4565.A0A3B6KFL8	0	0	0.145	0.654	0	0.175	0.685	0.151	0.786
TaMAPKKKK6	TaMEKK24	4565.A0A3B6DEP0	4565.A0A3B6LLV5	0	0	0.145	0.655	0	0.175	0.685	0.151	0.786
TaMAPKKKK6	TaMEKK29	4565.A0A3B6DEP0	4565.A0A3B6MSP6	0	0	0.146	0.654	0	0.175	0.685	0.151	0.786
TaMAPKKKK7	TaZIK9	4565.A0A3B6IX47	4565.A0A3B5ZP32	0	0	0.134	0.569	0	0.699	0	0.151	0.759
TaMAPKKKK7	TaZIK3	4565.A0A3B6IX47	4565.A0A3B6AUS6	0	0	0.131	0.558	0	0.699	0	0.151	0.758
TaMAPKKKK7	TaZIK8	4565.A0A3B6IX47	4565.A0A3B6C5U6	0	0	0.123	0.578	0	0.699	0	0.151	0.756

TaMAPKKKK7	TaZIK7	4565.A0A3B6IX47	4565.A0A3B6C620	0	0	0.129	0.558	0	0.699	0	0.151	0.758
TaMAPKKKK7	TaZIK5	4565.A0A3B6IX47	4565.A0A3B6DA61	0	0	0.126	0.578	0	0.699	0	0.151	0.757
TaMAPKKKK7	TaZIK11	4565.A0A3B6IX47	4565.A0A3B6LI77	0	0	0	0.556	0	0.699	0	0.151	0.733
TaMAPKKKK7	TaZIK1	4565.A0A3B6IX47	4565.Q84XZ4	0	0	0	0.556	0	0.699	0	0.151	0.733
TaMAPKKKK7	TaZIK10	4565.A0A3B6IX47	4565.A0A3B6NT46	0	0	0.124	0.564	0	0.699	0	0.151	0.756
TaMAPKKKK7	TaZIK2	4565.A0A3B6IX47	4565.A0A3B6QG64	0	0	0.124	0.564	0	0.699	0	0.151	0.756
TaMAPKKKK7	TaZIK4	4565.A0A3B6IX47	4565.A0A3B6PML2	0	0	0.127	0.565	0	0.699	0	0.151	0.757
TaMAPKKKK8	TaZIK9	4565.A0A3B6J0T1	4565.A0A3B5ZP32	0	0	0.145	0.578	0	0.699	0	0.151	0.762
TaMAPKKKK8	TaZIK3	4565.A0A3B6J0T1	4565.A0A3B6AUS6	0	0	0.149	0.565	0	0.699	0	0.151	0.763
TaMAPKKKK8	TaZIK8	4565.A0A3B6J0T1	4565.A0A3B6C5U6	0	0	0.133	0.613	0	0.699	0	0.151	0.759
TaMAPKKKK8	TaZIK7	4565.A0A3B6J0T1	4565.A0A3B6C620	0	0	0.148	0.565	0	0.699	0	0.151	0.763
TaMAPKKKK8	TaZIK5	4565.A0A3B6J0T1	4565.A0A3B6DA61	0	0	0.137	0.622	0	0.699	0	0.151	0.76
TaMAPKKKK8	TaZIK1	4565.A0A3B6J0T1	4565.Q84XZ4	0	0	0.124	0.564	0	0.699	0	0.151	0.756
TaMAPKKKK8	TaZIK11	4565.A0A3B6J0T1	4565.A0A3B6LI77	0	0	0.124	0.564	0	0.699	0	0.151	0.756
TaMAPKKKK8	TaZIK10	4565.A0A3B6J0T1	4565.A0A3B6NT46	0	0	0.142	0.571	0	0.699	0	0.151	0.761
TaMAPKKKK8	TaZIK2	4565.A0A3B6J0T1	4565.A0A3B6QG64	0	0	0.143	0.571	0	0.699	0	0.151	0.761
TaMAPKKKK8	TaZIK4	4565.A0A3B6J0T1	4565.A0A3B6PML2	0	0	0.144	0.574	0	0.699	0	0.151	0.762
TaMAPKKKK9	TaZIK9	4565.A0A3B6KID5	4565.A0A3B5ZP32	0	0	0.11	0.572	0	0.699	0	0.151	0.752

TaMAPKKKK9	TaZIK3	4565.A0A3B6KID5	4565.A0A3B6AUS6	0	0	0.119	0.572	0	0.699	0	0.151	0.755
TaMAPKKKK9	TaZIK8	4565.A0A3B6KID5	4565.A0A3B6C5U6	0	0	0.112	0.596	0	0.699	0	0.151	0.753
TaMAPKKKK9	TaZIK7	4565.A0A3B6KID5	4565.A0A3B6C620	0	0	0.118	0.572	0	0.699	0	0.151	0.755
TaMAPKKKK9	TaZIK5	4565.A0A3B6KID5	4565.A0A3B6DA61	0	0	0.116	0.599	0	0.699	0	0.151	0.754
TaMAPKKKK9	TaZIK1	4565.A0A3B6KID5	4565.Q84XZ4	0	0	0	0.562	0	0.699	0	0.151	0.733
TaMAPKKKK9	TaZIK11	4565.A0A3B6KID5	4565.A0A3B6LI77	0	0	0	0.565	0	0.699	0	0.151	0.733
TaMAPKKKK9	TaZIK10	4565.A0A3B6KID5	4565.A0A3B6NT46	0	0	0.111	0.57	0	0.699	0	0.151	0.753
TaMAPKKKK9	TaZIK2	4565.A0A3B6KID5	4565.A0A3B6QG64	0	0	0.111	0.571	0	0.699	0	0.151	0.753
TaMAPKKKK9	TaZIK4	4565.A0A3B6KID5	4565.A0A3B6PML2	0	0	0.111	0.57	0	0.699	0	0.151	0.753
TaMEKK1	TaMEKK17	4565.A0A3B6B3I4	4565.A0A3B6KFL8	0	0	0.158	0.757	0	0	0.59	0	0.64
TaMEKK1	TaMEKK29	4565.A0A3B6B3I4	4565.A0A3B6MSP6	0	0	0.158	0.756	0	0	0.59	0	0.64
TaMEKK1	TaMEKK24	4565.A0A3B6B3I4	4565.A0A3B6LLV5	0	0	0.158	0.757	0	0	0.59	0	0.64
TaMEKK1	TaMEKK4-1	4565.A0A3B6B3I4	4565.A0A3B6PNI6	0	0	0.052	0.977	0	0	0.927	0	0.927
TaMEKK1	TaMEKK4	4565.A0A3B6B3I4	4565.A0A3B6NRN9	0	0	0.054	0.973	0	0	0.927	0	0.928
TaMEKK10	TaRaf18	4565.A0A3B6JM32	4565.A0A077RY41	0	0	0.197	0.61	0	0.133	0.52	0	0.636
TaMEKK10	TaRaf29	4565.A0A3B6JM32	4565.A0A1D5UHD7	0	0	0.168	0.597	0	0.133	0.52	0	0.623
TaMEKK10	TaRaf88	4565.A0A3B6JM32	4565.A0A3B5Z5X1	0	0	0.196	0.62	0	0.133	0.52	0	0.636
TaMEKK10	TaRaf30	4565.A0A3B6JM32	4565.A0A3B6A1Z4	0	0	0.194	0.617	0	0.133	0.52	0	0.635

TaMEKK10	TaRaf79	4565.A0A3B6JM32	4565.A0A3B6B9C7	0	0	0.165	0.607	0	0.133	0.52	0	0.622
TaMEKK10	TaRaf59	4565.A0A3B6JM32	4565.A0A3B6E9E2	0	0	0.144	0.654	0	0.133	0.52	0	0.612
TaMEKK10	TaRaf60	4565.A0A3B6JM32	4565.A0A3B6EHW0	0	0	0.207	0.574	0	0.133	0.52	0	0.641
TaMEKK10	TaRaf5	4565.A0A3B6JM32	4565.A0A3B6GYQ1	0	0	0.214	0.575	0	0.133	0.52	0	0.644
TaMEKK10	TaRaf100	4565.A0A3B6JM32	4565.A0A3B6ML06	0	0	0	0.573	0	0.133	0.52	0	0.566
TaMEKK10	TaRaf91	4565.A0A3B6JM32	4565.A0A3B6PMI5	0	0	0.13	0.575	0	0.133	0.52	0	0.606
TaMEKK10	TaRaf1	4565.A0A3B6JM32	4565.A0A3B6NP57	0	0	0.141	0.652	0	0.133	0.52	0	0.611
TaMEKK10	TaRaf52	4565.A0A3B6JM32	4565.A0A3B6LRR0	0	0	0.144	0.608	0	0.133	0.52	0	0.612
TaMEKK10	TaRaf21	4565.A0A3B6JM32	4565.A0A3B6TVC5	0	0	0.145	0.633	0	0.133	0.52	0	0.613
TaMEKK10	TaRaf63	4565.A0A3B6JM32	4565.A0A3B6RKW0	0	0	0.147	0.599	0	0.133	0.52	0	0.614
TaMEKK10	TaRaf58	4565.A0A3B6JM32	4565.A0A3B6N1Y2	0	0	0.16	0.608	0	0.133	0.52	0	0.62
TaMEKK10	TaRaf46	4565.A0A3B6JM32	4565.A0A3B6MW69	0	0	0.188	0.623	0	0.133	0.52	0	0.632
TaMEKK10	TaRaf62	4565.A0A3B6JM32	4565.A9RAA9	0	0	0.19	0.632	0	0.133	0.52	0	0.633
TaMEKK11	TaRaf41	4565.A0A3B6N0D8	4565.A0A3B6KLD7	0	0	0.305	0	0	0.114	0.109	0	0.403
TaMEKK12	TaRaf18	4565.A0A3B6HQC5	4565.A0A077RY41	0	0	0.196	0.61	0	0.133	0.52	0	0.636
TaMEKK12	TaRaf29	4565.A0A3B6HQC5	4565.A0A1D5UHD7	0	0	0.169	0.595	0	0.133	0.52	0	0.624
TaMEKK12	TaRaf88	4565.A0A3B6HQC5	4565.A0A3B5Z5X1	0	0	0.193	0.625	0	0.133	0.52	0	0.634
TaMEKK12	TaRaf30	4565.A0A3B6HQC5	4565.A0A3B6A1Z4	0	0	0.193	0.618	0	0.133	0.52	0	0.635

TaMEKK12	TaRaf79	4565.A0A3B6HQC5	4565.A0A3B6B9C7	0	0	0.165	0.606	0	0.133	0.52	0	0.622
TaMEKK12	TaRaf59	4565.A0A3B6HQC5	4565.A0A3B6E9E2	0	0	0.144	0.655	0	0.133	0.52	0	0.612
TaMEKK12	TaRaf60	4565.A0A3B6HQC5	4565.A0A3B6EHW0	0	0	0.208	0.575	0	0.133	0.52	0	0.641
TaMEKK12	TaRaf5	4565.A0A3B6HQC5	4565.A0A3B6GYQ1	0	0	0.214	0.576	0	0.133	0.52	0	0.644
TaMEKK12	TaRaf100	4565.A0A3B6HQC5	4565.A0A3B6ML06	0	0	0	0.573	0	0.133	0.52	0	0.566
TaMEKK12	TaRaf91	4565.A0A3B6HQC5	4565.A0A3B6PMI5	0	0	0.128	0.573	0	0.133	0.52	0	0.605
TaMEKK12	TaRaf1	4565.A0A3B6HQC5	4565.A0A3B6NP57	0	0	0.139	0.654	0	0.133	0.52	0	0.61
TaMEKK12	TaRaf21	4565.A0A3B6HQC5	4565.A0A3B6TVC5	0	0	0.144	0.633	0	0.133	0.52	0	0.612
TaMEKK12	TaRaf52	4565.A0A3B6HQC5	4565.A0A3B6LRR0	0	0	0.146	0.607	0	0.133	0.52	0	0.613
TaMEKK12	TaRaf63	4565.A0A3B6HQC5	4565.A0A3B6RKW0	0	0	0.147	0.599	0	0.133	0.52	0	0.614
TaMEKK12	TaRaf58	4565.A0A3B6HQC5	4565.A0A3B6N1Y2	0	0	0.159	0.608	0	0.133	0.52	0	0.619
TaMEKK12	TaRaf46	4565.A0A3B6HQC5	4565.A0A3B6MW69	0	0	0.186	0.624	0	0.133	0.52	0	0.632
TaMEKK12	TaRaf62	4565.A0A3B6HQC5	4565.A9RAA9	0	0	0.189	0.632	0	0.133	0.52	0	0.633
TaMEKK15	TaRaf41	4565.A0A3B6KPK7	4565.A0A3B6KLD7	0	0	0.307	0	0	0.114	0.109	0	0.405
TaMEKK16	TaRaf41	4565.A0A3B6LW00	4565.A0A3B6KLD7	0	0	0.312	0	0	0.114	0.109	0	0.409
TaMEKK16	TaRaf7	4565.A0A3B6LW00	4565.A0A3B6TBH5	0	0	0.303	0	0	0.114	0.109	0	0.401
TaMEKK17	TaMEKK4-1	4565.A0A3B6KFL8	4565.A0A3B6PNI6	0	0	0.158	0.756	0	0	0.59	0	0.64
TaMEKK17	TaMEKK4	4565.A0A3B6KFL8	4565.A0A3B6NRN9	0	0	0.16	0.753	0	0	0.59	0	0.641

TaMEKK24	TaMEKK4-1	4565.A0A3B6LLV5	4565.A0A3B6PNI6	0	0	0.158	0.756	0	0	0.59	0	0.64
TaMEKK24	TaMEKK4	4565.A0A3B6LLV5	4565.A0A3B6NRN9	0	0	0.16	0.753	0	0	0.59	0	0.641
TaMEKK25	TaZIK9	4565.A0A3B6LLP7	4565.A0A3B5ZP32	0	0	0.112	0.572	0	0.699	0	0.151	0.753
TaMEKK25	TaZIK3	4565.A0A3B6LLP7	4565.A0A3B6AUS6	0	0	0.123	0.571	0	0.699	0	0.151	0.756
TaMEKK25	TaZIK8	4565.A0A3B6LLP7	4565.A0A3B6C5U6	0	0	0.115	0.598	0	0.699	0	0.151	0.754
TaMEKK25	TaZIK7	4565.A0A3B6LLP7	4565.A0A3B6C620	0	0	0.122	0.571	0	0.699	0	0.151	0.756
TaMEKK25	TaZIK5	4565.A0A3B6LLP7	4565.A0A3B6DA61	0	0	0.12	0.6	0	0.699	0	0.151	0.755
TaMEKK25	TaZIK11	4565.A0A3B6LLP7	4565.A0A3B6LI77	0	0	0	0.564	0	0.699	0	0.151	0.733
TaMEKK25	TaZIK1	4565.A0A3B6LLP7	4565.Q84XZ4	0	0	0	0.561	0	0.699	0	0.151	0.733
TaMEKK25	TaZIK4	4565.A0A3B6LLP7	4565.A0A3B6PML2	0	0	0.114	0.57	0	0.699	0	0.151	0.753
TaMEKK25	TaZIK2	4565.A0A3B6LLP7	4565.A0A3B6QG64	0	0	0.114	0.57	0	0.699	0	0.151	0.753
TaMEKK25	TaZIK10	4565.A0A3B6LLP7	4565.A0A3B6NT46	0	0	0.114	0.569	0	0.699	0	0.151	0.753
TaMEKK29	TaMEKK4-1	4565.A0A3B6MSP6	4565.A0A3B6PNI6	0	0	0.159	0.755	0	0	0.59	0	0.64
TaMEKK29	TaMEKK4	4565.A0A3B6MSP6	4565.A0A3B6NRN9	0	0	0.16	0.752	0	0	0.59	0	0.641
TaMEKK3	TaRaf18	4565.A0A3B6ISF1	4565.A0A077RY41	0	0	0.196	0.61	0	0.133	0.52	0	0.636
TaMEKK3	TaRaf29	4565.A0A3B6ISF1	4565.A0A1D5UHD7	0	0	0.17	0.596	0	0.133	0.52	0	0.624
TaMEKK3	TaRaf88	4565.A0A3B6ISF1	4565.A0A3B5Z5X1	0	0	0.194	0.62	0	0.133	0.52	0	0.635
TaMEKK3	TaRaf30	4565.A0A3B6ISF1	4565.A0A3B6A1Z4	0	0	0.193	0.617	0	0.133	0.52	0	0.635

TaMEKK3	TaRaf79	4565.A0A3 B6ISF1	4565.A0A3 B6B9C7	0	0	0.166	0.607	0	0.133	0.52	0	0.622
TaMEKK3	TaRaf59	4565.A0A3 B6ISF1	4565.A0A3 B6E9E2	0	0	0.144	0.654	0	0.133	0.52	0	0.612
TaMEKK3	TaRaf60	4565.A0A3 B6ISF1	4565.A0A3 B6EHW0	0	0	0.207	0.574	0	0.133	0.52	0	0.641
TaMEKK3	TaRaf5	4565.A0A3 B6ISF1	4565.A0A3 B6GYQ1	0	0	0.214	0.575	0	0.133	0.52	0	0.644
TaMEKK3	TaRaf100	4565.A0A3 B6ISF1	4565.A0A3 B6ML06	0	0	0	0.573	0	0.133	0.52	0	0.566
TaMEKK3	TaRaf91	4565.A0A3 B6ISF1	4565.A0A3 B6PMI5	0	0	0.127	0.575	0	0.133	0.52	0	0.605
TaMEKK3	TaRaf1	4565.A0A3 B6ISF1	4565.A0A3 B6NP57	0	0	0.14	0.652	0	0.133	0.52	0	0.611
TaMEKK3	TaRaf21	4565.A0A3 B6ISF1	4565.A0A3 B6TVC5	0	0	0.144	0.632	0	0.133	0.52	0	0.612
TaMEKK3	TaRaf52	4565.A0A3 B6ISF1	4565.A0A3 B6LRR0	0	0	0.146	0.607	0	0.133	0.52	0	0.613
TaMEKK3	TaRaf63	4565.A0A3 B6ISF1	4565.A0A3 B6RKW0	0	0	0.148	0.599	0	0.133	0.52	0	0.614
TaMEKK3	TaRaf58	4565.A0A3 B6ISF1	4565.A0A3 B6N1Y2	0	0	0.161	0.606	0	0.133	0.52	0	0.62
TaMEKK3	TaRaf46	4565.A0A3 B6ISF1	4565.A0A3 B6MW69	0	0	0.187	0.623	0	0.133	0.52	0	0.632
TaMEKK3	TaRaf62	4565.A0A3 B6ISF1	4565.A9RA A9	0	0	0.19	0.632	0	0.133	0.52	0	0.633
TaMEKK5	TaRaf41	4565.A0A3 B6AWC1	4565.A0A3 B6KLD7	0	0	0.309	0	0	0.114	0.109	0	0.406
TaMEKK9	TaRaf14	4565.A0A0 77S2G5	4565.A0A3 B6TYA5	0	0	0.289	0	0	0.139	0.137	0	0.425
TaRaf114	TaRaf51	4565.A0A3 B6FV36	4565.A0A3 B6JHE9	0	0	0.084	0.911	0	0	0.54	0	0.56
