# Supplementary material for: Bioinformatic Identification and Expression Analyses of the MAPK–MAP4K Gene Family Reveal a Putative Functional MAP4K10-MAP3K7/8-MAP2K1/11-MAPK3/6 Cascade in Wheat (Triticum aestivum L.)
Source: Plants (Basel). 2024 Mar 24;13(7):941. doi: 10.3390/plants13070941 (PMC11013086; doi:10.3390/plants13070941)
Supplement: Supplementary file 1 [file plants-13-00941-s001.zip › plants-2867660-supplementary/Supplementary Figure S1-S10 and Supplementary table S1-S7/Supplementary table S1-S7/Supplementary table 6.pdf]

Table S5 The expression profile data of MAPK-MAP4Ks.

gene id	gene	6dhs2	6dhs1	1dhs2	1dhs1	6h2	6h1	1h2	1h1	6d2	6d1	1d2	1d1	c2	c1
**TraesCS6B02G296700.2**	**TaMAPK1**	4.23195	5.5986	2.93146	3.00281	5.36652	4.37479	2.30859	2.31239	5.12847	5.54706	8.79167	6.85185	5.99579	9.1542
**TraesCS4A02G336800.2**	**TaMAPK2**	0	2.26E-07	0	0.27777	0	0.12886	0.01781	0	2.06E-09	0	0	0	0	0
**TraesCS4A02G106400.1**	**TaMAPK3**	7.52281	6.79529	3.42336	4.91829	9.40216	9.57002	6.72631	4.25848	4.82013	4.49729	38.1351	29.3051	9.64112	12.2491
**TraesCS1D02G088000.2**	**TaMAPK4**	0	0	0	0	0	0	0	0	0	0	0.13285	0.1911	0	0
**TraesCS1D02G422800.1**	**TaMAPK5**	0.0481	0.05089	0	0	0	0.05709	0	0.03243	0.16597	0	0.26064	0.22346	0.24746	0.3424
**TraesCS7B02G009200.1**	**TaMAPK6**	7.21125	7.3137	1.58199	1.80781	8.30898	7.67016	3.06793	2.82839	4.9529	5.96049	5.77572	4.01002	4.51255	6.29247
**TraesCS7D02G342800.3**	**TaMAPK7**	4.78129	4.88311	1.94705	1.93348	5.02468	5.38939	1.6256	1.35235	3.68614	3.54684	3.37577	2.79714	2.71373	3.87964
**TraesCS3D02G225600.1**	**TaMAPK8**	4.44048	4.40988	1.41612	1.83542	10.9193	10.5782	2.10702	1.91478	1.94834	2.12059	2.47205	2.0767	2.18523	2.66536
**TraesCS6B02G146300.1**	**TaMAPK10**	12.4553	15.1263	5.52391	5.2575	15.6588	18.4475	6.98601	5.73359	7.32178	7.40639	11.2116	8.72001	6.98657	8.45537
**TraesCS1A02G086500.1**	**TaMAPK11**	1.58332	1.6488	1.01691	0.8972	2.1937	2.19314	1.36336	1.3095	2.45412	2.63541	3.22123	2.34123	1.89717	2.70034
**TraesCS7A02G422500.1**	**TaMAPK12**	0.52592	0.81962	1.46766	1.59519	0.78016	0.79401	3.54849	2.7636	11.4136	10.0916	16.2846	13.4388	6.91316	7.42971

**TraesCS6D02G245500.3**	**TaMAPK13**	7.59712	9.19514	1.88416	3.04018	6.11007	7.97333	2.14702	3.85856	4.4303	6.1805	8.03121	4.39587	5.23465	7.81215
**TraesCS1A02G184500.1**	**TaMAPK14**	6.18036	6.37175	3.25418	3.24831	8.01795	8.41098	5.21467	4.1883	4.3158	4.14187	9.98283	8.05199	6.19477	8.4653
**TraesCS3B02G270200.1**	**TaMAPK16**	2.93462	3.13547	1.77221	1.82908	3.33936	2.81853	3.48574	2.76808	6.91275	6.74878	9.44762	6.92497	9.0083	12.6339
**TraesCS6B02G127800.1**	**TaMAPK17**	0	0	0	0.01161	0	0.00885	0	0	0	0	0	0	0.00811	0
**TraesCS7D02G044100.1**	**TaMAPK18**	36.7621	37.6997	15.3365	17.6187	34.153	33.9526	12.8063	8.85031	34.3126	32.708	38.7491	31.8673	24.3932	31.5904
**TraesCS7A02G111300.1**	**TaMAPK19**	0.48408	0.6424	0.35618	0.17223	0.71562	0.70617	0.4959	0.24626	0.52304	0.46932	0.49554	0.39186	0.58151	0.79753
**TraesCS7D02G403700.1**	**TaMAPK20**	0	0	0.13939	0.46897	0.38733	0	0	0.61179	0.19132	0	0.32293	0	0	0.3691
**TraesCS7D02G414900.1**	**TaMAPK22**	0.1314	0.10205	0.01654	0.0328	0.10429	0.05069	0.03603	0.08189	0.97597	0.92162	0.27566	0.27698	0.07183	0.08988
**TraesCS3D02G221700.1**	**TaMAPK23**	0.81414	0.8702	0.58578	0.67828	1.1271	1.12849	1.27288	1.10799	0.9085	0.7681	1.23184	1.00786	0.98504	1.40208
**TraesCS3D02G242200.2**	**TaMAPK24**	5.08755	5.80447	2.66261	2.35555	5.0612	4.97478	3.59982	2.90991	7.53011	7.29655	9.68926	6.869	9.39714	13.1337
**TraesCS4D02G198600.1**	**TaMAPK25**	9.30833	9.7448	6.26549	7.6517	15.8908	16.6113	14.0556	10.2497	13.1424	11.4694	58.814	46.7737	13.816	19.2555
**TraesCS5D02G534000.2**	**TaMAPK26**	0.07056	0	0	0.20026	0	0	0	0.28926	0.15063	0.16221	0.73291	0.20936	0.24837	0.09393

**TraesCS1B02G104900.1**	**TaMAPK27**	3.21833	3.84264	1.95737	1.49807	3.99496	4.00491	3.17776	2.9718	5.65883	6.03874	9.2461	6.91724	6.05644	7.99628
**TraesCS1B02G192600.3**	**TaMAPK28**	19.2486	18.8315	8.40185	10.846	22.6783	24.749	14.3783	11.2447	9.57706	9.6599	24.4988	20.829	14.5758	20.1423
**TraesCS1B02G431400.2**	**TaMAPK29**	0	0	0	0	0	0	0	0	0	0	0	0	0	0
**TraesCS7A02G335300.2**	**TaMAPK30**	1.37221	2.07075	0.65552	0.67418	1.18988	0.47827	0.46454	0.81131	0.28059	0	0.42468	0.44881	0.077	0.54622
**TraesCS4A02G434800.1**	**TaMAPK31**	32.9484	34.1062	16.0239	17.8452	44.5389	43.5812	14.1792	11.2272	43.0727	41.3292	55.0736	40.2052	33.971	47.2982
**TraesCS1D02G410100.1**	**TaMAPK33**	0.95091	1.11258	4.14187	4.7573	3.26301	3.07644	4.68439	3.38876	0.80345	0.76813	3.65716	2.91019	3.91623	5.25675
**TraesCS1D02G428900.1**	**TaMAPK34**	1.89667	1.69602	1.50406	1.84487	2.57524	2.70178	1.58173	1.06395	1.50273	1.50103	1.8226	1.443	1.42618	1.70925
**TraesCS6A02G099600.1**	**TaMAPK35**	0.51874	0.53596	0.3101	0.23908	0.78886	0.75821	0.09613	0.14483	0.36408	0.37313	0.37118	0.15077	0.2648	0.35862
**TraesCS6A02G118100.1**	**TaMAPK36**	1.13388	0.45369	0.21184	0.88075	0.50791	0.58227	0.52206	0.18516	0	0.10854	0.14207	0.45025	0.07588	0.13511
**TraesCS6A02G269400.1**	**TaMAPK37**	3.52737	3.98609	2.11914	2.23084	2.65294	2.47348	3.02621	2.4121	3.30345	3.49093	6.56911	4.81278	4.3483	6.05093
**TraesCS3B02G256700.1**	**TaMAPK38**	4.6966	5.38759	2.32133	2.04594	6.49266	6.918	1.29142	1.6659	1.89907	1.97591	2.38167	1.71959	2.16352	3.31616
**TraesCS3A02G242100.1**	**TaMAPK39**	12.4671	13.8946	8.88259	9.24868	9.25483	8.69729	7.64156	6.71677	20.7972	21.3127	16.2988	11.7206	9.24374	13.7131

**TraesCS3A02G231700.1**	**TaMAPK40**	0.3752	0.33016	0.3027	0.2201	0.42919	0.52147	0.31193	0.29772	0.32683	0.39243	0.37615	0.40505	0.3795	0.68424
**TraesCS1A02G402400.2**	**TaMAPK41**	0.40289	0	0	0	0	0	0	0	0	0	0.11868	0	0	0
**TraesCS1A02G415300.1**	**TaMAPK42**	0.07339	0	0	0	0.03259	0.0922	0	0.02428	0.1432	0.20452	0.17696	0.25845	0.15821	0.17444
**TraesCS1A02G421000.1**	**TaMAPK43**	0.82996	0.77403	0.95563	1.11445	1.76808	1.58475	1.01659	0.94738	2.261	2.18709	2.27788	1.79634	1.4595	1.77985
**TraesCS6D02G082900.2**	**TaMAPK44**	0.16112	0.07454	0.02309	0	0.1212	0.12676	0.02092	0.03161	0.037	0.05628	0.18376	0.03995	0.10932	0.26929
**TraesCS6D02G108100.1**	**TaMAPK45**	14.804	14.7754	5.92981	6.45907	19.5433	21.8583	7.97544	5.94758	8.45869	7.37898	11.8397	10.0048	7.62172	8.83977
**TraesCS7A02G049000.1**	**TaMAPK46**	0.18617	0.46169	0	0	0.46561	0.22121	0	0	0.22833	0	0	0	0	0.13147
**TraesCS7A02G029700.1**	**TaMAPK47**	0.04989	0	0	0	0.0222	0.09747	0	0.02476	0.0198	0.04476	0	0.02119	0.0344	0
**TraesCS7A02G410700.2**	**TaMAPK49**	4.26898	4.2015	12.033	10.45	10.745	8.19399	14.576	7.96733	3.77313	4.13073	21.7066	18.0642	9.66043	9.25449
**TraesCS5B02G536500.1**	**TaMAPK50**	2.80573	2.792	2.17274	1.96763	2.60119	2.58541	2.89455	2.29606	2.86623	2.99652	5.04365	4.16383	3.92986	5.4127
**TraesCS7B02G246900.3**	**TaMAPK52**	4.23252	4.57851	1.40634	1.29474	5.20338	5.30493	0.98536	0.97205	4.9182	4.74551	3.72055	2.37989	2.83742	4.16063
**TraesCS7B02G309900.1**	**TaMAPK53**	2.85226	2.62992	9.29011	9.58135	4.24272	4.04917	8.7766	7.1969	5.81906	5.4976	42.0679	32.0539	9.18617	13.6186

**TraesCS7B02G322900.1**	**TaMAPK54**	0.87103	0.97932	0.90343	1.01354	0.91602	1.00237	2.29356	1.97854	17.2182	16.582	15.6355	11.9364	4.90468	5.09804
**TraesCS6D02G328800.1**	**TaMAPKK1**	0.26629	0.34945	0.22523	0.33555	0.6079	0.757	1.41682	0.79399	1.2526	1.02375	4.59049	3.55217	1.87216	1.96624
**TraesCS5B02G565100.3**	**TaMAPKK2**	0.84445	0.50026	0.3594	0.25633	0.57414	0.84319	0.12836	0.29566	0.20103	0.18114	0.39747	0.30431	0.27313	0.45475
**TraesCS5D02G130900.2**	**TaMAPKK3**	0.1215	0.22894	0	0.06634	0.42473	0.31952	0.18507	0	0.093	0	0.043	0.13559	0.04376	0.23045
**TraesCS5A02G122700.4**	**TaMAPKK4**	0	0.18777	0	0	0.05426	0.3629	0	0	0	0	0.24274	0	0	0
**TraesCS4B02G049000.1**	**TaMAPKK5**	0	0	0	0	0	0	0	0	0	0	0	0	0	0
**TraesCS4B02G048100.1**	**TaMAPKK6**	0	0	0	0	0	0	0	0	0	0	0	0	0	0
**TraesCS4B02G048600.1**	**TaMAPKK7**	0	0	0	0	0	0	0	0	0	0	0	0	0	0
**TraesCS4B02G048900.1**	**TaMAPKK8**	0	0	0	0	0.02453	0	0	0	0	0	0	0	0	0
**TraesCS3B02G066300.1**	**TaMAPKK9**	0	0	0	0	0	0	0	0	0	0	0	0	0	0
**TraesCS4D02G048800.1**	**TaMAPKK11**	0	0	0	0	0	0	0	0	0	0	0	0.02351	0	0.01796
**TraesCS4D02G048500.1**	**TaMAPKK12**	0	0	0	0	0	0	0	0	0	0	0	0	0	0

**TraesCS5D02G549600.1**	**TaMAPKK13**	1.4285	1.08623	0.43273	0.57456	1.15162	1.15724	0.39329	0.2215	1.12809	0.97402	0.91868	0.67468	0.6426	0.85863
**TraesCS4A02G265900.1**	**TaMAPKK14**	0	0	0	0	0	0	0	0	0	0	0	0.04644	0	0
**TraesCS4A02G266000.1**	**TaMAPKK15**	0	0.02358	0.02297	0	0.04079	0.05359	0	0	0	0	0.08348	0.03881	0.0317	0.07495
**TraesCS4A02G266100.1**	**TaMAPKK16**	0	0.01679	0	0	0.02916	0.01268	0.01759	0.01592	0.01275	0	0.03563	0.01373	0.03437	0.06468
**TraesCS4A02G266200.1**	**TaMAPKK17**	0	0	0	0	0	0	0	0	0	0	0	0	0	0
**TraesCS5B02G122600.1**	**TaMAPKK18**	0.22857	0.38865	0.5181	0.20987	0.25588	0.35272	0.26193	0.09163	0.51424	0.26547	0	0.15297	0.09215	0.18051
**TraesCS2A02G407600.1**	**TaMAPKKK1**	2.95166	3.1499	0.70939	0.57702	3.48565	3.31548	0.79137	0.84885	1.4631	1.26065	2.27143	1.25669	1.36714	1.97031
**TraesCS4D02G027600.1**	**TaMAPKKK2**	0.00846	0.03622	0.22378	0.26093	0.03094	0.04001	0.11175	0.13909	0.02661	0.06112	0.18549	0.26641	0.1254	0.1958
**TraesCS4B02G210600.2**	**TaMAPKKK3**	2.80564	3.22986	1.11361	1.39338	2.62476	2.77854	0.89878	1.26365	5.03704	5.54443	4.73014	3.42137	2.59243	3.55501
**TraesCS6A02G245000.3**	**TaMAPKKK4**	0.62307	0.59135	0.371	0.41442	0.99573	0.86181	0.80747	0.62668	0.93455	0.98732	1.19488	0.90388	0.97676	1.33874
**TraesCS6B02G279300.1**	**TaMAPKKK4-1**	1.48528	1.47287	0.76028	0.76786	1.64736	2.04093	0.85856	0.76733	1.12824	1.07014	1.348	1.11436	1.0329	1.56196
**TraesCS2A02G199700.1**	**TaMAPKKK5**	2.34899	2.94583	3.48032	3.38813	7.70029	6.9214	4.46107	3.62434	1.32672	1.04253	12.3163	9.48559	3.73646	4.85665

**TraesCS3B02G289500.1**	**TaMAPKKK7**	0.0148	0.03083	2.21757	2.90414	0.05356	0.11627	3.56013	2.56524	0.51389	0.43997	0.25063	0.13847	0.06329	0.03965
**TraesCS3B02G288100.1**	**TaMAPKKK8**	0	0	0.04787	0.0656	0.07124	0.04954	0.49815	0.12436	0.08717	0.0142	0.04641	0.08048	0.05603	0.07377
**TraesCS3B02G288300.1**	**TaMAPKKK9**	0	0	0	0.0257	0.02173	0	0.0267	0.03229	0	0.02189	0.03562	0.06217	0	0.03735
**TraesCS4D02G211300.2**	**TaMAPKKK10**	2.11936	2.26382	0.84054	0.13872	2.41683	2.72473	1.10759	0.88326	3.8288	3.01133	3.39022	2.62115	1.51695	2.62477
**TraesCS5D02G475900.1**	**TaMAPKKK11**	12.5603	13.2346	22.68	23.35	19.0274	18.6883	21.8191	18.3273	6.28282	6.55711	10.7954	9.12894	3.40582	5.27303
**TraesCS4A02G093800.2**	**TaMAPKKK12**	7.25974	6.98022	1.38128	1.38264	6.00668	6.4917	1.86018	1.80312	4.51145	4.71248	4.32523	3.33251	4.01453	5.64931
**TraesCS5A02G118200.1**	**TaMAPKKK14**	0	0	0	0	0	0	0	0	0	0	0	0	0	0
**TraesCS5A02G463100.2**	**TaMAPKKK15**	2.14226	2.21302	2.81747	3.42899	3.91184	2.7717	3.21233	2.48363	1.31363	1.24381	3.68881	3.00342	1.48382	2.04732
**TraesCS5B02G474500.1**	**TaMAPKKK16**	7.3675	8.13186	19.216	21.471	12.8103	12.6426	16.2963	12.9086	10.2903	10.4042	9.79586	7.6715	3.19681	4.18913
**TraesCS5A02G200800.1**	**TaMAPKKK17**	0.016	0.01814	0	0	0.03088	0	9.40E-11	3.90E-10	0	0.05912	0	0	0	0
**TraesCS2B02G526200.3**	**TaMAPKKK18**	0	0.34004	0.19468	0.09461	0.10172	0.19012	0	0	0.20953	0.29801	0.16943	0.34713	0.26679	0.08739
**TraesCS2A02G498000.3**	**TaMAPKKK20**	1.08719	1.00178	0.78158	0.89191	0.73398	0.93592	0.6472	0.98187	0.68028	0.62625	3.08488	2.43553	1.72868	2.2504

**TraesCS6A02G149900.1**	**TaMAPKKK21**	0.16657	0.04676	0	0.02292	0.08994	0	0.07235	0.08722	0.58202	0.52172	0.39441	0.32351	0.25506	0.28667
**TraesCS5A02G392500.1**	**TaMAPKKK22**	0.7601	0.58024	0.29207	0.30806	0.64475	0.66001	0.43531	0.3089	0.76629	0.69898	1.11569	1.01564	0.88683	0.93256
**TraesCS6D02G139200.1**	**TaMAPKKK23**	0.02584	0	0	0	0.01172	0	0	0.0127	0.02904	0.08819	0.10594	0.04777	0.04632	0.02176
**TraesCS5B02G199400.1**	**TaMAPKKK24**	0	0	0	0	0	0	0	0	0.00929	9.43E-11	0.03976	0.04302	0	0
**TraesCS5B02G196400.1**	**TaMAPKKK25**	1.22451	0.99063	0.25574	0.37532	1.36751	1.25358	0.15854	0.09436	0.58637	0.5304	0.98308	0.92545	0.49395	0.74495
**TraesCS2D02G093700.1**	**TaMAPKKK26**	0.20579	0.31203	0.02693	0.0371	0.11214	0.10852	0.02234	0.03526	0.07591	0.04805	0.05386	0.05976	0.07859	0.08197
**TraesCS2B02G110500.1**	**TaMAPKKK27**	0.2436	0.29044	0.14075	0.09415	0.43193	0.28081	0.15672	0.17304	0.19477	0.25345	0.23275	0.18613	0.19664	0.32167
**TraesCS2A02G095300.1**	**TaMAPKKK28**	0.07039	0.06707	0.02701	0.0424	0.05522	0.05018	0.09653	0.06647	0.12912	0.10616	0.0677	0.12448	0.14254	0.15332
**TraesCS5D02G206500.1**	**TaMAPKKK29**	0	0	0	0	0	0	0	0	0	0	0	0	0	0
**TraesCS5D02G145100.1**	**TaMAPKKK30**	85.6204	68.9509	31.2419	37.3132	88.8787	89.7952	35.8657	20.8951	35.8742	32.4089	21.924	17.2075	15.0639	20.7541
**TraesCS6D02G236400.1**	**TaMAPKKK31**	0.11676	0.06122	0.07636	0.01294	0.01672	0.06066	0.08136	0.09803	0.31347	0.28367	0.39927	0.27528	0.33714	0.4655
**TraesCS2A02G195900.2**	**TaMAPKKK32**	2.52344	2.52717	1.29054	1.43314	3.07368	2.74186	3.38007	2.28611	2.52392	2.40763	4.76928	4.16436	4.90255	5.94954

**TraesCS6B02G270400.1**	**TaMAPKKK33**	0.14262	0.22873	0.0512	0.06036	0.21473	0.16835	0.05457	0.00822	0.07381	0.0606	0.11178	0.05711	0.15631	0.13547
**TraesCS2D02G197600.1**	**TaMAPKKK34**	1.0691	0.55699	4.52867	7.0015	2.02996	2.00328	8.66455	5.78854	3.60931	3.23168	8.18385	7.30094	0.12836	0.20744
**TraesCS2B02G223600.1**	**TaMAPKKK36**	3.66219	3.74196	1.15269	1.19271	3.4584	3.36586	2.83179	2.17899	7.15687	6.34714	5.75296	4.04936	4.22243	6.01412
**TraesCS2B02G216800.1**	**TaMAPKKK37**	3.31017	2.04273	29.4994	42.3796	2.67358	2.59689	30.1557	14.7691	3.4181	3.39264	22.4231	19.0127	0.30991	0.36422
**TraesCS1D02G026200.2**	**TaMAPKKK38**	0.7568	0.11732	0	0	0.4242	0.17879	0.19019	0.32198	0.26463	0.31866	0.38902	0.11016	0.31533	0.08102
**TraesCS6A02G255100.2**	**TaMAPKKK39**	0.09268	0.01242	0.00982	0.00185	0.00304	0.03523	0.03653	0.04127	0.07949	0.01076	0.07685	0.0732	0.07912	0.15078
**TraesCS5B02G146100.1**	**TaMAPKKK40**	10.8564	9.25424	5.76834	6.78848	12.7382	11.6772	9.94851	6.10198	10.7125	10.3458	10.6998	8.09682	8.06928	10.3538
**TraesCS6A02G172600.1**	**TaMAPKKK41**	0.10754	0.1548	0.67831	0.47399	0.13243	0.13891	1.06503	0.2204	0.02136	0	0.05878	0.02855	0.03684	0
**TraesCSU02G203100.1**	**TaMAPKKK42**	0	0	0	0	0	0	0	0	0	0	0	0	0	0
**TraesCS3B02G110300.1**	**TaMAPKKK43**	0.23945	0.06976	0.03411	0	0	0.05946	0	0.03421	0.05152	0	0.1149	0.10655	0.08702	0.14615
**TraesCS2D02G003900.1**	**TaMAPKKK44**	0	0	0	0	0	0	0	0	0	0	0	0	0	0
**TraesCS3D02G273200.1**	**TaMAPKKK45**	2.43647	2.1997	3.34843	3.61467	5.50032	5.34547	9.57479	8.07539	1.38536	1.37893	2.21519	1.62491	0.73621	0.93203

**TraesCS 2D02G0 50700.1**	**TaMAP KKK46**	0.01557	0.02451	0.09509	0.12792	0.05699	0.0982	0.17717	0.09911	0.08567	0.07032	0.42092	0.35779	0.37492	0.34503
**TraesCS 7D02G0 22200.1**	**TaMAP KKK47**	0	0	0	0	0	0	0	0	0	0	0	0	0	0
**TraesCS 7D02G0 79100.1**	**TaMAP KKK48**	0.23672	0.15849	0	0.06893	0.19647	0.11903	0.04342	0.02618	0.10239	0.16278	0.31778	0.15187	0.2116	0.25256
**TraesCS 7D02G0 99200.2**	**TaMAP KKK50**	4.10313	3.70457	0.40353	0.43919	6.3839	6.80607	0.62369	0.67724	1.07243	0.97957	9.34686	5.99207	7.28494	10.906
**TraesCS 7D02G2 30200.1**	**TaMAP KKK51**	0.4365	0.35632	0.12128	0.12193	0.76423	0.73428	0.11654	0.06944	0.52448	0.48268	0.28417	0.29488	0.12011	0.12142
**TraesCS 7D02G2 30500.1**	**TaMAP KKK52**	0.46321	0.44993	0.07811	0.09627	0.41862	0.47283	0.1681	0.07572	0.07312	0.06952	0.28402	0.22322	0.26352	0.22714
**TraesCS 1B02G3 72400.1**	**TaMAP KKK53**	0.87391	1.06556	0.39259	0.47703	3.52223	3.58072	0.58016	0.47799	0.50742	0.59013	0.4839	0.40199	0.49959	0.5098
**TraesCS 7D02G5 03600.1**	**TaMAP KKK54**	0	0	0	0	0	0	0	0.00753	0	0	0.0114	0.0131	0.01123	0
**TraesCS 3A02G0 39100.1**	**TaMAP KKK56**	0.13957	0.10169	0	0.02825	0.24328	0.38574	0.15415	0.1073	0.24977	0.30524	1.83748	1.33946	0.64881	0.69696
**TraesCS 3D02G0 40600.1**	**TaMAP KKK57**	0	0	0	0	0	0	0	0	0	0	0	0	0	0
**TraesCS 3B02G2 59800.1**	**TaMAP KKK58**	0.97491	0.92203	0.32781	0.53834	1.48314	1.73635	0.47654	0.27643	0.8264	0.66004	2.12968	1.37318	1.46892	2.07762
**TraesCS 2A02G2 16900.1**	**TaMAP KKK59**	0	0	0.00986	0.00999	0.00885	0	0	0.00951	0.03052	0	0.03586	0.03301	0.00703	0.02632

**TraesCS2A02G217000.1**	**TaMAPKKK60**	0.01016	0	0.01032	0.02094	0	0	0.03308	0	0.06396	0.01833	1.70449	1.21878	0.26471	0.32358
**TraesCS7D02G384700.1**	**TaMAPKKK61**	1.41644	1.42899	0.13669	0.10516	3.8911	3.80627	0.16976	0.15123	1.79832	1.97933	0.85743	0.50621	0.61796	0.77611
**TraesCS4A02G313900.1**	**TaMAPKKK62**	0	0	0	0	0	0	0	0	0	0	0	0.00488	0	0
**TraesCS4A02G383000.1**	**TaMAPKKK63**	0.05147	0.08057	0	0	0.0326	0.04047	0.01399	0.01265	0.03289	0.05799	0.12831	0.01152	0.07639	0.06062
**TraesCS4A02G465900.2**	**TaMAPKKK64**	0	0	0	0	0	0	0	0	0	0	0	0	0	0
**TraesCS4A02G464700.1**	**TaMAPKKK64-1**	0.08889	0.1542	0	0	0.08881	0.13645	0	0.04953	0.03959	0.02238	0.20029	0.10597	0.10319	0.11404
**TraesCS4A02G465000.1**	**TaMAPKKK65**	0.03249	0.00852	0.00826	0	0.052	0.01928	0.03278	0.00796	0.00639	0	0.02406	0.01382	0.01184	0.00555
**TraesCS1D02G273800.2**	**TaMAPKKK66**	0.08006	0.12332	0.29939	0.22601	0.15022	0.12404	0.14104	0.07142	0.78504	0.74489	3.6652	2.88276	1.05919	1.18647
**TraesCS1D02G360600.1**	**TaMAPKKK67**	0.39304	0.35583	0.16472	0.2532	2.63055	2.27179	0.57619	0.36219	0.05451	0.08352	0.11986	0.11805	0.24184	0.32582
**TraesCS1D02G431400.1**	**TaMAPKKK68**	0	0.00993	0.06758	0.03922	0.29254	0.19502	0.25434	0.21835	0.09672	0.12091	0.67571	0.42215	0.77051	0.89233
**TraesCS2D02G588200.1**	**TaMAPKKK69**	0.24021	0	1.63628	1.40156	0.08948	0.11531	1.17113	0.78922	0	0	0.1432	0.03882	0.0397	0.027
**TraesCS1D02G423800.1**	**TaMAPKKK70**	1.91931	1.69617	1.27247	1.62078	3.16768	3.06723	1.8489	1.06356	3.53494	3.74194	1.92088	1.10623	1.64582	2.14677

**TraesCS6A02G004500.1**	**TaMAPKKK71**	0	0	0	0	0	0	0	0	0.03165	0	0	0.03909	0	0
**TraesCS3A02G003900.1**	**TaMAPKKK72**	0.64138	0.61881	0.27906	0.41986	0.9006	0.87449	0.33903	0.28551	0.66909	0.55071	0.90694	0.66643	0.56422	0.7327
**TraesCS3A02G039200.1**	**TaMAPKKK73**	0.07853	0.05864	0	0	0.03062	0.01766	0	0.01102	0.03534	0.06071	0.729	0.55355	0.03236	0.06824
**TraesCS3A02G039400.1**	**TaMAPKKK74**	0	0	0	0	0	0.01754	0	0	0	0	0	0	0	0
**TraesCS3A02G096500.1**	**TaMAPKKK75**	0.22117	0.28695	0.1341	0.11512	0.24979	0.3224	0.16529	0.24256	1.39391	1.26809	0.83838	0.62178	0.35578	0.35061
**TraesCS3A02G246100.1**	**TaMAPKKK76**	0	0	0.00932	0.01887	0.08208	0.02888	0.01988	0.00899	0	0	0.02154	0.00803	0.03994	0.01971
**TraesCS3D02G501400.1**	**TaMAPKKK78**	0	0	0	0.01764	0.00766	0.00661	0.01897	0.0588	0.02636	0.05779	0.20517	0.18702	0.13894	0.13742
**TraesCS3A02G493900.1**	**TaMAPKKK79**	0.01614	0	0	0.05029	0.03452	0.01579	0.10034	0.05544	0.02371	0	0.34897	0.23238	0.11641	0.14963
**TraesCS2A02G217600.1**	**TaMAPKKK80**	0.19688	0.23732	0.09424	0.18065	0.44567	0.57172	0.05594	0.0708	0.08488	0.10231	0.35804	0.40638	0.17262	0.22568
**TraesCS5A02G292500.1**	**TaMAPKKK81**	0	0	0	0	0	0	0	0	0	0	0	0	0	0
**TraesCSU02G072500.1**	**TaMAPKKK82**	0	0	0	0	0	0	0	0	0	0	0	0	0	0
**TraesCS5A02G351500.1**	**TaMAPKKK83**	0	0	0	0	0	0	0	0	0	0	0	0	0	0

**TraesCS5A02G351000.1**	**TaMAPKKK84**	0	0	0	0	0	0	0	0	0	0	0	0	0	0
**TraesCS5A02G352000.1**	**TaMAPKKK85**	0	0	0	0	0	0	0	0	0	0	0	0	0	0
**TraesCS5D02G386800.1**	**TaMAPKKK86**	8.62561	6.75547	4.05397	4.20793	12.3849	11.6175	6.49278	4.05719	4.43276	4.2128	4.77906	3.41961	2.42781	3.36972
**TraesCS1A02G422800.1**	**TaMAPKKK88**	0	0	0.30396	0	0.42522	0.318	0	0.82093	0.20925	0.21578	3.21276	2.18828	1.98226	2.2712
**TraesCS7A02G152100.1**	**TaMAPKKK90**	0	0	0	0	0	0	0	0.02457	0	0	0	0	0	0
**TraesCS4D02G089300.1**	**TaMAPKKK91**	0.41006	0.50335	0.16897	0.05582	0.4248	0.62209	0.05933	0.1606	1.1614	1.09704	8.27991	6.30753	1.62198	2.08146
**TraesCS5B02G337300.1**	**TaMAPKKK92**	7.00148	8.07734	4.9548	5.24568	9.72469	9.60211	10.578	8.43876	6.05662	5.59606	10.7583	8.00647	9.10318	12.7884
**TraesCS5D02G018800.1**	**TaMAPKKK93**	0.88644	0.69727	0.04175	0.03373	0.60791	0.54335	0.08007	0.08843	0.4323	0.3928	0.55294	0.40509	0.22125	0.35774
**TraesCS5B02G012000.1**	**TaMAPKKK94**	7.32708	5.98234	0.95357	1.08694	28.9352	22.9126	2.16728	1.26321	7.41194	7.34099	6.64124	5.44529	8.00655	9.60441
**TraesCS5B02G204900.1**	**TaMAPKKK95**	1.08188	0.74391	2.49955	3.07212	1.34324	1.27314	2.19199	1.65722	2.76657	2.74523	4.43549	3.74259	0.8813	1.49211
**TraesCS5B02G292000.2**	**TaMAPKKK96**	0.29092	0.23839	0.06082	0	0.09405	0.22501	0	0	0	0.05525	0.08621	0.25184	0.03644	0.21825
**TraesCS5B02G353800.1**	**TaMAPKKK97**	0	0	0	0	0	0	0	0	0	0	0	0	0	0

**TraesCS5D02G482000.1**	**TaMAPKKK98**	4.74231	4.56576	12.2959	14.2296	4.07181	4.70544	13.0377	11.08	6.80238	7.02045	23.774	19.7878	9.55695	13.0233
**TraesCS3A02G001500.1**	**TaMAPKKK99**	1.70786	1.81275	0.29146	0.2849	2.24816	2.43482	1.55215	0.86069	4.43424	4.41477	3.99988	3.17627	3.34339	4.71911
**TraesCS3A02G274000.1**	**TaMAPKKK100**	3.20608	3.06695	2.46266	2.47077	6.44812	5.87213	5.62298	4.39283	1.82817	2.1272	5.50488	4.68964	1.7347	2.20892
**TraesCS5D02G019400.1**	**TaMAPKKK101**	5.43302	4.99033	1.51429	1.65934	21.2365	17.0822	4.05096	2.24142	8.35315	8.18604	9.18916	7.7869	8.93939	9.89305
**TraesCS7D02G474700.1**	**TaMAPKKK102**	3.24916	3.25969	1.66059	1.58951	4.30951	3.67804	2.12014	1.8193	3.82723	3.73805	4.82737	4.01616	3.29634	3.74187
**TraesCS7A02G326700.1**	**TaMAPKKK103**	1.56968	1.75478	0.24194	0.30612	1.58481	1.51786	0.41615	0.37125	0.81184	0.81675	0.91464	0.96256	1.03668	1.42182
**TraesCS1D02G273600.1**	**TaMAPKKK104**	0	0	0	0	0	0.10441	0.01398	0	0.00603	0	0	0	0	0
**TraesCS1B02G283400.1**	**TaMAPKKK104-1**	0.0614	0.04466	0.05198	0.09493	0.13057	0.06197	0.16659	0.06024	0.58294	0.56324	3.81542	3.38243	1.07729	1.52166
**TraesCS3A02G045200.1**	**TaMAPKKK105**	2.37358	2.37077	1.16227	1.31166	2.38588	2.31901	2.94864	1.70425	2.43348	2.75599	5.64181	4.76838	1.46062	2.00966
**TraesCS4A02G456900.1**	**TaMAPKKK106**	0	0	0	0	0	0	0	0	0	0	0	0	0	0
**TraesCS7A02G032700.1**	**TaMAPKKK107**	0	0	0	0	0	0	0	0	0	0	0	0	0	0
**TraesCS3D02G023600.1**	**TaMAPKKK108**	0	0	0	0	0	0	0	0	0	0	0	0	0.00608	0.01136

**TraesCS2D02G219800.1**	**TaMAPKKK109**	0.4262	0.42039	0.3439	0.28562	0.83379	0.71119	0.74341	0.48211	1.65817	1.81043	3.78708	2.99825	2.88728	3.4216
**TraesCS4B02G289100.1**	**TaMAPKKK110**	0.05523	0.02893	0.05615	0.04736	0.01681	0.06526	0.06988	0.03609	0.07964	0.07478	0.62642	0.39413	0.35424	0.34392
**TraesCS3A02G315100.1**	**TaMAPKKK111**	0.41988	0.43497	0.18603	0.16826	0.8096	0.67212	0.2042	0.17256	0.44913	0.47843	1.48027	1.08762	0.85998	1.34854
**TraesCS5D02G359500.1**	**TaMAPKKK112**	0	0	0	0	0	0	0	0	0	0.02253	0	0	0	0
**TraesCS3D02G108500.1**	**TaMAPKKK113**	0.13904	0.11847	3.78331	6.01423	0.20773	0.38144	9.15155	5.49174	0	0.01885	0.318	0.35752	0.44983	0.44206
**TraesCS3A02G229800.1**	**TaMAPKKK115**	0	0	0	0	0	0	0	0	0	0	0	0	0	0
**TraesCS1B02G454000.2**	**TaMAPKKK116**	0	0.00341	0	0.00719	0.02776	0.02628	0.00356	0.01695	0.01792	0	7.56E-09	0.05956	4.94E-08	0
**TraesCS3D02G097000.1**	**TaMAPKKK117**	0.34534	0.3697	0.16404	0.18719	0.38838	0.46085	0.40519	0.45193	2.42467	2.76027	1.31021	0.95755	0.39036	0.49077
**TraesCS2A02G577000.1**	**TaMAPKKK119**	0.26218	0.41025	1.09746	1.0219	0.04099	0.09408	0.26348	0.28715	2.13145	2.14525	0.32285	0.28127	0.20477	0.23516
**TraesCS4A02G317600.1**	**TaMAPKKK120**	1.46858	1.32984	0.86638	0.94202	1.47341	1.35683	0.29493	0.13328	0.53474	0.56463	3.13182	2.37577	1.41587	1.97744
**TraesCS2D02G066900.1**	**TaMAPKKK121**	0.24894	0.32399	0.02399	0.04216	0.31758	0.36157	0.05094	0.05752	0.19547	0.23396	1.60469	1.18537	0.74686	0.83618
**TraesCS2A02G214000.1**	**TaMAPKKK122**	0.02248	0.01175	0.13688	0.24363	0.10225	0.05306	0.42712	0.29053	0.06195	0.05068	0.57257	0.61348	0.32417	0.58589

**TraesCS5D02G358700.1**	**TaMAPKKK123**	0	0	0	0	0	0	0	0	0	0	0	0	0	0
**TraesCS5D02G358200.1**	**TaMAPKKK123-1**	0	0	0	0	0	0	0	0	0	0	0	0	0	0
**TraesCS7D02G153800.1**	**TaMAPKKK125**	0.02678	0.02806	0.00908	0.00918	0.02446	0.00703	0.03871	0.02781	0	0.04834	0.01981	0	0.01297	0.00607
**TraesCSU02G011500.1**	**TaMAPKKK126**	0	0	0	0	0	0	0	0	0	0	0	0	0	0
**TraesCS6D02G339600.1**	**TaMAPKKK127**	0	0	0	0	0	0	0	0	0	0	0	0.00589	0	0
**TraesCS1B02G446500.1**	**TaMAPKKK128**	0.24121	0.20506	0.10544	0.12208	0.59533	0.53415	0.176	0.10472	0.15128	0.19865	0.20567	0.17757	0.15213	0.42048
**TraesCS6B02G320800.1**	**TaMAPKKK129**	0.08121	0.13175	0.01503	0.00758	0.07436	0.09314	0.008	0.02891	0.06382	0.04096	0.63995	0.4838	0.81853	1.03294
**TraesCS7A02G044600.1**	**TaMAPKKK130**	0	0	0	0	0	0	0	0	0	0	0	0	0	0
**TraesCS6B02G215100.2**	**TaMAPKKK131**	1.92534	2.32343	1.65133	1.86876	2.81617	2.6634	1.53695	1.89744	1.69118	1.7388	2.76982	2.38548	3.05617	4.24127
**TraesCS5B02G353600.1**	**TaMAPKKK132**	0	0	0	0	0	0	0	0	0	0	0	0	0	0
**TraesCS1D02G004300.1**	**TaMAPKKK133**	2.56131	2.54671	1.08298	1.31171	5.72206	5.55753	1.29972	1.18712	2.36427	2.15406	2.45614	2.09492	1.87532	2.20017
**TraesCS5D02G547500.1**	**TaMAPKKK134**	0	0	0	0	0	0.00774	0	0	0	0	0.00726	0	0	0

**TraesCS2D02G598800.1**	**TaMAPKKK135**	0	0	0	0	0	0	0	0.01096	0.01758	0	0.00824	0	0	0
**TraesCS2B02G241600.1**	**TaMAPKKK136**	0.06166	0.08603	0.07307	0.03178	0.23412	0.27506	0.24535	0.09074	0.12132	0.09272	0.675	0.54206	0.3643	0.57085
**TraesCS3D02G472000.1**	**TaMAPKKK137**	0.25739	0.32565	1.62195	1.47226	0.46179	0.35077	2.0938	1.62917	0.19108	0.1159	1.19322	0.87867	0.66079	0.78402
**TraesCS3A02G493500.1**	**TaMAPKKK139**	0	0	0	0.01708	0.03047	0	0.03534	0	0	0.01489	0.06024	0.01373	0.07312	0.03369
**TraesCS5D02G097900.1**	**TaMAPKKK140**	1.23504	1.66278	0.51463	0.55156	2.04946	2.0126	1.24707	1.02752	1.14462	1.29091	2.80952	2.45627	2.96012	4.07955
**TraesCS7D02G000800.1**	**TaMAPKKK141**	0	0	0	0	0	0	0	0	0	0	0	0	0	0
**TraesCS2A02G032200.1**	**TaMAPKKK142**	0	0	0	0	0	0	0	0	0	0	0	0	0	0
**TraesCS3D02G501100.1**	**TaMAPKKK144**	0	4.06E-05	6.28E-05	0	0.0511	3.25E-05	0	0.00011	0	0.13066	0	4.73E-05	0	0
**TraesCS2B02G242300.1**	**TaMAPKKK145**	0	0	0	0	0	0	0.03738	0	0	0	0.00847	0.00976	0	0
**TraesCS2B02G241400.1**	**TaMAPKKK146**	0.60248	0.73194	0.72949	0.90637	4.77495	5.51763	1.38907	1.32402	2.79314	2.86358	11.9779	10.2665	4.15872	5.69921
**TraesCS2A02G216600.1**	**TaMAPKKK147**	0.43675	0.18354	0.37694	0.16683	0.9913	0.94122	0	0.26345	1.26619	0.45762	0.45409	0.31642	0.06958	0
**TraesCS1A02G003900.1**	**TaMAPKKK148**	0	0	0	0	0	0	0	0	0	0	0	0	0	0

**TraesCS3B02G008600.2**	**TaMAPKKK150**	1.77208	1.62286	6.54584	6.0289	3.88328	2.90688	11.5323	7.90162	1.00099	0.8964	0.93935	0.51635	0.40509	0.9369
**TraesCS3B02G123800.1**	**TaMAPKKK151**	0.27004	0.24059	0.15002	0.19715	0.31872	0.24242	0.54368	0.54371	0.61723	0.539	4.18164	3.26446	1.46764	1.66453
**TraesCS3B02G259100.1**	**TaMAPKKK152**	0	0	0	0.00874	0	0	0.00922	0.01667	0	0	0	0	0	0
**TraesCS3B02G351800.1**	**TaMAPKKK153**	0	0	0	0	0.02815	0	0	0	0	0	0	0	0.08629	0.06162
**TraesCS3B02G478400.1**	**TaMAPKKK154**	0	0	0	0	0	0	0	0	0	0	0	0	0	0
**TraesCS6B02G217100.1**	**TaMAPKKK155**	0.69852	0.78108	0.0314	0	0.73707	0.99453	0.236	0.06549	0.22604	0.25597	1.21791	0.81562	1.14207	1.57291
**TraesCS1A02G181900.1**	**TaMAPKKKK1**	1.62504	2.11758	0.91637	1.0585	1.79878	1.83868	1.81083	1.3964	2.54547	2.43345	2.82126	2.01443	2.18498	2.79802
**TraesCS1B02G199100.2**	**TaMAPKKKK2**	0	0.13191	0.16029	0.1407	0.16915	0	0.48077	0.18765	0	0	0	0	0	0
**TraesCS1D02G185000.2**	**TaMAPKKKK3**	0	0	0.12277	0	0.03953	0.07642	0.0499	0	0.16435	0.03991	0.13696	0.04491	0	0.04216
**TraesCS2A02G233400.1**	**TaMAPKKKK4**	1.86152	2.11298	10.2224	9.82802	3.98018	4.1295	14.8158	15.318	1.50253	1.55684	2.25338	1.58938	1.53512	2.11029
**TraesCS2B02G249900.1**	**TaMAPKKKK5**	1.55142	2.10435	0.74058	0.80034	1.87058	1.87486	1.00206	1.08793	1.9831	1.56232	2.70628	1.97032	1.67978	2.38912
**TraesCS2D02G232200.1**	**TaMAPKKKK6**	0.90934	1.3684	0.51578	0.36557	0.99944	1.19529	0.78229	0.61027	1.19426	0.99193	1.6943	1.37059	1.23345	1.31221

**TraesCS4B02G395600.1**	**TaMAPKKKK7**	0.02491	0.01299	0.01261	0.01286	0	0.01958	0.02708	0	0	0	0.01852	0.01061	0.02738	0.03374
**TraesCS4B02G398400.3**	**TaMAPKKKK8**	10.8455	11.352	2.95191	2.90618	10.279	9.69322	4.07049	3.64961	6.40615	5.63819	5.92384	4.78263	4.38146	5.61746
**TraesCS5A02G187400.1**	**TaMAPKKKK9**	0.59195	0.31586	0.12411	0.19138	0.85462	0.76518	0.30106	0.1716	0.53685	0.54269	1.0049	0.90888	0.81647	1.06739
**TraesCS5A02G392500.1**	**TaMAPKKKK10**	0.7601	0.58024	0.29207	0.30806	0.64475	0.66001	0.43531	0.3089	0.76629	0.69898	1.11569	1.01564	0.88683	0.93256
**TraesCS5A02G556400.5**	**TaMAPKKKK11**	2.291	1.80467	0.89871	0.76402	1.95998	2.11002	0.32158	0.28028	0.59176	0.7218	0.78317	0.4944	0.47644	0.45092
**TraesCS5B02G397300.1**	**TaMAPKKKK12**	0.53629	0.53127	0.31022	0.27832	0.59363	0.57389	0.45787	0.32331	0.59828	0.66049	1.29169	0.8702	0.69796	1.07908
**TraesCS5D02G203600.1**	**TaMAPKKKK13**	0.98052	1.00271	0.63288	0.94447	1.70779	1.73185	0.56178	0.46077	1.03268	0.82323	2.13495	1.6657	1.14092	1.562
**TraesCS5D02G402300.1**	**TaMAPKKKK14**	0.56409	0.52604	0.27717	0.2854	0.67374	0.69601	0.39938	0.35014	0.87105	0.71165	1.28211	1.1839	0.88425	1.17366
**TraesCS6A02G149900.1**	**TaMAPKKKK15**	0.16657	0.04676	0	0.02292	0.08994	0	0.07235	0.08722	0.58202	0.52172	0.39441	0.32351	0.25506	0.28667
**TraesCS6A02G353400.1**	**TaMAPKKKK16**	0	0	0	0.00832	0	0	0.02865	0	0	0	0.01982	0	0	0.03586
**TraesCS6A02G353500.1**	**TaMAPKKKK17**	0	0	0	0.01247	0	0	0	0	0	0	0	0.01089	0	0
**TraesCS6B02G177800.1**	**TaMAPKKKK18**	0	0	0	0	0	0.11377	0.0511	0	0.19455	0.04878	0.18413	0.10482	0.09224	0.13239

**TraesCS 6B02G3 86100.1**	**TaMAP KKKK19**	0	0	0	0	0	0	0	0.05847	0	0	6.96E-05	0	0.03412	0.03117
**TraesCS 6D02G3 35800.1**	**TaMAP KKKK20**	0.11115	0.05378	0.19782	0.18328	0	0.16169	0.2688	0.28607	0.29463	0.26156	0.35259	0.25477	0.45652	0.46012
**TraesCS 6D02G1 39200.1**	**TaMAP KKKK21**	0.02584	0	0	0	0.01172	0	0	0.0127	0.02904	0.08819	0.10594	0.04777	0.04632	0.02176
**TraesCS 7A02G2 32300.1**	**TaMAP KKKK22**	2.87128	3.26008	2.06441	1.78999	3.90491	4.15381	1.86117	1.9852	3.45884	3.09586	4.34205	3.31826	3.09631	3.86221
**TraesCS 7B02G1 30700.1**	**TaMAP KKKK23**	4.34625	4.42757	2.36408	2.30354	5.00861	4.8075	1.96832	2.05646	4.84156	4.50334	5.84287	4.61368	4.43647	5.80512
**TraesCS 7D02G2 32400.1**	**TaMAP KKKK24**	1.13985	1.14621	0.7667	0.64502	1.92786	1.66159	0.73727	0.49652	1.32838	1.36351	2.50593	1.88067	1.61911	1.92075
**TraesCS U02G11 5300.1**	**TaMAP KKKK25**	2.58611	2.87015	0.6063	1.41354	2.65969	1.3235	1.38767	0.85941	0.7496	0.81622	2.01977	2.15227	0.50453	1.86912

Table S5 The expression profile data of MAPK-MAP4Ks.

Locus ID	gene name	grain_Z71_rep1	grain_Z71_rep2	grain_Z75_rep1	grain_Z75_rep2	grain_Z85_rep1	grain_Z85_rep2	leaf_Z10_rep1	leaf_Z10_rep2	leaf_Z23_rep1	leaf_Z23_rep2	leaf_Z71_rep1	leaf_Z71_rep2	root_Z10_rep1	root_Z10_rep2
**TraesCS6B02G296700.2**	**TaMAPK1**	24.4108	18.2798	0.61531	1.07984	13.9459	10.0219	11.142	13.5966	16.4973	20.7801	8.54232	6.02991	16.6236	20.5462
**TraesCS4A02G336800.2**	**TaMAPK2**	8.92267	6.83392	0.36245	0.23007	1.10328	1.14248	4.20125	1.85965	4.08424	5.03315	3.05025	2.11314	4.54081	5.05101
**TraesCS4A02G106400.1**	**TaMAPK3**	21.3555	20.3193	3.17351	3.12641	2.28869	2.50991	7.53268	10.4741	30.2178	28.3146	45.9295	42.2849	59.2289	55.2361
**TraesCS1D02G088000.2**	**TaMAPK4**	0	0	0	0	0	0.17562	0	0	0	0	0	0	0	0
**TraesCS1D02G422800.1**	**TaMAPK5**	0	0.23106	0.05498	0.01662	0	0	0.58104	0.60318	0.47781	0.51079	0	0.10952	1.77232	1.14527
**TraesCS7B02G009200.1**	**TaMAPK6**	20.5689	16.6073	2.57137	2.74313	9.82822	8.57782	11.1836	10.9977	10.9509	11.5053	8.76512	7.50133	15.2859	15.9999
**TraesCS7D02G342800.3**	**TaMAPK7**	8.60731	5.41097	1.30939	1.07319	8.87654	10.1143	3.16595	4.51884	2.74175	3.44316	2.98436	2.77692	6.19151	7.08251
**TraesCS3D02G225600.1**	**TaMAPK8**	10.7137	8.75814	0.62243	0.6643	7.44689	8.04799	4.23561	5.44132	4.54847	5.59256	1.48717	1.42698	8.32236	8.1398
**TraesCS6B02G146300.1**	**TaMAPK10**	18.4335	13.6394	3.20426	3.22607	6.94167	9.64951	9.28622	13.8863	9.22067	11.0656	16.7953	13.4871	17.4774	18.52
**TraesCS1A02G086500.1**	**TaMAPK11**	12.8121	10.2356	1.99606	1.84006	4.47383	4.69139	5.2771	5.49469	5.50953	6.62221	5.22618	4.85364	10.616	10.5671

**TraesCS7A02G422500.1**	**TaMAPK12**	29.6771	25.6298	2.96297	2.82358	5.19904	5.50976	5.12509	6.45095	27.4061	29.6161	32.7462	31.7244	26.1714	24.5724
**TraesCS6D02G245500.3**	**TaMAPK13**	33.5378	24.6461	1.34139	1.8994	20.6173	22.3282	6.53845	13.9472	14.7489	19.0317	5.5225	8.71895	15.7334	25.4527
**TraesCS1A02G184500.1**	**TaMAPK14**	4.2521	4.04878	0.66599	0.57822	2.13001	2.22837	4.49362	5.20652	5.9231	6.65699	5.70827	4.83869	9.40606	9.18744
**TraesCS3B02G270200.1**	**TaMAPK16**	11.1753	8.13023	0.58925	0.59799	0.71078	0.7682	5.73943	6.09576	8.31498	10.5884	3.61948	3.38482	13.3213	14.4698
**TraesCS6B02G127800.1**	**TaMAPK17**	0.60389	0.2953	1.083	1.06043	0.819	0.70023	0.51143	0.74152	0.41557	0.48546	0.21989	0.11025	0.68097	0.56471
**TraesCS7D02G044100.1**	**TaMAPK18**	33.2428	27.6054	8.33467	7.88306	28.099	37.5967	14.3107	23.5606	27.9787	29.0474	22.8587	22.2115	106.637	113.635
**TraesCS7A02G111300.1**	**TaMAPK19**	18.8547	14.9183	4.31642	3.67391	7.0426	7.34511	12.2515	13.6982	11.4215	12.7252	9.45014	9.10178	19.3921	19.1353
**TraesCS7D02G403700.1**	**TaMAPK20**	3.11329	3.96929	0.65968	0.63587	0.19671	0	0.70744	1.65627	4.20388	4.61771	2.19633	3.07653	3.91603	4.30549
**TraesCS7D02G414900.1**	**TaMAPK22**	1.24048	1.08416	0.22017	0.13985	0.24788	0.24669	0	0.03832	0.05419	0.06245	0.33598	0.2137	0.25221	0.16403
**TraesCS3D02G221700.1**	**TaMAPK23**	2.36118	1.93581	0.07175	0.09719	2.63888	2.77886	1.17186	1.24163	0.61368	0.57397	0.79886	0.77835	1.4628	1.55693
**TraesCS3D02G242200.2**	**TaMAPK24**	6.94279	5.85862	0.52798	0.55959	1.0201	1.15541	6.11051	9.97922	8.93664	10.1875	5.04361	5.25339	10.8371	13.1103
**TraesCS4D02G198600.1**	**TaMAPK25**	22.2729	21.3672	2.46341	2.03852	2.25425	3.46337	8.57298	10.584	47.3954	45.4849	60.7443	58.9115	60.0802	57.118

**TraesCS5D02G534000.2**	**TaMAPK26**	1.37636	0.82281	0	0	0.16345	0.55456	0.17258	0.67698	0.88757	0.18371	0.29226	0	0.80615	0.84788
**TraesCS1B02G104900.1**	**TaMAPK27**	8.01739	6.21115	1.65215	1.66301	5.21658	5.63829	4.87328	6.52552	5.67313	6.29126	6.52998	5.62767	11.2066	11.1558
**TraesCS1B02G192600.3**	**TaMAPK28**	8.11931	5.63032	1.41354	1.40239	3.54922	3.53752	9.21038	11.8714	13.3909	15.2318	14.9928	13.7273	21.284	20.1527
**TraesCS1B02G431400.2**	**TaMAPK29**	0	1.16448	7.96723	7.38679	1.92871	3.68807	0	1.02E-08	0	0	0	2.19E-06	0	0.00358
**TraesCS7A02G335300.2**	**TaMAPK30**	1.98227	1.50666	0.30223	0.94259	2.14211	2.09495	0.28659	1.86537	6.44E-06	1.65E-09	0	0.38293	2.09326	0.86134
**TraesCS4A02G434800.1**	**TaMAPK31**	37.1206	29.4098	6.64643	6.41026	24.5694	28.0097	19.8967	25.8702	34.7553	40.0466	24.0043	22.661	86.3673	90.2965
**TraesCS1D02G410100.1**	**TaMAPK33**	26.6062	22.7873	2.39683	2.382	10.1892	11.5303	12.6991	18.0488	25.1851	26.3399	0.91019	0.53648	22.0251	23.4647
**TraesCS1D02G428900.1**	**TaMAPK34**	11.4807	9.40497	1.21851	0.9519	1.52258	1.45662	5.06587	5.8041	5.28603	6.43792	7.50665	6.93921	15.4448	15.0399
**TraesCS6A02G099600.1**	**TaMAPK35**	0.63887	0.41254	2.41155	2.18999	1.3446	0.975	0.34516	0.33885	0.42215	0.31095	0.34916	0.50219	0.62353	0.74022
**TraesCS6A02G118100.1**	**TaMAPK36**	1.3753	0.44134	0.07446	0.06822	1.26551	0.17686	0.10484	0.79046	0.30391	0.20826	0	0	1.21496	0.34452
**TraesCS6A02G269400.1**	**TaMAPK37**	26.1507	20.5706	2.09788	1.55947	18.49	20.2267	10.6613	14.7988	17.6558	21.0793	10.3974	9.2032	26.4	26.9177
**TraesCS3B02G256700.1**	**TaMAPK38**	5.12743	3.50662	0.36871	0.33047	5.42377	4.4376	1.67841	2.60153	3.17613	3.78062	1.31615	0.9651	5.20825	5.56444

**TraesCS3A02G242100.1**	**TaMAPK39**	17.1361	10.8036	0.55149	0.49624	1.97364	2.16782	5.77238	11.5286	12.6882	13.4176	7.75854	7.13522	16.0331	16.6173
**TraesCS3A02G231700.1**	**TaMAPK40**	1.05532	0.82364	0.01741	0.04714	0.34248	0.38254	0.17706	0.13682	0.2653	0.22644	0.26333	0.30616	0.67361	0.49321
**TraesCS1A02G402400.2**	**TaMAPK41**	0.92585	1.47717	7.77735	7.34089	0.22812	0	0.28215	0.83411	0.99867	0.83869	0	0	0	0.5862
**TraesCS1A02G415300.1**	**TaMAPK42**	0	0.08273	0	0.06346	0	0	1.26867	1.33715	1.04253	0.44337	0.07567	0	2.31725	1.59044
**TraesCS1A02G421000.1**	**TaMAPK43**	20.5566	17.3681	1.04052	0.91519	1.71602	1.90335	6.33271	7.2999	6.79957	7.67153	5.38904	4.84162	16.0454	16.3349
**TraesCS6D02G082900.2**	**TaMAPK44**	0.03203	0	0.99812	1.19173	0.1726	0.07236	0.06296	0.02895	0.46789	0.2794	0.49238	0.87078	0.04015	0.07563
**TraesCS6D02G108100.1**	**TaMAPK45**	16.0884	11.6068	2.13492	2.12899	5.80279	8.09188	6.21092	7.90734	5.77277	7.8933	11.6375	10.1107	13.6191	12.0754
**TraesCS7A02G049000.1**	**TaMAPK46**	8.55907	7.28036	1.73392	0.97897	8.18916	5.25697	4.33672	2.35018	6.89166	7.19077	3.84691	4.31197	2.28244	8.88081
**TraesCS7A02G029700.1**	**TaMAPK47**	0.03121	0	0	0	0	0	0	0	0	0	0.03013	0.03798	0	0
**TraesCS7A02G410700.2**	**TaMAPK49**	21.5791	20.0382	3.51649	3.02103	2.67142	1.93742	3.27595	3.91124	13.5825	12.779	20.137	18.4074	19.4592	17.6976
**TraesCS5B02G536500.1**	**TaMAPK50**	24.9451	19.5727	1.02854	1.08844	1.93162	2.25525	5.37792	5.77288	6.32381	8.02527	5.37952	5.0492	10.1896	11.3577
**TraesCS7B02G246900.3**	**TaMAPK52**	5.87491	4.64332	0.9177	0.93156	7.51252	7.23931	2.74299	3.22119	2.25912	2.70103	1.50278	1.39424	4.93037	5.02366

**TraesCS7B02G309900.1**	**TaMAPK53**	88.1217	68.4065	18.249	15.744	5.31442	6.13227	16.2192	19.6957	79.5443	88.8381	99.6791	98.61	105.556	106.447
**TraesCS7B02G322900.1**	**TaMAPK54**	13.7707	12.4374	2.26827	2.29116	3.75945	4.16632	2.03287	2.13678	11.9547	14.0399	16.3686	16.38	16.786	18.0564
**TraesCS6D02G328800.1**	**TaMAPKK1**	6.91179	7.6271	0.5506	0.58448	2.12781	1.44832	3.99169	1.99874	6.04786	5.29396	2.60024	2.62553	7.53843	5.14681
**TraesCS5B02G565100.3**	**TaMAPKK2**	0.14474	0.08512	0.09182	0.10668	0.21938	0.66847	0	0.23442	0.10443	0.08455	0	0	0.15765	0.18389
**TraesCS5D02G130900.2**	**TaMAPKK3**	0.25736	0.25786	0	6.65E-08	0.73442	1.67051	0.17321	0.63027	0.5339	0.05	0	0.61817	0.61937	0.74701
**TraesCS5A02G122700.4**	**TaMAPKK4**	0	0	0	0	0.51749	0.90832	0	0	0.29952	0	0.3184	0	0	0
**TraesCS4B02G049000.1**	**TaMAPKK5**	0	0	0	0	0	0	0	0	0	0	0	0	0	0
**TraesCS4B02G048100.1**	**TaMAPKK6**	0	0	0	0	0	0	0	0	0	0	0	0	0	0
**TraesCS4B02G048600.1**	**TaMAPKK7**	0	0	0	0	0	0	0	0	0.05601	0	0	0	0	0
**TraesCS4B02G048900.1**	**TaMAPKK8**	0.13666	0.03856	0	0	0	0	0	0	0	0	0	0	0.06916	0.09856
**TraesCS3B02G066300.1**	**TaMAPKK9**	0	0	0	0	0	0	0	0	0	0	0	0	0	0
**TraesCS4D02G048800.1**	**TaMAPKK11**	0	0	0	0	0	0	0.03366	0	0	0	0	0	0.10374	0

**TraesCS4D02G048500.1**	**TaMAPKK12**	0	0	0	0	0	0	0	0	0	0	0	0	0	0
**TraesCS5D02G549600.1**	**TaMAPKK13**	4.29793	2.27704	0.64701	0.55724	2.25353	2.33976	1.18381	1.62496	2.14455	2.01219	1.69744	1.37434	2.66849	2.80538
**TraesCS4A02G265900.1**	**TaMAPKK14**	0	0	0	0	0	0	0	0	0	0	0	0	0	0
**TraesCS4A02G266000.1**	**TaMAPKK15**	0	0	0	0	0	0	0.02853	0	0	0	0	0	0.12204	0.13869
**TraesCS4A02G266100.1**	**TaMAPKK16**	0	0	0	0	0	0	0	0	0	0	0	0	0.1434	0.0811
**TraesCS4A02G266200.1**	**TaMAPKK17**	0	0	0	0	0	0	0	0	0	0	0	0	0	0
**TraesCS5B02G122600.1**	**TaMAPKK18**	0.68029	0.65457	0.00246	0.01802	2.31229	3.10588	0.49587	0.82718	0.27317	0.3805	0.30593	0.33925	0.41242	0.46941
**TraesCS2A02G407600.1**	**TaMAPKKK1**	2.35174	1.88039	0.33788	0.25596	2.15925	2.98777	1.10483	1.71051	1.65506	1.86873	1.48841	1.41758	3.10411	3.40259
**TraesCS4D02G027600.1**	**TaMAPKKK2**	0.01153	0.07805	0.00608	0.01758	0	0	0.47405	0.27864	0.7237	0.78453	0.35406	0.35375	2.04002	1.27056
**TraesCS4B02G210600.2**	**TaMAPKKK3**	5.69314	4.86494	1.15456	0.74299	9.00142	8.10682	2.24118	2.8522	3.69646	3.54357	4.696	4.71112	4.17251	4.23056
**TraesCS6A02G245000.3**	**TaMAPKKK4**	4.10394	3.595	0.73318	0.58149	1.48813	1.47595	2.4135	2.33779	1.47386	2.0824	1.27535	1.26692	4.05505	3.95451
**TraesCS6B02G279300.1**	**TaMAPKKK4-1**	4.50905	4.14894	1.0288	0.80005	3.14617	3.69181	1.62378	2.24084	1.35569	1.76678	0.98482	1.3221	2.8807	3.24199

**TraesCS2A02G199700.1**	**TaMAPKKK5**	75.602	60.1258	4.09574	2.88322	4.86827	4.42538	9.38417	10.5375	51.9077	63.1839	46.4819	43.5181	52.3298	55.3493
**TraesCS3B02G289500.1**	**TaMAPKKK7**	7.51801	8.50026	0.2857	0.26679	0	0.02019	2.91711	1.38952	5.51597	5.37164	3.10806	3.5224	6.55672	6.44169
**TraesCS3B02G288100.1**	**TaMAPKKK8**	6.88357	8.80792	0.45385	0.17531	0.01833	0	3.11613	1.84471	15.9909	16.0437	2.93322	3.10185	4.77258	2.78701
**TraesCS3B02G288300.1**	**TaMAPKKK9**	5.507	7.50469	0.21283	0.16671	0.1363	0.03189	2.15553	1.11933	10.5797	9.99206	1.02239	1.47239	2.81208	2.50142
**TraesCS4D02G211300.2**	**TaMAPKKK10**	3.71133	3.26215	0.5339	0.46183	5.36661	3.74851	0.75756	0.77318	2.37329	2.97444	3.61218	3.69995	1.78158	2.8203
**TraesCS5D02G475900.1**	**TaMAPKKK11**	14.2653	12.458	1.77172	1.81622	6.03408	6.50953	2.04141	2.8935	4.19042	4.81538	11.2977	11.0184	12.8364	13.1886
**TraesCS4A02G093800.2**	**TaMAPKKK12**	4.71409	4.05759	1.12309	1.15958	10.0147	10.8669	2.10535	2.7136	2.851	4.00701	5.38596	5.09952	4.82465	4.69392
**TraesCS5A02G118200.1**	**TaMAPKKK14**	0.57274	0.40958	0.00779	0	0	0	0	0	0.01236	0	0	0	0.01958	0.01724
**TraesCS5A02G463100.2**	**TaMAPKKK15**	4.02595	5.24187	0.63387	0.43493	1.44456	1.64272	0.94048	1.44922	2.18147	2.8053	2.8002	2.84668	3.37647	3.24116
**TraesCS5B02G474500.1**	**TaMAPKKK16**	14.3485	13.8914	1.6091	1.48336	3.60566	3.48611	2.91344	3.32762	6.93994	7.90799	17.1147	16.6678	14.2411	15.2137
**TraesCS5A02G200800.1**	**TaMAPKKK17**	4.78737	4.2042	0.42432	0.35805	6.74E-10	0.17073	3.42787	3.84077	1.53946	1.72752	0	0.04781	8.00209	8.57009
**TraesCS2B02G526200.3**	**TaMAPKKK18**	0.44461	0.20204	1.19E-10	0.32536	0.33791	0.28457	0.17921	0.97267	0.85071	0.86697	0.56525	1.23087	1.41885	1.13299

**TraesCS2A02G498000.3**	**TaMAPKKK20**	3.20797	1.79821	0.79956	1.07742	1.70347	2.172	1.4243	1.84587	3.07766	2.47634	4.61248	2.326	4.51206	4.47563
**TraesCS6A02G149900.1**	**TaMAPKKK21**	0.01491	0	0	0	0	0	0.20916	0.51871	0.08155	0.06032	0	0	0	0
**TraesCS5A02G392500.1**	**TaMAPKKK22**	2.8722	2.65297	0.9929	0.98402	0.69559	0.71462	2.0858	2.61465	1.79127	1.79001	1.37183	1.32328	3.34958	3.58375
**TraesCS6D02G139200.1**	**TaMAPKKK23**	0.01718	0.01939	0	0.00813	0	0.01777	0.29641	0.44582	0.20665	0.38949	0.11671	0.10457	0	0
**TraesCS5B02G199400.1**	**TaMAPKKK24**	3.28821	2.93767	0.24729	0.3374	0.25548	0.36205	2.38569	2.59669	1.26638	1.64795	0	0.03158	6.15245	6.02918
**TraesCS5B02G196400.1**	**TaMAPKKK25**	0.14441	0	0	0	0	0	0.12281	0.17517	0.42101	0.44867	0.37712	0.58057	0	0
**TraesCS2D02G093700.1**	**TaMAPKKK26**	3.74786	3.12338	0.18037	0.16359	0.43393	0.27281	1.72835	1.49211	1.29068	1.55037	0.16603	0.06784	3.41781	3.53014
**TraesCS2B02G110500.1**	**TaMAPKKK27**	4.07051	3.39519	0.1717	0.14141	3.2664	3.20587	1.21616	1.3566	1.1188	1.17164	0.30742	0.35167	3.54698	3.52324
**TraesCS2A02G095300.1**	**TaMAPKKK28**	3.18705	2.48468	0.1935	0.11797	0.98105	0.94226	1.14681	1.34419	1.02177	1.36532	0.12055	0.15312	3.01986	3.08257
**TraesCS5D02G206500.1**	**TaMAPKKK29**	4.58349	3.20572	0.261	0.28067	1.53E-10	1.42E-10	3.22232	3.71632	1.52816	1.8422	0	0	7.86587	7.56048
**TraesCS5D02G145100.1**	**TaMAPKKK30**	1.00668	1.20584	0.48835	0.44176	11.3585	13.6861	1.28374	1.60318	6.84991	6.03831	36.3768	35.6926	5.66349	4.94882
**TraesCS6D02G236400.1**	**TaMAPKKK31**	1.60911	1.64189	0.43379	0.4306	0.36475	0.26763	1.25351	1.21139	0.68951	0.8343	0.21331	0.23451	0.96546	1.02575

**TraesCS 2A02G1 95900.2**	**TaMAP KKK32**	7.8317	6.80864	4.12605	3.69463	11.2165	15.1163	9.11636	12.367	6.10787	7.80143	2.98725	3.00183	19.6101	21.0264
**TraesCS 6B02G2 70400.1**	**TaMAP KKK33**	3.10731	2.18732	0.09877	0.09955	0.12333	0.05129	1.64309	1.38671	0.81009	1.19715	1.12914	0.81779	0.29239	0.35434
**TraesCS 2D02G1 97600.1**	**TaMAP KKK34**	3.53481	4.53489	2.17238	1.77715	0.41401	0.42059	0.63951	0.58887	0.31627	0.40665	0.32656	0.32781	9.58534	8.04465
**TraesCS 2B02G2 23600.1**	**TaMAP KKK36**	8.9444	7.45825	3.40428	3.15649	7.36075	8.91951	6.81383	10.6064	5.30304	5.36642	3.79457	3.75189	12.2586	12.2802
**TraesCS 2B02G2 16800.1**	**TaMAP KKK37**	3.31017	2.04273	29.4994	42.3796	2.67358	2.59689	30.1557	14.7691	3.4181	3.39264	22.4231	19.0127	0.30991	0.36422
**TraesCS 1D02G0 26200.2**	**TaMAP KKK38**	0.96568	0.01192	0	0	0.39958	0.11429	0.14013	0.11098	0	0	0.19183	0	0.64058	0.63435
**TraesCS 6A02G2 55100.2**	**TaMAP KKK39**	1.74022	1.47047	0.34115	0.23157	0.06505	0.11593	0.67173	0.98343	0.35974	0.54489	0.15737	0.08762	0.6592	0.732
**TraesCS 5B02G1 46100.1**	**TaMAP KKK40**	0.75462	0.66387	0.17576	0.13766	1.09354	1.83625	1.26347	1.37777	2.52451	2.78511	11.3043	10.8743	6.1067	5.17908
**TraesCS 6A02G1 72600.1**	**TaMAP KKK41**	0	0	0	0	0	0	0.06567	0	0.02761	0.15743	0	0.04055	0	0
**TraesCS U02G20 3100.1**	**TaMAP KKK42**	0	0	0	0	0	0	0	0	0	0	0	0.05147	0	0
**TraesCS 3B02G1 10300.1**	**TaMAP KKK43**	0.08186	0.09237	0	0	0	0	0.12044	0	0	0.04047	7.41E-10	0	0.13767	0.03939
**TraesCS 2D02G0 03900.1**	**TaMAP KKK44**	0	0	0	0	0.01367	0	0	0	0	0	0	0	0	0

**TraesCS3D02G273200.1**	**TaMAPKKK45**	3.14383	3.37244	1.0329	0.92159	4.83344	5.31382	0.40775	0.65886	1.196	0.97715	2.2432	1.79617	12.0578	12.5461
**TraesCS2D02G050700.1**	**TaMAPKKK46**	0	0	0	0	0	0	0	0	0.17784	0.19656	0.78669	0.77726	0	0
**TraesCS7D02G022200.1**	**TaMAPKKK47**	0.78357	0.67906	0.12384	0.0229	0.38933	0.28132	0.31148	0.35519	0.13834	0.13962	0.06901	0.02893	0.48343	0.57822
**TraesCS7D02G079100.1**	**TaMAPKKK48**	0.53595	0.59393	0.12849	0.08804	0.01564	0.07311	0.17666	0.27549	0.29801	0.15518	0.36529	0.3441	0.313	0.23178
**TraesCS7D02G099200.2**	**TaMAPKKK50**	0	0	0	0	0	0	0	0.01415	0.82171	0.81504	2.03274	1.68136	0	0
**TraesCS7D02G230200.1**	**TaMAPKKK51**	4.99257	4.499	0.05035	0.01692	0.04753	0.02727	2.44957	1.58453	1.57502	1.72997	0.45103	0.64943	1.28717	1.07893
**TraesCS7D02G230500.1**	**TaMAPKKK52**	0	0	0	0	0	0	0	0	0.08438	0.11885	0.29835	0.09868	0	0
**TraesCS1B02G372400.1**	**TaMAPKKK53**	0.26894	0.19428	0.05113	0.05135	0.01898	0	1.18205	1.07535	0.23366	0.15685	0.07323	0.10475	9.84023	8.63609
**TraesCS7D02G503600.1**	**TaMAPKKK54**	0	0	0.00556	0.00502	0	0	0	0	0.02638	0.02046	0.03071	0.03843	16.9281	16.5026
**TraesCS3A02G039100.1**	**TaMAPKKK56**	0.03613	0.02039	0	0	0	0	0	0	0.25834	0.24962	0.64009	0.67153	0.01535	0.11165
**TraesCS3D02G040600.1**	**TaMAPKKK57**	0	0	0	0	0	0	0	0	0	0	0	0	0.60381	0.64193
**TraesCS3B02G259800.1**	**TaMAPKKK58**	1.05051	1.20716	0.10371	0.11447	0.28619	0.23422	0.69445	0.61309	1.72022	1.3187	0.99455	0.66987	1.46116	1.58187

**TraesCS2A02G216900.1**	**TaMAPKKK59**	0	0	0	0	0	0	0	0	0.41626	0.54925	0.43356	0.39932	0	0
**TraesCS2A02G217000.1**	**TaMAPKKK60**	0	0	0	0	0	0	0	0	0.03433	0.0267	0.33303	0.43394	0	0
**TraesCS7D02G384700.1**	**TaMAPKKK61**	0.70369	0.94332	0	0.01672	0	0	0.71171	0.36209	0.23421	0.31093	0.26805	0.21046	0.03458	0.05086
**TraesCS4A02G313900.1**	**TaMAPKKK62**	0	0	0	0	0	0	0	0	0	0	0.00772	0	1.12459	1.02621
**TraesCS4A02G383000.1**	**TaMAPKKK63**	0.10272	0.03865	0.01796	0.02557	0.03031	0.03377	0	0.15649	0.03204	0.0668	0.08501	0.06698	0.04367	0.08212
**TraesCS4A02G465900.2**	**TaMAPKKK64**	0	0.17745	0.10262	0.15299	0.01461	0.00355	0	0	0	0.01569	0.00534	0.01629	0.02983	0
**TraesCS4A02G464700.1**	**TaMAPKKK64-1**	0.4808	0.21485	0.25821	0.27026	0	0.03413	0	0	0.05177	0.03068	0	0	0.07907	0.12533
**TraesCS4A02G465000.1**	**TaMAPKKK65**	0.03325	0	0.02926	0.01062	0.04891	0.01142	0.0222	0.03022	0.06964	0.09699	0.03233	0	4.1301	4.15439
**TraesCS1D02G273800.2**	**TaMAPKKK66**	1.70989	1.66046	0.06102	0.08237	0.47496	0.48955	1.17339	0.93309	5.90434	6.84849	8.12333	9.16127	1.11013	1.17692
**TraesCS1D02G360600.1**	**TaMAPKKK67**	0.04216	0.02379	0.02227	0.0151	0.0279	0.01086	0.24218	0.1724	0.02642	0.07171	0.13326	0.06414	2.1786	2.21447
**TraesCS1D02G431400.1**	**TaMAPKKK68**	0.11234	0.04227	0	0.01189	0	0	0.37055	0.32907	0.2996	0.23442	1.67548	1.30855	4.45429	4.4509
**TraesCS2D02G588200.1**	**TaMAPKKK69**	0	0	0	0	0.38018	0.11596	0	0	0	0	0.16726	0	0.2878	0.38783

**TraesCS1D02G423800.1**	**TaMAPKKK70**	2.43771	3.10006	1.13459	0.93365	8.39569	7.40727	1.76985	2.00039	1.74975	2.11401	3.31512	2.8613	2.63523	2.50199
**TraesCS6A02G004500.1**	**TaMAPKKK71**	0	0	0	0	3.64E-07	0	0	0	0	0	0	0	0.18794	0
**TraesCS3A02G003900.1**	**TaMAPKKK72**	8.58484	8.04143	0.69868	0.70771	1.70068	1.67427	2.16786	1.71954	1.59502	1.95965	0.90712	0.86184	3.30424	3.86852
**TraesCS3A02G039200.1**	**TaMAPKKK73**	0	0	0.00792	0	0	0	0	0	0.0628	0	0.36551	0.31154	0.21784	0.25994
**TraesCS3A02G039400.1**	**TaMAPKKK74**	0	0	0	0	0	0	0	0	0	0	0	0	0	0
**TraesCS3A02G096500.1**	**TaMAPKKK75**	3.03843	2.47025	0.18762	0.13337	0.09867	0.11315	0.27455	0.42809	0.43788	0.39695	0.70437	0.56117	1.03704	0.82018
**TraesCS3A02G246100.1**	**TaMAPKKK76**	0	0	0	0	0	0	0.05354	0.03392	0.01038	0.0121	0.08204	0.08102	0.05299	0.02476
**TraesCS3D02G501400.1**	**TaMAPKKK78**	0.50952	0.53312	0	0	0	0	0.06501	0	0.03816	0.03581	0.2639	0.31265	0.01948	0
**TraesCS3A02G493900.1**	**TaMAPKKK79**	5.18046	4.01847	0.04363	0.02465	0	0.01063	0.22467	0.1407	0.31726	0.28343	1.29589	1.089	0.23774	0.22901
**TraesCS2A02G217600.1**	**TaMAPKKK80**	0.01412	0.07846	0.01464	0.01322	0.02457	0	0.08886	0.03948	0.0116	0.08701	0.06752	0.15229	0.05919	0.06666
**TraesCS5A02G292500.1**	**TaMAPKKK81**	0	0	0	0	0	0	0	0	0	0	0	0	1.11244	1.11887
**TraesCSU02G072500.1**	**TaMAPKKK82**	0	0	0	0	0	0	0	0	0	0	0	0	0	0

**TraesCS5A02G351500.1**	**TaMAPKKK83**	0	0	0	0	0	0	0	0	0	0	0	0	0.02079	0.02356
**TraesCS5A02G351000.1**	**TaMAPKKK84**	0	0	0	0	0	0	0	0	0	0	0	0	0.25327	0.34378
**TraesCS5A02G352000.1**	**TaMAPKKK85**	0	0	0	0	0	0	0	0	0	0	0	0	0.03782	0.05861
**TraesCS5D02G386800.1**	**TaMAPKKK86**	3.98383	5.1795	0.24804	0.22154	10.8325	6.49831	2.3334	2.14553	3.00199	3.84607	6.37948	6.64424	4.32614	3.87485
**TraesCS1A02G422800.1**	**TaMAPKKK88**	0	0	0	0	0	0	0	0	0.54033	0.43465	1.35613	1.07731	0.93148	0.80886
**TraesCS7A02G152100.1**	**TaMAPKKK90**	0	0.06997	0	0	0	0	0	0	0	0	0	0.03773	0.1058	0.14898
**TraesCS4D02G089300.1**	**TaMAPKKK91**	0.3068	0.203	0	0.02525	0	0	0	0	1.00761	1.24733	15.4918	13.9645	1.44331	1.46103
**TraesCS5B02G337300.1**	**TaMAPKKK92**	9.72086	8.56627	1.3444	1.18644	50.1767	47.4539	17.1589	19.1392	11.9001	14.8359	17.451	18.5569	51.2447	58.5409
**TraesCS5D02G018800.1**	**TaMAPKKK93**	0.08956	0.06318	0.01182	0.01068	0.0494	0.04614	0.02236	0.02035	0.10286	0.03265	0.10885	0.1635	0.02863	0.08585
**TraesCS5B02G012000.1**	**TaMAPKKK94**	20.0577	23.9055	0.40397	0.37257	10.4063	10.5323	2.6272	1.64649	7.30104	6.46417	6.97869	7.37463	11.4601	10.264
**TraesCS5B02G204900.1**	**TaMAPKKK95**	10.9977	12.3229	1.20951	0.97431	0.41462	0.44303	1.99106	1.45318	7.35668	7.26881	13.0382	12.8533	2.95814	1.7996
**TraesCS5B02G292000.2**	**TaMAPKKK96**	0	0	0	0	0	0	0	0	0	0.10207	0	0.1345	0	0

**TraesCS5B02G353800.1**	**TaMAPKKK97**	0	0	0	0	0	0	0	0	0	0	0	0	0	0
**TraesCS5D02G482000.1**	**TaMAPKKK98**	21.5974	19.1707	1.63063	1.3914	5.93599	5.78704	5.84366	4.24742	14.2471	13.9253	21.215	20.3637	15.5599	13.8471
**TraesCS3A02G001500.1**	**TaMAPKKK99**	7.46099	7.3328	0.86174	0.52191	0.60157	0.79659	5.02424	6.78208	2.43452	2.97719	1.7239	1.67914	7.78754	6.53631
**TraesCS3A02G274000.1**	**TaMAPKKK100**	7.45335	6.46407	0.6481	0.6226	3.08862	3.3732	1.30397	1.34302	2.0575	1.76051	1.44866	1.4085	7.73655	8.13349
**TraesCS5D02G019400.1**	**TaMAPKKK101**	16.0054	17.4106	0.67718	0.57773	1.49106	1.4811	3.44181	2.31543	11.5566	10.7258	11.4425	11.5348	9.87933	8.40903
**TraesCS7D02G474700.1**	**TaMAPKKK102**	5.54887	5.50222	1.85514	1.58988	3.01266	2.99567	3.65657	3.89054	3.79904	4.06982	3.53496	3.8881	3.82787	4.67993
**TraesCS7A02G326700.1**	**TaMAPKKK103**	0.97122	0.83205	0.13053	0.10852	0.58354	0.62146	1.39831	1.36023	1.24802	1.58659	2.23294	1.88118	0.81183	0.78298
**TraesCS1D02G273600.1**	**TaMAPKKK104**	0	0	0.0065	0.00345	0	0	0.0077	0	0.08801	0	0	0	0	0
**TraesCS1B02G283400.1**	**TaMAPKKK104-1**	0.1933	0.12688	0.10903	0.04595	0.29282	0.32774	0.02753	0.05074	0.70862	0.86184	11.5287	11.6331	0.21955	0.29368
**TraesCS3A02G045200.1**	**TaMAPKKK105**	0.81043	0.79296	0.3367	0.35974	14.1226	14.2031	0.29201	0.18435	4.40762	4.4279	17.6246	19.2819	0.11486	0.09718
**TraesCS4A02G456900.1**	**TaMAPKKK106**	0	0	0	0	0	0	0	0	0	0	0	0	0	0
**TraesCS7A02G032700.1**	**TaMAPKKK107**	0	0	0	0	0	0	0	0	0	0	0	0	0	0

**TraesCS3D02G023600.1**	**TaMAPKKK108**	0	0	0	0	0	0	0	0	0	0	0.01106	0	0.01939	0
**TraesCS2D02G219800.1**	**TaMAPKKK109**	1.28862	1.05641	0.07258	0.06578	0.23871	0.23832	0.01495	0.0546	0.30047	0.32403	6.03829	6.24553	0.06387	0
**TraesCS4B02G289100.1**	**TaMAPKKK110**	0.07964	0.04229	0.01975	0.01189	0.11028	0.10303	0.03738	0.04541	0.45875	0.42526	1.56349	1.67192	0.03192	0
**TraesCS3A02G315100.1**	**TaMAPKKK111**	0.28545	0.26006	0.01778	0.01608	0.0666	0.01727	0.29394	0.28216	1.29163	1.48331	1.59346	1.46124	0.15769	0.24939
**TraesCS5D02G359500.1**	**TaMAPKKK112**	0.04511	0.10239	0.02392	0.01082	0.01001	0	0	0.03092	0.02841	0.02205	0.02205	0	0.12567	0.11957
**TraesCS3D02G108500.1**	**TaMAPKKK113**	0.20823	0.09401	0	0	0	0	0.02768	0.01262	0.06951	0.05407	0.09439	0.08448	0	0
**TraesCS3A02G229800.1**	**TaMAPKKK115**	0.02193	0.12374	0	0	0	0	0	0	0	0	0.02132	0	0.00935	0
**TraesCS1B02G454000.2**	**TaMAPKKK116**	0.004	0.00486	0	0	0	0	0.00424	0.00189	0	0	0.07432	0.02692	0	0.113
**TraesCS3D02G097000.1**	**TaMAPKKK117**	1.84902	1.196	0.23807	0.28747	0.07356	0.04218	0.22979	0.2063	0.41241	0.66668	1.11766	1.28857	0.32364	0.29404
**TraesCS2A02G577000.1**	**TaMAPKKK119**	0.08415	0.01726	0.14021	0.05294	0.78224	0.65264	0.11317	0.05192	0.34997	0.26647	0.43515	0.44534	6.2847	6.39633
**TraesCS4A02G317600.1**	**TaMAPKKK120**	0.03692	0	0	0	0	0	0.09826	0.10069	6.76168	7.47151	40.5375	38.0032	0	0
**TraesCS2D02G066900.1**	**TaMAPKKK121**	0.82117	0.64697	0.06198	0.07395	0.36445	0.30091	0.3544	0.30261	0.66359	0.6728	1.6118	1.27764	0.38968	0.31146

**TraesCS2A02G214000.1**	**TaMAPKKK122**	0.95653	0.80003	0.02379	0.03579	0	0	0.01503	0.01372	0.11322	0.08817	1.91071	2.00076	0.01283	0.01446
**TraesCS5D02G358700.1**	**TaMAPKKK123**	0	0	0	0	0	0	0	0	0	0	0	0	0.02049	0.04611
**TraesCS5D02G358200.1**	**TaMAPKKK123-1**	0	0	0	0	0	0	0	0	0	0	0	0	0.17993	0.29442
**TraesCS7D02G153800.1**	**TaMAPKKK125**	0.30317	0.38326	0.02351	0.01156	0.03212	0.0125	0	0	0	0.0118	0	0.01476	0.60926	0.84087
**TraesCSU02G011500.1**	**TaMAPKKK126**	0	0	0	0	0	0	0	0	0	0	0	0	0	0
**TraesCS6D02G339600.1**	**TaMAPKKK127**	0	0	0.05528	0.0318	0.05028	0.10761	0.22799	0.27627	0.14296	0.12923	0.39756	0.42794	3.50291	3.75212
**TraesCS1B02G446500.1**	**TaMAPKKK128**	2.99868	3.06142	0.57807	0.57957	21.2924	21.7879	1.02279	1.17629	0.87317	1.00298	0.80955	0.94914	1.84919	1.69197
**TraesCS6B02G320800.1**	**TaMAPKKK129**	0.15172	0.20551	0.01604	0.01933	0.04461	0	0	0	0.70997	0.89439	2.85923	2.70806	0	0
**TraesCS7A02G044600.1**	**TaMAPKKK130**	0	0	0	0	0	0	0	0	0	0	0	0	0	0
**TraesCS6B02G215100.2**	**TaMAPKKK131**	4.39641	3.40953	0.86764	0.99427	1.68233	1.35706	2.62723	2.61796	3.79121	4.88499	2.44438	2.48662	4.43897	5.32502
**TraesCS5B02G353600.1**	**TaMAPKKK132**	0	0	0	0	0	0	0	0	0	0	0	0	1.63638	1.67805
**TraesCS1D02G004300.1**	**TaMAPKKK133**	4.07468	3.67054	0.41576	0.49629	0.48041	0.63785	3.7692	3.74816	2.47779	2.56763	0.92998	0.79792	5.4117	4.66218

**TraesCS5D02G547500.1**	**TaMAPKKK134**	0	0	0	0	0	0	0	0	0.01109	0	0.03919	0.04873	0	0
**TraesCS2D02G598800.1**	**TaMAPKKK135**	0	0	0	0	0	0	0	0	0	0	0	0	0.01509	0.02874
**TraesCS2B02G241600.1**	**TaMAPKKK136**	0.33267	0.32855	0.2481	0.19109	0.83109	0.62946	0.41462	0.8445	0.13458	0.08098	0.02693	0.03374	2.2806	2.21401
**TraesCS3D02G472000.1**	**TaMAPKKK137**	0	0.01311	0	0	0	0	0.01159	0.01055	0.07942	0.18066	2.45868	2.28977	0	0
**TraesCS3A02G493500.1**	**TaMAPKKK139**	5.76298	3.67002	0.06132	0.06671	0	0	1.82375	2.436	1.18914	1.30284	1.49451	1.25761	0.01584	0
**TraesCS5D02G097900.1**	**TaMAPKKK140**	3.01934	2.7234	0.27135	0.28302	9.40875	8.67148	5.03646	5.86696	7.62267	9.19599	7.87631	7.03619	7.39227	7.97755
**TraesCS7D02G000800.1**	**TaMAPKKK141**	0	0	0.07441	0.10315	0.00827	0	0	0	0	0.02733	0	0	3.53755	3.74816
**TraesCS2A02G032200.1**	**TaMAPKKK142**	0	0	0	0	0	0	0	0	0	0	0	0	0	0
**TraesCS3D02G501100.1**	**TaMAPKKK144**	0.25657	0	0	5.20E-05	0	0	0	0	0.19208	0	0.12306	0	0	7.21E-06
**TraesCS2B02G242300.1**	**TaMAPKKK145**	0	0	0	0	0	0	0	0	0.02565	0.02997	0.02986	0.01872	0.01309	0
**TraesCS2B02G241400.1**	**TaMAPKKK146**	0.87989	1.2131	0	0.03045	0.10244	0.07953	0.20425	0.26754	1.0592	0.99967	2.07509	2.05562	0.897	1.07671
**TraesCS2A02G216600.1**	**TaMAPKKK147**	0.29877	0	0	0	2.70797	3.3302	0	0	0	0	0.12623	0	0	0.08719

**TraesCS1A02G003900.1**	**TaMAPKKK148**	0	0	0	0	0	0	0	0	0	0	0	0	0.08433	0.03203
**TraesCS3B02G008600.2**	**TaMAPKKK150**	10.6019	11.5836	1.22839	0.87764	2.68728	2.45564	2.75748	1.88679	2.1812	1.69276	0.97067	1.16586	6.79894	6.58454
**TraesCS3B02G123800.1**	**TaMAPKKK151**	0.43865	0.32209	0.06561	0.06666	0.05055	0	0.66365	0.65135	7.57625	8.04817	6.48776	6.77957	3.43963	3.64179
**TraesCS3B02G259100.1**	**TaMAPKKK152**	0.03475	0.02615	0	0	0	0	0	0.01053	0	0.01126	0	0	0	0
**TraesCS3B02G351800.1**	**TaMAPKKK153**	0.08216	0	0	0	0	0	0	0	0.12797	0.11458	0.26071	0.14122	0	0
**TraesCS3B02G478400.1**	**TaMAPKKK154**	0.03823	0.02868	0.02013	0.00607	0	0.01311	0.02544	0.02315	0.02119	0.11137	0	0.03096	1.03802	0.94909
**TraesCS6B02G217100.1**	**TaMAPKKK155**	0	0	0	0	0.03242	0	0	0.08851	0.42036	0.42493	1.36858	1.26551	0	0
**TraesCS1A02G181900.1**	**TaMAPKKKK1**	10.4092	7.22061	4.85381	4.4649	6.40321	7.74615	4.01949	6.35472	4.37574	4.99867	7.0492	6.12511	8.83577	10.0115
**TraesCS1B02G199100.2**	**TaMAPKKKK2**	0.09334	0.29541	0	0	0.14281	0.68513	0	0.1059	0	0	0.3334	0.10225	0	0.10189
**TraesCS1D02G185000.2**	**TaMAPKKKK3**	0.15467	0.35274	0.07568	0.04603	1.6627	1.41101	0.87096	0.08132	0.0682	0.12317	0.15549	0	0.30223	0.71389
**TraesCS2A02G233400.1**	**TaMAPKKKK4**	8.36674	6.93887	1.7164	1.55141	4.05354	4.78097	3.80637	3.96043	3.86049	4.4492	3.29856	3.11483	6.17328	6.68226
**TraesCS2B02G249900.1**	**TaMAPKKKK5**	8.07995	6.04508	1.28561	1.18312	3.73972	4.0003	3.85599	5.14908	4.30031	5.2378	3.65141	2.85223	7.11359	7.51037

**TraesCS2D02G232200.1**	**TaMAPKKKK6**	6.0911	3.37563	0.99381	0.93922	2.60534	1.78599	2.42687	2.54669	2.50905	2.31407	1.69735	1.42179	4.22524	4.41955
**TraesCS4B02G395600.1**	**TaMAPKKKK7**	0.04978	0.15046	0.00864	0.01562	0.10271	0.05137	0	0.03001	0.04247	0.04874	0.03235	0.02029	0.08555	0.01584
**TraesCS4B02G398400.3**	**TaMAPKKKK8**	2.7169	0.75444	1.23518	1.36935	5.43E-08	3.10E-08	1.38905	1.44588	1.7584	0.69352	1.56266	2.02216	2.20747	1.29356
**TraesCS5A02G187400.1**	**TaMAPKKKK9**	0	0	0	0	0	0	0.05617	0.22291	0.48118	0.61465	0.43085	0.70276	0	0
**TraesCS5A02G392500.1**	**TaMAPKKKK10**	2.8722	2.65297	0.9929	0.98402	0.69559	0.71462	2.0858	2.61465	1.79127	1.79001	1.37183	1.32328	3.34958	3.58375
**TraesCS5A02G556400.5**	**TaMAPKKKK11**	0	0.30568	0.10375	0	1.16944	1.44432	0	0	0.31141	0	0.41309	0	0	0.13892
**TraesCS5B02G397300.1**	**TaMAPKKKK12**	2.23882	1.69136	0.96295	0.86512	0.58147	0.39064	1.71095	1.96674	1.95909	1.75305	1.1706	1.08701	2.76776	2.98708
**TraesCS5D02G203600.1**	**TaMAPKKKK13**	0.31236	0.47698	0.00963	0	0	0	0.34459	0.30103	0.7226	1.16098	0.95533	0.7363	0	0
**TraesCS5D02G402300.1**	**TaMAPKKKK14**	2.55004	2.11723	0.84909	0.85037	0.50155	0.72753	1.91236	2.16896	1.88488	2.21538	1.37462	1.46595	3.14192	3.09779
**TraesCS6A02G149900.1**	**TaMAPKKKK15**	0.01491	0	0	0	0	0	0.20916	0.51871	0.08155	0.06032	0	0	0	0
**TraesCS6A02G353400.1**	**TaMAPKKKK16**	0	0.14356	0	0	0.02945	0	0	0	0	0	0	0	0.04284	0.09375
**TraesCS6A02G353500.1**	**TaMAPKKKK17**	0.17618	0.11985	0	0	0	0	0	0	0	0	0	0	0	0

**TraesCS6B02G177800.1**	**TaMAPKKKK18**	0	0	0	0	0	0	0.12541	0.14695	0.03488	0	0.10696	0.11494	0	0
**TraesCS6B02G386100.1**	**TaMAPKKKK19**	0	0	0	0	0	0	0	0	0	0	0	0	0	0
**TraesCS6D02G335800.1**	**TaMAPKKKK20**	0.29582	0.4219	0.12283	0	0.83166	1.3741	0	0.14811	0.475	0.4473	0.42491	0.52418	0.1394	0.34591
**TraesCS6D02G139200.1**	**TaMAPKKKK21**	0.01718	0.01939	0	0.00813	0	0.01777	0.29641	0.44582	0.20665	0.38949	0.11671	0.10457	0	0
**TraesCS7A02G232300.1**	**TaMAPKKKK22**	11.9071	8.51867	1.65205	1.42042	8.39651	8.79259	4.19249	5.98501	7.48615	8.41833	9.41127	7.85262	8.7987	9.515
**TraesCS7B02G130700.1**	**TaMAPKKKK23**	10.5672	8.08974	1.50889	1.4467	6.92037	7.55393	4.35357	5.9919	7.00833	7.86371	7.63091	7.02302	7.6167	8.23585
**TraesCS7D02G232400.1**	**TaMAPKKKK24**	3.02309	1.93368	0.56678	0.31506	1.91074	1.84477	1.31711	0.83064	1.86956	4.10924	1.85262	2.11178	1.67245	1.45145
**TraesCSU02G115300.1**	**TaMAPKKKK25**	0.00032	0.00927	0.45046	0.22645	3.50E-05	0	0.42355	0.51336	2.41803	0.23834	0.72617	0.01878	1.46907	7.69E-06

Table S5 The expression profile data of MAPK-MAP4Ks.

root_Z13_rep1	root_Z13_rep2	root_Z39_rep1	root_Z39_rep2	spike_Z32_rep1	spike_Z32_rep2	spike_Z39_rep1	spike_Z39_rep2	spike_Z65_rep1	spike_Z65_rep2	stem_Z30_rep1	stem_Z30_rep2	stem_Z32_rep1	stem_Z32_rep2	stem_Z65_rep1	stem_Z65_rep2
14.0576	22.9723	22.1792	27.2761	37.1546	36.2331	42.7221	52.3714	48.9568	53.9285	30.7933	47.1066	32.0245	36.9101	39.7736	46.2367
10.1703	5.00269	3.95589	7.69367	6.46213	4.82619	11.8936	5.32985	10.7492	5.51821	1.51538	3.89698	8.18444	8.32315	5.46189	6.05371
32.5263	41.0413	44.6531	33.5171	8.85154	8.15926	11.6509	7.41388	3.20477	1.79714	17.4968	12.0993	54.6245	49.6663	5.19284	6.18819
0	0	0	0	0	0	0	0.12116	0	0	0	0.13367	0	0	0.14968	0
0.36499	2.13147	2.62574	0.49341	0.30519	0.69435	0.28664	0.98645	11.0262	11.7988	1.59177	0.87819	0.42364	0.64464	0	0
13.549	15.4127	13.2908	17.9273	22.8265	21.3338	25.7197	28.3975	15.0877	17.1789	28.8898	28.9146	18.2811	15.6186	14.3469	14.2561
2.30134	3.48672	3.36777	3.68395	6.98779	6.58382	5.50714	5.8255	5.95509	7.47205	4.86032	3.77625	6.8592	6.09792	4.60356	4.69741
7.31342	8.94774	8.25053	8.19043	9.93684	9.17727	18.836	16.2593	12.2242	12.0835	11.1487	10.7141	9.36791	8.14553	25.9531	26.5437
13.8199	9.29107	8.94326	20.1928	16.0299	10.7788	14.2919	16.5587	21.7805	25.0142	12.0621	9.93373	20.7812	16.6688	19.5257	23.9406
10.7639	13.3439	11.0751	15.901	15.7035	17.5045	24.3351	26.8325	13.2993	14.4258	19.9516	21.467	12.9838	12.3061	9.7203	10.381

24.0902	25.3378	23.9822	33.6276	19.0815	16.5207	18.4821	15.1146	19.5765	13.7705	18.6693	16.7534	40.7658	32.4535	7.45564	8.08425
12.5053	25.9503	21.626	30.0871	36.7938	37.2883	39.833	58.4175	69.0164	64.8055	35.0836	44.5384	31.1204	27.03	27.6593	38.0913
6.32855	6.6507	5.56969	9.57094	4.86748	4.21284	3.5248	4.84081	3.11702	3.01407	9.02475	9.94738	9.51446	8.40546	5.3066	5.69076
8.58182	18.1894	15.8747	11.6758	7.54856	7.94033	17.7033	21.9284	8.88704	10.8637	10.168	11.5424	5.31873	4.22497	7.29184	6.8308
0.79154	0.65082	0.74348	0.99345	1.37682	1.62356	1.12628	1.17349	0.67242	0.91249	1.28816	1.4493	0.97965	0.64055	0.47027	0.50734
52.9615	62.6989	60.8521	70.652	28.4164	22.6431	39.7819	38.0917	50.338	46.6747	24.5709	20.681	67.2601	59.846	53.8692	59.0489
14.2095	18.7772	15.2006	21.1137	21.0291	21.0541	23.0943	26.1332	16.1501	17.8644	28.8697	28.6705	17.5802	16.9757	16.1029	13.6409
5.57257	8.67627	5.61391	8.7792	2.13153	2.84777	2.10053	0.5288	2.63845	1.79479	3.56709	1.52024	11.8881	11.5776	1.63299	0.85939
0.08499	0.32372	0.4002	0.06363	0.33665	0.32471	0.54751	0.32585	21.9951	15.4058	0.2368	0.14587	0.18004	0.14365	0	0
0.88323	1.23589	1.15421	1.49841	2.28682	2.40537	2.7148	2.83971	5.66631	5.84598	1.27452	1.62092	1.87995	1.25784	1.79268	1.4753
8.30378	16.2075	12.1836	11.3179	7.4409	8.85821	14.8682	19.3539	7.69033	9.4422	14.6577	15.0993	7.24922	5.61451	7.36457	8.82504
33.7577	50.655	58.0777	36.453	4.64479	3.23831	3.86203	2.04963	3.11042	1.79633	13.0732	10.3215	76.7707	65.322	6.55273	6.14035

0.62472	1.28935	0.88232	0.90522	1.60758	0.64577	1.46513	1.77423	1.29635	1.0705	1.18448	0.29424	0.29884	0	0.86128	0.18669
16.3427	16.2049	13.6885	26.6282	14.8044	15.7607	20.8572	26.011	12.4557	13.2281	18.2147	19.2127	13.0774	11.366	9.03355	10.7712
15.7717	14.2223	13.5521	19.3605	12.4882	10.9612	9.09481	12.4313	6.00338	6.23063	22.1211	22.4987	20.675	20.1499	15.2287	15.6558
0	0	0	6.57E-10	0	0	0	0	1.31618	0	0	0	0.00025	0	0	0
0	0.6983	2.09E-08	0.003	0.37663	1.85623	1.17159	1.86785	0.80242	2.19985	1.64478	0.25706	0	0.24944	0.77836	0.06954
43.9209	51.8266	50.9193	51.6315	23.0208	22.6468	36.0987	32.4788	41.4214	39.4187	20.7803	18.2666	66.8496	60.9824	36.2715	39.6468
12.4311	22.862	21.6745	13.2093	55.1925	40.2436	17.1197	13.0938	4.93167	5.3688	11.427	8.53401	15.56	14.7932	3.92097	4.88575
8.43114	13.3243	13.3383	11.0112	5.69005	5.78832	14.5519	16.0229	6.26097	7.59664	6.50875	6.76517	4.73595	4.80104	13.3684	13.7364
1.03061	0.63088	0.54725	0.86061	1.10138	1.1398	0.88493	0.88434	0.81958	1.007	0.91735	0.79191	0.63164	0.66575	0.58678	0.61476
0.20511	0	0.10961	0	0.74438	1.37115	0.25011	0.44685	0.23327	0.2706	0.14058	0.6704	0.82133	0.3603	0.15497	0.32164
18.6271	30.7584	26.0151	27.6464	40.6612	44.9507	47.3841	46.3589	57.6588	51.1392	39.2523	40.9586	41.7219	43.2595	41.6816	38.9761
3.94253	6.16013	5.42609	6.61387	7.24097	7.1274	13.0353	12.2284	12.0754	10.9814	8.7584	9.75816	5.27966	4.19019	24.2173	23.6627

12.0377	16.7408	14.4721	18.697	10.2147	9.15399	18.4385	20.7458	10.8537	13.1278	17.2249	17.5212	10.5892	9.6068	6.36206	8.61252
0.47234	0.447	0.62279	0.43399	1.13109	1.45044	1.26493	1.12331	3.85323	4.48897	1.01241	1.04558	0.60668	0.57733	0.47505	0.39269
0	0.32518	0.5722	0.25817	0.83629	0.58499	0	0	0	0	0.25643	0.28932	0.47702	0.70699	0.33973	0
1.3427	3.04467	2.80768	0.45381	0.10526	0.23244	0.09454	0.34023	7.94791	7.84695	1.94921	0.77217	0.06982	0.17257	0.47282	0.22939
7.14051	15.2545	14.4761	8.33568	11.7254	11.2528	19.4263	18.599	8.47178	9.91083	7.00629	6.89793	9.34688	8.42388	12.0682	12.1826
0.2989	0.03409	0.09474	0.07997	0.16298	0.27117	0.22557	0.13325	0.18222	0.29854	0.16227	0	0.11832	0.03965	0.42697	0.63457
10.286	9.29099	7.47628	14.5168	14.8509	12.0121	14.2621	14.8244	15.3387	17.183	10.919	10.0554	13.2486	12.5764	13.1732	15.4645
12.7175	28.9865	24.5922	15.5731	5.35308	6.97664	12.8783	14.3081	14.4492	17.5289	3.50094	7.13594	15.8736	16.3121	8.51669	15.6452
0	0	0.03199	0.03482	0.08567	0.09414	0.51186	1.22328	2.26986	1.94162	0	0.0355	0.12888	0.03494	0	0.03287
8.04926	20.2037	27.9511	9.88855	19.5781	28.3442	21.1992	12.0302	6.10164	7.22337	9.38093	6.59862	23.8175	19.41	0.60106	0.66572
8.6319	8.68428	7.27279	10.3083	12.3209	11.5027	19.9157	18.7944	13.5337	13.8607	8.41928	8.78858	12.921	10.9464	8.46573	8.76201
2.12319	3.67889	3.24023	3.83571	4.98892	5.65274	4.84077	4.99677	7.13488	7.23675	3.52553	3.81175	6.91636	6.12625	4.70974	5.30154

20.4004	77.0491	71.9115	32.9191	88.6762	89.1804	63.3789	41.0361	32.5935	29.9804	32.5293	31.5968	141.457	121.727	2.52643	2.6277
18.0362	17.3841	15.7286	22.9914	10.9087	9.20789	13.1832	11.2079	17.0485	14.5834	10.6369	11.7654	18.0484	16.0681	2.38002	3.05635
8.48958	9.65638	14.3385	4.14694	4.36056	7.08405	3.26295	2.18852	4.31266	3.32475	5.4736	3.43794	9.42157	7.81568	4.63062	3.96596
0.18402	0.17936	0.29406	0	0.13181	0.81868	0.21507	0.8019	0.38049	0.09286	0	0.40484	0.11817	0.12271	0.14567	0
0.22986	0.30022	0.37552	0.46224	0.00636	0.3023	0.27799	0.72617	0.41759	0.48041	0.74285	1.27869	2.69E-08	0.54733	0.09651	0.58743
0	0.12905	0	0	0.27888	0	0.38966	0	0.39174	0	0	0	0	0.43215	0	0
0	0	0	0	0	0	0	0	0	0	0	0	0	0.03772	0	0
0	0	0	0	0	0	0	0	0	0	0	0	0	0	0	0
0	0.07316	0.03476	0	0.12367	0.13585	0.03694	0	0.01707	0	0.15169	0.11546	0.02788	0.03786	0	0
0	0.07397	0.07031	0	0	0	0.07467	0.03439	0.20701	0	0	0	0.01409	0	0	0
0	0	0	0	0	0	0	0	0	0	0	0	0	0	0	0
0	0	0.07031	0	0	0	0	0	0.0345	0.03134	0	0	0.01409	0	0	0

0	0	0	0	0	0	0	0	0	0	0	0	0	0	0	0
2.95681	2.34366	2.57277	4.74523	5.54771	6.09242	5.04609	7.48869	3.44445	4.29391	5.38401	6.29798	3.65125	3.58856	2.15878	2.70607
0	0	0	0	0	0	0	0	0.03413	0	0	0	0	0	0	0
0.0139	0	0.02951	0.029	0	0	0	0	0	0	0	0	0	0	0	0
0	0.06838	0.07485	0	0	0	0	0.02112	0.06409	0	0	0	0	0	0	0
0	0	0	0	0.03092	0	0	0.03402	0.01707	0	0	0	0	0	0	0
0.62858	0.43547	0.35553	0.66982	0.77783	1.03607	0.47734	1.03137	0.45488	0.69847	1.05403	0.96418	1.18991	0.45617	0.59094	0.41426
3.13187	3.36214	3.87593	4.00049	4.6001	6.22037	5.07724	6.43118	2.83339	3.51532	3.92868	3.99396	3.4476	2.95965	1.82681	2.41107
2.36026	2.3643	3.01112	1.97529	0.24298	0.21425	0.2264	0.06878	0.03501	0	0.98473	0.56757	0.21127	0.51254	0.0125	0
3.76406	4.41433	3.59965	3.98238	5.35388	5.40034	5.82423	7.436	5.87516	6.19959	4.28913	4.83012	4.89309	3.9698	3.43011	5.05833
0.97113	3.82586	3.03757	2.87109	5.33624	5.35962	5.98941	6.71458	2.30262	4.17244	3.81552	4.41101	3.48646	3.30705	3.03123	3.34222
1.53981	2.74265	2.05859	1.90496	4.02233	4.89647	5.3936	6.02601	2.90485	4.38319	3.16997	3.62127	3.7334	3.07846	2.26364	2.69655

25.761	72.0143	65.1546	34.4606	32.2067	42.0509	41.3399	32.5693	10.6267	9.5426	14.1444	17.7826	58.7771	55.6511	7.29324	7.40727
6.9106	20.3338	25.8455	4.59055	3.78532	3.73516	1.45802	1.32418	2.65331	1.20821	1.81794	1.57403	14.5884	17.0357	0.38078	0.59245
16.6304	27.8412	30.2591	9.24037	8.65584	17.3857	3.12582	1.52889	0.96187	0.63712	7.574	5.03184	20.7984	25.4847	0.04249	0.10478
16.6156	17.994	20.2664	7.99184	3.11151	7.66023	1.04327	0.54184	5.40941	3.03261	3.65296	2.99487	11.4647	17.6432	0.07447	0
1.25721	3.8241	3.48682	2.82243	3.85624	4.42628	4.60408	6.07459	3.17409	4.81855	2.34012	4.86171	1.45114	3.1523	2.21307	1.71798
12.8614	11.1338	9.1205	16.4105	4.94359	4.31963	6.52379	6.81954	13.3804	13.2177	3.75147	3.50718	10.4185	10.5798	4.44343	5.01251
4.41032	4.31942	4.22995	5.56601	6.38255	8.16576	6.88242	8.21442	6.66642	6.27513	4.69181	5.83209	2.33192	4.86906	4.9984	4.83535
0	0	0	0	0.01358	0	0.04377	0	51.8711	33.8269	0	0.01673	0	0	0	0.02993
9.57149	5.17012	5.57619	8.87404	3.05572	3.24475	5.51867	5.77902	6.48723	6.44677	3.02682	2.54086	1.98584	2.65123	2.25395	2.13337
11.8212	14.7163	13.0151	15.1069	8.50628	7.29474	12.3128	10.6701	13.9085	12.8759	6.60179	7.31553	33.7789	34.2633	9.18251	9.81397
2.05893	6.04571	5.24301	2.73077	7.56601	9.57444	2.90609	3.42954	1.52162	1.84917	9.14049	9.25645	2.02847	1.94698	3.45E-10	2.26E-10
0.72608	1.35051	0.6556	1.61926	0.6846	2.18286	0.78161	2.26024	0.65986	0	0.6048	1.4572	0.68081	1.04693	0.25185	0.93369

3.19644	3.60828	3.51254	3.49187	6.47548	6.38529	4.97641	9.01766	3.28399	4.4263	4.94017	6.7395	7.4551	6.58843	1.60229	2.2087
0	0	0	0	0.02066	0	0.45413	0	5.38208	4.09372	0	0.03368	0.01241	0	0.0846	0
2.34812	3.25082	3.65747	3.39388	4.62704	4.71786	4.41641	4.64122	3.39698	3.50035	5.38835	5.46683	4.14041	3.8707	1.58496	1.3341
0	0	0	0	0.03149	0.05201	0.21636	0	16.4279	17.4734	0	0.05827	0	0	0.15535	0.07817
1.80804	4.33642	4.1145	2.5301	5.68374	6.79071	2.38222	2.70672	1.0411	1.55474	7.53864	7.31929	1.65432	1.54419	2.00E-10	3.91E-10
0	0	0	0	0.0147	0.04858	0.14078	0.04799	0.21112	0.14487	0.07278	0.03626	0	0.07167	0.37707	0.25945
1.06987	3.09208	2.7638	1.2675	7.96871	10.7681	1.93102	2.68224	1.88161	2.54639	6.06971	6.93211	1.47946	1.56898	0.01016	0.07794
1.56216	3.00204	2.81571	1.73091	7.27284	10.2819	2.62211	4.16203	3.22177	3.70183	4.87263	7.0273	2.02045	2.02238	0.08834	0.09239
1.17104	2.38395	2.37317	1.54958	7.08032	10.1343	2.58807	3.6489	2.23401	2.36768	4.86669	5.70871	1.5818	1.8852	0.10595	0.04384
2.12404	5.96357	5.5652	3.51627	7.35895	8.49577	2.51522	3.24118	1.61691	1.70846	8.61926	7.66265	1.46376	2.05698	0	0
11.8056	8.69837	9.26185	10.5192	0.34277	0.26141	2.14975	1.60215	11.0379	6.67219	4.44007	3.15279	8.71236	8.36083	5.19022	4.68512
1.03718	1.11735	0.73065	0.891	2.75147	3.60935	3.35631	4.27141	10.6418	11.5808	1.06335	1.47566	2.04448	1.3295	1.43713	1.41368

9.03842	14.8326	13.8957	13.159	10.9909	10.2201	12.3911	13.0263	15.2704	17.7009	20.0636	16.6677	13.9805	13.3569	7.10789	8.5237
0.24159	0.22456	0.18067	0.40925	2.48485	2.52269	4.24894	5.57335	12.7671	11.8763	1.14946	1.94774	1.96628	1.90583	2.9788	2.99537
4.97501	5.69666	7.3981	3.49592	0.38807	0.3063	2.27294	1.61735	0.80036	0.88728	0.82798	0.4983	1.84934	1.5998	0.03301	0.07909
6.78963	8.13253	7.98239	7.05718	8.10653	8.2134	13.2711	14.0081	15.5569	15.75	10.5968	10.2471	9.27536	9.25077	6.52064	7.88371

0	0.16794	0	0.6928	0.40705	0.47861	0.46351	1.32828	0.76243	0.43752	0.54592	0.85594	0.37431	0	0.64304	0.3987
0.47461	0.47542	0.30956	0.79085	1.98631	2.03267	2.42468	3.40147	11.2949	11.762	0.9672	1.18741	1.14506	1.38016	1.16793	1.41617
9.05479	9.18674	11.3385	7.59386	0.54933	0.28804	1.88273	1.54494	4.20586	3.30333	3.84919	2.86478	5.53577	5.09708	4.78854	4.8696
0	0	0.11985	0	0	0	0.16827	0.13409	0.03785	0.068	0	0	0	0	0.10257	0
0	0	0	0.02136	0	0	0	0	0.046	0.00421	0	0.0101	0.059	0.05884	0.04341	0
0.06163	0.09006	0.02123	0.16845	0.0374	0	0.04467	0.0827	0	0	0	0	0	0.04589	0	0
0	0	0	0	0	0	0	0	0	0	0	0	0	0	0	0

7.91766	14.0224	15.3904	8.16332	0.43495	0.38024	0.92257	0.86464	2.58255	1.92961	1.23968	1.3642	4.68914	4.9063	0.67189	0.476
0.03071	0	0	0.12704	0	0	0	0	0	0	0	0	0	0	0	0
0.71715	1.2575	0.56361	1.41899	1.13441	1.57131	1.31789	1.98272	0.70436	1.08011	0.94925	1.18553	0.39121	0.51021	0.17874	0.43457
0.13601	0.21836	0.21248	0.37047	0.30378	0.72256	0.83501	0.80191	0.88226	0.8794	0.32538	0.50952	0.74285	0.96636	0.92699	0.91808
0.03139	0	0	0.10622	0.01426	0	0	0.06203	0.01575	0.04516	0	0	0	0.05211	1.55149	1.23384
1.67623	1.12481	1.03319	1.45034	0.77502	0.42809	11.3457	6.6058	1.76744	1.65876	2.32334	1.95818	1.1592	1.23335	2.13843	1.86732
0	0.02221	0.01025	0.02054	0	0	0	0	0	0.01847	0	0	0	0	0.0625	0
10.2473	6.93552	8.0153	8.67956	0	0	0.02362	0.03209	0.18856	0.18802	0.20559	0.21827	0.79803	0.5521	0.09963	0.20583
16.0012	22.6029	20.4156	24.5132	0.0193	0.0319	0.01155	0.05231	0.07461	0.03782	0.68967	0.5219	0.93869	0.88058	0.20577	0.18014
0.48684	0.02254	0.08201	0.73382	0	0	0	0	0.03656	0	0.01637	0.04086	0	0.02018	0	0
1.33619	0.51473	0.87032	1.70489	0.01327	0	0	0	0	0	0	0	0	0	0	0
1.25129	1.38547	1.33189	1.60417	3.76071	3.27008	2.45966	2.60373	2.30937	2.3164	1.37442	1.46777	2.16335	1.41499	1.54776	1.52239

0	0	0	0	0	0	0	0	0	0	0	0	0	0	0	0
0	0	0.00688	0	0.01257	0	0	0	0	0	0.01246	0	0	0	0	0
0.12375	0.04781	0.02947	0.06222	0.04373	0	0.07207	0.12235	0.12247	0.09585	0	0.07134	0.1172	0.09995	0.32325	0.39174
1.02602	0.76823	0.50192	1.52861	0	0	0.00872	0	0	0.00711	0	0	0	0.01769	0	0
0	0.03692	0.05175	0.01709	0.19803	0.07036	0.05925	0.18133	0.03745	0.22257	0.09313	0.03995	0.04707	0.01912	0.12485	0.19662
0.02429	0.02309	0.05224	0	0.0159	0	0	0.34721	0	0	0.00865	0.01309	0.02069	0.02898	0	0.04826
0.93027	0.37132	0.31992	0.72751	0.47028	0.28241	0.20474	0.53501	0.09591	0.19987	0.31075	0.21299	0.27332	0.21254	0.06797	0.06373
5.74583	3.85485	3.75607	5.57881	0.0508	0.0896	0.73031	0.62826	0.01186	0.05978	0.33303	0.42078	0.26829	0.18603	0.06844	0.08932
0.25458	1.47186	1.58073	0.06803	1.21645	0.64115	0.67997	0.4751	0.41561	0.2528	0.79691	0.76903	1.28715	1.34028	0	0.06383
4.84775	1.48164	2.19142	3.74052	0	0.01065	0.08101	0.08382	0.06695	0.02841	0.09593	0.11879	0.0615	0.07055	0	0
3.21657	2.8193	2.6501	3.71301	0	0.02522	0.0274	0.02484	0.10112	0.06741	0.4484	0.38771	0.21967	0.25569	0.99661	0.77803
1.30446	1.41753	0.81515	2.31438	0	0.08601	0.16387	0.11613	0	0	0	0	0.13672	0.24374	0	0

4.88622	1.44872	1.13084	4.71214	5.50254	6.23137	4.57106	4.02568	3.18629	3.02506	4.01208	4.00994	2.29825	3.25184	2.39008	1.94562
0	0	0	0	0	0	0.09329	0	0	0	0	0	0	0	0	0
2.17445	2.81668	2.85545	2.2114	6.21849	6.34386	7.56596	6.94989	5.04385	4.68066	6.08903	5.48135	5.58577	6.04635	1.71295	1.85551
0.05795	0	0.00764	0.1369	0	0	0	0	0	0	0.05467	0	0.06263	0.01681	0	0
0.31629	0	0.00751	0.55126	0	0	0	0	0	0	0.01357	0	0	0.03337	0	0
2.80631	0.72372	0.75593	3.54555	0.16499	0.08304	0.28676	0.6551	0.9609	0.6298	0.34863	0.33179	0.69621	0.43663	0.92314	0.81478
0.18643	0.11359	0.20559	0.10543	0	0	0.0273	0.01238	0.02519	0.01119	0.01131	0	0	0	0	0.02667
0.20101	0	0	0.11519	0.13955	0.1174	0.13305	0.27513	0.09649	0.10858	0.08144	0.03864	0.13906	0.19366	0.06284	0.02368
0.31851	0.12224	0.15472	0.53382	0.76873	0.56184	0.52034	0.95786	0.75065	0.73247	0.40444	0.46809	0.98942	0.79901	0.48091	0.38602
0.22734	0.17974	0.10763	0.16623	0.04174	0.07381	0.03518	0.02932	0.02878	0.09408	0.10531	0.01569	0.11572	0.0776	0.07868	0.11353
0.17883	1.38239	0.99448	0.56503	0.02841	0	0	0	0	0	0	0	0	0	0	0
0	0	0.00601	0	0	0	0	0	0	0	0	0	0	0	0	0

0	0	0.01275	0.12292	0	0	0	0	0	0	0	0	0	0	0	0
0.82308	1.10079	1.1853	0.97159	0	0	0	0	0	0.01069	0	0	0	0	0	0
0.06728	0.19103	0.12933	0.30904	0	0	0	0	0	0	0	0	0	0	0	0
7.58568	5.84894	4.66133	5.77646	3.32921	2.70994	4.7006	4.59474	7.53622	7.47281	3.00965	4.0138	7.89486	6.32173	7.47946	7.09181
3.40551	1.45087	1.86159	3.72457	0.0122	0	0	0	0	0.03598	0.33568	0.18033	0	0.41617	0	0.1209
0.22391	0.1006	0.03177	0.06788	0	0.03117	0.04025	0.03116	0	0.07368	0	0	0	0	0.03182	0.03165
0.7122	1.04784	0.81279	0.99961	0.48492	0.07484	0.10454	0	0.15001	0	0	0.07155	0	0.31868	0	0
12.5028	25.1366	22.0935	16.8522	9.60023	10.5102	12.778	14.8484	11.4318	13.5474	23.7327	27.6907	30.5449	29.0638	11.3815	12.1295
0.50064	0.19286	0.22395	0.83534	0.12314	0.09049	0.11061	0.10019	0.0907	0.17109	0.02037	0.10097	0.72767	0.49963	0.43774	0.65404
16.9325	7.35593	10.2391	9.83255	6.42432	2.58925	3.70593	2.10971	7.60795	6.39664	3.61231	2.57331	21.1678	23.2017	8.32024	7.59311
3.29816	2.43597	3.26452	2.74995	6.71605	6.51215	5.69941	2.13627	2.59074	1.82511	1.77641	1.39302	10.4826	10.8463	1.52255	1.7413
0	0	0	0.08086	0.20506	0.21458	0	0	0	0	0	0	0.08969	0	0	0

0	0.06163	0	0.05776	0	0	0	0	0	0	0	0	0	0	0	0
18.7227	15.2743	14.6759	19.2419	10.0584	8.18144	9.58147	6.94799	14.4299	14.9723	8.01666	8.3439	15.7612	14.8446	35.6986	36.5816
3.61558	6.56378	6.6421	4.26529	14.1513	12.0196	15.3303	14.1487	15.8381	17.7587	8.04795	7.11268	12.7142	11.0381	6.68	7.03446
6.68574	10.7407	10.4297	7.59839	1.80238	2.81999	1.96805	2.77302	3.63585	2.93502	2.85898	2.94327	4.73605	5.07851	2.06896	1.94424
15.5419	7.23823	10.4136	8.77315	5.74793	3.10997	3.21565	2.19556	5.93945	4.6605	3.74638	3.54179	23.5989	26.957	10.8635	10.3153
4.97426	5.87961	5.38825	5.9198	6.26783	5.35226	6.8996	7.2618	6.04208	7.14322	5.89972	6.21835	5.58736	5.69671	4.55575	4.98482
1.3057	1.28828	1.26906	2.25706	0.92138	1.07501	0.91614	1.09573	1.87661	1.98234	1.3288	1.13461	1.57501	1.2741	2.02143	1.92003
0.02669	0	0.03981	0.01102	0	0	0	0	0	0.01092	0	0	0	0	0	0
0.58761	0.28396	0.3185	0.16507	0.06327	0.04182	0.0303	0	0.16774	0.08431	0.10132	0.14026	0.44028	0.57589	0.01565	0.02963
0.81222	0.41868	0.15305	0.5729	0.57525	0.34072	0.56751	0.33975	1.60109	1.0707	0.26229	0.40076	1.2516	1.32545	0.41669	0.5197
0.01535	0	0.01605	0	0.04386	0.03104	0	0	0	0	0	0	0	0	0	0
0	0	0	0	0.05445	0.14086	0	0	0	0	0	0	0	0	0	0

0.27888	0.26938	0.30141	0.28287	0	0	0	0	0	0	0	0	0	0	0	0
0.74357	0.1876	0.20289	0.46386	0.05342	0.0606	0.03293	0.02991	3.50673	2.45614	0	0.01695	0.16232	0.21255	1.85909	1.74308
0.22821	0.04035	0.04379	0.16163	0	0.01261	0.01371	0.01243	0.1138	0.04496	0.07947	0.01409	0.72806	0.64109	0.15418	0.10072
0.08477	0.0271	0.09206	0.13345	0.51576	0.56873	0.32277	0.48353	0.41677	0.36895	0.43613	0.32143	0.48336	0.40682	0.14692	0.16883
0.15498	0.43955	0.45515	0.28118	0.19749	0.361	0.07469	0.11277	0.04575	0.13254	0.17537	0.13947	0.06614	0.06326	0.04643	0.02285
0.02671	0	0.0209	0.01383	0	0.01402	0.01523	0	0.01405	0	0	0.03134	0.03466	0.0155	0	0
0	0	0.00548	0	0	0	0	0	9.21155	4.9695	0	0	0	0	0	0.01104
0.778	0.25463	0	0.71035	0.00181	0	0	0	0.00197	0	0.00515	0.00223	0.02472	0.03289	0	0.009
2.30198	0.59858	0.32051	3.62036	0	0.02752	0.18529	0.24422	1.65138	1.08695	0.24759	0.40834	0.39671	0.52604	0.86907	0.96352
9.97288	14.2876	12.8571	13.8382	0.08333	0.22699	0.45463	0.25396	0.3024	0.25926	0.55498	0.58754	0.83361	0.46716	0.2415	0.24833
0	0	0	0	0	0	0	0	1.34595	0.79743	0	0	0.0205	0	1.96273	1.79897
0.46795	0.19639	0.26123	0.70187	1.08031	1.04973	1.09698	1.3385	1.68841	1.61185	0.94836	0.5764	0.83422	0.73066	0.87834	1.16503

0.07979	0.08126	0.01516	0.03007	0	0	0	0	0.19923	0.21991	0	0	0.01255	0	0.09307	0.03116
0.32379	0.18128	0.1264	0.40689	0	0	0	0	0	0	0	0	0	0	0	0
0.78324	1.19701	0.86228	1.27567	0	0	0	0	0	0	0	0	0	0	0	0
0.32596	0.68388	0.5829	0.31479	0.09084	0.147	0.05676	0.14479	0.34384	0.41901	0	0.02736	0.06061	0	0.07486	0.02445
0	0	0	0	0	0	0	0	0	0	0	0	0	0	0	0
1.90074	3.86617	4.06935	2.69209	0.05227	0.05763	0.11479	0.05665	0.0385	0.09384	0.15578	0.1927	0	0.0848	0.20539	0.10518
1.24906	1.08684	1.01021	0.95735	6.39113	8.08816	6.67698	4.40283	2.82264	3.63198	5.19185	4.97566	2.48523	2.65081	0.99586	0.98782
0	0	0	0	0.05564	0.08178	0.02221	0.07037	0.12966	0.19987	0.01842	0	0	0.05642	0	0
0	0	0	0.05296	0	0	0.02889	0	0	0	0	0	0	0	0	0
2.32854	3.7797	3.32796	3.66364	3.6371	4.12425	3.76079	4.89069	3.29742	3.9329	2.85394	2.9861	2.36787	2.22716	1.98965	2.27346
2.57705	4.76426	3.98977	2.00841	0	0	0	0	0	0	0	0	0	0	0	0
4.84481	4.40796	5.37058	4.4885	7.83923	10.0207	7.53088	8.56643	4.13738	4.69097	10.26	9.25812	4.34758	5.01292	3.60182	3.441

0	0	0	0	0	0	0	0	0	0	0	0	0	0	0	0
0.1946	0.09689	0.15058	0.22409	0	0	0	0	0	0	0.0136	0.01692	0	0	0	0
1.24303	0.89605	0.90031	2.38904	0.30488	0.33591	0.19772	0.05876	0.07017	0.19717	1.05492	1.32992	0.41898	0.49514	0.04278	0.05595
0	0	0	0.01155	0.02129	0.01173	0.01275	0.01155	0.02352	0.01044	0	0.01309	0	0	0.01195	0
0.03571	0	0	0	3.81353	2.44263	3.05141	4.52711	1.4205	1.32887	3.16433	2.45881	2.22464	2.80234	2.32288	1.9088
6.15919	9.79482	8.48547	8.23523	6.28734	7.56384	6.29469	7.68309	5.98011	6.23682	8.69235	9.16368	4.97315	5.09722	4.71732	4.93982
5.55581	6.71288	6.10325	8.10113	0.40415	0.19909	1.38022	1.92885	0.13301	0.21047	0.10251	0.10566	0.03129	0.06277	0	0
0	0.03854	0.03668	0.03588	0	0	0	0	0	0	0	0	0	0	0	0
4.59E-06	7.86E-06	2.20E-06	9.68E-06	0.09079	0.09249	0	0.1329	0	0	0.1708	0.27379	0.30777	0	0	0
0.04438	0	0	0.03067	0	0	0	0	0.01557	0	0.02791	0.01737	0.02558	0	0	0
3.90884	1.03327	0.70488	4.31554	0.11727	0.18092	0.1197	0.15988	0.32388	0.2419	0.27912	0.17323	0.82986	1.13072	0.27643	0.27905
0	0	0.07813	0.69858	0	0.38046	0	0	0.4849	0	0.12987	0	0	0	0	0

0	0.28844	0.18834	0.20127	0	0	0.03641	0	0	0	0	0	0.02749	0	0	0
3.35022	3.78545	4.10714	2.71196	6.81017	5.9982	8.8569	6.51771	5.2531	5.1292	6.06509	6.04223	14.0718	13.8262	2.98058	2.71753
1.83581	4.69551	3.46111	2.20116	1.36069	1.90506	0.81029	0.71213	3.78346	3.53421	2.06896	1.90214	2.89426	2.58033	0.16179	0.01544
0	0	0	0	0	0	0.01271	0.01152	6.60024	4.42111	0	0	0	0	0	0
0	0	0	0	0.10607	0.03882	0	0.03996	0.11708	0.14249	0	0	0	0	0	0
0.3601	0.91691	0.7436	0.93572	0.10475	0.14116	0.12557	0.22764	0.07747	0.06871	0.1156	0.20144	0.1691	0.21288	0.13125	0.11549
0	0	0.01963	0	0	0.1202	0.05286	0.39911	0.10989	0.03335	0.13009	0	0.08968	0	0.21932	0.20671
8.10155	10.8258	8.61844	15.0877	10.9288	11.4235	10.6347	14.19	9.71206	10.2822	13.9978	13.9439	11.7349	11.367	9.28293	9.76561
0.41422	0	0	0.25203	0.00015	0	0	0.37608	0.42538	0.0734	0.27266	0.59311	0	0.02963	0	0.08045
0.52654	0.53158	0.07405	0.42708	0.20145	0.24315	0.37829	0.52383	0.16755	0.38308	0.43102	0.55113	0.05194	0.34895	0.14687	0.13328
5.03013	5.71944	5.75152	6.42023	9.85414	11.2781	9.32168	10.937	7.51924	8.44769	8.6742	9.58565	8.05154	6.52744	4.78707	5.44439
4.5729	6.98754	6.25231	7.51812	9.2083	10.1418	9.53409	10.9157	6.9518	8.12441	9.92944	11.3859	7.5789	6.23448	5.67332	5.44836

3.64031	4.38747	3.68578	3.33974	5.40513	7.91945	6.27305	7.38399	5.7239	5.54927	5.3013	5.95327	3.79644	3.35627	3.40056	2.45649
0.03996	0.0715	0.00839	0.06611	0.06125	0.16987	0.05591	0.05029	0.08435	0.06007	0.03077	0.03758	0.0693	0	0.01699	0
5.78257	5.62327	3.478	6.22121	1.11551	2.81303	3.49411	6.31962	3.70734	3.83339	2.9602	4.82024	2.72E-09	0.77016	0	0.44602
0.02357	0.05283	0.00822	0.0163	0	0.01648	0.08957	0.02116	0.05239	0.13857	0	0	0	0	0.1808	0.41277
2.34812	3.25082	3.65747	3.39388	4.62704	4.71786	4.41641	4.64122	3.39698	3.50035	5.38835	5.46683	4.14041	3.8707	1.58496	1.3341
0	0.43422	0.3884	0.61243	0.1741	0.13491	0.22894	0	0.72327	0.35184	0	0.25768	0	0.14268	0	0.11322
2.17013	2.94378	2.47192	2.79715	3.93436	3.88434	4.72834	4.11772	3.09686	3.00879	4.93158	4.46393	3.79363	3.05064	1.65173	1.49235
0	0	0	0.01835	0	0	0.04029	0.04954	0.25714	0.15968	0.01665	0.04156	0.03053	0.08211	0.32844	0.35336
2.71663	3.39306	3.03929	3.78862	3.58047	5.16556	4.85308	4.88457	2.79473	3.00974	4.78346	4.62643	3.09671	3.48447	1.59686	1.5382
0	0	0	0	0.02066	0	0.45413	0	5.38208	4.09372	0	0.03368	0.01241	0	0.0846	0
0	0	0.03565	0	0.06144	0.19756	0.1255	0.07907	1.45694	1.97827	0.00436	0	0	0	0	0
0	0	0	0.01558	0	0.02333	0.07679	0.0267	1.34242	1.49212	0.02755	0	0	0	0	0

0	0	0	0	0	0	0.21008	0.31102	24.3923	28.7429	0	0	0.01309	0	0	0
0	0	0	0	0	0	0	0	2.3091	1.79455	0	0	0	0	0	0
0.50377	0.26322	0.22636	0.71697	1.18795	0.99844	0.7372	1.21373	1.59483	2.76197	0.655	0.2883	0.71766	0.26824	0.3237	0.27817
0	0	0	0	0.03149	0.05201	0.21636	0	16.4279	17.4734	0	0.05827	0	0	0.15535	0.07817
6.98154	8.46923	6.92043	10.5431	11.5312	11.3102	12.5518	13.5712	14.5213	14.7811	10.8609	12.223	9.70404	8.22371	7.45009	7.85811
6.46121	7.95549	6.66761	10.2028	10.3853	11.795	11.5382	13.4275	12.6455	13.0036	9.92934	10.235	8.00562	7.6103	5.59586	6.37301
1.40439	2.73531	2.07338	1.52232	2.7471	5.05326	4.09187	4.8219	3.91224	3.93711	2.36022	3.54483	1.79428	2.21543	1.17735	1.32474
1.31201	2.54379	1.99771	2.24793	0.35581	1.67E-09	0.63387	1.68097	0.00135	0.00408	1.34637	2.54599	2.36896	1.10E-10	2.129	1.64067
