# Supplementary material for: Bioinformatic Identification and Expression Analyses of the MAPK–MAP4K Gene Family Reveal a Putative Functional MAP4K10-MAP3K7/8-MAP2K1/11-MAPK3/6 Cascade in Wheat (Triticum aestivum L.)
Source: Plants (Basel). 2024 Mar 24;13(7):941. doi: 10.3390/plants13070941 (PMC11013086; doi:10.3390/plants13070941)
Supplement: Supplementary file 1 [file plants-13-00941-s001.zip › plants-2867660-supplementary/Supplementary Figure S1-S10 and Supplementary table S1-S7/Supplementary table S1-S7/Supplementary table 7.pdf]

Table S6 The primers of MAPK-MAP4Ks and refrerence gene for the qRT-PCR analysis

Primer name	F-Primer sequence (5'-3')	R-Primer sequence (5'-3')
**TaMAPK3**	CGCTCCAACCAAGAACTCTC	GACCACGTACTCCGTCATCA
**TaMAPK6**	GCTTTATCGGAGGAGCACTG	CTGGTGCCCTGTACCATCTT
**TaMAPK14**	CGTTTGACAACCACATCGAC	GCGAATGATCTGGTGGAGAT
**TaMAP2K1**	CTCAACGACGGCAACTACAA	CGACTCGGAGTAGCAGATGG
**TaMAP2K11**	TACATGAGCCCCGAGAGATT	GAGCTCACCGAAGCAGATG
**TaMAP3K1**	CGCTTCTACTCCCCTGTCTG	GACCAAACTGCCCAAGGTTA
**TaMAP3K7**	CCTTTCGGTGACGATACCAT	GAGACGCACTGTGTTCTCCA
**TaMAP3K8**	CGAAACAGAGGACATGCTGA	CGGATTCCAATCCTTCTTCA
**TaRaf76**	TTCGGTGTCATGGTTCTTGA	TGAGAAGCTGGTGTTCATGC
**TaMAP4K3**	GAAAATCCGCAAGTGTTGGT	TGTTGCACGAGGTTCTCAAG
**TaMAP4K10**	GGCATCAATGAGCTCTCTCC	CCCAAAGTAGCGGACAACAT
**TaMAP4K24**	CACGTAAGAGGGGATTTCCA	GCGCGAAGAAGATTAGGATG
**TaRP15**	GCACACGTGCTTTGCAGATAAG	GCCCTCAAGCTCAACCATAACT
