# Supplementary material for: Bioinformatic Identification and Expression Analyses of the MAPK–MAP4K Gene Family Reveal a Putative Functional MAP4K10-MAP3K7/8-MAP2K1/11-MAPK3/6 Cascade in Wheat (Triticum aestivum L.)
Source: Plants (Basel). 2024 Mar 24;13(7):941. doi: 10.3390/plants13070941 (PMC11013086; doi:10.3390/plants13070941)
Supplement: Supplementary file 1 [file plants-13-00941-s001.zip › plants-2867660-supplementary/Supplementary Figure S1-S10 and Supplementary table S1-S7/Supplementary table S1-S7/Supplementary table S1-S7.pdf]

Table S1. Physicochemical property aspects of TaMAPK-TaMAP4K											
**Gene Name**			**Gene ID**	**Chr start**	**Chr end**	**Length (aa)**	**MW (kDa)**	**pI**	**Subcellular Localizatio**	**GRAGY**	**Transmem brane**
**TaMAPK1**			TraesCS6B02G296700.2	532338556	532341647	460	51.60	9.71	Nucleus	-0.388	none
**TaMAPK2**			TraesCS4A02G336800.2	619184426	619188858	445	50.90	6.14	Nucleus	-0.345	none
**TaMAPK3**			TraesCS4A	120604688	120608133	369	42.83	5.46	Nucleus	-0.298	none
**TaMAPK4**			TraesCS1D02G088000.2	73674523	73680096	553	62.75	8.85	Nucleus	-0.513	none
**TaMAPK5**			TraesCS1D02G422800.1	478142725	478147790	605	68.33	7.71	Nucleus	-0.561	none
**TaMAPK6**			TraesCS7B	5513957	5521157	393	44.24	5.50	Nucleus	-0.238	none
**TaMAPK7**			TraesCS7D02G342800.3	439463570	439478002	421	48.54	8.91	Nucleus	-0.426	none
**TaMAPK8**			TraesCS3D02G225600.1	307226730	307233549	613	69.88	9.04	Nucleus	-0.554	none
**TaMAPK10**			TraesCS6B02G146300.1	146837429	146841338	369	42.36	6.67	Nucleus	-0.187	none
**TaMAPK11**			TraesCS1A02G086500.1	73295476	73300550	549	62.31	8.74	Nucleus	-0.492	none
**TaMAPK12**			TraesCS7A02G422500.1	612990989	612994043	824	47.32	8.24	Nucleus	-0.195	none
**TaMAPK13**			TraesCS6D02G245500.3	347684447	347687540	460	51.56	9.71	Nucleus	-0.406	none

**TaMAPK14**	TraesCS1A02G184500.1	334446534	334452739	377	42.87	5.75	Nucleus	-0.328	none
**TaMAPK16**	TraesCS3B02G270200.1	434980667	434986997	586	67.17	9.32	Nucleus	-0.551	none
**TaMAPK17**	TraesCS6B02G127800.1	123702609	123715378	598	56.88	6.43	Nucleus	-0.374	none
**TaMAPK18**	TraesCS7D02G044100.1	22581173	22585094	485	55.11	9.49	Nucleus	-0.487	none
**TaMAPK19**	TraesCS7A02G111300.1	68086983	68094361	396	44.44	5.50	Nucleus	-0.223	none
**TaMAPK20**	TraesCS7D02G403700.1	520827434	520832219	581	65.47	7.32	Nucleus	-0.482	none
**TaMAPK22**	TraesCS7D02G414900.1	533432518	533434635	377	43.02	8.04	Nucleus	-0.157	none
**TaMAPK23**	TraesCS3D02G221700.1	301965173	301974102	488	55.77	6.82	Nucleus	-0.425	none
**TaMAPK24**	TraesCS3D02G242200.2	337303815	337310057	584	66.87	9.32	Nucleus	-0.539	none
**TaMAPK25**	TraesCS4D02G198600.1	344940376	344943564	369	42.82	5.46	Nucleus	-0.312	none
**TaMAPK26**	TraesCS5D02G534000.2	548349748	548354652	441	50.24	5.74	Nucleus	-0.322	none
**TaMAPK27**	TraesCS1B02G104900.1	115953191	115959231	549	62.38	8.92	Nucleus	-0.492	none

**TaMAPK28**	TraesCS1B02G192600.3	344838254	344842121	376	42.86	5.75	Nucleus	-0.329	none
**TaMAPK29**	TraesCS1B02G431400.2	655781234	655786592	556	63.02	9.04	Nucleus	-0.513	none
**TaMAPK30**	TraesCS7A02G335300.2	490831796	490841117	421	48.45	8.83	Nucleus	-0.414	none
**TaMAPK31**	TraesCS4A02G434800.1	705502495	705508717	484	54.84	9.50	Nucleus	-0.476	none
**TaMAPK33**	TraesCS1D02G410100.1	471618853	471623653	555	62.75	9.33	Nucleus	-0.523	none
**TaMAPK34**	TraesCS1D02G428900.1	480888268	480894402	599	66.65	9.22	Nucleus	-0.416	none
**TaMAPK35**	TraesCS6A02G099600.1	67126390	67137086	500	57.25	6.36	Nucleus	-0.429	none
**TaMAPK36**	TraesCS6A02G118100.1	88854990	88859282	380	43.50	7.20	Nucleus	-0.181	none
**TaMAPK37**	TraesCS6A02G269400.1	495685844	495688911	460	51.54	9.71	Nucleus	-0.4	none
**TaMAPK38**	TraesCS3B02G256700.1	414020367	414026235	602	68.57	8.99	Nucleus	-0.534	none
**TaMAPK39**	TraesCS3A02G242100.1	454510686	454516596	582	66.51	9.25	Nucleus	-0.522	none
**TaMAPK40**	TraesCS3A02G231700.1	432855223	432868359	490	56.03	6.17	Nucleus	-0.429	none

**TaMAPK41**	TraesCS1A02G402400.2	566009663	566015714	548	62.14	9.06	Nucleus	-0.49	none
**TaMAPK42**	TraesCS1A02G415300.1	574351875	574357108	606	68.45	7.71	Nucleus	-0.581	none
**TaMAPK43**	TraesCS1A02G421000.1	577468378	577474524	598	66.51	9.27	Nucleus	-0.409	none
**TaMAPK44**	TraesCS6D02G082900.2	47937796	47944111	505	58.04	6.48	Nucleus	-0.437	none
**TaMAPK45**	TraesCS6D02G108100.1	72229215	72231632	382	43.82	7.63	Nucleus	-0.205	none
**TaMAPK46**	TraesCS7A02G049000.1	22930536	22936407	492	56.02	9.46	Nucleus	-0.457	none
**TaMAPK47**	TraesCS7A02G029700.1	12115497	12118204	324	37.21	9.16	Nucleus	-0.237	none
**TaMAPK49**	TraesCS7A02G410700.2	598216576	598222568	578	65.15	6.85	Nucleus	-0.496	none
**TaMAPK50**	TraesCS5B02G536500.1	692559588	692565223	443	50.42	6.04	Nucleus	-0.351	none
**TaMAPK52**	TraesCS7B02G246900.3	457243859	457252780	421	48.49	8.91	Nucleus	-0.418	none
**TaMAPK53**	TraesCS7B02G309900.1	553883880	553888100	578	65.21	6.85	Nucleus	-0.485	none
**TaMAPK54**	TraesCS7B02G322900.1	573261339	573264067	377	43.19	7.20	Nucleus	-0.188	none

**TaMAPKK1**	TraesCS6D02G328800.1	433408096	433409202	368	39.62	9.30	Cytoplasm. Nucleus	-0.323	none
**TaMAPKK2**	TraesCS5B02G565100.3	710226104	710231942	525	58.65	7.89	Nucleus	-0.329	none
**TaMAPKK3**	TraesCS5D02G130900.2	207244486	207252504	525	58.60	5.34	Nucleus	-0.242	none
**TaMAPKK4**	TraesCS5A02G122700.4	267940928	267946212	299	33.60	5.89	Nucleus	-0.039	none
**TaMAPKK5**	TraesCS4B02G049000.1	37296414	37297430	338	36.34	8.23	Nucleus	-0.117	none
**TaMAPKK6**	TraesCS4B02G048100.1	36012946	36013977	343	36.19	8.95	Nucleus	0.076	none
**TaMAPKK7**	TraesCS4B02G048600.1	37084618	37085631	337	35.63	8.81	Nucleus	0.09	none
**TaMAPKK8**	TraesCS4B02G048900.1	37173459	37174463	334	35.92	8.62	Nucleus	-0.106	none
**TaMAPKK9**	TraesCS3B02G066300.1	39545184	39546200	338	36.26	7.72	Nucleus	-0.052	none
**TaMAPKK11**	TraesCS4D02G048800.1	25193488	25194492	334	35.95	7.76	Nucleus	-0.096	none
**TaMAPKK12**	TraesCS4D02G048500.1	25159546	25160577	343	36.26	8.76	Nucleus	0.077	none
**TaMAPKK13**	TraesCS5D02G549600.1	556549608	556556744	523	58.42	5.71	Nucleus	-0.228	none

**TaMAPKK14**			TraesCS4A02G265900.1	578007015	578008028	337	36.11	7.15	Nucleus	-0.061	none
**TaMAPKK15**			TraesCS4A02G266000.1	578175110	578176265	337	36.26	8.21	Nucleus	-0.07	none
**TaMAPKK16**			TraesCS4A02G266100.1	578233482	578234994	334	35.77	8.84	Nucleus	-0.082	none
**TaMAPKK17**			TraesCS4A02G266200.1	578382450	578383463	337	35.60	8.88	Nucleus	0.098	none
**TaMAPKK18**			TraesCS5B02G122600.1	220811093	220817726	476	53.82	5.74	Nucleus	-0.311	none
**TaMAPKK**	**MEKK**	TaMEKK1	TraesCS2A	663319194	663325688	827	89.98	9.73	Nucleus	-0.671	none
**TaMAPKKK2**		TaMEKK2	TraesCS4D02G027600.1	12408039	12412107	570	63.22	8.89	Nucleus	-0.375	none
**TaMAPKKK3**		TaMEKK3	TraesCS4B02G210600.2	449128767	449134380	705	77.22	6.53	Nucleus	-0.286	none
**TaMAPKKK4**		TaMEKK4	TraesCS6A02G245000.3	456595800	456610297	878	94.93	9.94	Nucleus	-0.624	none
**TaMAPKKK4-1**		TaMEKK4-1	TraesCS6B02G279300.1	504591712	504606525	786	84.73	9.83	Nucleus	-0.606	none
**TaMAPKKK5**		TaMEKK5	TraesCS2A02G199700.1	172214508	172218294	543	60.97	6.59	Nucleus	-0.452	none
**TaMAPKKK7**		TaMEKK7	TraesCS3B02G289500.1	464615550	464617173	473	49.83	4.64	Nucleus	-0.147	none
**TaMAPKKK8**		TaMEKK8	TraesCS3B02G288100.1	462866244	462867785	477	51.60	5.26	Nucleus	-0.138	none

**TaMAPKKK9**	TaMEKK9	TraesCS3B02G288300.1	462890522	462891675	366	38.24	4.55	Nucleus	-0.172	none
**TaMAPKKK10**	TaMEKK10	TraesCS4D02G211300.2	363679689	363686267	710	77.70	6.55	Nucleus	-0.295	none
**TaMAPKKK11**	TaMEKK11	TraesCS5D02G475900.1	515114772	515119147	534	59.81	6.43	Nucleus	-0.45	none
**TaMAPKKK12**	TaMEKK12	TraesCS4A02G093800.2	101741028	101746878	710	77.72	6.55	Nucleus	-0.306	none
**TaMAPKKK14**	TaMEKK14	TraesCS5A02G118200.1	242531195	242538426	553	62.10	6.37	Nucleus	-0.344	none
**TaMAPKKK15**	TaMEKK15	TraesCS5A02G463100.2	643328443	643333346	534	59.83	6.43	Nucleus	-0.448	none
**TaMAPKKK16**	TaMEKK16	TraesCS5B02G474500.1	647940529	647944797	535	59.93	6.31	Nucleus	-0.464	none
**TaMAPKKK17**	TaMEKK17	TraesCS5A02G200800.1	406667546	406672895	682	74.39	6.29	Nucleus	-0.559	none
**TaMAPKKK18**	TaMEKK18	TraesCS2B02G526200.3	720473250	720486136	1324	146.41	5.83	Nucleus	-0.298	none
**TaMAPKKK20**	TaMEKK20	TraesCS2A02G498000.3	728546777	728559173	1323	146.44	5.86	Nucleus	-0.301	none
**TaMAPKKK21**	TaMEKK21	TraesCS6A02G149900.1	133414193	133419353	543	61.18	6.83	Nucleus	-0.552	none
**TaMAPKKK22**	TaMEKK22	TraesCS5A02G392500.1	588731002	588738966	825	89.69	5.31	Nucleus	-0.504	none

**TaMAPKKK23**		TaMEKK23	TraesCS6D02G139200.1	108447173	108452128	509	57.60	6.71	Nucleus	-0.504	none
**TaMAPKKK24**		TaMEKK24	TraesCS5B02G199400.1	359309183	359314533	680	74.21	6.29	Nucleus	-0.554	none
**TaMAPKKK25**		TaMEKK25	TraesCS5B02G196400.1	354227906	354229839	518	56.47	6.01	Nucleus	-0.16	none
**TaMAPKKK26**		TaMEKK26	TraesCS2D02G093700.1	45528501	45538616	1335	146.06	8.01	Nucleus	-0.051	none
**TaMAPKKK27**		TaMEKK27	TraesCS2B02G110500.1	72570247	72580245	1335	146.06	8.01	Nucleus	-0.049	none
**TaMAPKKK28**		TaMEKK28	TraesCS2A02G095300.1	48855363	48865404	1332	145.87	8.09	Nucleus	-0.042	none
**TaMAPKKK29**		TaMEKK29	TraesCS5D02G206500.1	312603930	312609217	682	74.38	6.29	Nucleus	-0.547	none
**TaMAPKKK30**	**ZIK**	TaZIK1	TraesCS5D02G145100.1	231743581	231750603	640	70.63	5.71	Nucleus	-0.606	none
**TaMAPKKK31**		TaZIK2	TraesCS6D02G236400.1	332952908	332957161	616	68.85	4.86	Nucleus	-0.325	none
**TaMAPKKK32**		TaZIK3	TraesCS2A02G195900.2	165847896	165850719	701	78.40	5.53	Nucleus	-0.506	none
**TaMAPKKK33**		TaZIK4	TraesCS6B02G270400.1	487199066	487203196	617	69.03	4.89	Nucleus	-0.367	none
**TaMAPKKK34**		TaZIK5	TraesCS2D02G197600.1	145361837	145364153	319	35.94	6.46	Nucleus	-0.443	none

**TaMAPKKK36**		TaZIK7	TraesCS2B02G223600.1	213252738	213256205	703	78.63	5.61	Cell membrane. Nucleus	-0.529	none
**TaMAPKKK37**		TaZIK8	TraesCS2B02G216800.1	202919276	202921526	322	36.36	6.41	Nucleus	-0.466	none
**TaMAPKKK38**		TaZIK9	TraesCS1D02G026200.2	10715309	10722269	615	68.76	5.04	Nucleus	-0.631	none
**TaMAPKKK39**		TaZIK10	TraesCS6A02G255100.2	472517606	472521858	616	68.94	4.82	Nucleus	-0.35	none
**TaMAPKKK40**		TaZIK11	TraesCS5B02G146100.1	273714479	273721500	640	70.50	5.55	Nucleus	-0.6	none
**TaMAPKKK41**	**Raf**	**TaRaf1**	TraesCS6A02G172600.1	183765974	183767047	341	38.49	8.87	Nucleus	-0.263	none
**TaMAPKKK42**		**TaRaf2**	TraesCSU02G203100.1	302673848	302675171	281	31.63	8.28	Nucleus	-0.288	none
**TaMAPKKK43**		**TaRaf3**	TraesCS3B02G110300.1	76121219	76122267	146	16.52	6.20	Nucleus	-0.304	none
**TaMAPKKK44**		**TaRaf4**	TraesCS2D02G003900.1	2448415	2452565	666	73.48	6.20	Nucleus	-0.047	yes
**TaMAPKKK45**		**TaRaf5**	TraesCS3D02G273200.1	378922472	378925538	601	67.50	9.28	Nucleus	-0.586	none
**TaMAPKKK46**		**TaRaf6**	TraesCS2D02G050700.1	18755065	18763417	842	93.01	6.62	Chloroplast. Nucleus.	-0.078	yes
**TaMAPKKK47**		**TaRaf7**	TraesCS7D02G022200.1	10480193	10483005	454	50.76	5.30	Cell membrane. Cytoplasm. Nucleus.	-0.418	none

**TaMAPKKK48**	**TaRaf8**	TraesCS7D02G079100.1	46798827	46802577	590	66.29	5.16	Nucleus	-0.153	yes
**TaMAPKKK50**	**TaRaf10**	TraesCS7D02G099200.2	59413722	59416749	662	73.14	5.78	Cell membrane. Nucleus.	-0.05	yes
**TaMAPKKK51**	**TaRaf11**	TraesCS7D02G230200.1	191491327	191494118	411	45.94	7.61	Cell membrane. Nucleus.	-0.084	yes
**TaMAPKKK52**	**TaRaf12**	TraesCS7D02G230500.1	191554886	191558690	421	46.94	8.84	Cell membrane. Nucleus.	-0.037	yes
**TaMAPKKK53**	**TaRaf13**	TraesCS1B02G372400.1	603091105	603093899	804	88.12	5.43	Nucleus	-0.064	yes
**TaMAPKKK54**	**TaRaf14**	TraesCS7D02G503600.1	608808978	608814007	714	79.66	6.28	Chloroplast. Cytoplasm.	-0.306	yes
**TaMAPKKK56**	**TaRaf16**	TraesCS3A02G039100.1	20834670	20837048	483	54.10	7.10	Cell membrane. Nucleus.	-0.083	yes
**TaMAPKK**	**TaRaf17**	TraesCS3D	15371189	15373968	644	70.45	7.43	Cell	0.016	yes
**TaMAPKKK58**	**TaRaf18**	TraesCS3B02G259800.1	417712407	417716508	385	42.62	7.57	Nucleus	-0.375	none
**TaMAPKKK59**	**TaRaf19**	TraesCS2A02G216900.1	203718668	203726292	669	72.89	6.26	Nucleus	-0.092	yes
**TaMAPKKK60**	**TaRaf20**	TraesCS2A02G217000.1	203756927	203768418	687	76.38	5.89	Nucleus	-0.231	yes
**TaMAPKK**	**TaRaf21**	TraesCS7D	498527161	498529159	396	44.67	9.19	Nucleus	-0.357	none
**TaMAPKKK62**	**TaRaf22**	TraesCS4A02G313900.1	604208153	604212003	1095	118.89	6.14	Cell membrane.	0.239	yes

**TaMAPKKK63**	**TaRaf23**	TraesCS4A02G383000.1	660923030	660926301	607	67.95	6.00	Nucleus	0.011	yes
**TaMAPKKK64**	**TaRaf24**	TraesCS4A02G465900.2	729138021	729142814	722	80.84	5.28	Nucleus	-0.2	none
**TaMAPKK**	**TaRaf24-1**	TraesCS4A	728455965	728458756	168	18.72	5.85	Nucleus	-0.07	none
**TaMAPKKK65**	**TaRaf25**	TraesCS4A02G465000.1	728517691	728524395	741	82.77	5.79	Nucleus	-0.107	none
**TaMAPKKK66**	**TaRaf26**	TraesCS1D02G273800.2	368278445	368281075	421	46.94	8.84	Cell membrane. Nucleus.	-0.037	yes
**TaMAPKKK67**	**TaRaf27**	TraesCS1D02G360600.1	443716098	443718946	804	88.21	5.44	Nucleus	-0.071	yes
**TaMAPKKK68**	**TaRaf28**	TraesCS1D02G431400.1	481556383	481559743	710	76.79	6.00	Nucleus	-0.097	yes
**TaMAPKKK69**	**TaRaf29**	TraesCS2D02G588200.1	645705545	645712161	597	66.95	5.48	Nucleus	-0.416	none
**TaMAPKKK70**	**TaRaf30**	TraesCS1D02G423800.1	478411677	478415662	374	41.10	7.05	Cytoplasm. Nucleus.	-0.299	none
**TaMAPKKK71**	**TaRaf31**	TraesCS6A02G004500.1	1877233	1883101	794	90.75	5.59	Nucleus	-0.357	none
**TaMAPKKK72**	**TaRaf32**	TraesCS3A02G003900.1	1924585	1928794	687	72.42	6.26	Nucleus	-0.429	yes
**TaMAPKKK73**	**TaRaf33**	TraesCS3A02G039200.1	20841799	20844493	682	73.97	7.78	Cell membrane. Nucleus.	0.019	yes
**TaMAPKKK74**	**TaRaf34**	TraesCS3A02G039400.1	20931615	20935238	632	69.75	8.19	Chloroplast.	-0.007	yes

**TaMAPKKK75**	**TaRaf35**	TraesCS3A02G096500.1	61445227	61448967	653	69.90	8.98	Chloroplast.	0.034	yes
**TaMAPKKK76**	**TaRaf36**	TraesCS3A02G246100.1	461839239	461841696	813	89.58	6.40	Chloroplast.	-0.155	yes
**TaMAPKKK78**	**TaRaf38**	TraesCS3D02G501400.1	590305938	590309917	716	79.31	5.85	Cell membrane. Nucleus.	-0.111	yes
**TaMAPKKK79**	**TaRaf39**	TraesCS3A02G493900.1	720213465	720223668	704	78.64	5.75	Cell membrane.	-0.082	yes
**TaMAPKKK80**	**TaRaf40**	TraesCS2A02G217600.1	204885066	204888307	477	50.96	8.00	Nucleus	-0.255	yes
**TaMAPKKK81**	**TaRaf41**	TraesCS5A02G292500.1	502584783	502592915	775	88.02	6.28	Nucleus	-0.284	none
**TaMAPKKK82**	**TaRaf42**	TraesCSU02G072500.1	63290513	63296324	840	92.42	5.99	Cell membrane. Nucleus.	-0.135	yes
**TaMAPKKK83**	**TaRaf43**	TraesCS5A02G351500.1	554181335	554183861	310	34.93	7.61	Nucleus	-0.235	none
**TaMAPKKK84**	**TaRaf44**	TraesCS5A02G351000.1	553490210	553499037	848	93.63	7.05	Cell membrane. Nucleus.	-0.176	yes
**TaMAPKKK85**	**TaRaf45**	TraesCS5A02G352000.1	554234499	554238433	836	92.61	7.09	Nucleus	-0.204	yes
**TaMAPKKK86**	**TaRaf46**	TraesCS5D02G386800.1	456607644	456612314	373	41.35	8.53	Nucleus	-0.355	none
**TaMAPKKK88**	**TaRaf48**	TraesCS1A02G422800.1	578196408	578199395	690	75.15	5.95	Nucleus	-0.066	yes

**TaMAPKKK90**	**TaRaf50**	TraesCS7A02G152100.1	105511739	105513870	362	40.41	7.04	Nucleus	-0.274	none
**TaMAPKKK91**	**TaRaf51**	TraesCS4D02G089300.1	64706830	64719914	755	85.85	6.45	Nucleus	-0.263	none
**TaMAPKKK92**	**TaRaf52**	TraesCS5B02G337300.1	520868787	520875955	598	65.62	5.78	Nucleus	-0.393	none
**TaMAPKKK93**	**TaRaf53**	TraesCS5D02G018800.1	11301422	11304114	645	72.62	6.00	Nucleus	-0.296	yes
**TaMAPKKK94**	**TaRaf54**	TraesCS5B02G012000.1	11764765	11774531	1017	106.30	4.67	Cell membrane	0.122	yes
**TaMAPKKK95**	**TaRaf55**	TraesCS5B02G204900.1	373215862	373220653	439	48.20	9.72	Nucleus	-0.515	none
**TaMAPKKK96**	**TaRaf56**	TraesCS5B02G292000.2	477512642	477518372	788	88.84	8.46	Nucleus	-0.263	none
**TaMAPKKK97**	**TaRaf57**	TraesCS5B02G353800.1	533375142	533376026	225	25.29	6.52	Nucleus	-0.145	none
**TaMAPKKK98**	**TaRaf58**	TraesCS5D02G482000.1	519649924	519653802	418	45.49	8.00	Nucleus	-0.276	none
**TaMAPKKK99**	**TaRaf59**	TraesCS3A02G001500.1	1028128	1031945	365	40.72	9.31	Nucleus	-0.247	none
**TaMAPKKK100**	**TaRaf60**	TraesCS3A02G274000.1	502835637	502839127	601	67.35	9.31	Nucleus	-0.593	none
**TaMAPKKK101**	**TaRaf61**	TraesCS5D02G019400.1	11949643	11952687	1014	105.97	6.33	Cell membrane	0.123	yes

**TaMAPKKK102**	**TaRaf62**	TraesCS7D02G474700.1	587069445	587072872	425	47.20	6.43	Nucleus	-0.185	none
**TaMAPKKK103**	**TaRaf63**	TraesCS7A02G326700.1	474729873	474737049	536	60.15	5.48	Nucleus	-0.332	none
**TaMAPKKK104**	**TaRaf64**	TraesCS1D02G273600.1	367915349	367918308	681	73.38	6.00	Nucleus	-0.117	none
**TaMAPKK**	**TaRaf64-1**	TraesCS1B	492327989	492331022	678	73.33	6.38	Nucleus	-0.141	yes
**TaMAPKKK105**	**TaRaf65**	TraesCS3A02G045200.1	24205959	24209204	399	43.36	6.72	Nucleus	-0.193	none
**TaMAPKKK106**	**TaRaf66**	TraesCS4A02G456900.1	722287491	722294625	647	71.63	6.20	Cell membrane. Nucleus	-0.133	yes
**TaMAPKKK107**	**TaRaf67**	TraesCS7A02G032700.1	13832914	13835159	480	54.35	8.59	Cell membrane. Nucleus	-0.128	yes
**TaMAPKKK108**	**TaRaf68**	TraesCS3D02G023600.1	7496834	7501605	830	91.83	7.19	Chloroplast. Nucleus	-0.208	yes
**TaMAPKKK109**	**TaRaf69**	TraesCS2D02G219800.1	186995581	186997635	684	74.88	6.00	Cell membrane. Cytoplasm. Nucleus	-0.064	yes
**TaMAPKKK110**	**TaRaf70**	TraesCS4B02G289100.1	574169282	574171714	680	72.78	6.51	Nucleus	0.061	yes
**TaMAPKKK111**	**TaRaf71**	TraesCS3A02G315100.1	556354721	556361160	810	89.49	6.45	Nucleus	-0.054	yes
**TaMAPKKK112**	**TaRaf72**	TraesCS5D02G359500.1	438720779	438724678	846	93.64	6.60	Nucleus	-0.159	yes

**TaMAPKKK113**	**TaRaf73**	TraesCS3D02G108500.1	61696216	61699845	669	72.21	8.21	Nucleus	-0.039	yes
**TaMAPKKK115**	**TaRaf75**	TraesCS3A02G229800.1	429615380	429618754	720	74.97	8.84	Nucleus	-0.402	yes
**TaMAPKKK116**	**TaRaf76**	TraesCS1B02G454000.2	670025362	670028705	663	72.92	7.09	Nucleus	-0.105	yes
**TaMAPKKK117**	**TaRaf77**	TraesCS3D02G097000.1	48958881	48962702	648	69.64	8.85	Chloroplast. Nucleus	0.04	yes
**TaMAPKKK119**	**TaRaf79**	TraesCS2A02G577000.1	771236940	771242936	558	62.29	5.51	Nucleus	-0.427	none
**TaMAPKKK120**	**TaRaf80**	TraesCS4A02G317600.1	606796989	606806238	674	73.40	8.38	Chloroplast. Nucleus	-0.119	yes
**TaMAPKKK121**	**TaRaf81**	TraesCS2D02G066900.1	27998176	28002775	997	108.03	6.01	Cell membrane	0.119	yes
**TaMAPKKK122**	**TaRaf82**	TraesCS2A02G214000.1	199713870	199715915	681	74.62	6.07	Cytoplasm. Extracell. Nucleus	-0.088	yes
**TaMAPKKK123**	**TaRaf83**	TraesCS5D02G358700.1	438557604	438562390	839	92.71	6.63	Nucleus	-0.161	yes
**TaMAPKK**	**TaRaf83-1**	TraesCS5D	438454390	438456577	457	50.56	8.22	Nucleus	-0.216	yes
**TaMAPKKK125**	**TaRaf85**	TraesCS7D02G153800.1	101736527	101739826	826	90.93	6.86	Nucleus	-0.133	yes
**TaMAPKKK126**	**TaRaf86**	TraesCSU02G011500.1	15720917	15724380	644	72.97	6.33	Nucleus	0.023	yes
**TaMAPKKK127**	**TaRaf87**	TraesCS6D02G339600.1	438942402	438946634	928	104.30	5.77	Chloroplast. Cytoplasm. Nucleus	-0.412	none

**TaMAPKKK128**	**TaRaf88**	TraesCS1B02G446500.1	666535726	666539402	372	40.91	7.54	Nucleus	-0.281	none
**TaMAPKKK129**	**TaRaf89**	TraesCS6B02G320800.1	567847786	567851826	986	108.30	6.21	Cell membrane	0.086	yes
**TaMAPKKK130**	**TaRaf90**	TraesCS7A02G044600.1	20510405	20512958	405	46.16	6.25	Nucleus	-0.169	yes
**TaMAPKKK131**	**TaRaf91**	TraesCS6B02G215100.2	290710332	290718338	860	93.43	5.41	Nucleus	-0.228	none
**TaMAPKKK132**	**TaRaf92**	TraesCS5B02G353600.1	533033821	533038318	846	93.47	5.85	Nucleus	-0.136	yes
**TaMAPKKK133**	**TaRaf93**	TraesCS1D02G004300.1	2108471	2112601	656	68.93	9.64	Nucleus	-0.394	yes
**TaMAPKKK134**	**TaRaf94**	TraesCS5D02G547500.1	555555819	555559333	765	83.36	6.02	Cell membrane	0.191	yes
**TaMAPKKK135**	**TaRaf95**	TraesCS2D02G598800.1	650601306	650604027	499	55.24	5.84	Nucleus	-0.286	none
**TaMAPKKK136**	**TaRaf96**	TraesCS2B02G241600.1	245686727	245691716	675	72.70	5.94	Nucleus	0.015	yes
**TaMAPKKK137**	**TaRaf97**	TraesCS3D02G472000.1	574118271	574125655	746	80.84	5.82	Nucleus	-0.239	yes
**TaMAPKKK139**	**TaRaf99**	TraesCS3A02G493500.1	719931689	719937693	711	79.40	6.02	Cell membrane	-0.137	yes
**TaMAPKKK140**	**TaRaf100**	TraesCS5D02G097900.1	108527463	108535238	753	83.11	7.94	Nucleus	-0.646	none

**TaMAPKKK141**	**TaRaf101**	TraesCS7D02G000800.1	981342	987541	965	107.83	6.39	Cytoplasm. Nucleus	-0.301	yes
**TaMAPKKK142**	**TaRaf102**	TraesCS2A02G032200.1	14729078	14730418	323	36.46	5.40	Nucleus	-0.275	none
**TaMAPKKK144**	**TaRaf104**	TraesCS3D02G501100.1	590199543	590205678	713	79.61	5.82	Cell membrane. Nucleus	-0.134	yes
**TaMAPKKK145**	**TaRaf105**	TraesCS2B02G242300.1	246113761	246119395	669	72.81	6.05	Cell membrane. Nucleus	-0.096	yes
**TaMAPKKK146**	**TaRaf106**	TraesCS2B02G241400.1	245317184	245320539	667	72.23	6.12	Chloroplast. Nucleus	0.058	yes
**TaMAPKKK147**	**TaRaf107**	TraesCS2A02G216600.1	203325595	203329022	668	73.47	8.53	Nucleus	-0.128	yes
**TaMAPKKK148**	**TaRaf108**	TraesCS1A02G003900.1	2391500	2393497	341	39.38	6.23	Nucleus	-0.331	none
**TaMAPKKK150**	**TaRaf110**	TraesCS3B02G008600.2	4323030	4327039	686	71.89	6.26	Nucleus	-0.438	yes
**TaMAPKKK151**	**TaRaf111**	TraesCS3B02G123800.1	96812311	96816428	577	63.83	5.79	Nucleus	-0.202	none
**TaMAPKKK152**	**TaRaf112**	TraesCS3B02G259100.1	416805836	416810951	698	72.98	5.89	Nucleus	-0.449	yes
**TaMAPKKK153**	**TaRaf113**	TraesCS3B02G351800.1	561590530	561591713	302	33.43	6.25	Nucleus	0.002	none
**TaMAPKKK154**	**TaRaf114**	TraesCS3B02G478400.1	726770459	726780334	770	87.44	5.95	Nucleus	-0.318	none

**TaMAPKKK155**		**TaRaf115**	TraesCS6B02G217100.1	298820612	298821648	318	36.07	5.20	Nucleus	-0.025	none
**TaMAPKKK1**			TraesCS1A02G181900.1	328244901	328253761	714	79.25	6.11	Nucleus	-0.543	none
**TaMAPKKK2**			TraesCS1B02G199100.2	357209635	357218446	724	80.56	6.27	Nucleus	-0.537	none
**TaMAPKKK3**			TraesCS1D02G185000.2	255824893	255833850	731	81.45	6.30	Nucleus	-0.555	none
**TaMAPKKK4**			TraesCS2A02G233400.1	278445853	278467939	692	76.02	6.97	Nucleus	-0.665	none
**TaMAPKKK5**			TraesCS2B02G249900.1	259009416	259028116	692	76.08	7.00	Nucleus	-0.672	none
**TaMAPKKK6**			TraesCS2D02G232200.1	204990636	205004380	692	76.09	7.00	Nucleus	-0.654	none
**TaMAPKKK7**			TraesCS4B02G395600.1	670201548	670229484	629	69.54	9.51	Nucleus	-0.468	none
**TaMAPKKK8**			TraesCS4B02G398400.3	672296531	672306045	744	82.04	6.52	Nucleus	-0.471	none
**TaMAPKKK9**			TraesCS5A02G187400.1	389131980	389133955	518	56.50	5.80	Nucleus	-0.191	none
**TaMAPKKK10**			TraesCS5A02G392500.1	588731002	588738966	825	89.69	5.31	Nucleus	-0.504	none
**TaMAPKKK11**			TraesCS5A02G556400.5	707802354	707810757	742	82.08	6.89	Nucleus	-0.448	none

**TaMAPKKKK12**	TraesCS5B02G397300.1	574952105	574960822	858	93.17	5.31	Nucleus	-0.522	none
**TaMAPKKKK13**	TraesCS5D02G203600.1	308863403	308865114	518	56.49	5.75	Nucleus	-0.201	none
**TaMAPKKKK14**	TraesCS5D02G402300.1	468166467	468175209	822	89.42	5.24	Nucleus	-0.519	none
**TaMAPKKKK15**	TraesCS6A02G149900.1	133414193	133419353	543	61.18	6.83	Nucleus	-0.552	none
**TaMAPKKKK16**	TraesCS6A02G353400.1	585174093	585176268	348	38.89	8.97	Nucleus	-0.25	none
**TaMAPKKKK17**	TraesCS6A02G353500.1	585187497	585195069	672	74.09	6.39	Nucleus	-0.444	none
**TaMAPKKKK18**	TraesCS6B02G177800.1	195805196	195810462	542	61.16	7.02	Nucleus	-0.579	none
**TaMAPKKKK19**	TraesCS6B02G386100.1	660664103	660676384	695	76.96	6.31	Nucleus	-0.415	none
**TaMAPKKKK20**	TraesCS6D02G335800.1	436870500	436887069	695	76.92	6.26	Nucleus	-0.413	none
**TaMAPKKKK21**	TraesCS6D02G139200.1	108447173	108452128	509	57.60	6.71	Nucleus	-0.504	none
**TaMAPKKKK22**	TraesCS7A02G232300.1	203511879	203521492	700	77.96	6.18	Nucleus	-0.559	none
**TaMAPKKKK23**	TraesCS7B02G130700.1	157674223	157684150	700	77.91	6.11	Nucleus	-0.578	none

**TaMAPKKKK24**	TraesCS7D02G232400.1	193922055	193930244	709	78.76	5.99	Nucleus	-0.417	none
**TaMAPKKKK25**	TraesCSU02G115300.1	99866931	99876736	741	81.70	6.34	Nucleus	-0.486	none

1(A,B,D)chromosomes	**TaMAPK41**			TraesCS1A02G402400.2
	**TaMAPK42**			TraesCS1A02G415300.1
	**TaMAPK43**			TraesCS1A02G421000.1
	**TaMAPK11**			TraesCS1A02G086500.1
	**TaMAPK14**			TraesCS1A02G184500.1
	**TaMAPK27**			TraesCS1B02G104900.1
	**TaMAPK28**			TraesCS1B02G192600.3
	**TaMAPK29**			TraesCS1B02G431400.2
	**TaMAPK4**			TraesCS1D02G088000.2
	**TaMAPK5**			TraesCS1D02G422800.1
	**TaMAPK33**			TraesCS1D02G410100.1
	**TaMAPK34**			TraesCS1D02G428900.1

3(A,B,D)chromosomes	**TaMAPK39**			TraesCS3A02G242100.1
	**TaMAPK40**			TraesCS3A02G231700.1
	**TaMAPK16**			TraesCS3B02G270200.1
	**TaMAPK38**			TraesCS3B02G256700.1
	**TaMAPK8**			TraesCS3D02G225600.1
	**TaMAPK23**			TraesCS3D02G221700.1
	**TaMAPK24**			TraesCS3D02G242200.2

4(A,B,D)chromosomes	**TaMAPK31**			TraesCS4A02G434800.1
	**TaMAPK2**			TraesCS4A02G336800.2
	**TaMAPK3**			TraesCS4A02G106400.1
	**TaMAPK25**			TraesCS4D02G198600.1

5(A,B,D)chromosomes	**TaMAPK50**			TraesCS5B02G536500.1
	**TaMAPK26**			TraesCS5D02G534000.2

6(A,B,D)chromosomes	**TaMAPK35**			TraesCS6A02G099600.1
	**TaMAPK36**			TraesCS6A02G118100.1
	**TaMAPK37**			TraesCS6A02G269400.1
	**TaMAPK17**			TraesCS6B02G127800.1

6(A,B,D)chromosomes	**TaMAPK1**			TraesCS6B02G296700.2
	**TaMAPK10**			TraesCS6B02G146300.1
	**TaMAPK13**			TraesCS6D02G245500.3
	**TaMAPK44**			TraesCS6D02G082900.2

7(A,B,D)chromosomes	**TaMAPK19**			TraesCS7A02G111300.1
	**TaMAPK12**			TraesCS7A02G422500.1
	**TaMAPK30**			TraesCS7A02G335300.2
	**TaMAPK46**			TraesCS7A02G049000.1
	**TaMAPK47**			TraesCS7A02G029700.1
	**TaMAPK49**			TraesCS7A02G410700.2
	**TaMAPK52**			TraesCS7B02G246900.3
	**TaMAPK53**			TraesCS7B02G309900.1
	**TaMAPK54**			TraesCS7B02G322900.1
	**TaMAPK6**			TraesCS7B02G009200.1
	**TaMAPK7**			TraesCS7D02G342800.3
	**TaMAPK18**			TraesCS7D02G044100.1
	**TaMAPK20**			TraesCS7D02G403700.1
	**TaMAPK22**			TraesCS7D02G414900.1

3(A,B,D)chromosomes	**TaMAPKK9**			TraesCS3B02G066300.1

4(A,B,D)chromosomes	**TaMAPKK14**			TraesCS4A02G265900.1
	**TaMAPKK15**			TraesCS4A02G266000.1
	**TaMAPKK16**			TraesCS4A02G266100.1
	**TaMAPKK17**			TraesCS4A02G266200.1
	**TaMAPKK5**			TraesCS4B02G049000.1
	**TaMAPKK6**			TraesCS4B02G048100.1
	**TaMAPKK7**			TraesCS4B02G048600.1
	**TaMAPKK8**			TraesCS4B02G048900.1
	**TaMAPKK11**			TraesCS4D02G048800.1
	**TaMAPKK12**			TraesCS4D02G048500.1

5(A,B,D)chromosomes	**TaMAPKK4**			TraesCS5A02G122700.4
	**TaMAPKK2**			TraesCS5B02G565100.3
	**TaMAPKK18**			TraesCS5B02G122600.1
	**TaMAPKK3**			TraesCS5D02G130900.2
	**TaMAPKK13**			TraesCS5D02G549600.1

6(A,B,D)chromosomes	**TaMAPKK1**			TraesCS6D02G328800.1

2(A,B,D)chromosomes	**TaMAPKKK5**	**MEKK**	TaMEKK5	TraesCS2A02G199700.1
	**TaMAPKKK1**		TaMEKK1	TraesCS2A02G407600.1
	**TaMAPKKK28**		TaMEKK28	TraesCS2A02G095300.1
	**TaMAPKKK20**		TaMEKK20	TraesCS2A02G498000.3
	**TaMAPKKK27**		TaMEKK27	TraesCS2B02G110500.1
	**TaMAPKKK18**		TaMEKK18	TraesCS2B02G526200.3
	**TaMAPKKK26**		TaMEKK26	TraesCS2D02G093700.1

3(A,B,D)chromosomes	**TaMAPKKK7**		TaMEKK7	TraesCS3B02G289500.1
	**TaMAPKKK8**		TaMEKK8	TraesCS3B02G288100.1
	**TaMAPKKK9**		TaMEKK9	TraesCS3B02G288300.1

4(A,B,D)chromosomes	**TaMAPKKK12**		TaMEKK12	TraesCS4A02G093800.2
	**TaMAPKKK3**		TaMEKK3	TraesCS4B02G210600.2
	**TaMAPKKK2**		TaMEKK2	TraesCS4D02G027600.1
	**TaMAPKKK10**		TaMEKK10	TraesCS4D02G211300.2

5(A,B,D)chromosomes	**TaMAPKKK17**		TaMEKK17	TraesCS5A02G200800.1
	**TaMAPKKK22**		TaMEKK22	TraesCS5A02G392500.1
	**TaMAPKKK14**		TaMEKK14	TraesCS5A02G118200.1
	**TaMAPKKK15**		TaMEKK15	TraesCS5A02G463100.2
	**TaMAPKKK16**		TaMEKK16	TraesCS5B02G474500.1
	**TaMAPKKK24**		TaMEKK24	TraesCS5B02G199400.1

	**TaMAPKKK25**		TaMEKK25	TraesCS5B02G196400.1
	**TaMAPKKK11**		TaMEKK11	TraesCS5D02G475900.1
	**TaMAPKKK29**		TaMEKK29	TraesCS5D02G206500.1
6(A,B,D)chromosomes	**TaMAPKKK21**		TaMEKK21	TraesCS6A02G149900.1
	**TaMAPKKK4**		TaMEKK4	TraesCS6A02G245000.3
	**TaMAPKKK4-1**		TaMEKK4-1	TraesCS6B02G279300.1
	**TaMAPKKK23**		TaMEKK23	TraesCS6D02G139200.1
1(A,B,D)chromosomes	**TaMAPKKK38**	**ZIK**	TaZIK9	TraesCS1D02G026200.2
2(A,B,D)chromosomes	**TaMAPKKK32**		TaZIK3	TraesCS2A02G195900.2
	**TaMAPKKK36**		TaZIK7	TraesCS2B02G223600.1
	**TaMAPKKK37**		TaZIK8	TraesCS2B02G216800.1
	**TaMAPKKK34**		TaZIK5	TraesCS2D02G197600.1
5(A,B,D)chromosomes	**TaMAPKKK40**		TaZIK11	TraesCS5B02G146100.1
	**TaMAPKKK30**		TaZIK1	TraesCS5D02G145100.1
6(A,B,D)chromosomes	**TaMAPKKK39**		TaZIK10	TraesCS6A02G255100.2
	**TaMAPKKK33**		TaZIK4	TraesCS6B02G270400.1
	**TaMAPKKK31**		TaZIK2	TraesCS6D02G236400.1
1(A,B,D)chromosomes	**TaMAPKKK148**	**Raf**	**TaRaf108**	TraesCS1A02G003900.1
	**TaMAPKKK88**		**TaRaf48**	TraesCS1A02G422800.1
	**TaMAPKKK53**		**TaRaf13**	TraesCS1B02G372400.1
	**TaMAPKKK104-1**		**TaRaf64-1**	TraesCS1B02G283400.1
	**TaMAPKKK128**		**TaRaf88**	TraesCS1B02G446500.1
	**TaMAPKKK116**		**TaRaf76**	TraesCS1B02G454000.2
	**TaMAPKKK104**		**TaRaf64**	TraesCS1D02G273600.1
	**TaMAPKKK66**		**TaRaf26**	TraesCS1D02G273800.2
	**TaMAPKKK67**		**TaRaf27**	TraesCS1D02G360600.1
	**TaMAPKKK68**		**TaRaf28**	TraesCS1D02G431400.1

	**TaMAPKKK70**	**TaRaf30**	TraesCS1D02G423800.1
	**TaMAPKKK133**	**TaRaf93**	TraesCS1D02G004300.1
2(A,B,D)chromosomes	**TaMAPKKK119**	**TaRaf79**	TraesCS2A02G577000.1
	**TaMAPKKK80**	**TaRaf40**	TraesCS2A02G217600.1
	**TaMAPKKK59**	**TaRaf19**	TraesCS2A02G216900.1
	**TaMAPKKK60**	**TaRaf20**	TraesCS2A02G217000.1
	**TaMAPKKK147**	**TaRaf107**	TraesCS2A02G216600.1
	**TaMAPKKK142**	**TaRaf102**	TraesCS2A02G032200.1
	**TaMAPKKK122**	**TaRaf82**	TraesCS2A02G214000.1
	**TaMAPKKK145**	**TaRaf105**	TraesCS2B02G242300.1
	**TaMAPKKK146**	**TaRaf106**	TraesCS2B02G241400.1
	**TaMAPKKK136**	**TaRaf96**	TraesCS2B02G241600.1
	**TaMAPKKK69**	**TaRaf29**	TraesCS2D02G588200.1
	**TaMAPKKK135**	**TaRaf95**	TraesCS2D02G598800.1
	**TaMAPKKK44**	**TaRaf4**	TraesCS2D02G003900.1
	**TaMAPKKK46**	**TaRaf6**	TraesCS2D02G050700.1
	**TaMAPKKK109**	**TaRaf69**	TraesCS2D02G219800.1
	**TaMAPKKK121**	**TaRaf81**	TraesCS2D02G066900.1
	**TaMAPKKK111**	**TaRaf71**	TraesCS3A02G315100.1
	**TaMAPKKK105**	**TaRaf65**	TraesCS3A02G045200.1
	**TaMAPKKK99**	**TaRaf59**	TraesCS3A02G001500.1
	**TaMAPKKK100**	**TaRaf60**	TraesCS3A02G274000.1
	**TaMAPKKK115**	**TaRaf75**	TraesCS3A02G229800.1
	**TaMAPKKK79**	**TaRaf39**	TraesCS3A02G493900.1
	**TaMAPKKK72**	**TaRaf32**	TraesCS3A02G003900.1
	**TaMAPKKK73**	**TaRaf33**	TraesCS3A02G039200.1
	**TaMAPKKK74**	**TaRaf34**	TraesCS3A02G039400.1
	**TaMAPKKK75**	**TaRaf35**	TraesCS3A02G096500.1
	**TaMAPKKK76**	**TaRaf36**	TraesCS3A02G246100.1
	**TaMAPKKK56**	**TaRaf16**	TraesCS3A02G039100.1
	**TaMAPKKK139**	**TaRaf99**	TraesCS3A02G493500.1

3(A,B,D)chromosomes	**TaMAPKKK150**	**TaRaf110**	TraesCS3B02G008600.2
	**TaMAPKKK151**	**TaRaf111**	TraesCS3B02G123800.1
	**TaMAPKKK152**	**TaRaf112**	TraesCS3B02G259100.1
	**TaMAPKKK153**	**TaRaf113**	TraesCS3B02G351800.1
	**TaMAPKKK154**	**TaRaf114**	TraesCS3B02G478400.1
	**TaMAPKKK43**	**TaRaf3**	TraesCS3B02G110300.1
	**TaMAPKKK58**	**TaRaf18**	TraesCS3B02G259800.1
	**TaMAPKKK144**	**TaRaf104**	TraesCS3D02G501100.1
	**TaMAPKKK137**	**TaRaf97**	TraesCS3D02G472000.1
	**TaMAPKKK45**	**TaRaf5**	TraesCS3D02G273200.1
	**TaMAPKKK57**	**TaRaf17**	TraesCS3D02G040600.1
	**TaMAPKKK78**	**TaRaf38**	TraesCS3D02G501400.1
	**TaMAPKKK113**	**TaRaf73**	TraesCS3D02G108500.1
	**TaMAPKKK117**	**TaRaf77**	TraesCS3D02G097000.1
	**TaMAPKKK108**	**TaRaf68**	TraesCS3D02G023600.1
	**TaMAPKKK106**	**TaRaf66**	TraesCS4A02G456900.1
	**TaMAPKKK62**	**TaRaf22**	TraesCS4A02G313900.1
	**TaMAPKKK63**	**TaRaf23**	TraesCS4A02G383000.1
4(A,B,D)chromosomes	**TaMAPKKK64**	**TaRaf24**	TraesCS4A02G465900.2
	**TaMAPKKK64-1**	**TaRaf24-1**	TraesCS4A02G464700.1
	**TaMAPKKK65**	**TaRaf25**	TraesCS4A02G465000.1
	**TaMAPKKK91**	**TaRaf51**	TraesCS4D02G089300.1
	**TaMAPKKK81**	**TaRaf41**	TraesCS5A02G292500.1
	**TaMAPKKK83**	**TaRaf43**	TraesCS5A02G351500.1
	**TaMAPKKK84**	**TaRaf44**	TraesCS5A02G351000.1
	**TaMAPKKK85**	**TaRaf45**	TraesCS5A02G352000.1
	**TaMAPKKK92**	**TaRaf52**	TraesCS5B02G337300.1
	**TaMAPKKK94**	**TaRaf54**	TraesCS5B02G012000.1
	**TaMAPKKK95**	**TaRaf55**	TraesCS5B02G204900.1
	**TaMAPKKK96**	**TaRaf56**	TraesCS5B02G292000.2
5(A,B,D)chromosomes	**TaMAPKKK97**	**TaRaf57**	TraesCS5B02G353800.1

5(A,B,D)chromosomes	**TaMAPKKK132**	**TaRaf92**	TraesCS5B02G353600.1
	**TaMAPKKK98**	**TaRaf58**	TraesCS5D02G482000.1
	**TaMAPKKK101**	**TaRaf61**	TraesCS5D02G019400.1
	**TaMAPKKK93**	**TaRaf53**	TraesCS5D02G018800.1
	**TaMAPKKK86**	**TaRaf46**	TraesCS5D02G386800.1
	**TaMAPKKK123**	**TaRaf83**	TraesCS5D02G358700.1
	**TaMAPKKK123-1**	**TaRaf83-1**	TraesCS5D02G358200.1
	**TaMAPKKK134**	**TaRaf94**	TraesCS5D02G547500.1
	**TaMAPKKK140**	**TaRaf100**	TraesCS5D02G097900.1
6(A,B,D)chromosomes	**TaMAPKKK71**	**TaRaf31**	TraesCS6A02G004500.1
	**TaMAPKKK41**	**TaRaf1**	TraesCS6A02G172600.1
	**TaMAPKKK155**	**TaRaf115**	TraesCS6B02G217100.1
	**TaMAPKKK131**	**TaRaf91**	TraesCS6B02G215100.2
	**TaMAPKKK129**	**TaRaf89**	TraesCS6B02G320800.1
	**TaMAPKKK127**	**TaRaf87**	TraesCS6D02G339600.1
7(A,B,D)chromosomes	**TaMAPKKK90**	**TaRaf50**	TraesCS7A02G152100.1
	**TaMAPKKK130**	**TaRaf90**	TraesCS7A02G044600.1
	**TaMAPKKK103**	**TaRaf63**	TraesCS7A02G326700.1
	**TaMAPKKK107**	**TaRaf67**	TraesCS7A02G032700.1
	**TaMAPKKK102**	**TaRaf62**	TraesCS7D02G474700.1
	**TaMAPKKK47**	**TaRaf7**	TraesCS7D02G022200.1
	**TaMAPKKK48**	**TaRaf8**	TraesCS7D02G079100.1
	**TaMAPKKK50**	**TaRaf10**	TraesCS7D02G099200.2
	**TaMAPKKK51**	**TaRaf11**	TraesCS7D02G230200.1
	**TaMAPKKK52**	**TaRaf12**	TraesCS7D02G230500.1
	**TaMAPKKK61**	**TaRaf21**	TraesCS7D02G384700.1
	**TaMAPKKK125**	**TaRaf85**	TraesCS7D02G153800.1
	**TaMAPKKK54**	**TaRaf14**	TraesCS7D02G503600.1
	**TaMAPKKK141**	**TaRaf101**	TraesCS7D02G000800.1
	**TaMAPKKK82**	**TaRaf42**	TraesCSU02G072500.1

Un	**TaMAPKKK42**		**TaRaf2**	TraesCSU02G203100.1
	**TaMAPKKK126**		**TaRaf86**	TraesCSU02G011500.1

1(A,B,D)chromosomes	**TaMAPKKKK1**			TraesCS1A02G181900.1
	**TaMAPKKKK2**			TraesCS1B02G199100.2
	**TaMAPKKKK3**			TraesCS1D02G185000.2

2(A,B,D)chromosomes	**TaMAPKKKK4**			TraesCS2A02G233400.1
	**TaMAPKKKK5**			TraesCS2B02G249900.1
	**TaMAPKKKK6**			TraesCS2D02G232200.1

4(A,B,D)chromosomes	**TaMAPKKKK7**			TraesCS4B02G395600.1
	**TaMAPKKKK8**			TraesCS4B02G398400.3

5(A,B,D)chromosomes	**TaMAPKKKK9**			TraesCS5A02G187400.1
	**TaMAPKKKK10**			TraesCS5A02G392500.1
	**TaMAPKKKK11**			TraesCS5A02G556400.5
	**TaMAPKKKK12**			TraesCS5B02G397300.1
	**TaMAPKKKK13**			TraesCS5D02G203600.1
	**TaMAPKKKK14**			TraesCS5D02G402300.1

6(A,B,D)chromosomes	**TaMAPKKKK15**			TraesCS6A02G149900.1
	**TaMAPKKKK16**			TraesCS6A02G353400.1
	**TaMAPKKKK17**			TraesCS6A02G353500.1
	**TaMAPKKKK18**			TraesCS6B02G177800.1
	**TaMAPKKKK19**			TraesCS6B02G386100.1
	**TaMAPKKKK20**			TraesCS6D02G335800.1
	**TaMAPKKKK21**			TraesCS6D02G139200.1

7(A,B,D)chromosomes	**TaMAPKKKK22**			TraesCS7A02G232300.1
	**TaMAPKKKK23**			TraesCS7B02G130700.1
	**TaMAPKKKK24**			TraesCS7D02G232400.1
	**TaMAPKKKK25**			TraesCSU02G115300.1

RefSeq version			IWGSC version		TGACv1 version	
**TaMAPK1**		TraesCS6B02G296700.2	**TaMAPK1**	Traes_6BL_BF59BFB93.2		
**TaMAPK2**		TraesCS4A02G336800.2	**TaMAPK2**	Traes_4AL_6F3D0ACCA.1		
**TaMAPK3**		TraesCS4A02G106400.1	**TaMAPK3**	Traes_7AL_F5620757F.2	**TaMAPK3**	TRIAE_CS42_4AS_TGACv1_306486_AA1008960/4BL_TGACv1_320270_AA1033300/4DL_TGACv1_344691_AA1148960
**TaMAPK4**		TraesCS1D02G088000.2	**TaMAPK4**	Traes_1DS_66BD773BF.2	**TaMAPK4**	TRIAE_CS42_1AL_TGACv1_000118_AA0003960/1BL_TGACv1_030274_AA0084790/1DL_TGACv1_061983_AA0206840
**TaMAPK5**		TraesCS1D02G422800.1	**TaMAPK5**	Traes_1DL_52D511CDD.1		
**TaMAPK6**		TraesCS7B02G009200.1	**TaMAPK6**	Traes_1BL_7FA80EF00.2	**TaMAPK6**	TRIAE_CS42_7AS_TGACv1_570369_AA1834630/7BS_TGACv1_593766_AA1954300/7DS_TGACv1_622461_AA2040040
**TaMAPK7**		TraesCS7D02G342800.3	**TaMAPK7**	Traes_7DL_FE0ECD387.2	**TaMAPK7**	TRIAE_CS42_7AL_TGACv1_558385_AA1792950/7BL_TGACv1_576902_AA1859240/7DL_TGACv1_603035_AA1974260/7DL_TGACv1_603254_AA1979200
**TaMAPK8**		TraesCS3D02G225600.1	**TaMAPK8**	Traes_3DL_5D82311EA.1		
**TaMAPK10**		TraesCS6B02G146300.1	**TaMAPK10**	Traes_6BS_17C1E5829.1		
**TaMAPK11**		TraesCS1A02G086500.1	**TaMAPK11**	Traes_1AS_C9CFD7AC8.1	**TaMAPK11**	TRIAE_CS42_7AL_TGACv1_555982_AA1751700/7BL_TGACv1_580661_AA1914980/7DL_TGACv1_603600_AA1986180
**TaMAPK12**		TraesCS7A02G422500.1	**TaMAPK12**	Traes_7AL_4AF13CC8B.2		
**TaMAPK13**		TraesCS6D02G245500.3	**TaMAPK13**	Traes_6DL_F48A5E31E.2		
**TaMAPK14**		TraesCS1A02G184500.1	**TaMAPK14**	Traes_1AL_89DDB4243.1	**TaMAPK14**	TRIAE_CS42_6AS_TGACv1_487866_AA1573320/6BS_TGACv1_514340_AA1658600/6DS_TGACv1_544171_AA1747030
**TaMAPK16**		TraesCS3B02G270200.1	**TaMAPK16**	TRAES3BF058500020CFD	**TaMAPK16**	TRIAE_CS42_1AS_TGACv1_020601_AA0078640/1BS_TGACv1_049733_AA0160590/U_TGACv1_642759_AA2122920

**TaMAPK17**		TraesCS6B02G127800.1	**TaMAPK17**	Traes_6BS_2D0054D1F.2	**TaMAPK17**	TRIAE_CS42_7AL_TGACv1_556562_AA1765430/U_TGACv1_641980_AA2108810/7DL_TGACv1_604680_AA2000630
**TaMAPK18**		TraesCS7D02G044100.1	**TaMAPK18**	Traes_7DS_C268073F4.3		
**TaMAPK19**		TraesCS7A02G111300.1	**TaMAPK19**	Traes_7DS_1D8A8BFA2.1		
**TaMAPK20**		TraesCS7D02G403700.1	**TaMAPK20**	Traes_7DL_FB75EA9C3.2	**TaMAPK20**	TaMAPK20-1: TRIAE_CS42_3AL_TGACv1_194002_AA0624350/3B_TGACv1_224428_AA0796670/3DL_TGACv1_253300_AA0894090. TaMAPK20-2: TRIAE_CS42_1AL_TGACv1_002534_AA0042880/1BL_TGACv1_030959_AA0104540/1DL_TGACv1_063491_AA0227870. TaMAPK20-3: TRIAE_CS42_7AL_TGACv1_557823_AA1786610/7BL_TGACv1_577050_AA1864020/7DL_TGACv1_604962_AA2003380. TaMAPK20-4: TRIAE_CS42_3AL_TGACv1_195023_AA0643580/3B_TGACv1_225288_AA0806810/3DL_TGACv1_250331_AA0866300. TaMAPK20-5: TRIAE_CS42_1AL_TGACv1_001596_AA0032670/1BL_TGACv1_032512_AA0130820/U_TGACv1_643598_AA2133390
**TaMAPK22**		TraesCS7D02G414900.1	**TaMAPK22**	Traes_7DL_73DF29BF0.1		
**TaMAPK23**		TraesCS3D02G221700.1	**TaMAPK23**	Traes_3DL_281B6BCBF.2		
**TaMAPK24**		TraesCS3D02G242200.2	**TaMAPK24**	Traes_3DL_38B762939.1	**TaMAPK24**	TRIAE_CS42_U_TGACv1_640751_AA2072490 & AA2072480/BS_TGACv1_513631_AA1646180/6DS_TGACv1_543835_AA1744530
**TaMAPK25**		TraesCS4D02G198600.1	**TaMAPK25**	Traes_4DL_15045954F.1	**TaMAPK25**	TRIAE_CS42_5AL_TGACv1_375843_AA1227850/5BL_TGACv1_405765_AA1334990
**TaMAPK26**		TraesCS5D02G534000.2	**TaMAPK26**	Traes_5DL_8DC610F26.1		

**TaMAPK27**		TraesCS1B02G104900.1	**TaMAPK27**	Traes_1BS_26C55B2B1.1		
**TaMAPK28**		TraesCS1B02G192600.3	**TaMAPK28**	Traes_1BL_C5CD09285.1		
**TaMAPK29**		TraesCS1B02G431400.2	**TaMAPK29**	Traes_1BL_9360D8CEC.1		
**TaMAPK30**		TraesCS7A02G335300.2	**TaMAPK30**	Traes_4AS_5015DF7A2.1		
**TaMAPK31**		TraesCS4A02G434800.1	**TaMAPK31**	Traes_4AL_E432524A0.2		
**TaMAPK33**		TraesCS1D02G410100.1	**TaMAPK33**	Traes_1DL_8C6B737E9.1		
**TaMAPK34**		TraesCS1D02G428900.1	**TaMAPK34**	Traes_1DL_73AAC8631.1		
**TaMAPK35**		TraesCS6A02G099600.1	**TaMAPK35**	Traes_6AS_50BE5D59F.1		
**TaMAPK36**		TraesCS6A02G118100.1	**TaMAPK36**	Traes_6AS_8225741A6.2		
**TaMAPK37**		TraesCS6A02G269400.1	**TaMAPK37**	Traes_6AL_BE97161B9.2		
**TaMAPK38**		TraesCS3B02G256700.1	**TaMAPK38**	Traes_3AL_F88B0A8E6.1		
**TaMAPK39**		TraesCS3A02G242100.1	**TaMAPK39**	Traes_3AL_669FE0293.1		
**TaMAPK40**		TraesCS3A02G231700.1	**TaMAPK40**	Traes_3AL_532CA9EE7.1		
**TaMAPK41**		TraesCS1A02G402400.2	**TaMAPK41**	Traes_1AL_30C4B017F.1		
**TaMAPK42**		TraesCS1A02G415300.1	**TaMAPK42**	Traes_1AL_14DCD6020.1		
**TaMAPK43**		TraesCS1A02G421000.1	**TaMAPK43**	Traes_1AL_A7173BBAE.1		
**TaMAPK44**		TraesCS6D02G082900.2	**TaMAPK44**	Traes_6DS_6424C38F2.2		

**TaMAPK45**		TraesCS6D02G108100.1	**TaMAPK45**	Traes_6DS_A61864DB2.2		
**TaMAPK46**		TraesCS7A02G049000.1	**TaMAPK46**	Traes_7AS_E1135D559.1		
**TaMAPK47**		TraesCS7A02G029700.1	**TaMAPK47**	Traes_7AS_71B4C13A6.1		
**TaMAPK49**		TraesCS7A02G410700.2	**TaMAPK49**	Traes_7AL_81A545A54.2		
**TaMAPK50**		TraesCS5B02G536500.1	**TaMAPK50**	Traes_5BL_A39EC2FCD.1		
**TaMAPK52**		TraesCS7B02G246900.3	**TaMAPK52**	Traes_7BL_44A22C7FC.2		
**TaMAPK53**		TraesCS7B02G309900.1	**TaMAPK53**	Traes_7BL_992E1F443.4		
**TaMAPK54**		TraesCS7B02G322900.1	**TaMAPK54**	Traes_7BL_95B1705A2.2		
**TaMAPKK1**		TraesCS6D02G328800.1	**TaMAPKK1**	Traes_6DL_8D2D914D7.2	**TaMKK1**	TaMKK1-1: TRIAE_CS42_4AL_TGACv1_290148_AA0982230/7AS_TGACv1_570427_AA1835590/7DS_TGACv1_622118_AA2033130. TaMKK1-2: TRIAE_CS42_6AS_TGACv1_485693_AA1550190/6BS_TGACv1_513382_AA1639520/U_TGACv1_644387_AA2139180. TaMKK1-3: TRIAE_CS42_7DS_TGACv1_623511_AA2053970
**TaMAPKK2**		TraesCS5B02G565100.3	**TaMAPKK2**	Traes_5BL_BB574E77D.2		
**TaMAPKK3**		TraesCS5D02G130900.2	**TaMAPKK3**	Traes_5DL_0886CB561.2	**TaMKK3**	TaMKK3-1: TRIAE_CS42_3B_TGACv1_228257_AA0826360. TaMKK3-2: TRIAE_CS42_5BL_TGACv1_405566_AA1330480/5DL_TGACv1_432926_AA1394740. TaMKK3-3: TRIAE_CS42_5AL_TGACv1_373997_AA1187200/5BL_TGACv1_405070_AA1319320/5DL_TGACv1_435057_AA1445420
**TaMAPKK4**		TraesCS5A02G122700.4	**TaMAPKK4**	Traes_5AL_5EFCEDAFB.2	**TaMKK4**	TRIAE_CS42_6AL_TGACv1_472053_AA1517260/6BL_TGACv1_502626_AA1625820/6DL_TGACv1_528809_AA1716310

**TaMAPKK5**		TraesCS4B02G049000.1	**TaMAPKK5**	Traes_4BS_EE048CC01.1	**TaMKK5**	TRIAE_CS42_7AS_TGACv1_572257_AA1851770/7BS_TGACv1_592906_AA1946090/7DS_TGACv1_623332_AA2052160
**TaMAPKK6**		TraesCS4B02G048100.1	**TaMAPKK6**	Traes_4BS_59F3F68D1.1	**TaMKK6**	TRIAE_CS42_4AL_TGACv1_290485_AA0985860/4BL_TGACv1_320624_AA1044950/4DL_TGACv1_342786_AA1122060
**TaMAPKK7**		TraesCS4B02G048600.1	**TaMAPKK7**	Traes_4BS_84D913A2E.1		
**TaMAPKK8**		TraesCS4B02G048900.1	**TaMAPKK8**	Traes_4BS_BDB56BC90.1		
**TaMAPKK9**		TraesCS3B02G066300.1	**TaMAPKK9**	TRAES3BF024700110CFD		
**TaMAPKK11**		TraesCS4D02G048800.1	**TaMAPKK11**	Traes_4DS_7F2F2671B.2		
**TaMAPKK12**		TraesCS4D02G048500.1	**TaMAPKK12**	Traes_4DS_7A016C2E4.1		
**TaMAPKK13**		TraesCS5D02G549600.1	**TaMAPKK13**	Traes_5DL_F89F21E65.1		
**TaMAPKK14**		TraesCS4A02G265900.1	**TaMAPKK14**	Traes_4AL_75E7BE9EE.1		
**TaMAPKK15**		TraesCS4A02G266000.1	**TaMAPKK15**	Traes_4AL_429683D72.1		
**TaMAPKK16**		TraesCS4A02G266100.1	**TaMAPKK16**	Traes_4AL_B7C432896.2		
**TaMAPKK17**		TraesCS4A02G266200.1	**TaMAPKK17**	Traes_4AL_84DFF6A541.1		
**TaMAPKK18**		TraesCS5B02G122600.1	**TaMAPKK18**	Traes_5BL_EC9896AB3.2		
**TaMAPKKK1**	TaMEKK1	TraesCS2A02G407600.1	TaMEKK1	Traes_2BL_23D01E7F4		
**TaMAPKKK2**	TaMEKK2	TraesCS4D02G027600.1	TaMEKK2	Traes_4DS_63F7CF3CE		
**TaMAPKKK3**	TaMEKK3	TraesCS4B02G210600.2	TaMEKK3	Traes_4BL_A7AE389EE		
**TaMAPKKK4**	TaMEKK4	TraesCS6A02G245000.3	TaMEKK4	Traes_6BL_93505FEAF		

**TaMAPKKK4-1**	TaMEKK4-1	TraesCS6B02 G279300.1				
**TaMAPKKK5**	TaMEKK5	TraesCS2A02 G199700.1	TaMEKK5	Traes_2AS_6D A49285E		
**TaMAPKKK7**	TaMEKK7	TraesCS3B02 G289500.1	TaMEKK7	TRAES3BF169 900020CFD_g		
**TaMAPKKK8**	TaMEKK8	TraesCS3B02 G288100.1	TaMEKK8	TRAES3BF036 800120CFD_g		
**TaMAPKKK9**	TaMEKK9	TraesCS3B02 G288300.1	TaMEKK9	TRAES3BF036 800100CFD_g		
**TaMAPKKK10**	TaMEKK10	TraesCS4D02 G211300.2	TaMEKK10	Traes_4DL_94 E10E6EB		
**TaMAPKKK11**	TaMEKK11	TraesCS5D02 G475900.1	TaMEKK11	Traes_5DL_A DFFAE33D		
**TaMAPKKK12**	TaMEKK12	TraesCS4A02 G093800.2	TaMEKK12	Traes_4AS_DF 85CBD39		
**TaMAPKKK14**	TaMEKK14	TraesCS5A02 G118200.1	TaMEKK14	Traes_5AS_9A 8A9187C		
**TaMAPKKK15**	TaMEKK15	TraesCS5A02 G463100.2	TaMEKK15	Traes_5AL_DE DF36AD2		
**TaMAPKKK16**	TaMEKK16	TraesCS5B02 G474500.1	TaMEKK16	Traes_5BL_35 A6B4387		
**TaMAPKKK17**	TaMEKK17	TraesCS5A02 G200800.1	TaMEKK17	Traes_5AL_4D 0919BA1		
**TaMAPKKK18**	TaMEKK18	TraesCS2B02 G526200.3	TaMEKK18	Traes_2BL_84 B12F4F8		
**TaMAPKKK20**	TaMEKK20	TraesCS2A02 G498000.3	TaMEKK20	Traes_2AL_66 079157A		
**TaMAPKKK21**	TaMEKK21	TraesCS6A02 G149900.1	TaMEKK21	Traes_6AS_E6 90A27CA		
**TaMAPKKK22**	TaMEKK22	TraesCS5A02 G392500.1	TaMEKK22	Traes_5AL_F9 C2BEAF3		
**TaMAPKKK23**	TaMEKK23	TraesCS6D02 G139200.1	TaMEKK23	Traes_6DS_18 5723D1E		

**TaMAPKKK24**	TaMEKK24	TraesCS5B02 G199400.1	TaMEKK24	Traes_5BL_3E FFD8013		
**TaMAPKKK25**	TaMEKK25	TraesCS5B02 G196400.1	TaMEKK25	Traes_5BL_38 DB82ACF		
**TaMAPKKK26**	TaMEKK26	TraesCS2D02 G093700.1	TaMEKK26	Traes_2DS_12 2AEE879		
**TaMAPKKK27**	TaMEKK27	TraesCS2B02 G110500.1	TaMEKK27	Traes_2BS_850 6C57C5		
**TaMAPKKK28**	TaMEKK28	TraesCS2A02 G095300.1	TaMEKK28	Traes_2AS_F0 521C4F2		
**TaMAPKKK29**	TaMEKK29	TraesCS5D02 G206500.1	TaMEKK29	Traes_5DL_24 3735D6C		
**TaMAPKKK30**	TaZIK1	TraesCS5D02 G145100.1	TaZIK1	Traes_5DL_98 24E97A8		
**TaMAPKKK31**	TaZIK2	TraesCS6D02 G236400.1	TaZIK2	Traes_6DL_F7 0F83614		
**TaMAPKKK32**	TaZIK3	TraesCS2A02 G195900.2	TaZIK3	Traes_2AS_2B 84A0A98		
**TaMAPKKK33**	TaZIK4	TraesCS6B02 G270400.1	TaZIK4	Traes_6BL_4A 17F7221		
**TaMAPKKK34**	TaZIK5	TraesCS2D02 G197600.1	TaZIK5	Traes_2DS_AA 3E486F3		
**TaMAPKKK36**	TaZIK7	TraesCS2B02 G223600.1	TaZIK7	Traes_2BS_182 64AA5C		
**TaMAPKKK37**	TaZIK8	TraesCS2B02 G216800.1	TaZIK8	Traes_2BS_1E 887CFE5		
**TaMAPKKK38**	TaZIK9	TraesCS1D02 G026200.2	TaZIK9	Traes_1DS_34 EFDA767		
**TaMAPKKK39**	TaZIK10	TraesCS6A02 G255100.2	TaZIK10	Traes_6AL_48 165ABE5		
**TaMAPKKK40**	TaZIK11	TraesCS5B02 G146100.1	TaZIK11	Traes_5BL_40 02B5518		
**TaMAPKKK41**	**TaRaf1**	TraesCS6A02 G172600.1	TaRaf1	Traes_6DS_D8 750EB5A		

**TaMAPKKK42**	**TaRaf2**	TraesCSU02 G203100.1	TaRaf2	Traes_2BL_4C AF2C184		
**TaMAPKKK43**	**TaRaf3**	TraesCS3B02 G110300.1	TaRaf3	Traes_6BL_01 E6CE316		
**TaMAPKKK44**	**TaRaf4**	TraesCS2D02 G003900.1	TaRaf4	Traes_2DS_DF E006BB6		
**TaMAPKKK45**	**TaRaf5**	TraesCS3D02 G273200.1	TaRaf5	Traes_3DL_CF CA7AA6B		
**TaMAPKKK46**	**TaRaf6**	TraesCS2D02 G050700.1	TaRaf6	Traes_2DS_0B FF3B23D		
**TaMAPKKK47**	**TaRaf7**	TraesCS7D02 G022200.1	TaRaf7	Traes_7DS_36 1EC0618		
**TaMAPKKK48**	**TaRaf8**	TraesCS7D02 G079100.1	TaRaf8	Traes_7DS_A3 EB5BFEB		
**TaMAPKKK50**	**TaRaf10**	TraesCS7D02 G099200.2	TaRaf10	Traes_7DS_D5 6FBFFD4		
**TaMAPKKK51**	**TaRaf11**	TraesCS7D02 G230200.1	TaRaf11	Traes_7DS_5A 97B2141		
**TaMAPKKK52**	**TaRaf12**	TraesCS7D02 G230500.1	TaRaf12	Traes_7DS_34 2F25C32		
**TaMAPKKK53**	**TaRaf13**	TraesCS1B02 G372400.1	TaRaf13	Traes_1BL_C9 B36DE76		
**TaMAPKKK54**	**TaRaf14**	TraesCS7D02 G503600.1	TaRaf14	Traes_7DL_F0 110933B		
**TaMAPKKK56**	**TaRaf16**	TraesCS3A02 G039100.1	TaRaf16	Traes_3DS_4E 61EE6EA		
**TaMAPKKK57**	**TaRaf17**	TraesCS3D02 G040600.1	TaRaf17	Traes_3DS_68 01BD0D2		
**TaMAPKKK58**	**TaRaf18**	TraesCS3B02 G259800.1	TaRaf18	Traes_3DL_B2 8036C5B		
**TaMAPKKK59**	**TaRaf19**	TraesCS2A02 G216900.1	TaRaf19	Traes_2AS_92 19695D6		
**TaMAPKKK60**	**TaRaf20**	TraesCS2A02 G217000.1	TaRaf20	Traes_2AS_79 A94F84A		

**TaMAPKKK61**	**TaRaf21**	TraesCS7D02 G384700.1	TaRaf21	Traes_7DL_70 5BA7CDD		
**TaMAPKKK62**	**TaRaf22**	TraesCS4A02 G313900.1	TaRaf22	Traes_4AL_1C 557F688		
**TaMAPKKK63**	**TaRaf23**	TraesCS4A02 G383000.1	TaRaf23	Traes_4AL_06 A8F8B8F		
**TaMAPKKK64**	**TaRaf24**	TraesCS4A02 G465900.2	TaRaf24	Traes_4AL_FE FC21AAB		
**TaMAPKKK64-1**	**TaRaf24-1**	TraesCS4A02 G464700.1				
**TaMAPKKK65**	**TaRaf25**	TraesCS4A02 G465000.1	TaRaf25	Traes_4AL_C2 17A20A1		
**TaMAPKKK66**	**TaRaf26**	TraesCS1D02 G273800.2	TaRaf26	Traes_1DL_FB 90601E7		
**TaMAPKKK67**	**TaRaf27**	TraesCS1D02 G360600.1	TaRaf27	Traes_1DL_F4 9D0E56A		
**TaMAPKKK68**	**TaRaf28**	TraesCS1D02 G431400.1	TaRaf28	Traes_1DL_A0 FB3E1D3		
**TaMAPKKK69**	**TaRaf29**	TraesCS2D02 G588200.1	TaRaf29	Traes_2DL_C5 A0BDC60		
**TaMAPKKK70**	**TaRaf30**	TraesCS1D02 G423800.1	TaRaf30	Traes_1DL_56 B195A26		
**TaMAPKKK71**	**TaRaf31**	TraesCS6A02 G004500.1	TaRaf31	Traes_6AS_00 6C344A3		
**TaMAPKKK72**	**TaRaf32**	TraesCS3A02 G003900.1	TaRaf32	Traes_3AS_A2 CECBF17		
**TaMAPKKK73**	**TaRaf33**	TraesCS3A02 G039200.1	TaRaf33	Traes_3AS_76 9E90DDD		
**TaMAPKKK74**	**TaRaf34**	TraesCS3A02 G039400.1	TaRaf34	Traes_3AS_5A F26B2FC		
**TaMAPKKK75**	**TaRaf35**	TraesCS3A02 G096500.1	TaRaf35	Traes_3AS_A5 42EC6F6		
**TaMAPKKK76**	**TaRaf36**	TraesCS3A02 G246100.1	TaRaf36	Traes_3AL_7F 6E774BB		

**TaMAPKKK78**	**TaRaf38**	TraesCS3D02G501400.1	TaRaf38	Traes_3AL_60BB7086F		
**TaMAPKKK79**	**TaRaf39**	TraesCS3A02G493900.1	TaRaf39	Traes_3AL_F384515F5		
**TaMAPKKK80**	**TaRaf40**	TraesCS2A02G217600.1	TaRaf40	Traes_2AS_0C8932B8E		
**TaMAPKKK81**	**TaRaf41**	TraesCS5A02G292500.1	TaRaf41	Traes_5AL_3FE725FD4		
**TaMAPKKK82**	**TaRaf42**	TraesCSU02G072500.1	TaRaf42	Traes_5AL_A236B0387		
**TaMAPKKK83**	**TaRaf43**	TraesCS5A02G351500.1	TaRaf43	Traes_5AL_CDD4A02E7		
**TaMAPKKK84**	**TaRaf44**	TraesCS5A02G351000.1	TaRaf44	Traes_5AL_13784C39B		
**TaMAPKKK85**	**TaRaf45**	TraesCS5A02G352000.1	TaRaf45	Traes_5AL_68C659562		
**TaMAPKKK86**	**TaRaf46**	TraesCS5D02G386800.1	TaRaf46	Traes_5AL_7B1C0342F		
**TaMAPKKK88**	**TaRaf48**	TraesCS1A02G422800.1	TaRaf48	Traes_1AL_C21696173		
**TaMAPKKK90**	**TaRaf50**	TraesCS7A02G152100.1	TaRaf50	Traes_7AS_81545C211		
**TaMAPKKK91**	**TaRaf51**	TraesCS4D02G089300.1	TaRaf51	Traes_4DS_7D8A5F90B		
**TaMAPKKK92**	**TaRaf52**	TraesCS5B02G337300.1	TaRaf52	Traes_5DL_3191490FE		
**TaMAPKKK93**	**TaRaf53**	TraesCS5D02G018800.1	TaRaf53	Traes_5BS_0B466F42F		
**TaMAPKKK94**	**TaRaf54**	TraesCS5B02G012000.1	TaRaf54	Traes_5BS_43731B6AC		
**TaMAPKKK95**	**TaRaf55**	TraesCS5B02G204900.1	TaRaf55	Traes_5BL_E44E042FD		
**TaMAPKKK96**	**TaRaf56**	TraesCS5B02G292000.2	TaRaf56	Traes_5BL_2DA8896EE		

**TaMAPKKK97**	**TaRaf57**	TraesCS5B02G353800.1	TaRaf57	Traes_5BL_11A7A1F5C		
**TaMAPKKK98**	**TaRaf58**	TraesCS5D02G482000.1	TaRaf58	Traes_5DL_294C4EDB3		
**TaMAPKKK99**	**TaRaf59**	TraesCS3A02G001500.1	TaRaf59	Traes_3AS_2A0765E10		
**TaMAPKKK100**	**TaRaf60**	TraesCS3A02G274000.1	TaRaf60	Traes_3AL_82306B917		
**TaMAPKKK101**	**TaRaf61**	TraesCS5D02G019400.1	TaRaf61	Traes_5DS_53F8C78FA		
**TaMAPKKK102**	**TaRaf62**	TraesCS7D02G474700.1	TaRaf62	Traes_7BL_46880A4FE		
**TaMAPKKK103**	**TaRaf63**	TraesCS7A02G326700.1	TaRaf63	Traes_7AL_9AD23808D		
**TaMAPKKK104**	**TaRaf64**	TraesCS1D02G273600.1	TaRaf64	Traes_1DL_0162A6BAC		
**TaMAPKKK104-1**	**TaRaf64-1**	TraesCS1B02G283400.1				
**TaMAPKKK105**	**TaRaf65**	TraesCS3A02G045200.1	TaRaf65	Traes_3AS_A0EA6D12C		
**TaMAPKKK106**	**TaRaf66**	TraesCS4A02G456900.1	TaRaf66	Traes_4AL_48E7FB1C6		
**TaMAPKKK107**	**TaRaf67**	TraesCS7A02G032700.1	TaRaf67	Traes_4AL_83D9333FE		
**TaMAPKKK108**	**TaRaf68**	TraesCS3D02G023600.1	TaRaf68	Traes_5DL_62B6846F6		
**TaMAPKKK109**	**TaRaf69**	TraesCS2D02G219800.1	TaRaf69	Traes_2DS_42A9CC22D		
**TaMAPKKK110**	**TaRaf70**	TraesCS4B02G289100.1	TaRaf70	Traes_4BL_3626CDB73		
**TaMAPKKK111**	**TaRaf71**	TraesCS3A02G315100.1	TaRaf71	Traes_3AL_5DC02A5FC		
**TaMAPKKK112**	**TaRaf72**	TraesCS5D02G359500.1	TaRaf72	Traes_5DL_0A74AE348		

**TaMAPKKK113**	**TaRaf73**	TraesCS3D02G108500.1	TaRaf73	Traes_3AS_C492FCE9A		
**TaMAPKKK115**	**TaRaf75**	TraesCS3A02G229800.1	TaRaf75	Traes_3AL_0187ECBAC		
**TaMAPKKK116**	**TaRaf76**	TraesCS1B02G454000.2	TaRaf76	Traes_1BL_1E2841006		
**TaMAPKKK117**	**TaRaf77**	TraesCS3D02G097000.1	TaRaf77	Traes_3DS_0B1914F50		
**TaMAPKKK119**	**TaRaf79**	TraesCS2A02G577000.1	TaRaf79	Traes_2AL_0E43EBBB6		
**TaMAPKKK120**	**TaRaf80**	TraesCS4A02G317600.1	TaRaf80	Traes_4AL_9601B9873		
**TaMAPKKK121**	**TaRaf81**	TraesCS2D02G066900.1	TaRaf81	Traes_2DS_964FA3D25		
**TaMAPKKK122**	**TaRaf82**	TraesCS2A02G214000.1	TaRaf82	Traes_2AS_DCD2F10331		
**TaMAPKKK123**	**TaRaf83**	TraesCS5D02G358700.1	TaRaf83	Traes_5DL_A367964F5		
**TaMAPKKK123-1**	**TaRaf83-1**	TraesCS5D02G358200.1				
**TaMAPKKK125**	**TaRaf85**	TraesCS7D02G153800.1	TaRaf85	Traes_7DS_81C827CE6		
**TaMAPKKK126**	**TaRaf86**	TraesCSU02G011500.1	TaRaf86	Traes_6BS_511AB47D71		
**TaMAPKKK127**	**TaRaf87**	TraesCS6D02G339600.1	TaRaf87	Traes_6DL_7662129AC		
**TaMAPKKK128**	**TaRaf88**	TraesCS1B02G446500.1	TaRaf88	Traes_1BL_CDC566E72		
**TaMAPKKK129**	**TaRaf89**	TraesCS6B02G320800.1	TaRaf89	Traes_6BL_658AE8589		
**TaMAPKKK130**	**TaRaf90**	TraesCS7A02G044600.1	TaRaf90	Traes_7AS_0BE0D89AC		
**TaMAPKKK131**	**TaRaf91**	TraesCS6B02G215100.2	TaRaf91	Traes_6BS_EAABDE59A		

**TaMAPKKK132**	**TaRaf92**	TraesCS5B02G353600.1	TaRaf92	Traes_5BL_17A56822E		
**TaMAPKKK133**	**TaRaf93**	TraesCS1D02G004300.1	TaRaf93	Traes_1BS_EA26D2661		
**TaMAPKKK134**	**TaRaf94**	TraesCS5D02G547500.1	TaRaf94	Traes_5DL_383D5A71F		
**TaMAPKKK135**	**TaRaf95**	TraesCS2D02G598800.1	TaRaf95	Traes_2DL_77990F25A		
**TaMAPKKK136**	**TaRaf96**	TraesCS2B02G241600.1	TaRaf96	Traes_2BS_C0AED9734		
**TaMAPKKK137**	**TaRaf97**	TraesCS3D02G472000.1	TaRaf97	Traes_3DL_73ACAB95C		
**TaMAPKKK139**	**TaRaf99**	TraesCS3A02G493500.1	TaRaf99	Traes_3AL_AB54706CA		
**TaMAPKKK140**	**TaRaf100**	TraesCS5D02G097900.1	TaRaf100	Traes_5BS_F1687AA56		
**TaMAPKKK141**	**TaRaf101**	TraesCS7D02G000800.1	TaRaf101	Traes_7DS_A46AFAE10		
**TaMAPKKK142**	**TaRaf102**	TraesCS2A02G032200.1	TaRaf102	Traes_2AS_CC27D1C41		
**TaMAPKKK144**	**TaRaf104**	TraesCS3D02G501100.1	TaRaf104	Traes_3DL_3D1CAD68F		
**TaMAPKKK145**	**TaRaf105**	TraesCS2B02G242300.1	TaRaf105	Traes_2BS_5C64FC44A		
**TaMAPKKK146**	**TaRaf106**	TraesCS2B02G241400.1	TaRaf106	Traes_4BS_C5AB35B0C		
**TaMAPKKK147**	**TaRaf107**	TraesCS2A02G216600.1	TaRaf107	Traes_2AS_E5AB3458C		
**TaMAPKKK148**	**TaRaf108**	TraesCS1A02G003900.1	TaRaf108	Traes_1BS_41E5F1990		
**TaMAPKKK150**	**TaRaf110**	TraesCS3B02G008600.2	TaRaf110	TRAES3BF061500080CFD_t1		
**TaMAPKKK151**	**TaRaf111**	TraesCS3B02G123800.1	TaRaf111	TRAES3BF104900080CFD_t1		

**TaMAPKKK152**	**TaRaf112**	TraesCS3B02G259100.1	TaRaf112	TRAES3BF026200090CFD_t1		
**TaMAPKKK153**	**TaRaf113**	TraesCS3B02G351800.1	TaRaf113	TRAES3BF086600060CFD_t1		
**TaMAPKKK154**	**TaRaf114**	TraesCS3B02G478400.1	TaRaf114	TRAES3BF078400040CFD_t1		
**TaMAPKKK155**	**TaRaf115**	TraesCS6B02G217100.1	TaRaf115	Traes_6BS_5BFDC774A		

Table S2 The Ka/Ks ratio of MAPK-MAP4K gene pairs in wheat											
***TaMAPK-TaMAP4K* Gene ID**	**Gene Name**	***TaMAPK-TaMAP4K* Gene ID**	**Gene Name**	**Ka**	**Ks**	**Ka/Ks**	**EffectiveLen**	**AverageS-sites**	**AverageN-sites**	**Selection pressure**	**Mya**
TraesCS1A02G003900	TaMAPKKK148	TraesCS1B02G004000	/	0.21	0.65	0.32	996.00	219.17	776.83	Purifying selection	49.63
TraesCS1A02G003900	TaMAPKKK148	TraesCS1B02G003200	/	0.21	0.46	0.44	978.00	217.83	760.17	Purifying selection	35.67
TraesCS1A02G025900	/	TraesCS1D02G026200	TaMAPKKK38	0.01	0.06	0.09	1845.00	421.33	1423.67	Purifying selection	4.75
TraesCS1A02G086500	TaMAPK11	TraesCS1B02G104900	TaMAPK27	0.01	0.10	0.06	1647.00	387.50	1259.50	Purifying selection	7.63
TraesCS1A02G086500	TaMAPK11	TraesCS1D02G088000	TaMAPK4	0.01	0.10	0.06	1647.00	386.67	1260.33	Purifying selection	7.65
TraesCS1A02G181900	TaMAPKKKK1	TraesCS1B02G199100	TaMAPKKKK2	0.00	0.05	0.08	2142.00	492.83	1649.17	Purifying selection	3.87
TraesCS1A02G181900	TaMAPKKKK1	TraesCS1D02G185000	TaMAPKKKK3	0.00	0.04	0.05	2142.00	493.08	1648.92	Purifying selection	2.71
TraesCS1A02G184500	TaMAPK14	TraesCS1B02G192600	TaMAPK28	0.00	0.07	0.03	1128.00	267.83	860.17	Purifying selection	5.42
TraesCS1A02G184500	TaMAPK14	TraesCS1D02G192200	/	0.00	0.03	0.03	1128.00	267.83	860.17	Purifying selection	2.64
TraesCS1A02G273600	/	TraesCS1D02G273600	TaMAPKKK104	0.08	0.15	0.53	2019.00	514.67	1504.33	Purifying selection	11.64
TraesCS1A02G273600	/	TraesCS1B02G283400	TaMAPKKK104-1	0.08	0.17	0.47	2025.00	516.33	1508.67	Purifying selection	12.70
TraesCS1A02G339600	/	TraesCS3A02G274000	TaMAPKKK100	0.33	1.29	0.26	1734.00	411.00	1323.00	Purifying selection	99.09
TraesCS1A02G339600	/	TraesCS3D02G273200	TaMAPKKK45	0.33	1.32	0.25	1740.00	412.83	1327.17	Purifying selection	101.42
TraesCS1A02G356300	/	TraesCS1B02G372400	TaMAPKKK53	0.03	0.14	0.23	2400.00	592.92	1807.08	Purifying selection	10.92
TraesCS1A02G366900	/	TraesCS3B02G288100	TaMAPKKK8	0.31	0.73	0.43	1338.00	337.83	1000.17	Purifying selection	56.52
TraesCS1A02G402400	TaMAPK41	TraesCS1B02G431400	TaMAPK29	0.02	0.15	0.11	1644.00	385.17	1258.83	Purifying selection	11.45

TraesCS1A02G402400	TaMAPK41	TraesCS1D02G410100	TaMAPK33	0.01	0.14	0.10	1644.00	385.33	1258.67	Purifying selection	10.48
TraesCS1A02G402400	TaMAPK41	TraesCS3A02G242100	TaMAPK39	0.12	1.02	0.12	1608.00	369.00	1239.00	Purifying selection	78.42
TraesCS1A02G402400	TaMAPK41	TraesCS3B02G270200	TaMAPK16	0.13	1.05	0.12	1620.00	371.17	1248.83	Purifying selection	80.72
TraesCS1A02G402400	TaMAPK41	TraesCS3D02G242200	TaMAPK24	0.13	1.03	0.12	1614.00	370.25	1243.75	Purifying selection	78.94
TraesCS1A02G415300	TaMAPK42	TraesCS1B02G445300	/	0.01	0.04	0.38	1812.00	424.33	1387.67	Purifying selection	2.79
TraesCS1A02G415300	TaMAPK42	TraesCS1D02G422800	TaMAPK5	0.01	0.03	0.38	1815.00	423.67	1391.33	Purifying selection	2.03
TraesCS1A02G415300	TaMAPK42	TraesCS3A02G231700	TaMAPK40	0.13	1.22	0.11	1440.00	327.83	1112.17	Purifying selection	93.60
TraesCS1A02G415300	TaMAPK42	TraesCS3B02G260900	/	0.12	1.28	0.09	1440.00	329.92	1110.08	Purifying selection	98.85
TraesCS1A02G415300	TaMAPK42	TraesCS3D02G221700	TaMAPK23	0.12	1.32	0.09	1425.00	326.25	1098.75	Purifying selection	101.76
TraesCS1A02G416200	/	TraesCS1B02G446500	TaMAPKKK128	0.01	0.16	0.05	1113.00	265.00	848.00	Purifying selection	12.06
TraesCS1A02G416200	/	TraesCS3B02G259800	TaMAPKKK58	0.10	0.70	0.15	1113.00	265.75	847.25	Purifying selection	53.77
TraesCS1A02G416200	/	TraesCS1D02G423800	TaMAPKKK70	0.01	0.15	0.05	1119.00	266.00	853.00	Purifying selection	11.83
TraesCS1A02G421000	TaMAPK43	TraesCS1B02G452200	TaMAPK6	0.01	0.13	0.08	1794.00	434.42	1359.58	Purifying selection	10.03
TraesCS1A02G421000	TaMAPK43	TraesCS1D02G428900	TaMAPK34	0.01	0.10	0.08	1791.00	431.25	1359.75	Purifying selection	7.62
TraesCS1A02G421000	TaMAPK43	TraesCS3A02G228000	/	0.15	1.00	0.15	1767.00	419.92	1347.08	Purifying selection	76.63
TraesCS1A02G421000	TaMAPK43	TraesCS3B02G256700	TaMAPK38	0.15	1.01	0.15	1761.00	419.08	1341.92	Purifying selection	77.77
TraesCS1A02G421000	TaMAPK43	TraesCS3D02G225600	TaMAPK8	0.15	1.03	0.15	1767.00	420.25	1346.75	Purifying selection	78.97
TraesCS1B02G001500	/	TraesCS1D02G004300	TaMAPKKK133	0.01	0.31	0.02	1077.00	254.50	822.50	Purifying selection	23.56

TraesCS1B02G032500	/	TraesCS1D02G026200	TaMAPKKK38	0.02	0.09	0.19	1845.00	423.58	1421.42	Purifying selection	7.25
TraesCS1B02G104900	TaMAPK27	TraesCS1D02G088000	TaMAPK4	0.00	0.11	0.04	1647.00	387.83	1259.17	Purifying selection	8.31
TraesCS1B02G192600	TaMAPK28	TraesCS1D02G192200	/	0.00	0.07	0.02	1128.00	268.17	859.83	Purifying selection	5.41
TraesCS1B02G199100	TaMAPKKK2	TraesCS1D02G185000	TaMAPKKK3	0.00	0.04	0.09	2142.00	492.42	1649.58	Purifying selection	3.05
TraesCS1B02G283400	TaMAPKKK104-1	TraesCS1D02G273600	TaMAPKKK104	0.07	0.16	0.42	2028.00	513.83	1514.17	Purifying selection	12.21
TraesCS1B02G351900	/	TraesCS3A02G274000	TaMAPKKK100	0.33	1.29	0.26	1734.00	408.58	1325.42	Purifying selection	99.25
TraesCS1B02G351900	/	TraesCS3D02G273200	TaMAPKKK45	0.33	1.31	0.25	1740.00	410.75	1329.25	Purifying selection	100.57
TraesCS1B02G372400	TaMAPKKK53	TraesCS1D02G360300	/	0.13	0.22	0.61	1800.00	453.25	1346.75	Purifying selection	16.64
TraesCS1B02G372400	TaMAPKKK53	TraesCS3A02G246100	TaMAPKKK76	0.12	0.34	0.35	2391.00	591.00	1800.00	Purifying selection	26.40
TraesCS1B02G372400	TaMAPKKK53	TraesCS3B02G273200	/	0.11	0.35	0.32	2406.00	596.25	1809.75	Purifying selection	26.92
TraesCS1B02G372400	TaMAPKKK53	TraesCS3D02G245300	/	0.12	0.34	0.34	2406.00	594.75	1811.25	Purifying selection	26.15
TraesCS1B02G384700	/	TraesCS3B02G288100	TaMAPKKK8	0.34	0.66	0.51	1314.00	330.58	983.42	Purifying selection	50.53
TraesCS1B02G431400	TaMAPK29	TraesCS1D02G410100	TaMAPK33	0.01	0.11	0.09	1665.00	391.33	1273.67	Purifying selection	8.45
TraesCS1B02G431400	TaMAPK29	TraesCS3A02G242100	TaMAPK39	0.12	1.19	0.10	1632.00	376.00	1256.00	Purifying selection	91.16
TraesCS1B02G431400	TaMAPK29	TraesCS3B02G270200	TaMAPK16	0.13	1.22	0.11	1644.00	378.17	1265.83	Purifying selection	93.90
TraesCS1B02G431400	TaMAPK29	TraesCS3D02G242200	TaMAPK24	0.13	1.22	0.11	1638.00	377.25	1260.75	Purifying selection	93.78
TraesCS1B02G445300	/	TraesCS3D02G221700	TaMAPK23	0.12	1.29	0.09	1425.00	326.25	1098.75	Purifying selection	98.85
TraesCS1B02G445300	/	TraesCS1D02G422800	TaMAPK5	0.01	0.04	0.28	1812.00	424.00	1388.00	Purifying selection	3.36

TraesCS1B02G445300	/	TraesCS3A02G231700	TaMAPK40	0.13	1.28	0.10	1440.00	327.50	1112.50	Purifying selection	98.29
TraesCS1B02G446500	TaMAPKKK128	TraesCS1D02G423800	TaMAPKKK70	0.01	0.14	0.05	1113.00	264.67	848.33	Purifying selection	10.84
TraesCS1B02G446500	TaMAPKKK128	TraesCS3A02G230500	/	0.10	0.68	0.15	1107.00	263.50	843.50	Purifying selection	52.57
TraesCS1B02G446500	TaMAPKKK128	TraesCS3B02G259800	TaMAPKKK58	0.10	0.70	0.14	1107.00	263.25	843.75	Purifying selection	53.75
TraesCS1B02G446500	TaMAPKKK128	TraesCS3D02G222800	/	0.10	0.69	0.15	1107.00	263.58	843.42	Purifying selection	52.90
TraesCS1B02G452200	TaMAPK6	TraesCS1D02G428900	TaMAPK34	0.01	0.10	0.06	1797.00	434.50	1362.50	Purifying selection	7.96
TraesCS1B02G452200	TaMAPK6	TraesCS3A02G228000	/	0.15	1.08	0.14	1770.00	423.08	1346.92	Purifying selection	82.85
TraesCS1B02G452200	TaMAPK6	TraesCS3B02G256700	TaMAPK38	0.15	1.11	0.13	1764.00	422.25	1341.75	Purifying selection	85.71
TraesCS1B02G452200	TaMAPK6	TraesCS3D02G225600	TaMAPK8	0.15	1.11	0.13	1770.00	423.42	1346.58	Purifying selection	85.43
TraesCS1B02G454000	TaMAPKKK116	TraesCS1D02G430800	/	0.21	0.60	0.34	1932.00	473.17	1458.83	Purifying selection	45.88
TraesCS1D02G341600	/	TraesCS3A02G274000	TaMAPKKK100	0.33	1.31	0.25	1725.00	407.25	1317.75	Purifying selection	100.66
TraesCS1D02G341600	/	TraesCS3D02G273200	TaMAPKKK45	0.33	1.29	0.26	1731.00	409.08	1321.92	Purifying selection	98.93
TraesCS1D02G360400	/	TraesCS3A02G246100	TaMAPKKK76	0.10	0.33	0.31	2373.00	590.67	1782.33	Purifying selection	25.33
TraesCS1D02G372900	/	TraesCS3B02G288100	TaMAPKKK8	0.37	0.79	0.47	1308.00	329.58	978.42	Purifying selection	60.51
TraesCS1D02G410100	TaMAPK33	TraesCS3A02G242100	TaMAPK39	0.12	1.22	0.10	1632.00	375.83	1256.17	Purifying selection	93.80
TraesCS1D02G410100	TaMAPK33	TraesCS3B02G270200	TaMAPK16	0.13	1.27	0.10	1644.00	378.00	1266.00	Purifying selection	97.75
TraesCS1D02G410100	TaMAPK33	TraesCS3D02G242200	TaMAPK24	0.13	1.23	0.10	1638.00	377.08	1260.92	Purifying selection	94.40
TraesCS1D02G422800	TaMAPK5	TraesCS3A02G231700	TaMAPK40	0.13	1.25	0.11	1440.00	327.50	1112.50	Purifying selection	96.28

TraesCS1D02G422800	TaMAPK5	TraesCS3B02G260900	/	0.12	1.32	0.09	1440.00	329.58	1110.42	Purifying selection	101.77
TraesCS1D02G422800	TaMAPK5	TraesCS3D02G221700	TaMAPK23	0.12	1.36	0.09	1425.00	325.92	1099.08	Purifying selection	104.88
TraesCS1D02G423800	TaMAPKKK70	TraesCS3A02G230500	/	0.10	0.69	0.15	1107.00	264.00	843.00	Purifying selection	53.25
TraesCS1D02G423800	TaMAPKKK70	TraesCS3B02G259800	TaMAPKKK58	0.10	0.71	0.14	1113.00	264.58	848.42	Purifying selection	54.54
TraesCS1D02G423800	TaMAPKKK70	TraesCS3D02G222800	/	0.10	0.72	0.14	1113.00	264.92	848.08	Purifying selection	55.17
TraesCS1D02G428900	TaMAPK34	TraesCS3A02G228000	/	0.16	1.01	0.15	1770.00	420.33	1349.67	Purifying selection	77.75
TraesCS1D02G428900	TaMAPK34	TraesCS3B02G256700	TaMAPK38	0.15	1.02	0.15	1764.00	419.67	1344.33	Purifying selection	78.13
TraesCS1D02G428900	TaMAPK34	TraesCS3D02G225600	TaMAPK8	0.16	1.04	0.15	1770.00	420.67	1349.33	Purifying selection	80.13
TraesCS2A02G051000	/	TraesCS2D02G050700	TaMAPKKK46	0.15	0.48	0.32	315.00	75.92	239.08	Purifying selection	36.77
TraesCS2A02G095300	TaMAPKKK28	TraesCS2B02G110500	TaMAPKKK27	0.01	0.07	0.11	3996.00	966.50	3029.50	Purifying selection	5.60
TraesCS2A02G095300	TaMAPKKK28	TraesCS2D02G093700	TaMAPKKK26	0.01	0.07	0.10	3996.00	965.42	3030.58	Purifying selection	5.69
TraesCS2A02G190200	/	TraesCS2D02G197600	TaMAPKKK34	0.00	0.04	0.09	957.00	232.67	724.33	Purifying selection	3.40
TraesCS2A02G190200	/	TraesCS2B02G216800	TaMAPKKK37	0.01	0.08	0.14	966.00	234.42	731.58	Purifying selection	6.23
TraesCS2A02G195900	TaMAPKKK32	TraesCS2B02G223600	TaMAPKKK36	0.01	0.08	0.09	2103.00	484.75	1618.25	Purifying selection	6.02
TraesCS2A02G195900	TaMAPKKK32	TraesCS2D02G203900	/	0.01	0.08	0.07	2103.00	485.83	1617.17	Purifying selection	6.53
TraesCS2A02G199700	TaMAPKKK5	TraesCS2B02G227000	/	0.00	0.06	0.06	1629.00	374.83	1254.17	Purifying selection	4.26
TraesCS2A02G199700	TaMAPKKK5	TraesCS2D02G207400	/	0.00	0.06	0.07	1629.00	374.67	1254.33	Purifying selection	4.48
TraesCS2A02G214000	TaMAPKKK122	TraesCS2D02G219800	TaMAPKKK109	0.02	0.10	0.22	2043.00	510.92	1532.08	Purifying selection	7.72

TraesCS2A02G216300	/	TraesCS2B02G241400	TaMAPKKK146	0.04	0.10	0.36	2001.00	489.92	1511.08	Purifying selection	7.81
TraesCS2A02G217000	TaMAPKKK60	TraesCS2B02G242400	/	0.07	0.15	0.49	1128.00	275.17	852.83	Purifying selection	11.68
TraesCS2A02G217600	TaMAPKKK80	TraesCS2B02G243100	/	0.01	0.09	0.09	1017.00	231.25	785.75	Purifying selection	6.69
TraesCS2A02G233400	TaMAPKKKK4	TraesCS2B02G249900	TaMAPKKKK5	0.00	0.05	0.08	2076.00	496.17	1579.83	Purifying selection	3.52
TraesCS2A02G233400	TaMAPKKKK4	TraesCS2D02G232200	TaMAPKKKK6	0.00	0.03	0.15	2076.00	495.92	1580.08	Purifying selection	2.13
TraesCS2A02G407600	TaMAPKKK1	TraesCS2B02G425500	/	0.02	0.08	0.31	2460.00	586.50	1873.50	Purifying selection	6.08
TraesCS2A02G407600	TaMAPKKK1	TraesCS2D02G404700	/	0.02	0.06	0.41	2481.00	591.58	1889.42	Purifying selection	4.48
TraesCS2A02G407600	TaMAPKKK1	TraesCS6A02G245000	TaMAPKKK4	0.15	0.91	0.17	2427.00	580.08	1846.92	Purifying selection	69.77
TraesCS2A02G407600	TaMAPKKK1	TraesCS6B02G279300	TaMAPKKK4-1	0.13	0.85	0.16	2343.00	561.42	1781.58	Purifying selection	65.46
TraesCS2A02G407600	TaMAPKKK1	TraesCS6D02G227300	/	0.15	0.88	0.17	2427.00	581.92	1845.08	Purifying selection	67.69
TraesCS2A02G498000	TaMAPKKK20	TraesCS2B02G526200	TaMAPKKK18	0.01	0.08	0.10	3969.00	917.92	3051.08	Purifying selection	6.28
TraesCS2A02G498000	TaMAPKKK20	TraesCS2D02G498100	/	0.01	0.07	0.10	3861.00	895.75	2965.25	Purifying selection	5.49
TraesCS2A02G578900	/	TraesCS2D02G598800	TaMAPKKK135	0.36	1.10	0.33	1290.00	301.00	989.00	Purifying selection	84.89
TraesCS2B02G082200	/	TraesCS2D02G066900	TaMAPKKK121	0.20	0.64	0.30	795.00	189.42	605.58	Purifying selection	49.51
TraesCS2B02G110500	TaMAPKKK27	TraesCS2D02G093700	TaMAPKKK26	0.00	0.04	0.11	4005.00	967.92	3037.08	Purifying selection	3.44
TraesCS2B02G216800	TaMAPKKK37	TraesCS2D02G197600	TaMAPKKK34	0.01	0.04	0.19	957.00	232.92	724.08	Purifying selection	3.40
TraesCS2B02G223600	TaMAPKKK36	TraesCS2D02G203900	/	0.00	0.07	0.06	2109.00	486.75	1622.25	Purifying selection	5.64
TraesCS2B02G241400	TaMAPKKK146	TraesCS2D02G221900	/	0.03	0.10	0.36	2001.00	491.33	1509.67	Purifying selection	7.34

TraesCS2B02G249900	TaMAPKKKK5	TraesCS2D02G232200	TaMAPKKKK6	0.01	0.05	0.09	2076.00	495.42	1580.58	Purifying selection	4.19
TraesCS2B02G425500	/	TraesCS6B02G279300	TaMAPKKK4-1	0.14	0.85	0.16	2355.00	566.33	1788.67	Purifying selection	65.10
TraesCS2B02G425500	/	TraesCS6A02G245000	TaMAPKKK4	0.16	0.86	0.18	2616.00	628.58	1987.42	Purifying selection	66.22
TraesCS2B02G526200	TaMAPKKK18	TraesCS2D02G498100	/	0.01	0.05	0.10	3864.00	897.67	2966.33	Purifying selection	3.90
TraesCS2B02G606100	/	TraesCS2D02G598800	TaMAPKKK135	0.25	1.21	0.21	1035.00	244.08	790.92	Purifying selection	92.80
TraesCS2B02G610200	/	TraesCS2D02G588200	TaMAPKKK69	0.02	0.11	0.19	1638.00	389.92	1248.08	Purifying selection	8.30
TraesCS2D02G404700	/	TraesCS6A02G245000	TaMAPKKK4	0.15	0.83	0.18	2616.00	627.92	1988.08	Purifying selection	64.01
TraesCS2D02G404700	/	TraesCS6B02G279300	TaMAPKKK4-1	0.13	0.82	0.16	2355.00	566.00	1789.00	Purifying selection	62.71
TraesCS3A02G001500	TaMAPKKK99	TraesCS3B02G000400	/	0.02	0.16	0.10	1095.00	268.17	826.83	Purifying selection	12.26
TraesCS3A02G001500	TaMAPKKK99	TraesCS3D02G010000	/	0.01	0.17	0.07	1092.00	268.67	823.33	Purifying selection	12.77
TraesCS3A02G003900	TaMAPKKK72	TraesCS3B02G008600	TaMAPKKK150	0.03	0.16	0.20	2043.00	532.83	1510.17	Purifying selection	12.53
TraesCS3A02G003900	TaMAPKKK72	TraesCS3D02G005400	/	0.02	0.14	0.18	1203.00	285.67	917.33	Purifying selection	10.61
TraesCS3A02G039100	TaMAPKKK56	TraesCS3B02G043700	/	0.11	0.37	0.30	1440.00	339.33	1100.67	Purifying selection	28.76
TraesCS3A02G039100	TaMAPKKK56	TraesCS3D02G040300	/	0.08	0.37	0.23	1284.00	297.75	986.25	Purifying selection	28.55
TraesCS3A02G039200	TaMAPKKK73	TraesCS3B02G043600	/	0.12	0.34	0.36	2031.00	490.42	1540.58	Purifying selection	26.43
TraesCS3A02G039200	TaMAPKKK73	TraesCS3D02G040400	/	0.02	0.14	0.14	540.00	119.67	420.33	Purifying selection	10.55
TraesCS3A02G045200	TaMAPKKK105	TraesCS3B02G038900	/	0.01	0.13	0.12	1185.00	298.75	886.25	Purifying selection	9.80
TraesCS3A02G045200	TaMAPKKK105	TraesCS3D02G036000	/	0.01	0.10	0.07	1197.00	302.25	894.75	Purifying selection	7.32

TraesCS3A02G094800	/	TraesCS3B02G110300	TaMAPKKK43	0.04	0.11	0.36	435.00	92.58	342.42	Purifying selection	8.57
TraesCS3A02G096500	TaMAPKKK75	TraesCS3B02G112300	/	0.01	0.11	0.11	1941.00	488.92	1452.08	Purifying selection	8.09
TraesCS3A02G096500	TaMAPKKK75	TraesCS3D02G097000	TaMAPKKK117	0.01	0.06	0.22	1944.00	488.92	1455.08	Purifying selection	4.75
TraesCS3A02G105400	/	TraesCS3B02G123800	TaMAPKKK151	0.02	0.09	0.26	1731.00	425.25	1305.75	Purifying selection	6.57
TraesCS3A02G228000	/	TraesCS3D02G225600	TaMAPK8	0.00	0.05	0.02	1839.00	433.67	1405.33	Purifying selection	3.47
TraesCS3A02G228000	/	TraesCS3B02G256700	TaMAPK38	0.00	0.05	0.03	1806.00	425.67	1380.33	Purifying selection	3.54
TraesCS3A02G229800	TaMAPKKK115	TraesCS3B02G259100	TaMAPKKK152	0.04	0.10	0.41	2094.00	567.58	1526.42	Purifying selection	7.59
TraesCS3A02G230500	/	TraesCS3B02G259800	TaMAPKKK58	0.00	0.08	0.04	1155.00	276.58	878.42	Purifying selection	6.47
TraesCS3A02G231700	TaMAPK40	TraesCS3B02G260900	/	0.02	0.08	0.25	1470.00	337.75	1132.25	Purifying selection	5.99
TraesCS3A02G231700	TaMAPK40	TraesCS3D02G221700	TaMAPK23	0.02	0.08	0.29	1464.00	336.58	1127.42	Purifying selection	6.02
TraesCS3A02G242100	TaMAPK39	TraesCS3B02G270200	TaMAPK16	0.00	0.03	0.09	1746.00	395.00	1351.00	Purifying selection	2.59
TraesCS3A02G242100	TaMAPK39	TraesCS3B02G256700	TaMAPK38	0.19	2.64	0.07	1692.00	389.83	1302.17	Purifying selection	202.72
TraesCS3A02G242100	TaMAPK39	TraesCS3D02G242200	TaMAPK24	0.00	0.02	0.11	1746.00	395.75	1350.25	Purifying selection	1.58
TraesCS3A02G242100	TaMAPK39	TraesCS3D02G225600	TaMAPK8	0.19	2.61	0.07	1722.00	397.08	1324.92	Purifying selection	200.95
TraesCS3A02G246100	TaMAPKKK76	TraesCS3B02G273200	/	0.03	0.12	0.22	2439.00	603.00	1836.00	Purifying selection	9.25
TraesCS3A02G246100	TaMAPKKK76	TraesCS3D02G245300	/	0.03	0.12	0.23	2439.00	601.50	1837.50	Purifying selection	9.05
TraesCS3A02G255600	/	TraesCS3B02G288100	TaMAPKKK8	0.03	0.13	0.27	1383.00	343.58	1039.42	Purifying selection	10.00
TraesCS3A02G274000	TaMAPKKK100	TraesCS3B02G307700	/	0.02	0.07	0.33	1800.00	417.00	1383.00	Purifying selection	5.01

TraesCS3A02G274000	TaMAPKKK100	TraesCS3D02G273200	TaMAPKKK45	0.02	0.09	0.20	1800.00	417.92	1382.08	Purifying selection	6.83
TraesCS3A02G315100	TaMAPKKK111	TraesCS3B02G351800	TaMAPKKK153	0.02	0.11	0.14	906.00	213.08	692.92	Purifying selection	8.54
TraesCS3A02G315100	TaMAPKKK111	TraesCS3D02G316400	/	0.04	0.09	0.47	2418.00	553.00	1865.00	Purifying selection	6.55
TraesCS3A02G477300	/	TraesCS3D02G472000	TaMAPKKK137	0.03	0.12	0.23	2235.00	537.83	1697.17	Purifying selection	8.96
TraesCS3A02G493500	TaMAPKKK139	TraesCS3B02G555500	/	0.03	0.09	0.34	2121.00	492.83	1628.17	Purifying selection	6.87
TraesCS3A02G493800	/	TraesCS3D02G501100	TaMAPKKK144	0.10	0.23	0.42	2106.00	489.75	1616.25	Purifying selection	17.69
TraesCS3B02G008600	TaMAPKKK150	TraesCS3D02G005400	/	0.03	0.20	0.13	1197.00	284.92	912.08	Purifying selection	15.67
TraesCS3B02G022400	/	TraesCS3D02G023600	TaMAPKKK108	0.03	0.10	0.26	2484.00	601.83	1882.17	Purifying selection	8.06
TraesCS3B02G110300	TaMAPKKK43	TraesCS3D02G095000	/	0.04	0.11	0.36	435.00	92.75	342.25	Purifying selection	8.55
TraesCS3B02G112300	/	TraesCS3D02G097000	TaMAPKKK117	0.01	0.10	0.11	1941.00	489.33	1451.67	Purifying selection	7.73
TraesCS3B02G123800	TaMAPKKK151	TraesCS3D02G107500	/	0.02	0.05	0.32	1731.00	426.17	1304.83	Purifying selection	4.02
TraesCS3B02G256700	TaMAPK38	TraesCS3B02G270200	TaMAPK16	0.19	2.55	0.07	1692.00	389.17	1302.83	Purifying selection	196.35
TraesCS3B02G256700	TaMAPK38	TraesCS3D02G225600	TaMAPK8	0.00	0.03	0.05	1806.00	426.00	1380.00	Purifying selection	2.21
TraesCS3B02G256700	TaMAPK38	TraesCS3D02G242200	TaMAPK24	0.19	2.97	0.06	1692.00	389.92	1302.08	Purifying selection	228.16
TraesCS3B02G259800	TaMAPKKK58	TraesCS3D02G222800	/	0.00	0.04	0.08	1155.00	276.67	878.33	Purifying selection	3.44
TraesCS3B02G260900	/	TraesCS3D02G221700	TaMAPK23	0.01	0.06	0.14	1464.00	338.67	1125.33	Purifying selection	4.49
TraesCS3B02G270200	TaMAPK16	TraesCS3D02G242200	TaMAPK24	0.00	0.05	0.03	1752.00	395.92	1356.08	Purifying selection	3.61
TraesCS3B02G270200	TaMAPK16	TraesCS3D02G225600	TaMAPK8	0.20	2.46	0.08	1722.00	396.42	1325.58	Purifying selection	189.45

TraesCS3B02G288100	TaMAPKKK8	TraesCS3D02G256400	/	0.30	0.67	0.45	1329.00	331.25	997.75	Purifying selection	51.63
TraesCS3B02G307700	/	TraesCS3D02G273200	TaMAPKKK45	0.01	0.07	0.19	1803.00	418.08	1384.92	Purifying selection	5.40
TraesCS3B02G351800	TaMAPKKK153	TraesCS3D02G316400	/	0.01	0.12	0.09	906.00	212.50	693.50	Purifying selection	9.42
TraesCS3B02G555800	/	TraesCS3D02G501100	TaMAPKKK144	0.09	0.22	0.41	2103.00	487.67	1615.33	Purifying selection	17.03
TraesCS3D02G225600	TaMAPK8	TraesCS3D02G242200	TaMAPK24	0.20	2.92	0.07	1722.00	397.17	1324.83	Purifying selection	224.88
TraesCS4A02G093800	TaMAPKKK12	TraesCS4B02G210600	TaMAPKKK3	0.01	0.07	0.11	2115.00	513.00	1602.00	Purifying selection	5.50
TraesCS4A02G093800	TaMAPKKK12	TraesCS4D02G211300	TaMAPKKK10	0.01	0.06	0.11	2130.00	516.33	1613.67	Purifying selection	4.65
TraesCS4A02G106400	TaMAPK30	TraesCS4B02G197800	/	0.00	0.08	0.00	1107.00	258.33	848.67	Purifying selection	6.29
TraesCS4A02G106400	TaMAPK30	TraesCS4D02G198600	TaMAPK25	0.00	0.06	0.02	1107.00	259.00	848.00	Purifying selection	4.64
TraesCS4A02G265900	TaMAPKK14	TraesCS4B02G048600	TaMAPKK7	0.23	0.62	0.38	975.00	265.42	709.58	Purifying selection	47.93
TraesCS4A02G265900	TaMAPKK14	TraesCS4D02G048800	TaMAPKK11	0.06	0.35	0.18	1002.00	271.92	730.08	Purifying selection	27.01
TraesCS4A02G266000	TaMAPKK15	TraesCS4B02G048100	TaMAPKK6	0.24	0.54	0.44	975.00	267.92	707.08	Purifying selection	41.27
TraesCS4A02G266000	TaMAPKK15	TraesCS4D02G047900	/	0.23	0.51	0.45	975.00	268.92	706.08	Purifying selection	38.90
TraesCS4A02G283400	/	TraesCS4D02G027600	TaMAPKKK2	0.01	0.14	0.07	1704.00	431.00	1273.00	Purifying selection	10.76
TraesCS4A02G313900	TaMAPKKK62	TraesCS5D02G547600	/	0.30	1.34	0.23	3252.00	779.92	2472.08	Purifying selection	102.76
TraesCS4A02G336800	TaMAPK2	TraesCS5B02G536500	TaMAPK50	0.02	0.08	0.24	1320.00	312.08	1007.92	Purifying selection	5.79
TraesCS4A02G336800	TaMAPK2	TraesCS5D02G534000	TaMAPK26	0.01	0.05	0.23	1320.00	311.17	1008.83	Purifying selection	4.19
TraesCS4A02G383000	TaMAPKKK63	TraesCS7D02G079100	TaMAPKKK48	0.13	0.35	0.36	1653.00	373.08	1279.92	Purifying selection	27.28

TraesCS4A02G434800	TaMAPK31	TraesCS7A02G049000	TaMAPK46	0.02	0.10	0.18	1449.00	344.75	1104.25	Purifying selection	7.62
TraesCS4A02G434800	TaMAPK31	TraesCS7D02G044100	TaMAPK18	0.01	0.08	0.16	1449.00	343.50	1105.50	Purifying selection	6.51
TraesCS4A02G456900	TaMAPKKK106	TraesCS7A02G032700	TaMAPKKK107	0.02	0.10	0.23	1440.00	332.92	1107.08	Purifying selection	7.65
TraesCS4A02G456900	TaMAPKKK106	TraesCS7D02G029300	/	0.02	0.05	0.31	1437.00	332.42	1104.58	Purifying selection	3.83
TraesCS4A02G464700	TaMAPKKK64-1	TraesCS7D02G020600	/	0.11	0.60	0.19	504.00	116.67	387.33	Purifying selection	45.88
TraesCS4A02G464700	TaMAPKKK64-1	TraesCS7D02G022000	/	0.13	0.60	0.21	504.00	117.33	386.67	Purifying selection	45.97
TraesCS4A02G465000	TaMAPKKK65	TraesCS7D02G022200	TaMAPKKK47	0.44	1.04	0.43	1239.00	283.33	955.67	Purifying selection	80.30
TraesCS4A02G465900	TaMAPKKK64	TraesCS7A02G023400	/	0.08	0.28	0.31	792.00	176.75	615.25	Purifying selection	21.24
TraesCS4B02G030100	/	TraesCS4D02G027600	TaMAPKKK2	0.01	0.15	0.09	1704.00	430.83	1273.17	Purifying selection	11.41
TraesCS4B02G048100	TaMAPKK6	TraesCS4D02G047900	/	0.04	0.13	0.31	1029.00	284.50	744.50	Purifying selection	9.69
TraesCS4B02G048900	TaMAPKK8	TraesCS4D02G048800	TaMAPKK11	0.02	0.15	0.17	1002.00	273.50	728.50	Purifying selection	11.30
TraesCS4B02G210600	TaMAPKKK3	TraesCS4D02G211300	TaMAPKKK10	0.01	0.05	0.16	2115.00	513.67	1601.33	Purifying selection	4.19
TraesCS4B02G289100	TaMAPKKK110	TraesCS4D02G288200	/	0.02	0.11	0.20	2004.00	526.50	1477.50	Purifying selection	8.12
TraesCS4B02G395600	TaMAPKKKK7	TraesCS5A02G556400	TaMAPKKKK11	0.11	0.18	0.58	1779.00	419.42	1359.58	Purifying selection	14.03
TraesCS4B02G398400	TaMAPKKKK8	TraesCS5A02G556400	TaMAPKKKK11	0.01	0.08	0.16	2187.00	511.25	1675.75	Purifying selection	5.94
TraesCS4B02G398400	TaMAPKKKK8	TraesCSU02G115300	TaMAPKKKK25	0.01	0.08	0.17	2223.00	519.08	1703.92	Purifying selection	6.25
TraesCS5A02G085900	/	TraesCS5D02G097900	TaMAPKKK140	0.00	0.03	0.13	2259.00	531.83	1727.17	Purifying selection	2.66
TraesCS5A02G118200	TaMAPKKK14	TraesCS5B02G112900	/	0.01	0.04	0.24	1638.00	383.33	1254.67	Purifying selection	3.09

TraesCS5A02G122700	TaMAPKK4	TraesCS5B02G122600	TaMAPKK18	0.02	0.06	0.36	897.00	213.17	683.83	Purifying selection	4.44
TraesCS5A02G122700	TaMAPKK4	TraesCS5D02G130900	TaMAPKK3	0.02	0.07	0.25	897.00	212.42	684.58	Purifying selection	5.64
TraesCS5A02G147500	/	TraesCS5D02G145100	TaMAPKKK30	0.01	0.05	0.22	1908.00	478.83	1429.17	Purifying selection	3.48
TraesCS5A02G147500	/	TraesCS5B02G146100	TaMAPKKK40	0.01	0.09	0.10	1908.00	477.75	1430.25	Purifying selection	6.65
TraesCS5A02G187400	TaMAPKKKK9	TraesCS5B02G196400	TaMAPKKK25	0.02	0.09	0.17	1554.00	383.08	1170.92	Purifying selection	7.27
TraesCS5A02G187400	TaMAPKKKK9	TraesCS5D02G203600	TaMAPKKKK13	0.02	0.09	0.17	1554.00	380.67	1173.33	Purifying selection	7.09
TraesCS5A02G200800	TaMAPKKK17	TraesCS5B02G199400	TaMAPKKK24	0.00	0.06	0.02	2040.00	474.92	1565.08	Purifying selection	4.37
TraesCS5A02G200800	TaMAPKKK17	TraesCS5D02G206500	TaMAPKKK29	0.00	0.03	0.04	2046.00	477.50	1568.50	Purifying selection	2.47
TraesCS5A02G206700	/	TraesCS5B02G204900	TaMAPKKK95	0.01	0.07	0.20	1317.00	349.25	967.75	Purifying selection	5.54
TraesCS5A02G292500	TaMAPKKK81	TraesCS5D02G299800	/	0.03	0.06	0.51	1047.00	228.58	818.42	Purifying selection	4.55
TraesCS5A02G338800	/	TraesCS5B02G337300	TaMAPKKK92	0.00	0.08	0.05	1785.00	416.67	1368.33	Purifying selection	6.44
TraesCS5A02G351100	/	TraesCS5D02G358200	TaMAPKKK123-1	0.10	0.30	0.34	1350.00	308.42	1041.58	Purifying selection	23.17
TraesCS5A02G392500	TaMAPKKK22	TraesCS5B02G397300	TaMAPKKKK12	0.01	0.06	0.10	2475.00	596.33	1878.67	Purifying selection	4.28
TraesCS5A02G392500	TaMAPKKK22	TraesCS5D02G402300	TaMAPKKKK14	0.01	0.05	0.11	2466.00	593.58	1872.42	Purifying selection	3.75
TraesCS5A02G463100	TaMAPKKK15	TraesCS5B02G474500	TaMAPKKK16	0.00	0.10	0.01	1602.00	371.42	1230.58	Purifying selection	7.75
TraesCS5A02G463100	TaMAPKKK15	TraesCS5D02G475900	TaMAPKKK11	0.00	0.07	0.06	1602.00	371.67	1230.33	Purifying selection	5.65
TraesCS5A02G469200	/	TraesCS5D02G482000	TaMAPKKK98	0.01	0.05	0.14	1242.00	296.83	945.17	Purifying selection	4.02
TraesCS5A02G556400	TaMAPKKKK11	TraesCSU02G115300	TaMAPKKKK25	0.01	0.05	0.14	2187.00	508.50	1678.50	Purifying selection	4.15

TraesCS5B02G012000	TaMAPKKK94	TraesCS5D02G019400	TaMAPKKK101	0.03	0.12	0.25	3039.00	812.67	2226.33	Purifying selection	9.00
TraesCS5B02G091700	/	TraesCS5D02G097900	TaMAPKKK140	0.01	0.07	0.08	2259.00	532.00	1727.00	Purifying selection	5.30
TraesCS5B02G122600	TaMAPKK18	TraesCS5D02G130900	TaMAPKK3	0.03	0.07	0.39	1386.00	322.33	1063.67	Purifying selection	5.03
TraesCS5B02G146100	TaMAPKKK40	TraesCS5D02G145100	TaMAPKKK30	0.01	0.08	0.13	1920.00	481.08	1438.92	Purifying selection	5.89
TraesCS5B02G196400	TaMAPKKK25	TraesCS5D02G203600	TaMAPKKKK13	0.01	0.07	0.20	1554.00	382.92	1171.08	Purifying selection	5.70
TraesCS5B02G199400	TaMAPKKK24	TraesCS5D02G206500	TaMAPKKK29	0.00	0.07	0.02	2040.00	475.58	1564.42	Purifying selection	5.25
TraesCS5B02G204900	TaMAPKKK95	TraesCS5D02G212900	/	0.01	0.05	0.19	1317.00	349.17	967.83	Purifying selection	3.87
TraesCS5B02G292000	TaMAPKKK96	TraesCS5D02G299800	/	0.12	0.35	0.35	1047.00	230.50	816.50	Purifying selection	26.85
TraesCS5B02G337300	TaMAPKKK92	TraesCS5D02G343000	/	0.00	0.05	0.03	1788.00	416.83	1371.17	Purifying selection	4.21
TraesCS5B02G353600	TaMAPKKK132	TraesCS5D02G357900	/	0.17	0.65	0.26	861.00	221.25	639.75	Purifying selection	50.33
TraesCS5B02G353800	TaMAPKKK97	TraesCS5D02G358600	/	0.13	0.54	0.24	672.00	156.25	515.75	Purifying selection	41.39
TraesCS5B02G354400	/	TraesCS5D02G358200	TaMAPKKK123-1	0.12	0.43	0.28	1362.00	310.58	1051.42	Purifying selection	32.94
TraesCS5B02G380400	/	TraesCS5D02G386800	TaMAPKKK86	0.00	0.10	0.01	1119.00	263.50	855.50	Purifying selection	7.47
TraesCS5B02G397300	TaMAPKKKK12	TraesCS5D02G402300	TaMAPKKKK14	0.00	0.03	0.09	2466.00	593.42	1872.58	Purifying selection	2.58
TraesCS5B02G474500	TaMAPKKK16	TraesCS5D02G475900	TaMAPKKK11	0.00	0.08	0.04	1602.00	371.92	1230.08	Purifying selection	6.33
TraesCS5B02G481700	/	TraesCS5D02G482000	TaMAPKKK98	0.01	0.07	0.10	1245.00	299.00	946.00	Purifying selection	5.67
TraesCS5B02G536500	TaMAPK50	TraesCS5D02G534000	TaMAPK26	0.01	0.06	0.17	1320.00	312.08	1007.92	Purifying selection	4.88
TraesCS5B02G565100	TaMAPKK2	TraesCS5D02G549600	TaMAPKK13	0.02	0.06	0.37	1164.00	271.42	892.58	Purifying selection	4.34

TraesCS6A02G004500	TaMAPKKK71	TraesCS6B02G013900	/	0.20	0.26	0.74	420.00	93.50	326.50	Purifying selection	20.24
TraesCS6A02G099600	TaMAPK35	TraesCS6B02G127800	TaMAPK17	0.03	0.08	0.36	1494.00	350.42	1143.58	Purifying selection	6.25
TraesCS6A02G099600	TaMAPK35	TraesCS6D02G082900	TaMAPK44	0.01	0.06	0.19	1500.00	349.33	1150.67	Purifying selection	4.58
TraesCS6A02G118100	TaMAPK36	TraesCS6B02G146300	TaMAPK10	0.00	0.10	0.01	1107.00	254.83	852.17	Purifying selection	7.40
TraesCS6A02G118100	TaMAPK36	TraesCS6D02G108100	TaMAPK45	0.01	0.11	0.08	1140.00	263.17	876.83	Purifying selection	8.15
TraesCS6A02G118100	TaMAPK36	TraesCS7A02G422500	TaMAPK12	0.05	1.03	0.04	1107.00	254.92	852.08	Purifying selection	79.31
TraesCS6A02G118100	TaMAPK36	TraesCS7B02G322900	TaMAPK54	0.05	1.14	0.04	1107.00	254.58	852.42	Purifying selection	87.91
TraesCS6A02G118100	TaMAPK36	TraesCS7D02G414700	/	0.05	1.05	0.04	1107.00	254.92	852.08	Purifying selection	81.13
TraesCS6A02G149900	TaMAPKKK21	TraesCS6B02G177800	TaMAPKKK18	0.01	0.12	0.10	1626.00	362.17	1263.83	Purifying selection	9.57
TraesCS6A02G149900	TaMAPKKK21	TraesCS6D02G139200	TaMAPKKK23	0.00	0.11	0.04	1527.00	338.33	1188.67	Purifying selection	8.56
TraesCS6A02G155200	/	TraesCS7D02G474700	TaMAPKKK102	0.09	0.77	0.11	1254.00	292.42	961.58	Purifying selection	59.41
TraesCS6A02G172600	TaMAPKKK41	TraesCS7A02G388800	/	0.19	0.60	0.32	1014.00	243.33	770.67	Purifying selection	46.04
TraesCS6A02G172600	TaMAPKKK41	TraesCS7B02G290700	/	0.19	0.59	0.32	1014.00	243.67	770.33	Purifying selection	45.25
TraesCS6A02G172600	TaMAPKKK41	TraesCS7D02G384700	TaMAPKKK61	0.19	0.59	0.32	1014.00	243.67	770.33	Purifying selection	45.25
TraesCS6A02G186100	/	TraesCS6B02G215100	TaMAPKKK131	0.01	0.03	0.28	2580.00	621.58	1958.42	Purifying selection	2.66
TraesCS6A02G187400	/	TraesCS6B02G217100	TaMAPKKK155	0.07	0.09	0.72	909.00	217.67	691.33	Purifying selection	7.14
TraesCS6A02G245000	TaMAPKKK4	TraesCS6B02G279300	TaMAPKKK4-1	0.01	0.04	0.17	2358.00	568.58	1789.42	Purifying selection	3.06
TraesCS6A02G245000	TaMAPKKK4	TraesCS6D02G227300	/	0.01	0.04	0.20	2634.00	635.83	1998.17	Purifying selection	2.72

TraesCS6A02G255100	TaMAPKKK39	TraesCS6B02G270400	TaMAPKKK33	0.03	0.08	0.39	1848.00	419.25	1428.75	Purifying selection	5.99
TraesCS6A02G255100	TaMAPKKK39	TraesCS6D02G236400	TaMAPKKK31	0.01	0.04	0.19	1845.00	418.83	1426.17	Purifying selection	3.40
TraesCS6A02G269400	TaMAPK37	TraesCS6B02G296700	TaMAPK1	0.01	0.05	0.13	1380.00	326.00	1054.00	Purifying selection	3.90
TraesCS6A02G269400	TaMAPK37	TraesCS6D02G245500	TaMAPK13	0.00	0.04	0.12	1380.00	326.17	1053.83	Purifying selection	3.15
TraesCS6A02G276300	/	TraesCS7D02G230200	TaMAPKKK51	0.18	0.36	0.50	1206.00	295.58	910.42	Purifying selection	27.65
TraesCS6A02G290500	/	TraesCS6B02G320800	TaMAPKKK129	0.08	0.12	0.61	2223.00	518.83	1704.17	Purifying selection	9.57
TraesCS6A02G353400	TaMAPKKKK16	TraesCS6B02G386100	TaMAPKKKK19	0.01	0.06	0.20	1044.00	243.17	800.83	Purifying selection	4.89
TraesCS6A02G353400	TaMAPKKKK16	TraesCS6D02G335800	TaMAPKKKK20	0.01	0.07	0.19	1044.00	242.83	801.17	Purifying selection	5.25
TraesCS6B02G127800	TaMAPK17	TraesCS6D02G082900	TaMAPK44	0.03	0.09	0.31	1494.00	349.50	1144.50	Purifying selection	6.76
TraesCS6B02G146300	TaMAPK10	TraesCS6D02G108100	TaMAPK45	0.00	0.07	0.02	1107.00	254.67	852.33	Purifying selection	5.05
TraesCS6B02G146300	TaMAPK10	TraesCS7A02G422500	TaMAPK12	0.04	0.98	0.05	1107.00	254.42	852.58	Purifying selection	75.03
TraesCS6B02G146300	TaMAPK10	TraesCS7B02G322900	TaMAPK54	0.05	1.03	0.04	1107.00	254.08	852.92	Purifying selection	79.27
TraesCS6B02G146300	TaMAPK10	TraesCS7D02G414700	/	0.05	1.03	0.04	1107.00	254.42	852.58	Purifying selection	79.05
TraesCS6B02G177800	TaMAPKKKK18	TraesCS6D02G139200	TaMAPKKK23	0.01	0.09	0.13	1527.00	339.00	1188.00	Purifying selection	7.12
TraesCS6B02G183300	/	TraesCS7D02G474700	TaMAPKKK102	0.09	0.80	0.11	1254.00	292.25	961.75	Purifying selection	61.35
TraesCS6B02G200100	/	TraesCS7D02G384700	TaMAPKKK61	0.19	0.59	0.31	1014.00	243.50	770.50	Purifying selection	45.64
TraesCS6B02G215100	TaMAPKKK131	TraesCS6D02G173100	/	0.01	0.04	0.28	2577.00	620.17	1956.83	Purifying selection	2.80
TraesCS6B02G217100	TaMAPKKK155	TraesCS6D02G174300	/	0.09	0.13	0.67	948.00	225.58	722.42	Purifying selection	9.79

TraesCS6B02G217100	TaMAPKKK155	TraesCS7B02G270900	/	0.26	3.26	0.08	879.00	213.33	665.67	Purifying selection	250.45
TraesCS6B02G270400	TaMAPKKK33	TraesCS6D02G236400	TaMAPKKK31	0.03	0.07	0.41	1848.00	418.75	1429.25	Purifying selection	5.19
TraesCS6B02G279300	TaMAPKKK4-1	TraesCS6D02G227300	/	0.01	0.02	0.27	2358.00	570.67	1787.33	Purifying selection	1.78
TraesCS6B02G296700	TaMAPK1	TraesCS6D02G245500	TaMAPK13	0.00	0.03	0.14	1380.00	326.17	1053.83	Purifying selection	2.16
TraesCS6B02G303800	/	TraesCS7D02G230200	TaMAPKKK51	0.17	0.43	0.39	1218.00	297.92	920.08	Purifying selection	32.85
TraesCS6B02G320800	TaMAPKKK129	TraesCS6D02G271500	/	0.07	0.15	0.49	2952.00	706.42	2245.58	Purifying selection	11.39
TraesCS6B02G379500	/	TraesCS6D02G328800	TaMAPKK1	0.01	0.10	0.07	1104.00	300.08	803.92	Purifying selection	7.81
TraesCS6B02G386100	TaMAPKKK19	TraesCS6D02G335800	TaMAPKKK20	0.00	0.04	0.09	2085.00	477.42	1607.58	Purifying selection	3.32
TraesCS6D02G108100	TaMAPK45	TraesCS7A02G422500	TaMAPK12	0.05	1.00	0.05	1107.00	254.75	852.25	Purifying selection	76.70
TraesCS6D02G108100	TaMAPK45	TraesCS7B02G322900	TaMAPK54	0.05	1.07	0.04	1107.00	254.42	852.58	Purifying selection	82.31
TraesCS6D02G108100	TaMAPK45	TraesCS7D02G414700	/	0.05	1.02	0.05	1107.00	254.75	852.25	Purifying selection	78.43
TraesCS6D02G145100	/	TraesCS7D02G474700	TaMAPKKK102	0.09	0.77	0.11	1254.00	292.75	961.25	Purifying selection	59.29
TraesCS6D02G161600	/	TraesCS7D02G384700	TaMAPKKK61	0.21	0.58	0.37	1098.00	267.67	830.33	Purifying selection	44.75
TraesCS6D02G256700	/	TraesCS7D02G230200	TaMAPKKK51	0.18	0.36	0.49	1215.00	297.58	917.42	Purifying selection	27.76
TraesCS6D02G328800	TaMAPKK1	TraesCS7D02G155100	/	0.12	0.42	0.29	1035.00	278.50	756.50	Purifying selection	31.96
TraesCS7A02G032700	TaMAPKKK107	TraesCS7D02G029300	/	0.01	0.06	0.22	1440.00	336.25	1103.75	Purifying selection	4.52
TraesCS7A02G049000	TaMAPK46	TraesCS7D02G044100	TaMAPK18	0.01	0.09	0.15	1452.00	345.42	1106.58	Purifying selection	6.85
TraesCS7A02G111300	TaMAPK19	TraesCS7B02G009200	/	0.00	0.09	0.04	1179.00	272.42	906.58	Purifying selection	7.21

TraesCS7A02G111300	TaMAPK19	TraesCS7D02G106400	/	0.00	0.06	0.05	1179.00	272.50	906.50	Purifying selection	4.70
TraesCS7A02G229600	/	TraesCS7D02G230200	TaMAPKKK51	0.00	0.03	0.13	1233.00	304.50	928.50	Purifying selection	2.58
TraesCS7A02G232300	TaMAPKKKK22	TraesCS7B02G130700	TaMAPKKKK23	0.01	0.06	0.12	2100.00	482.33	1617.67	Purifying selection	4.48
TraesCS7A02G232300	TaMAPKKKK22	TraesCS7D02G232400	TaMAPKKKK24	0.02	0.05	0.37	2067.00	476.33	1590.67	Purifying selection	3.55
TraesCS7A02G326700	TaMAPKKK103	TraesCS7B02G227300	/	0.56	1.01	0.55	537.00	127.08	409.92	Purifying selection	77.84
TraesCS7A02G326700	TaMAPKKK103	TraesCS7D02G323500	/	0.02	0.06	0.28	1602.00	374.58	1227.42	Purifying selection	4.48
TraesCS7A02G335300	TaMAPK3	TraesCS7B02G246900	TaMAPK52	0.00	0.04	0.05	1263.00	287.50	975.50	Purifying selection	3.30
TraesCS7A02G335300	TaMAPK3	TraesCS7D02G342800	TaMAPK7	0.01	0.04	0.14	1263.00	287.67	975.33	Purifying selection	2.74
TraesCS7A02G388800	/	TraesCS7D02G384700	TaMAPKKK61	0.00	0.03	0.03	1188.00	286.33	901.67	Purifying selection	2.47
TraesCS7A02G410700	TaMAPK49	TraesCS7B02G309900	TaMAPK53	0.00	0.10	0.03	1734.00	409.08	1324.92	Purifying selection	8.06
TraesCS7A02G410700	TaMAPK49	TraesCS7D02G403700	TaMAPK20	0.00	0.07	0.05	1734.00	409.83	1324.17	Purifying selection	5.72
TraesCS7A02G422500	TaMAPK12	TraesCS7B02G322900	TaMAPK54	0.01	0.12	0.10	1131.00	260.33	870.67	Purifying selection	8.93
TraesCS7A02G422500	TaMAPK12	TraesCS7D02G414700	/	0.00	0.08	0.04	1107.00	254.50	852.50	Purifying selection	5.88
TraesCS7A02G488200	/	TraesCS7D02G474700	TaMAPKKK102	0.00	0.16	0.01	1275.00	299.08	975.92	Purifying selection	12.28
TraesCS7B02G130700	TaMAPKKKK23	TraesCS7D02G232400	TaMAPKKKK24	0.02	0.09	0.21	2067.00	476.50	1590.50	Purifying selection	6.55
TraesCS7B02G195500	/	TraesCS7D02G230200	TaMAPKKK51	0.00	0.07	0.02	1233.00	303.83	929.17	Purifying selection	5.02
TraesCS7B02G246900	TaMAPK52	TraesCS7D02G342800	TaMAPK7	0.00	0.03	0.11	1263.00	287.67	975.33	Purifying selection	2.18
TraesCS7B02G290700	/	TraesCS7D02G384700	TaMAPKKK61	0.00	0.05	0.05	1188.00	286.67	901.33	Purifying selection	3.60

TraesCS7B02G309900	TaMAPK53	TraesCS7D02G403700	TaMAPK20	0.01	0.09	0.06	1734.00	409.92	1324.08	Purifying selection	6.97
TraesCS7B02G322900	TaMAPK54	TraesCS7D02G414700	/	0.00	0.10	0.04	1107.00	254.17	852.83	Purifying selection	7.42
TraesCS7B02G391300	/	TraesCS7D02G474700	TaMAPKKK102	0.00	0.09	0.02	1275.00	299.42	975.58	Purifying selection	6.81
TraesCS7B02G424500	/	TraesCS7D02G503600	TaMAPKKK54	0.14	0.35	0.41	1350.00	304.25	1045.75	Purifying selection	26.78
TraesCS7B02G425200	/	TraesCS7D02G503600	TaMAPKKK54	0.13	0.46	0.28	1269.00	295.83	973.17	Purifying selection	35.76
TraesCS7B02G427800	/	TraesCS7D02G503600	TaMAPKKK54	0.14	0.42	0.34	1974.00	459.42	1514.58	Purifying selection	32.09
TraesCS7B02G430300	/	TraesCS7D02G503600	TaMAPKKK54	0.14	0.40	0.34	2136.00	493.08	1642.92	Purifying selection	30.81

The Ka/Ks ratio and divergence times between MAPK-MAP4K genes in wheat and *T.dicoccoides*															
**Gene Name**	**Gene Name**	**Gene ID**	**Gene ID**	**Ka**	**Ks**	**Ka/Ks**	**EffectiveLen**	**AverageS-sites**	**Average N-sites**	**cN**	**cS**	**pN**	**pS**	**Divergence times (Mya)**
TaMAPK1		Ta--TraesCS6B02G296700.2	Td--TRIDC6AG041340.3	0.00742	0.05016795112393 05	0.14799	1236	288.5	947.5	7	14	0.007	0.049	3.85907316
TaMAPK1		Ta--TraesCS6B02G296700.2	Td--TRIDC6BG048300.1	0.00104	0		1254	292.67	961.333	1	0	0.001	0	0
TaMAPK2		Ta--TraesCS4A02G336800.2	Td--TRIDC5BG079810.1	0.01875	0.07521017361124 73	0.249335237585 456	1320	312.08	1007.92	18.67	22.33	0.019	0.072	5.78539797
TaMAPK3		Ta--TraesCS4A02G106400.1	Td--TRIDC4AG014520.1	0	0.00391901610199 883	0	1098	255.83	842.167	0	1	0	0.004	0.30146278
TaMAPK3		Ta--TraesCS4A02G106400.1	Td--TRIDC4BG035610.1	0	0.08171338784141 59	0	1107	258.33	848.667	0	20	0	0.077	6.28564522
TaMAPK4		Ta--TraesCS1D02G088000.2	Td--TRIDC1AG012310.2	0.00637	0.10236857568974 9	0.062270480206 5927	1647	386.67	1260.33	8	37	0.006	0.096	7.87450582
TaMAPK4		Ta--TraesCS1D02G088000.2	Td--TRIDC1BG015140.6	0.00398	0.10499874460090 9	0.037918828505 5864	1647	387.83	1259.17	5	38	0.004	0.098	8.07682651

TaMAPK5	Ta--TraesCS1D02G422800.1	Td--TRIDC1AG061100.1	0.05941	0.15305196525909 5	0.388161796728 085	1686	402.08	1283.92	73.33	55.67	0.057	0.138	11.7732281
TaMAPK5	Ta--TraesCS1D02G422800.1	Td--TRIDC1BG070050.1	0.06018	0.15785773034545 3	0.381246126850 021	1680	400.42	1279.58	74	57	0.058	0.142	12.1429023
TaMAPK5	Ta--TraesCS1D02G422800.1	Td--TRIDC3AG034650.7	0.14228	1.25243872352443	0.113600673522 565	1470	337	1133	146.8	205.2	0.13	0.609	96.3414403
TaMAPK5	Ta--TraesCS1D02G422800.1	Td--TRIDC3BG039070.1 2	0.13336	1.3757077464143	0.096942581267 3929	1482	344.33	1137.67	139	217	0.122	0.63	105.823673
TaMAPK6	Ta--TraesCS7B02G009200.1	Td--TRIDC7AG012990.2	0.12506	0.30602701793458 1	0.408641154673 493	1032	243.42	788.583	90.83	61.17	0.115	0.251	23.5405398
TaMAPK6	Ta--TraesCS7B02G009200.1	Td--TRIDC7BG001200.2	0	0	NaN	1077	246.33	830.667	0	0	0	0	0
TaMAPK7	Ta--TraesCS7D02G342800.3	Td--TRIDC7AG047060.4	0.00514	0.03924795542276 89	0.131065504737 014	1263	287.67	975.333	5	11	0.005	0.038	3.01907349
TaMAPK7	Ta--TraesCS7D02G342800.3	Td--TRIDC7BG040680.1	0.00308	0.02833867217098 47	0.108762898278 818	1263	287.67	975.333	3	8	0.003	0.028	2.17989786

TaMAPK8	Ta--TraesCS3D02G225600.1	Td--TRIDC1AG061940.2	0.14901	1.03601193131669	0.143829560602657	1767	420.25	1346.75	182	236	0.135	0.562	79.6932255
TaMAPK8	Ta--TraesCS3D02G225600.1	Td--TRIDC1BG071030.1	0.14681	1.10004784487189	0.133456406945343	1770	423.75	1346.25	179.5	244.5	0.133	0.577	84.619065
TaMAPK8	Ta--TraesCS3D02G225600.1	Td--TRIDC3AG036190.14	0.19656	2.89929980159109	0.067795825020655	1707	395.17	1311.83	226.8	290.2	0.173	0.734	223.023062
TaMAPK8	Ta--TraesCS3D02G225600.1	Td--TRIDC3AG034020.1	0.00142	0.0451532097387966	0.031546313445 7483	1839	433.58	1405.42	2	19	0.001	0.044	3.47332383
TaMAPK8	Ta--TraesCS3D02G225600.1	Td--TRIDC3BG040600.18	0.2013	2.44373001620979	0.082373895670 3154	1722	396.58	1325.42	234	286	0.177	0.721	187.979232
TaMAPK8	Ta--TraesCS3D02G225600.1	Td--TRIDC3BG038510.2	0.00357	0.0281173106948522	0.126943531733047	1839	434.83	1404.17	5	12	0.004	0.028	2.16287005
TaMAPK10	Ta--TraesCS6B02G146300.1	Td--TRIDC6AG015690.2	0	0.096232086482528	0	1107	254.67	852.333	0	23	0	0.09	7.40246819
TaMAPK10	Ta--TraesCS6B02G146300.1	Td--TRIDC6BG021470.2	0	0.0118814365992434	0	1107	254.5	852.5	0	3	0	0.012	0.91395666

TaMAPK10	Ta--TraesCS6B02G146300.1	Td--TRIDC7AG058780.3	0.04491	0.98990566347023 4	0.045369209752 9397	1107	254.42	852.583	37.17	139.8	0.044	0.55	76.1465895
TaMAPK10	Ta--TraesCS6B02G146300.1	Td--TRIDC7BG051510.2	0.04552	1.000038 26810639	0.045514051473 1277	1107	254.08	852.917	37.67	140.3	0.044	0.552	76.9260206
TaMAPK11	Ta--TraesCS1A02G086500.1	Td--TRIDC1AG012310.2	0.00079	0.00259291528789 334	0.306084379777 707	1647	386.33	1260.67	1	1	0.001	0.003	0.19945502
TaMAPK11	Ta--TraesCS1A02G086500.1	Td--TRIDC1BG015140.6	0.00478	0.09624168715841 6	0.049656119211 2174	1647	387.5	1259.5	6	35	0.005	0.09	7.4032067
TaMAPK12	Ta--TraesCS7A02G422500.1	Td--TRIDC6AG015690.2	0.05572	1.052637 60077497	0.052936062441 8997	1134	261.92	872.083	46.83	148.2	0.054	0.566	80.9721231
TaMAPK12	Ta--TraesCS7A02G422500.1	Td--TRIDC6BG021470.2	0.06406	0.99781042704407 4	0.064200307641 729	1161	270.67	890.333	54.67	149.3	0.061	0.552	76.7546482
TaMAPK12	Ta--TraesCS7A02G422500.1	Td--TRIDC7AG058780.3	0.02452	0.02921313302862 25	0.839272417346 312	1152	267.58	884.417	21.33	7.667	0.024	0.029	2.24716408
TaMAPK12	Ta--TraesCS7A02G422500.1	Td--TRIDC7BG051510.2	0.00963	0.08987399964540 92	0.107122380206 223	1236	295.17	940.833	9	25	0.01	0.085	6.91338459

TaMAPK13	Ta--TraesCS6D02G245500.3	Td--TRIDC6AG041340.3	0.0053	0.04276692896976 59	0.123848784815 936	1236	288.67	947.333	5	12	0.005	0.042	3.28976377
TaMAPK13	Ta--TraesCS6D02G245500.3	Td--TRIDC6BG048300.1	0.00313	0.02782927954047 14	0.112389526965 24	1254	292.83	961.167	3	8	0.003	0.027	2.14071381
TaMAPK14	Ta--TraesCS1A02G184500.1	Td--TRIDC1AG027930.1	0	0	NaN	1044	244.5	799.5	0	0	0	0	0
TaMAPK14	Ta--TraesCS1A02G184500.1	Td--TRIDC1BG032170.1	0.00233	0.07040997353804 11	0.033074041835 4893	1128	267.83	860.167	2	18	0.002	0.067	5.41615181
TaMAPK16	Ta--TraesCS3B02G270200.1	Td--TRIDC1AG059290.6	0.11603	1.064362 57300333	0.109009107519 06	1587	365.25	1221.75	131.3	207.7	0.107	0.569	81.8740441
TaMAPK16	Ta--TraesCS3B02G270200.1	Td--TRIDC1BG068060.2	0.13001	1.213490 6196101	0.107138444752 693	1647	378.5	1268.5	151.4	227.6	0.119	0.601	93.3454323
TaMAPK16	Ta--TraesCS3B02G270200.1	Td--TRIDC3AG036190.1 4	0.01956	0.05448622699735 64	0.358909602911 44	1752	396.42	1355.58	26.17	20.83	0.019	0.053	4.19124823
TaMAPK16	Ta--TraesCS3B02G270200.1	Td--TRIDC3AG034020.1	0.19677	2.759121 83869536	0.071317328430 7232	1722	396	1326	229.5	289.5	0.173	0.731	212.240141

TaMAPK16	Ta--TraesCS3B02G270200.1	Td--TRIDC3BG040600.18	0.01727	0.0149152642820377	1.15782553175512	1752	395	1357	23.17	5.833	0.017	0.015	1.14732802
TaMAPK16	Ta--TraesCS3B02G270200.1	Td--TRIDC3BG038510.2	0.19226	2.43413962795926	0.0789831845357364	1722	397.25	1324.75	224.7	286.3	0.17	0.721	187.24151
TaMAPK17	Ta--TraesCS6B02G127800.1	Td--TRIDC6BG018000.3	0.02338	0.0348020073141141	0.671757693922535	1446	338.17	1107.83	25.5	11.5	0.023	0.034	2.67707749
TaMAPK18	Ta--TraesCS7D02G044100.1	Td--TRIDC4AG065120.1	0.01415	0.0814097621275007	0.173855279698641	1449	343.5	1105.5	15.5	26.5	0.014	0.077	6.26228939
TaMAPK18	Ta--TraesCS7D02G044100.1	Td--TRIDC7AG005070.14	0.0146	0.0890387506427807	0.163974799304442	1452	345.42	1106.58	16	29	0.014	0.084	6.84913466
TaMAPK19	Ta--TraesCS7A02G111300.1	Td--TRIDC7AG012990.2	0.12238	0.20642145853537	0.592883157990451	1032	243.83	788.167	89	44	0.113	0.18	15.8785737
TaMAPK19	Ta--TraesCS7A02G111300.1	Td--TRIDC7BG001200.2	0.0012	0.0951942122214532	0.0126526258242493	1077	246.08	830.917	1	22	0.001	0.089	7.32263171
TaMAPK20	Ta--TraesCS7D02G403700.1	Td--TRIDC7AG057270.3	0.00454	0.0716351513763928	0.0634450338639796	1734	409.83	1324.17	6	28	0.005	0.068	5.51039626

TaMAPK20	Ta--TraesCS7D02G403700.1	Td--TRIDC7BG049670.7	0.00531	0.09064693841771 07	0.058528142988 5363	1734	409.92	1324.08	7	35	0.005	0.085	6.97284142
TaMAPK23	Ta--TraesCS3D02G221700.1	Td--TRIDC1AG061100.1	0.12144	1.29162944784723	0.094023009198 9617	1428	327.25	1100.75	123.4	201.6	0.112	0.616	99.3561114
TaMAPK23	Ta--TraesCS3D02G221700.1	Td--TRIDC1BG070050.1	0.12329	1.27516804392155	0.096682257209 2444	1428	327.75	1100.25	125.1	200.9	0.114	0.613	98.0898495
TaMAPK23	Ta--TraesCS3D02G221700.1	Td--TRIDC3AG034650.7	0.02343	0.07484782846617 32	0.313043225405 584	1464	336.92	1127.08	26	24	0.023	0.071	5.75752527
TaMAPK23	Ta--TraesCS3D02G221700.1	Td--TRIDC3BG039070.12	0.02064	0.07621556842761 55	0.270859093568 191	1446	336.92	1109.08	22.58	24.42	0.02	0.072	5.86273603
TaMAPK24	Ta--TraesCS3D02G242200.2	Td--TRIDC1AG059290.6	0.11534	1.0223955958083	0.112815898147 014	1584	364.92	1219.08	130.3	203.7	0.107	0.558	78.6458151
TaMAPK24	Ta--TraesCS3D02G242200.2	Td--TRIDC1BG068060.2	0.13027	1.21194965965697	0.107487091284 717	1641	377.58	1263.42	151.1	226.9	0.12	0.601	93.2268969
TaMAPK24	Ta--TraesCS3D02G242200.2	Td--TRIDC3AG036190.14	0.00264	0.02201280150770 55	0.120014005113 195	1719	391.83	1327.17	3.5	8.5	0.003	0.022	1.69329242

TaMAPK24	Ta--TraesCS3D02G242200.2	Td--TRIDC3AG034020.1	0.19625	3.61641987712469	0.054265477911 6772	1722	396.75	1325.25	228.8	295.2	0.173	0.744	278.186144
TaMAPK24	Ta--TraesCS3D02G242200.2	Td--TRIDC3BG040600.18	0.01158	0.0511673778753061	0.226337143047591	1743	394.25	1348.75	15.5	19.5	0.011	0.049	3.93595214
TaMAPK24	Ta--TraesCS3D02G242200.2	Td--TRIDC3BG038510.2	0.19173	2.87022581694835	0.066799542312 1429	1722	398	1324	224	292	0.169	0.734	220.786601
TaMAPK25	Ta--TraesCS4D02G198600.1	Td--TRIDC4AG014520.1	0.00119	0.0609678778859364	0.019499207364 8951	1098	256.17	841.833	1	15	0.001	0.059	4.68983676
TaMAPK25	Ta--TraesCS4D02G198600.1	Td--TRIDC4BG035610.1	0.00118	0.0687813198671241	0.017151593639 4262	1107	258.67	848.333	1	17	0.001	0.066	5.29087076
TaMAPK26	Ta--TraesCS5D02G534000.2	Td--TRIDC5BG079810.1	0.012	0.0634946176153795	0.189012180999 085	1320	312.08	1007.92	12	19	0.012	0.061	4.88420136
TaMAPK27	Ta--TraesCS1B02G104900.1	Td--TRIDC1AG012310.2	0.00638	0.102132733646731	0.062455745230 2269	1647	387.5	1259.5	8	37	0.006	0.095	7.85636413
TaMAPK27	Ta--TraesCS1B02G104900.1	Td--TRIDC1BG015140.6	0.0008	0.0025773221239359	0.308507544009 172	1647	388.67	1258.33	1	1	0.001	0.003	0.19825555

TaMAPK28	Ta--TraesCS1B02G192600.3	Td--TRIDC1AG027930.1	0	0.0728623919963763	0	1044	244.83	799.167	0	17	0	0.069	5.60479938
TaMAPK28	Ta--TraesCS1B02G192600.3	Td--TRIDC1BG032170.1	0	0	NaN	1128	268.17	859.833	0	0	0	0	0
TaMAPK30	Ta--TraesCS7A02G335300.2	Td--TRIDC7AG047060.4	0	0.00348635142598846	0	1263	287.5	975.5	0	1	0	0.003	0.26818088
TaMAPK30	Ta--TraesCS7A02G335300.2	Td--TRIDC7BG040680.1	0.00205	0.0429455406475612	0.0478056169421855	1263	287.5	975.5	2	12	0.002	0.042	3.30350313
TaMAPK31	Ta--TraesCS4A02G434800.1	Td--TRIDC4AG065120.1	0.0009	0.00291121180663198	0.310251040394682	1452	344.17	1107.83	1	1	0.001	0.003	0.22393937
TaMAPK31	Ta--TraesCS4A02G434800.1	Td--TRIDC7AG005070.14	0.01833	0.0990874493794773	0.185029679252049	1449	344.75	1104.25	20	32	0.018	0.093	7.62211149
TaMAPK33	Ta--TraesCS1D02G410100.1	Td--TRIDC1BG068060.2	0.00948	0.112849792914552	0.0840169518000014	1665	391.33	1273.67	12	41	0.009	0.105	8.6807533
TaMAPK33	Ta--TraesCS1D02G410100.1	Td--TRIDC3AG036190.14	0.12441	1.23950861579893	0.100372044934739	1632	378.08	1253.92	143.8	229.2	0.115	0.606	95.3468166

TaMAPK33	Ta--TraesCS1D02G410100.1	Td--TRIDC3BG040600.18	0.12406	1.27739892174097	0.097117671844 1689	1629	376.17	1252.83	143.3	230.7	0.114	0.613	98.2614555
TaMAPK34	Ta--TraesCS1D02G428900.1	Td--TRIDC1AG061940.2	0.00813	0.0990106916882497	0.082149388108 0352	1791	431.25	1359.75	11	40	0.008	0.093	7.61620705
TaMAPK34	Ta--TraesCS1D02G428900.1	Td--TRIDC1BG071030.1	0.00664	0.100764797139255	0.065860260255 9447	1797	434.83	1362.17	9	41	0.007	0.094	7.75113824
TaMAPK34	Ta--TraesCS1D02G428900.1	Td--TRIDC3AG034020.1	0.15651	1.01117136838978	0.154783225167 804	1770	420.25	1349.75	190.7	233.3	0.141	0.555	77.782413
TaMAPK34	Ta--TraesCS1D02G428900.1	Td--TRIDC3BG038510.2	0.15439	1.00940089350214	0.152954661439 063	1770	421.5	1348.5	188.2	233.8	0.14	0.555	77.6462226
TaMAPK35	Ta--TraesCS6A02G099600.1	Td--TRIDC6BG018000.3	0.02935	0.0617791840474554	0.475084377523 57	1449	337.25	1111.75	32	20	0.029	0.059	4.75224493
TaMAPK36	Ta--TraesCS6A02G118100.1	Td--TRIDC6AG015690.2	0.00117	0.00787925828566064	0.149107725072 188	1107	255.17	851.833	1	2	0.001	0.008	0.60609679
TaMAPK36	Ta--TraesCS6A02G118100.1	Td--TRIDC6BG021470.2	0.00117	0.0872351457577674	0.013465080666 2304	1107	255	852	1	21	0.001	0.082	6.71039583

TaMAPK36	Ta--TraesCS6A02G118100.1	Td--TRIDC7AG058780.3	0.04619	1.04669985672484	0.0441248709000351	1107	254.92	852.083	38.17	143.8	0.045	0.564	80.5153736
TaMAPK36	Ta--TraesCS6A02G118100.1	Td--TRIDC7BG051510.2	0.04679	1.10759507837085	0.0422455256133219	1107	254.58	852.417	38.67	147.3	0.045	0.579	85.1996214
TaMAPK37	Ta--TraesCS6A02G269400.1	Td--TRIDC6AG041340.3	0	0	NaN	1236	288.5	947.5	0	0	0	0	0
TaMAPK37	Ta--TraesCS6A02G269400.1	Td--TRIDC6BG048300.1	0.00627	0.0494296489342025	0.126795275271909	1254	292.67	961.333	6	14	0.006	0.048	3.80228069
TaMAPK38	Ta--TraesCS3B02G256700.1	Td--TRIDC1AG061940.2	0.14718	1.02024043069682	0.144261217630693	1761	419.08	1341.92	179.3	233.7	0.134	0.558	78.4800331
TaMAPK38	Ta--TraesCS3B02G256700.1	Td--TRIDC1BG071030.1	0.14498	1.10354201974222	0.131374418531363	1764	422.58	1341.42	176.8	244.2	0.132	0.578	84.8878477
TaMAPK38	Ta--TraesCS3B02G256700.1	Td--TRIDC3AG034020.1	0.00218	0.0460285687371147	0.0472839308150044	1806	425.58	1380.42	3	19	0.002	0.045	3.54065913
TaMAPK38	Ta--TraesCS3B02G256700.1	Td--TRIDC3AG036190.14	0.19047	2.94021897914399	0.0647806723151071	1677	387.92	1289.08	216.8	285.2	0.168	0.735	226.170691

TaMAPK38	Ta--TraesCS3B02G256700.1	Td--TRIDC3BG038510.2	0.00145	0		1806	426.83	1379.17	2	0	0.001	0	0
TaMAPK38	Ta--TraesCS3B02G256700.1	Td--TRIDC3BG040600.18	0.19532	2.5306265319557	0.0771809137839172	1692	389.33	1302.67	224	282	0.172	0.724	194.663579
TaMAPK39	Ta--TraesCS3A02G242100.1	Td--TRIDC1AG059290.6	0.12871	1.02630359727683	0.125412317214093	1608	369.33	1238.67	146.5	206.5	0.118	0.559	78.9464306
TaMAPK39	Ta--TraesCS3A02G242100.1	Td--TRIDC3AG036190.14	0.00264	0.00641166546451419	0.411959428703249	1719	391.58	1327.42	3.5	2.5	0.003	0.006	0.49320504
TaMAPK39	Ta--TraesCS3A02G242100.1	Td--TRIDC3AG034020.1	0.19346	2.99299959956051	0.0646376983010901	1722	396.67	1325.33	226	292	0.171	0.736	230.230738
TaMAPK39	Ta--TraesCS3A02G242100.1	Td--TRIDC3BG040600.18	0.00415	0.0327134279906905	0.126904346259797	1719	390.5	1328.5	5.5	12.5	0.004	0.032	2.51641754
TaMAPK39	Ta--TraesCS3A02G242100.1	Td--TRIDC3BG038510.2	0.18896	2.57727262393975	0.073317037835729 8	1722	397.92	1324.08	221.2	288.8	0.167	0.726	198.25174
TaMAPK40	Ta--TraesCS3A02G231700.1	Td--TRIDC1AG061100.1	0.13542	1.23525197428024	0.109628540014771	1443	328.5	1114.5	138.1	198.9	0.124	0.606	95.0193826

TaMAPK40	Ta--TraesCS3A02G231700.1	Td--TRIDC1BG070050.1	0.13728	1.26799751426752	0.108265165489214	1443	329	1114	139.8	201.3	0.125	0.612	97.5382703
TaMAPK40	Ta--TraesCS3A02G231700.1	Td--TRIDC3AG034650.7	0.00088	0.00298211128472955	0.2958819972499 1	1470	336	1134	1	1	0.001	0.003	0.22939318
TaMAPK40	Ta--TraesCS3A02G231700.1	Td--TRIDC3BG039070.12	0.03908	0.09729470696099 99	0.401692319318875	1449	335.17	1113.83	42.42	30.58	0.038	0.091	7.48420823
TaMAPK42	Ta--TraesCS1A02G415300.1	Td--TRIDC1AG061100.1	0.05393	0.13957788828226	0.386410524665208	1686	401.75	1284.25	66.83	51.17	0.052	0.127	10.7367606
TaMAPK42	Ta--TraesCS1A02G415300.1	Td--TRIDC1BG070050.1	0.06061	0.153177502270747	0.395713402283722	1680	400.58	1279.42	74.5	55.5	0.058	0.139	11.7828848
TaMAPK42	Ta--TraesCS1A02G415300.1	Td--TRIDC3AG034650.7	0.14524	1.27658932962714	0.113775712787159	1473	337.67	1135.33	149.9	207.1	0.132	0.613	98.1991792
TaMAPK42	Ta--TraesCS1A02G415300.1	Td--TRIDC3BG039070.12	0.13028	1.33481156665734	0.0976045111485081	1482	344.83	1137.17	136	215	0.12	0.623	102.677813
TaMAPK43	Ta--TraesCS1A02G421000.1	Td--TRIDC1AG061940.2	0	0.00463143115923732	0	1794	433.17	1360.83	0	2	0	0.005	0.35626394

TaMAPK43	Ta--TraesCS1A02G421000.1	Td--TRIDC1BG071030.1	0.01037	0.127568052158476	0.0812991626219065	1794	434.75	1359.25	14	51	0.01	0.117	9.81292709
TaMAPK43	Ta--TraesCS1A02G421000.1	Td--TRIDC3AG034020.1	0.15032	0.996620422827743	0.15082720205762	1767	419.83	1347.17	183.5	231.5	0.136	0.551	76.6631094
TaMAPK43	Ta--TraesCS1A02G421000.1	Td--TRIDC3BG038510.2	0.14905	0.997397036629437	0.149438936255203	1767	420.58	1346.42	182	232	0.135	0.552	76.722849
TaMAPK44	Ta--TraesCS6D02G082900.2	Td--TRIDC6BG018000.3	0.02699	0.0733432163085657	0.368034708880667	1449	336.33	1112.67	29.5	23.5	0.027	0.07	5.64178587
TaMAPK45	Ta--TraesCS6D02G108100.1	Td--TRIDC6AG015690.2	0.00117	0.105066594878011	0.0111798452773931	1107	255	852	1	25	0.001	0.098	8.08204576
TaMAPK45	Ta--TraesCS6D02G108100.1	Td--TRIDC6BG021470.2	0.00117	0.0528315849197334	0.0222290957711989	1107	254.83	852.167	1	13	0.001	0.051	4.06396807
TaMAPK45	Ta--TraesCS6D02G108100.1	Td--TRIDC7AG058780.3	0.04659	1.01202457519198	0.0460386591681408	1107	254.75	852.25	38.5	141.5	0.045	0.555	77.8480442
TaMAPK45	Ta--TraesCS6D02G108100.1	Td--TRIDC7BG051510.2	0.0472	1.03800311918125	0.0454697531536604	1107	254.42	852.583	39	143	0.046	0.562	79.8463938

TaMAPK46	Ta--TraesCS7A02G049000.1	Td--TRIDC4AG065120.1	0.01833	0.09908744937947 73	0.185029679252049	1449	344.75	1104.25	20	32	0.018	0.093	7.62211149
TaMAPK46	Ta--TraesCS7A02G049000.1	Td--TRIDC7AG005070.14	0.00181	0.00289017698699247	0.626811123282763	1452	346.67	1105.33	2	1	0.002	0.003	0.22232131
TaMAPK47	Ta--TraesCS7A02G029700.1	Td--TRIDC7AG002740.1	0.02167	0.0320781997245268	0.675520783767096	972	222.92	749.083	16	7	0.021	0.031	2.46755382
TaMAPK49	Ta--TraesCS7A02G410700.2	Td--TRIDC7AG057270.3	0.00076	0.00244898176780432	0.308330987928357	1734	409	1325	1	1	0.001	0.002	0.18838321
TaMAPK49	Ta--TraesCS7A02G410700.2	Td--TRIDC7BG049670.7	0.00303	0.104767985475068	0.0288747618679432	1734	409.08	1324.92	4	40	0.003	0.098	8.05907581
TaMAPK50	Ta--TraesCS5B02G536500.1	Td--TRIDC5BG079810.1	0.00099	0.00955426933307228	0.103390121521531	1329	316	1013	1	3	0.001	0.009	0.73494379
TaMAPK52	Ta--TraesCS7B02G246900.3	Td--TRIDC7AG047060.4	0.00205	0.046637855797338	0.0440208502400651	1263	287.5	975.5	2	13	0.002	0.045	3.58752737
TaMAPK52	Ta--TraesCS7B02G246900.3	Td--TRIDC7BG040680.1	0	0.00698898476240196	0	1263	287.5	975.5	0	2	0	0.007	0.53761421

TaMAPK53	Ta--TraesCS7B02G309900.1	Td--TRIDC7AG057270.3	0.00378	0.107584229103314	0.035166387714 3551	1734	409.08	1324.92	5	41	0.004	0.1	8.27570993
TaMAPK53	Ta--TraesCS7B02G309900.1	Td--TRIDC7BG049670.7	0	0	NaN	1734	409.17	1324.83	0	0	0	0	0
TaMAPK54	Ta--TraesCS7B02G322900.1	Td--TRIDC6AG015690.2	0.05496	1.11459355060578	0.0493138690160926	1131	259.92	871.083	46.17	150.8	0.053	0.58	85.7379654
TaMAPK54	Ta--TraesCS7B02G322900.1	Td--TRIDC6BG021470.2	0.05436	1.08793777907221	0.0499645459299791	1131	260.08	870.917	45.67	149.3	0.052	0.574	83.6875215
TaMAPK54	Ta--TraesCS7B02G322900.1	Td--TRIDC7AG058780.3	0.00701	0.10028824829668	0.0698652976751843	1116	255.67	860.333	6	24	0.007	0.094	7.71448064
TaMAPK54	Ta--TraesCS7B02G322900.1	Td--TRIDC7BG051510.2	0.00578	0.0235313419258153	0.245456626690206	1128	259	869	5	6	0.006	0.023	1.81010323
TaMAPKK1	Ta--TraesCS6D02G328800.1	Td--TRIDC6AG052120.1	0.00499	0.0736788296875599	0.0676860827306238	1104	299.25	804.75	4	21	0.005	0.07	5.66760228
TaMAPKK1	Ta--TraesCS6D02G328800.1	Td--TRIDC6BG060870.1	0.00687	0.105373589984994	0.0652241261882785	1104	300.08	803.917	5.5	29.5	0.007	0.098	8.10566077

TaMAPKK 2	Ta--TraesCS5B02G565100.3	Td--TRIDC4AG047480.2	0.06775	0.2100996055787 62	0.322489104402995	1296	304.25	991.75	64.25	55.75	0.065	0.183	16.1615081
TaMAPKK 2	Ta--TraesCS5B02G565100.3	Td--TRIDC5BG083460.4	0.04287	0.101395307637	0.42282706860533	1239	289.08	949.917	39.58	27.42	0.042	0.095	7.79963905
TaMAPKK 3	Ta--TraesCS5D02G130900.2	Td--TRIDC5AG020820.4	0.01676	0.0737858123438149	0.227084085437421	885	211.08	673.917	11.17	14.83	0.017	0.07	5.67583172
TaMAPKK 3	Ta--TraesCS5D02G130900.2	Td--TRIDC5BG021620.3	0.04206	0.11534673911063	0.364649363323799	1224	286.83	937.167	38.33	30.67	0.041	0.107	8.87282609
TaMAPKK 4	Ta--TraesCS5A02G122700.4	Td--TRIDC5AG020820.4	0.0542	0.086588072808598	0.625979776360957	843	200.75	642.25	33.58	16.42	0.052	0.082	6.66062099
TaMAPKK 4	Ta--TraesCS5A02G122700.4	Td--TRIDC5BG021620.3	0.02157	0.057212478562223	0.376955266216645	816	196.67	619.333	13.17	10.83	0.021	0.055	4.40095989
TaMAPKK 6	Ta--TraesCS4B02G048100.1	Td--TRIDC4AG041540.1	0.23616	0.512803675557325	0.460526017572017	984	270.33	713.667	144.6	100.4	0.203	0.371	39.4464366
TaMAPKK 6	Ta--TraesCS4B02G048100.1	Td--TRIDC4BG007520.1	0.00134	0.00353774240860418	0.379417742290736	1029	283.33	745.667	1	1	0.001	0.004	0.27213403

TaMAPKK9		Ta--TraesCS3B02G066300.1	Td--TRIDC3BG008530.2	0.03565	0.0358722624266235	0.993698297982508	975	268.83	706.167	24.58	9.417	0.035	0.035	2.7594048
TaMAPKK13		Ta--TraesCS5D02G549600.1	Td--TRIDC4AG047480.2	0.01255	0.161924562122982	0.077515842976551	1569	363.92	1205.08	15	53	0.012	0.146	12.4557355
TaMAPKK13		Ta--TraesCS5D02G549600.1	Td--TRIDC5BG083460.4	0.00835	0.0687503913943679	0.121516475656622	1569	365.33	1203.67	10	24	0.008	0.066	5.28849165
TaMAPKK14		Ta--TraesCS4A02G265900.1	Td--TRIDC4AG041540.1	0.03051	0.135016924595119	0.225974124026006	1011	275.17	735.833	22	34	0.03	0.124	10.3859173
TaMAPKK14		Ta--TraesCS4A02G265900.1	Td--TRIDC4BG007520.1	0.23736	0.532852466532255	0.445459242121528	984	268.5	715.5	145.6	102.4	0.203	0.381	40.9886513
TaMAPKK18		Ta--TraesCS5B02G122600.1	Td--TRIDC5AG020820.4	0.01677	0.0632731980111069	0.264978963642157	885	211.5	673.5	11.17	12.83	0.017	0.061	4.86716908
TaMAPKK18		Ta--TraesCS5B02G122600.1	Td--TRIDC5BG021620.3	0.01353	0.0452310953758704	0.299033136571675	1266	296.17	969.833	13	13	0.013	0.044	3.47931503
TaMAPKKK1	TaMEKK1	Ta--TraesCS2A02G407600.1	Td--TRIDC2AG058830.1	0	0.00679506346830184	0	2481	591.33	1889.67	0	4	0	0.007	0.52269719

TaMAPKKK1	TaMEKK1	Ta--TraesCS2A02G407600.1	Td--TRIDC2BG061830.1	0.02386	0.07712883466883 74	0.309393814125996	2460	586.67	1873.33	44	43	0.023	0.073	5.93298728
TaMAPKKK1	TaMEKK1	Ta--TraesCS2A02G407600.1	Td--TRIDC6AG037840.4	0.15108	0.907555974135041	0.166471534986 3	2427	579.92	1847.08	252.8	305.3	0.137	0.526	69.811998
TaMAPKKK1	TaMEKK1	Ta--TraesCS2A02G407600.1	Td--TRIDC6BG044800.1	0.13714	0.873582894625792	0.156991213467956	2364	567.5	1796.5	225.2	292.8	0.125	0.516	67.1986842
TaMAPKKK2	TaMEKK2	Ta--TraesCS4D02G027600.1	Td--TRIDC4AG043860.6	0.01028	0.142620354827718	0.0721048219900967	1704	431.17	1272.83	13	56	0.01	0.13	10.9707965
TaMAPKKK2	TaMEKK2	Ta--TraesCS4D02G027600.1	Td--TRIDC4BG004990.4	0.01347	0.142772317164011	0.0943595615042622	1704	430.75	1273.25	17	56	0.013	0.13	10.9824859
TaMAPKKK3	TaMEKK3	Ta--TraesCS4B02G210600.2	Td--TRIDC4AG012750.6	0.00816	0.0715313693800487	0.114062911879357	2115	513	1602	13	35	0.008	0.068	5.50241303
TaMAPKKK3	TaMEKK3	Ta--TraesCS4B02G210600.2	Td--TRIDC4BG037560.5	0.00065	0.00413651513929987	0.157031115488524	2025	484.83	1540.17	1	2	0.001	0.004	0.31819347
TaMAPKKK4	TaMEKK4	Ta--TraesCS6A02G245000.3	Td--TRIDC2AG058830.1	0.1511	0.907048947969418	0.166581241928488	2427	580.08	1846.92	252.8	305.3	0.137	0.526	69.772996

TaMAPKKK4	TaMEKK4	Ta--TraesCS6A02G245000.3	Td--TRIDC2BG061830.1	0.15803	0.860417656801211	0.183667254687451	2616	628.75	1987.25	283.2	321.8	0.142	0.512	66.1859736
TaMAPKKK4	TaMEKK4	Ta--TraesCS6A02G245000.3	Td--TRIDC6AG037840.4	0.0005	0		2634	634	2000	1	0	0.001	0	0
TaMAPKKK4	TaMEKK4	Ta--TraesCS6A02G245000.3	Td--TRIDC6BG044800.1	0.00704	0.0371043401542991	0.189670133469085	2634	635.33	1998.67	14	23	0.007	0.036	2.85418001
TaMAPKKK4-1	TaMEKK4-1	Ta--TraesCS6B02G279300.1	Td--TRIDC2AG058830.1	0.13381	0.850934018800818	0.157247342927983	2343	561.42	1781.58	218.3	285.7	0.123	0.509	65.456463
TaMAPKKK4-1	TaMEKK4-1	Ta--TraesCS6B02G279300.1	Td--TRIDC2BG061830.1	0.1348	0.845901079883277	0.159357535114766	2355	566.5	1788.5	220.7	287.3	0.123	0.507	65.0693138
TaMAPKKK4-1	TaMEKK4-1	Ta--TraesCS6B02G279300.1	Td--TRIDC6AG037840.4	0.00617	0.0397384137080505	0.155315924428594	2358	568.42	1789.58	11	22	0.006	0.039	3.05680105
TaMAPKKK4-1	TaMEKK4-1	Ta--TraesCS6B02G279300.1	Td--TRIDC6BG044800.1	0.00056	0		2358	570.33	1787.67	1	0	0.001	0	0
TaMAPKKK5	TaMEKK5	Ta--TraesCS2A02G199700.1	Td--TRIDC2AG025680.4	0.0032	0.00267022978989298	1.19728308453262	1629	375.17	1253.83	4	1	0.003	0.003	0.20540229

TaMAPKKK5	TaMEKK5	Ta--TraesCS2A02G199700.1	Td--TRIDC2BG030100.5	0.0064	0.05828173824548 73	0.109885336644 952	1629	374.5	1254.5	8	21	0.006	0.056	4.48321063
TaMAPKKK7	TaMEKK7	Ta--TraesCS3B02G289500.1	Td--TRIDC1BG061690.1	0.23262	0.60761085358837 8	0.382837460611 696	1047	266.17	780.833	156.2	110.8	0.2	0.416	46.7392964
TaMAPKKK7	TaMEKK7	Ta--TraesCS3B02G289500.1	Td--TRIDC3BG043440.2	0.00515	0		1305	331.08	973.917	5	0	0.005	0	0
TaMAPKKK8	TaMEKK8	Ta--TraesCS3B02G288100.1	Td--TRIDC1AG053880.1	0.28819	0.66451708882114 4	0.433689228405 294	1251	315.92	935.083	223.7	139.3	0.239	0.441	51.1166991
TaMAPKKK8	TaMEKK8	Ta--TraesCS3B02G288100.1	Td--TRIDC3AG038300.1	0.02495	0.12445843606034 8	0.200507404436 402	1251	313.92	937.083	23	36	0.025	0.115	9.57372585
TaMAPKKK8	TaMEKK8	Ta--TraesCS3B02G288100.1	Td--TRIDC3BG043280.1	0.00104	0.00628275925353 592	0.166201630629 482	1278	319.67	958.333	1	2	0.001	0.006	0.48328917
TaMAPKKK10	TaMEKK10	Ta--TraesCS4D02G211300.2	Td--TRIDC4AG012750.6	0.00685	0.06047599686525 1	0.113234038018 108	2130	516.33	1613.67	11	30	0.007	0.058	4.65199976
TaMAPKKK10	TaMEKK10	Ta--TraesCS4D02G211300.2	Td--TRIDC4BG037560.5	0.00981	0.05998913561944 91	0.163501857133 416	2025	485.67	1539.33	15	28	0.01	0.058	4.61454889

TaMAPKKK11	TaMEKK11	Ta--TraesCS5D02G475900.1	Td--TRIDC5AG066400.8	0.00489	0.0735744981914521	0.066463149503 7367	1602	371	1231	6	26	0.005	0.07	5.65957678
TaMAPKKK11	TaMEKK11	Ta--TraesCS5D02G475900.1	Td--TRIDC5BG071320.5	0.04094	0.125711557053194	0.325667883327722	1548	360	1188	47.33	41.67	0.04	0.116	9.67011977
TaMAPKKK12	TaMEKK12	Ta--TraesCS4A02G093800.2	Td--TRIDC4AG012750.6	0	0	NaN	2130	515.67	1614.33	0	0	0	0	0
TaMAPKKK12	TaMEKK12	Ta--TraesCS4A02G093800.2	Td--TRIDC4BG037560.5	0.00783	0.071328011617978	0.109816174503288	2025	485	1540	12	33	0.008	0.068	5.48677012
TaMAPKKK14	TaMEKK14	Ta--TraesCS5A02G118200.1	Td--TRIDC5AG020280.1	0.00162	0		1611	376	1235	2	0	0.002	0	0
TaMAPKKK15	TaMEKK15	Ta--TraesCS5A02G463100.2	Td--TRIDC5AG066400.8	0.00244	0.00541763078059518	0.450384774775975	1602	370.5	1231.5	3	2	0.002	0.005	0.41674083
TaMAPKKK15	TaMEKK15	Ta--TraesCS5A02G463100.2	Td--TRIDC5BG071320.5	0.03826	0.135837678629234	0.281670712740502	1548	359.5	1188.5	44.33	44.67	0.037	0.124	10.4490522
TaMAPKKK16	TaMEKK16	Ta--TraesCS5B02G474500.1	Td--TRIDC5AG066400.8	0.00163	0.100895472487721	0.016116947849 7392	1602	370.75	1231.25	2	35	0.002	0.094	7.76119019

TaMAPKKK16	TaMEKK16	Ta--TraesCS5B02G474500.1	Td--TRIDC5BG071320.5	0.0373	0.03602872963299 93	1.035252136515 78	1551	360.08	1190.92	43.33	12.67	0.036	0.035	2.77144074
TaMAPKKK17	TaMEKK17	Ta--TraesCS5A02G200800.1	Td--TRIDC5AG032410.5	0.00064	0.00212766100139 979	0.301927010830 03	2028	470.67	1557.33	1	1	0.001	0.002	0.16366623
TaMAPKKK17	TaMEKK17	Ta--TraesCS5A02G200800.1	Td--TRIDC5BG034210.7	0.00192	0.05686818508009 71	0.033746107538 3591	2040	474.75	1565.25	3	26	0.002	0.055	4.37447578
TaMAPKKK18	TaMEKK18	Ta--TraesCS2B02G526200.3	Td--TRIDC2AG070140.10	0.00891	0.08165067844261 2	0.109126597570 206	3966	917.75	3048.25	27	71	0.009	0.077	6.28082142
TaMAPKKK18	TaMEKK18	Ta--TraesCS2B02G526200.3	Td--TRIDC2BG075830.1	0	0.00108833684978 57	0	3969	919.5	3049.5	0	1	0	0.001	0.08371822
TaMAPKKK20	TaMEKK20	Ta--TraesCS2A02G498000.3	Td--TRIDC2AG070140.10	0.00098	0		3966	916.83	3049.17	3	0	0.001	0	0
TaMAPKKK20	TaMEKK20	Ta--TraesCS2A02G498000.3	Td--TRIDC2BG075830.1	0.00858	0.08045214028031 23	0.106620963632 334	3966	917.58	3048.42	26	70	0.009	0.076	6.18862618
TaMAPKKK21/TaMAPKKKK15	TaMEKK21	Ta--TraesCS6A02G149900.1	Td--TRIDC6AG020750.1	0.00079	0		1626	361.33	1264.67	1	0	0.001	0	0

TaMAPKKK21/TaMAPKKKK15	TaMEKK21	Ta--TraesCS6A02G149900.1	Td--TRIDC6BG026600.3	0.01237	0.121067579367253	0.102145258798394	1626	362.25	1263.75	15.5	40.5	0.012	0.112	9.31289072
TaMAPKKK22/TaMAPKKKK10	TaMEKK22	Ta--TraesCS5A02G392500.1	Td--TRIDC5AG057130.2	0.00348	0.00421349422816744	0.825101821093778	2469	595	1874	6.5	2.5	0.003	0.004	0.32411494
TaMAPKKK22/TaMAPKKKK10	TaMEKK22	Ta--TraesCS5A02G392500.1	Td--TRIDC5BG061270.2	0.00683	0.05965876642654	0.114547461718917	2322	558	1764	12	32	0.007	0.057	4.58913588
TaMAPKKK23/TaMAPKKKK21	TaMEKK23	Ta--TraesCS6D02G139200.1	Td--TRIDC6AG020750.1	0.00506	0.111315003838704	0.0454991230793017	1527	338.33	1188.67	6	35	0.005	0.103	8.5626926
TaMAPKKK23/TaMAPKKKK21	TaMEKK23	Ta--TraesCS6D02G139200.1	Td--TRIDC6BG026600.3	0.01231	0.0891441126405373	0.138053566865557	1527	339.08	1187.92	14.5	28.5	0.012	0.084	6.85723943
TaMAPKKK24	TaMEKK24	Ta--TraesCS5B02G199400.1	Td--TRIDC5AG032410.5	0.00193	0.0619707176641425	0.0311268203039521	2028	470.75	1557.25	3	28	0.002	0.059	4.76697828
TaMAPKKK24	TaMEKK24	Ta--TraesCS5B02G199400.1	Td--TRIDC5BG034210.7	0.00064	0		2040	474.83	1565.17	1	0	0.001	0	0
TaMAPKKK25	TaMEKK25	Ta--TraesCS5B02G196400.1	Td--TRIDC5AG032000.2	0.02019	0.0936472148657488	0.215545948366101	1554	382.42	1171.58	23.33	33.67	0.02	0.088	7.20363191

TaMAPKKK25	TaMEKK25	Ta--TraesCS5B02G196400.1	Td--TRIDC5BG033660.2	0.00086	0		1554	385.33	1168.67	1	0	0.001	0	0
TaMAPKKK26	TaMEKK26	Ta--TraesCS2D02G093700.1	Td--TRIDC2AG010980.4	0.00663	0.07743886598097 46	0.085585968921 0269	3996	965	3031	20	71	0.007	0.074	5.95683584
TaMAPKKK26	TaMEKK26	Ta--TraesCS2D02G093700.1	Td--TRIDC2BG013430.2	0.00529	0.04467822018399 91	0.118346929228 289	4005	968.33	3036.67	16	42	0.005	0.043	3.43678617
TaMAPKKK27	TaMEKK27	Ta--TraesCS2B02G110500.1	Td--TRIDC2AG010980.4	0.0073	0.07620071317294 13	0.095751093825 3888	3996	966.08	3029.92	22	70	0.007	0.072	5.86159332
TaMAPKKK27	TaMEKK27	Ta--TraesCS2B02G110500.1	Td--TRIDC2BG013430.2	0.00231	0.03591919564766 26	0.064298047411 3478	4005	969.42	3035.58	7	34	0.002	0.035	2.76301505
TaMAPKKK28	TaMEKK28	Ta--TraesCS2A02G095300.1	Td--TRIDC2AG010980.4	0.00066	0.00519437623026 219	0.127125652210 642	3996	965.92	3030.08	2	5	0.001	0.005	0.3995674
TaMAPKKK28	TaMEKK28	Ta--TraesCS2A02G095300.1	Td--TRIDC2BG013430.2	0.00797	0.07613159092122 91	0.104625916491 222	3996	966.92	3029.08	24	70	0.008	0.072	5.85627622
TaMAPKKK29	TaMEKK29	Ta--TraesCS5D02G206500.1	Td--TRIDC5AG032410.5	0.00193	0.03473848126803 23	0.055548633491 0182	2028	471.33	1556.67	3	16	0.002	0.034	2.67219087

TaMAPKKK29	TaMEKK29	Ta--TraesCS5D02G206500.1	Td--TRIDC5BG034210.7	0.00192	0.06821631341323 87	0.028144274555 5598	2040	475.42	1564.58	3	31	0.002	0.065	5.24740872
TaMAPKKK30	TaZIK1	Ta--TraesCS5D02G145100.1	Td--TRIDC5AG024780.6	0.01057	0.04739552240345 18	0.223037936651 595	1908	479	1429	15	22	0.01	0.046	3.64580942
TaMAPKKK30	TaZIK1	Ta--TraesCS5D02G145100.1	Td--TRIDC5BG025890.5	0.00979	0.07883275503936 9	0.124227580090 338	1920	481.08	1438.92	14	36	0.01	0.075	6.06405808
TaMAPKKK31	TaZIK2	Ta--TraesCS6D02G236400.1	Td--TRIDC6AG039240.3	0.03393	0.05555069505076 19	0.610712438933 908	1164	264.58	899.417	29.83	14.17	0.033	0.054	4.27313039
TaMAPKKK31	TaZIK2	Ta--TraesCS6D02G236400.1	Td--TRIDC6BG046090.3	0.0278	0.06741903645111 25	0.412284227224 11	1848	418.75	1429.25	39	27	0.027	0.064	5.18607973
TaMAPKKK32	TaZIK3	Ta--TraesCS2A02G195900.2	Td--TRIDC2AG025210.3	0	0.00417682945176 646	0	2079	480.17	1598.83	0	2	0	0.004	0.32129457
TaMAPKKK32	TaZIK3	Ta--TraesCS2A02G195900.2	Td--TRIDC2BG029650.2	0.00683	0.07820425883144 67	0.087315673044 2644	2103	484.75	1618.25	11	36	0.007	0.074	6.01571222
TaMAPKKK33	TaZIK4	Ta--TraesCS6B02G270400.1	Td--TRIDC6AG039240.3	0.06121	0.07946559163798 32	0.770213064745 879	1167	265.25	901.75	53	20	0.059	0.075	6.11273782

TaMAPKKK33	TaZIK4	Ta--TraesCS6B02G270400.1	Td--TRIDC6BG046090.3	0	0	NaN	1851	419.17	1431.83	0	0	0	0	0
TaMAPKKK34	TaZIK5	Ta--TraesCS2D02G197600.1	Td--TRIDC2AG024350.1	0.00415	0.04426062857779 13	0.093835490945 5968	957	232.67	724.333	3	10	0.004	0.043	3.40466374
TaMAPKKK34	TaZIK5	Ta--TraesCS2D02G197600.1	Td--TRIDC2BG028790.2	0.00974	0.03952616023433 05	0.246457692889 926	957	233.75	723.25	7	9	0.01	0.039	3.04047386
TaMAPKKK36	TaZIK7	Ta--TraesCS2B02G223600.1	Td--TRIDC2AG025210.3	0.00691	0.07906381339576 63	0.087397230415 9756	2079	479.75	1599.25	11	36	0.007	0.075	6.0818318
TaMAPKKK36	TaZIK7	Ta--TraesCS2B02G223600.1	Td--TRIDC2BG029650.2	0	0.00412939790589 186	0	2109	485.67	1623.33	0	2	0	0.004	0.31764599
TaMAPKKK37	TaZIK8	Ta--TraesCS2B02G216800.1	Td--TRIDC2AG025210.3	0.44259	0.74047632563800 6	0.597711932056 96	864	206.67	657.333	219.8	97.25	0.334	0.471	56.9597174
TaMAPKKK37	TaZIK8	Ta--TraesCS2B02G216800.1	Td--TRIDC2AG024350.1	0.01102	0.08100785008529 73	0.135982976313 737	966	234.42	731.583	8	18	0.011	0.077	6.23137308
TaMAPKKK37	TaZIK8	Ta--TraesCS2B02G216800.1	Td--TRIDC2BG028790.2	0.00274	0.00426743683325 892	0.642301403218 437	966	235	731	2	1	0.003	0.004	0.32826437

TaMAPKKK38	TaZIK9	Ta--TraesCS1D02G026200.2	Td--TRIDC1AG002650.1	0.00573	0.0656432185905535	0.0873005167963942	1815	413.67	1401.33	8	26	0.006	0.063	5.04947835
TaMAPKKK39	TaZIK10	Ta--TraesCS6A02G255100.2	Td--TRIDC6AG039240.3	0.02347	0.019717056650243	1.19049999211312	1167	265.5	901.5	20.83	5.167	0.023	0.019	1.51669667
TaMAPKKK39	TaZIK10	Ta--TraesCS6A02G255100.2	Td--TRIDC6BG046090.3	0.03072	0.0778452659565614	0.394587263577064	1848	419.25	1428.75	43	31	0.03	0.074	5.98809738
TaMAPKKK40	TaZIK11	Ta--TraesCS5B02G146100.1	Td--TRIDC5AG024780.6	0.00915	0.0887460629299847	0.103057175454398	1908	477.92	1430.08	13	40	0.009	0.084	6.82662023
TaMAPKKK40	TaZIK11	Ta--TraesCS5B02G146100.1	Td--TRIDC5BG025890.5	0	0.0020862322214076	0	1920	480	1440	0	1	0	0.002	0.1604794
TaMAPKKK41	TaRaf1	Ta--TraesCS6A02G172600.1	Td--TRIDC6AG024780.3	0.102	0.193907531568042	0.526022867649668	729	178.5	550.5	52.5	30.5	0.095	0.171	14.915964
TaMAPKKK41	TaRaf1	Ta--TraesCS6A02G172600.1	Td--TRIDC7AG054160.3	0.18886	0.598500351155931	0.315549772086887	1014	243.33	770.667	128.7	100.3	0.167	0.412	46.0384886
TaMAPKKK41	TaRaf1	Ta--TraesCS6A02G172600.1	Td--TRIDC7BG046810.3	0.19239	0.578073130868244	0.332816160737427	1014	244	770	130.7	98.33	0.17	0.403	44.4671639

TaMAPKKK45	TaRaf5	Ta--TraesCS3D02G273200.1	Td--TRIDC1AG050190.1	0.33168	1.31851456219555	0.251554379526124	1740	412.83	1327.17	355.7	256.3	0.268	0.621	101.424197
TaMAPKKK45	TaRaf5	Ta--TraesCS3D02G273200.1	Td--TRIDC1BG057000.1	0.32772	1.29084951575606	0.25388224420573	1740	411.08	1328.92	352.8	253.2	0.266	0.616	99.2961166
TaMAPKKK45	TaRaf5	Ta--TraesCS3D02G273200.1	Td--TRIDC3AG040710.1	0.01757	0.0887671886532731	0.197949512506304	1800	418.08	1381.92	24	35	0.017	0.084	6.82824528
TaMAPKKK45	TaRaf5	Ta--TraesCS3D02G273200.1	Td--TRIDC3BG045870.3	0.01238	0.0701533371907363	0.176423091941957	1803	418.08	1384.92	17	28	0.012	0.067	5.39641055
TaMAPKKK46	TaRaf6	Ta--TraesCS2D02G050700.1	Td--TRIDC2AG005450.2	0.03582	0.0801537454781221	0.446920837241676	2454	588.67	1865.33	65.25	44.75	0.035	0.076	6.16567273
TaMAPKKK48	TaRaf8	Ta--TraesCS7D02G079100.1	Td--TRIDC7AG009510.2	0.12735	0.296344597734739	0.429729677562347	1596	360.83	1235.17	144.7	88.33	0.117	0.245	22.7957383
TaMAPKKK50	TaRaf10	Ta--TraesCS7D02G099200.2	Td--TRIDC7AG012240.3	0.03	0.0730025354605975	0.410906376592397	1974	472	1502	44.17	32.83	0.029	0.07	5.61557965
TaMAPKKK51	TaRaf11	Ta--TraesCS7D02G230200.1	Td--TRIDC6AG042270.3	0.23143	0.524580545441436	0.441171081324421	1074	270.08	803.917	160.1	101.9	0.199	0.377	40.3523496

TaMAPKKK51	TaRaf11	Ta--TraesCS7D02G230200.1	Td--TRIDC6BG049330.2	0.16598	0.43299307974882 6	0.383339541419749	1218	297.92	920.083	137	98	0.149	0.329	33.30716
TaMAPKKK51	TaRaf11	Ta--TraesCS7D02G230200.1	Td--TRIDC7AG029400.6	0.00432	0.0336002435665247	0.128560567134661	1233	304.33	928.667	4	10	0.004	0.033	2.58463412
TaMAPKKK51	TaRaf11	Ta--TraesCS7D02G230200.1	Td--TRIDC7BG020300.3	0	0.0708160668935029	0	1143	281.17	861.833	0	19	0	0.068	5.44738976
TaMAPKKK53	TaRaf13	Ta--TraesCS1B02G372400.1	Td--TRIDC1AG052430.1	0.03779	0.14906312327034	0.253545912196605	2403	594.25	1808.75	66.67	80.33	0.037	0.135	11.4663941
TaMAPKKK53	TaRaf13	Ta--TraesCS1B02G372400.1	Td--TRIDC1BG059980.1	0.00804	0.0178024747012926	0.451873127713191	2409	596.83	1812.17	14.5	10.5	0.008	0.018	1.36942113
TaMAPKKK53	TaRaf13	Ta--TraesCS1B02G372400.1	Td--TRIDC3AG036880.3	0.22015	0.583900094619452	0.377032129826563	1986	489.08	1496.92	285.6	198.4	0.191	0.406	44.9153919
TaMAPKKK53	TaRaf13	Ta--TraesCS1B02G372400.1	Td--TRIDC3BG040940.1	0.35817	2.53907974502931	0.141063724130798	2340	556.17	1783.83	508	403	0.285	0.725	195.313827
TaMAPKKK54	TaRaf14	Ta--TraesCS7D02G503600.1	Td--TRIDC7BG067700.1	0.1817	0.646276906192314	0.281143112833595	585	134.67	450.333	72.67	58.33	0.161	0.433	49.7136082

TaMAPKKK58	TaRaf18	Ta--TraesCS3B02G259800.1	Td--TRIDC1AG061210.1	0.06104	0.79841495180714 1	0.076451825058 3799	894	211.67	682.333	40	104	0.059	0.491	61.4165348
TaMAPKKK58	TaRaf18	Ta--TraesCS3B02G259800.1	Td--TRIDC1BG070170.4	0.06253	0.72169844838785 1	0.086640679127 3554	918	217.92	700.083	42	101	0.06	0.463	55.5152653
TaMAPKKK58	TaRaf18	Ta--TraesCS3B02G259800.1	Td--TRIDC3AG034530.2	0	0.08071021096555 15	0	945	222.17	722.833	0	17	0	0.077	6.20847777
TaMAPKKK58	TaRaf18	Ta--TraesCS3B02G259800.1	Td--TRIDC3BG038930.3	0	0	NaN	942	221	721	0	0	0	0	0
TaMAPKKK60	TaRaf20	Ta--TraesCS2A02G217000.1	Td--TRIDC2AG028890.5	0.4403	2.600605 37632376	0.169306611165 478	1944	463.92	1480.08	492.9	337.1	0.333	0.727	200.046567
TaMAPKKK60	TaRaf20	Ta--TraesCS2A02G217000.1	Td--TRIDC2BG032550.5	0.45131	2.989994 31599426	0.150939077570 784	1947	465.42	1481.58	502.4	342.6	0.339	0.736	229.999563
TaMAPKKK61	TaRaf21	Ta--TraesCS7D02G384700.1	Td--TRIDC6AG024780.3	0.31839	0.91348602561009 3	0.348546700934 176	846	210.33	635.667	164.9	111.1	0.259	0.528	70.2681558
TaMAPKKK61	TaRaf21	Ta--TraesCS7D02G384700.1	Td--TRIDC6BG030200.2	0.20273	0.58316855242967 4	0.347632136087 723	1098	267.67	830.333	147.5	108.5	0.178	0.405	44.8591194

TaMAPKKK61	TaRaf21	Ta--TraesCS7D02G384700.1	Td--TRIDC7AG054160.3	0.00111	0.03210954098038 91	0.034565368488 2206	1188	286.33	901.667	1	9	0.001	0.031	2.46996469
TaMAPKKK61	TaRaf21	Ta--TraesCS7D02G384700.1	Td--TRIDC7BG046810.3	0.00445	0.04302254615994 01	0.103496971777 634	1188	287	901	4	12	0.004	0.042	3.30942663
TaMAPKKK62	TaRaf22	Ta--TraesCS4A02G313900.1	Td--TRIDC4AG047400.2	0.00122	0.00256465306544 853	0.473806433284 811	3252	781.17	2470.83	3	2	0.001	0.003	0.19728101
TaMAPKKK62	TaRaf22	Ta--TraesCS4A02G313900.1	Td--TRIDC5BG083500.1	0.14962	0.40191448113130 8	0.372262343345 239	2946	714.58	2231.42	302.7	222.3	0.136	0.311	30.9164985
TaMAPKKK64-1	TaRaf24-1	Ta--TraesCS4A02G464700.1	Td--TRIDC7AG002140.2	0.11648	0.51568210163286 7	0.225875712229 118	504	119.33	384.667	41.5	44.5	0.108	0.373	39.667854
TaMAPKKK69	TaRaf29	Ta--TraesCS2D02G588200.1	Td--TRIDC2AG080090.1	0.09901	0.22218817375519 9	0.445607455410 613	1635	385.25	1249.75	115.9	74.08	0.093	0.192	17.091398
TaMAPKKK69	TaRaf29	Ta--TraesCS2D02G588200.1	Td--TRIDC2BG088230.2	0.01956	0.10525571421331 3	0.185793037493 819	1479	347.92	1131.08	21.83	34.17	0.019	0.098	8.0965934
TaMAPKKK70	TaRaf30	Ta--TraesCS1D02G423800.1	Td--TRIDC1AG061210.1	0.00438	0.16706520881242 5	0.026240298958 9348	900	213.67	686.333	3	32	0.004	0.15	12.8511699

TaMAPKKK70	TaRaf30	Ta--TraesCS1D02G423800.1	Td--TRIDC1BG070170.4	0.00858	0.12812717807393 3	0.066970301078 2587	924	220.75	703.25	6	26	0.009	0.118	9.85593677
TaMAPKKK70	TaRaf30	Ta--TraesCS1D02G423800.1	Td--TRIDC3AG034530.2	0.06587	0.70906563114623 2	0.092901181690 7382	945	223.5	721.5	45.5	102.5	0.063	0.459	54.5435101
TaMAPKKK70	TaRaf30	Ta--TraesCS1D02G423800.1	Td--TRIDC3BG038930.3	0.06603	0.72797166817751	0.090707471809 3406	942	222.17	719.833	45.5	103.5	0.063	0.466	55.9978206
TaMAPKKK71	TaRaf31	Ta--TraesCS6A02G004500.1	Td--TRIDC6AG000550.4	0.00799	0.01080038300719 21	0.739939016430 358	2337	512.92	1824.08	14.5	5.5	0.008	0.011	0.83079869
TaMAPKKK72	TaRaf32	Ta--TraesCS3A02G003900.1	Td--TRIDC3BG001050.1	0.0296	0.18253636016640 1	0.162177982005 81	1554	382.67	1171.33	34	62	0.029	0.162	14.0412585
TaMAPKKK73	TaRaf33	Ta--TraesCS3A02G039200.1	Td--TRIDC3AG004290.4	0.0425	0.19146086381136 3	0.221985064171 511	2034	493.17	1540.83	63.67	83.33	0.041	0.169	14.7277588
TaMAPKKK73	TaRaf33	Ta--TraesCS3A02G039200.1	Td--TRIDC3BG006020.1	0.0403	0.19478468635940 4	0.206875464695 253	2019	489.67	1529.33	60	84	0.039	0.172	14.9834374
TaMAPKKK75	TaRaf35	Ta--TraesCS3A02G096500.1	Td--TRIDC3AG011590.6	0.00138	0.00205198486415 606	0.672802347368 806	1938	488	1450	2	1	0.001	0.002	0.15784499

TaMAPKKK75	TaRaf35	Ta--TraesCS3A02G096500.1	Td--TRIDC3BG015640.4	0.0111	0.1028745229051	0.107902258986652	1941	488.92	1452.08	16	47	0.011	0.096	7.91342484
TaMAPKKK76	TaRaf36	Ta--TraesCS3A02G246100.1	Td--TRIDC1AG052430.1	0.11719	0.358375135872824	0.326997444928042	2388	589.08	1798.92	195.2	167.8	0.108	0.285	27.5673181
TaMAPKKK76	TaRaf36	Ta--TraesCS3A02G246100.1	Td--TRIDC1BG059980.1	0.12166	0.3357883548587755	0.362325434285263	2391	590.17	1800.83	202.3	159.8	0.112	0.271	25.8298735
TaMAPKKK76	TaRaf36	Ta--TraesCS3A02G246100.1	Td--TRIDC3AG036880.3	0.12118	0.1848314310864766	0.655614425450289	2037	502.08	1534.92	171.8	82.25	0.112	0.164	14.2178024
TaMAPKKK76	TaRaf36	Ta--TraesCS3A02G246100.1	Td--TRIDC3BG040940.1	0.33313	1.90622751011775	0.174757289806033	2355	560.58	1794.42	482.7	387.3	0.269	0.691	146.632885
TaMAPKKK81	TaRaf41	Ta--TraesCS5A02G292500.1	Td--TRIDC5BG047840.2	0.12347	0.3153806097774288	0.391485496445416	1884	421.42	1462.58	166.5	108.5	0.114	0.257	24.2600469
TaMAPKKK86	TaRaf46	Ta--TraesCS5D02G386800.1	Td--TRIDC5AG055160.3	0.00117	0.1059745441914014	0.0110333005290468	1119	263.08	855.917	1	26	0.001	0.099	8.15188801
TaMAPKKK86	TaRaf46	Ta--TraesCS5D02G386800.1	Td--TRIDC5AG056200.2	0.35279	1.69527549905343	0.208100281006245	1056	250.58	805.417	226.7	168.3	0.281	0.672	130.405808

TaMAPKKK86	TaRaf46	Ta--TraesCS5D02G386800.1	Td--TRIDC5BG059170.3	0.00117	0.09703959165214 61	0.012057419237 4066	1119	263.67	855.333	1	24	0.001	0.091	7.46458397
TaMAPKKK92	TaRaf52	Ta--TraesCS5B02G337300.1	Td--TRIDC5AG049770.1	0.00525	0.08510623350483 43	0.061649329090 7322	1749	410.17	1338.83	7	33	0.005	0.08	6.54663335
TaMAPKKK92	TaRaf52	Ta--TraesCS5B02G337300.1	Td--TRIDC5BG053430.2	0	0.00250836354213 543	0	1719	399.33	1319.67	0	1	0	0.003	0.19295104
TaMAPKKK93	TaRaf53	Ta--TraesCS5D02G018800.1	Td--TRIDC5BG001980.1	0.04126	0.16050134351708 6	0.257066562002 931	939	204.17	734.833	29.5	29.5	0.04	0.144	12.3462572
TaMAPKKK95	TaRaf55	Ta--TraesCS5B02G204900.1	Td--TRIDC5AG033270.1	0.01936	0.07049902815488 56	0.274675491516 263	1317	349.25	967.75	18.5	23.5	0.019	0.067	5.42300217
TaMAPKKK95	TaRaf55	Ta--TraesCS5B02G204900.1	Td--TRIDC5BG035150.3	0	0	NaN	1317	348.33	968.667	0	0	0	0	0
TaMAPKKK96	TaRaf56	Ta--TraesCS5B02G292000.2	Td--TRIDC5BG047840.2	0.01238	0.06549613768639 25	0.189082635126 752	1896	430.5	1465.5	18	27	0.012	0.063	5.03816444
TaMAPKKK97	TaRaf57	Ta--TraesCS5B02G353800.1	Td--TRIDC5AG051290.3	0.14812	0.65233720638802 1	0.227054775682 187	672	154.92	517.083	69.5	67.5	0.134	0.436	50.1797851

TaMAPKKK98	TaRaf58	Ta--TraesCS5D02G482000.1	Td--TRIDC5AG067100.1	0.00892	0.05998913561944 89	0.148743367833738	1032	242.83	789.167	7	14	0.009	0.058	4.61454889
TaMAPKKK98	TaRaf58	Ta--TraesCS5D02G482000.1	Td--TRIDC5BG072290.4	0.05698	0.144037958994054	0.395616243807195	1014	241	773	42.42	31.58	0.055	0.131	11.079843
TaMAPKKK99	TaRaf59	Ta--TraesCS3A02G001500.1	Td--TRIDC3AG003160.1	0	0.00534285429588826	0	777	187.83	589.167	0	1	0	0.005	0.41098879
TaMAPKKK99	TaRaf59	Ta--TraesCS3A02G001500.1	Td--TRIDC3BG000190.2	0.01651	0.150192333936826	0.109910384499504	1095	268.17	826.833	13.5	36.5	0.016	0.136	11.5532565
TaMAPKKK100	TaRaf60	Ta--TraesCS3A02G274000.1	Td--TRIDC1AG050190.1	0.33457	1.28815173176702	0.25972541647255	1734	411	1323	357.1	252.9	0.27	0.615	99.0885948
TaMAPKKK100	TaRaf60	Ta--TraesCS3A02G274000.1	Td--TRIDC1BG057000.1	0.3343	1.27396442395981	0.262405958611797	1734	408.92	1325.08	357.4	250.6	0.27	0.613	97.9972634
TaMAPKKK100	TaRaf60	Ta--TraesCS3A02G274000.1	Td--TRIDC3AG040710.1	0.00145	0		1803	418.5	1384.5	2	0	0.001	0	0
TaMAPKKK100	TaRaf60	Ta--TraesCS3A02G274000.1	Td--TRIDC3BG045870.3	0.02052	0.0650950450031555	0.315294729559683	1800	417	1383	28	26	0.02	0.062	5.00731115

TaMAPKKK101	TaRaf61	Ta--TraesCS5D02G019400.1	Td--TRIDC5BG002040.2	0.02745	0.1140006839180773	0.240810397495702	3039	813.17	2225.83	60	86	0.027	0.106	8.76928338
TaMAPKKK102	TaRaf62	Ta--TraesCS7D02G474700.1	Td--TRIDC6AG021710.1	0.08935	0.762826430901621	0.117134827726129	1254	292.42	961.583	81	140	0.084	0.479	58.6789562
TaMAPKKK102	TaRaf62	Ta--TraesCS7D02G474700.1	Td--TRIDC6BG027410.1	0.08758	0.78764160407195	0.111195495151347	1254	292.25	961.75	79.5	142.5	0.083	0.488	60.5878157
TaMAPKKK102	TaRaf62	Ta--TraesCS7D02G474700.1	Td--TRIDC7AG068050.1	0.00205	0.167932556374681	0.0122201457953167	1275	299.08	975.917	2	45	0.002	0.15	12.917889
TaMAPKKK102	TaRaf62	Ta--TraesCS7D02G474700.1	Td--TRIDC7BG061870.1	0.00308	0.0884676775076339	0.0348368165719975	1275	299.58	975.417	3	25	0.003	0.083	6.80520596
TaMAPKKK103	TaRaf63	Ta--TraesCS7A02G326700.1	Td--TRIDC7AG045740.2	0.00081	0.00266016683228101	0.305436773783536	1608	376.58	1231.42	1	1	0.001	0.003	0.20462822
TaMAPKKK103	TaRaf63	Ta--TraesCS7A02G326700.1	Td--TRIDC7BG037680.2	0.01874	0.0455223847945452	0.411643577964265	1551	362.25	1188.75	22	16	0.019	0.044	3.50172191
TaMAPKKK104	TaRaf64	Ta--TraesCS1D02G273600.1	Td--TRIDC1AG040730.2	0.0795	0.148992338401155	0.533588031702845	2019	514.33	1504.67	113.5	69.5	0.075	0.135	11.4609491

TaMAPKKK104	TaRaf64	Ta--TraesCS1D02G273600.1	Td--TRIDC1BG046350.4	0.06725	0.1576746090867 85	0.426532760271 581	2010	509.83	1500.17	96.5	72.5	0.064	0.142	12.1288161
TaMAPKKK104-1	TaRaf64-1	Ta--TraesCS1B02G283400.1	Td--TRIDC1AG040730.2	0.07779	0.162769016101706	0.477904584580205	2025	516	1509	111.5	75.5	0.074	0.146	12.5206935
TaMAPKKK104-1	TaRaf64-1	Ta--TraesCS1B02G283400.1	Td--TRIDC1BG046350.4	0	0.00195694827271029	0	2016	511.67	1504.33	0	1	0	0.002	0.15053448
TaMAPKKK105	TaRaf65	Ta--TraesCS3A02G045200.1	Td--TRIDC3AG005110.2	0.00112	0.00663721145773687	0.168592739268112	1197	302.67	894.333	1	2	0.001	0.007	0.51055473
TaMAPKKK105	TaRaf65	Ta--TraesCS3A02G045200.1	Td--TRIDC3BG005340.1	0.01481	0.127385536926584	0.116291770564942	1185	298.75	886.25	13	35	0.015	0.117	9.79888746
TaMAPKKK106	TaRaf66	Ta--TraesCS4A02G456900.1	Td--TRIDC4AG067540.2	0.3227	0.984266095198941	0.32785369434115	1899	447.75	1451.25	380.6	245.4	0.262	0.548	75.7127766
TaMAPKKK106	TaRaf66	Ta--TraesCS4A02G456900.1	Td--TRIDC7AG003050.2	0.02652	0.105767110789391	0.250770718092588	1851	437.58	1413.42	36.83	43.17	0.026	0.099	8.1359316
TaMAPKKK107	TaRaf67	Ta--TraesCS7A02G032700.1	Td--TRIDC4AG067560.1	0.1037	0.490098949476573	0.21158453168996	1428	333.5	1094.5	106	120	0.097	0.36	37.6999192

TaMAPKKK107	TaRaf67	Ta--TraesCS7A02G032700.1	Td--TRIDC7AG003050.2	0.01065	0.00963868687768 53	1.105419471748 2	1353	313.25	1039.75	11	3	0.011	0.01	0.74143745
TaMAPKKK108	TaRaf68	Ta--TraesCS3D02G023600.1	Td--TRIDC3BG003740.4	0.03168	0.11547366941486 4	0.274380277132 693	1266	299	967	30	32	0.031	0.107	8.88258995
TaMAPKKK109	TaRaf69	Ta--TraesCS2D02G219800.1	Td--TRIDC2AG028370.1	0.02252	0.09817575500704 07	0.229430005811 456	2043	510.75	1532.25	34	47	0.022	0.092	7.55198115
TaMAPKKK110	TaRaf70	Ta--TraesCS4B02G289100.1	Td--TRIDC4AG002010.1	0.15163	0.27100759047618 7	0.559498637007 512	1080	285.42	794.583	109.1	64.92	0.137	0.227	20.8467377
TaMAPKKK110	TaRaf70	Ta--TraesCS4B02G289100.1	Td--TRIDC4BG049660.1	0.00067	0.00374065613327 928	0.177826694026 476	2040	536	1504	1	2	0.001	0.004	0.28774278
TaMAPKKK111	TaRaf71	Ta--TraesCS3A02G315100.1	Td--TRIDC3AG046380.3	0.00268	0		2430	558.58	1871.42	5	0	0.003	0	0
TaMAPKKK111	TaRaf71	Ta--TraesCS3A02G315100.1	Td--TRIDC3BG052620.2	0.04005	0.04887630003322 92	0.819436304664 022	1320	302.92	1017.08	39.67	14.33	0.039	0.047	3.75971539
TaMAPKKK111	TaRaf71	Ta--TraesCS3A02G315100.1	Td--TRIDC3BG052880.9	0.50524	NaN	NaN	2292	544.5	1747.5	642.4	464.6	0.368	0.853	High Sequence Divergence Value (pS>=0.75)

TaMAPKKK115	TaRaf75	Ta--TraesCS3A02G229800.1	Td--TRIDC3AG034390.3	0	0.0121435561228019	0	1587	415.08	1171.92	0	5	0	0.012	0.9341197
TaMAPKKK115	TaRaf75	Ta--TraesCS3A02G229800.1	Td--TRIDC3BG038810.4	0.03875	0.103856701377086	0.37307533631623	1596	417.58	1178.42	44.5	40.5	0.038	0.097	7.98897703
TaMAPKKK116	TaRaf76	Ta--TraesCS1B02G454000.2	Td--TRIDC1BG071250.6	0.20881	0.702612082577192	0.297191749689681	1848	449.83	1398.17	254.8	205.2	0.182	0.456	54.0470833
TaMAPKKK117	TaRaf77	Ta--TraesCS3D02G097000.1	Td--TRIDC3AG011590.6	0.01815	0.0619238254351119	0.293066611084727	1938	487.92	1450.08	26	29	0.018	0.059	4.76337119
TaMAPKKK117	TaRaf77	Ta--TraesCS3D02G097000.1	Td--TRIDC3BG015640.4	0.0111	0.0981077268826218	0.113177662699222	1941	489.33	1451.67	16	45	0.011	0.092	7.54674822
TaMAPKKK119	TaRaf79	Ta--TraesCS2A02G577000.1	Td--TRIDC2BG088230.2	0.01001	0.100726559810219	0.0993737675828018	1512	355.42	1156.58	11.5	33.5	0.01	0.094	7.74819691
TaMAPKKK121	TaRaf81	Ta--TraesCS2D02G066900.1	Td--TRIDC2AG007500.5	0.33726	1.32954169740596	0.253664636552142	2886	715.42	2170.58	589.6	445.4	0.272	0.623	102.272438
TaMAPKKK122	TaRaf82	Ta--TraesCS2A02G214000.1	Td--TRIDC2AG028370.1	0.00131	0.00195758675574277	0.667683048829233	2043	511.5	1531.5	2	1	0.001	0.002	0.1505836

TaMAPKKK123-1	TaRaf83-1	Ta--TraesCS5D02G358200.1	Td--TRIDC5AG051310.6	0.18928	0.739125229079247	0.256088825761523	1371	315.92	1055.08	176.5	148.5	0.167	0.47	56.8557869
TaMAPKKK125	TaRaf85	Ta--TraesCS7D02G153800.1	Td--TRIDC7AG018660.25	0.04654	0.165799485838111	0.280682683058412	1353	337.25	1015.75	45.83	50.17	0.045	0.149	12.7538066
TaMAPKKK128	TaRaf88	Ta--TraesCS1B02G446500.1	Td--TRIDC1AG061210.1	0.00292	0.160959796072858	0.0181482163088325	900	214	686	2	31	0.003	0.145	12.3815228
TaMAPKKK128	TaRaf88	Ta--TraesCS1B02G446500.1	Td--TRIDC1BG070170.4	0.00285	0.0811278807381575	0.0351383305338462	924	221.08	702.917	2	17	0.003	0.077	6.24060621
TaMAPKKK128	TaRaf88	Ta--TraesCS1B02G446500.1	Td--TRIDC3AG034530.2	0.06439	0.718870721894625	0.089574355804219	945	223.83	721.167	44.5	103.5	0.062	0.462	55.2977478
TaMAPKKK128	TaRaf88	Ta--TraesCS1B02G446500.1	Td--TRIDC3BG038930.3	0.06455	0.714389752627958	0.0903542474016787	942	222.5	719.5	44.5	102.5	0.062	0.461	54.9530579
TaMAPKKK129	TaRaf89	Ta--TraesCS6B02G320800.1	Td--TRIDC6BG052000.1	0.10106	0.12669376669724	0.797651372832766	1269	318.83	950.167	89.83	37.17	0.095	0.117	9.74567436
TaMAPKKK131	TaRaf91	Ta--TraesCS6B02G215100.2	Td--TRIDC6AG027120.2	0.01183	0.0271742485110101	0.435227614815954	2229	524.58	1704.42	20	14	0.012	0.027	2.09032681

TaMAPKKK131	TaRaf91	Ta--TraesCS6B02G215100.2	Td--TRIDC6BG033330.6	0.00073	0.00681048947680986	0.106567559573051	1821	442.5	1378.5	1	3	0.001	0.007	0.52388381
TaMAPKKK133	TaRaf93	Ta--TraesCS1D02G004300.1	Td--TRIDC1BG000450.3	0.01755	0.239527146517152	0.0732878829635221	1620	409.67	1210.33	21	84	0.017	0.205	18.4251651
TaMAPKKK134	TaRaf94	Ta--TraesCS5D02G547500.1	Td--TRIDC4AG047400.2	0.31104	1.47185660992628	0.211326242963726	2178	514	1664	423.7	331.3	0.255	0.645	113.219739
TaMAPKKK137	TaRaf97	Ta--TraesCS3D02G472000.1	Td--TRIDC3AG067170.2	0.04134	0.143042227087951	0.289015156943727	870	211.17	658.833	26.5	27.5	0.04	0.13	11.0032482
TaMAPKKK140	TaRaf100	Ta--TraesCS5D02G097900.1	Td--TRIDC5AG013160.4	0.00581	0.0405178085329921	0.1434989826801 1	2259	532.42	1726.58	10	21	0.006	0.039	3.1167545
TaMAPKKK140	TaRaf100	Ta--TraesCS5D02G097900.1	Td--TRIDC5BG015110.1	0.00669	0.0729987511184199	0.0916522435833342	1947	445.67	1501.33	10	31	0.007	0.07	5.61528855
TaMAPKKK146	TaRaf106	Ta--TraesCS2B02G241400.1	Td--TRIDC2AG028730.4	0.03695	0.0992190295197042	0.372454155456731	2001	489.58	1511.42	54.5	45.5	0.036	0.093	7.63223304
TaMAPKKK146	TaRaf106	Ta--TraesCS2B02G241400.1	Td--TRIDC2BG032450.3	0.00199	0		2001	490.83	1510.17	3	0	0.002	0	0

TaMAPKKK148	TaRaf108	Ta--TraesCS1A02G003900.1	Td--TRIDC1BG000340.1	0.11217	0.39633677302590 5	0.283008185313 808	1023	227.92	795.083	82.83	70.17	0.104	0.308	30.4874441
TaMAPKKK150	TaRaf110	Ta--TraesCS3B02G008600.2	Td--TRIDC3AG002440.3	0.02705	0.15867555612490 8	0.170472108279 048	2037	531.42	1505.58	40	76	0.027	0.143	12.205812
TaMAPKKK150	TaRaf110	Ta--TraesCS3B02G008600.2	Td--TRIDC3BG001050.1	0	0	NaN	1560	385	1175	0	0	0	0	0
TaMAPKKK151	TaRaf111	Ta--TraesCS3B02G123800.1	Td--TRIDC3AG012960.1	0.02228	0.08542187463344 56	0.260845510697 612	1731	425.25	1305.75	28.67	34.33	0.022	0.081	6.57091343
TaMAPKKK151	TaRaf111	Ta--TraesCS3B02G123800.1	Td--TRIDC3BG017420.1	0.00153	0		1731	425.67	1305.33	2	0	0.002	0	0
TaMAPKKK152	TaRaf112	Ta--TraesCS3B02G259100.1	Td--TRIDC3AG034390.3	0.04165	0.10468576338775 9	0.397828011104 016	1587	414.5	1172.5	47.5	40.5	0.041	0.098	8.05275103
TaMAPKKK152	TaRaf112	Ta--TraesCS3B02G259100.1	Td--TRIDC3BG038810.4	0.00212	0.00120048044838 44	1.768578298172 42	1596	416.83	1179.17	2.5	0.5	0.002	0.001	0.09234465
TaMAPKKK153	TaRaf113	Ta--TraesCS3B02G351800.1	Td--TRIDC3AG046380.3	0.01605	0.11108039055768	0.144448022233 575	906	213.08	692.917	11	22	0.016	0.103	8.54464543

TaMAPKKK154	TaRaf114	Ta--TraesCS3B02G478400.1	Td--TRIDC3BG070490.1	0.00323	0		792	172.33	619.667	2	0	0.003	0	0
TaMAPKKK155	TaRaf115	Ta--TraesCS6B02G217100.1	Td--TRIDC7AG049870.2	0.26127	2.59651603904645	0.100624156919516	867	209	658	145.2	151.8	0.221	0.726	199.732003
TaMAPKKK155	TaRaf115	Ta--TraesCS6B02G217100.1	Td--TRIDC7BG043940.1	0.2597	3.21278750775947	0.0808341227119502	879	213.5	665.5	146.1	157.9	0.22	0.74	247.137501
TaMAPKKKK1		Ta--TraesCS1A02G181900.1	Td--TRIDC1AG027610.11	0	0.00211491151775357	0	2067	473.5	1593.5	0	1	0	0.002	0.1626855
TaMAPKKKK1		Ta--TraesCS1A02G181900.1	Td--TRIDC1BG033320.4	0.00475	0.0496439547286897	0.0955855024765835	1917	437.17	1479.83	7	21	0.005	0.048	3.81876575
TaMAPKKKK2		Ta--TraesCS1B02G199100.2	Td--TRIDC1AG027610.11	0.00377	0.0457658297442697	0.0824543745325147	2067	473	1594	6	21	0.004	0.044	3.52044844
TaMAPKKKK2		Ta--TraesCS1B02G199100.2	Td--TRIDC1BG033320.4	0.00068	0		1917	436.67	1480.33	1	0	0.001	0	0
TaMAPKKKK3		Ta--TraesCS1D02G185000.2	Td--TRIDC1AG027610.11	0.00188	0.0346069679327911	0.0544549800224439	2067	473.08	1593.92	3	16	0.002	0.034	2.66207446

TaMAPKKKK3	Ta--TraesCS1D02G185000.2	Td--TRIDC1BG033320.4	0.00406	0.0399702803998843	0.101684605405365	1917	436.75	1480.25	6	17	0.004	0.039	3.07463695
TaMAPKKKK4	Ta--TraesCS2A02G233400.1	Td--TRIDC2AG030300.1	0.00063	0.00201748608540676	0.313911587860861	2076	496.33	1579.67	1	1	0.001	0.002	0.15519124
TaMAPKKKK4	Ta--TraesCS2A02G233400.1	Td--TRIDC2BG033980.3	0.00444	0.0478498463029498	0.0928735937118374	2076	496.17	1579.83	7	23	0.004	0.046	3.68075741
TaMAPKKKK5	Ta--TraesCS2B02G249900.1	Td--TRIDC2AG030300.1	0.00444	0.0435958062133858	0.101914549522575	2076	495.83	1580.17	7	21	0.004	0.042	3.35352355
TaMAPKKKK5	Ta--TraesCS2B02G249900.1	Td--TRIDC2BG033980.3	0.00063	0.00202020324166721	0.313357077504967	2076	495.67	1580.33	1	1	0.001	0.002	0.15540025
TaMAPKKKK6	Ta--TraesCS2D02G232200.1	Td--TRIDC2AG030300.1	0.00476	0.0256566833991391	0.185552483236487	2076	495.58	1580.42	7.5	12.5	0.005	0.025	1.97359103
TaMAPKKKK6	Ta--TraesCS2D02G232200.1	Td--TRIDC2BG033980.3	0.00572	0.0565811929013991	0.101019883145075	2076	495.42	1580.58	9	27	0.006	0.054	4.35239945
TaMAPKKKK7	Ta--TraesCS4B02G395600.1	Td--TRIDC1AG027610.11	0.2646	1.03634927928802	0.255316613349468	1719	400.75	1318.25	293.9	225.1	0.223	0.562	79.7191753

TaMAPKKKK8	Ta--TraesCS4B02G398400.3	Td--TRIDC1AG027610.11	0.18079	0.99534553783817 9	0.181639346371077	2028	466.67	1561.33	250.8	257.2	0.161	0.551	76.5650414
TaMAPKKKK8	Ta--TraesCS4B02G398400.3	Td--TRIDC1BG033320.4	0.18405	1.08413721642466	0.169762417820955	1878	430.42	1447.58	236.3	246.8	0.163	0.573	83.3951705
TaMAPKKKK8	Ta--TraesCS4B02G398400.3	Td--TRIDC5AG078780.1	0.02808	0.0983929473184089	0.285369651533873	2061	482.58	1578.42	43.5	44.5	0.028	0.092	7.56868826
TaMAPKKKK9	Ta--TraesCS5A02G187400.1	Td--TRIDC5AG032000.2	0.0037	0.00970744541864889	0.38122531414588	1554	380.17	1173.83	4.333	3.667	0.004	0.01	0.74672657
TaMAPKKKK9	Ta--TraesCS5A02G187400.1	Td--TRIDC5BG033660.2	0.01728	0.0944599268834398	0.182915004632196	1554	383.08	1170.92	20	34	0.017	0.089	7.26614822
TaMAPKKKK11	Ta--TraesCS5A02G556400.5	Td--TRIDC1AG027610.11	0.17268	0.924049900272294	0.186867878381902	2013	462.92	1550.08	239.1	245.9	0.154	0.531	71.0807616
TaMAPKKKK11	Ta--TraesCS5A02G556400.5	Td--TRIDC5AG078780.1	0.01354	0.0256755327798694	0.527492781220761	2040	475.42	1564.58	21	12	0.013	0.025	1.97504098
TaMAPKKKK12	Ta--TraesCS5B02G397300.1	Td--TRIDC5AG057130.2	0.00886	0.060364830835347	0.146707831087657	2469	594.83	1874.17	16.5	34.5	0.009	0.058	4.64344853

TaMAPKKKK12	Ta--TraesCS5B02G397300.1	Td--TRIDC5BG061270.2	0.00113	0.00901724117215036	0.125818684235447	2322	557.83	1764.17	2	5	0.001	0.009	0.69363394
TaMAPKKKK13	Ta--TraesCS5D02G203600.1	Td--TRIDC5AG032000.2	0.01927	0.0913036616244838	0.211039705262639	1554	380	1174	22.33	32.67	0.019	0.086	7.02335859
TaMAPKKKK13	Ta--TraesCS5D02G203600.1	Td--TRIDC5BG033660.2	0.01379	0.0740495906961091	0.186206824911701	1554	382.92	1171.08	16	27	0.014	0.071	5.69612236
TaMAPKKKK14	Ta--TraesCS5D02G402300.1	Td--TRIDC5AG057130.2	0.00889	0.0533674998893562	0.1665021141103	2460	592.08	1867.92	16.5	30.5	0.009	0.052	4.1051923
TaMAPKKKK14	Ta--TraesCS5D02G402300.1	Td--TRIDC5BG061270.2	0.0037	0.0377785985155642	0.0980233215300578	2316	556.42	1759.58	6.5	20.5	0.004	0.037	2.90604604
TaMAPKKKK16	Ta--TraesCS6A02G353400.1	Td--TRIDC6AG053160.4	0.01192	0.0292393982367445	0.407668280835926	894	203.42	690.583	8.167	5.833	0.012	0.029	2.24918448
TaMAPKKKK16	Ta--TraesCS6A02G353400.1	Td--TRIDC6BG061710.3	0.01028	0.0636394776412797	0.161597211809352	912	210.33	701.667	7.167	12.83	0.01	0.061	4.89534443
TaMAPKKKK18	Ta--TraesCS6B02G177800.1	Td--TRIDC6AG020750.1	0.01317	0.124349849267513	0.105914773423427	1626	362.17	1263.83	16.5	41.5	0.013	0.115	9.56537302

TaMAPKKKK18	Ta--TraesCS6B02G177800.1	Td--TRIDC6BG026600.3	0.00159	0.00275925811338641	0.574542138041081	1626	363.08	1262.92	2	1	0.002	0.003	0.21225062
TaMAPKKKK19	Ta--TraesCS6B02G386100.1	Td--TRIDC6AG053160.4	0.00216	0.03252542183634 74	0.066520061548 5585	1797	408.42	1388.58	3	13	0.002	0.032	2.50195553
TaMAPKKKK19	Ta--TraesCS6B02G386100.1	Td--TRIDC6BG061710.3	0.00066	0.00450451804515894	0.147312108258894	1953	445.33	1507.67	1	2	0.001	0.004	0.34650139
TaMAPKKKK20	Ta--TraesCS6D02G335800.1	Td--TRIDC6AG053160.4	0.00289	0.0376963760856662	0.076540846274 0315	1797	408	1389	4	15	0.003	0.037	2.89972124
TaMAPKKKK20	Ta--TraesCS6D02G335800.1	Td--TRIDC6BG061710.3	0.00399	0.0392170822682073	0.101719705964875	1953	444.92	1508.08	6	17	0.004	0.038	3.01669864
TaMAPKKKK22	Ta--TraesCS7A02G232300.1	Td--TRIDC7AG029890.1	0.00062	0		2100	482.33	1617.67	1	0	0.001	0	0
TaMAPKKKK22	Ta--TraesCS7A02G232300.1	Td--TRIDC7BG020630.1	0.00683	0.0604208668478506	0.113055827432374	2100	482.33	1617.67	11	28	0.007	0.058	4.64775899
TaMAPKKKK23	Ta--TraesCS7B02G130700.1	Td--TRIDC7AG029890.1	0.00621	0.0581561403468654	0.106747151690557	2100	482.5	1617.5	10	27	0.006	0.056	4.47354926

TaMAPKKKK23	Ta--TraesCS7B02G130700.1	Td--TRIDC7BG020630.1	0.00124	0.00207540775812 04	0.596266641452943	2100	482.5	1617.5	2	1	0.001	0.002	0.15964675
TaMAPKKKK24	Ta--TraesCS7D02G232400.1	Td--TRIDC7AG029890.1	0.01631	0.0461628678233344	0.353392829344622	2067	476.5	1590.5	25.67	21.33	0.016	0.045	3.55098983
TaMAPKKKK24	Ta--TraesCS7D02G232400.1	Td--TRIDC7BG020630.1	0.0176	0.0874524368939637	0.201250496811154	2067	476.5	1590.5	27.67	39.33	0.017	0.083	6.72711053
TaMAPKKKK25	Ta--TraesCSU02G115300.1	Td--TRIDC5AG078780.1	0.02771	0.0743642698449401	0.372596261921256	2061	480.25	1580.75	43	34	0.027	0.071	5.72032845
													Average=27.6055561113674

The Ka/Ks ratio and divergence times between the genes of *T.dicoccoides* and *T.turgidum*

Gene ID	Gene ID	Ka	Ks	Ka/Ks	EffectiveLen	AverageS-sites	AverageN-sites	cN	cS	pN	pS	Divergence times (Mya)
Td--TRIDC6AG041340.3	Tt--TRITD6Av1G170870.1	0.1065743	0.208721734135453	0.510604793066788	1131	261.16667	869.83333	86.42	47.58	0.099	0.182	16.055518
Td--TRIDC6AG041340.3	Tt--TRITD6Bv1G157460.4	0.0074245	0.0501679511239305	0.147992700003166	1236	288.5	947.5	7	14	0.007	0.049	3.85907316
Td--TRIDC6BG048300.1	Tt--TRITD6Av1G170870.1	0.1057604	0.2417510902655	0.437476287221586	1149	265.33333	883.66667	87.17	54.83	0.099	0.207	18.5962377
Td--TRIDC6BG048300.1	Tt--TRITD6Bv1G157460.4	0.0010409	0		1254	292.66667	961.33333	1	0	0.001	0	0
Td--TRIDC5BG079810.1	Tt--TRITD4Av1G214900.3	0.014998	0.0703247651031883	0.213267689904842	1323	312.83333	1010.1667	15	21	0.015	0.067	5.40959732
Td--TRIDC5BG079810.1	Tt--TRITD5Bv1G245660.3	0.0019773	0.00635260547548787	0.311252232754272	1329	316.16667	1012.8333	2	2	0.002	0.006	0.48866196
Td--TRIDC4AG014520.1	Tt--TRITD4Av1G049530.4	0.0236666	0.0284498004157667	0.831872335058616	984	232.83333	751.16667	17.5	6.5	0.023	0.028	2.18844619
Td--TRIDC4AG014520.1	Tt--TRITD4Bv1G124550.2	0	0.0870541284395653	0	1098	255.5	842.5	0	21	0	0.082	6.69647142
Td--TRIDC4BG035610.1	Tt--TRITD4Av1G049530.4	0.0250362	0.103533165597153	0.241817720611919	984	232.66667	751.33333	18.5	22.5	0.025	0.097	7.96408966
Td--TRIDC4BG035610.1	Tt--TRITD4Bv1G124550.2	0	0	NaN	1107	258	849	0	0	0	0	0

Td--TRIDC1AG012310.2	Tt--TRITD1Av1G032550.8	0.0007937	0.00259291528789334	0.306084379777707	1647	386.33333	1260.6667	1	1	0.001	0.003	0.19945502
Td--TRIDC1AG012310.2	Tt--TRITD1Bv1G041580.2	0.0043428	0.0962278867899456	0.0451304008214176	1509	354.33333	1154.6667	5	32	0.004	0.09	7.40214514
Td--TRIDC1BG015140.6	Tt--TRITD1Av1G032550.8	0.004779	0.096241687158416	0.0496561192112174	1647	387.5	1259.5	6	35	0.005	0.09	7.4032067
Td--TRIDC1BG015140.6	Tt--TRITD1Bv1G041580.2	0.0092335	0.0171118876470109	0.539596130336913	1545	364.5	1180.5	10.83	6.167	0.009	0.017	1.31629905
Td--TRIDC1AG061100.1	Tt--TRITD1Av1G220750.2	0.0539344	0.145598490894623	0.370432170769167	1686	401.75	1284.25	66.83	53.17	0.052	0.132	11.1998839
Td--TRIDC1AG061100.1	Tt--TRITD1Bv1G218290.1	0.0376254	0.111714311714502	0.336800571574661	1602	375.75	1226.25	45	39	0.037	0.104	8.59340859
Td--TRIDC1AG061100.1	Tt--TRITD3Av1G155110.1	0.1354189	1.23525197428024	0.109628540014771	1443	328.5	1114.5	138.1	198.9	0.124	0.606	95.0193826
Td--TRIDC1AG061100.1	Tt--TRITD3Bv1G135620.1	0.1265958	1.27125495123956	0.0995833668165323	1443	330.58333	1112.4167	129.6	202.4	0.116	0.612	97.7888424
Td--TRIDC1BG070050.1	Tt--TRITD1Av1G220750.2	0.0606144	0.159326652781303	0.380440996649074	1680	400.58333	1279.4167	74.5	57.5	0.058	0.144	12.2558964
Td--TRIDC1BG070050.1	Tt--TRITD1Bv1G218290.1	0.0624474	0.138259104546158	0.451669352754335	1689	403.91667	1285.0833	77	51	0.06	0.126	10.6353157
Td--TRIDC1BG070050.1	Tt--TRITD3Av1G155110.1	0.13728	1.26799751426752	0.108265165489214	1443	329	1114	139.8	201.3	0.125	0.612	97.5382703

Td--TRIDC1BG070050.1	Tt--TRITD3Av1G155110.1	0.13728	1.26799751426752	0.108265165489214	1443	329	1114	139.8	201.3	0.125	0.612	97.5382703
Td--TRIDC1BG070050.1	Tt--TRITD3Bv1G135620.1	0.1284346	1.28834650155023	0.0996895185728013	1443	331.08333	1111.9167	131.3	203.8	0.118	0.615	99.103577
Td--TRIDC3AG034650.7	Tt--TRITD1Av1G220750.2	0.1452449	1.29301334360786	0.112330519737356	1473	337.66667	1135.3333	149.9	208.1	0.132	0.616	99.4625649
Td--TRIDC3AG034650.7	Tt--TRITD1Bv1G218290.1	0.134263	1.2333260658534	0.10886253079586	1464	336	1128	138.7	203.3	0.123	0.605	94.8712358
Td--TRIDC3AG034650.7	Tt--TRITD3Av1G155110.1	0.0008824	0		1470	336	1134	1	0	0.001	0	0
Td--TRIDC3AG034650.7	Tt--TRITD3Bv1G135620.1	0.0205998	0.074576263040085	0.276225252490908	1470	338.08333	1131.9167	23	24	0.02	0.071	5.73663562
Td--TRIDC3BG039070.12	Tt--TRITD1Av1G220750.2	0.1302836	1.35220365034652	0.0963491190144152	1482	344.83333	1137.1667	136	216	0.12	0.626	104.015665
Td--TRIDC3BG039070.12	Tt--TRITD1Bv1G218290.1	0.1303671	1.29446660129644	0.100711056079871	1482	345.5	1136.5	136	213	0.12	0.616	99.5743539
Td--TRIDC3BG039070.12	Tt--TRITD3Av1G155110.1	0.0390825	0.0939055089510034	0.416190029032335	1449	335.16667	1113.8333	42.42	29.58	0.038	0.088	7.22350069
Td--TRIDC3BG039070.12	Tt--TRITD3Bv1G135620.1	0.018593	0.02282975827682	0.814420121405706	1449	337.25	1111.75	20.42	7.583	0.018	0.022	1.75613525
Td--TRIDC7AG012990.2	Tt--TRITD7Av1G032080.4	0.1223838	0.19569741523526	0.625372624703907	1032	243.83333	788.16667	89	42	0.113	0.172	15.0536473

Td--TRIDC7AG012990.2	Tt--TRITD7Bv1G002620.4	0.1114694	0.288879048267642	0.385868852164262	1017	239.83333	777.16667	80.5	57.5	0.104	0.24	22.2214653
Td--TRIDC7BG001200.2	Tt--TRITD7Av1G032080.4	0.0012045	0.0905947344103324	0.0132949972833615	1077	246.08333	830.91667	1	21	0.001	0.085	6.96882572
Td--TRIDC7BG001200.2	Tt--TRITD7Bv1G002620.4	0	0	NaN	1002	227.5	774.5	0	0	0	0	0
Td--TRIDC7AG047060.4	Tt--TRITD7Av1G178880.7	0.001084	0.00370142639004825	0.292863051858581	1194	270.83333	923.16667	1	1	0.001	0.004	0.28472511
Td--TRIDC7AG047060.4	Tt--TRITD7Bv1G140030.6	0.002053	0.046637855797338	0.0440208502400651	1263	287.5	975.5	2	13	0.002	0.045	3.58752737
Td--TRIDC7BG040680.1	Tt--TRITD7Av1G178880.7	0.0010838	0.0417829552605042	0.0259391709072096	1194	270.66667	923.33333	1	11	0.001	0.041	3.21407348
Td--TRIDC7BG040680.1	Tt--TRITD7Bv1G140030.6	0	0.00698898476240196	0	1263	287.5	975.5	0	2	0	0.007	0.53761421
Td--TRIDC1AG061940.2	Tt--TRITD1Av1G222580.3	0	0	NaN	1794	433.16667	1360.8333	0	0	0	0	0
Td--TRIDC1AG061940.2	Tt--TRITD1Bv1G220000.2	0.0103686	0.130408872715655	0.0795085120382178	1794	434.41667	1359.5833	14	52	0.01	0.12	10.0314517
Td--TRIDC1AG061940.2	Tt--TRITD3Av1G151000.4	0.1574447	1.01236019368328	0.155522381456451	1767	420.91667	1346.0833	191.2	233.8	0.142	0.556	77.8738611
Td--TRIDC1AG061940.2	Tt--TRITD3Bv1G133770.2	0.1570731	1.01354713755119	0.154973678433744	1767	421.58333	1345.4167	190.7	234.3	0.142	0.556	77.9651644

Td--TRIDC1BG071030.1	Tt--TRITD1Av1G222580.3	0.0103712	0.127568052158476	0.0812991626219065	1794	434.75	1359.25	14	51	0.01	0.117	9.81292709
Td--TRIDC1BG071030.1	Tt--TRITD1Bv1G220000.2	0.0014634	0.00227100854823082	0.644389955989513	1809	441	1368	2	1	0.001	0.002	0.17469297
Td--TRIDC1BG071030.1	Tt--TRITD3Av1G151000.4	0.1547643	1.07938269349323	0.143382194654205	1770	424.41667	1345.5833	188.2	242.8	0.14	0.572	83.029438
Td--TRIDC1BG071030.1	Tt--TRITD3Bv1G133770.2	0.1562218	1.09056278359719	0.143248794480683	1770	425.08333	1344.9167	189.7	244.3	0.141	0.575	83.8894449
Td--TRIDC3AG036190.14	Tt--TRITD1Bv1G214990.2	0.1277327	1.20443932889738	0.106051564562706	1632	378.25	1253.75	147.3	226.7	0.117	0.599	92.6491791
Td--TRIDC3AG036190.14	Tt--TRITD3Av1G162410.11	0.0018861	0.00383796146691783	0.491427997516935	1719	391.83333	1327.1667	2.5	1.5	0.002	0.004	0.29522781
Td--TRIDC3AG036190.14	Tt--TRITD3Av1G151000.4	0.1966788	3.51600464367557	0.0559381427433389	1722	398.33333	1323.6667	229	296	0.173	0.743	270.461896
Td--TRIDC3AG036190.14	Tt--TRITD3Bv1G140820.6	0.0195556	0.0544862269973564	0.35890960291144	1752	396.41667	1355.5833	26.17	20.83	0.019	0.053	4.19124823
Td--TRIDC3AG036190.14	Tt--TRITD3Bv1G133770.2	0.193118	2.84012649172185	0.0679962580274387	1722	399.5	1322.5	225.2	292.8	0.17	0.733	218.471269
Td--TRIDC3AG034020.1	Tt--TRITD1Av1G222580.3	0.1503175	1.00567030638758	0.149469929592073	1767	419.83333	1347.1667	183.5	232.5	0.136	0.554	77.3592543
Td--TRIDC3AG034020.1	Tt--TRITD1Bv1G220000.2	0.1482243	1.07749635951193	0.137563641918064	1770	423	1347	181.2	241.8	0.134	0.572	82.8843353

Td--TRIDC3AG034020.1	Tt--TRITD3Av1G151000.4	0.0108752	0.0065532058424920 2	1.6595187 7570267	1839	434.25	1404.75	15.17	2.833	0.011	0.007	0.50409276
Td--TRIDC3AG034020.1	Tt--TRITD3Av1G162410.11	0.1925261	3.2933216 6767146	0.0584595 43209101	1722	396.91667	1325.0833	225	294	0.17	0.741	253.332436
Td--TRIDC3AG034020.1	Tt--TRITD3Bv1G133770.2	0.0137814	0.0518984 629605249	0.2655448 58451341	1839	435.41667	1403.5833	19.17	21.83	0.014	0.05	3.99218946
Td--TRIDC3AG034020.1	Tt--TRITD3Bv1G140820.6	0.1967732	2.7591218 3869536	0.0713173 284307232	1722	396	1326	229.5	289.5	0.173	0.731	212.240141
Td--TRIDC3BG040600.18	Tt--TRITD1Av1G209190.3	0.1327002	1.0567579 8332382	0.1255729 12115734	1626	373.5	1252.5	152.3	211.7	0.122	0.567	81.2890756
Td--TRIDC3BG040600.18	Tt--TRITD1Bv1G214990.2	0.1273799	1.2265950 1334797	0.1038483 99585952	1629	376.33333	1252.6667	146.8	227.2	0.117	0.604	94.3534626
Td--TRIDC3BG040600.18	Tt--TRITD3Av1G162410.11	0.0033956	0.0353700 117425536	0.0960019 907100345	1719	390.75	1328.25	4.5	13.5	0.003	0.035	2.72077013
Td--TRIDC3BG040600.18	Tt--TRITD3Av1G151000.4	0.1970924	2.8852001 3464622	0.0683115 275885759	1722	396.91667	1325.0833	229.7	291.3	0.173	0.734	221.938472
Td--TRIDC3BG040600.18	Tt--TRITD3Bv1G140820.6	0.0172693	0.0149152 642820377	1.1578255 3175512	1752	395	1357	23.17	5.833	0.017	0.015	1.14732802
Td--TRIDC3BG040600.18	Tt--TRITD3Bv1G133770.2	0.1935338	2.5181806 5472993	0.0768546 286579587	1722	398.08333	1323.9167	225.8	288.2	0.171	0.724	193.706204
Td--TRIDC3BG038510.2	Tt--TRITD1Av1G222580.3	0.14905	1.0064401 0021721	0.1480961 97822231	1767	420.58333	1346.4167	182	233	0.135	0.554	77.4184692

Td--TRIDC3BG038510.2	Tt--TRITD1Bv1G220000.2	0.1487684	1.08820660651699	0.136709683435741		1770	423.75	1346.25	181.7	243.3	0.135	0.574	83.7082005
Td--TRIDC3BG038510.2	Tt--TRITD3Av1G151000.4	0.0130568	0.0518881805508732	0.251633954457995		1839	435.5	1403.5	18.17	21.83	0.013	0.05	3.9913985
Td--TRIDC3BG038510.2	Tt--TRITD3Av1G162410.11	0.1880273	2.73460036435454	0.068758616061087		1722	398.16667	1323.8333	220.2	290.8	0.166	0.73	210.353874
Td--TRIDC3BG038510.2	Tt--TRITD3Bv1G133770.2	0.0101709	0.00651678007566091	1.56071926804005		1839	436.66667	1402.3333	14.17	2.833	0.01	0.006	0.50129078
Td--TRIDC3BG038510.2	Tt--TRITD3Bv1G140820.6	0.1922561	2.43413962795926	0.0789831845357364		1722	397.25	1324.75	224.7	286.3	0.17	0.721	187.24151
Td--TRIDC6AG015690.2	Tt--TRITD6Av1G037430.3	0.0011749	0.00787925828566064	0.149107725072188		1107	255.16667	851.83333	1	2	0.001	0.008	0.60609679
Td--TRIDC6AG015690.2	Tt--TRITD6Bv1G050580.1	0	0.0917810348426671	0		1107	254.66667	852.33333	0	22	0	0.086	7.0600796
Td--TRIDC6AG015690.2	Tt--TRITD7Av1G227590.3	0.0557225	1.05263760077497	0.0529360624418997		1134	261.91667	872.08333	46.83	148.2	0.054	0.566	80.9721231
Td--TRIDC6BG021470.2	Tt--TRITD6Av1G037430.3	0.0011746	0.0872351457577674	0.0134650806662304		1107	255	852	1	21	0.001	0.082	6.71039583
Td--TRIDC6BG021470.2	Tt--TRITD6Bv1G050580.1	0	0.00790000720941963	0		1107	254.5	852.5	0	2	0	0.008	0.60769286
Td--TRIDC6BG021470.2	Tt--TRITD7Av1G227590.3	0.0640597	0.997810427044074	0.064200307641729		1161	270.66667	890.33333	54.67	149.3	0.061	0.552	76.7546482

Td--TRIDC7AG058780.3	Tt--TRITD6Av1G037430.3	0.0461855	1.04669985672484	0.0441248709000351	1107	254.91667	852.08333	38.17	143.8	0.045	0.564	80.5153736
Td--TRIDC7AG058780.3	Tt--TRITD6Bv1G050580.1	0.0449112	1.00476376392246	0.044698305506437	1107	254.41667	852.58333	37.17	140.8	0.044	0.554	77.2895203
Td--TRIDC7AG058780.3	Tt--TRITD7Av1G227590.3	0.0245178	0.0292131330286225	0.839272417346312	1152	267.58333	884.41667	21.33	7.667	0.024	0.029	2.24716408
Td--TRIDC7BG051510.2	Tt--TRITD6Av1G037430.3	0.0467909	1.10759507837085	0.0422455256133219	1107	254.58333	852.41667	38.67	147.3	0.045	0.579	85.1996214
Td--TRIDC7BG051510.2	Tt--TRITD6Bv1G050580.1	0.0455158	1.01512046885208	0.044837824284207	1107	254.08333	852.91667	37.67	141.3	0.044	0.556	78.0861899
Td--TRIDC7BG051510.2	Tt--TRITD7Av1G227590.3	0.0096275	0.0898739996454092	0.107122380206223	1236	295.16667	940.83333	9	25	0.01	0.085	6.91338459
Td--TRIDC1AG027930.1	Tt--TRITD1Bv1G113560.1	0	0.0728623919963763	0	1044	244.83333	799.16667	0	17	0	0.069	5.60479938
Td--TRIDC1BG032170.1	Tt--TRITD1Av1G120860.1	0.0023287	0.0704099735380411	0.0330740418354893	1128	267.83333	860.16667	2	18	0.002	0.067	5.41615181
Td--TRIDC1BG032170.1	Tt--TRITD1Bv1G113560.1	0	0	NaN	1128	268.16667	859.83333	0	0	0	0	0
Td--TRIDC1AG059290.6	Tt--TRITD1Av1G209190.3	0.0032584	0.005355970095081	0.608375263720713	1605	374.75	1230.25	4	2	0.003	0.005	0.4119977
Td--TRIDC1AG059290.6	Tt--TRITD3Av1G162410.11	0.1153425	1.0117602624987	0.114001786468474	1584	364.91667	1219.0833	130.3	202.7	0.107	0.555	77.8277125

Td--TRIDC1AG059290.6	Tt--TRITD3Bv1G140820.6	0.1160252	1.06436257300333	0.10900910751906	1587	365.25	1221.75	131.3	207.7	0.107	0.569	81.8740441
Td--TRIDC1BG068060.2	Tt--TRITD1Bv1G214990.2	0.0015692	0.00255428088523709	0.61435820229795	1668	392.16667	1275.8333	2	1	0.002	0.003	0.19648315
Td--TRIDC1BG068060.2	Tt--TRITD3Av1G162410.11	0.1231475	1.16995122268611	0.1052586386829	1635	376.58333	1258.4167	142.9	223.1	0.114	0.592	89.9962479
Td--TRIDC1BG068060.2	Tt--TRITD3Bv1G140820.6	0.1300115	1.2134906196101	0.107138444752693	1647	378.5	1268.5	151.4	227.6	0.119	0.601	93.3454323
Td--TRIDC6BG018000.3	Tt--TRITD6Av1G027670.1	0.0302909	0.0617473768900052	0.490562113552078	1449	337.41667	1111.5833	33	20	0.03	0.059	4.74979822
Td--TRIDC6BG018000.3	Tt--TRITD6Bv1G041120.1	0.0233785	0.0348020073141141	0.671757693922535	1446	338.16667	1107.8333	25.5	11.5	0.023	0.034	2.67707749
Td--TRIDC4AG065120.1	Tt--TRITD4Av1G251160.7	0.0043789	0.00338928917659708	1.29198571027517	1452	345	1107	4.833	1.167	0.004	0.003	0.26071455
Td--TRIDC4AG065120.1	Tt--TRITD7Av1G011740.1	0.0251763	0.117991980172379	0.213373233276526	1449	345	1104	27.33	37.67	0.025	0.109	9.07630617
Td--TRIDC7AG005070.14	Tt--TRITD4Av1G251160.7	0.0219167	0.10269342006903	0.213418820044597	1449	345.58333	1103.4167	23.83	33.17	0.022	0.096	7.89949385
Td--TRIDC7AG005070.14	Tt--TRITD7Av1G011740.1	0.0158514	0.0194673918947138	0.814255430943479	1452	346.91667	1105.0833	17.33	6.667	0.016	0.019	1.49749168
Td--TRIDC7AG057270.3	Tt--TRITD7Av1G221430.1	0.0117685	0.0216651642448627	0.543197301074161	1719	405.83333	1313.1667	15.33	8.667	0.012	0.021	1.6665511

Td--TRIDC7AG057270.3	Tt--TRITD7Bv1G170250.1	0.0204151	0.125392217062182	0.162809668231129	1734	409.91667	1324.0833	26.67	47.33	0.02	0.115	9.64555516
Td--TRIDC7BG049670.7	Tt--TRITD7Av1G221430.1	0.0140936	0.11899948460855	0.118434492438824	1719	405.91667	1313.0833	18.33	44.67	0.014	0.11	9.15380651
Td--TRIDC7BG049670.7	Tt--TRITD7Bv1G170250.1	0.0163527	0.0237459614298487	0.68865249879315	1734	410	1324	21.42	9.583	0.016	0.023	1.82661242
Td--TRIDC6AG052120.1	Tt--TRITD6Bv1G202800.1	0.0068126	0.0997973386553715	0.0682643299265672	1116	305	811	5.5	28.5	0.007	0.093	7.67671836
Td--TRIDC6AG052120.1	Tt--TRITD7Av1G048910.1	0.12237	0.453365248935098	0.269914818893932	1035	277.75	757.25	85.5	94.5	0.113	0.34	34.8742499
Td--TRIDC6AG052120.1	Tt--TRITD7Bv1G022360.1	0.1100307	0.408021422500717	0.269668913604755	1017	272.75	744.25	76.17	85.83	0.102	0.315	31.3862633
Td--TRIDC6BG060870.1	Tt--TRITD6Bv1G202800.1	0	0.00327690308299791	0	1116	305.83333	810.16667	0	1	0	0.003	0.25206947
Td--TRIDC6BG060870.1	Tt--TRITD7Av1G048910.1	0.1194667	0.418760099697434	0.28528676932556	1035	278.91667	756.08333	83.5	89.5	0.11	0.321	32.2123154
Td--TRIDC6BG060870.1	Tt--TRITD7Bv1G022360.1	0.1071057	0.399471013689992	0.268118796897122	1017	273.91667	743.08333	74.17	84.83	0.1	0.31	30.7285395
Td--TRIDC4AG047480.2	Tt--TRITD4Av1G206810.2	0	0	NaN	1569	361.16667	1207.8333	0	0	0	0	0
Td--TRIDC4AG047480.2	Tt--TRITD5Bv1G252270.3	0.0677548	0.210099605578762	0.322489104402995	1296	304.25	991.75	64.25	55.75	0.065	0.183	16.1615081

Td--TRIDC5BG083460.4	Tt--TRITD4Av1G206810.2	0.0125377	0.16947222514417	0.0739810322579846	1569	362.58333	1206.4167	15	55	0.012	0.152	13.036325
Td--TRIDC5BG083460.4	Tt--TRITD5Bv1G252270.3	0.0428727	0.101395307637	0.42282706860533	1239	289.08333	949.91667	39.58	27.42	0.042	0.095	7.79963905
Td--TRIDC5AG020820.4	Tt--TRITD5Av1G091640.3	0.0542024	0.086588072808598	0.625979776360957	843	200.75	642.25	33.58	16.42	0.052	0.082	6.66062099
Td--TRIDC5AG020820.4	Tt--TRITD5Bv1G075270.1	0.0167661	0.0684352487877035	0.244991678006738	885	211.5	673.5	11.17	13.83	0.017	0.065	5.26424991
Td--TRIDC5BG021620.3	Tt--TRITD5Av1G091640.3	0.0215665	0.057212478562223	0.376955266216645	816	196.66667	619.33333	13.17	10.83	0.021	0.055	4.40095989
Td--TRIDC5BG021620.3	Tt--TRITD5Bv1G075270.1	0.0135256	0.0488260693050803	0.277015875193783	1266	296.16667	969.83333	13	14	0.013	0.047	3.75585149
Td--TRIDC4AG041540.1	Tt--TRITD4Av1G192120.1	0.0305103	0.135016924595119	0.225974124026006	1011	275.16667	735.83333	22	34	0.03	0.124	10.3859173
Td--TRIDC4AG041540.1	Tt--TRITD4Bv1G014100.1	0.2379509	0.506401622956579	0.469885710906565	984	270	714	145.6	99.42	0.204	0.368	38.953971
Td--TRIDC4BG007520.1	Tt--TRITD4Av1G192120.1	0.2392845	0.525311556754041	0.45550961817642	984	268.5	715.5	146.6	101.4	0.205	0.378	40.4085813
Td--TRIDC4BG007520.1	Tt--TRITD4Bv1G014100.1	0.0013417	0.0142691581083784	0.0940267058401689	1029	283	746	1	4	0.001	0.014	1.09762755
Td--TRIDC3BG008530.2	Tt--TRITD3Bv1G017840.1	0.0326584	0.0281619247560474	1.15966450543059	975	268.33333	706.66667	22.58	7.417	0.032	0.028	2.1663019

Td--TRIDC2AG058830.1	Tt--TRITD2Bv1G201730.10	0.0244122	0.0790440680872091	0.308843474581227	2460	586.5	1873.5	45	44	0.024	0.075	6.08031293
Td--TRIDC2AG058830.1	Tt--TRITD6Av1G156460.10	0.1517447	0.907555974135041	0.167201525921507	2427	579.91667	1847.0833	253.8	305.3	0.137	0.526	69.811998
Td--TRIDC2AG058830.1	Tt--TRITD6Bv1G142910.11	0.1371448	0.873582894625792	0.156991213467956	2364	567.5	1796.5	225.2	292.8	0.125	0.516	67.1986842
Td--TRIDC2BG061830.1	Tt--TRITD2Bv1G201730.10	0.0004933	0.00156658020404368	0.314863428603852	2667	639	2028	1	1	5E-04	0.002	0.12050617
Td--TRIDC2BG061830.1	Tt--TRITD6Av1G156460.10	0.1586372	0.860845215308567	0.184280777719342	2616	628.58333	1987.4167	284.2	321.8	0.143	0.512	66.2188627
Td--TRIDC2BG061830.1	Tt--TRITD6Bv1G142910.11	0.1587558	0.832942555202414	0.190596379634392	2616	629.91667	1986.0833	284.2	316.8	0.143	0.503	64.0725042
Td--TRIDC6AG037840.4	Tt--TRITD2Bv1G201730.10	0.1586224	0.8612732446822	0.18417199541233	2616	628.41667	1987.5833	284.2	321.8	0.143	0.512	66.2517881
Td--TRIDC6AG037840.4	Tt--TRITD6Av1G156460.10	0.0005001	0		2634	633.83333	2000.1667	1	0	5E-04	0	0
Td--TRIDC6AG037840.4	Tt--TRITD6Bv1G142910.11	0.0065322	0.0371143211932183	0.17600099691641	2634	635.16667	1998.8333	13	23	0.007	0.036	2.85494778
Td--TRIDC6BG044800.1	Tt--TRITD2Bv1G201730.10	0.1593634	0.833346822418081	0.191232979049828	2616	629.75	1986.25	285.2	316.8	0.144	0.503	64.1036017
Td--TRIDC6BG044800.1	Tt--TRITD6Av1G156460.10	0.007037	0.0371143211932183	0.189603240847122	2634	635.16667	1998.8333	14	23	0.007	0.036	2.85494778

Td--TRIDC6BG044800.1	Tt--TRITD6Bv1G142910.11	0	0	NaN	2634	636.5	1997.5	0	0	0	0	0
Td--TRIDC4AG043860.6	Tt--TRITD4Av1G198600.3	0.0007756	0.00692845573870227	0.111943371064782	1725	435	1290	1	3	0.001	0.007	0.53295813
Td--TRIDC4BG004990.4	Tt--TRITD4Av1G198600.3	0.0203814	0.178250233899183	0.114341632462028	1728	434.91667	1293.0833	26	69	0.02	0.159	13.7115565
Td--TRIDC4AG012750.6	Tt--TRITD4Av1G042360.2	0	0	NaN	2130	515.66667	1614.3333	0	0	0	0	0
Td--TRIDC4AG012750.6	Tt--TRITD4Bv1G128900.2	0.0081591	0.0715313693800487	0.114062911879357	2115	513	1602	13	35	0.008	0.068	5.50241303
Td--TRIDC4BG037560.5	Tt--TRITD4Av1G042360.2	0.007833	0.071328011617978	0.109816174503288	2025	485	1540	12	33	0.008	0.068	5.48677012
Td--TRIDC4BG037560.5	Tt--TRITD4Bv1G128900.2	0.0006496	0.00413651513929987	0.157031115488524	2025	484.83333	1540.1667	1	2	0.001	0.004	0.31819347
Td--TRIDC2AG025680.4	Tt--TRITD2Av1G072210.1	0.0380104	0.0606793079162398	0.626413831984514	1527	348.83333	1178.1667	43.67	20.33	0.037	0.058	4.66763907
Td--TRIDC2AG025680.4	Tt--TRITD2Bv1G081010.1	0.0104356	0.0640611878648543	0.162901272124366	1629	374.58333	1254.4167	13	23	0.01	0.061	4.92778368
Td--TRIDC2BG030100.5	Tt--TRITD2Av1G072210.1	0.0397752	0.11236887939933	0.353969672394324	1527	348.16667	1178.8333	45.67	36.33	0.039	0.104	8.64375995
Td--TRIDC2BG030100.5	Tt--TRITD2Bv1G081010.1	0.0072053	0.00806640085646106	0.893253313547796	1629	373.91667	1255.0833	9	3	0.007	0.008	0.62049237

Td--TRIDC1BG061690.1	Tt--TRITD3Av1G171650.1	0.2284842	0.578995916315227	0.394621362950363	1035	267.08333	767.91667	151.2	107.8	0.197	0.403	44.5381474
Td--TRIDC1BG061690.1	Tt--TRITD3Bv1G151660.1	0.2292926	0.58678582296574	0.39076036136575	1044	266.16667	777.83333	153.7	108.3	0.198	0.407	45.137371
Td--TRIDC3BG043440.2	Tt--TRITD3Bv1G151660.1	0.0051516	0		1305	331.08333	973.91667	5	0	0.005	0	0
Td--TRIDC1AG053880.1	Tt--TRITD1Av1G205950.1	0.0021284	0		609	138.5	470.5	1	0	0.002	0	0
Td--TRIDC1AG053880.1	Tt--TRITD1Bv1G200860.1	0.0284177	0.100148544468392	0.283755478883934	1485	373.33333	1111.6667	31	35	0.028	0.094	7.70373419
Td--TRIDC1AG053880.1	Tt--TRITD3Av1G171180.1	0.2861648	0.56346660719439	0.507864782488311	1092	279.33333	812.66667	193.3	110.7	0.238	0.396	43.3435852
Td--TRIDC1AG053880.1	Tt--TRITD3Bv1G150780.1	0.3071565	0.576841599716673	0.532479769666476	1035	266.91667	768.08333	193.6	107.4	0.252	0.402	44.3724307
Td--TRIDC3AG038300.1	Tt--TRITD1Bv1G200860.1	0.3239761	0.54031312010137	0.599608128188567	1239	311.83333	927.16667	243.9	120.1	0.263	0.385	41.5625477
Td--TRIDC3AG038300.1	Tt--TRITD3Av1G171180.1	0.2227947	0.537274102996463	0.414676108156998	1098	279.58333	818.41667	157.8	107.3	0.193	0.384	41.3287772
Td--TRIDC3AG038300.1	Tt--TRITD3Bv1G150780.1	0.2326162	0.52818246389706	0.440408783191285	1041	266.83333	774.16667	154.8	101.2	0.2	0.379	40.6294203
Td--TRIDC3BG043280.1	Tt--TRITD1Bv1G200860.1	0.277579	0.622344559877688	0.446021476202718	1167	299.91667	867.08333	201.2	126.8	0.232	0.423	47.8726585

Td--TRIDC3BG043280.1	Tt--TRITD3Av1G171180.1	0.2402904	0.573503774226506	0.418986562156567	1113	282.5	830.5	170.8	113.3	0.206	0.401	44.1156749
Td--TRIDC3BG043280.1	Tt--TRITD3Bv1G150780.1	0.2475903	0.606865769753381	0.407981946869876	1056	269.58333	786.41667	165.8	112.2	0.211	0.416	46.6819823
Td--TRIDC5AG066400.8	Tt--TRITD5Av1G228010.3	0.0031244	0.00525281201683735	0.594804130834112	1665	382.08333	1282.9167	4	2	0.003	0.005	0.40406246
Td--TRIDC5AG066400.8	Tt--TRITD5Bv1G226960.4	0.0016261	0.100895472487721	0.0161169478497392	1602	370.75	1231.25	2	35	0.002	0.094	7.76119019
Td--TRIDC5BG071320.5	Tt--TRITD5Av1G228010.3	0.0503831	0.179806254805906	0.280207954752309	1620	378.41667	1241.5833	60.5	60.5	0.049	0.16	13.8312504
Td--TRIDC5BG071320.5	Tt--TRITD5Bv1G226960.4	0.0372988	0.0418790883787865	0.890631118594394	1551	360.08333	1190.9167	43.33	14.67	0.036	0.041	3.22146834
Td--TRIDC5AG020280.1	Tt--TRITD5Av1G086470.1	0.0495012	0.0652585327422225	0.758539647030267	1596	380	1216	58.25	23.75	0.048	0.063	5.01988713
Td--TRIDC5AG032410.5	Tt--TRITD5Bv1G121250.4	0.0019256	0.0619250067196664	0.0310964782888502	2031	471.08333	1559.9167	3	28	0.002	0.059	4.76346206
Td--TRIDC5AG032410.5	Tt--TRITD0Uv1G042030.1	0.0245754	0.0289733090894047	0.848208089307106	1632	363.58333	1268.4167	30.67	10.33	0.024	0.028	2.22871608
Td--TRIDC5BG034210.7	Tt--TRITD5Bv1G121250.4	0.0006381	0		2043	475.16667	1567.8333	1	0	0.001	0	0
Td--TRIDC5BG034210.7	Tt--TRITD0Uv1G042030.1	0.026203	0.0884877733817011	0.29611949321391	1632	363.41667	1268.5833	32.67	30.33	0.026	0.083	6.8067518

Td--TRIDC2AG070140.10	Tt--TRITD2Av1G270510.6	0.0013128	0		3966	916.5	3049.5	4	0	0.001	0	0
Td--TRIDC2AG070140.10	Tt--TRITD2Bv1G233690.10	0.0082466	0.0828666073744607	0.0995165506588824	3966	917.75	3048.25	25	72	0.008	0.078	6.37435441
Td--TRIDC2BG075830.1	Tt--TRITD2Av1G270510.6	0.0089088	0.0804830033446936	0.110691578926508	3966	917.25	3048.75	27	70	0.009	0.076	6.19100026
Td--TRIDC2BG075830.1	Tt--TRITD2Bv1G233690.10	0.0006561	0.00217825529772948	0.301219119629607	3969	919.5	3049.5	2	2	0.001	0.002	0.1675581
Td--TRIDC6AG020750.1	Tt--TRITD6Av1G054950.1	0.0007911	0		1626	361.33333	1264.6667	1	0	0.001	0	0
Td--TRIDC6AG020750.1	Tt--TRITD6Bv1G065890.1	0.013174	0.120976973211965	0.108896685544764	1626	362.5	1263.5	16.5	40.5	0.013	0.112	9.30592102
Td--TRIDC6BG026600.3	Tt--TRITD6Av1G054950.1	0.0123665	0.121067579367253	0.102145258798394	1626	362.25	1263.75	15.5	40.5	0.012	0.112	9.31289072
Td--TRIDC6BG026600.3	Tt--TRITD6Bv1G065890.1	0.0015857	0		1626	363.41667	1262.5833	2	0	0.002	0	0
Td--TRIDC5AG057130.2	Tt--TRITD5Av1G205000.3	0.0022727	0.00734399992846323	0.309467461059725	2310	547.33333	1762.6667	4	4	0.002	0.007	0.56492307
Td--TRIDC5AG057130.2	Tt--TRITD5Bv1G200940.1	0.008856	0.060364830835347	0.146707831087657	2469	594.83333	1874.1667	16.5	34.5	0.009	0.058	4.64344853
Td--TRIDC5BG061270.2	Tt--TRITD5Av1G205000.3	0.0072934	0.0634324681209124	0.114979168161857	2163	509.66667	1653.3333	12	31	0.007	0.061	4.87942062

Td--TRIDC5BG061270.2	Tt--TRITD5Bv1G200940.1	0.0011345	0.00901724117215036	0.125818684235447	2322	557.83333	1764.1667	2	5	0.001	0.009	0.69363394
Td--TRIDC5AG032000.2	Tt--TRITD5Bv1G119650.1	0.0201853	0.0936472148657488	0.215545948366101	1554	382.41667	1171.5833	23.33	33.67	0.02	0.088	7.20363191
Td--TRIDC5AG032000.2	Tt--TRITD0Uv1G047610.1	0.0037007	0.00970744541864889	0.38122531414588	1554	380.16667	1173.8333	4.333	3.667	0.004	0.01	0.74672657
Td--TRIDC5BG033660.2	Tt--TRITD5Bv1G119650.1	0.0008562	0		1554	385.33333	1168.6667	1	0	0.001	0	0
Td--TRIDC5BG033660.2	Tt--TRITD0Uv1G047610.1	0.0172781	0.0944599268834398	0.182915004632196	1554	383.08333	1170.9167	20	34	0.017	0.089	7.26614822
Td--TRIDC2AG010980.4	Tt--TRITD2Av1G024070.6	0	0.00207451078759352	0	3996	965.41667	3030.5833	0	2	0	0.002	0.15957775
Td--TRIDC2AG010980.4	Tt--TRITD2Bv1G031320.6	0.0072973	0.0796098003311754	0.0916634636871207	3996	966.5	3029.5	22	73	0.007	0.076	6.12383079
Td--TRIDC2BG013430.2	Tt--TRITD2Av1G024070.6	0.0072971	0.0796170426266173	0.0916525921802457	3996	966.41667	3029.5833	22	73	0.007	0.076	6.12438789
Td--TRIDC2BG013430.2	Tt--TRITD2Bv1G031320.6	0	0	NaN	4005	969.83333	3035.1667	0	0	0	0	0
Td--TRIDC5AG024780.6	Tt--TRITD5Av1G112160.1	0.0006988	0.0062849529800631	0.111188114269111	1911	479.33333	1431.6667	1	3	0.001	0.006	0.48345792
Td--TRIDC5AG024780.6	Tt--TRITD5Bv1G092620.2	0.0091459	0.0887460629299847	0.103057175454398	1908	477.91667	1430.0833	13	40	0.009	0.084	6.82662023

Td--TRIDC5BG025890.5	Tt--TRITD5Av1G112160.1	0.0084374	0.0887789295178338	0.0950385889968959	1908	477.75	1430.25	12	40	0.008	0.084	6.82914842
Td--TRIDC5BG025890.5	Tt--TRITD5Bv1G092620.2	0	0.0020862322214076	0	1920	480	1440	0	1	0	0.002	0.1604794
Td--TRIDC6AG039240.3	Tt--TRITD6Av1G162640.1	0.0234732	0.019717056650243	1.19049999211312	1167	265.5	901.5	20.83	5.167	0.023	0.019	1.51669667
Td--TRIDC6AG039240.3	Tt--TRITD6Bv1G148270.1	0.0623976	0.0795182990242753	0.784695177566379	1167	265.08333	901.91667	54	20	0.06	0.075	6.11679223
Td--TRIDC6BG046090.3	Tt--TRITD6Av1G162640.1	0.0307168	0.0778452659565614	0.394587263577064	1848	419.25	1428.75	43	31	0.03	0.074	5.98809738
Td--TRIDC6BG046090.3	Tt--TRITD6Bv1G148270.1	0.0006986	0		1851	419	1432	1	0	0.001	0	0
Td--TRIDC2AG025210.3	Tt--TRITD2Av1G069790.2	0	0	NaN	2079	480.16667	1598.8333	0	0	0	0	0
Td--TRIDC2AG025210.3	Tt--TRITD2Bv1G079430.3	0.0091225	0.0825309635481236	0.11053400565534	2079	479.83333	1599.1667	14.5	37.5	0.009	0.078	6.34853566
Td--TRIDC2BG029650.2	Tt--TRITD2Av1G069790.2	0.0068285	0.0782042588314467	0.0873156730442644	2103	484.75	1618.25	11	36	0.007	0.074	6.01571222
Td--TRIDC2BG029650.2	Tt--TRITD2Bv1G079430.3	0.0021593	0.0072401872384622	0.298234484444953	2109	485.75	1623.25	3.5	3.5	0.002	0.007	0.55693748
Td--TRIDC2AG024350.1	Tt--TRITD2Av1G066770.1	0.0869896	0.11193563419003	0.777139866011752	972	236.41667	735.58333	60.42	24.58	0.082	0.104	8.6104334

Td--TRIDC2AG024350.1	Tt--TRITD2Bv1G076660.1	0.0109879	0.075872891291839	0.144820496226415	969	235.58333	733.41667	8	17	0.011	0.072	5.83637625
Td--TRIDC2BG028790.2	Tt--TRITD2Av1G069790.2	0.4536053	0.741593361128086	0.611663123071611	888	212.5	675.5	229.9	100.1	0.34	0.471	57.0456432
Td--TRIDC2BG028790.2	Tt--TRITD2Av1G066770.1	0.0775346	0.197701006191836	0.392181160108862	969	235.91667	733.08333	54	41	0.074	0.174	15.2077697
Td--TRIDC2BG028790.2	Tt--TRITD2Bv1G076660.1	0.0013667	0		969	236.66667	732.33333	1	0	0.001	0	0
Td--TRIDC1AG002650.1	Tt--TRITD0Uv1G000510.7	0.0014288	0		1815	413.91667	1401.0833	2	0	0.001	0	0
Td--TRIDC1AG002650.1	Tt--TRITD0Uv1G004130.5	0.0184536	0.118196062931122	0.156126715834684	1815	416.08333	1398.9167	25.5	45.5	0.018	0.109	9.09200484
Td--TRIDC6AG024780.3	Tt--TRITD6Av1G073760.2	0.1832943	0.27129041935898	0.675638645537406	822	205	617	100.3	46.67	0.163	0.228	20.8684938
Td--TRIDC6AG024780.3	Tt--TRITD7Av1G209280.1	0.3155857	0.934037145898307	0.337872772954532	846	210	636	163.8	112.2	0.258	0.534	71.8490112
Td--TRIDC6AG024780.3	Tt--TRITD7Bv1G162110.1	0.3153921	0.950547545282606	0.331800484979582	846	210.33333	635.66667	163.7	113.3	0.257	0.539	73.1190419
Td--TRIDC7AG054160.3	Tt--TRITD6Av1G073760.2	0.2072379	0.585928508332545	0.353691419239156	1098	266.83333	831.16667	150.5	108.5	0.181	0.407	45.0714237
Td--TRIDC7AG054160.3	Tt--TRITD6Bv1G077930.1	0.2046463	0.590843810669946	0.346362784436033	1098	267	831	148.8	109.2	0.179	0.409	45.4495239

Td--TRIDC7AG054160.3	Tt--TRITD7Av1G209280.1	0	0.00702581250270012	0	1188	286	902	0	2	0	0.007	0.54044712
Td--TRIDC7AG054160.3	Tt--TRITD7Bv1G162110.1	0.0011099	0.0505606560281201	0.0219514184182557	1188	286.33333	901.66667	1	14	0.001	0.049	3.88928123
Td--TRIDC7BG046810.3	Tt--TRITD6Av1G073760.2	0.2106117	0.567610355823165	0.371049794291614	1098	267.5	830.5	152.5	106.5	0.184	0.398	43.6623351
Td--TRIDC7BG046810.3	Tt--TRITD6Bv1G077930.1	0.2080071	0.57240603590732	0.363390740687512	1098	267.66667	830.33333	150.8	107.2	0.182	0.4	44.0312335
Td--TRIDC7BG046810.3	Tt--TRITD7Av1G209280.1	0.0033358	0.0617782480748455	0.0539965101411712	1188	286.66667	901.33333	3	17	0.003	0.059	4.75217293
Td--TRIDC7BG046810.3	Tt--TRITD7Bv1G162110.1	0.002223	0.00349243937246322	0.63653136473622	1188	287	901	2	1	0.002	0.003	0.26864918
Td--TRIDC1AG050190.1	Tt--TRITD1Av1G197990.2	0.0014657	0.00230680689353918	0.635397282851082	1800	434.16667	1365.8333	2	1	0.001	0.002	0.17744668
Td--TRIDC1AG050190.1	Tt--TRITD1Bv1G187410.2	0.007386	0.134508289498696	0.0549114011556151	1791	430.41667	1360.5833	10	53	0.007	0.123	10.3467915
Td--TRIDC1AG050190.1	Tt--TRITD3Av1G181840.2	0.3345657	1.28815173176702	0.25972541647255	1734	411	1323	357.1	252.9	0.27	0.615	99.0885948
Td--TRIDC1AG050190.1	Tt--TRITD3Bv1G162740.2	0.3259125	1.25624537336748	0.259433814973163	1731	409.75	1321.25	349.3	249.7	0.264	0.61	96.6342595
Td--TRIDC1BG057000.1	Tt--TRITD1Av1G197990.2	0.0059023	0.129023890418143	0.0457455942832789	1791	430.25	1360.75	8	51	0.006	0.119	9.92491465

Td--TRIDC1BG057000.1	Tt--TRITD1Bv1G187410.2	0	0.00232829057765231	0	1794	430.16667	1363.8333	0	1	0	0.002	0.17909928
Td--TRIDC1BG057000.1	Tt--TRITD3Av1G181840.2	0.3342959	1.27396442395981	0.262405958611797	1734	408.91667	1325.0833	357.4	250.6	0.27	0.613	97.9972634
Td--TRIDC1BG057000.1	Tt--TRITD3Bv1G162740.2	0.3230436	1.24413075712993	0.259654043033045	1731	408	1323	347.2	247.7	0.262	0.607	95.7023659
Td--TRIDC3AG040710.1	Tt--TRITD1Av1G197990.2	0.3313186	1.30007197761386	0.254846344381073	1734	410.66667	1323.3333	354.4	253.6	0.268	0.617	100.005537
Td--TRIDC3AG040710.1	Tt--TRITD1Bv1G187410.2	0.3168141	1.30084734326506	0.243544394626008	1704	402.75	1301.25	336.2	248.8	0.258	0.618	100.06518
Td--TRIDC3AG040710.1	Tt--TRITD3Av1G181840.2	0.001446	0		1803	418.5	1384.5	2	0	0.001	0	0
Td--TRIDC3AG040710.1	Tt--TRITD3Bv1G162740.2	0.0208983	0.0663762067337091	0.314846759708261	1800	417.16667	1382.8333	28.5	26.5	0.021	0.064	5.10586206
Td--TRIDC3BG045870.3	Tt--TRITD1Av1G197990.2	0.322262	1.24723019157164	0.258382101941531	1731	409.25	1321.75	346.3	248.7	0.262	0.608	95.940784
Td--TRIDC3BG045870.3	Tt--TRITD1Bv1G187410.2	0.3212048	1.26479921517767	0.253957108814932	1731	408	1323	345.7	249.3	0.261	0.611	97.2922473
Td--TRIDC3BG045870.3	Tt--TRITD3Av1G181840.2	0.0205241	0.0650950450031555	0.315294729559683	1800	417	1383	28	26	0.02	0.062	5.00731115
Td--TRIDC3BG045870.3	Tt--TRITD3Bv1G162740.2	0.0007219	0		1803	417.16667	1385.8333	1	0	0.001	0	0

Td--TRIDC7AG009510.2	Tt--TRITD7Av1G024300.1	0.0051209	0.0113746256035582	0.450206490956674	765	177.16667	587.83333	3	2	0.005	0.011	0.8749712
Td--TRIDC7AG012240.3	Tt--TRITD7Av1G030560.1	0.0157936	0.0297212119320387	0.531392181055493	1962	469	1493	23.33	13.67	0.016	0.029	2.28624707
Td--TRIDC6AG042270.3	Tt--TRITD6Av1G173820.1	0.1118106	0.178767375370161	0.62545314169559	1182	293.91667	888.08333	92.25	46.75	0.104	0.159	13.7513366
Td--TRIDC6AG042270.3	Tt--TRITD6Bv1G161420.1	0.1472336	0.281974552836387	0.522152146382099	1188	297.83333	890.16667	119	70	0.134	0.235	21.6903502
Td--TRIDC6AG042270.3	Tt--TRITD7Av1G083120.2	0.2577164	0.545085748949433	0.472799677421988	1146	289.75	856.25	186.8	112.3	0.218	0.387	41.929673
Td--TRIDC6AG042270.3	Tt--TRITD7Bv1G058080.2	0.4579181	1.22730677190666	0.373108094901961	591	158.66667	432.33333	148.2	95.83	0.343	0.604	94.4082132
Td--TRIDC6BG049330.2	Tt--TRITD6Av1G173820.1	0.0202785	0.086438668040675	0.234600233027205	1287	312.33333	974.66667	19.5	25.5	0.02	0.082	6.64912831
Td--TRIDC6BG049330.2	Tt--TRITD6Bv1G161420.1	0.0010471	0.00326087470207754	0.321116474752615	1263	307.33333	955.66667	1	1	0.001	0.003	0.25083652
Td--TRIDC6BG049330.2	Tt--TRITD7Av1G083120.2	0.1867691	0.448504124127258	0.416426642189408	1281	314.75	966.25	159.8	106.3	0.165	0.338	34.5003172
Td--TRIDC6BG049330.2	Tt--TRITD7Bv1G058080.2	0.4300403	1.33915637446288	0.32112774504063	597	160.33333	436.66667	142.9	100.1	0.327	0.624	103.012029
Td--TRIDC7AG029400.6	Tt--TRITD6Av1G173820.1	0.1931971	0.365466659887451	0.528631273689721	1257	310.25	946.75	161.3	89.75	0.17	0.289	28.11282

Td--TRIDC7AG029400.6	Tt--TRITD6Bv1G161420.1	0.1677721	0.426692427367973	0.393192004991309	1251	308.08333	942.91667	141.8	100.3	0.15	0.325	32.8224944
Td--TRIDC7AG029400.6	Tt--TRITD7Av1G083120.2	0.0010373	0		1284	319.33333	964.66667	1	0	0.001	0	0
Td--TRIDC7AG029400.6	Tt--TRITD7Bv1G058080.2	0.3318493	0.707801022400452	0.468845411243417	663	180.08333	482.91667	129.5	82.5	0.268	0.458	54.4462325
Td--TRIDC7BG020300.3	Tt--TRITD6Av1G173820.1	0.157325	0.334822578745814	0.469875631139311	1143	279.25	863.75	122.6	75.42	0.142	0.27	25.755583
Td--TRIDC7BG020300.3	Tt--TRITD6Bv1G161420.1	0.1559019	0.376109821010814	0.414511533793417	1143	278.08333	864.91667	121.8	82.25	0.141	0.296	28.9315247
Td--TRIDC7BG020300.3	Tt--TRITD7Av1G083120.2	0.0011612	0.0669174226371055	0.0173529641708794	1143	281.16667	861.83333	1	18	0.001	0.064	5.14749405
Td--TRIDC7BG020300.3	Tt--TRITD7Bv1G058080.2	0.381206	0.821231940768545	0.464187926158886	507	137.25	369.75	110.5	68.5	0.299	0.499	63.1716878
Td--TRIDC1AG052430.1	Tt--TRITD1Av1G203640.1	0.0211809	0.0373429983772616	0.567199481468634	2343	583.33333	1759.6667	36.75	21.25	0.021	0.036	2.87253834
Td--TRIDC1AG052430.1	Tt--TRITD3Av1G164820.1	0.1147778	0.395707852589574	0.290056801465112	1602	380.5	1221.5	130	117	0.106	0.307	30.4390656
Td--TRIDC1AG052430.1	Tt--TRITD3Bv1G142220.1	0.3514902	2.11776390663665	0.165972335986967	2346	557.08333	1788.9167	502	393	0.281	0.705	162.904916
Td--TRIDC1BG059980.1	Tt--TRITD1Av1G203640.1	0.0545668	0.143480067903826	0.380309408704363	2331	581.33333	1749.6667	92.08	75.92	0.053	0.131	11.0369283

Td--TRIDC1BG059980.1	Tt--TRITD1Av1G201620.1	0.0487389	0.176322278082073	0.276419691227632	372	85.916667	286.08333	13.5	13.5	0.047	0.157	13.5632522
Td--TRIDC1BG059980.1	Tt--TRITD1Bv1G199190.1	0.1210778	0.317467594792552	0.381386267631744	2376	587.25	1788.75	200	152	0.112	0.259	24.4205842
Td--TRIDC1BG059980.1	Tt--TRITD3Av1G164820.1	0.1203193	0.357928291418113	0.33615475446977	1605	383.83333	1221.1667	135.8	109.3	0.111	0.285	27.5329455
Td--TRIDC3AG036880.3	Tt--TRITD1Av1G203640.1	0.2588967	0.648928981782857	0.398959982918882	1953	489.5	1463.5	320.4	212.6	0.219	0.434	49.917614
Td--TRIDC3AG036880.3	Tt--TRITD1Bv1G199190.1	0.2108178	0.600137735044423	0.351282334416608	2019	501.33333	1517.6667	278.9	207.1	0.184	0.413	46.1644412
Td--TRIDC3AG036880.3	Tt--TRITD3Av1G164820.1	0.006696	0.00424299271808696	1.57813720236106	1644	393.91667	1250.0833	8.333	1.667	0.007	0.004	0.32638406
Td--TRIDC3AG036880.3	Tt--TRITD3Bv1G142350.1	0.1260918	0.301569733812641	0.418118256727455	2043	505.75	1537.25	178.4	125.6	0.116	0.248	23.1976718
Td--TRIDC3BG040940.1	Tt--TRITD1Bv1G199190.1	0.3541204	1.80737790825242	0.195930450882675	2349	556.91667	1792.0833	505.8	380.2	0.282	0.683	139.02907
Td--TRIDC3BG040940.1	Tt--TRITD3Av1G164850.1	0.2528121	1.54787213105319	0.163328814856083	2337	534.91667	1802.0833	386.8	350.3	0.215	0.655	119.067087
Td--TRIDC3BG040940.1	Tt--TRITD3Bv1G142220.1	0	0.00179910131248118	0	2433	556.5	1876.5	0	1	0	0.002	0.13839241
Td--TRIDC7BG067700.1	Tt--TRITD7Bv1G217320.3	0.168896	0.589916692738967	0.286304884632633	585	134.25	450.75	68.17	54.83	0.151	0.408	45.3782071

Td--TRIDC7BG067700.1	Tt--TRITD7Bv1G218360.3	0.1743293	0.592152417662069	0.294399335647513	585	133.91667	451.08333	70.17	54.83	0.156	0.409	45.550186
Td--TRIDC1AG061210.1	Tt--TRITD1Bv1G218630.1	0.0019934	0.154389040653111	0.012911257474677	660	157.66667	502.33333	1	22	0.002	0.14	11.8760801
Td--TRIDC1AG061210.1	Tt--TRITD3Av1G155690.2	0.0610403	0.798414951807141	0.0764518250583799	894	211.66667	682.33333	40	104	0.059	0.491	61.4165348
Td--TRIDC1AG061210.1	Tt--TRITD3Bv1G135290.1	0.0610403	0.798414951807141	0.0764518250583799	894	211.66667	682.33333	40	104	0.059	0.491	61.4165348
Td--TRIDC1BG070170.4	Tt--TRITD1Bv1G218630.1	0	0.117143011731485	0	660	156.75	503.25	0	17	0	0.108	9.0110009
Td--TRIDC1BG070170.4	Tt--TRITD3Av1G155690.2	0.0625284	0.733807692263891	0.0852109406220229	918	217.91667	700.08333	42	102	0.06	0.468	56.4467456
Td--TRIDC1BG070170.4	Tt--TRITD3Bv1G135290.1	0.0625284	0.721698448387851	0.0866406791273554	918	217.91667	700.08333	42	101	0.06	0.463	55.5152653
Td--TRIDC3AG034530.2	Tt--TRITD1Bv1G218630.1	0.0648148	0.709558818050817	0.0913452342297723	654	154.75	499.25	31	71	0.062	0.459	54.5814475
Td--TRIDC3AG034530.2	Tt--TRITD3Av1G155690.2	0	0	NaN	945	222.33333	722.66667	0	0	0	0	0
Td--TRIDC3AG034530.2	Tt--TRITD3Bv1G135290.1	0	0.0760143706952155	0	933	221.33333	711.66667	0	16	0	0.072	5.84725928
Td--TRIDC3BG038930.3	Tt--TRITD1Bv1G218630.1	0.0648148	0.693097251872527	0.0935147502887126	654	154.75	499.25	31	70	0.062	0.452	53.3151732

Td--TRIDC3BG038930.3	Tt--TRITD3Av1G155690.2	0	0.0761350440528097	0	942	221	721	0	16	0	0.072	5.85654185
Td--TRIDC3BG038930.3	Tt--TRITD3Bv1G135290.1	0.0097903	0		942	222.33333	719.66667	7	0	0.01	0	0
Td--TRIDC2AG028890.5	Tt--TRITD2Av1G084730.2	0.4427342	2.60060537632376	0.170242735787871	1944	463.91667	1480.0833	494.9	337.1	0.334	0.727	200.046567
Td--TRIDC2BG032550.5	Tt--TRITD2Av1G084730.2	0.453775	2.98999431599426	0.151764509822024	1947	465.41667	1481.5833	504.4	342.6	0.34	0.736	229.999563
Td--TRIDC2BG032550.5	Tt--TRITD2Bv1G090370.1	0	0	NaN	2100	518.33333	1581.6667	0	0	0	0	0
Td--TRIDC6BG030200.2	Tt--TRITD6Bv1G077930.1	0.001183	0.00359282124197445	0.32925811368404	1125	279	846	1	1	0.001	0.004	0.27637086
Td--TRIDC6BG030200.2	Tt--TRITD7Av1G209280.1	0.2010591	0.609133407760861	0.330073993985945	1098	267.33333	830.66667	146.5	111.5	0.176	0.417	46.856416
Td--TRIDC6BG030200.2	Tt--TRITD7Bv1G162110.1	0.2011517	0.583168552429674	0.344928871658064	1098	267.66667	830.33333	146.5	108.5	0.176	0.405	44.8591194
Td--TRIDC4AG047400.2	Tt--TRITD4Av1G206190.2	0.0008098	0.0025652013035985	0.315696848032724	3252	781	2471	2	2	0.001	0.003	0.19732318
Td--TRIDC5BG083500.1	Tt--TRITD4Av1G206190.2	0.1490584	0.402038536940989	0.370756551794264	2946	714.41667	2231.5833	301.7	222.3	0.135	0.311	30.9260413
Td--TRIDC7AG002140.2	Tt--TRITD7Av1G004690.5	0.0179356	0.0364042045856986	0.492680379335556	1008	232.16667	775.83333	13.75	8.25	0.018	0.036	2.80032343

Td--TRIDC2AG080090.1	Tt--TRITD2Av1G291610.2	0.0566158	0.104705627478431	0.540714145124144	1287	296.75	990.25	54	29	0.055	0.098	8.05427904
Td--TRIDC2AG080090.1	Tt--TRITD2Bv1G261380.1	0.0677454	0.163713170069208	0.41380539240602	588	132.58333	455.41667	29.5	19.5	0.065	0.147	12.5933208
Td--TRIDC2BG088230.2	Tt--TRITD2Av1G291610.2	0.059962	0.161909614151363	0.370342612796858	1293	301	992	57.17	43.83	0.058	0.146	12.4545857
Td--TRIDC6AG000550.4	Tt--TRITD6Av1G000920.4	0.0038486	0.0137573460987946	0.279750856630974	2337	513.5	1823.5	7	7	0.004	0.014	1.05825739
Td--TRIDC3BG001050.1	Tt--TRITD3Bv1G001440.2	0	0	NaN	1560	385	1175	0	0	0	0	0
Td--TRIDC3AG004290.4	Tt--TRITD3Av1G008980.2	0.0417496	0.19105651781488	0.218519471248368	1737	417.5	1319.5	53.58	70.42	0.041	0.169	14.6966552
Td--TRIDC3AG004290.4	Tt--TRITD3Bv1G011530.1	0.0993272	0.329132910186198	0.301784428714638	2001	483.58333	1517.4167	141.2	128.8	0.093	0.266	25.3179162
Td--TRIDC3BG006020.1	Tt--TRITD3Av1G008980.2	0.042369	0.179891216186298	0.235525567137798	1722	413.16667	1308.8333	53.92	66.08	0.041	0.16	13.8377859
Td--TRIDC3BG006020.1	Tt--TRITD3Bv1G011530.1	0.0957248	0.325156319881328	0.294396100619572	1986	480.08333	1505.9167	135.3	126.7	0.09	0.264	25.0120246
Td--TRIDC3AG011590.6	Tt--TRITD3Av1G028450.2	0.0013806	0.00205198486415606	0.672802347368806	1938	488	1450	2	1	0.001	0.002	0.15784499
Td--TRIDC3BG015640.4	Tt--TRITD3Av1G028450.2	0.0111004	0.1028745229051	0.107902258986652	1941	488.91667	1452.0833	16	47	0.011	0.096	7.91342484

Td--TRIDC5BG047840.2	Tt--TRITD5Av1G170840.1	0.1234669	0.315380609777428	0.391485496445416		1884	421.41667	1462.5833	166.5	108.5	0.114	0.257	24.2600469
Td--TRIDC5BG047840.2	Tt--TRITD5Bv1G162020.4	0.0123813	0.0604922649238134	0.204676461677485		1896	430.16667	1465.8333	18	25	0.012	0.058	4.65325115
Td--TRIDC5AG055160.3	Tt--TRITD5Av1G199510.5	0	0.0076531276306813	0		1119	262.66667	856.33333	0	2	0	0.008	0.58870213
Td--TRIDC5AG055160.3	Tt--TRITD5Av1G202570.2	0.4379037	NaN	NaN		1065	249.25	815.75	270.6	194.4	0.332	0.78	High Sequence Divergence Value (pS>=0.75)
Td--TRIDC5AG055160.3	Tt--TRITD5Bv1G194390.4	0.0023403	0.123694083778229	0.0189202556965995		1119	263.08333	855.91667	2	30	0.002	0.114	9.51492952
Td--TRIDC5AG055160.3	Tt--TRITD5Bv1G198000.2	0.353001	1.7928682933406	0.196891739015922		1074	254.16667	819.83333	230.8	173.2	0.282	0.681	137.912946
Td--TRIDC5AG056200.2	Tt--TRITD5Av1G202570.2	0.1453941	0.238307937901449	0.610110041530824		1032	244.5	787.5	104.1	49.92	0.132	0.204	18.3313798
Td--TRIDC5AG056200.2	Tt--TRITD5Av1G199510.5	0.3518928	1.57557323336148	0.223342735258737		1056	250.16667	805.83333	226.3	164.7	0.281	0.658	121.197941
Td--TRIDC5AG056200.2	Tt--TRITD5Bv1G198000.2	0.0048241	0.0843697968021209	0.0571784858824653		1095	263.16667	831.83333	4	21	0.005	0.08	6.48998437
Td--TRIDC5AG056200.2	Tt--TRITD5Bv1G194390.4	0.3509878	1.64991377837595	0.212731023459429		1056	250.91667	805.08333	225.7	167.3	0.28	0.667	126.916444

Td--TRIDC5BG059170.3	Tt--TRITD5Av1G199510.5	0.0023408	0.123608948443107	0.0189369801974374	1119	263.25	855.75	2	30	0.002	0.114	9.50838065
Td--TRIDC5BG059170.3	Tt--TRITD5Bv1G194390.4	0	0.00762395383263804	0	1119	263.66667	855.33333	0	2	0	0.008	0.58645799
Td--TRIDC5AG049770.1	Tt--TRITD5Av1G187760.2	0	0.0024174074111765	0	1761	414.33333	1346.6667	0	1	0	0.002	0.18595442
Td--TRIDC5AG049770.1	Tt--TRITD5Bv1G177650.5	0.006	0.0878043629503894	0.0683340986563195	1749	410.33333	1338.6667	8	34	0.006	0.083	6.75418177
Td--TRIDC5BG053430.2	Tt--TRITD5Av1G187760.2	0.0065987	0.074437593881568	0.0886480588887442	1719	399.83333	1319.1667	8.667	28.33	0.007	0.071	5.72596876
Td--TRIDC5BG053430.2	Tt--TRITD5Bv1G177650.5	0.0007582	0.00502304096064115	0.150953574727698	1719	399.5	1319.5	1	2	0.001	0.005	0.38638777
Td--TRIDC5AG033270.1	Tt--TRITD5Bv1G125410.2	0.167217	0.225227150593817	0.742437287929162	1167	315.25	851.75	127.7	61.33	0.15	0.195	17.3251654
Td--TRIDC5AG033270.1	Tt--TRITD0Uv1G046260.2	0.147273	0.198073199569571	0.743528128034025	1155	311.16667	843.83333	112.8	54.17	0.134	0.174	15.2364
Td--TRIDC5BG035150.3	Tt--TRITD5Bv1G125410.2	0.1547054	0.208702678702075	0.741271683100083	1164	313.33333	850.66667	118.9	57.08	0.14	0.182	16.0540522
Td--TRIDC5BG035150.3	Tt--TRITD0Uv1G046260.2	0.1619175	0.245864643775762	0.65856342272815	1167	313.25	853.75	124.3	65.67	0.146	0.21	18.9126649
Td--TRIDC5AG051290.3	Tt--TRITD5Bv1G183720.1	0.1219701	0.674239178141445	0.180900353429972	510	116.16667	393.83333	44.33	51.67	0.113	0.445	51.8645522

Td--TRIDC5AG067100.1	Tt--TRITD5Av1G229390.2	0	0.00413508973058175	0	1032	242.5	789.5	0	1	0	0.004	0.31808383
Td--TRIDC5AG067100.1	Tt--TRITD5Bv1G228940.2	0.0063668	0.0870541284395653	0.0731356640725363	1032	243.33333	788.66667	5	20	0.006	0.082	6.69647142
Td--TRIDC5BG072290.4	Tt--TRITD5Av1G229390.2	0.0551004	0.140907844978917	0.391038298255491	1014	240.66667	773.33333	41.08	30.92	0.053	0.128	10.839065
Td--TRIDC5BG072290.4	Tt--TRITD5Bv1G228940.2	0.0337764	0.0792939168477378	0.425964515464878	999	237	762	25.17	17.83	0.033	0.075	6.09953207
Td--TRIDC3AG003160.1	Tt--TRITD3Av1G003600.2	0	0	NaN	777	187.83333	589.16667	0	0	0	0	0
Td--TRIDC3BG000190.2	Tt--TRITD3Av1G003600.2	0.0256962	0.15185405301196	0.169216236315174	1059	260.66667	798.33333	20.17	35.83	0.025	0.137	11.681081
Td--TRIDC3BG000190.2	Tt--TRITD0Uv1G022400.1	0.0095351	0.0128400311303118	0.742609152080375	1071	261.83333	809.16667	7.667	3.333	0.009	0.013	0.9876947
Td--TRIDC6AG021710.1	Tt--TRITD6Av1G059560.3	0.001039	0.00690851171492774	0.150388571081626	1254	290.83333	963.16667	1	2	0.001	0.007	0.53142398
Td--TRIDC6AG021710.1	Tt--TRITD6Bv1G068340.3	0.0031207	0.0913536554131526	0.0341603215575783	1254	290.66667	963.33333	3	25	0.003	0.086	7.02720426
Td--TRIDC6AG021710.1	Tt--TRITD7Av1G255570.2	0.0911213	0.730086859285578	0.124808846421006	1254	292.5	961.5	82.5	136.5	0.086	0.467	56.1605276
Td--TRIDC6AG021710.1	Tt--TRITD7Bv1G205360.3	0.0917426	0.82995924850139	0.110538631359594	1254	292.83333	961.16667	83	147	0.086	0.502	63.8430191

Td--TRIDC6BG027410.1	Tt--TRITD6Av1G059560.3	0.002079	0.0759685005383254	0.02736665059236	1254	290.66667	963.33333	2	21	0.002	0.072	5.84373081
Td--TRIDC6BG027410.1	Tt--TRITD6Bv1G068340.3	0	0.0138973287208706	0	1254	290.5	963.5	0	4	0	0.014	1.06902529
Td--TRIDC6BG027410.1	Tt--TRITD7Av1G255570.2	0.0893453	0.735336931205108	0.12150255993614	1254	292.33333	961.66667	81	137	0.084	0.469	56.5643793
Td--TRIDC6BG027410.1	Tt--TRITD7Bv1G205360.3	0.0899644	0.825669593525126	0.108959307579007	1254	292.66667	961.33333	81.5	146.5	0.085	0.501	63.5130457
Td--TRIDC7AG068050.1	Tt--TRITD6Av1G059560.3	0.0899478	0.739191675198536	0.121684026087941	1254	292.5	961.5	81.5	137.5	0.085	0.47	56.8608981
Td--TRIDC7AG068050.1	Tt--TRITD6Bv1G068340.3	0.0893453	0.744511357531834	0.120005314429586	1254	292.33333	961.66667	81	138	0.084	0.472	57.2701044
Td--TRIDC7AG068050.1	Tt--TRITD7Av1G255570.2	0	0.00667041639863754	0	1281	301.16667	979.83333	0	2	0	0.007	0.51310895
Td--TRIDC7AG068050.1	Tt--TRITD7Bv1G205360.3	0.0030817	0.163506964677769	0.018847322132892	1275	299.5	975.5	3	44	0.003	0.147	12.5774588
Td--TRIDC7BG061870.1	Tt--TRITD6Bv1G068340.3	0.089981	0.856231636998301	0.105089507948703	1254	292.83333	961.16667	81.5	149.5	0.085	0.511	65.8639721
Td--TRIDC7BG061870.1	Tt--TRITD7Av1G255570.2	0.0025676	0.157206976847221	0.016332712390595	1275	299.66667	975.33333	2.5	42.5	0.003	0.142	12.0928444
Td--TRIDC7BG061870.1	Tt--TRITD7Bv1G205360.3	0.0020541	0		1275	300	975	2	0	0.002	0	0

Td--TRIDC7AG045740.2	Tt--TRITD7Av1G173340.3	0.0008125	0.00266016683228101	0.305436773783536	1608	376.58333	1231.4167	1	1	0.001	0.003	0.20462822
Td--TRIDC7AG045740.2	Tt--TRITD7Bv1G130380.4	0.0190882	0.0470634079226066	0.405584042148974	1593	372.66667	1220.3333	23	17	0.019	0.046	3.62026215
Td--TRIDC7BG037680.2	Tt--TRITD7Av1G173340.3	0.018739	0.0455223847945452	0.411643577964265	1551	362.25	1188.75	22	16	0.019	0.044	3.50172191
Td--TRIDC7BG037680.2	Tt--TRITD7Bv1G130380.4	0	0.00276498009016413	0	1551	362.33333	1188.6667	0	1	0	0.003	0.21269078
Td--TRIDC1AG040730.2	Tt--TRITD1Av1G170240.2	0.0013263	0.00191938684411613	0.690981235295084	2031	521.66667	1509.3333	2	1	0.001	0.002	0.14764514
Td--TRIDC1AG040730.2	Tt--TRITD1Bv1G156400.1	0.0777881	0.162769016101706	0.477904584580205	2025	516	1509	111.5	75.5	0.074	0.146	12.5206935
Td--TRIDC1BG046350.4	Tt--TRITD1Av1G170240.2	0.0785745	0.16164589643209	0.486090217715244	2007	512.33333	1494.6667	111.5	74.5	0.075	0.145	12.4342997
Td--TRIDC1BG046350.4	Tt--TRITD1Bv1G156400.1	0	0.00195694827271029	0	2016	511.66667	1504.3333	0	1	0	0.002	0.15053448
Td--TRIDC3AG005110.2	Tt--TRITD3Av1G011010.3	0.001119	0.00663721145773687	0.168592739268112	1197	302.66667	894.33333	1	2	0.001	0.007	0.51055473
Td--TRIDC3AG005110.2	Tt--TRITD3Bv1G009870.4	0.0170996	0.119928508409559	0.14258185550982	1185	297.75	887.25	15	33	0.017	0.111	9.22526988
Td--TRIDC3BG005340.1	Tt--TRITD3Av1G011010.3	0.0148139	0.127385536926584	0.116291770564942	1185	298.75	886.25	13	35	0.015	0.117	9.79888746

Td--TRIDC3BG005340.1	Tt--TRITD3Bv1G009870.4	0.0136497	0.0521344743064754	0.261816504845455	1185	297.83333	887.16667	12	15	0.014	0.05	4.01034418
Td--TRIDC4AG067540.2	Tt--TRITD4Av1G255960.1	0.0330435	0.0841249275951196	0.392790723555208	1959	458.66667	1500.3333	48.5	36.5	0.032	0.08	6.47114828
Td--TRIDC4AG067540.2	Tt--TRITD7Av1G006960.1	0.033066	0.0790808194415211	0.418129493874075	1959	459.66667	1499.3333	48.5	34.5	0.032	0.075	6.08313996
Td--TRIDC3BG003740.4	Tt--TRITD3Av1G001260.2	0.0454819	0.124556559659091	0.36515085286017	1266	299.16667	966.83333	42.67	34.33	0.044	0.115	9.58127382
Td--TRIDC2AG028370.1	Tt--TRITD2Av1G083210.1	0.0006532	0.00195758675574277	0.333696075523793	2043	511.5	1531.5	1	1	0.001	0.002	0.1505836
Td--TRIDC4AG002010.1	Tt--TRITD4Bv1G170030.2	0.1516284	0.271007590476187	0.559498637007512	1080	285.41667	794.58333	109.1	64.92	0.137	0.227	20.8467377
Td--TRIDC4BG049660.1	Tt--TRITD4Av1G004390.1	0.0315283	0.10623788779727	0.296770630047815	795	212	583	18	21	0.031	0.099	8.17214522
Td--TRIDC4BG049660.1	Tt--TRITD4Bv1G170030.2	0.0006652	0.00374065613327928	0.177826694026476	2040	536	1504	1	2	0.001	0.004	0.28774278
Td--TRIDC3AG046380.3	Tt--TRITD3Av1G202040.8	0.0010527	0		2466	564.83333	1901.1667	2	0	0.001	0	0
Td--TRIDC3AG046380.3	Tt--TRITD3Bv1G178340.3	0.0497061	0.0673883262055209	0.73760654264633	1623	363.33333	1259.6667	60.58	23.42	0.048	0.064	5.1837174
Td--TRIDC3BG052620.2	Tt--TRITD3Av1G202040.8	0.0389654	0.049085600209437	0.793826287436215	1320	301.66667	1018.3333	38.67	14.33	0.038	0.048	3.7758154

Td--TRIDC3BG052620.2	Tt--TRITD3Bv1G178340.3	0.0350502	0.0370966312101704	0.944834650270738		1326	303.91667	1022.0833	35	11	0.034	0.036	2.85358702
Td--TRIDC5AG051310.6	Tt--TRITD5Bv1G183000.5	0.2351317	0.724758240067772	0.324427785588307		1803	445.5	1357.5	274	207	0.202	0.465	55.7506339
Td--TRIDC7AG018660.25	Tt--TRITD7Bv1G021510.1	0.1975801	0.381903658401193	0.517355829158243		180	45.666667	134.33333	23.33	13.67	0.174	0.299	29.3772045
Td--TRIDC6BG052000.1	Tt--TRITD6Av1G181680.1	0.4503656	0.669904210998038	0.672283602392527		381	95.75	285.25	96.58	42.42	0.339	0.443	51.5310932
Td--TRIDC6BG052000.1	Tt--TRITD6Bv1G171100.1	0.1320585	0.187122184471385	0.705734226482892		795	196.25	598.75	72.5	32.5	0.121	0.166	14.3940142
Td--TRIDC6AG027120.2	Tt--TRITD6Av1G085990.3	0.0011735	0.00383142595704184	0.306277511004383		2229	523.33333	1705.6667	2	2	0.001	0.004	0.29472507
Td--TRIDC6AG027120.2	Tt--TRITD6Bv1G090340.3	0.011827	0.0271742485110101	0.435227614815954		2229	524.58333	1704.4167	20	14	0.012	0.027	2.09032681
Td--TRIDC6BG033330.6	Tt--TRITD6Av1G085990.3	0.0139003	0.0419282461401825	0.331525773699893		1821	441.41667	1379.5833	19	18	0.014	0.041	3.2252497
Td--TRIDC6BG033330.6	Tt--TRITD6Bv1G090340.3	0.0007258	0.00681048947680986	0.106567559573051		1821	442.5	1378.5	1	3	0.001	0.007	0.52388381
Td--TRIDC1BG000450.3	Tt--TRITD1Av1G000530.1	0.0097931	0.282694718241685	0.0346418204517112		1077	254.75	822.25	8	60	0.01	0.236	21.7457476
Td--TRIDC3AG067170.2	Tt--TRITD3Av1G263280.2	0	0.0156527420441852	0		795	193.66667	601.33333	0	3	0	0.015	1.20405708

Td--TRIDC5AG013160.4	Tt--TRITD5Av1G045620.11	0.0197938	0.0276829253871922	0.715019975198079	2202	521.25	1680.75	32.83	14.17	0.02	0.027	2.1294558
Td--TRIDC5AG013160.4	Tt--TRITD5Bv1G043010.13	0.0381645	0.120502436525644	0.316711530575384	2247	531.5	1715.5	63.83	59.17	0.037	0.111	9.26941819
Td--TRIDC5BG015110.1	Tt--TRITD4Av1G134780.6	0.2192454	0.97357992212686	0.225195039032581	1923	440.5	1482.5	281.8	240.2	0.19	0.545	74.8907632
Td--TRIDC5BG015110.1	Tt--TRITD5Av1G045620.11	0.0294258	0.11412809800106	0.257831692528109	1890	434.5	1455.5	42	46	0.029	0.106	8.77908446
Td--TRIDC5BG015110.1	Tt--TRITD5Bv1G043010.13	0.0277551	0.0359738765094755	0.77153450662917	1893	434.16667	1458.8333	39.75	15.25	0.027	0.035	2.76722127
Td--TRIDC2AG028730.4	Tt--TRITD2Av1G084500.2	0.0013233	0.00205058227535112	0.645346038019313	2001	488.33333	1512.6667	2	1	0.001	0.002	0.1577371
Td--TRIDC2AG028730.4	Tt--TRITD2Bv1G089940.2	0.0369545	0.0992190295197042	0.372454155456731	2001	489.58333	1511.4167	54.5	45.5	0.036	0.093	7.63223304
Td--TRIDC2BG032450.3	Tt--TRITD2Av1G084500.2	0.0376499	0.101554116052796	0.370737409304238	2001	489.58333	1511.4167	55.5	46.5	0.037	0.095	7.81185508
Td--TRIDC2BG032450.3	Tt--TRITD2Bv1G089940.2	0.0019892	0		2001	490.83333	1510.1667	3	0	0.002	0	0
Td--TRIDC3AG002440.3	Tt--TRITD3Bv1G001440.2	0.0270498	0.158675556124908	0.170472108279048	2037	531.41667	1505.5833	40	76	0.027	0.143	12.205812
Td--TRIDC3AG012960.1	Tt--TRITD3Av1G032230.2	0.0023021	0		1731	425.83333	1305.1667	3	0	0.002	0	0

Td--TRIDC3AG012960.1	Tt--TRITD3Bv1G039450.3	0.0222819	0.0854218746334456	0.260845510697612	1731	425.25	1305.75	28.67	34.33	0.022	0.081	6.57091343
Td--TRIDC3BG017420.1	Tt--TRITD3Av1G032230.2	0.0199343	0.0852096429892616	0.233944158885565	1731	426.25	1304.75	25.67	34.33	0.02	0.081	6.55458792
Td--TRIDC3BG017420.1	Tt--TRITD3Bv1G039450.3	0.0015337	0		1731	425.66667	1305.3333	2	0	0.002	0	0
Td--TRIDC7AG049870.2	Tt--TRITD7Av1G192070.4	0.000429	0.00391858951726676	0.109490022899238	3099	767.58333	2331.4167	1	3	4E-04	0.004	0.30142996
Td--TRIDC7AG049870.2	Tt--TRITD7Bv1G152370.2	0.0497018	0.121284736946398	0.409794251879709	3084	762	2322	111.7	85.33	0.048	0.112	9.32959515
Td--TRIDC7BG043940.1	Tt--TRITD7Av1G192070.4	0.0485464	0.12520568586254	0.387732826915449	2898	714	2184	102.7	82.33	0.047	0.115	9.6312066
Td--TRIDC7BG043940.1	Tt--TRITD7Bv1G152370.2	0.0009139	0.0028057082054386	0.325717427557193	2904	714.16667	2189.8333	2	2	0.001	0.003	0.21582371
Td--TRIDC1AG027610.11	Tt--TRITD1Av1G118910.11	0	0	NaN	2067	473.5	1593.5	0	0	0	0	0
Td--TRIDC1AG027610.11	Tt--TRITD1Bv1G117690.3	0.0044044	0.0457658297442697	0.0962371948287374	2067	473	1594	7	21	0.004	0.044	3.52044844
Td--TRIDC1BG033320.4	Tt--TRITD1Av1G118910.11	0.0047452	0.0472039363097519	0.100526412173093	1917	437.16667	1479.8333	7	20	0.005	0.046	3.63107202
Td--TRIDC1BG033320.4	Tt--TRITD1Bv1G117690.3	0.0013523	0		1917	436.66667	1480.3333	2	0	0.001	0	0

Td--TRIDC2AG030300.1	Tt--TRITD2Av1G095350.5	0.0098801	0.0131983101377534	0.74858796567626		2076	496.83333	1579.1667	15.5	6.5	0.01	0.013	1.01525463
Td--TRIDC2AG030300.1	Tt--TRITD2Bv1G096060.3	0.004443	0.0435958062133858	0.101914549522575		2076	495.83333	1580.1667	7	21	0.004	0.042	3.35352355
Td--TRIDC2BG033980.3	Tt--TRITD2Av1G095350.5	0.0130938	0.0596965353346621	0.219339303481989		2076	496.66667	1579.3333	20.5	28.5	0.013	0.057	4.59204118
Td--TRIDC2BG033980.3	Tt--TRITD2Bv1G096060.3	0.000633	0.00202020324166721	0.313357077504967		2076	495.66667	1580.3333	1	1	0.001	0.002	0.15540025
Td--TRIDC5AG078780.1	Tt--TRITD4Bv1G207930.11	0.0280784	0.0983929473184089	0.285369651533873		2061	482.58333	1578.4167	43.5	44.5	0.028	0.092	7.56868826
Td--TRIDC5AG078780.1	Tt--TRITD5Av1G258690.15	0.0135437	0.0256755327798694	0.527492781220761		2040	475.41667	1564.5833	21	12	0.013	0.025	1.97504098
Td--TRIDC6AG053160.4	Tt--TRITD6Av1G209030.4	0	0	NaN		1806	410.16667	1395.8333	0	0	0	0	0
Td--TRIDC6AG053160.4	Tt--TRITD6Bv1G205270.3	0.0021636	0.0325254218363474	0.0665200615485585		1797	408.41667	1388.5833	3	13	0.002	0.032	2.50195553
Td--TRIDC6BG061710.3	Tt--TRITD6Av1G209030.4	0.002658	0.0391718894193417	0.0678536905882627		1953	445.41667	1507.5833	4	17	0.003	0.038	3.01322226
Td--TRIDC6BG061710.3	Tt--TRITD6Bv1G205270.3	0.0006636	0.00224887724719241	0.295067261119309		1953	445.33333	1507.6667	1	1	0.001	0.002	0.17299056
Td--TRIDC7AG029890.1	Tt--TRITD7Av1G084620.26	0.0118291	0.0198475846758864	0.595999400914857		2067	476.5	1590.5	18.67	9.333	0.012	0.02	1.52673728

Td--TRIDC7AG029890.1	Tt--TRITD7Bv1G059260.26	0.0175961	0.0804704712156252	0.218665489880116	2067	476.16667	1590.8333	27.67	36.33	0.017	0.076	6.19003625
Td--TRIDC7BG020630.1	Tt--TRITD7Av1G084620.26	0.0182438	0.082750841243805	0.220466452986428	2067	476.5	1590.5	28.67	37.33	0.018	0.078	6.36544933
Td--TRIDC7BG020630.1	Tt--TRITD7Bv1G059260.26	0.0124655	0.0220212338645908	0.566067945203166	2067	476.16667	1590.8333	19.67	10.33	0.012	0.022	1.69394107
												Average=27.6340178125438

The Ka/Ks ratio and divergence times between the genes of *T.turgidum* and *A.speltoides*												
**Gene ID**	**Gene ID**	**Ka**	**Ks**	**Ka/Ks**	**EffectiveLen**	**Average S-sites**	**Average N-sites**	**cN**	**cS**	**pN**	**pS**	**Divergence times (Mya)**
Tt--TRITD6Av1G170870.1	Aes-CH6S01G388700.1	0.105272	0.250568389028305	0.42013262960469	1140	270.333	869.667	85.4167	57.5833	0.098	0.21	19.2744915
Tt--TRITD6Bv1G157460.4	Aes-CH6S01G388700.1	0.009032	0.036058446260535	0.250489244720249	1245	298.25	946.75	8.5	10.5	0.009	0.04	2.77372664
Tt--TRITD4Av1G214900.3	Aes-CH5S01G635300.1	0.009957	0.0462701767540049	0.215192605160078	1323	312	1011	10	14	0.01	0.04	3.55924437
Tt--TRITD5Bv1G245660.3	Aes-CH5S01G635300.1	0.008967	0.0359545967168393	0.249404198620978	1323	313.333	1009.67	9	11	0.009	0.04	2.76573821
Tt--TRITD4Av1G049530.4	Aes-CH4S01G275300.1	0.025036	0.103533165597153	0.241817720611919	984	232.667	751.333	18.5	22.5	0.025	0.1	7.96408966
Tt--TRITD4Bv1G124550.2	Aes-CH4S01G275300.1	0	0.0236240002945282	0	1107	258	849	0	6	0	0.02	1.81723079
Tt--TRITD1Av1G032550.8	Aes-CH1S01G121300.1	0.004774	0.0762734268698046	0.0625853913690585	1647	386.083	1260.92	6	28	0.005	0.07	5.86718668
Tt--TRITD1Bv1G041580.2	Aes-CH1S01G121300.1	0.000867	0.0616936821058823	0.0140490721543162	1509	354.583	1154.42	1	21	0.001	0.06	4.74566785
Tt--TRITD1Av1G220750.2	Aes-CH1S01G422500.1	0.010845	0.0386403355785428	0.280661875092429	1818	424.833	1393.17	15	16	0.011	0.04	2.97233351
Tt--TRITD1Av1G220750.2	Aes-CH3S01G287600.1	0.116746	1.37796524082199	0.0847232693703563	1185	267.75	917.25	99.1667	168.833	0.108	0.63	105.997326

Tt--TRITD1Bv1G218290.1	Aes-CH1S01G422500.1	0.005792	0.0311920202169871	0.185696001956616	1812	425.5	1386.5	8	13	0.006	0.03	2.39938617
Tt--TRITD1Bv1G218290.1	Aes-CH3S01G287600.1	0.120198	1.33524278151607	0.0900193726488985	1185	268.083	916.917	101.833	167.167	0.111	0.62	102.710983
Tt--TRITD3Av1G155110.1	Aes-CH1S01G422500.1	0.131744	1.30052768422985	0.1013001229066	1440	328.167	1111.83	134.333	202.667	0.121	0.62	100.040591
Tt--TRITD3Av1G155110.1	Aes-CH3S01G287600.1	0.016172	0.0646583257422691	0.250109791081995	1212	274.417	937.583	15	17	0.016	0.06	4.97371736
Tt--TRITD3Bv1G135620.1	Aes-CH1S01G422500.1	0.122936	1.32163028928653	0.0930182171527697	1440	330.25	1109.75	125.833	205.167	0.113	0.62	101.663868
Tt--TRITD3Bv1G135620.1	Aes-CH3S01G287600.1	0.003213	0.029515028952746	0.108874360126327	1212	276.417	935.583	3	8	0.003	0.03	2.27038684
Tt--TRITD7Av1G032080.4	Aes-CH7S01G034800.1	0.004416	0.118550524752603	0.0372519852884539	1182	273.583	908.417	4	30	0.004	0.11	9.11927113
Tt--TRITD7Av1G032080.4	Aes-CH7S01G040500.1	0.014617	0.120863906464681	0.12093589077302	174	35.8333	138.167	2	4	0.014	0.11	9.29722357
Tt--TRITD7Bv1G002620.4	Aes-CH7S01G034800.1	0.003538	0.0615098637456262	0.057515042192818	1104	254	850	3	15	0.004	0.06	4.73152798
Tt--TRITD7Bv1G002620.4	Aes-CH7S01G040500.1	0.014617	0.0579999025523778	0.25201394394436	174	35.8333	138.167	2	2	0.014	0.06	4.46153097
Tt--TRITD7Av1G178880.7	Aes-CH7S01G479200.1	0	0.0526531940049364	0	435	98.3333	336.667	0	5	0	0.05	4.05024569

Tt--TRITD7Av1G178880.7	Aes-CH7S01G477100.1	0.001084	0.0378868426026762	0.0286066386918701	1194	270.667	923.333	1	10	0.001	0.04	2.91437251
Tt--TRITD7Bv1G140030.6	Aes-CH7S01G479100.1	0.012498	0.0444574574471552	0.281129130147292	675	150.583	524.417	6.5	6.5	0.012	0.04	3.41980442
Tt--TRITD7Bv1G140030.6	Aes-CH7S01G477100.1	0	0.0247518043527187	0	1263	287.5	975.5	0	7	0	0.02	1.90398495
Tt--TRITD1Av1G222580.3	Aes-CH1S01G431400.1	0.008873	0.0958357091220637	0.0925809593007284	1794	433.5	1360.5	12	39	0.009	0.09	7.37197762
Tt--TRITD1Av1G222580.3	Aes-CH3S01G281200.1	0.157947	1.03385847950683	0.152773865744122	1767	421.25	1345.75	191.667	236.333	0.142	0.56	79.5275753
Tt--TRITD1Bv1G220000.2	Aes-CH1S01G431400.1	0.002927	0.0892438790498591	0.0327978959523383	1809	439.75	1369.25	4	37	0.003	0.08	6.86491377
Tt--TRITD1Bv1G220000.2	Aes-CH3S01G281200.1	0.155374	1.13434289729373	0.136972255185271	1770	424.417	1345.58	188.833	248.167	0.14	0.58	87.2571459
Tt--TRITD3Av1G151000.4	Aes-CH1S01G431400.1	0.155093	1.02255020345943	0.15167283869453	1770	423.417	1346.58	188.667	236.333	0.14	0.56	78.657708
Tt--TRITD3Av1G151000.4	Aes-CH3S01G281200.1	0.000713	0.0572159775533474	0.0124602924012231	1839	435.667	1403.33	1	24	0.001	0.06	4.40122904
Tt--TRITD3Av1G151000.4	Aes-CH3S01G300500.1	0.195384	2.73473987977138	0.0714452200488085	1701	392.917	1308.08	225	287	0.172	0.73	210.364606
Tt--TRITD3Bv1G133770.2	Aes-CH1S01G431400.1	0.15655	1.00548754279242	0.15569596695384	1770	424.083	1345.92	190.167	234.833	0.141	0.55	77.3451956

Tt--TRITD3Bv1G133770.2	Aes-CH3S01G281200.1	0.003574	0.0161980842889023	0.220668935044077		1839	436.833	1402.17	5	7	0.004	0.02	1.24600648
Tt--TRITD3Bv1G133770.2	Aes-CH3S01G300300.1	0.132592	2.44057814362181	0.0543282886721166		1473	340.25	1132.75	137.667	245.333	0.122	0.72	187.73678
Tt--TRITD1Bv1G214990.2	Aes-CH1S01G408400.1	0.010244	0.0754167015730403	0.135828976729899		1668	390.25	1277.75	13	28	0.01	0.07	5.80128474
Tt--TRITD1Bv1G214990.2	Aes-CH3S01G300300.1	0.126641	1.1634683506924	0.108848219217405		1494	343.333	1150.67	134.083	202.917	0.117	0.59	89.4975654
Tt--TRITD3Av1G162410.11	Aes-CH1S01G408400.1	0.135474	1.25740218445295	0.107740977267222		1638	376.667	1261.33	156.333	229.667	0.124	0.61	96.723245
Tt--TRITD3Av1G162410.11	Aes-CH3S01G300300.1	0.007797	0.0358544744535693	0.217471914046156		1503	342.75	1160.25	9	12	0.008	0.04	2.7580365
Tt--TRITD3Bv1G140820.6	Aes-CH1S01G408400.1	0.135671	1.30910986782328	0.10363574291475		1650	378.25	1271.75	157.833	234.167	0.124	0.62	100.700759
Tt--TRITD3Bv1G140820.6	Aes-CH3S01G300300.1	0.008584	0.0450135072946896	0.190693531792783		1515	343.333	1171.67	10	15	0.009	0.04	3.46257748
Tt--TRITD1Av1G209190.3	Aes-CH3S01G300300.1	0.128141	1.05006576243917	0.122031403208924		1491	340.667	1150.33	135.5	192.5	0.118	0.57	80.7742894
Tt--TRITD6Av1G037430.3	Aes-CH6S01G223700.1	0.003205	0.114947230865338	0.0278835171487635		813	187.667	625.333	2	20	0.003	0.11	8.84209468
Tt--TRITD6Av1G037430.3	Aes-CH7S01G615700.1	0.046758	1.11343770157145	0.0419941943560718		1107	254	853	38.6667	147.333	0.045	0.58	85.649054

Tt-- TRITD6Av1G0374 30.3	Aes- CH7S01G592100.1	0.04681	1.07084141 346941	0.04371312 45361176	1107	254.917	852.083	38.6667	145.333	0.045	0.57	82.3724164
Tt-- TRITD6Bv1G0505 80.1	Aes- CH6S01G223700.1	0.0016	0.04400895 68527774	0.03634655 68784514	813	187.167	625.833	1	8	0.002	0.04	3.38530437
Tt-- TRITD6Bv1G0505 80.1	Aes- CH7S01G615700.1	0.045484	1.02009166 299726	0.04458787 77141053	1107	253.5	853.5	37.6667	141.333	0.044	0.56	78.4685895
Tt-- TRITD6Bv1G0505 80.1	Aes- CH7S01G592100.1	0.045534	0.99729792 0439844	0.04565750 99732291	1107	254.417	852.583	37.6667	140.333	0.044	0.55	76.7152246
Tt-- TRITD7Av1G2275 90.3	Aes- CH6S01G223700.1	0.051366	1.01719459 446228	0.05049753 9500752	813	188.583	624.417	31	105	0.05	0.56	78.245738
Tt-- TRITD7Av1G2275 90.3	Aes- CH7S01G615700.1	0.046856	0.23530988 8194804	0.19912631 0713873	1248	295.833	952.167	43.25	59.75	0.045	0.2	18.1007606
Tt-- TRITD7Av1G2275 90.3	Aes- CH7S01G592100.1	0.04804	0.18518713 5365258	0.25941486 0232998	1248	297.083	950.917	44.25	48.75	0.047	0.16	14.2451643
Tt-- TRITD6Av1G0276 70.1	Aes- CH6S01G197100.1	0.109455	0.18051133 6279558	0.60635852 6767644	1086	262.833	823.167	83.8333	42.1667	0.102	0.16	13.8854874
Tt-- TRITD6Av1G0276 70.1	Aes- CH6S01G225500.1	0.029791	0.05481214 25538575	0.54350219 1735965	1497	350	1147	33.5	18.5	0.029	0.05	4.21631866
Tt-- TRITD6Bv1G0411 20.1	Aes- CH6S01G197100.1	0.113074	0.23236198 7661441	0.48662916 2493911	1101	268.167	832.833	87.4167	53.5833	0.105	0.2	17.8739991
Tt-- TRITD6Bv1G0411 20.1	Aes- CH6S01G225500.1	0.019046	0.03951169 6280512	0.48203018 8890218	1494	350.75	1143.25	21.5	13.5	0.019	0.04	3.03936125

Tt--TRITD4Av1G251160.7	Aes-CH7S01G875500.1	0.011221	0.0531062273628451	0.211284965323988	1452	344.583	1107.42	12.3333	17.6667	0.011	0.05	4.08509441
Tt--TRITD7Av1G011740.1	Aes-CH7S01G875500.1	0.021414	0.11505765997465	0.186116987396476	1449	343.75	1105.25	23.3333	36.6667	0.021	0.11	8.85058923
Tt--TRITD7Av1G221430.1	Aes-CH7S01G583200.1	0.013574	0.115236495246923	0.117791436367129	1719	405.667	1313.33	17.6667	43.3333	0.013	0.11	8.86434579
Tt--TRITD7Av1G221430.1	Aes-CH7S01G560300.1	0.012542	0.110412056839335	0.113596464923078	1719	405.833	1313.17	16.3333	41.6667	0.012	0.1	8.49323514
Tt--TRITD7Bv1G170250.1	Aes-CH7S01G560300.1	0.017897	0.0731871521317476	0.244537983139636	1734	409.917	1324.08	23.4167	28.5833	0.018	0.07	5.62978093
Tt--TRITD7Bv1G170250.1	Aes-CH7S01G583200.1	0.015835	0.0669660604809241	0.236466746132782	1734	409.75	1324.25	20.75	26.25	0.016	0.06	5.15123542
Tt--TRITD6Bv1G202800.1	Aes-CH6S01G486800.1	0.15913	0.256252859532278	0.620987834911066	1059	287.167	771.833	110.667	62.3333	0.143	0.22	19.7117584
Tt--TRITD6Bv1G202800.1	Aes-CH6S01G486100.1	0.004331	0.107105405370392	0.0404391215116197	1116	305.583	810.417	3.5	30.5	0.004	0.1	8.23887734
Tt--TRITD6Bv1G202800.1	Aes-CH7S01G128000.1	0.109122	0.400651023322969	0.272360697175106	1020	274.917	745.083	75.6667	85.3333	0.102	0.31	30.8193095
Tt--TRITD7Av1G048910.1	Aes-CH7S01G128000.1	0.002633	0.0961622513292521	0.0273780649076557	1038	277	761	2	25	0.003	0.09	7.39709626
Tt--TRITD7Bv1G022360.1	Aes-CH6S01G486800.1	0.266712	0.502971237768445	0.530271943876969	1002	268.333	733.667	164.667	98.3333	0.224	0.37	38.6900952

Tt--TRITD7Bv1G022360.1	Aes-CH7S01G128000.1	0.001319	0.0409531005261388	0.0321998002948671	1035	276	759	1	11	0.001	0.04	3.1502385
Tt--TRITD4Av1G206810.2	Aes-CH5S01G665200.1	0.011695	0.187009442781175	0.0625343561241292	1569	362.5	1206.5	14	60	0.012	0.17	14.3853418
Tt--TRITD5Bv1G252270.3	Aes-CH5S01G665200.1	0.019564	0.036904322238023	0.530140549196177	1164	270.75	893.25	17.25	9.75	0.019	0.04	2.83879402
Tt--TRITD5Av1G091640.3	Aes-CH5S01G146600.1	0.020066	0.0696419467394346	0.288124314098006	873	208	665	13.1667	13.8333	0.02	0.07	5.35707283
Tt--TRITD5Bv1G075270.1	Aes-CH5S01G146600.1	0.018257	0.0326895821136056	0.558500104085056	1362	317.833	1044.17	18.8333	10.1667	0.018	0.03	2.51458324
Tt--TRITD4Av1G192120.1	Aes-CH4S01G066000.1	0.240159	0.538902310923573	0.445643859839499	987	269.25	717.75	147.5	103.5	0.206	0.38	41.4540239
Tt--TRITD4Bv1G014100.1	Aes-CH4S01G066000.1	0.020384	0.114252748719646	0.178411438827204	1029	283.083	745.917	15	30	0.02	0.11	8.78867298
Tt--TRITD2Bv1G201730.10	Aes-CH2S01G410800.1	0.005443	0.0319792646706658	0.170214134658332	2667	638.833	2028.17	11	20	0.005	0.03	2.45994344
Tt--TRITD2Bv1G201730.10	Aes-CH6S01G361000.1	0.158178	0.808067960217736	0.195748662274665	2616	630.417	1985.58	283.167	311.833	0.143	0.49	62.1590739
Tt--TRITD6Av1G156460.10	Aes-CH2S01G410800.1	0.154284	0.871375824822425	0.177057507565776	2616	628.417	1987.58	277.167	323.833	0.139	0.52	67.0289096
Tt--TRITD6Av1G156460.10	Aes-CH6S01G361000.1	0.007039	0.0453824621193559	0.15511177933772	2634	635.833	1998.17	14	28	0.007	0.04	3.49095862

Tt--TRITD6Bv1G142910.11	Aes-CH2S01G410800.1	0.154399	0.828538471502112	0.18635058692384	2616	629.75	1986.25	277.167	315.833	0.14	0.5	63.7337286
Tt--TRITD6Bv1G142910.11	Aes-CH6S01G361000.1	0.003011	0.014259737710894	0.211139416707664	2634	637.167	1996.83	6	9	0.003	0.01	1.0969029
Tt--TRITD4Av1G198600.3	Aes-CH4S01G045900.1	0.012248	0.141219140034339	0.0867332536499366	1707	431.167	1275.83	15.5	55.5	0.012	0.13	10.8630108
Tt--TRITD4Av1G042360.2	Aes-CH4S01G288500.1	0.062643	0.126900351623443	0.493638236498916	549	141.333	407.667	24.5	16.5	0.06	0.12	9.76156551
Tt--TRITD4Bv1G128900.2	Aes-CH4S01G288500.1	0.060058	0.109906222020403	0.546444172010317	549	141.833	407.167	23.5	14.5	0.058	0.1	8.45432477
Tt--TRITD2Av1G072210.1	Aes-CH2S01G206600.1	0.0362	0.122509241501212	0.295491537153017	1527	348	1179	41.6667	39.3333	0.035	0.11	9.42378781
Tt--TRITD2Bv1G081010.1	Aes-CH2S01G206600.1	0.003994	0.044079702765306	0.0906059987749453	1629	373.75	1255.25	5	16	0.004	0.04	3.39074637
Tt--TRITD3Av1G171650.1	Aes-CH3S01G320300.1	0.266838	0.638027132594916	0.418223555174867	1191	305.083	885.917	198.917	131.083	0.225	0.43	49.0790102
Tt--TRITD1Bv1G200860.1	Aes-CH1S01G358800.1	0.023772	0.0916866770674624	0.25927505800895	1575	399.75	1175.25	27.5	34.5	0.023	0.09	7.05282131
Tt--TRITD3Av1G171180.1	Aes-CH1S01G358800.1	0.304656	0.652118830121486	0.46717818717868	1134	291.917	842.083	210.833	127.167	0.25	0.44	50.1629869
Tt--TRITD3Av1G171180.1	Aes-CH3S01G320300.1	0.022482	0.125522378871155	0.179111140417861	1152	294.167	857.833	19	34	0.022	0.12	9.65556761

Tt--TRITD3Bv1G150780.1	Aes-CH1S01G358800.1	0.325805	0.647798321944976	0.502941885672488	1077	279.5	797.5	210.75	121.25	0.264	0.43	49.8306401
Tt--TRITD3Bv1G150780.1	Aes-CH3S01G320300.1	0.044364	0.139667222025121	0.317637244003039	1095	282.5	812.5	35	36	0.043	0.13	10.7436325
Tt--TRITD5Av1G228010.3	Aes-CH5S01G571400.1	0.001627	0.0854407908989227	0.0190438228057703	1602	371.5	1230.5	2	30	0.002	0.08	6.57236853
Tt--TRITD5Bv1G226960.4	Aes-CH5S01G571400.1	0.000813	0.0587302022696126	0.0138477934849731	1602	371.75	1230.25	1	21	0.001	0.06	4.51770787
Tt--TRITD5Av1G086470.1	Aes-CH5S01G135500.1	0.033137	0.070830453628411	0.467833323532586	1569	373.583	1195.42	38.75	25.25	0.032	0.07	5.44849643
Tt--TRITD5Bv1G121250.4	Aes-CH5S01G228100.1	0.00192	0.0388366462279456	0.0494405063438468	2040	475.583	1564.42	3	18	0.002	0.04	2.98743433
Tt--TRITD0Uv1G042030.1	Aes-CH5S01G228100.1	0.02851	0.0740121482122232	0.38521164647193	1629	363	1266	35.4167	25.5833	0.028	0.07	5.69324217
Tt--TRITD2Av1G270510.6	Aes-CH2S01G510600.1	0.00827	0.0746742164637182	0.110754028654497	3954	914.5	3039.5	25	65	0.008	0.07	5.7441705
Tt--TRITD2Bv1G233690.10	Aes-CH2S01G510600.1	0.001976	0.0277783810997533	0.0711388830397603	3957	916.75	3040.25	6	25	0.002	0.03	2.13679855
Tt--TRITD6Av1G054950.1	Aes-CH6S01G239300.1	0.01196	0.109961698922761	0.108761430418351	1626	361.75	1264.25	15	37	0.012	0.1	8.45859222
Tt--TRITD6Bv1G065890.1	Aes-CH6S01G239300.1	0.006361	0.0167176645225422	0.380471962132943	1626	362.917	1263.08	8	6	0.006	0.02	1.28597419

Tt--TRITD5Av1G205000.3	Aes-CH5S01G459300.1	0.006821	0.0548102751379005	0.1244440469502	2316	548.667	1767.33	12	29	0.007	0.05	4.21617501
Tt--TRITD5Bv1G200940.1	Aes-CH5S01G459300.1	0.00213	0.0289891158257094	0.0734928971921728	2478	597.833	1880.17	4	17	0.002	0.03	2.22993199
Tt--TRITD5Bv1G119650.1	Aes-CH5S01G223800.1	0.00719	0.0482869864807181	0.148901177759489	1299	320.75	978.25	7	15	0.007	0.05	3.71438358
Tt--TRITD0Uv1G047610.1	Aes-CH5S01G223800.1	0.013374	0.0725242061553695	0.184403168413933	1299	318.25	980.75	13	22	0.013	0.07	5.57878509
Tt--TRITD5Av1G112160.1	Aes-CH5S01G171800.1	0.007828	0.0641866136241277	0.121954707796117	1884	471.417	1412.58	11	29	0.008	0.06	4.93743182
Tt--TRITD5Bv1G092620.2	Aes-CH5S01G171800.1	0.003524	0.0367773749447345	0.0958093029027097	1896	473.667	1422.33	5	17	0.004	0.04	2.82902884
Tt--TRITD6Av1G162640.1	Aes-CH6S01G373100.1	0.059463	0.0946611667118609	0.628168446467165	1536	352.333	1183.67	67.6667	31.3333	0.057	0.09	7.28162821
Tt--TRITD6Bv1G148270.1	Aes-CH6S01G373100.1	0.023727	0.0510208930518217	0.465044724753242	1536	351.417	1184.58	27.6667	17.3333	0.023	0.05	3.92468408
Tt--TRITD2Av1G069790.2	Aes-CH2S01G202900.1	0.0064	0.102940134689408	0.0621724350359807	2037	467.833	1569.17	10	45	0.006	0.1	7.9184719
Tt--TRITD2Bv1G079430.3	Aes-CH2S01G202900.1	0.005101	0.0714959540977767	0.0713425693667414	2043	469.25	1573.75	8	32	0.005	0.07	5.49968878
Tt--TRITD2Av1G066770.1	Aes-CH2S01G196500.1	0.079502	0.181517481848183	0.437983440544963	966	234.667	731.333	55.1667	37.8333	0.075	0.16	13.9628832

Tt--TRITD2Bv1G076660.1	Aes-CH2S01G196500.1	0.001369	0.0216153289016188	0.0633168028439896	966	234.667	731.333	1	5	0.001	0.02	1.66271761
Tt--TRITD0Uv1G000510.7	Aes-CH1S01G030400.1	0.016327	0.0961742563439188	0.16976658836637	1761	398.833	1362.17	22	36	0.016	0.09	7.39801972
Tt--TRITD0Uv1G004130.5	Aes-CH1S01G030400.1	0.010736	0.0774966998216712	0.138530061495741	1761	400.667	1360.33	14.5	29.5	0.011	0.07	5.9612846
Tt--TRITD6Av1G073760.2	Aes-CH6S01G267500.1	0.011514	0.0495755651565165	0.232258187977808	1167	291.833	875.167	10	14	0.011	0.05	3.81350501
Tt--TRITD6Av1G073760.2	Aes-CH7S01G572000.1	0.207406	0.567874847945159	0.365231085699519	1098	267.417	830.583	150.5	106.5	0.181	0.4	43.6826806
Tt--TRITD6Av1G073760.2	Aes-CH7S01G534900.1	0.207334	0.593029256307023	0.34961792639015	1098	267.167	830.833	150.5	109.5	0.181	0.41	45.6176351
Tt--TRITD7Av1G209280.1	Aes-CH6S01G267500.1	0.206134	0.591968280834634	0.348217465262988	1098	266.667	831.333	149.833	109.167	0.18	0.41	45.5360216
Tt--TRITD7Av1G209280.1	Aes-CH7S01G534900.1	0	0.0543059821634854	0	1188	286.333	901.667	0	15	0	0.05	4.17738324
Tt--TRITD7Av1G209280.1	Aes-CH7S01G572000.1	0	0.054256852862061	0	1188	286.583	901.417	0	15	0	0.05	4.17360407
Tt--TRITD7Bv1G162110.1	Aes-CH6S01G267500.1	0.206229	0.566539253690104	0.364015138196783	1098	267	831	149.833	106.167	0.18	0.4	43.5799426
Tt--TRITD7Bv1G162110.1	Aes-CH7S01G534900.1	0.00111	0.0393885257125847	0.028188129861152	1188	286.667	901.333	1	11	0.001	0.04	3.02988659

Tt--TRITD7Bv1G162110.1	Aes-CH7S01G572000.1	0.001111	0.0541914852970241	0.020493941856555	1188	286.917	901.083	1	15	0.001	0.05	4.16857579
Tt--TRITD6Bv1G077930.1	Aes-CH6S01G267500.1	0.003413	0.0033975143038125	1.0045506413814	1176	295	881	3	1	0.003	0	0.26134725
Tt--TRITD6Bv1G077930.1	Aes-CH7S01G572000.1	0.204812	0.572673557981767	0.357641109038273	1098	267.583	830.417	148.833	107.167	0.179	0.4	44.0518122
Tt--TRITD6Bv1G077930.1	Aes-CH7S01G534900.1	0.204741	0.597980785901273	0.342386805593968	1098	267.333	830.667	148.833	110.167	0.179	0.41	45.998522
Tt--TRITD1Av1G197990.2	Aes-CH1S01G324900.1	0.010316	0.125211941650912	0.082391539189843	1800	433.583	1366.42	14	50	0.01	0.12	9.63168782
Tt--TRITD1Av1G197990.2	Aes-CH3S01G359100.1	0.274845	1.2543756472696	0.219109387999411	1293	307.25	985.75	226.833	187.167	0.23	0.61	96.4904344
Tt--TRITD1Bv1G187410.2	Aes-CH1S01G324900.1	0.005151	0.109876198997481	0.0468845690315337	1794	430.5	1363.5	7	44	0.005	0.1	8.45201531
Tt--TRITD1Bv1G187410.2	Aes-CH3S01G359100.1	0.272404	1.32648478026529	0.205357453951548	1293	306.5	986.5	225.333	190.667	0.228	0.62	102.037291
Tt--TRITD3Av1G181840.2	Aes-CH1S01G324900.1	0.321732	1.34918169196563	0.238464178165405	1731	409.417	1321.58	345.75	256.25	0.262	0.63	103.783207
Tt--TRITD3Av1G181840.2	Aes-CH3S01G359100.1	0.015966	0.0716861350579924	0.222721049163425	1320	307.167	1012.83	16	21	0.016	0.07	5.51431808
Tt--TRITD3Bv1G162740.2	Aes-CH1S01G324900.1	0.303096	1.29630321663795	0.233815614882939	1713	405.167	1307.83	326.083	249.917	0.249	0.62	99.715632

Tt--TRITD3Bv1G162740.2	Aes-CH3S01G359100.1	0.003951	0.0164210172052585	0.240583538110935	1323	307.833	1015.17	4	5	0.004	0.02	1.26315517
Tt--TRITD6Av1G173820.1	Aes-CH6S01G396400.1	0.053081	0.0845966206090083	0.627465016964092	324	83.3333	240.667	12.3333	6.66667	0.051	0.08	6.50743235
Tt--TRITD6Av1G173820.1	Aes-CH7S01G293100.1	0.221359	0.448766688942792	0.493260373832198	1356	336.083	1019.92	195.5	113.5	0.192	0.34	34.5205145
Tt--TRITD6Av1G173820.1	Aes-CH7S01G292300.1	0.234012	0.489995908361605	0.4775785084182	1386	342.583	1043.42	209.75	123.25	0.201	0.36	37.691993
Tt--TRITD6Bv1G161420.1	Aes-CH6S01G396400.1	0.053081	0.0981512064037792	0.540812710616793	324	83.3333	240.667	12.3333	7.66667	0.051	0.09	7.5500928
Tt--TRITD6Bv1G161420.1	Aes-CH7S01G293100.1	0.169421	0.421975101311121	0.4014962583378	1254	308.833	945.167	143.333	99.6667	0.152	0.32	32.4596232
Tt--TRITD6Bv1G161420.1	Aes-CH7S01G292300.1	0.169388	0.463200269427842	0.365690444618567	1254	308.667	945.333	143.333	106.667	0.152	0.35	35.63079
Tt--TRITD7Av1G083120.2	Aes-CH6S01G396400.1	0.168767	0.37502085796009	0.450020656336791	324	83.5833	240.417	36.3333	24.6667	0.151	0.3	28.8477583
Tt--TRITD7Av1G083120.2	Aes-CH7S01G293100.1	0.033803	0.0867283306824468	0.389758427116124	1413	354.083	1058.92	35	29	0.033	0.08	6.67141005
Tt--TRITD7Av1G083120.2	Aes-CH7S01G292300.1	0.032124	0.0699041085036541	0.45954824254818	1416	353.333	1062.67	33.4167	23.5833	0.031	0.07	5.37723912
Tt--TRITD1Av1G203640.1	Aes-CH1S01G345800.1	0.052485	0.158936909681011	0.33022642471187	2331	581.833	1749.17	88.6667	83.3333	0.051	0.14	12.2259161

Tt--TRITD3Av1G164820.1	Aes-CH1S01G345800.1	0.119795	0.336132169650911	0.356391718216885	1605	382.667	1222.33	135.333	103.667	0.111	0.27	25.8563207
Tt--TRITD3Av1G164820.1	Aes-CH3S01G303500.1	0.030853	0.172646032765129	0.178706943220297	1578	381.5	1196.5	36.1667	58.8333	0.03	0.15	13.2804641
Tt--TRITD3Bv1G142220.1	Aes-CH3S01G303200.1	0.010199	0.0500804044045429	0.203647747629072	2433	557.333	1875.67	19	27	0.01	0.05	3.8523388
Tt--TRITD1Bv1G199190.1	Aes-CH1S01G345800.1	0.122627	0.302985638665569	0.404729441850311	2385	589.083	1795.92	203.167	146.833	0.113	0.25	23.3065876
Tt--TRITD3Av1G164850.1	Aes-CH3S01G303200.1	0.250197	1.56164432419734	0.160213528491737	2337	535.917	1801.08	383.167	351.833	0.213	0.66	120.126486
Tt--TRITD1Bv1G218630.1	Aes-CH1S01G423700.1	0.003988	0.116806960772631	0.0341421886387693	660	157.167	502.833	2	17	0.004	0.11	8.98515083
Tt--TRITD1Bv1G218630.1	Aes-CH3S01G286400.1	0.064815	0.676989250164609	0.095739801509245	654	154.75	499.25	31	69	0.062	0.45	52.0760962
Tt--TRITD3Av1G155690.2	Aes-CH1S01G423700.1	0.067549	0.789155341376171	0.0855969547681668	699	164.917	534.083	34.5	80.5	0.065	0.49	60.704257
Tt--TRITD3Av1G155690.2	Aes-CH3S01G286400.1	0.023523	0.10274396690584	0.228951969644575	990	234.333	755.667	17.5	22.5	0.023	0.1	7.90338207
Tt--TRITD3Bv1G135290.1	Aes-CH1S01G423700.1	0.067549	0.789155341376171	0.0855969547681668	699	164.917	534.083	34.5	80.5	0.065	0.49	60.704257
Tt--TRITD3Bv1G135290.1	Aes-CH3S01G286400.1	0	0.0303948974249952	0	984	235	749	0	7	0	0.03	2.33806903

Tt--TRITD2Av1G084730.2	Aes-CH2S01G225300.1	0.437188	3.00372926185952	0.145548428193269	1908	446.583	1461.42	484.167	328.833	0.331	0.74	231.056097
Tt--TRITD2Bv1G090370.1	Aes-CH2S01G225200.1	0.01405	0.0795852977293504	0.176545642529286	2097	516.5	1580.5	22	39	0.014	0.08	6.12194598
Tt--TRITD7Av1G004690.5	Aes-CH7S01G846700.1	0.077608	0.196655836641592	0.39463647419282	1005	230.75	774.25	57.0833	39.9167	0.074	0.17	15.127372
Tt--TRITD7Av1G004690.5	Aes-CH7S01G847900.1	0.114489	0.356430553297247	0.321211134083509	981	224.417	756.583	80.3333	63.6667	0.106	0.28	27.4177349
Tt--TRITD2Av1G291610.2	Aes-CH2S01G633500.1	0.058331	0.159801748239495	0.365020782994529	1293	301.083	991.917	55.6667	43.3333	0.056	0.14	12.2924422
Tt--TRITD6Av1G000920.4	Aes-CH6S01G007900.1	0.099679	0.181289952665359	0.549832888941937	489	108.667	380.333	35.5	17.5	0.093	0.16	13.945381
Tt--TRITD3Bv1G011530.1	Aes-CH3S01G029900.1	0.077347	0.369654123214705	0.209241298364992	1242	287.25	954.75	70.1667	83.8333	0.073	0.29	28.4349326
Tt--TRITD3Av1G028450.2	Aes-CH3S01G103500.1	0.013197	0.107660269622227	0.122580744872265	1941	488.583	1452.42	19	49	0.013	0.1	8.2815592
Tt--TRITD5Av1G170840.1	Aes-CH5S01G330400.1	0.04464	0.0888767065112392	0.502268851571693	1308	304.25	1003.75	43.5	25.5	0.043	0.08	6.83666973
Tt--TRITD5Av1G199510.5	Aes-CH5S01G438200.1	0.002342	0.110139826373957	0.0212610992027364	1119	263.583	855.417	2	27	0.002	0.1	8.47229434
Tt--TRITD5Av1G202570.2	Aes-CH5S01G446700.1	0.14269	0.292256479781646	0.488235034561334	1053	251.333	801.667	104.167	60.8333	0.13	0.24	22.4812677

Tt--TRITD5Bv1G194390.4	Aes-CH5S01G438200.1	0.002343	0.0969087986100044	0.0241756865100649	1119	264	855	2	24	0.002	0.09	7.45452297
Tt--TRITD5Bv1G198000.2	Aes-CH5S01G446700.1	0.003468	0.0570979485169519	0.0607297852177565	1140	272.833	867.167	3	15	0.003	0.05	4.39214989
Tt--TRITD5Av1G187760.2	Aes-CH5S01G383000.1	0.005869	0.122268780825224	0.0480014342340689	1785	416.583	1368.42	8	47	0.006	0.11	9.40529083
Tt--TRITD5Bv1G177650.5	Aes-CH5S01G383000.1	0.002185	0.0779105392690779	0.0280432572423906	1794	418.917	1375.08	3	31	0.002	0.07	5.99311841
Tt--TRITD5Bv1G125410.2	Aes-CH5S01G234600.1	0.165494	0.213148885020312	0.776423733139837	1167	315.75	851.25	126.417	58.5833	0.149	0.19	16.3960681
Tt--TRITD0Uv1G046260.2	Aes-CH5S01G234600.1	0.162903	0.237969956799587	0.684553246209904	1155	311	844	123.583	63.4167	0.146	0.2	18.3053813
Tt--TRITD5Av1G229390.2	Aes-CH5S01G579200.1	0.004247	0.0704329495874262	0.0602991539710432	1242	297.5	944.5	4	20	0.004	0.07	5.4179192
Tt--TRITD5Bv1G228940.2	Aes-CH5S01G579200.1	0.004243	0.058989929712129	0.0719324944206817	1245	299.667	945.333	4	17	0.004	0.06	4.5376869
Tt--TRITD6Av1G059560.3	Aes-CH6S01G246400.1	0.002079	0.0875309117576116	0.0237475337883332	1254	290.5	963.5	2	24	0.002	0.08	6.73314706
Tt--TRITD6Bv1G068340.3	Aes-CH6S01G246400.1	0	0.0316576890012354	0	1254	290.333	963.667	0	9	0	0.03	2.43520685
Tt--TRITD7Av1G255570.2	Aes-CH6S01G246400.1	0.089329	0.726972633199035	0.122877910390838	1254	292.167	961.833	81	136	0.084	0.47	55.9209718

Tt--TRITD7Bv1G205360.3	Aes-CH6S01G246400.1	0.089948	0.85805599705865	0.104827446457087		1254	292.5	961.5	81.5	149.5	0.085	0.51	66.0043075
Tt--TRITD7Av1G173340.3	Aes-CH7S01G417300.1	0.016078	0.0314755425123037	0.510795489868722		1599	373.083	1225.92	19.5	11.5	0.016	0.03	2.42119558
Tt--TRITD7Bv1G130380.4	Aes-CH7S01G417300.1	0.00782	0.0232153263596053	0.336849608321048		1593	371.833	1221.17	9.5	8.5	0.008	0.02	1.78579434
Tt--TRITD1Av1G170240.2	Aes-CH1S01G249300.1	0.079011	0.164270367467962	0.480982484841285		2022	515.167	1506.83	113	76	0.075	0.15	12.6361821
Tt--TRITD1Bv1G156400.1	Aes-CH1S01G249300.1	0.066631	0.136692609763071	0.487454073676192		2031	514.833	1516.17	96.6667	64.3333	0.064	0.12	10.5148161
Tt--TRITD3Av1G011010.3	Aes-CH3S01G024500.1	0.020725	0.112336048214884	0.184494265450723		1155	290.75	864.25	17.6667	30.3333	0.02	0.1	8.64123448
Tt--TRITD3Bv1G009870.4	Aes-CH3S01G024500.1	0.022885	0.117412175920595	0.194908431144324		1155	289.833	865.167	19.5	31.5	0.023	0.11	9.03170584
Tt--TRITD7Av1G006960.1	Aes-CH7S01G856400.1	0.015368	0.0511688378479657	0.300339457224188		1977	465	1512	23	23	0.015	0.05	3.93606445
Tt--TRITD3Av1G001260.2	Aes-CH3S01G011500.1	0.048228	0.130521723808777	0.369503022911355		2301	554.417	1746.58	81.5833	66.4167	0.047	0.12	10.0401326
Tt--TRITD2Av1G083210.1	Aes-CH2S01G221400.1	0.224394	1.89681134612709	0.118300459412427		552	136.917	415.083	80.5	94.5	0.194	0.69	145.908565
Tt--TRITD4Bv1G170030.2	Aes-CH4S01G415500.1	0.013459	0.0746811837582654	0.180213488120449		2034	534.583	1499.42	20	38	0.013	0.07	5.74470644

Tt--TRITD4Bv1G170030.2	Aes-CH4S01G404900.1	0.015802	0.0919972787562797	0.171761161384436	2040	537.083	1502.92	23.5	46.5	0.016	0.09	7.07671375
Tt--TRITD3Av1G202040.8	Aes-CH3S01G393100.1	0.017683	0.0926017809171765	0.190962140179784	2424	554.833	1869.17	32.6667	48.3333	0.017	0.09	7.12321392
Tt--TRITD3Bv1G178340.3	Aes-CH3S01G393100.1	0.052979	0.106234123237836	0.498699954773756	1617	360.917	1256.08	64.25	35.75	0.051	0.1	8.17185563
Tt--TRITD6Av1G085990.3	Aes-CH6S01G288200.1	0.012091	0.0473776098543071	0.25521192018948	2580	620.75	1959.25	23.5	28.5	0.012	0.05	3.64443153
Tt--TRITD6Bv1G090340.3	Aes-CH6S01G288200.1	0.008732	0.032872969220032	0.265635503836382	2580	621.833	1958.17	17	20	0.009	0.03	2.52868994
Tt--TRITD1Av1G000530.1	Aes-CH1S01G005100.1	0.011445	0.179100270476067	0.0639013751181213	1077	255.25	821.75	9.33333	40.6667	0.011	0.16	13.7769439
Tt--TRITD5Av1G045620.11	Aes-CH5S01G108100.1	0.025412	0.109302673120492	0.232492578738636	2202	521.083	1680.92	42	53	0.025	0.1	8.40789793
Tt--TRITD5Bv1G043010.13	Aes-CH5S01G108100.1	0.026749	0.0868843829832222	0.307873770895023	2205	521.083	1683.92	44.25	42.75	0.026	0.08	6.68341408
Tt--TRITD4Av1G134780.6	Aes-CH4S01G202500.1	0.01012	0.0379196153814435	0.266885754058882	2205	513.833	1691.17	17	19	0.01	0.04	2.91689349
Tt--TRITD4Av1G134780.6	Aes-CH5S01G108100.1	0.244969	0.968594800616026	0.252911662025259	2163	504.583	1658.42	346.583	274.417	0.209	0.54	74.5072924
Tt--TRITD2Av1G084500.2	Aes-CH2S01G224000.1	0.034953	0.106149161901035	0.329283519483	1998	490	1508	51.5	48.5	0.034	0.1	8.16532015

Tt--TRITD2Bv1G089940.2	Aes-CH2S01G224000.1	0.010022	0.0206375875560002	0.485612129089382	1998	491.25	1506.75	15	10	0.01	0.02	1.58750674
Tt--TRITD3Av1G032230.2	Aes-CH3S01G115500.1	0.017055	0.0948894656543	0.179735425310561	1731	426.333	1304.67	22	38	0.017	0.09	7.29918967
Tt--TRITD3Bv1G039450.3	Aes-CH3S01G115500.1	0.00925	0.0688302547185429	0.134395180687933	1731	425.75	1305.25	12	28	0.009	0.07	5.29463498
Tt--TRITD7Av1G192070.4	Aes-CH7S01G509000.1	0.046324	0.105046765345887	0.440987838379926	3066	758.333	2307.67	103.667	74.3333	0.045	0.1	8.08052041
Tt--TRITD7Av1G192070.4	Aes-CH7S01G458800.1	0.039171	0.10919260571489	0.358733572120284	2649	644.583	2004.42	76.5	65.5	0.038	0.1	8.39943121
Tt--TRITD7Bv1G152370.2	Aes-CH7S01G509000.1	0.012666	0.0491252062355354	0.257838329358305	3066	757.083	2308.92	29	36	0.013	0.05	3.77886202
Tt--TRITD7Bv1G152370.2	Aes-CH7S01G458800.1	0.009518	0.0446346831567349	0.213250557218117	2655	646.167	2008.83	19	28	0.009	0.04	3.43343717
Tt--TRITD2Av1G095350.5	Aes-CH2S01G234400.1	0.094022	0.182411273964383	0.515441047626232	558	131.75	426.25	37.6667	21.3333	0.088	0.16	14.0316365
Tt--TRITD2Bv1G096060.3	Aes-CH2S01G234400.1	0.094022	0.14465756334155	0.649964343233419	558	131.75	426.25	37.6667	17.3333	0.088	0.13	11.1275049
Tt--TRITD4Bv1G207930.11	Aes-CH4S01G568500.1	0.007702	0.0683432012778051	0.112699578510867	2217	520.5	1696.5	13	34	0.008	0.07	5.25716933
Tt--TRITD5Av1G258690.15	Aes-CH4S01G568500.1	0.009345	0.0455528752698572	0.205156001408206	2178	509.083	1668.92	15.5	22.5	0.009	0.04	3.50406733

Tt--TRITD6Av1G209030.4	Aes-CH6S01G495000.1	0.003211	0.0420541646048345	0.0763571660392743	2025	464.583	1560.42	5	19	0.003	0.04	3.23493574
Tt--TRITD6Bv1G205270.3	Aes-CH6S01G495000.1	0.001283	0.0240633108479577	0.0533067467454556	2025	464.5	1560.5	2	11	0.001	0.02	1.85102391
Tt--TRITD7Av1G084620.26	Aes-CH7S01G296700.1	0.02818	0.0986309890251347	0.285709848995147	2067	476.083	1590.92	44	44	0.028	0.09	7.58699916
Tt--TRITD7Av1G084620.26	Aes-CH7S01G268600.1	0.03355	0.0943993052449784	0.355401616577939	2121	490.417	1630.58	53.5	43.5	0.033	0.09	7.26148502
Tt--TRITD7Bv1G059260.26	Aes-CH7S01G296700.1	0.022324	0.0868116356111995	0.257157498518642	2067	475.75	1591.25	35	39	0.022	0.08	6.67781812
Tt--TRITD7Bv1G059260.26	Aes-CH7S01G268600.1	0.029068	0.0829842524189104	0.350279129930066	2121	490.083	1630.92	46.5	38.5	0.029	0.08	6.38340403
												Average=24.4088924598461

The Ka/Ks ratio and divergence times between the genes of *A.speltoides* and *T.urartu*													
**Gene ID**	**Gene ID**	**Ka**	**Ks**	**Ka/Ks**	**EffectiveLen**	**AverageS-sites**	**AverageN-sites**	**cN**	**cS**	**pN**	**pS**	**Divergence times (Mya)**
Aes-CH4S01G275300.1	Tu--TuG1812G0400002351.01.T01	0.0011783	0.0819367870481388	0.0143808320594586	1107	257.66667	849.33333	1	20	0.001	0.078	6.30282977
Aes-CH1S01G121300.1	Tu--TuG1812G0100001066.01.T01	0.0039759	0.0762734268698046	0.0521267909096672	1647	386.08333	1260.9167	5	28	0.004	0.073	5.86718668
Aes-CH1S01G422500.1	Tu--TuG1812G0100004520.01.T02	0.0169465	0.0452053417446256	0.374877251480609	1818	425.5	1392.5	23.33	18.67	0.017	0.044	3.47733398
Aes-CH1S01G422500.1	Tu--TuG1812G0300002745.01.T01	0.1306509	1.30230703060867	0.100322621026937	1440	328	1112	133.3	202.7	0.12	0.618	100.177464
Aes-CH3S01G287600.1	Tu--TuG1812G0300002745.01.T01	0.0150799	0.0646993644273757	0.233077163374837	1212	274.25	937.75	14	17	0.015	0.062	4.97687419
Aes-CH7S01G477100.1	Tu--TuG1812G0700003597.01.T01	0.0041544	0.0469750134988024	0.0884388468198459	1251	285.5	965.5	4	13	0.004	0.046	3.61346258
Aes-CH1S01G431400.1	Tu--TuG1812G0100004629.01.T01	0.0081272	0.0985425040468546	0.0824736244558751	1794	433.16667	1360.8333	11	40	0.008	0.092	7.58019262

Aes-CH3S01G281200.1	Tu--TuG1812G0100004629.01.T01	0.1574447	1.0309130593167	0.152723517071063		1767	420.91667	1346.0833	191.2	235.8	0.142	0.56	79.3010046	
Aes-CH3S01G281200.1	Tu--TuG1812G0300002551.01.T01	0.0108758	0.0619691682854432	0.17550369512793		1839	434.33333	1404.6667	15.17	25.83	0.011	0.059	4.7668591	
Aes-CH7S01G615700.1	Tu--TuG1812G0700004566.01.T01	0.0468564	0.230698036614541	0.203107016419973		1248	295.83333	952.16667	43.25	58.75	0.045	0.199	17.7460028	
Aes-CH7S01G592100.1	Tu--TuG1812G0700004566.01.T01	0.0480403	0.180890668191215	0.265576413189793		1248	297.08333	950.91667	44.25	47.75	0.047	0.161	13.9146668	
Aes-CH6S01G225500.1	Tu--TuG1812G0600001016.01.T02	0.0297773	0.0487616299489188	0.610670272798829		1497	349.5	1147.5	33.5	16.5	0.029	0.047	3.75089461	
Aes-CH7S01G583200.1	Tu--TuG1812G0700004443.01.T01	0.0053	0.0992967804246585	0.0533757586321119		1734	408.58333	1325.4167	7	38	0.005	0.093	7.63821388	
Aes-CH7S01G560300.1	Tu--TuG1812G0700004443.01.T01	0.0030244	0.0936888934181125	0.0322811817364989		1734	408.75	1325.25	4	36	0.003	0.088	7.20683796	
Aes-CH6S01G486800.1	Tu--TuG1812G0600003691.01.T01	0.1621645	0.257053184937537	0.63085988150873		1059	286.41667	772.58333	112.7	62.33	0.146	0.218	19.7733219	

Aes-CH6S01G486800.1	Tu--TuG1812G0700001616.01.T01	0.2789343	0.545030532789184	0.511777409949768	1002	269.33333	732.66667	170.7	104.3	0.233	0.387	41.9254256
Aes-CH7S01G128000.1	Tu--TuG1812G0700001616.01.T01	0.0013149	0.0880551203114362	0.0149329729380583	1038	276.83333	761.16667	1	23	0.001	0.083	6.77347079
Aes-CH4S01G066000.1	Tu--TuG1812G0400000463.01.T01	0.2343334	0.537286601069677	0.436142323385515	984	268.5	715.5	144	103	0.201	0.384	41.3297385
Aes-CH2S01G410800.1	Tu--TuG1812G0600002720.01.T01	0.1537231	0.854579852023839	0.17988146200477	2616	629.08333	1986.9167	276.2	320.8	0.139	0.51	65.7369117
Aes-CH6S01G361000.1	Tu--TuG1812G0600002720.01.T01	0.0065365	0.0436663323580672	0.149692828654531	2634	636.5	1997.5	13	27	0.007	0.042	3.35894864
Aes-CH3S01G320300.1	Tu--TuG1812G0100004071.01.T01	0.29772	0.6120213011709	0.486453640276547	1191	303.16667	887.83333	218.2	126.8	0.246	0.418	47.0785616
Aes-CH3S01G320300.1	Tu--TuG1812G0300003030.01.T01	0.311075	0.678576320758312	0.458422961761321	1185	298.41667	886.58333	225.8	133.3	0.255	0.447	52.1981785
Aes-CH1S01G358800.1	Tu--TuG1812G0100004071.01.T01	0.0409964	0.119134409751092	0.344118762660927	1458	367.66667	1090.3333	43.5	40.5	0.04	0.11	9.16418537

Aes-CH1S01G358800.1	Tu--TuG1812G0300003030.01.T01	0.3148171	0.78074362539595	0.403227278288915	1260	320.33333	939.66667	241.6	155.4	0.257	0.485	60.057202
Aes-CH5S01G571400.1	Tu--TuG1812G0500004830.01.T01	0.000813	0.0824693581318243	0.00985830650451029	1602	371.33333	1230.6667	1	29	0.001	0.078	6.34379678
Aes-CH5S01G228100.1	Tu--TuG1812G0500002311.01.T01	0.0038351	0.0364748313303763	0.105143719656632	2046	477.5	1568.5	6	17	0.004	0.036	2.80575626
Aes-CH2S01G510600.1	Tu--TuG1812G0200005431.01.T03	0.0072812	0.0698311478482388	0.104268784888706	3951	914.83333	3036.1667	22	61	0.007	0.067	5.37162676
Aes-CH5S01G223800.1	Tu--TuG1812G0500002254.01.T01	0.0123619	0.0658497822801345	0.187728121938305	1296	317.25	978.75	12	20	0.012	0.063	5.06536787
Aes-CH5S01G171800.1	Tu--TuG1812G0500001767.01.T01	0.0063985	0.0618793631984733	0.103403167077156	1884	471.41667	1412.5833	9	28	0.006	0.059	4.75995102
Aes-CH2S01G202900.1	Tu--TuG1812G0200002096.01.T01	0.4406804	0.831022624873256	0.530286859009922	822	195.58333	626.41667	208.8	98.25	0.333	0.502	63.9248173
Aes-CH2S01G196500.1	Tu--TuG1812G0200002096.01.T01	0.0130932	0.0693230556785387	0.188872895819973	966	234.08333	731.91667	9.5	15.5	0.013	0.066	5.33254274

Aes-CH6S01G267500.1	Tu--TuG1812G0600001715.01.T01	0.0092008	0.0495170953733576	0.185810685625442	1167	292.16667	874.83333	8	14	0.009	0.048	3.80900734
Aes-CH6S01G267500.1	Tu--TuG1812G0700004249.01.T01	0.2061337	0.583756519967381	0.353115874885377	1098	266.66667	831.33333	149.8	108.2	0.18	0.406	44.9043477
Aes-CH7S01G572000.1	Tu--TuG1812G0700004249.01.T01	0	0.0467917834558258	0	1188	286.58333	901.41667	0	13	0	0.045	3.59936796
Aes-CH1S01G324900.1	Tu--TuG1812G0100003823.01.T01	0.0159659	0.129501396370546	0.123287855603543	1794	433	1361	21.5	51.5	0.016	0.119	9.96164587
Aes-CH3S01G359100.1	Tu--TuG1812G0100003823.01.T01	0.277234	1.3653680454514	0.203047109000297	1284	306	978	226.7	192.3	0.232	0.629	105.028311
Aes-CH1S01G345800.1	Tu--TuG1812G0100003984.01.T01	0.0721759	0.278953321268972	0.25873844532606	2385	590.25	1794.75	123.5	137.5	0.069	0.233	21.4579478
Aes-CH1S01G345800.1	Tu--TuG1812G0100003933.01.T01	0.0353006	0.122096350832699	0.289121115436606	303	71	232	8	8	0.034	0.113	9.39202699
Aes-CH3S01G303200.1	Tu--TuG1812G0300002902.01.T01	0.251813	1.53109020096301	0.164466490426571	2337	535.91667	1801.0833	385.3	349.8	0.214	0.653	117.776169

Aes-CH1S01G423700.1	Tu--TuG1812G0100004544.01.T01	0.0018634	0.166240206114792	0.0112088106679786	705	167.66667	537.33333	1	25	0.002	0.149	12.7877082
Aes-CH1S01G423700.1	Tu--TuG1812G0300002735.01.T01	0.0675493	0.789155341376171	0.0855969547681668	699	164.91667	534.08333	34.5	80.5	0.065	0.488	60.704257
Aes-CH2S01G225200.1	Tu--TuG1812G0200002657.01.T01	0.4053403	0.912481096235994	0.444217731974035	1929	465.58333	1463.4167	458.3	245.8	0.313	0.528	70.1908536
Aes-CH2S01G225200.1	Tu--TuG1812G0200002474.01.T01	0.3549384	NaN	NaN	1407	327.5	1079.5	305.3	270.7	0.283	0.827	High Sequence Divergence Value (pS>=0.75)
Aes-CH7S01G846700.1	Tu--TuG1812G0700000286.01.T01	0.1121885	0.413743832750576	0.271154608185072	462	106.91667	355.08333	37	34	0.104	0.318	31.8264487
Aes-CH7S01G847900.1	Tu--TuG1812G0700000286.01.T01	0.1326304	0.489010644309415	0.271221954960689	465	107.16667	357.83333	43.5	38.5	0.122	0.359	37.6162034
Aes-CH2S01G633500.1	Tu--TuG1812G0200006101.01.T01	0.0086957	0.0941251378043485	0.0923849865034429	1668	395.66667	1272.3333	11	35	0.009	0.088	7.24039522
Aes-CH3S01G029900.1	Tu--TuG1812G0300000417.01.T01	0.0242467	0.173851210542993	0.139467912019651	1254	290	964	23	45	0.024	0.155	13.37317

Aes-CH3S01G103500.1	Tu--TuG1812G0300001074.01.T02	0.0082367	0.104157408587433	0.0790793319101409	1785	442.16667	1342.8333	11	43	0.008	0.097	8.01210835
Aes-CH5S01G330400.1	Tu--TuG1812G0500003267.01.T02	0.0303455	0.0730665847556012	0.415312680741179	696	158	538	16	11	0.03	0.07	5.62050652
Aes-CH5S01G383000.1	Tu--TuG1812G0500003673.01.T01	0.0058691	0.122268780825224	0.0480014342340689	1785	416.58333	1368.4167	8	47	0.006	0.113	9.40529083
Aes-CH5S01G234600.1	Tu--TuG1812G0500002335.01.T01	0.0135373	0.0779922851430357	0.1735727300046	1320	351	969	13	26	0.013	0.074	5.99940655
Aes-CH5S01G579200.1	Tu--TuG1812G0500004860.01.T01	0.0073762	0.0697366111169322	0.105772918938188	1254	300.33333	953.66667	7	20	0.007	0.067	5.3643547
Aes-CH6S01G246400.1	Tu--TuG1812G0600001625.01.T01	0.0010386	0.0836723823466833	0.0124127159646731	1254	290.5	963.5	1	23	0.001	0.079	6.4363371
Aes-CH7S01G856400.1	Tu--TuG1812G0700000347.01.T01	0.0169365	0.0583471668574607	0.29027076423613	1956	463.16667	1492.8333	25	26	0.017	0.056	4.4882436
Aes-CH1S01G005100.1	Tu--TuG1812G0100000037.01.T01	0.0323787	0.18273948209003 7	0.177184994745305	1911	501.5	1409.5	44.67	81.33	0.032	0.162	14.0568832

Aes-CH5S01G108100.1	Tu--TuG1812G0500000997.01.T01	0.0069832	0.0896439612271905	0.0778995355442498	2259	532.58333	1726.4167	12	45	0.007	0.084	6.89568933
Aes-CH2S01G224000.1	Tu--TuG1812G0200002486.01.T01	0.0342704	0.103688266402051	0.330513303772615	1998	490.5	1507.5	50.5	47.5	0.033	0.097	7.97602049
Aes-CH3S01G115500.1	Tu--TuG1812G0300001179.01.T01	0.0135383	0.0988104531232662	0.137012566041847	1731	426.66667	1304.3333	17.5	39.5	0.013	0.093	7.60080409
Aes-CH7S01G509000.1	Tu--TuG1812G0700003889.01.T01	0.0458635	0.0990034222564522	0.463252067661587	3066	758.33333	2307.6667	102.7	70.33	0.044	0.093	7.61564787
Aes-CH7S01G458800.1	Tu--TuG1812G0700003889.01.T01	0.0386456	0.102048631473971	0.378697782157535	2649	644.58333	2004.4167	75.5	61.5	0.038	0.095	7.84989473
Aes-CH2S01G234400.1	Tu--TuG1812G0200002891.01.T01	0.0962172	0.164552423773864	0.584720380915915	327	77.833333	249.16667	22.5	11.5	0.09	0.148	12.6578788
Aes-CH4S01G568500.1	Tu--TuG1812G0500005695.01.T01	0.0095136	0.0480644914227494	0.197933132886887	2208	515.5	1692.5	16	24	0.009	0.047	3.69726857
Aes-CH6S01G495000.1	Tu--TuG1812G0600003785.01.T06	0.0025682	0.0420309009556518	0.0611031586212621	2025	464.83333	1560.1667	4	19	0.003	0.041	3.23314623
												Average=22.1596285155407

The Ka/Ks ratio and divergence times between the genes of *T.urartu* and *A.tauschii*												
**Gene ID**	**Gene ID**	**Ka**	**Ks**	**Ka/Ks**	**Effective Len**	**AverageS-sites**	**Average N-sites**	**cN**	**cS**	**pN**	**pS**	**Divergence times (Mya)**
Tu--TuG1812G0400002351.01.T01	Aet-:AET4Gv20512600.2	0	0.0689208432992557	0	1107	258.16667	848.833	0	17	0	0.0658	5.30160333
Tu--TuG1812G0100001066.01.T01	Aet-:AET1Gv20214200.10	0.0048	0.0935276088 75718	0.0510631972220011	1647	386.66667	1260.33	6	34	0.0048	0.0879	7.19443145
Tu--TuG1812G0100004520.01.T02	Aet-:AET1Gv20987400.1	0.017	0.0377546106718633	0.450964061209805	1725	398.33333	1326.67	22.333	14.667	0.0168	0.0368	2.90420082
Tu--TuG1812G0100004520.01.T02	Aet-:AET3Gv20567900.18	0.1255	1.26283408254075	0.0993922257548571	1440	330.33333	1109.67	128.25	201.75	0.1156	0.6107	97.1410833
Tu--TuG1812G0300002745.01.T01	Aet-:AET1Gv20987400.1	0.1309	1.25327918270647	0.104463539926869	1440	327.33333	1112.67	133.67	199.33	0.1201	0.609	96.406091
Tu--TuG1812G0300002745.01.T01	Aet-:AET3Gv20567900.18	0.0363	0.0858754406979184	0.422546403567249	1458	335.83333	1122.17	39.75	27.25	0.0354	0.0811	6.60580313
Tu--TuG1812G0700003597.01.T01	Aet-:AET7Gv20853400.1	0.0073	0.043229251154466	0.168558641312925	1251	285.66667	965.333	7	12	0.0073	0.042	3.32532701
Tu--TuG1812G0100004629.01.T01	Aet-:AET1Gv20998400.2	0.0089	0.126082259559629	0.0703885638903304	1791	430.83333	1360.17	12	50	0.0088	0.1161	9.69863535
Tu--TuG1812G0100004629.01.T01	Aet-:AET3Gv20558100.1	0.1485	1.01440376366681	0.146406949757957	1767	419.91667	1347.08	181.5	233.5	0.1347	0.5561	78.0310587
Tu--TuG1812G0300002551.01.T01	Aet-:AET3Gv20558100.1	0.0014	0.0427330760569053	0.0333269650444869	1839	433.33333	1405.67	2	18	0.0014	0.0415	3.2871597

Tu--TuG1812G0700004566.01.T01	Aet-:AET7Gv21020800.4	0.0029	0.0721214136778505	0.0407410143095772	1107	254.5	852.5	2.5	17.5	0.0029	0.0688	5.54780105
Tu--TuG1812G0600001016.01.T02	Aet-:AET6Gv20256900.11	0.0114	0.0534880057367498	0.21270402880801	1500	348.66667	1151.33	13	18	0.0113	0.0516	4.11446198
Tu--TuG1812G0700004443.01.T01	Aet-:AET7Gv20996900.4	0.0045	0.0689994663193708	0.0658560759696207	1734	409.58333	1324.42	6	27	0.0045	0.0659	5.30765126
Tu--TuG1812G0600003691.01.T01	Aet-:AET6Gv20859000.1	0.0096	0.0812125508979431	0.117994177104405	1008	272.83333	735.167	7	21	0.0095	0.077	6.2471193
Tu--TuG1812G0700001616.01.T01	Aet-:AET7Gv20388900.1	0.0229	0.144541788542599	0.158770153659166	1026	273.83333	752.167	17	36	0.0226	0.1315	11.1185991
Tu--TuG1812G0400000463.01.T01	Aet-:AET4Gv20097400.1	0.2413	0.531471610109033	0.45393406287235	984	269.41667	714.583	147.42	102.58	0.2063	0.3808	40.8824315
Tu--TuG1812G0600002720.01.T01	Aet-:AET2Gv20903900.5	0.1538	0.81663532615612	0.188295525832228	2616	628.41667	1987.58	276.33	312.67	0.139	0.4975	62.818102
Tu--TuG1812G0600002720.01.T01	Aet-:AET6Gv20637800.1	0.0065	0.0337496682818668	0.193660839041328	2634	636.33333	1997.67	13	21	0.0065	0.033	2.59612833
Tu--TuG1812G0100004071.01.T01	Aet-:AET1Gv20883200.1	0.027	0.117426795518655	0.230042598326359	1461	368	1093	29	40	0.0265	0.1087	9.03283042
Tu--TuG1812G0100004071.01.T01	Aet-:AET3Gv20622200.1	0.3013	0.650759760758272	0.462975095537248	1260	317.58333	942.417	233.83	138.17	0.2481	0.4351	50.0584431
Tu--TuG1812G0300003030.01.T01	Aet-:AET1Gv20883200.1	0.3035	0.71738402605807	0.422999337790689	1230	311.08333	918.917	229.33	143.67	0.2496	0.4618	55.1833866

Tu--TuG1812G0300003030.01.T01	Aet-:AET3Gv20622200.1	0.2948	0.688156918212449	0.428428371489293	1194	299.75	894.25	218	135	0.2438	0.4504	52.9351476
Tu--TuG1812G0500004830.01.T01	Aet-:AET5Gv21075800.4	0.0033	0.0705074951700408	0.0462046801684237	1602	371.5	1230.5	4	25	0.0033	0.0673	5.42365347
Tu--TuG1812G0500002311.01.T01	Aet-:AET5Gv20490900.10	0.0026	0.0301873302205371	0.085203762352627	2031	473.16667	1557.83	4	14	0.0026	0.0296	2.32210232
Tu--TuG1812G0200005431.01.T03	Aet-:AET2Gv21094700.6	0.0119	0.073152065664957	0.162756801087456	3966	918.25	3047.75	36	64	0.0118	0.0697	5.62708197
Tu--TuG1812G0500002254.01.T01	Aet-:AET5Gv20485300.2	0.0147	0.086446553978471	0.169534100202832	1551	379.66667	1171.33	17	31	0.0145	0.0817	6.64973492
Tu--TuG1812G0500001767.01.T01	Aet-:AET5Gv20380700.1	0.0092	0.04295322878838	0.213102296440828	1908	479.08333	1428.92	13	20	0.0091	0.0417	3.30409452
Tu--TuG1812G0200002096.01.T01	Aet-:AET2Gv20411300.2	0.4684	0.776603063734958	0.603115062118616	945	227.58333	717.417	249.92	110.08	0.3484	0.4837	59.7386972
Tu--TuG1812G0200002096.01.T01	Aet-:AET2Gv20397600.3	0.0109	0.0625277380608518	0.173582970034577	879	213.91667	665.083	7.1667	12.833	0.0108	0.06	4.809826
Tu--TuG1812G0600001715.01.T01	Aet-:AET6Gv20435900.1	0.0506	0.11468206723452	0.441185946972528	1107	275.83333	831.167	40.667	29.333	0.0489	0.1063	8.82169748
Tu--TuG1812G0700004249.01.T01	Aet-:AET6Gv20435900.1	0.228	0.719696062505103	0.316868129660233	1116	272.66667	843.333	165.83	126.17	0.1966	0.4627	55.3612356
Tu--TuG1812G0100003823.01.T01	Aet-:AET1Gv20814900.1	0.0126	0.12030733072903	0.104608084987805	1794	431.83333	1362.17	17	48	0.0125	0.1112	9.25441006

Tu--TuG1812G0100003823.01.T01	Aet-:AET3Gv20654200.3	0.3323	1.32564508688524	0.250669932744008	1731	411.08333	1319.92	354.33	255.67	0.2685	0.6219	101.972699
Tu--TuG1812G0100003984.01.T01	Aet-:AET1Gv20861500.1	0.2889	0.520595450298151	0.555010591037392	1179	311.91667	867.083	207.92	117.08	0.2398	0.3754	40.0458039
Tu--TuG1812G0300002902.01.T01	Aet-:AET3Gv20596900.1	0.2492	1.424770224899	0.174885440119388	2337	536.75	1800.25	381.67	342.33	0.212	0.6378	109.59771
Tu--TuG1812G0100004544.01.T01	Aet-:AET1Gv20988700.4	0.0035	0.157766368269016	0.022373363685757	1119	267.08333	851.917	3	38	0.0035	0.1423	12.1358745
Tu--TuG1812G0100004544.01.T01	Aet-:AET3Gv20565300.1	0.1068	0.758017187858094	0.140858427551491	1119	266.58333	852.417	84.833	127.17	0.0995	0.477	58.3090145
Tu--TuG1812G0300002735.01.T01	Aet-:AET1Gv20988700.4	0.0957	0.673627687173771	0.142036339268602	1113	265.08333	847.917	76.167	117.83	0.0898	0.4445	51.8175144
Tu--TuG1812G0200002657.01.T01	Aet-:AET2Gv20463700.1	0.2872	NaN	NaN	990	226.25	763.75	182.25	189.75	0.2386	0.8387	High Sequence Divergence Value (pS>=0.75)
Tu--TuG1812G0200002657.01.T01	Aet-:AET2Gv20462800.7	0.4162	0.953097440882319	0.436717095386415	1941	467.83333	1473.17	470.58	252.42	0.3194	0.5395	73.3151878
Tu--TuG1812G0200002474.01.T01	Aet-:AET2Gv20462900.1	0.3861	NaN	NaN	1443	331.91667	1111.08	335.33	260.67	0.3018	0.7853	High Sequence Divergence Value (pS>=0.75)
Tu--TuG1812G0200006101.01.T01	Aet-:AET2Gv21265400.2	0.0714	0.149238265816286	0.478733567325328	1536	362.08333	1173.92	80	49	0.0681	0.1353	11.4798666

Tu--TuG1812G0300001074.01.T02	Aet-:AET3Gv20208200.4	0.0105	0.0515929085464107	0.203354434926737	1785	441.25	1343.75	14	22	0.0104	0.0499	3.96868527
Tu--TuG1812G0500003267.01.T02	Aet-:AET5Gv20680900.1	0.0437	0.134231405074957	0.325265902887181	198	44.75	153.25	6.5	5.5	0.0424	0.1229	10.3254927
Tu--TuG1812G0500003673.01.T01	Aet-:AET5Gv20774000.3	0.0044	0.0758220876535267	0.0579727769805434	1785	416	1369	6	30	0.0044	0.0721	5.83246828
Tu--TuG1812G0500002335.01.T01	Aet-:AET5Gv20505600.1	0.0104	0.0561700471395463	0.185018110220011	1320	351.08333	968.917	10	19	0.0103	0.0541	4.32077286
Tu--TuG1812G0500004860.01.T01	Aet-:AET5Gv21088300.4	0.0128	0.0560026828092803	0.228566026851822	1242	296.5	945.5	12	16	0.0127	0.054	4.30789868
Tu--TuG1812G0600001625.01.T01	Aet-:AET6Gv20396400.1	0.001	0.0645173681433777	0.0161091683246037	1254	291.16667	962.833	1	18	0.001	0.0618	4.96287447
Tu--TuG1812G0600001625.01.T01	Aet-:AET7Gv21189400.1	0.0882	0.77234281551536	0.114175885447156	1254	292.41667	961.583	80	141	0.0832	0.4822	59.4109858
Tu--TuG1812G0700000347.01.T01	Aet-:AET7Gv20063100.2	0.0128	0.0652830325380597	0.196720184341483	1956	463.83333	1492.17	19	29	0.0127	0.0625	5.02177173
Tu--TuG1812G0100000037.01.T01	Aet-:AET1Gv20001400.6	0.0199	0.221015774276258	0.0898804075221016	1185	283.83333	901.167	17.667	54.333	0.0196	0.1914	17.0012134
Tu--TuG1812G0500000997.01.T01	Aet-:AET5Gv20234400.6	0.0058	0.0366043750210879	0.158786837991951	2259	531.83333	1727.17	10	19	0.0058	0.0357	2.81572116
Tu--TuG1812G0300001179.01.T01	Aet-:AET3Gv20232400.2	0.0198	0.077639544852257	0.255230186457891	1731	427.08333	1303.92	25.5	31.5	0.0196	0.0738	5.97227268

Tu--TuG1812G0700003889.01.T01	Aet-:AET7Gv20906800.1	0.022	0.0660140212071353	0.332941355640537		3099	767.5	2331.5	50.5	48.5	0.0217	0.0632	5.07800163
Tu--TuG1812G0500005695.01.T01	Aet-:AET4Gv20885100.12	0.0053	0.0481851575604212	0.110081205880307		2217	514.25	1702.75	9	24	0.0053	0.0467	3.70655058
Tu--TuG1812G0600003785.01.T06	Aet-:AET6Gv20872100.4	0.0019	0.0378208023040004	0.0508769919960789		2022	460.91667	1561.08	3	17	0.0019	0.0369	2.90929248
													Average=25.3955129918934

Table S3 The cis-regulatory element of the MAPK-MAP4K cascade gene promoter region in wheat

	Name						Function
TaMAPK1	CGTCA-motif	CGTCA	894	5	+	Hordeum vulgare	the MeJA responsive
TaMAPK1	CGTCA-motif	CGTCA	1816	5	-	Hordeum vulgare	the MeJA responsive
TaMAPK1	TGACG-motif	TGACG	894	5	-	Hordeum vulgare	the MeJA responsive
TaMAPK1	TGACG-motif	TGACG	1816	5	+	Hordeum vulgare	the MeJA responsive
TaMAPK1	O2-site	GATGACATGG	1018	10	-	Zea mays	zein metabolism regulation
TaMAPK1	O2-site	GATGATGTGG	1052	9	-	Zea mays	zein metabolism regulation
TaMAPK1	MSA-like	TCAAACGGT	450	9	-	Catharanthus roseus	cell cycle regulation
TaMAPK1	ARE	AAACCA	587	6	-	Zea mays	anaerobic induction
TaMAPK1	P-box	CCTTTTG	180	7	+	Oryza sativa	gibberellin responsive
TaMAPK1	ABRE	CACGTG	745	6	+	Arabidopsis thaliana	abscisic acid responsive
TaMAPK1	ABRE	ACGTG	746	5	+	Arabidopsis thaliana	abscisic acid responsive
TaMAPK1	TGA-element	AACGAC	1270	6	+	Brassica oleracea	auxin responsive
TaMAPK10	TCA-element	TCAGAAGAGG	1467	9	-	Brassica oleracea	salicylic acid responsive
TaMAPK10	MBS	CAACTG	1408	6	+	Arabidopsis thaliana	drought inducibility
TaMAPK10	ABRE	CGCACGTGTC	253	9	+	Hordeum vulgare	abscisic acid responsive
TaMAPK10	ABRE	CACGTG	255	6	+	Arabidopsis thaliana	abscisic acid responsive
TaMAPK10	ABRE	ACGTG	256	5	+	Arabidopsis thaliana	abscisic acid responsive
TaMAPK10	ABRE	ACGTG	976	5	+	Arabidopsis thaliana	abscisic acid responsive

TaMAPK10	GC-motif	CCCCCG	302	6 +	Zea mays	anoxic specific inducibility
TaMAPK10	GC-motif	CCCCCG	1080	6 -	Zea mays	anoxic specific inducibility
TaMAPK10	TC-rich repeats	ATTCTCTAAC	1646	9 -	Nicotiana tabacum	defense and stress responsive
TaMAPK10	GCN4_motif	TGAGTCA	72	7 -	Oryza sativa	endosperm expression
TaMAPK10	O2-site	GATGACATGG	1420	9 +	Zea mays	zein metabolism regulation
TaMAPK10	ARE	AAACCA	1639	6 -	Zea mays	anaerobic induction
TaMAPK11	ABRE	ACGTG	618	5 -	Arabidopsis thaliana	abscisic acid responsive
TaMAPK11	ABRE	CGTACGTGCA	1401	9 -	Hordeum vulgare	abscisic acid responsive
TaMAPK11	ABRE	CACGTG	1698	6 -	Arabidopsis thaliana	abscisic acid responsive
TaMAPK11	ABRE	ACGTG	1699	5 +	Arabidopsis thaliana	abscisic acid responsive
TaMAPK11	GC-motif	CCCCCG	1035	6 -	Zea mays	anoxic specific inducibility
TaMAPK11	CGTCA-motif	CGTCA	1427	5 -	Hordeum vulgare	the MeJA responsive
TaMAPK11	CGTCA-motif	CGTCA	1456	5 +	Hordeum vulgare	the MeJA responsive
TaMAPK11	CGTCA-motif	CGTCA	1760	5 -	Hordeum vulgare	the MeJA responsive
TaMAPK11	CGTCA-motif	CGTCA	1948	5 +	Hordeum vulgare	the MeJA responsive
TaMAPK11	ARE	AAACCA	81	6 -	Zea mays	anaerobic induction
TaMAPK11	ARE	AAACCA	379	6 +	Zea mays	anaerobic induction
TaMAPK11	ARE	AAACCA	1441	6 -	Zea mays	anaerobic induction
TaMAPK11	TGACG-motif	TGACG	1427	5 +	Hordeum vulgare	the MeJA responsive

TaMAPK11	TGACG-motif	TGACG	1456	5 -	Hordeum vulgare	the MeJA responsive
TaMAPK11	TGACG-motif	TGACG	1760	5 +	Hordeum vulgare	the MeJA responsive
TaMAPK11	TGACG-motif	TGACG	1948	5 -	Hordeum vulgare	the MeJA responsive
TaMAPK11	O2-site	GATGACATGG	1923	9 -	Zea mays	zein metabolism regulation
TaMAPK11	GCN4_motif	TGAGTCA	49	7 -	Oryza sativa	endosperm expression
TaMAPK11	LTR	CCGAAA	824	6 +	Hordeum vulgare	low temperature responsive
TaMAPK11	circadian	CAAAGATATC	1045	9 +	Lycopersicon esculentum	circadian control
TaMAPK11	HD-Zip 1	CAAT(A/T)ATTG	146	8 +	Arabidopsis thaliana	differentiation of the palisade mesophyll cells
TaMAPK12	GC-motif	CCCCCG	1889	6 +	Zea mays	anoxic specific inducibility
TaMAPK12	O2-site	GTTGACGTGA	1047	9 +	Zea mays	zein metabolism regulation
TaMAPK12	ARE	AAACCA	495	6 -	Zea mays	anaerobic induction
TaMAPK12	ABRE	ACGTG	1051	5 +	Arabidopsis thaliana	abscisic acid responsive
TaMAPK12	ABRE	GCCGCGTGGC	1543	9 -	Oryza sativa	abscisic acid responsive
TaMAPK12	ABRE	CACGTG	1606	6 -	Arabidopsis thaliana	abscisic acid responsive
TaMAPK12	ABRE	ACGTG	1607	5 +	Arabidopsis thaliana	abscisic acid responsive
TaMAPK12	ABRE	GACACGTGGC	1702	9 -	Triticum aestivum	abscisic acid responsive
TaMAPK12	TGA-element	AACGAC	1480	6 +	Brassica oleracea	auxin responsive
TaMAPK12	HD-Zip 1	CAAT(A/T)ATTG	30	8 +	Arabidopsis thaliana	differentiation of the palisade mesophyll cells

TaMAPK12	CGTCA-motif	CGTCA	170	5 +	Hordeum vulgare	the MeJA responsive
TaMAPK12	CGTCA-motif	CGTCA	1049	5 -	Hordeum vulgare	the MeJA responsive
TaMAPK12	CGTCA-motif	CGTCA	1381	5 +	Hordeum vulgare	the MeJA responsive
TaMAPK12	CGTCA-motif	CGTCA	1663	5 -	Hordeum vulgare	the MeJA responsive
TaMAPK12	TGACG-motif	TGACG	170	5 -	Hordeum vulgare	the MeJA responsive
TaMAPK12	TGACG-motif	TGACG	1049	5 +	Hordeum vulgare	the MeJA responsive
TaMAPK12	TGACG-motif	TGACG	1381	5 -	Hordeum vulgare	the MeJA responsive
TaMAPK12	TGACG-motif	TGACG	1663	5 +	Hordeum vulgare	the MeJA responsive
TaMAPK12	LTR	CCGAAA	1973	6 +	Hordeum vulgare	low temperature responsive
TaMAPK12	AuxRR-core	GGTCCAT	139	7 +	Nicotiana tabacum	auxin responsive
TaMAPK12	CAT-box	GCCACT	1698	6 -	Arabidopsis thaliana	meristem expression
TaMAPK13	TATC-box	TATCCCA	1921	7 +	Oryza sativa	gibberellin responsive
TaMAPK13	P-box	CCTTTTG	315	7 +	Oryza sativa	gibberellin responsive
TaMAPK13	ABRE	CACGTG	15	6 +	Arabidopsis thaliana	abscisic acid responsive
TaMAPK13	ABRE	ACGTG	16	5 +	Arabidopsis thaliana	abscisic acid responsive
TaMAPK13	ABRE	CACGTG	873	6 +	Arabidopsis thaliana	abscisic acid responsive
TaMAPK13	ABRE	ACGTG	874	5 +	Arabidopsis thaliana	abscisic acid responsive
TaMAPK13	GC-motif	CCCCCG	97	6 +	Zea mays	anoxic specific inducibility
TaMAPK13	TGA-element	AACGAC	674	6 -	Brassica oleracea	auxin responsive
TaMAPK13	TGA-element	AACGAC	1376	6 +	Brassica oleracea	auxin responsive

TaMAPK13	TGACG-motif	TGACG	1022	5 -	Hordeum vulgare	the MeJA responsive
TaMAPK13	TGACG-motif	TGACG	1227	5 -	Hordeum vulgare	the MeJA responsive
TaMAPK13	CGTCA-motif	CGTCA	1022	5 +	Hordeum vulgare	the MeJA responsive
TaMAPK13	CGTCA-motif	CGTCA	1227	5 +	Hordeum vulgare	the MeJA responsive
TaMAPK13	ARE	AAACCA	715	6 -	Zea mays	anaerobic induction
TaMAPK13	O2-site	GATGACATGG	1120	9 -	Zea mays	zein metabolism regulation
TaMAPK13	O2-site	GATGATGTGG	1154	9 -	Zea mays	zein metabolism regulation
TaMAPK13	MSA-like	TCAAACGGT	578	9 -	Catharanthus roseus	cell cycle regulation
TaMAPK14	TC-rich repeats	ATTCTCTAAC	29	9 +	Nicotiana tabacum	defense and stress responsive
TaMAPK14	TC-rich repeats	GTTTTCTTAC	260	9 +	Nicotiana tabacum	defense and stress responsive
TaMAPK14	RY-element	CATGCATG	1068	8 -	Helianthus annuus	seed specific regulation
TaMAPK14	ABRE	ACGTG	38	5 -	Arabidopsis thaliana	abscisic acid responsive
TaMAPK14	ABRE	ACGTG	68	5 +	Arabidopsis thaliana	abscisic acid responsive
TaMAPK14	ABRE	ACGTG	206	5 -	Arabidopsis thaliana	abscisic acid responsive
TaMAPK14	ABRE	GCAACGTGTC	304	9 -	Hordeum vulgare	abscisic acid responsive
TaMAPK14	ABRE	ACGTG	764	5 +	Arabidopsis thaliana	abscisic acid responsive
TaMAPK14	ABRE	GACACGTGGC	880	9 -	Triticum aestivum	abscisic acid responsive
TaMAPK14	ABRE	CACGTG	882	6 +	Arabidopsis thaliana	abscisic acid responsive
TaMAPK14	ABRE	ACGTG	883	5 +	Arabidopsis thaliana	abscisic acid responsive

TaMAPK14	LTR	CCGAAA	47	6 +	Hordeum vulgare	low temperature responsive
TaMAPK14	GARE-motif	TCTGTTG	828	7 -	Brassica oleracea	gibberellin responsive
TaMAPK14	TGACG-motif	TGACG	40	5 -	Hordeum vulgare	the MeJA responsive
TaMAPK14	TGACG-motif	TGACG	208	5 -	Hordeum vulgare	the MeJA responsive
TaMAPK14	TGACG-motif	TGACG	638	5 -	Hordeum vulgare	the MeJA responsive
TaMAPK14	MBS	CAACTG	117	6 -	Arabidopsis thaliana	drought inducibility
TaMAPK14	CGTCA-motif	CGTCA	40	5 +	Hordeum vulgare	the MeJA responsive
TaMAPK14	CGTCA-motif	CGTCA	208	5 +	Hordeum vulgare	the MeJA responsive
TaMAPK14	CGTCA-motif	CGTCA	638	5 +	Hordeum vulgare	the MeJA responsive
TaMAPK14	TGA-element	AACGAC	278	6 -	Brassica oleracea	auxin responsive
TaMAPK14	TGA-element	AACGAC	1637	6 -	Brassica oleracea	auxin responsive
TaMAPK14	TGA-element	AACGAC	1908	6 +	Brassica oleracea	auxin responsive
TaMAPK14	P-box	CCTTTTG	712	7 -	Oryza sativa	gibberellin responsive
TaMAPK14	O2-site	GATGACATGG	1059	9 -	Zea mays	zein metabolism regulation
TaMAPK14	TCA-element	CCATCTTTTT	1864	9 -	Nicotiana tabacum	salicylic acid responsive
TaMAPK14	ARE	AAACCA	84	6 -	Zea mays	anaerobic induction
TaMAPK14	ARE	AAACCA	202	6 +	Zea mays	anaerobic induction
TaMAPK16	ARE	AAACCA	1438	6 -	Zea mays	anaerobic induction
TaMAPK16	ARE	AAACCA	1470	6 +	Zea mays	anaerobic induction
TaMAPK16	ARE	AAACCA	1767	6 +	Zea mays	anaerobic induction

TaMAPK16	ABRE	ACGTG	325	5 -	Arabidopsis thaliana	abscisic acid responsive
TaMAPK16	ABRE	AACCCGG	892	7 -	Arabidopsis thaliana	abscisic acid responsive
TaMAPK16	ABRE	GCCGCGTGGC	1500	9 -	Oryza sativa	abscisic acid responsive
TaMAPK16	ABRE	GCCGCGTGGC	1580	9 -	Oryza sativa	abscisic acid responsive
TaMAPK16	ABRE	ACGTG	1734	5 -	Arabidopsis thaliana	abscisic acid responsive
TaMAPK16	ABRE	CGCACGTGTC	1837	9 -	Hordeum vulgare	abscisic acid responsive
TaMAPK16	CGTCA-motif	CGTCA	247	5 +	Hordeum vulgare	the MeJA responsive
TaMAPK16	CGTCA-motif	CGTCA	1076	5 +	Hordeum vulgare	the MeJA responsive
TaMAPK16	CGTCA-motif	CGTCA	1557	5 +	Hordeum vulgare	the MeJA responsive
TaMAPK16	LTR	CCGAAA	124	6 -	Hordeum vulgare	low temperature responsive
TaMAPK16	TCA-element	CCATCTTTTT	1021	9 -	Nicotiana tabacum	salicylic acid responsive
TaMAPK16	O2-site	GATGATGTGG	378	9 +	Zea mays	zein metabolism regulation
TaMAPK16	O2-site	GATGACATGG	991	9 +	Zea mays	zein metabolism regulation
TaMAPK16	O2-site	GATGACATGG	1129	9 +	Zea mays	zein metabolism regulation
TaMAPK16	O2-site	GATGATGTGG	1551	9 -	Zea mays	zein metabolism regulation
TaMAPK16	GC-motif	CCCCCG	58	6 -	Zea mays	anoxic specific inducibility
TaMAPK16	MBS	CAACTG	331	6 -	Arabidopsis thaliana	drought inducibility
TaMAPK16	TGACG-motif	TGACG	247	5 -	Hordeum vulgare	the MeJA responsive
TaMAPK16	TGACG-motif	TGACG	1076	5 -	Hordeum vulgare	the MeJA responsive

TaMAPK16	TGACG-motif	TGACG	1557	5 -	Hordeum vulgare	the MeJA responsive
TaMAPK17	TATC-box	TATCCCA	875	7 +	Oryza sativa	gibberellin responsive
TaMAPK17	TCA-element	TCAGAAGAGG	34	9 +	Brassica oleracea	salicylic acid responsive
TaMAPK17	TCA-element	CCATCTTTTT	291	9 +	Nicotiana tabacum	salicylic acid responsive
TaMAPK17	TCA-element	TCAGAAGAGG	1518	9 +	Brassica oleracea	salicylic acid responsive
TaMAPK17	TGA-element	AACGAC	1940	6 -	Brassica oleracea	auxin responsive
TaMAPK17	ABRE	ACGTG	240	5 +	Arabidopsis thaliana	abscisic acid responsive
TaMAPK17	ABRE	ACGTG	821	5 +	Arabidopsis thaliana	abscisic acid responsive
TaMAPK17	ABRE	ACGTG	1198	5 -	Arabidopsis thaliana	abscisic acid responsive
TaMAPK17	TGACG-motif	TGACG	304	5 -	Hordeum vulgare	the MeJA responsive
TaMAPK17	TGACG-motif	TGACG	1946	5 -	Hordeum vulgare	the MeJA responsive
TaMAPK17	CGTCA-motif	CGTCA	304	5 +	Hordeum vulgare	the MeJA responsive
TaMAPK17	CGTCA-motif	CGTCA	1946	5 +	Hordeum vulgare	the MeJA responsive
TaMAPK17	ARE	AAACCA	1434	6 -	Zea mays	anaerobic induction
TaMAPK17	ARE	AAACCA	1550	6 -	Zea mays	anaerobic induction
TaMAPK18	TGACG-motif	TGACG	792	5 -	Hordeum vulgare	the MeJA responsive
TaMAPK18	TGACG-motif	TGACG	1230	5 -	Hordeum vulgare	the MeJA responsive
TaMAPK18	CGTCA-motif	CGTCA	792	5 +	Hordeum vulgare	the MeJA responsive
TaMAPK18	CGTCA-motif	CGTCA	1230	5 +	Hordeum vulgare	the MeJA responsive

TaMAPK18	TATC-box	TATCCCA	1154	7 +	Oryza sativa	gibberellin responsive
TaMAPK18	ARE	AAACCA	371	6 -	Zea mays	anaerobic induction
TaMAPK18	ARE	AAACCA	469	6 -	Zea mays	anaerobic induction
TaMAPK18	ARE	AAACCA	1570	6 -	Zea mays	anaerobic induction
TaMAPK18	ABRE	ACGTG	69	5 +	Arabidopsis thaliana	abscisic acid responsive
TaMAPK18	ABRE	ACGTG	156	5 +	Arabidopsis thaliana	abscisic acid responsive
TaMAPK18	ABRE	ACGTG	1023	5 -	Arabidopsis thaliana	abscisic acid responsive
TaMAPK18	ABRE	ACGTG	1159	5 -	Arabidopsis thaliana	abscisic acid responsive
TaMAPK18	ABRE	ACGTG	1694	5 +	Arabidopsis thaliana	abscisic acid responsive
TaMAPK18	GC-motif	CCCCCG	40	6 -	Zea mays	anoxic specific inducibility
TaMAPK18	GC-motif	CCCCCG	92	6 -	Zea mays	anoxic specific inducibility
TaMAPK18	O2-site	GATGACATGG	1215	9 -	Zea mays	zein metabolism regulation
TaMAPK18	GCN4_motif	TGAGTCA	912	7 +	Oryza sativa	endosperm expression
TaMAPK18	GCN4_motif	TGAGTCA	1739	7 -	Oryza sativa	endosperm expression
TaMAPK18	GARE-motif	TCTGTTG	1041	7 +	Brassica oleracea	gibberellin responsive
TaMAPK18	LTR	CCGAAA	472	6 -	Hordeum vulgare	low temperature responsive
TaMAPK19	ABRE	CGTACGTGCA	348	9 +	Hordeum vulgare	abscisic acid responsive
TaMAPK19	ABRE	ACGTG	640	5 +	Arabidopsis thaliana	abscisic acid responsive
TaMAPK19	ABRE	ACGTG	1437	5 +	Arabidopsis thaliana	abscisic acid responsive

TaMAPK19	circadian	CAAAGATATC	105	9 -	Lycopersicon esculentum	circadian control
TaMAPK19	circadian	CAAAGATATC	711	9 -	Lycopersicon esculentum	circadian control
TaMAPK19	GC-motif	CCCCCG	407	6 +	Zea mays	anoxic specific inducibility
TaMAPK19	GC-motif	CCCCCG	1094	6 -	Zea mays	anoxic specific inducibility
TaMAPK19	GC-motif	CCCCCG	1975	6 +	Zea mays	anoxic specific inducibility
TaMAPK19	O2-site	GATGA(C/T)(A/G)TG(A/G)	507	8 +	Zea mays	zein metabolism regulation
TaMAPK19	O2-site	GATGACATGG	1841	9 -	Zea mays	zein metabolism regulation
TaMAPK19	CGTCA-motif	CGTCA	859	5 -	Hordeum vulgare	the MeJA responsive
TaMAPK19	CGTCA-motif	CGTCA	881	5 -	Hordeum vulgare	the MeJA responsive
TaMAPK19	CGTCA-motif	CGTCA	1003	5 -	Hordeum vulgare	the MeJA responsive
TaMAPK19	CGTCA-motif	CGTCA	1435	5 -	Hordeum vulgare	the MeJA responsive
TaMAPK19	TGACG-motif	TGACG	859	5 +	Hordeum vulgare	the MeJA responsive
TaMAPK19	TGACG-motif	TGACG	881	5 +	Hordeum vulgare	the MeJA responsive
TaMAPK19	TGACG-motif	TGACG	1003	5 +	Hordeum vulgare	the MeJA responsive
TaMAPK19	TGACG-motif	TGACG	1435	5 +	Hordeum vulgare	the MeJA responsive
TaMAPK19	AuxRR-core	GGTCCAT	499	7 -	Nicotiana tabacum	auxin responsive
TaMAPK19	LTR	CCGAAA	180	6 +	Hordeum vulgare	low temperature responsive
TaMAPK19	LTR	CCGAAA	387	6 +	Hordeum vulgare	low temperature responsive
TaMAPK19	MBS	CAACTG	433	6 +	Arabidopsis thaliana	drought inducibility

TaMAPK19	CAT-box	GCCACT	301	6 -	Arabidopsis thaliana	meristem expression
TaMAPK19	CAT-box	GCCACT	1768	6 +	Arabidopsis thaliana	meristem expression
TaMAPK19	CAT-box	GCCACT	1781	6 +	Arabidopsis thaliana	meristem expression
TaMAPK2	ABRE	CACGTG	1334	6 -	Arabidopsis thaliana	abscisic acid responsive
TaMAPK2	ABRE	ACGTG	1335	5 +	Arabidopsis thaliana	abscisic acid responsive
TaMAPK2	TGA-element	AACGAC	1929	6 +	Brassica oleracea	auxin responsive
TaMAPK2	CGTCA-motif	CGTCA	126	5 +	Hordeum vulgare	the MeJA responsive
TaMAPK2	CGTCA-motif	CGTCA	359	5 +	Hordeum vulgare	the MeJA responsive
TaMAPK2	CGTCA-motif	CGTCA	1839	5 +	Hordeum vulgare	the MeJA responsive
TaMAPK2	LTR	CCGAAA	766	6 -	Hordeum vulgare	low temperature responsive
TaMAPK2	LTR	CCGAAA	902	6 -	Hordeum vulgare	low temperature responsive
TaMAPK2	MBS	CAACTG	1357	6 -	Arabidopsis thaliana	drought inducibility
TaMAPK2	MBS	CAACTG	1742	6 -	Arabidopsis thaliana	drought inducibility
TaMAPK2	GARE-motif	TCTGTTG	1393	7 +	Brassica oleracea	gibberellin responsive
TaMAPK2	TGACG-motif	TGACG	126	5 -	Hordeum vulgare	the MeJA responsive
TaMAPK2	TGACG-motif	TGACG	359	5 -	Hordeum vulgare	the MeJA responsive
TaMAPK2	TGACG-motif	TGACG	1839	5 -	Hordeum vulgare	the MeJA responsive
TaMAPK2	TC-rich repeats	GTTTTCTTAC	1880	9 +	Nicotiana tabacum	defense and stress responsive
TaMAPK2	GC-motif	CCCCCG	270	6 +	Zea mays	anoxic specific inducibility

TaMAPK2	GC-motif	CCCCCG	299	6 +	Zea mays	anoxic specific inducibility
TaMAPK2	GC-motif	CCCCCG	378	6 +	Zea mays	anoxic specific inducibility
TaMAPK2	GC-motif	CCCCCG	545	6 +	Zea mays	anoxic specific inducibility
TaMAPK2	GC-motif	CCCCCG	629	6 +	Zea mays	anoxic specific inducibility
TaMAPK2	GC-motif	CCCCCG	1784	6 +	Zea mays	anoxic specific inducibility
TaMAPK2	ARE	AAACCA	25	6 +	Zea mays	anaerobic induction
TaMAPK2	ARE	AAACCA	115	6 +	Zea mays	anaerobic induction
TaMAPK2	CAT-box	GCCACT	1296	6 -	Arabidopsis thaliana	meristem expression
TaMAPK2	CAT-box	GCCACT	1300	6 +	Arabidopsis thaliana	meristem expression
TaMAPK20	CAT-box	GCCACT	1885	6 -	Arabidopsis thaliana	meristem expression
TaMAPK20	LTR	CCGAAA	1720	6 +	Hordeum vulgare	low temperature responsive
TaMAPK20	GARE-motif	TCTGTTG	29	7 +	Brassica oleracea	gibberellin responsive
TaMAPK20	GCN4_motif	TGAGTCA	750	7 +	Oryza sativa	endosperm expression
TaMAPK20	TATC-box	TATCCCA	704	7 -	Oryza sativa	gibberellin responsive
TaMAPK20	CGTCA-motif	CGTCA	449	5 -	Hordeum vulgare	the MeJA responsive
TaMAPK20	CGTCA-motif	CGTCA	565	5 -	Hordeum vulgare	the MeJA responsive
TaMAPK20	CGTCA-motif	CGTCA	1634	5 +	Hordeum vulgare	the MeJA responsive
TaMAPK20	TGACG-motif	TGACG	449	5 +	Hordeum vulgare	the MeJA responsive
TaMAPK20	TGACG-motif	TGACG	565	5 +	Hordeum vulgare	the MeJA responsive

TaMAPK20	TGACG-motif	TGACG	1634	5 -	Hordeum vulgare	the MeJA responsive
TaMAPK20	O2-site	GATGACATGG	771	9 +	Zea mays	zein metabolism regulation
TaMAPK20	GC-motif	CCCCCG	234	6 -	Zea mays	anoxic specific inducibility
TaMAPK20	TGA-element	AACGAC	322	6 -	Brassica oleracea	auxin responsive
TaMAPK20	TGA-element	AACGAC	380	6 -	Brassica oleracea	auxin responsive
TaMAPK22	LTR	CCGAAA	1787	6 +	Hordeum vulgare	low temperature responsive
TaMAPK22	GARE-motif	TCTGTTG	959	7 -	Brassica oleracea	gibberellin responsive
TaMAPK22	MBS	CAACTG	84	6 -	Arabidopsis thaliana	drought inducibility
TaMAPK22	CAT-box	GCCACT	1550	6 -	Arabidopsis thaliana	meristem expression
TaMAPK22	TGA-element	AACGAC	1322	6 +	Brassica oleracea	auxin responsive
TaMAPK22	ABRE	ACGTG	554	5 +	Arabidopsis thaliana	abscisic acid responsive
TaMAPK22	ABRE	ACGTG	980	5 +	Arabidopsis thaliana	abscisic acid responsive
TaMAPK22	ABRE	ACGTG	1091	5 -	Arabidopsis thaliana	abscisic acid responsive
TaMAPK22	ABRE	ACGTG	1150	5 +	Arabidopsis thaliana	abscisic acid responsive
TaMAPK22	ABRE	ACGTG	1171	5 +	Arabidopsis thaliana	abscisic acid responsive
TaMAPK22	ABRE	CGTACGTGCA	1455	9 -	Hordeum vulgare	abscisic acid responsive
TaMAPK22	ABRE	ACGTG	1457	5 -	Arabidopsis thaliana	abscisic acid responsive
TaMAPK22	ABRE	GACACGTGGC	1554	9 -	Triticum aestivum	abscisic acid responsive
TaMAPK22	ABRE	ACGTG	1744	5 +	Arabidopsis thaliana	abscisic acid responsive
TaMAPK22	O2-site	GTTGACGTGA	550	9 +	Zea mays	zein metabolism regulation

TaMAPK22	GC-motif	CCCCCG	1263	6 -	Zea mays	anoxic specific inducibility
TaMAPK22	GC-motif	CCCCCG	1393	6 +	Zea mays	anoxic specific inducibility
TaMAPK22	CGTCA-motif	CGTCA	552	5 -	Hordeum vulgare	the MeJA responsive
TaMAPK22	CGTCA-motif	CGTCA	943	5 -	Hordeum vulgare	the MeJA responsive
TaMAPK22	CGTCA-motif	CGTCA	1148	5 -	Hordeum vulgare	the MeJA responsive
TaMAPK22	TGACG-motif	TGACG	552	5 +	Hordeum vulgare	the MeJA responsive
TaMAPK22	TGACG-motif	TGACG	943	5 +	Hordeum vulgare	the MeJA responsive
TaMAPK22	TGACG-motif	TGACG	1148	5 +	Hordeum vulgare	the MeJA responsive
TaMAPK23	CAT-box	GCCACT	522	6 -	Arabidopsis thaliana	meristem expression
TaMAPK23	MBS	CAACTG	101	6 -	Arabidopsis thaliana	drought inducibility
TaMAPK23	MBS	CAACTG	1244	6 -	Arabidopsis thaliana	drought inducibility
TaMAPK23	TGACG-motif	TGACG	283	5 +	Hordeum vulgare	the MeJA responsive
TaMAPK23	TGACG-motif	TGACG	337	5 +	Hordeum vulgare	the MeJA responsive
TaMAPK23	TGACG-motif	TGACG	455	5 +	Hordeum vulgare	the MeJA responsive
TaMAPK23	TGACG-motif	TGACG	942	5 -	Hordeum vulgare	the MeJA responsive
TaMAPK23	TGACG-motif	TGACG	1337	5 -	Hordeum vulgare	the MeJA responsive
TaMAPK23	TGACG-motif	TGACG	1467	5 -	Hordeum vulgare	the MeJA responsive
TaMAPK23	ARE	AAACCA	1577	6 -	Zea mays	anaerobic induction
TaMAPK23	GC-motif	CCCCCG	140	6 -	Zea mays	anoxic specific inducibility

TaMAPK23	GC-motif	CCCCCG	736	6 -	Zea mays	anoxic specific inducibility
TaMAPK23	GC-motif	CCCCCG	1630	6 +	Zea mays	anoxic specific inducibility
TaMAPK23	ABRE	GCCGCGTGGC	298	9 +	Oryza sativa	abscisic acid responsive
TaMAPK23	ABRE	GCCGCGTGGC	1109	9 +	Oryza sativa	abscisic acid responsive
TaMAPK23	ABRE	CACGTG	1543	6 -	Arabidopsis thaliana	abscisic acid responsive
TaMAPK23	ABRE	ACGTG	1544	5 +	Arabidopsis thaliana	abscisic acid responsive
TaMAPK23	ABRE	CACGTG	1668	6 -	Arabidopsis thaliana	abscisic acid responsive
TaMAPK23	ABRE	ACGTG	1669	5 +	Arabidopsis thaliana	abscisic acid responsive
TaMAPK23	CGTCA-motif	CGTCA	283	5 -	Hordeum vulgare	the MeJA responsive
TaMAPK23	CGTCA-motif	CGTCA	337	5 -	Hordeum vulgare	the MeJA responsive
TaMAPK23	CGTCA-motif	CGTCA	455	5 -	Hordeum vulgare	the MeJA responsive
TaMAPK23	CGTCA-motif	CGTCA	942	5 +	Hordeum vulgare	the MeJA responsive
TaMAPK23	CGTCA-motif	CGTCA	1337	5 +	Hordeum vulgare	the MeJA responsive
TaMAPK23	CGTCA-motif	CGTCA	1467	5 +	Hordeum vulgare	the MeJA responsive
TaMAPK23	LTR	CCGAAA	1207	6 -	Hordeum vulgare	low temperature responsive
TaMAPK23	LTR	CCGAAA	1488	6 +	Hordeum vulgare	low temperature responsive
TaMAPK23	TGA-element	AACGAC	195	6 -	Brassica oleracea	auxin responsive
TaMAPK24	TATC-box	TATCCCA	1248	7 -	Oryza sativa	gibberellin responsive
TaMAPK24	TCA-element	CCATCTTTTT	1007	9 -	Nicotiana tabacum	salicylic acid responsive

TaMAPK24	GCN4_motif	TGAGTCA	443	7 +	Oryza sativa	endosperm expression
TaMAPK24	ABRE	CGTACGTGCA	136	10 +	Hordeum vulgare	abscisic acid responsive
TaMAPK24	ABRE	ACGTG	139	5 +	Arabidopsis thaliana	abscisic acid responsive
TaMAPK24	ABRE	ACGTG	248	5 -	Arabidopsis thaliana	abscisic acid responsive
TaMAPK24	ABRE	GCCGCGTGGC	1478	9 -	Oryza sativa	abscisic acid responsive
TaMAPK24	ABRE	GCCGCGTGGC	1558	9 -	Oryza sativa	abscisic acid responsive
TaMAPK24	ABRE	CGCACGTGTC	1829	9 -	Hordeum vulgare	abscisic acid responsive
TaMAPK24	CGTCA-motif	CGTCA	174	5 +	Hordeum vulgare	the MeJA responsive
TaMAPK24	CGTCA-motif	CGTCA	1062	5 +	Hordeum vulgare	the MeJA responsive
TaMAPK24	CGTCA-motif	CGTCA	1535	5 +	Hordeum vulgare	the MeJA responsive
TaMAPK24	CGTCA-motif	CGTCA	1618	5 +	Hordeum vulgare	the MeJA responsive
TaMAPK24	LTR	CCGAAA	150	6 -	Hordeum vulgare	low temperature responsive
TaMAPK24	P-box	CCTTTTG	841	7 -	Oryza sativa	gibberellin responsive
TaMAPK24	ARE	AAACCA	1416	6 -	Zea mays	anaerobic induction
TaMAPK24	ARE	AAACCA	1759	6 +	Zea mays	anaerobic induction
TaMAPK24	TC-rich repeats	GTTTTCTTAC	1576	9 -	Nicotiana tabacum	defense and stress responsive
TaMAPK24	TGACG-motif	TGACG	174	5 -	Hordeum vulgare	the MeJA responsive
TaMAPK24	TGACG-motif	TGACG	1062	5 -	Hordeum vulgare	the MeJA responsive
TaMAPK24	TGACG-motif	TGACG	1535	5 -	Hordeum vulgare	the MeJA responsive

TaMAPK24	TGACG-motif	TGACG	1618	5 -	Hordeum vulgare	the MeJA responsive
TaMAPK24	O2-site	GATGACATGG	977	9 +	Zea mays	zein metabolism regulation
TaMAPK24	O2-site	GATGACATGG	1115	9 +	Zea mays	zein metabolism regulation
TaMAPK24	O2-site	GATGATGTGG	1529	9 -	Zea mays	zein metabolism regulation
TaMAPK25	TC-rich repeats	GTTTTCTTAC	1086	9 +	Nicotiana tabacum	defense and stress responsive
TaMAPK25	TGACG-motif	TGACG	1534	5 -	Hordeum vulgare	the MeJA responsive
TaMAPK25	ARE	AAACCA	1357	6 +	Zea mays	anaerobic induction
TaMAPK25	ABRE	ACGTG	1245	5 -	Arabidopsis thaliana	abscisic acid responsive
TaMAPK25	CGTCA-motif	CGTCA	1534	5 +	Hordeum vulgare	the MeJA responsive
TaMAPK25	circadian	CAAAGATATC	1082	9 +	Lycopersicon esculentum	circadian control
TaMAPK26	TC-rich repeats	GTTTTCTTAC	1945	9 +	Nicotiana tabacum	defense and stress responsive
TaMAPK26	LTR	CCGAAA	830	6 -	Hordeum vulgare	low temperature responsive
TaMAPK26	LTR	CCGAAA	875	6 -	Hordeum vulgare	low temperature responsive
TaMAPK26	CAT-box	GCCACT	1379	6 -	Arabidopsis thaliana	meristem expression
TaMAPK26	CAT-box	GCCACT	1383	6 +	Arabidopsis thaliana	meristem expression
TaMAPK26	GARE-motif	TCTGTTG	1475	7 +	Brassica oleracea	gibberellin responsive
TaMAPK26	MBS	CAACTG	1088	6 +	Arabidopsis thaliana	drought inducibility
TaMAPK26	MBS	CAACTG	1439	6 -	Arabidopsis thaliana	drought inducibility
TaMAPK26	MBS	CAACTG	1807	6 -	Arabidopsis thaliana	drought inducibility

TaMAPK26	ABRE	GCCGCGTGGC	140	9 +	Oryza sativa	abscisic acid responsive
TaMAPK26	ABRE	CACGTG	1416	6 -	Arabidopsis thaliana	abscisic acid responsive
TaMAPK26	ABRE	ACGTG	1417	5 +	Arabidopsis thaliana	abscisic acid responsive
TaMAPK26	ABRE	ACGTG	1712	5 +	Arabidopsis thaliana	abscisic acid responsive
TaMAPK26	GC-motif	CCCCCG	17	6 +	Zea mays	anoxic specific inducibility
TaMAPK26	GC-motif	CCCCCG	46	6 +	Zea mays	anoxic specific inducibility
TaMAPK26	GC-motif	CCCCCG	125	6 +	Zea mays	anoxic specific inducibility
TaMAPK26	GC-motif	CCCCCG	588	6 +	Zea mays	anoxic specific inducibility
TaMAPK26	GC-motif	CCCCCG	1849	6 +	Zea mays	anoxic specific inducibility
TaMAPK26	TGA-element	AACGAC	1994	6 +	Brassica oleracea	auxin responsive
TaMAPK27	ARE	AAACCA	99	6 +	Zea mays	anaerobic induction
TaMAPK27	TGACG-motif	TGACG	11	5 -	Hordeum vulgare	the MeJA responsive
TaMAPK27	TGACG-motif	TGACG	1443	5 +	Hordeum vulgare	the MeJA responsive
TaMAPK27	TGACG-motif	TGACG	1948	5 -	Hordeum vulgare	the MeJA responsive
TaMAPK27	GCN4_motif	TGAGTCA	1507	7 +	Oryza sativa	endosperm expression
TaMAPK27	O2-site	GATGACATGG	1923	9 -	Zea mays	zein metabolism regulation
TaMAPK27	LTR	CCGAAA	778	6 +	Hordeum vulgare	low temperature responsive
TaMAPK27	circadian	CAAAGATATC	999	9 +	Lycopersicon esculentum	circadian control
TaMAPK27	P-box	CCTTTTG	1853	7 +	Oryza sativa	gibberellin responsive

TaMAPK27	ABRE	ACGTG	339	5 +	Arabidopsis thaliana	abscisic acid responsive
TaMAPK27	ABRE	ACGTG	567	5 -	Arabidopsis thaliana	abscisic acid responsive
TaMAPK27	GC-motif	CCCCCG	961	6 +	Zea mays	anoxic specific inducibility
TaMAPK27	TGA-element	AACGAC	1556	6 -	Brassica oleracea	auxin responsive
TaMAPK27	CGTCA-motif	CGTCA	11	5 +	Hordeum vulgare	the MeJA responsive
TaMAPK27	CGTCA-motif	CGTCA	1443	5 -	Hordeum vulgare	the MeJA responsive
TaMAPK27	CGTCA-motif	CGTCA	1948	5 +	Hordeum vulgare	the MeJA responsive
TaMAPK28	TATC-box	TATCCCA	1737	7 -	Oryza sativa	gibberellin responsive
TaMAPK28	AuxRR-core	GGTCCAT	1640	7 -	Nicotiana tabacum	auxin responsive
TaMAPK28	LTR	CCGAAA	41	6 +	Hordeum vulgare	low temperature responsive
TaMAPK28	MBSI	aaaAaaC(G/C)GTTA	262	10.5 -	Petunia hybrida	flavonoid biosynthetic genes regulation
TaMAPK28	TGACG-motif	TGACG	34	5 -	Hordeum vulgare	the MeJA responsive
TaMAPK28	TGACG-motif	TGACG	205	5 -	Hordeum vulgare	the MeJA responsive
TaMAPK28	TGACG-motif	TGACG	637	5 -	Hordeum vulgare	the MeJA responsive
TaMAPK28	TCA-element	CCATCTTTTT	1865	9 -	Nicotiana tabacum	salicylic acid responsive
TaMAPK28	ARE	AAACCA	78	6 -	Zea mays	anaerobic induction
TaMAPK28	ARE	AAACCA	199	6 +	Zea mays	anaerobic induction
TaMAPK28	RY-element	CATGCATG	1182	8 -	Helianthus annuus	seed specific regulation
TaMAPK28	RY-element	CATGCATG	1307	8 -	Helianthus annuus	seed specific regulation

TaMAPK28	MBS	CAACTG	111	6 -	Arabidopsis thaliana	drought inducibility
TaMAPK28	TGA-element	AACGAC	1908	6 +	Brassica oleracea	auxin responsive
TaMAPK28	CGTCA-motif	CGTCA	34	5 +	Hordeum vulgare	the MeJA responsive
TaMAPK28	CGTCA-motif	CGTCA	205	5 +	Hordeum vulgare	the MeJA responsive
TaMAPK28	CGTCA-motif	CGTCA	637	5 +	Hordeum vulgare	the MeJA responsive
TaMAPK28	P-box	CCTTTTG	805	7 -	Oryza sativa	gibberellin responsive
TaMAPK28	ABRE	ACGTG	32	5 -	Arabidopsis thaliana	abscisic acid responsive
TaMAPK28	ABRE	ACGTG	203	5 -	Arabidopsis thaliana	abscisic acid responsive
TaMAPK28	ABRE	ACGTG	343	5 -	Arabidopsis thaliana	abscisic acid responsive
TaMAPK29	GC-motif	CCCCCG	1348	6 +	Zea mays	anoxic specific inducibility
TaMAPK29	ABRE	ACGTG	30	5 +	Arabidopsis thaliana	abscisic acid responsive
TaMAPK29	ABRE	ACGTG	244	5 -	Arabidopsis thaliana	abscisic acid responsive
TaMAPK29	ABRE	ACGTG	1457	5 -	Arabidopsis thaliana	abscisic acid responsive
TaMAPK29	ABRE	CACGTG	1616	6 -	Arabidopsis thaliana	abscisic acid responsive
TaMAPK29	ABRE	ACGTG	1617	5 +	Arabidopsis thaliana	abscisic acid responsive
TaMAPK29	ABRE	ACGTG	1663	5 -	Arabidopsis thaliana	abscisic acid responsive
TaMAPK29	CGTCA-motif	CGTCA	741	5 -	Hordeum vulgare	the MeJA responsive
TaMAPK29	CGTCA-motif	CGTCA	1255	5 -	Hordeum vulgare	the MeJA responsive
TaMAPK29	CAT-box	GCCACT	1568	6 -	Arabidopsis thaliana	meristem expression

TaMAPK29	TGACG-motif	TGACG	741	5 +	Hordeum vulgare	the MeJA responsive
TaMAPK29	TGACG-motif	TGACG	1255	5 +	Hordeum vulgare	the MeJA responsive
TaMAPK29	LTR	CCGAAA	899	6 -	Hordeum vulgare	low temperature responsive
TaMAPK3	AuxRR-core	GGTCCAT	1153	7 +	Nicotiana tabacum	auxin responsive
TaMAPK3	AuxRR-core	GGTCCAT	1165	7 -	Nicotiana tabacum	auxin responsive
TaMAPK3	CAT-box	GCCACT	1066	6 +	Arabidopsis thaliana	meristem expression
TaMAPK3	GC-motif	CCCCCG	999	6 +	Zea mays	anoxic specific inducibility
TaMAPK3	TCA-element	CCATCTTTTT	828	9 -	Nicotiana tabacum	salicylic acid responsive
TaMAPK3	ARE	AAACCA	273	6 +	Zea mays	anaerobic induction
TaMAPK3	ARE	AAACCA	1335	6 +	Zea mays	anaerobic induction
TaMAPK3	ARE	AAACCA	1389	6 -	Zea mays	anaerobic induction
TaMAPK3	TGA-element	AACGAC	411	6 +	Brassica oleracea	auxin responsive
TaMAPK3	TGA-element	AACGAC	590	6 +	Brassica oleracea	auxin responsive
TaMAPK3	TGA-element	AACGAC	1840	6 +	Brassica oleracea	auxin responsive
TaMAPK3	ABRE	GACACGTGGC	809	9 +	Triticum aestivum	abscisic acid responsive
TaMAPK3	ABRE	AACCCGG	1774	7 +	Arabidopsis thaliana	abscisic acid responsive
TaMAPK3	P-box	CCTTTTG	1047	7 -	Oryza sativa	gibberellin responsive
TaMAPK3	CGTCA-motif	CGTCA	38	5 +	Hordeum vulgare	the MeJA responsive
TaMAPK3	CGTCA-motif	CGTCA	773	5 -	Hordeum vulgare	the MeJA responsive
TaMAPK3	CGTCA-motif	CGTCA	1635	5 +	Hordeum vulgare	the MeJA responsive

TaMAPK3	TGACG-motif	TGACG	38	5 -	Hordeum vulgare	the MeJA responsive
TaMAPK3	TGACG-motif	TGACG	773	5 +	Hordeum vulgare	the MeJA responsive
TaMAPK3	TGACG-motif	TGACG	1635	5 -	Hordeum vulgare	the MeJA responsive
TaMAPK30	ABRE	ACGTG	111	5 +	Arabidopsis thaliana	abscisic acid responsive
TaMAPK30	ABRE	ACGTG	965	5 -	Arabidopsis thaliana	abscisic acid responsive
TaMAPK30	ABRE	GCCGCGTGGC	1546	9 -	Oryza sativa	abscisic acid responsive
TaMAPK30	ABRE	GCCGCGTGGC	1665	9 -	Oryza sativa	abscisic acid responsive
TaMAPK30	TGA-element	AACGAC	931	6 +	Brassica oleracea	auxin responsive
TaMAPK30	circadian	CAAAGATATC	474	9 +	Lycopersicon esculentum	circadian control
TaMAPK30	TATC-box	TATCCCA	377	7 +	Oryza sativa	gibberellin responsive
TaMAPK30	MBS	CAACTG	702	6 -	Arabidopsis thaliana	drought inducibility
TaMAPK30	MBS	CAACTG	847	6 +	Arabidopsis thaliana	drought inducibility
TaMAPK30	TC-rich repeats	ATTCTCTAAC	255	9 +	Nicotiana tabacum	defense and stress responsive
TaMAPK30	ARE	AAACCA	233	6 +	Zea mays	anaerobic induction
TaMAPK30	ARE	AAACCA	1357	6 +	Zea mays	anaerobic induction
TaMAPK30	GC-motif	CCCCCG	575	6 +	Zea mays	anoxic specific inducibility
TaMAPK30	CAT-box	GCCACT	788	6 +	Arabidopsis thaliana	meristem expression
TaMAPK30	CAT-box	GCCACT	976	6 +	Arabidopsis thaliana	meristem expression
TaMAPK31	MBS	CAACTG	700	6 -	Arabidopsis thaliana	drought inducibility

TaMAPK31	GARE-motif	TCTGTTG	1039	7 +	Brassica oleracea	gibberellin responsive
TaMAPK31	O2-site	GATGACATGG	1211	9 -	Zea mays	zein metabolism regulation
TaMAPK31	TGACG-motif	TGACG	747	5 +	Hordeum vulgare	the MeJA responsive
TaMAPK31	TGACG-motif	TGACG	1226	5 -	Hordeum vulgare	the MeJA responsive
TaMAPK31	GC-motif	CCCCCG	104	6 -	Zea mays	anoxic specific inducibility
TaMAPK31	GC-motif	CCCCCG	119	6 -	Zea mays	anoxic specific inducibility
TaMAPK31	GC-motif	CCCCCG	613	6 -	Zea mays	anoxic specific inducibility
TaMAPK31	ARE	AAACCA	581	6 -	Zea mays	anaerobic induction
TaMAPK31	ARE	AAACCA	1568	6 -	Zea mays	anaerobic induction
TaMAPK31	NON-box	AGATCGACG	562	9 +	Arabidopsis thaliana	meristem specific activation
TaMAPK31	ABRE	ACGTG	294	5 +	Arabidopsis thaliana	abscisic acid responsive
TaMAPK31	ABRE	ACGTG	777	5 +	Arabidopsis thaliana	abscisic acid responsive
TaMAPK31	ABRE	ACGTG	1155	5 -	Arabidopsis thaliana	abscisic acid responsive
TaMAPK31	ABRE	ACGTG	1692	5 +	Arabidopsis thaliana	abscisic acid responsive
TaMAPK31	CGTCA-motif	CGTCA	747	5 -	Hordeum vulgare	the MeJA responsive
TaMAPK31	CGTCA-motif	CGTCA	1226	5 +	Hordeum vulgare	the MeJA responsive
TaMAPK31	TATC-box	TATCCCA	1150	7 +	Oryza sativa	gibberellin responsive
TaMAPK31	GCN4_motif	TGAGTCA	850	7 +	Oryza sativa	endosperm expression
TaMAPK31	GCN4_motif	TGAGTCA	1738	7 -	Oryza sativa	endosperm expression

TaMAPK33	CGTCA-motif	CGTCA	956	5 -	Hordeum vulgare	the MeJA responsive
TaMAPK33	CGTCA-motif	CGTCA	1313	5 -	Hordeum vulgare	the MeJA responsive
TaMAPK33	CGTCA-motif	CGTCA	1414	5 +	Hordeum vulgare	the MeJA responsive
TaMAPK33	CGTCA-motif	CGTCA	1916	5 -	Hordeum vulgare	the MeJA responsive
TaMAPK33	CAT-box	GCCACT	438	6 -	Arabidopsis thaliana	meristem expression
TaMAPK33	CAT-box	GCCACT	661	6 +	Arabidopsis thaliana	meristem expression
TaMAPK33	CAT-box	GCCACT	1766	6 +	Arabidopsis thaliana	meristem expression
TaMAPK33	TGA-element	AACGAC	9	6 +	Brassica oleracea	auxin responsive
TaMAPK33	GC-motif	CCCCCG	186	6 +	Zea mays	anoxic specific inducibility
TaMAPK33	GC-motif	CCCCCG	1973	6 +	Zea mays	anoxic specific inducibility
TaMAPK33	ABRE	ACGTG	532	5 -	Arabidopsis thaliana	abscisic acid responsive
TaMAPK33	ABRE	CGCACGTGTC	1176	10 +	Hordeum vulgare	abscisic acid responsive
TaMAPK33	ABRE	CACGTG	1178	6 -	Arabidopsis thaliana	abscisic acid responsive
TaMAPK33	ABRE	ACGTG	1179	5 +	Arabidopsis thaliana	abscisic acid responsive
TaMAPK33	TGACG-motif	TGACG	956	5 +	Hordeum vulgare	the MeJA responsive
TaMAPK33	TGACG-motif	TGACG	1313	5 +	Hordeum vulgare	the MeJA responsive
TaMAPK33	TGACG-motif	TGACG	1414	5 -	Hordeum vulgare	the MeJA responsive
TaMAPK33	TGACG-motif	TGACG	1916	5 +	Hordeum vulgare	the MeJA responsive
TaMAPK33	LTR	CCGAAA	1708	6 +	Hordeum vulgare	low temperature responsive

TaMAPK33	GCN4_motif	TGAGTCA	928	7 +	Oryza sativa	endosperm expression
TaMAPK34	GC-motif	CCCCCG	1294	6 +	Zea mays	anoxic specific inducibility
TaMAPK34	GC-motif	CCCCCG	1839	6 +	Zea mays	anoxic specific inducibility
TaMAPK34	ABRE	CACGTG	32	6 +	Arabidopsis thaliana	abscisic acid responsive
TaMAPK34	ABRE	ACGTG	33	5 +	Arabidopsis thaliana	abscisic acid responsive
TaMAPK34	ABRE	CACGTG	147	6 +	Arabidopsis thaliana	abscisic acid responsive
TaMAPK34	ABRE	ACGTG	148	5 +	Arabidopsis thaliana	abscisic acid responsive
TaMAPK34	ABRE	ACGTG	170	5 +	Arabidopsis thaliana	abscisic acid responsive
TaMAPK34	ABRE	ACGTG	718	5 +	Arabidopsis thaliana	abscisic acid responsive
TaMAPK34	ABRE	CACGTG	1161	6 -	Arabidopsis thaliana	abscisic acid responsive
TaMAPK34	ABRE	ACGTG	1162	5 +	Arabidopsis thaliana	abscisic acid responsive
TaMAPK34	ABRE	ACGTG	1881	5 +	Arabidopsis thaliana	abscisic acid responsive
TaMAPK34	TC-rich repeats	ATTCTCTAAC	62	9 -	Nicotiana tabacum	defense and stress responsive
TaMAPK34	TGACG-motif	TGACG	151	5 +	Hordeum vulgare	the MeJA responsive
TaMAPK34	TGACG-motif	TGACG	336	5 -	Hordeum vulgare	the MeJA responsive
TaMAPK34	TGACG-motif	TGACG	422	5 -	Hordeum vulgare	the MeJA responsive
TaMAPK34	AuxRR-core	GGTCCAT	1049	7 -	Nicotiana tabacum	auxin responsive
TaMAPK34	TGA-element	AACGAC	1302	6 +	Brassica oleracea	auxin responsive
TaMAPK34	CGTCA-motif	CGTCA	151	5 -	Hordeum vulgare	the MeJA responsive

TaMAPK34	CGTCA-motif	CGTCA	336	5 +	Hordeum vulgare	the MeJA responsive
TaMAPK34	CGTCA-motif	CGTCA	422	5 +	Hordeum vulgare	the MeJA responsive
TaMAPK34	CAT-box	GCCACT	828	6 -	Arabidopsis thaliana	meristem expression
TaMAPK34	MBS	CAACTG	372	6 +	Arabidopsis thaliana	drought inducibility
TaMAPK34	MBS	CAACTG	1503	6 -	Arabidopsis thaliana	drought inducibility
TaMAPK34	ARE	AAACCA	730	6 -	Zea mays	anaerobic induction
TaMAPK34	ARE	AAACCA	1675	6 +	Zea mays	anaerobic induction
TaMAPK34	TCA-element	CCATCTTTTT	400	9 -	Nicotiana tabacum	salicylic acid responsive
TaMAPK34	TCA-element	CCATCTTTTT	904	9 +	Nicotiana tabacum	salicylic acid responsive
TaMAPK35	GARE-motif	TCTGTTG	1629	7 +	Brassica oleracea	gibberellin responsive
TaMAPK35	TCA-element	CCATCTTTTT	262	9 +	Nicotiana tabacum	salicylic acid responsive
TaMAPK35	TCA-element	TCAGAAGAGG	1514	9 +	Brassica oleracea	salicylic acid responsive
TaMAPK35	TATC-box	TATCCCA	879	7 +	Oryza sativa	gibberellin responsive
TaMAPK35	TGA-element	AACGAC	1940	6 -	Brassica oleracea	auxin responsive
TaMAPK35	TGACG-motif	TGACG	1946	5 -	Hordeum vulgare	the MeJA responsive
TaMAPK35	CGTCA-motif	CGTCA	1946	5 +	Hordeum vulgare	the MeJA responsive
TaMAPK35	ARE	AAACCA	1430	6 -	Zea mays	anaerobic induction
TaMAPK35	ARE	AAACCA	1546	6 -	Zea mays	anaerobic induction
TaMAPK35	ARE	AAACCA	1783	6 -	Zea mays	anaerobic induction

TaMAPK36	MBS	CAACTG	1412	6 +	Arabidopsis thaliana	drought inducibility
TaMAPK36	TGACG-motif	TGACG	1004	5 +	Hordeum vulgare	the MeJA responsive
TaMAPK36	TGACG-motif	TGACG	1380	5 -	Hordeum vulgare	the MeJA responsive
TaMAPK36	ARE	AAACCA	1162	6 -	Zea mays	anaerobic induction
TaMAPK36	ARE	AAACCA	1636	6 -	Zea mays	anaerobic induction
TaMAPK36	ABRE	CGCACGTGTC	292	9 +	Hordeum vulgare	abscisic acid responsive
TaMAPK36	ABRE	CACGTG	294	6 +	Arabidopsis thaliana	abscisic acid responsive
TaMAPK36	ABRE	ACGTG	295	5 +	Arabidopsis thaliana	abscisic acid responsive
TaMAPK36	GC-motif	CCCCCG	1077	6 -	Zea mays	anoxic specific inducibility
TaMAPK36	CGTCA-motif	CGTCA	1004	5 -	Hordeum vulgare	the MeJA responsive
TaMAPK36	CGTCA-motif	CGTCA	1380	5 +	Hordeum vulgare	the MeJA responsive
TaMAPK36	GCN4_motif	TGAGTCA	86	7 -	Oryza sativa	endosperm expression
TaMAPK36	CAT-box	GCCACT	1264	6 -	Arabidopsis thaliana	meristem expression
TaMAPK36	O2-site	GATGACATGG	1424	9 +	Zea mays	zein metabolism regulation
TaMAPK36	O2-site	GTTGACGTGA	1718	9 -	Zea mays	zein metabolism regulation
TaMAPK37	ARE	AAACCA	874	6 -	Zea mays	anaerobic induction
TaMAPK37	ARE	AAACCA	1757	6 +	Zea mays	anaerobic induction
TaMAPK37	O2-site	GATGACATGG	989	10 -	Zea mays	zein metabolism regulation
TaMAPK37	O2-site	GATGATGTGG	1023	9 -	Zea mays	zein metabolism regulation

TaMAPK37	TGACG-motif	TGACG	891	5 -	Hordeum vulgare	the MeJA responsive
TaMAPK37	TGACG-motif	TGACG	1783	5 +	Hordeum vulgare	the MeJA responsive
TaMAPK37	CGTCA-motif	CGTCA	891	5 +	Hordeum vulgare	the MeJA responsive
TaMAPK37	CGTCA-motif	CGTCA	1783	5 -	Hordeum vulgare	the MeJA responsive
TaMAPK37	TGA-element	AACGAC	528	6 -	Brassica oleracea	auxin responsive
TaMAPK37	TGA-element	AACGAC	1241	6 +	Brassica oleracea	auxin responsive
TaMAPK37	ABRE	CACGTG	730	6 +	Arabidopsis thaliana	abscisic acid responsive
TaMAPK37	ABRE	ACGTG	731	5 +	Arabidopsis thaliana	abscisic acid responsive
TaMAPK37	MBS	CAACTG	894	6 -	Arabidopsis thaliana	drought inducibility
TaMAPK37	TATC-box	TATCCCA	449	7 -	Oryza sativa	gibberellin responsive
TaMAPK38	TGA-element	AACGAC	1930	6 -	Brassica oleracea	auxin responsive
TaMAPK38	ABRE	ACGTG	31	5 -	Arabidopsis thaliana	abscisic acid responsive
TaMAPK38	ABRE	ACGTG	76	5 -	Arabidopsis thaliana	abscisic acid responsive
TaMAPK38	ABRE	ACGTG	92	5 -	Arabidopsis thaliana	abscisic acid responsive
TaMAPK38	ARE	AAACCA	1274	6 +	Zea mays	anaerobic induction
TaMAPK38	circadian	CAAAGATATC	524	9 +	Lycopersicon esculentum	circadian control
TaMAPK38	GC-motif	CCCCCG	1956	6 -	Zea mays	anoxic specific inducibility
TaMAPK38	TCA-element	TCAGAAGAGG	1430	9 +	Brassica oleracea	salicylic acid responsive
TaMAPK38	P-box	CCTTTTG	158	7 -	Oryza sativa	gibberellin responsive
TaMAPK38	P-box	CCTTTTG	1301	7 -	Oryza sativa	gibberellin responsive

TaMAPK38	LTR	CCGAAA	878	6 +	Hordeum vulgare	low temperature responsive
TaMAPK39	TGACG-motif	TGACG	622	5 -	Hordeum vulgare	the MeJA responsive
TaMAPK39	TGACG-motif	TGACG	1077	5 -	Hordeum vulgare	the MeJA responsive
TaMAPK39	TGACG-motif	TGACG	1640	5 -	Hordeum vulgare	the MeJA responsive
TaMAPK39	ARE	AAACCA	107	6 +	Zea mays	anaerobic induction
TaMAPK39	ARE	AAACCA	215	6 +	Zea mays	anaerobic induction
TaMAPK39	ARE	AAACCA	318	6 +	Zea mays	anaerobic induction
TaMAPK39	ARE	AAACCA	1449	6 -	Zea mays	anaerobic induction
TaMAPK39	ARE	AAACCA	1759	6 +	Zea mays	anaerobic induction
TaMAPK39	ABRE	GACACGTGGC	514	9 +	Triticum aestivum	abscisic acid responsive
TaMAPK39	ABRE	CACGTG	516	6 +	Arabidopsis thaliana	abscisic acid responsive
TaMAPK39	ABRE	ACGTG	517	5 +	Arabidopsis thaliana	abscisic acid responsive
TaMAPK39	ABRE	ACGTG	699	5 -	Arabidopsis thaliana	abscisic acid responsive
TaMAPK39	ABRE	AACCCGG	785	7 -	Arabidopsis thaliana	abscisic acid responsive
TaMAPK39	ABRE	AACCCGG	897	7 -	Arabidopsis thaliana	abscisic acid responsive
TaMAPK39	ABRE	GCCGCGTGGC	1512	9 -	Oryza sativa	abscisic acid responsive
TaMAPK39	ABRE	GCCGCGTGGC	1592	9 -	Oryza sativa	abscisic acid responsive
TaMAPK39	CAT-box	GCCACT	285	6 +	Arabidopsis thaliana	meristem expression
TaMAPK39	LTR	CCGAAA	891	6 -	Hordeum vulgare	low temperature responsive

TaMAPK39	CGTCA-motif	CGTCA	622	5 +	Hordeum vulgare	the MeJA responsive
TaMAPK39	CGTCA-motif	CGTCA	1077	5 +	Hordeum vulgare	the MeJA responsive
TaMAPK39	CGTCA-motif	CGTCA	1640	5 +	Hordeum vulgare	the MeJA responsive
TaMAPK39	MBS	CAACTG	435	6 -	Arabidopsis thaliana	drought inducibility
TaMAPK39	TCA-element	CCATCTTTTT	1022	9 -	Nicotiana tabacum	salicylic acid responsive
TaMAPK39	O2-site	GATGATGTGG	377	9 -	Zea mays	zein metabolism regulation
TaMAPK39	O2-site	GATGATGTGG	752	9 +	Zea mays	zein metabolism regulation
TaMAPK39	O2-site	GATGACATGG	992	9 +	Zea mays	zein metabolism regulation
TaMAPK39	O2-site	GATGACATGG	1126	9 +	Zea mays	zein metabolism regulation
TaMAPK39	GC-motif	CCCCCG	1912	6 -	Zea mays	anoxic specific inducibility
TaMAPK4	CGTCA-motif	CGTCA	581	5 +	Hordeum vulgare	the MeJA responsive
TaMAPK4	CGTCA-motif	CGTCA	1760	5 -	Hordeum vulgare	the MeJA responsive
TaMAPK4	ABRE	ACGTG	579	5 -	Arabidopsis thaliana	abscisic acid responsive
TaMAPK4	ABRE	CACGTG	1698	6 -	Arabidopsis thaliana	abscisic acid responsive
TaMAPK4	ABRE	ACGTG	1699	5 +	Arabidopsis thaliana	abscisic acid responsive
TaMAPK4	GC-motif	CCCCCG	614	6 +	Zea mays	anoxic specific inducibility
TaMAPK4	GC-motif	CCCCCG	994	6 +	Zea mays	anoxic specific inducibility
TaMAPK4	GC-motif	CCCCCG	1033	6 -	Zea mays	anoxic specific inducibility
TaMAPK4	TC-rich repeats	GTTTTCTTAC	52	9 +	Nicotiana tabacum	defense and stress responsive

TaMAPK4	TGACG-motif	TGACG	581	5 -	Hordeum vulgare	the MeJA responsive
TaMAPK4	TGACG-motif	TGACG	1760	5 +	Hordeum vulgare	the MeJA responsive
TaMAPK4	ARE	AAACCA	50	6 -	Zea mays	anaerobic induction
TaMAPK4	ARE	AAACCA	257	6 -	Zea mays	anaerobic induction
TaMAPK4	ARE	AAACCA	1420	6 -	Zea mays	anaerobic induction
TaMAPK4	circadian	CAAAGATATC	494	9 +	Lycopersicon esculentum	circadian control
TaMAPK4	GARE-motif	TCTGTTG	548	7 -	Brassica oleracea	gibberellin responsive
TaMAPK4	GCN4_motif	TGAGTCA	1963	7 -	Oryza sativa	endosperm expression
TaMAPK4	O2-site	GATGACATGG	1923	9 -	Zea mays	zein metabolism regulation
TaMAPK4	LTR	CCGAAA	792	6 +	Hordeum vulgare	low temperature responsive
TaMAPK40	O2-site	GATGATGTGG	320	9 -	Zea mays	zein metabolism regulation
TaMAPK40	GC-motif	CCCCCG	1304	6 +	Zea mays	anoxic specific inducibility
TaMAPK40	MBS	CAACTG	870	6 -	Arabidopsis thaliana	drought inducibility
TaMAPK40	CGTCA-motif	CGTCA	134	5 -	Hordeum vulgare	the MeJA responsive
TaMAPK40	CGTCA-motif	CGTCA	1144	5 +	Hordeum vulgare	the MeJA responsive
TaMAPK40	LTR	CCGAAA	1165	6 +	Hordeum vulgare	low temperature responsive
TaMAPK40	ABRE	CACGTG	256	6 +	Arabidopsis thaliana	abscisic acid responsive
TaMAPK40	ABRE	ACGTG	257	5 +	Arabidopsis thaliana	abscisic acid responsive
TaMAPK40	ABRE	GCCGCGTGGC	741	9 +	Oryza sativa	abscisic acid responsive

TaMAPK40	ABRE	ACGTG	990	5 +	Arabidopsis thaliana	abscisic acid responsive
TaMAPK40	ABRE	GACACGTACG T	1086	10 -	Oryza sativa	abscisic acid responsive
TaMAPK40	ABRE	TACGTGTC	1089	8 +	Oryza sativa	abscisic acid responsive
TaMAPK40	ABRE	ACGTG	1090	5 +	Arabidopsis thaliana	abscisic acid responsive
TaMAPK40	ABRE	CACGTG	1218	6 -	Arabidopsis thaliana	abscisic acid responsive
TaMAPK40	ABRE	ACGTG	1219	5 +	Arabidopsis thaliana	abscisic acid responsive
TaMAPK40	ABRE	CACGTG	1340	6 -	Arabidopsis thaliana	abscisic acid responsive
TaMAPK40	ABRE	ACGTG	1341	5 +	Arabidopsis thaliana	abscisic acid responsive
TaMAPK40	GARE-motif	TCTGTTG	835	7 +	Brassica oleracea	gibberellin responsive
TaMAPK40	GARE-motif	TCTGTTG	1123	7 +	Brassica oleracea	gibberellin responsive
TaMAPK40	TGACG-motif	TGACG	134	5 +	Hordeum vulgare	the MeJA responsive
TaMAPK40	TGACG-motif	TGACG	1144	5 -	Hordeum vulgare	the MeJA responsive
TaMAPK41	P-box	CCTTTTG	971	7 -	Oryza sativa	gibberellin responsive
TaMAPK41	ABRE	ACGTG	1114	5 -	Arabidopsis thaliana	abscisic acid responsive
TaMAPK41	ABRE	ACGTG	1130	5 -	Arabidopsis thaliana	abscisic acid responsive
TaMAPK41	ABRE	ACGTG	1169	5 +	Arabidopsis thaliana	abscisic acid responsive
TaMAPK41	ABRE	ACGTG	1454	5 -	Arabidopsis thaliana	abscisic acid responsive
TaMAPK41	CAT-box	GCCACT	195	6 -	Arabidopsis thaliana	meristem expression
TaMAPK41	CAT-box	GCCACT	1563	6 -	Arabidopsis thaliana	meristem expression

TaMAPK41	RY-element	CATGCATG	962	8 +	Helianthus annuus	seed specific regulation
TaMAPK41	ARE	AAACCA	42	6 -	Zea mays	anaerobic induction
TaMAPK41	O2-site	GATGATGTGG	946	9 -	Zea mays	zein metabolism regulation
TaMAPK41	GARE-motif	TCTGTTG	580	7 +	Brassica oleracea	gibberellin responsive
TaMAPK42	TATC-box	TATCCCA	1446	7 -	Oryza sativa	gibberellin responsive
TaMAPK42	HD-Zip 1	CAAT(A/T)ATTG	1243	8 -	Arabidopsis thaliana	differentiation of the palisade mesophyll cells
TaMAPK42	TGACG-motif	TGACG	1757	5 -	Hordeum vulgare	the MeJA responsive
TaMAPK42	CGTCA-motif	CGTCA	1757	5 +	Hordeum vulgare	the MeJA responsive
TaMAPK42	P-box	CCTTTTG	1652	7 +	Oryza sativa	gibberellin responsive
TaMAPK42	ABRE	ACGTG	1737	5 +	Arabidopsis thaliana	abscisic acid responsive
TaMAPK42	ABRE	ACGTG	1809	5 -	Arabidopsis thaliana	abscisic acid responsive
TaMAPK43	GC-motif	CCCCCG	1106	6 -	Zea mays	anoxic specific inducibility
TaMAPK43	GC-motif	CCCCCG	1917	6 -	Zea mays	anoxic specific inducibility
TaMAPK43	ABRE	CGTACGTGCA	153	9 -	Hordeum vulgare	abscisic acid responsive
TaMAPK43	ABRE	ACGTG	155	5 -	Arabidopsis thaliana	abscisic acid responsive
TaMAPK43	ABRE	AACCCGG	956	7 +	Arabidopsis thaliana	abscisic acid responsive
TaMAPK43	ABRE	AACCCGG	1003	7 +	Arabidopsis thaliana	abscisic acid responsive
TaMAPK43	ABRE	ACGTG	1875	5 +	Arabidopsis thaliana	abscisic acid responsive

TaMAPK43	TC-rich repeats	GTTTTCTTAC	923	9 +	Nicotiana tabacum	defense and stress responsive
TaMAPK43	TGACG-motif	TGACG	394	5 -	Hordeum vulgare	the MeJA responsive
TaMAPK43	TGACG-motif	TGACG	778	5 +	Hordeum vulgare	the MeJA responsive
TaMAPK43	TGA-element	AACGAC	1387	6 +	Brassica oleracea	auxin responsive
TaMAPK43	CGTCA-motif	CGTCA	394	5 +	Hordeum vulgare	the MeJA responsive
TaMAPK43	CGTCA-motif	CGTCA	778	5 -	Hordeum vulgare	the MeJA responsive
TaMAPK43	P-box	CCTTTTG	657	7 -	Oryza sativa	gibberellin responsive
TaMAPK43	ARE	AAACCA	161	6 -	Zea mays	anaerobic induction
TaMAPK43	ARE	AAACCA	1068	6 +	Zea mays	anaerobic induction
TaMAPK43	ARE	AAACCA	1708	6 +	Zea mays	anaerobic induction
TaMAPK43	TATC-box	TATCCCA	994	7 -	Oryza sativa	gibberellin responsive
TaMAPK43	O2-site	GATGA(C/T)(A/G)TG(A/G)	49	8 +	Zea mays	zein metabolism regulation
TaMAPK44	CGTCA-motif	CGTCA	756	5 +	Hordeum vulgare	the MeJA responsive
TaMAPK44	CGTCA-motif	CGTCA	1698	5 +	Hordeum vulgare	the MeJA responsive
TaMAPK44	TGACG-motif	TGACG	756	5 -	Hordeum vulgare	the MeJA responsive
TaMAPK44	TGACG-motif	TGACG	1698	5 -	Hordeum vulgare	the MeJA responsive
TaMAPK44	LTR	CCGAAA	1898	6 +	Hordeum vulgare	low temperature responsive
TaMAPK44	TATC-box	TATCCCA	1372	7 +	Oryza sativa	gibberellin responsive
TaMAPK44	circadian	CAAAGATATC	295	9 -	Lycopersicon esculentum	circadian control

TaMAPK44	TCA-element	CCATCTTTTT	744	9 +	Nicotiana tabacum	salicylic acid responsive
TaMAPK44	P-box	CCTTTTG	1529	7 +	Oryza sativa	gibberellin responsive
TaMAPK44	ABRE	ACGTG	693	5 +	Arabidopsis thaliana	abscisic acid responsive
TaMAPK44	ABRE	ACGTG	1318	5 +	Arabidopsis thaliana	abscisic acid responsive
TaMAPK44	ABRE	ACGTG	1696	5 -	Arabidopsis thaliana	abscisic acid responsive
TaMAPK45	O2-site	GATGACATGG	1432	9 +	Zea mays	zein metabolism regulation
TaMAPK45	GC-motif	CCCCCG	1091	6 -	Zea mays	anoxic specific inducibility
TaMAPK45	ABRE	CGCACGTGTC	307	9 +	Hordeum vulgare	abscisic acid responsive
TaMAPK45	ABRE	CACGTG	309	6 +	Arabidopsis thaliana	abscisic acid responsive
TaMAPK45	ABRE	ACGTG	310	5 +	Arabidopsis thaliana	abscisic acid responsive
TaMAPK45	ARE	AAACCA	1176	6 -	Zea mays	anaerobic induction
TaMAPK45	MBS	CAACTG	1420	6 +	Arabidopsis thaliana	drought inducibility
TaMAPK45	GCN4_motif	TGAGTCA	126	7 -	Oryza sativa	endosperm expression
TaMAPK46	TC-rich repeats	GTTTTCTTAC	935	9 +	Nicotiana tabacum	defense and stress responsive
TaMAPK46	GCN4_motif	TGAGTCA	1739	7 -	Oryza sativa	endosperm expression
TaMAPK46	LTR	CCGAAA	41	6 +	Hordeum vulgare	low temperature responsive
TaMAPK46	MSA-like	TCCAACGGT	1786	9 +	Catharanthus roseus	cell cycle regulation
TaMAPK46	ARE	AAACCA	73	6 +	Zea mays	anaerobic induction
TaMAPK46	ARE	AAACCA	737	6 -	Zea mays	anaerobic induction

TaMAPK46	ARE	AAACCA	1570	6 -	Zea mays	anaerobic induction
TaMAPK46	ABRE	ACGTG	1	5 +	Arabidopsis thaliana	abscisic acid responsive
TaMAPK46	ABRE	ACGTG	116	5 -	Arabidopsis thaliana	abscisic acid responsive
TaMAPK46	ABRE	ACGTG	729	5 +	Arabidopsis thaliana	abscisic acid responsive
TaMAPK46	ABRE	ACGTG	1159	5 -	Arabidopsis thaliana	abscisic acid responsive
TaMAPK46	ABRE	ACGTG	1694	5 +	Arabidopsis thaliana	abscisic acid responsive
TaMAPK46	GC-motif	CCCCCG	433	6 -	Zea mays	anoxic specific inducibility
TaMAPK46	GC-motif	CCCCCG	534	6 -	Zea mays	anoxic specific inducibility
TaMAPK46	GC-motif	CCCCCG	616	6 -	Zea mays	anoxic specific inducibility
TaMAPK46	O2-site	GATGA(C/T)(A/G)TG(A/G)	949	8 +	Zea mays	zein metabolism regulation
TaMAPK46	O2-site	GATGACATGG	1215	9 -	Zea mays	zein metabolism regulation
TaMAPK46	TATC-box	TATCCCA	1154	7 +	Oryza sativa	gibberellin responsive
TaMAPK46	CGTCA-motif	CGTCA	1018	5 +	Hordeum vulgare	the MeJA responsive
TaMAPK46	CGTCA-motif	CGTCA	1230	5 +	Hordeum vulgare	the MeJA responsive
TaMAPK46	TGACG-motif	TGACG	1018	5 -	Hordeum vulgare	the MeJA responsive
TaMAPK46	TGACG-motif	TGACG	1230	5 -	Hordeum vulgare	the MeJA responsive
TaMAPK46	P-box	CCTTTTG	810	7 -	Oryza sativa	gibberellin responsive
TaMAPK47	LTR	CCGAAA	47	6 +	Hordeum vulgare	low temperature responsive
TaMAPK47	MBS	CAACTG	221	6 +	Arabidopsis thaliana	drought inducibility

TaMAPK47	ABRE	ACGTG	999	5 -	Arabidopsis thaliana	abscisic acid responsive
TaMAPK47	ABRE	GACACGTGGC	1950	9 -	Triticum aestivum	abscisic acid responsive
TaMAPK47	ABRE	CACGTG	1952	6 -	Arabidopsis thaliana	abscisic acid responsive
TaMAPK47	ABRE	ACGTG	1953	5 +	Arabidopsis thaliana	abscisic acid responsive
TaMAPK47	ARE	AAACCA	51	6 +	Zea mays	anaerobic induction
TaMAPK47	ARE	AAACCA	1207	6 +	Zea mays	anaerobic induction
TaMAPK47	TGACG-motif	TGACG	255	5 -	Hordeum vulgare	the MeJA responsive
TaMAPK47	TGACG-motif	TGACG	1031	5 +	Hordeum vulgare	the MeJA responsive
TaMAPK47	TGACG-motif	TGACG	1716	5 -	Hordeum vulgare	the MeJA responsive
TaMAPK47	TGACG-motif	TGACG	1766	5 -	Hordeum vulgare	the MeJA responsive
TaMAPK47	CGTCA-motif	CGTCA	255	5 +	Hordeum vulgare	the MeJA responsive
TaMAPK47	CGTCA-motif	CGTCA	1031	5 -	Hordeum vulgare	the MeJA responsive
TaMAPK47	CGTCA-motif	CGTCA	1716	5 +	Hordeum vulgare	the MeJA responsive
TaMAPK47	CGTCA-motif	CGTCA	1766	5 +	Hordeum vulgare	the MeJA responsive
TaMAPK47	TATC-box	TATCCCA	992	7 -	Oryza sativa	gibberellin responsive
TaMAPK49	GCN4_motif	TGAGTCA	73	7 +	Oryza sativa	endosperm expression
TaMAPK49	LTR	CCGAAA	562	6 +	Hordeum vulgare	low temperature responsive
TaMAPK49	LTR	CCGAAA	1030	6 +	Hordeum vulgare	low temperature responsive
TaMAPK49	NON-box	AGATCGACG	1455	9 +	Arabidopsis thaliana	meristem specific activation

TaMAPK49	CAT-box	GCCACT	1881	6 -	Arabidopsis thaliana	meristem expression
TaMAPK49	TCA-element	CCATCTTTTT	194	9 -	Nicotiana tabacum	salicylic acid responsive
TaMAPK49	TCA-element	CCATCTTTTT	777	9 -	Nicotiana tabacum	salicylic acid responsive
TaMAPK49	GC-motif	CCCCCG	1567	6 +	Zea mays	anoxic specific inducibility
TaMAPK49	O2-site	GATGACATGG	1021	9 -	Zea mays	zein metabolism regulation
TaMAPK49	ABRE	ACGTG	299	5 -	Arabidopsis thaliana	abscisic acid responsive
TaMAPK49	ABRE	ACGTG	638	5 -	Arabidopsis thaliana	abscisic acid responsive
TaMAPK49	P-box	CCTTTTG	253	7 -	Oryza sativa	gibberellin responsive
TaMAPK49	P-box	CCTTTTG	933	7 +	Oryza sativa	gibberellin responsive
TaMAPK49	TGACG-motif	TGACG	474	5 -	Hordeum vulgare	the MeJA responsive
TaMAPK49	TGACG-motif	TGACG	640	5 -	Hordeum vulgare	the MeJA responsive
TaMAPK49	TGACG-motif	TGACG	942	5 +	Hordeum vulgare	the MeJA responsive
TaMAPK49	TGACG-motif	TGACG	1297	5 +	Hordeum vulgare	the MeJA responsive
TaMAPK49	TGACG-motif	TGACG	1315	5 -	Hordeum vulgare	the MeJA responsive
TaMAPK49	TGACG-motif	TGACG	1629	5 -	Hordeum vulgare	the MeJA responsive
TaMAPK49	CGTCA-motif	CGTCA	474	5 +	Hordeum vulgare	the MeJA responsive
TaMAPK49	CGTCA-motif	CGTCA	640	5 +	Hordeum vulgare	the MeJA responsive
TaMAPK49	CGTCA-motif	CGTCA	942	5 -	Hordeum vulgare	the MeJA responsive
TaMAPK49	CGTCA-motif	CGTCA	1297	5 -	Hordeum vulgare	the MeJA responsive

TaMAPK49	CGTCA-motif	CGTCA	1315	5	+	Hordeum vulgare	the MeJA responsive
TaMAPK49	CGTCA-motif	CGTCA	1629	5	+	Hordeum vulgare	the MeJA responsive
TaMAPK5	LTR	CCGAAA	655	6	-	Hordeum vulgare	low temperature responsive
TaMAPK5	LTR	CCGAAA	737	6	-	Hordeum vulgare	low temperature responsive
TaMAPK5	LTR	CCGAAA	904	6	-	Hordeum vulgare	low temperature responsive
TaMAPK5	ARE	AAACCA	1031	6	+	Zea mays	anaerobic induction
TaMAPK5	ARE	AAACCA	1255	6	-	Zea mays	anaerobic induction
TaMAPK5	TGACG-motif	TGACG	797	5	+	Hordeum vulgare	the MeJA responsive
TaMAPK5	TGACG-motif	TGACG	1520	5	-	Hordeum vulgare	the MeJA responsive
TaMAPK5	MBSI	TTTTTACGGTTA	901	11	+	Petunia hybrida	flavonoid biosynthetic genes regulation
TaMAPK5	TGA-box	TGACGTAA	797	8	+	Glycine max	auxin responsive
TaMAPK5	ABRE	ACGTG	515	5	-	Arabidopsis thaliana	abscisic acid responsive
TaMAPK5	ABRE	ACGTG	691	5	-	Arabidopsis thaliana	abscisic acid responsive
TaMAPK5	ABRE	ACGTG	886	5	-	Arabidopsis thaliana	abscisic acid responsive
TaMAPK5	ABRE	CACGTG	1286	6	-	Arabidopsis thaliana	abscisic acid responsive
TaMAPK5	ABRE	ACGTG	1287	5	+	Arabidopsis thaliana	abscisic acid responsive
TaMAPK5	ABRE	ACGTG	1742	5	+	Arabidopsis thaliana	abscisic acid responsive
TaMAPK5	ABRE	ACGTG	1804	5	-	Arabidopsis thaliana	abscisic acid responsive
TaMAPK5	GC-motif	CCCCCG	1856	6	+	Zea mays	anoxic specific inducibility

TaMAPK5	CGTCA-motif	CGTCA	797	5 -	Hordeum vulgare	the MeJA responsive
TaMAPK5	CGTCA-motif	CGTCA	1520	5 +	Hordeum vulgare	the MeJA responsive
TaMAPK5	TGA-element	AACGAC	694	6 -	Brassica oleracea	auxin responsive
TaMAPK50	TC-rich repeats	GTTTTCTTAC	1945	9 +	Nicotiana tabacum	defense and stress responsive
TaMAPK50	TGACG-motif	TGACG	41	5 -	Hordeum vulgare	the MeJA responsive
TaMAPK50	TGACG-motif	TGACG	1904	5 -	Hordeum vulgare	the MeJA responsive
TaMAPK50	CGTCA-motif	CGTCA	41	5 +	Hordeum vulgare	the MeJA responsive
TaMAPK50	CGTCA-motif	CGTCA	1904	5 +	Hordeum vulgare	the MeJA responsive
TaMAPK50	LTR	CCGAAA	197	6 +	Hordeum vulgare	low temperature responsive
TaMAPK50	LTR	CCGAAA	844	6 -	Hordeum vulgare	low temperature responsive
TaMAPK50	LTR	CCGAAA	997	6 -	Hordeum vulgare	low temperature responsive
TaMAPK50	CAT-box	GCCACT	1415	6 +	Arabidopsis thaliana	meristem expression
TaMAPK50	GARE-motif	TCTGTTG	1494	7 +	Brassica oleracea	gibberellin responsive
TaMAPK50	MBS	CAACTG	1807	6 -	Arabidopsis thaliana	drought inducibility
TaMAPK50	TCA-element	TCAGAAGAGG	1062	9 -	Brassica oleracea	salicylic acid responsive
TaMAPK50	TCA-element	CCATCTTTTT	1424	9 +	Nicotiana tabacum	salicylic acid responsive
TaMAPK50	ABRE	CACGTG	1450	6 -	Arabidopsis thaliana	abscisic acid responsive
TaMAPK50	ABRE	ACGTG	1451	5 +	Arabidopsis thaliana	abscisic acid responsive
TaMAPK50	ABRE	ACGTG	1712	5 +	Arabidopsis thaliana	abscisic acid responsive
TaMAPK50	TGA-element	AACGAC	1264	6 +	Brassica oleracea	auxin responsive

TaMAPK50	TGA-element	AACGAC	1994	6 +	Brassica oleracea	auxin responsive
TaMAPK50	GC-motif	CCCCCG	214	6 +	Zea mays	anoxic specific inducibility
TaMAPK50	GC-motif	CCCCCG	768	6 -	Zea mays	anoxic specific inducibility
TaMAPK50	GC-motif	CCCCCG	1849	6 +	Zea mays	anoxic specific inducibility
TaMAPK52	LTR	CCGAAA	1147	6 +	Hordeum vulgare	low temperature responsive
TaMAPK52	GCN4_motif	TGAGTCA	276	7 +	Oryza sativa	endosperm expression
TaMAPK52	MBS	CAACTG	95	6 +	Arabidopsis thaliana	drought inducibility
TaMAPK52	ABRE	TACGGTC	1238	7 +	Arabidopsis thaliana	abscisic acid responsive
TaMAPK52	ABRE	ACGTG	1437	5 +	Arabidopsis thaliana	abscisic acid responsive
TaMAPK52	ABRE	AACCCGG	1767	7 +	Arabidopsis thaliana	abscisic acid responsive
TaMAPK52	ABRE	ACGTG	1796	5 -	Arabidopsis thaliana	abscisic acid responsive
TaMAPK52	ARE	AAACCA	657	6 -	Zea mays	anaerobic induction
TaMAPK52	O2-site	GATGACATGG	428	9 -	Zea mays	zein metabolism regulation
TaMAPK52	O2-site	GATGACATGG	1460	9 -	Zea mays	zein metabolism regulation
TaMAPK52	O2-site	GATGATGTGG	1630	9 -	Zea mays	zein metabolism regulation
TaMAPK52	CGTCA-motif	CGTCA	1428	5 +	Hordeum vulgare	the MeJA responsive
TaMAPK52	CGTCA-motif	CGTCA	1636	5 +	Hordeum vulgare	the MeJA responsive
TaMAPK52	GARE-motif	TCTGTTG	1166	7 -	Brassica oleracea	gibberellin responsive
TaMAPK52	CAT-box	GCCACT	360	6 +	Arabidopsis thaliana	meristem expression
TaMAPK52	TGA-element	AACGAC	1833	6 +	Brassica oleracea	auxin responsive

TaMAPK52	GC-motif	CCCCCG	415	6 +	Zea mays	anoxic specific inducibility
TaMAPK52	GC-motif	CCCCCG	1386	6 -	Zea mays	anoxic specific inducibility
TaMAPK52	TGACG-motif	TGACG	1428	5 -	Hordeum vulgare	the MeJA responsive
TaMAPK52	TGACG-motif	TGACG	1636	5 -	Hordeum vulgare	the MeJA responsive
TaMAPK53	GCN4_motif	TGAGTCA	768	7 +	Oryza sativa	endosperm expression
TaMAPK53	LTR	CCGAAA	1715	6 +	Hordeum vulgare	low temperature responsive
TaMAPK53	TC-rich repeats	GTTTTCTTAC	745	9 -	Nicotiana tabacum	defense and stress responsive
TaMAPK53	CAT-box	GCCACT	323	6 +	Arabidopsis thaliana	meristem expression
TaMAPK53	CAT-box	GCCACT	1881	6 -	Arabidopsis thaliana	meristem expression
TaMAPK53	O2-site	GATGATGTGG	974	10 +	Zea mays	zein metabolism regulation
TaMAPK53	ABRE	ACGTG	955	5 -	Arabidopsis thaliana	abscisic acid responsive
TaMAPK53	P-box	CCTTTTG	226	7 -	Oryza sativa	gibberellin responsive
TaMAPK53	CGTCA-motif	CGTCA	1629	5 +	Hordeum vulgare	the MeJA responsive
TaMAPK53	TGACG-motif	TGACG	1629	5 -	Hordeum vulgare	the MeJA responsive
TaMAPK54	GC-motif	CCCCCG	827	6 -	Zea mays	anoxic specific inducibility
TaMAPK54	GC-motif	CCCCCG	1281	6 +	Zea mays	anoxic specific inducibility
TaMAPK54	GC-motif	CCCCCG	1378	6 -	Zea mays	anoxic specific inducibility
TaMAPK54	O2-site	GATGACATGG	596	9 -	Zea mays	zein metabolism regulation
TaMAPK54	O2-site	GATGATGTGG	1032	9 -	Zea mays	zein metabolism regulation

TaMAPK54	ARE	AAACCA	232	6 +	Zea mays	anaerobic induction
TaMAPK54	ARE	AAACCA	584	6 +	Zea mays	anaerobic induction
TaMAPK54	TGA-element	AACGAC	713	6 +	Brassica oleracea	auxin responsive
TaMAPK54	ABRE	CGCACGTGTC	287	9 -	Hordeum vulgare	abscisic acid responsive
TaMAPK54	ABRE	ACGTG	430	5 +	Arabidopsis thaliana	abscisic acid responsive
TaMAPK54	ABRE	ACGTG	618	5 -	Arabidopsis thaliana	abscisic acid responsive
TaMAPK54	ABRE	CACGTG	847	6 +	Arabidopsis thaliana	abscisic acid responsive
TaMAPK54	ABRE	ACGTG	848	5 +	Arabidopsis thaliana	abscisic acid responsive
TaMAPK54	ABRE	GACACGTGGC	963	9 -	Triticum aestivum	abscisic acid responsive
TaMAPK54	ABRE	ACGTG	1154	5 +	Arabidopsis thaliana	abscisic acid responsive
TaMAPK54	ABRE	ACGTG	1294	5 +	Arabidopsis thaliana	abscisic acid responsive
TaMAPK54	RY-element	CATGCATG	236	8 +	Helianthus annuus	seed specific regulation
TaMAPK54	RY-element	CATGCATG	588	8 +	Helianthus annuus	seed specific regulation
TaMAPK54	TGACG-motif	TGACG	611	5 -	Hordeum vulgare	the MeJA responsive
TaMAPK54	TGACG-motif	TGACG	1124	5 -	Hordeum vulgare	the MeJA responsive
TaMAPK54	CGTCA-motif	CGTCA	611	5 +	Hordeum vulgare	the MeJA responsive
TaMAPK54	CGTCA-motif	CGTCA	1124	5 +	Hordeum vulgare	the MeJA responsive
TaMAPK54	GCN4_motif	TGAGTCA	514	7 -	Oryza sativa	endosperm expression
TaMAPK54	LTR	CCGAAA	1193	6 +	Hordeum vulgare	low temperature responsive

TaMAPK54	WUN-motif	AAATTTCCT	299	9 -	Brassica oleracea	wound responsive
TaMAPK6	GARE-motif	TCTGTTG	1314	7 -	Brassica oleracea	gibberellin responsive
TaMAPK6	O2-site	GATGACATGG	707	9 +	Zea mays	zein metabolism regulation
TaMAPK6	TGACG-motif	TGACG	456	5 -	Hordeum vulgare	the MeJA responsive
TaMAPK6	TGACG-motif	TGACG	653	5 +	Hordeum vulgare	the MeJA responsive
TaMAPK6	TGACG-motif	TGACG	736	5 -	Hordeum vulgare	the MeJA responsive
TaMAPK6	TGACG-motif	TGACG	1875	5 -	Hordeum vulgare	the MeJA responsive
TaMAPK6	ARE	AAACCA	1669	6 +	Zea mays	anaerobic induction
TaMAPK6	RY-element	CATGCATG	819	8 +	Helianthus annuus	seed specific regulation
TaMAPK6	MBS	CAACTG	199	6 +	Arabidopsis thaliana	drought inducibility
TaMAPK6	CGTCA-motif	CGTCA	456	5 +	Hordeum vulgare	the MeJA responsive
TaMAPK6	CGTCA-motif	CGTCA	653	5 -	Hordeum vulgare	the MeJA responsive
TaMAPK6	CGTCA-motif	CGTCA	736	5 +	Hordeum vulgare	the MeJA responsive
TaMAPK6	CGTCA-motif	CGTCA	1875	5 +	Hordeum vulgare	the MeJA responsive
TaMAPK6	TGA-element	AACGAC	1370	6 +	Brassica oleracea	auxin responsive
TaMAPK6	ABRE	TACGGTC	1373	7 -	Arabidopsis thaliana	abscisic acid responsive
TaMAPK6	GC-motif	CCCCCG	1361	6 +	Zea mays	anoxic specific inducibility
TaMAPK7	CAT-box	GCCACT	476	6 -	Arabidopsis thaliana	meristem expression
TaMAPK7	CAT-box	GCCACT	499	6 -	Arabidopsis thaliana	meristem expression

TaMAPK7	CAT-box	GCCACT	570	6 -	Arabidopsis thaliana	meristem expression
TaMAPK7	CAT-box	GCCACT	1094	6 +	Arabidopsis thaliana	meristem expression
TaMAPK7	AuxRR-core	GGTCCAT	748	7 +	Nicotiana tabacum	auxin responsive
TaMAPK7	LTR	CCGAAA	971	6 +	Hordeum vulgare	low temperature responsive
TaMAPK7	TGACG-motif	TGACG	1638	5 -	Hordeum vulgare	the MeJA responsive
TaMAPK7	CGTCA-motif	CGTCA	1638	5 +	Hordeum vulgare	the MeJA responsive
TaMAPK7	TGA-element	AACGAC	1068	6 +	Brassica oleracea	auxin responsive
TaMAPK7	TGA-element	AACGAC	1835	6 +	Brassica oleracea	auxin responsive
TaMAPK7	ABRE	ACGTG	195	5 +	Arabidopsis thaliana	abscisic acid responsive
TaMAPK7	ABRE	AACCCGG	1769	7 +	Arabidopsis thaliana	abscisic acid responsive
TaMAPK7	ABRE	ACGTG	1798	5 -	Arabidopsis thaliana	abscisic acid responsive
TaMAPK7	ARE	AAACCA	1350	6 +	Zea mays	anaerobic induction
TaMAPK7	ARE	AAACCA	1404	6 -	Zea mays	anaerobic induction
TaMAPK7	O2-site	GATGATGTGG	1632	9 -	Zea mays	zein metabolism regulation
TaMAPK7	GC-motif	CCCCCG	1228	6 +	Zea mays	anoxic specific inducibility
TaMAPK7	GC-motif	CCCCCG	1239	6 +	Zea mays	anoxic specific inducibility
TaMAPK8	TGACG-motif	TGACG	1359	5 -	Hordeum vulgare	the MeJA responsive
TaMAPK8	circadian	CAAAGATATC	522	9 +	Lycopersicon esculentum	circadian control
TaMAPK8	ARE	AAACCA	1289	6 +	Zea mays	anaerobic induction
TaMAPK8	ABRE	ACGTG	65	5 +	Arabidopsis thaliana	abscisic acid responsive

TaMAPK8	ABRE	ACGTG	107	5 -	Arabidopsis thaliana	abscisic acid responsive
TaMAPK8	ABRE	ACGTG	156	5 -	Arabidopsis thaliana	abscisic acid responsive
TaMAPK8	ABRE	ACGTG	172	5 -	Arabidopsis thaliana	abscisic acid responsive
TaMAPK8	ABRE	CACGTG	885	6 +	Arabidopsis thaliana	abscisic acid responsive
TaMAPK8	ABRE	ACGTG	886	5 +	Arabidopsis thaliana	abscisic acid responsive
TaMAPK8	ABRE	GCCGCGTGGC	1485	9 -	Oryza sativa	abscisic acid responsive
TaMAPK8	CGTCA-motif	CGTCA	1359	5 +	Hordeum vulgare	the MeJA responsive
TaMAPK8	LTR	CCGAAA	798	6 -	Hordeum vulgare	low temperature responsive
TaMAPK8	LTR	CCGAAA	1005	6 -	Hordeum vulgare	low temperature responsive
TaMAPK8	LTR	CCGAAA	1850	6 -	Hordeum vulgare	low temperature responsive
TaMAPK8	P-box	CCTTTTG	238	7 -	Oryza sativa	gibberellin responsive
TaMAPK8	P-box	CCTTTTG	1316	7 -	Oryza sativa	gibberellin responsive
TaMAPK8	P-box	TTCCAACAAA CCCC	1468	13 +	Petroselinum crispum	gibberellin responsive
TaMAPK8	GC-motif	CCCCCG	1770	6 +	Zea mays	anoxic specific inducibility
TaMAPK8	GC-motif	CCCCCG	1956	6 -	Zea mays	anoxic specific inducibility
TaMAPK8	TC-rich repeats	GTTTTCTTAC	462	9 +	Nicotiana tabacum	defense and stress responsive
TaMAPKK1	LTR	CCGAAA	1978	6 -	Hordeum vulgare	low temperature responsive
TaMAPKK1	ABRE	GACACGTACG T	14	10 -	Oryza sativa	abscisic acid responsive
TaMAPKK1	ABRE	CACGTG	400	6 +	Arabidopsis thaliana	abscisic acid responsive

TaMAPKK1	ABRE	ACGTG	401	5 +	Arabidopsis thaliana	abscisic acid responsive
TaMAPKK1	ABRE	CGCACGTGTC	679	9 +	Hordeum vulgare	abscisic acid responsive
TaMAPKK1	ABRE	ACGTG	681	5 -	Arabidopsis thaliana	abscisic acid responsive
TaMAPKK1	ABRE	CGCACGTGTC	1037	9 -	Hordeum vulgare	abscisic acid responsive
TaMAPKK1	ABRE	CACGTG	1039	6 -	Arabidopsis thaliana	abscisic acid responsive
TaMAPKK1	ABRE	ACGTG	1040	5 +	Arabidopsis thaliana	abscisic acid responsive
TaMAPKK1	ABRE	ACGTG	1160	5 +	Arabidopsis thaliana	abscisic acid responsive
TaMAPKK1	ABRE	CACGTG	1486	6 -	Arabidopsis thaliana	abscisic acid responsive
TaMAPKK1	ABRE	ACGTG	1487	5 +	Arabidopsis thaliana	abscisic acid responsive
TaMAPKK1	TGA-element	AACGAC	959	6 +	Brassica oleracea	auxin responsive
TaMAPKK1	ARE	AAACCA	1024	6 -	Zea mays	anaerobic induction
TaMAPKK1	GC-motif	CCCCCG	1665	6 -	Zea mays	anoxic specific inducibility
TaMAPKK1	GC-motif	CCCCCG	1728	6 +	Zea mays	anoxic specific inducibility
TaMAPKK1	CGTCA-motif	CGTCA	228	5 +	Hordeum vulgare	the MeJA responsive
TaMAPKK1	CGTCA-motif	CGTCA	492	5 -	Hordeum vulgare	the MeJA responsive
TaMAPKK1	CGTCA-motif	CGTCA	1172	5 +	Hordeum vulgare	the MeJA responsive
TaMAPKK1	CGTCA-motif	CGTCA	1278	5 +	Hordeum vulgare	the MeJA responsive
TaMAPKK1	CGTCA-motif	CGTCA	1735	5 +	Hordeum vulgare	the MeJA responsive
TaMAPKK1	TGACG-motif	TGACG	228	5 -	Hordeum vulgare	the MeJA responsive

TaMAPKK1	TGACG-motif	TGACG	492	5 +	Hordeum vulgare	the MeJA responsive
TaMAPKK1	TGACG-motif	TGACG	1172	5 -	Hordeum vulgare	the MeJA responsive
TaMAPKK1	TGACG-motif	TGACG	1278	5 -	Hordeum vulgare	the MeJA responsive
TaMAPKK1	TGACG-motif	TGACG	1735	5 -	Hordeum vulgare	the MeJA responsive
TaMAPKK11	CAT-box	GCCACT	42	6 -	Arabidopsis thaliana	meristem expression
TaMAPKK11	O2-site	GATGACATGG	570	10 -	Zea mays	zein metabolism regulation
TaMAPKK11	TGACG-motif	TGACG	26	5 -	Hordeum vulgare	the MeJA responsive
TaMAPKK11	TGACG-motif	TGACG	423	5 -	Hordeum vulgare	the MeJA responsive
TaMAPKK11	TGACG-motif	TGACG	1271	5 +	Hordeum vulgare	the MeJA responsive
TaMAPKK11	TGACG-motif	TGACG	1274	5 -	Hordeum vulgare	the MeJA responsive
TaMAPKK11	TGACG-motif	TGACG	1325	5 +	Hordeum vulgare	the MeJA responsive
TaMAPKK11	TGACG-motif	TGACG	1827	5 -	Hordeum vulgare	the MeJA responsive
TaMAPKK11	GC-motif	CCCCCG	1901	6 +	Zea mays	anoxic specific inducibility
TaMAPKK11	ARE	AAACCA	1084	6 +	Zea mays	anaerobic induction
TaMAPKK11	ARE	AAACCA	1639	6 -	Zea mays	anaerobic induction
TaMAPKK11	TATC-box	TATCCCA	1473	7 +	Oryza sativa	gibberellin responsive
TaMAPKK11	TCA-element	TCAGAAGAGG	774	9 -	Brassica oleracea	salicylic acid responsive
TaMAPKK11	LTR	CCGAAA	1904	6 +	Hordeum vulgare	low temperature responsive
TaMAPKK11	CGTCA-motif	CGTCA	26	5 +	Hordeum vulgare	the MeJA responsive

TaMAPKK11	CGTCA-motif	CGTCA	423	5 +	Hordeum vulgare	the MeJA responsive
TaMAPKK11	CGTCA-motif	CGTCA	1271	5 -	Hordeum vulgare	the MeJA responsive
TaMAPKK11	CGTCA-motif	CGTCA	1274	5 +	Hordeum vulgare	the MeJA responsive
TaMAPKK11	CGTCA-motif	CGTCA	1325	5 -	Hordeum vulgare	the MeJA responsive
TaMAPKK11	CGTCA-motif	CGTCA	1827	5 +	Hordeum vulgare	the MeJA responsive
TaMAPKK12	ARE	AAACCA	77	6 -	Zea mays	anaerobic induction
TaMAPKK12	TGACG-motif	TGACG	194	5 +	Hordeum vulgare	the MeJA responsive
TaMAPKK12	TGACG-motif	TGACG	1186	5 -	Hordeum vulgare	the MeJA responsive
TaMAPKK12	TGACG-motif	TGACG	1234	5 -	Hordeum vulgare	the MeJA responsive
TaMAPKK12	TGACG-motif	TGACG	1548	5 +	Hordeum vulgare	the MeJA responsive
TaMAPKK12	GARE-motif	TCTGTTG	1344	7 -	Brassica oleracea	gibberellin responsive
TaMAPKK12	LTR	CCGAAA	1291	6 -	Hordeum vulgare	low temperature responsive
TaMAPKK12	CGTCA-motif	CGTCA	194	5 -	Hordeum vulgare	the MeJA responsive
TaMAPKK12	CGTCA-motif	CGTCA	1186	5 +	Hordeum vulgare	the MeJA responsive
TaMAPKK12	CGTCA-motif	CGTCA	1234	5 +	Hordeum vulgare	the MeJA responsive
TaMAPKK12	CGTCA-motif	CGTCA	1548	5 -	Hordeum vulgare	the MeJA responsive
TaMAPKK12	AuxRR-core	GGTCCAT	261	7 -	Nicotiana tabacum	auxin responsive
TaMAPKK12	AuxRR-core	GGTCCAT	819	7 -	Nicotiana tabacum	auxin responsive
TaMAPKK12	ABRE	ACGTG	292	5 +	Arabidopsis thaliana	abscisic acid responsive

TaMAPKK12	ABRE	ACGTG	316	5 +	Arabidopsis thaliana	abscisic acid responsive
TaMAPKK12	ABRE	ACGTG	641	5 +	Arabidopsis thaliana	abscisic acid responsive
TaMAPKK12	ABRE	ACGTG	1472	5 -	Arabidopsis thaliana	abscisic acid responsive
TaMAPKK12	ABRE	ACGTG	1484	5 -	Arabidopsis thaliana	abscisic acid responsive
TaMAPKK12	ABRE	ACGTG	1626	5 -	Arabidopsis thaliana	abscisic acid responsive
TaMAPKK12	ABRE	ACGTG	1905	5 -	Arabidopsis thaliana	abscisic acid responsive
TaMAPKK13	MBS	CAACTG	1643	6 -	Arabidopsis thaliana	drought inducibility
TaMAPKK13	MBS	CAACTG	1804	6 +	Arabidopsis thaliana	drought inducibility
TaMAPKK13	ARE	AAACCA	846	6 +	Zea mays	anaerobic induction
TaMAPKK13	ARE	AAACCA	873	6 +	Zea mays	anaerobic induction
TaMAPKK13	ARE	AAACCA	1179	6 -	Zea mays	anaerobic induction
TaMAPKK13	TGACG-motif	TGACG	138	5 -	Hordeum vulgare	the MeJA responsive
TaMAPKK13	TGACG-motif	TGACG	1037	5 -	Hordeum vulgare	the MeJA responsive
TaMAPKK13	LTR	CCGAAA	767	6 +	Hordeum vulgare	low temperature responsive
TaMAPKK13	LTR	CCGAAA	900	6 -	Hordeum vulgare	low temperature responsive
TaMAPKK13	ABRE	AACCCGG	145	7 -	Arabidopsis thaliana	abscisic acid responsive
TaMAPKK13	ABRE	CACGTG	877	6 +	Arabidopsis thaliana	abscisic acid responsive
TaMAPKK13	ABRE	ACGTG	878	5 +	Arabidopsis thaliana	abscisic acid responsive
TaMAPKK13	ABRE	GCCGCGTGGC	1470	9 -	Oryza sativa	abscisic acid responsive

TaMAPKK13	GC-motif	CCCCCG	1400	6 +	Zea mays	anoxic specific inducibility
TaMAPKK13	GC-motif	CCCCCG	1437	6 +	Zea mays	anoxic specific inducibility
TaMAPKK13	GARE-motif	TCTGTTG	1709	7 +	Brassica oleracea	gibberellin responsive
TaMAPKK13	circadian	CAAAGATATC	1248	10 -	Lycopersicon esculentum	circadian control
TaMAPKK13	CAT-box	GCCACT	1374	6 -	Arabidopsis thaliana	meristem expression
TaMAPKK13	O2-site	GATGACATGG	1634	9 -	Zea mays	zein metabolism regulation
TaMAPKK13	TC-rich repeats	ATTCTCTAAC	1675	10 -	Nicotiana tabacum	defense and stress responsive
TaMAPKK13	CGTCA-motif	CGTCA	138	5 +	Hordeum vulgare	the MeJA responsive
TaMAPKK13	CGTCA-motif	CGTCA	1037	5 +	Hordeum vulgare	the MeJA responsive
TaMAPKK14	TCA-element	TCAGAAGAGG	271	9 +	Brassica oleracea	salicylic acid responsive
TaMAPKK14	TCA-element	CCATCTTTTT	552	9 +	Nicotiana tabacum	salicylic acid responsive
TaMAPKK14	LTR	CCGAAA	118	6 +	Hordeum vulgare	low temperature responsive
TaMAPKK14	CGTCA-motif	CGTCA	1349	5 -	Hordeum vulgare	the MeJA responsive
TaMAPKK14	ABRE	ACGTG	196	5 -	Arabidopsis thaliana	abscisic acid responsive
TaMAPKK14	ARE	AAACCA	181	6 +	Zea mays	anaerobic induction
TaMAPKK14	ARE	AAACCA	1452	6 +	Zea mays	anaerobic induction
TaMAPKK14	TGACG-motif	TGACG	1349	5 +	Hordeum vulgare	the MeJA responsive
TaMAPKK14	O2-site	GATGATGTGG	99	9 +	Zea mays	zein metabolism regulation
TaMAPKK14	O2-site	GATGATGTGG	698	9 -	Zea mays	zein metabolism regulation

TaMAPKK15	ABRE	ACGTG	170	5 -	Arabidopsis thaliana	abscisic acid responsive
TaMAPKK15	LTR	CCGAAA	92	6 +	Hordeum vulgare	low temperature responsive
TaMAPKK15	CGTCA-motif	CGTCA	1342	5 -	Hordeum vulgare	the MeJA responsive
TaMAPKK15	TCA-element	TCAGAAGAGG	245	9 +	Brassica oleracea	salicylic acid responsive
TaMAPKK15	TCA-element	CCATCTTTTT	539	9 +	Nicotiana tabacum	salicylic acid responsive
TaMAPKK15	GC-motif	CCCCCG	1679	6 -	Zea mays	anoxic specific inducibility
TaMAPKK15	ARE	AAACCA	155	6 +	Zea mays	anaerobic induction
TaMAPKK15	ARE	AAACCA	1443	6 +	Zea mays	anaerobic induction
TaMAPKK15	MBS	CAACTG	1186	6 -	Arabidopsis thaliana	drought inducibility
TaMAPKK15	O2-site	GATGATGTGG	685	9 -	Zea mays	zein metabolism regulation
TaMAPKK15	TGACG-motif	TGACG	1342	5 +	Hordeum vulgare	the MeJA responsive
TaMAPKK16	GC-motif	CCCCCG	1899	6 +	Zea mays	anoxic specific inducibility
TaMAPKK16	ARE	AAACCA	1401	6 -	Zea mays	anaerobic induction
TaMAPKK16	TGACG-motif	TGACG	839	5 -	Hordeum vulgare	the MeJA responsive
TaMAPKK16	TGACG-motif	TGACG	1835	5 -	Hordeum vulgare	the MeJA responsive
TaMAPKK16	O2-site	GATGACATGG	1022	9 -	Zea mays	zein metabolism regulation
TaMAPKK16	P-box	CCTTTTG	65	7 +	Oryza sativa	gibberellin responsive
TaMAPKK16	P-box	CCTTTTG	459	7 +	Oryza sativa	gibberellin responsive
TaMAPKK16	CAT-box	GCCACT	1149	6 +	Arabidopsis thaliana	meristem expression

TaMAPKK16	ABRE	AACCCGG	1477	7 -	Arabidopsis thaliana	abscisic acid responsive
TaMAPKK16	CGTCA-motif	CGTCA	839	5 +	Hordeum vulgare	the MeJA responsive
TaMAPKK16	CGTCA-motif	CGTCA	1835	5 +	Hordeum vulgare	the MeJA responsive
TaMAPKK16	LTR	CCGAAA	123	6 +	Hordeum vulgare	low temperature responsive
TaMAPKK16	LTR	CCGAAA	690	6 +	Hordeum vulgare	low temperature responsive
TaMAPKK16	LTR	CCGAAA	888	6 +	Hordeum vulgare	low temperature responsive
TaMAPKK16	LTR	CCGAAA	1135	6 +	Hordeum vulgare	low temperature responsive
TaMAPKK16	LTR	CCGAAA	1902	6 +	Hordeum vulgare	low temperature responsive
TaMAPKK16	TATC-box	TATCCCA	1539	7 +	Oryza sativa	gibberellin responsive
TaMAPKK16	circadian	CAAAGATATC	1592	9 +	Lycopersicon esculentum	circadian control
TaMAPKK17	circadian	CAAAGATATC	1814	9 -	Lycopersicon esculentum	circadian control
TaMAPKK17	TGA-element	AACGAC	1405	6 +	Brassica oleracea	auxin responsive
TaMAPKK17	AuxRR-core	GGTCCAT	168	7 -	Nicotiana tabacum	auxin responsive
TaMAPKK17	CGTCA-motif	CGTCA	101	5 -	Hordeum vulgare	the MeJA responsive
TaMAPKK17	CGTCA-motif	CGTCA	1463	5 -	Hordeum vulgare	the MeJA responsive
TaMAPKK17	LTR	CCGAAA	1289	6 -	Hordeum vulgare	low temperature responsive
TaMAPKK17	ABRE	ACGTG	199	5 +	Arabidopsis thaliana	abscisic acid responsive
TaMAPKK17	ABRE	ACGTG	223	5 +	Arabidopsis thaliana	abscisic acid responsive
TaMAPKK17	ABRE	GCAACGTGTC	1469	9 -	Hordeum vulgare	abscisic acid responsive

TaMAPKK17	ABRE	ACGTG	1471	5 -	Arabidopsis thaliana	abscisic acid responsive
TaMAPKK17	P-box	CCTTTTG	563	7 +	Oryza sativa	gibberellin responsive
TaMAPKK17	P-box	CCTTTTG	1333	7 +	Oryza sativa	gibberellin responsive
TaMAPKK17	TGACG-motif	TGACG	101	5 +	Hordeum vulgare	the MeJA responsive
TaMAPKK17	TGACG-motif	TGACG	1463	5 +	Hordeum vulgare	the MeJA responsive
TaMAPKK17	TC-rich repeats	GTTTTCTTAC	1157	9 +	Nicotiana tabacum	defense and stress responsive
TaMAPKK17	GARE-motif	TCTGTTG	1343	7 -	Brassica oleracea	gibberellin responsive
TaMAPKK17	ARE	AAACCA	551	6 -	Zea mays	anaerobic induction
TaMAPKK17	ARE	AAACCA	926	6 -	Zea mays	anaerobic induction
TaMAPKK17	ARE	AAACCA	1001	6 -	Zea mays	anaerobic induction
TaMAPKK17	ARE	AAACCA	1083	6 -	Zea mays	anaerobic induction
TaMAPKK17	ARE	AAACCA	1118	6 -	Zea mays	anaerobic induction
TaMAPKK17	ARE	AAACCA	1179	6 -	Zea mays	anaerobic induction
TaMAPKK18	TGACG-motif	TGACG	674	5 -	Hordeum vulgare	the MeJA responsive
TaMAPKK18	TGACG-motif	TGACG	923	5 -	Hordeum vulgare	the MeJA responsive
TaMAPKK18	TGACG-motif	TGACG	1090	5 +	Hordeum vulgare	the MeJA responsive
TaMAPKK18	TGACG-motif	TGACG	1378	5 -	Hordeum vulgare	the MeJA responsive
TaMAPKK18	TGACG-motif	TGACG	1603	5 +	Hordeum vulgare	the MeJA responsive
TaMAPKK18	CGTCA-motif	CGTCA	674	5 +	Hordeum vulgare	the MeJA responsive

TaMAPKK18	CGTCA-motif	CGTCA	923	5 +	Hordeum vulgare	the MeJA responsive
TaMAPKK18	CGTCA-motif	CGTCA	1090	5 -	Hordeum vulgare	the MeJA responsive
TaMAPKK18	CGTCA-motif	CGTCA	1378	5 +	Hordeum vulgare	the MeJA responsive
TaMAPKK18	CGTCA-motif	CGTCA	1603	5 -	Hordeum vulgare	the MeJA responsive
TaMAPKK18	LTR	CCGAAA	1703	6 -	Hordeum vulgare	low temperature responsive
TaMAPKK18	LTR	CCGAAA	1755	6 -	Hordeum vulgare	low temperature responsive
TaMAPKK18	CAT-box	GCCACT	297	6 -	Arabidopsis thaliana	meristem expression
TaMAPKK18	ARE	AAACCA	528	6 +	Zea mays	anaerobic induction
TaMAPKK18	TCA-element	CCATCTTTTT	212	9 +	Nicotiana tabacum	salicylic acid responsive
TaMAPKK18	P-box	CCTTTTG	216	7 +	Oryza sativa	gibberellin responsive
TaMAPKK18	ABRE	ACGTG	1254	5 +	Arabidopsis thaliana	abscisic acid responsive
TaMAPKK18	ABRE	CGCACGTGTC	1406	9 +	Hordeum vulgare	abscisic acid responsive
TaMAPKK18	ABRE	AACCCGG	1673	7 -	Arabidopsis thaliana	abscisic acid responsive
TaMAPKK18	GC-motif	CCCCCG	700	6 +	Zea mays	anoxic specific inducibility
TaMAPKK18	GC-motif	CCCCCG	737	6 +	Zea mays	anoxic specific inducibility
TaMAPKK18	GC-motif	CCCCCG	767	6 -	Zea mays	anoxic specific inducibility
TaMAPKK18	GC-motif	CCCCCG	1133	6 +	Zea mays	anoxic specific inducibility
TaMAPKK18	GC-motif	CCCCCG	1652	6 +	Zea mays	anoxic specific inducibility
TaMAPKK18	TGA-element	AACGAC	27	6 -	Brassica oleracea	auxin responsive
TaMAPKK18	TGA-element	AACGAC	1333	6 +	Brassica oleracea	auxin responsive

TaMAPKK2	TGA-element	AACGAC	1942	6 -	Brassica oleracea	auxin responsive
TaMAPKK2	ABRE	ACGTG	153	5 -	Arabidopsis thaliana	abscisic acid responsive
TaMAPKK2	ABRE	GACACGTGGC	618	9 -	Triticum aestivum	abscisic acid responsive
TaMAPKK2	ABRE	CACGTG	620	6 +	Arabidopsis thaliana	abscisic acid responsive
TaMAPKK2	ABRE	ACGTG	621	5 +	Arabidopsis thaliana	abscisic acid responsive
TaMAPKK2	ABRE	ACGTG	665	5 -	Arabidopsis thaliana	abscisic acid responsive
TaMAPKK2	ABRE	CACGTG	927	6 +	Arabidopsis thaliana	abscisic acid responsive
TaMAPKK2	ABRE	ACGTG	928	5 +	Arabidopsis thaliana	abscisic acid responsive
TaMAPKK2	ABRE	ACGTG	982	5 +	Arabidopsis thaliana	abscisic acid responsive
TaMAPKK2	ABRE	CACGTG	993	6 +	Arabidopsis thaliana	abscisic acid responsive
TaMAPKK2	ABRE	ACGTG	994	5 +	Arabidopsis thaliana	abscisic acid responsive
TaMAPKK2	ABRE	CACGTG	1021	6 -	Arabidopsis thaliana	abscisic acid responsive
TaMAPKK2	ABRE	ACGTG	1022	5 +	Arabidopsis thaliana	abscisic acid responsive
TaMAPKK2	MBS	CAACTG	675	6 +	Arabidopsis thaliana	drought inducibility
TaMAPKK2	MBS	CAACTG	743	6 +	Arabidopsis thaliana	drought inducibility
TaMAPKK2	MBS	CAACTG	749	6 -	Arabidopsis thaliana	drought inducibility
TaMAPKK2	MBS	CAACTG	1710	6 +	Arabidopsis thaliana	drought inducibility
TaMAPKK2	circadian	CAAAGATATC	1905	9 -	Lycopersicon esculentum	circadian control
TaMAPKK2	ARE	AAACCA	842	6 -	Zea mays	anaerobic induction

TaMAPKK2	ARE	AAACCA	1600	6 +	Zea mays	anaerobic induction
TaMAPKK2	LTR	CCGAAA	334	6 +	Hordeum vulgare	low temperature responsive
TaMAPKK2	TGACG-motif	TGACG	155	5 -	Hordeum vulgare	the MeJA responsive
TaMAPKK2	TGACG-motif	TGACG	444	5 +	Hordeum vulgare	the MeJA responsive
TaMAPKK2	TC-rich repeats	GTTTTCTTAC	1661	9 -	Nicotiana tabacum	defense and stress responsive
TaMAPKK2	CGTCA-motif	CGTCA	155	5 +	Hordeum vulgare	the MeJA responsive
TaMAPKK2	CGTCA-motif	CGTCA	444	5 -	Hordeum vulgare	the MeJA responsive
TaMAPKK3	P-box	CCTTTTG	1640	7 +	Oryza sativa	gibberellin responsive
TaMAPKK3	TCA-element	CCATCTTTTT	1476	9 +	Nicotiana tabacum	salicylic acid responsive
TaMAPKK3	TCA-element	CCATCTTTTT	1636	9 +	Nicotiana tabacum	salicylic acid responsive
TaMAPKK3	GARE-motif	TCTGTTG	73	7 +	Brassica oleracea	gibberellin responsive
TaMAPKK3	MBS	CAACTG	221	6 +	Arabidopsis thaliana	drought inducibility
TaMAPKK3	ABRE	ACGTG	339	5 -	Arabidopsis thaliana	abscisic acid responsive
TaMAPKK3	ABRE	GCCGCGTGGC	1088	9 -	Oryza sativa	abscisic acid responsive
TaMAPKK3	TGA-element	AACGAC	581	6 +	Brassica oleracea	auxin responsive
TaMAPKK3	GC-motif	CCCCCG	635	6 +	Zea mays	anoxic specific inducibility
TaMAPKK3	GC-motif	CCCCCG	1270	6 +	Zea mays	anoxic specific inducibility
TaMAPKK3	CGTCA-motif	CGTCA	822	5 -	Hordeum vulgare	the MeJA responsive
TaMAPKK3	CGTCA-motif	CGTCA	853	5 +	Hordeum vulgare	the MeJA responsive

TaMAPKK3	TGACG-motif	TGACG	822	5 +	Hordeum vulgare	the MeJA responsive
TaMAPKK3	TGACG-motif	TGACG	853	5 -	Hordeum vulgare	the MeJA responsive
TaMAPKK3	O2-site	GATGACATGG	744	9 -	Zea mays	zein metabolism regulation
TaMAPKK3	O2-site	GATGA(C/T)(A/G)TG(A/G)	1456	8.5 -	Zea mays	zein metabolism regulation
TaMAPKK3	O2-site	GATGACATGG	1508	9 +	Zea mays	zein metabolism regulation
TaMAPKK3	CAT-box	GCCACT	162	6 +	Arabidopsis thaliana	meristem expression
TaMAPKK3	CAT-box	GCCACT	351	6 +	Arabidopsis thaliana	meristem expression
TaMAPKK3	CAT-box	GCCACT	1721	6 -	Arabidopsis thaliana	meristem expression
TaMAPKK4	CGTCA-motif	CGTCA	104	5 -	Hordeum vulgare	the MeJA responsive
TaMAPKK4	CGTCA-motif	CGTCA	132	5 +	Hordeum vulgare	the MeJA responsive
TaMAPKK4	CGTCA-motif	CGTCA	228	5 +	Hordeum vulgare	the MeJA responsive
TaMAPKK4	CGTCA-motif	CGTCA	399	5 -	Hordeum vulgare	the MeJA responsive
TaMAPKK4	CGTCA-motif	CGTCA	471	5 +	Hordeum vulgare	the MeJA responsive
TaMAPKK4	CGTCA-motif	CGTCA	486	5 +	Hordeum vulgare	the MeJA responsive
TaMAPKK4	CGTCA-motif	CGTCA	1678	5 -	Hordeum vulgare	the MeJA responsive
TaMAPKK4	ABRE	GCCGCGTGGC	187	9 +	Oryza sativa	abscisic acid responsive
TaMAPKK4	ABRE	AACCCGG	275	7 -	Arabidopsis thaliana	abscisic acid responsive
TaMAPKK4	ABRE	ACGTG	527	5 +	Arabidopsis thaliana	abscisic acid responsive
TaMAPKK4	ABRE	GCAACGTGTC	555	9 -	Hordeum vulgare	abscisic acid responsive

TaMAPKK4	ABRE	ACGTG	1154	5 +	Arabidopsis thaliana	abscisic acid responsive
TaMAPKK4	TCA-element	CCATCTTTTT	1605	9 +	Nicotiana tabacum	salicylic acid responsive
TaMAPKK4	GC-motif	CCCCCG	1641	6 +	Zea mays	anoxic specific inducibility
TaMAPKK4	ARE	AAACCA	1233	6 -	Zea mays	anaerobic induction
TaMAPKK4	TGACG-motif	TGACG	104	5 +	Hordeum vulgare	the MeJA responsive
TaMAPKK4	TGACG-motif	TGACG	132	5 -	Hordeum vulgare	the MeJA responsive
TaMAPKK4	TGACG-motif	TGACG	228	5 -	Hordeum vulgare	the MeJA responsive
TaMAPKK4	TGACG-motif	TGACG	399	5 +	Hordeum vulgare	the MeJA responsive
TaMAPKK4	TGACG-motif	TGACG	471	5 -	Hordeum vulgare	the MeJA responsive
TaMAPKK4	TGACG-motif	TGACG	486	5 -	Hordeum vulgare	the MeJA responsive
TaMAPKK4	TGACG-motif	TGACG	1678	5 +	Hordeum vulgare	the MeJA responsive
TaMAPKK4	O2-site	GTTGACGTGA	985	9 +	Zea mays	zein metabolism regulation
TaMAPKK4	GARE-motif	TCTGTTG	1171	7 +	Brassica oleracea	gibberellin responsive
TaMAPKK5	TCA-element	CCATCTTTTT	551	9 +	Nicotiana tabacum	salicylic acid responsive
TaMAPKK5	ABRE	ACGTG	675	5 -	Arabidopsis thaliana	abscisic acid responsive
TaMAPKK5	CGTCA-motif	CGTCA	677	5 +	Hordeum vulgare	the MeJA responsive
TaMAPKK5	CGTCA-motif	CGTCA	1326	5 -	Hordeum vulgare	the MeJA responsive
TaMAPKK5	CAT-box	GCCACT	1852	6 +	Arabidopsis thaliana	meristem expression
TaMAPKK5	MBS	CAACTG	945	6 +	Arabidopsis thaliana	drought inducibility

TaMAPKK5	TGACG-motif	TGACG	677	5 -	Hordeum vulgare	the MeJA responsive
TaMAPKK5	TGACG-motif	TGACG	1326	5 +	Hordeum vulgare	the MeJA responsive
TaMAPKK6	TGA-box	TGACGTAA	1186	8 -	Glycine max	auxin responsive
TaMAPKK6	ABRE	TACGGTC	958	7 -	Arabidopsis thaliana	abscisic acid responsive
TaMAPKK6	ABRE	CGCACGTGTC	971	9 +	Hordeum vulgare	abscisic acid responsive
TaMAPKK6	ABRE	ACGTG	1550	5 -	Arabidopsis thaliana	abscisic acid responsive
TaMAPKK6	ABRE	ACGTG	1695	5 -	Arabidopsis thaliana	abscisic acid responsive
TaMAPKK6	ABRE	GACACGTACG T	1696	10 -	Oryza sativa	abscisic acid responsive
TaMAPKK6	ABRE	ACGTG	1700	5 +	Arabidopsis thaliana	abscisic acid responsive
TaMAPKK6	ABRE	GACACGTGGC	1704	9 +	Triticum aestivum	abscisic acid responsive
TaMAPKK6	ABRE	ACGTG	1868	5 -	Arabidopsis thaliana	abscisic acid responsive
TaMAPKK6	CGTCA-motif	CGTCA	355	5 -	Hordeum vulgare	the MeJA responsive
TaMAPKK6	CGTCA-motif	CGTCA	784	5 -	Hordeum vulgare	the MeJA responsive
TaMAPKK6	CGTCA-motif	CGTCA	799	5 -	Hordeum vulgare	the MeJA responsive
TaMAPKK6	CGTCA-motif	CGTCA	1127	5 -	Hordeum vulgare	the MeJA responsive
TaMAPKK6	CGTCA-motif	CGTCA	1189	5 +	Hordeum vulgare	the MeJA responsive
TaMAPKK6	CGTCA-motif	CGTCA	1552	5 +	Hordeum vulgare	the MeJA responsive
TaMAPKK6	CGTCA-motif	CGTCA	1962	5 -	Hordeum vulgare	the MeJA responsive
TaMAPKK6	LTR	CCGAAA	1438	6 -	Hordeum vulgare	low temperature responsive
TaMAPKK6	TGA-element	AACGAC	955	6 +	Brassica oleracea	auxin responsive

TaMAPKK6	CAT-box	GCCACT	475	6 -	Arabidopsis thaliana	meristem expression
TaMAPKK6	GARE-motif	TCTGTTG	175	7 +	Brassica oleracea	gibberellin responsive
TaMAPKK6	MBS	CAACTG	1555	6 -	Arabidopsis thaliana	drought inducibility
TaMAPKK6	O2-site	GATGATGTGG	1199	9 -	Zea mays	zein metabolism regulation
TaMAPKK6	TGACG-motif	TGACG	355	5 +	Hordeum vulgare	the MeJA responsive
TaMAPKK6	TGACG-motif	TGACG	784	5 +	Hordeum vulgare	the MeJA responsive
TaMAPKK6	TGACG-motif	TGACG	799	5 +	Hordeum vulgare	the MeJA responsive
TaMAPKK6	TGACG-motif	TGACG	1127	5 +	Hordeum vulgare	the MeJA responsive
TaMAPKK6	TGACG-motif	TGACG	1189	5 -	Hordeum vulgare	the MeJA responsive
TaMAPKK6	TGACG-motif	TGACG	1552	5 -	Hordeum vulgare	the MeJA responsive
TaMAPKK6	TGACG-motif	TGACG	1962	5 +	Hordeum vulgare	the MeJA responsive
TaMAPKK7	MBS	CAACTG	53	6 -	Arabidopsis thaliana	drought inducibility
TaMAPKK7	MBSI	TTTTTACGGTTA	825	11 +	Petunia hybrida	flavonoid biosynthetic genes regulation
TaMAPKK7	ABRE	AACCCGG	725	7 -	Arabidopsis thaliana	abscisic acid responsive
TaMAPKK7	ABRE	CACGTG	1104	6 -	Arabidopsis thaliana	abscisic acid responsive
TaMAPKK7	ABRE	ACGTG	1105	5 +	Arabidopsis thaliana	abscisic acid responsive
TaMAPKK7	ABRE	GCAACGTGTC	1434	9 -	Hordeum vulgare	abscisic acid responsive
TaMAPKK7	ABRE	ACGTG	1436	5 -	Arabidopsis thaliana	abscisic acid responsive

TaMAPKK7	ABRE	ACGTG	1449	5 -	Arabidopsis thaliana	abscisic acid responsive
TaMAPKK7	ABRE	ACGTG	1532	5 -	Arabidopsis thaliana	abscisic acid responsive
TaMAPKK7	ABRE	ACGTG	1886	5 -	Arabidopsis thaliana	abscisic acid responsive
TaMAPKK7	AuxRR-core	GGTCCAT	370	7 -	Nicotiana tabacum	auxin responsive
TaMAPKK7	TCA-element	CCATCTTTTT	1165	9 +	Nicotiana tabacum	salicylic acid responsive
TaMAPKK7	TGACG-motif	TGACG	828	5 +	Hordeum vulgare	the MeJA responsive
TaMAPKK7	TGACG-motif	TGACG	1995	5 +	Hordeum vulgare	the MeJA responsive
TaMAPKK7	GC-motif	CCCCCG	704	6 -	Zea mays	anoxic specific inducibility
TaMAPKK7	ARE	AAACCA	319	6 +	Zea mays	anaerobic induction
TaMAPKK7	CAT-box	GCCACT	1190	6 -	Arabidopsis thaliana	meristem expression
TaMAPKK7	CAT-box	GCCACT	1550	6 +	Arabidopsis thaliana	meristem expression
TaMAPKK7	LTR	CCGAAA	1250	6 -	Hordeum vulgare	low temperature responsive
TaMAPKK7	CGTCA-motif	CGTCA	828	5 -	Hordeum vulgare	the MeJA responsive
TaMAPKK7	CGTCA-motif	CGTCA	1995	5 -	Hordeum vulgare	the MeJA responsive
TaMAPKK8	motif I	gGTACGTGGCG	1053	10 -	Oryza sativa	root specific
TaMAPKK8	CAT-box	GCCACT	543	6 +	Arabidopsis thaliana	meristem expression
TaMAPKK8	GC-motif	CCCCCG	493	6 +	Zea mays	anoxic specific inducibility
TaMAPKK8	GC-motif	CCCCCG	497	6 -	Zea mays	anoxic specific inducibility
TaMAPKK8	GC-motif	CCCCCG	963	6 +	Zea mays	anoxic specific inducibility

TaMAPKK8	TGACG-motif	TGACG	55	5 +	Hordeum vulgare	the MeJA responsive
TaMAPKK8	TGACG-motif	TGACG	549	5 -	Hordeum vulgare	the MeJA responsive
TaMAPKK8	TGACG-motif	TGACG	1476	5 +	Hordeum vulgare	the MeJA responsive
TaMAPKK8	TGACG-motif	TGACG	1835	5 -	Hordeum vulgare	the MeJA responsive
TaMAPKK8	O2-site	GATGACATGG	79	9 +	Zea mays	zein metabolism regulation
TaMAPKK8	O2-site	GATGACATGG	1030	9 +	Zea mays	zein metabolism regulation
TaMAPKK8	ABRE	ACGTG	57	5 +	Arabidopsis thaliana	abscisic acid responsive
TaMAPKK8	ABRE	ACGTG	435	5 -	Arabidopsis thaliana	abscisic acid responsive
TaMAPKK8	ABRE	TACGGTC	463	7 +	Arabidopsis thaliana	abscisic acid responsive
TaMAPKK8	CGTCA-motif	CGTCA	55	5 -	Hordeum vulgare	the MeJA responsive
TaMAPKK8	CGTCA-motif	CGTCA	549	5 +	Hordeum vulgare	the MeJA responsive
TaMAPKK8	CGTCA-motif	CGTCA	1476	5 -	Hordeum vulgare	the MeJA responsive
TaMAPKK8	CGTCA-motif	CGTCA	1835	5 +	Hordeum vulgare	the MeJA responsive
TaMAPKK8	TGA-element	AACGAC	944	6 +	Brassica oleracea	auxin responsive
TaMAPKK9	ABRE	ACGTG	832	5 -	Arabidopsis thaliana	abscisic acid responsive
TaMAPKK9	ABRE	ACGTG	873	5 +	Arabidopsis thaliana	abscisic acid responsive
TaMAPKK9	ABRE	CACGTG	899	6 +	Arabidopsis thaliana	abscisic acid responsive
TaMAPKK9	ABRE	ACGTG	900	5 +	Arabidopsis thaliana	abscisic acid responsive
TaMAPKK9	ABRE	ACGTG	1864	5 -	Arabidopsis thaliana	abscisic acid responsive

TaMAPKK9	ABRE	ACGTG	1977	5 -	Arabidopsis thaliana	abscisic acid responsive
TaMAPKK9	ARE	AAACCA	496	6 +	Zea mays	anaerobic induction
TaMAPKK9	TGACG-motif	TGACG	1534	5 -	Hordeum vulgare	the MeJA responsive
TaMAPKK9	TCA-element	CCATCTTTTT	797	9 -	Nicotiana tabacum	salicylic acid responsive
TaMAPKK9	TCA-element	CCATCTTTTT	984	9 +	Nicotiana tabacum	salicylic acid responsive
TaMAPKK9	O2-site	GATGATGTGG	1129	9 -	Zea mays	zein metabolism regulation
TaMAPKK9	GCN4_motif	TGAGTCA	1	7 -	Oryza sativa	endosperm expression
TaMAPKK9	MBS	CAACTG	79	6 -	Arabidopsis thaliana	drought inducibility
TaMAPKK9	MBS	CAACTG	171	6 -	Arabidopsis thaliana	drought inducibility
TaMAPKK9	MBS	CAACTG	201	6 +	Arabidopsis thaliana	drought inducibility
TaMAPKK9	MBS	CAACTG	250	6 -	Arabidopsis thaliana	drought inducibility
TaMAPKK9	MBS	CAACTG	280	6 +	Arabidopsis thaliana	drought inducibility
TaMAPKK9	MBS	CAACTG	359	6 +	Arabidopsis thaliana	drought inducibility
TaMAPKK9	MBS	CAACTG	416	6 -	Arabidopsis thaliana	drought inducibility
TaMAPKK9	MBS	CAACTG	436	6 +	Arabidopsis thaliana	drought inducibility
TaMAPKK9	MBS	CAACTG	565	6 +	Arabidopsis thaliana	drought inducibility
TaMAPKK9	MBS	CAACTG	610	6 +	Arabidopsis thaliana	drought inducibility
TaMAPKK9	MBS	CAACTG	632	6 +	Arabidopsis thaliana	drought inducibility
TaMAPKK9	MBS	CAACTG	674	6 +	Arabidopsis thaliana	drought inducibility

TaMAPKK9	MBS	CAACTG	1285	6 -	Arabidopsis thaliana	drought inducibility
TaMAPKK9	CGTCA-motif	CGTCA	1534	5 +	Hordeum vulgare	the MeJA responsive
TaMAPKKK1	TCA-element	CCATCTTTTT	1024	9 +	Nicotiana tabacum	salicylic acid responsive
TaMAPKKK1	TCA-element	TCAGAAGAGG	1902	9 -	Brassica oleracea	salicylic acid responsive
TaMAPKKK1	ARE	AAACCA	143	6 +	Zea mays	anaerobic induction
TaMAPKKK1	ARE	AAACCA	976	6 +	Zea mays	anaerobic induction
TaMAPKKK1	ARE	AAACCA	1727	6 -	Zea mays	anaerobic induction
TaMAPKKK1	ARE	AAACCA	1818	6 -	Zea mays	anaerobic induction
TaMAPKKK1	LTR	CCGAAA	714	6 +	Hordeum vulgare	low temperature responsive
TaMAPKKK1	CAT-box	GCCACT	859	6 -	Arabidopsis thaliana	meristem expression
TaMAPKKK1	P-box	CCTTTTG	318	7 +	Oryza sativa	gibberellin responsive
TaMAPKKK1	P-box	CCTTTTG	1669	7 +	Oryza sativa	gibberellin responsive
TaMAPKKK1	ABRE	ACGTG	1582	5 -	Arabidopsis thaliana	abscisic acid responsive
TaMAPKKK1	GC-motif	CCCCCG	1103	6 +	Zea mays	anoxic specific inducibility
TaMAPKKK1	GC-motif	CCCCCG	1587	6 -	Zea mays	anoxic specific inducibility
TaMAPKKK1	MBS	CAACTG	947	6 -	Arabidopsis thaliana	drought inducibility
TaMAPKKK10	TC-rich repeats	ATTCTCTAAC	1619	9 -	Nicotiana tabacum	defense and stress responsive
TaMAPKKK10	TGACG-motif	TGACG	1155	5 -	Hordeum vulgare	the MeJA responsive
TaMAPKKK10	TGACG-motif	TGACG	1400	5 +	Hordeum vulgare	the MeJA responsive

TaMAPKKK10	TGACG-motif	TGACG	1661	5 +	Hordeum vulgare	the MeJA responsive
TaMAPKKK10	O2-site	GTTGACGTGA	1398	9 +	Zea mays	zein metabolism regulation
TaMAPKKK10	GARE-motif	TCTGTTG	408	7 -	Brassica oleracea	gibberellin responsive
TaMAPKKK10	MBS	CAACTG	429	6 +	Arabidopsis thaliana	drought inducibility
TaMAPKKK10	MBS	CAACTG	508	6 +	Arabidopsis thaliana	drought inducibility
TaMAPKKK10	MBS	CAACTG	935	6 -	Arabidopsis thaliana	drought inducibility
TaMAPKKK10	ARE	AAACCA	1632	6 +	Zea mays	anaerobic induction
TaMAPKKK10	ARE	AAACCA	1772	6 +	Zea mays	anaerobic induction
TaMAPKKK10	GC-motif	CCCCCG	1439	6 +	Zea mays	anoxic specific inducibility
TaMAPKKK10	GC-motif	CCCCCG	1731	6 -	Zea mays	anoxic specific inducibility
TaMAPKKK10	GC-motif	CCCCCG	1931	6 +	Zea mays	anoxic specific inducibility
TaMAPKKK10	GC-motif	CCCCCG	1983	6 +	Zea mays	anoxic specific inducibility
TaMAPKKK10	CAT-box	GCCACT	1316	6 +	Arabidopsis thaliana	meristem expression
TaMAPKKK10	P-box	CCTTTTG	68	7 -	Oryza sativa	gibberellin responsive
TaMAPKKK10	CGTCA-motif	CGTCA	1155	5 +	Hordeum vulgare	the MeJA responsive
TaMAPKKK10	CGTCA-motif	CGTCA	1400	5 -	Hordeum vulgare	the MeJA responsive
TaMAPKKK10	CGTCA-motif	CGTCA	1661	5 -	Hordeum vulgare	the MeJA responsive
TaMAPKKK10	ABRE	AACCCGG	713	7 +	Arabidopsis thaliana	abscisic acid responsive
TaMAPKKK10	ABRE	GCCGCGTGGC	1348	9 +	Oryza sativa	abscisic acid responsive

TaMAPKKK10	ABRE	ACGTG	1360	5 +		Arabidopsis thaliana	abscisic acid responsive
TaMAPKKK10	ABRE	ACGTG	1402	5 +		Arabidopsis thaliana	abscisic acid responsive
TaMAPKKK10	TCA-element	TCAGAAGAGG	566	9 +		Brassica oleracea	salicylic acid responsive
TaMAPKKK100	MBS	CAACTG	789	6 +		Arabidopsis thaliana	drought inducibility
TaMAPKKK100	P-box	CCTTTTG	1625	7 +		Oryza sativa	gibberellin responsive
TaMAPKKK100	motif I	gGTACGTGGCG	824	10 -		Oryza sativa	root specific
TaMAPKKK100	GC-motif	CCCCCG	1312	6 +		Zea mays	anoxic specific inducibility
TaMAPKKK100	GC-motif	CCCCCG	1662	6 -		Zea mays	anoxic specific inducibility
TaMAPKKK100	CGTCA-motif	CGTCA	943	5 +		Hordeum vulgare	the MeJA responsive
TaMAPKKK100	AuxRR-core	GGTCCAT	1981	7 +		Nicotiana tabacum	auxin responsive
TaMAPKKK100	GARE-motif	TCTGTTG	692	7 +		Brassica oleracea	gibberellin responsive
TaMAPKKK100	ABRE	GCAACGTGTC	24	9 +		Hordeum vulgare	abscisic acid responsive
TaMAPKKK100	ABRE	ACGTG	27	5 +		Arabidopsis thaliana	abscisic acid responsive
TaMAPKKK100	ABRE	CACGTG	64	6 +		Arabidopsis thaliana	abscisic acid responsive
TaMAPKKK100	ABRE	ACGTG	65	5 +		Arabidopsis thaliana	abscisic acid responsive
TaMAPKKK100	ABRE	TACGTGTC	768	8 -		Oryza sativa	abscisic acid responsive
TaMAPKKK100	ABRE	ACGTG	770	5 -		Arabidopsis thaliana	abscisic acid responsive
TaMAPKKK100	ABRE	ACGTG	827	5 -		Arabidopsis thaliana	abscisic acid responsive
TaMAPKKK100	ABRE	CACGTG	875	6 +		Arabidopsis thaliana	abscisic acid responsive

TaMAPKKK100	ABRE	ACGTG	876	5 +	Arabidopsis thaliana	abscisic acid responsive
TaMAPKKK100	ABRE	ACGTG	1392	5 -	Arabidopsis thaliana	abscisic acid responsive
TaMAPKKK100	ABRE	ACGTG	1460	5 -	Arabidopsis thaliana	abscisic acid responsive
TaMAPKKK100	ABRE	ACGTG	1492	5 -	Arabidopsis thaliana	abscisic acid responsive
TaMAPKKK100	ABRE	AACCCGG	1885	7 -	Arabidopsis thaliana	abscisic acid responsive
TaMAPKKK100	TGACG-motif	TGACG	943	5 -	Hordeum vulgare	the MeJA responsive
TaMAPKKK101	ABRE	ACGTG	1144	5 -	Arabidopsis thaliana	abscisic acid responsive
TaMAPKKK101	TGA-element	AACGAC	1139	6 +	Brassica oleracea	auxin responsive
TaMAPKKK101	TATC-box	TATCCCA	293	7 -	Oryza sativa	gibberellin responsive
TaMAPKKK101	TCA-element	CCATCTTTTT	356	9 +	Nicotiana tabacum	salicylic acid responsive
TaMAPKKK101	O2-site	GATGATGTGG	100	9 -	Zea mays	zein metabolism regulation
TaMAPKKK101	CAT-box	GCCACT	1580	6 -	Arabidopsis thaliana	meristem expression
TaMAPKKK101	ARE	AAACCA	568	6 -	Zea mays	anaerobic induction
TaMAPKKK101	ARE	AAACCA	1710	6 -	Zea mays	anaerobic induction
TaMAPKKK101	CGTCA-motif	CGTCA	516	5 +	Hordeum vulgare	the MeJA responsive
TaMAPKKK101	CGTCA-motif	CGTCA	609	5 -	Hordeum vulgare	the MeJA responsive
TaMAPKKK101	TGACG-motif	TGACG	516	5 -	Hordeum vulgare	the MeJA responsive
TaMAPKKK101	TGACG-motif	TGACG	609	5 +	Hordeum vulgare	the MeJA responsive
TaMAPKKK102	CAT-box	GCCACT	1559	6 +	Arabidopsis thaliana	meristem expression

TaMAPKKK102	GC-motif	CCCCCG	1007	6 +	Zea mays	anoxic specific inducibility
TaMAPKKK102	GC-motif	CCCCCG	1329	6 +	Zea mays	anoxic specific inducibility
TaMAPKKK102	GC-motif	CCCCCG	1340	6 +	Zea mays	anoxic specific inducibility
TaMAPKKK102	GC-motif	CCCCCG	1357	6 +	Zea mays	anoxic specific inducibility
TaMAPKKK102	TGA-element	AACGAC	135	6 -	Brassica oleracea	auxin responsive
TaMAPKKK102	TGACG-motif	TGACG	284	5 +	Hordeum vulgare	the MeJA responsive
TaMAPKKK102	LTR	CCGAAA	241	6 +	Hordeum vulgare	low temperature responsive
TaMAPKKK102	LTR	CCGAAA	412	6 -	Hordeum vulgare	low temperature responsive
TaMAPKKK102	MBS	CAACTG	130	6 -	Arabidopsis thaliana	drought inducibility
TaMAPKKK102	ARE	AAACCA	1675	6 -	Zea mays	anaerobic induction
TaMAPKKK102	ABRE	ACGTG	772	5 -	Arabidopsis thaliana	abscisic acid responsive
TaMAPKKK102	ABRE	ACGTG	932	5 -	Arabidopsis thaliana	abscisic acid responsive
TaMAPKKK102	CGTCA-motif	CGTCA	284	5 -	Hordeum vulgare	the MeJA responsive
TaMAPKKK103	LTR	CCGAAA	772	6 +	Hordeum vulgare	low temperature responsive
TaMAPKKK103	TC-rich repeats	ATTCTCTAAC	512	9 -	Nicotiana tabacum	defense and stress responsive
TaMAPKKK103	MBS	CAACTG	643	6 +	Arabidopsis thaliana	drought inducibility
TaMAPKKK103	MSA-like	TCCAACGGT	822	9 +	Catharanthus roseus	cell cycle regulation
TaMAPKKK103	CAT-box	GCCACT	709	6 +	Arabidopsis thaliana	meristem expression
TaMAPKKK103	ABRE	ACGTG	968	5 +	Arabidopsis thaliana	abscisic acid responsive

TaMAPKKK103	ABRE	ACGTG	1581	5 -	Arabidopsis thaliana	abscisic acid responsive
TaMAPKKK103	ABRE	GACACGTGGC	1780	9 -	Triticum aestivum	abscisic acid responsive
TaMAPKKK103	GC-motif	CCCCCG	862	6 +	Zea mays	anoxic specific inducibility
TaMAPKKK103	TGACG-motif	TGACG	943	5 -	Hordeum vulgare	the MeJA responsive
TaMAPKKK103	TGACG-motif	TGACG	1524	5 +	Hordeum vulgare	the MeJA responsive
TaMAPKKK103	TGACG-motif	TGACG	1727	5 -	Hordeum vulgare	the MeJA responsive
TaMAPKKK103	CGTCA-motif	CGTCA	943	5 +	Hordeum vulgare	the MeJA responsive
TaMAPKKK103	CGTCA-motif	CGTCA	1524	5 -	Hordeum vulgare	the MeJA responsive
TaMAPKKK103	CGTCA-motif	CGTCA	1727	5 +	Hordeum vulgare	the MeJA responsive
TaMAPKKK104	MBS	CAACTG	113	6 -	Arabidopsis thaliana	drought inducibility
TaMAPKKK104	MBS	CAACTG	913	6 -	Arabidopsis thaliana	drought inducibility
TaMAPKKK104	P-box	CCTTTTG	1892	7 +	Oryza sativa	gibberellin responsive
TaMAPKKK104	GC-motif	CCCCCG	1148	6 -	Zea mays	anoxic specific inducibility
TaMAPKKK104	ABRE	ACGTG	1808	5 -	Arabidopsis thaliana	abscisic acid responsive
TaMAPKKK104	MSA-like	(T/C)C(T/C)AAC GG(T/C)(T/C)A	626	9 +	Catharanthus roseus	cell cycle regulation
TaMAPKKK104	CGTCA-motif	CGTCA	501	5 +	Hordeum vulgare	the MeJA responsive
TaMAPKKK104	CGTCA-motif	CGTCA	944	5 +	Hordeum vulgare	the MeJA responsive
TaMAPKKK104	CGTCA-motif	CGTCA	1762	5 -	Hordeum vulgare	the MeJA responsive

TaMAPKKK104	AuxRR-core	GGTCCAT	614	7 +	Nicotiana tabacum	auxin responsive
TaMAPKKK104	O2-site	GATGATGTGG	294	9 -	Zea mays	zein metabolism regulation
TaMAPKKK104	O2-site	GATGACATGG	799	9 +	Zea mays	zein metabolism regulation
TaMAPKKK104	O2-site	GATGATGTGG	994	9 +	Zea mays	zein metabolism regulation
TaMAPKKK104	GCN4_motif	TGAGTCA	1483	7 -	Oryza sativa	endosperm expression
TaMAPKKK104	TGACG-motif	TGACG	501	5 -	Hordeum vulgare	the MeJA responsive
TaMAPKKK104	TGACG-motif	TGACG	944	5 -	Hordeum vulgare	the MeJA responsive
TaMAPKKK104	TGACG-motif	TGACG	1762	5 +	Hordeum vulgare	the MeJA responsive
TaMAPKKK104-1	MBS	CAACTG	1187	6 +	Arabidopsis thaliana	drought inducibility
TaMAPKKK104-1	MBS	CAACTG	1650	6 -	Arabidopsis thaliana	drought inducibility
TaMAPKKK104-1	TGA-element	AACGAC	1192	6 -	Brassica oleracea	auxin responsive
TaMAPKKK104-1	TGA-element	AACGAC	1383	6 +	Brassica oleracea	auxin responsive
TaMAPKKK104-1	TGA-element	AACGAC	1475	6 +	Brassica oleracea	auxin responsive
TaMAPKKK104-1	CAT-box	GCCACT	483	6 +	Arabidopsis thaliana	meristem expression
TaMAPKKK104-1	CAT-box	GCCACT	734	6 +	Arabidopsis thaliana	meristem expression
TaMAPKKK104-1	CAT-box	GCCACT	1294	6 +	Arabidopsis thaliana	meristem expression
TaMAPKKK104-1	CGTCA-motif	CGTCA	958	5 -	Hordeum vulgare	the MeJA responsive
TaMAPKKK104-1	CGTCA-motif	CGTCA	1055	5 -	Hordeum vulgare	the MeJA responsive
TaMAPKKK104-1	CGTCA-motif	CGTCA	1197	5 -	Hordeum vulgare	the MeJA responsive

TaMAPKKK104-1	CGTCA-motif	CGTCA	1208	5 +	Hordeum vulgare	the MeJA responsive
TaMAPKKK104-1	CGTCA-motif	CGTCA	1492	5 +	Hordeum vulgare	the MeJA responsive
TaMAPKKK104-1	CGTCA-motif	CGTCA	1541	5 +	Hordeum vulgare	the MeJA responsive
TaMAPKKK104-1	P-box	CCTTTTG	1901	7 +	Oryza sativa	gibberellin responsive
TaMAPKKK104-1	ABRE	CACGTG	138	6 +	Arabidopsis thaliana	abscisic acid responsive
TaMAPKKK104-1	ABRE	ACGTG	139	5 +	Arabidopsis thaliana	abscisic acid responsive
TaMAPKKK104-1	ABRE	ACGTG	1206	5 -	Arabidopsis thaliana	abscisic acid responsive
TaMAPKKK104-1	ABRE	CGTACGTGCA	1982	9 -	Hordeum vulgare	abscisic acid responsive
TaMAPKKK104-1	GC-motif	CCCCCG	1456	6 -	Zea mays	anoxic specific inducibility
TaMAPKKK104-1	GARE-motif	TCTGTTG	908	7 -	Brassica oleracea	gibberellin responsive
TaMAPKKK104-1	O2-site	GATGATGTGG	1749	9 +	Zea mays	zein metabolism regulation
TaMAPKKK104-1	TGACG-motif	TGACG	958	5 +	Hordeum vulgare	the MeJA responsive
TaMAPKKK104-1	TGACG-motif	TGACG	1055	5 +	Hordeum vulgare	the MeJA responsive
TaMAPKKK104-1	TGACG-motif	TGACG	1197	5 +	Hordeum vulgare	the MeJA responsive
TaMAPKKK104-1	TGACG-motif	TGACG	1208	5 -	Hordeum vulgare	the MeJA responsive
TaMAPKKK104-1	TGACG-motif	TGACG	1492	5 -	Hordeum vulgare	the MeJA responsive
TaMAPKKK104-1	TGACG-motif	TGACG	1541	5 -	Hordeum vulgare	the MeJA responsive
TaMAPKKK104-1	ARE	AAACCA	1973	6 -	Zea mays	anaerobic induction
TaMAPKKK105	ABRE	ACGTG	183	5 -	Arabidopsis thaliana	abscisic acid responsive

TaMAPKKK105	ABRE	GCCGCGTGGC	590	9 +	Oryza sativa	abscisic acid responsive
TaMAPKKK105	ABRE	ACGTG	1808	5 -	Arabidopsis thaliana	abscisic acid responsive
TaMAPKKK105	ABRE	ACGTG	1813	5 -	Arabidopsis thaliana	abscisic acid responsive
TaMAPKKK105	MSA-like	(T/C)C(T/C)AACGG(T/C)(T/C)A	753	8.5 -	Catharanthus roseus	cell cycle regulation
TaMAPKKK105	TGA-element	AACGAC	1320	6 -	Brassica oleracea	auxin responsive
TaMAPKKK105	TGACG-motif	TGACG	1692	5 +	Hordeum vulgare	the MeJA responsive
TaMAPKKK105	TGACG-motif	TGACG	1810	5 -	Hordeum vulgare	the MeJA responsive
TaMAPKKK105	MBS	CAACTG	308	6 +	Arabidopsis thaliana	drought inducibility
TaMAPKKK105	MBS	CAACTG	1102	6 +	Arabidopsis thaliana	drought inducibility
TaMAPKKK105	GC-motif	CCCCCG	1503	6 -	Zea mays	anoxic specific inducibility
TaMAPKKK105	TC-rich repeats	ATTCTCTAAC	758	9 -	Nicotiana tabacum	defense and stress responsive
TaMAPKKK105	TC-rich repeats	ATTCTCTAAC	929	9 +	Nicotiana tabacum	defense and stress responsive
TaMAPKKK105	LTR	CCGAAA	554	6 -	Hordeum vulgare	low temperature responsive
TaMAPKKK105	CGTCA-motif	CGTCA	1692	5 -	Hordeum vulgare	the MeJA responsive
TaMAPKKK105	CGTCA-motif	CGTCA	1810	5 +	Hordeum vulgare	the MeJA responsive
TaMAPKKK105	HD-Zip 1	CAAT(A/T)ATTG	1182	8 -	Arabidopsis thaliana	differentiation of the palisade mesophyll cells
TaMAPKKK106	TGA-box	TGACGTAA	955	8 +	Glycine max	auxin responsive
TaMAPKKK106	ABRE	ACGTG	63	5 +	Arabidopsis thaliana	abscisic acid responsive
TaMAPKKK106	ABRE	ACGTG	197	5 +	Arabidopsis thaliana	abscisic acid responsive

TaMAPKKK106	ABRE	ACGTG	1288	5 -	Arabidopsis thaliana	abscisic acid responsive
TaMAPKKK106	LTR	CCGAAA	177	6 +	Hordeum vulgare	low temperature responsive
TaMAPKKK106	CGTCA-motif	CGTCA	99	5 +	Hordeum vulgare	the MeJA responsive
TaMAPKKK106	CGTCA-motif	CGTCA	450	5 -	Hordeum vulgare	the MeJA responsive
TaMAPKKK106	CGTCA-motif	CGTCA	776	5 +	Hordeum vulgare	the MeJA responsive
TaMAPKKK106	CGTCA-motif	CGTCA	955	5 -	Hordeum vulgare	the MeJA responsive
TaMAPKKK106	CGTCA-motif	CGTCA	1801	5 -	Hordeum vulgare	the MeJA responsive
TaMAPKKK106	TGA-element	AACGAC	857	6 +	Brassica oleracea	auxin responsive
TaMAPKKK106	TGA-element	AACGAC	920	6 +	Brassica oleracea	auxin responsive
TaMAPKKK106	MSA-like	TCCAACGGT	797	9 -	Catharanthus roseus	cell cycle regulation
TaMAPKKK106	GARE-motif	TCTGTTG	159	7 -	Brassica oleracea	gibberellin responsive
TaMAPKKK106	MBS	CAACTG	537	6 +	Arabidopsis thaliana	drought inducibility
TaMAPKKK106	MBS	CAACTG	1888	6 +	Arabidopsis thaliana	drought inducibility
TaMAPKKK106	O2-site	GATGA(C/T)(A/G)TG(A/G)	201	8 +	Zea mays	zein metabolism regulation
TaMAPKKK106	TGACG-motif	TGACG	99	5 -	Hordeum vulgare	the MeJA responsive
TaMAPKKK106	TGACG-motif	TGACG	450	5 +	Hordeum vulgare	the MeJA responsive
TaMAPKKK106	TGACG-motif	TGACG	776	5 -	Hordeum vulgare	the MeJA responsive
TaMAPKKK106	TGACG-motif	TGACG	955	5 +	Hordeum vulgare	the MeJA responsive
TaMAPKKK106	TGACG-motif	TGACG	1801	5 +	Hordeum vulgare	the MeJA responsive
TaMAPKKK106	ARE	AAACCA	1248	6 -	Zea mays	anaerobic induction

TaMAPKKK107	TGA-element	AACGAC	1677	6 +	Brassica oleracea	auxin responsive
TaMAPKKK107	TGA-element	AACGAC	1740	6 +	Brassica oleracea	auxin responsive
TaMAPKKK107	ABRE	ACGTG	885	5 +	Arabidopsis thaliana	abscisic acid responsive
TaMAPKKK107	ABRE	GCCGCGTGGC	1837	9 -	Oryza sativa	abscisic acid responsive
TaMAPKKK107	ARE	AAACCA	523	6 -	Zea mays	anaerobic induction
TaMAPKKK107	O2-site	GATGACATGG	915	9 -	Zea mays	zein metabolism regulation
TaMAPKKK107	O2-site	GATGA(C/T)(A/G)TG(A/G)	1023	8 +	Zea mays	zein metabolism regulation
TaMAPKKK107	TGACG-motif	TGACG	1176	5 +	Hordeum vulgare	the MeJA responsive
TaMAPKKK107	TGACG-motif	TGACG	1271	5 +	Hordeum vulgare	the MeJA responsive
TaMAPKKK107	TGACG-motif	TGACG	1772	5 +	Hordeum vulgare	the MeJA responsive
TaMAPKKK107	CGTCA-motif	CGTCA	1176	5 -	Hordeum vulgare	the MeJA responsive
TaMAPKKK107	CGTCA-motif	CGTCA	1271	5 -	Hordeum vulgare	the MeJA responsive
TaMAPKKK107	CGTCA-motif	CGTCA	1772	5 -	Hordeum vulgare	the MeJA responsive
TaMAPKKK107	LTR	CCGAAA	999	6 +	Hordeum vulgare	low temperature responsive
TaMAPKKK107	GARE-motif	TCTGTTG	981	7 -	Brassica oleracea	gibberellin responsive
TaMAPKKK107	MBS	CAACTG	474	6 -	Arabidopsis thaliana	drought inducibility
TaMAPKKK107	MBS	CAACTG	1357	6 +	Arabidopsis thaliana	drought inducibility
TaMAPKKK108	TGACG-motif	TGACG	238	5 -	Hordeum vulgare	the MeJA responsive
TaMAPKKK108	TGACG-motif	TGACG	625	5 -	Hordeum vulgare	the MeJA responsive
TaMAPKKK108	AuxRR-core	GGTCCAT	572	7 +	Nicotiana tabacum	auxin responsive

TaMAPKKK108	LTR	CCGAAA	781	6 +	Hordeum vulgare	low temperature responsive
TaMAPKKK108	LTR	CCGAAA	856	6 -	Hordeum vulgare	low temperature responsive
TaMAPKKK108	LTR	CCGAAA	1620	6 +	Hordeum vulgare	low temperature responsive
TaMAPKKK108	CGTCA-motif	CGTCA	238	5 +	Hordeum vulgare	the MeJA responsive
TaMAPKKK108	CGTCA-motif	CGTCA	625	5 +	Hordeum vulgare	the MeJA responsive
TaMAPKKK108	GC-motif	CCCCCG	621	6 +	Zea mays	anoxic specific inducibility
TaMAPKKK108	GC-motif	CCCCCG	664	6 +	Zea mays	anoxic specific inducibility
TaMAPKKK108	GC-motif	CCCCCG	694	6 +	Zea mays	anoxic specific inducibility
TaMAPKKK108	MBS	CAACTG	23	6 -	Arabidopsis thaliana	drought inducibility
TaMAPKKK108	MBS	CAACTG	769	6 -	Arabidopsis thaliana	drought inducibility
TaMAPKKK108	ARE	AAACCA	1067	6 +	Zea mays	anaerobic induction
TaMAPKKK108	ARE	AAACCA	1161	6 -	Zea mays	anaerobic induction
TaMAPKKK108	TGA-element	AACGAC	1763	6 -	Brassica oleracea	auxin responsive
TaMAPKKK108	ABRE	ACGTG	47	5 -	Arabidopsis thaliana	abscisic acid responsive
TaMAPKKK108	ABRE	ACGTG	383	5 -	Arabidopsis thaliana	abscisic acid responsive
TaMAPKKK108	ABRE	ACGTG	1822	5 +	Arabidopsis thaliana	abscisic acid responsive
TaMAPKKK108	ABRE	GCAACGTGTC	1833	9 +	Hordeum vulgare	abscisic acid responsive
TaMAPKKK108	ABRE	ACGTG	1836	5 +	Arabidopsis thaliana	abscisic acid responsive
TaMAPKKK108	ABRE	ACGTG	1915	5 -	Arabidopsis thaliana	abscisic acid responsive

TaMAPKKK108	CAT-box	GCCACT	733	6 -	Arabidopsis thaliana	meristem expression
TaMAPKKK109	CGTCA-motif	CGTCA	345	5 +	Hordeum vulgare	the MeJA responsive
TaMAPKKK109	CGTCA-motif	CGTCA	479	5 -	Hordeum vulgare	the MeJA responsive
TaMAPKKK109	CGTCA-motif	CGTCA	712	5 +	Hordeum vulgare	the MeJA responsive
TaMAPKKK109	CGTCA-motif	CGTCA	860	5 +	Hordeum vulgare	the MeJA responsive
TaMAPKKK109	CGTCA-motif	CGTCA	1680	5 +	Hordeum vulgare	the MeJA responsive
TaMAPKKK109	GC-motif	CCCCCG	509	6 +	Zea mays	anoxic specific inducibility
TaMAPKKK109	GC-motif	CCCCCG	1056	6 +	Zea mays	anoxic specific inducibility
TaMAPKKK109	P-box	CCTTTTG	1209	7 +	Oryza sativa	gibberellin responsive
TaMAPKKK109	MBS	CAACTG	674	6 +	Arabidopsis thaliana	drought inducibility
TaMAPKKK109	MBS	CAACTG	680	6 -	Arabidopsis thaliana	drought inducibility
TaMAPKKK109	MBS	CAACTG	1937	6 +	Arabidopsis thaliana	drought inducibility
TaMAPKKK109	ARE	AAACCA	1019	6 -	Zea mays	anaerobic induction
TaMAPKKK109	ARE	AAACCA	1281	6 +	Zea mays	anaerobic induction
TaMAPKKK109	ARE	AAACCA	1383	6 -	Zea mays	anaerobic induction
TaMAPKKK109	ARE	AAACCA	1630	6 -	Zea mays	anaerobic induction
TaMAPKKK109	TGACG-motif	TGACG	345	5 -	Hordeum vulgare	the MeJA responsive
TaMAPKKK109	TGACG-motif	TGACG	479	5 +	Hordeum vulgare	the MeJA responsive
TaMAPKKK109	TGACG-motif	TGACG	712	5 -	Hordeum vulgare	the MeJA responsive

TaMAPKKK109	TGACG-motif	TGACG	860	5 -	Hordeum vulgare	the MeJA responsive
TaMAPKKK109	TGACG-motif	TGACG	1680	5 -	Hordeum vulgare	the MeJA responsive
TaMAPKKK109	ABRE	ACGTG	291	5 +	Arabidopsis thaliana	abscisic acid responsive
TaMAPKKK109	ABRE	ACGTG	1102	5 -	Arabidopsis thaliana	abscisic acid responsive
TaMAPKKK109	ABRE	GCCGCGTGGC	1854	9 -	Oryza sativa	abscisic acid responsive
TaMAPKKK109	CAT-box	GCCACT	565	6 -	Arabidopsis thaliana	meristem expression
TaMAPKKK11	LTR	CCGAAA	1495	6 +	Hordeum vulgare	low temperature responsive
TaMAPKKK11	TGACG-motif	TGACG	900	5 -	Hordeum vulgare	the MeJA responsive
TaMAPKKK11	ARE	AAACCA	727	6 +	Zea mays	anaerobic induction
TaMAPKKK11	ARE	AAACCA	1415	6 +	Zea mays	anaerobic induction
TaMAPKKK11	MBS	CAACTG	1012	6 +	Arabidopsis thaliana	drought inducibility
TaMAPKKK11	MBS	CAACTG	1571	6 +	Arabidopsis thaliana	drought inducibility
TaMAPKKK11	CGTCA-motif	CGTCA	900	5 +	Hordeum vulgare	the MeJA responsive
TaMAPKKK11	CAT-box	GCCACT	1119	6 +	Arabidopsis thaliana	meristem expression
TaMAPKKK11	O2-site	GATGATGTGG	444	10 -	Zea mays	zein metabolism regulation
TaMAPKKK11	O2-site	GATGATGTGG	644	9 -	Zea mays	zein metabolism regulation
TaMAPKKK11	O2-site	GATGA(C/T)(A/G)TG(A/G)	1040	8 -	Zea mays	zein metabolism regulation
TaMAPKKK11	O2-site	GATGATGTGG	1577	9 -	Zea mays	zein metabolism regulation
TaMAPKKK11	circadian	CAAAGATATC	1700	9 +	Lycopersicon esculentum	circadian control

TaMAPKKK11	P-box	CCTTTTG	1343	7 -	Oryza sativa	gibberellin responsive
TaMAPKKK11	ABRE	CACGTG	556	6 +	Arabidopsis thaliana	abscisic acid responsive
TaMAPKKK11	ABRE	ACGTG	557	5 +	Arabidopsis thaliana	abscisic acid responsive
TaMAPKKK11	ABRE	GACACGTGGC	1119	9 -	Triticum aestivum	abscisic acid responsive
TaMAPKKK110	GCN4_motif	TGAGTCA	262	7 -	Oryza sativa	endosperm expression
TaMAPKKK110	ABRE	AACCCGG	89	7 -	Arabidopsis thaliana	abscisic acid responsive
TaMAPKKK110	ABRE	ACGTG	195	5 -	Arabidopsis thaliana	abscisic acid responsive
TaMAPKKK110	ABRE	CACGTG	610	6 +	Arabidopsis thaliana	abscisic acid responsive
TaMAPKKK110	ABRE	ACGTG	611	5 +	Arabidopsis thaliana	abscisic acid responsive
TaMAPKKK110	TGA-element	AACGAC	219	6 -	Brassica oleracea	auxin responsive
TaMAPKKK110	CGTCA-motif	CGTCA	1523	5 -	Hordeum vulgare	the MeJA responsive
TaMAPKKK110	CGTCA-motif	CGTCA	1641	5 -	Hordeum vulgare	the MeJA responsive
TaMAPKKK110	P-box	CCTTTTG	156	7 +	Oryza sativa	gibberellin responsive
TaMAPKKK110	P-box	CCTTTTG	622	7 +	Oryza sativa	gibberellin responsive
TaMAPKKK110	MBS	CAACTG	915	6 -	Arabidopsis thaliana	drought inducibility
TaMAPKKK110	MBS	CAACTG	978	6 -	Arabidopsis thaliana	drought inducibility
TaMAPKKK110	GARE-motif	TCTGTTG	1543	7 +	Brassica oleracea	gibberellin responsive
TaMAPKKK110	TGACG-motif	TGACG	1523	5 +	Hordeum vulgare	the MeJA responsive
TaMAPKKK110	TGACG-motif	TGACG	1641	5 +	Hordeum vulgare	the MeJA responsive

TaMAPKKK110	ARE	AAACCA	903	6 -	Zea mays	anaerobic induction
TaMAPKKK110	ARE	AAACCA	1733	6 +	Zea mays	anaerobic induction
TaMAPKKK110	ARE	AAACCA	1958	6 +	Zea mays	anaerobic induction
TaMAPKKK110	ARE	AAACCA	1963	6 +	Zea mays	anaerobic induction
TaMAPKKK111	TC-rich repeats	ATTCTCTAAC	1199	9 -	Nicotiana tabacum	defense and stress responsive
TaMAPKKK111	TCA-element	CCATCTTTTT	79	9 +	Nicotiana tabacum	salicylic acid responsive
TaMAPKKK111	CGTCA-motif	CGTCA	1098	5 +	Hordeum vulgare	the MeJA responsive
TaMAPKKK111	ABRE	AACCCGG	115	7 +	Arabidopsis thaliana	abscisic acid responsive
TaMAPKKK111	ABRE	CACGTG	399	6 +	Arabidopsis thaliana	abscisic acid responsive
TaMAPKKK111	ABRE	ACGTG	400	5 +	Arabidopsis thaliana	abscisic acid responsive
TaMAPKKK111	ABRE	ACGTG	852	5 +	Arabidopsis thaliana	abscisic acid responsive
TaMAPKKK111	TGACG-motif	TGACG	1098	5 -	Hordeum vulgare	the MeJA responsive
TaMAPKKK111	AuxRE	TGTCTCAATA AG	797	11 -	Glycine max	auxin responsive
TaMAPKKK112	CAT-box	GCCACT	799	6 +	Arabidopsis thaliana	meristem expression
TaMAPKKK112	ARE	AAACCA	849	6 +	Zea mays	anaerobic induction
TaMAPKKK112	TC-rich repeats	ATTCTCTAAC	671	9 -	Nicotiana tabacum	defense and stress responsive
TaMAPKKK112	TGACG-motif	TGACG	190	5 +	Hordeum vulgare	the MeJA responsive
TaMAPKKK112	TGACG-motif	TGACG	1749	5 +	Hordeum vulgare	the MeJA responsive
TaMAPKKK112	TGACG-motif	TGACG	1844	5 -	Hordeum vulgare	the MeJA responsive

TaMAPKKK112	CGTCA-motif	CGTCA	190	5 -	Hordeum vulgare	the MeJA responsive
TaMAPKKK112	CGTCA-motif	CGTCA	1749	5 -	Hordeum vulgare	the MeJA responsive
TaMAPKKK112	CGTCA-motif	CGTCA	1844	5 +	Hordeum vulgare	the MeJA responsive
TaMAPKKK112	MBS	CAACTG	563	6 -	Arabidopsis thaliana	drought inducibility
TaMAPKKK112	MBS	CAACTG	1490	6 -	Arabidopsis thaliana	drought inducibility
TaMAPKKK112	P-box	CCTTTTG	495	7 +	Oryza sativa	gibberellin responsive
TaMAPKKK112	TCA-element	CCATCTTTTT	1516	9 -	Nicotiana tabacum	salicylic acid responsive
TaMAPKKK113	TGACG-motif	TGACG	861	5 +	Hordeum vulgare	the MeJA responsive
TaMAPKKK113	TGACG-motif	TGACG	1238	5 +	Hordeum vulgare	the MeJA responsive
TaMAPKKK113	TGACG-motif	TGACG	1636	5 -	Hordeum vulgare	the MeJA responsive
TaMAPKKK113	ARE	AAACCA	1177	6 -	Zea mays	anaerobic induction
TaMAPKKK113	ARE	AAACCA	1376	6 +	Zea mays	anaerobic induction
TaMAPKKK113	ABRE	ACGTG	318	5 -	Arabidopsis thaliana	abscisic acid responsive
TaMAPKKK113	ABRE	ACGTG	919	5 -	Arabidopsis thaliana	abscisic acid responsive
TaMAPKKK113	ABRE	ACGTG	1044	5 -	Arabidopsis thaliana	abscisic acid responsive
TaMAPKKK113	ABRE	ACGTG	1240	5 +	Arabidopsis thaliana	abscisic acid responsive
TaMAPKKK113	ABRE	CACGTG	1626	6 -	Arabidopsis thaliana	abscisic acid responsive
TaMAPKKK113	ABRE	ACGTG	1627	5 +	Arabidopsis thaliana	abscisic acid responsive
TaMAPKKK113	ABRE	ACGTG	1775	5 -	Arabidopsis thaliana	abscisic acid responsive

TaMAPKKK113	TGA-element	AACGAC	1013	6 +	Brassica oleracea	auxin responsive
TaMAPKKK113	CGTCA-motif	CGTCA	861	5 -	Hordeum vulgare	the MeJA responsive
TaMAPKKK113	CGTCA-motif	CGTCA	1238	5 -	Hordeum vulgare	the MeJA responsive
TaMAPKKK113	CGTCA-motif	CGTCA	1636	5 +	Hordeum vulgare	the MeJA responsive
TaMAPKKK113	P-box	CCTTTTG	148	7 -	Oryza sativa	gibberellin responsive
TaMAPKKK113	P-box	CCTTTTG	298	7 -	Oryza sativa	gibberellin responsive
TaMAPKKK113	MBS	CAACTG	1222	6 +	Arabidopsis thaliana	drought inducibility
TaMAPKKK113	TC-rich repeats	GTTTTCTTAC	196	9 +	Nicotiana tabacum	defense and stress responsive
TaMAPKKK113	TC-rich repeats	GTTTTCTTAC	402	9 +	Nicotiana tabacum	defense and stress responsive
TaMAPKKK113	TC-rich repeats	ATTCTCTAAC	634	9 +	Nicotiana tabacum	defense and stress responsive
TaMAPKKK113	TCA-element	CCATCTTTTT	846	9 -	Nicotiana tabacum	salicylic acid responsive
TaMAPKKK113	TCA-element	CCATCTTTTT	1049	9 +	Nicotiana tabacum	salicylic acid responsive
TaMAPKKK113	O2-site	GATGATGTGG	1891	9 +	Zea mays	zein metabolism regulation
TaMAPKKK113	GC-motif	CCCCCG	1581	6 -	Zea mays	anoxic specific inducibility
TaMAPKKK115	ABRE	ACGTG	319	5 -	Arabidopsis thaliana	abscisic acid responsive
TaMAPKKK115	TGA-element	AACGAC	1482	6 +	Brassica oleracea	auxin responsive
TaMAPKKK115	TGA-element	AACGAC	1771	6 +	Brassica oleracea	auxin responsive
TaMAPKKK115	TGA-element	AACGAC	1972	6 +	Brassica oleracea	auxin responsive
TaMAPKKK115	TGACG-motif	TGACG	39	5 +	Hordeum vulgare	the MeJA responsive
TaMAPKKK115	ARE	AAACCA	219	6 -	Zea mays	anaerobic induction
TaMAPKKK115	O2-site	GATGA(C/T)(A/G)TG(A/G)	1290	8 +	Zea mays	zein metabolism regulation

TaMAPKKK115	TCA-element	CCATCTTTTT	139	9 +	Nicotiana tabacum	salicylic acid responsive
TaMAPKKK115	LTR	CCGAAA	1213	6 -	Hordeum vulgare	low temperature responsive
TaMAPKKK115	LTR	CCGAAA	1310	6 -	Hordeum vulgare	low temperature responsive
TaMAPKKK115	CGTCA-motif	CGTCA	39	5 -	Hordeum vulgare	the MeJA responsive
TaMAPKKK116	ABRE	CACGTG	68	6 +	Arabidopsis thaliana	abscisic acid responsive
TaMAPKKK116	ABRE	ACGTG	69	5 +	Arabidopsis thaliana	abscisic acid responsive
TaMAPKKK116	ABRE	CACGTG	99	6 +	Arabidopsis thaliana	abscisic acid responsive
TaMAPKKK116	ABRE	ACGTG	100	5 +	Arabidopsis thaliana	abscisic acid responsive
TaMAPKKK116	ABRE	GCAACGTGTC	191	9 +	Hordeum vulgare	abscisic acid responsive
TaMAPKKK116	ABRE	CACGTG	193	6 +	Arabidopsis thaliana	abscisic acid responsive
TaMAPKKK116	ABRE	ACGTG	194	5 +	Arabidopsis thaliana	abscisic acid responsive
TaMAPKKK116	ABRE	ACGTG	389	5 -	Arabidopsis thaliana	abscisic acid responsive
TaMAPKKK116	ABRE	ACGTG	723	5 +	Arabidopsis thaliana	abscisic acid responsive
TaMAPKKK116	ABRE	GACACGTGGC	935	9 -	Triticum aestivum	abscisic acid responsive
TaMAPKKK116	ABRE	CACGTG	937	6 +	Arabidopsis thaliana	abscisic acid responsive
TaMAPKKK116	ABRE	ACGTG	938	5 +	Arabidopsis thaliana	abscisic acid responsive
TaMAPKKK116	ABRE	ACGTG	1184	5 +	Arabidopsis thaliana	abscisic acid responsive
TaMAPKKK116	ABRE	ACGTG	1580	5 +	Arabidopsis thaliana	abscisic acid responsive
TaMAPKKK116	ABRE	ACGTG	1651	5 +	Arabidopsis thaliana	abscisic acid responsive

TaMAPKKK116	GC-motif	CCCCCG	485	6 +	Zea mays	anoxic specific inducibility
TaMAPKKK116	GC-motif	CCCCCG	1172	6 -	Zea mays	anoxic specific inducibility
TaMAPKKK116	CAT-box	GCCACT	682	6 -	Arabidopsis thaliana	meristem expression
TaMAPKKK116	CAT-box	GCCACT	852	6 +	Arabidopsis thaliana	meristem expression
TaMAPKKK116	CGTCA-motif	CGTCA	210	5 -	Hordeum vulgare	the MeJA responsive
TaMAPKKK116	CGTCA-motif	CGTCA	227	5 -	Hordeum vulgare	the MeJA responsive
TaMAPKKK116	CGTCA-motif	CGTCA	242	5 +	Hordeum vulgare	the MeJA responsive
TaMAPKKK116	CGTCA-motif	CGTCA	341	5 +	Hordeum vulgare	the MeJA responsive
TaMAPKKK116	CGTCA-motif	CGTCA	505	5 -	Hordeum vulgare	the MeJA responsive
TaMAPKKK116	CGTCA-motif	CGTCA	721	5 -	Hordeum vulgare	the MeJA responsive
TaMAPKKK116	CGTCA-motif	CGTCA	969	5 +	Hordeum vulgare	the MeJA responsive
TaMAPKKK116	MBS	CAACTG	1227	6 -	Arabidopsis thaliana	drought inducibility
TaMAPKKK116	RY-element	CATGCATG	354	8 +	Helianthus annuus	seed specific regulation
TaMAPKKK116	ARE	AAACCA	1483	6 -	Zea mays	anaerobic induction
TaMAPKKK116	TGACG-motif	TGACG	210	5 +	Hordeum vulgare	the MeJA responsive
TaMAPKKK116	TGACG-motif	TGACG	227	5 +	Hordeum vulgare	the MeJA responsive
TaMAPKKK116	TGACG-motif	TGACG	242	5 -	Hordeum vulgare	the MeJA responsive
TaMAPKKK116	TGACG-motif	TGACG	341	5 -	Hordeum vulgare	the MeJA responsive
TaMAPKKK116	TGACG-motif	TGACG	505	5 +	Hordeum vulgare	the MeJA responsive

TaMAPKKK116	TGACG-motif	TGACG	721	5 +	Hordeum vulgare	the MeJA responsive
TaMAPKKK116	TGACG-motif	TGACG	969	5 -	Hordeum vulgare	the MeJA responsive
TaMAPKKK116	LTR	CCGAAA	1101	6 -	Hordeum vulgare	low temperature responsive
TaMAPKKK116	LTR	CCGAAA	1143	6 -	Hordeum vulgare	low temperature responsive
TaMAPKKK117	TGA-element	AACGAC	506	6 -	Brassica oleracea	auxin responsive
TaMAPKKK117	GARE-motif	TCTGTTG	1743	7 +	Brassica oleracea	gibberellin responsive
TaMAPKKK117	ABRE	ACGTG	265	5 +	Arabidopsis thaliana	abscisic acid responsive
TaMAPKKK117	ABRE	ACGTG	476	5 -	Arabidopsis thaliana	abscisic acid responsive
TaMAPKKK117	ABRE	ACGTG	688	5 +	Arabidopsis thaliana	abscisic acid responsive
TaMAPKKK117	ABRE	ACGTG	1244	5 -	Arabidopsis thaliana	abscisic acid responsive
TaMAPKKK117	ABRE	ACGTG	1648	5 -	Arabidopsis thaliana	abscisic acid responsive
TaMAPKKK117	ABRE	CACGTG	1762	6 -	Arabidopsis thaliana	abscisic acid responsive
TaMAPKKK117	ABRE	ACGTG	1763	5 +	Arabidopsis thaliana	abscisic acid responsive
TaMAPKKK117	ABRE	ACGTG	1768	5 +	Arabidopsis thaliana	abscisic acid responsive
TaMAPKKK117	ARE	AAACCA	764	6 -	Zea mays	anaerobic induction
TaMAPKKK117	TGACG-motif	TGACG	680	5 -	Hordeum vulgare	the MeJA responsive
TaMAPKKK117	TGACG-motif	TGACG	833	5 +	Hordeum vulgare	the MeJA responsive
TaMAPKKK117	TGACG-motif	TGACG	1077	5 +	Hordeum vulgare	the MeJA responsive
TaMAPKKK117	TGACG-motif	TGACG	1766	5 +	Hordeum vulgare	the MeJA responsive

TaMAPKKK117	GC-motif	CCCCCG	445	6 +	Zea mays	anoxic specific inducibility
TaMAPKKK117	O2-site	GATGATGTGG	1419	10 -	Zea mays	zein metabolism regulation
TaMAPKKK117	TCA-element	CCATCTTTTT	864	9 -	Nicotiana tabacum	salicylic acid responsive
TaMAPKKK117	P-box	CCTTTTG	1869	7 +	Oryza sativa	gibberellin responsive
TaMAPKKK117	AuxRR-core	GGTCCAT	51	7 -	Nicotiana tabacum	auxin responsive
TaMAPKKK117	CGTCA-motif	CGTCA	680	5 +	Hordeum vulgare	the MeJA responsive
TaMAPKKK117	CGTCA-motif	CGTCA	833	5 -	Hordeum vulgare	the MeJA responsive
TaMAPKKK117	CGTCA-motif	CGTCA	1077	5 -	Hordeum vulgare	the MeJA responsive
TaMAPKKK117	CGTCA-motif	CGTCA	1766	5 -	Hordeum vulgare	the MeJA responsive
TaMAPKKK119	TGACG-motif	TGACG	1892	5 -	Hordeum vulgare	the MeJA responsive
TaMAPKKK119	TGACG-motif	TGACG	1900	5 -	Hordeum vulgare	the MeJA responsive
TaMAPKKK119	LTR	CCGAAA	1244	6 -	Hordeum vulgare	low temperature responsive
TaMAPKKK119	CAT-box	GCCACT	979	6 -	Arabidopsis thaliana	meristem expression
TaMAPKKK119	CGTCA-motif	CGTCA	1892	5 +	Hordeum vulgare	the MeJA responsive
TaMAPKKK119	CGTCA-motif	CGTCA	1900	5 +	Hordeum vulgare	the MeJA responsive
TaMAPKKK119	ABRE	ACGTG	1131	5 +	Arabidopsis thaliana	abscisic acid responsive
TaMAPKKK119	ABRE	CACGTG	1228	6 -	Arabidopsis thaliana	abscisic acid responsive
TaMAPKKK119	ABRE	ACGTG	1229	5 +	Arabidopsis thaliana	abscisic acid responsive
TaMAPKKK119	ABRE	ACGTG	1365	5 +	Arabidopsis thaliana	abscisic acid responsive

TaMAPKKK119	ABRE	ACGTG	1382	5 -	Arabidopsis thaliana	abscisic acid responsive
TaMAPKKK119	ABRE	ACGTG	1399	5 +	Arabidopsis thaliana	abscisic acid responsive
TaMAPKKK119	ABRE	GCCGCGTGGC	1599	9 -	Oryza sativa	abscisic acid responsive
TaMAPKKK119	GC-motif	CCCCCG	1351	6 -	Zea mays	anoxic specific inducibility
TaMAPKKK119	GC-motif	CCCCCG	1840	6 +	Zea mays	anoxic specific inducibility
TaMAPKKK119	MBS	CAACTG	251	6 +	Arabidopsis thaliana	drought inducibility
TaMAPKKK119	MBS	CAACTG	312	6 -	Arabidopsis thaliana	drought inducibility
TaMAPKKK119	MBS	CAACTG	927	6 +	Arabidopsis thaliana	drought inducibility
TaMAPKKK119	MBS	CAACTG	1289	6 -	Arabidopsis thaliana	drought inducibility
TaMAPKKK12	O2-site	GTTGACGTGA	1377	9 +	Zea mays	zein metabolism regulation
TaMAPKKK12	TGACG-motif	TGACG	1142	5 -	Hordeum vulgare	the MeJA responsive
TaMAPKKK12	TGACG-motif	TGACG	1379	5 +	Hordeum vulgare	the MeJA responsive
TaMAPKKK12	TGACG-motif	TGACG	1688	5 +	Hordeum vulgare	the MeJA responsive
TaMAPKKK12	MBS	CAACTG	64	6 +	Arabidopsis thaliana	drought inducibility
TaMAPKKK12	MBS	CAACTG	143	6 +	Arabidopsis thaliana	drought inducibility
TaMAPKKK12	GARE-motif	TCTGTTG	43	7 -	Brassica oleracea	gibberellin responsive
TaMAPKKK12	GC-motif	CCCCCG	1418	6 +	Zea mays	anoxic specific inducibility
TaMAPKKK12	GC-motif	CCCCCG	1931	6 +	Zea mays	anoxic specific inducibility
TaMAPKKK12	GC-motif	CCCCCG	1983	6 +	Zea mays	anoxic specific inducibility

TaMAPKKK12	ARE	AAACCA	1780	6 +	Zea mays	anaerobic induction
TaMAPKKK12	CAT-box	GCCACT	1302	6 +	Arabidopsis thaliana	meristem expression
TaMAPKKK12	MSA-like	TCAAACGGT	972	9 -	Catharanthus roseus	cell cycle regulation
TaMAPKKK12	CGTCA-motif	CGTCA	1142	5 +	Hordeum vulgare	the MeJA responsive
TaMAPKKK12	CGTCA-motif	CGTCA	1379	5 -	Hordeum vulgare	the MeJA responsive
TaMAPKKK12	CGTCA-motif	CGTCA	1688	5 -	Hordeum vulgare	the MeJA responsive
TaMAPKKK12	LTR	CCGAAA	283	6 +	Hordeum vulgare	low temperature responsive
TaMAPKKK12	ABRE	AACCCGG	348	7 +	Arabidopsis thaliana	abscisic acid responsive
TaMAPKKK12	ABRE	AACCCGG	409	7 -	Arabidopsis thaliana	abscisic acid responsive
TaMAPKKK12	ABRE	ACGTG	1176	5 +	Arabidopsis thaliana	abscisic acid responsive
TaMAPKKK12	ABRE	ACGTG	1343	5 +	Arabidopsis thaliana	abscisic acid responsive
TaMAPKKK12	ABRE	ACGTG	1381	5 +	Arabidopsis thaliana	abscisic acid responsive
TaMAPKKK12	ABRE	ACGTG	1514	5 +	Arabidopsis thaliana	abscisic acid responsive
TaMAPKKK120	CGTCA-motif	CGTCA	156	5 -	Hordeum vulgare	the MeJA responsive
TaMAPKKK120	CGTCA-motif	CGTCA	816	5 -	Hordeum vulgare	the MeJA responsive
TaMAPKKK120	CGTCA-motif	CGTCA	1469	5 -	Hordeum vulgare	the MeJA responsive
TaMAPKKK120	TGA-element	AACGAC	286	6 +	Brassica oleracea	auxin responsive
TaMAPKKK120	TGA-element	AACGAC	744	6 -	Brassica oleracea	auxin responsive
TaMAPKKK120	ABRE	ACGTG	378	5 -	Arabidopsis thaliana	abscisic acid responsive
TaMAPKKK120	ABRE	ACGTG	843	5 -	Arabidopsis thaliana	abscisic acid responsive

TaMAPKKK120	ABRE	ACGTG	1069	5 -	Arabidopsis thaliana	abscisic acid responsive
TaMAPKKK120	ABRE	GCCGCGTGGC	1831	9 -	Oryza sativa	abscisic acid responsive
TaMAPKKK120	TGACG-motif	TGACG	156	5 +	Hordeum vulgare	the MeJA responsive
TaMAPKKK120	TGACG-motif	TGACG	816	5 +	Hordeum vulgare	the MeJA responsive
TaMAPKKK120	TGACG-motif	TGACG	1469	5 +	Hordeum vulgare	the MeJA responsive
TaMAPKKK120	ARE	AAACCA	1714	6 +	Zea mays	anaerobic induction
TaMAPKKK120	ARE	AAACCA	1739	6 -	Zea mays	anaerobic induction
TaMAPKKK120	GC-motif	CCCCCG	1619	6 -	Zea mays	anoxic specific inducibility
TaMAPKKK121	TGACG-motif	TGACG	24	5 +	Hordeum vulgare	the MeJA responsive
TaMAPKKK121	TGACG-motif	TGACG	1067	5 +	Hordeum vulgare	the MeJA responsive
TaMAPKKK121	TGACG-motif	TGACG	1597	5 +	Hordeum vulgare	the MeJA responsive
TaMAPKKK121	TGACG-motif	TGACG	1812	5 +	Hordeum vulgare	the MeJA responsive
TaMAPKKK121	ARE	AAACCA	532	6 -	Zea mays	anaerobic induction
TaMAPKKK121	ABRE	ACGTG	402	5 -	Arabidopsis thaliana	abscisic acid responsive
TaMAPKKK121	ABRE	ACGTG	549	5 +	Arabidopsis thaliana	abscisic acid responsive
TaMAPKKK121	ABRE	AACCCGG	1096	7 -	Arabidopsis thaliana	abscisic acid responsive
TaMAPKKK121	ABRE	GCAACGTGTC	1346	9 -	Hordeum vulgare	abscisic acid responsive
TaMAPKKK121	ABRE	ACGTG	1348	5 -	Arabidopsis thaliana	abscisic acid responsive
TaMAPKKK121	TGA-element	AACGAC	1343	6 +	Brassica oleracea	auxin responsive

TaMAPKKK121	LTR	CCGAAA	1589	6 +	Hordeum vulgare	low temperature responsive
TaMAPKKK121	CGTCA-motif	CGTCA	24	5 -	Hordeum vulgare	the MeJA responsive
TaMAPKKK121	CGTCA-motif	CGTCA	1067	5 -	Hordeum vulgare	the MeJA responsive
TaMAPKKK121	CGTCA-motif	CGTCA	1597	5 -	Hordeum vulgare	the MeJA responsive
TaMAPKKK121	CGTCA-motif	CGTCA	1812	5 -	Hordeum vulgare	the MeJA responsive
TaMAPKKK121	MBS	CAACTG	1235	6 +	Arabidopsis thaliana	drought inducibility
TaMAPKKK121	GCN4_motif	TGAGTCA	426	7 +	Oryza sativa	endosperm expression
TaMAPKKK121	TCA-element	CCATCTTTTT	1529	9 +	Nicotiana tabacum	salicylic acid responsive
TaMAPKKK122	CAT-box	GCCACT	520	6 -	Arabidopsis thaliana	meristem expression
TaMAPKKK122	CGTCA-motif	CGTCA	973	5 +	Hordeum vulgare	the MeJA responsive
TaMAPKKK122	CGTCA-motif	CGTCA	1541	5 +	Hordeum vulgare	the MeJA responsive
TaMAPKKK122	ABRE	ACGTG	879	5 +	Arabidopsis thaliana	abscisic acid responsive
TaMAPKKK122	ABRE	GCCGCGTGGC	1738	9 -	Oryza sativa	abscisic acid responsive
TaMAPKKK122	TC-rich repeats	GTTTTCTTAC	1495	9 +	Nicotiana tabacum	defense and stress responsive
TaMAPKKK122	MBS	CAACTG	1940	6 +	Arabidopsis thaliana	drought inducibility
TaMAPKKK122	TGACG-motif	TGACG	973	5 -	Hordeum vulgare	the MeJA responsive
TaMAPKKK122	TGACG-motif	TGACG	1541	5 -	Hordeum vulgare	the MeJA responsive
TaMAPKKK122	ARE	AAACCA	1433	6 -	Zea mays	anaerobic induction
TaMAPKKK122	GARE-motif	TCTGTTG	425	7 -	Brassica oleracea	gibberellin responsive

TaMAPKKK123	MBS	CAACTG	1105	6 +	Arabidopsis thaliana	drought inducibility
TaMAPKKK123	TCA-element	CCATCTTTTT	696	9 +	Nicotiana tabacum	salicylic acid responsive
TaMAPKKK123	P-box	CCTTTTG	249	7 +	Oryza sativa	gibberellin responsive
TaMAPKKK123	ARE	AAACCA	270	6 +	Zea mays	anaerobic induction
TaMAPKKK123	ARE	AAACCA	1080	6 +	Zea mays	anaerobic induction
TaMAPKKK123	ARE	AAACCA	1878	6 +	Zea mays	anaerobic induction
TaMAPKKK123	GCN4_motif	TGAGTCA	402	7 -	Oryza sativa	endosperm expression
TaMAPKKK123	TC-rich repeats	ATTCTCTAAC	1426	9 -	Nicotiana tabacum	defense and stress responsive
TaMAPKKK123	TGACG-motif	TGACG	567	5 -	Hordeum vulgare	the MeJA responsive
TaMAPKKK123	TGACG-motif	TGACG	1629	5 +	Hordeum vulgare	the MeJA responsive
TaMAPKKK123	TGACG-motif	TGACG	1692	5 +	Hordeum vulgare	the MeJA responsive
TaMAPKKK123	TGACG-motif	TGACG	1874	5 -	Hordeum vulgare	the MeJA responsive
TaMAPKKK123	TGACG-motif	TGACG	1991	5 +	Hordeum vulgare	the MeJA responsive
TaMAPKKK123	CGTCA-motif	CGTCA	567	5 +	Hordeum vulgare	the MeJA responsive
TaMAPKKK123	CGTCA-motif	CGTCA	1629	5 -	Hordeum vulgare	the MeJA responsive
TaMAPKKK123	CGTCA-motif	CGTCA	1692	5 -	Hordeum vulgare	the MeJA responsive
TaMAPKKK123	CGTCA-motif	CGTCA	1874	5 +	Hordeum vulgare	the MeJA responsive
TaMAPKKK123	CGTCA-motif	CGTCA	1991	5 -	Hordeum vulgare	the MeJA responsive
TaMAPKKK123-1	P-box	CCTTTTG	919	7 +	Oryza sativa	gibberellin responsive

TaMAPKKK123-1	TATC-box	TATCCCA	52	7 -	Oryza sativa	gibberellin responsive
TaMAPKKK123-1	MBS	CAACTG	1506	6 -	Arabidopsis thaliana	drought inducibility
TaMAPKKK123-1	GC-motif	CCCCCG	1922	6 -	Zea mays	anoxic specific inducibility
TaMAPKKK123-1	TGA-element	AACGAC	1355	6 +	Brassica oleracea	auxin responsive
TaMAPKKK123-1	ABRE	ACGTG	454	5 +	Arabidopsis thaliana	abscisic acid responsive
TaMAPKKK123-1	ABRE	ACGTG	1644	5 +	Arabidopsis thaliana	abscisic acid responsive
TaMAPKKK123-1	LTR	CCGAAA	771	6 -	Hordeum vulgare	low temperature responsive
TaMAPKKK123-1	CGTCA-motif	CGTCA	432	5 -	Hordeum vulgare	the MeJA responsive
TaMAPKKK123-1	CGTCA-motif	CGTCA	1408	5 -	Hordeum vulgare	the MeJA responsive
TaMAPKKK123-1	CGTCA-motif	CGTCA	1708	5 +	Hordeum vulgare	the MeJA responsive
TaMAPKKK123-1	TGACG-motif	TGACG	432	5 +	Hordeum vulgare	the MeJA responsive
TaMAPKKK123-1	TGACG-motif	TGACG	1408	5 +	Hordeum vulgare	the MeJA responsive
TaMAPKKK123-1	TGACG-motif	TGACG	1708	5 -	Hordeum vulgare	the MeJA responsive
TaMAPKKK125	ARE	AAACCA	229	6 +	Zea mays	anaerobic induction
TaMAPKKK125	ARE	AAACCA	773	6 +	Zea mays	anaerobic induction
TaMAPKKK125	ARE	AAACCA	1018	6 +	Zea mays	anaerobic induction
TaMAPKKK125	ABRE	ACGTG	27	5 +	Arabidopsis thaliana	abscisic acid responsive
TaMAPKKK125	ABRE	ACGTG	1595	5 -	Arabidopsis thaliana	abscisic acid responsive
TaMAPKKK125	ABRE	ACGTG	1638	5 -	Arabidopsis thaliana	abscisic acid responsive

TaMAPKKK125	O2-site	GTTGACGTGA	891	9 -	Zea mays	zein metabolism regulation
TaMAPKKK125	CGTCA-motif	CGTCA	1158	5 +	Hordeum vulgare	the MeJA responsive
TaMAPKKK125	TGACG-motif	TGACG	1158	5 -	Hordeum vulgare	the MeJA responsive
TaMAPKKK125	LTR	CCGAAA	1201	6 +	Hordeum vulgare	low temperature responsive
TaMAPKKK125	LTR	CCGAAA	1816	6 -	Hordeum vulgare	low temperature responsive
TaMAPKKK125	MBS	CAACTG	511	6 +	Arabidopsis thaliana	drought inducibility
TaMAPKKK125	WUN-motif	AAATTTCCT	1109	9 +	Brassica oleracea	wound responsive
TaMAPKKK126	TGACG-motif	TGACG	447	5 -	Hordeum vulgare	the MeJA responsive
TaMAPKKK126	TGACG-motif	TGACG	1852	5 +	Hordeum vulgare	the MeJA responsive
TaMAPKKK126	TGA-element	AACGAC	1585	6 +	Brassica oleracea	auxin responsive
TaMAPKKK126	CAT-box	GCCACT	451	6 -	Arabidopsis thaliana	meristem expression
TaMAPKKK126	GARE-motif	TCTGTTG	1621	7 +	Brassica oleracea	gibberellin responsive
TaMAPKKK126	CGTCA-motif	CGTCA	447	5 +	Hordeum vulgare	the MeJA responsive
TaMAPKKK126	CGTCA-motif	CGTCA	1852	5 -	Hordeum vulgare	the MeJA responsive
TaMAPKKK126	P-box	CCTTTTG	265	7 -	Oryza sativa	gibberellin responsive
TaMAPKKK126	ABRE	ACGTG	445	5 -	Arabidopsis thaliana	abscisic acid responsive
TaMAPKKK126	ABRE	CACGTG	1453	6 -	Arabidopsis thaliana	abscisic acid responsive
TaMAPKKK126	ABRE	ACGTG	1454	5 +	Arabidopsis thaliana	abscisic acid responsive
TaMAPKKK126	ARE	AAACCA	760	6 +	Zea mays	anaerobic induction

TaMAPKKK126	ARE	AAACCA	1319	6	+	Zea mays	anaerobic induction
TaMAPKKK126	TCA-element	TCAGAAGAGG	501	9	+	Brassica oleracea	salicylic acid responsive
TaMAPKKK126	O2-site	GATGACATGG	1654	9	-	Zea mays	zein metabolism regulation
TaMAPKKK126	O2-site	GATGA(C/T)(A/G)TG(A/G)	1659	8	-	Zea mays	zein metabolism regulation
TaMAPKKK126	MBS	CAACTG	727	6	+	Arabidopsis thaliana	drought inducibility
TaMAPKKK126	MBS	CAACTG	998	6	-	Arabidopsis thaliana	drought inducibility
TaMAPKKK126	LTR	CCGAAA	1678	6	+	Hordeum vulgare	low temperature responsive
TaMAPKKK127	MSA-like	(T/C)C(T/C)AACGG(T/C)(T/C)A	1152	8.5	+	Catharanthus roseus	cell cycle regulation
TaMAPKKK127	MSA-like	(T/C)C(T/C)AACGG(T/C)(T/C)A	1210	8.5	+	Catharanthus roseus	cell cycle regulation
TaMAPKKK127	ARE	AAACCA	212	6	-	Zea mays	anaerobic induction
TaMAPKKK127	CGTCA-motif	CGTCA	54	5	+	Hordeum vulgare	the MeJA responsive
TaMAPKKK127	CGTCA-motif	CGTCA	553	5	-	Hordeum vulgare	the MeJA responsive
TaMAPKKK127	CGTCA-motif	CGTCA	976	5	-	Hordeum vulgare	the MeJA responsive
TaMAPKKK127	CGTCA-motif	CGTCA	1376	5	+	Hordeum vulgare	the MeJA responsive
TaMAPKKK127	TC-rich repeats	GTTTTCTTAC	214	9	+	Nicotiana tabacum	defense and stress responsive
TaMAPKKK127	TGACG-motif	TGACG	54	5	-	Hordeum vulgare	the MeJA responsive
TaMAPKKK127	TGACG-motif	TGACG	553	5	+	Hordeum vulgare	the MeJA responsive
TaMAPKKK127	TGACG-motif	TGACG	976	5	+	Hordeum vulgare	the MeJA responsive

TaMAPKKK127	TGACG-motif	TGACG	1376	5 -	Hordeum vulgare	the MeJA responsive
TaMAPKKK127	TGA-element	AACGAC	1868	6 -	Brassica oleracea	auxin responsive
TaMAPKKK127	ABRE	ACGTG	599	5 -	Arabidopsis thaliana	abscisic acid responsive
TaMAPKKK127	TCA-element	TCAGAAGAGG	1124	9 +	Brassica oleracea	salicylic acid responsive
TaMAPKKK127	circadian	CAAAGATATC	827	9 +	Lycopersicon esculentum	circadian control
TaMAPKKK127	circadian	CAAAGATATC	1102	9 +	Lycopersicon esculentum	circadian control
TaMAPKKK127	MBS	CAACTG	564	6 +	Arabidopsis thaliana	drought inducibility
TaMAPKKK127	GARE-motif	TCTGTTG	415	7 -	Brassica oleracea	gibberellin responsive
TaMAPKKK128	MBS	CAACTG	70	6 +	Arabidopsis thaliana	drought inducibility
TaMAPKKK128	MBS	CAACTG	133	6 -	Arabidopsis thaliana	drought inducibility
TaMAPKKK128	CAT-box	GCCACT	165	6 -	Arabidopsis thaliana	meristem expression
TaMAPKKK128	CAT-box	GCCACT	251	6 -	Arabidopsis thaliana	meristem expression
TaMAPKKK128	CGTCA-motif	CGTCA	1175	5 -	Hordeum vulgare	the MeJA responsive
TaMAPKKK128	CGTCA-motif	CGTCA	1613	5 -	Hordeum vulgare	the MeJA responsive
TaMAPKKK128	CGTCA-motif	CGTCA	1659	5 +	Hordeum vulgare	the MeJA responsive
TaMAPKKK128	MSA-like	TCAAACGGT	1596	9 +	Catharanthus roseus	cell cycle regulation
TaMAPKKK128	ABRE	ACGTG	312	5 +	Arabidopsis thaliana	abscisic acid responsive
TaMAPKKK128	ABRE	ACGTG	356	5 +	Arabidopsis thaliana	abscisic acid responsive
TaMAPKKK128	ABRE	CACGTG	438	6 +	Arabidopsis thaliana	abscisic acid responsive

TaMAPKKK128	ABRE	ACGTG	439	5 +	Arabidopsis thaliana	abscisic acid responsive
TaMAPKKK128	ABRE	GACACGTACGT	1177	10 -	Oryza sativa	abscisic acid responsive
TaMAPKKK128	ABRE	CGCACGTGTC	1178	9 +	Hordeum vulgare	abscisic acid responsive
TaMAPKKK128	ABRE	ACGTG	1181	5 +	Arabidopsis thaliana	abscisic acid responsive
TaMAPKKK128	ABRE	CGTACGTGCA	1287	9 +	Hordeum vulgare	abscisic acid responsive
TaMAPKKK128	ABRE	ACGTG	1380	5 -	Arabidopsis thaliana	abscisic acid responsive
TaMAPKKK128	GC-motif	CCCCCG	1842	6 +	Zea mays	anoxic specific inducibility
TaMAPKKK128	P-box	CCTTTTG	393	7 -	Oryza sativa	gibberellin responsive
TaMAPKKK128	LTR	CCGAAA	459	6 +	Hordeum vulgare	low temperature responsive
TaMAPKKK128	LTR	CCGAAA	605	6 -	Hordeum vulgare	low temperature responsive
TaMAPKKK128	O2-site	GATGATGTGG	715	9 +	Zea mays	zein metabolism regulation
TaMAPKKK128	TGACG-motif	TGACG	1175	5 +	Hordeum vulgare	the MeJA responsive
TaMAPKKK128	TGACG-motif	TGACG	1613	5 +	Hordeum vulgare	the MeJA responsive
TaMAPKKK128	TGACG-motif	TGACG	1659	5 -	Hordeum vulgare	the MeJA responsive
TaMAPKKK128	TCA-element	CCATCTTTTT	60	9 -	Nicotiana tabacum	salicylic acid responsive
TaMAPKKK129	ARE	AAACCA	668	6 -	Zea mays	anaerobic induction
TaMAPKKK129	ARE	AAACCA	701	6 +	Zea mays	anaerobic induction
TaMAPKKK129	LTR	CCGAAA	1384	6 -	Hordeum vulgare	low temperature responsive
TaMAPKKK129	LTR	CCGAAA	1965	6 +	Hordeum vulgare	low temperature responsive

TaMAPKKK129	CGTCA-motif	CGTCA	1095	5 +	Hordeum vulgare	the MeJA responsive
TaMAPKKK129	CGTCA-motif	CGTCA	1574	5 -	Hordeum vulgare	the MeJA responsive
TaMAPKKK129	TGACG-motif	TGACG	1095	5 -	Hordeum vulgare	the MeJA responsive
TaMAPKKK129	TGACG-motif	TGACG	1574	5 +	Hordeum vulgare	the MeJA responsive
TaMAPKKK129	TC-rich repeats	ATTCTCTAAC	1302	9 +	Nicotiana tabacum	defense and stress responsive
TaMAPKKK129	ABRE	ACGTG	545	5 +	Arabidopsis thaliana	abscisic acid responsive
TaMAPKKK129	ABRE	ACGTG	853	5 -	Arabidopsis thaliana	abscisic acid responsive
TaMAPKKK129	ABRE	ACGTG	1253	5 +	Arabidopsis thaliana	abscisic acid responsive
TaMAPKKK129	TCA-element	CCATCTTTTT	110	9 -	Nicotiana tabacum	salicylic acid responsive
TaMAPKKK130	MBS	CAACTG	644	6 -	Arabidopsis thaliana	drought inducibility
TaMAPKKK130	CGTCA-motif	CGTCA	468	5 -	Hordeum vulgare	the MeJA responsive
TaMAPKKK130	CGTCA-motif	CGTCA	1109	5 -	Hordeum vulgare	the MeJA responsive
TaMAPKKK130	CGTCA-motif	CGTCA	1715	5 +	Hordeum vulgare	the MeJA responsive
TaMAPKKK130	CGTCA-motif	CGTCA	1757	5 -	Hordeum vulgare	the MeJA responsive
TaMAPKKK130	O2-site	GATGATGTGG	479	9 -	Zea mays	zein metabolism regulation
TaMAPKKK130	ABRE	ACGTG	1056	5 +	Arabidopsis thaliana	abscisic acid responsive
TaMAPKKK130	ABRE	ACGTG	1118	5 +	Arabidopsis thaliana	abscisic acid responsive
TaMAPKKK130	ABRE	ACGTG	1480	5 +	Arabidopsis thaliana	abscisic acid responsive
TaMAPKKK130	ABRE	ACGTG	1560	5 +	Arabidopsis thaliana	abscisic acid responsive

TaMAPKKK130	ABRE	ACGTG	1916	5 -	Arabidopsis thaliana	abscisic acid responsive
TaMAPKKK130	ARE	AAACCA	1128	6 -	Zea mays	anaerobic induction
TaMAPKKK130	CAT-box	GCCACT	19	6 +	Arabidopsis thaliana	meristem expression
TaMAPKKK130	CAT-box	GCCACT	1183	6 +	Arabidopsis thaliana	meristem expression
TaMAPKKK130	GARE-motif	TCTGTTG	856	7 +	Brassica oleracea	gibberellin responsive
TaMAPKKK130	SARE	TTCGACCATCT T	518	11 -	Nicotiana tabacum	salicylic acid responsive
TaMAPKKK130	AuxRR-core	GGTCCAT	1797	7 -	Nicotiana tabacum	auxin responsive
TaMAPKKK130	TGACG-motif	TGACG	468	5 +	Hordeum vulgare	the MeJA responsive
TaMAPKKK130	TGACG-motif	TGACG	1109	5 +	Hordeum vulgare	the MeJA responsive
TaMAPKKK130	TGACG-motif	TGACG	1715	5 -	Hordeum vulgare	the MeJA responsive
TaMAPKKK130	TGACG-motif	TGACG	1757	5 +	Hordeum vulgare	the MeJA responsive
TaMAPKKK130	GC-motif	CCCCCG	1474	6 +	Zea mays	anoxic specific inducibility
TaMAPKKK130	TGA-element	AACGAC	1362	6 +	Brassica oleracea	auxin responsive
TaMAPKKK130	TGA-element	AACGAC	1973	6 -	Brassica oleracea	auxin responsive
TaMAPKKK131	P-box	CCTTTTG	1279	7 -	Oryza sativa	gibberellin responsive
TaMAPKKK131	MBS	CAACTG	1795	6 +	Arabidopsis thaliana	drought inducibility
TaMAPKKK131	ABRE	ACGTG	309	5 -	Arabidopsis thaliana	abscisic acid responsive
TaMAPKKK131	ABRE	ACGTG	1167	5 +	Arabidopsis thaliana	abscisic acid responsive
TaMAPKKK131	ABRE	GCCGCGTGGC	1505	9 -	Oryza sativa	abscisic acid responsive
TaMAPKKK131	ABRE	ACGTG	1553	5 -	Arabidopsis thaliana	abscisic acid responsive

TaMAPKKK131	ABRE	ACGTG	1625	5 -	Arabidopsis thaliana	abscisic acid responsive
TaMAPKKK131	GC-motif	CCCCCG	1818	6 -	Zea mays	anoxic specific inducibility
TaMAPKKK131	CGTCA-motif	CGTCA	1165	5 -	Hordeum vulgare	the MeJA responsive
TaMAPKKK131	CGTCA-motif	CGTCA	1555	5 +	Hordeum vulgare	the MeJA responsive
TaMAPKKK131	CGTCA-motif	CGTCA	1571	5 +	Hordeum vulgare	the MeJA responsive
TaMAPKKK131	TGACG-motif	TGACG	1165	5 +	Hordeum vulgare	the MeJA responsive
TaMAPKKK131	TGACG-motif	TGACG	1555	5 -	Hordeum vulgare	the MeJA responsive
TaMAPKKK131	TGACG-motif	TGACG	1571	5 -	Hordeum vulgare	the MeJA responsive
TaMAPKKK131	LTR	CCGAAA	346	6 +	Hordeum vulgare	low temperature responsive
TaMAPKKK131	LTR	CCGAAA	1815	6 -	Hordeum vulgare	low temperature responsive
TaMAPKKK131	O2-site	GTTGACGTGA	1552	9 -	Zea mays	zein metabolism regulation
TaMAPKKK131	MSA-like	TCAAACGGT	1322	9 -	Catharanthus roseus	cell cycle regulation
TaMAPKKK131	CAT-box	GCCACT	473	6 -	Arabidopsis thaliana	meristem expression
TaMAPKKK132	ABRE	ACGTG	274	5 -	Arabidopsis thaliana	abscisic acid responsive
TaMAPKKK132	ABRE	AACCCGG	605	7 +	Arabidopsis thaliana	abscisic acid responsive
TaMAPKKK132	ABRE	GCCGCGTGGC	721	9 -	Oryza sativa	abscisic acid responsive
TaMAPKKK132	ABRE	ACGTG	1787	5 +	Arabidopsis thaliana	abscisic acid responsive
TaMAPKKK132	CGTCA-motif	CGTCA	1765	5 -	Hordeum vulgare	the MeJA responsive
TaMAPKKK132	TGACG-motif	TGACG	1765	5 +	Hordeum vulgare	the MeJA responsive

TaMAPKKK132	ARE	AAACCA	1047	6 +	Zea mays	anaerobic induction
TaMAPKKK132	GC-motif	CCCCCG	357	6 -	Zea mays	anoxic specific inducibility
TaMAPKKK132	GC-motif	CCCCCG	950	6 +	Zea mays	anoxic specific inducibility
TaMAPKKK133	GC-motif	CCCCCG	1947	6 +	Zea mays	anoxic specific inducibility
TaMAPKKK133	ABRE	ACGTG	792	5 +	Arabidopsis thaliana	abscisic acid responsive
TaMAPKKK133	CGTCA-motif	CGTCA	165	5 +	Hordeum vulgare	the MeJA responsive
TaMAPKKK133	CGTCA-motif	CGTCA	390	5 +	Hordeum vulgare	the MeJA responsive
TaMAPKKK133	CGTCA-motif	CGTCA	1788	5 -	Hordeum vulgare	the MeJA responsive
TaMAPKKK133	CAT-box	GCCACT	1163	6 -	Arabidopsis thaliana	meristem expression
TaMAPKKK133	MBS	CAACTG	9	6 -	Arabidopsis thaliana	drought inducibility
TaMAPKKK133	MBS	CAACTG	1267	6 -	Arabidopsis thaliana	drought inducibility
TaMAPKKK133	TC-rich repeats	GTTTTCTTAC	1206	9 +	Nicotiana tabacum	defense and stress responsive
TaMAPKKK133	TCA-element	CCATCTTTTT	283	9 +	Nicotiana tabacum	salicylic acid responsive
TaMAPKKK133	TGACG-motif	TGACG	165	5 -	Hordeum vulgare	the MeJA responsive
TaMAPKKK133	TGACG-motif	TGACG	390	5 -	Hordeum vulgare	the MeJA responsive
TaMAPKKK133	TGACG-motif	TGACG	1788	5 +	Hordeum vulgare	the MeJA responsive
TaMAPKKK133	LTR	CCGAAA	591	6 -	Hordeum vulgare	low temperature responsive
TaMAPKKK134	MSA-like	(T/C)C(T/C)AAC GG(T/C)(T/C)A	1916	8.5 -	Catharanthus roseus	cell cycle regulation

TaMAPKKK134	TGACG-motif	TGACG	1670	5 -	Hordeum vulgare	the MeJA responsive
TaMAPKKK134	MBS	CAACTG	1892	6 +	Arabidopsis thaliana	drought inducibility
TaMAPKKK134	CAT-box	GCCACT	587	6 -	Arabidopsis thaliana	meristem expression
TaMAPKKK134	CGTCA-motif	CGTCA	1670	5 +	Hordeum vulgare	the MeJA responsive
TaMAPKKK134	GC-motif	CCCCCG	1107	6 -	Zea mays	anoxic specific inducibility
TaMAPKKK134	ABRE	CACGTG	448	6 +	Arabidopsis thaliana	abscisic acid responsive
TaMAPKKK134	ABRE	ACGTG	449	5 +	Arabidopsis thaliana	abscisic acid responsive
TaMAPKKK134	ABRE	ACGTG	608	5 -	Arabidopsis thaliana	abscisic acid responsive
TaMAPKKK134	ABRE	ACGTG	1229	5 -	Arabidopsis thaliana	abscisic acid responsive
TaMAPKKK134	ABRE	CACGTG	1292	6 -	Arabidopsis thaliana	abscisic acid responsive
TaMAPKKK134	ABRE	ACGTG	1293	5 +	Arabidopsis thaliana	abscisic acid responsive
TaMAPKKK134	ABRE	GACACGTGGC	1445	9 -	Triticum aestivum	abscisic acid responsive
TaMAPKKK134	ABRE	ACGTG	1907	5 -	Arabidopsis thaliana	abscisic acid responsive
TaMAPKKK134	ABRE	GCAACGTGTC	1952	10 +	Hordeum vulgare	abscisic acid responsive
TaMAPKKK134	ABRE	ACGTG	1955	5 +	Arabidopsis thaliana	abscisic acid responsive
TaMAPKKK134	P-box	CCTTTTG	416	7 -	Oryza sativa	gibberellin responsive
TaMAPKKK134	P-box	CCTTTTG	1011	7 -	Oryza sativa	gibberellin responsive
TaMAPKKK134	TCA-element	CCATCTTTTT	1753	9 +	Nicotiana tabacum	salicylic acid responsive
TaMAPKKK135	GCN4_motif	TGAGTCA	1839	7 -	Oryza sativa	endosperm expression

TaMAPKKK135	TCA-element	CCATCTTTTT	134	9 +	Nicotiana tabacum	salicylic acid responsive
TaMAPKKK135	TCA-element	CCATCTTTTT	769	9 -	Nicotiana tabacum	salicylic acid responsive
TaMAPKKK135	TCA-element	CCATCTTTTT	1218	10 -	Nicotiana tabacum	salicylic acid responsive
TaMAPKKK135	AuxRR-core	GGTCCAT	698	7 +	Nicotiana tabacum	auxin responsive
TaMAPKKK135	CGTCA-motif	CGTCA	70	5 +	Hordeum vulgare	the MeJA responsive
TaMAPKKK135	CGTCA-motif	CGTCA	103	5 +	Hordeum vulgare	the MeJA responsive
TaMAPKKK135	ABRE	AACCCGG	1654	7 -	Arabidopsis thaliana	abscisic acid responsive
TaMAPKKK135	CAT-box	GCCACT	1967	6 -	Arabidopsis thaliana	meristem expression
TaMAPKKK135	ARE	AAACCA	10	6 +	Zea mays	anaerobic induction
TaMAPKKK135	ARE	AAACCA	26	6 +	Zea mays	anaerobic induction
TaMAPKKK135	TGACG-motif	TGACG	70	5 -	Hordeum vulgare	the MeJA responsive
TaMAPKKK135	TGACG-motif	TGACG	103	5 -	Hordeum vulgare	the MeJA responsive
TaMAPKKK136	TGA-element	AACGAC	703	6 +	Brassica oleracea	auxin responsive
TaMAPKKK136	TGA-element	AACGAC	1821	6 +	Brassica oleracea	auxin responsive
TaMAPKKK136	CAT-box	GCCACT	801	6 +	Arabidopsis thaliana	meristem expression
TaMAPKKK136	CAT-box	GCCACT	1848	6 +	Arabidopsis thaliana	meristem expression
TaMAPKKK136	CGTCA-motif	CGTCA	795	5 -	Hordeum vulgare	the MeJA responsive
TaMAPKKK136	CGTCA-motif	CGTCA	1540	5 +	Hordeum vulgare	the MeJA responsive
TaMAPKKK136	CGTCA-motif	CGTCA	1800	5 -	Hordeum vulgare	the MeJA responsive
TaMAPKKK136	ABRE	GCCGCGTGGC	121	9 -	Oryza sativa	abscisic acid responsive

TaMAPKKK136	ABRE	ACGTG	289	5 +	Arabidopsis thaliana	abscisic acid responsive
TaMAPKKK136	ABRE	CGTACGTGCA	1424	9 -	Hordeum vulgare	abscisic acid responsive
TaMAPKKK136	ABRE	ACGTG	1426	5 -	Arabidopsis thaliana	abscisic acid responsive
TaMAPKKK136	ABRE	ACGTG	1682	5 +	Arabidopsis thaliana	abscisic acid responsive
TaMAPKKK136	GC-motif	CCCCCG	206	6 -	Zea mays	anoxic specific inducibility
TaMAPKKK136	GC-motif	CCCCCG	374	6 +	Zea mays	anoxic specific inducibility
TaMAPKKK136	GC-motif	CCCCCG	881	6 +	Zea mays	anoxic specific inducibility
TaMAPKKK136	TGACG-motif	TGACG	795	5 +	Hordeum vulgare	the MeJA responsive
TaMAPKKK136	TGACG-motif	TGACG	1540	5 -	Hordeum vulgare	the MeJA responsive
TaMAPKKK136	TGACG-motif	TGACG	1800	5 +	Hordeum vulgare	the MeJA responsive
TaMAPKKK136	TCA-element	CCATCTTTTT	846	9 +	Nicotiana tabacum	salicylic acid responsive
TaMAPKKK136	ARE	AAACCA	1446	6 +	Zea mays	anaerobic induction
TaMAPKKK136	ARE	AAACCA	1631	6 -	Zea mays	anaerobic induction
TaMAPKKK136	O2-site	GATGA(C/T)(A/G)TG(A/G)	1005	8 -	Zea mays	zein metabolism regulation
TaMAPKKK136	O2-site	GATGACATGG	1246	9 +	Zea mays	zein metabolism regulation
TaMAPKKK136	LTR	CCGAAA	1768	6 +	Hordeum vulgare	low temperature responsive
TaMAPKKK137	CGTCA-motif	CGTCA	1385	5 -	Hordeum vulgare	the MeJA responsive
TaMAPKKK137	MBS	CAACTG	240	6 +	Arabidopsis thaliana	drought inducibility
TaMAPKKK137	MBS	CAACTG	410	6 -	Arabidopsis thaliana	drought inducibility

TaMAPKKK137	TCA-element	CCATCTTTTT	1959	9 +	Nicotiana tabacum	salicylic acid responsive
TaMAPKKK137	TGACG-motif	TGACG	1385	5 +	Hordeum vulgare	the MeJA responsive
TaMAPKKK137	CAT-box	GCCACT	942	6 -	Arabidopsis thaliana	meristem expression
TaMAPKKK137	CAT-box	GCCACT	1284	6 -	Arabidopsis thaliana	meristem expression
TaMAPKKK137	ABRE	ACGTG	1726	5 -	Arabidopsis thaliana	abscisic acid responsive
TaMAPKKK137	ABRE	ACGTG	1884	5 -	Arabidopsis thaliana	abscisic acid responsive
TaMAPKKK139	CGTCA-motif	CGTCA	157	5 +	Hordeum vulgare	the MeJA responsive
TaMAPKKK139	CGTCA-motif	CGTCA	477	5 -	Hordeum vulgare	the MeJA responsive
TaMAPKKK139	CGTCA-motif	CGTCA	519	5 -	Hordeum vulgare	the MeJA responsive
TaMAPKKK139	CGTCA-motif	CGTCA	1685	5 -	Hordeum vulgare	the MeJA responsive
TaMAPKKK139	TC-rich repeats	GTTTTCTTAC	1437	9 -	Nicotiana tabacum	defense and stress responsive
TaMAPKKK139	TCA-element	CCATCTTTTT	464	9 +	Nicotiana tabacum	salicylic acid responsive
TaMAPKKK139	TCA-element	TCAGAAGAGG	1914	9 -	Brassica oleracea	salicylic acid responsive
TaMAPKKK139	TGACG-motif	TGACG	157	5 -	Hordeum vulgare	the MeJA responsive
TaMAPKKK139	TGACG-motif	TGACG	477	5 +	Hordeum vulgare	the MeJA responsive
TaMAPKKK139	TGACG-motif	TGACG	519	5 +	Hordeum vulgare	the MeJA responsive
TaMAPKKK139	TGACG-motif	TGACG	1685	5 +	Hordeum vulgare	the MeJA responsive
TaMAPKKK139	ARE	AAACCA	932	6 +	Zea mays	anaerobic induction
TaMAPKKK139	ABRE	TACGGTC	166	7 -	Arabidopsis thaliana	abscisic acid responsive

TaMAPKKK139	ABRE	ACGTG	172	5 +	Arabidopsis thaliana	abscisic acid responsive
TaMAPKKK139	ABRE	ACGTG	231	5 -	Arabidopsis thaliana	abscisic acid responsive
TaMAPKKK139	WUN-motif	AAATTTCCT	356	9 -	Brassica oleracea	wound responsive
TaMAPKKK139	GARE-motif	TCTGTTG	386	7 -	Brassica oleracea	gibberellin responsive
TaMAPKKK139	CAT-box	GCCACT	710	6 -	Arabidopsis thaliana	meristem expression
TaMAPKKK14	ABRE	ACGTG	108	5 +	Arabidopsis thaliana	abscisic acid responsive
TaMAPKKK14	ABRE	ACGTG	538	5 -	Arabidopsis thaliana	abscisic acid responsive
TaMAPKKK14	ABRE	ACGTG	1416	5 +	Arabidopsis thaliana	abscisic acid responsive
TaMAPKKK14	LTR	CCGAAA	806	6 -	Hordeum vulgare	low temperature responsive
TaMAPKKK14	CGTCA-motif	CGTCA	1982	5 -	Hordeum vulgare	the MeJA responsive
TaMAPKKK14	TGA-element	AACGAC	323	6 +	Brassica oleracea	auxin responsive
TaMAPKKK14	TGA-element	AACGAC	1968	6 +	Brassica oleracea	auxin responsive
TaMAPKKK14	ARE	AAACCA	1753	6 +	Zea mays	anaerobic induction
TaMAPKKK14	ARE	AAACCA	1801	6 +	Zea mays	anaerobic induction
TaMAPKKK14	O2-site	GATGATGTGG	454	9 +	Zea mays	zein metabolism regulation
TaMAPKKK14	O2-site	GATGACATGG	495	9 -	Zea mays	zein metabolism regulation
TaMAPKKK14	TGACG-motif	TGACG	1982	5 +	Hordeum vulgare	the MeJA responsive
TaMAPKKK14	P-box	CCTTTTG	970	7 +	Oryza sativa	gibberellin responsive
TaMAPKKK14	CAT-box	GCCACT	1583	6 -	Arabidopsis thaliana	meristem expression
TaMAPKKK14	CAT-box	GCCACT	1623	6 -	Arabidopsis thaliana	meristem expression

TaMAPKKK140	RY-element	CATGCATG	383	8 +	Helianthus annuus	seed specific regulation
TaMAPKKK140	ABRE	GCAACGTGTC	192	9 +	Hordeum vulgare	abscisic acid responsive
TaMAPKKK140	ABRE	ACGTG	569	5 -	Arabidopsis thaliana	abscisic acid responsive
TaMAPKKK140	MBS	CAACTG	13	6 +	Arabidopsis thaliana	drought inducibility
TaMAPKKK140	ARE	AAACCA	927	6 -	Zea mays	anaerobic induction
TaMAPKKK140	ARE	AAACCA	1596	6 -	Zea mays	anaerobic induction
TaMAPKKK140	GCN4_motif	TGAGTCA	594	7 +	Oryza sativa	endosperm expression
TaMAPKKK140	LTR	CCGAAA	540	6 -	Hordeum vulgare	low temperature responsive
TaMAPKKK140	TGACG-motif	TGACG	1228	5 +	Hordeum vulgare	the MeJA responsive
TaMAPKKK140	CGTCA-motif	CGTCA	1228	5 -	Hordeum vulgare	the MeJA responsive
TaMAPKKK140	AuxRR-core	GGTCCAT	1182	7 -	Nicotiana tabacum	auxin responsive
TaMAPKKK141	TC-rich repeats	GTTTTCTTAC	718	9 +	Nicotiana tabacum	defense and stress responsive
TaMAPKKK141	TC-rich repeats	ATTCTCTAAC	1735	9 +	Nicotiana tabacum	defense and stress responsive
TaMAPKKK141	O2-site	GATGATGTGG	1370	9 +	Zea mays	zein metabolism regulation
TaMAPKKK141	TCA-element	CCATCTTTTT	1590	9 +	Nicotiana tabacum	salicylic acid responsive
TaMAPKKK141	ABRE	ACGTG	203	5 -	Arabidopsis thaliana	abscisic acid responsive
TaMAPKKK141	TGA-element	AACGAC	441	6 +	Brassica oleracea	auxin responsive
TaMAPKKK141	TGA-element	AACGAC	820	6 -	Brassica oleracea	auxin responsive
TaMAPKKK141	ARE	AAACCA	988	6 -	Zea mays	anaerobic induction
TaMAPKKK141	ARE	AAACCA	1130	6 -	Zea mays	anaerobic induction

TaMAPKKK141	ARE	AAACCA	1877	6 -	Zea mays	anaerobic induction
TaMAPKKK141	P-box	CCTTTTG	839	7 -	Oryza sativa	gibberellin responsive
TaMAPKKK141	P-box	CCTTTTG	1340	7 +	Oryza sativa	gibberellin responsive
TaMAPKKK141	TGACG-motif	TGACG	290	5 -	Hordeum vulgare	the MeJA responsive
TaMAPKKK141	TGACG-motif	TGACG	355	5 -	Hordeum vulgare	the MeJA responsive
TaMAPKKK141	TGACG-motif	TGACG	460	5 -	Hordeum vulgare	the MeJA responsive
TaMAPKKK141	TGACG-motif	TGACG	502	5 +	Hordeum vulgare	the MeJA responsive
TaMAPKKK141	TGACG-motif	TGACG	898	5 +	Hordeum vulgare	the MeJA responsive
TaMAPKKK141	TGACG-motif	TGACG	958	5 +	Hordeum vulgare	the MeJA responsive
TaMAPKKK141	TGACG-motif	TGACG	1410	5 -	Hordeum vulgare	the MeJA responsive
TaMAPKKK141	CGTCA-motif	CGTCA	290	5 +	Hordeum vulgare	the MeJA responsive
TaMAPKKK141	CGTCA-motif	CGTCA	355	5 +	Hordeum vulgare	the MeJA responsive
TaMAPKKK141	CGTCA-motif	CGTCA	460	5 +	Hordeum vulgare	the MeJA responsive
TaMAPKKK141	CGTCA-motif	CGTCA	502	5 -	Hordeum vulgare	the MeJA responsive
TaMAPKKK141	CGTCA-motif	CGTCA	898	5 -	Hordeum vulgare	the MeJA responsive
TaMAPKKK141	CGTCA-motif	CGTCA	958	5 -	Hordeum vulgare	the MeJA responsive
TaMAPKKK141	CGTCA-motif	CGTCA	1410	5 +	Hordeum vulgare	the MeJA responsive
TaMAPKKK142	TCA-element	TCAGAAGAGG	1933	9 -	Brassica oleracea	salicylic acid responsive
TaMAPKKK142	ARE	AAACCA	470	6 -	Zea mays	anaerobic induction

TaMAPKKK142	ARE	AAACCA	560	6 -	Zea mays	anaerobic induction
TaMAPKKK142	ARE	AAACCA	733	6 -	Zea mays	anaerobic induction
TaMAPKKK142	ARE	AAACCA	1347	6 -	Zea mays	anaerobic induction
TaMAPKKK142	MBS	CAACTG	1523	6 -	Arabidopsis thaliana	drought inducibility
TaMAPKKK142	CGTCA-motif	CGTCA	169	5 -	Hordeum vulgare	the MeJA responsive
TaMAPKKK142	CGTCA-motif	CGTCA	212	5 +	Hordeum vulgare	the MeJA responsive
TaMAPKKK142	CGTCA-motif	CGTCA	1959	5 +	Hordeum vulgare	the MeJA responsive
TaMAPKKK142	CAT-box	GCCACT	373	6 -	Arabidopsis thaliana	meristem expression
TaMAPKKK142	CAT-box	GCCACT	699	6 -	Arabidopsis thaliana	meristem expression
TaMAPKKK142	TGA-element	AACGAC	1715	6 +	Brassica oleracea	auxin responsive
TaMAPKKK142	P-box	CCTTTTG	1799	7 -	Oryza sativa	gibberellin responsive
TaMAPKKK142	circadian	CAAAGATATC	1234	9 -	Lycopersicon esculentum	circadian control
TaMAPKKK142	LTR	CCGAAA	77	6 -	Hordeum vulgare	low temperature responsive
TaMAPKKK142	AuxRR-core	GGTCCAT	411	7 +	Nicotiana tabacum	auxin responsive
TaMAPKKK142	TGACG-motif	TGACG	169	5 +	Hordeum vulgare	the MeJA responsive
TaMAPKKK142	TGACG-motif	TGACG	212	5 -	Hordeum vulgare	the MeJA responsive
TaMAPKKK142	TGACG-motif	TGACG	1959	5 -	Hordeum vulgare	the MeJA responsive
TaMAPKKK142	motif I	gGTACGTGGCG	295	10 +	Oryza sativa	root specific
TaMAPKKK142	GC-motif	CCCCCG	614	6 -	Zea mays	anoxic specific inducibility

TaMAPKKK142	GC-motif	CCCCCG	1138	6 +	Zea mays	anoxic specific inducibility
TaMAPKKK142	ABRE	ACGTG	298	5 +	Arabidopsis thaliana	abscisic acid responsive
TaMAPKKK142	ABRE	ACGTG	453	5 +	Arabidopsis thaliana	abscisic acid responsive
TaMAPKKK142	ABRE	ACGTG	718	5 +	Arabidopsis thaliana	abscisic acid responsive
TaMAPKKK142	ABRE	ACGTG	1211	5 -	Arabidopsis thaliana	abscisic acid responsive
TaMAPKKK144	TGA-element	AACGAC	1769	6 -	Brassica oleracea	auxin responsive
TaMAPKKK144	CAT-box	GCCACT	878	6 -	Arabidopsis thaliana	meristem expression
TaMAPKKK144	ABRE	ACGTG	372	5 -	Arabidopsis thaliana	abscisic acid responsive
TaMAPKKK144	ABRE	AACCCGG	1063	7 +	Arabidopsis thaliana	abscisic acid responsive
TaMAPKKK144	ARE	AAACCA	193	6 +	Zea mays	anaerobic induction
TaMAPKKK144	ARE	AAACCA	1106	6 -	Zea mays	anaerobic induction
TaMAPKKK144	TGACG-motif	TGACG	482	5 +	Hordeum vulgare	the MeJA responsive
TaMAPKKK144	TGACG-motif	TGACG	575	5 -	Hordeum vulgare	the MeJA responsive
TaMAPKKK144	TGACG-motif	TGACG	695	5 +	Hordeum vulgare	the MeJA responsive
TaMAPKKK144	TGACG-motif	TGACG	1681	5 +	Hordeum vulgare	the MeJA responsive
TaMAPKKK144	O2-site	GATGACATGG	470	9 -	Zea mays	zein metabolism regulation
TaMAPKKK144	TATC-box	TATCCCA	1190	7 +	Oryza sativa	gibberellin responsive
TaMAPKKK144	CGTCA-motif	CGTCA	482	5 -	Hordeum vulgare	the MeJA responsive
TaMAPKKK144	CGTCA-motif	CGTCA	575	5 +	Hordeum vulgare	the MeJA responsive

TaMAPKKK144	CGTCA-motif	CGTCA	695	5 -	Hordeum vulgare	the MeJA responsive
TaMAPKKK144	CGTCA-motif	CGTCA	1681	5 -	Hordeum vulgare	the MeJA responsive
TaMAPKKK145	MBS	CAACTG	1673	6 +	Arabidopsis thaliana	drought inducibility
TaMAPKKK145	P-box	CCTTTTG	770	7 +	Oryza sativa	gibberellin responsive
TaMAPKKK145	GC-motif	CCCCCG	615	6 -	Zea mays	anoxic specific inducibility
TaMAPKKK145	ABRE	ACGTG	493	5 -	Arabidopsis thaliana	abscisic acid responsive
TaMAPKKK145	ABRE	ACGTG	1045	5 +	Arabidopsis thaliana	abscisic acid responsive
TaMAPKKK145	TGA-element	AACGAC	551	6 +	Brassica oleracea	auxin responsive
TaMAPKKK145	CGTCA-motif	CGTCA	505	5 +	Hordeum vulgare	the MeJA responsive
TaMAPKKK145	CGTCA-motif	CGTCA	1858	5 +	Hordeum vulgare	the MeJA responsive
TaMAPKKK145	O2-site	GATGATGTGG	1933	9 -	Zea mays	zein metabolism regulation
TaMAPKKK145	GCN4_motif	TGAGTCA	1157	7 -	Oryza sativa	endosperm expression
TaMAPKKK145	GCN4_motif	TGAGTCA	1190	7 -	Oryza sativa	endosperm expression
TaMAPKKK145	GCN4_motif	TGAGTCA	1234	7 +	Oryza sativa	endosperm expression
TaMAPKKK145	LTR	CCGAAA	284	6 -	Hordeum vulgare	low temperature responsive
TaMAPKKK145	LTR	CCGAAA	649	6 -	Hordeum vulgare	low temperature responsive
TaMAPKKK145	TCA-element	CCATCTTTTT	1056	9 +	Nicotiana tabacum	salicylic acid responsive
TaMAPKKK145	TGACG-motif	TGACG	505	5 -	Hordeum vulgare	the MeJA responsive
TaMAPKKK145	TGACG-motif	TGACG	1858	5 -	Hordeum vulgare	the MeJA responsive

TaMAPKKK146	TCA-element	CCATCTTTTT	76	9 -	Nicotiana tabacum	salicylic acid responsive
TaMAPKKK146	TCA-element	CCATCTTTTT	990	9 +	Nicotiana tabacum	salicylic acid responsive
TaMAPKKK146	TGACG-motif	TGACG	1198	5 -	Hordeum vulgare	the MeJA responsive
TaMAPKKK146	TGACG-motif	TGACG	1889	5 +	Hordeum vulgare	the MeJA responsive
TaMAPKKK146	TGACG-motif	TGACG	1928	5 -	Hordeum vulgare	the MeJA responsive
TaMAPKKK146	ARE	AAACCA	192	6 +	Zea mays	anaerobic induction
TaMAPKKK146	ARE	AAACCA	374	6 +	Zea mays	anaerobic induction
TaMAPKKK146	ARE	AAACCA	469	6 +	Zea mays	anaerobic induction
TaMAPKKK146	ARE	AAACCA	900	6 -	Zea mays	anaerobic induction
TaMAPKKK146	HD-Zip 1	CAAT(A/T)ATTG	893	8 +	Arabidopsis thaliana	differentiation of the palisade mesophyll cells
TaMAPKKK146	LTR	CCGAAA	1683	6 +	Hordeum vulgare	low temperature responsive
TaMAPKKK146	O2-site	GATGACATGG	195	9 -	Zea mays	zein metabolism regulation
TaMAPKKK146	O2-site	GATGA(C/T)(A/G)TG(A/G)	1755	8 -	Zea mays	zein metabolism regulation
TaMAPKKK146	CGTCA-motif	CGTCA	1198	5 +	Hordeum vulgare	the MeJA responsive
TaMAPKKK146	CGTCA-motif	CGTCA	1889	5 -	Hordeum vulgare	the MeJA responsive
TaMAPKKK146	CGTCA-motif	CGTCA	1928	5 +	Hordeum vulgare	the MeJA responsive
TaMAPKKK146	CAT-box	GCCACT	1731	6 -	Arabidopsis thaliana	meristem expression
TaMAPKKK146	TGA-element	AACGAC	1352	6 -	Brassica oleracea	auxin responsive
TaMAPKKK146	GC-motif	CCCCCG	1984	6 +	Zea mays	anoxic specific inducibility

TaMAPKKK146	ABRE	GCCGCGTGGC	1735	9 -	Oryza sativa	abscisic acid responsive
TaMAPKKK146	P-box	CCTTTTG	10	7 -	Oryza sativa	gibberellin responsive
TaMAPKKK146	TC-rich repeats	GTTTTCTTAC	1403	9 +	Nicotiana tabacum	defense and stress responsive
TaMAPKKK146	MBS	CAACTG	716	6 +	Arabidopsis thaliana	drought inducibility
TaMAPKKK147	ABRE	ACGTG	483	5 +	Arabidopsis thaliana	abscisic acid responsive
TaMAPKKK147	ABRE	ACGTG	1135	5 -	Arabidopsis thaliana	abscisic acid responsive
TaMAPKKK147	ABRE	AACCCGG	1426	7 -	Arabidopsis thaliana	abscisic acid responsive
TaMAPKKK147	ABRE	GCAACGTGTC	1890	9 -	Hordeum vulgare	abscisic acid responsive
TaMAPKKK147	ABRE	ACGTG	1892	5 -	Arabidopsis thaliana	abscisic acid responsive
TaMAPKKK147	GC-motif	CCCCCG	134	6 -	Zea mays	anoxic specific inducibility
TaMAPKKK147	GC-motif	CCCCCG	291	6 +	Zea mays	anoxic specific inducibility
TaMAPKKK147	CAT-box	GCCACT	536	6 +	Arabidopsis thaliana	meristem expression
TaMAPKKK147	CAT-box	GCCACT	952	6 -	Arabidopsis thaliana	meristem expression
TaMAPKKK147	CGTCA-motif	CGTCA	407	5 -	Hordeum vulgare	the MeJA responsive
TaMAPKKK147	CGTCA-motif	CGTCA	456	5 -	Hordeum vulgare	the MeJA responsive
TaMAPKKK147	CGTCA-motif	CGTCA	481	5 -	Hordeum vulgare	the MeJA responsive
TaMAPKKK147	CGTCA-motif	CGTCA	1313	5 +	Hordeum vulgare	the MeJA responsive
TaMAPKKK147	CGTCA-motif	CGTCA	1855	5 +	Hordeum vulgare	the MeJA responsive
TaMAPKKK147	CGTCA-motif	CGTCA	1947	5 +	Hordeum vulgare	the MeJA responsive

TaMAPKKK147	ARE	AAACCA	1280	6 +	Zea mays	anaerobic induction
TaMAPKKK147	ARE	AAACCA	1905	6 +	Zea mays	anaerobic induction
TaMAPKKK147	TGACG-motif	TGACG	407	5 +	Hordeum vulgare	the MeJA responsive
TaMAPKKK147	TGACG-motif	TGACG	456	5 +	Hordeum vulgare	the MeJA responsive
TaMAPKKK147	TGACG-motif	TGACG	481	5 +	Hordeum vulgare	the MeJA responsive
TaMAPKKK147	TGACG-motif	TGACG	1313	5 -	Hordeum vulgare	the MeJA responsive
TaMAPKKK147	TGACG-motif	TGACG	1855	5 -	Hordeum vulgare	the MeJA responsive
TaMAPKKK147	TGACG-motif	TGACG	1947	5 -	Hordeum vulgare	the MeJA responsive
TaMAPKKK147	O2-site	GATGACATGG	878	9 -	Zea mays	zein metabolism regulation
TaMAPKKK147	GCN4_motif	TGAGTCA	1090	7 +	Oryza sativa	endosperm expression
TaMAPKKK147	AuxRR-core	GGTCCAT	32	7 -	Nicotiana tabacum	auxin responsive
TaMAPKKK147	LTR	CCGAAA	1712	6 -	Hordeum vulgare	low temperature responsive
TaMAPKKK148	CGTCA-motif	CGTCA	621	5 -	Hordeum vulgare	the MeJA responsive
TaMAPKKK148	CGTCA-motif	CGTCA	653	5 -	Hordeum vulgare	the MeJA responsive
TaMAPKKK148	CGTCA-motif	CGTCA	663	5 -	Hordeum vulgare	the MeJA responsive
TaMAPKKK148	CGTCA-motif	CGTCA	772	5 -	Hordeum vulgare	the MeJA responsive
TaMAPKKK148	TGA-element	AACGAC	935	6 +	Brassica oleracea	auxin responsive
TaMAPKKK148	GC-motif	CCCCCG	1236	6 +	Zea mays	anoxic specific inducibility
TaMAPKKK148	ABRE	CACGTG	473	6 +	Arabidopsis thaliana	abscisic acid responsive

TaMAPKKK148	ABRE	ACGTG	474	5 +	Arabidopsis thaliana	abscisic acid responsive
TaMAPKKK148	ABRE	CGCACGTGTC	877	9 +	Hordeum vulgare	abscisic acid responsive
TaMAPKKK148	ABRE	CACGTG	879	6 +	Arabidopsis thaliana	abscisic acid responsive
TaMAPKKK148	ABRE	ACGTG	880	5 +	Arabidopsis thaliana	abscisic acid responsive
TaMAPKKK148	ABRE	AACCCGG	1044	7 +	Arabidopsis thaliana	abscisic acid responsive
TaMAPKKK148	ABRE	ACGTG	1123	5 -	Arabidopsis thaliana	abscisic acid responsive
TaMAPKKK148	ABRE	CACGTG	1486	6 -	Arabidopsis thaliana	abscisic acid responsive
TaMAPKKK148	ABRE	ACGTG	1487	5 +	Arabidopsis thaliana	abscisic acid responsive
TaMAPKKK148	TGA-box	TGACGTAA	653	8 +	Glycine max	auxin responsive
TaMAPKKK148	MBS	CAACTG	149	6 +	Arabidopsis thaliana	drought inducibility
TaMAPKKK148	MBS	CAACTG	1725	6 -	Arabidopsis thaliana	drought inducibility
TaMAPKKK148	TCA-element	TCAGAAGAGG	230	9 -	Brassica oleracea	salicylic acid responsive
TaMAPKKK148	TGACG-motif	TGACG	621	5 +	Hordeum vulgare	the MeJA responsive
TaMAPKKK148	TGACG-motif	TGACG	653	5 +	Hordeum vulgare	the MeJA responsive
TaMAPKKK148	TGACG-motif	TGACG	663	5 +	Hordeum vulgare	the MeJA responsive
TaMAPKKK148	TGACG-motif	TGACG	772	5 +	Hordeum vulgare	the MeJA responsive
TaMAPKKK148	ARE	AAACCA	1864	6 -	Zea mays	anaerobic induction
TaMAPKKK148	circadian	CAAAGATATC	1557	9 +	Lycopersicon esculentum	circadian control
TaMAPKKK148	O2-site	GTTGACGTGA	1483	9 +	Zea mays	zein metabolism regulation

TaMAPKKK15	CGTCA-motif	CGTCA	303	5 +	Hordeum vulgare	the MeJA responsive
TaMAPKKK15	CGTCA-motif	CGTCA	690	5 -	Hordeum vulgare	the MeJA responsive
TaMAPKKK15	ABRE	ACGTG	85	5 -	Arabidopsis thaliana	abscisic acid responsive
TaMAPKKK15	circadian	CAAAGATATC	1706	9 +	Lycopersicon esculentum	circadian control
TaMAPKKK15	GC-motif	CCCCCG	1962	6 +	Zea mays	anoxic specific inducibility
TaMAPKKK15	ARE	AAACCA	1027	6 +	Zea mays	anaerobic induction
TaMAPKKK15	TGACG-motif	TGACG	303	5 -	Hordeum vulgare	the MeJA responsive
TaMAPKKK15	TGACG-motif	TGACG	690	5 +	Hordeum vulgare	the MeJA responsive
TaMAPKKK15	MBS	CAACTG	1182	6 +	Arabidopsis thaliana	drought inducibility
TaMAPKKK150	CGTCA-motif	CGTCA	538	5 +	Hordeum vulgare	the MeJA responsive
TaMAPKKK150	CGTCA-motif	CGTCA	616	5 -	Hordeum vulgare	the MeJA responsive
TaMAPKKK150	AuxRR-core	GGTCCAT	575	7 -	Nicotiana tabacum	auxin responsive
TaMAPKKK150	MBS	CAACTG	231	6 +	Arabidopsis thaliana	drought inducibility
TaMAPKKK150	TC-rich repeats	ATTCTCTAAC	1325	9 -	Nicotiana tabacum	defense and stress responsive
TaMAPKKK150	TGACG-motif	TGACG	538	5 -	Hordeum vulgare	the MeJA responsive
TaMAPKKK150	TGACG-motif	TGACG	616	5 +	Hordeum vulgare	the MeJA responsive
TaMAPKKK150	ARE	AAACCA	1103	6 -	Zea mays	anaerobic induction
TaMAPKKK150	ARE	AAACCA	1490	6 -	Zea mays	anaerobic induction
TaMAPKKK150	ABRE	ACGTG	509	5 -	Arabidopsis thaliana	abscisic acid responsive

TaMAPKKK150	ABRE	GCCGCGTGGC	1236	9 +	Oryza sativa	abscisic acid responsive
TaMAPKKK150	TGA-element	AACGAC	560	6 +	Brassica oleracea	auxin responsive
TaMAPKKK151	LTR	CCGAAA	1603	6 -	Hordeum vulgare	low temperature responsive
TaMAPKKK151	CGTCA-motif	CGTCA	785	5 +	Hordeum vulgare	the MeJA responsive
TaMAPKKK151	O2-site	GATGATGTGG	637	9 +	Zea mays	zein metabolism regulation
TaMAPKKK151	O2-site	GATGATGTGG	1064	9 +	Zea mays	zein metabolism regulation
TaMAPKKK151	O2-site	GATGA(C/T)(A/G)TG(A/G)	1219	8 -	Zea mays	zein metabolism regulation
TaMAPKKK151	GC-motif	CCCCCG	1610	6 -	Zea mays	anoxic specific inducibility
TaMAPKKK151	P-box	CCTTTTG	1139	7 +	Oryza sativa	gibberellin responsive
TaMAPKKK151	MBS	CAACTG	331	6 +	Arabidopsis thaliana	drought inducibility
TaMAPKKK151	MBS	CAACTG	1944	6 +	Arabidopsis thaliana	drought inducibility
TaMAPKKK151	ARE	AAACCA	838	6 -	Zea mays	anaerobic induction
TaMAPKKK151	ARE	AAACCA	1199	6 +	Zea mays	anaerobic induction
TaMAPKKK151	ARE	AAACCA	1647	6 -	Zea mays	anaerobic induction
TaMAPKKK151	TGACG-motif	TGACG	785	5 -	Hordeum vulgare	the MeJA responsive
TaMAPKKK152	TGA-element	AACGAC	823	6 +	Brassica oleracea	auxin responsive
TaMAPKKK152	TGA-element	AACGAC	862	6 +	Brassica oleracea	auxin responsive
TaMAPKKK152	TGA-element	AACGAC	1425	6 +	Brassica oleracea	auxin responsive
TaMAPKKK152	TGA-element	AACGAC	1772	6 +	Brassica oleracea	auxin responsive
TaMAPKKK152	ABRE	ACGTG	474	5 -	Arabidopsis thaliana	abscisic acid responsive
TaMAPKKK152	ABRE	ACGTG	562	5 +	Arabidopsis thaliana	abscisic acid responsive

TaMAPKKK152	ABRE	ACGTG	1272	5 -	Arabidopsis thaliana	abscisic acid responsive
TaMAPKKK152	CAT-box	GCCACT	171	6 -	Arabidopsis thaliana	meristem expression
TaMAPKKK152	CAT-box	GCCACT	739	6 +	Arabidopsis thaliana	meristem expression
TaMAPKKK152	CAT-box	GCCACT	1254	6 -	Arabidopsis thaliana	meristem expression
TaMAPKKK152	ARE	AAACCA	635	6 -	Zea mays	anaerobic induction
TaMAPKKK152	ARE	AAACCA	1587	6 +	Zea mays	anaerobic induction
TaMAPKKK152	TCA-element	CCATCTTTTT	142	9 +	Nicotiana tabacum	salicylic acid responsive
TaMAPKKK152	TCA-element	TCAGAAGAGG	383	9 +	Brassica oleracea	salicylic acid responsive
TaMAPKKK152	TCA-element	CCATCTTTTT	1308	9 +	Nicotiana tabacum	salicylic acid responsive
TaMAPKKK152	CGTCA-motif	CGTCA	420	5 +	Hordeum vulgare	the MeJA responsive
TaMAPKKK152	LTR	CCGAAA	93	6 +	Hordeum vulgare	low temperature responsive
TaMAPKKK152	LTR	CCGAAA	872	6 +	Hordeum vulgare	low temperature responsive
TaMAPKKK152	MSA-like	(T/C)C(T/C)AAC GG(T/C)(T/C)A	1168	8.5 -	Catharanthus roseus	cell cycle regulation
TaMAPKKK152	TGACG-motif	TGACG	420	5 -	Hordeum vulgare	the MeJA responsive
TaMAPKKK153	GARE-motif	TCTGTTG	271	7 -	Brassica oleracea	gibberellin responsive
TaMAPKKK153	TGACG-motif	TGACG	676	5 +	Hordeum vulgare	the MeJA responsive
TaMAPKKK153	TGACG-motif	TGACG	1065	5 -	Hordeum vulgare	the MeJA responsive
TaMAPKKK153	motif I	gGTACGTGGCG	1459	10 +	Oryza sativa	root specific

TaMAPKKK153	O2-site	GATGATGTGG	1756	9 -	Zea mays	zein metabolism regulation
TaMAPKKK153	GC-motif	CCCCCG	1206	6 -	Zea mays	anoxic specific inducibility
TaMAPKKK153	GC-motif	CCCCCG	1872	6 -	Zea mays	anoxic specific inducibility
TaMAPKKK153	P-box	CCTTTTG	624	7 -	Oryza sativa	gibberellin responsive
TaMAPKKK153	MBS	CAACTG	126	6 +	Arabidopsis thaliana	drought inducibility
TaMAPKKK153	TATC-box	TATCCCA	205	7 -	Oryza sativa	gibberellin responsive
TaMAPKKK153	CGTCA-motif	CGTCA	676	5 -	Hordeum vulgare	the MeJA responsive
TaMAPKKK153	CGTCA-motif	CGTCA	1065	5 +	Hordeum vulgare	the MeJA responsive
TaMAPKKK154	CAT-box	GCCACT	1939	6 -	Arabidopsis thaliana	meristem expression
TaMAPKKK154	ABRE	ACGTG	722	5 -	Arabidopsis thaliana	abscisic acid responsive
TaMAPKKK154	ABRE	ACGTG	833	5 -	Arabidopsis thaliana	abscisic acid responsive
TaMAPKKK154	ABRE	ACGTG	1983	5 -	Arabidopsis thaliana	abscisic acid responsive
TaMAPKKK154	TGACG-motif	TGACG	308	5 -	Hordeum vulgare	the MeJA responsive
TaMAPKKK154	O2-site	GATGATGTGG	1734	10 +	Zea mays	zein metabolism regulation
TaMAPKKK154	O2-site	GATGA(C/T)(A/G)TG(A/G)	1856	8 +	Zea mays	zein metabolism regulation
TaMAPKKK154	RY-element	CATGCATG	1352	8 -	Helianthus annuus	seed specific regulation
TaMAPKKK154	MBS	CAACTG	287	6 -	Arabidopsis thaliana	drought inducibility
TaMAPKKK154	P-box	CCTTTTG	50	7 +	Oryza sativa	gibberellin responsive
TaMAPKKK154	P-box	CCTTTTG	1136	7 +	Oryza sativa	gibberellin responsive

TaMAPKKK154	P-box	CCTTTTG	1361	7 -	Oryza sativa	gibberellin responsive
TaMAPKKK154	CGTCA-motif	CGTCA	308	5 +	Hordeum vulgare	the MeJA responsive
TaMAPKKK154	LTR	CCGAAA	530	6 +	Hordeum vulgare	low temperature responsive
TaMAPKKK155	GC-motif	CCCCCG	1728	6 -	Zea mays	anoxic specific inducibility
TaMAPKKK155	circadian	CAAAGATATC	1858	9 +	Lycopersicon esculentum	circadian control
TaMAPKKK155	TCA-element	CCATCTTTTT	1884	9 +	Nicotiana tabacum	salicylic acid responsive
TaMAPKKK155	MBS	CAACTG	244	6 -	Arabidopsis thaliana	drought inducibility
TaMAPKKK155	MBS	CAACTG	1926	6 -	Arabidopsis thaliana	drought inducibility
TaMAPKKK155	ARE	AAACCA	108	6 -	Zea mays	anaerobic induction
TaMAPKKK155	ARE	AAACCA	1182	6 -	Zea mays	anaerobic induction
TaMAPKKK155	ARE	AAACCA	1576	6 +	Zea mays	anaerobic induction
TaMAPKKK155	ARE	AAACCA	1933	6 -	Zea mays	anaerobic induction
TaMAPKKK155	LTR	CCGAAA	1185	6 -	Hordeum vulgare	low temperature responsive
TaMAPKKK16	ARE	AAACCA	1409	6 +	Zea mays	anaerobic induction
TaMAPKKK16	TGACG-motif	TGACG	61	5 +	Hordeum vulgare	the MeJA responsive
TaMAPKKK16	TGACG-motif	TGACG	121	5 -	Hordeum vulgare	the MeJA responsive
TaMAPKKK16	TGACG-motif	TGACG	996	5 -	Hordeum vulgare	the MeJA responsive
TaMAPKKK16	TGACG-motif	TGACG	1110	5 +	Hordeum vulgare	the MeJA responsive
TaMAPKKK16	LTR	CCGAAA	271	6 -	Hordeum vulgare	low temperature responsive

TaMAPKKK16	MBS	CAACTG	1556	6 +	Arabidopsis thaliana	drought inducibility
TaMAPKKK16	CAT-box	GCCACT	1306	6 +	Arabidopsis thaliana	meristem expression
TaMAPKKK16	O2-site	GATGATGTGG	547	9 -	Zea mays	zein metabolism regulation
TaMAPKKK16	O2-site	GATGATGTGG	728	9 -	Zea mays	zein metabolism regulation
TaMAPKKK16	O2-site	GATGATGTGG	1566	9 -	Zea mays	zein metabolism regulation
TaMAPKKK16	CGTCA-motif	CGTCA	61	5 -	Hordeum vulgare	the MeJA responsive
TaMAPKKK16	CGTCA-motif	CGTCA	121	5 +	Hordeum vulgare	the MeJA responsive
TaMAPKKK16	CGTCA-motif	CGTCA	996	5 +	Hordeum vulgare	the MeJA responsive
TaMAPKKK16	CGTCA-motif	CGTCA	1110	5 -	Hordeum vulgare	the MeJA responsive
TaMAPKKK16	ABRE	GCCGCGTGGC	99	9 +	Oryza sativa	abscisic acid responsive
TaMAPKKK16	ABRE	CGCACGTGTC	137	9 +	Hordeum vulgare	abscisic acid responsive
TaMAPKKK16	ABRE	ACGTG	263	5 +	Arabidopsis thaliana	abscisic acid responsive
TaMAPKKK16	ABRE	ACGTG	290	5 -	Arabidopsis thaliana	abscisic acid responsive
TaMAPKKK16	ABRE	CGTACGTGCA	351	10 +	Hordeum vulgare	abscisic acid responsive
TaMAPKKK16	ABRE	ACGTG	354	5 +	Arabidopsis thaliana	abscisic acid responsive
TaMAPKKK16	ABRE	CACGTG	648	6 +	Arabidopsis thaliana	abscisic acid responsive
TaMAPKKK16	ABRE	ACGTG	649	5 +	Arabidopsis thaliana	abscisic acid responsive
TaMAPKKK16	TCA-element	TCAGAAGAGG	1024	9 +	Brassica oleracea	salicylic acid responsive
TaMAPKKK16	circadian	CAAAGATATC	1710	9 +	Lycopersicon esculentum	circadian control

TaMAPKKK17	TGACG-motif	TGACG	397	5 +	Hordeum vulgare	the MeJA responsive
TaMAPKKK17	TGACG-motif	TGACG	1301	5 +	Hordeum vulgare	the MeJA responsive
TaMAPKKK17	GC-motif	CCCCCG	1333	6 +	Zea mays	anoxic specific inducibility
TaMAPKKK17	GC-motif	CCCCCG	1434	6 +	Zea mays	anoxic specific inducibility
TaMAPKKK17	CAT-box	GCCACT	88	6 +	Arabidopsis thaliana	meristem expression
TaMAPKKK17	MSA-like	(T/C)C(T/C)AACGG(T/C)(T/C)A	282	8.5 -	Catharanthus roseus	cell cycle regulation
TaMAPKKK17	ABRE	ACGTG	399	5 +	Arabidopsis thaliana	abscisic acid responsive
TaMAPKKK17	ABRE	ACGTG	1303	5 +	Arabidopsis thaliana	abscisic acid responsive
TaMAPKKK17	ABRE	GACACGTACGT	1354	10 +	Oryza sativa	abscisic acid responsive
TaMAPKKK17	ABRE	ACGTG	1356	5 -	Arabidopsis thaliana	abscisic acid responsive
TaMAPKKK17	AuxRR-core	GGTCCAT	1421	7 +	Nicotiana tabacum	auxin responsive
TaMAPKKK17	CGTCA-motif	CGTCA	397	5 -	Hordeum vulgare	the MeJA responsive
TaMAPKKK17	CGTCA-motif	CGTCA	1301	5 -	Hordeum vulgare	the MeJA responsive
TaMAPKKK17	TATC-box	TATCCCA	1066	7 +	Oryza sativa	gibberellin responsive
TaMAPKKK17	GCN4_motif	TGAGTCA	901	7 +	Oryza sativa	endosperm expression
TaMAPKKK18	CGTCA-motif	CGTCA	1329	5 -	Hordeum vulgare	the MeJA responsive
TaMAPKKK18	GC-motif	CCCCCG	1312	6 +	Zea mays	anoxic specific inducibility
TaMAPKKK18	GC-motif	CCCCCG	1484	6 +	Zea mays	anoxic specific inducibility

TaMAPKKK18	P-box	CCTTTTG	805	7 -	Oryza sativa	gibberellin responsive
TaMAPKKK18	TGACG-motif	TGACG	1329	5 +	Hordeum vulgare	the MeJA responsive
TaMAPKKK18	TGA-element	AACGAC	53	6 -	Brassica oleracea	auxin responsive
TaMAPKKK18	ABRE	ACGTG	259	5 +	Arabidopsis thaliana	abscisic acid responsive
TaMAPKKK18	ABRE	ACGTG	1095	5 +	Arabidopsis thaliana	abscisic acid responsive
TaMAPKKK18	ABRE	GCAACGTGTC	1269	9 -	Hordeum vulgare	abscisic acid responsive
TaMAPKKK18	ABRE	CACGTG	1271	6 -	Arabidopsis thaliana	abscisic acid responsive
TaMAPKKK18	ABRE	ACGTG	1272	5 +	Arabidopsis thaliana	abscisic acid responsive
TaMAPKKK2	ABRE	ACGTG	157	5 +	Arabidopsis thaliana	abscisic acid responsive
TaMAPKKK2	ABRE	ACGTG	350	5 +	Arabidopsis thaliana	abscisic acid responsive
TaMAPKKK2	CGTCA-motif	CGTCA	348	5 -	Hordeum vulgare	the MeJA responsive
TaMAPKKK2	CGTCA-motif	CGTCA	1288	5 +	Hordeum vulgare	the MeJA responsive
TaMAPKKK2	CGTCA-motif	CGTCA	1364	5 -	Hordeum vulgare	the MeJA responsive
TaMAPKKK2	LTR	CCGAAA	89	6 -	Hordeum vulgare	low temperature responsive
TaMAPKKK2	AuxRR-core	GGTCCAT	1745	7 -	Nicotiana tabacum	auxin responsive
TaMAPKKK2	TGACG-motif	TGACG	348	5 +	Hordeum vulgare	the MeJA responsive
TaMAPKKK2	TGACG-motif	TGACG	1288	5 -	Hordeum vulgare	the MeJA responsive
TaMAPKKK2	TGACG-motif	TGACG	1364	5 +	Hordeum vulgare	the MeJA responsive
TaMAPKKK2	ARE	AAACCA	455	6 -	Zea mays	anaerobic induction

TaMAPKKK2	ARE	AAACCA	524	6 -	Zea mays	anaerobic induction
TaMAPKKK2	ARE	AAACCA	650	6 -	Zea mays	anaerobic induction
TaMAPKKK2	GC-motif	CCCCCG	1611	6 +	Zea mays	anoxic specific inducibility
TaMAPKKK2	GC-motif	CCCCCG	1705	6 +	Zea mays	anoxic specific inducibility
TaMAPKKK20	CGTCA-motif	CGTCA	10	5 +	Hordeum vulgare	the MeJA responsive
TaMAPKKK20	CGTCA-motif	CGTCA	889	5 +	Hordeum vulgare	the MeJA responsive
TaMAPKKK20	CGTCA-motif	CGTCA	984	5 -	Hordeum vulgare	the MeJA responsive
TaMAPKKK20	CGTCA-motif	CGTCA	1130	5 +	Hordeum vulgare	the MeJA responsive
TaMAPKKK20	CGTCA-motif	CGTCA	1573	5 -	Hordeum vulgare	the MeJA responsive
TaMAPKKK20	GC-motif	CCCCCG	1556	6 +	Zea mays	anoxic specific inducibility
TaMAPKKK20	GC-motif	CCCCCG	1626	6 +	Zea mays	anoxic specific inducibility
TaMAPKKK20	GC-motif	CCCCCG	1728	6 +	Zea mays	anoxic specific inducibility
TaMAPKKK20	ABRE	ACGTG	887	5 -	Arabidopsis thaliana	abscisic acid responsive
TaMAPKKK20	P-box	CCTTTTG	739	7 +	Oryza sativa	gibberellin responsive
TaMAPKKK20	P-box	CCTTTTG	1247	7 -	Oryza sativa	gibberellin responsive
TaMAPKKK20	TGACG-motif	TGACG	10	5 -	Hordeum vulgare	the MeJA responsive
TaMAPKKK20	TGACG-motif	TGACG	889	5 -	Hordeum vulgare	the MeJA responsive
TaMAPKKK20	TGACG-motif	TGACG	984	5 +	Hordeum vulgare	the MeJA responsive
TaMAPKKK20	TGACG-motif	TGACG	1130	5 -	Hordeum vulgare	the MeJA responsive

TaMAPKKK20	TGACG-motif	TGACG	1573	5 +	Hordeum vulgare	the MeJA responsive
TaMAPKKK20	circadian	CAAAGATATC	892	9 +	Lycopersicon esculentum	circadian control
TaMAPKKK20	O2-site	GTTGACGTGA	886	9 -	Zea mays	zein metabolism regulation
TaMAPKKK20	O2-site	GATGATGTGG	1031	9 +	Zea mays	zein metabolism regulation
TaMAPKKK23	ARE	AAACCA	856	6 +	Zea mays	anaerobic induction
TaMAPKKK23	GCN4_motif	TGAGTCA	780	7 +	Oryza sativa	endosperm expression
TaMAPKKK23	TGA-element	AACGAC	1051	6 +	Brassica oleracea	auxin responsive
TaMAPKKK23	TGA-element	AACGAC	1096	6 +	Brassica oleracea	auxin responsive
TaMAPKKK23	GC-motif	CCCCCG	325	6 +	Zea mays	anoxic specific inducibility
TaMAPKKK23	ABRE	CACGTG	487	6 +	Arabidopsis thaliana	abscisic acid responsive
TaMAPKKK23	ABRE	ACGTG	488	5 +	Arabidopsis thaliana	abscisic acid responsive
TaMAPKKK23	ABRE	ACGTG	943	5 +	Arabidopsis thaliana	abscisic acid responsive
TaMAPKKK23	ABRE	CACGTG	970	6 +	Arabidopsis thaliana	abscisic acid responsive
TaMAPKKK23	ABRE	ACGTG	971	5 +	Arabidopsis thaliana	abscisic acid responsive
TaMAPKKK23	ABRE	ACGTG	1010	5 +	Arabidopsis thaliana	abscisic acid responsive
TaMAPKKK23	P-box	CCTTTTG	165	7 -	Oryza sativa	gibberellin responsive
TaMAPKKK24	ARE	AAACCA	364	6 -	Zea mays	anaerobic induction
TaMAPKKK24	ARE	AAACCA	612	6 -	Zea mays	anaerobic induction
TaMAPKKK24	ARE	AAACCA	940	6 +	Zea mays	anaerobic induction
TaMAPKKK24	GC-motif	CCCCCG	1750	6 +	Zea mays	anoxic specific inducibility

TaMAPKKK24	GC-motif	CCCCCG	1851	6 +	Zea mays	anoxic specific inducibility
TaMAPKKK24	GC-motif	CCCCCG	1917	6 +	Zea mays	anoxic specific inducibility
TaMAPKKK24	TC-rich repeats	GTTTTCTTAC	614	9 +	Nicotiana tabacum	defense and stress responsive
TaMAPKKK24	O2-site	GATGACATGG	215	9 +	Zea mays	zein metabolism regulation
TaMAPKKK24	TGACG-motif	TGACG	1300	5 +	Hordeum vulgare	the MeJA responsive
TaMAPKKK24	TGACG-motif	TGACG	1718	5 +	Hordeum vulgare	the MeJA responsive
TaMAPKKK24	ABRE	ACGTG	1524	5 +	Arabidopsis thaliana	abscisic acid responsive
TaMAPKKK24	ABRE	ACGTG	1720	5 +	Arabidopsis thaliana	abscisic acid responsive
TaMAPKKK24	ABRE	GACACGTACGT	1771	10 +	Oryza sativa	abscisic acid responsive
TaMAPKKK24	ABRE	ACGTG	1773	5 -	Arabidopsis thaliana	abscisic acid responsive
TaMAPKKK24	CGTCA-motif	CGTCA	1300	5 -	Hordeum vulgare	the MeJA responsive
TaMAPKKK24	CGTCA-motif	CGTCA	1718	5 -	Hordeum vulgare	the MeJA responsive
TaMAPKKK24	AuxRR-core	GGTCCAT	869	7 -	Nicotiana tabacum	auxin responsive
TaMAPKKK24	AuxRR-core	GGTCCAT	1838	7 +	Nicotiana tabacum	auxin responsive
TaMAPKKK24	WUN-motif	AAATTTCCT	509	9 +	Brassica oleracea	wound responsive
TaMAPKKK24	TATC-box	TATCCCA	1329	7 -	Oryza sativa	gibberellin responsive
TaMAPKKK24	GCN4_motif	TGAGTCA	1352	7 +	Oryza sativa	endosperm expression
TaMAPKKK25	CGTCA-motif	CGTCA	52	5 -	Hordeum vulgare	the MeJA responsive
TaMAPKKK25	CGTCA-motif	CGTCA	1413	5 -	Hordeum vulgare	the MeJA responsive

TaMAPKKK25	CGTCA-motif	CGTCA	1484	5 +	Hordeum vulgare	the MeJA responsive
TaMAPKKK25	TCA-element	CCATCTTTTT	1782	9 +	Nicotiana tabacum	salicylic acid responsive
TaMAPKKK25	P-box	CCTTTTG	1240	7 +	Oryza sativa	gibberellin responsive
TaMAPKKK25	GARE-motif	TCTGTTG	1598	7 +	Brassica oleracea	gibberellin responsive
TaMAPKKK25	ABRE	ACGTG	54	5 +	Arabidopsis thaliana	abscisic acid responsive
TaMAPKKK25	ABRE	ACGTG	572	5 -	Arabidopsis thaliana	abscisic acid responsive
TaMAPKKK25	ABRE	GACACGTGGC	596	9 -	Triticum aestivum	abscisic acid responsive
TaMAPKKK25	ABRE	CACGTG	598	6 +	Arabidopsis thaliana	abscisic acid responsive
TaMAPKKK25	ABRE	ACGTG	599	5 +	Arabidopsis thaliana	abscisic acid responsive
TaMAPKKK25	ABRE	ACGTG	837	5 -	Arabidopsis thaliana	abscisic acid responsive
TaMAPKKK25	ABRE	ACGTG	1040	5 +	Arabidopsis thaliana	abscisic acid responsive
TaMAPKKK25	ABRE	CACGTG	1360	6 -	Arabidopsis thaliana	abscisic acid responsive
TaMAPKKK25	ABRE	ACGTG	1361	5 +	Arabidopsis thaliana	abscisic acid responsive
TaMAPKKK25	ABRE	CGCACGTGTC	1504	9 -	Hordeum vulgare	abscisic acid responsive
TaMAPKKK25	ABRE	CACGTG	1506	6 -	Arabidopsis thaliana	abscisic acid responsive
TaMAPKKK25	ABRE	ACGTG	1507	5 +	Arabidopsis thaliana	abscisic acid responsive
TaMAPKKK25	AuxRR-core	GGTCCAT	1340	7 -	Nicotiana tabacum	auxin responsive
TaMAPKKK25	TGACG-motif	TGACG	52	5 +	Hordeum vulgare	the MeJA responsive
TaMAPKKK25	TGACG-motif	TGACG	1413	5 +	Hordeum vulgare	the MeJA responsive

TaMAPKKK25	TGACG-motif	TGACG	1484	5 -	Hordeum vulgare	the MeJA responsive
TaMAPKKK25	LTR	CCGAAA	1433	6 -	Hordeum vulgare	low temperature responsive
TaMAPKKK25	MSA-like	(T/C)C(T/C)AAC GG(T/C)(T/C)A	1568	8.5 -	Catharanthus roseus	cell cycle regulation
TaMAPKKK25	ARE	AAACCA	222	6 +	Zea mays	anaerobic induction
TaMAPKKK25	ARE	AAACCA	1130	6 -	Zea mays	anaerobic induction
TaMAPKKK25	TATC-box	TATCCCA	1552	7 -	Oryza sativa	gibberellin responsive
TaMAPKKK25	MBS	CAACTG	226	6 +	Arabidopsis thaliana	drought inducibility
TaMAPKKK25	RY-element	CATGCATG	171	8 +	Helianthus annuus	seed specific regulation
TaMAPKKK26	LTR	CCGAAA	503	6 +	Hordeum vulgare	low temperature responsive
TaMAPKKK26	LTR	CCGAAA	628	6 -	Hordeum vulgare	low temperature responsive
TaMAPKKK26	CGTCA-motif	CGTCA	535	5 +	Hordeum vulgare	the MeJA responsive
TaMAPKKK26	CGTCA-motif	CGTCA	841	5 +	Hordeum vulgare	the MeJA responsive
TaMAPKKK26	TC-rich repeats	GTTTTCTTAC	1352	9 -	Nicotiana tabacum	defense and stress responsive
TaMAPKKK26	P-box	CCTTTTG	791	7 -	Oryza sativa	gibberellin responsive
TaMAPKKK26	MBS	CAACTG	217	6 +	Arabidopsis thaliana	drought inducibility
TaMAPKKK26	MBS	CAACTG	468	6 -	Arabidopsis thaliana	drought inducibility
TaMAPKKK26	MBS	CAACTG	597	6 -	Arabidopsis thaliana	drought inducibility
TaMAPKKK26	ARE	AAACCA	99	6 +	Zea mays	anaerobic induction

TaMAPKKK26	ARE	AAACCA	270	6 -	Zea mays	anaerobic induction
TaMAPKKK26	TGACG-motif	TGACG	535	5 -	Hordeum vulgare	the MeJA responsive
TaMAPKKK26	TGACG-motif	TGACG	841	5 -	Hordeum vulgare	the MeJA responsive
TaMAPKKK26	ABRE	ACGTG	282	5 -	Arabidopsis thaliana	abscisic acid responsive
TaMAPKKK26	ABRE	ACGTG	953	5 -	Arabidopsis thaliana	abscisic acid responsive
TaMAPKKK26	ABRE	ACGTG	982	5 -	Arabidopsis thaliana	abscisic acid responsive
TaMAPKKK26	ABRE	ACGTG	1780	5 -	Arabidopsis thaliana	abscisic acid responsive
TaMAPKKK26	CAT-box	GCCACT	1547	6 +	Arabidopsis thaliana	meristem expression
TaMAPKKK27	TGACG-motif	TGACG	818	5 -	Hordeum vulgare	the MeJA responsive
TaMAPKKK27	TGACG-motif	TGACG	1783	5 -	Hordeum vulgare	the MeJA responsive
TaMAPKKK27	TCA-element	CCATCTTTTT	82	9 -	Nicotiana tabacum	salicylic acid responsive
TaMAPKKK27	ARE	AAACCA	67	6 +	Zea mays	anaerobic induction
TaMAPKKK27	ARE	AAACCA	581	6 -	Zea mays	anaerobic induction
TaMAPKKK27	ARE	AAACCA	602	6 -	Zea mays	anaerobic induction
TaMAPKKK27	ARE	AAACCA	611	6 -	Zea mays	anaerobic induction
TaMAPKKK27	circadian	CAAAGATATC	1390	9 +	Lycopersicon esculentum	circadian control
TaMAPKKK27	CAT-box	GCCACT	1555	6 +	Arabidopsis thaliana	meristem expression
TaMAPKKK27	CGTCA-motif	CGTCA	818	5 +	Hordeum vulgare	the MeJA responsive
TaMAPKKK27	CGTCA-motif	CGTCA	1783	5 +	Hordeum vulgare	the MeJA responsive

TaMAPKKK27	ABRE	ACGTG	233	5 -	Arabidopsis thaliana	abscisic acid responsive
TaMAPKKK27	ABRE	ACGTG	1778	5 -	Arabidopsis thaliana	abscisic acid responsive
TaMAPKKK27	MBS	CAACTG	440	6 -	Arabidopsis thaliana	drought inducibility
TaMAPKKK27	MBS	CAACTG	569	6 -	Arabidopsis thaliana	drought inducibility
TaMAPKKK28	TC-rich repeats	GTTTTCTTAC	1361	9 -	Nicotiana tabacum	defense and stress responsive
TaMAPKKK28	MBS	CAACTG	223	6 -	Arabidopsis thaliana	drought inducibility
TaMAPKKK28	CGTCA-motif	CGTCA	590	5 +	Hordeum vulgare	the MeJA responsive
TaMAPKKK28	CGTCA-motif	CGTCA	853	5 -	Hordeum vulgare	the MeJA responsive
TaMAPKKK28	ABRE	ACGTG	44	5 -	Arabidopsis thaliana	abscisic acid responsive
TaMAPKKK28	ABRE	CACGTG	202	6 +	Arabidopsis thaliana	abscisic acid responsive
TaMAPKKK28	ABRE	ACGTG	203	5 +	Arabidopsis thaliana	abscisic acid responsive
TaMAPKKK28	ABRE	GCAACGTGTC	584	9 +	Hordeum vulgare	abscisic acid responsive
TaMAPKKK28	ABRE	ACGTG	873	5 -	Arabidopsis thaliana	abscisic acid responsive
TaMAPKKK28	ABRE	ACGTG	1778	5 -	Arabidopsis thaliana	abscisic acid responsive
TaMAPKKK28	P-box	CCTTTTG	540	7 -	Oryza sativa	gibberellin responsive
TaMAPKKK28	LTR	CCGAAA	258	6 +	Hordeum vulgare	low temperature responsive
TaMAPKKK28	LTR	CCGAAA	369	6 -	Hordeum vulgare	low temperature responsive
TaMAPKKK28	LTR	CCGAAA	656	6 -	Hordeum vulgare	low temperature responsive
TaMAPKKK28	TGACG-motif	TGACG	590	5 -	Hordeum vulgare	the MeJA responsive

TaMAPKKK28	TGACG-motif	TGACG	853	5 +	Hordeum vulgare	the MeJA responsive
TaMAPKKK28	ARE	AAACCA	381	6 -	Zea mays	anaerobic induction
TaMAPKKK29	CGTCA-motif	CGTCA	1141	5 -	Hordeum vulgare	the MeJA responsive
TaMAPKKK29	CGTCA-motif	CGTCA	1704	5 -	Hordeum vulgare	the MeJA responsive
TaMAPKKK29	AuxRR-core	GGTCCAT	855	7 -	Nicotiana tabacum	auxin responsive
TaMAPKKK29	AuxRR-core	GGTCCAT	1828	7 +	Nicotiana tabacum	auxin responsive
TaMAPKKK29	TGACG-motif	TGACG	1141	5 +	Hordeum vulgare	the MeJA responsive
TaMAPKKK29	TGACG-motif	TGACG	1704	5 +	Hordeum vulgare	the MeJA responsive
TaMAPKKK29	TC-rich repeats	GTTTTCTTAC	286	9 +	Nicotiana tabacum	defense and stress responsive
TaMAPKKK29	LTR	CCGAAA	1781	6 +	Hordeum vulgare	low temperature responsive
TaMAPKKK29	GCN4_motif	TGAGTCA	1347	7 +	Oryza sativa	endosperm expression
TaMAPKKK29	ARE	AAACCA	34	6 -	Zea mays	anaerobic induction
TaMAPKKK29	ARE	AAACCA	284	6 -	Zea mays	anaerobic induction
TaMAPKKK29	ARE	AAACCA	926	6 +	Zea mays	anaerobic induction
TaMAPKKK29	CAT-box	GCCACT	457	6 -	Arabidopsis thaliana	meristem expression
TaMAPKKK29	TATC-box	TATCCCA	1324	7 -	Oryza sativa	gibberellin responsive
TaMAPKKK29	WUN-motif	AAATTTCCT	180	9 +	Brassica oleracea	wound responsive
TaMAPKKK29	ABRE	ACGTG	1517	5 +	Arabidopsis thaliana	abscisic acid responsive
TaMAPKKK29	ABRE	ACGTG	1706	5 +	Arabidopsis thaliana	abscisic acid responsive

TaMAPKKK29	ABRE	ACGTG	1766	5 -	Arabidopsis thaliana	abscisic acid responsive
TaMAPKKK29	GC-motif	CCCCCG	1743	6 +	Zea mays	anoxic specific inducibility
TaMAPKKK29	GC-motif	CCCCCG	1841	6 +	Zea mays	anoxic specific inducibility
TaMAPKKK29	GC-motif	CCCCCG	1918	6 +	Zea mays	anoxic specific inducibility
TaMAPKKK3	CAT-box	GCCACT	554	6 +	Arabidopsis thaliana	meristem expression
TaMAPKKK3	GC-motif	CCCCCG	673	6 +	Zea mays	anoxic specific inducibility
TaMAPKKK3	GC-motif	CCCCCG	1931	6 +	Zea mays	anoxic specific inducibility
TaMAPKKK3	GC-motif	CCCCCG	1983	6 +	Zea mays	anoxic specific inducibility
TaMAPKKK3	ARE	AAACCA	1327	6 -	Zea mays	anaerobic induction
TaMAPKKK3	ARE	AAACCA	1784	6 +	Zea mays	anaerobic induction
TaMAPKKK3	O2-site	GTTGACGTGA	632	9 +	Zea mays	zein metabolism regulation
TaMAPKKK3	TGACG-motif	TGACG	634	5 +	Hordeum vulgare	the MeJA responsive
TaMAPKKK3	TGACG-motif	TGACG	895	5 +	Hordeum vulgare	the MeJA responsive
TaMAPKKK3	GARE-motif	TCTGTTG	170	7 +	Brassica oleracea	gibberellin responsive
TaMAPKKK3	CGTCA-motif	CGTCA	634	5 -	Hordeum vulgare	the MeJA responsive
TaMAPKKK3	CGTCA-motif	CGTCA	895	5 -	Hordeum vulgare	the MeJA responsive
TaMAPKKK3	MBS	CAACTG	1191	6 -	Arabidopsis thaliana	drought inducibility
TaMAPKKK3	ABRE	ACGTG	596	5 +	Arabidopsis thaliana	abscisic acid responsive
TaMAPKKK3	ABRE	ACGTG	636	5 +	Arabidopsis thaliana	abscisic acid responsive

TaMAPKKK3	ABRE	TACGTGTC	750	8 -	Oryza sativa	abscisic acid responsive
TaMAPKKK3	ABRE	ACGTG	752	5 -	Arabidopsis thaliana	abscisic acid responsive
TaMAPKKK3	ABRE	ACGTG	770	5 -	Arabidopsis thaliana	abscisic acid responsive
TaMAPKKK3	ABRE	ACGTG	935	5 +	Arabidopsis thaliana	abscisic acid responsive
TaMAPKKK3	ABRE	CACGTG	950	6 +	Arabidopsis thaliana	abscisic acid responsive
TaMAPKKK3	ABRE	ACGTG	951	5 +	Arabidopsis thaliana	abscisic acid responsive
TaMAPKKK3	ABRE	ACGTG	983	5 -	Arabidopsis thaliana	abscisic acid responsive
TaMAPKKK3	ABRE	GCCGCGTGGC	1264	9 +	Oryza sativa	abscisic acid responsive
TaMAPKKK3	ABRE	CGTACGTGCA	1313	9 +	Hordeum vulgare	abscisic acid responsive
TaMAPKKK30	CGTCA-motif	CGTCA	223	5 -	Hordeum vulgare	the MeJA responsive
TaMAPKKK30	CGTCA-motif	CGTCA	426	5 +	Hordeum vulgare	the MeJA responsive
TaMAPKKK30	CGTCA-motif	CGTCA	1614	5 -	Hordeum vulgare	the MeJA responsive
TaMAPKKK30	CGTCA-motif	CGTCA	1759	5 +	Hordeum vulgare	the MeJA responsive
TaMAPKKK30	CGTCA-motif	CGTCA	1811	5 +	Hordeum vulgare	the MeJA responsive
TaMAPKKK30	TGACG-motif	TGACG	223	5 +	Hordeum vulgare	the MeJA responsive
TaMAPKKK30	TGACG-motif	TGACG	426	5 -	Hordeum vulgare	the MeJA responsive
TaMAPKKK30	TGACG-motif	TGACG	1614	5 +	Hordeum vulgare	the MeJA responsive
TaMAPKKK30	TGACG-motif	TGACG	1759	5 -	Hordeum vulgare	the MeJA responsive
TaMAPKKK30	TGACG-motif	TGACG	1811	5 -	Hordeum vulgare	the MeJA responsive

TaMAPKKK30	TC-rich repeats	ATTCTCTAAC	545	9 -	Nicotiana tabacum	defense and stress responsive
TaMAPKKK30	O2-site	GATGATGTGG	1436	9 -	Zea mays	zein metabolism regulation
TaMAPKKK30	ARE	AAACCA	323	6 +	Zea mays	anaerobic induction
TaMAPKKK30	ARE	AAACCA	412	6 +	Zea mays	anaerobic induction
TaMAPKKK30	ARE	AAACCA	719	6 +	Zea mays	anaerobic induction
TaMAPKKK30	HD-Zip 1	CAAT(A/T)ATT G	167	8.5 +	Arabidopsis thaliana	differentiation of the palisade mesophyll cells
TaMAPKKK30	GARE-motif	TCTGTTG	1291	7 -	Brassica oleracea	gibberellin responsive
TaMAPKKK30	MBS	CAACTG	507	6 -	Arabidopsis thaliana	drought inducibility
TaMAPKKK30	ABRE	CACGTG	998	6 +	Arabidopsis thaliana	abscisic acid responsive
TaMAPKKK30	ABRE	ACGTG	999	5 +	Arabidopsis thaliana	abscisic acid responsive
TaMAPKKK31	TGACG-motif	TGACG	883	5 -	Hordeum vulgare	the MeJA responsive
TaMAPKKK31	TGACG-motif	TGACG	1856	5 -	Hordeum vulgare	the MeJA responsive
TaMAPKKK31	CGTCA-motif	CGTCA	883	5 +	Hordeum vulgare	the MeJA responsive
TaMAPKKK31	CGTCA-motif	CGTCA	1856	5 +	Hordeum vulgare	the MeJA responsive
TaMAPKKK31	GC-motif	CCCCCG	257	6 +	Zea mays	anoxic specific inducibility
TaMAPKKK31	GC-motif	CCCCCG	1008	6 +	Zea mays	anoxic specific inducibility
TaMAPKKK31	GC-motif	CCCCCG	1785	6 +	Zea mays	anoxic specific inducibility
TaMAPKKK31	TGA-element	AACGAC	861	6 -	Brassica oleracea	auxin responsive
TaMAPKKK31	ABRE	AACCCGG	706	7 +	Arabidopsis thaliana	abscisic acid responsive

TaMAPKKK31	ARE	AAACCA	1019	6 -	Zea mays	anaerobic induction
TaMAPKKK31	CAT-box	GCCACT	731	6 -	Arabidopsis thaliana	meristem expression
TaMAPKKK31	MBS	CAACTG	62	6 -	Arabidopsis thaliana	drought inducibility
TaMAPKKK32	ARE	AAACCA	1317	6 +	Zea mays	anaerobic induction
TaMAPKKK32	ARE	AAACCA	1461	6 -	Zea mays	anaerobic induction
TaMAPKKK32	TGACG-motif	TGACG	731	5 +	Hordeum vulgare	the MeJA responsive
TaMAPKKK32	TCA-element	TCAGAAGAGG	1350	9 -	Brassica oleracea	salicylic acid responsive
TaMAPKKK32	LTR	CCGAAA	1284	6 -	Hordeum vulgare	low temperature responsive
TaMAPKKK32	O2-site	GATGA(C/T)(A/G)TG(A/G)	787	8 -	Zea mays	zein metabolism regulation
TaMAPKKK32	O2-site	GATGATGTGG	1729	9 -	Zea mays	zein metabolism regulation
TaMAPKKK32	ABRE	ACGTG	33	5 +	Arabidopsis thaliana	abscisic acid responsive
TaMAPKKK32	ABRE	GCCGCGTGGC	65	9 -	Oryza sativa	abscisic acid responsive
TaMAPKKK32	ABRE	ACGTG	1324	5 +	Arabidopsis thaliana	abscisic acid responsive
TaMAPKKK32	GC-motif	CCCCCG	963	6 -	Zea mays	anoxic specific inducibility
TaMAPKKK32	GC-motif	CCCCCG	1861	6 +	Zea mays	anoxic specific inducibility
TaMAPKKK32	CAT-box	GCCACT	420	6 +	Arabidopsis thaliana	meristem expression
TaMAPKKK32	CGTCA-motif	CGTCA	731	5 -	Hordeum vulgare	the MeJA responsive
TaMAPKKK32	TGA-element	AACGAC	1279	6 -	Brassica oleracea	auxin responsive
TaMAPKKK33	GC-motif	CCCCCG	1797	6 +	Zea mays	anoxic specific inducibility
TaMAPKKK33	TGA-element	AACGAC	934	6 +	Brassica oleracea	auxin responsive

TaMAPKKK33	ABRE	GCCGCGTGGC	36	9 -	Oryza sativa	abscisic acid responsive
TaMAPKKK33	ABRE	GACACGTGGC	205	9 +	Triticum aestivum	abscisic acid responsive
TaMAPKKK33	ABRE	ACGTG	219	5 -	Arabidopsis thaliana	abscisic acid responsive
TaMAPKKK33	ABRE	ACGTG	858	5 -	Arabidopsis thaliana	abscisic acid responsive
TaMAPKKK33	P-box	CCTTTTG	195	7 +	Oryza sativa	gibberellin responsive
TaMAPKKK33	TCA-element	CCATCTTTTT	1926	9 +	Nicotiana tabacum	salicylic acid responsive
TaMAPKKK33	TCA-element	CCATCTTTTT	1950	9 +	Nicotiana tabacum	salicylic acid responsive
TaMAPKKK33	circadian	CAAAGATATC	1230	9 -	Lycopersicon esculentum	circadian control
TaMAPKKK33	MBS	CAACTG	131	6 +	Arabidopsis thaliana	drought inducibility
TaMAPKKK33	MBS	CAACTG	1070	6 -	Arabidopsis thaliana	drought inducibility
TaMAPKKK33	GARE-motif	TCTGTTG	1099	7 +	Brassica oleracea	gibberellin responsive
TaMAPKKK33	GARE-motif	TCTGTTG	1265	7 +	Brassica oleracea	gibberellin responsive
TaMAPKKK33	CAT-box	GCCACT	257	6 -	Arabidopsis thaliana	meristem expression
TaMAPKKK33	CGTCA-motif	CGTCA	1868	5 +	Hordeum vulgare	the MeJA responsive
TaMAPKKK33	TGACG-motif	TGACG	1868	5 -	Hordeum vulgare	the MeJA responsive
TaMAPKKK34	TGACG-motif	TGACG	1101	5 +	Hordeum vulgare	the MeJA responsive
TaMAPKKK34	O2-site	GATGACATGG	388	9 -	Zea mays	zein metabolism regulation
TaMAPKKK34	TC-rich repeats	GTTTTCTTAC	220	9 +	Nicotiana tabacum	defense and stress responsive
TaMAPKKK34	RY-element	CATGCATG	835	8 +	Helianthus annuus	seed specific regulation

TaMAPKKK34	MBS	CAACTG	408	6 +	Arabidopsis thaliana	drought inducibility
TaMAPKKK34	MBS	CAACTG	1364	6 -	Arabidopsis thaliana	drought inducibility
TaMAPKKK34	LTR	CCGAAA	606	6 -	Hordeum vulgare	low temperature responsive
TaMAPKKK34	CGTCA-motif	CGTCA	1101	5 -	Hordeum vulgare	the MeJA responsive
TaMAPKKK34	TGA-element	AACGAC	1245	6 +	Brassica oleracea	auxin responsive
TaMAPKKK34	CAT-box	GCCACT	167	6 +	Arabidopsis thaliana	meristem expression
TaMAPKKK34	ABRE	ACGTG	163	5 +	Arabidopsis thaliana	abscisic acid responsive
TaMAPKKK34	ABRE	CACGTG	1182	6 -	Arabidopsis thaliana	abscisic acid responsive
TaMAPKKK34	ABRE	ACGTG	1183	5 +	Arabidopsis thaliana	abscisic acid responsive
TaMAPKKK34	ABRE	ACGTG	1576	5 +	Arabidopsis thaliana	abscisic acid responsive
TaMAPKKK34	ABRE	GCCGCGTGGC	1688	9 -	Oryza sativa	abscisic acid responsive
TaMAPKKK34	ABRE	ACGTG	1765	5 +	Arabidopsis thaliana	abscisic acid responsive
TaMAPKKK34	ABRE	CGCACGTGTC	1836	9 +	Hordeum vulgare	abscisic acid responsive
TaMAPKKK34	ABRE	CACGTG	1838	6 -	Arabidopsis thaliana	abscisic acid responsive
TaMAPKKK34	ABRE	ACGTG	1839	5 +	Arabidopsis thaliana	abscisic acid responsive
TaMAPKKK34	GARE-motif	TCTGTTG	313	7 -	Brassica oleracea	gibberellin responsive
TaMAPKKK34	ARE	AAACCA	972	6 +	Zea mays	anaerobic induction
TaMAPKKK34	P-box	CCTTTTG	501	7 -	Oryza sativa	gibberellin responsive
TaMAPKKK36	GC-motif	CCCCCG	980	6 -	Zea mays	anoxic specific inducibility

TaMAPKKK36	GC-motif	CCCCCG	1233	6 +	Zea mays	anoxic specific inducibility
TaMAPKKK36	O2-site	GATGACATGG	186	9 -	Zea mays	zein metabolism regulation
TaMAPKKK36	O2-site	GATGATGTGG	1721	9 -	Zea mays	zein metabolism regulation
TaMAPKKK36	CGTCA-motif	CGTCA	581	5 -	Hordeum vulgare	the MeJA responsive
TaMAPKKK36	CGTCA-motif	CGTCA	740	5 +	Hordeum vulgare	the MeJA responsive
TaMAPKKK36	CGTCA-motif	CGTCA	760	5 -	Hordeum vulgare	the MeJA responsive
TaMAPKKK36	LTR	CCGAAA	1299	6 -	Hordeum vulgare	low temperature responsive
TaMAPKKK36	TGA-element	AACGAC	215	6 +	Brassica oleracea	auxin responsive
TaMAPKKK36	TGA-element	AACGAC	1294	6 -	Brassica oleracea	auxin responsive
TaMAPKKK36	TGA-element	AACGAC	1567	6 +	Brassica oleracea	auxin responsive
TaMAPKKK36	ABRE	GCCGCGTGGC	267	9 +	Oryza sativa	abscisic acid responsive
TaMAPKKK36	ABRE	GCAACGTGTC	386	10 -	Hordeum vulgare	abscisic acid responsive
TaMAPKKK36	ABRE	ACGTG	388	5 -	Arabidopsis thaliana	abscisic acid responsive
TaMAPKKK36	CAT-box	GCCACT	527	6 -	Arabidopsis thaliana	meristem expression
TaMAPKKK36	ARE	AAACCA	1332	6 +	Zea mays	anaerobic induction
TaMAPKKK36	TGACG-motif	TGACG	581	5 +	Hordeum vulgare	the MeJA responsive
TaMAPKKK36	TGACG-motif	TGACG	740	5 -	Hordeum vulgare	the MeJA responsive
TaMAPKKK36	TGACG-motif	TGACG	760	5 +	Hordeum vulgare	the MeJA responsive
TaMAPKKK37	ABRE	AACCCGG	594	7 +	Arabidopsis thaliana	abscisic acid responsive
TaMAPKKK37	ABRE	ACGTG	824	5 -	Arabidopsis thaliana	abscisic acid responsive

TaMAPKKK37	ABRE	AACCCGG	1397	7 -	Arabidopsis thaliana	abscisic acid responsive
TaMAPKKK37	ABRE	GCCGCGTGGC	1697	9 -	Oryza sativa	abscisic acid responsive
TaMAPKKK37	ABRE	CGCACGTGTC	1840	9 +	Hordeum vulgare	abscisic acid responsive
TaMAPKKK37	ABRE	CACGTG	1842	6 -	Arabidopsis thaliana	abscisic acid responsive
TaMAPKKK37	ABRE	ACGTG	1843	5 +	Arabidopsis thaliana	abscisic acid responsive
TaMAPKKK37	TGA-element	AACGAC	98	6 -	Brassica oleracea	auxin responsive
TaMAPKKK37	TGA-element	AACGAC	215	6 -	Brassica oleracea	auxin responsive
TaMAPKKK37	CGTCA-motif	CGTCA	934	5 -	Hordeum vulgare	the MeJA responsive
TaMAPKKK37	CGTCA-motif	CGTCA	1300	5 +	Hordeum vulgare	the MeJA responsive
TaMAPKKK37	CGTCA-motif	CGTCA	1619	5 +	Hordeum vulgare	the MeJA responsive
TaMAPKKK37	O2-site	GATGATGTGG	632	9 +	Zea mays	zein metabolism regulation
TaMAPKKK37	TGACG-motif	TGACG	934	5 +	Hordeum vulgare	the MeJA responsive
TaMAPKKK37	TGACG-motif	TGACG	1300	5 -	Hordeum vulgare	the MeJA responsive
TaMAPKKK37	TGACG-motif	TGACG	1619	5 -	Hordeum vulgare	the MeJA responsive
TaMAPKKK38	GC-motif	CCCCCG	1483	6 -	Zea mays	anoxic specific inducibility
TaMAPKKK38	ABRE	AACCCGG	473	7 -	Arabidopsis thaliana	abscisic acid responsive
TaMAPKKK38	ABRE	ACGTG	623	5 -	Arabidopsis thaliana	abscisic acid responsive
TaMAPKKK38	ABRE	ACGTG	761	5 +	Arabidopsis thaliana	abscisic acid responsive
TaMAPKKK38	ABRE	ACGTG	842	5 +	Arabidopsis thaliana	abscisic acid responsive
TaMAPKKK38	ABRE	ACGTG	1630	5 +	Arabidopsis thaliana	abscisic acid responsive

TaMAPKKK38	ABRE	ACGTG	1695	5 -	Arabidopsis thaliana	abscisic acid responsive
TaMAPKKK38	CGTCA-motif	CGTCA	817	5 -	Hordeum vulgare	the MeJA responsive
TaMAPKKK38	CGTCA-motif	CGTCA	826	5 +	Hordeum vulgare	the MeJA responsive
TaMAPKKK38	CGTCA-motif	CGTCA	840	5 -	Hordeum vulgare	the MeJA responsive
TaMAPKKK38	TC-rich repeats	ATTCTCTAAC	540	9 -	Nicotiana tabacum	defense and stress responsive
TaMAPKKK38	ARE	AAACCA	379	6 -	Zea mays	anaerobic induction
TaMAPKKK38	ARE	AAACCA	448	6 -	Zea mays	anaerobic induction
TaMAPKKK38	ARE	AAACCA	1244	6 -	Zea mays	anaerobic induction
TaMAPKKK38	ARE	AAACCA	1260	6 +	Zea mays	anaerobic induction
TaMAPKKK38	TCA-element	CCATCTTTTT	711	9 +	Nicotiana tabacum	salicylic acid responsive
TaMAPKKK38	TGACG-motif	TGACG	817	5 +	Hordeum vulgare	the MeJA responsive
TaMAPKKK38	TGACG-motif	TGACG	826	5 -	Hordeum vulgare	the MeJA responsive
TaMAPKKK38	TGACG-motif	TGACG	840	5 +	Hordeum vulgare	the MeJA responsive
TaMAPKKK38	LTR	CCGAAA	438	6 +	Hordeum vulgare	low temperature responsive
TaMAPKKK38	GCN4_motif	TGAGTCA	1877	7 -	Oryza sativa	endosperm expression
TaMAPKKK38	O2-site	GATGA(C/T)(A/G)TG(A/G)	838	8 +	Zea mays	zein metabolism regulation
TaMAPKKK39	TCA-element	TCAGAAGAGG	771	9 +	Brassica oleracea	salicylic acid responsive
TaMAPKKK39	GC-motif	CCCCCG	1797	6 +	Zea mays	anoxic specific inducibility
TaMAPKKK39	ABRE	ACGTG	815	5 -	Arabidopsis thaliana	abscisic acid responsive

TaMAPKKK39	ABRE	ACGTG	877	5 +	Arabidopsis thaliana	abscisic acid responsive
TaMAPKKK39	ABRE	CACGTG	1026	6 -	Arabidopsis thaliana	abscisic acid responsive
TaMAPKKK39	ABRE	ACGTG	1027	5 +	Arabidopsis thaliana	abscisic acid responsive
TaMAPKKK39	ABRE	GCAACGTGTC	1058	9 +	Hordeum vulgare	abscisic acid responsive
TaMAPKKK39	RY-element	CATGCATG	1212	8 -	Helianthus annuus	seed specific regulation
TaMAPKKK39	LTR	CCGAAA	715	6 -	Hordeum vulgare	low temperature responsive
TaMAPKKK39	AuxRR-core	GGTCCAT	24	7 +	Nicotiana tabacum	auxin responsive
TaMAPKKK39	CGTCA-motif	CGTCA	690	5 -	Hordeum vulgare	the MeJA responsive
TaMAPKKK39	CGTCA-motif	CGTCA	1230	5 +	Hordeum vulgare	the MeJA responsive
TaMAPKKK39	CGTCA-motif	CGTCA	1868	5 +	Hordeum vulgare	the MeJA responsive
TaMAPKKK39	TGACG-motif	TGACG	690	5 +	Hordeum vulgare	the MeJA responsive
TaMAPKKK39	TGACG-motif	TGACG	1230	5 -	Hordeum vulgare	the MeJA responsive
TaMAPKKK39	TGACG-motif	TGACG	1868	5 -	Hordeum vulgare	the MeJA responsive
TaMAPKKK39	MSA-like	(T/C)C(T/C)AAC GG(T/C)(T/C)A	934	8.5 -	Catharanthus roseus	cell cycle regulation
TaMAPKKK39	MSA-like	TCAAACGGT	936	9 -	Catharanthus roseus	cell cycle regulation
TaMAPKKK39	CAT-box	GCCACT	626	6 +	Arabidopsis thaliana	meristem expression
TaMAPKKK39	ARE	AAACCA	335	6 -	Zea mays	anaerobic induction
TaMAPKKK39	ARE	AAACCA	827	6 -	Zea mays	anaerobic induction

TaMAPKKK39	ARE	AAACCA	1079	6 -	Zea mays	anaerobic induction
TaMAPKKK4	TGA-element	AACGAC	941	6 -	Brassica oleracea	auxin responsive
TaMAPKKK4	MBS	CAACTG	538	6 -	Arabidopsis thaliana	drought inducibility
TaMAPKKK4	MBS	CAACTG	1687	6 -	Arabidopsis thaliana	drought inducibility
TaMAPKKK4	MBS	CAACTG	1904	6 +	Arabidopsis thaliana	drought inducibility
TaMAPKKK4	ARE	AAACCA	595	6 -	Zea mays	anaerobic induction
TaMAPKKK4	ARE	AAACCA	1398	6 +	Zea mays	anaerobic induction
TaMAPKKK4	LTR	CCGAAA	73	6 -	Hordeum vulgare	low temperature responsive
TaMAPKKK4	TGACG-motif	TGACG	1164	5 +	Hordeum vulgare	the MeJA responsive
TaMAPKKK4	CGTCA-motif	CGTCA	1164	5 -	Hordeum vulgare	the MeJA responsive
TaMAPKKK40	TC-rich repeats	ATTCTCTAAC	1557	9 -	Nicotiana tabacum	defense and stress responsive
TaMAPKKK40	TGACG-motif	TGACG	74	5 +	Hordeum vulgare	the MeJA responsive
TaMAPKKK40	TGACG-motif	TGACG	1245	5 +	Hordeum vulgare	the MeJA responsive
TaMAPKKK40	TGACG-motif	TGACG	1437	5 -	Hordeum vulgare	the MeJA responsive
TaMAPKKK40	TGACG-motif	TGACG	1742	5 -	Hordeum vulgare	the MeJA responsive
TaMAPKKK40	TGACG-motif	TGACG	1794	5 -	Hordeum vulgare	the MeJA responsive
TaMAPKKK40	O2-site	GATGATGTGG	29	9 -	Zea mays	zein metabolism regulation
TaMAPKKK40	MBS	CAACTG	70	6 -	Arabidopsis thaliana	drought inducibility
TaMAPKKK40	MBS	CAACTG	531	6 +	Arabidopsis thaliana	drought inducibility

TaMAPKKK40	MBS	CAACTG	609	6 -	Arabidopsis thaliana	drought inducibility
TaMAPKKK40	MBS	CAACTG	744	6 +	Arabidopsis thaliana	drought inducibility
TaMAPKKK40	MBS	CAACTG	1518	6 -	Arabidopsis thaliana	drought inducibility
TaMAPKKK40	ARE	AAACCA	1261	6 -	Zea mays	anaerobic induction
TaMAPKKK40	ARE	AAACCA	1328	6 +	Zea mays	anaerobic induction
TaMAPKKK40	ARE	AAACCA	1423	6 +	Zea mays	anaerobic induction
TaMAPKKK40	GC-motif	CCCCCG	1788	6 -	Zea mays	anoxic specific inducibility
TaMAPKKK40	HD-Zip 1	CAAT(A/T)ATT G	1160	8.5 -	Arabidopsis thaliana	differentiation of the palisade mesophyll cells
TaMAPKKK40	CGTCA-motif	CGTCA	74	5 -	Hordeum vulgare	the MeJA responsive
TaMAPKKK40	CGTCA-motif	CGTCA	1245	5 -	Hordeum vulgare	the MeJA responsive
TaMAPKKK40	CGTCA-motif	CGTCA	1437	5 +	Hordeum vulgare	the MeJA responsive
TaMAPKKK40	CGTCA-motif	CGTCA	1742	5 +	Hordeum vulgare	the MeJA responsive
TaMAPKKK40	CGTCA-motif	CGTCA	1794	5 +	Hordeum vulgare	the MeJA responsive
TaMAPKKK40	TGA-element	AACGAC	971	6 +	Brassica oleracea	auxin responsive
TaMAPKKK40	ABRE	ACGTG	1135	5 +	Arabidopsis thaliana	abscisic acid responsive
TaMAPKKK41	ARE	AAACCA	84	6 +	Zea mays	anaerobic induction
TaMAPKKK41	CGTCA-motif	CGTCA	733	5 -	Hordeum vulgare	the MeJA responsive
TaMAPKKK41	CGTCA-motif	CGTCA	1877	5 +	Hordeum vulgare	the MeJA responsive
TaMAPKKK41	TGACG-motif	TGACG	733	5 +	Hordeum vulgare	the MeJA responsive

TaMAPKKK41	TGACG-motif	TGACG	1877	5 -	Hordeum vulgare	the MeJA responsive
TaMAPKKK41	ABRE	CGTACGTGCA	1754	9 +	Hordeum vulgare	abscisic acid responsive
TaMAPKKK41	RY-element	CATGCATG	1124	8 -	Helianthus annuus	seed specific regulation
TaMAPKKK41	GC-motif	CCCCCG	111	6 +	Zea mays	anoxic specific inducibility
TaMAPKKK41	TGA-element	AACGAC	1323	6 -	Brassica oleracea	auxin responsive
TaMAPKKK41	TCA-element	CCATCTTTTT	959	9 -	Nicotiana tabacum	salicylic acid responsive
TaMAPKKK4-1	ARE	AAACCA	120	6 +	Zea mays	anaerobic induction
TaMAPKKK4-1	ARE	AAACCA	619	6 -	Zea mays	anaerobic induction
TaMAPKKK4-1	ARE	AAACCA	1422	6 +	Zea mays	anaerobic induction
TaMAPKKK4-1	LTR	CCGAAA	392	6 -	Hordeum vulgare	low temperature responsive
TaMAPKKK4-1	LTR	CCGAAA	734	6 +	Hordeum vulgare	low temperature responsive
TaMAPKKK4-1	CGTCA-motif	CGTCA	243	5 -	Hordeum vulgare	the MeJA responsive
TaMAPKKK4-1	CGTCA-motif	CGTCA	296	5 -	Hordeum vulgare	the MeJA responsive
TaMAPKKK4-1	CGTCA-motif	CGTCA	604	5 +	Hordeum vulgare	the MeJA responsive
TaMAPKKK4-1	CGTCA-motif	CGTCA	840	5 +	Hordeum vulgare	the MeJA responsive
TaMAPKKK4-1	CGTCA-motif	CGTCA	1193	5 -	Hordeum vulgare	the MeJA responsive
TaMAPKKK4-1	CGTCA-motif	CGTCA	1652	5 +	Hordeum vulgare	the MeJA responsive
TaMAPKKK4-1	TGACG-motif	TGACG	243	5 +	Hordeum vulgare	the MeJA responsive
TaMAPKKK4-1	TGACG-motif	TGACG	296	5 +	Hordeum vulgare	the MeJA responsive

TaMAPKKK4-1	TGACG-motif	TGACG	604	5 -	Hordeum vulgare	the MeJA responsive
TaMAPKKK4-1	TGACG-motif	TGACG	840	5 -	Hordeum vulgare	the MeJA responsive
TaMAPKKK4-1	TGACG-motif	TGACG	1193	5 +	Hordeum vulgare	the MeJA responsive
TaMAPKKK4-1	TGACG-motif	TGACG	1652	5 -	Hordeum vulgare	the MeJA responsive
TaMAPKKK4-1	TGA-element	AACGAC	973	6 -	Brassica oleracea	auxin responsive
TaMAPKKK4-1	GC-motif	CCCCCG	358	6 -	Zea mays	anoxic specific inducibility
TaMAPKKK4-1	GC-motif	CCCCCG	1028	6 +	Zea mays	anoxic specific inducibility
TaMAPKKK4-1	GARE-motif	TCTGTTG	1853	7 +	Brassica oleracea	gibberellin responsive
TaMAPKKK4-1	MBS	CAACTG	565	6 -	Arabidopsis thaliana	drought inducibility
TaMAPKKK4-1	MBS	CAACTG	1711	6 -	Arabidopsis thaliana	drought inducibility
TaMAPKKK4-1	MBS	CAACTG	1910	6 +	Arabidopsis thaliana	drought inducibility
TaMAPKKK42	LTR	CCGAAA	857	6 -	Hordeum vulgare	low temperature responsive
TaMAPKKK42	GARE-motif	TCTGTTG	433	7 -	Brassica oleracea	gibberellin responsive
TaMAPKKK42	GARE-motif	TCTGTTG	966	7 +	Brassica oleracea	gibberellin responsive
TaMAPKKK42	GARE-motif	TCTGTTG	1761	7 -	Brassica oleracea	gibberellin responsive
TaMAPKKK42	AuxRR-core	GGTCCAT	372	7 +	Nicotiana tabacum	auxin responsive
TaMAPKKK42	AuxRR-core	GGTCCAT	689	7 +	Nicotiana tabacum	auxin responsive
TaMAPKKK42	MBS	CAACTG	460	6 +	Arabidopsis thaliana	drought inducibility
TaMAPKKK42	O2-site	GATGACATGG	1268	9 +	Zea mays	zein metabolism regulation

TaMAPKKK42	ABRE	GCAACGTGTC	600	9 -	Hordeum vulgare	abscisic acid responsive
TaMAPKKK42	ABRE	ACGTG	1377	5 +	Arabidopsis thaliana	abscisic acid responsive
TaMAPKKK42	TGA-element	AACGAC	470	6 +	Brassica oleracea	auxin responsive
TaMAPKKK42	ARE	AAACCA	1188	6 +	Zea mays	anaerobic induction
TaMAPKKK42	ARE	AAACCA	1981	6 -	Zea mays	anaerobic induction
TaMAPKKK42	CGTCA-motif	CGTCA	70	5 -	Hordeum vulgare	the MeJA responsive
TaMAPKKK42	CGTCA-motif	CGTCA	222	5 -	Hordeum vulgare	the MeJA responsive
TaMAPKKK42	CGTCA-motif	CGTCA	356	5 -	Hordeum vulgare	the MeJA responsive
TaMAPKKK42	CGTCA-motif	CGTCA	1012	5 -	Hordeum vulgare	the MeJA responsive
TaMAPKKK42	CGTCA-motif	CGTCA	1018	5 -	Hordeum vulgare	the MeJA responsive
TaMAPKKK42	CGTCA-motif	CGTCA	1137	5 +	Hordeum vulgare	the MeJA responsive
TaMAPKKK42	CGTCA-motif	CGTCA	1528	5 +	Hordeum vulgare	the MeJA responsive
TaMAPKKK42	CGTCA-motif	CGTCA	1726	5 +	Hordeum vulgare	the MeJA responsive
TaMAPKKK42	TGACG-motif	TGACG	70	5 +	Hordeum vulgare	the MeJA responsive
TaMAPKKK42	TGACG-motif	TGACG	222	5 +	Hordeum vulgare	the MeJA responsive
TaMAPKKK42	TGACG-motif	TGACG	356	5 +	Hordeum vulgare	the MeJA responsive
TaMAPKKK42	TGACG-motif	TGACG	1012	5 +	Hordeum vulgare	the MeJA responsive
TaMAPKKK42	TGACG-motif	TGACG	1018	5 +	Hordeum vulgare	the MeJA responsive
TaMAPKKK42	TGACG-motif	TGACG	1137	5 -	Hordeum vulgare	the MeJA responsive

TaMAPKKK42	TGACG-motif	TGACG	1528	5 -	Hordeum vulgare	the MeJA responsive
TaMAPKKK42	TGACG-motif	TGACG	1726	5 -	Hordeum vulgare	the MeJA responsive
TaMAPKKK43	LTR	CCGAAA	829	6 +	Hordeum vulgare	low temperature responsive
TaMAPKKK43	LTR	CCGAAA	1353	6 -	Hordeum vulgare	low temperature responsive
TaMAPKKK43	LTR	CCGAAA	1932	6 -	Hordeum vulgare	low temperature responsive
TaMAPKKK43	MBS	CAACTG	1476	6 -	Arabidopsis thaliana	drought inducibility
TaMAPKKK43	O2-site	GATGATGTGG	890	9 +	Zea mays	zein metabolism regulation
TaMAPKKK43	GC-motif	CCCCCG	1489	6 -	Zea mays	anoxic specific inducibility
TaMAPKKK43	ABRE	CACGTG	1090	6 -	Arabidopsis thaliana	abscisic acid responsive
TaMAPKKK43	ABRE	ACGTG	1091	5 +	Arabidopsis thaliana	abscisic acid responsive
TaMAPKKK43	ABRE	AACCCGG	1270	7 +	Arabidopsis thaliana	abscisic acid responsive
TaMAPKKK43	CAT-box	GCCACT	1231	6 -	Arabidopsis thaliana	meristem expression
TaMAPKKK43	CAT-box	GCCACT	1463	6 -	Arabidopsis thaliana	meristem expression
TaMAPKKK43	TGA-element	AACGAC	1155	6 +	Brassica oleracea	auxin responsive
TaMAPKKK43	TGA-element	AACGAC	1385	6 +	Brassica oleracea	auxin responsive
TaMAPKKK43	MSA-like	(T/C)C(T/C)AAC GG(T/C)(T/C)A	872	8.5 -	Catharanthus roseus	cell cycle regulation
TaMAPKKK44	CAT-box	GCCACT	1858	6 +	Arabidopsis thaliana	meristem expression
TaMAPKKK44	ABRE	ACGTG	303	5 +	Arabidopsis thaliana	abscisic acid responsive
TaMAPKKK44	ABRE	ACGTG	840	5 +	Arabidopsis thaliana	abscisic acid responsive

TaMAPKKK44	ABRE	ACGTG	1299	5 -	Arabidopsis thaliana	abscisic acid responsive
TaMAPKKK44	ABRE	CGTACGTGCA	1652	9 -	Hordeum vulgare	abscisic acid responsive
TaMAPKKK44	TGA-element	AACGAC	276	6 -	Brassica oleracea	auxin responsive
TaMAPKKK44	TGA-element	AACGAC	1036	6 +	Brassica oleracea	auxin responsive
TaMAPKKK44	TGACG-motif	TGACG	736	5 +	Hordeum vulgare	the MeJA responsive
TaMAPKKK44	TGACG-motif	TGACG	1141	5 +	Hordeum vulgare	the MeJA responsive
TaMAPKKK44	TGACG-motif	TGACG	1814	5 +	Hordeum vulgare	the MeJA responsive
TaMAPKKK44	ARE	AAACCA	89	6 +	Zea mays	anaerobic induction
TaMAPKKK44	O2-site	GATGA(C/T)(A/G)TG(A/G)	1272	8 -	Zea mays	zein metabolism regulation
TaMAPKKK44	CGTCA-motif	CGTCA	736	5 -	Hordeum vulgare	the MeJA responsive
TaMAPKKK44	CGTCA-motif	CGTCA	1141	5 -	Hordeum vulgare	the MeJA responsive
TaMAPKKK44	CGTCA-motif	CGTCA	1814	5 -	Hordeum vulgare	the MeJA responsive
TaMAPKKK44	LTR	CCGAAA	1308	6 +	Hordeum vulgare	low temperature responsive
TaMAPKKK44	AuxRR-core	GGTCCAT	488	7 +	Nicotiana tabacum	auxin responsive
TaMAPKKK45	ARE	AAACCA	491	6 -	Zea mays	anaerobic induction
TaMAPKKK45	ARE	AAACCA	1431	6 -	Zea mays	anaerobic induction
TaMAPKKK45	ABRE	TACGTGTC	737	8 -	Oryza sativa	abscisic acid responsive
TaMAPKKK45	ABRE	ACGTG	739	5 -	Arabidopsis thaliana	abscisic acid responsive
TaMAPKKK45	ABRE	CACGTG	781	6 +	Arabidopsis thaliana	abscisic acid responsive
TaMAPKKK45	ABRE	ACGTG	782	5 +	Arabidopsis thaliana	abscisic acid responsive

TaMAPKKK45	ABRE	ACGTG	1229	5 -	Arabidopsis thaliana	abscisic acid responsive
TaMAPKKK45	ABRE	ACGTG	1306	5 -	Arabidopsis thaliana	abscisic acid responsive
TaMAPKKK45	ABRE	ACGTG	1326	5 -	Arabidopsis thaliana	abscisic acid responsive
TaMAPKKK45	ABRE	AACCCGG	1885	7 -	Arabidopsis thaliana	abscisic acid responsive
TaMAPKKK45	ABRE	ACGTG	1923	5 -	Arabidopsis thaliana	abscisic acid responsive
TaMAPKKK45	P-box	CCTTTTG	1456	7 +	Oryza sativa	gibberellin responsive
TaMAPKKK45	TGACG-motif	TGACG	839	5 -	Hordeum vulgare	the MeJA responsive
TaMAPKKK45	TGACG-motif	TGACG	1925	5 -	Hordeum vulgare	the MeJA responsive
TaMAPKKK45	CGTCA-motif	CGTCA	839	5 +	Hordeum vulgare	the MeJA responsive
TaMAPKKK45	CGTCA-motif	CGTCA	1925	5 +	Hordeum vulgare	the MeJA responsive
TaMAPKKK45	MBS	CAACTG	758	6 +	Arabidopsis thaliana	drought inducibility
TaMAPKKK45	MBS	CAACTG	1119	6 +	Arabidopsis thaliana	drought inducibility
TaMAPKKK45	GC-motif	CCCCCG	1161	6 +	Zea mays	anoxic specific inducibility
TaMAPKKK45	GC-motif	CCCCCG	1280	6 +	Zea mays	anoxic specific inducibility
TaMAPKKK46	TC-rich repeats	GTTTTCTTAC	295	9 +	Nicotiana tabacum	defense and stress responsive
TaMAPKKK46	MBS	CAACTG	1644	6 -	Arabidopsis thaliana	drought inducibility
TaMAPKKK46	CGTCA-motif	CGTCA	39	5 -	Hordeum vulgare	the MeJA responsive
TaMAPKKK46	TGA-element	AACGAC	427	6 +	Brassica oleracea	auxin responsive
TaMAPKKK46	TGA-element	AACGAC	497	6 -	Brassica oleracea	auxin responsive
TaMAPKKK46	CAT-box	GCCACT	1413	6 -	Arabidopsis thaliana	meristem expression

TaMAPKKK46	ABRE	ACGTG	41	5 +	Arabidopsis thaliana	abscisic acid responsive
TaMAPKKK46	ABRE	AACCCGG	369	7 -	Arabidopsis thaliana	abscisic acid responsive
TaMAPKKK46	ABRE	TACGTGTC	430	8 -	Oryza sativa	abscisic acid responsive
TaMAPKKK46	ABRE	ACGTG	432	5 -	Arabidopsis thaliana	abscisic acid responsive
TaMAPKKK46	ABRE	ACGTG	1274	5 +	Arabidopsis thaliana	abscisic acid responsive
TaMAPKKK46	ABRE	CGTACGTGCA	1746	9 -	Hordeum vulgare	abscisic acid responsive
TaMAPKKK46	ABRE	CACGTG	1830	6 -	Arabidopsis thaliana	abscisic acid responsive
TaMAPKKK46	ABRE	ACGTG	1831	5 +	Arabidopsis thaliana	abscisic acid responsive
TaMAPKKK46	ARE	AAACCA	568	6 -	Zea mays	anaerobic induction
TaMAPKKK46	circadian	CAAAGATATC	621	9 -	Lycopersicon esculentum	circadian control
TaMAPKKK46	TGACG-motif	TGACG	39	5 +	Hordeum vulgare	the MeJA responsive
TaMAPKKK47	TGA-element	AACGAC	500	6 -	Brassica oleracea	auxin responsive
TaMAPKKK47	ARE	AAACCA	755	6 -	Zea mays	anaerobic induction
TaMAPKKK47	ARE	AAACCA	1388	6 -	Zea mays	anaerobic induction
TaMAPKKK47	GC-motif	CCCCCG	48	6 +	Zea mays	anoxic specific inducibility
TaMAPKKK47	GC-motif	CCCCCG	1874	6 -	Zea mays	anoxic specific inducibility
TaMAPKKK47	CGTCA-motif	CGTCA	926	5 +	Hordeum vulgare	the MeJA responsive
TaMAPKKK47	CGTCA-motif	CGTCA	967	5 -	Hordeum vulgare	the MeJA responsive
TaMAPKKK47	TGACG-motif	TGACG	926	5 -	Hordeum vulgare	the MeJA responsive

TaMAPKKK47	TGACG-motif	TGACG	967	5 +	Hordeum vulgare	the MeJA responsive
TaMAPKKK47	MBS	CAACTG	1427	6 +	Arabidopsis thaliana	drought inducibility
TaMAPKKK47	MBS	CAACTG	1527	6 +	Arabidopsis thaliana	drought inducibility
TaMAPKKK47	CAT-box	GCCACT	766	6 +	Arabidopsis thaliana	meristem expression
TaMAPKKK48	ARE	AAACCA	450	6 +	Zea mays	anaerobic induction
TaMAPKKK48	ARE	AAACCA	517	6 +	Zea mays	anaerobic induction
TaMAPKKK48	ARE	AAACCA	635	6 +	Zea mays	anaerobic induction
TaMAPKKK48	ARE	AAACCA	786	6 +	Zea mays	anaerobic induction
TaMAPKKK48	ARE	AAACCA	1472	6 -	Zea mays	anaerobic induction
TaMAPKKK48	ARE	AAACCA	1915	6 +	Zea mays	anaerobic induction
TaMAPKKK48	ABRE	ACGTG	703	5 -	Arabidopsis thaliana	abscisic acid responsive
TaMAPKKK48	O2-site	GATGATGTGG	166	10 +	Zea mays	zein metabolism regulation
TaMAPKKK48	O2-site	GATGATGTGG	1370	9 -	Zea mays	zein metabolism regulation
TaMAPKKK48	TGACG-motif	TGACG	371	5 -	Hordeum vulgare	the MeJA responsive
TaMAPKKK48	TGACG-motif	TGACG	705	5 -	Hordeum vulgare	the MeJA responsive
TaMAPKKK48	CGTCA-motif	CGTCA	371	5 +	Hordeum vulgare	the MeJA responsive
TaMAPKKK48	CGTCA-motif	CGTCA	705	5 +	Hordeum vulgare	the MeJA responsive
TaMAPKKK48	P-box	CCTTTTG	1987	7 +	Oryza sativa	gibberellin responsive
TaMAPKKK48	LTR	CCGAAA	1587	6 -	Hordeum vulgare	low temperature responsive

TaMAPKKK48	TC-rich repeats	GTTTTCTTAC	1055	9 +	Nicotiana tabacum	defense and stress responsive
TaMAPKKK5	circadian	CAAAGATATC	382	9 -	Lycopersicon esculentum	circadian control
TaMAPKKK5	circadian	CAAAGATATC	572	9 -	Lycopersicon esculentum	circadian control
TaMAPKKK5	circadian	CAAAGATATC	1660	9 -	Lycopersicon esculentum	circadian control
TaMAPKKK5	O2-site	GATGATGTGG	935	9 -	Zea mays	zein metabolism regulation
TaMAPKKK5	O2-site	GATGA(C/T)(A/G)TG(A/G)	1088	8 +	Zea mays	zein metabolism regulation
TaMAPKKK5	O2-site	GATGACATGG	1093	9 +	Zea mays	zein metabolism regulation
TaMAPKKK5	O2-site	GATGA(C/T)(A/G)TG(A/G)	1710	8 -	Zea mays	zein metabolism regulation
TaMAPKKK5	TGACG-motif	TGACG	49	5 -	Hordeum vulgare	the MeJA responsive
TaMAPKKK5	TCA-element	CCATCTTTTT	531	9 +	Nicotiana tabacum	salicylic acid responsive
TaMAPKKK5	ARE	AAACCA	433	6 +	Zea mays	anaerobic induction
TaMAPKKK5	RY-element	CATGCATG	1178	8 -	Helianthus annuus	seed specific regulation
TaMAPKKK5	TGA-element	AACGAC	290	6 +	Brassica oleracea	auxin responsive
TaMAPKKK5	CGTCA-motif	CGTCA	49	5 +	Hordeum vulgare	the MeJA responsive
TaMAPKKK5	ABRE	CGTACGTGCA	30	9 -	Hordeum vulgare	abscisic acid responsive
TaMAPKKK5	ABRE	ACGTG	32	5 -	Arabidopsis thaliana	abscisic acid responsive
TaMAPKKK5	ABRE	ACGTG	359	5 -	Arabidopsis thaliana	abscisic acid responsive
TaMAPKKK5	GC-motif	CCCCCG	1824	6 +	Zea mays	anoxic specific inducibility
TaMAPKKK50	TCA-element	CCATCTTTTT	1889	9 -	Nicotiana tabacum	salicylic acid responsive

TaMAPKKK50	O2-site	GATGACATGG	919	9 -	Zea mays	zein metabolism regulation
TaMAPKKK50	O2-site	GATGACATGG	1564	9 +	Zea mays	zein metabolism regulation
TaMAPKKK50	GC-motif	CCCCCG	877	6 +	Zea mays	anoxic specific inducibility
TaMAPKKK50	ABRE	ACGTG	309	5 -	Arabidopsis thaliana	abscisic acid responsive
TaMAPKKK50	ABRE	ACGTG	1627	5 -	Arabidopsis thaliana	abscisic acid responsive
TaMAPKKK50	ARE	AAACCA	354	6 +	Zea mays	anaerobic induction
TaMAPKKK50	ARE	AAACCA	1896	6 -	Zea mays	anaerobic induction
TaMAPKKK50	CGTCA-motif	CGTCA	235	5 +	Hordeum vulgare	the MeJA responsive
TaMAPKKK50	CGTCA-motif	CGTCA	247	5 +	Hordeum vulgare	the MeJA responsive
TaMAPKKK50	CGTCA-motif	CGTCA	1348	5 +	Hordeum vulgare	the MeJA responsive
TaMAPKKK50	CGTCA-motif	CGTCA	1375	5 +	Hordeum vulgare	the MeJA responsive
TaMAPKKK50	TGACG-motif	TGACG	235	5 -	Hordeum vulgare	the MeJA responsive
TaMAPKKK50	TGACG-motif	TGACG	247	5 -	Hordeum vulgare	the MeJA responsive
TaMAPKKK50	TGACG-motif	TGACG	1348	5 -	Hordeum vulgare	the MeJA responsive
TaMAPKKK50	TGACG-motif	TGACG	1375	5 -	Hordeum vulgare	the MeJA responsive
TaMAPKKK50	LTR	CCGAAA	1521	6 -	Hordeum vulgare	low temperature responsive
TaMAPKKK50	GCN4_motif	TGAGTCA	1365	7 +	Oryza sativa	endosperm expression
TaMAPKKK50	AACA_motif	TAACAAACTCCA	1134	11 +	Oryza sativa	endosperm expression
TaMAPKKK50	MBS	CAACTG	1857	6 +	Arabidopsis thaliana	drought inducibility

TaMAPKKK51	ABRE	ACGTG	1875	5 -	Arabidopsis thaliana	abscisic acid responsive
TaMAPKKK51	ABRE	ACGTG	1911	5 -	Arabidopsis thaliana	abscisic acid responsive
TaMAPKKK51	ARE	AAACCA	269	6 +	Zea mays	anaerobic induction
TaMAPKKK51	ARE	AAACCA	1272	6 +	Zea mays	anaerobic induction
TaMAPKKK51	ARE	AAACCA	1620	6 -	Zea mays	anaerobic induction
TaMAPKKK51	TCA-element	CCATCTTTTT	272	9 +	Nicotiana tabacum	salicylic acid responsive
TaMAPKKK51	CGTCA-motif	CGTCA	668	5 +	Hordeum vulgare	the MeJA responsive
TaMAPKKK51	CGTCA-motif	CGTCA	1913	5 +	Hordeum vulgare	the MeJA responsive
TaMAPKKK51	CGTCA-motif	CGTCA	1923	5 +	Hordeum vulgare	the MeJA responsive
TaMAPKKK51	TGACG-motif	TGACG	668	5 -	Hordeum vulgare	the MeJA responsive
TaMAPKKK51	TGACG-motif	TGACG	1913	5 -	Hordeum vulgare	the MeJA responsive
TaMAPKKK51	TGACG-motif	TGACG	1923	5 -	Hordeum vulgare	the MeJA responsive
TaMAPKKK51	MBS	CAACTG	1789	6 +	Arabidopsis thaliana	drought inducibility
TaMAPKKK51	CAT-box	GCCACT	1486	6 -	Arabidopsis thaliana	meristem expression
TaMAPKKK52	MSA-like	(T/C)C(T/C)AAC GG(T/C)(T/C)A	17	8.5 -	Catharanthus roseus	cell cycle regulation
TaMAPKKK52	CGTCA-motif	CGTCA	1539	5 +	Hordeum vulgare	the MeJA responsive
TaMAPKKK52	CGTCA-motif	CGTCA	1674	5 -	Hordeum vulgare	the MeJA responsive
TaMAPKKK52	TGACG-motif	TGACG	1539	5 -	Hordeum vulgare	the MeJA responsive

TaMAPKKK52	TGACG-motif	TGACG	1674	5 +	Hordeum vulgare	the MeJA responsive
TaMAPKKK52	RY-element	CATGCATG	1698	8 -	Helianthus annuus	seed specific regulation
TaMAPKKK52	TGA-element	AACGAC	1878	6 -	Brassica oleracea	auxin responsive
TaMAPKKK52	ABRE	TACGGTC	915	7 +	Arabidopsis thaliana	abscisic acid responsive
TaMAPKKK52	ARE	AAACCA	179	6 +	Zea mays	anaerobic induction
TaMAPKKK52	ARE	AAACCA	253	6 +	Zea mays	anaerobic induction
TaMAPKKK52	ARE	AAACCA	1629	6 +	Zea mays	anaerobic induction
TaMAPKKK52	TCA-element	CCATCTTTTT	1801	9 -	Nicotiana tabacum	salicylic acid responsive
TaMAPKKK53	O2-site	GATGA(C/T)(A/G)TG(A/G)	1318	8 +	Zea mays	zein metabolism regulation
TaMAPKKK53	O2-site	GATGA(C/T)(A/G)TG(A/G)	1778	8 -	Zea mays	zein metabolism regulation
TaMAPKKK53	GCN4_motif	TGAGTCA	1479	7 +	Oryza sativa	endosperm expression
TaMAPKKK53	TGACG-motif	TGACG	479	5 +	Hordeum vulgare	the MeJA responsive
TaMAPKKK53	TGACG-motif	TGACG	971	5 +	Hordeum vulgare	the MeJA responsive
TaMAPKKK53	TGACG-motif	TGACG	1870	5 +	Hordeum vulgare	the MeJA responsive
TaMAPKKK53	ARE	AAACCA	890	6 -	Zea mays	anaerobic induction
TaMAPKKK53	RY-element	CATGCATG	1690	8 -	Helianthus annuus	seed specific regulation
TaMAPKKK53	TGA-box	TGACGTAA	1870	8 +	Glycine max	auxin responsive
TaMAPKKK53	CGTCA-motif	CGTCA	479	5 -	Hordeum vulgare	the MeJA responsive
TaMAPKKK53	CGTCA-motif	CGTCA	971	5 -	Hordeum vulgare	the MeJA responsive
TaMAPKKK53	CGTCA-motif	CGTCA	1870	5 -	Hordeum vulgare	the MeJA responsive

TaMAPKKK53	CAT-box	GCCACT	564	6 +	Arabidopsis thaliana	meristem expression
TaMAPKKK53	CAT-box	GCCACT	1339	6 -	Arabidopsis thaliana	meristem expression
TaMAPKKK53	CAT-box	GCCACT	1863	6 -	Arabidopsis thaliana	meristem expression
TaMAPKKK53	P-box	CCTTTTG	533	7 -	Oryza sativa	gibberellin responsive
TaMAPKKK53	ABRE	GACACGTGGC	545	9 +	Triticum aestivum	abscisic acid responsive
TaMAPKKK53	ABRE	CACGTG	547	6 +	Arabidopsis thaliana	abscisic acid responsive
TaMAPKKK53	ABRE	ACGTG	548	5 +	Arabidopsis thaliana	abscisic acid responsive
TaMAPKKK53	ABRE	ACGTG	1369	5 +	Arabidopsis thaliana	abscisic acid responsive
TaMAPKKK54	MBS	CAACTG	556	6 -	Arabidopsis thaliana	drought inducibility
TaMAPKKK54	MBS	CAACTG	1159	6 -	Arabidopsis thaliana	drought inducibility
TaMAPKKK54	CAT-box	GCCACT	822	6 -	Arabidopsis thaliana	meristem expression
TaMAPKKK54	CAT-box	GCCACT	971	6 +	Arabidopsis thaliana	meristem expression
TaMAPKKK54	CAT-box	GCCACT	1539	6 +	Arabidopsis thaliana	meristem expression
TaMAPKKK54	GARE-motif	TCTGTTG	647	7 +	Brassica oleracea	gibberellin responsive
TaMAPKKK54	TC-rich repeats	ATTCTCTAAC	1052	9 -	Nicotiana tabacum	defense and stress responsive
TaMAPKKK54	TGACG-motif	TGACG	58	5 +	Hordeum vulgare	the MeJA responsive
TaMAPKKK54	TGACG-motif	TGACG	83	5 +	Hordeum vulgare	the MeJA responsive
TaMAPKKK54	CGTCA-motif	CGTCA	58	5 -	Hordeum vulgare	the MeJA responsive
TaMAPKKK54	CGTCA-motif	CGTCA	83	5 -	Hordeum vulgare	the MeJA responsive

TaMAPKKK54	TATC-box	TATCCCA	1819	7 +	Oryza sativa	gibberellin responsive
TaMAPKKK54	TGA-element	AACGAC	118	6 +	Brassica oleracea	auxin responsive
TaMAPKKK54	ABRE	ACGTG	1108	5 -	Arabidopsis thaliana	abscisic acid responsive
TaMAPKKK54	ABRE	ACGTG	1498	5 +	Arabidopsis thaliana	abscisic acid responsive
TaMAPKKK54	O2-site	GATGACATGG	1703	9 -	Zea mays	zein metabolism regulation
TaMAPKKK56	ARE	AAACCA	1088	6 -	Zea mays	anaerobic induction
TaMAPKKK56	ARE	AAACCA	1356	6 +	Zea mays	anaerobic induction
TaMAPKKK56	TGACG-motif	TGACG	1446	5 +	Hordeum vulgare	the MeJA responsive
TaMAPKKK56	TGACG-motif	TGACG	1602	5 -	Hordeum vulgare	the MeJA responsive
TaMAPKKK56	TGACG-motif	TGACG	1926	5 -	Hordeum vulgare	the MeJA responsive
TaMAPKKK56	TGACG-motif	TGACG	1934	5 -	Hordeum vulgare	the MeJA responsive
TaMAPKKK56	TGACG-motif	TGACG	1961	5 -	Hordeum vulgare	the MeJA responsive
TaMAPKKK56	ABRE	ACGTG	698	5 +	Arabidopsis thaliana	abscisic acid responsive
TaMAPKKK56	CGTCA-motif	CGTCA	1446	5 -	Hordeum vulgare	the MeJA responsive
TaMAPKKK56	CGTCA-motif	CGTCA	1602	5 +	Hordeum vulgare	the MeJA responsive
TaMAPKKK56	CGTCA-motif	CGTCA	1926	5 +	Hordeum vulgare	the MeJA responsive
TaMAPKKK56	CGTCA-motif	CGTCA	1934	5 +	Hordeum vulgare	the MeJA responsive
TaMAPKKK56	CGTCA-motif	CGTCA	1961	5 +	Hordeum vulgare	the MeJA responsive
TaMAPKKK56	TC-rich repeats	GTTTTCTTAC	279	9 -	Nicotiana tabacum	defense and stress responsive

TaMAPKKK56	TC-rich repeats	GTTTTCTTAC	1304	9 -	Nicotiana tabacum	defense and stress responsive
TaMAPKKK56	O2-site	GATGATGTGG	787	10 -	Zea mays	zein metabolism regulation
TaMAPKKK56	GC-motif	CCCCCG	1682	6 +	Zea mays	anoxic specific inducibility
TaMAPKKK56	TCA-element	CCATCTTTTT	635	9 -	Nicotiana tabacum	salicylic acid responsive
TaMAPKKK56	P-box	CCTTTTG	503	7 -	Oryza sativa	gibberellin responsive
TaMAPKKK56	MBS	CAACTG	148	6 +	Arabidopsis thaliana	drought inducibility
TaMAPKKK56	MBS	CAACTG	967	6 +	Arabidopsis thaliana	drought inducibility
TaMAPKKK56	MBS	CAACTG	1758	6 +	Arabidopsis thaliana	drought inducibility
TaMAPKKK57	CAT-box	GCCACT	620	6 +	Arabidopsis thaliana	meristem expression
TaMAPKKK57	CAT-box	GCCACT	670	6 -	Arabidopsis thaliana	meristem expression
TaMAPKKK57	CAT-box	GCCACT	1883	6 -	Arabidopsis thaliana	meristem expression
TaMAPKKK57	MBS	CAACTG	1633	6 +	Arabidopsis thaliana	drought inducibility
TaMAPKKK57	O2-site	GATGACATGG	225	9 +	Zea mays	zein metabolism regulation
TaMAPKKK57	TGACG-motif	TGACG	35	5 +	Hordeum vulgare	the MeJA responsive
TaMAPKKK57	TGACG-motif	TGACG	597	5 -	Hordeum vulgare	the MeJA responsive
TaMAPKKK57	TGACG-motif	TGACG	612	5 -	Hordeum vulgare	the MeJA responsive
TaMAPKKK57	TGACG-motif	TGACG	629	5 +	Hordeum vulgare	the MeJA responsive
TaMAPKKK57	TGACG-motif	TGACG	1042	5 +	Hordeum vulgare	the MeJA responsive
TaMAPKKK57	TGACG-motif	TGACG	1045	5 -	Hordeum vulgare	the MeJA responsive

TaMAPKKK57	TC-rich repeats	ATTCTCTAAC	1262	9 +	Nicotiana tabacum	defense and stress responsive
TaMAPKKK57	ARE	AAACCA	701	6 +	Zea mays	anaerobic induction
TaMAPKKK57	ARE	AAACCA	928	6 -	Zea mays	anaerobic induction
TaMAPKKK57	ARE	AAACCA	963	6 -	Zea mays	anaerobic induction
TaMAPKKK57	ARE	AAACCA	1344	6 -	Zea mays	anaerobic induction
TaMAPKKK57	TCA-element	TCAGAAGAGG	55	9 -	Brassica oleracea	salicylic acid responsive
TaMAPKKK57	TCA-element	CCATCTTTTT	949	9 -	Nicotiana tabacum	salicylic acid responsive
TaMAPKKK57	ABRE	AACCCGG	433	7 -	Arabidopsis thaliana	abscisic acid responsive
TaMAPKKK57	ABRE	ACGTG	1309	5 -	Arabidopsis thaliana	abscisic acid responsive
TaMAPKKK57	ABRE	CGCACGTGTC	1688	9 +	Hordeum vulgare	abscisic acid responsive
TaMAPKKK57	ABRE	CACGTG	1690	6 -	Arabidopsis thaliana	abscisic acid responsive
TaMAPKKK57	ABRE	ACGTG	1691	5 +	Arabidopsis thaliana	abscisic acid responsive
TaMAPKKK57	ABRE	ACGTG	1929	5 -	Arabidopsis thaliana	abscisic acid responsive
TaMAPKKK57	TGA-element	AACGAC	836	6 +	Brassica oleracea	auxin responsive
TaMAPKKK57	CGTCA-motif	CGTCA	35	5 -	Hordeum vulgare	the MeJA responsive
TaMAPKKK57	CGTCA-motif	CGTCA	597	5 +	Hordeum vulgare	the MeJA responsive
TaMAPKKK57	CGTCA-motif	CGTCA	612	5 +	Hordeum vulgare	the MeJA responsive
TaMAPKKK57	CGTCA-motif	CGTCA	629	5 -	Hordeum vulgare	the MeJA responsive
TaMAPKKK57	CGTCA-motif	CGTCA	1042	5 -	Hordeum vulgare	the MeJA responsive

TaMAPKKK57	CGTCA-motif	CGTCA	1045	5 +	Hordeum vulgare	the MeJA responsive
TaMAPKKK58	TGACG-motif	TGACG	7	5 -	Hordeum vulgare	the MeJA responsive
TaMAPKKK58	TGACG-motif	TGACG	59	5 +	Hordeum vulgare	the MeJA responsive
TaMAPKKK58	TGACG-motif	TGACG	563	5 -	Hordeum vulgare	the MeJA responsive
TaMAPKKK58	TGACG-motif	TGACG	726	5 +	Hordeum vulgare	the MeJA responsive
TaMAPKKK58	TGACG-motif	TGACG	733	5 +	Hordeum vulgare	the MeJA responsive
TaMAPKKK58	TGACG-motif	TGACG	1276	5 +	Hordeum vulgare	the MeJA responsive
TaMAPKKK58	GARE-motif	TCTGTTG	695	7 -	Brassica oleracea	gibberellin responsive
TaMAPKKK58	ABRE	GACACGTGGC	114	9 -	Triticum aestivum	abscisic acid responsive
TaMAPKKK58	ABRE	ACGTG	509	5 -	Arabidopsis thaliana	abscisic acid responsive
TaMAPKKK58	ABRE	GCCGCGTGGC	1967	9 +	Oryza sativa	abscisic acid responsive
TaMAPKKK58	CAT-box	GCCACT	114	6 +	Arabidopsis thaliana	meristem expression
TaMAPKKK58	CAT-box	GCCACT	672	6 -	Arabidopsis thaliana	meristem expression
TaMAPKKK58	CAT-box	GCCACT	853	6 -	Arabidopsis thaliana	meristem expression
TaMAPKKK58	TGA-element	AACGAC	994	6 -	Brassica oleracea	auxin responsive
TaMAPKKK58	CGTCA-motif	CGTCA	7	5 +	Hordeum vulgare	the MeJA responsive
TaMAPKKK58	CGTCA-motif	CGTCA	59	5 -	Hordeum vulgare	the MeJA responsive
TaMAPKKK58	CGTCA-motif	CGTCA	563	5 +	Hordeum vulgare	the MeJA responsive
TaMAPKKK58	CGTCA-motif	CGTCA	726	5 -	Hordeum vulgare	the MeJA responsive

TaMAPKKK58	CGTCA-motif	CGTCA	733	5 -	Hordeum vulgare	the MeJA responsive
TaMAPKKK58	CGTCA-motif	CGTCA	1276	5 -	Hordeum vulgare	the MeJA responsive
TaMAPKKK58	AuxRR-core	GGTCCAT	410	7 +	Nicotiana tabacum	auxin responsive
TaMAPKKK58	P-box	CCTTTTG	353	7 +	Oryza sativa	gibberellin responsive
TaMAPKKK58	MBS	CAACTG	12	6 -	Arabidopsis thaliana	drought inducibility
TaMAPKKK58	MBS	CAACTG	481	6 +	Arabidopsis thaliana	drought inducibility
TaMAPKKK58	GCN4_motif	TGAGTCA	1491	7 +	Oryza sativa	endosperm expression
TaMAPKKK58	GC-motif	CCCCCG	1341	6 +	Zea mays	anoxic specific inducibility
TaMAPKKK58	GC-motif	CCCCCG	1424	6 -	Zea mays	anoxic specific inducibility
TaMAPKKK58	GC-motif	CCCCCG	1793	6 +	Zea mays	anoxic specific inducibility
TaMAPKKK58	O2-site	GTTGACGTGA	508	9 -	Zea mays	zein metabolism regulation
TaMAPKKK58	O2-site	GATGATGTGG	848	9 +	Zea mays	zein metabolism regulation
TaMAPKKK59	TGACG-motif	TGACG	321	5 +	Hordeum vulgare	the MeJA responsive
TaMAPKKK59	TGACG-motif	TGACG	359	5 -	Hordeum vulgare	the MeJA responsive
TaMAPKKK59	TGACG-motif	TGACG	603	5 +	Hordeum vulgare	the MeJA responsive
TaMAPKKK59	TGACG-motif	TGACG	896	5 -	Hordeum vulgare	the MeJA responsive
TaMAPKKK59	TGACG-motif	TGACG	1088	5 -	Hordeum vulgare	the MeJA responsive
TaMAPKKK59	TGACG-motif	TGACG	1174	5 -	Hordeum vulgare	the MeJA responsive
TaMAPKKK59	O2-site	GATGATGTGG	1944	9 -	Zea mays	zein metabolism regulation

TaMAPKKK59	TATC-box	TATCCCA	1761	7 +	Oryza sativa	gibberellin responsive
TaMAPKKK59	ABRE	TACGGTC	212	7 -	Arabidopsis thaliana	abscisic acid responsive
TaMAPKKK59	ABRE	ACGTG	250	5 +	Arabidopsis thaliana	abscisic acid responsive
TaMAPKKK59	ABRE	ACGTG	513	5 -	Arabidopsis thaliana	abscisic acid responsive
TaMAPKKK59	ABRE	ACGTG	532	5 +	Arabidopsis thaliana	abscisic acid responsive
TaMAPKKK59	ABRE	ACGTG	1076	5 -	Arabidopsis thaliana	abscisic acid responsive
TaMAPKKK59	TGA-element	AACGAC	858	6 -	Brassica oleracea	auxin responsive
TaMAPKKK59	CAT-box	GCCACT	40	6 -	Arabidopsis thaliana	meristem expression
TaMAPKKK59	CAT-box	GCCACT	82	6 -	Arabidopsis thaliana	meristem expression
TaMAPKKK59	CAT-box	GCCACT	877	6 +	Arabidopsis thaliana	meristem expression
TaMAPKKK59	CGTCA-motif	CGTCA	321	5 -	Hordeum vulgare	the MeJA responsive
TaMAPKKK59	CGTCA-motif	CGTCA	359	5 +	Hordeum vulgare	the MeJA responsive
TaMAPKKK59	CGTCA-motif	CGTCA	603	5 -	Hordeum vulgare	the MeJA responsive
TaMAPKKK59	CGTCA-motif	CGTCA	896	5 +	Hordeum vulgare	the MeJA responsive
TaMAPKKK59	CGTCA-motif	CGTCA	1088	5 +	Hordeum vulgare	the MeJA responsive
TaMAPKKK59	CGTCA-motif	CGTCA	1174	5 +	Hordeum vulgare	the MeJA responsive
TaMAPKKK59	MBS	CAACTG	1680	6 +	Arabidopsis thaliana	drought inducibility
TaMAPKKK59	RY-element	CATGCATG	475	8 +	Helianthus annuus	seed specific regulation
TaMAPKKK60	MBS	CAACTG	1597	6 +	Arabidopsis thaliana	drought inducibility

TaMAPKKK60	RY-element	CATGCATG	1704	8 -	Helianthus annuus	seed specific regulation
TaMAPKKK60	TC-rich repeats	ATTCTCTAAC	1425	9 +	Nicotiana tabacum	defense and stress responsive
TaMAPKKK60	P-box	CCTTTTG	220	7 +	Oryza sativa	gibberellin responsive
TaMAPKKK60	ABRE	GACACGTGGC	51	9 +	Triticum aestivum	abscisic acid responsive
TaMAPKKK60	ABRE	CACGTG	53	6 +	Arabidopsis thaliana	abscisic acid responsive
TaMAPKKK60	ABRE	ACGTG	54	5 +	Arabidopsis thaliana	abscisic acid responsive
TaMAPKKK60	ABRE	GACACGTGGC	755	9 +	Triticum aestivum	abscisic acid responsive
TaMAPKKK60	ABRE	ACGTG	1009	5 +	Arabidopsis thaliana	abscisic acid responsive
TaMAPKKK60	ABRE	ACGTG	1715	5 +	Arabidopsis thaliana	abscisic acid responsive
TaMAPKKK60	TGA-element	AACGAC	1432	6 +	Brassica oleracea	auxin responsive
TaMAPKKK60	CGTCA-motif	CGTCA	434	5 -	Hordeum vulgare	the MeJA responsive
TaMAPKKK60	CGTCA-motif	CGTCA	726	5 -	Hordeum vulgare	the MeJA responsive
TaMAPKKK60	CGTCA-motif	CGTCA	735	5 +	Hordeum vulgare	the MeJA responsive
TaMAPKKK60	CGTCA-motif	CGTCA	1245	5 -	Hordeum vulgare	the MeJA responsive
TaMAPKKK60	CGTCA-motif	CGTCA	1286	5 +	Hordeum vulgare	the MeJA responsive
TaMAPKKK60	CAT-box	GCCACT	237	6 -	Arabidopsis thaliana	meristem expression
TaMAPKKK60	O2-site	GATGACATGG	376	9 -	Zea mays	zein metabolism regulation
TaMAPKKK60	O2-site	GATGACATGG	1694	9 -	Zea mays	zein metabolism regulation
TaMAPKKK60	LTR	CCGAAA	121	6 -	Hordeum vulgare	low temperature responsive

TaMAPKKK60	LTR	CCGAAA	947	6 +	Hordeum vulgare	low temperature responsive
TaMAPKKK60	LTR	CCGAAA	1442	6 -	Hordeum vulgare	low temperature responsive
TaMAPKKK60	LTR	CCGAAA	1655	6 +	Hordeum vulgare	low temperature responsive
TaMAPKKK60	circadian	CAAAGATATC	766	9 -	Lycopersicon esculentum	circadian control
TaMAPKKK60	ARE	AAACCA	538	6 +	Zea mays	anaerobic induction
TaMAPKKK60	ARE	AAACCA	548	6 +	Zea mays	anaerobic induction
TaMAPKKK60	TCA-element	CCATCTTTTT	1526	9 -	Nicotiana tabacum	salicylic acid responsive
TaMAPKKK60	TGACG-motif	TGACG	434	5 +	Hordeum vulgare	the MeJA responsive
TaMAPKKK60	TGACG-motif	TGACG	726	5 +	Hordeum vulgare	the MeJA responsive
TaMAPKKK60	TGACG-motif	TGACG	735	5 -	Hordeum vulgare	the MeJA responsive
TaMAPKKK60	TGACG-motif	TGACG	1245	5 +	Hordeum vulgare	the MeJA responsive
TaMAPKKK60	TGACG-motif	TGACG	1286	5 -	Hordeum vulgare	the MeJA responsive
TaMAPKKK61	LTR	CCGAAA	660	6 +	Hordeum vulgare	low temperature responsive
TaMAPKKK61	MBS	CAACTG	1115	6 +	Arabidopsis thaliana	drought inducibility
TaMAPKKK61	MBS	CAACTG	1456	6 +	Arabidopsis thaliana	drought inducibility
TaMAPKKK61	CAT-box	GCCACT	602	6 +	Arabidopsis thaliana	meristem expression
TaMAPKKK61	TGA-element	AACGAC	614	6 +	Brassica oleracea	auxin responsive
TaMAPKKK61	O2-site	GTTGACGTGA	1109	9 -	Zea mays	zein metabolism regulation
TaMAPKKK61	CGTCA-motif	CGTCA	1112	5 +	Hordeum vulgare	the MeJA responsive

TaMAPKKK61	CGTCA-motif	CGTCA	1137	5 -	Hordeum vulgare	the MeJA responsive
TaMAPKKK61	TGACG-motif	TGACG	1112	5 -	Hordeum vulgare	the MeJA responsive
TaMAPKKK61	TGACG-motif	TGACG	1137	5 +	Hordeum vulgare	the MeJA responsive
TaMAPKKK61	RY-element	CATGCATG	419	8 +	Helianthus annuus	seed specific regulation
TaMAPKKK61	RY-element	CATGCATG	1416	8 -	Helianthus annuus	seed specific regulation
TaMAPKKK61	P-box	CCTTTTG	695	7 +	Oryza sativa	gibberellin responsive
TaMAPKKK62	ABRE	ACGTG	56	5 -	Arabidopsis thaliana	abscisic acid responsive
TaMAPKKK62	ABRE	ACGTG	85	5 +	Arabidopsis thaliana	abscisic acid responsive
TaMAPKKK62	ABRE	ACGTG	431	5 +	Arabidopsis thaliana	abscisic acid responsive
TaMAPKKK62	ABRE	ACGTG	643	5 -	Arabidopsis thaliana	abscisic acid responsive
TaMAPKKK62	ABRE	CACGTG	801	6 +	Arabidopsis thaliana	abscisic acid responsive
TaMAPKKK62	ABRE	ACGTG	802	5 +	Arabidopsis thaliana	abscisic acid responsive
TaMAPKKK62	ABRE	ACGTG	1576	5 +	Arabidopsis thaliana	abscisic acid responsive
TaMAPKKK62	ABRE	ACGTG	1740	5 -	Arabidopsis thaliana	abscisic acid responsive
TaMAPKKK62	TC-rich repeats	GTTTTCTTAC	443	9 -	Nicotiana tabacum	defense and stress responsive
TaMAPKKK62	MBS	CAACTG	834	6 +	Arabidopsis thaliana	drought inducibility
TaMAPKKK62	CGTCA-motif	CGTCA	83	5 -	Hordeum vulgare	the MeJA responsive
TaMAPKKK62	CGTCA-motif	CGTCA	1554	5 -	Hordeum vulgare	the MeJA responsive
TaMAPKKK62	TGACG-motif	TGACG	83	5 +	Hordeum vulgare	the MeJA responsive

TaMAPKKK62	TGACG-motif	TGACG	1554	5 +	Hordeum vulgare	the MeJA responsive
TaMAPKKK62	O2-site	GATGATGTGG	602	9 +	Zea mays	zein metabolism regulation
TaMAPKKK62	O2-site	GATGA(C/T)(A/G)TG(A/G)	1970	8 -	Zea mays	zein metabolism regulation
TaMAPKKK62	GC-motif	CCCCCG	465	6 -	Zea mays	anoxic specific inducibility
TaMAPKKK62	GC-motif	CCCCCG	1152	6 +	Zea mays	anoxic specific inducibility
TaMAPKKK62	GC-motif	CCCCCG	1484	6 +	Zea mays	anoxic specific inducibility
TaMAPKKK62	GC-motif	CCCCCG	1681	6 -	Zea mays	anoxic specific inducibility
TaMAPKKK62	CAT-box	GCCACT	1729	6 -	Arabidopsis thaliana	meristem expression
TaMAPKKK62	CAT-box	GCCACT	1783	6 -	Arabidopsis thaliana	meristem expression
TaMAPKKK62	P-box	CCTTTTG	1549	7 +	Oryza sativa	gibberellin responsive
TaMAPKKK63	ARE	AAACCA	1153	6 +	Zea mays	anaerobic induction
TaMAPKKK63	TGACG-motif	TGACG	70	5 -	Hordeum vulgare	the MeJA responsive
TaMAPKKK63	TGACG-motif	TGACG	1221	5 -	Hordeum vulgare	the MeJA responsive
TaMAPKKK63	TGACG-motif	TGACG	1337	5 -	Hordeum vulgare	the MeJA responsive
TaMAPKKK63	CAT-box	GCCACT	85	6 +	Arabidopsis thaliana	meristem expression
TaMAPKKK63	CAT-box	GCCACT	1352	6 +	Arabidopsis thaliana	meristem expression
TaMAPKKK63	MBSI	aaaAaaC(G/C)GTTA	499	10.5 +	Petunia hybrida	flavonoid biosynthetic genes regulation
TaMAPKKK63	AuxRR-core	GGTCCAT	669	7 +	Nicotiana tabacum	auxin responsive

TaMAPKKK63	CGTCA-motif	CGTCA	70	5 +	Hordeum vulgare	the MeJA responsive
TaMAPKKK63	CGTCA-motif	CGTCA	1221	5 +	Hordeum vulgare	the MeJA responsive
TaMAPKKK63	CGTCA-motif	CGTCA	1337	5 +	Hordeum vulgare	the MeJA responsive
TaMAPKKK63	LTR	CCGAAA	374	6 +	Hordeum vulgare	low temperature responsive
TaMAPKKK63	LTR	CCGAAA	1589	6 -	Hordeum vulgare	low temperature responsive
TaMAPKKK63	circadian	CAAAGATATC	976	9 +	Lycopersicon esculentum	circadian control
TaMAPKKK64	TCA-element	TCAGAAGAGG	415	9 -	Brassica oleracea	salicylic acid responsive
TaMAPKKK64	TCA-element	CCATCTTTTT	489	9 -	Nicotiana tabacum	salicylic acid responsive
TaMAPKKK64	TCA-element	CCATCTTTTT	725	9 -	Nicotiana tabacum	salicylic acid responsive
TaMAPKKK64	TCA-element	CCATCTTTTT	1624	9 +	Nicotiana tabacum	salicylic acid responsive
TaMAPKKK64	CGTCA-motif	CGTCA	422	5 -	Hordeum vulgare	the MeJA responsive
TaMAPKKK64	CGTCA-motif	CGTCA	430	5 +	Hordeum vulgare	the MeJA responsive
TaMAPKKK64	CGTCA-motif	CGTCA	1301	5 -	Hordeum vulgare	the MeJA responsive
TaMAPKKK64	ABRE	CGTACGTGCA	1929	9 -	Hordeum vulgare	abscisic acid responsive
TaMAPKKK64	TGACG-motif	TGACG	422	5 +	Hordeum vulgare	the MeJA responsive
TaMAPKKK64	TGACG-motif	TGACG	430	5 -	Hordeum vulgare	the MeJA responsive
TaMAPKKK64	TGACG-motif	TGACG	1301	5 +	Hordeum vulgare	the MeJA responsive
TaMAPKKK64	O2-site	GATGACATGG	158	9 -	Zea mays	zein metabolism regulation
TaMAPKKK64	O2-site	GATGACATGG	201	9 +	Zea mays	zein metabolism regulation

TaMAPKKK64	GARE-motif	TCTGTTG	136	7 -	Brassica oleracea	gibberellin responsive
TaMAPKKK64	MBS	CAACTG	19	6 -	Arabidopsis thaliana	drought inducibility
TaMAPKKK64	MBS	CAACTG	501	6 +	Arabidopsis thaliana	drought inducibility
TaMAPKKK64	MBS	CAACTG	532	6 -	Arabidopsis thaliana	drought inducibility
TaMAPKKK64	MBS	CAACTG	1892	6 +	Arabidopsis thaliana	drought inducibility
TaMAPKKK64	ARE	AAACCA	1067	6 -	Zea mays	anaerobic induction
TaMAPKKK64	ARE	AAACCA	1853	6 -	Zea mays	anaerobic induction
TaMAPKKK64-1	ABRE	TACGGTC	428	7 +	Arabidopsis thaliana	abscisic acid responsive
TaMAPKKK64-1	ABRE	CGTACGTGCA	1929	9 -	Hordeum vulgare	abscisic acid responsive
TaMAPKKK64-1	CGTCA-motif	CGTCA	157	5 -	Hordeum vulgare	the MeJA responsive
TaMAPKKK64-1	CGTCA-motif	CGTCA	1096	5 -	Hordeum vulgare	the MeJA responsive
TaMAPKKK64-1	CGTCA-motif	CGTCA	1394	5 +	Hordeum vulgare	the MeJA responsive
TaMAPKKK64-1	CGTCA-motif	CGTCA	1435	5 -	Hordeum vulgare	the MeJA responsive
TaMAPKKK64-1	LTR	CCGAAA	173	6 -	Hordeum vulgare	low temperature responsive
TaMAPKKK64-1	P-box	CCTTTTG	864	7 -	Oryza sativa	gibberellin responsive
TaMAPKKK64-1	P-box	CCTTTTG	1866	7 +	Oryza sativa	gibberellin responsive
TaMAPKKK64-1	CAT-box	GCCACT	193	6 -	Arabidopsis thaliana	meristem expression
TaMAPKKK64-1	ARE	AAACCA	580	6 -	Zea mays	anaerobic induction
TaMAPKKK64-1	ARE	AAACCA	1221	6 -	Zea mays	anaerobic induction

TaMAPKKK64-1	ARE	AAACCA	1532	6 -	Zea mays	anaerobic induction
TaMAPKKK64-1	ARE	AAACCA	1853	6 -	Zea mays	anaerobic induction
TaMAPKKK64-1	MBS	CAACTG	4	6 +	Arabidopsis thaliana	drought inducibility
TaMAPKKK64-1	MBS	CAACTG	35	6 -	Arabidopsis thaliana	drought inducibility
TaMAPKKK64-1	MBS	CAACTG	1892	6 +	Arabidopsis thaliana	drought inducibility
TaMAPKKK64-1	TGACG-motif	TGACG	157	5 +	Hordeum vulgare	the MeJA responsive
TaMAPKKK64-1	TGACG-motif	TGACG	1096	5 +	Hordeum vulgare	the MeJA responsive
TaMAPKKK64-1	TGACG-motif	TGACG	1394	5 -	Hordeum vulgare	the MeJA responsive
TaMAPKKK64-1	TGACG-motif	TGACG	1435	5 +	Hordeum vulgare	the MeJA responsive
TaMAPKKK64-1	O2-site	GTTGACGTGA	977	9 -	Zea mays	zein metabolism regulation
TaMAPKKK65	ABRE	ACGTG	219	5 -	Arabidopsis thaliana	abscisic acid responsive
TaMAPKKK65	ABRE	ACGTG	1234	5 -	Arabidopsis thaliana	abscisic acid responsive
TaMAPKKK65	WUN-motif	AAATTTCCT	5	9 +	Brassica oleracea	wound responsive
TaMAPKKK65	TGA-element	AACGAC	945	6 +	Brassica oleracea	auxin responsive
TaMAPKKK65	CGTCA-motif	CGTCA	1516	5 +	Hordeum vulgare	the MeJA responsive
TaMAPKKK65	CGTCA-motif	CGTCA	1667	5 -	Hordeum vulgare	the MeJA responsive
TaMAPKKK65	CGTCA-motif	CGTCA	1847	5 +	Hordeum vulgare	the MeJA responsive
TaMAPKKK65	LTR	CCGAAA	1865	6 +	Hordeum vulgare	low temperature responsive
TaMAPKKK65	ARE	AAACCA	235	6 -	Zea mays	anaerobic induction

TaMAPKKK65	ARE	AAACCA	312	6 -	Zea mays	anaerobic induction
TaMAPKKK65	ARE	AAACCA	1138	6 -	Zea mays	anaerobic induction
TaMAPKKK65	ARE	AAACCA	1823	6 -	Zea mays	anaerobic induction
TaMAPKKK65	GARE-motif	TCTGTTG	805	7 -	Brassica oleracea	gibberellin responsive
TaMAPKKK65	TGACG-motif	TGACG	1516	5 -	Hordeum vulgare	the MeJA responsive
TaMAPKKK65	TGACG-motif	TGACG	1667	5 +	Hordeum vulgare	the MeJA responsive
TaMAPKKK65	TGACG-motif	TGACG	1847	5 -	Hordeum vulgare	the MeJA responsive
TaMAPKKK65	O2-site	GATGACATGG	341	9 -	Zea mays	zein metabolism regulation
TaMAPKKK65	O2-site	GATGATGTGG	423	9 +	Zea mays	zein metabolism regulation
TaMAPKKK65	P-box	CCTTTTG	685	7 +	Oryza sativa	gibberellin responsive
TaMAPKKK65	CAT-box	GCCACT	798	6 +	Arabidopsis thaliana	meristem expression
TaMAPKKK65	CAT-box	GCCACT	1163	6 -	Arabidopsis thaliana	meristem expression
TaMAPKKK66	TGACG-motif	TGACG	313	5 +	Hordeum vulgare	the MeJA responsive
TaMAPKKK66	TGACG-motif	TGACG	766	5 +	Hordeum vulgare	the MeJA responsive
TaMAPKKK66	TGACG-motif	TGACG	1024	5 -	Hordeum vulgare	the MeJA responsive
TaMAPKKK66	TGACG-motif	TGACG	1697	5 +	Hordeum vulgare	the MeJA responsive
TaMAPKKK66	ARE	AAACCA	1896	6 -	Zea mays	anaerobic induction
TaMAPKKK66	circadian	CAAAGATATC	770	9 -	Lycopersicon esculentum	circadian control
TaMAPKKK66	circadian	CAAAGATATC	799	9 -	Lycopersicon esculentum	circadian control

TaMAPKKK66	circadian	CAAAGATATC	1015	9 +	Lycopersicon esculentum	circadian control
TaMAPKKK66	LTR	CCGAAA	110	6 +	Hordeum vulgare	low temperature responsive
TaMAPKKK66	LTR	CCGAAA	934	6 -	Hordeum vulgare	low temperature responsive
TaMAPKKK66	GCN4_motif	TGAGTCA	542	7 +	Oryza sativa	endosperm expression
TaMAPKKK66	CGTCA-motif	CGTCA	313	5 -	Hordeum vulgare	the MeJA responsive
TaMAPKKK66	CGTCA-motif	CGTCA	766	5 -	Hordeum vulgare	the MeJA responsive
TaMAPKKK66	CGTCA-motif	CGTCA	1024	5 +	Hordeum vulgare	the MeJA responsive
TaMAPKKK66	CGTCA-motif	CGTCA	1697	5 -	Hordeum vulgare	the MeJA responsive
TaMAPKKK66	MSA-like	(T/C)C(T/C)AAC GG(T/C)(T/C)A	345	8.5 -	Catharanthus roseus	cell cycle regulation
TaMAPKKK66	MSA-like	(T/C)C(T/C)AAC GG(T/C)(T/C)A	1420	9 -	Catharanthus roseus	cell cycle regulation
TaMAPKKK66	ABRE	GCAACGTGTC	257	9 -	Hordeum vulgare	abscisic acid responsive
TaMAPKKK66	ABRE	ACGTG	645	5 -	Arabidopsis thaliana	abscisic acid responsive
TaMAPKKK66	ABRE	CGTACGTGCA	1905	9 -	Hordeum vulgare	abscisic acid responsive
TaMAPKKK66	P-box	CCTTTTG	1824	7 +	Oryza sativa	gibberellin responsive
TaMAPKKK66	MBS	CAACTG	1527	6 +	Arabidopsis thaliana	drought inducibility
TaMAPKKK67	TGA-box	TGACGTAA	1868	8 +	Glycine max	auxin responsive
TaMAPKKK67	MBS	CAACTG	1452	6 +	Arabidopsis thaliana	drought inducibility
TaMAPKKK67	CGTCA-motif	CGTCA	1868	5 -	Hordeum vulgare	the MeJA responsive

TaMAPKKK67	CAT-box	GCCACT	1097	6 -	Arabidopsis thaliana	meristem expression
TaMAPKKK67	CAT-box	GCCACT	1590	6 -	Arabidopsis thaliana	meristem expression
TaMAPKKK67	CAT-box	GCCACT	1861	6 -	Arabidopsis thaliana	meristem expression
TaMAPKKK67	ABRE	GCAACGTGTC	619	9 -	Hordeum vulgare	abscisic acid responsive
TaMAPKKK67	ABRE	ACGTG	621	5 -	Arabidopsis thaliana	abscisic acid responsive
TaMAPKKK67	ABRE	ACGTG	1383	5 -	Arabidopsis thaliana	abscisic acid responsive
TaMAPKKK67	ABRE	ACGTG	1625	5 +	Arabidopsis thaliana	abscisic acid responsive
TaMAPKKK67	O2-site	GATGA(C/T)(A/G)TG(A/G)	1569	8 +	Zea mays	zein metabolism regulation
TaMAPKKK67	GCN4_motif	TGAGTCA	1749	7 +	Oryza sativa	endosperm expression
TaMAPKKK67	GARE-motif	TCTGTTG	78	7 +	Brassica oleracea	gibberellin responsive
TaMAPKKK67	LTR	CCGAAA	762	6 -	Hordeum vulgare	low temperature responsive
TaMAPKKK67	LTR	CCGAAA	1397	6 +	Hordeum vulgare	low temperature responsive
TaMAPKKK67	TGACG-motif	TGACG	1868	5 +	Hordeum vulgare	the MeJA responsive
TaMAPKKK67	ARE	AAACCA	543	6 -	Zea mays	anaerobic induction
TaMAPKKK67	ARE	AAACCA	927	6 +	Zea mays	anaerobic induction
TaMAPKKK67	ARE	AAACCA	1411	6 -	Zea mays	anaerobic induction
TaMAPKKK68	TGACG-motif	TGACG	85	5 -	Hordeum vulgare	the MeJA responsive
TaMAPKKK68	TGACG-motif	TGACG	337	5 -	Hordeum vulgare	the MeJA responsive
TaMAPKKK68	TGACG-motif	TGACG	728	5 -	Hordeum vulgare	the MeJA responsive

TaMAPKKK68	TGACG-motif	TGACG	1757	5 +	Hordeum vulgare	the MeJA responsive
TaMAPKKK68	LTR	CCGAAA	1158	6 -	Hordeum vulgare	low temperature responsive
TaMAPKKK68	AuxRE	TGTCTCAATAAG	1456	11 -	Glycine max	auxin responsive
TaMAPKKK68	TATC-box	TATCCCA	635	7 +	Oryza sativa	gibberellin responsive
TaMAPKKK68	ABRE	ACGTG	775	5 -	Arabidopsis thaliana	abscisic acid responsive
TaMAPKKK68	GC-motif	CCCCCG	1605	6 -	Zea mays	anoxic specific inducibility
TaMAPKKK68	CGTCA-motif	CGTCA	85	5 +	Hordeum vulgare	the MeJA responsive
TaMAPKKK68	CGTCA-motif	CGTCA	337	5 +	Hordeum vulgare	the MeJA responsive
TaMAPKKK68	CGTCA-motif	CGTCA	728	5 +	Hordeum vulgare	the MeJA responsive
TaMAPKKK68	CGTCA-motif	CGTCA	1757	5 -	Hordeum vulgare	the MeJA responsive
TaMAPKKK68	MBS	CAACTG	1669	6 +	Arabidopsis thaliana	drought inducibility
TaMAPKKK69	CGTCA-motif	CGTCA	328	5 +	Hordeum vulgare	the MeJA responsive
TaMAPKKK69	CGTCA-motif	CGTCA	722	5 -	Hordeum vulgare	the MeJA responsive
TaMAPKKK69	CGTCA-motif	CGTCA	1359	5 +	Hordeum vulgare	the MeJA responsive
TaMAPKKK69	CGTCA-motif	CGTCA	1883	5 +	Hordeum vulgare	the MeJA responsive
TaMAPKKK69	CGTCA-motif	CGTCA	1891	5 +	Hordeum vulgare	the MeJA responsive
TaMAPKKK69	GCN4_motif	TGAGTCA	271	7 +	Oryza sativa	endosperm expression
TaMAPKKK69	GC-motif	CCCCCG	1836	6 +	Zea mays	anoxic specific inducibility
TaMAPKKK69	GC-motif	CCCCCG	1980	6 +	Zea mays	anoxic specific inducibility

TaMAPKKK69	TGACG-motif	TGACG	328	5 -	Hordeum vulgare	the MeJA responsive
TaMAPKKK69	TGACG-motif	TGACG	722	5 +	Hordeum vulgare	the MeJA responsive
TaMAPKKK69	TGACG-motif	TGACG	1359	5 -	Hordeum vulgare	the MeJA responsive
TaMAPKKK69	TGACG-motif	TGACG	1883	5 -	Hordeum vulgare	the MeJA responsive
TaMAPKKK69	TGACG-motif	TGACG	1891	5 -	Hordeum vulgare	the MeJA responsive
TaMAPKKK69	ABRE	ACGTG	310	5 -	Arabidopsis thaliana	abscisic acid responsive
TaMAPKKK69	ABRE	ACGTG	1021	5 +	Arabidopsis thaliana	abscisic acid responsive
TaMAPKKK69	ABRE	ACGTG	1136	5 +	Arabidopsis thaliana	abscisic acid responsive
TaMAPKKK69	ABRE	ACGTG	1304	5 +	Arabidopsis thaliana	abscisic acid responsive
TaMAPKKK69	ABRE	ACGTG	1314	5 -	Arabidopsis thaliana	abscisic acid responsive
TaMAPKKK69	ABRE	ACGTG	1331	5 +	Arabidopsis thaliana	abscisic acid responsive
TaMAPKKK69	ABRE	CGCACGTGTC	1603	9 -	Hordeum vulgare	abscisic acid responsive
TaMAPKKK69	ABRE	CACGTG	1605	6 -	Arabidopsis thaliana	abscisic acid responsive
TaMAPKKK69	ABRE	ACGTG	1606	5 +	Arabidopsis thaliana	abscisic acid responsive
TaMAPKKK7	NON-box	AGATCGACG	750	9 -	Arabidopsis thaliana	meristem specific activation
TaMAPKKK7	ARE	AAACCA	721	6 +	Zea mays	anaerobic induction
TaMAPKKK7	TGACG-motif	TGACG	288	5 +	Hordeum vulgare	the MeJA responsive
TaMAPKKK7	TGACG-motif	TGACG	631	5 -	Hordeum vulgare	the MeJA responsive
TaMAPKKK7	TGACG-motif	TGACG	1076	5 -	Hordeum vulgare	the MeJA responsive

TaMAPKKK7	TGACG-motif	TGACG	1156	5 -	Hordeum vulgare	the MeJA responsive
TaMAPKKK7	TGACG-motif	TGACG	1212	5 +	Hordeum vulgare	the MeJA responsive
TaMAPKKK7	TGACG-motif	TGACG	1577	5 +	Hordeum vulgare	the MeJA responsive
TaMAPKKK7	TGACG-motif	TGACG	1607	5 -	Hordeum vulgare	the MeJA responsive
TaMAPKKK7	TGA-element	AACGAC	117	6 +	Brassica oleracea	auxin responsive
TaMAPKKK7	CAT-box	GCCACT	4	6 +	Arabidopsis thaliana	meristem expression
TaMAPKKK7	CAT-box	GCCACT	1106	6 -	Arabidopsis thaliana	meristem expression
TaMAPKKK7	ABRE	ACGTG	26	5 -	Arabidopsis thaliana	abscisic acid responsive
TaMAPKKK7	ABRE	ACGTG	1201	5 +	Arabidopsis thaliana	abscisic acid responsive
TaMAPKKK7	CGTCA-motif	CGTCA	288	5 -	Hordeum vulgare	the MeJA responsive
TaMAPKKK7	CGTCA-motif	CGTCA	631	5 +	Hordeum vulgare	the MeJA responsive
TaMAPKKK7	CGTCA-motif	CGTCA	1076	5 +	Hordeum vulgare	the MeJA responsive
TaMAPKKK7	CGTCA-motif	CGTCA	1156	5 +	Hordeum vulgare	the MeJA responsive
TaMAPKKK7	CGTCA-motif	CGTCA	1212	5 -	Hordeum vulgare	the MeJA responsive
TaMAPKKK7	CGTCA-motif	CGTCA	1577	5 -	Hordeum vulgare	the MeJA responsive
TaMAPKKK7	CGTCA-motif	CGTCA	1607	5 +	Hordeum vulgare	the MeJA responsive
TaMAPKKK7	TCA-element	CCATCTTTTT	1390	9 -	Nicotiana tabacum	salicylic acid responsive
TaMAPKKK7	TCA-element	CCATCTTTTT	1681	9 -	Nicotiana tabacum	salicylic acid responsive
TaMAPKKK7	GC-motif	CCCCCG	337	6 +	Zea mays	anoxic specific inducibility

TaMAPKKK7	TC-rich repeats	ATTCTCTAAC	342	9 -	Nicotiana tabacum	defense and stress responsive
TaMAPKKK7	MBS	CAACTG	101	6 +	Arabidopsis thaliana	drought inducibility
TaMAPKKK70	ARE	AAACCA	1765	6 +	Zea mays	anaerobic induction
TaMAPKKK70	TGACG-motif	TGACG	1452	5 +	Hordeum vulgare	the MeJA responsive
TaMAPKKK70	TGACG-motif	TGACG	1455	5 -	Hordeum vulgare	the MeJA responsive
TaMAPKKK70	TGACG-motif	TGACG	1908	5 -	Hordeum vulgare	the MeJA responsive
TaMAPKKK70	LTR	CCGAAA	89	6 +	Hordeum vulgare	low temperature responsive
TaMAPKKK70	LTR	CCGAAA	354	6 -	Hordeum vulgare	low temperature responsive
TaMAPKKK70	LTR	CCGAAA	824	6 -	Hordeum vulgare	low temperature responsive
TaMAPKKK70	O2-site	GATGACATGG	4	9 +	Zea mays	zein metabolism regulation
TaMAPKKK70	O2-site	GATGATGTGG	937	9 +	Zea mays	zein metabolism regulation
TaMAPKKK70	ABRE	ACGTG	184	5 +	Arabidopsis thaliana	abscisic acid responsive
TaMAPKKK70	GC-motif	CCCCCG	1703	6 +	Zea mays	anoxic specific inducibility
TaMAPKKK70	CGTCA-motif	CGTCA	1452	5 -	Hordeum vulgare	the MeJA responsive
TaMAPKKK70	CGTCA-motif	CGTCA	1455	5 +	Hordeum vulgare	the MeJA responsive
TaMAPKKK70	CGTCA-motif	CGTCA	1908	5 +	Hordeum vulgare	the MeJA responsive
TaMAPKKK70	TGA-element	AACGAC	916	6 -	Brassica oleracea	auxin responsive
TaMAPKKK70	RY-element	CATGCATG	510	8 +	Helianthus annuus	seed specific regulation
TaMAPKKK71	TATC-box	TATCCCA	815	7 +	Oryza sativa	gibberellin responsive

TaMAPKKK71	TCA-element	TCAGAAGAGG	1054	9	-	Brassica oleracea	salicylic acid responsive
TaMAPKKK71	MBS	CAACTG	1751	6	-	Arabidopsis thaliana	drought inducibility
TaMAPKKK71	TGA-element	AACGAC	1331	6	-	Brassica oleracea	auxin responsive
TaMAPKKK71	CGTCA-motif	CGTCA	687	5	-	Hordeum vulgare	the MeJA responsive
TaMAPKKK71	CGTCA-motif	CGTCA	966	5	-	Hordeum vulgare	the MeJA responsive
TaMAPKKK71	CGTCA-motif	CGTCA	1488	5	+	Hordeum vulgare	the MeJA responsive
TaMAPKKK71	CGTCA-motif	CGTCA	1606	5	-	Hordeum vulgare	the MeJA responsive
TaMAPKKK71	TGACG-motif	TGACG	687	5	+	Hordeum vulgare	the MeJA responsive
TaMAPKKK71	TGACG-motif	TGACG	966	5	+	Hordeum vulgare	the MeJA responsive
TaMAPKKK71	TGACG-motif	TGACG	1488	5	-	Hordeum vulgare	the MeJA responsive
TaMAPKKK71	TGACG-motif	TGACG	1606	5	+	Hordeum vulgare	the MeJA responsive
TaMAPKKK71	O2-site	GATGACATGG	1111	9	+	Zea mays	zein metabolism regulation
TaMAPKKK71	MSA-like	(T/C)C(T/C)AACGG(T/C)(T/C)A	664	8.5	-	Catharanthus roseus	cell cycle regulation
TaMAPKKK72	CGTCA-motif	CGTCA	881	5	+	Hordeum vulgare	the MeJA responsive
TaMAPKKK72	P-box	CCTTTTG	439	7	-	Oryza sativa	gibberellin responsive
TaMAPKKK72	GC-motif	CCCCCG	1830	6	+	Zea mays	anoxic specific inducibility
TaMAPKKK72	O2-site	GATGA(C/T)(A/G)TG(A/G)	451	8	-	Zea mays	zein metabolism regulation
TaMAPKKK72	TC-rich repeats	GTTTTCTTAC	136	9	-	Nicotiana tabacum	defense and stress responsive
TaMAPKKK72	TC-rich repeats	ATTCTCTAAC	1415	9	-	Nicotiana tabacum	defense and stress responsive

TaMAPKKK72	TGACG-motif	TGACG	881	5 -	Hordeum vulgare	the MeJA responsive
TaMAPKKK72	ARE	AAACCA	930	6 +	Zea mays	anaerobic induction
TaMAPKKK72	ARE	AAACCA	1573	6 -	Zea mays	anaerobic induction
TaMAPKKK72	CAT-box	GCCACT	741	6 -	Arabidopsis thaliana	meristem expression
TaMAPKKK72	ABRE	GCAACGTGTC	1296	9 -	Hordeum vulgare	abscisic acid responsive
TaMAPKKK72	ABRE	GCCGCGTGGC	1340	9 +	Oryza sativa	abscisic acid responsive
TaMAPKKK72	ABRE	AACCCGG	1800	7 -	Arabidopsis thaliana	abscisic acid responsive
TaMAPKKK73	TGACG-motif	TGACG	45	5 -	Hordeum vulgare	the MeJA responsive
TaMAPKKK73	TGACG-motif	TGACG	608	5 +	Hordeum vulgare	the MeJA responsive
TaMAPKKK73	TGACG-motif	TGACG	1570	5 +	Hordeum vulgare	the MeJA responsive
TaMAPKKK73	TGACG-motif	TGACG	1838	5 +	Hordeum vulgare	the MeJA responsive
TaMAPKKK73	circadian	CAAAGATATC	36	9 +	Lycopersicon esculentum	circadian control
TaMAPKKK73	ARE	AAACCA	216	6 +	Zea mays	anaerobic induction
TaMAPKKK73	CAT-box	GCCACT	1512	6 -	Arabidopsis thaliana	meristem expression
TaMAPKKK73	ABRE	CACGTG	1632	6 -	Arabidopsis thaliana	abscisic acid responsive
TaMAPKKK73	ABRE	ACGTG	1633	5 +	Arabidopsis thaliana	abscisic acid responsive
TaMAPKKK73	ABRE	GACACGTACG T	1981	10 +	Oryza sativa	abscisic acid responsive
TaMAPKKK73	ABRE	ACGTG	1983	5 -	Arabidopsis thaliana	abscisic acid responsive
TaMAPKKK73	TGA-element	AACGAC	1886	6 +	Brassica oleracea	auxin responsive

TaMAPKKK73	CGTCA-motif	CGTCA	45	5 +	Hordeum vulgare	the MeJA responsive
TaMAPKKK73	CGTCA-motif	CGTCA	608	5 -	Hordeum vulgare	the MeJA responsive
TaMAPKKK73	CGTCA-motif	CGTCA	1570	5 -	Hordeum vulgare	the MeJA responsive
TaMAPKKK73	CGTCA-motif	CGTCA	1838	5 -	Hordeum vulgare	the MeJA responsive
TaMAPKKK73	LTR	CCGAAA	1462	6 -	Hordeum vulgare	low temperature responsive
TaMAPKKK73	TCA-element	CCATCTTTTT	66	9 -	Nicotiana tabacum	salicylic acid responsive
TaMAPKKK74	MBS	CAACTG	1258	6 +	Arabidopsis thaliana	drought inducibility
TaMAPKKK74	GC-motif	CCCCCG	1743	6 +	Zea mays	anoxic specific inducibility
TaMAPKKK74	O2-site	GATGA(C/T)(A/G)TG(A/G)	1928	8 -	Zea mays	zein metabolism regulation
TaMAPKKK74	TCA-element	CCATCTTTTT	1431	9 +	Nicotiana tabacum	salicylic acid responsive
TaMAPKKK74	CGTCA-motif	CGTCA	390	5 +	Hordeum vulgare	the MeJA responsive
TaMAPKKK74	LTR	CCGAAA	1795	6 -	Hordeum vulgare	low temperature responsive
TaMAPKKK74	ABRE	ACGTG	924	5 +	Arabidopsis thaliana	abscisic acid responsive
TaMAPKKK74	TGA-element	AACGAC	1822	6 -	Brassica oleracea	auxin responsive
TaMAPKKK74	TGACG-motif	TGACG	390	5 -	Hordeum vulgare	the MeJA responsive
TaMAPKKK74	ARE	AAACCA	1298	6 +	Zea mays	anaerobic induction
TaMAPKKK74	ARE	AAACCA	1439	6 -	Zea mays	anaerobic induction
TaMAPKKK74	ARE	AAACCA	1466	6 +	Zea mays	anaerobic induction
TaMAPKKK75	ARE	AAACCA	38	6 -	Zea mays	anaerobic induction

TaMAPKKK75	ARE	AAACCA	1390	6 -	Zea mays	anaerobic induction
TaMAPKKK75	ARE	AAACCA	1407	6 -	Zea mays	anaerobic induction
TaMAPKKK75	ARE	AAACCA	1938	6 +	Zea mays	anaerobic induction
TaMAPKKK75	TGA-element	AACGAC	1153	6 -	Brassica oleracea	auxin responsive
TaMAPKKK75	CAT-box	GCCACT	318	6 +	Arabidopsis thaliana	meristem expression
TaMAPKKK75	ABRE	GACACGTGGC	344	9 -	Triticum aestivum	abscisic acid responsive
TaMAPKKK75	ABRE	CACGTG	346	6 +	Arabidopsis thaliana	abscisic acid responsive
TaMAPKKK75	ABRE	ACGTG	347	5 +	Arabidopsis thaliana	abscisic acid responsive
TaMAPKKK75	ABRE	ACGTG	504	5 +	Arabidopsis thaliana	abscisic acid responsive
TaMAPKKK75	ABRE	ACGTG	1084	5 -	Arabidopsis thaliana	abscisic acid responsive
TaMAPKKK75	ABRE	ACGTG	1302	5 +	Arabidopsis thaliana	abscisic acid responsive
TaMAPKKK75	ABRE	CGTACGTGCA	1376	9 +	Hordeum vulgare	abscisic acid responsive
TaMAPKKK75	ABRE	CGTACGTGCA	1650	9 -	Hordeum vulgare	abscisic acid responsive
TaMAPKKK75	ABRE	ACGTG	1652	5 -	Arabidopsis thaliana	abscisic acid responsive
TaMAPKKK75	ABRE	ACGTG	1766	5 +	Arabidopsis thaliana	abscisic acid responsive
TaMAPKKK75	P-box	CCTTTTG	758	7 +	Oryza sativa	gibberellin responsive
TaMAPKKK75	TGACG-motif	TGACG	343	5 -	Hordeum vulgare	the MeJA responsive
TaMAPKKK75	TGACG-motif	TGACG	1515	5 +	Hordeum vulgare	the MeJA responsive
TaMAPKKK75	CGTCA-motif	CGTCA	343	5 +	Hordeum vulgare	the MeJA responsive

TaMAPKKK75	CGTCA-motif	CGTCA	1515	5 -	Hordeum vulgare	the MeJA responsive
TaMAPKKK75	LTR	CCGAAA	524	6 -	Hordeum vulgare	low temperature responsive
TaMAPKKK75	LTR	CCGAAA	1871	6 -	Hordeum vulgare	low temperature responsive
TaMAPKKK75	O2-site	GATGACATGG	1098	9 -	Zea mays	zein metabolism regulation
TaMAPKKK75	MBS	CAACTG	887	6 -	Arabidopsis thaliana	drought inducibility
TaMAPKKK76	LTR	CCGAAA	995	6 +	Hordeum vulgare	low temperature responsive
TaMAPKKK76	LTR	CCGAAA	1248	6 +	Hordeum vulgare	low temperature responsive
TaMAPKKK76	LTR	CCGAAA	1444	6 +	Hordeum vulgare	low temperature responsive
TaMAPKKK76	CGTCA-motif	CGTCA	765	5 -	Hordeum vulgare	the MeJA responsive
TaMAPKKK76	CGTCA-motif	CGTCA	1139	5 -	Hordeum vulgare	the MeJA responsive
TaMAPKKK76	CGTCA-motif	CGTCA	1314	5 -	Hordeum vulgare	the MeJA responsive
TaMAPKKK76	CGTCA-motif	CGTCA	1403	5 -	Hordeum vulgare	the MeJA responsive
TaMAPKKK76	CGTCA-motif	CGTCA	1460	5 +	Hordeum vulgare	the MeJA responsive
TaMAPKKK76	CGTCA-motif	CGTCA	1896	5 -	Hordeum vulgare	the MeJA responsive
TaMAPKKK76	MBS	CAACTG	1366	6 +	Arabidopsis thaliana	drought inducibility
TaMAPKKK76	MBS	CAACTG	1901	6 +	Arabidopsis thaliana	drought inducibility
TaMAPKKK76	P-box	CCTTTTG	1166	7 -	Oryza sativa	gibberellin responsive
TaMAPKKK76	ARE	AAACCA	527	6 +	Zea mays	anaerobic induction
TaMAPKKK76	TGACG-motif	TGACG	765	5 +	Hordeum vulgare	the MeJA responsive

TaMAPKKK76	TGACG-motif	TGACG	1139	5 +	Hordeum vulgare	the MeJA responsive
TaMAPKKK76	TGACG-motif	TGACG	1314	5 +	Hordeum vulgare	the MeJA responsive
TaMAPKKK76	TGACG-motif	TGACG	1403	5 +	Hordeum vulgare	the MeJA responsive
TaMAPKKK76	TGACG-motif	TGACG	1460	5 -	Hordeum vulgare	the MeJA responsive
TaMAPKKK76	TGACG-motif	TGACG	1896	5 +	Hordeum vulgare	the MeJA responsive
TaMAPKKK76	TGA-element	AACGAC	1273	6 -	Brassica oleracea	auxin responsive
TaMAPKKK76	GARE-motif	TCTGTTG	172	7 +	Brassica oleracea	gibberellin responsive
TaMAPKKK76	GARE-motif	TCTGTTG	1591	7 +	Brassica oleracea	gibberellin responsive
TaMAPKKK76	ABRE	ACGTG	254	5 -	Arabidopsis thaliana	abscisic acid responsive
TaMAPKKK76	ABRE	ACGTG	568	5 +	Arabidopsis thaliana	abscisic acid responsive
TaMAPKKK76	ABRE	ACGTG	1728	5 +	Arabidopsis thaliana	abscisic acid responsive
TaMAPKKK76	ABRE	TACGTGTC	1906	8 -	Oryza sativa	abscisic acid responsive
TaMAPKKK76	ABRE	ACGTG	1908	5 -	Arabidopsis thaliana	abscisic acid responsive
TaMAPKKK78	CGTCA-motif	CGTCA	317	5 -	Hordeum vulgare	the MeJA responsive
TaMAPKKK78	CGTCA-motif	CGTCA	399	5 +	Hordeum vulgare	the MeJA responsive
TaMAPKKK78	CGTCA-motif	CGTCA	1770	5 +	Hordeum vulgare	the MeJA responsive
TaMAPKKK78	CGTCA-motif	CGTCA	1996	5 -	Hordeum vulgare	the MeJA responsive
TaMAPKKK78	LTR	CCGAAA	14	6 -	Hordeum vulgare	low temperature responsive
TaMAPKKK78	TATC-box	TATCCCA	50	7 +	Oryza sativa	gibberellin responsive
TaMAPKKK78	TGA-element	AACGAC	1593	6 -	Brassica oleracea	auxin responsive

TaMAPKKK78	CAT-box	GCCACT	1689	6 +	Arabidopsis thaliana	meristem expression
TaMAPKKK78	ABRE	AACCCGG	153	7 +	Arabidopsis thaliana	abscisic acid responsive
TaMAPKKK78	ABRE	GCCGCGTGGC	1426	9 +	Oryza sativa	abscisic acid responsive
TaMAPKKK78	ARE	AAACCA	1414	6 +	Zea mays	anaerobic induction
TaMAPKKK78	TGACG-motif	TGACG	317	5 +	Hordeum vulgare	the MeJA responsive
TaMAPKKK78	TGACG-motif	TGACG	399	5 -	Hordeum vulgare	the MeJA responsive
TaMAPKKK78	TGACG-motif	TGACG	1770	5 -	Hordeum vulgare	the MeJA responsive
TaMAPKKK78	TGACG-motif	TGACG	1996	5 +	Hordeum vulgare	the MeJA responsive
TaMAPKKK79	CGTCA-motif	CGTCA	1042	5 +	Hordeum vulgare	the MeJA responsive
TaMAPKKK79	TC-rich repeats	GTTTTCTTAC	1074	9 -	Nicotiana tabacum	defense and stress responsive
TaMAPKKK79	ARE	AAACCA	535	6 +	Zea mays	anaerobic induction
TaMAPKKK79	TGACG-motif	TGACG	1042	5 -	Hordeum vulgare	the MeJA responsive
TaMAPKKK79	TGA-element	AACGAC	861	6 -	Brassica oleracea	auxin responsive
TaMAPKKK79	ABRE	ACGTG	23	5 +	Arabidopsis thaliana	abscisic acid responsive
TaMAPKKK79	ABRE	ACGTG	1554	5 -	Arabidopsis thaliana	abscisic acid responsive
TaMAPKKK79	CAT-box	GCCACT	978	6 +	Arabidopsis thaliana	meristem expression
TaMAPKKK8	LTR	CCGAAA	15	6 -	Hordeum vulgare	low temperature responsive
TaMAPKKK8	LTR	CCGAAA	1562	6 +	Hordeum vulgare	low temperature responsive
TaMAPKKK8	CGTCA-motif	CGTCA	193	5 +	Hordeum vulgare	the MeJA responsive

TaMAPKKK8	CGTCA-motif	CGTCA	945	5 +	Hordeum vulgare	the MeJA responsive
TaMAPKKK8	CGTCA-motif	CGTCA	1769	5 -	Hordeum vulgare	the MeJA responsive
TaMAPKKK8	GC-motif	CCCCCG	63	6 +	Zea mays	anoxic specific inducibility
TaMAPKKK8	GC-motif	CCCCCG	1772	6 -	Zea mays	anoxic specific inducibility
TaMAPKKK8	MBS	CAACTG	41	6 +	Arabidopsis thaliana	drought inducibility
TaMAPKKK8	TGACG-motif	TGACG	193	5 -	Hordeum vulgare	the MeJA responsive
TaMAPKKK8	TGACG-motif	TGACG	945	5 -	Hordeum vulgare	the MeJA responsive
TaMAPKKK8	TGACG-motif	TGACG	1769	5 +	Hordeum vulgare	the MeJA responsive
TaMAPKKK8	TGA-element	AACGAC	986	6 +	Brassica oleracea	auxin responsive
TaMAPKKK8	CAT-box	GCCACT	474	6 -	Arabidopsis thaliana	meristem expression
TaMAPKKK8	ABRE	ACGTG	1575	5 +	Arabidopsis thaliana	abscisic acid responsive
TaMAPKKK8	ABRE	CGCACGTGTC	1822	9 +	Hordeum vulgare	abscisic acid responsive
TaMAPKKK8	ABRE	ACGTG	1824	5 -	Arabidopsis thaliana	abscisic acid responsive
TaMAPKKK8	ABRE	ACGTG	1917	5 -	Arabidopsis thaliana	abscisic acid responsive
TaMAPKKK80	TGACG-motif	TGACG	893	5 -	Hordeum vulgare	the MeJA responsive
TaMAPKKK80	TGACG-motif	TGACG	963	5 +	Hordeum vulgare	the MeJA responsive
TaMAPKKK80	GARE-motif	TCTGTTG	869	7 -	Brassica oleracea	gibberellin responsive
TaMAPKKK80	LTR	CCGAAA	1325	6 -	Hordeum vulgare	low temperature responsive
TaMAPKKK80	TATC-box	TATCCCA	98	7 -	Oryza sativa	gibberellin responsive

TaMAPKKK80	TATC-box	TATCCCA	795	7 -	Oryza sativa	gibberellin responsive
TaMAPKKK80	P-box	CCTTTTG	1060	7 -	Oryza sativa	gibberellin responsive
TaMAPKKK80	ABRE	ACGTG	582	5 +	Arabidopsis thaliana	abscisic acid responsive
TaMAPKKK80	ABRE	CACGTG	1025	6 -	Arabidopsis thaliana	abscisic acid responsive
TaMAPKKK80	ABRE	ACGTG	1026	5 +	Arabidopsis thaliana	abscisic acid responsive
TaMAPKKK80	ABRE	TACGGTC	1279	7 +	Arabidopsis thaliana	abscisic acid responsive
TaMAPKKK80	ABRE	ACGTG	1726	5 +	Arabidopsis thaliana	abscisic acid responsive
TaMAPKKK80	GC-motif	CCCCCG	1583	6 -	Zea mays	anoxic specific inducibility
TaMAPKKK80	TGA-element	AACGAC	1476	6 +	Brassica oleracea	auxin responsive
TaMAPKKK80	MSA-like	(T/C)C(T/C)AAC GG(T/C)(T/C)A	1154	8.5 -	Catharanthus roseus	cell cycle regulation
TaMAPKKK80	CAT-box	GCCACT	49	6 +	Arabidopsis thaliana	meristem expression
TaMAPKKK80	CGTCA-motif	CGTCA	893	5 +	Hordeum vulgare	the MeJA responsive
TaMAPKKK80	CGTCA-motif	CGTCA	963	5 -	Hordeum vulgare	the MeJA responsive
TaMAPKKK80	MBS	CAACTG	206	6 -	Arabidopsis thaliana	drought inducibility
TaMAPKKK81	ABRE	ACGTG	1202	5 -	Arabidopsis thaliana	abscisic acid responsive
TaMAPKKK81	WUN-motif	AAATTTCCT	1692	9 +	Brassica oleracea	wound responsive
TaMAPKKK81	CGTCA-motif	CGTCA	639	5 +	Hordeum vulgare	the MeJA responsive
TaMAPKKK81	CGTCA-motif	CGTCA	1840	5 +	Hordeum vulgare	the MeJA responsive
TaMAPKKK81	CGTCA-motif	CGTCA	1962	5 +	Hordeum vulgare	the MeJA responsive

TaMAPKKK81	TATC-box	TATCCCA	1049	7 -	Oryza sativa	gibberellin responsive
TaMAPKKK81	TATC-box	TATCCCA	1876	7 +	Oryza sativa	gibberellin responsive
TaMAPKKK81	TCA-element	CCATCTTTTT	281	10 -	Nicotiana tabacum	salicylic acid responsive
TaMAPKKK81	O2-site	GATGACATGG	1207	9 -	Zea mays	zein metabolism regulation
TaMAPKKK81	TGACG-motif	TGACG	639	5 -	Hordeum vulgare	the MeJA responsive
TaMAPKKK81	TGACG-motif	TGACG	1840	5 -	Hordeum vulgare	the MeJA responsive
TaMAPKKK81	TGACG-motif	TGACG	1962	5 -	Hordeum vulgare	the MeJA responsive
TaMAPKKK81	CAT-box	GCCACT	985	6 +	Arabidopsis thaliana	meristem expression
TaMAPKKK82	WUN-motif	AAATTTCCT	29	9 -	Brassica oleracea	wound responsive
TaMAPKKK82	MBS	CAACTG	789	6 +	Arabidopsis thaliana	drought inducibility
TaMAPKKK82	GARE-motif	TCTGTTG	1286	7 +	Brassica oleracea	gibberellin responsive
TaMAPKKK82	HD-Zip 1	CAAT(A/T)ATTG	1433	8 -	Arabidopsis thaliana	differentiation of the palisade mesophyll cells
TaMAPKKK82	TGACG-motif	TGACG	716	5 -	Hordeum vulgare	the MeJA responsive
TaMAPKKK82	TGACG-motif	TGACG	1211	5 +	Hordeum vulgare	the MeJA responsive
TaMAPKKK82	CGTCA-motif	CGTCA	716	5 +	Hordeum vulgare	the MeJA responsive
TaMAPKKK82	CGTCA-motif	CGTCA	1211	5 -	Hordeum vulgare	the MeJA responsive
TaMAPKKK82	TCA-element	CCATCTTTTT	1706	9 -	Nicotiana tabacum	salicylic acid responsive
TaMAPKKK82	TGA-element	AACGAC	184	6 -	Brassica oleracea	auxin responsive
TaMAPKKK82	ABRE	ACGTG	853	5 +	Arabidopsis thaliana	abscisic acid responsive

TaMAPKKK82	ARE	AAACCA	660	6 -	Zea mays	anaerobic induction
TaMAPKKK82	ARE	AAACCA	869	6 +	Zea mays	anaerobic induction
TaMAPKKK82	ARE	AAACCA	1793	6 +	Zea mays	anaerobic induction
TaMAPKKK83	CAT-box	GCCACT	14	6 -	Arabidopsis thaliana	meristem expression
TaMAPKKK83	CAT-box	GCCACT	269	6 -	Arabidopsis thaliana	meristem expression
TaMAPKKK83	TGACG-motif	TGACG	287	5 +	Hordeum vulgare	the MeJA responsive
TaMAPKKK83	TGACG-motif	TGACG	485	5 +	Hordeum vulgare	the MeJA responsive
TaMAPKKK83	TGACG-motif	TGACG	620	5 +	Hordeum vulgare	the MeJA responsive
TaMAPKKK83	TGACG-motif	TGACG	784	5 +	Hordeum vulgare	the MeJA responsive
TaMAPKKK83	TGACG-motif	TGACG	1383	5 -	Hordeum vulgare	the MeJA responsive
TaMAPKKK83	TGACG-motif	TGACG	1901	5 +	Hordeum vulgare	the MeJA responsive
TaMAPKKK83	MBS	CAACTG	886	6 +	Arabidopsis thaliana	drought inducibility
TaMAPKKK83	GARE-motif	TCTGTTG	64	7 -	Brassica oleracea	gibberellin responsive
TaMAPKKK83	ARE	AAACCA	926	6 -	Zea mays	anaerobic induction
TaMAPKKK83	ARE	AAACCA	1661	6 -	Zea mays	anaerobic induction
TaMAPKKK83	AuxRR-core	GGTCCAT	1035	7 +	Nicotiana tabacum	auxin responsive
TaMAPKKK83	CGTCA-motif	CGTCA	287	5 -	Hordeum vulgare	the MeJA responsive
TaMAPKKK83	CGTCA-motif	CGTCA	485	5 -	Hordeum vulgare	the MeJA responsive
TaMAPKKK83	CGTCA-motif	CGTCA	620	5 -	Hordeum vulgare	the MeJA responsive

TaMAPKKK83	CGTCA-motif	CGTCA	784	5 -	Hordeum vulgare	the MeJA responsive
TaMAPKKK83	CGTCA-motif	CGTCA	1383	5 +	Hordeum vulgare	the MeJA responsive
TaMAPKKK83	CGTCA-motif	CGTCA	1901	5 -	Hordeum vulgare	the MeJA responsive
TaMAPKKK84	GC-motif	CCCCCG	549	6 -	Zea mays	anoxic specific inducibility
TaMAPKKK84	TGACG-motif	TGACG	430	5 -	Hordeum vulgare	the MeJA responsive
TaMAPKKK84	TGACG-motif	TGACG	911	5 +	Hordeum vulgare	the MeJA responsive
TaMAPKKK84	TGACG-motif	TGACG	1754	5 +	Hordeum vulgare	the MeJA responsive
TaMAPKKK84	TATC-box	TATCCCA	127	7 -	Oryza sativa	gibberellin responsive
TaMAPKKK84	TATC-box	TATCCCA	574	7 +	Oryza sativa	gibberellin responsive
TaMAPKKK84	circadian	CAAAGATATC	1506	9 -	Lycopersicon esculentum	circadian control
TaMAPKKK84	TGA-element	AACGAC	1895	6 -	Brassica oleracea	auxin responsive
TaMAPKKK84	CGTCA-motif	CGTCA	430	5 +	Hordeum vulgare	the MeJA responsive
TaMAPKKK84	CGTCA-motif	CGTCA	911	5 -	Hordeum vulgare	the MeJA responsive
TaMAPKKK84	CGTCA-motif	CGTCA	1754	5 -	Hordeum vulgare	the MeJA responsive
TaMAPKKK84	LTR	CCGAAA	487	6 -	Hordeum vulgare	low temperature responsive
TaMAPKKK84	ABRE	ACGTG	990	5 +	Arabidopsis thaliana	abscisic acid responsive
TaMAPKKK84	ABRE	ACGTG	1776	5 +	Arabidopsis thaliana	abscisic acid responsive
TaMAPKKK85	MBS	CAACTG	1457	6 -	Arabidopsis thaliana	drought inducibility
TaMAPKKK85	TGACG-motif	TGACG	1338	5 +	Hordeum vulgare	the MeJA responsive

TaMAPKKK85	TGACG-motif	TGACG	1713	5 +	Hordeum vulgare	the MeJA responsive
TaMAPKKK85	TGACG-motif	TGACG	1808	5 -	Hordeum vulgare	the MeJA responsive
TaMAPKKK85	TC-rich repeats	ATTCTCTAAC	668	9 -	Nicotiana tabacum	defense and stress responsive
TaMAPKKK85	ARE	AAACCA	855	6 +	Zea mays	anaerobic induction
TaMAPKKK85	ARE	AAACCA	1131	6 -	Zea mays	anaerobic induction
TaMAPKKK85	ABRE	CGTACGTGCA	244	9 -	Hordeum vulgare	abscisic acid responsive
TaMAPKKK85	ABRE	ACGTG	246	5 -	Arabidopsis thaliana	abscisic acid responsive
TaMAPKKK85	ABRE	ACGTG	1340	5 +	Arabidopsis thaliana	abscisic acid responsive
TaMAPKKK85	TGA-element	AACGAC	70	6 -	Brassica oleracea	auxin responsive
TaMAPKKK85	CGTCA-motif	CGTCA	1338	5 -	Hordeum vulgare	the MeJA responsive
TaMAPKKK85	CGTCA-motif	CGTCA	1713	5 -	Hordeum vulgare	the MeJA responsive
TaMAPKKK85	CGTCA-motif	CGTCA	1808	5 +	Hordeum vulgare	the MeJA responsive
TaMAPKKK85	RY-element	CATGCATG	981	8 +	Helianthus annuus	seed specific regulation
TaMAPKKK86	TGA-element	AACGAC	314	6 -	Brassica oleracea	auxin responsive
TaMAPKKK86	MBS	CAACTG	372	6 +	Arabidopsis thaliana	drought inducibility
TaMAPKKK86	ARE	AAACCA	230	6 -	Zea mays	anaerobic induction
TaMAPKKK86	TGACG-motif	TGACG	344	5 +	Hordeum vulgare	the MeJA responsive
TaMAPKKK86	TGACG-motif	TGACG	1431	5 -	Hordeum vulgare	the MeJA responsive
TaMAPKKK86	TGACG-motif	TGACG	1747	5 -	Hordeum vulgare	the MeJA responsive
TaMAPKKK86	TGACG-motif	TGACG	1834	5 -	Hordeum vulgare	the MeJA responsive

TaMAPKKK86	LTR	CCGAAA	1737	6 -	Hordeum vulgare	low temperature responsive
TaMAPKKK86	ABRE	ACGTG	42	5 +	Arabidopsis thaliana	abscisic acid responsive
TaMAPKKK86	ABRE	TACGGTC	796	7 +	Arabidopsis thaliana	abscisic acid responsive
TaMAPKKK86	ABRE	ACGTG	1179	5 +	Arabidopsis thaliana	abscisic acid responsive
TaMAPKKK86	ABRE	TACGGTC	1637	7 +	Arabidopsis thaliana	abscisic acid responsive
TaMAPKKK86	GC-motif	CCCCCG	1513	6 +	Zea mays	anoxic specific inducibility
TaMAPKKK86	P-box	CCTTTTG	1789	7 +	Oryza sativa	gibberellin responsive
TaMAPKKK86	TCA-element	CCATCTTTTT	763	9 -	Nicotiana tabacum	salicylic acid responsive
TaMAPKKK86	O2-site	GATGA(C/T)(A/G)TG(A/G)	208	8 +	Zea mays	zein metabolism regulation
TaMAPKKK86	O2-site	GATGATGTGG	967	9 -	Zea mays	zein metabolism regulation
TaMAPKKK86	TC-rich repeats	GTTTTCTTAC	123	9 -	Nicotiana tabacum	defense and stress responsive
TaMAPKKK86	CGTCA-motif	CGTCA	344	5 -	Hordeum vulgare	the MeJA responsive
TaMAPKKK86	CGTCA-motif	CGTCA	1431	5 +	Hordeum vulgare	the MeJA responsive
TaMAPKKK86	CGTCA-motif	CGTCA	1747	5 +	Hordeum vulgare	the MeJA responsive
TaMAPKKK86	CGTCA-motif	CGTCA	1834	5 +	Hordeum vulgare	the MeJA responsive
TaMAPKKK88	CGTCA-motif	CGTCA	68	5 -	Hordeum vulgare	the MeJA responsive
TaMAPKKK88	CGTCA-motif	CGTCA	325	5 -	Hordeum vulgare	the MeJA responsive
TaMAPKKK88	CGTCA-motif	CGTCA	1918	5 +	Hordeum vulgare	the MeJA responsive
TaMAPKKK88	CAT-box	GCCACT	507	6 -	Arabidopsis thaliana	meristem expression

TaMAPKKK88	GC-motif	CCCCCG	623	6 -	Zea mays	anoxic specific inducibility
TaMAPKKK88	ABRE	ACGTG	1043	5 -	Arabidopsis thaliana	abscisic acid responsive
TaMAPKKK88	ABRE	ACGTG	1190	5 -	Arabidopsis thaliana	abscisic acid responsive
TaMAPKKK88	TGACG-motif	TGACG	68	5 +	Hordeum vulgare	the MeJA responsive
TaMAPKKK88	TGACG-motif	TGACG	325	5 +	Hordeum vulgare	the MeJA responsive
TaMAPKKK88	TGACG-motif	TGACG	1918	5 -	Hordeum vulgare	the MeJA responsive
TaMAPKKK88	ARE	AAACCA	1055	6 -	Zea mays	anaerobic induction
TaMAPKKK88	ARE	AAACCA	1818	6 +	Zea mays	anaerobic induction
TaMAPKKK88	LTR	CCGAAA	525	6 -	Hordeum vulgare	low temperature responsive
TaMAPKKK88	O2-site	GATGA(C/T)(A/G)TG(A/G)	819	8 -	Zea mays	zein metabolism regulation
TaMAPKKK88	O2-site	GATGACATGG	1029	9 +	Zea mays	zein metabolism regulation
TaMAPKKK88	O2-site	GATGACATGG	1048	9 +	Zea mays	zein metabolism regulation
TaMAPKKK9	ARE	AAACCA	566	6 +	Zea mays	anaerobic induction
TaMAPKKK9	TGACG-motif	TGACG	772	5 +	Hordeum vulgare	the MeJA responsive
TaMAPKKK9	TGACG-motif	TGACG	1095	5 +	Hordeum vulgare	the MeJA responsive
TaMAPKKK9	TGACG-motif	TGACG	1150	5 -	Hordeum vulgare	the MeJA responsive
TaMAPKKK9	TGACG-motif	TGACG	1449	5 -	Hordeum vulgare	the MeJA responsive
TaMAPKKK9	TGACG-motif	TGACG	1621	5 +	Hordeum vulgare	the MeJA responsive
TaMAPKKK9	TGA-element	AACGAC	1703	6 +	Brassica oleracea	auxin responsive

TaMAPKKK9	GARE-motif	TCTGTTG	871	7 -	Brassica oleracea	gibberellin responsive
TaMAPKKK9	ABRE	AACCCGG	240	7 -	Arabidopsis thaliana	abscisic acid responsive
TaMAPKKK9	ABRE	ACGTG	774	5 +	Arabidopsis thaliana	abscisic acid responsive
TaMAPKKK9	ABRE	GACACGTGGC	1034	9 -	Triticum aestivum	abscisic acid responsive
TaMAPKKK9	ABRE	CACGTG	1036	6 -	Arabidopsis thaliana	abscisic acid responsive
TaMAPKKK9	ABRE	ACGTG	1037	5 +	Arabidopsis thaliana	abscisic acid responsive
TaMAPKKK9	ABRE	ACGTG	1245	5 +	Arabidopsis thaliana	abscisic acid responsive
TaMAPKKK9	ABRE	ACGTG	1285	5 +	Arabidopsis thaliana	abscisic acid responsive
TaMAPKKK9	ABRE	ACGTG	1327	5 -	Arabidopsis thaliana	abscisic acid responsive
TaMAPKKK9	ABRE	ACGTG	1447	5 -	Arabidopsis thaliana	abscisic acid responsive
TaMAPKKK9	ABRE	CACGTG	1452	6 -	Arabidopsis thaliana	abscisic acid responsive
TaMAPKKK9	ABRE	ACGTG	1453	5 +	Arabidopsis thaliana	abscisic acid responsive
TaMAPKKK9	ABRE	CACGTG	1617	6 -	Arabidopsis thaliana	abscisic acid responsive
TaMAPKKK9	ABRE	ACGTG	1618	5 +	Arabidopsis thaliana	abscisic acid responsive
TaMAPKKK9	ABRE	ACGTG	1752	5 -	Arabidopsis thaliana	abscisic acid responsive
TaMAPKKK9	ABRE	CGCACGTGTC	1845	9 +	Hordeum vulgare	abscisic acid responsive
TaMAPKKK9	ABRE	CACGTG	1847	6 -	Arabidopsis thaliana	abscisic acid responsive
TaMAPKKK9	ABRE	ACGTG	1848	5 +	Arabidopsis thaliana	abscisic acid responsive
TaMAPKKK9	CAT-box	GCCACT	1894	6 +	Arabidopsis thaliana	meristem expression

TaMAPKKK9	CGTCA-motif	CGTCA	772	5 -	Hordeum vulgare	the MeJA responsive
TaMAPKKK9	CGTCA-motif	CGTCA	1095	5 -	Hordeum vulgare	the MeJA responsive
TaMAPKKK9	CGTCA-motif	CGTCA	1150	5 +	Hordeum vulgare	the MeJA responsive
TaMAPKKK9	CGTCA-motif	CGTCA	1449	5 +	Hordeum vulgare	the MeJA responsive
TaMAPKKK9	CGTCA-motif	CGTCA	1621	5 -	Hordeum vulgare	the MeJA responsive
TaMAPKKK9	LTR	CCGAAA	1196	6 +	Hordeum vulgare	low temperature responsive
TaMAPKKK9	GC-motif	CCCCCG	359	6 -	Zea mays	anoxic specific inducibility
TaMAPKKK9	GC-motif	CCCCCG	1193	6 +	Zea mays	anoxic specific inducibility
TaMAPKKK9	O2-site	GATGACATGG	616	10 +	Zea mays	zein metabolism regulation
TaMAPKKK9	O2-site	GATGA(C/T)(A/G)TG(A/G)	770	8 +	Zea mays	zein metabolism regulation
TaMAPKKK9	TCA-element	TCAGAAGAGG	1309	9 -	Brassica oleracea	salicylic acid responsive
TaMAPKKK9	P-box	CCTTTTG	960	7 -	Oryza sativa	gibberellin responsive
TaMAPKKK9	MBS	CAACTG	570	6 +	Arabidopsis thaliana	drought inducibility
TaMAPKKK90	CGTCA-motif	CGTCA	309	5 +	Hordeum vulgare	the MeJA responsive
TaMAPKKK90	CGTCA-motif	CGTCA	1228	5 +	Hordeum vulgare	the MeJA responsive
TaMAPKKK90	CGTCA-motif	CGTCA	1405	5 -	Hordeum vulgare	the MeJA responsive
TaMAPKKK90	O2-site	GATGATGTGG	910	9 -	Zea mays	zein metabolism regulation
TaMAPKKK90	TCA-element	TCAGAAGAGG	654	9 -	Brassica oleracea	salicylic acid responsive
TaMAPKKK90	ABRE	ACGTG	208	5 -	Arabidopsis thaliana	abscisic acid responsive

TaMAPKKK90	ABRE	ACGTG	251	5 -	Arabidopsis thaliana	abscisic acid responsive
TaMAPKKK90	ABRE	GCAACGTGTC	968	9 -	Hordeum vulgare	abscisic acid responsive
TaMAPKKK90	ABRE	ACGTG	970	5 -	Arabidopsis thaliana	abscisic acid responsive
TaMAPKKK90	ABRE	CGCACGTGTC	1009	10 -	Hordeum vulgare	abscisic acid responsive
TaMAPKKK90	ABRE	CACGTG	1011	6 -	Arabidopsis thaliana	abscisic acid responsive
TaMAPKKK90	ABRE	ACGTG	1012	5 +	Arabidopsis thaliana	abscisic acid responsive
TaMAPKKK90	ABRE	ACGTG	1430	5 +	Arabidopsis thaliana	abscisic acid responsive
TaMAPKKK90	ABRE	GCAACGTGTC	1589	9 -	Hordeum vulgare	abscisic acid responsive
TaMAPKKK90	ABRE	ACGTG	1591	5 -	Arabidopsis thaliana	abscisic acid responsive
TaMAPKKK90	ARE	AAACCA	1447	6 -	Zea mays	anaerobic induction
TaMAPKKK90	ARE	AAACCA	1534	6 -	Zea mays	anaerobic induction
TaMAPKKK90	ARE	AAACCA	1559	6 +	Zea mays	anaerobic induction
TaMAPKKK90	MBS	CAACTG	218	6 -	Arabidopsis thaliana	drought inducibility
TaMAPKKK90	MBS	CAACTG	517	6 +	Arabidopsis thaliana	drought inducibility
TaMAPKKK90	MBS	CAACTG	1627	6 +	Arabidopsis thaliana	drought inducibility
TaMAPKKK90	TGACG-motif	TGACG	309	5 -	Hordeum vulgare	the MeJA responsive
TaMAPKKK90	TGACG-motif	TGACG	1228	5 -	Hordeum vulgare	the MeJA responsive
TaMAPKKK90	TGACG-motif	TGACG	1405	5 +	Hordeum vulgare	the MeJA responsive
TaMAPKKK90	TGA-box	TGACGTAA	306	8 -	Glycine max	auxin responsive

TaMAPKKK90	CAT-box	GCCACT	1368	6 -	Arabidopsis thaliana	meristem expression
TaMAPKKK90	CAT-box	GCCACT	1667	6 -	Arabidopsis thaliana	meristem expression
TaMAPKKK91	ABRE	ACGTG	598	5 -	Arabidopsis thaliana	abscisic acid responsive
TaMAPKKK91	ABRE	ACGTG	921	5 +	Arabidopsis thaliana	abscisic acid responsive
TaMAPKKK91	ABRE	ACGTG	1366	5 -	Arabidopsis thaliana	abscisic acid responsive
TaMAPKKK91	ABRE	CACGTG	1385	6 -	Arabidopsis thaliana	abscisic acid responsive
TaMAPKKK91	ABRE	ACGTG	1386	5 +	Arabidopsis thaliana	abscisic acid responsive
TaMAPKKK91	CGTCA-motif	CGTCA	405	5 -	Hordeum vulgare	the MeJA responsive
TaMAPKKK91	CGTCA-motif	CGTCA	501	5 -	Hordeum vulgare	the MeJA responsive
TaMAPKKK91	CGTCA-motif	CGTCA	1205	5 -	Hordeum vulgare	the MeJA responsive
TaMAPKKK91	CGTCA-motif	CGTCA	1208	5 +	Hordeum vulgare	the MeJA responsive
TaMAPKKK91	TGA-element	AACGAC	212	6 +	Brassica oleracea	auxin responsive
TaMAPKKK91	TGA-element	AACGAC	1164	6 +	Brassica oleracea	auxin responsive
TaMAPKKK91	ARE	AAACCA	332	6 +	Zea mays	anaerobic induction
TaMAPKKK91	ARE	AAACCA	1112	6 -	Zea mays	anaerobic induction
TaMAPKKK91	ARE	AAACCA	1989	6 -	Zea mays	anaerobic induction
TaMAPKKK91	TGACG-motif	TGACG	405	5 +	Hordeum vulgare	the MeJA responsive
TaMAPKKK91	TGACG-motif	TGACG	501	5 +	Hordeum vulgare	the MeJA responsive
TaMAPKKK91	TGACG-motif	TGACG	1205	5 +	Hordeum vulgare	the MeJA responsive
TaMAPKKK91	TGACG-motif	TGACG	1208	5 -	Hordeum vulgare	the MeJA responsive

TaMAPKKK91	CAT-box	GCCACT	1231	6 +	Arabidopsis thaliana	meristem expression
TaMAPKKK92	TGACG-motif	TGACG	425	5 +	Hordeum vulgare	the MeJA responsive
TaMAPKKK92	TGACG-motif	TGACG	924	5 -	Hordeum vulgare	the MeJA responsive
TaMAPKKK92	CGTCA-motif	CGTCA	425	5 -	Hordeum vulgare	the MeJA responsive
TaMAPKKK92	CGTCA-motif	CGTCA	924	5 +	Hordeum vulgare	the MeJA responsive
TaMAPKKK92	AuxRR-core	GGTCCAT	837	7 +	Nicotiana tabacum	auxin responsive
TaMAPKKK92	LTR	CCGAAA	1044	6 -	Hordeum vulgare	low temperature responsive
TaMAPKKK92	LTR	CCGAAA	1962	6 +	Hordeum vulgare	low temperature responsive
TaMAPKKK92	ARE	AAACCA	1820	6 +	Zea mays	anaerobic induction
TaMAPKKK92	MSA-like	(T/C)C(T/C)AAC GG(T/C)(T/C)A	680	8.5 +	Catharanthus roseus	cell cycle regulation
TaMAPKKK92	MSA-like	(T/C)C(T/C)AAC GG(T/C)(T/C)A	895	8.5 +	Catharanthus roseus	cell cycle regulation
TaMAPKKK92	WUN-motif	AAATTTCCT	1218	9 +	Brassica oleracea	wound responsive
TaMAPKKK92	ABRE	ACGTG	107	5 -	Arabidopsis thaliana	abscisic acid responsive
TaMAPKKK92	ABRE	ACGTG	439	5 -	Arabidopsis thaliana	abscisic acid responsive
TaMAPKKK92	ABRE	CACGTG	644	6 +	Arabidopsis thaliana	abscisic acid responsive
TaMAPKKK92	ABRE	ACGTG	645	5 +	Arabidopsis thaliana	abscisic acid responsive
TaMAPKKK92	ABRE	ACGTG	888	5 +	Arabidopsis thaliana	abscisic acid responsive
TaMAPKKK92	ABRE	ACGTG	1101	5 +	Arabidopsis thaliana	abscisic acid responsive

TaMAPKKK92	ABRE	CGTACGTGCA	1804	9 -	Hordeum vulgare	abscisic acid responsive
TaMAPKKK92	ABRE	ACGTG	1810	5 -	Arabidopsis thaliana	abscisic acid responsive
TaMAPKKK92	GC-motif	CCCCCG	179	6 -	Zea mays	anoxic specific inducibility
TaMAPKKK93	CGTCA-motif	CGTCA	242	5 -	Hordeum vulgare	the MeJA responsive
TaMAPKKK93	CGTCA-motif	CGTCA	286	5 +	Hordeum vulgare	the MeJA responsive
TaMAPKKK93	CGTCA-motif	CGTCA	420	5 +	Hordeum vulgare	the MeJA responsive
TaMAPKKK93	TGACG-motif	TGACG	242	5 +	Hordeum vulgare	the MeJA responsive
TaMAPKKK93	TGACG-motif	TGACG	286	5 -	Hordeum vulgare	the MeJA responsive
TaMAPKKK93	TGACG-motif	TGACG	420	5 -	Hordeum vulgare	the MeJA responsive
TaMAPKKK93	P-box	CCTTTTG	476	7 +	Oryza sativa	gibberellin responsive
TaMAPKKK93	MBS	CAACTG	1331	6 -	Arabidopsis thaliana	drought inducibility
TaMAPKKK93	MBS	CAACTG	1466	6 -	Arabidopsis thaliana	drought inducibility
TaMAPKKK93	TGA-element	AACGAC	1151	6 +	Brassica oleracea	auxin responsive
TaMAPKKK93	ABRE	TACGGTC	351	7 -	Arabidopsis thaliana	abscisic acid responsive
TaMAPKKK93	ABRE	ACGTG	385	5 +	Arabidopsis thaliana	abscisic acid responsive
TaMAPKKK93	ABRE	CACGTG	1028	6 -	Arabidopsis thaliana	abscisic acid responsive
TaMAPKKK93	ABRE	ACGTG	1029	5 +	Arabidopsis thaliana	abscisic acid responsive
TaMAPKKK93	ABRE	ACGTG	1155	5 +	Arabidopsis thaliana	abscisic acid responsive
TaMAPKKK94	TATC-box	TATCCCA	314	7 -	Oryza sativa	gibberellin responsive

TaMAPKKK94	TCA-element	TCAGAAGAGG	1044	9 +	Brassica oleracea	salicylic acid responsive
TaMAPKKK94	ABRE	ACGTG	289	5 -	Arabidopsis thaliana	abscisic acid responsive
TaMAPKKK94	ABRE	TACGGTC	1890	7 +	Arabidopsis thaliana	abscisic acid responsive
TaMAPKKK94	TGA-element	AACGAC	1143	6 +	Brassica oleracea	auxin responsive
TaMAPKKK94	CGTCA-motif	CGTCA	524	5 +	Hordeum vulgare	the MeJA responsive
TaMAPKKK94	CGTCA-motif	CGTCA	553	5 +	Hordeum vulgare	the MeJA responsive
TaMAPKKK94	CGTCA-motif	CGTCA	617	5 -	Hordeum vulgare	the MeJA responsive
TaMAPKKK94	CAT-box	GCCACT	971	6 +	Arabidopsis thaliana	meristem expression
TaMAPKKK94	CAT-box	GCCACT	1586	6 -	Arabidopsis thaliana	meristem expression
TaMAPKKK94	GARE-motif	TCTGTTG	769	7 -	Brassica oleracea	gibberellin responsive
TaMAPKKK94	TGACG-motif	TGACG	524	5 -	Hordeum vulgare	the MeJA responsive
TaMAPKKK94	TGACG-motif	TGACG	553	5 -	Hordeum vulgare	the MeJA responsive
TaMAPKKK94	TGACG-motif	TGACG	617	5 +	Hordeum vulgare	the MeJA responsive
TaMAPKKK94	ARE	AAACCA	834	6 +	Zea mays	anaerobic induction
TaMAPKKK94	ARE	AAACCA	1716	6 -	Zea mays	anaerobic induction
TaMAPKKK94	ARE	AAACCA	1760	6 +	Zea mays	anaerobic induction
TaMAPKKK95	ABRE	CGCACGTGTC	163	9 -	Hordeum vulgare	abscisic acid responsive
TaMAPKKK95	ABRE	ACGTG	1200	5 +	Arabidopsis thaliana	abscisic acid responsive
TaMAPKKK95	ABRE	CGTACGTGCA	1305	9 -	Hordeum vulgare	abscisic acid responsive

TaMAPKKK95	ABRE	ACGTG	1612	5 -	Arabidopsis thaliana	abscisic acid responsive
TaMAPKKK95	ABRE	CGTACGTGCA	1624	9 -	Hordeum vulgare	abscisic acid responsive
TaMAPKKK95	ABRE	CGTACGTGCA	1693	9 +	Hordeum vulgare	abscisic acid responsive
TaMAPKKK95	ABRE	CGCACGTGTC	1851	9 -	Hordeum vulgare	abscisic acid responsive
TaMAPKKK95	TGA-element	AACGAC	390	6 +	Brassica oleracea	auxin responsive
TaMAPKKK95	CGTCA-motif	CGTCA	522	5 +	Hordeum vulgare	the MeJA responsive
TaMAPKKK95	CGTCA-motif	CGTCA	622	5 +	Hordeum vulgare	the MeJA responsive
TaMAPKKK95	CGTCA-motif	CGTCA	895	5 -	Hordeum vulgare	the MeJA responsive
TaMAPKKK95	CGTCA-motif	CGTCA	1341	5 -	Hordeum vulgare	the MeJA responsive
TaMAPKKK95	CAT-box	GCCACT	1299	6 +	Arabidopsis thaliana	meristem expression
TaMAPKKK95	GC-motif	CCCCCG	1452	6 -	Zea mays	anoxic specific inducibility
TaMAPKKK95	ARE	AAACCA	279	6 +	Zea mays	anaerobic induction
TaMAPKKK95	GARE-motif	TCTGTTG	59	7 -	Brassica oleracea	gibberellin responsive
TaMAPKKK95	GARE-motif	TCTGTTG	1597	7 +	Brassica oleracea	gibberellin responsive
TaMAPKKK95	TGACG-motif	TGACG	522	5 -	Hordeum vulgare	the MeJA responsive
TaMAPKKK95	TGACG-motif	TGACG	622	5 -	Hordeum vulgare	the MeJA responsive
TaMAPKKK95	TGACG-motif	TGACG	895	5 +	Hordeum vulgare	the MeJA responsive
TaMAPKKK95	TGACG-motif	TGACG	1341	5 +	Hordeum vulgare	the MeJA responsive
TaMAPKKK96	TC-rich repeats	GTTTTCTTAC	797	9 +	Nicotiana tabacum	defense and stress responsive

TaMAPKKK96	ARE	AAACCA	959	6 +	Zea mays	anaerobic induction
TaMAPKKK96	ARE	AAACCA	1109	6 +	Zea mays	anaerobic induction
TaMAPKKK96	ARE	AAACCA	1285	6 -	Zea mays	anaerobic induction
TaMAPKKK96	CAT-box	GCCACT	147	6 +	Arabidopsis thaliana	meristem expression
TaMAPKKK96	CAT-box	GCCACT	917	6 +	Arabidopsis thaliana	meristem expression
TaMAPKKK96	TATC-box	TATCCCA	1871	7 +	Oryza sativa	gibberellin responsive
TaMAPKKK96	circadian	CAAAGATATC	1977	9 -	Lycopersicon esculentum	circadian control
TaMAPKKK96	TGA-element	AACGAC	1572	6 +	Brassica oleracea	auxin responsive
TaMAPKKK96	ABRE	ACGTG	395	5 -	Arabidopsis thaliana	abscisic acid responsive
TaMAPKKK96	ABRE	AACCCGG	404	7 +	Arabidopsis thaliana	abscisic acid responsive
TaMAPKKK96	ABRE	ACGTG	794	5 -	Arabidopsis thaliana	abscisic acid responsive
TaMAPKKK97	AuxRR-core	GGTCCAT	730	7 -	Nicotiana tabacum	auxin responsive
TaMAPKKK97	CGTCA-motif	CGTCA	129	5 -	Hordeum vulgare	the MeJA responsive
TaMAPKKK97	CGTCA-motif	CGTCA	1461	5 -	Hordeum vulgare	the MeJA responsive
TaMAPKKK97	TGACG-motif	TGACG	129	5 +	Hordeum vulgare	the MeJA responsive
TaMAPKKK97	TGACG-motif	TGACG	1461	5 +	Hordeum vulgare	the MeJA responsive
TaMAPKKK97	CAT-box	GCCACT	101	6 -	Arabidopsis thaliana	meristem expression
TaMAPKKK97	ARE	AAACCA	835	6 +	Zea mays	anaerobic induction
TaMAPKKK97	ARE	AAACCA	1269	6 -	Zea mays	anaerobic induction

TaMAPKKK97	ARE	AAACCA	1927	6 +	Zea mays	anaerobic induction
TaMAPKKK97	TATC-box	TATCCCA	1165	7 -	Oryza sativa	gibberellin responsive
TaMAPKKK97	MBS	CAACTG	1946	6 +	Arabidopsis thaliana	drought inducibility
TaMAPKKK97	ABRE	ACGTG	64	5 +	Arabidopsis thaliana	abscisic acid responsive
TaMAPKKK98	GC-motif	CCCCCG	1455	6 -	Zea mays	anoxic specific inducibility
TaMAPKKK98	GC-motif	CCCCCG	1491	6 +	Zea mays	anoxic specific inducibility
TaMAPKKK98	GC-motif	CCCCCG	1585	6 +	Zea mays	anoxic specific inducibility
TaMAPKKK98	GC-motif	CCCCCG	1689	6 +	Zea mays	anoxic specific inducibility
TaMAPKKK98	TGA-element	AACGAC	946	6 -	Brassica oleracea	auxin responsive
TaMAPKKK98	ABRE	GCCGCGTGGC	156	9 -	Oryza sativa	abscisic acid responsive
TaMAPKKK98	ABRE	ACGTG	289	5 -	Arabidopsis thaliana	abscisic acid responsive
TaMAPKKK98	ABRE	ACGTG	305	5 -	Arabidopsis thaliana	abscisic acid responsive
TaMAPKKK98	ABRE	CACGTG	344	6 +	Arabidopsis thaliana	abscisic acid responsive
TaMAPKKK98	ABRE	ACGTG	345	5 +	Arabidopsis thaliana	abscisic acid responsive
TaMAPKKK98	ABRE	ACGTG	513	5 -	Arabidopsis thaliana	abscisic acid responsive
TaMAPKKK98	ABRE	ACGTG	541	5 -	Arabidopsis thaliana	abscisic acid responsive
TaMAPKKK98	ABRE	ACGTG	902	5 -	Arabidopsis thaliana	abscisic acid responsive
TaMAPKKK98	ABRE	ACGTG	1021	5 -	Arabidopsis thaliana	abscisic acid responsive
TaMAPKKK98	ABRE	GCCGCGTGGC	1701	9 -	Oryza sativa	abscisic acid responsive

TaMAPKKK98	LTR	CCGAAA	319	6 +	Hordeum vulgare	low temperature responsive
TaMAPKKK98	TGACG-motif	TGACG	448	5 -	Hordeum vulgare	the MeJA responsive
TaMAPKKK98	TGACG-motif	TGACG	482	5 -	Hordeum vulgare	the MeJA responsive
TaMAPKKK98	TGACG-motif	TGACG	876	5 -	Hordeum vulgare	the MeJA responsive
TaMAPKKK98	TGACG-motif	TGACG	904	5 -	Hordeum vulgare	the MeJA responsive
TaMAPKKK98	TGACG-motif	TGACG	1023	5 -	Hordeum vulgare	the MeJA responsive
TaMAPKKK98	CGTCA-motif	CGTCA	448	5 +	Hordeum vulgare	the MeJA responsive
TaMAPKKK98	CGTCA-motif	CGTCA	482	5 +	Hordeum vulgare	the MeJA responsive
TaMAPKKK98	CGTCA-motif	CGTCA	876	5 +	Hordeum vulgare	the MeJA responsive
TaMAPKKK98	CGTCA-motif	CGTCA	904	5 +	Hordeum vulgare	the MeJA responsive
TaMAPKKK98	CGTCA-motif	CGTCA	1023	5 +	Hordeum vulgare	the MeJA responsive
TaMAPKKK98	CAT-box	GCCACT	562	6 -	Arabidopsis thaliana	meristem expression
TaMAPKKK99	ABRE	ACGTG	261	5 +	Arabidopsis thaliana	abscisic acid responsive
TaMAPKKK99	ABRE	ACGTG	373	5 -	Arabidopsis thaliana	abscisic acid responsive
TaMAPKKK99	ABRE	ACGTG	1098	5 +	Arabidopsis thaliana	abscisic acid responsive
TaMAPKKK99	GARE-motif	TCTGTTG	998	7 -	Brassica oleracea	gibberellin responsive
TaMAPKKK99	TGACG-motif	TGACG	1105	5 -	Hordeum vulgare	the MeJA responsive
TaMAPKKK99	TGACG-motif	TGACG	1129	5 +	Hordeum vulgare	the MeJA responsive
TaMAPKKK99	ARE	AAACCA	431	6 -	Zea mays	anaerobic induction

TaMAPKKK99	ARE	AAACCA	1575	6 +	Zea mays	anaerobic induction
TaMAPKKK99	ARE	AAACCA	1658	6 +	Zea mays	anaerobic induction
TaMAPKKK99	MBS	CAACTG	418	6 +	Arabidopsis thaliana	drought inducibility
TaMAPKKK99	MBS	CAACTG	1213	6 -	Arabidopsis thaliana	drought inducibility
TaMAPKKK99	GC-motif	CCCCCG	117	6 +	Zea mays	anoxic specific inducibility
TaMAPKKK99	GC-motif	CCCCCG	1955	6 +	Zea mays	anoxic specific inducibility
TaMAPKKK99	CGTCA-motif	CGTCA	1105	5 +	Hordeum vulgare	the MeJA responsive
TaMAPKKK99	CGTCA-motif	CGTCA	1129	5 -	Hordeum vulgare	the MeJA responsive
TaMAPKKKK1	TC-rich repeats	ATTCTCTAAC	553	9 -	Nicotiana tabacum	defense and stress responsive
TaMAPKKKK1	TC-rich repeats	GTTTTCTTAC	1124	9 +	Nicotiana tabacum	defense and stress responsive
TaMAPKKKK1	CGTCA-motif	CGTCA	367	5 +	Hordeum vulgare	the MeJA responsive
TaMAPKKKK1	CGTCA-motif	CGTCA	417	5 -	Hordeum vulgare	the MeJA responsive
TaMAPKKKK1	CGTCA-motif	CGTCA	1395	5 +	Hordeum vulgare	the MeJA responsive
TaMAPKKKK1	CAT-box	GCCACT	1270	6 +	Arabidopsis thaliana	meristem expression
TaMAPKKKK1	CAT-box	GCCACT	1284	6 +	Arabidopsis thaliana	meristem expression
TaMAPKKKK1	CAT-box	GCCACT	1299	6 +	Arabidopsis thaliana	meristem expression
TaMAPKKKK1	GC-motif	CCCCCG	1325	6 -	Zea mays	anoxic specific inducibility
TaMAPKKKK1	GC-motif	CCCCCG	1380	6 +	Zea mays	anoxic specific inducibility
TaMAPKKKK1	ABRE	ACGTG	365	5 -	Arabidopsis thaliana	abscisic acid responsive

TaMAPKKKK1	ABRE	TACGGTC	809	7 -	Arabidopsis thaliana	abscisic acid responsive
TaMAPKKKK1	ABRE	ACGTG	1036	5 +	Arabidopsis thaliana	abscisic acid responsive
TaMAPKKKK1	ABRE	ACGTG	1369	5 -	Arabidopsis thaliana	abscisic acid responsive
TaMAPKKKK1	ABRE	ACGTG	1393	5 -	Arabidopsis thaliana	abscisic acid responsive
TaMAPKKKK1	O2-site	GTTGACGTGA	364	9 -	Zea mays	zein metabolism regulation
TaMAPKKKK1	GCN4_motif	TGAGTCA	160	7 -	Oryza sativa	endosperm expression
TaMAPKKKK1	LTR	CCGAAA	1403	6 +	Hordeum vulgare	low temperature responsive
TaMAPKKKK1	TGACG-motif	TGACG	367	5 -	Hordeum vulgare	the MeJA responsive
TaMAPKKKK1	TGACG-motif	TGACG	417	5 +	Hordeum vulgare	the MeJA responsive
TaMAPKKKK1	TGACG-motif	TGACG	1395	5 -	Hordeum vulgare	the MeJA responsive
TaMAPKKKK10	O2-site	GATGACATGG	1228	9 +	Zea mays	zein metabolism regulation
TaMAPKKKK10	TGACG-motif	TGACG	43	5 -	Hordeum vulgare	the MeJA responsive
TaMAPKKKK10	TGACG-motif	TGACG	59	5 -	Hordeum vulgare	the MeJA responsive
TaMAPKKKK10	TGACG-motif	TGACG	985	5 -	Hordeum vulgare	the MeJA responsive
TaMAPKKKK10	CAT-box	GCCACT	486	6 +	Arabidopsis thaliana	meristem expression
TaMAPKKKK10	ABRE	ACGTG	41	5 -	Arabidopsis thaliana	abscisic acid responsive
TaMAPKKKK10	CGTCA-motif	CGTCA	43	5 +	Hordeum vulgare	the MeJA responsive
TaMAPKKKK10	CGTCA-motif	CGTCA	59	5 +	Hordeum vulgare	the MeJA responsive
TaMAPKKKK10	CGTCA-motif	CGTCA	985	5 +	Hordeum vulgare	the MeJA responsive

TaMAPKKKK10	LTR	CCGAAA	1138	6 +	Hordeum vulgare	low temperature responsive
TaMAPKKKK10	LTR	CCGAAA	1386	6 +	Hordeum vulgare	low temperature responsive
TaMAPKKKK10	LTR	CCGAAA	1605	6 -	Hordeum vulgare	low temperature responsive
TaMAPKKKK10	TGA-element	AACGAC	644	6 +	Brassica oleracea	auxin responsive
TaMAPKKKK10	TCA-element	CCATCTTTTT	830	9 -	Nicotiana tabacum	salicylic acid responsive
TaMAPKKKK11	TCA-element	TCAGAAGAGG	1725	9 +	Brassica oleracea	salicylic acid responsive
TaMAPKKKK11	CGTCA-motif	CGTCA	174	5 -	Hordeum vulgare	the MeJA responsive
TaMAPKKKK11	CGTCA-motif	CGTCA	397	5 +	Hordeum vulgare	the MeJA responsive
TaMAPKKKK11	CGTCA-motif	CGTCA	523	5 +	Hordeum vulgare	the MeJA responsive
TaMAPKKKK11	CGTCA-motif	CGTCA	649	5 +	Hordeum vulgare	the MeJA responsive
TaMAPKKKK11	CGTCA-motif	CGTCA	775	5 +	Hordeum vulgare	the MeJA responsive
TaMAPKKKK11	CGTCA-motif	CGTCA	901	5 +	Hordeum vulgare	the MeJA responsive
TaMAPKKKK11	CGTCA-motif	CGTCA	1244	5 -	Hordeum vulgare	the MeJA responsive
TaMAPKKKK11	CGTCA-motif	CGTCA	1551	5 +	Hordeum vulgare	the MeJA responsive
TaMAPKKKK11	CGTCA-motif	CGTCA	1656	5 +	Hordeum vulgare	the MeJA responsive
TaMAPKKKK11	LTR	CCGAAA	110	6 +	Hordeum vulgare	low temperature responsive
TaMAPKKKK11	LTR	CCGAAA	254	6 +	Hordeum vulgare	low temperature responsive
TaMAPKKKK11	TGA-element	AACGAC	1160	6 -	Brassica oleracea	auxin responsive
TaMAPKKKK11	TGA-element	AACGAC	1188	6 -	Brassica oleracea	auxin responsive

TaMAPKKKK11	TGA-element	AACGAC	1943	6 +	Brassica oleracea	auxin responsive
TaMAPKKKK11	ABRE	ACGTG	1387	5 -	Arabidopsis thaliana	abscisic acid responsive
TaMAPKKKK11	ABRE	ACGTG	1879	5 -	Arabidopsis thaliana	abscisic acid responsive
TaMAPKKKK11	CAT-box	GCCACT	1796	6 +	Arabidopsis thaliana	meristem expression
TaMAPKKKK11	TGACG-motif	TGACG	174	5 +	Hordeum vulgare	the MeJA responsive
TaMAPKKKK11	TGACG-motif	TGACG	397	5 -	Hordeum vulgare	the MeJA responsive
TaMAPKKKK11	TGACG-motif	TGACG	523	5 -	Hordeum vulgare	the MeJA responsive
TaMAPKKKK11	TGACG-motif	TGACG	649	5 -	Hordeum vulgare	the MeJA responsive
TaMAPKKKK11	TGACG-motif	TGACG	775	5 -	Hordeum vulgare	the MeJA responsive
TaMAPKKKK11	TGACG-motif	TGACG	901	5 -	Hordeum vulgare	the MeJA responsive
TaMAPKKKK11	TGACG-motif	TGACG	1244	5 +	Hordeum vulgare	the MeJA responsive
TaMAPKKKK11	TGACG-motif	TGACG	1551	5 -	Hordeum vulgare	the MeJA responsive
TaMAPKKKK11	TGACG-motif	TGACG	1656	5 -	Hordeum vulgare	the MeJA responsive
TaMAPKKKK11	GARE-motif	TCTGTTG	1893	7 +	Brassica oleracea	gibberellin responsive
TaMAPKKKK11	GC-motif	CCCCCG	1506	6 -	Zea mays	anoxic specific inducibility
TaMAPKKKK12	P-box	CCTTTTG	813	7 -	Oryza sativa	gibberellin responsive
TaMAPKKKK12	ABRE	ACGTG	836	5 -	Arabidopsis thaliana	abscisic acid responsive
TaMAPKKKK12	ABRE	GCAACGTGTC	876	9 -	Hordeum vulgare	abscisic acid responsive
TaMAPKKKK12	ABRE	ACGTG	878	5 -	Arabidopsis thaliana	abscisic acid responsive

TaMAPKKKK12	LTR	CCGAAA	944	6 +	Hordeum vulgare	low temperature responsive
TaMAPKKKK12	LTR	CCGAAA	1458	6 +	Hordeum vulgare	low temperature responsive
TaMAPKKKK12	TGACG-motif	TGACG	564	5 +	Hordeum vulgare	the MeJA responsive
TaMAPKKKK12	TGACG-motif	TGACG	854	5 -	Hordeum vulgare	the MeJA responsive
TaMAPKKKK12	TGACG-motif	TGACG	1587	5 -	Hordeum vulgare	the MeJA responsive
TaMAPKKKK12	CGTCA-motif	CGTCA	564	5 -	Hordeum vulgare	the MeJA responsive
TaMAPKKKK12	CGTCA-motif	CGTCA	854	5 +	Hordeum vulgare	the MeJA responsive
TaMAPKKKK12	CGTCA-motif	CGTCA	1587	5 +	Hordeum vulgare	the MeJA responsive
TaMAPKKKK12	ARE	AAACCA	344	6 -	Zea mays	anaerobic induction
TaMAPKKKK13	CAT-box	GCCACT	676	6 +	Arabidopsis thaliana	meristem expression
TaMAPKKKK13	CAT-box	GCCACT	1273	6 -	Arabidopsis thaliana	meristem expression
TaMAPKKKK13	TGACG-motif	TGACG	51	5 +	Hordeum vulgare	the MeJA responsive
TaMAPKKKK13	TGACG-motif	TGACG	871	5 -	Hordeum vulgare	the MeJA responsive
TaMAPKKKK13	TGACG-motif	TGACG	1384	5 +	Hordeum vulgare	the MeJA responsive
TaMAPKKKK13	CGTCA-motif	CGTCA	51	5 -	Hordeum vulgare	the MeJA responsive
TaMAPKKKK13	CGTCA-motif	CGTCA	871	5 +	Hordeum vulgare	the MeJA responsive
TaMAPKKKK13	CGTCA-motif	CGTCA	1384	5 -	Hordeum vulgare	the MeJA responsive
TaMAPKKKK13	LTR	CCGAAA	1407	6 -	Hordeum vulgare	low temperature responsive
TaMAPKKKK13	ABRE	ACGTG	53	5 +	Arabidopsis thaliana	abscisic acid responsive

TaMAPKKKK13	ABRE	ACGTG	481	5 +	Arabidopsis thaliana	abscisic acid responsive
TaMAPKKKK13	ABRE	ACGTG	563	5 -	Arabidopsis thaliana	abscisic acid responsive
TaMAPKKKK13	ABRE	ACGTG	726	5 -	Arabidopsis thaliana	abscisic acid responsive
TaMAPKKKK13	ABRE	ACGTG	1034	5 +	Arabidopsis thaliana	abscisic acid responsive
TaMAPKKKK13	ABRE	CACGTG	1332	6 -	Arabidopsis thaliana	abscisic acid responsive
TaMAPKKKK13	ABRE	ACGTG	1333	5 +	Arabidopsis thaliana	abscisic acid responsive
TaMAPKKKK13	ABRE	ACGTG	1504	5 +	Arabidopsis thaliana	abscisic acid responsive
TaMAPKKKK13	TGA-element	AACGAC	896	6 -	Brassica oleracea	auxin responsive
TaMAPKKKK13	MBS	CAACTG	338	6 -	Arabidopsis thaliana	drought inducibility
TaMAPKKKK13	GARE-motif	TCTGTTG	1594	7 +	Brassica oleracea	gibberellin responsive
TaMAPKKKK13	TCA-element	CCATCTTTTT	1732	9 +	Nicotiana tabacum	salicylic acid responsive
TaMAPKKKK13	TCA-element	CCATCTTTTT	1788	9 +	Nicotiana tabacum	salicylic acid responsive
TaMAPKKKK14	LTR	CCGAAA	154	6 +	Hordeum vulgare	low temperature responsive
TaMAPKKKK14	LTR	CCGAAA	1664	6 -	Hordeum vulgare	low temperature responsive
TaMAPKKKK14	LTR	CCGAAA	1678	6 -	Hordeum vulgare	low temperature responsive
TaMAPKKKK14	ABRE	TACGGTC	648	7 +	Arabidopsis thaliana	abscisic acid responsive
TaMAPKKKK14	ABRE	ACGTG	1588	5 +	Arabidopsis thaliana	abscisic acid responsive
TaMAPKKKK15	AuxRR-core	GGTCCAT	1100	7 -	Nicotiana tabacum	auxin responsive
TaMAPKKKK15	CGTCA-motif	CGTCA	113	5 -	Hordeum vulgare	the MeJA responsive

TaMAPKKKK15	CGTCA-motif	CGTCA	907	5 -	Hordeum vulgare	the MeJA responsive
TaMAPKKKK15	CGTCA-motif	CGTCA	951	5 -	Hordeum vulgare	the MeJA responsive
TaMAPKKKK15	TGACG-motif	TGACG	113	5 +	Hordeum vulgare	the MeJA responsive
TaMAPKKKK15	TGACG-motif	TGACG	907	5 +	Hordeum vulgare	the MeJA responsive
TaMAPKKKK15	TGACG-motif	TGACG	951	5 +	Hordeum vulgare	the MeJA responsive
TaMAPKKKK15	LTR	CCGAAA	1405	6 +	Hordeum vulgare	low temperature responsive
TaMAPKKKK15	O2-site	GATGA(C/T)(A/G)TG(A/G)	520	8 -	Zea mays	zein metabolism regulation
TaMAPKKKK15	O2-site	GATGACATGG	1361	9 -	Zea mays	zein metabolism regulation
TaMAPKKKK15	CAT-box	GCCACT	75	6 +	Arabidopsis thaliana	meristem expression
TaMAPKKKK15	circadian	CAAAGATATC	1200	9 +	Lycopersicon esculentum	circadian control
TaMAPKKKK15	MBS	CAACTG	1018	6 -	Arabidopsis thaliana	drought inducibility
TaMAPKKKK15	ABRE	CACGTG	409	6 +	Arabidopsis thaliana	abscisic acid responsive
TaMAPKKKK15	ABRE	ACGTG	410	5 +	Arabidopsis thaliana	abscisic acid responsive
TaMAPKKKK15	GC-motif	CCCCCG	862	6 -	Zea mays	anoxic specific inducibility
TaMAPKKKK15	TGA-element	AACGAC	935	6 -	Brassica oleracea	auxin responsive
TaMAPKKKK15	TGA-element	AACGAC	1598	6 +	Brassica oleracea	auxin responsive
TaMAPKKKK15	TGA-element	AACGAC	1641	6 +	Brassica oleracea	auxin responsive
TaMAPKKKK16	CAT-box	GCCACT	1548	6 +	Arabidopsis thaliana	meristem expression
TaMAPKKKK16	CAT-box	GCCACT	1574	6 +	Arabidopsis thaliana	meristem expression

TaMAPKKKK16	CGTCA-motif	CGTCA	168	5 -	Hordeum vulgare	the MeJA responsive
TaMAPKKKK16	CGTCA-motif	CGTCA	194	5 -	Hordeum vulgare	the MeJA responsive
TaMAPKKKK16	CGTCA-motif	CGTCA	762	5 +	Hordeum vulgare	the MeJA responsive
TaMAPKKKK16	CGTCA-motif	CGTCA	1447	5 -	Hordeum vulgare	the MeJA responsive
TaMAPKKKK16	CGTCA-motif	CGTCA	1891	5 +	Hordeum vulgare	the MeJA responsive
TaMAPKKKK16	TGACG-motif	TGACG	168	5 +	Hordeum vulgare	the MeJA responsive
TaMAPKKKK16	TGACG-motif	TGACG	194	5 +	Hordeum vulgare	the MeJA responsive
TaMAPKKKK16	TGACG-motif	TGACG	762	5 -	Hordeum vulgare	the MeJA responsive
TaMAPKKKK16	TGACG-motif	TGACG	1447	5 +	Hordeum vulgare	the MeJA responsive
TaMAPKKKK16	TGACG-motif	TGACG	1891	5 -	Hordeum vulgare	the MeJA responsive
TaMAPKKKK16	TC-rich repeats	GTTTTCTTAC	990	9 -	Nicotiana tabacum	defense and stress responsive
TaMAPKKKK16	TGA-element	AACGAC	81	6 -	Brassica oleracea	auxin responsive
TaMAPKKKK16	RY-element	CATGCATG	372	8 +	Helianthus annuus	seed specific regulation
TaMAPKKKK16	ABRE	CACGTG	266	6 +	Arabidopsis thaliana	abscisic acid responsive
TaMAPKKKK16	ABRE	ACGTG	267	5 +	Arabidopsis thaliana	abscisic acid responsive
TaMAPKKKK16	ABRE	ACGTG	1347	5 +	Arabidopsis thaliana	abscisic acid responsive
TaMAPKKKK16	TCA-element	TCAGAAGAGG	1590	9 -	Brassica oleracea	salicylic acid responsive
TaMAPKKKK16	MBS	CAACTG	216	6 -	Arabidopsis thaliana	drought inducibility
TaMAPKKKK17	ABRE	ACGTG	71	5 +	Arabidopsis thaliana	abscisic acid responsive

TaMAPKKKK17	CAT-box	GCCACT	272	6 +	Arabidopsis thaliana	meristem expression
TaMAPKKKK17	CAT-box	GCCACT	298	6 +	Arabidopsis thaliana	meristem expression
TaMAPKKKK17	CGTCA-motif	CGTCA	171	5 -	Hordeum vulgare	the MeJA responsive
TaMAPKKKK17	CGTCA-motif	CGTCA	1891	5 +	Hordeum vulgare	the MeJA responsive
TaMAPKKKK17	TCA-element	TCAGAAGAGG	314	9 -	Brassica oleracea	salicylic acid responsive
TaMAPKKKK17	TGACG-motif	TGACG	171	5 +	Hordeum vulgare	the MeJA responsive
TaMAPKKKK17	TGACG-motif	TGACG	1891	5 -	Hordeum vulgare	the MeJA responsive
TaMAPKKKK18	ARE	AAACCA	376	6 -	Zea mays	anaerobic induction
TaMAPKKKK18	TGACG-motif	TGACG	1053	5 -	Hordeum vulgare	the MeJA responsive
TaMAPKKKK18	TGACG-motif	TGACG	1071	5 -	Hordeum vulgare	the MeJA responsive
TaMAPKKKK18	CGTCA-motif	CGTCA	1053	5 +	Hordeum vulgare	the MeJA responsive
TaMAPKKKK18	CGTCA-motif	CGTCA	1071	5 +	Hordeum vulgare	the MeJA responsive
TaMAPKKKK18	LTR	CCGAAA	276	6 +	Hordeum vulgare	low temperature responsive
TaMAPKKKK18	ABRE	ACGTG	480	5 +	Arabidopsis thaliana	abscisic acid responsive
TaMAPKKKK18	ABRE	ACGTG	520	5 +	Arabidopsis thaliana	abscisic acid responsive
TaMAPKKKK18	ABRE	CACGTG	882	6 +	Arabidopsis thaliana	abscisic acid responsive
TaMAPKKKK18	ABRE	ACGTG	883	5 +	Arabidopsis thaliana	abscisic acid responsive
TaMAPKKKK18	GARE-motif	TCTGTTG	1916	7 -	Brassica oleracea	gibberellin responsive
TaMAPKKKK19	ARE	AAACCA	980	6 -	Zea mays	anaerobic induction

TaMAPKKKK19	CAT-box	GCCACT	1699	6 -	Arabidopsis thaliana	meristem expression
TaMAPKKKK19	TGACG-motif	TGACG	218	5 +	Hordeum vulgare	the MeJA responsive
TaMAPKKKK19	TGACG-motif	TGACG	1869	5 -	Hordeum vulgare	the MeJA responsive
TaMAPKKKK19	CGTCA-motif	CGTCA	218	5 -	Hordeum vulgare	the MeJA responsive
TaMAPKKKK19	CGTCA-motif	CGTCA	1869	5 +	Hordeum vulgare	the MeJA responsive
TaMAPKKKK19	LTR	CCGAAA	461	6 -	Hordeum vulgare	low temperature responsive
TaMAPKKKK19	LTR	CCGAAA	1562	6 -	Hordeum vulgare	low temperature responsive
TaMAPKKKK19	ABRE	TACGGTC	17	7 +	Arabidopsis thaliana	abscisic acid responsive
TaMAPKKKK19	ABRE	CACGTG	29	6 +	Arabidopsis thaliana	abscisic acid responsive
TaMAPKKKK19	ABRE	ACGTG	30	5 +	Arabidopsis thaliana	abscisic acid responsive
TaMAPKKKK19	ABRE	ACGTG	429	5 +	Arabidopsis thaliana	abscisic acid responsive
TaMAPKKKK19	ABRE	AACCCGG	1626	7 -	Arabidopsis thaliana	abscisic acid responsive
TaMAPKKKK19	ABRE	GCCGCGTGGC	1695	9 +	Oryza sativa	abscisic acid responsive
TaMAPKKKK19	GC-motif	CCCCCG	1448	6 +	Zea mays	anoxic specific inducibility
TaMAPKKKK19	P-box	CCTTTTG	727	7 +	Oryza sativa	gibberellin responsive
TaMAPKKKK19	P-box	CCTTTTG	1134	7 -	Oryza sativa	gibberellin responsive
TaMAPKKKK2	LTR	CCGAAA	555	6 -	Hordeum vulgare	low temperature responsive
TaMAPKKKK2	LTR	CCGAAA	1721	6 +	Hordeum vulgare	low temperature responsive
TaMAPKKKK2	TGACG-motif	TGACG	1629	5 +	Hordeum vulgare	the MeJA responsive

TaMAPKKKK2	TGACG-motif	TGACG	1713	5 -	Hordeum vulgare	the MeJA responsive
TaMAPKKKK2	TCA-element	CCATCTTTTT	1017	9 +	Nicotiana tabacum	salicylic acid responsive
TaMAPKKKK2	ARE	AAACCA	608	6 +	Zea mays	anaerobic induction
TaMAPKKKK2	ARE	AAACCA	708	6 -	Zea mays	anaerobic induction
TaMAPKKKK2	ARE	AAACCA	1441	6 +	Zea mays	anaerobic induction
TaMAPKKKK2	CAT-box	GCCACT	1589	6 +	Arabidopsis thaliana	meristem expression
TaMAPKKKK2	CAT-box	GCCACT	1603	6 +	Arabidopsis thaliana	meristem expression
TaMAPKKKK2	CGTCA-motif	CGTCA	1629	5 -	Hordeum vulgare	the MeJA responsive
TaMAPKKKK2	CGTCA-motif	CGTCA	1713	5 +	Hordeum vulgare	the MeJA responsive
TaMAPKKKK2	TGA-element	AACGAC	696	6 -	Brassica oleracea	auxin responsive
TaMAPKKKK2	ABRE	GACACGTGGC	619	9 +	Triticum aestivum	abscisic acid responsive
TaMAPKKKK2	ABRE	ACGTG	1312	5 +	Arabidopsis thaliana	abscisic acid responsive
TaMAPKKKK2	ABRE	ACGTG	1689	5 -	Arabidopsis thaliana	abscisic acid responsive
TaMAPKKKK2	ABRE	ACGTG	1711	5 -	Arabidopsis thaliana	abscisic acid responsive
TaMAPKKKK2	GC-motif	CCCCCG	1218	6 +	Zea mays	anoxic specific inducibility
TaMAPKKKK2	GC-motif	CCCCCG	1645	6 -	Zea mays	anoxic specific inducibility
TaMAPKKKK2	GC-motif	CCCCCG	1704	6 +	Zea mays	anoxic specific inducibility
TaMAPKKKK20	LTR	CCGAAA	44	6 +	Hordeum vulgare	low temperature responsive
TaMAPKKKK20	LTR	CCGAAA	163	6 +	Hordeum vulgare	low temperature responsive

TaMAPKKKK20	LTR	CCGAAA	264	6 -	Hordeum vulgare	low temperature responsive
TaMAPKKKK20	LTR	CCGAAA	395	6 +	Hordeum vulgare	low temperature responsive
TaMAPKKKK20	LTR	CCGAAA	1128	6 -	Hordeum vulgare	low temperature responsive
TaMAPKKKK20	TC-rich repeats	GTTTTCTTAC	1205	9 +	Nicotiana tabacum	defense and stress responsive
TaMAPKKKK20	CAT-box	GCCACT	806	6 +	Arabidopsis thaliana	meristem expression
TaMAPKKKK20	CAT-box	GCCACT	1661	6 -	Arabidopsis thaliana	meristem expression
TaMAPKKKK20	ABRE	ACGTG	580	5 +	Arabidopsis thaliana	abscisic acid responsive
TaMAPKKKK20	ABRE	CACGTG	1294	6 -	Arabidopsis thaliana	abscisic acid responsive
TaMAPKKKK20	ABRE	ACGTG	1295	5 +	Arabidopsis thaliana	abscisic acid responsive
TaMAPKKKK20	ABRE	GCCGCGTGGC	1657	9 +	Oryza sativa	abscisic acid responsive
TaMAPKKKK20	ARE	AAACCA	541	6 -	Zea mays	anaerobic induction
TaMAPKKKK20	TGACG-motif	TGACG	1277	5 +	Hordeum vulgare	the MeJA responsive
TaMAPKKKK20	TGACG-motif	TGACG	1871	5 -	Hordeum vulgare	the MeJA responsive
TaMAPKKKK20	CGTCA-motif	CGTCA	1277	5 -	Hordeum vulgare	the MeJA responsive
TaMAPKKKK20	CGTCA-motif	CGTCA	1871	5 +	Hordeum vulgare	the MeJA responsive
TaMAPKKKK21	CAT-box	GCCACT	799	6 -	Arabidopsis thaliana	meristem expression
TaMAPKKKK21	MBS	CAACTG	91	6 -	Arabidopsis thaliana	drought inducibility
TaMAPKKKK21	MBS	CAACTG	1024	6 -	Arabidopsis thaliana	drought inducibility
TaMAPKKKK21	TC-rich repeats	GTTTTCTTAC	258	9 +	Nicotiana tabacum	defense and stress responsive

TaMAPKKKK21	CGTCA-motif	CGTCA	923	5 -	Hordeum vulgare	the MeJA responsive
TaMAPKKKK21	TGACG-motif	TGACG	923	5 +	Hordeum vulgare	the MeJA responsive
TaMAPKKKK21	TCA-element	CCATCTTTTT	1737	9 +	Nicotiana tabacum	salicylic acid responsive
TaMAPKKKK21	circadian	CAAAGATATC	480	9 +	Lycopersicon esculentum	circadian control
TaMAPKKKK21	TGA-element	AACGAC	1450	6 -	Brassica oleracea	auxin responsive
TaMAPKKKK21	TGA-element	AACGAC	1483	6 -	Brassica oleracea	auxin responsive
TaMAPKKKK21	TGA-element	AACGAC	1498	6 -	Brassica oleracea	auxin responsive
TaMAPKKKK21	TGA-element	AACGAC	1507	6 -	Brassica oleracea	auxin responsive
TaMAPKKKK21	ARE	AAACCA	1329	6 -	Zea mays	anaerobic induction
TaMAPKKKK22	MBS	CAACTG	94	6 +	Arabidopsis thaliana	drought inducibility
TaMAPKKKK22	P-box	CCTTTTG	572	7 +	Oryza sativa	gibberellin responsive
TaMAPKKKK22	P-box	CCTTTTG	589	7 +	Oryza sativa	gibberellin responsive
TaMAPKKKK22	TGACG-motif	TGACG	196	5 -	Hordeum vulgare	the MeJA responsive
TaMAPKKKK22	TGACG-motif	TGACG	1979	5 +	Hordeum vulgare	the MeJA responsive
TaMAPKKKK22	CGTCA-motif	CGTCA	196	5 +	Hordeum vulgare	the MeJA responsive
TaMAPKKKK22	CGTCA-motif	CGTCA	1979	5 -	Hordeum vulgare	the MeJA responsive
TaMAPKKKK22	GC-motif	CCCCCG	828	6 +	Zea mays	anoxic specific inducibility
TaMAPKKKK22	GC-motif	CCCCCG	977	6 +	Zea mays	anoxic specific inducibility
TaMAPKKKK22	ABRE	ACGTG	130	5 +	Arabidopsis thaliana	abscisic acid responsive

TaMAPKKKK22	ABRE	ACGTG	283	5 -	Arabidopsis thaliana	abscisic acid responsive
TaMAPKKKK22	ABRE	AACCCGG	1083	7 -	Arabidopsis thaliana	abscisic acid responsive
TaMAPKKKK22	TGA-element	AACGAC	308	6 -	Brassica oleracea	auxin responsive
TaMAPKKKK22	ARE	AAACCA	694	6 +	Zea mays	anaerobic induction
TaMAPKKKK22	ARE	AAACCA	1888	6 -	Zea mays	anaerobic induction
TaMAPKKKK23	GC-motif	CCCCCG	828	6 +	Zea mays	anoxic specific inducibility
TaMAPKKKK23	GC-motif	CCCCCG	980	6 +	Zea mays	anoxic specific inducibility
TaMAPKKKK23	ARE	AAACCA	181	6 -	Zea mays	anaerobic induction
TaMAPKKKK23	ARE	AAACCA	702	6 +	Zea mays	anaerobic induction
TaMAPKKKK23	ARE	AAACCA	1549	6 -	Zea mays	anaerobic induction
TaMAPKKKK23	ABRE	ACGTG	17	5 +	Arabidopsis thaliana	abscisic acid responsive
TaMAPKKKK23	ABRE	AACCCGG	1085	7 -	Arabidopsis thaliana	abscisic acid responsive
TaMAPKKKK23	ABRE	CGCACGTGTC	1374	9 +	Hordeum vulgare	abscisic acid responsive
TaMAPKKKK23	ABRE	ACGTG	1675	5 +	Arabidopsis thaliana	abscisic acid responsive
TaMAPKKKK23	P-box	CCTTTTG	580	7 +	Oryza sativa	gibberellin responsive
TaMAPKKKK23	P-box	CCTTTTG	597	7 +	Oryza sativa	gibberellin responsive
TaMAPKKKK23	TGACG-motif	TGACG	204	5 -	Hordeum vulgare	the MeJA responsive
TaMAPKKKK23	TGACG-motif	TGACG	1979	5 +	Hordeum vulgare	the MeJA responsive
TaMAPKKKK23	CGTCA-motif	CGTCA	204	5 +	Hordeum vulgare	the MeJA responsive

TaMAPKKKK23	CGTCA-motif	CGTCA	1979	5 -	Hordeum vulgare	the MeJA responsive
TaMAPKKKK24	CAT-box	GCCACT	961	6 +	Arabidopsis thaliana	meristem expression
TaMAPKKKK24	GC-motif	CCCCCG	852	6 +	Zea mays	anoxic specific inducibility
TaMAPKKKK24	GC-motif	CCCCCG	997	6 +	Zea mays	anoxic specific inducibility
TaMAPKKKK24	TCA-element	CCATCTTTTT	99	9 -	Nicotiana tabacum	salicylic acid responsive
TaMAPKKKK24	TGA-element	AACGAC	413	6 -	Brassica oleracea	auxin responsive
TaMAPKKKK24	ABRE	ACGTG	299	5 -	Arabidopsis thaliana	abscisic acid responsive
TaMAPKKKK24	ABRE	ACGTG	388	5 -	Arabidopsis thaliana	abscisic acid responsive
TaMAPKKKK24	ABRE	AACCCGG	1102	7 -	Arabidopsis thaliana	abscisic acid responsive
TaMAPKKKK24	ARE	AAACCA	726	6 +	Zea mays	anaerobic induction
TaMAPKKKK24	P-box	CCTTTTG	604	7 +	Oryza sativa	gibberellin responsive
TaMAPKKKK24	P-box	CCTTTTG	621	7 +	Oryza sativa	gibberellin responsive
TaMAPKKKK24	TGACG-motif	TGACG	1979	5 +	Hordeum vulgare	the MeJA responsive
TaMAPKKKK24	CGTCA-motif	CGTCA	1979	5 -	Hordeum vulgare	the MeJA responsive
TaMAPKKKK25	LTR	CCGAAA	1203	6 +	Hordeum vulgare	low temperature responsive
TaMAPKKKK25	LTR	CCGAAA	1372	6 -	Hordeum vulgare	low temperature responsive
TaMAPKKKK25	LTR	CCGAAA	1394	6 -	Hordeum vulgare	low temperature responsive
TaMAPKKKK25	O2-site	GATGATGTGG	1457	9 +	Zea mays	zein metabolism regulation
TaMAPKKKK25	GC-motif	CCCCCG	1005	6 +	Zea mays	anoxic specific inducibility

TaMAPKKKK25	GC-motif	CCCCCG	1646	6 -	Zea mays	anoxic specific inducibility
TaMAPKKKK25	ABRE	AACCCGG	220	7 +	Arabidopsis thaliana	abscisic acid responsive
TaMAPKKKK25	ABRE	ACGTG	691	5 +	Arabidopsis thaliana	abscisic acid responsive
TaMAPKKKK25	ABRE	GCCGCGTGGC	1685	9 -	Oryza sativa	abscisic acid responsive
TaMAPKKKK25	ABRE	ACGTG	1796	5 -	Arabidopsis thaliana	abscisic acid responsive
TaMAPKKKK25	TGA-element	AACGAC	1933	6 +	Brassica oleracea	auxin responsive
TaMAPKKKK25	ARE	AAACCA	147	6 +	Zea mays	anaerobic induction
TaMAPKKKK25	TGACG-motif	TGACG	198	5 -	Hordeum vulgare	the MeJA responsive
TaMAPKKKK25	TGACG-motif	TGACG	259	5 -	Hordeum vulgare	the MeJA responsive
TaMAPKKKK25	TGACG-motif	TGACG	306	5 -	Hordeum vulgare	the MeJA responsive
TaMAPKKKK25	TGACG-motif	TGACG	449	5 +	Hordeum vulgare	the MeJA responsive
TaMAPKKKK25	TGACG-motif	TGACG	1048	5 -	Hordeum vulgare	the MeJA responsive
TaMAPKKKK25	TGACG-motif	TGACG	1163	5 -	Hordeum vulgare	the MeJA responsive
TaMAPKKKK25	TGACG-motif	TGACG	1217	5 -	Hordeum vulgare	the MeJA responsive
TaMAPKKKK25	CGTCA-motif	CGTCA	198	5 +	Hordeum vulgare	the MeJA responsive
TaMAPKKKK25	CGTCA-motif	CGTCA	259	5 +	Hordeum vulgare	the MeJA responsive
TaMAPKKKK25	CGTCA-motif	CGTCA	306	5 +	Hordeum vulgare	the MeJA responsive
TaMAPKKKK25	CGTCA-motif	CGTCA	449	5 -	Hordeum vulgare	the MeJA responsive
TaMAPKKKK25	CGTCA-motif	CGTCA	1048	5 +	Hordeum vulgare	the MeJA responsive

TaMAPKKKK25	CGTCA-motif	CGTCA	1163	5 +	Hordeum vulgare	the MeJA responsive
TaMAPKKKK25	CGTCA-motif	CGTCA	1217	5 +	Hordeum vulgare	the MeJA responsive
TaMAPKKKK3	TATC-box	TATCCCA	95	7 +	Oryza sativa	gibberellin responsive
TaMAPKKKK3	LTR	CCGAAA	1720	6 +	Hordeum vulgare	low temperature responsive
TaMAPKKKK3	GCN4_motif	TGAGTCA	479	7 -	Oryza sativa	endosperm expression
TaMAPKKKK3	O2-site	GTTGACGTGA	145	9 -	Zea mays	zein metabolism regulation
TaMAPKKKK3	O2-site	GTTGACGTGA	683	9 -	Zea mays	zein metabolism regulation
TaMAPKKKK3	TGACG-motif	TGACG	686	5 -	Hordeum vulgare	the MeJA responsive
TaMAPKKKK3	TGACG-motif	TGACG	736	5 +	Hordeum vulgare	the MeJA responsive
TaMAPKKKK3	TGACG-motif	TGACG	1112	5 +	Hordeum vulgare	the MeJA responsive
TaMAPKKKK3	TGACG-motif	TGACG	1712	5 -	Hordeum vulgare	the MeJA responsive
TaMAPKKKK3	MBSI	TTTTTACGGTTA	1178	11 +	Petunia hybrida	flavonoid biosynthetic genes regulation
TaMAPKKKK3	ARE	AAACCA	192	6 +	Zea mays	anaerobic induction
TaMAPKKKK3	TC-rich repeats	ATTCTCTAAC	871	9 -	Nicotiana tabacum	defense and stress responsive
TaMAPKKKK3	TC-rich repeats	ATTCTCTAAC	886	9 +	Nicotiana tabacum	defense and stress responsive
TaMAPKKKK3	TC-rich repeats	GTTTTCTTAC	1439	9 +	Nicotiana tabacum	defense and stress responsive
TaMAPKKKK3	RY-element	CATGCATG	103	8 +	Helianthus annuus	seed specific regulation
TaMAPKKKK3	RY-element	CATGCATG	196	8 +	Helianthus annuus	seed specific regulation

TaMAPKKKK3	CGTCA-motif	CGTCA	686	5 +	Hordeum vulgare	the MeJA responsive
TaMAPKKKK3	CGTCA-motif	CGTCA	736	5 -	Hordeum vulgare	the MeJA responsive
TaMAPKKKK3	CGTCA-motif	CGTCA	1112	5 -	Hordeum vulgare	the MeJA responsive
TaMAPKKKK3	CGTCA-motif	CGTCA	1712	5 +	Hordeum vulgare	the MeJA responsive
TaMAPKKKK3	CAT-box	GCCACT	936	6 -	Arabidopsis thaliana	meristem expression
TaMAPKKKK3	CAT-box	GCCACT	1587	6 +	Arabidopsis thaliana	meristem expression
TaMAPKKKK3	CAT-box	GCCACT	1601	6 +	Arabidopsis thaliana	meristem expression
TaMAPKKKK3	CAT-box	GCCACT	1617	6 +	Arabidopsis thaliana	meristem expression
TaMAPKKKK3	GC-motif	CCCCCG	1643	6 -	Zea mays	anoxic specific inducibility
TaMAPKKKK3	GC-motif	CCCCCG	1697	6 +	Zea mays	anoxic specific inducibility
TaMAPKKKK3	GC-motif	CCCCCG	1703	6 +	Zea mays	anoxic specific inducibility
TaMAPKKKK3	ABRE	CACGTG	351	6 +	Arabidopsis thaliana	abscisic acid responsive
TaMAPKKKK3	ABRE	ACGTG	352	5 +	Arabidopsis thaliana	abscisic acid responsive
TaMAPKKKK3	ABRE	ACGTG	501	5 +	Arabidopsis thaliana	abscisic acid responsive
TaMAPKKKK3	ABRE	ACGTG	684	5 -	Arabidopsis thaliana	abscisic acid responsive
TaMAPKKKK3	ABRE	CACGTG	1084	6 -	Arabidopsis thaliana	abscisic acid responsive
TaMAPKKKK3	ABRE	ACGTG	1085	5 +	Arabidopsis thaliana	abscisic acid responsive
TaMAPKKKK3	ABRE	ACGTG	1109	5 +	Arabidopsis thaliana	abscisic acid responsive
TaMAPKKKK3	ABRE	ACGTG	1351	5 +	Arabidopsis thaliana	abscisic acid responsive

TaMAPKKKK3	ABRE	ACGTG	1687	5 -	Arabidopsis thaliana	abscisic acid responsive
TaMAPKKKK3	ABRE	ACGTG	1710	5 -	Arabidopsis thaliana	abscisic acid responsive
TaMAPKKKK4	P-box	CCTTTTG	458	7 -	Oryza sativa	gibberellin responsive
TaMAPKKKK4	P-box	CCTTTTG	506	7 -	Oryza sativa	gibberellin responsive
TaMAPKKKK4	P-box	CCTTTTG	1106	7 -	Oryza sativa	gibberellin responsive
TaMAPKKKK4	ABRE	ACGTG	153	5 +	Arabidopsis thaliana	abscisic acid responsive
TaMAPKKKK4	ABRE	ACGTG	601	5 -	Arabidopsis thaliana	abscisic acid responsive
TaMAPKKKK4	ABRE	ACGTG	661	5 -	Arabidopsis thaliana	abscisic acid responsive
TaMAPKKKK4	ABRE	CACGTG	996	6 +	Arabidopsis thaliana	abscisic acid responsive
TaMAPKKKK4	ABRE	ACGTG	997	5 +	Arabidopsis thaliana	abscisic acid responsive
TaMAPKKKK4	ABRE	ACGTG	1276	5 +	Arabidopsis thaliana	abscisic acid responsive
TaMAPKKKK4	ABRE	ACGTG	1589	5 +	Arabidopsis thaliana	abscisic acid responsive
TaMAPKKKK4	ABRE	GCCGCGTGGC	1752	9 -	Oryza sativa	abscisic acid responsive
TaMAPKKKK4	CAT-box	GCCACT	1336	6 -	Arabidopsis thaliana	meristem expression
TaMAPKKKK4	CAT-box	GCCACT	1774	6 +	Arabidopsis thaliana	meristem expression
TaMAPKKKK4	CGTCA-motif	CGTCA	151	5 -	Hordeum vulgare	the MeJA responsive
TaMAPKKKK4	CGTCA-motif	CGTCA	169	5 +	Hordeum vulgare	the MeJA responsive
TaMAPKKKK4	MBS	CAACTG	1561	6 -	Arabidopsis thaliana	drought inducibility
TaMAPKKKK4	ARE	AAACCA	419	6 -	Zea mays	anaerobic induction

TaMAPKKKK4	ARE	AAACCA	898	6 +	Zea mays	anaerobic induction
TaMAPKKKK4	ARE	AAACCA	1518	6 -	Zea mays	anaerobic induction
TaMAPKKKK4	ARE	AAACCA	1799	6 +	Zea mays	anaerobic induction
TaMAPKKKK4	ARE	AAACCA	1844	6 +	Zea mays	anaerobic induction
TaMAPKKKK4	TGACG-motif	TGACG	151	5 +	Hordeum vulgare	the MeJA responsive
TaMAPKKKK4	TGACG-motif	TGACG	169	5 -	Hordeum vulgare	the MeJA responsive
TaMAPKKKK4	TCA-element	CCATCTTTTT	1350	9 -	Nicotiana tabacum	salicylic acid responsive
TaMAPKKKK4	O2-site	GTTGACGTGA	923	9 +	Zea mays	zein metabolism regulation
TaMAPKKKK4	LTR	CCGAAA	1053	6 -	Hordeum vulgare	low temperature responsive
TaMAPKKKK4	circadian	CAAAGATATC	1533	9 -	Lycopersicon esculentum	circadian control
TaMAPKKKK5	LTR	CCGAAA	1488	6 -	Hordeum vulgare	low temperature responsive
TaMAPKKKK5	TGACG-motif	TGACG	477	5 +	Hordeum vulgare	the MeJA responsive
TaMAPKKKK5	TGACG-motif	TGACG	675	5 -	Hordeum vulgare	the MeJA responsive
TaMAPKKKK5	ARE	AAACCA	1799	6 +	Zea mays	anaerobic induction
TaMAPKKKK5	MBS	CAACTG	343	6 +	Arabidopsis thaliana	drought inducibility
TaMAPKKKK5	CGTCA-motif	CGTCA	477	5 -	Hordeum vulgare	the MeJA responsive
TaMAPKKKK5	CGTCA-motif	CGTCA	675	5 +	Hordeum vulgare	the MeJA responsive
TaMAPKKKK5	CAT-box	GCCACT	252	6 +	Arabidopsis thaliana	meristem expression
TaMAPKKKK5	CAT-box	GCCACT	1774	6 +	Arabidopsis thaliana	meristem expression

TaMAPKKKK5	GC-motif	CCCCCG	1894	6 +	Zea mays	anoxic specific inducibility
TaMAPKKKK5	ABRE	ACGTG	18	5 -	Arabidopsis thaliana	abscisic acid responsive
TaMAPKKKK5	ABRE	ACGTG	624	5 +	Arabidopsis thaliana	abscisic acid responsive
TaMAPKKKK5	ABRE	AACCCGG	659	7 +	Arabidopsis thaliana	abscisic acid responsive
TaMAPKKKK5	ABRE	ACGTG	1070	5 -	Arabidopsis thaliana	abscisic acid responsive
TaMAPKKKK5	ABRE	GCCGCGTGGC	1752	9 -	Oryza sativa	abscisic acid responsive
TaMAPKKKK6	O2-site	GTTGACGTGA	893	9 +	Zea mays	zein metabolism regulation
TaMAPKKKK6	P-box	CCTTTTG	229	7 -	Oryza sativa	gibberellin responsive
TaMAPKKKK6	P-box	CCTTTTG	277	7 -	Oryza sativa	gibberellin responsive
TaMAPKKKK6	P-box	CCTTTTG	1076	7 -	Oryza sativa	gibberellin responsive
TaMAPKKKK6	LTR	CCGAAA	1023	6 -	Hordeum vulgare	low temperature responsive
TaMAPKKKK6	CGTCA-motif	CGTCA	900	5 -	Hordeum vulgare	the MeJA responsive
TaMAPKKKK6	CGTCA-motif	CGTCA	1483	5 +	Hordeum vulgare	the MeJA responsive
TaMAPKKKK6	CGTCA-motif	CGTCA	1881	5 -	Hordeum vulgare	the MeJA responsive
TaMAPKKKK6	TGA-element	AACGAC	401	6 +	Brassica oleracea	auxin responsive
TaMAPKKKK6	TGA-element	AACGAC	1333	6 +	Brassica oleracea	auxin responsive
TaMAPKKKK6	ABRE	CACGTG	966	6 +	Arabidopsis thaliana	abscisic acid responsive
TaMAPKKKK6	ABRE	ACGTG	967	5 +	Arabidopsis thaliana	abscisic acid responsive
TaMAPKKKK6	ABRE	CACGTG	1006	6 -	Arabidopsis thaliana	abscisic acid responsive
TaMAPKKKK6	ABRE	ACGTG	1007	5 +	Arabidopsis thaliana	abscisic acid responsive

TaMAPKKKK6	ABRE	ACGTG	1635	5 +	Arabidopsis thaliana	abscisic acid responsive
TaMAPKKKK6	ABRE	GCCGCGTGGC	1752	9 -	Oryza sativa	abscisic acid responsive
TaMAPKKKK6	CAT-box	GCCACT	426	6 -	Arabidopsis thaliana	meristem expression
TaMAPKKKK6	CAT-box	GCCACT	608	6 +	Arabidopsis thaliana	meristem expression
TaMAPKKKK6	CAT-box	GCCACT	731	6 +	Arabidopsis thaliana	meristem expression
TaMAPKKKK6	CAT-box	GCCACT	1305	6 -	Arabidopsis thaliana	meristem expression
TaMAPKKKK6	CAT-box	GCCACT	1774	6 +	Arabidopsis thaliana	meristem expression
TaMAPKKKK6	ARE	AAACCA	514	6 +	Zea mays	anaerobic induction
TaMAPKKKK6	ARE	AAACCA	1799	6 +	Zea mays	anaerobic induction
TaMAPKKKK6	TGACG-motif	TGACG	900	5 +	Hordeum vulgare	the MeJA responsive
TaMAPKKKK6	TGACG-motif	TGACG	1483	5 -	Hordeum vulgare	the MeJA responsive
TaMAPKKKK6	TGACG-motif	TGACG	1881	5 +	Hordeum vulgare	the MeJA responsive
TaMAPKKKK7	TGACG-motif	TGACG	753	5 +	Hordeum vulgare	the MeJA responsive
TaMAPKKKK7	TGACG-motif	TGACG	1472	5 -	Hordeum vulgare	the MeJA responsive
TaMAPKKKK7	TGACG-motif	TGACG	1496	5 -	Hordeum vulgare	the MeJA responsive
TaMAPKKKK7	GARE-motif	TCTGTTG	684	7 +	Brassica oleracea	gibberellin responsive
TaMAPKKKK7	ARE	AAACCA	554	6 -	Zea mays	anaerobic induction
TaMAPKKKK7	ARE	AAACCA	1192	6 +	Zea mays	anaerobic induction
TaMAPKKKK7	GC-motif	CCCCCG	247	6 +	Zea mays	anoxic specific inducibility

TaMAPKKKK7	GC-motif	CCCCCG	1362	6 +	Zea mays	anoxic specific inducibility
TaMAPKKKK7	CAT-box	GCCACT	366	6 -	Arabidopsis thaliana	meristem expression
TaMAPKKKK7	CAT-box	GCCACT	1440	6 -	Arabidopsis thaliana	meristem expression
TaMAPKKKK7	P-box	CCTTTTG	648	7 +	Oryza sativa	gibberellin responsive
TaMAPKKKK7	CGTCA-motif	CGTCA	753	5 -	Hordeum vulgare	the MeJA responsive
TaMAPKKKK7	CGTCA-motif	CGTCA	1472	5 +	Hordeum vulgare	the MeJA responsive
TaMAPKKKK7	CGTCA-motif	CGTCA	1496	5 +	Hordeum vulgare	the MeJA responsive
TaMAPKKKK7	TGA-element	AACGAC	565	6 -	Brassica oleracea	auxin responsive
TaMAPKKKK7	TCA-element	CCATCTTTTT	1573	9 -	Nicotiana tabacum	salicylic acid responsive
TaMAPKKKK8	TGACG-motif	TGACG	963	5 -	Hordeum vulgare	the MeJA responsive
TaMAPKKKK8	TGA-element	AACGAC	1355	6 +	Brassica oleracea	auxin responsive
TaMAPKKKK8	CGTCA-motif	CGTCA	963	5 +	Hordeum vulgare	the MeJA responsive
TaMAPKKKK8	ABRE	ACGTG	332	5 -	Arabidopsis thaliana	abscisic acid responsive
TaMAPKKKK8	ABRE	ACGTG	348	5 -	Arabidopsis thaliana	abscisic acid responsive
TaMAPKKKK8	ABRE	ACGTG	481	5 +	Arabidopsis thaliana	abscisic acid responsive
TaMAPKKKK8	ABRE	ACGTG	535	5 -	Arabidopsis thaliana	abscisic acid responsive
TaMAPKKKK8	ABRE	CACGTG	599	6 +	Arabidopsis thaliana	abscisic acid responsive
TaMAPKKKK8	ABRE	ACGTG	600	5 +	Arabidopsis thaliana	abscisic acid responsive
TaMAPKKKK8	ABRE	ACGTG	1103	5 -	Arabidopsis thaliana	abscisic acid responsive
TaMAPKKKK8	ABRE	ACGTG	1594	5 -	Arabidopsis thaliana	abscisic acid responsive

TaMAPKKKK8	TATC-box	TATCCCA	1468	7 -	Oryza sativa	gibberellin responsive
TaMAPKKKK9	MBSI	aaaAaaC(G/C)GTTA	865	10.5 +	Petunia hybrida	flavonoid biosynthetic genes regulation
TaMAPKKKK9	MBS	CAACTG	39	6 -	Arabidopsis thaliana	drought inducibility
TaMAPKKKK9	GARE-motif	TCTGTTG	1604	7 +	Brassica oleracea	gibberellin responsive
TaMAPKKKK9	O2-site	GTTGACGTGA	1248	9 +	Zea mays	zein metabolism regulation
TaMAPKKKK9	TGACG-motif	TGACG	564	5 -	Hordeum vulgare	the MeJA responsive
TaMAPKKKK9	TGACG-motif	TGACG	1250	5 +	Hordeum vulgare	the MeJA responsive
TaMAPKKKK9	TGACG-motif	TGACG	1302	5 +	Hordeum vulgare	the MeJA responsive
TaMAPKKKK9	ARE	AAACCA	43	6 -	Zea mays	anaerobic induction
TaMAPKKKK9	TATC-box	TATCCCA	1558	7 -	Oryza sativa	gibberellin responsive
TaMAPKKKK9	TCA-element	CCATCTTTTT	1738	9 +	Nicotiana tabacum	salicylic acid responsive
TaMAPKKKK9	TCA-element	CCATCTTTTT	1789	9 +	Nicotiana tabacum	salicylic acid responsive
TaMAPKKKK9	ABRE	ACGTG	729	5 +	Arabidopsis thaliana	abscisic acid responsive
TaMAPKKKK9	ABRE	ACGTG	1252	5 +	Arabidopsis thaliana	abscisic acid responsive
TaMAPKKKK9	ABRE	GACACGTACGT	1920	10 +	Oryza sativa	abscisic acid responsive
TaMAPKKKK9	CGTCA-motif	CGTCA	564	5 +	Hordeum vulgare	the MeJA responsive
TaMAPKKKK9	CGTCA-motif	CGTCA	1250	5 -	Hordeum vulgare	the MeJA responsive
TaMAPKKKK9	CGTCA-motif	CGTCA	1302	5 -	Hordeum vulgare	the MeJA responsive

Table S4 Parameters for MAPK-MAP4K protein interaction analysis in wheat			
degree.layo	name	selected	shared name
75	TaMAPKK11	FALSE	TaMAPKK11
74	TaMAPKK16	FALSE	TaMAPKK16
73	TaMAPKK15	FALSE	TaMAPKK15
73	TaMAPKK14	FALSE	TaMAPKK14
72	TaMAPKK5	FALSE	TaMAPKK5
71	TaMAPKK12	FALSE	TaMAPKK12
70	TaMAPKK8	FALSE	TaMAPKK8
69	TaMAPKK9	FALSE	TaMAPKK9
67	TaMAPKK17	FALSE	TaMAPKK17
67	TaMAPKK7	FALSE	TaMAPKK7
66	TaMAPKK6	FALSE	TaMAPKK6
61	TaMAPKK1	FALSE	TaMAPKK1
58	TaMEKK29	FALSE	TaMEKK29
58	TaMEKK17	FALSE	TaMEKK17
55	TaMEKK24	FALSE	TaMEKK24
54	TaMAPKK18	FALSE	TaMAPKK18
54	TaMAPKK2	FALSE	TaMAPKK2
54	TaMAPKK3	FALSE	TaMAPKK3
53	TaMAPKK4	FALSE	TaMAPKK4
53	TaMAPKK13	FALSE	TaMAPKK13
39	TaMEKK16	FALSE	TaMEKK16
39	TaRaf87	FALSE	TaRaf87
38	TaMEKK5	FALSE	TaMEKK5
38	TaMEKK14	FALSE	TaMEKK14
38	TaMEKK15	FALSE	TaMEKK15
38	TaMEKK11	FALSE	TaMEKK11
37	TaMEKK2	FALSE	TaMEKK2
35	TaMEKK1	FALSE	TaMEKK1
34	TaMEKK4	FALSE	TaMEKK4
34	TaMEKK4-1	FALSE	TaMEKK4-1
33	TaMAPK47	FALSE	TaMAPK47
33	TaMAPK25	FALSE	TaMAPK25
33	TaMAPK3	FALSE	TaMAPK3
33	TaMAPKKKK4	FALSE	TaMAPKKKK4
33	TaMAPKKKK5	FALSE	TaMAPKKKK5
33	TaMAPKKKK6	FALSE	TaMAPKKKK6
32	TaMAPK12	FALSE	TaMAPK12
31	TaMAPK35	FALSE	TaMAPK35
30	TaMAPK17	FALSE	TaMAPK17
30	TaMAPK34	FALSE	TaMAPK34
30	TaMAPK44	FALSE	TaMAPK44
29	TaMAPK19	FALSE	TaMAPK19
29	TaMAPK6	FALSE	TaMAPK6
29	TaMAPK20	FALSE	TaMAPK20
29	TaMAPK29	FALSE	TaMAPK29
29	TaMAPK33	FALSE	TaMAPK33
29	TaMAPK39	FALSE	TaMAPK39
29	TaMAPK40	FALSE	TaMAPK40
29	TaMAPK41	FALSE	TaMAPK41
29	TaMAPK49	FALSE	TaMAPK49
29	TaMAPK53	FALSE	TaMAPK53
28	TaMAPK10	FALSE	TaMAPK10
28	TaMAPK14	FALSE	TaMAPK14
28	TaMAPK28	FALSE	TaMAPK28

28	TaMAPK24	FALSE	TaMAPK24
28	TaMAPK43	FALSE	TaMAPK43
28	TaMAPKKKK10	FALSE	TaMAPKKKK10
28	TaMAPKKKK12	FALSE	TaMAPKKKK12
28	TaMAPKKKK14	FALSE	TaMAPKKKK14
27	TaMAPK22	FALSE	TaMAPK22
27	TaMAPK54	FALSE	TaMAPK54
27	TaMAPK11	FALSE	TaMAPK11
27	TaMAPK16	FALSE	TaMAPK16
27	TaRaf30	FALSE	TaRaf30
27	TaMAPK23	FALSE	TaMAPK23
27	TaMAPK27	FALSE	TaMAPK27
27	TaMAPK30	FALSE	TaMAPK30
27	TaMAPK38	FALSE	TaMAPK38
27	TaMAPK4	FALSE	TaMAPK4
27	TaMAPK42	FALSE	TaMAPK42
27	TaMAPK5	FALSE	TaMAPK5
27	TaMAPK52	FALSE	TaMAPK52
27	TaMAPK7	FALSE	TaMAPK7
27	TaMAPK8	FALSE	TaMAPK8
25	TaRaf88	FALSE	TaRaf88
24	TaMEKK9	FALSE	TaMEKK9
23	TaMAPK36	FALSE	TaMAPK36
23	TaMEKK7	FALSE	TaMEKK7
23	TaMEKK20	FALSE	TaMEKK20
23	TaMEKK18	FALSE	TaMEKK18
23	TaMEKK8	FALSE	TaMEKK8
22	TaMAPK45	FALSE	TaMAPK45
22	TaMAPKKKK16	FALSE	TaMAPKKKK16
19	TaZIK1	FALSE	TaZIK1
19	TaZIK11	FALSE	TaZIK11
19	TaZIK8	FALSE	TaZIK8
19	TaZIK5	FALSE	TaZIK5
19	TaZIK10	FALSE	TaZIK10
19	TaZIK2	FALSE	TaZIK2
19	TaZIK9	FALSE	TaZIK9
19	TaZIK7	FALSE	TaZIK7
19	TaZIK4	FALSE	TaZIK4
19	TaZIK3	FALSE	TaZIK3
18	TaRaf91	FALSE	TaRaf91
18	TaRaf62	FALSE	TaRaf62
18	TaMEKK12	FALSE	TaMEKK12
18	TaMEKK3	FALSE	TaMEKK3
18	TaMEKK10	FALSE	TaMEKK10
17	TaRaf18	FALSE	TaRaf18
17	TaRaf60	FALSE	TaRaf60
17	TaRaf5	FALSE	TaRaf5
16	TaRaf41	FALSE	TaRaf41
15	TaRaf46	FALSE	TaRaf46
13	TaRaf7	FALSE	TaRaf7
12	TaRaf56	FALSE	TaRaf56
11	TaMAPKKKK1	FALSE	TaMAPKKKK1
11	TaMAPKKKK11	FALSE	TaMAPKKKK11
11	TaMAPKKKK13	FALSE	TaMAPKKKK13
11	TaMAPKKKK15	FALSE	TaMAPKKKK15
11	TaMAPKKKK2	FALSE	TaMAPKKKK2

11	TaMAPKKKK3	FALSE	TaMAPKKKK3
11	TaMAPKKKK7	FALSE	TaMAPKKKK7
11	TaMAPKKKK8	FALSE	TaMAPKKKK8
11	TaMAPKKKK9	FALSE	TaMAPKKKK9
11	TaMEKK25	FALSE	TaMEKK25
11	TaMAPKKKK20	FALSE	TaMAPKKKK20
11	TaMAPKKKK19	FALSE	TaMAPKKKK19
11	TaMAPKKKK17	FALSE	TaMAPKKKK17
11	TaMAPKKKK23	FALSE	TaMAPKKKK23
11	TaMAPKKKK24	FALSE	TaMAPKKKK24
11	TaMAPKKKK22	FALSE	TaMAPKKKK22
11	TaMAPKKKK25	FALSE	TaMAPKKKK25
11	TaMAPKKKK18	FALSE	TaMAPKKKK18
11	TaMAPKKKK21	FALSE	TaMAPKKKK21
10	TaRaf21	FALSE	TaRaf21
10	TaRaf95	FALSE	TaRaf95
10	TaRaf1	FALSE	TaRaf1
9	TaRaf59	FALSE	TaRaf59
9	TaRaf52	FALSE	TaRaf52
7	TaRaf111	FALSE	TaRaf111
7	TaRaf102	FALSE	TaRaf102
7	TaRaf42	FALSE	TaRaf42
7	TaRaf50	FALSE	TaRaf50
7	TaRaf43	FALSE	TaRaf43
7	TaRaf29	FALSE	TaRaf29
7	TaRaf79	FALSE	TaRaf79
7	TaRaf58	FALSE	TaRaf58
6	TaRaf105	FALSE	TaRaf105
6	TaRaf100	FALSE	TaRaf100
6	TaRaf63	FALSE	TaRaf63
5	TaRaf19	FALSE	TaRaf19
4	TaRaf73	FALSE	TaRaf73
3	TaRaf45	FALSE	TaRaf45
3	TaRaf72	FALSE	TaRaf72
3	TaRaf22	FALSE	TaRaf22
3	TaRaf81	FALSE	TaRaf81
3	TaRaf94	FALSE	TaRaf94
3	TaRaf89	FALSE	TaRaf89
2	TaRaf14	FALSE	TaRaf14
2	TaRaf83	FALSE	TaRaf83
2	TaRaf44	FALSE	TaRaf44
1	TaRaf113	FALSE	TaRaf113
1	TaRaf71	FALSE	TaRaf71

Table S4 Parameters for MAPK-MAP4K protein interaction analysis in wheat												
node1	node2	node1_string_id	node2_string_id	neighborhood_on_chromosome	gene_fusion	phylogenetic_cooccurrence	homology	coexpression	experimentally_determined_interaction	database_annotated	automated_textmining	combined_score
TaMAPK10	TaMAPKK9	4565.A0A3B6PGY7	4565.A0A077RVQ4	0	0	0.108	0.667	0	0.705	0.583	0.146	0.893
TaMAPK10	TaMEKK5	4565.A0A3B6PGY7	4565.A0A3B6AWC1	0	0	0.124	0.636	0	0.222	0.186	0.065	0.412
TaMAPK10	TaMAPKK15	4565.A0A3B6PGY7	4565.A0A3B6HW51	0	0	0.112	0.66	0	0.705	0.583	0.146	0.894
TaMAPK10	TaMAPKK14	4565.A0A3B6PGY7	4565.A0A3B6HY95	0	0	0.117	0.652	0	0.705	0.583	0.146	0.894
TaMAPK10	TaMAPKK16	4565.A0A3B6PGY7	4565.A0A3B6HZP7	0	0	0.115	0.666	0	0.705	0.583	0.146	0.894
TaMAPK10	TaMAPKK17	4565.A0A3B6PGY7	4565.A0A3B6I0M7	0	0	0.117	0.636	0	0.705	0.583	0.146	0.894
TaMAPK10	TaMAPKK8	4565.A0A3B6PGY7	4565.A0A3B6IK39	0	0	0.112	0.656	0	0.705	0.583	0.146	0.894
TaMAPK10	TaMAPKK6	4565.A0A3B6PGY7	4565.A0A3B6ILF0	0	0	0.114	0.635	0	0.705	0.583	0.146	0.894
TaMAPK10	TaMAPKK5	4565.A0A3B6PGY7	4565.A0A3B6IMW7	0	0	0.117	0.643	0	0.705	0.583	0.146	0.894
TaMAPK10	TaMAPKK7	4565.A0A3B6PGY7	4565.A0A3B6INV0	0	0	0.118	0.635	0	0.705	0.583	0.146	0.894
TaMAPK10	TaMEKK2	4565.A0A3B6PGY7	4565.A0A3B6JCC4	0	0	0.111	0.608	0	0.222	0.186	0.065	0.403
TaMAPK10	TaMAPKK11	4565.A0A3B6PGY7	4565.A0A3B6JEH0	0	0	0.119	0.656	0	0.705	0.583	0.146	0.895
TaMAPK10	TaMAPKK12	4565.A0A3B6PGY7	4565.A0A3B6JG06	0	0	0.119	0.63	0	0.705	0.583	0.146	0.895
TaMAPK10	TaMEKK14	4565.A0A3B6PGY7	4565.A0A3B6KF43	0	0	0.123	0.622	0	0.222	0.186	0.065	0.411
TaMAPK10	TaMAPKK4	4565.A0A3B6PGY7	4565.A0A3B6KFB5	0	0	0	0.613	0	0.705	0.825	0.195	0.954
TaMAPK10	TaMEKK15	4565.A0A3B6PGY7	4565.A0A3B6KPK7	0	0	0.112	0.634	0	0.222	0.186	0.065	0.403

TaMAPK10	TaMAPKK18	4565.A0A3B6PGY7	4565.A0A3B6LJ27	0	0	0	0.596	0	0.705	0.825	0.195	0.954
TaMAPK10	TaMEKK16	4565.A0A3B6PGY7	4565.A0A3B6LW00	0	0	0.11	0.634	0	0.222	0.186	0.065	0.402
TaMAPK10	TaMAPKK2	4565.A0A3B6PGY7	4565.A0A3B6LYW0	0	0	0	0.606	0	0.705	0.825	0.195	0.954
TaMAPK10	TaMAPKK3	4565.A0A3B6PGY7	4565.A0A3B6MNP8	0	0	0	0.595	0	0.705	0.825	0.195	0.954
TaMAPK10	TaMEKK11	4565.A0A3B6PGY7	4565.A0A3B6N0D8	0	0	0.11	0.633	0	0.222	0.186	0.065	0.402
TaMAPK10	TaMAPKK13	4565.A0A3B6PGY7	4565.A0A3B6N2X8	0	0	0	0.603	0	0.705	0.825	0.195	0.954
TaMAPK10	TaRaf87	4565.A0A3B6PGY7	4565.A0A3B6QMZ9	0	0	0	0.584	0.16	0.134	0.389	0.088	0.54
TaMAPK10	TaMAPK22	4565.A0A3B6PGY7	4565.A0A3B6TPT1	0	0	0.048	0.984	0	0	0.793	0	0.794
TaMAPK10	TaMAPK12	4565.A0A3B6PGY7	4565.A0A3B6RP80	0	0	0.048	0.985	0	0	0.793	0	0.794
TaMAPK10	TaMAPK54	4565.A0A3B6PGY7	4565.A0A3B6SKC9	0	0	0.048	0.985	0	0	0.793	0	0.794
TaMAPK10	TaMAPK47	4565.A0A3B6PGY7	4565.A0A3B6RAZ7	0	0	0.053	0.975	0	0	0.793	0	0.795
TaMAPK10	TaMAPKK1	4565.A0A3B6PGY7	4565.A0A3B6QJ87	0	0	0.131	0.609	0	0.705	0.583	0.146	0.896
TaMAPK11	TaMAPKK9	4565.A0A3B5XVG6	4565.A0A077RVQ4	0	0	0.126	0.623	0	0.705	0.583	0.146	0.896
TaMAPK11	TaMEKK24	4565.A0A3B5XVG6	4565.A0A3B6LLV5	0	0	0.177	0.59	0	0.27	0.063	0.088	0.417
TaMAPK11	TaMEKK29	4565.A0A3B5XVG6	4565.A0A3B6MSP6	0	0	0.177	0.59	0	0.27	0.063	0.088	0.418
TaMAPK11	TaMEKK17	4565.A0A3B5XVG6	4565.A0A3B6KFL8	0	0	0.177	0.59	0	0.27	0.063	0.088	0.418
TaMAPK11	TaMEKK2	4565.A0A3B5XVG6	4565.A0A3B6JCC4	0	0	0.137	0.568	0	0.222	0.186	0.065	0.42
TaMAPK11	TaMEKK11	4565.A0A3B5XVG6	4565.A0A3B6N0D8	0	0	0.149	0.57	0	0.222	0.186	0.065	0.428

TaMAPK11	TaMEKK15	4565.A0A3B5XVG6	4565.A0A3B6KPK7	0	0	0.152	0.573	0	0.222	0.186	0.065	0.43
TaMAPK11	TaMEKK16	4565.A0A3B5XVG6	4565.A0A3B6LW00	0	0	0.15	0.572	0	0.222	0.186	0.065	0.43
TaMAPK11	TaMEKK14	4565.A0A3B5XVG6	4565.A0A3B6KF43	0	0	0.153	0.58	0	0.222	0.186	0.065	0.431
TaMAPK11	TaMEKK5	4565.A0A3B5XVG6	4565.A0A3B6AWC1	0	0	0.159	0.574	0	0.222	0.186	0.065	0.435
TaMAPK11	TaRaf87	4565.A0A3B5XVG6	4565.A0A3B6QMZ9	0	0	0	0.565	0.3	0.134	0.389	0.088	0.617
TaMAPK11	TaMAPKK8	4565.A0A3B5XVG6	4565.A0A3B6IK39	0	0	0.123	0.616	0	0.705	0.583	0.146	0.895
TaMAPK11	TaMAPKK15	4565.A0A3B5XVG6	4565.A0A3B6HW51	0	0	0.134	0.615	0	0.705	0.583	0.146	0.896
TaMAPK11	TaMAPKK16	4565.A0A3B5XVG6	4565.A0A3B6HZP7	0	0	0.132	0.621	0	0.705	0.583	0.146	0.896
TaMAPK11	TaMAPKK5	4565.A0A3B5XVG6	4565.A0A3B6IMW7	0	0	0.128	0.609	0	0.705	0.583	0.146	0.896
TaMAPK11	TaMAPKK14	4565.A0A3B5XVG6	4565.A0A3B6HY95	0	0	0.133	0.611	0	0.705	0.583	0.146	0.896
TaMAPK11	TaMAPKK11	4565.A0A3B5XVG6	4565.A0A3B6JEH0	0	0	0.127	0.621	0	0.705	0.583	0.146	0.896
TaMAPK11	TaMAPKK7	4565.A0A3B5XVG6	4565.A0A3B6INV0	0	0	0.139	0.611	0	0.705	0.583	0.146	0.897
TaMAPK11	TaMAPKK17	4565.A0A3B5XVG6	4565.A0A3B6I0M7	0	0	0.137	0.613	0	0.705	0.583	0.146	0.897
TaMAPK11	TaMAPKK6	4565.A0A3B5XVG6	4565.A0A3B6ILF0	0	0	0.14	0.607	0	0.705	0.583	0.146	0.897
TaMAPK11	TaMAPKK1	4565.A0A3B5XVG6	4565.A0A3B6QJ87	0	0	0.136	0.615	0	0.705	0.583	0.146	0.897
TaMAPK11	TaMAPKK12	4565.A0A3B5XVG6	4565.A0A3B6JG06	0	0	0.146	0.608	0	0.705	0.583	0.146	0.898
TaMAPK11	TaMAPKK13	4565.A0A3B5XVG6	4565.A0A3B6N2X8	0	0	0.108	0.58	0	0.705	0.612	0.146	0.901
TaMAPK11	TaMAPKK2	4565.A0A3B5XVG6	4565.A0A3B6LYW0	0	0	0.123	0.581	0	0.705	0.612	0.146	0.902

TaMAPK11	TaMAPKK 3	4565.A0A3 B5XVG6	4565.A0A3 B6MNP8	0	0	0.131	0.577	0	0.705	0.612	0.146	0.903
TaMAPK11	TaMAPKK 4	4565.A0A3 B5XVG6	4565.A0A3 B6KFB5	0	0	0.13	0.63	0	0.705	0.612	0.146	0.903
TaMAPK11	TaMAPKK 18	4565.A0A3 B5XVG6	4565.A0A3 B6LJ27	0	0	0.137	0.583	0	0.705	0.612	0.146	0.904
TaMAPK12	TaMAPKK 9	4565.A0A3 B6RP80	4565.A0A0 77RVQ4	0	0	0.123	0.65	0	0.705	0.583	0.146	0.895
TaMAPK12	TaMEKK5	4565.A0A3 B6RP80	4565.A0A3 B6AWC1	0	0	0.132	0.623	0	0.222	0.186	0.065	0.417
TaMAPK12	TaMEKK1	4565.A0A3 B6RP80	4565.A0A3 B6B3I4	0	0	0.152	0.613	0	0.27	0.063	0.088	0.4
TaMAPK12	TaMAPKK 15	4565.A0A3 B6RP80	4565.A0A3 B6HW51	0	0	0.127	0.643	0	0.705	0.583	0.146	0.896
TaMAPK12	TaMAPKK 14	4565.A0A3 B6RP80	4565.A0A3 B6HY95	0	0	0.132	0.633	0	0.705	0.583	0.146	0.896
TaMAPK12	TaMAPKK 16	4565.A0A3 B6RP80	4565.A0A3 B6HZP7	0	0	0.129	0.648	0	0.705	0.583	0.146	0.896
TaMAPK12	TaMAPKK 17	4565.A0A3 B6RP80	4565.A0A3 B6I0M7	0	0	0.131	0.623	0	0.705	0.583	0.146	0.896
TaMAPK12	TaMAPKK 8	4565.A0A3 B6RP80	4565.A0A3 B6IK39	0	0	0.125	0.638	0	0.705	0.583	0.146	0.895
TaMAPK12	TaMAPKK 6	4565.A0A3 B6RP80	4565.A0A3 B6ILF0	0	0	0.129	0.625	0	0.705	0.583	0.146	0.896
TaMAPK12	TaMAPKK 5	4565.A0A3 B6RP80	4565.A0A3 B6IMW7	0	0	0.131	0.626	0	0.705	0.583	0.146	0.896
TaMAPK12	TaMAPKK 7	4565.A0A3 B6RP80	4565.A0A3 B6INV0	0	0	0.13	0.622	0	0.705	0.583	0.146	0.896
TaMAPK12	TaMEKK2	4565.A0A3 B6RP80	4565.A0A3 B6JCC4	0	0	0.115	0.604	0	0.222	0.186	0.065	0.406
TaMAPK12	TaMAPKK 11	4565.A0A3 B6RP80	4565.A0A3 B6JEH0	0	0	0.133	0.641	0	0.705	0.583	0.146	0.896
TaMAPK12	TaMAPKK 12	4565.A0A3 B6RP80	4565.A0A3 B6JG06	0	0	0.133	0.615	0	0.705	0.583	0.146	0.896
TaMAPK12	TaMEKK1 4	4565.A0A3 B6RP80	4565.A0A3 B6KF43	0	0	0.131	0.611	0	0.222	0.186	0.065	0.416

TaMAPK12	TaMAPKK 4	4565.A0A3 B6RP80	4565.A0A3 B6KFB5	0	0	0.104	0.611	0	0.705	0.739	0.195	0.937
TaMAPK12	TaMEKK1 7	4565.A0A3 B6RP80	4565.A0A3 B6KFL8	0	0	0.153	0.619	0	0.27	0.063	0.088	0.4
TaMAPK12	TaMEKK1 5	4565.A0A3 B6RP80	4565.A0A3 B6KPK7	0	0	0.122	0.617	0	0.222	0.186	0.065	0.41
TaMAPK12	TaMAPKK 18	4565.A0A3 B6RP80	4565.A0A3 B6LJ27	0	0	0	0.582	0	0.705	0.739	0.195	0.932
TaMAPK12	TaMEKK1 6	4565.A0A3 B6RP80	4565.A0A3 B6LW00	0	0	0.12	0.616	0	0.222	0.186	0.065	0.409
TaMAPK12	TaMAPKK 2	4565.A0A3 B6RP80	4565.A0A3 B6LYW0	0	0	0	0.593	0	0.705	0.886	0.195	0.97
TaMAPK12	TaMAPKK 3	4565.A0A3 B6RP80	4565.A0A3 B6MNP8	0	0	0	0.581	0	0.705	0.739	0.195	0.932
TaMAPK12	TaMEKK2 9	4565.A0A3 B6RP80	4565.A0A3 B6MSP6	0	0	0.153	0.62	0	0.27	0.063	0.088	0.401
TaMAPK12	TaMEKK1 1	4565.A0A3 B6RP80	4565.A0A3 B6N0D8	0	0	0.12	0.616	0	0.222	0.186	0.065	0.409
TaMAPK12	TaMAPKK 13	4565.A0A3 B6RP80	4565.A0A3 B6N2X8	0	0	0	0.588	0	0.705	0.886	0.195	0.97
TaMAPK12	TaMAPK3 6	4565.A0A3 B6RP80	4565.A0A3 B6NN33	0	0	0.048	0.984	0	0	0.793	0	0.794
TaMAPK12	TaMEKK4	4565.A0A3 B6RP80	4565.A0A3 B6NRN9	0	0	0.155	0.608	0	0.27	0.063	0.088	0.402
TaMAPK12	TaMEKK4-1	4565.A0A3 B6RP80	4565.A0A3 B6PNI6	0	0	0.154	0.61	0	0.27	0.063	0.088	0.402
TaMAPK12	TaMAPK4 5	4565.A0A3 B6RP80	4565.A0A3 B6QBD2	0	0	0.049	0.983	0	0	0.793	0	0.794
TaMAPK12	TaMAPKK 1	4565.A0A3 B6RP80	4565.A0A3 B6QJ87	0	0	0.142	0.611	0	0.705	0.583	0.146	0.897
TaMAPK12	TaRaf87	4565.A0A3 B6RP80	4565.A0A3 B6QMZ9	0	0	0	0.573	0.16	0.134	0.389	0.088	0.54
TaMAPK14	TaMAPKK 9	4565.A0A3 B5Y080	4565.A0A0 77RVQ4	0	0	0.137	0.61	0	0.705	0.583	0.146	0.897
TaMAPK14	TaMEKK1 7	4565.A0A3 B5Y080	4565.A0A3 B6KFL8	0	0	0.152	0.64	0	0.27	0.063	0.088	0.4

TaMAPK14	TaMEKK29	4565.A0A3B5Y080	4565.A0A3B6MSP6	0	0	0.152	0.64	0	0.27	0.063	0.088	0.4
TaMAPK14	TaMEKK2	4565.A0A3B5Y080	4565.A0A3B6JCC4	0	0	0.108	0.616	0	0.222	0.186	0.065	0.401
TaMAPK14	TaMEKK16	4565.A0A3B5Y080	4565.A0A3B6LW00	0	0	0.113	0.622	0	0.222	0.186	0.065	0.404
TaMAPK14	TaMEKK11	4565.A0A3B5Y080	4565.A0A3B6N0D8	0	0	0.113	0.621	0	0.222	0.186	0.065	0.404
TaMAPK14	TaMEKK15	4565.A0A3B5Y080	4565.A0A3B6KPK7	0	0	0.113	0.622	0	0.222	0.186	0.065	0.405
TaMAPK14	TaMEKK5	4565.A0A3B5Y080	4565.A0A3B6AWC1	0	0	0.12	0.634	0	0.222	0.186	0.065	0.409
TaMAPK14	TaMEKK14	4565.A0A3B5Y080	4565.A0A3B6KF43	0	0	0.12	0.628	0	0.222	0.186	0.065	0.409
TaMAPK14	TaRaf87	4565.A0A3B5Y080	4565.A0A3B6QMZ9	0	0	0	0.604	0.16	0.134	0.389	0.088	0.54
TaMAPK14	TaMAPK19	4565.A0A3B5Y080	4565.A0A3B6RDB9	0	0	0.054	0.973	0	0.139	0.841	0	0.859
TaMAPK14	TaMAPK6	4565.A0A3B5Y080	4565.A0A3B6SCW0	0	0	0.054	0.972	0	0.139	0.841	0	0.859
TaMAPK14	TaMAPKK13	4565.A0A3B5Y080	4565.A0A3B6N2X8	0	0	0	0.61	0	0.705	0.583	0.146	0.885
TaMAPK14	TaMAPKK2	4565.A0A3B5Y080	4565.A0A3B6LYW0	0	0	0.111	0.61	0	0.705	0.583	0.146	0.894
TaMAPK14	TaMAPKK3	4565.A0A3B5Y080	4565.A0A3B6MNP8	0	0	0.111	0.606	0	0.705	0.583	0.146	0.894
TaMAPK14	TaMAPKK4	4565.A0A3B5Y080	4565.A0A3B6KFB5	0	0	0.125	0.629	0	0.705	0.583	0.146	0.895
TaMAPK14	TaMAPKK18	4565.A0A3B5Y080	4565.A0A3B6LJ27	0	0	0.119	0.604	0	0.705	0.583	0.146	0.895
TaMAPK14	TaMAPKK1	4565.A0A3B5Y080	4565.A0A3B6QJ87	0	0	0.141	0.617	0	0.705	0.583	0.146	0.897
TaMAPK14	TaMAPKK14	4565.A0A3B5Y080	4565.A0A3B6HY95	0	0	0.143	0.607	0	0.705	0.583	0.146	0.897
TaMAPK14	TaMAPKK11	4565.A0A3B5Y080	4565.A0A3B6JEH0	0	0	0.139	0.618	0	0.705	0.583	0.146	0.897

TaMAPK14	TaMAPKK 12	4565.A0A3 B5Y080	4565.A0A3 B6JG06	0	0	0.139	0.615	0	0.705	0.583	0.146	0.897
TaMAPK14	TaMAPKK 7	4565.A0A3 B5Y080	4565.A0A3 B6INV0	0	0	0.138	0.615	0	0.705	0.583	0.146	0.897
TaMAPK14	TaMAPKK 15	4565.A0A3 B5Y080	4565.A0A3 B6HW51	0	0	0.14	0.612	0	0.705	0.583	0.146	0.897
TaMAPK14	TaMAPKK 6	4565.A0A3 B5Y080	4565.A0A3 B6ILF0	0	0	0.135	0.613	0	0.705	0.583	0.146	0.897
TaMAPK14	TaMAPKK 8	4565.A0A3 B5Y080	4565.A0A3 B6IK39	0	0	0.137	0.607	0	0.705	0.583	0.146	0.897
TaMAPK14	TaMAPKK 5	4565.A0A3 B5Y080	4565.A0A3 B6IMW7	0	0	0.141	0.6	0	0.705	0.583	0.146	0.897
TaMAPK14	TaMAPKK 17	4565.A0A3 B5Y080	4565.A0A3 B6I0M7	0	0	0.136	0.617	0	0.705	0.583	0.146	0.897
TaMAPK14	TaMAPKK 16	4565.A0A3 B5Y080	4565.A0A3 B6HZP7	0	0	0.147	0.608	0	0.705	0.583	0.146	0.898
TaMAPK16	TaMEKK2	4565.A0A0 77RTL3	4565.A0A3 B6JCC4	0	0	0.127	0.578	0	0.222	0.186	0.065	0.414
TaMAPK16	TaMEKK1 6	4565.A0A0 77RTL3	4565.A0A3 B6LW00	0	0	0.133	0.574	0	0.222	0.186	0.065	0.417
TaMAPK16	TaMEKK1 1	4565.A0A0 77RTL3	4565.A0A3 B6N0D8	0	0	0.132	0.572	0	0.222	0.186	0.065	0.417
TaMAPK16	TaMEKK1 5	4565.A0A0 77RTL3	4565.A0A3 B6KPK7	0	0	0.134	0.575	0	0.222	0.186	0.065	0.418
TaMAPK16	TaMEKK5	4565.A0A0 77RTL3	4565.A0A3 B6AWC1	0	0	0.144	0.579	0	0.222	0.186	0.065	0.425
TaMAPK16	TaMEKK1 7	4565.A0A0 77RTL3	4565.A0A3 B6KFL8	0	0	0.189	0.574	0	0.27	0.063	0.088	0.426
TaMAPK16	TaMEKK2 4	4565.A0A0 77RTL3	4565.A0A3 B6LLV5	0	0	0.189	0.574	0	0.27	0.063	0.088	0.426
TaMAPK16	TaMEKK1 4	4565.A0A0 77RTL3	4565.A0A3 B6KF43	0	0	0.145	0.586	0	0.222	0.186	0.065	0.426
TaMAPK16	TaMEKK2 9	4565.A0A0 77RTL3	4565.A0A3 B6MSP6	0	0	0.189	0.574	0	0.27	0.063	0.088	0.426
TaMAPK16	TaRaf87	4565.A0A0 77RTL3	4565.A0A3 B6QMZ9	0	0	0	0.564	0.277	0.134	0.389	0.088	0.604

TaMAPK16	TaMAPKK4	4565.A0A077RTL3	4565.A0A3B6KFB5	0	0	0	0.639	0	0.705	0.583	0.146	0.885
TaMAPK16	TaMAPKK18	4565.A0A077RTL3	4565.A0A3B6LJ27	0	0	0	0.586	0	0.705	0.583	0.146	0.885
TaMAPK16	TaMAPKK13	4565.A0A077RTL3	4565.A0A3B6N2X8	0	0	0	0.583	0	0.705	0.583	0.146	0.885
TaMAPK16	TaMAPKK2	4565.A0A077RTL3	4565.A0A3B6LYW0	0	0	0	0.584	0	0.705	0.583	0.146	0.885
TaMAPK16	TaMAPKK3	4565.A0A077RTL3	4565.A0A3B6MNP8	0	0	0	0.581	0	0.705	0.583	0.146	0.885
TaMAPK16	TaMAPKK14	4565.A0A077RTL3	4565.A0A3B6HY95	0	0	0.116	0.619	0	0.705	0.583	0.146	0.894
TaMAPK16	TaMAPKK9	4565.A0A077RTL3	4565.A0A077RVQ4	0	0	0.115	0.622	0	0.705	0.583	0.146	0.894
TaMAPK16	TaMAPKK8	4565.A0A077RTL3	4565.A0A3B6IK39	0	0	0.113	0.621	0	0.705	0.583	0.146	0.894
TaMAPK16	TaMAPKK16	4565.A0A077RTL3	4565.A0A3B6HZP7	0	0	0.117	0.621	0	0.705	0.583	0.146	0.894
TaMAPK16	TaMAPKK5	4565.A0A077RTL3	4565.A0A3B6IMW7	0	0	0.115	0.614	0	0.705	0.583	0.146	0.894
TaMAPK16	TaMAPKK11	4565.A0A077RTL3	4565.A0A3B6JEH0	0	0	0.118	0.618	0	0.705	0.583	0.146	0.895
TaMAPK16	TaMAPKK12	4565.A0A077RTL3	4565.A0A3B6JG06	0	0	0.125	0.61	0	0.705	0.583	0.146	0.895
TaMAPK16	TaMAPKK7	4565.A0A077RTL3	4565.A0A3B6INV0	0	0	0.123	0.613	0	0.705	0.583	0.146	0.895
TaMAPK16	TaMAPKK15	4565.A0A077RTL3	4565.A0A3B6HW51	0	0	0.118	0.621	0	0.705	0.583	0.146	0.895
TaMAPK16	TaMAPKK6	4565.A0A077RTL3	4565.A0A3B6ILF0	0	0	0.122	0.611	0	0.705	0.583	0.146	0.895
TaMAPK16	TaMAPKK17	4565.A0A077RTL3	4565.A0A3B6I0M7	0	0	0.121	0.615	0	0.705	0.583	0.146	0.895
TaMAPK16	TaMAPKK1	4565.A0A077RTL3	4565.A0A3B6QJ87	0	0	0.128	0.619	0	0.705	0.583	0.146	0.896
TaMAPK17	TaMAPKK9	4565.A0A3B6PHX1	4565.A0A077RVQ4	0	0	0.151	0.614	0	0.705	0.583	0.146	0.898

TaMAPK17	TaMEKK5	4565.A0A3B6PHX1	4565.A0A3B6AWC1	0	0	0.17	0.58	0	0.222	0.186	0.065	0.443
TaMAPK17	TaMEKK1	4565.A0A3B6PHX1	4565.A0A3B6B3I4	0	0	0.16	0.615	0	0.27	0.063	0.088	0.406
TaMAPK17	TaMAPKK15	4565.A0A3B6PHX1	4565.A0A3B6HW51	0	0	0.157	0.608	0	0.705	0.583	0.146	0.899
TaMAPK17	TaMAPKK14	4565.A0A3B6PHX1	4565.A0A3B6HY95	0	0	0.156	0.604	0	0.705	0.583	0.146	0.899
TaMAPK17	TaMAPKK16	4565.A0A3B6PHX1	4565.A0A3B6HZP7	0	0	0.152	0.618	0	0.705	0.583	0.146	0.899
TaMAPK17	TaMAPKK17	4565.A0A3B6PHX1	4565.A0A3B6I0M7	0	0	0.15	0.609	0	0.705	0.583	0.146	0.898
TaMAPK17	TaMAPKK8	4565.A0A3B6PHX1	4565.A0A3B6IK39	0	0	0.146	0.609	0	0.705	0.583	0.146	0.898
TaMAPK17	TaMAPKK6	4565.A0A3B6PHX1	4565.A0A3B6ILF0	0	0	0.149	0.606	0	0.705	0.583	0.146	0.898
TaMAPK17	TaMAPKK5	4565.A0A3B6PHX1	4565.A0A3B6IMW7	0	0	0.151	0.601	0	0.705	0.583	0.146	0.898
TaMAPK17	TaMAPKK7	4565.A0A3B6PHX1	4565.A0A3B6INV0	0	0	0.153	0.607	0	0.705	0.583	0.146	0.899
TaMAPK17	TaMEKK2	4565.A0A3B6PHX1	4565.A0A3B6JCC4	0	0	0.153	0.579	0	0.222	0.186	0.065	0.431
TaMAPK17	TaMAPKK11	4565.A0A3B6PHX1	4565.A0A3B6JEH0	0	0	0.15	0.613	0	0.705	0.583	0.146	0.898
TaMAPK17	TaMAPKK12	4565.A0A3B6PHX1	4565.A0A3B6JG06	0	0	0.154	0.605	0	0.705	0.583	0.146	0.899
TaMAPK17	TaMEKK14	4565.A0A3B6PHX1	4565.A0A3B6KF43	0	0	0.17	0.588	0	0.222	0.186	0.065	0.443
TaMAPK17	TaMAPKK4	4565.A0A3B6PHX1	4565.A0A3B6KFB5	0	0	0.119	0.638	0	0.705	0.612	0.146	0.902
TaMAPK17	TaMEKK17	4565.A0A3B6PHX1	4565.A0A3B6KFL8	0	0	0.18	0.607	0	0.27	0.063	0.088	0.42
TaMAPK17	TaMEKK15	4565.A0A3B6PHX1	4565.A0A3B6KPK7	0	0	0.165	0.582	0	0.222	0.186	0.065	0.439
TaMAPK17	TaMAPKK18	4565.A0A3B6PHX1	4565.A0A3B6LJ27	0	0	0.119	0.585	0	0.705	0.612	0.146	0.902

TaMAPK17	TaMEKK24	4565.A0A3B6PHX1	4565.A0A3B6LLV5	0	0	0.18	0.607	0	0.27	0.063	0.088	0.42
TaMAPK17	TaMEKK16	4565.A0A3B6PHX1	4565.A0A3B6LW00	0	0	0.164	0.582	0	0.222	0.186	0.065	0.438
TaMAPK17	TaMAPKK2	4565.A0A3B6PHX1	4565.A0A3B6LYW0	0	0	0.107	0.583	0	0.705	0.612	0.146	0.901
TaMAPK17	TaMAPKK3	4565.A0A3B6PHX1	4565.A0A3B6MNP8	0	0	0.109	0.582	0	0.705	0.612	0.146	0.901
TaMAPK17	TaMEKK29	4565.A0A3B6PHX1	4565.A0A3B6MSP6	0	0	0.181	0.607	0	0.27	0.063	0.088	0.421
TaMAPK17	TaMEKK11	4565.A0A3B6PHX1	4565.A0A3B6N0D8	0	0	0.162	0.58	0	0.222	0.186	0.065	0.437
TaMAPK17	TaMAPKK13	4565.A0A3B6PHX1	4565.A0A3B6N2X8	0	0	0	0.581	0	0.705	0.612	0.146	0.893
TaMAPK17	TaMEKK4	4565.A0A3B6PHX1	4565.A0A3B6NRN9	0	0	0.16	0.597	0	0.27	0.063	0.088	0.406
TaMAPK17	TaMEKK4-1	4565.A0A3B6PHX1	4565.A0A3B6PNI6	0	0	0.162	0.6	0	0.27	0.063	0.088	0.407
TaMAPK17	TaRaf87	4565.A0A3B6PHX1	4565.A0A3B6QMZ9	0	0	0	0.587	0.3	0.134	0.389	0.088	0.617
TaMAPK17	TaMAPKK1	4565.A0A3B6PHX1	4565.A0A3B6QJ87	0	0	0.156	0.607	0	0.705	0.583	0.146	0.899
TaMAPK19	TaMAPKK9	4565.A0A3B6RDB9	4565.A0A077RVQ4	0	0	0.13	0.633	0	0.705	0.763	0.146	0.941
TaMAPK19	TaMAPK28	4565.A0A3B6RDB9	4565.A0A3B5YXF4	0	0	0.054	0.973	0	0.139	0.841	0	0.859
TaMAPK19	TaMEKK5	4565.A0A3B6RDB9	4565.A0A3B6AWC1	0	0	0.126	0.634	0	0.222	0.186	0.065	0.413
TaMAPK19	TaRaf111	4565.A0A3B6RDB9	4565.A0A3B6FHS8	0	0	0	0	0.07	0.141	0.15	0.297	0.458
TaMAPK19	TaMAPKK15	4565.A0A3B6RDB9	4565.A0A3B6HW51	0	0	0.131	0.645	0	0.705	0.763	0.146	0.941
TaMAPK19	TaMAPKK14	4565.A0A3B6RDB9	4565.A0A3B6HY95	0	0	0.132	0.649	0	0.705	0.763	0.146	0.941
TaMAPK19	TaMAPKK16	4565.A0A3B6RDB9	4565.A0A3B6HZP7	0	0	0.136	0.652	0	0.705	0.763	0.146	0.941

TaMAPK19	TaMAPKK 17	4565.A0A3 B6RDB9	4565.A0A3 B6I0M7	0	0	0.13	0.639	0	0.705	0.763	0.146	0.941
TaMAPK19	TaMAPKK 8	4565.A0A3 B6RDB9	4565.A0A3 B6IK39	0	0	0.128	0.643	0	0.705	0.763	0.146	0.94
TaMAPK19	TaMAPKK 6	4565.A0A3 B6RDB9	4565.A0A3 B6ILF0	0	0	0.129	0.633	0	0.705	0.763	0.146	0.941
TaMAPK19	TaMAPKK 5	4565.A0A3 B6RDB9	4565.A0A3 B6IMW7	0	0	0.128	0.65	0	0.705	0.763	0.146	0.941
TaMAPK19	TaMAPKK 7	4565.A0A3 B6RDB9	4565.A0A3 B6INV0	0	0	0.131	0.638	0	0.705	0.763	0.146	0.941
TaMAPK19	TaMEKK2	4565.A0A3 B6RDB9	4565.A0A3 B6JCC4	0	0	0.108	0.623	0	0.222	0.186	0.065	0.401
TaMAPK19	TaMAPKK 11	4565.A0A3 B6RDB9	4565.A0A3 B6JEH0	0	0	0.134	0.645	0	0.705	0.763	0.146	0.941
TaMAPK19	TaMAPKK 12	4565.A0A3 B6RDB9	4565.A0A3 B6JG06	0	0	0.134	0.636	0	0.705	0.946	0.146	0.986
TaMAPK19	TaMAPK2 5	4565.A0A3 B6RDB9	4565.A0A3 B6JLL7	0	0	0.052	0.976	0	0	0.946	0.446	0.969
TaMAPK19	TaMEKK1 4	4565.A0A3 B6RDB9	4565.A0A3 B6KF43	0	0	0.124	0.62	0	0.222	0.186	0.065	0.412
TaMAPK19	TaMAPKK 4	4565.A0A3 B6RDB9	4565.A0A3 B6KFB5	0	0	0.101	0.636	0	0.705	0.825	0.209	0.958
TaMAPK19	TaMEKK1 5	4565.A0A3 B6RDB9	4565.A0A3 B6KPK7	0	0	0.117	0.62	0	0.222	0.186	0.065	0.407
TaMAPK19	TaMAPKK 18	4565.A0A3 B6RDB9	4565.A0A3 B6LJ27	0	0	0	0.602	0	0.705	0.825	0.209	0.955
TaMAPK19	TaMEKK1 6	4565.A0A3 B6RDB9	4565.A0A3 B6LW00	0	0	0.115	0.621	0	0.222	0.186	0.065	0.406
TaMAPK19	TaMAPKK 2	4565.A0A3 B6RDB9	4565.A0A3 B6LYW0	0	0	0	0.611	0	0.705	0.927	0.209	0.981
TaMAPK19	TaMAPKK 3	4565.A0A3 B6RDB9	4565.A0A3 B6MNP8	0	0	0	0.604	0	0.705	0.825	0.209	0.955
TaMAPK19	TaMEKK1 1	4565.A0A3 B6RDB9	4565.A0A3 B6N0D8	0	0	0.115	0.619	0	0.222	0.186	0.065	0.406
TaMAPK19	TaMAPKK 13	4565.A0A3 B6RDB9	4565.A0A3 B6N2X8	0	0	0	0.615	0	0.705	0.927	0.209	0.981

TaMAPK19	TaMAPKK 1	4565.A0A3 B6RDB9	4565.A0A3 B6QJ87	0	0	0.148	0.625	0	0.705	0.883	0.146	0.971
TaMAPK19	TaRaf87	4565.A0A3 B6RDB9	4565.A0A3 B6QMZ9	0	0	0	0.598	0.16	0.134	0.389	0.088	0.54
TaMAPK19	TaMAPK3	4565.A0A3 B6RDB9	4565.A7L5 U5	0	0	0.052	0.976	0	0	0.946	0.446	0.969
TaMAPK20	TaMAPKK 9	4565.A0A3 B6TNR5	4565.A0A0 77RVQ4	0	0	0.12	0.616	0	0.705	0.583	0.146	0.895
TaMAPK20	TaRaf88	4565.A0A3 B6TNR5	4565.A0A3 B5Z5X1	0	0	0.285	0	0	0.15	0.177	0.05	0.461
TaMAPK20	TaRaf30	4565.A0A3 B6TNR5	4565.A0A3 B6A1Z4	0	0	0.282	0	0	0.15	0.177	0.05	0.458
TaMAPK20	TaMEKK5	4565.A0A3 B6TNR5	4565.A0A3 B6AWC1	0	0	0.164	0.575	0	0.222	0.186	0.065	0.438
TaMAPK20	TaMAPKK 15	4565.A0A3 B6TNR5	4565.A0A3 B6HW51	0	0	0.126	0.611	0	0.705	0.583	0.146	0.895
TaMAPK20	TaMAPKK 14	4565.A0A3 B6TNR5	4565.A0A3 B6HY95	0	0	0.125	0.607	0	0.705	0.583	0.146	0.895
TaMAPK20	TaMAPKK 16	4565.A0A3 B6TNR5	4565.A0A3 B6HZP7	0	0	0.124	0.616	0	0.705	0.583	0.146	0.895
TaMAPK20	TaMAPKK 17	4565.A0A3 B6TNR5	4565.A0A3 B6I0M7	0	0	0.12	0.624	0	0.705	0.583	0.146	0.895
TaMAPK20	TaMAPKK 8	4565.A0A3 B6TNR5	4565.A0A3 B6IK39	0	0	0.118	0.615	0	0.705	0.583	0.146	0.894
TaMAPK20	TaMAPKK 6	4565.A0A3 B6TNR5	4565.A0A3 B6ILF0	0	0	0.122	0.609	0	0.705	0.583	0.146	0.895
TaMAPK20	TaMAPKK 5	4565.A0A3 B6TNR5	4565.A0A3 B6IMW7	0	0	0.122	0.605	0	0.705	0.583	0.146	0.895
TaMAPK20	TaMAPKK 7	4565.A0A3 B6TNR5	4565.A0A3 B6INV0	0	0	0.122	0.626	0	0.705	0.583	0.146	0.895
TaMAPK20	TaMEKK2	4565.A0A3 B6TNR5	4565.A0A3 B6JCC4	0	0	0.144	0.572	0	0.222	0.186	0.065	0.425
TaMAPK20	TaMAPKK 11	4565.A0A3 B6TNR5	4565.A0A3 B6JEH0	0	0	0.122	0.615	0	0.705	0.583	0.146	0.895
TaMAPK20	TaMAPKK 12	4565.A0A3 B6TNR5	4565.A0A3 B6JG06	0	0	0.126	0.621	0	0.705	0.583	0.146	0.895

TaMAPK20	TaMEKK14	4565.A0A3B6TNR5	4565.A0A3B6KF43	0	0	0.155	0.583	0	0.222	0.186	0.065	0.433
TaMAPK20	TaMAPKK4	4565.A0A3B6TNR5	4565.A0A3B6KFB5	0	0	0.105	0.637	0	0.705	0.612	0.146	0.9
TaMAPK20	TaMEKK17	4565.A0A3B6TNR5	4565.A0A3B6KFL8	0	0	0.17	0.582	0	0.27	0.063	0.088	0.413
TaMAPK20	TaMEKK15	4565.A0A3B6TNR5	4565.A0A3B6KPK7	0	0	0.156	0.575	0	0.222	0.186	0.065	0.433
TaMAPK20	TaMAPKK18	4565.A0A3B6TNR5	4565.A0A3B6LJ27	0	0	0	0.585	0	0.705	0.612	0.146	0.893
TaMAPK20	TaMEKK24	4565.A0A3B6TNR5	4565.A0A3B6LLV5	0	0	0.169	0.582	0	0.27	0.063	0.088	0.412
TaMAPK20	TaMEKK16	4565.A0A3B6TNR5	4565.A0A3B6LW00	0	0	0.154	0.575	0	0.222	0.186	0.065	0.432
TaMAPK20	TaMAPKK2	4565.A0A3B6TNR5	4565.A0A3B6LYW0	0	0	0	0.575	0	0.705	0.612	0.205	0.901
TaMAPK20	TaMAPKK3	4565.A0A3B6TNR5	4565.A0A3B6MNP8	0	0	0	0.58	0	0.705	0.612	0.146	0.893
TaMAPK20	TaMEKK29	4565.A0A3B6TNR5	4565.A0A3B6MSP6	0	0	0.171	0.581	0	0.27	0.063	0.088	0.414
TaMAPK20	TaMEKK11	4565.A0A3B6TNR5	4565.A0A3B6N0D8	0	0	0.153	0.574	0	0.222	0.186	0.065	0.431
TaMAPK20	TaMAPKK13	4565.A0A3B6TNR5	4565.A0A3B6N2X8	0	0	0	0.576	0	0.705	0.612	0.205	0.901
TaMAPK20	TaMAPKK1	4565.A0A3B6TNR5	4565.A0A3B6QJ87	0	0	0.126	0.619	0	0.705	0.583	0.146	0.895
TaMAPK20	TaRaf87	4565.A0A3B6TNR5	4565.A0A3B6QMZ9	0	0	0	0.565	0.3	0.134	0.389	0.088	0.617
TaMAPK22	TaMAPKK9	4565.A0A3B6TPT1	4565.A0A077RVQ4	0	0	0.118	0.646	0	0.705	0.583	0.146	0.895
TaMAPK22	TaMEKK5	4565.A0A3B6TPT1	4565.A0A3B6AWC1	0	0	0.125	0.634	0	0.222	0.186	0.065	0.412
TaMAPK22	TaMAPKK15	4565.A0A3B6TPT1	4565.A0A3B6HW51	0	0	0.122	0.638	0	0.705	0.583	0.146	0.895
TaMAPK22	TaMAPKK14	4565.A0A3B6TPT1	4565.A0A3B6HY95	0	0	0.127	0.629	0	0.705	0.583	0.146	0.896

TaMAPK22	TaMAPKK16	4565.A0A3B6TPT1	4565.A0A3B6HZP7	0	0	0.126	0.644	0	0.705	0.583	0.146	0.895
TaMAPK22	TaMAPKK17	4565.A0A3B6TPT1	4565.A0A3B6I0M7	0	0	0.127	0.621	0	0.705	0.583	0.146	0.896
TaMAPK22	TaMAPKK8	4565.A0A3B6TPT1	4565.A0A3B6IK39	0	0	0.12	0.635	0	0.705	0.583	0.146	0.895
TaMAPK22	TaMAPKK6	4565.A0A3B6TPT1	4565.A0A3B6ILF0	0	0	0.126	0.622	0	0.705	0.583	0.146	0.895
TaMAPK22	TaMAPKK5	4565.A0A3B6TPT1	4565.A0A3B6IMW7	0	0	0.125	0.624	0	0.705	0.583	0.146	0.895
TaMAPK22	TaMAPKK7	4565.A0A3B6TPT1	4565.A0A3B6INV0	0	0	0.127	0.619	0	0.705	0.583	0.146	0.896
TaMAPK22	TaMEKK2	4565.A0A3B6TPT1	4565.A0A3B6JCC4	0	0	0.11	0.607	0	0.222	0.186	0.065	0.403
TaMAPK22	TaMAPKK11	4565.A0A3B6TPT1	4565.A0A3B6JEH0	0	0	0.131	0.632	0	0.705	0.583	0.146	0.896
TaMAPK22	TaMAPKK12	4565.A0A3B6TPT1	4565.A0A3B6JG06	0	0	0.129	0.615	0	0.705	0.583	0.146	0.896
TaMAPK22	TaMEKK14	4565.A0A3B6TPT1	4565.A0A3B6KF43	0	0	0.125	0.621	0	0.222	0.186	0.065	0.413
TaMAPK22	TaMAPKK4	4565.A0A3B6TPT1	4565.A0A3B6KFB5	0	0	0.103	0.612	0	0.705	0.739	0.195	0.937
TaMAPK22	TaMEKK15	4565.A0A3B6TPT1	4565.A0A3B6KPK7	0	0	0.115	0.63	0	0.222	0.186	0.065	0.406
TaMAPK22	TaMAPKK18	4565.A0A3B6TPT1	4565.A0A3B6LJ27	0	0	0	0.591	0	0.705	0.739	0.195	0.932
TaMAPK22	TaMEKK16	4565.A0A3B6TPT1	4565.A0A3B6LW00	0	0	0.113	0.63	0	0.222	0.186	0.065	0.405
TaMAPK22	TaMAPKK2	4565.A0A3B6TPT1	4565.A0A3B6LYW0	0	0	0	0.604	0	0.705	0.886	0.195	0.97
TaMAPK22	TaMAPKK3	4565.A0A3B6TPT1	4565.A0A3B6MNP8	0	0	0	0.591	0	0.705	0.739	0.195	0.932
TaMAPK22	TaMEKK11	4565.A0A3B6TPT1	4565.A0A3B6N0D8	0	0	0.114	0.629	0	0.222	0.186	0.065	0.405
TaMAPK22	TaMAPKK13	4565.A0A3B6TPT1	4565.A0A3B6N2X8	0	0	0	0.6	0	0.705	0.886	0.195	0.97

TaMAPK22	TaMAPK36	4565.A0A3B6TPT1	4565.A0A3B6NN33	0	0	0.049	0.983	0	0	0.793	0	0.794
TaMAPK22	TaMAPK45	4565.A0A3B6TPT1	4565.A0A3B6QBD2	0	0	0.049	0.983	0	0	0.793	0	0.794
TaMAPK22	TaMAPKK1	4565.A0A3B6TPT1	4565.A0A3B6QJ87	0	0	0.138	0.608	0	0.705	0.583	0.146	0.897
TaMAPK22	TaRaf87	4565.A0A3B6TPT1	4565.A0A3B6QMZ9	0	0	0	0.579	0.16	0.134	0.389	0.088	0.54
TaMAPK23	TaMAPKK9	4565.A0A3B6GWR5	4565.A0A077RVQ4	0	0	0.12	0.625	0	0.705	0.583	0.146	0.895
TaMAPK23	TaMEKK5	4565.A0A3B6GWR5	4565.A0A3B6AWC1	0	0	0.148	0.583	0	0.222	0.186	0.065	0.428
TaMAPK23	TaMEKK17	4565.A0A3B6GWR5	4565.A0A3B6KFL8	0	0	0.161	0.605	0	0.27	0.063	0.088	0.406
TaMAPK23	TaMEKK24	4565.A0A3B6GWR5	4565.A0A3B6LLV5	0	0	0.161	0.605	0	0.27	0.063	0.088	0.406
TaMAPK23	TaMEKK29	4565.A0A3B6GWR5	4565.A0A3B6MSP6	0	0	0.161	0.605	0	0.27	0.063	0.088	0.407
TaMAPK23	TaMEKK2	4565.A0A3B6GWR5	4565.A0A3B6JCC4	0	0	0.133	0.577	0	0.222	0.186	0.065	0.418
TaMAPK23	TaMEKK16	4565.A0A3B6GWR5	4565.A0A3B6LW00	0	0	0.143	0.58	0	0.222	0.186	0.065	0.424
TaMAPK23	TaMEKK11	4565.A0A3B6GWR5	4565.A0A3B6N0D8	0	0	0.142	0.578	0	0.222	0.186	0.065	0.424
TaMAPK23	TaMEKK15	4565.A0A3B6GWR5	4565.A0A3B6KPK7	0	0	0.144	0.581	0	0.222	0.186	0.065	0.425
TaMAPK23	TaMEKK14	4565.A0A3B6GWR5	4565.A0A3B6KF43	0	0	0.148	0.596	0	0.222	0.186	0.065	0.428
TaMAPK23	TaRaf87	4565.A0A3B6GWR5	4565.A0A3B6QMZ9	0	0	0	0.571	0.3	0.134	0.389	0.088	0.617
TaMAPK23	TaMAPKK13	4565.A0A3B6GWR5	4565.A0A3B6N2X8	0	0	0	0.579	0	0.705	0.612	0.146	0.893
TaMAPK23	TaMAPKK2	4565.A0A3B6GWR5	4565.A0A3B6LYW0	0	0	0	0.579	0	0.705	0.612	0.146	0.893
TaMAPK23	TaMAPKK3	4565.A0A3B6GWR5	4565.A0A3B6MNP8	0	0	0	0.577	0	0.705	0.612	0.146	0.893

TaMAPK23	TaMAPKK8	4565.A0A3B6GWR5	4565.A0A3B6IK39	0	0	0.117	0.616	0	0.705	0.583	0.146	0.894
TaMAPK23	TaMAPKK14	4565.A0A3B6GWR5	4565.A0A3B6HY95	0	0	0.125	0.613	0	0.705	0.583	0.146	0.895
TaMAPK23	TaMAPKK11	4565.A0A3B6GWR5	4565.A0A3B6JEH0	0	0	0.121	0.618	0	0.705	0.583	0.146	0.895
TaMAPK23	TaMAPKK7	4565.A0A3B6GWR5	4565.A0A3B6INV0	0	0	0.126	0.612	0	0.705	0.583	0.146	0.895
TaMAPK23	TaMAPKK15	4565.A0A3B6GWR5	4565.A0A3B6HW51	0	0	0.126	0.617	0	0.705	0.583	0.146	0.895
TaMAPK23	TaMAPKK17	4565.A0A3B6GWR5	4565.A0A3B6I0M7	0	0	0.125	0.613	0	0.705	0.583	0.146	0.895
TaMAPK23	TaMAPKK6	4565.A0A3B6GWR5	4565.A0A3B6ILF0	0	0	0.125	0.606	0	0.705	0.583	0.146	0.895
TaMAPK23	TaMAPKK16	4565.A0A3B6GWR5	4565.A0A3B6HZP7	0	0	0.123	0.621	0	0.705	0.583	0.146	0.895
TaMAPK23	TaMAPKK5	4565.A0A3B6GWR5	4565.A0A3B6IMW7	0	0	0.121	0.61	0	0.705	0.583	0.146	0.895
TaMAPK23	TaMAPKK1	4565.A0A3B6GWR5	4565.A0A3B6QJ87	0	0	0.133	0.613	0	0.705	0.583	0.146	0.896
TaMAPK23	TaMAPKK12	4565.A0A3B6GWR5	4565.A0A3B6JG06	0	0	0.131	0.608	0	0.705	0.583	0.146	0.896
TaMAPK23	TaMAPKK18	4565.A0A3B6GWR5	4565.A0A3B6LJ27	0	0	0.108	0.576	0	0.705	0.612	0.146	0.901
TaMAPK23	TaMAPKK4	4565.A0A3B6GWR5	4565.A0A3B6KFB5	0	0	0.111	0.62	0	0.705	0.612	0.146	0.901
TaMAPK24	TaMAPKK9	4565.A0A3B6GX04	4565.A0A077RVQ4	0	0	0.114	0.622	0	0.705	0.583	0.146	0.894
TaMAPK24	TaRaf30	4565.A0A3B6GX04	4565.A0A3B6A1Z4	0	0	0.228	0	0	0.15	0.177	0.05	0.418
TaMAPK24	TaMEKK5	4565.A0A3B6GX04	4565.A0A3B6AWC1	0	0	0.144	0.579	0	0.222	0.186	0.065	0.425
TaMAPK24	TaMEKK2	4565.A0A3B6GX04	4565.A0A3B6JCC4	0	0	0.126	0.578	0	0.222	0.186	0.065	0.413
TaMAPK24	TaMEKK11	4565.A0A3B6GX04	4565.A0A3B6N0D8	0	0	0.131	0.572	0	0.222	0.186	0.065	0.417

TaMAPK24	TaMEKK16	4565.A0A3B6GX04	4565.A0A3B6LW00	0	0	0.132	0.574	0	0.222	0.186	0.065	0.417
TaMAPK24	TaMEKK15	4565.A0A3B6GX04	4565.A0A3B6KPK7	0	0	0.134	0.575	0	0.222	0.186	0.065	0.418
TaMAPK24	TaMEKK29	4565.A0A3B6GX04	4565.A0A3B6MSP6	0	0	0.188	0.574	0	0.27	0.063	0.088	0.425
TaMAPK24	TaMEKK14	4565.A0A3B6GX04	4565.A0A3B6KF43	0	0	0.144	0.586	0	0.222	0.186	0.065	0.425
TaMAPK24	TaMEKK24	4565.A0A3B6GX04	4565.A0A3B6LLV5	0	0	0.188	0.573	0	0.27	0.063	0.088	0.425
TaMAPK24	TaMEKK17	4565.A0A3B6GX04	4565.A0A3B6KFL8	0	0	0.188	0.574	0	0.27	0.063	0.088	0.425
TaMAPK24	TaRaf87	4565.A0A3B6GX04	4565.A0A3B6QMZ9	0	0	0	0.564	0.277	0.134	0.389	0.088	0.604
TaMAPK24	TaMAPKK2	4565.A0A3B6GX04	4565.A0A3B6LYW0	0	0	0	0.584	0	0.705	0.583	0.146	0.885
TaMAPK24	TaMAPKK3	4565.A0A3B6GX04	4565.A0A3B6MNP8	0	0	0	0.581	0	0.705	0.583	0.146	0.885
TaMAPK24	TaMAPKK13	4565.A0A3B6GX04	4565.A0A3B6N2X8	0	0	0	0.583	0	0.705	0.583	0.146	0.885
TaMAPK24	TaMAPKK18	4565.A0A3B6GX04	4565.A0A3B6LJ27	0	0	0	0.587	0	0.705	0.583	0.146	0.885
TaMAPK24	TaMAPKK4	4565.A0A3B6GX04	4565.A0A3B6KFB5	0	0	0	0.639	0	0.705	0.583	0.146	0.885
TaMAPK24	TaMAPKK15	4565.A0A3B6GX04	4565.A0A3B6HW51	0	0	0.117	0.621	0	0.705	0.583	0.146	0.894
TaMAPK24	TaMAPKK5	4565.A0A3B6GX04	4565.A0A3B6IMW7	0	0	0.115	0.613	0	0.705	0.583	0.146	0.894
TaMAPK24	TaMAPKK16	4565.A0A3B6GX04	4565.A0A3B6HZP7	0	0	0.117	0.621	0	0.705	0.583	0.146	0.894
TaMAPK24	TaMAPKK8	4565.A0A3B6GX04	4565.A0A3B6IK39	0	0	0.113	0.621	0	0.705	0.583	0.146	0.894
TaMAPK24	TaMAPKK14	4565.A0A3B6GX04	4565.A0A3B6HY95	0	0	0.115	0.619	0	0.705	0.583	0.146	0.894
TaMAPK24	TaMAPKK11	4565.A0A3B6GX04	4565.A0A3B6JEH0	0	0	0.118	0.618	0	0.705	0.583	0.146	0.894

TaMAPK24	TaMAPKK7	4565.A0A3B6GX04	4565.A0A3B6INV0	0	0	0.123	0.613	0	0.705	0.583	0.146	0.895
TaMAPK24	TaMAPKK17	4565.A0A3B6GX04	4565.A0A3B6I0M7	0	0	0.12	0.615	0	0.705	0.583	0.146	0.895
TaMAPK24	TaMAPKK6	4565.A0A3B6GX04	4565.A0A3B6ILF0	0	0	0.121	0.611	0	0.705	0.583	0.146	0.895
TaMAPK24	TaMAPKK12	4565.A0A3B6GX04	4565.A0A3B6JG06	0	0	0.124	0.61	0	0.705	0.583	0.146	0.895
TaMAPK24	TaMAPKK1	4565.A0A3B6GX04	4565.A0A3B6QJ87	0	0	0.128	0.619	0	0.705	0.583	0.146	0.896
TaMAPK25	TaMAPKK9	4565.A0A3B6JLL7	4565.A0A077RVQ4	0	0	0.127	0.63	0	0.705	0.791	0.146	0.947
TaMAPK25	TaMEKK5	4565.A0A3B6JLL7	4565.A0A3B6AWC1	0	0	0.118	0.652	0	0.222	0.186	0.065	0.408
TaMAPK25	TaMEKK1	4565.A0A3B6JLL7	4565.A0A3B6B3I4	0	0	0.127	0.658	0	0.27	0.063	0.137	0.416
TaMAPK25	TaRaf111	4565.A0A3B6JLL7	4565.A0A3B6FHS8	0	0	0	0	0.051	0.141	0.15	0.403	0.53
TaMAPK25	TaMAPKK15	4565.A0A3B6JLL7	4565.A0A3B6HW51	0	0	0.123	0.649	0	0.705	0.791	0.146	0.947
TaMAPK25	TaMAPKK14	4565.A0A3B6JLL7	4565.A0A3B6HY95	0	0	0.128	0.645	0	0.705	0.791	0.146	0.947
TaMAPK25	TaMAPKK16	4565.A0A3B6JLL7	4565.A0A3B6HZP7	0	0	0.133	0.643	0	0.705	0.791	0.146	0.948
TaMAPK25	TaMAPKK17	4565.A0A3B6JLL7	4565.A0A3B6I0M7	0	0	0.125	0.635	0	0.705	0.791	0.146	0.947
TaMAPK25	TaMAPKK8	4565.A0A3B6JLL7	4565.A0A3B6IK39	0	0	0.127	0.644	0	0.705	0.791	0.146	0.947
TaMAPK25	TaMAPKK6	4565.A0A3B6JLL7	4565.A0A3B6ILF0	0	0	0.125	0.634	0	0.705	0.791	0.146	0.947
TaMAPK25	TaMAPKK5	4565.A0A3B6JLL7	4565.A0A3B6IMW7	0	0	0.126	0.639	0	0.705	0.791	0.146	0.947
TaMAPK25	TaMAPKK7	4565.A0A3B6JLL7	4565.A0A3B6INV0	0	0	0.127	0.634	0	0.705	0.791	0.146	0.947
TaMAPK25	TaMEKK2	4565.A0A3B6JLL7	4565.A0A3B6JCC4	0	0	0.106	0.624	0	0.222	0.186	0.074	0.406

TaMAPK25	TaMAPKK11	4565.A0A3B6JLL7	4565.A0A3B6JEH0	0	0	0.134	0.633	0	0.705	0.791	0.146	0.948
TaMAPK25	TaMAPKK12	4565.A0A3B6JLL7	4565.A0A3B6JG06	0	0	0.127	0.63	0	0.705	0.959	0.146	0.989
TaMAPK25	TaMEKK11	4565.A0A3B6JLL7	4565.A0A3B6N0D8	0	0	0.106	0.638	0	0.222	0.186	0.065	0.4
TaMAPK25	TaMEKK14	4565.A0A3B6JLL7	4565.A0A3B6KF43	0	0	0.116	0.654	0	0.222	0.186	0.065	0.406
TaMAPK25	TaMEKK4-1	4565.A0A3B6JLL7	4565.A0A3B6PNI6	0	0	0.125	0.666	0	0.27	0.063	0.137	0.414
TaMAPK25	TaMEKK4	4565.A0A3B6JLL7	4565.A0A3B6NRN9	0	0	0.126	0.661	0	0.27	0.063	0.137	0.415
TaMAPK25	TaMEKK24	4565.A0A3B6JLL7	4565.A0A3B6LLV5	0	0	0.142	0.636	0	0.27	0.063	0.136	0.425
TaMAPK25	TaMEKK29	4565.A0A3B6JLL7	4565.A0A3B6MSP6	0	0	0.143	0.636	0	0.27	0.063	0.136	0.425
TaMAPK25	TaMEKK17	4565.A0A3B6JLL7	4565.A0A3B6KFL8	0	0	0.142	0.637	0	0.27	0.063	0.136	0.425
TaMAPK25	TaMEKK15	4565.A0A3B6JLL7	4565.A0A3B6KPK7	0	0	0.107	0.639	0.111	0.222	0.186	0.065	0.444
TaMAPK25	TaMEKK16	4565.A0A3B6JLL7	4565.A0A3B6LW00	0	0	0.106	0.639	0.114	0.222	0.186	0.065	0.445
TaMAPK25	TaRaf87	4565.A0A3B6JLL7	4565.A0A3B6QMZ9	0	0	0	0.598	0.16	0.134	0.389	0.088	0.54
TaMAPK25	TaMAPKK2	4565.A0A3B6JLL7	4565.A0A3B6LYW0	0	0	0	0.621	0	0.705	0.583	0.149	0.886
TaMAPK25	TaMAPKK3	4565.A0A3B6JLL7	4565.A0A3B6MNP8	0	0	0	0.62	0	0.705	0.583	0.149	0.886
TaMAPK25	TaMAPKK18	4565.A0A3B6JLL7	4565.A0A3B6LJ27	0	0	0	0.618	0	0.705	0.583	0.149	0.886
TaMAPK25	TaMAPKK13	4565.A0A3B6JLL7	4565.A0A3B6N2X8	0	0	0	0.618	0	0.705	0.583	0.149	0.886
TaMAPK25	TaMAPKK4	4565.A0A3B6JLL7	4565.A0A3B6KFB5	0	0	0.102	0.645	0	0.705	0.583	0.149	0.893
TaMAPK25	TaMAPK6	4565.A0A3B6JLL7	4565.A0A3B6SCW0	0	0	0.052	0.976	0	0	0.946	0.446	0.969

TaMAPK25	TaMAPKK1	4565.A0A3B6JLL7	4565.A0A3B6QJ87	0	0	0.141	0.615	0	0.705	0.888	0.146	0.972
TaMAPK27	TaMAPKK9	4565.A9RAB0	4565.A0A077RVQ4	0	0	0.127	0.623	0	0.705	0.583	0.146	0.896
TaMAPK27	TaMEKK5	4565.A9RAB0	4565.A0A3B6AWC1	0	0	0.159	0.574	0	0.222	0.186	0.065	0.435
TaMAPK27	TaMAPKK15	4565.A9RAB0	4565.A0A3B6HW51	0	0	0.134	0.615	0	0.705	0.583	0.146	0.896
TaMAPK27	TaMAPKK14	4565.A9RAB0	4565.A0A3B6HY95	0	0	0.134	0.61	0	0.705	0.583	0.146	0.896
TaMAPK27	TaMAPKK16	4565.A9RAB0	4565.A0A3B6HZP7	0	0	0.133	0.621	0	0.705	0.583	0.146	0.896
TaMAPK27	TaMAPKK17	4565.A9RAB0	4565.A0A3B6I0M7	0	0	0.138	0.613	0	0.705	0.583	0.146	0.897
TaMAPK27	TaMAPKK8	4565.A9RAB0	4565.A0A3B6IK39	0	0	0.124	0.615	0	0.705	0.583	0.146	0.895
TaMAPK27	TaMAPKK6	4565.A9RAB0	4565.A0A3B6ILF0	0	0	0.14	0.607	0	0.705	0.583	0.146	0.897
TaMAPK27	TaMAPKK5	4565.A9RAB0	4565.A0A3B6IMW7	0	0	0.129	0.609	0	0.705	0.583	0.146	0.896
TaMAPK27	TaMAPKK7	4565.A9RAB0	4565.A0A3B6INV0	0	0	0.139	0.611	0	0.705	0.583	0.146	0.897
TaMAPK27	TaMEKK2	4565.A9RAB0	4565.A0A3B6JCC4	0	0	0.137	0.568	0	0.222	0.186	0.065	0.42
TaMAPK27	TaMAPKK11	4565.A9RAB0	4565.A0A3B6JEH0	0	0	0.128	0.621	0	0.705	0.583	0.146	0.896
TaMAPK27	TaMAPKK12	4565.A9RAB0	4565.A0A3B6JG06	0	0	0.146	0.608	0	0.705	0.583	0.146	0.898
TaMAPK27	TaMEKK14	4565.A9RAB0	4565.A0A3B6KF43	0	0	0.153	0.581	0	0.222	0.186	0.065	0.431
TaMAPK27	TaMAPKK4	4565.A9RAB0	4565.A0A3B6KFB5	0	0	0.131	0.629	0	0.705	0.612	0.146	0.903
TaMAPK27	TaMEKK17	4565.A9RAB0	4565.A0A3B6KFL8	0	0	0.178	0.59	0	0.27	0.063	0.088	0.418
TaMAPK27	TaMEKK15	4565.A9RAB0	4565.A0A3B6KPK7	0	0	0.152	0.573	0	0.222	0.186	0.065	0.431

TaMAPK27	TaMAPKK18	4565.A9RAB0	4565.A0A3B6LJ27	0	0	0.138	0.582	0	0.705	0.612	0.146	0.904
TaMAPK27	TaMEKK24	4565.A9RAB0	4565.A0A3B6LLV5	0	0	0.177	0.59	0	0.27	0.063	0.088	0.418
TaMAPK27	TaMEKK16	4565.A9RAB0	4565.A0A3B6LW00	0	0	0.151	0.572	0	0.222	0.186	0.065	0.43
TaMAPK27	TaMAPKK2	4565.A9RAB0	4565.A0A3B6LYW0	0	0	0.124	0.581	0	0.705	0.612	0.146	0.903
TaMAPK27	TaMAPKK3	4565.A9RAB0	4565.A0A3B6MNP8	0	0	0.131	0.577	0	0.705	0.612	0.146	0.903
TaMAPK27	TaMEKK29	4565.A9RAB0	4565.A0A3B6MSP6	0	0	0.178	0.59	0	0.27	0.063	0.088	0.418
TaMAPK27	TaMEKK11	4565.A9RAB0	4565.A0A3B6N0D8	0	0	0.149	0.57	0	0.222	0.186	0.065	0.428
TaMAPK27	TaMAPKK13	4565.A9RAB0	4565.A0A3B6N2X8	0	0	0.109	0.58	0	0.705	0.612	0.146	0.901
TaMAPK27	TaMAPKK1	4565.A9RAB0	4565.A0A3B6QJ87	0	0	0.137	0.614	0	0.705	0.583	0.146	0.897
TaMAPK27	TaRaf87	4565.A9RAB0	4565.A0A3B6QMZ9	0	0	0	0.565	0.3	0.134	0.389	0.088	0.617
TaMAPK28	TaMAPKK9	4565.A0A3B5YXF4	4565.A0A077RVQ4	0	0	0.136	0.61	0	0.705	0.583	0.146	0.897
TaMAPK28	TaMEKK17	4565.A0A3B5YXF4	4565.A0A3B6KFL8	0	0	0.152	0.641	0	0.27	0.063	0.088	0.4
TaMAPK28	TaMEKK29	4565.A0A3B5YXF4	4565.A0A3B6MSP6	0	0	0.152	0.641	0	0.27	0.063	0.088	0.4
TaMAPK28	TaMEKK2	4565.A0A3B5YXF4	4565.A0A3B6JCC4	0	0	0.108	0.612	0	0.222	0.186	0.065	0.401
TaMAPK28	TaMEKK16	4565.A0A3B5YXF4	4565.A0A3B6LW00	0	0	0.112	0.623	0	0.222	0.186	0.065	0.404
TaMAPK28	TaMEKK15	4565.A0A3B5YXF4	4565.A0A3B6KPK7	0	0	0.113	0.623	0	0.222	0.186	0.065	0.404
TaMAPK28	TaMEKK11	4565.A0A3B5YXF4	4565.A0A3B6N0D8	0	0	0.112	0.622	0	0.222	0.186	0.065	0.404
TaMAPK28	TaMEKK5	4565.A0A3B5YXF4	4565.A0A3B6AWC1	0	0	0.121	0.633	0	0.222	0.186	0.065	0.409

TaMAPK28	TaMEKK14	4565.A0A3B5YXF4	4565.A0A3B6KF43	0	0	0.12	0.629	0	0.222	0.186	0.065	0.409
TaMAPK28	TaRaf87	4565.A0A3B5YXF4	4565.A0A3B6QMZ9	0	0	0	0.604	0.16	0.134	0.389	0.088	0.54
TaMAPK28	TaMAPK6	4565.A0A3B5YXF4	4565.A0A3B6SCW0	0	0	0.054	0.973	0	0.139	0.841	0	0.859
TaMAPK28	TaMAPKK13	4565.A0A3B5YXF4	4565.A0A3B6N2X8	0	0	0	0.61	0	0.705	0.583	0.146	0.885
TaMAPK28	TaMAPKK3	4565.A0A3B5YXF4	4565.A0A3B6MNP8	0	0	0.11	0.606	0	0.705	0.583	0.146	0.894
TaMAPK28	TaMAPKK2	4565.A0A3B5YXF4	4565.A0A3B6LYW0	0	0	0.111	0.61	0	0.705	0.583	0.146	0.894
TaMAPK28	TaMAPKK18	4565.A0A3B5YXF4	4565.A0A3B6LJ27	0	0	0.118	0.604	0	0.705	0.583	0.146	0.895
TaMAPK28	TaMAPKK4	4565.A0A3B5YXF4	4565.A0A3B6KFB5	0	0	0.125	0.629	0	0.705	0.583	0.146	0.895
TaMAPK28	TaMAPKK11	4565.A0A3B5YXF4	4565.A0A3B6JEH0	0	0	0.139	0.618	0	0.705	0.583	0.146	0.897
TaMAPK28	TaMAPKK12	4565.A0A3B5YXF4	4565.A0A3B6JG06	0	0	0.139	0.615	0	0.705	0.583	0.146	0.897
TaMAPK28	TaMAPKK14	4565.A0A3B5YXF4	4565.A0A3B6HY95	0	0	0.143	0.607	0	0.705	0.583	0.146	0.897
TaMAPK28	TaMAPKK1	4565.A0A3B5YXF4	4565.A0A3B6QJ87	0	0	0.141	0.617	0	0.705	0.583	0.146	0.897
TaMAPK28	TaMAPKK5	4565.A0A3B5YXF4	4565.A0A3B6IMW7	0	0	0.14	0.6	0	0.705	0.583	0.146	0.897
TaMAPK28	TaMAPKK6	4565.A0A3B5YXF4	4565.A0A3B6ILF0	0	0	0.135	0.613	0	0.705	0.583	0.146	0.897
TaMAPK28	TaMAPKK8	4565.A0A3B5YXF4	4565.A0A3B6IK39	0	0	0.137	0.607	0	0.705	0.583	0.146	0.897
TaMAPK28	TaMAPKK17	4565.A0A3B5YXF4	4565.A0A3B6I0M7	0	0	0.135	0.617	0	0.705	0.583	0.146	0.897
TaMAPK28	TaMAPKK15	4565.A0A3B5YXF4	4565.A0A3B6HW51	0	0	0.14	0.612	0	0.705	0.583	0.146	0.897
TaMAPK28	TaMAPKK7	4565.A0A3B5YXF4	4565.A0A3B6INV0	0	0	0.138	0.615	0	0.705	0.583	0.146	0.897

TaMAPK28	TaMAPKK16	4565.A0A3B5YXF4	4565.A0A3B6HZP7	0	0	0.146	0.608	0	0.705	0.583	0.146	0.898
TaMAPK29	TaMAPKK9	4565.A0A3B5Z4C1	4565.A0A077RVQ4	0	0	0.117	0.617	0	0.705	0.583	0.146	0.894
TaMAPK29	TaMEKK2	4565.A0A3B5Z4C1	4565.A0A3B6JCC4	0	0	0.128	0.576	0	0.222	0.186	0.065	0.414
TaMAPK29	TaMEKK17	4565.A0A3B5Z4C1	4565.A0A3B6KFL8	0	0	0.175	0.581	0	0.27	0.063	0.088	0.416
TaMAPK29	TaMEKK24	4565.A0A3B5Z4C1	4565.A0A3B6LLV5	0	0	0.174	0.581	0	0.27	0.063	0.088	0.416
TaMAPK29	TaMEKK29	4565.A0A3B5Z4C1	4565.A0A3B6MSP6	0	0	0.175	0.581	0	0.27	0.063	0.088	0.417
TaMAPK29	TaMEKK11	4565.A0A3B5Z4C1	4565.A0A3B6N0D8	0	0	0.136	0.572	0	0.222	0.186	0.065	0.42
TaMAPK29	TaMEKK16	4565.A0A3B5Z4C1	4565.A0A3B6LW00	0	0	0.137	0.573	0	0.222	0.186	0.065	0.421
TaMAPK29	TaMEKK15	4565.A0A3B5Z4C1	4565.A0A3B6KPK7	0	0	0.139	0.574	0	0.222	0.186	0.065	0.422
TaMAPK29	TaMEKK5	4565.A0A3B5Z4C1	4565.A0A3B6AWC1	0	0	0.146	0.578	0	0.222	0.186	0.065	0.427
TaMAPK29	TaMEKK14	4565.A0A3B5Z4C1	4565.A0A3B6KF43	0	0	0.15	0.582	0	0.222	0.186	0.065	0.429
TaMAPK29	TaRaf88	4565.A0A3B5Z4C1	4565.A0A3B5Z5X1	0	0	0.254	0	0	0.15	0.177	0.05	0.437
TaMAPK29	TaRaf30	4565.A0A3B5Z4C1	4565.A0A3B6A1Z4	0	0	0.255	0	0	0.15	0.177	0.05	0.438
TaMAPK29	TaRaf87	4565.A0A3B5Z4C1	4565.A0A3B6QMZ9	0	0	0	0.569	0.277	0.134	0.389	0.088	0.604
TaMAPK29	TaMAPKK18	4565.A0A3B5Z4C1	4565.A0A3B6LJ27	0	0	0	0.583	0	0.705	0.583	0.146	0.885
TaMAPK29	TaMAPKK13	4565.A0A3B5Z4C1	4565.A0A3B6N2X8	0	0	0	0.58	0	0.705	0.583	0.146	0.885
TaMAPK29	TaMAPKK2	4565.A0A3B5Z4C1	4565.A0A3B6LYW0	0	0	0	0.581	0	0.705	0.583	0.146	0.885
TaMAPK29	TaMAPKK3	4565.A0A3B5Z4C1	4565.A0A3B6MNP8	0	0	0	0.577	0	0.705	0.583	0.146	0.885

TaMAPK29	TaMAPKK4	4565.A0A3B5Z4C1	4565.A0A3B6KFB5	0	0	0.101	0.634	0	0.705	0.583	0.146	0.893
TaMAPK29	TaMAPKK8	4565.A0A3B5Z4C1	4565.A0A3B6IK39	0	0	0.115	0.621	0	0.705	0.583	0.146	0.894
TaMAPK29	TaMAPKK5	4565.A0A3B5Z4C1	4565.A0A3B6IMW7	0	0	0.118	0.607	0	0.705	0.583	0.146	0.894
TaMAPK29	TaMAPKK14	4565.A0A3B5Z4C1	4565.A0A3B6HY95	0	0	0.119	0.616	0	0.705	0.583	0.146	0.895
TaMAPK29	TaMAPKK11	4565.A0A3B5Z4C1	4565.A0A3B6JEH0	0	0	0.119	0.619	0	0.705	0.583	0.146	0.895
TaMAPK29	TaMAPKK12	4565.A0A3B5Z4C1	4565.A0A3B6JG06	0	0	0.123	0.613	0	0.705	0.583	0.146	0.895
TaMAPK29	TaMAPKK7	4565.A0A3B5Z4C1	4565.A0A3B6INV0	0	0	0.12	0.618	0	0.705	0.583	0.146	0.895
TaMAPK29	TaMAPKK15	4565.A0A3B5Z4C1	4565.A0A3B6HW51	0	0	0.119	0.62	0	0.705	0.583	0.146	0.895
TaMAPK29	TaMAPKK6	4565.A0A3B5Z4C1	4565.A0A3B6ILF0	0	0	0.119	0.616	0	0.705	0.583	0.146	0.895
TaMAPK29	TaMAPKK16	4565.A0A3B5Z4C1	4565.A0A3B6HZP7	0	0	0.12	0.621	0	0.705	0.583	0.146	0.895
TaMAPK29	TaMAPKK17	4565.A0A3B5Z4C1	4565.A0A3B6I0M7	0	0	0.118	0.618	0	0.705	0.583	0.146	0.895
TaMAPK29	TaMAPKK1	4565.A0A3B5Z4C1	4565.A0A3B6QJ87	0	0	0.129	0.613	0	0.705	0.583	0.146	0.896
TaMAPK3	TaMAPKK9	4565.A7L5U5	4565.A0A077RVQ4	0	0	0.126	0.629	0	0.705	0.791	0.146	0.947
TaMAPK3	TaMEKK5	4565.A7L5U5	4565.A0A3B6AWC1	0	0	0.119	0.65	0	0.222	0.186	0.065	0.408
TaMAPK3	TaMEKK1	4565.A7L5U5	4565.A0A3B6B3I4	0	0	0.124	0.658	0	0.27	0.063	0.137	0.414
TaMAPK3	TaRaf111	4565.A7L5U5	4565.A0A3B6FHS8	0	0	0	0	0.051	0.141	0.15	0.403	0.53
TaMAPK3	TaMAPKK15	4565.A7L5U5	4565.A0A3B6HW51	0	0	0.122	0.647	0	0.705	0.791	0.146	0.947
TaMAPK3	TaMAPKK14	4565.A7L5U5	4565.A0A3B6HY95	0	0	0.128	0.643	0	0.705	0.791	0.146	0.947

TaMAPK3	TaMAPKK 16	4565.A7L5 U5	4565.A0A3 B6HZP7	0	0	0.134	0.64	0	0.705	0.791	0.146	0.948
TaMAPK3	TaMAPKK 17	4565.A7L5 U5	4565.A0A3 B6I0M7	0	0	0.124	0.635	0	0.705	0.791	0.146	0.947
TaMAPK3	TaMAPKK 8	4565.A7L5 U5	4565.A0A3 B6IK39	0	0	0.128	0.642	0	0.705	0.791	0.146	0.947
TaMAPK3	TaMAPKK 6	4565.A7L5 U5	4565.A0A3 B6ILF0	0	0	0.126	0.63	0	0.705	0.791	0.146	0.947
TaMAPK3	TaMAPKK 5	4565.A7L5 U5	4565.A0A3 B6IMW7	0	0	0.126	0.636	0	0.705	0.791	0.146	0.947
TaMAPK3	TaMAPKK 7	4565.A7L5 U5	4565.A0A3 B6INV0	0	0	0.127	0.634	0	0.705	0.791	0.146	0.947
TaMAPK3	TaMEKK2	4565.A7L5 U5	4565.A0A3 B6JCC4	0	0	0.107	0.624	0	0.222	0.186	0.074	0.406
TaMAPK3	TaMAPKK 11	4565.A7L5 U5	4565.A0A3 B6JEH0	0	0	0.135	0.629	0	0.705	0.791	0.146	0.948
TaMAPK3	TaMAPKK 12	4565.A7L5 U5	4565.A0A3 B6JG06	0	0	0.127	0.63	0	0.705	0.959	0.146	0.989
TaMAPK3	TaMEKK1 4	4565.A7L5 U5	4565.A0A3 B6KF43	0	0	0.117	0.654	0	0.222	0.186	0.065	0.407
TaMAPK3	TaMAPKK 4	4565.A7L5 U5	4565.A0A3 B6KFB5	0	0	0.103	0.64	0	0.705	0.583	0.149	0.893
TaMAPK3	TaMEKK1 7	4565.A7L5 U5	4565.A0A3 B6KFL8	0	0	0.142	0.636	0	0.27	0.063	0.136	0.425
TaMAPK3	TaMEKK1 5	4565.A7L5 U5	4565.A0A3 B6KPK7	0	0	0.108	0.638	0.111	0.222	0.186	0.065	0.445
TaMAPK3	TaMAPKK 18	4565.A7L5 U5	4565.A0A3 B6LJ27	0	0	0	0.614	0	0.705	0.583	0.149	0.886
TaMAPK3	TaMEKK2 4	4565.A7L5 U5	4565.A0A3 B6LLV5	0	0	0.141	0.636	0	0.27	0.063	0.136	0.424
TaMAPK3	TaMEKK1 6	4565.A7L5 U5	4565.A0A3 B6LW00	0	0	0.107	0.638	0.114	0.222	0.186	0.065	0.446
TaMAPK3	TaMAPKK 2	4565.A7L5 U5	4565.A0A3 B6LYW0	0	0	0	0.622	0	0.705	0.583	0.149	0.886
TaMAPK3	TaMAPKK 3	4565.A7L5 U5	4565.A0A3 B6MNP8	0	0	0	0.616	0	0.705	0.583	0.149	0.886

TaMAPK3	TaMEKK29	4565.A7L5U5	4565.A0A3B6MSP6	0	0	0.142	0.636	0	0.27	0.063	0.136	0.425
TaMAPK3	TaMEKK11	4565.A7L5U5	4565.A0A3B6N0D8	0	0	0.107	0.637	0	0.222	0.186	0.065	0.4
TaMAPK3	TaMAPKK13	4565.A7L5U5	4565.A0A3B6N2X8	0	0	0	0.618	0	0.705	0.583	0.149	0.886
TaMAPK3	TaMEKK4	4565.A7L5U5	4565.A0A3B6NRN9	0	0	0.123	0.66	0	0.27	0.063	0.137	0.413
TaMAPK3	TaMEKK4-1	4565.A7L5U5	4565.A0A3B6PNI6	0	0	0.122	0.666	0	0.27	0.063	0.137	0.412
TaMAPK3	TaMAPKK1	4565.A7L5U5	4565.A0A3B6QJ87	0	0	0.14	0.615	0	0.705	0.888	0.146	0.972
TaMAPK3	TaRaf87	4565.A7L5U5	4565.A0A3B6QMZ9	0	0	0	0.597	0.16	0.134	0.389	0.088	0.54
TaMAPK3	TaMAPK6	4565.A7L5U5	4565.A0A3B6SCW0	0	0	0.052	0.976	0	0	0.946	0.446	0.969
TaMAPK30	TaMAPKK9	4565.A0A3B6RL73	4565.A0A077RVQ4	0	0	0.117	0.625	0	0.705	0.583	0.146	0.894
TaMAPK30	TaMEKK5	4565.A0A3B6RL73	4565.A0A3B6AWC1	0	0	0.151	0.595	0	0.222	0.186	0.065	0.43
TaMAPK30	TaMAPKK15	4565.A0A3B6RL73	4565.A0A3B6HW51	0	0	0.122	0.623	0	0.705	0.583	0.146	0.895
TaMAPK30	TaMAPKK14	4565.A0A3B6RL73	4565.A0A3B6HY95	0	0	0.12	0.619	0	0.705	0.583	0.146	0.895
TaMAPK30	TaMAPKK16	4565.A0A3B6RL73	4565.A0A3B6HZP7	0	0	0.121	0.624	0	0.705	0.583	0.146	0.895
TaMAPK30	TaMAPKK17	4565.A0A3B6RL73	4565.A0A3B6I0M7	0	0	0.12	0.626	0	0.705	0.583	0.146	0.895
TaMAPK30	TaMAPKK8	4565.A0A3B6RL73	4565.A0A3B6IK39	0	0	0.113	0.624	0	0.705	0.583	0.146	0.894
TaMAPK30	TaMAPKK6	4565.A0A3B6RL73	4565.A0A3B6ILF0	0	0	0.12	0.619	0	0.705	0.583	0.146	0.895
TaMAPK30	TaMAPKK5	4565.A0A3B6RL73	4565.A0A3B6IMW7	0	0	0.116	0.616	0	0.705	0.583	0.146	0.894
TaMAPK30	TaMAPKK7	4565.A0A3B6RL73	4565.A0A3B6INV0	0	0	0.122	0.623	0	0.705	0.583	0.146	0.895

TaMAPK30	TaMEKK2	4565.A0A3B6RL73	4565.A0A3B6JCC4	0	0	0.131	0.596	0	0.222	0.186	0.065	0.417
TaMAPK30	TaMAPKK11	4565.A0A3B6RL73	4565.A0A3B6JEH0	0	0	0.118	0.624	0	0.705	0.583	0.146	0.895
TaMAPK30	TaMAPKK12	4565.A0A3B6RL73	4565.A0A3B6JG06	0	0	0.124	0.619	0	0.705	0.583	0.146	0.895
TaMAPK30	TaMEKK14	4565.A0A3B6RL73	4565.A0A3B6KF43	0	0	0.145	0.609	0	0.222	0.186	0.065	0.426
TaMAPK30	TaMAPKK4	4565.A0A3B6RL73	4565.A0A3B6KFB5	0	0	0.103	0.626	0	0.705	0.583	0.146	0.893
TaMAPK30	TaMEKK17	4565.A0A3B6RL73	4565.A0A3B6KFL8	0	0	0.16	0.609	0	0.27	0.063	0.088	0.406
TaMAPK30	TaMEKK15	4565.A0A3B6RL73	4565.A0A3B6KPK7	0	0	0.142	0.594	0	0.222	0.186	0.065	0.424
TaMAPK30	TaMAPKK18	4565.A0A3B6RL73	4565.A0A3B6LJ27	0	0	0	0.592	0	0.705	0.583	0.146	0.885
TaMAPK30	TaMEKK24	4565.A0A3B6RL73	4565.A0A3B6LLV5	0	0	0.159	0.609	0	0.27	0.063	0.088	0.405
TaMAPK30	TaMEKK16	4565.A0A3B6RL73	4565.A0A3B6LW00	0	0	0.141	0.593	0	0.222	0.186	0.065	0.423
TaMAPK30	TaMAPKK2	4565.A0A3B6RL73	4565.A0A3B6LYW0	0	0	0	0.594	0	0.705	0.583	0.146	0.885
TaMAPK30	TaMAPKK3	4565.A0A3B6RL73	4565.A0A3B6MNP8	0	0	0	0.593	0	0.705	0.583	0.146	0.885
TaMAPK30	TaMEKK29	4565.A0A3B6RL73	4565.A0A3B6MSP6	0	0	0.161	0.608	0	0.27	0.063	0.088	0.406
TaMAPK30	TaMEKK11	4565.A0A3B6RL73	4565.A0A3B6N0D8	0	0	0.141	0.591	0	0.222	0.186	0.065	0.423
TaMAPK30	TaMAPKK13	4565.A0A3B6RL73	4565.A0A3B6N2X8	0	0	0	0.594	0	0.705	0.583	0.146	0.885
TaMAPK30	TaMAPKK1	4565.A0A3B6RL73	4565.A0A3B6QJ87	0	0	0.132	0.625	0	0.705	0.583	0.146	0.896
TaMAPK30	TaRaf87	4565.A0A3B6RL73	4565.A0A3B6QMZ9	0	0	0	0.585	0.277	0.134	0.389	0.088	0.604
TaMAPK33	TaMAPKK9	4565.A0A3B6A1J3	4565.A0A077RVQ4	0	0	0.118	0.617	0	0.705	0.583	0.146	0.894

TaMAPK33	TaRaf88	4565.A0A3B6A1J3	4565.A0A3B5Z5X1	0	0	0.254	0	0	0.15	0.177	0.05	0.437
TaMAPK33	TaMEKK2	4565.A0A3B6A1J3	4565.A0A3B6JCC4	0	0	0.128	0.577	0	0.222	0.186	0.065	0.414
TaMAPK33	TaMEKK24	4565.A0A3B6A1J3	4565.A0A3B6LLV5	0	0	0.174	0.581	0	0.27	0.063	0.088	0.416
TaMAPK33	TaMEKK29	4565.A0A3B6A1J3	4565.A0A3B6MSP6	0	0	0.175	0.581	0	0.27	0.063	0.088	0.416
TaMAPK33	TaMEKK17	4565.A0A3B6A1J3	4565.A0A3B6KFL8	0	0	0.175	0.581	0	0.27	0.063	0.088	0.416
TaMAPK33	TaMEKK11	4565.A0A3B6A1J3	4565.A0A3B6N0D8	0	0	0.136	0.572	0	0.222	0.186	0.065	0.419
TaMAPK33	TaMEKK16	4565.A0A3B6A1J3	4565.A0A3B6LW00	0	0	0.136	0.574	0	0.222	0.186	0.065	0.42
TaMAPK33	TaMEKK15	4565.A0A3B6A1J3	4565.A0A3B6KPK7	0	0	0.138	0.574	0	0.222	0.186	0.065	0.421
TaMAPK33	TaMEKK5	4565.A0A3B6A1J3	4565.A0A3B6AWC1	0	0	0.147	0.579	0	0.222	0.186	0.065	0.427
TaMAPK33	TaMEKK14	4565.A0A3B6A1J3	4565.A0A3B6KF43	0	0	0.15	0.582	0	0.222	0.186	0.065	0.429
TaMAPK33	TaRaf30	4565.A0A3B6A1J3	4565.A0A3B6A1Z4	0	0	0.255	0	0	0.15	0.177	0.05	0.438
TaMAPK33	TaRaf87	4565.A0A3B6A1J3	4565.A0A3B6QMZ9	0	0	0	0.569	0.277	0.134	0.389	0.088	0.604
TaMAPK33	TaMAPKK2	4565.A0A3B6A1J3	4565.A0A3B6LYW0	0	0	0	0.583	0	0.705	0.583	0.146	0.885
TaMAPK33	TaMAPKK3	4565.A0A3B6A1J3	4565.A0A3B6MNP8	0	0	0	0.578	0	0.705	0.583	0.146	0.885
TaMAPK33	TaMAPKK18	4565.A0A3B6A1J3	4565.A0A3B6LJ27	0	0	0	0.583	0	0.705	0.583	0.146	0.885
TaMAPK33	TaMAPKK13	4565.A0A3B6A1J3	4565.A0A3B6N2X8	0	0	0	0.581	0	0.705	0.583	0.146	0.885
TaMAPK33	TaMAPKK4	4565.A0A3B6A1J3	4565.A0A3B6KFB5	0	0	0.102	0.634	0	0.705	0.583	0.146	0.893
TaMAPK33	TaMAPKK8	4565.A0A3B6A1J3	4565.A0A3B6IK39	0	0	0.115	0.621	0	0.705	0.583	0.146	0.894

TaMAPK33	TaMAPKK15	4565.A0A3B6A1J3	4565.A0A3B6HW51	0	0	0.12	0.62	0	0.705	0.583	0.146	0.895
TaMAPK33	TaMAPKK7	4565.A0A3B6A1J3	4565.A0A3B6INV0	0	0	0.121	0.617	0	0.705	0.583	0.146	0.895
TaMAPK33	TaMAPKK5	4565.A0A3B6A1J3	4565.A0A3B6IMW7	0	0	0.119	0.607	0	0.705	0.583	0.146	0.895
TaMAPK33	TaMAPKK16	4565.A0A3B6A1J3	4565.A0A3B6HZP7	0	0	0.12	0.621	0	0.705	0.583	0.146	0.895
TaMAPK33	TaMAPKK6	4565.A0A3B6A1J3	4565.A0A3B6ILF0	0	0	0.12	0.616	0	0.705	0.583	0.146	0.895
TaMAPK33	TaMAPKK17	4565.A0A3B6A1J3	4565.A0A3B6I0M7	0	0	0.119	0.618	0	0.705	0.583	0.146	0.895
TaMAPK33	TaMAPKK14	4565.A0A3B6A1J3	4565.A0A3B6HY95	0	0	0.12	0.616	0	0.705	0.583	0.146	0.895
TaMAPK33	TaMAPKK11	4565.A0A3B6A1J3	4565.A0A3B6JEH0	0	0	0.12	0.62	0	0.705	0.583	0.146	0.895
TaMAPK33	TaMAPKK12	4565.A0A3B6A1J3	4565.A0A3B6JG06	0	0	0.123	0.613	0	0.705	0.583	0.146	0.895
TaMAPK33	TaMAPKK1	4565.A0A3B6A1J3	4565.A0A3B6QJ87	0	0	0.129	0.612	0	0.705	0.583	0.146	0.896
TaMAPK34	TaMAPKK9	4565.A0A3B6A3E4	4565.A0A077RVQ4	0	0	0.117	0.619	0	0.705	0.583	0.146	0.894
TaMAPK34	TaMAPK43	4565.A0A3B6A3E4	4565.A0A3B5Y7D4	0	0	0.047	0.987	0	0	0.54	0	0.543
TaMAPK34	TaRaf30	4565.A0A3B6A3E4	4565.A0A3B6A1Z4	0	0	0.235	0	0	0.15	0.177	0.05	0.423
TaMAPK34	TaRaf14	4565.A0A3B6A3E4	4565.A0A3B6TYA5	0	0	0.213	0	0.041	0.15	0.177	0	0.401
TaMAPK34	TaMEKK2	4565.A0A3B6A3E4	4565.A0A3B6JCC4	0	0	0.128	0.574	0	0.222	0.186	0.065	0.414
TaMAPK34	TaMEKK11	4565.A0A3B6A3E4	4565.A0A3B6N0D8	0	0	0.132	0.572	0	0.222	0.186	0.065	0.417
TaMAPK34	TaMEKK16	4565.A0A3B6A3E4	4565.A0A3B6LW00	0	0	0.133	0.573	0	0.222	0.186	0.065	0.418
TaMAPK34	TaMEKK17	4565.A0A3B6A3E4	4565.A0A3B6KFL8	0	0	0.177	0.575	0	0.27	0.063	0.088	0.418

TaMAPK34	TaMEKK24	4565.A0A3B6A3E4	4565.A0A3B6LLV5	0	0	0.177	0.575	0	0.27	0.063	0.088	0.418
TaMAPK34	TaMEKK29	4565.A0A3B6A3E4	4565.A0A3B6MSP6	0	0	0.177	0.575	0	0.27	0.063	0.088	0.418
TaMAPK34	TaMEKK15	4565.A0A3B6A3E4	4565.A0A3B6KPK7	0	0	0.135	0.574	0	0.222	0.186	0.065	0.419
TaMAPK34	TaMEKK5	4565.A0A3B6A3E4	4565.A0A3B6AWC1	0	0	0.14	0.582	0	0.222	0.186	0.065	0.423
TaMAPK34	TaMEKK14	4565.A0A3B6A3E4	4565.A0A3B6KF43	0	0	0.142	0.582	0	0.222	0.186	0.065	0.424
TaMAPK34	TaRaf87	4565.A0A3B6A3E4	4565.A0A3B6QMZ9	0	0	0	0.56	0.277	0.134	0.389	0.088	0.604
TaMAPK34	TaMAPKK18	4565.A0A3B6A3E4	4565.A0A3B6LJ27	0	0	0	0.589	0	0.705	0.583	0.146	0.885
TaMAPK34	TaMAPKK13	4565.A0A3B6A3E4	4565.A0A3B6N2X8	0	0	0	0.583	0	0.705	0.583	0.146	0.885
TaMAPK34	TaMAPKK3	4565.A0A3B6A3E4	4565.A0A3B6MNP8	0	0	0	0.583	0	0.705	0.583	0.146	0.885
TaMAPK34	TaMAPKK2	4565.A0A3B6A3E4	4565.A0A3B6LYW0	0	0	0	0.585	0	0.705	0.583	0.146	0.885
TaMAPK34	TaMAPKK4	4565.A0A3B6A3E4	4565.A0A3B6KFB5	0	0	0.097	0.644	0	0.705	0.583	0.146	0.892
TaMAPK34	TaMAPKK11	4565.A0A3B6A3E4	4565.A0A3B6JEH0	0	0	0.118	0.615	0	0.705	0.583	0.146	0.894
TaMAPK34	TaMAPKK8	4565.A0A3B6A3E4	4565.A0A3B6IK39	0	0	0.117	0.612	0	0.705	0.583	0.146	0.894
TaMAPK34	TaMAPKK12	4565.A0A3B6A3E4	4565.A0A3B6JG06	0	0	0.122	0.607	0	0.705	0.583	0.146	0.895
TaMAPK34	TaMAPKK14	4565.A0A3B6A3E4	4565.A0A3B6HY95	0	0	0.119	0.612	0	0.705	0.583	0.146	0.895
TaMAPK34	TaMAPKK6	4565.A0A3B6A3E4	4565.A0A3B6ILF0	0	0	0.121	0.608	0	0.705	0.583	0.146	0.895
TaMAPK34	TaMAPKK16	4565.A0A3B6A3E4	4565.A0A3B6HZP7	0	0	0.12	0.616	0	0.705	0.583	0.146	0.895
TaMAPK34	TaMAPKK5	4565.A0A3B6A3E4	4565.A0A3B6IMW7	0	0	0.12	0.609	0	0.705	0.583	0.146	0.895

TaMAPK34	TaMAPKK17	4565.A0A3B6A3E4	4565.A0A3B6I0M7	0	0	0.12	0.612	0	0.705	0.583	0.146	0.895
TaMAPK34	TaMAPKK7	4565.A0A3B6A3E4	4565.A0A3B6INV0	0	0	0.123	0.61	0	0.705	0.583	0.146	0.895
TaMAPK34	TaMAPKK15	4565.A0A3B6A3E4	4565.A0A3B6HW51	0	0	0.121	0.617	0	0.705	0.583	0.146	0.895
TaMAPK34	TaMAPKK1	4565.A0A3B6A3E4	4565.A0A3B6QJ87	0	0	0.127	0.605	0	0.705	0.583	0.146	0.896
TaMAPK35	TaMAPKK9	4565.A0A3B6NJX2	4565.A0A077RVQ4	0	0	0.144	0.619	0	0.705	0.583	0.146	0.898
TaMAPK35	TaMEKK5	4565.A0A3B6NJX2	4565.A0A3B6AWC1	0	0	0.173	0.574	0	0.222	0.186	0.065	0.445
TaMAPK35	TaMEKK1	4565.A0A3B6NJX2	4565.A0A3B6B3I4	0	0	0.16	0.615	0	0.27	0.063	0.088	0.406
TaMAPK35	TaMAPKK15	4565.A0A3B6NJX2	4565.A0A3B6HW51	0	0	0.151	0.613	0	0.705	0.583	0.146	0.898
TaMAPK35	TaMAPKK14	4565.A0A3B6NJX2	4565.A0A3B6HY95	0	0	0.15	0.607	0	0.705	0.583	0.146	0.898
TaMAPK35	TaMAPKK16	4565.A0A3B6NJX2	4565.A0A3B6HZP7	0	0	0.146	0.62	0	0.705	0.583	0.146	0.898
TaMAPK35	TaMAPKK17	4565.A0A3B6NJX2	4565.A0A3B6I0M7	0	0	0.146	0.602	0	0.705	0.583	0.146	0.898
TaMAPK35	TaMAPKK8	4565.A0A3B6NJX2	4565.A0A3B6IK39	0	0	0.14	0.613	0	0.705	0.583	0.146	0.897
TaMAPK35	TaMAPKK6	4565.A0A3B6NJX2	4565.A0A3B6ILF0	0	0	0.141	0.612	0	0.705	0.583	0.146	0.897
TaMAPK35	TaMAPKK5	4565.A0A3B6NJX2	4565.A0A3B6IMW7	0	0	0.146	0.604	0	0.705	0.583	0.146	0.898
TaMAPK35	TaMAPKK7	4565.A0A3B6NJX2	4565.A0A3B6INV0	0	0	0.147	0.602	0	0.705	0.583	0.146	0.898
TaMAPK35	TaMEKK2	4565.A0A3B6NJX2	4565.A0A3B6JCC4	0	0	0.152	0.574	0	0.222	0.186	0.065	0.431
TaMAPK35	TaMAPKK11	4565.A0A3B6NJX2	4565.A0A3B6JEH0	0	0	0.144	0.618	0	0.705	0.583	0.146	0.898
TaMAPK35	TaMAPKK12	4565.A0A3B6NJX2	4565.A0A3B6JG06	0	0	0.149	0.6	0	0.705	0.583	0.146	0.898

TaMAPK35	TaMEKK14	4565.A0A3B6NJX2	4565.A0A3B6KF43	0	0	0.17	0.578	0	0.222	0.186	0.065	0.443
TaMAPK35	TaMAPKK4	4565.A0A3B6NJX2	4565.A0A3B6KFB5	0	0	0.117	0.628	0	0.705	0.612	0.146	0.902
TaMAPK35	TaMEKK17	4565.A0A3B6NJX2	4565.A0A3B6KFL8	0	0	0.178	0.607	0	0.27	0.063	0.088	0.419
TaMAPK35	TaMEKK15	4565.A0A3B6NJX2	4565.A0A3B6KPK7	0	0	0.165	0.575	0	0.222	0.186	0.065	0.44
TaMAPK35	TaMAPKK18	4565.A0A3B6NJX2	4565.A0A3B6LJ27	0	0	0.115	0.581	0	0.705	0.612	0.146	0.901
TaMAPK35	TaMEKK24	4565.A0A3B6NJX2	4565.A0A3B6LLV5	0	0	0.178	0.607	0	0.27	0.063	0.088	0.418
TaMAPK35	TaMEKK16	4565.A0A3B6NJX2	4565.A0A3B6LW00	0	0	0.165	0.575	0	0.222	0.186	0.065	0.439
TaMAPK35	TaMAPKK2	4565.A0A3B6NJX2	4565.A0A3B6LYW0	0	0	0	0.579	0	0.705	0.612	0.146	0.893
TaMAPK35	TaMAPKK3	4565.A0A3B6NJX2	4565.A0A3B6MNP8	0	0	0	0.578	0	0.705	0.612	0.146	0.893
TaMAPK35	TaMEKK29	4565.A0A3B6NJX2	4565.A0A3B6MSP6	0	0	0.179	0.607	0	0.27	0.063	0.088	0.419
TaMAPK35	TaMEKK11	4565.A0A3B6NJX2	4565.A0A3B6N0D8	0	0	0.163	0.573	0	0.222	0.186	0.065	0.438
TaMAPK35	TaMAPKK13	4565.A0A3B6NJX2	4565.A0A3B6N2X8	0	0	0	0.579	0	0.705	0.612	0.146	0.893
TaMAPK35	TaMEKK4	4565.A0A3B6NJX2	4565.A0A3B6NRN9	0	0	0.161	0.597	0	0.27	0.063	0.088	0.407
TaMAPK35	TaMEKK4-1	4565.A0A3B6NJX2	4565.A0A3B6PNI6	0	0	0.164	0.599	0	0.27	0.063	0.088	0.408
TaMAPK35	TaRaf21	4565.A0A3B6NJX2	4565.A0A3B6TVC5	0	0	0.229	0	0	0.15	0.177	0.05	0.419
TaMAPK35	TaRaf87	4565.A0A3B6NJX2	4565.A0A3B6QMZ9	0	0	0	0.578	0.3	0.134	0.389	0.088	0.617
TaMAPK35	TaMAPKK1	4565.A0A3B6NJX2	4565.A0A3B6QJ87	0	0	0.147	0.612	0	0.705	0.583	0.146	0.898
TaMAPK36	TaMAPKK9	4565.A0A3B6NN33	4565.A0A077RVQ4	0	0	0	0.661	0	0.705	0.583	0.146	0.885

TaMAPK36	TaMAPKK15	4565.A0A3B6NN33	4565.A0A3B6HW51	0	0	0	0.653	0	0.705	0.583	0.146	0.885
TaMAPK36	TaMAPKK14	4565.A0A3B6NN33	4565.A0A3B6HY95	0	0	0	0.644	0	0.705	0.583	0.146	0.885
TaMAPK36	TaMAPKK16	4565.A0A3B6NN33	4565.A0A3B6HZP7	0	0	0.095	0.661	0	0.705	0.583	0.146	0.892
TaMAPK36	TaMAPKK17	4565.A0A3B6NN33	4565.A0A3B6I0M7	0	0	0	0.633	0	0.705	0.583	0.146	0.885
TaMAPK36	TaMAPKK8	4565.A0A3B6NN33	4565.A0A3B6IK39	0	0	0.097	0.651	0	0.705	0.583	0.146	0.892
TaMAPK36	TaMAPKK6	4565.A0A3B6NN33	4565.A0A3B6ILF0	0	0	0	0.632	0	0.705	0.583	0.146	0.885
TaMAPK36	TaMAPKK5	4565.A0A3B6NN33	4565.A0A3B6IMW7	0	0	0	0.636	0	0.705	0.583	0.146	0.885
TaMAPK36	TaMAPKK7	4565.A0A3B6NN33	4565.A0A3B6INV0	0	0	0	0.634	0	0.705	0.583	0.146	0.885
TaMAPK36	TaMAPKK11	4565.A0A3B6NN33	4565.A0A3B6JEH0	0	0	0.102	0.654	0	0.705	0.583	0.146	0.893
TaMAPK36	TaMAPKK12	4565.A0A3B6NN33	4565.A0A3B6JG06	0	0	0	0.626	0	0.705	0.583	0.146	0.885
TaMAPK36	TaMEKK14	4565.A0A3B6NN33	4565.A0A3B6KF43	0	0	0.107	0.608	0	0.222	0.186	0.065	0.4
TaMAPK36	TaMAPKK4	4565.A0A3B6NN33	4565.A0A3B6KFB5	0	0	0	0.605	0	0.705	0.825	0.195	0.954
TaMAPK36	TaMAPKK18	4565.A0A3B6NN33	4565.A0A3B6LJ27	0	0	0	0.588	0	0.705	0.825	0.195	0.954
TaMAPK36	TaMAPKK2	4565.A0A3B6NN33	4565.A0A3B6LYW0	0	0	0	0.593	0	0.705	0.825	0.195	0.954
TaMAPK36	TaMAPKK3	4565.A0A3B6NN33	4565.A0A3B6MNP8	0	0	0	0.586	0	0.705	0.825	0.195	0.954
TaMAPK36	TaMAPKK13	4565.A0A3B6NN33	4565.A0A3B6N2X8	0	0	0	0.589	0	0.705	0.825	0.195	0.954
TaMAPK36	TaRaf87	4565.A0A3B6NN33	4565.A0A3B6QMZ9	0	0	0	0.58	0.16	0.134	0.389	0.088	0.54
TaMAPK36	TaMAPK54	4565.A0A3B6NN33	4565.A0A3B6SKC9	0	0	0.048	0.984	0	0	0.793	0	0.794

TaMAPK36	TaMAPK47	4565.A0A3B6NN33	4565.A0A3B6RAZ7	0	0	0.053	0.974	0	0	0.793	0	0.795
TaMAPK36	TaMAPKK1	4565.A0A3B6NN33	4565.A0A3B6QJ87	0	0	0.106	0.608	0	0.705	0.583	0.146	0.893
TaMAPK38	TaMAPKK9	4565.A0A3B6FMN5	4565.A0A077RVQ4	0	0	0.112	0.617	0	0.705	0.583	0.146	0.894
TaMAPK38	TaMEKK5	4565.A0A3B6FMN5	4565.A0A3B6AWC1	0	0	0.14	0.584	0	0.222	0.186	0.065	0.422
TaMAPK38	TaMEKK2	4565.A0A3B6FMN5	4565.A0A3B6JCC4	0	0	0.127	0.575	0	0.222	0.186	0.065	0.414
TaMAPK38	TaMEKK16	4565.A0A3B6FMN5	4565.A0A3B6LW00	0	0	0.129	0.578	0	0.222	0.186	0.065	0.415
TaMAPK38	TaMEKK11	4565.A0A3B6FMN5	4565.A0A3B6N0D8	0	0	0.129	0.576	0	0.222	0.186	0.065	0.415
TaMAPK38	TaMEKK15	4565.A0A3B6FMN5	4565.A0A3B6KPK7	0	0	0.13	0.579	0	0.222	0.186	0.065	0.416
TaMAPK38	TaMEKK24	4565.A0A3B6FMN5	4565.A0A3B6LLV5	0	0	0.178	0.579	0	0.27	0.063	0.088	0.418
TaMAPK38	TaMEKK17	4565.A0A3B6FMN5	4565.A0A3B6KFL8	0	0	0.179	0.579	0	0.27	0.063	0.088	0.419
TaMAPK38	TaMEKK29	4565.A0A3B6FMN5	4565.A0A3B6MSP6	0	0	0.179	0.579	0	0.27	0.063	0.088	0.419
TaMAPK38	TaMEKK14	4565.A0A3B6FMN5	4565.A0A3B6KF43	0	0	0.139	0.586	0	0.222	0.186	0.065	0.422
TaMAPK38	TaRaf87	4565.A0A3B6FMN5	4565.A0A3B6QMZ9	0	0	0	0.562	0.277	0.134	0.389	0.088	0.604
TaMAPK38	TaMAPKK4	4565.A0A3B6FMN5	4565.A0A3B6KFB5	0	0	0	0.647	0	0.705	0.583	0.146	0.885
TaMAPK38	TaMAPKK18	4565.A0A3B6FMN5	4565.A0A3B6LJ27	0	0	0	0.589	0	0.705	0.583	0.146	0.885
TaMAPK38	TaMAPKK13	4565.A0A3B6FMN5	4565.A0A3B6N2X8	0	0	0	0.581	0	0.705	0.583	0.146	0.885
TaMAPK38	TaMAPKK2	4565.A0A3B6FMN5	4565.A0A3B6LYW0	0	0	0	0.585	0	0.705	0.583	0.146	0.885
TaMAPK38	TaMAPKK3	4565.A0A3B6FMN5	4565.A0A3B6MNP8	0	0	0	0.582	0	0.705	0.583	0.146	0.885

TaMAPK38	TaMAPKK14	4565.A0A3B6FMN5	4565.A0A3B6HY95	0	0	0.115	0.607	0	0.705	0.583	0.146	0.894
TaMAPK38	TaMAPKK11	4565.A0A3B6FMN5	4565.A0A3B6JEH0	0	0	0.115	0.614	0	0.705	0.583	0.146	0.894
TaMAPK38	TaMAPKK15	4565.A0A3B6FMN5	4565.A0A3B6HW51	0	0	0.117	0.614	0	0.705	0.583	0.146	0.894
TaMAPK38	TaMAPKK5	4565.A0A3B6FMN5	4565.A0A3B6IMW7	0	0	0.115	0.606	0	0.705	0.583	0.146	0.894
TaMAPK38	TaMAPKK16	4565.A0A3B6FMN5	4565.A0A3B6HZP7	0	0	0.115	0.616	0	0.705	0.583	0.146	0.894
TaMAPK38	TaMAPKK8	4565.A0A3B6FMN5	4565.A0A3B6IK39	0	0	0.111	0.616	0	0.705	0.583	0.146	0.894
TaMAPK38	TaMAPKK6	4565.A0A3B6FMN5	4565.A0A3B6ILF0	0	0	0.118	0.607	0	0.705	0.583	0.146	0.894
TaMAPK38	TaMAPKK17	4565.A0A3B6FMN5	4565.A0A3B6I0M7	0	0	0.117	0.611	0	0.705	0.583	0.146	0.894
TaMAPK38	TaMAPKK12	4565.A0A3B6FMN5	4565.A0A3B6JG06	0	0	0.121	0.605	0	0.705	0.583	0.146	0.895
TaMAPK38	TaMAPKK7	4565.A0A3B6FMN5	4565.A0A3B6INV0	0	0	0.12	0.609	0	0.705	0.583	0.146	0.895
TaMAPK38	TaMAPKK1	4565.A0A3B6FMN5	4565.A0A3B6QJ87	0	0	0.129	0.611	0	0.705	0.583	0.146	0.896
TaMAPK39	TaMAPKK9	4565.A0A3B6EGM9	4565.A0A077RVQ4	0	0	0.115	0.619	0	0.705	0.583	0.146	0.894
TaMAPK39	TaRaf88	4565.A0A3B6EGM9	4565.A0A3B5Z5X1	0	0	0.231	0	0	0.15	0.177	0.05	0.42
TaMAPK39	TaRaf30	4565.A0A3B6EGM9	4565.A0A3B6A1Z4	0	0	0.232	0	0	0.15	0.177	0.05	0.421
TaMAPK39	TaMEKK5	4565.A0A3B6EGM9	4565.A0A3B6AWC1	0	0	0.149	0.576	0	0.222	0.186	0.065	0.429
TaMAPK39	TaMEKK2	4565.A0A3B6EGM9	4565.A0A3B6JCC4	0	0	0.131	0.579	0	0.222	0.186	0.065	0.416
TaMAPK39	TaMEKK16	4565.A0A3B6EGM9	4565.A0A3B6LW00	0	0	0.137	0.571	0	0.222	0.186	0.065	0.42
TaMAPK39	TaMEKK11	4565.A0A3B6EGM9	4565.A0A3B6N0D8	0	0	0.136	0.57	0	0.222	0.186	0.065	0.42

TaMAPK39	TaMEKK15	4565.A0A3B6EGM9	4565.A0A3B6KPK7	0	0	0.138	0.572	0	0.222	0.186	0.065	0.421
TaMAPK39	TaMEKK17	4565.A0A3B6EGM9	4565.A0A3B6KFL8	0	0	0.189	0.573	0	0.27	0.063	0.088	0.426
TaMAPK39	TaMEKK24	4565.A0A3B6EGM9	4565.A0A3B6LLV5	0	0	0.188	0.573	0	0.27	0.063	0.088	0.426
TaMAPK39	TaMEKK29	4565.A0A3B6EGM9	4565.A0A3B6MSP6	0	0	0.189	0.572	0	0.27	0.063	0.088	0.426
TaMAPK39	TaMEKK14	4565.A0A3B6EGM9	4565.A0A3B6KF43	0	0	0.151	0.583	0	0.222	0.186	0.065	0.43
TaMAPK39	TaRaf87	4565.A0A3B6EGM9	4565.A0A3B6QMZ9	0	0	0	0.565	0.277	0.134	0.389	0.088	0.604
TaMAPK39	TaMAPKK4	4565.A0A3B6EGM9	4565.A0A3B6KFB5	0	0	0	0.638	0	0.705	0.583	0.146	0.885
TaMAPK39	TaMAPKK18	4565.A0A3B6EGM9	4565.A0A3B6LJ27	0	0	0	0.586	0	0.705	0.583	0.146	0.885
TaMAPK39	TaMAPKK13	4565.A0A3B6EGM9	4565.A0A3B6N2X8	0	0	0	0.587	0	0.705	0.583	0.146	0.885
TaMAPK39	TaMAPKK2	4565.A0A3B6EGM9	4565.A0A3B6LYW0	0	0	0	0.587	0	0.705	0.583	0.146	0.885
TaMAPK39	TaMAPKK3	4565.A0A3B6EGM9	4565.A0A3B6MNP8	0	0	0	0.581	0	0.705	0.583	0.146	0.885
TaMAPK39	TaMAPKK14	4565.A0A3B6EGM9	4565.A0A3B6HY95	0	0	0.117	0.616	0	0.705	0.583	0.146	0.894
TaMAPK39	TaMAPKK8	4565.A0A3B6EGM9	4565.A0A3B6IK39	0	0	0.114	0.618	0	0.705	0.583	0.146	0.894
TaMAPK39	TaMAPKK16	4565.A0A3B6EGM9	4565.A0A3B6HZP7	0	0	0.118	0.618	0	0.705	0.583	0.146	0.894
TaMAPK39	TaMAPKK5	4565.A0A3B6EGM9	4565.A0A3B6IMW7	0	0	0.115	0.61	0	0.705	0.583	0.146	0.894
TaMAPK39	TaMAPKK11	4565.A0A3B6EGM9	4565.A0A3B6JEH0	0	0	0.119	0.615	0	0.705	0.583	0.146	0.895
TaMAPK39	TaMAPKK12	4565.A0A3B6EGM9	4565.A0A3B6JG06	0	0	0.125	0.612	0	0.705	0.583	0.146	0.895
TaMAPK39	TaMAPKK7	4565.A0A3B6EGM9	4565.A0A3B6INV0	0	0	0.123	0.615	0	0.705	0.583	0.146	0.895

TaMAPK39	TaMAPKK15	4565.A0A3B6EGM9	4565.A0A3B6HW51	0	0	0.118	0.618	0	0.705	0.583	0.146	0.895
TaMAPK39	TaMAPKK6	4565.A0A3B6EGM9	4565.A0A3B6ILF0	0	0	0.122	0.611	0	0.705	0.583	0.146	0.895
TaMAPK39	TaMAPKK17	4565.A0A3B6EGM9	4565.A0A3B6I0M7	0	0	0.122	0.616	0	0.705	0.583	0.146	0.895
TaMAPK39	TaMAPKK1	4565.A0A3B6EGM9	4565.A0A3B6QJ87	0	0	0.13	0.613	0	0.705	0.583	0.146	0.896
TaMAPK4	TaMAPKK9	4565.A0A3B5ZRD7	4565.A0A077RVQ4	0	0	0.126	0.622	0	0.705	0.583	0.146	0.895
TaMAPK4	TaMEKK29	4565.A0A3B5ZRD7	4565.A0A3B6MSP6	0	0	0.178	0.589	0	0.27	0.063	0.088	0.418
TaMAPK4	TaMEKK24	4565.A0A3B5ZRD7	4565.A0A3B6LLV5	0	0	0.177	0.589	0	0.27	0.063	0.088	0.418
TaMAPK4	TaMEKK17	4565.A0A3B5ZRD7	4565.A0A3B6KFL8	0	0	0.178	0.589	0	0.27	0.063	0.088	0.418
TaMAPK4	TaMEKK2	4565.A0A3B5ZRD7	4565.A0A3B6JCC4	0	0	0.135	0.568	0	0.222	0.186	0.065	0.419
TaMAPK4	TaMEKK11	4565.A0A3B5ZRD7	4565.A0A3B6N0D8	0	0	0.148	0.57	0	0.222	0.186	0.065	0.428
TaMAPK4	TaMEKK16	4565.A0A3B5ZRD7	4565.A0A3B6LW00	0	0	0.149	0.572	0	0.222	0.186	0.065	0.429
TaMAPK4	TaMEKK14	4565.A0A3B5ZRD7	4565.A0A3B6KF43	0	0	0.152	0.581	0	0.222	0.186	0.065	0.43
TaMAPK4	TaMEKK15	4565.A0A3B5ZRD7	4565.A0A3B6KPK7	0	0	0.151	0.573	0	0.222	0.186	0.065	0.43
TaMAPK4	TaMEKK5	4565.A0A3B5ZRD7	4565.A0A3B6AWC1	0	0	0.158	0.574	0	0.222	0.186	0.065	0.435
TaMAPK4	TaRaf87	4565.A0A3B5ZRD7	4565.A0A3B6QMZ9	0	0	0	0.565	0.3	0.134	0.389	0.088	0.617
TaMAPK4	TaMAPKK8	4565.A0A3B5ZRD7	4565.A0A3B6IK39	0	0	0.123	0.615	0	0.705	0.583	0.146	0.895
TaMAPK4	TaMAPKK15	4565.A0A3B5ZRD7	4565.A0A3B6HW51	0	0	0.133	0.615	0	0.705	0.583	0.146	0.896
TaMAPK4	TaMAPKK5	4565.A0A3B5ZRD7	4565.A0A3B6IMW7	0	0	0.127	0.609	0	0.705	0.583	0.146	0.896

TaMAPK4	TaMAPKK16	4565.A0A3B5ZRD7	4565.A0A3B6HZP7	0	0	0.131	0.621	0	0.705	0.583	0.146	0.896
TaMAPK4	TaMAPKK1	4565.A0A3B5ZRD7	4565.A0A3B6QJ87	0	0	0.134	0.615	0	0.705	0.583	0.146	0.896
TaMAPK4	TaMAPKK14	4565.A0A3B5ZRD7	4565.A0A3B6HY95	0	0	0.133	0.61	0	0.705	0.583	0.146	0.896
TaMAPK4	TaMAPKK11	4565.A0A3B5ZRD7	4565.A0A3B6JEH0	0	0	0.127	0.62	0	0.705	0.583	0.146	0.896
TaMAPK4	TaMAPKK7	4565.A0A3B5ZRD7	4565.A0A3B6INV0	0	0	0.138	0.611	0	0.705	0.583	0.146	0.897
TaMAPK4	TaMAPKK17	4565.A0A3B5ZRD7	4565.A0A3B6I0M7	0	0	0.136	0.613	0	0.705	0.583	0.146	0.897
TaMAPK4	TaMAPKK6	4565.A0A3B5ZRD7	4565.A0A3B6ILF0	0	0	0.138	0.607	0	0.705	0.583	0.146	0.897
TaMAPK4	TaMAPKK12	4565.A0A3B5ZRD7	4565.A0A3B6JG06	0	0	0.144	0.608	0	0.705	0.583	0.146	0.898
TaMAPK4	TaMAPKK13	4565.A0A3B5ZRD7	4565.A0A3B6N2X8	0	0	0.108	0.58	0	0.705	0.612	0.146	0.901
TaMAPK4	TaMAPKK2	4565.A0A3B5ZRD7	4565.A0A3B6LYW0	0	0	0.123	0.581	0	0.705	0.612	0.146	0.902
TaMAPK4	TaMAPKK3	4565.A0A3B5ZRD7	4565.A0A3B6MNP8	0	0	0.131	0.577	0	0.705	0.612	0.146	0.903
TaMAPK4	TaMAPKK4	4565.A0A3B5ZRD7	4565.A0A3B6KFB5	0	0	0.13	0.629	0	0.705	0.612	0.146	0.903
TaMAPK4	TaMAPKK18	4565.A0A3B5ZRD7	4565.A0A3B6LJ27	0	0	0.137	0.582	0	0.705	0.612	0.146	0.904
TaMAPK40	TaMAPKK9	4565.A0A3B6EH95	4565.A0A077RVQ4	0	0	0.114	0.622	0	0.705	0.583	0.146	0.894
TaMAPK40	TaRaf88	4565.A0A3B6EH95	4565.A0A3B5Z5X1	0	0	0.29	0	0	0.15	0.177	0.05	0.465
TaMAPK40	TaRaf30	4565.A0A3B6EH95	4565.A0A3B6A1Z4	0	0	0.292	0	0	0.15	0.177	0.05	0.466
TaMAPK40	TaMEKK5	4565.A0A3B6EH95	4565.A0A3B6AWC1	0	0	0.151	0.586	0	0.222	0.186	0.065	0.43
TaMAPK40	TaMEKK24	4565.A0A3B6EH95	4565.A0A3B6LLV5	0	0	0.156	0.609	0	0.27	0.063	0.088	0.403

TaMAPK40	TaMEKK29	4565.A0A3B6EH95	4565.A0A3B6MSP6	0	0	0.157	0.609	0	0.27	0.063	0.088	0.404
TaMAPK40	TaMEKK17	4565.A0A3B6EH95	4565.A0A3B6KFL8	0	0	0.157	0.609	0	0.27	0.063	0.088	0.404
TaMAPK40	TaMEKK2	4565.A0A3B6EH95	4565.A0A3B6JCC4	0	0	0.135	0.582	0	0.222	0.186	0.065	0.419
TaMAPK40	TaMEKK11	4565.A0A3B6EH95	4565.A0A3B6N0D8	0	0	0.144	0.583	0	0.222	0.186	0.065	0.425
TaMAPK40	TaMEKK15	4565.A0A3B6EH95	4565.A0A3B6KPK7	0	0	0.146	0.585	0	0.222	0.186	0.065	0.426
TaMAPK40	TaMEKK16	4565.A0A3B6EH95	4565.A0A3B6LW00	0	0	0.145	0.585	0	0.222	0.186	0.065	0.426
TaMAPK40	TaMEKK14	4565.A0A3B6EH95	4565.A0A3B6KF43	0	0	0.151	0.594	0	0.222	0.186	0.065	0.43
TaMAPK40	TaRaf87	4565.A0A3B6EH95	4565.A0A3B6QMZ9	0	0	0	0.572	0.3	0.134	0.389	0.088	0.617
TaMAPK40	TaMAPKK2	4565.A0A3B6EH95	4565.A0A3B6LYW0	0	0	0	0.582	0	0.705	0.612	0.146	0.893
TaMAPK40	TaMAPKK3	4565.A0A3B6EH95	4565.A0A3B6MNP8	0	0	0	0.58	0	0.705	0.612	0.146	0.893
TaMAPK40	TaMAPKK13	4565.A0A3B6EH95	4565.A0A3B6N2X8	0	0	0	0.582	0	0.705	0.612	0.146	0.893
TaMAPK40	TaMAPKK18	4565.A0A3B6EH95	4565.A0A3B6LJ27	0	0	0	0.579	0	0.705	0.612	0.146	0.893
TaMAPK40	TaMAPKK8	4565.A0A3B6EH95	4565.A0A3B6IK39	0	0	0.112	0.615	0	0.705	0.583	0.146	0.894
TaMAPK40	TaMAPKK5	4565.A0A3B6EH95	4565.A0A3B6IMW7	0	0	0.117	0.609	0	0.705	0.583	0.146	0.894
TaMAPK40	TaMAPKK11	4565.A0A3B6EH95	4565.A0A3B6JEH0	0	0	0.115	0.618	0	0.705	0.583	0.146	0.894
TaMAPK40	TaMAPKK7	4565.A0A3B6EH95	4565.A0A3B6INV0	0	0	0.124	0.611	0	0.705	0.583	0.146	0.895
TaMAPK40	TaMAPKK15	4565.A0A3B6EH95	4565.A0A3B6HW51	0	0	0.12	0.616	0	0.705	0.583	0.146	0.895
TaMAPK40	TaMAPKK17	4565.A0A3B6EH95	4565.A0A3B6I0M7	0	0	0.122	0.613	0	0.705	0.583	0.146	0.895

TaMAPK40	TaMAPKK6	4565.A0A3B6EH95	4565.A0A3B6ILF0	0	0	0.122	0.611	0	0.705	0.583	0.146	0.895
TaMAPK40	TaMAPKK16	4565.A0A3B6EH95	4565.A0A3B6HZP7	0	0	0.118	0.622	0	0.705	0.583	0.146	0.895
TaMAPK40	TaMAPKK14	4565.A0A3B6EH95	4565.A0A3B6HY95	0	0	0.121	0.612	0	0.705	0.583	0.146	0.895
TaMAPK40	TaMAPKK1	4565.A0A3B6EH95	4565.A0A3B6QJ87	0	0	0.128	0.616	0	0.705	0.583	0.146	0.896
TaMAPK40	TaMAPKK12	4565.A0A3B6EH95	4565.A0A3B6JG06	0	0	0.129	0.607	0	0.705	0.583	0.146	0.896
TaMAPK40	TaMAPKK4	4565.A0A3B6EH95	4565.A0A3B6KFB5	0	0	0.108	0.628	0	0.705	0.612	0.146	0.901
TaMAPK41	TaMAPKK9	4565.A0A3B5Y6A3	4565.A0A077RVQ4	0	0	0.119	0.619	0	0.705	0.583	0.146	0.895
TaMAPK41	TaMEKK24	4565.A0A3B5Y6A3	4565.A0A3B6LLV5	0	0	0.177	0.582	0	0.27	0.063	0.088	0.418
TaMAPK41	TaMEKK17	4565.A0A3B5Y6A3	4565.A0A3B6KFL8	0	0	0.178	0.582	0	0.27	0.063	0.088	0.418
TaMAPK41	TaMEKK2	4565.A0A3B5Y6A3	4565.A0A3B6JCC4	0	0	0.133	0.577	0	0.222	0.186	0.065	0.418
TaMAPK41	TaMEKK29	4565.A0A3B5Y6A3	4565.A0A3B6MSP6	0	0	0.178	0.582	0	0.27	0.063	0.088	0.419
TaMAPK41	TaMEKK11	4565.A0A3B5Y6A3	4565.A0A3B6N0D8	0	0	0.142	0.572	0	0.222	0.186	0.065	0.423
TaMAPK41	TaMEKK16	4565.A0A3B5Y6A3	4565.A0A3B6LW00	0	0	0.142	0.573	0	0.222	0.186	0.065	0.424
TaMAPK41	TaMEKK15	4565.A0A3B5Y6A3	4565.A0A3B6KPK7	0	0	0.144	0.574	0	0.222	0.186	0.065	0.425
TaMAPK41	TaMEKK5	4565.A0A3B5Y6A3	4565.A0A3B6AWC1	0	0	0.151	0.58	0	0.222	0.186	0.065	0.43
TaMAPK41	TaMEKK14	4565.A0A3B5Y6A3	4565.A0A3B6KF43	0	0	0.153	0.584	0	0.222	0.186	0.065	0.431
TaMAPK41	TaRaf30	4565.A0A3B5Y6A3	4565.A0A3B6A1Z4	0	0	0.267	0	0	0.15	0.177	0.05	0.447
TaMAPK41	TaRaf88	4565.A0A3B5Y6A3	4565.A0A3B5Z5X1	0	0	0.267	0	0	0.15	0.177	0.05	0.447

TaMAPK41	TaRaf87	4565.A0A3B5Y6A3	4565.A0A3B6QMZ9	0	0	0	0.57	0.277	0.134	0.389	0.088	0.604
TaMAPK41	TaMAPKK3	4565.A0A3B5Y6A3	4565.A0A3B6MNP8	0	0	0	0.579	0	0.705	0.583	0.146	0.885
TaMAPK41	TaMAPKK2	4565.A0A3B5Y6A3	4565.A0A3B6LYW0	0	0	0	0.583	0	0.705	0.583	0.146	0.885
TaMAPK41	TaMAPKK13	4565.A0A3B5Y6A3	4565.A0A3B6N2X8	0	0	0	0.582	0	0.705	0.583	0.146	0.885
TaMAPK41	TaMAPKK18	4565.A0A3B5Y6A3	4565.A0A3B6LJ27	0	0	0	0.585	0	0.705	0.583	0.146	0.885
TaMAPK41	TaMAPKK4	4565.A0A3B5Y6A3	4565.A0A3B6KFB5	0	0	0.102	0.638	0	0.705	0.583	0.146	0.893
TaMAPK41	TaMAPKK8	4565.A0A3B5Y6A3	4565.A0A3B6IK39	0	0	0.116	0.621	0	0.705	0.583	0.146	0.894
TaMAPK41	TaMAPKK17	4565.A0A3B5Y6A3	4565.A0A3B6I0M7	0	0	0.121	0.619	0	0.705	0.583	0.146	0.895
TaMAPK41	TaMAPKK6	4565.A0A3B5Y6A3	4565.A0A3B6ILF0	0	0	0.122	0.617	0	0.705	0.583	0.146	0.895
TaMAPK41	TaMAPKK16	4565.A0A3B5Y6A3	4565.A0A3B6HZP7	0	0	0.122	0.622	0	0.705	0.583	0.146	0.895
TaMAPK41	TaMAPKK5	4565.A0A3B5Y6A3	4565.A0A3B6IMW7	0	0	0.119	0.609	0	0.705	0.583	0.146	0.895
TaMAPK41	TaMAPKK7	4565.A0A3B5Y6A3	4565.A0A3B6INV0	0	0	0.124	0.618	0	0.705	0.583	0.146	0.895
TaMAPK41	TaMAPKK15	4565.A0A3B5Y6A3	4565.A0A3B6HW51	0	0	0.122	0.621	0	0.705	0.583	0.146	0.895
TaMAPK41	TaMAPKK11	4565.A0A3B5Y6A3	4565.A0A3B6JEH0	0	0	0.12	0.62	0	0.705	0.583	0.146	0.895
TaMAPK41	TaMAPKK14	4565.A0A3B5Y6A3	4565.A0A3B6HY95	0	0	0.122	0.618	0	0.705	0.583	0.146	0.895
TaMAPK41	TaMAPKK12	4565.A0A3B5Y6A3	4565.A0A3B6JG06	0	0	0.127	0.614	0	0.705	0.583	0.146	0.896
TaMAPK41	TaMAPKK1	4565.A0A3B5Y6A3	4565.A0A3B6QJ87	0	0	0.131	0.615	0	0.705	0.583	0.146	0.896
TaMAPK42	TaMAPKK9	4565.A0A3B5Y6P8	4565.A0A077RVQ4	0	0	0.115	0.63	0	0.705	0.583	0.146	0.894

TaMAPK42	TaMEKK24	4565.A0A3B5Y6P8	4565.A0A3B6LLV5	0	0	0.176	0.582	0	0.27	0.063	0.088	0.417
TaMAPK42	TaMEKK17	4565.A0A3B5Y6P8	4565.A0A3B6KFL8	0	0	0.176	0.582	0	0.27	0.063	0.088	0.417
TaMAPK42	TaMEKK29	4565.A0A3B5Y6P8	4565.A0A3B6MSP6	0	0	0.177	0.582	0	0.27	0.063	0.088	0.418
TaMAPK42	TaMEKK2	4565.A0A3B5Y6P8	4565.A0A3B6JCC4	0	0	0.138	0.576	0	0.222	0.186	0.065	0.421
TaMAPK42	TaMEKK11	4565.A0A3B5Y6P8	4565.A0A3B6N0D8	0	0	0.151	0.577	0	0.222	0.186	0.065	0.43
TaMAPK42	TaMEKK16	4565.A0A3B5Y6P8	4565.A0A3B6LW00	0	0	0.153	0.579	0	0.222	0.186	0.065	0.431
TaMAPK42	TaMEKK15	4565.A0A3B5Y6P8	4565.A0A3B6KPK7	0	0	0.154	0.579	0	0.222	0.186	0.065	0.432
TaMAPK42	TaMEKK5	4565.A0A3B5Y6P8	4565.A0A3B6AWC1	0	0	0.158	0.578	0	0.222	0.186	0.065	0.434
TaMAPK42	TaMEKK14	4565.A0A3B5Y6P8	4565.A0A3B6KF43	0	0	0.158	0.59	0	0.222	0.186	0.065	0.435
TaMAPK42	TaRaf87	4565.A0A3B5Y6P8	4565.A0A3B6QMZ9	0	0	0	0.564	0.3	0.134	0.389	0.088	0.617
TaMAPK42	TaMAPKK2	4565.A0A3B5Y6P8	4565.A0A3B6LYW0	0	0	0	0.578	0	0.705	0.612	0.146	0.893
TaMAPK42	TaMAPKK3	4565.A0A3B5Y6P8	4565.A0A3B6MNP8	0	0	0	0.577	0	0.705	0.612	0.146	0.893
TaMAPK42	TaMAPKK13	4565.A0A3B5Y6P8	4565.A0A3B6N2X8	0	0	0	0.578	0	0.705	0.612	0.146	0.893
TaMAPK42	TaMAPKK18	4565.A0A3B5Y6P8	4565.A0A3B6LJ27	0	0	0	0.583	0	0.705	0.612	0.146	0.893
TaMAPK42	TaMAPKK8	4565.A0A3B5Y6P8	4565.A0A3B6IK39	0	0	0.116	0.619	0	0.705	0.583	0.146	0.894
TaMAPK42	TaMAPKK7	4565.A0A3B5Y6P8	4565.A0A3B6INV0	0	0	0.125	0.615	0	0.705	0.583	0.146	0.895
TaMAPK42	TaMAPKK15	4565.A0A3B5Y6P8	4565.A0A3B6HW51	0	0	0.122	0.623	0	0.705	0.583	0.146	0.895
TaMAPK42	TaMAPKK17	4565.A0A3B5Y6P8	4565.A0A3B6I0M7	0	0	0.124	0.616	0	0.705	0.583	0.146	0.895

TaMAPK42	TaMAPKK6	4565.A0A3B5Y6P8	4565.A0A3B6ILF0	0	0	0.123	0.612	0	0.705	0.583	0.146	0.895
TaMAPK42	TaMAPKK16	4565.A0A3B5Y6P8	4565.A0A3B6HZP7	0	0	0.121	0.624	0	0.705	0.583	0.146	0.895
TaMAPK42	TaMAPKK5	4565.A0A3B5Y6P8	4565.A0A3B6IMW7	0	0	0.119	0.616	0	0.705	0.583	0.146	0.895
TaMAPK42	TaMAPKK14	4565.A0A3B5Y6P8	4565.A0A3B6HY95	0	0	0.123	0.616	0	0.705	0.583	0.146	0.895
TaMAPK42	TaMAPKK11	4565.A0A3B5Y6P8	4565.A0A3B6JEH0	0	0	0.118	0.626	0	0.705	0.583	0.146	0.895
TaMAPK42	TaMAPKK1	4565.A0A3B5Y6P8	4565.A0A3B6QJ87	0	0	0.135	0.617	0	0.705	0.583	0.146	0.896
TaMAPK42	TaMAPKK12	4565.A0A3B5Y6P8	4565.A0A3B6JG06	0	0	0.131	0.613	0	0.705	0.583	0.146	0.896
TaMAPK42	TaMAPKK4	4565.A0A3B5Y6P8	4565.A0A3B6KFB5	0	0	0.107	0.63	0	0.705	0.612	0.146	0.901
TaMAPK43	TaMAPKK9	4565.A0A3B5Y7D4	4565.A0A077RVQ4	0	0	0.117	0.618	0	0.705	0.583	0.146	0.894
TaMAPK43	TaMEKK2	4565.A0A3B5Y7D4	4565.A0A3B6JCC4	0	0	0.128	0.574	0	0.222	0.186	0.065	0.415
TaMAPK43	TaMEKK11	4565.A0A3B5Y7D4	4565.A0A3B6N0D8	0	0	0.133	0.572	0	0.222	0.186	0.065	0.417
TaMAPK43	TaMEKK17	4565.A0A3B5Y7D4	4565.A0A3B6KFL8	0	0	0.178	0.576	0	0.27	0.063	0.088	0.418
TaMAPK43	TaMEKK16	4565.A0A3B5Y7D4	4565.A0A3B6LW00	0	0	0.133	0.573	0	0.222	0.186	0.065	0.418
TaMAPK43	TaMEKK24	4565.A0A3B5Y7D4	4565.A0A3B6LLV5	0	0	0.177	0.576	0	0.27	0.063	0.088	0.418
TaMAPK43	TaMEKK29	4565.A0A3B5Y7D4	4565.A0A3B6MSP6	0	0	0.178	0.576	0	0.27	0.063	0.088	0.418
TaMAPK43	TaMEKK15	4565.A0A3B5Y7D4	4565.A0A3B6KPK7	0	0	0.135	0.574	0	0.222	0.186	0.065	0.419
TaMAPK43	TaMEKK5	4565.A0A3B5Y7D4	4565.A0A3B6AWC1	0	0	0.141	0.582	0	0.222	0.186	0.065	0.423
TaMAPK43	TaMEKK14	4565.A0A3B5Y7D4	4565.A0A3B6KF43	0	0	0.142	0.581	0	0.222	0.186	0.065	0.424

TaMAPK43	TaRaf87	4565.A0A3B5Y7D4	4565.A0A3B6QMZ9	0	0	0	0.56	0.277	0.134	0.389	0.088	0.604
TaMAPK43	TaMAPKK18	4565.A0A3B5Y7D4	4565.A0A3B6LJ27	0	0	0	0.589	0	0.705	0.583	0.146	0.885
TaMAPK43	TaMAPKK13	4565.A0A3B5Y7D4	4565.A0A3B6N2X8	0	0	0	0.583	0	0.705	0.583	0.146	0.885
TaMAPK43	TaMAPKK2	4565.A0A3B5Y7D4	4565.A0A3B6LYW0	0	0	0	0.585	0	0.705	0.583	0.146	0.885
TaMAPK43	TaMAPKK3	4565.A0A3B5Y7D4	4565.A0A3B6MNP8	0	0	0	0.583	0	0.705	0.583	0.146	0.885
TaMAPK43	TaMAPKK4	4565.A0A3B5Y7D4	4565.A0A3B6KFB5	0	0	0.097	0.644	0	0.705	0.583	0.146	0.892
TaMAPK43	TaMAPKK11	4565.A0A3B5Y7D4	4565.A0A3B6JEH0	0	0	0.118	0.614	0	0.705	0.583	0.146	0.894
TaMAPK43	TaMAPKK8	4565.A0A3B5Y7D4	4565.A0A3B6IK39	0	0	0.116	0.612	0	0.705	0.583	0.146	0.894
TaMAPK43	TaMAPKK14	4565.A0A3B5Y7D4	4565.A0A3B6HY95	0	0	0.119	0.611	0	0.705	0.583	0.146	0.895
TaMAPK43	TaMAPKK12	4565.A0A3B5Y7D4	4565.A0A3B6JG06	0	0	0.122	0.608	0	0.705	0.583	0.146	0.895
TaMAPK43	TaMAPKK15	4565.A0A3B5Y7D4	4565.A0A3B6HW51	0	0	0.121	0.617	0	0.705	0.583	0.146	0.895
TaMAPK43	TaMAPKK7	4565.A0A3B5Y7D4	4565.A0A3B6INV0	0	0	0.122	0.611	0	0.705	0.583	0.146	0.895
TaMAPK43	TaMAPKK5	4565.A0A3B5Y7D4	4565.A0A3B6IMW7	0	0	0.119	0.608	0	0.705	0.583	0.146	0.895
TaMAPK43	TaMAPKK16	4565.A0A3B5Y7D4	4565.A0A3B6HZP7	0	0	0.12	0.616	0	0.705	0.583	0.146	0.895
TaMAPK43	TaMAPKK6	4565.A0A3B5Y7D4	4565.A0A3B6ILF0	0	0	0.119	0.611	0	0.705	0.583	0.146	0.895
TaMAPK43	TaMAPKK17	4565.A0A3B5Y7D4	4565.A0A3B6I0M7	0	0	0.119	0.612	0	0.705	0.583	0.146	0.895
TaMAPK43	TaMAPKK1	4565.A0A3B5Y7D4	4565.A0A3B6QJ87	0	0	0.127	0.605	0	0.705	0.583	0.146	0.896
TaMAPK44	TaMAPKK9	4565.A0A3B6QDH5	4565.A0A077RVQ4	0	0	0.137	0.618	0	0.705	0.583	0.146	0.897

TaMAPK44	TaMEKK5	4565.A0A3B6QDH5	4565.A0A3B6AWC1	0	0	0.169	0.573	0	0.222	0.186	0.065	0.442
TaMAPK44	TaMEKK1	4565.A0A3B6QDH5	4565.A0A3B6B3I4	0	0	0.157	0.612	0	0.27	0.063	0.088	0.403
TaMAPK44	TaMAPKK15	4565.A0A3B6QDH5	4565.A0A3B6HW51	0	0	0.143	0.615	0	0.705	0.583	0.146	0.898
TaMAPK44	TaMAPKK14	4565.A0A3B6QDH5	4565.A0A3B6HY95	0	0	0.142	0.608	0	0.705	0.583	0.146	0.897
TaMAPK44	TaMAPKK16	4565.A0A3B6QDH5	4565.A0A3B6HZP7	0	0	0.138	0.622	0	0.705	0.583	0.146	0.897
TaMAPK44	TaMAPKK17	4565.A0A3B6QDH5	4565.A0A3B6I0M7	0	0	0.141	0.606	0	0.705	0.583	0.146	0.897
TaMAPK44	TaMAPKK8	4565.A0A3B6QDH5	4565.A0A3B6IK39	0	0	0.132	0.613	0	0.705	0.583	0.146	0.896
TaMAPK44	TaMAPKK6	4565.A0A3B6QDH5	4565.A0A3B6ILF0	0	0	0.135	0.613	0	0.705	0.583	0.146	0.897
TaMAPK44	TaMAPKK5	4565.A0A3B6QDH5	4565.A0A3B6IMW7	0	0	0.136	0.606	0	0.705	0.583	0.146	0.897
TaMAPK44	TaMAPKK7	4565.A0A3B6QDH5	4565.A0A3B6INV0	0	0	0.142	0.603	0	0.705	0.583	0.146	0.897
TaMAPK44	TaMEKK2	4565.A0A3B6QDH5	4565.A0A3B6JCC4	0	0	0.15	0.572	0	0.222	0.186	0.065	0.429
TaMAPK44	TaMAPKK11	4565.A0A3B6QDH5	4565.A0A3B6JEH0	0	0	0.137	0.618	0	0.705	0.583	0.146	0.897
TaMAPK44	TaMAPKK12	4565.A0A3B6QDH5	4565.A0A3B6JG06	0	0	0.145	0.603	0	0.705	0.583	0.146	0.898
TaMAPK44	TaMEKK14	4565.A0A3B6QDH5	4565.A0A3B6KF43	0	0	0.165	0.579	0	0.222	0.186	0.065	0.439
TaMAPK44	TaMAPKK4	4565.A0A3B6QDH5	4565.A0A3B6KFB5	0	0	0.113	0.629	0	0.705	0.612	0.146	0.901
TaMAPK44	TaMEKK17	4565.A0A3B6QDH5	4565.A0A3B6KFL8	0	0	0.176	0.601	0	0.27	0.063	0.088	0.417
TaMAPK44	TaMEKK15	4565.A0A3B6QDH5	4565.A0A3B6KPK7	0	0	0.163	0.572	0	0.222	0.186	0.065	0.438
TaMAPK44	TaMAPKK18	4565.A0A3B6QDH5	4565.A0A3B6LJ27	0	0	0.113	0.581	0	0.705	0.612	0.146	0.901

TaMAPK44	TaMEKK24	4565.A0A3B6QDH5	4565.A0A3B6LLV5	0	0	0.175	0.601	0	0.27	0.063	0.088	0.416
TaMAPK44	TaMEKK16	4565.A0A3B6QDH5	4565.A0A3B6LW00	0	0	0.162	0.572	0	0.222	0.186	0.065	0.437
TaMAPK44	TaMAPKK2	4565.A0A3B6QDH5	4565.A0A3B6LYW0	0	0	0	0.58	0	0.705	0.612	0.146	0.893
TaMAPK44	TaMAPKK3	4565.A0A3B6QDH5	4565.A0A3B6MNP8	0	0	0	0.578	0	0.705	0.612	0.146	0.893
TaMAPK44	TaMEKK29	4565.A0A3B6QDH5	4565.A0A3B6MSP6	0	0	0.176	0.601	0	0.27	0.063	0.088	0.417
TaMAPK44	TaMEKK11	4565.A0A3B6QDH5	4565.A0A3B6N0D8	0	0	0.161	0.57	0	0.222	0.186	0.065	0.437
TaMAPK44	TaMAPKK13	4565.A0A3B6QDH5	4565.A0A3B6N2X8	0	0	0	0.579	0	0.705	0.612	0.146	0.893
TaMAPK44	TaMEKK4	4565.A0A3B6QDH5	4565.A0A3B6NRN9	0	0	0.158	0.596	0	0.27	0.063	0.088	0.404
TaMAPK44	TaMEKK4-1	4565.A0A3B6QDH5	4565.A0A3B6PNI6	0	0	0.16	0.599	0	0.27	0.063	0.088	0.406
TaMAPK44	TaRaf87	4565.A0A3B6QDH5	4565.A0A3B6QMZ9	0	0	0	0.58	0.3	0.134	0.389	0.088	0.617
TaMAPK44	TaMAPKK1	4565.A0A3B6QDH5	4565.A0A3B6QJ87	0	0	0.145	0.605	0	0.705	0.583	0.146	0.898
TaMAPK45	TaMAPKK9	4565.A0A3B6QBD2	4565.A0A077RVQ4	0	0	0	0.651	0	0.705	0.583	0.146	0.885
TaMAPK45	TaMAPKK15	4565.A0A3B6QBD2	4565.A0A3B6HW51	0	0	0	0.643	0	0.705	0.583	0.146	0.885
TaMAPK45	TaMAPKK14	4565.A0A3B6QBD2	4565.A0A3B6HY95	0	0	0	0.636	0	0.705	0.583	0.146	0.885
TaMAPK45	TaMAPKK16	4565.A0A3B6QBD2	4565.A0A3B6HZP7	0	0	0	0.651	0	0.705	0.583	0.146	0.885
TaMAPK45	TaMAPKK17	4565.A0A3B6QBD2	4565.A0A3B6I0M7	0	0	0	0.623	0	0.705	0.583	0.146	0.885
TaMAPK45	TaMAPKK8	4565.A0A3B6QBD2	4565.A0A3B6IK39	0	0	0	0.642	0	0.705	0.583	0.146	0.885
TaMAPK45	TaMAPKK6	4565.A0A3B6QBD2	4565.A0A3B6ILF0	0	0	0	0.623	0	0.705	0.583	0.146	0.885

TaMAPK45	TaMAPKK5	4565.A0A3B6QBD2	4565.A0A3B6IMW7	0	0	0	0.627	0	0.705	0.583	0.146	0.885
TaMAPK45	TaMAPKK7	4565.A0A3B6QBD2	4565.A0A3B6INV0	0	0	0	0.623	0	0.705	0.583	0.146	0.885
TaMAPK45	TaMAPKK11	4565.A0A3B6QBD2	4565.A0A3B6JEH0	0	0	0.101	0.642	0	0.705	0.583	0.146	0.892
TaMAPK45	TaMAPKK12	4565.A0A3B6QBD2	4565.A0A3B6JG06	0	0	0	0.617	0	0.705	0.583	0.146	0.885
TaMAPK45	TaMAPKK4	4565.A0A3B6QBD2	4565.A0A3B6KFB5	0	0	0	0.606	0	0.705	0.825	0.195	0.954
TaMAPK45	TaMAPKK18	4565.A0A3B6QBD2	4565.A0A3B6LJ27	0	0	0	0.589	0	0.705	0.825	0.195	0.954
TaMAPK45	TaMAPKK2	4565.A0A3B6QBD2	4565.A0A3B6LYW0	0	0	0	0.592	0	0.705	0.825	0.195	0.954
TaMAPK45	TaMAPKK3	4565.A0A3B6QBD2	4565.A0A3B6MNP8	0	0	0	0.588	0	0.705	0.825	0.195	0.954
TaMAPK45	TaMAPKK13	4565.A0A3B6QBD2	4565.A0A3B6N2X8	0	0	0	0.588	0	0.705	0.825	0.195	0.954
TaMAPK45	TaRaf87	4565.A0A3B6QBD2	4565.A0A3B6QMZ9	0	0	0	0.578	0.16	0.134	0.389	0.088	0.54
TaMAPK45	TaMAPK54	4565.A0A3B6QBD2	4565.A0A3B6SKC9	0	0	0.048	0.984	0	0	0.793	0	0.794
TaMAPK45	TaMAPK47	4565.A0A3B6QBD2	4565.A0A3B6RAZ7	0	0	0.053	0.974	0	0	0.793	0	0.795
TaMAPK45	TaMAPKK1	4565.A0A3B6QBD2	4565.A0A3B6QJ87	0	0	0	0.602	0	0.705	0.583	0.146	0.885
TaMAPK47	TaMAPKK9	4565.A0A3B6RAZ7	4565.A0A077RVQ4	0	0	0.153	0.651	0	0.705	0.583	0.146	0.899
TaMAPK47	TaMEKK5	4565.A0A3B6RAZ7	4565.A0A3B6AWC1	0	0	0.137	0.649	0	0.222	0.186	0.065	0.42
TaMAPK47	TaMEKK1	4565.A0A3B6RAZ7	4565.A0A3B6B3I4	0	0	0.156	0.66	0	0.27	0.063	0.088	0.403
TaMAPK47	TaMAPKK15	4565.A0A3B6RAZ7	4565.A0A3B6HW51	0	0	0.161	0.646	0	0.705	0.583	0.146	0.9
TaMAPK47	TaMAPKK14	4565.A0A3B6RAZ7	4565.A0A3B6HY95	0	0	0.161	0.643	0	0.705	0.583	0.146	0.9

TaMAPK47	TaMAPKK16	4565.A0A3B6RAZ7	4565.A0A3B6HZP7	0	0	0.16	0.643	0	0.705	0.583	0.146	0.9
TaMAPK47	TaMAPKK17	4565.A0A3B6RAZ7	4565.A0A3B6I0M7	0	0	0.156	0.63	0	0.705	0.583	0.146	0.899
TaMAPK47	TaMAPKK8	4565.A0A3B6RAZ7	4565.A0A3B6IK39	0	0	0.155	0.642	0	0.705	0.583	0.146	0.899
TaMAPK47	TaMAPKK6	4565.A0A3B6RAZ7	4565.A0A3B6ILF0	0	0	0.155	0.628	0	0.705	0.583	0.146	0.899
TaMAPK47	TaMAPKK5	4565.A0A3B6RAZ7	4565.A0A3B6IMW7	0	0	0.159	0.639	0	0.705	0.583	0.146	0.899
TaMAPK47	TaMAPKK7	4565.A0A3B6RAZ7	4565.A0A3B6INV0	0	0	0.155	0.629	0	0.705	0.583	0.146	0.899
TaMAPK47	TaMEKK2	4565.A0A3B6RAZ7	4565.A0A3B6JCC4	0	0	0.123	0.618	0	0.222	0.186	0.065	0.411
TaMAPK47	TaMAPKK11	4565.A0A3B6RAZ7	4565.A0A3B6JEH0	0	0	0.164	0.639	0	0.705	0.583	0.146	0.9
TaMAPK47	TaMAPKK12	4565.A0A3B6RAZ7	4565.A0A3B6JG06	0	0	0.159	0.628	0	0.705	0.583	0.146	0.899
TaMAPK47	TaMEKK14	4565.A0A3B6RAZ7	4565.A0A3B6KF43	0	0	0.14	0.64	0	0.222	0.186	0.065	0.423
TaMAPK47	TaMAPKK4	4565.A0A3B6RAZ7	4565.A0A3B6KFB5	0	0	0.12	0.606	0	0.705	0.739	0.195	0.938
TaMAPK47	TaMEKK17	4565.A0A3B6RAZ7	4565.A0A3B6KFL8	0	0	0.169	0.646	0	0.27	0.063	0.088	0.412
TaMAPK47	TaMEKK15	4565.A0A3B6RAZ7	4565.A0A3B6KPK7	0	0	0.13	0.636	0	0.222	0.186	0.065	0.416
TaMAPK47	TaMAPKK18	4565.A0A3B6RAZ7	4565.A0A3B6LJ27	0	0	0	0.604	0	0.705	0.739	0.195	0.932
TaMAPK47	TaMEKK24	4565.A0A3B6RAZ7	4565.A0A3B6LLV5	0	0	0.168	0.646	0	0.27	0.063	0.088	0.412
TaMAPK47	TaMEKK16	4565.A0A3B6RAZ7	4565.A0A3B6LW00	0	0	0.129	0.636	0	0.222	0.186	0.065	0.415
TaMAPK47	TaMAPKK2	4565.A0A3B6RAZ7	4565.A0A3B6LYW0	0	0	0	0.622	0	0.705	0.886	0.195	0.97
TaMAPK47	TaMAPKK3	4565.A0A3B6RAZ7	4565.A0A3B6MNP8	0	0	0	0.603	0	0.705	0.739	0.195	0.932

TaMAPK47	TaMEKK29	4565.A0A3B6RAZ7	4565.A0A3B6MSP6	0	0	0.17	0.646	0	0.27	0.063	0.088	0.413
TaMAPK47	TaMEKK11	4565.A0A3B6RAZ7	4565.A0A3B6N0D8	0	0	0.129	0.635	0	0.222	0.186	0.065	0.415
TaMAPK47	TaMAPKK13	4565.A0A3B6RAZ7	4565.A0A3B6N2X8	0	0	0	0.621	0	0.705	0.886	0.195	0.97
TaMAPK47	TaMEKK4	4565.A0A3B6RAZ7	4565.A0A3B6NRN9	0	0	0.153	0.668	0	0.27	0.063	0.088	0.401
TaMAPK47	TaMEKK4-1	4565.A0A3B6RAZ7	4565.A0A3B6PNI6	0	0	0.155	0.668	0	0.27	0.063	0.088	0.402
TaMAPK47	TaMAPKK1	4565.A0A3B6RAZ7	4565.A0A3B6QJ87	0	0	0.161	0.617	0	0.705	0.583	0.146	0.9
TaMAPK47	TaRaf87	4565.A0A3B6RAZ7	4565.A0A3B6QMZ9	0	0	0	0.616	0.16	0.134	0.389	0.088	0.54
TaMAPK49	TaMAPKK9	4565.A9RAB3	4565.A0A077RVQ4	0	0	0.12	0.616	0	0.705	0.583	0.146	0.895
TaMAPK49	TaRaf88	4565.A9RAB3	4565.A0A3B5Z5X1	0	0	0.284	0	0	0.15	0.177	0.05	0.46
TaMAPK49	TaRaf30	4565.A9RAB3	4565.A0A3B6A1Z4	0	0	0.281	0	0	0.15	0.177	0.05	0.458
TaMAPK49	TaMEKK5	4565.A9RAB3	4565.A0A3B6AWC1	0	0	0.165	0.575	0	0.222	0.186	0.065	0.439
TaMAPK49	TaMAPKK15	4565.A9RAB3	4565.A0A3B6HW51	0	0	0.125	0.614	0	0.705	0.583	0.146	0.895
TaMAPK49	TaMAPKK14	4565.A9RAB3	4565.A0A3B6HY95	0	0	0.125	0.606	0	0.705	0.583	0.146	0.895
TaMAPK49	TaMAPKK16	4565.A9RAB3	4565.A0A3B6HZP7	0	0	0.124	0.616	0	0.705	0.583	0.146	0.895
TaMAPK49	TaMAPKK17	4565.A9RAB3	4565.A0A3B6I0M7	0	0	0.122	0.62	0	0.705	0.583	0.146	0.895
TaMAPK49	TaMAPKK8	4565.A9RAB3	4565.A0A3B6IK39	0	0	0.117	0.615	0	0.705	0.583	0.146	0.894
TaMAPK49	TaMAPKK6	4565.A9RAB3	4565.A0A3B6ILF0	0	0	0.123	0.611	0	0.705	0.583	0.146	0.895
TaMAPK49	TaMAPKK5	4565.A9RAB3	4565.A0A3B6IMW7	0	0	0.122	0.605	0	0.705	0.583	0.146	0.895

TaMAPK49	TaMAPKK7	4565.A9RAB3	4565.A0A3B6INV0	0	0	0.124	0.621	0	0.705	0.583	0.146	0.895
TaMAPK49	TaMEKK2	4565.A9RAB3	4565.A0A3B6JCC4	0	0	0.144	0.572	0	0.222	0.186	0.065	0.425
TaMAPK49	TaMAPKK11	4565.A9RAB3	4565.A0A3B6JEH0	0	0	0.121	0.615	0	0.705	0.583	0.146	0.895
TaMAPK49	TaMAPKK12	4565.A9RAB3	4565.A0A3B6JG06	0	0	0.128	0.616	0	0.705	0.583	0.146	0.896
TaMAPK49	TaMEKK14	4565.A9RAB3	4565.A0A3B6KF43	0	0	0.155	0.583	0	0.222	0.186	0.065	0.432
TaMAPK49	TaMAPKK4	4565.A9RAB3	4565.A0A3B6KFB5	0	0	0.105	0.637	0	0.705	0.612	0.146	0.9
TaMAPK49	TaMEKK17	4565.A9RAB3	4565.A0A3B6KFL8	0	0	0.171	0.577	0	0.27	0.063	0.088	0.414
TaMAPK49	TaMEKK15	4565.A9RAB3	4565.A0A3B6KPK7	0	0	0.156	0.576	0	0.222	0.186	0.065	0.433
TaMAPK49	TaMAPKK18	4565.A9RAB3	4565.A0A3B6LJ27	0	0	0	0.584	0	0.705	0.612	0.146	0.893
TaMAPK49	TaMEKK24	4565.A9RAB3	4565.A0A3B6LLV5	0	0	0.17	0.577	0	0.27	0.063	0.088	0.413
TaMAPK49	TaMEKK16	4565.A9RAB3	4565.A0A3B6LW00	0	0	0.155	0.576	0	0.222	0.186	0.065	0.433
TaMAPK49	TaMAPKK2	4565.A9RAB3	4565.A0A3B6LYW0	0	0	0	0.575	0	0.705	0.612	0.205	0.901
TaMAPK49	TaMAPKK3	4565.A9RAB3	4565.A0A3B6MNP8	0	0	0	0.58	0	0.705	0.612	0.146	0.893
TaMAPK49	TaMEKK29	4565.A9RAB3	4565.A0A3B6MSP6	0	0	0.172	0.577	0	0.27	0.063	0.088	0.414
TaMAPK49	TaMEKK11	4565.A9RAB3	4565.A0A3B6N0D8	0	0	0.154	0.573	0	0.222	0.186	0.065	0.432
TaMAPK49	TaMAPKK13	4565.A9RAB3	4565.A0A3B6N2X8	0	0	0	0.576	0	0.705	0.612	0.205	0.901
TaMAPK49	TaMAPKK1	4565.A9RAB3	4565.A0A3B6QJ87	0	0	0.127	0.619	0	0.705	0.583	0.146	0.896
TaMAPK49	TaRaf87	4565.A9RAB3	4565.A0A3B6QMZ9	0	0	0	0.565	0.3	0.134	0.389	0.088	0.617

TaMAPK5	TaMAPKK9	4565.A0A3B6A1I9	4565.A0A077RVQ4	0	0	0.109	0.629	0	0.705	0.583	0.146	0.893
TaMAPK5	TaMEKK24	4565.A0A3B6A1I9	4565.A0A3B6LLV5	0	0	0.168	0.583	0	0.27	0.063	0.088	0.411
TaMAPK5	TaMEKK29	4565.A0A3B6A1I9	4565.A0A3B6MSP6	0	0	0.169	0.583	0	0.27	0.063	0.088	0.412
TaMAPK5	TaMEKK17	4565.A0A3B6A1I9	4565.A0A3B6KFL8	0	0	0.169	0.583	0	0.27	0.063	0.088	0.412
TaMAPK5	TaMEKK2	4565.A0A3B6A1I9	4565.A0A3B6JCC4	0	0	0.139	0.575	0	0.222	0.186	0.065	0.422
TaMAPK5	TaMEKK11	4565.A0A3B6A1I9	4565.A0A3B6N0D8	0	0	0.148	0.576	0	0.222	0.186	0.065	0.428
TaMAPK5	TaMEKK16	4565.A0A3B6A1I9	4565.A0A3B6LW00	0	0	0.149	0.578	0	0.222	0.186	0.065	0.429
TaMAPK5	TaMEKK15	4565.A0A3B6A1I9	4565.A0A3B6KPK7	0	0	0.151	0.578	0	0.222	0.186	0.065	0.43
TaMAPK5	TaMEKK5	4565.A0A3B6A1I9	4565.A0A3B6AWC1	0	0	0.154	0.578	0	0.222	0.186	0.065	0.432
TaMAPK5	TaMEKK14	4565.A0A3B6A1I9	4565.A0A3B6KF43	0	0	0.155	0.591	0	0.222	0.186	0.065	0.433
TaMAPK5	TaRaf87	4565.A0A3B6A1I9	4565.A0A3B6QMZ9	0	0	0	0.565	0.3	0.134	0.389	0.088	0.617
TaMAPK5	TaMAPKK3	4565.A0A3B6A1I9	4565.A0A3B6MNP8	0	0	0	0.574	0	0.705	0.612	0.146	0.893
TaMAPK5	TaMAPKK8	4565.A0A3B6A1I9	4565.A0A3B6IK39	0	0	0.109	0.621	0	0.705	0.583	0.146	0.893
TaMAPK5	TaMAPKK2	4565.A0A3B6A1I9	4565.A0A3B6LYW0	0	0	0	0.577	0	0.705	0.612	0.146	0.893
TaMAPK5	TaMAPKK18	4565.A0A3B6A1I9	4565.A0A3B6LJ27	0	0	0	0.579	0	0.705	0.612	0.146	0.893
TaMAPK5	TaMAPKK13	4565.A0A3B6A1I9	4565.A0A3B6N2X8	0	0	0	0.577	0	0.705	0.612	0.146	0.893
TaMAPK5	TaMAPKK16	4565.A0A3B6A1I9	4565.A0A3B6HZP7	0	0	0.115	0.623	0	0.705	0.583	0.146	0.894
TaMAPK5	TaMAPKK5	4565.A0A3B6A1I9	4565.A0A3B6IMW7	0	0	0.112	0.616	0	0.705	0.583	0.146	0.894

TaMAPK5	TaMAPKK6	4565.A0A3B6A1I9	4565.A0A3B6ILF0	0	0	0.118	0.612	0	0.705	0.583	0.146	0.894
TaMAPK5	TaMAPKK15	4565.A0A3B6A1I9	4565.A0A3B6HW51	0	0	0.115	0.623	0	0.705	0.583	0.146	0.894
TaMAPK5	TaMAPKK11	4565.A0A3B6A1I9	4565.A0A3B6JEH0	0	0	0.111	0.626	0	0.705	0.583	0.146	0.894
TaMAPK5	TaMAPKK14	4565.A0A3B6A1I9	4565.A0A3B6HY95	0	0	0.116	0.615	0	0.705	0.583	0.146	0.894
TaMAPK5	TaMAPKK17	4565.A0A3B6A1I9	4565.A0A3B6I0M7	0	0	0.118	0.614	0	0.705	0.583	0.146	0.895
TaMAPK5	TaMAPKK7	4565.A0A3B6A1I9	4565.A0A3B6INV0	0	0	0.119	0.614	0	0.705	0.583	0.146	0.895
TaMAPK5	TaMAPKK12	4565.A0A3B6A1I9	4565.A0A3B6JG06	0	0	0.124	0.612	0	0.705	0.583	0.146	0.895
TaMAPK5	TaMAPKK1	4565.A0A3B6A1I9	4565.A0A3B6QJ87	0	0	0.125	0.617	0	0.705	0.583	0.146	0.895
TaMAPK5	TaMAPKK4	4565.A0A3B6A1I9	4565.A0A3B6KFB5	0	0	0.104	0.622	0	0.705	0.612	0.146	0.9
TaMAPK52	TaMAPKK9	4565.A0A3B6SH79	4565.A0A077RVQ4	0	0	0.117	0.625	0	0.705	0.583	0.146	0.894
TaMAPK52	TaMEKK5	4565.A0A3B6SH79	4565.A0A3B6AWC1	0	0	0.151	0.595	0	0.222	0.186	0.065	0.43
TaMAPK52	TaMAPKK15	4565.A0A3B6SH79	4565.A0A3B6HW51	0	0	0.122	0.624	0	0.705	0.583	0.146	0.895
TaMAPK52	TaMAPKK14	4565.A0A3B6SH79	4565.A0A3B6HY95	0	0	0.12	0.619	0	0.705	0.583	0.146	0.895
TaMAPK52	TaMAPKK16	4565.A0A3B6SH79	4565.A0A3B6HZP7	0	0	0.12	0.625	0	0.705	0.583	0.146	0.895
TaMAPK52	TaMAPKK17	4565.A0A3B6SH79	4565.A0A3B6I0M7	0	0	0.12	0.626	0	0.705	0.583	0.146	0.895
TaMAPK52	TaMAPKK8	4565.A0A3B6SH79	4565.A0A3B6IK39	0	0	0.113	0.624	0	0.705	0.583	0.146	0.894
TaMAPK52	TaMAPKK6	4565.A0A3B6SH79	4565.A0A3B6ILF0	0	0	0.121	0.619	0	0.705	0.583	0.146	0.895
TaMAPK52	TaMAPKK5	4565.A0A3B6SH79	4565.A0A3B6IMW7	0	0	0.116	0.616	0	0.705	0.583	0.146	0.894

TaMAPK52	TaMAPKK7	4565.A0A3B6SH79	4565.A0A3B6INV0	0	0	0.122	0.624	0	0.705	0.583	0.146	0.895
TaMAPK52	TaMEKK2	4565.A0A3B6SH79	4565.A0A3B6JCC4	0	0	0.132	0.597	0	0.222	0.186	0.065	0.417
TaMAPK52	TaMAPKK11	4565.A0A3B6SH79	4565.A0A3B6JEH0	0	0	0.118	0.624	0	0.705	0.583	0.146	0.895
TaMAPK52	TaMAPKK12	4565.A0A3B6SH79	4565.A0A3B6JG06	0	0	0.124	0.619	0	0.705	0.583	0.146	0.895
TaMAPK52	TaMEKK14	4565.A0A3B6SH79	4565.A0A3B6KF43	0	0	0.145	0.609	0	0.222	0.186	0.065	0.426
TaMAPK52	TaMAPKK4	4565.A0A3B6SH79	4565.A0A3B6KFB5	0	0	0.103	0.626	0	0.705	0.583	0.146	0.893
TaMAPK52	TaMEKK17	4565.A0A3B6SH79	4565.A0A3B6KFL8	0	0	0.161	0.609	0	0.27	0.063	0.088	0.406
TaMAPK52	TaMEKK15	4565.A0A3B6SH79	4565.A0A3B6KPK7	0	0	0.142	0.594	0	0.222	0.186	0.065	0.424
TaMAPK52	TaMAPKK18	4565.A0A3B6SH79	4565.A0A3B6LJ27	0	0	0	0.591	0	0.705	0.583	0.146	0.885
TaMAPK52	TaMEKK24	4565.A0A3B6SH79	4565.A0A3B6LLV5	0	0	0.16	0.609	0	0.27	0.063	0.088	0.406
TaMAPK52	TaMEKK16	4565.A0A3B6SH79	4565.A0A3B6LW00	0	0	0.141	0.594	0	0.222	0.186	0.065	0.423
TaMAPK52	TaMAPKK2	4565.A0A3B6SH79	4565.A0A3B6LYW0	0	0	0	0.595	0	0.705	0.583	0.146	0.885
TaMAPK52	TaMAPKK3	4565.A0A3B6SH79	4565.A0A3B6MNP8	0	0	0	0.593	0	0.705	0.583	0.146	0.885
TaMAPK52	TaMEKK29	4565.A0A3B6SH79	4565.A0A3B6MSP6	0	0	0.161	0.609	0	0.27	0.063	0.088	0.406
TaMAPK52	TaMEKK11	4565.A0A3B6SH79	4565.A0A3B6N0D8	0	0	0.142	0.591	0	0.222	0.186	0.065	0.423
TaMAPK52	TaMAPKK13	4565.A0A3B6SH79	4565.A0A3B6N2X8	0	0	0	0.594	0	0.705	0.583	0.146	0.885
TaMAPK52	TaMAPKK1	4565.A0A3B6SH79	4565.A0A3B6QJ87	0	0	0.132	0.625	0	0.705	0.583	0.146	0.896
TaMAPK52	TaRaf87	4565.A0A3B6SH79	4565.A0A3B6QMZ9	0	0	0	0.585	0.277	0.134	0.389	0.088	0.604

TaMAPK53	TaMAPKK9	4565.A0A3B6SPB2	4565.A0A077RVQ4	0	0	0.12	0.616	0	0.705	0.583	0.146	0.895
TaMAPK53	TaRaf88	4565.A0A3B6SPB2	4565.A0A3B5Z5X1	0	0	0.286	0	0	0.15	0.177	0.05	0.462
TaMAPK53	TaRaf30	4565.A0A3B6SPB2	4565.A0A3B6A1Z4	0	0	0.282	0	0	0.15	0.177	0.05	0.458
TaMAPK53	TaMEKK5	4565.A0A3B6SPB2	4565.A0A3B6AWC1	0	0	0.164	0.575	0	0.222	0.186	0.065	0.439
TaMAPK53	TaMAPKK15	4565.A0A3B6SPB2	4565.A0A3B6HW51	0	0	0.126	0.614	0	0.705	0.583	0.146	0.895
TaMAPK53	TaMAPKK14	4565.A0A3B6SPB2	4565.A0A3B6HY95	0	0	0.125	0.607	0	0.705	0.583	0.146	0.895
TaMAPK53	TaMAPKK16	4565.A0A3B6SPB2	4565.A0A3B6HZP7	0	0	0.124	0.617	0	0.705	0.583	0.146	0.895
TaMAPK53	TaMAPKK17	4565.A0A3B6SPB2	4565.A0A3B6I0M7	0	0	0.122	0.62	0	0.705	0.583	0.146	0.895
TaMAPK53	TaMAPKK8	4565.A0A3B6SPB2	4565.A0A3B6IK39	0	0	0.118	0.615	0	0.705	0.583	0.146	0.894
TaMAPK53	TaMAPKK6	4565.A0A3B6SPB2	4565.A0A3B6ILF0	0	0	0.123	0.61	0	0.705	0.583	0.146	0.895
TaMAPK53	TaMAPKK5	4565.A0A3B6SPB2	4565.A0A3B6IMW7	0	0	0.123	0.605	0	0.705	0.583	0.146	0.895
TaMAPK53	TaMAPKK7	4565.A0A3B6SPB2	4565.A0A3B6INV0	0	0	0.124	0.621	0	0.705	0.583	0.146	0.895
TaMAPK53	TaMEKK2	4565.A0A3B6SPB2	4565.A0A3B6JCC4	0	0	0.144	0.572	0	0.222	0.186	0.065	0.425
TaMAPK53	TaMAPKK11	4565.A0A3B6SPB2	4565.A0A3B6JEH0	0	0	0.122	0.615	0	0.705	0.583	0.146	0.895
TaMAPK53	TaMAPKK12	4565.A0A3B6SPB2	4565.A0A3B6JG06	0	0	0.128	0.616	0	0.705	0.583	0.146	0.896
TaMAPK53	TaMEKK14	4565.A0A3B6SPB2	4565.A0A3B6KF43	0	0	0.155	0.583	0	0.222	0.186	0.065	0.433
TaMAPK53	TaMAPKK4	4565.A0A3B6SPB2	4565.A0A3B6KFB5	0	0	0.106	0.637	0	0.705	0.583	0.146	0.893
TaMAPK53	TaMEKK17	4565.A0A3B6SPB2	4565.A0A3B6KFL8	0	0	0.172	0.577	0	0.27	0.063	0.088	0.414

TaMAPK53	TaMEKK15	4565.A0A3B6SPB2	4565.A0A3B6KPK7	0	0	0.156	0.576	0	0.222	0.186	0.065	0.433
TaMAPK53	TaMAPKK18	4565.A0A3B6SPB2	4565.A0A3B6LJ27	0	0	0	0.584	0	0.705	0.583	0.146	0.885
TaMAPK53	TaMEKK24	4565.A0A3B6SPB2	4565.A0A3B6LLV5	0	0	0.171	0.577	0	0.27	0.063	0.088	0.413
TaMAPK53	TaMEKK16	4565.A0A3B6SPB2	4565.A0A3B6LW00	0	0	0.155	0.575	0	0.222	0.186	0.065	0.433
TaMAPK53	TaMAPKK2	4565.A0A3B6SPB2	4565.A0A3B6LYW0	0	0	0	0.576	0	0.705	0.583	0.146	0.885
TaMAPK53	TaMAPKK3	4565.A0A3B6SPB2	4565.A0A3B6MNP8	0	0	0	0.58	0	0.705	0.583	0.146	0.885
TaMAPK53	TaMEKK29	4565.A0A3B6SPB2	4565.A0A3B6MSP6	0	0	0.172	0.577	0	0.27	0.063	0.088	0.415
TaMAPK53	TaMEKK11	4565.A0A3B6SPB2	4565.A0A3B6N0D8	0	0	0.154	0.573	0	0.222	0.186	0.065	0.432
TaMAPK53	TaMAPKK13	4565.A0A3B6SPB2	4565.A0A3B6N2X8	0	0	0	0.576	0	0.705	0.583	0.146	0.885
TaMAPK53	TaMAPKK1	4565.A0A3B6SPB2	4565.A0A3B6QJ87	0	0	0.127	0.619	0	0.705	0.583	0.146	0.896
TaMAPK53	TaRaf87	4565.A0A3B6SPB2	4565.A0A3B6QMZ9	0	0	0	0.565	0.275	0.134	0.389	0.088	0.603
TaMAPK54	TaMAPKK9	4565.A0A3B6SKC9	4565.A0A077RVQ4	0	0	0.121	0.647	0	0.705	0.583	0.146	0.895
TaMAPK54	TaMEKK5	4565.A0A3B6SKC9	4565.A0A3B6AWC1	0	0	0.128	0.636	0	0.222	0.186	0.065	0.415
TaMAPK54	TaMAPKK15	4565.A0A3B6SKC9	4565.A0A3B6HW51	0	0	0.126	0.639	0	0.705	0.583	0.146	0.895
TaMAPK54	TaMAPKK14	4565.A0A3B6SKC9	4565.A0A3B6HY95	0	0	0.13	0.63	0	0.705	0.583	0.146	0.896
TaMAPK54	TaMAPKK16	4565.A0A3B6SKC9	4565.A0A3B6HZP7	0	0	0.128	0.644	0	0.705	0.583	0.146	0.896
TaMAPK54	TaMAPKK17	4565.A0A3B6SKC9	4565.A0A3B6I0M7	0	0	0.13	0.621	0	0.705	0.583	0.146	0.896
TaMAPK54	TaMAPKK8	4565.A0A3B6SKC9	4565.A0A3B6IK39	0	0	0.123	0.635	0	0.705	0.583	0.146	0.895

TaMAPK54	TaMAPKK6	4565.A0A3B6SKC9	4565.A0A3B6ILF0	0	0	0.127	0.625	0	0.705	0.583	0.146	0.896
TaMAPK54	TaMAPKK5	4565.A0A3B6SKC9	4565.A0A3B6IMW7	0	0	0.128	0.625	0	0.705	0.583	0.146	0.896
TaMAPK54	TaMAPKK7	4565.A0A3B6SKC9	4565.A0A3B6INV0	0	0	0.129	0.62	0	0.705	0.583	0.146	0.896
TaMAPK54	TaMEKK2	4565.A0A3B6SKC9	4565.A0A3B6JCC4	0	0	0.114	0.607	0	0.222	0.186	0.065	0.405
TaMAPK54	TaMAPKK11	4565.A0A3B6SKC9	4565.A0A3B6JEH0	0	0	0.133	0.634	0	0.705	0.583	0.146	0.896
TaMAPK54	TaMAPKK12	4565.A0A3B6SKC9	4565.A0A3B6JG06	0	0	0.131	0.615	0	0.705	0.583	0.146	0.896
TaMAPK54	TaMEKK14	4565.A0A3B6SKC9	4565.A0A3B6KF43	0	0	0.128	0.624	0	0.222	0.186	0.065	0.414
TaMAPK54	TaMAPKK4	4565.A0A3B6SKC9	4565.A0A3B6KFB5	0	0	0.105	0.61	0	0.705	0.739	0.195	0.937
TaMAPK54	TaMEKK15	4565.A0A3B6SKC9	4565.A0A3B6KPK7	0	0	0.119	0.632	0	0.222	0.186	0.065	0.408
TaMAPK54	TaMAPKK18	4565.A0A3B6SKC9	4565.A0A3B6LJ27	0	0	0	0.59	0	0.705	0.739	0.195	0.932
TaMAPK54	TaMEKK16	4565.A0A3B6SKC9	4565.A0A3B6LW00	0	0	0.118	0.631	0	0.222	0.186	0.065	0.407
TaMAPK54	TaMAPKK2	4565.A0A3B6SKC9	4565.A0A3B6LYW0	0	0	0	0.601	0	0.705	0.886	0.195	0.97
TaMAPK54	TaMAPKK3	4565.A0A3B6SKC9	4565.A0A3B6MNP8	0	0	0	0.589	0	0.705	0.739	0.195	0.932
TaMAPK54	TaMEKK11	4565.A0A3B6SKC9	4565.A0A3B6N0D8	0	0	0.117	0.63	0	0.222	0.186	0.065	0.407
TaMAPK54	TaMAPKK13	4565.A0A3B6SKC9	4565.A0A3B6N2X8	0	0	0	0.597	0	0.705	0.886	0.195	0.97
TaMAPK54	TaMAPKK1	4565.A0A3B6SKC9	4565.A0A3B6QJ87	0	0	0.141	0.606	0	0.705	0.583	0.146	0.897
TaMAPK54	TaRaf87	4565.A0A3B6SKC9	4565.A0A3B6QMZ9	0	0	0	0.579	0.16	0.134	0.389	0.088	0.54
TaMAPK6	TaMAPKK9	4565.A0A3B6SCW0	4565.A0A077RVQ4	0	0	0.13	0.633	0	0.705	0.763	0.146	0.941

TaMAPK6	TaMEKK5	4565.A0A3 B6SCW0	4565.A0A3 B6AWC1	0	0	0.126	0.635	0	0.222	0.186	0.065	0.413
TaMAPK6	TaRaf111	4565.A0A3 B6SCW0	4565.A0A3 B6FHS8	0	0	0	0	0.07	0.141	0.15	0.297	0.458
TaMAPK6	TaMAPKK 15	4565.A0A3 B6SCW0	4565.A0A3 B6HW51	0	0	0.13	0.645	0	0.705	0.763	0.146	0.941
TaMAPK6	TaMAPKK 14	4565.A0A3 B6SCW0	4565.A0A3 B6HY95	0	0	0.132	0.649	0	0.705	0.763	0.146	0.941
TaMAPK6	TaMAPKK 16	4565.A0A3 B6SCW0	4565.A0A3 B6HZP7	0	0	0.136	0.652	0	0.705	0.763	0.146	0.941
TaMAPK6	TaMAPKK 17	4565.A0A3 B6SCW0	4565.A0A3 B6I0M7	0	0	0.129	0.639	0	0.705	0.763	0.146	0.941
TaMAPK6	TaMAPKK 8	4565.A0A3 B6SCW0	4565.A0A3 B6IK39	0	0	0.128	0.643	0	0.705	0.763	0.146	0.94
TaMAPK6	TaMAPKK 6	4565.A0A3 B6SCW0	4565.A0A3 B6ILF0	0	0	0.129	0.633	0	0.705	0.763	0.146	0.941
TaMAPK6	TaMAPKK 5	4565.A0A3 B6SCW0	4565.A0A3 B6IMW7	0	0	0.128	0.65	0	0.705	0.763	0.146	0.941
TaMAPK6	TaMAPKK 7	4565.A0A3 B6SCW0	4565.A0A3 B6INV0	0	0	0.131	0.637	0	0.705	0.763	0.146	0.941
TaMAPK6	TaMEKK2	4565.A0A3 B6SCW0	4565.A0A3 B6JCC4	0	0	0.107	0.625	0	0.222	0.186	0.065	0.4
TaMAPK6	TaMAPKK 11	4565.A0A3 B6SCW0	4565.A0A3 B6JEH0	0	0	0.134	0.644	0	0.705	0.763	0.146	0.941
TaMAPK6	TaMAPKK 12	4565.A0A3 B6SCW0	4565.A0A3 B6JG06	0	0	0.133	0.636	0	0.705	0.946	0.146	0.986
TaMAPK6	TaMEKK1 4	4565.A0A3 B6SCW0	4565.A0A3 B6KF43	0	0	0.124	0.622	0	0.222	0.186	0.065	0.411
TaMAPK6	TaMAPKK 4	4565.A0A3 B6SCW0	4565.A0A3 B6KFB5	0	0	0.101	0.636	0	0.705	0.825	0.209	0.958
TaMAPK6	TaMEKK1 5	4565.A0A3 B6SCW0	4565.A0A3 B6KPK7	0	0	0.116	0.622	0	0.222	0.186	0.065	0.406
TaMAPK6	TaMAPKK 18	4565.A0A3 B6SCW0	4565.A0A3 B6LJ27	0	0	0	0.602	0	0.705	0.825	0.209	0.955
TaMAPK6	TaMEKK1 6	4565.A0A3 B6SCW0	4565.A0A3 B6LW00	0	0	0.115	0.622	0	0.222	0.186	0.065	0.406

TaMAPK6	TaMAPKK2	4565.A0A3B6SCW0	4565.A0A3B6LYW0	0	0	0	0.612	0	0.705	0.927	0.209	0.981
TaMAPK6	TaMAPKK3	4565.A0A3B6SCW0	4565.A0A3B6MNP8	0	0	0	0.605	0	0.705	0.825	0.209	0.955
TaMAPK6	TaMEKK11	4565.A0A3B6SCW0	4565.A0A3B6N0D8	0	0	0.115	0.62	0	0.222	0.186	0.065	0.405
TaMAPK6	TaMAPKK13	4565.A0A3B6SCW0	4565.A0A3B6N2X8	0	0	0	0.617	0	0.705	0.927	0.209	0.981
TaMAPK6	TaMAPKK1	4565.A0A3B6SCW0	4565.A0A3B6QJ87	0	0	0.148	0.625	0	0.705	0.883	0.146	0.971
TaMAPK6	TaRaf87	4565.A0A3B6SCW0	4565.A0A3B6QMZ9	0	0	0	0.599	0.16	0.134	0.389	0.088	0.54
TaMAPK7	TaMAPKK9	4565.A0A3B6TM72	4565.A0A077RVQ4	0	0	0.117	0.625	0	0.705	0.583	0.146	0.894
TaMAPK7	TaMEKK5	4565.A0A3B6TM72	4565.A0A3B6AWC1	0	0	0.151	0.595	0	0.222	0.186	0.065	0.43
TaMAPK7	TaMAPKK15	4565.A0A3B6TM72	4565.A0A3B6HW51	0	0	0.122	0.623	0	0.705	0.583	0.146	0.895
TaMAPK7	TaMAPKK14	4565.A0A3B6TM72	4565.A0A3B6HY95	0	0	0.12	0.619	0	0.705	0.583	0.146	0.895
TaMAPK7	TaMAPKK16	4565.A0A3B6TM72	4565.A0A3B6HZP7	0	0	0.121	0.625	0	0.705	0.583	0.146	0.895
TaMAPK7	TaMAPKK17	4565.A0A3B6TM72	4565.A0A3B6I0M7	0	0	0.12	0.626	0	0.705	0.583	0.146	0.895
TaMAPK7	TaMAPKK8	4565.A0A3B6TM72	4565.A0A3B6IK39	0	0	0.113	0.624	0	0.705	0.583	0.146	0.894
TaMAPK7	TaMAPKK6	4565.A0A3B6TM72	4565.A0A3B6ILF0	0	0	0.121	0.619	0	0.705	0.583	0.146	0.895
TaMAPK7	TaMAPKK5	4565.A0A3B6TM72	4565.A0A3B6IMW7	0	0	0.117	0.616	0	0.705	0.583	0.146	0.894
TaMAPK7	TaMAPKK7	4565.A0A3B6TM72	4565.A0A3B6INV0	0	0	0.122	0.624	0	0.705	0.583	0.146	0.895
TaMAPK7	TaMEKK2	4565.A0A3B6TM72	4565.A0A3B6JCC4	0	0	0.131	0.597	0	0.222	0.186	0.065	0.417
TaMAPK7	TaMAPKK11	4565.A0A3B6TM72	4565.A0A3B6JEH0	0	0	0.118	0.624	0	0.705	0.583	0.146	0.895

TaMAPK7	TaMAPKK 12	4565.A0A3 B6TM72	4565.A0A3 B6JG06	0	0	0.125	0.618	0	0.705	0.583	0.146	0.895
TaMAPK7	TaMEKK1 4	4565.A0A3 B6TM72	4565.A0A3 B6KF43	0	0	0.145	0.609	0	0.222	0.186	0.065	0.426
TaMAPK7	TaMAPKK 4	4565.A0A3 B6TM72	4565.A0A3 B6KFB5	0	0	0.103	0.626	0	0.705	0.583	0.146	0.893
TaMAPK7	TaMEKK1 7	4565.A0A3 B6TM72	4565.A0A3 B6KFL8	0	0	0.16	0.609	0	0.27	0.063	0.088	0.406
TaMAPK7	TaMEKK1 5	4565.A0A3 B6TM72	4565.A0A3 B6KPK7	0	0	0.142	0.594	0	0.222	0.186	0.065	0.424
TaMAPK7	TaMAPKK 18	4565.A0A3 B6TM72	4565.A0A3 B6LJ27	0	0	0	0.591	0	0.705	0.583	0.146	0.885
TaMAPK7	TaMEKK2 4	4565.A0A3 B6TM72	4565.A0A3 B6LLV5	0	0	0.159	0.609	0	0.27	0.063	0.088	0.405
TaMAPK7	TaMEKK1 6	4565.A0A3 B6TM72	4565.A0A3 B6LW00	0	0	0.141	0.594	0	0.222	0.186	0.065	0.423
TaMAPK7	TaMAPKK 2	4565.A0A3 B6TM72	4565.A0A3 B6LYW0	0	0	0	0.594	0	0.705	0.583	0.146	0.885
TaMAPK7	TaMAPKK 3	4565.A0A3 B6TM72	4565.A0A3 B6MNP8	0	0	0	0.593	0	0.705	0.583	0.146	0.885
TaMAPK7	TaMEKK2 9	4565.A0A3 B6TM72	4565.A0A3 B6MSP6	0	0	0.161	0.609	0	0.27	0.063	0.088	0.406
TaMAPK7	TaMEKK1 1	4565.A0A3 B6TM72	4565.A0A3 B6N0D8	0	0	0.141	0.591	0	0.222	0.186	0.065	0.423
TaMAPK7	TaMAPKK 13	4565.A0A3 B6TM72	4565.A0A3 B6N2X8	0	0	0	0.593	0	0.705	0.583	0.146	0.885
TaMAPK7	TaMAPKK 1	4565.A0A3 B6TM72	4565.A0A3 B6QJ87	0	0	0.132	0.625	0	0.705	0.583	0.146	0.896
TaMAPK7	TaRaf87	4565.A0A3 B6TM72	4565.A0A3 B6QMZ9	0	0	0	0.585	0.277	0.134	0.389	0.088	0.604
TaMAPK8	TaMAPKK 9	4565.A0A3 B6GTS3	4565.A0A0 77RVQ4	0	0	0.112	0.617	0	0.705	0.583	0.146	0.894
TaMAPK8	TaMEKK5	4565.A0A3 B6GTS3	4565.A0A3 B6AWC1	0	0	0.139	0.584	0	0.222	0.186	0.065	0.422
TaMAPK8	TaMEKK2	4565.A0A3 B6GTS3	4565.A0A3 B6JCC4	0	0	0.126	0.574	0	0.222	0.186	0.065	0.413

TaMAPK8	TaMEKK16	4565.A0A3B6GTS3	4565.A0A3B6LW00	0	0	0.128	0.578	0	0.222	0.186	0.065	0.414
TaMAPK8	TaMEKK11	4565.A0A3B6GTS3	4565.A0A3B6N0D8	0	0	0.127	0.576	0	0.222	0.186	0.065	0.414
TaMAPK8	TaMEKK15	4565.A0A3B6GTS3	4565.A0A3B6KPK7	0	0	0.129	0.578	0	0.222	0.186	0.065	0.415
TaMAPK8	TaMEKK17	4565.A0A3B6GTS3	4565.A0A3B6KFL8	0	0	0.179	0.577	0	0.27	0.063	0.088	0.419
TaMAPK8	TaMEKK24	4565.A0A3B6GTS3	4565.A0A3B6LLV5	0	0	0.178	0.577	0	0.27	0.063	0.088	0.419
TaMAPK8	TaMEKK29	4565.A0A3B6GTS3	4565.A0A3B6MSP6	0	0	0.179	0.577	0	0.27	0.063	0.088	0.419
TaMAPK8	TaMEKK14	4565.A0A3B6GTS3	4565.A0A3B6KF43	0	0	0.138	0.586	0	0.222	0.186	0.065	0.421
TaMAPK8	TaRaf87	4565.A0A3B6GTS3	4565.A0A3B6QMZ9	0	0	0	0.561	0.277	0.134	0.389	0.088	0.604
TaMAPK8	TaMAPKK18	4565.A0A3B6GTS3	4565.A0A3B6LJ27	0	0	0	0.59	0	0.705	0.583	0.146	0.885
TaMAPK8	TaMAPKK4	4565.A0A3B6GTS3	4565.A0A3B6KFB5	0	0	0	0.647	0	0.705	0.583	0.146	0.885
TaMAPK8	TaMAPKK13	4565.A0A3B6GTS3	4565.A0A3B6N2X8	0	0	0	0.581	0	0.705	0.583	0.146	0.885
TaMAPK8	TaMAPKK2	4565.A0A3B6GTS3	4565.A0A3B6LYW0	0	0	0	0.585	0	0.705	0.583	0.146	0.885
TaMAPK8	TaMAPKK3	4565.A0A3B6GTS3	4565.A0A3B6MNP8	0	0	0	0.582	0	0.705	0.583	0.146	0.885
TaMAPK8	TaMAPKK14	4565.A0A3B6GTS3	4565.A0A3B6HY95	0	0	0.114	0.607	0	0.705	0.583	0.146	0.894
TaMAPK8	TaMAPKK11	4565.A0A3B6GTS3	4565.A0A3B6JEH0	0	0	0.115	0.614	0	0.705	0.583	0.146	0.894
TaMAPK8	TaMAPKK15	4565.A0A3B6GTS3	4565.A0A3B6HW51	0	0	0.117	0.614	0	0.705	0.583	0.146	0.894
TaMAPK8	TaMAPKK8	4565.A0A3B6GTS3	4565.A0A3B6IK39	0	0	0.111	0.616	0	0.705	0.583	0.146	0.894
TaMAPK8	TaMAPKK6	4565.A0A3B6GTS3	4565.A0A3B6ILF0	0	0	0.118	0.607	0	0.705	0.583	0.146	0.894

TaMAPK8	TaMAPKK5	4565.A0A3B6GTS3	4565.A0A3B6IMW7	0	0	0.115	0.606	0	0.705	0.583	0.146	0.894
TaMAPK8	TaMAPKK16	4565.A0A3B6GTS3	4565.A0A3B6HZP7	0	0	0.115	0.616	0	0.705	0.583	0.146	0.894
TaMAPK8	TaMAPKK17	4565.A0A3B6GTS3	4565.A0A3B6I0M7	0	0	0.117	0.611	0	0.705	0.583	0.146	0.894
TaMAPK8	TaMAPKK12	4565.A0A3B6GTS3	4565.A0A3B6JG06	0	0	0.121	0.605	0	0.705	0.583	0.146	0.895
TaMAPK8	TaMAPKK7	4565.A0A3B6GTS3	4565.A0A3B6INV0	0	0	0.12	0.608	0	0.705	0.583	0.146	0.895
TaMAPK8	TaMAPKK1	4565.A0A3B6GTS3	4565.A0A3B6QJ87	0	0	0.13	0.611	0	0.705	0.583	0.146	0.896
TaMAPKK1	TaMEKK7	4565.A0A3B6QJ87	4565.A0A077RUI2	0	0	0.179	0.701	0.05	0.652	0.431	0.136	0.842
TaMAPKK1	TaRaf18	4565.A0A3B6QJ87	4565.A0A077RY41	0	0	0.191	0.657	0	0.172	0.176	0	0.4
TaMAPKK1	TaMEKK9	4565.A0A3B6QJ87	4565.A0A077S2G5	0	0	0.202	0.638	0.05	0.652	0.431	0.136	0.846
TaMAPKK1	TaMAPKKK4	4565.A0A3B6QJ87	4565.A0A3B6AY64	0	0	0.102	0.726	0	0.176	0.579	0.195	0.715
TaMAPKK1	TaMEKK1	4565.A0A3B6QJ87	4565.A0A3B6B3I4	0	0	0.154	0.745	0.05	0.652	0.883	0.136	0.966
TaMAPKK1	TaMEKK20	4565.A0A3B6QJ87	4565.A0A3B6B6T5	0	0	0	0.671	0.05	0.652	0.431	0.136	0.815
TaMAPKK1	TaMAPKKK5	4565.A0A3B6QJ87	4565.A0A3B6C4T6	0	0	0.101	0.726	0	0.176	0.579	0.195	0.715
TaMAPKK1	TaMEKK18	4565.A0A3B6QJ87	4565.A0A3B6CEA6	0	0	0	0.671	0.05	0.652	0.431	0.136	0.815
TaMAPKK1	TaMAPKKK6	4565.A0A3B6QJ87	4565.A0A3B6DEP0	0	0	0.102	0.726	0	0.176	0.579	0.195	0.715
TaMAPKK1	TaRaf60	4565.A0A3B6QJ87	4565.A0A3B6EHW0	0	0	0.207	0.623	0	0.172	0.176	0	0.412
TaMAPKK1	TaMEKK8	4565.A0A3B6QJ87	4565.A0A3B6FNU8	0	0	0.18	0.691	0.05	0.652	0.431	0.136	0.842
TaMAPKK1	TaRaf5	4565.A0A3B6QJ87	4565.A0A3B6GYQ1	0	0	0.206	0.625	0	0.172	0.176	0	0.411

TaMAPKK1	TaMEKK17	4565.A0A3B6QJ87	4565.A0A3B6KFL8	0	0	0.153	0.744	0.05	0.652	0.883	0.136	0.966
TaMAPKK1	TaRaf41	4565.A0A3B6QJ87	4565.A0A3B6KLD7	0	0	0.205	0.62	0	0.172	0.176	0	0.41
TaMAPKK1	TaMEKK24	4565.A0A3B6QJ87	4565.A0A3B6LLV5	0	0	0.153	0.744	0.05	0.652	0.883	0.136	0.966
TaMAPKK1	TaRaf56	4565.A0A3B6QJ87	4565.A0A3B6LPY3	0	0	0.217	0.593	0	0.172	0.176	0	0.419
TaMAPKK1	TaMEKK29	4565.A0A3B6QJ87	4565.A0A3B6MSP6	0	0	0.153	0.744	0.05	0.652	0.883	0.136	0.966
TaMAPKK1	TaMEKK4	4565.A0A3B6QJ87	4565.A0A3B6NRN9	0	0	0.157	0.73	0.05	0.652	0.883	0.136	0.966
TaMAPKK1	TaRaf91	4565.A0A3B6QJ87	4565.A0A3B6PMI5	0	0	0.125	0.644	0	0.172	0.489	0	0.597
TaMAPKK1	TaMEKK4-1	4565.A0A3B6QJ87	4565.A0A3B6PNI6	0	0	0.158	0.73	0.05	0.652	0.883	0.136	0.966
TaMAPKK1	TaRaf62	4565.A0A3B6QJ87	4565.A9RAA9	0	0	0.193	0.662	0	0.172	0.176	0	0.401
TaMAPKK1	TaRaf7	4565.A0A3B6QJ87	4565.A0A3B6TBH5	0	0	0.215	0.595	0	0.172	0.176	0	0.418
TaMAPKK11	TaMEKK7	4565.A0A3B6JEH0	4565.A0A077RUI2	0	0	0.176	0.695	0.05	0.652	0.431	0.136	0.841
TaMAPKK11	TaRaf18	4565.A0A3B6JEH0	4565.A0A077RY41	0	0	0.192	0.637	0	0.172	0.176	0	0.401
TaMAPKK11	TaMEKK9	4565.A0A3B6JEH0	4565.A0A077S2G5	0	0	0.19	0.652	0.05	0.652	0.431	0.136	0.844
TaMAPKK11	TaRaf95	4565.A0A3B6JEH0	4565.A0A1D5UN01	0	0	0.193	0.601	0	0.172	0.176	0	0.401
TaMAPKK11	TaRaf88	4565.A0A3B6JEH0	4565.A0A3B5Z5X1	0	0	0.197	0.618	0	0.172	0.176	0	0.404
TaMAPKK11	TaRaf30	4565.A0A3B6JEH0	4565.A0A3B6A1Z4	0	0	0.196	0.62	0	0.172	0.176	0	0.404
TaMAPKK11	TaRaf102	4565.A0A3B6JEH0	4565.A0A3B6AQT7	0	0	0.232	0	0	0.172	0.176	0	0.43
TaMAPKK11	TaMAPKKKK4	4565.A0A3B6JEH0	4565.A0A3B6AY64	0	0	0.109	0.682	0	0.176	0.579	0.195	0.718

TaMAPKK11	TaMEKK1	4565.A0A3B6JEH0	4565.A0A3B6B3I4	0	0	0.152	0.722	0.05	0.652	0.573	0.136	0.877
TaMAPKK11	TaMEKK20	4565.A0A3B6JEH0	4565.A0A3B6B6T5	0	0	0	0.702	0.05	0.652	0.431	0.136	0.815
TaMAPKK11	TaRaf105	4565.A0A3B6JEH0	4565.A0A3B6C472	0	0	0.254	0	0	0.172	0.176	0	0.446
TaMAPKK11	TaMAPKKK5	4565.A0A3B6JEH0	4565.A0A3B6C4T6	0	0	0.109	0.682	0	0.176	0.579	0.195	0.717
TaMAPKK11	TaMEKK18	4565.A0A3B6JEH0	4565.A0A3B6CEA6	0	0	0	0.703	0.05	0.652	0.431	0.136	0.815
TaMAPKK11	TaMAPKKK6	4565.A0A3B6JEH0	4565.A0A3B6DEP0	0	0	0.109	0.684	0	0.176	0.579	0.195	0.718
TaMAPKK11	TaRaf60	4565.A0A3B6JEH0	4565.A0A3B6EHW0	0	0	0.201	0.587	0	0.172	0.176	0	0.407
TaMAPKK11	TaMEKK8	4565.A0A3B6JEH0	4565.A0A3B6FNU8	0	0	0.168	0.71	0.05	0.652	0.431	0.136	0.84
TaMAPKK11	TaRaf73	4565.A0A3B6JEH0	4565.A0A3B6GRN8	0	0	0.244	0	0	0.172	0.176	0	0.439
TaMAPKK11	TaRaf5	4565.A0A3B6JEH0	4565.A0A3B6GYQ1	0	0	0.198	0.594	0	0.172	0.176	0	0.405
TaMAPKK11	TaRaf46	4565.A0A3B6JEH0	4565.A0A3B6MW69	0	0	0.194	0.628	0	0.172	0.176	0	0.402
TaMAPKK11	TaRaf83	4565.A0A3B6JEH0	4565.A0A3B6MWW7	0	0	0.204	0	0	0.172	0.176	0	0.409
TaMAPKK11	TaRaf44	4565.A0A3B6JEH0	4565.A0A3B6KNC2	0	0	0.205	0	0	0.172	0.176	0	0.41
TaMAPKK11	TaRaf62	4565.A0A3B6JEH0	4565.A9RAA9	0	0	0.206	0.598	0	0.172	0.176	0	0.411
TaMAPKK11	TaRaf41	4565.A0A3B6JEH0	4565.A0A3B6KLD7	0	0	0.216	0.586	0	0.172	0.176	0	0.418
TaMAPKK11	TaRaf56	4565.A0A3B6JEH0	4565.A0A3B6LPY3	0	0	0.216	0.584	0	0.172	0.176	0	0.418
TaMAPKK11	TaRaf7	4565.A0A3B6JEH0	4565.A0A3B6TBH5	0	0	0.216	0.593	0	0.172	0.176	0	0.418
TaMAPKK11	TaRaf42	4565.A0A3B6JEH0	4565.A0A3B6U554	0	0	0.223	0	0	0.172	0.176	0	0.423

TaMAPKK 11	TaRaf45	4565.A0A3 B6JEH0	4565.A0A3 B6KN82	0	0	0.235	0	0	0.172	0.176	0	0.432
TaMAPKK 11	TaRaf72	4565.A0A3 B6JEH0	4565.A0A3 B6MXW5	0	0	0.247	0	0	0.172	0.176	0	0.441
TaMAPKK 11	TaRaf50	4565.A0A3 B6JEH0	4565.A0A3 B6RET0	0	0	0.28	0	0	0.172	0.176	0	0.465
TaMAPKK 11	TaRaf43	4565.A0A3 B6JEH0	4565.A0A3 B6KNC6	0	0	0.292	0	0	0.172	0.176	0	0.474
TaMAPKK 11	TaRaf91	4565.A0A3 B6JEH0	4565.A0A3 B6PMI5	0	0	0.136	0.604	0	0.172	0.489	0	0.602
TaMAPKK 11	TaMEKK4-1	4565.A0A3 B6JEH0	4565.A0A3 B6PNI6	0	0	0.163	0.701	0.05	0.652	0.573	0.136	0.879
TaMAPKK 11	TaMEKK4	4565.A0A3 B6JEH0	4565.A0A3 B6NRN9	0	0	0.163	0.699	0.05	0.652	0.573	0.136	0.879
TaMAPKK 11	TaMEKK24	4565.A0A3 B6JEH0	4565.A0A3 B6LLV5	0	0	0.156	0.74	0.05	0.652	0.614	0.136	0.89
TaMAPKK 11	TaMEKK29	4565.A0A3 B6JEH0	4565.A0A3 B6MSP6	0	0	0.156	0.74	0.05	0.652	0.614	0.136	0.89
TaMAPKK 11	TaMEKK17	4565.A0A3 B6JEH0	4565.A0A3 B6KFL8	0	0	0.156	0.74	0.05	0.652	0.614	0.136	0.89
TaMAPKK 12	TaMEKK7	4565.A0A3 B6JG06	4565.A0A0 77RUI2	0	0	0.182	0.702	0.05	0.652	0.431	0.136	0.842
TaMAPKK 12	TaRaf18	4565.A0A3 B6JG06	4565.A0A0 77RY41	0	0	0.21	0.621	0	0.172	0.176	0	0.414
TaMAPKK 12	TaMEKK9	4565.A0A3 B6JG06	4565.A0A0 77S2G5	0	0	0.199	0.647	0.05	0.652	0.431	0.136	0.846
TaMAPKK 12	TaRaf29	4565.A0A3 B6JG06	4565.A0A1 D5UHD7	0	0	0.194	0.634	0	0.172	0.176	0	0.402
TaMAPKK 12	TaRaf95	4565.A0A3 B6JG06	4565.A0A1 D5UN01	0	0	0.192	0.603	0	0.172	0.176	0	0.401
TaMAPKK 12	TaRaf88	4565.A0A3 B6JG06	4565.A0A3 B5Z5X1	0	0	0.213	0.622	0	0.172	0.176	0	0.416
TaMAPKK 12	TaRaf30	4565.A0A3 B6JG06	4565.A0A3 B6A1Z4	0	0	0.211	0.621	0	0.172	0.176	0	0.415
TaMAPKK 12	TaMAPKKKK4	4565.A0A3 B6JG06	4565.A0A3 B6AY64	0	0	0.114	0.685	0	0.176	0.579	0.195	0.719

TaMAPKK12	TaMEKK1	4565.A0A3B6JG06	4565.A0A3B6B3I4	0	0	0.153	0.735	0.05	0.652	0.573	0.136	0.877
TaMAPKK12	TaMEKK20	4565.A0A3B6JG06	4565.A0A3B6B6T5	0	0	0	0.686	0.05	0.652	0.431	0.136	0.815
TaMAPKK12	TaRaf79	4565.A0A3B6JG06	4565.A0A3B6B9C7	0	0	0.194	0.63	0	0.172	0.176	0	0.402
TaMAPKK12	TaMAPKKK5	4565.A0A3B6JG06	4565.A0A3B6C4T6	0	0	0.113	0.684	0	0.176	0.579	0.195	0.719
TaMAPKK12	TaMEKK18	4565.A0A3B6JG06	4565.A0A3B6CEA6	0	0	0	0.686	0.05	0.652	0.431	0.136	0.815
TaMAPKK12	TaMAPKKK6	4565.A0A3B6JG06	4565.A0A3B6DEP0	0	0	0.114	0.684	0	0.176	0.579	0.195	0.719
TaMAPKK12	TaRaf59	4565.A0A3B6JG06	4565.A0A3B6E9E2	0	0	0.196	0.609	0	0.172	0.176	0	0.404
TaMAPKK12	TaRaf60	4565.A0A3B6JG06	4565.A0A3B6EHW0	0	0	0.218	0.591	0	0.172	0.176	0	0.419
TaMAPKK12	TaMEKK8	4565.A0A3B6JG06	4565.A0A3B6FNU8	0	0	0.181	0.695	0.05	0.652	0.431	0.136	0.842
TaMAPKK12	TaRaf5	4565.A0A3B6JG06	4565.A0A3B6GYQ1	0	0	0.218	0.595	0	0.172	0.176	0	0.42
TaMAPKK12	TaRaf58	4565.A0A3B6JG06	4565.A0A3B6N1Y2	0	0	0.195	0.589	0	0.172	0.176	0	0.403
TaMAPKK12	TaRaf46	4565.A0A3B6JG06	4565.A0A3B6MW69	0	0	0.198	0.647	0	0.172	0.176	0	0.405
TaMAPKK12	TaRaf21	4565.A0A3B6JG06	4565.A0A3B6TVC5	0	0	0.198	0.594	0	0.172	0.176	0	0.405
TaMAPKK12	TaRaf1	4565.A0A3B6JG06	4565.A0A3B6NP57	0	0	0.202	0.586	0	0.172	0.176	0	0.408
TaMAPKK12	TaRaf7	4565.A0A3B6JG06	4565.A0A3B6TBH5	0	0	0.211	0.601	0	0.172	0.176	0	0.414
TaMAPKK12	TaRaf41	4565.A0A3B6JG06	4565.A0A3B6KLD7	0	0	0.212	0.608	0	0.172	0.176	0	0.415
TaMAPKK12	TaRaf62	4565.A0A3B6JG06	4565.A9RAA9	0	0	0.213	0.615	0	0.172	0.176	0	0.416
TaMAPKK12	TaRaf56	4565.A0A3B6JG06	4565.A0A3B6LPY3	0	0	0.217	0.595	0	0.172	0.176	0	0.419

TaMAPKK12	TaRaf91	4565.A0A3B6JG06	4565.A0A3B6PMI5	0	0	0.151	0.597	0	0.172	0.489	0	0.609
TaMAPKK12	TaMEKK4	4565.A0A3B6JG06	4565.A0A3B6NRN9	0	0	0.165	0.708	0.05	0.652	0.573	0.136	0.879
TaMAPKK12	TaMEKK4-1	4565.A0A3B6JG06	4565.A0A3B6PNI6	0	0	0.165	0.71	0.05	0.652	0.573	0.136	0.879
TaMAPKK12	TaMEKK17	4565.A0A3B6JG06	4565.A0A3B6KFL8	0	0	0.166	0.729	0.05	0.652	0.614	0.136	0.891
TaMAPKK12	TaMEKK29	4565.A0A3B6JG06	4565.A0A3B6MSP6	0	0	0.166	0.729	0.05	0.652	0.614	0.136	0.891
TaMAPKK12	TaMEKK24	4565.A0A3B6JG06	4565.A0A3B6LLV5	0	0	0.166	0.729	0.05	0.652	0.614	0.136	0.891
TaMAPKK13	TaMEKK7	4565.A0A3B6N2X8	4565.A0A077RUI2	0	0	0.111	0.617	0.05	0.652	0.927	0.136	0.978
TaMAPKK13	TaMEKK9	4565.A0A3B6N2X8	4565.A0A077S2G5	0	0	0.109	0.654	0.05	0.652	0.791	0.136	0.937
TaMAPKK13	TaMAPKKK4	4565.A0A3B6N2X8	4565.A0A3B6AY64	0	0	0	0.654	0	0.176	0.579	0.195	0.696
TaMAPKK13	TaMEKK1	4565.A0A3B6N2X8	4565.A0A3B6B3I4	0	0	0	0.631	0.05	0.652	0.431	0.136	0.815
TaMAPKK13	TaMEKK20	4565.A0A3B6N2X8	4565.A0A3B6B6T5	0	0	0	0.631	0.05	0.652	0.431	0.136	0.815
TaMAPKK13	TaMAPKKK5	4565.A0A3B6N2X8	4565.A0A3B6C4T6	0	0	0	0.655	0	0.176	0.579	0.195	0.696
TaMAPKK13	TaMEKK18	4565.A0A3B6N2X8	4565.A0A3B6CEA6	0	0	0	0.631	0.05	0.652	0.431	0.136	0.815
TaMAPKK13	TaMAPKKK6	4565.A0A3B6N2X8	4565.A0A3B6DEP0	0	0	0	0.655	0	0.176	0.579	0.195	0.696
TaMAPKK13	TaMEKK8	4565.A0A3B6N2X8	4565.A0A3B6FNU8	0	0	0.107	0.628	0.05	0.652	0.946	0.136	0.983
TaMAPKK13	TaMEKK17	4565.A0A3B6N2X8	4565.A0A3B6KFL8	0	0	0.138	0.644	0.05	0.652	0.431	0.136	0.834
TaMAPKK13	TaMEKK24	4565.A0A3B6N2X8	4565.A0A3B6LLV5	0	0	0.14	0.643	0.05	0.652	0.431	0.136	0.834
TaMAPKK13	TaMEKK29	4565.A0A3B6N2X8	4565.A0A3B6MSP6	0	0	0.138	0.644	0.05	0.652	0.431	0.136	0.834

TaMAPKK13	TaMEKK4-1	4565.A0A3B6N2X8	4565.A0A3B6PNI6	0	0	0	0.625	0.05	0.652	0.431	0.136	0.815
TaMAPKK13	TaMEKK4	4565.A0A3B6N2X8	4565.A0A3B6NRN9	0	0	0	0.623	0.05	0.652	0.431	0.136	0.815
TaMAPKK14	TaMEKK7	4565.A0A3B6HY95	4565.A0A077RUI2	0	0	0.175	0.705	0.05	0.652	0.431	0.136	0.841
TaMAPKK14	TaRaf18	4565.A0A3B6HY95	4565.A0A077RY41	0	0	0.202	0.623	0	0.172	0.176	0	0.408
TaMAPKK14	TaMEKK9	4565.A0A3B6HY95	4565.A0A077S2G5	0	0	0.189	0.662	0.05	0.652	0.431	0.136	0.844
TaMAPKK14	TaRaf95	4565.A0A3B6HY95	4565.A0A1D5UN01	0	0	0.196	0.594	0	0.172	0.176	0	0.403
TaMAPKK14	TaRaf88	4565.A0A3B6HY95	4565.A0A3B5Z5X1	0	0	0.207	0.612	0	0.172	0.176	0	0.412
TaMAPKK14	TaRaf30	4565.A0A3B6HY95	4565.A0A3B6A1Z4	0	0	0.205	0.613	0	0.172	0.176	0	0.41
TaMAPKK14	TaRaf102	4565.A0A3B6HY95	4565.A0A3B6AQT7	0	0	0.214	0	0	0.172	0.176	0	0.416
TaMAPKK14	TaRaf19	4565.A0A3B6HY95	4565.A0A3B6AWY0	0	0	0.243	0	0	0.172	0.176	0	0.438
TaMAPKK14	TaMAPKKK4	4565.A0A3B6HY95	4565.A0A3B6AY64	0	0	0.109	0.69	0	0.176	0.579	0.195	0.718
TaMAPKK14	TaMEKK1	4565.A0A3B6HY95	4565.A0A3B6B3I4	0	0	0.158	0.728	0.05	0.652	0.573	0.136	0.878
TaMAPKK14	TaMEKK20	4565.A0A3B6HY95	4565.A0A3B6B6T5	0	0	0	0.699	0.05	0.652	0.431	0.136	0.815
TaMAPKK14	TaRaf105	4565.A0A3B6HY95	4565.A0A3B6C472	0	0	0.228	0	0	0.172	0.176	0	0.427
TaMAPKK14	TaMAPKKK5	4565.A0A3B6HY95	4565.A0A3B6C4T6	0	0	0.109	0.69	0	0.176	0.579	0.195	0.717
TaMAPKK14	TaMEKK18	4565.A0A3B6HY95	4565.A0A3B6CEA6	0	0	0	0.699	0.05	0.652	0.431	0.136	0.815
TaMAPKK14	TaMAPKKK6	4565.A0A3B6HY95	4565.A0A3B6DEP0	0	0	0.11	0.69	0	0.176	0.579	0.195	0.718
TaMAPKK14	TaRaf60	4565.A0A3B6HY95	4565.A0A3B6EHW0	0	0	0.209	0.585	0	0.172	0.176	0	0.413

TaMAPKK 14	TaMEKK8	4565.A0A3 B6HY95	4565.A0A3 B6FNU8	0	0	0.177	0.695	0.05	0.652	0.431	0.136	0.841
TaMAPKK 14	TaRaf73	4565.A0A3 B6HY95	4565.A0A3 B6GRN8	0	0	0.222	0	0	0.172	0.176	0	0.422
TaMAPKK 14	TaRaf5	4565.A0A3 B6HY95	4565.A0A3 B6GYQ1	0	0	0.21	0.589	0	0.172	0.176	0	0.414
TaMAPKK 14	TaRaf46	4565.A0A3 B6HY95	4565.A0A3 B6MW69	0	0	0.201	0.616	0	0.172	0.176	0	0.407
TaMAPKK 14	TaRaf62	4565.A0A3 B6HY95	4565.A9RA A9	0	0	0.212	0.601	0	0.172	0.176	0	0.415
TaMAPKK 14	TaRaf42	4565.A0A3 B6HY95	4565.A0A3 B6U554	0	0	0.213	0	0	0.172	0.176	0	0.416
TaMAPKK 14	TaRaf41	4565.A0A3 B6HY95	4565.A0A3 B6KLD7	0	0	0.213	0.592	0	0.172	0.176	0	0.416
TaMAPKK 14	TaRaf7	4565.A0A3 B6HY95	4565.A0A3 B6TBH5	0	0	0.216	0.594	0	0.172	0.176	0	0.418
TaMAPKK 14	TaRaf56	4565.A0A3 B6HY95	4565.A0A3 B6LPY3	0	0	0.217	0.586	0	0.172	0.176	0	0.419
TaMAPKK 14	TaRaf72	4565.A0A3 B6HY95	4565.A0A3 B6MXW5	0	0	0.232	0	0	0.172	0.176	0	0.43
TaMAPKK 14	TaRaf50	4565.A0A3 B6HY95	4565.A0A3 B6RET0	0	0	0.266	0	0	0.172	0.176	0	0.455
TaMAPKK 14	TaRaf43	4565.A0A3 B6HY95	4565.A0A3 B6KNC6	0	0	0.281	0	0	0.172	0.176	0	0.466
TaMAPKK 14	TaRaf91	4565.A0A3 B6HY95	4565.A0A3 B6PMI5	0	0	0.133	0.604	0	0.172	0.489	0	0.601
TaMAPKK 14	TaMEKK4-1	4565.A0A3 B6HY95	4565.A0A3 B6PNI6	0	0	0.174	0.695	0.05	0.652	0.573	0.136	0.88
TaMAPKK 14	TaMEKK4	4565.A0A3 B6HY95	4565.A0A3 B6NRN9	0	0	0.175	0.693	0.05	0.652	0.573	0.136	0.881
TaMAPKK 14	TaMEKK29	4565.A0A3 B6HY95	4565.A0A3 B6MSP6	0	0	0.16	0.739	0.05	0.652	0.614	0.136	0.89
TaMAPKK 14	TaMEKK24	4565.A0A3 B6HY95	4565.A0A3 B6LLV5	0	0	0.16	0.739	0.05	0.652	0.614	0.136	0.89
TaMAPKK 14	TaMEKK17	4565.A0A3 B6HY95	4565.A0A3 B6KFL8	0	0	0.16	0.739	0.05	0.652	0.614	0.136	0.89

TaMAPKK 15	TaMEKK7	4565.A0A3 B6HW51	4565.A0A0 77RUI2	0	0	0.174	0.709	0.05	0.652	0.431	0.136	0.841
TaMAPKK 15	TaRaf18	4565.A0A3 B6HW51	4565.A0A0 77RY41	0	0	0.201	0.619	0	0.172	0.176	0	0.407
TaMAPKK 15	TaMEKK9	4565.A0A3 B6HW51	4565.A0A0 77S2G5	0	0	0.191	0.661	0.05	0.652	0.431	0.136	0.844
TaMAPKK 15	TaRaf95	4565.A0A3 B6HW51	4565.A0A1 D5UN01	0	0	0.201	0.598	0	0.172	0.176	0	0.407
TaMAPKK 15	TaRaf88	4565.A0A3 B6HW51	4565.A0A3 B5Z5X1	0	0	0.206	0.608	0	0.172	0.176	0	0.411
TaMAPKK 15	TaRaf30	4565.A0A3 B6HW51	4565.A0A3 B6A1Z4	0	0	0.203	0.609	0	0.172	0.176	0	0.409
TaMAPKK 15	TaRaf102	4565.A0A3 B6HW51	4565.A0A3 B6AQT7	0	0	0.214	0	0	0.172	0.176	0	0.416
TaMAPKK 15	TaRaf19	4565.A0A3 B6HW51	4565.A0A3 B6AWY0	0	0	0.249	0	0	0.172	0.176	0	0.442
TaMAPKK 15	TaMAPKK KK4	4565.A0A3 B6HW51	4565.A0A3 B6AY64	0	0	0.111	0.683	0	0.176	0.579	0.195	0.718
TaMAPKK 15	TaMEKK1	4565.A0A3 B6HW51	4565.A0A3 B6B3I4	0	0	0.159	0.726	0.05	0.652	0.573	0.136	0.878
TaMAPKK 15	TaMEKK2 0	4565.A0A3 B6HW51	4565.A0A3 B6B6T5	0	0	0	0.699	0.05	0.652	0.431	0.136	0.815
TaMAPKK 15	TaRaf105	4565.A0A3 B6HW51	4565.A0A3 B6C472	0	0	0.235	0	0	0.172	0.176	0	0.432
TaMAPKK 15	TaMAPKK KK5	4565.A0A3 B6HW51	4565.A0A3 B6C4T6	0	0	0.111	0.682	0	0.176	0.579	0.195	0.718
TaMAPKK 15	TaMEKK1 8	4565.A0A3 B6HW51	4565.A0A3 B6CEA6	0	0	0	0.699	0.05	0.652	0.431	0.136	0.815
TaMAPKK 15	TaMAPKK KK6	4565.A0A3 B6HW51	4565.A0A3 B6DEP0	0	0	0.112	0.683	0	0.176	0.579	0.195	0.719
TaMAPKK 15	TaRaf60	4565.A0A3 B6HW51	4565.A0A3 B6EHW0	0	0	0.204	0.592	0	0.172	0.176	0	0.409
TaMAPKK 15	TaMEKK8	4565.A0A3 B6HW51	4565.A0A3 B6FNU8	0	0	0.172	0.703	0.05	0.652	0.431	0.136	0.84
TaMAPKK 15	TaRaf73	4565.A0A3 B6HW51	4565.A0A3 B6GRN8	0	0	0.229	0	0	0.172	0.176	0	0.428

TaMAPKK 15	TaRaf5	4565.A0A3 B6HW51	4565.A0A3 B6GYQ1	0	0	0.204	0.596	0	0.172	0.176	0	0.409
TaMAPKK 15	TaRaf46	4565.A0A3 B6HW51	4565.A0A3 B6MW69	0	0	0.198	0.615	0	0.172	0.176	0	0.405
TaMAPKK 15	TaRaf62	4565.A0A3 B6HW51	4565.A9RA A9	0	0	0.207	0.608	0	0.172	0.176	0	0.412
TaMAPKK 15	TaRaf42	4565.A0A3 B6HW51	4565.A0A3 B6U554	0	0	0.211	0	0	0.172	0.176	0	0.414
TaMAPKK 15	TaRaf41	4565.A0A3 B6HW51	4565.A0A3 B6KLD7	0	0	0.214	0.593	0	0.172	0.176	0	0.417
TaMAPKK 15	TaRaf56	4565.A0A3 B6HW51	4565.A0A3 B6LPY3	0	0	0.218	0.587	0	0.172	0.176	0	0.42
TaMAPKK 15	TaRaf7	4565.A0A3 B6HW51	4565.A0A3 B6TBH5	0	0	0.22	0.591	0	0.172	0.176	0	0.421
TaMAPKK 15	TaRaf72	4565.A0A3 B6HW51	4565.A0A3 B6MXW5	0	0	0.233	0	0	0.172	0.176	0	0.431
TaMAPKK 15	TaRaf50	4565.A0A3 B6HW51	4565.A0A3 B6RET0	0	0	0.268	0	0	0.172	0.176	0	0.456
TaMAPKK 15	TaRaf43	4565.A0A3 B6HW51	4565.A0A3 B6KNC6	0	0	0.278	0	0	0.172	0.176	0	0.464
TaMAPKK 15	TaRaf91	4565.A0A3 B6HW51	4565.A0A3 B6PMI5	0	0	0.139	0.597	0	0.172	0.489	0	0.604
TaMAPKK 15	TaMEKK4-1	4565.A0A3 B6HW51	4565.A0A3 B6PNI6	0	0	0.173	0.702	0.05	0.652	0.573	0.136	0.88
TaMAPKK 15	TaMEKK4	4565.A0A3 B6HW51	4565.A0A3 B6NRN9	0	0	0.174	0.699	0.05	0.652	0.573	0.136	0.88
TaMAPKK 15	TaMEKK2 4	4565.A0A3 B6HW51	4565.A0A3 B6LLV5	0	0	0.159	0.742	0.05	0.652	0.614	0.136	0.89
TaMAPKK 15	TaMEKK2 9	4565.A0A3 B6HW51	4565.A0A3 B6MSP6	0	0	0.159	0.741	0.05	0.652	0.614	0.136	0.89
TaMAPKK 15	TaMEKK1 7	4565.A0A3 B6HW51	4565.A0A3 B6KFL8	0	0	0.159	0.741	0.05	0.652	0.614	0.136	0.89
TaMAPKK 16	TaMEKK7	4565.A0A3 B6HZP7	4565.A0A0 77RUI2	0	0	0.183	0.687	0.05	0.652	0.431	0.136	0.843
TaMAPKK 16	TaRaf18	4565.A0A3 B6HZP7	4565.A0A0 77RY41	0	0	0.199	0.627	0	0.172	0.176	0	0.406

TaMAPKK16	TaMEKK9	4565.A0A3B6HZP7	4565.A0A077S2G5	0	0	0.197	0.641	0.05	0.652	0.431	0.136	0.845
TaMAPKK16	TaRaf95	4565.A0A3B6HZP7	4565.A0A1D5UN01	0	0	0.191	0.609	0	0.172	0.176	0	0.4
TaMAPKK16	TaRaf88	4565.A0A3B6HZP7	4565.A0A3B5Z5X1	0	0	0.205	0.612	0	0.172	0.176	0	0.41
TaMAPKK16	TaRaf30	4565.A0A3B6HZP7	4565.A0A3B6A1Z4	0	0	0.203	0.612	0	0.172	0.176	0	0.409
TaMAPKK16	TaRaf102	4565.A0A3B6HZP7	4565.A0A3B6AQT7	0	0	0.232	0	0	0.172	0.176	0	0.43
TaMAPKK16	TaRaf19	4565.A0A3B6HZP7	4565.A0A3B6AWY0	0	0	0.269	0	0	0.172	0.176	0	0.457
TaMAPKK16	TaMAPKKK4	4565.A0A3B6HZP7	4565.A0A3B6AY64	0	0	0.114	0.682	0	0.176	0.579	0.195	0.719
TaMAPKK16	TaMEKK1	4565.A0A3B6HZP7	4565.A0A3B6B3I4	0	0	0.155	0.732	0.05	0.652	0.573	0.136	0.878
TaMAPKK16	TaMEKK20	4565.A0A3B6HZP7	4565.A0A3B6B6T5	0	0	0	0.701	0.05	0.652	0.431	0.136	0.815
TaMAPKK16	TaRaf105	4565.A0A3B6HZP7	4565.A0A3B6C472	0	0	0.251	0	0	0.172	0.176	0	0.444
TaMAPKK16	TaMAPKKK5	4565.A0A3B6HZP7	4565.A0A3B6C4T6	0	0	0.113	0.683	0	0.176	0.579	0.195	0.719
TaMAPKK16	TaMEKK18	4565.A0A3B6HZP7	4565.A0A3B6CEA6	0	0	0	0.702	0.05	0.652	0.431	0.136	0.815
TaMAPKK16	TaMAPKKK6	4565.A0A3B6HZP7	4565.A0A3B6DEP0	0	0	0.114	0.683	0	0.176	0.579	0.195	0.719
TaMAPKK16	TaRaf60	4565.A0A3B6HZP7	4565.A0A3B6EHW0	0	0	0.203	0.587	0	0.172	0.176	0	0.409
TaMAPKK16	TaMEKK8	4565.A0A3B6HZP7	4565.A0A3B6FNU8	0	0	0.179	0.688	0.05	0.652	0.431	0.136	0.842
TaMAPKK16	TaRaf5	4565.A0A3B6HZP7	4565.A0A3B6GYQ1	0	0	0.204	0.594	0	0.172	0.176	0	0.409
TaMAPKK16	TaRaf46	4565.A0A3B6HZP7	4565.A0A3B6MW69	0	0	0.201	0.612	0	0.172	0.176	0	0.407
TaMAPKK16	TaRaf83	4565.A0A3B6HZP7	4565.A0A3B6MWW7	0	0	0.208	0	0	0.172	0.176	0	0.412

TaMAPKK16	TaRaf44	4565.A0A3B6HZP7	4565.A0A3B6KNC2	0	0	0.21	0	0	0.172	0.176	0	0.413
TaMAPKK16	TaRaf62	4565.A0A3B6HZP7	4565.A9RAA9	0	0	0.21	0.597	0	0.172	0.176	0	0.414
TaMAPKK16	TaRaf41	4565.A0A3B6HZP7	4565.A0A3B6KLD7	0	0	0.217	0.589	0	0.172	0.176	0	0.419
TaMAPKK16	TaRaf56	4565.A0A3B6HZP7	4565.A0A3B6LPY3	0	0	0.217	0.589	0	0.172	0.176	0	0.419
TaMAPKK16	TaRaf7	4565.A0A3B6HZP7	4565.A0A3B6TBH5	0	0	0.218	0.592	0	0.172	0.176	0	0.42
TaMAPKK16	TaRaf42	4565.A0A3B6HZP7	4565.A0A3B6U554	0	0	0.223	0	0	0.172	0.176	0	0.423
TaMAPKK16	TaRaf45	4565.A0A3B6HZP7	4565.A0A3B6KN82	0	0	0.238	0	0	0.172	0.176	0	0.434
TaMAPKK16	TaRaf50	4565.A0A3B6HZP7	4565.A0A3B6RET0	0	0	0.286	0	0	0.172	0.176	0	0.47
TaMAPKK16	TaRaf43	4565.A0A3B6HZP7	4565.A0A3B6KNC6	0	0	0.291	0	0	0.172	0.176	0	0.474
TaMAPKK16	TaRaf91	4565.A0A3B6HZP7	4565.A0A3B6PMI5	0	0	0.129	0.602	0	0.172	0.489	0	0.599
TaMAPKK16	TaMEKK4-1	4565.A0A3B6HZP7	4565.A0A3B6PNI6	0	0	0.166	0.709	0.05	0.652	0.573	0.136	0.879
TaMAPKK16	TaMEKK4	4565.A0A3B6HZP7	4565.A0A3B6NRN9	0	0	0.167	0.705	0.05	0.652	0.573	0.136	0.879
TaMAPKK16	TaMEKK24	4565.A0A3B6HZP7	4565.A0A3B6LLV5	0	0	0.158	0.741	0.05	0.652	0.614	0.136	0.89
TaMAPKK16	TaMEKK29	4565.A0A3B6HZP7	4565.A0A3B6MSP6	0	0	0.158	0.742	0.05	0.652	0.614	0.136	0.89
TaMAPKK16	TaMEKK17	4565.A0A3B6HZP7	4565.A0A3B6KFL8	0	0	0.158	0.741	0.05	0.652	0.614	0.136	0.89
TaMAPKK17	TaMEKK7	4565.A0A3B6I0M7	4565.A0A077RUI2	0	0	0.178	0.702	0.05	0.652	0.431	0.136	0.842
TaMAPKK17	TaRaf18	4565.A0A3B6I0M7	4565.A0A077RY41	0	0	0.201	0.633	0	0.172	0.176	0	0.407
TaMAPKK17	TaMEKK9	4565.A0A3B6I0M7	4565.A0A077S2G5	0	0	0.195	0.65	0.05	0.652	0.431	0.136	0.845

TaMAPKK17	TaRaf95	4565.A0A3B6I0M7	4565.A0A1D5UN01	0	0	0.191	0.606	0	0.172	0.176	0	0.4
TaMAPKK17	TaRaf88	4565.A0A3B6I0M7	4565.A0A3B5Z5X1	0	0	0.206	0.628	0	0.172	0.176	0	0.411
TaMAPKK17	TaRaf30	4565.A0A3B6I0M7	4565.A0A3B6A1Z4	0	0	0.203	0.629	0	0.172	0.176	0	0.409
TaMAPKK17	TaMAPKKK4	4565.A0A3B6I0M7	4565.A0A3B6AY64	0	0	0.108	0.692	0	0.176	0.579	0.195	0.717
TaMAPKK17	TaMEKK1	4565.A0A3B6I0M7	4565.A0A3B6B3I4	0	0	0.148	0.742	0.05	0.652	0.573	0.136	0.877
TaMAPKK17	TaMEKK20	4565.A0A3B6I0M7	4565.A0A3B6B6T5	0	0	0	0.691	0.05	0.652	0.431	0.136	0.815
TaMAPKK17	TaMAPKKK5	4565.A0A3B6I0M7	4565.A0A3B6C4T6	0	0	0.108	0.691	0	0.176	0.579	0.195	0.717
TaMAPKK17	TaMEKK18	4565.A0A3B6I0M7	4565.A0A3B6CEA6	0	0	0	0.693	0.05	0.652	0.431	0.136	0.815
TaMAPKK17	TaMAPKKK6	4565.A0A3B6I0M7	4565.A0A3B6DEP0	0	0	0.109	0.692	0	0.176	0.579	0.195	0.718
TaMAPKK17	TaRaf59	4565.A0A3B6I0M7	4565.A0A3B6E9E2	0	0	0.192	0.609	0	0.172	0.176	0	0.4
TaMAPKK17	TaRaf60	4565.A0A3B6I0M7	4565.A0A3B6EHW0	0	0	0.212	0.589	0	0.172	0.176	0	0.415
TaMAPKK17	TaMEKK8	4565.A0A3B6I0M7	4565.A0A3B6FNU8	0	0	0.178	0.697	0.05	0.652	0.431	0.136	0.842
TaMAPKK17	TaRaf5	4565.A0A3B6I0M7	4565.A0A3B6GYQ1	0	0	0.212	0.593	0	0.172	0.176	0	0.415
TaMAPKK17	TaRaf21	4565.A0A3B6I0M7	4565.A0A3B6TVC5	0	0	0.192	0.592	0	0.172	0.176	0	0.401
TaMAPKK17	TaRaf1	4565.A0A3B6I0M7	4565.A0A3B6NP57	0	0	0.194	0.589	0	0.172	0.176	0	0.402
TaMAPKK17	TaRaf62	4565.A0A3B6I0M7	4565.A9RAA9	0	0	0.207	0.62	0	0.172	0.176	0	0.411
TaMAPKK17	TaRaf7	4565.A0A3B6I0M7	4565.A0A3B6TBH5	0	0	0.207	0.602	0	0.172	0.176	0	0.412
TaMAPKK17	TaRaf41	4565.A0A3B6I0M7	4565.A0A3B6KLD7	0	0	0.209	0.607	0	0.172	0.176	0	0.413

TaMAPKK 17	TaRaf56	4565.A0A3 B6I0M7	4565.A0A3 B6LPY3	0	0	0.216	0.592	0	0.172	0.176	0	0.419
TaMAPKK 17	TaRaf91	4565.A0A3 B6I0M7	4565.A0A3 B6PMI5	0	0	0.151	0.597	0	0.172	0.489	0	0.609
TaMAPKK 17	TaMEKK4-1	4565.A0A3 B6I0M7	4565.A0A3 B6PNI6	0	0	0.161	0.716	0.05	0.652	0.573	0.136	0.879
TaMAPKK 17	TaMEKK4	4565.A0A3 B6I0M7	4565.A0A3 B6NRN9	0	0	0.161	0.714	0.05	0.652	0.573	0.136	0.879
TaMAPKK 17	TaMEKK2 4	4565.A0A3 B6I0M7	4565.A0A3 B6LLV5	0	0	0.162	0.732	0.05	0.652	0.614	0.136	0.89
TaMAPKK 17	TaMEKK2 9	4565.A0A3 B6I0M7	4565.A0A3 B6MSP6	0	0	0.162	0.732	0.05	0.652	0.614	0.136	0.89
TaMAPKK 17	TaMEKK1 7	4565.A0A3 B6I0M7	4565.A0A3 B6KFL8	0	0	0.162	0.732	0.05	0.652	0.614	0.136	0.89
TaMAPKK 18	TaMEKK7	4565.A0A3 B6LJ27	4565.A0A0 77RUI2	0	0	0.14	0.613	0.05	0.652	0.823	0.136	0.948
TaMAPKK 18	TaMEKK9	4565.A0A3 B6LJ27	4565.A0A0 77S2G5	0	0	0.139	0.633	0.05	0.652	0.791	0.136	0.939
TaMAPKK 18	TaMAPKK KK4	4565.A0A3 B6LJ27	4565.A0A3 B6AY64	0	0	0	0.673	0	0.176	0.579	0.195	0.696
TaMAPKK 18	TaMEKK1	4565.A0A3 B6LJ27	4565.A0A3 B6B3I4	0	0	0.1	0.651	0.05	0.652	0.431	0.136	0.827
TaMAPKK 18	TaMEKK2 0	4565.A0A3 B6LJ27	4565.A0A3 B6B6T5	0	0	0	0.634	0.05	0.652	0.431	0.136	0.815
TaMAPKK 18	TaMAPKK KK5	4565.A0A3 B6LJ27	4565.A0A3 B6C4T6	0	0	0	0.673	0	0.176	0.579	0.195	0.696
TaMAPKK 18	TaMEKK1 8	4565.A0A3 B6LJ27	4565.A0A3 B6CEA6	0	0	0	0.633	0.05	0.652	0.431	0.136	0.815
TaMAPKK 18	TaMAPKK KK6	4565.A0A3 B6LJ27	4565.A0A3 B6DEP0	0	0	0	0.673	0	0.176	0.579	0.195	0.696
TaMAPKK 18	TaMEKK8	4565.A0A3 B6LJ27	4565.A0A3 B6FNU8	0	0	0.139	0.602	0.05	0.652	0.791	0.136	0.939
TaMAPKK 18	TaMEKK1 7	4565.A0A3 B6LJ27	4565.A0A3 B6KFL8	0	0	0.157	0.644	0.05	0.652	0.431	0.136	0.838
TaMAPKK 18	TaRaf52	4565.A0A3 B6LJ27	4565.A0A3 B6LRR0	0	0	0.196	0.603	0	0.172	0.176	0	0.403

TaMAPKK18	TaMEKK4	4565.A0A3B6LJ27	4565.A0A3B6NRN9	0	0	0.107	0.645	0.05	0.652	0.431	0.136	0.828
TaMAPKK18	TaMEKK4-1	4565.A0A3B6LJ27	4565.A0A3B6PNI6	0	0	0.109	0.645	0.05	0.652	0.431	0.136	0.828
TaMAPKK18	TaMEKK24	4565.A0A3B6LJ27	4565.A0A3B6LLV5	0	0	0.157	0.644	0.05	0.652	0.431	0.136	0.838
TaMAPKK18	TaMEKK29	4565.A0A3B6LJ27	4565.A0A3B6MSP6	0	0	0.158	0.643	0.05	0.652	0.431	0.136	0.838
TaMAPKK2	TaMEKK7	4565.A0A3B6LYW0	4565.A0A077RUI2	0	0	0.154	0.622	0.05	0.652	0.927	0.136	0.979
TaMAPKK2	TaMEKK9	4565.A0A3B6LYW0	4565.A0A077S2G5	0	0	0.149	0.653	0.05	0.652	0.791	0.136	0.939
TaMAPKK2	TaMAPKKK4	4565.A0A3B6LYW0	4565.A0A3B6AY64	0	0	0	0.657	0	0.176	0.579	0.195	0.696
TaMAPKK2	TaMEKK1	4565.A0A3B6LYW0	4565.A0A3B6B3I4	0	0	0.109	0.639	0.05	0.652	0.431	0.136	0.828
TaMAPKK2	TaMEKK20	4565.A0A3B6LYW0	4565.A0A3B6B6T5	0	0	0	0.637	0.05	0.652	0.431	0.136	0.815
TaMAPKK2	TaMAPKKK5	4565.A0A3B6LYW0	4565.A0A3B6C4T6	0	0	0	0.657	0	0.176	0.579	0.195	0.696
TaMAPKK2	TaMEKK18	4565.A0A3B6LYW0	4565.A0A3B6CEA6	0	0	0	0.636	0.05	0.652	0.431	0.136	0.815
TaMAPKK2	TaMAPKKK6	4565.A0A3B6LYW0	4565.A0A3B6DEP0	0	0	0	0.657	0	0.176	0.579	0.195	0.696
TaMAPKK2	TaMEKK8	4565.A0A3B6LYW0	4565.A0A3B6FNU8	0	0	0.149	0.628	0.05	0.652	0.946	0.136	0.984
TaMAPKK2	TaMEKK17	4565.A0A3B6LYW0	4565.A0A3B6KFL8	0	0	0.166	0.648	0.05	0.652	0.431	0.136	0.839
TaMAPKK2	TaMEKK24	4565.A0A3B6LYW0	4565.A0A3B6LLV5	0	0	0.167	0.648	0.05	0.652	0.431	0.136	0.84
TaMAPKK2	TaRaf52	4565.A0A3B6LYW0	4565.A0A3B6LRR0	0	0	0.194	0.598	0	0.172	0.176	0	0.402
TaMAPKK2	TaMEKK4	4565.A0A3B6LYW0	4565.A0A3B6NRN9	0	0	0.114	0.632	0.05	0.652	0.431	0.136	0.829
TaMAPKK2	TaMEKK4-1	4565.A0A3B6LYW0	4565.A0A3B6PNI6	0	0	0.116	0.634	0.05	0.652	0.431	0.136	0.83

TaMAPKK2	TaMEKK29	4565.A0A3B6LYW0	4565.A0A3B6MSP6	0	0	0.166	0.648	0.05	0.652	0.431	0.136	0.839
TaMAPKK3	TaMEKK7	4565.A0A3B6MNP8	4565.A0A077RUI2	0	0	0.124	0.615	0.05	0.652	0.823	0.136	0.947
TaMAPKK3	TaMEKK9	4565.A0A3B6MNP8	4565.A0A077S2G5	0	0	0.124	0.627	0.05	0.652	0.791	0.136	0.938
TaMAPKK3	TaMAPKKK4	4565.A0A3B6MNP8	4565.A0A3B6AY64	0	0	0	0.653	0	0.176	0.579	0.195	0.696
TaMAPKK3	TaMEKK1	4565.A0A3B6MNP8	4565.A0A3B6B3I4	0	0	0	0.632	0.05	0.652	0.431	0.136	0.815
TaMAPKK3	TaMEKK20	4565.A0A3B6MNP8	4565.A0A3B6B6T5	0	0	0	0.62	0.05	0.652	0.431	0.136	0.815
TaMAPKK3	TaMAPKKK5	4565.A0A3B6MNP8	4565.A0A3B6C4T6	0	0	0	0.652	0	0.176	0.579	0.195	0.696
TaMAPKK3	TaMEKK18	4565.A0A3B6MNP8	4565.A0A3B6CEA6	0	0	0	0.62	0.05	0.652	0.431	0.136	0.815
TaMAPKK3	TaMAPKKK6	4565.A0A3B6MNP8	4565.A0A3B6DEP0	0	0	0	0.652	0	0.176	0.579	0.195	0.696
TaMAPKK3	TaMEKK8	4565.A0A3B6MNP8	4565.A0A3B6FNU8	0	0	0.12	0.602	0.05	0.652	0.791	0.136	0.937
TaMAPKK3	TaMEKK17	4565.A0A3B6MNP8	4565.A0A3B6KFL8	0	0	0.155	0.627	0.05	0.652	0.431	0.136	0.837
TaMAPKK3	TaMEKK24	4565.A0A3B6MNP8	4565.A0A3B6LLV5	0	0	0.155	0.627	0.05	0.652	0.431	0.136	0.837
TaMAPKK3	TaRaf52	4565.A0A3B6MNP8	4565.A0A3B6LRR0	0	0	0.2	0.59	0	0.172	0.176	0	0.406
TaMAPKK3	TaMEKK4-1	4565.A0A3B6MNP8	4565.A0A3B6PNI6	0	0	0.107	0.628	0.05	0.652	0.431	0.136	0.828
TaMAPKK3	TaMEKK4	4565.A0A3B6MNP8	4565.A0A3B6NRN9	0	0	0.105	0.627	0.05	0.652	0.431	0.136	0.828
TaMAPKK3	TaMEKK29	4565.A0A3B6MNP8	4565.A0A3B6MSP6	0	0	0.154	0.627	0.05	0.652	0.431	0.136	0.837
TaMAPKK4	TaMEKK7	4565.A0A3B6KFB5	4565.A0A077RUI2	0	0	0.135	0.689	0.05	0.652	0.823	0.136	0.948
TaMAPKK4	TaMEKK9	4565.A0A3B6KFB5	4565.A0A077S2G5	0	0	0.152	0.656	0.05	0.652	0.791	0.136	0.94

TaMAPKK4	TaMAPKKKK4	4565.A0A3B6KFB5	4565.A0A3B6AY64	0	0	0	0.776	0	0.176	0.579	0.195	0.696
TaMAPKK4	TaMEKK1	4565.A0A3B6KFB5	4565.A0A3B6B3I4	0	0	0.094	0.749	0.05	0.652	0.431	0.136	0.825
TaMAPKK4	TaMEKK20	4565.A0A3B6KFB5	4565.A0A3B6B6T5	0	0	0	0.724	0.05	0.652	0.431	0.136	0.815
TaMAPKK4	TaMAPKKKK5	4565.A0A3B6KFB5	4565.A0A3B6C4T6	0	0	0	0.776	0	0.176	0.579	0.195	0.696
TaMAPKK4	TaMEKK18	4565.A0A3B6KFB5	4565.A0A3B6CEA6	0	0	0	0.723	0.05	0.652	0.431	0.136	0.815
TaMAPKK4	TaMAPKKKK6	4565.A0A3B6KFB5	4565.A0A3B6DEP0	0	0	0	0.776	0	0.176	0.579	0.195	0.696
TaMAPKK4	TaMEKK8	4565.A0A3B6KFB5	4565.A0A3B6FNU8	0	0	0.14	0.66	0.05	0.652	0.791	0.136	0.939
TaMAPKK4	TaMEKK4-1	4565.A0A3B6KFB5	4565.A0A3B6PNI6	0	0	0.102	0.741	0.05	0.652	0.431	0.136	0.827
TaMAPKK4	TaMEKK4	4565.A0A3B6KFB5	4565.A0A3B6NRN9	0	0	0.1	0.74	0.05	0.652	0.431	0.136	0.827
TaMAPKK4	TaMEKK24	4565.A0A3B6KFB5	4565.A0A3B6LLV5	0	0	0.138	0.731	0.05	0.652	0.431	0.136	0.834
TaMAPKK4	TaMEKK29	4565.A0A3B6KFB5	4565.A0A3B6MSP6	0	0	0.138	0.73	0.05	0.652	0.431	0.136	0.834
TaMAPKK4	TaMEKK17	4565.A0A3B6KFB5	4565.A0A3B6KFL8	0	0	0.138	0.73	0.05	0.652	0.431	0.136	0.834
TaMAPKK5	TaMEKK7	4565.A0A3B6IMW7	4565.A0A077RUI2	0	0	0.181	0.689	0.05	0.652	0.431	0.136	0.842
TaMAPKK5	TaRaf18	4565.A0A3B6IMW7	4565.A0A077RY41	0	0	0.195	0.62	0	0.172	0.176	0	0.403
TaMAPKK5	TaMEKK9	4565.A0A3B6IMW7	4565.A0A077S2G5	0	0	0.192	0.652	0.05	0.652	0.431	0.136	0.844
TaMAPKK5	TaRaf95	4565.A0A3B6IMW7	4565.A0A1D5UN01	0	0	0.192	0.592	0	0.172	0.176	0	0.401
TaMAPKK5	TaRaf88	4565.A0A3B6IMW7	4565.A0A3B5Z5X1	0	0	0.201	0.607	0	0.172	0.176	0	0.407
TaMAPKK5	TaRaf30	4565.A0A3B6IMW7	4565.A0A3B6A1Z4	0	0	0.199	0.609	0	0.172	0.176	0	0.406

TaMAPKK5	TaRaf102	4565.A0A3B6IMW7	4565.A0A3B6AQT7	0	0	0.212	0	0	0.172	0.176	0	0.415
TaMAPKK5	TaRaf19	4565.A0A3B6IMW7	4565.A0A3B6AWY0	0	0	0.255	0	0	0.172	0.176	0	0.447
TaMAPKK5	TaMAPKKK4	4565.A0A3B6IMW7	4565.A0A3B6AY64	0	0	0.112	0.691	0	0.176	0.579	0.195	0.718
TaMAPKK5	TaMEKK1	4565.A0A3B6IMW7	4565.A0A3B6B3I4	0	0	0.16	0.72	0.05	0.652	0.573	0.136	0.878
TaMAPKK5	TaMEKK20	4565.A0A3B6IMW7	4565.A0A3B6B6T5	0	0	0	0.687	0.05	0.652	0.431	0.136	0.815
TaMAPKK5	TaRaf105	4565.A0A3B6IMW7	4565.A0A3B6C472	0	0	0.24	0	0	0.172	0.176	0	0.436
TaMAPKK5	TaMAPKKK5	4565.A0A3B6IMW7	4565.A0A3B6C4T6	0	0	0.111	0.691	0	0.176	0.579	0.195	0.718
TaMAPKK5	TaMEKK18	4565.A0A3B6IMW7	4565.A0A3B6CEA6	0	0	0	0.688	0.05	0.652	0.431	0.136	0.815
TaMAPKK5	TaMAPKKK6	4565.A0A3B6IMW7	4565.A0A3B6DEP0	0	0	0.113	0.692	0	0.176	0.579	0.195	0.719
TaMAPKK5	TaRaf60	4565.A0A3B6IMW7	4565.A0A3B6EHW0	0	0	0.201	0.588	0	0.172	0.176	0	0.407
TaMAPKK5	TaMEKK8	4565.A0A3B6IMW7	4565.A0A3B6FNU8	0	0	0.18	0.679	0.05	0.652	0.431	0.136	0.842
TaMAPKK5	TaRaf73	4565.A0A3B6IMW7	4565.A0A3B6GRN8	0	0	0.227	0	0	0.172	0.176	0	0.426
TaMAPKK5	TaRaf5	4565.A0A3B6IMW7	4565.A0A3B6GYQ1	0	0	0.201	0.594	0	0.172	0.176	0	0.407
TaMAPKK5	TaRaf46	4565.A0A3B6IMW7	4565.A0A3B6MW69	0	0	0.196	0.614	0	0.172	0.176	0	0.404
TaMAPKK5	TaRaf62	4565.A0A3B6IMW7	4565.A9RAA9	0	0	0.207	0.598	0	0.172	0.176	0	0.411
TaMAPKK5	TaRaf41	4565.A0A3B6IMW7	4565.A0A3B6KLD7	0	0	0.209	0.588	0	0.172	0.176	0	0.413
TaMAPKK5	TaRaf56	4565.A0A3B6IMW7	4565.A0A3B6LPY3	0	0	0.211	0.583	0	0.172	0.176	0	0.414
TaMAPKK5	TaRaf7	4565.A0A3B6IMW7	4565.A0A3B6TBH5	0	0	0.216	0.592	0	0.172	0.176	0	0.419

TaMAPKK5	TaRaf42	4565.A0A3B6IMW7	4565.A0A3B6U554	0	0	0.217	0	0	0.172	0.176	0	0.419
TaMAPKK5	TaRaf50	4565.A0A3B6IMW7	4565.A0A3B6RET0	0	0	0.261	0	0	0.172	0.176	0	0.451
TaMAPKK5	TaRaf43	4565.A0A3B6IMW7	4565.A0A3B6KNC6	0	0	0.277	0	0	0.172	0.176	0	0.463
TaMAPKK5	TaRaf91	4565.A0A3B6IMW7	4565.A0A3B6PMI5	0	0	0.13	0.595	0	0.172	0.489	0	0.599
TaMAPKK5	TaMEKK4-1	4565.A0A3B6IMW7	4565.A0A3B6PNI6	0	0	0.172	0.698	0.05	0.652	0.573	0.136	0.88
TaMAPKK5	TaMEKK4	4565.A0A3B6IMW7	4565.A0A3B6NRN9	0	0	0.173	0.695	0.05	0.652	0.573	0.136	0.88
TaMAPKK5	TaMEKK17	4565.A0A3B6IMW7	4565.A0A3B6KFL8	0	0	0.164	0.731	0.05	0.652	0.614	0.136	0.891
TaMAPKK5	TaMEKK24	4565.A0A3B6IMW7	4565.A0A3B6LLV5	0	0	0.164	0.731	0.05	0.652	0.614	0.136	0.891
TaMAPKK5	TaMEKK29	4565.A0A3B6IMW7	4565.A0A3B6MSP6	0	0	0.164	0.731	0.05	0.652	0.614	0.136	0.891
TaMAPKK6	TaMEKK7	4565.A0A3B6ILF0	4565.A0A077RUI2	0	0	0.181	0.697	0.05	0.652	0.431	0.136	0.842
TaMAPKK6	TaRaf18	4565.A0A3B6ILF0	4565.A0A077RY41	0	0	0.202	0.628	0	0.172	0.176	0	0.408
TaMAPKK6	TaMEKK9	4565.A0A3B6ILF0	4565.A0A077S2G5	0	0	0.196	0.649	0.05	0.652	0.431	0.136	0.845
TaMAPKK6	TaRaf95	4565.A0A3B6ILF0	4565.A0A1D5UN01	0	0	0.192	0.6	0	0.172	0.176	0	0.4
TaMAPKK6	TaRaf88	4565.A0A3B6ILF0	4565.A0A3B5Z5X1	0	0	0.205	0.627	0	0.172	0.176	0	0.41
TaMAPKK6	TaRaf30	4565.A0A3B6ILF0	4565.A0A3B6A1Z4	0	0	0.206	0.622	0	0.172	0.176	0	0.411
TaMAPKK6	TaMAPKKK4	4565.A0A3B6ILF0	4565.A0A3B6AY64	0	0	0.108	0.684	0	0.176	0.579	0.195	0.717
TaMAPKK6	TaMEKK1	4565.A0A3B6ILF0	4565.A0A3B6B3I4	0	0	0.151	0.735	0.05	0.652	0.573	0.136	0.877
TaMAPKK6	TaMEKK20	4565.A0A3B6ILF0	4565.A0A3B6B6T5	0	0	0	0.687	0.05	0.652	0.431	0.136	0.815

TaMAPKK6	TaMAPKKKK5	4565.A0A3B6ILF0	4565.A0A3B6C4T6	0	0	0.108	0.683	0	0.176	0.579	0.195	0.717
TaMAPKK6	TaMEKK18	4565.A0A3B6ILF0	4565.A0A3B6CEA6	0	0	0	0.684	0.05	0.652	0.431	0.136	0.815
TaMAPKK6	TaMAPKKKK6	4565.A0A3B6ILF0	4565.A0A3B6DEP0	0	0	0.109	0.684	0	0.176	0.579	0.195	0.717
TaMAPKK6	TaRaf60	4565.A0A3B6ILF0	4565.A0A3B6EHW0	0	0	0.209	0.589	0	0.172	0.176	0	0.413
TaMAPKK6	TaMEKK8	4565.A0A3B6ILF0	4565.A0A3B6FNU8	0	0	0.177	0.698	0.05	0.652	0.431	0.136	0.841
TaMAPKK6	TaRaf5	4565.A0A3B6ILF0	4565.A0A3B6GYQ1	0	0	0.21	0.593	0	0.172	0.176	0	0.414
TaMAPKK6	TaRaf1	4565.A0A3B6ILF0	4565.A0A3B6NP57	0	0	0.194	0.585	0	0.172	0.176	0	0.402
TaMAPKK6	TaRaf46	4565.A0A3B6ILF0	4565.A0A3B6MW69	0	0	0.194	0.642	0	0.172	0.176	0	0.402
TaMAPKK6	TaRaf41	4565.A0A3B6ILF0	4565.A0A3B6KLD7	0	0	0.208	0.607	0	0.172	0.176	0	0.413
TaMAPKK6	TaRaf62	4565.A0A3B6ILF0	4565.A9RAA9	0	0	0.209	0.609	0	0.172	0.176	0	0.413
TaMAPKK6	TaRaf7	4565.A0A3B6ILF0	4565.A0A3B6TBH5	0	0	0.212	0.596	0	0.172	0.176	0	0.415
TaMAPKK6	TaRaf56	4565.A0A3B6ILF0	4565.A0A3B6LPY3	0	0	0.214	0.591	0	0.172	0.176	0	0.416
TaMAPKK6	TaRaf91	4565.A0A3B6ILF0	4565.A0A3B6PMI5	0	0	0.147	0.596	0	0.172	0.489	0	0.607
TaMAPKK6	TaMEKK4	4565.A0A3B6ILF0	4565.A0A3B6NRN9	0	0	0.163	0.71	0.05	0.652	0.573	0.136	0.879
TaMAPKK6	TaMEKK4-1	4565.A0A3B6ILF0	4565.A0A3B6PNI6	0	0	0.163	0.711	0.05	0.652	0.573	0.136	0.879
TaMAPKK6	TaMEKK24	4565.A0A3B6ILF0	4565.A0A3B6LLV5	0	0	0.162	0.733	0.05	0.652	0.614	0.136	0.89
TaMAPKK6	TaMEKK29	4565.A0A3B6ILF0	4565.A0A3B6MSP6	0	0	0.162	0.733	0.05	0.652	0.614	0.136	0.89
TaMAPKK6	TaMEKK17	4565.A0A3B6ILF0	4565.A0A3B6KFL8	0	0	0.161	0.734	0.05	0.652	0.614	0.136	0.89

TaMAPKK7	TaMEKK7	4565.A0A3B6INV0	4565.A0A077RUI2	0	0	0.18	0.699	0.05	0.652	0.431	0.136	0.842
TaMAPKK7	TaRaf18	4565.A0A3B6INV0	4565.A0A077RY41	0	0	0.202	0.628	0	0.172	0.176	0	0.408
TaMAPKK7	TaMEKK9	4565.A0A3B6INV0	4565.A0A077S2G5	0	0	0.195	0.648	0.05	0.652	0.431	0.136	0.845
TaMAPKK7	TaRaf95	4565.A0A3B6INV0	4565.A0A1D5UN01	0	0	0.191	0.6	0	0.172	0.176	0	0.4
TaMAPKK7	TaRaf88	4565.A0A3B6INV0	4565.A0A3B5Z5X1	0	0	0.206	0.624	0	0.172	0.176	0	0.411
TaMAPKK7	TaRaf30	4565.A0A3B6INV0	4565.A0A3B6A1Z4	0	0	0.204	0.624	0	0.172	0.176	0	0.409
TaMAPKK7	TaMAPKKK4	4565.A0A3B6INV0	4565.A0A3B6AY64	0	0	0.11	0.689	0	0.176	0.579	0.195	0.718
TaMAPKK7	TaMEKK1	4565.A0A3B6INV0	4565.A0A3B6B3I4	0	0	0.148	0.741	0.05	0.652	0.573	0.136	0.877
TaMAPKK7	TaMEKK20	4565.A0A3B6INV0	4565.A0A3B6B6T5	0	0	0	0.693	0.05	0.652	0.431	0.136	0.815
TaMAPKK7	TaMAPKKK5	4565.A0A3B6INV0	4565.A0A3B6C4T6	0	0	0.11	0.688	0	0.176	0.579	0.195	0.718
TaMAPKK7	TaMEKK18	4565.A0A3B6INV0	4565.A0A3B6CEA6	0	0	0	0.692	0.05	0.652	0.431	0.136	0.815
TaMAPKK7	TaMAPKKK6	4565.A0A3B6INV0	4565.A0A3B6DEP0	0	0	0.111	0.69	0	0.176	0.579	0.195	0.718
TaMAPKK7	TaRaf59	4565.A0A3B6INV0	4565.A0A3B6E9E2	0	0	0.191	0.608	0	0.172	0.176	0	0.4
TaMAPKK7	TaRaf60	4565.A0A3B6INV0	4565.A0A3B6EHW0	0	0	0.211	0.588	0	0.172	0.176	0	0.415
TaMAPKK7	TaMEKK8	4565.A0A3B6INV0	4565.A0A3B6FNU8	0	0	0.177	0.698	0.05	0.652	0.431	0.136	0.842
TaMAPKK7	TaRaf5	4565.A0A3B6INV0	4565.A0A3B6GYQ1	0	0	0.212	0.591	0	0.172	0.176	0	0.415
TaMAPKK7	TaRaf21	4565.A0A3B6INV0	4565.A0A3B6TVC5	0	0	0.194	0.593	0	0.172	0.176	0	0.402
TaMAPKK7	TaRaf1	4565.A0A3B6INV0	4565.A0A3B6NP57	0	0	0.197	0.589	0	0.172	0.176	0	0.404

TaMAPKK7	TaRaf62	4565.A0A3B6INV0	4565.A9RAA9	0	0	0.206	0.616	0	0.172	0.176	0	0.411
TaMAPKK7	TaRaf7	4565.A0A3B6INV0	4565.A0A3B6TBH5	0	0	0.209	0.599	0	0.172	0.176	0	0.413
TaMAPKK7	TaRaf41	4565.A0A3B6INV0	4565.A0A3B6KLD7	0	0	0.208	0.605	0	0.172	0.176	0	0.413
TaMAPKK7	TaRaf56	4565.A0A3B6INV0	4565.A0A3B6LPY3	0	0	0.214	0.591	0	0.172	0.176	0	0.417
TaMAPKK7	TaRaf91	4565.A0A3B6INV0	4565.A0A3B6PMI5	0	0	0.149	0.596	0	0.172	0.489	0	0.608
TaMAPKK7	TaMEKK4	4565.A0A3B6INV0	4565.A0A3B6NRN9	0	0	0.161	0.715	0.05	0.652	0.573	0.136	0.879
TaMAPKK7	TaMEKK4-1	4565.A0A3B6INV0	4565.A0A3B6PNI6	0	0	0.161	0.716	0.05	0.652	0.573	0.136	0.879
TaMAPKK7	TaMEKK17	4565.A0A3B6INV0	4565.A0A3B6KFL8	0	0	0.163	0.731	0.05	0.652	0.614	0.136	0.89
TaMAPKK7	TaMEKK29	4565.A0A3B6INV0	4565.A0A3B6MSP6	0	0	0.163	0.731	0.05	0.652	0.614	0.136	0.89
TaMAPKK7	TaMEKK24	4565.A0A3B6INV0	4565.A0A3B6LLV5	0	0	0.163	0.731	0.05	0.652	0.614	0.136	0.89
TaMAPKK8	TaMEKK7	4565.A0A3B6IK39	4565.A0A077RUI2	0	0	0.178	0.694	0.05	0.652	0.431	0.136	0.842
TaMAPKK8	TaRaf113	4565.A0A3B6IK39	4565.A0A077RV42	0	0	0.304	0	0	0.172	0.176	0	0.483
TaMAPKK8	TaMEKK9	4565.A0A3B6IK39	4565.A0A077S2G5	0	0	0.196	0.642	0.05	0.652	0.431	0.136	0.845
TaMAPKK8	TaRaf88	4565.A0A3B6IK39	4565.A0A3B5Z5X1	0	0	0.193	0.617	0	0.172	0.176	0	0.401
TaMAPKK8	TaRaf30	4565.A0A3B6IK39	4565.A0A3B6A1Z4	0	0	0.193	0.617	0	0.172	0.176	0	0.401
TaMAPKK8	TaRaf102	4565.A0A3B6IK39	4565.A0A3B6AQT7	0	0	0.224	0	0	0.172	0.176	0	0.424
TaMAPKK8	TaRaf19	4565.A0A3B6IK39	4565.A0A3B6AWY0	0	0	0.265	0	0	0.172	0.176	0	0.454
TaMAPKK8	TaMAPKKKK4	4565.A0A3B6IK39	4565.A0A3B6AY64	0	0	0.109	0.691	0	0.176	0.579	0.195	0.717

TaMAPKK8	TaMEKK1	4565.A0A3B6IK39	4565.A0A3B6B3I4	0	0	0.151	0.735	0.05	0.652	0.573	0.136	0.877
TaMAPKK8	TaMEKK20	4565.A0A3B6IK39	4565.A0A3B6B6T5	0	0	0	0.705	0.05	0.652	0.431	0.136	0.815
TaMAPKK8	TaRaf105	4565.A0A3B6IK39	4565.A0A3B6C472	0	0	0.251	0	0	0.172	0.176	0	0.444
TaMAPKK8	TaMAPKKK5	4565.A0A3B6IK39	4565.A0A3B6C4T6	0	0	0.108	0.69	0	0.176	0.579	0.195	0.717
TaMAPKK8	TaMEKK18	4565.A0A3B6IK39	4565.A0A3B6CEA6	0	0	0	0.706	0.05	0.652	0.431	0.136	0.815
TaMAPKK8	TaMAPKKK6	4565.A0A3B6IK39	4565.A0A3B6DEP0	0	0	0.109	0.691	0	0.176	0.579	0.195	0.718
TaMAPKK8	TaRaf71	4565.A0A3B6IK39	4565.A0A3B6EJJ5	0	0	0.202	0	0	0.172	0.176	0	0.408
TaMAPKK8	TaMEKK8	4565.A0A3B6IK39	4565.A0A3B6FNU8	0	0	0.174	0.694	0.05	0.652	0.431	0.136	0.841
TaMAPKK8	TaRaf46	4565.A0A3B6IK39	4565.A0A3B6MW69	0	0	0.192	0.623	0	0.172	0.176	0	0.4
TaMAPKK8	TaRaf62	4565.A0A3B6IK39	4565.A9RAA9	0	0	0.2	0.597	0	0.172	0.176	0	0.406
TaMAPKK8	TaRaf56	4565.A0A3B6IK39	4565.A0A3B6LPY3	0	0	0.211	0.586	0	0.172	0.176	0	0.415
TaMAPKK8	TaRaf41	4565.A0A3B6IK39	4565.A0A3B6KLD7	0	0	0.212	0.591	0	0.172	0.176	0	0.415
TaMAPKK8	TaRaf42	4565.A0A3B6IK39	4565.A0A3B6U554	0	0	0.213	0	0	0.172	0.176	0	0.416
TaMAPKK8	TaRaf7	4565.A0A3B6IK39	4565.A0A3B6TBH5	0	0	0.216	0.59	0	0.172	0.176	0	0.419
TaMAPKK8	TaRaf45	4565.A0A3B6IK39	4565.A0A3B6KN82	0	0	0.224	0	0	0.172	0.176	0	0.424
TaMAPKK8	TaRaf50	4565.A0A3B6IK39	4565.A0A3B6RET0	0	0	0.27	0	0	0.172	0.176	0	0.458
TaMAPKK8	TaRaf43	4565.A0A3B6IK39	4565.A0A3B6KNC6	0	0	0.281	0	0	0.172	0.176	0	0.466
TaMAPKK8	TaRaf91	4565.A0A3B6IK39	4565.A0A3B6PMI5	0	0	0.125	0.606	0	0.172	0.489	0	0.597

TaMAPKK8	TaMEKK4-1	4565.A0A3B6IK39	4565.A0A3B6PNI6	0	0	0.164	0.709	0.05	0.652	0.573	0.136	0.879
TaMAPKK8	TaMEKK4	4565.A0A3B6IK39	4565.A0A3B6NRN9	0	0	0.165	0.706	0.05	0.652	0.573	0.136	0.879
TaMAPKK8	TaMEKK17	4565.A0A3B6IK39	4565.A0A3B6KFL8	0	0	0.156	0.743	0.05	0.652	0.614	0.136	0.889
TaMAPKK8	TaMEKK29	4565.A0A3B6IK39	4565.A0A3B6MSP6	0	0	0.155	0.744	0.05	0.652	0.614	0.136	0.889
TaMAPKK8	TaMEKK24	4565.A0A3B6IK39	4565.A0A3B6LLV5	0	0	0.156	0.743	0.05	0.652	0.614	0.136	0.89
TaMAPKK9	TaMEKK7	4565.A0A077RVQ4	4565.A0A077RUI2	0	0	0.176	0.704	0.05	0.652	0.431	0.136	0.841
TaMAPKK9	TaRaf46	4565.A0A077RVQ4	4565.A0A3B6MW69	0	0	0.195	0.622	0	0.172	0.176	0	0.403
TaMAPKK9	TaRaf95	4565.A0A077RVQ4	4565.A0A1D5UN01	0	0	0.197	0.602	0	0.172	0.176	0	0.404
TaMAPKK9	TaRaf30	4565.A0A077RVQ4	4565.A0A3B6A1Z4	0	0	0.198	0.619	0	0.172	0.176	0	0.405
TaMAPKK9	TaRaf18	4565.A0A077RVQ4	4565.A0A077RY41	0	0	0.199	0.622	0	0.172	0.176	0	0.406
TaMAPKK9	TaRaf88	4565.A0A077RVQ4	4565.A0A3B5Z5X1	0	0	0.2	0.618	0	0.172	0.176	0	0.407
TaMAPKK9	TaRaf5	4565.A0A077RVQ4	4565.A0A3B6GYQ1	0	0	0.202	0.592	0	0.172	0.176	0	0.408
TaMAPKK9	TaRaf60	4565.A0A077RVQ4	4565.A0A3B6EHW0	0	0	0.205	0.584	0	0.172	0.176	0	0.41
TaMAPKK9	TaRaf62	4565.A0A077RVQ4	4565.A9RAA9	0	0	0.204	0.608	0	0.172	0.176	0	0.41
TaMAPKK9	TaRaf42	4565.A0A077RVQ4	4565.A0A3B6U554	0	0	0.215	0	0	0.172	0.176	0	0.417
TaMAPKK9	TaRaf56	4565.A0A077RVQ4	4565.A0A3B6LPY3	0	0	0.216	0.585	0	0.172	0.176	0	0.418
TaMAPKK9	TaRaf41	4565.A0A077RVQ4	4565.A0A3B6KLD7	0	0	0.215	0.591	0	0.172	0.176	0	0.418
TaMAPKK9	TaRaf7	4565.A0A077RVQ4	4565.A0A3B6TBH5	0	0	0.218	0.594	0	0.172	0.176	0	0.419

TaMAPKK9	TaRaf102	4565.A0A077RVQ4	4565.A0A3B6AQT7	0	0	0.221	0	0	0.172	0.176	0	0.422
TaMAPKK9	TaRaf50	4565.A0A077RVQ4	4565.A0A3B6RET0	0	0	0.274	0	0	0.172	0.176	0	0.461
TaMAPKK9	TaRaf43	4565.A0A077RVQ4	4565.A0A3B6KNC6	0	0	0.285	0	0	0.172	0.176	0	0.469
TaMAPKK9	TaRaf91	4565.A0A077RVQ4	4565.A0A3B6PMI5	0	0	0.133	0.595	0	0.172	0.489	0	0.601
TaMAPKK9	TaMAPKKK4	4565.A0A077RVQ4	4565.A0A3B6AY64	0	0	0.11	0.689	0	0.176	0.579	0.195	0.718
TaMAPKK9	TaMAPKKK5	4565.A0A077RVQ4	4565.A0A3B6C4T6	0	0	0.11	0.689	0	0.176	0.579	0.195	0.718
TaMAPKK9	TaMAPKKK6	4565.A0A077RVQ4	4565.A0A3B6DEP0	0	0	0.111	0.689	0	0.176	0.579	0.195	0.718
TaMAPKK9	TaMEKK18	4565.A0A077RVQ4	4565.A0A3B6CEA6	0	0	0	0.702	0.05	0.652	0.431	0.136	0.815
TaMAPKK9	TaMEKK20	4565.A0A077RVQ4	4565.A0A3B6B6T5	0	0	0	0.702	0.05	0.652	0.431	0.136	0.815
TaMAPKK9	TaMEKK8	4565.A0A077RVQ4	4565.A0A3B6FNU8	0	0	0.174	0.699	0.05	0.652	0.431	0.136	0.841
TaMAPKK9	TaMEKK9	4565.A0A077RVQ4	4565.A0A077S2G5	0	0	0.19	0.658	0.05	0.652	0.431	0.136	0.844
TaMAPKK9	TaMEKK1	4565.A0A077RVQ4	4565.A0A3B6B3I4	0	0	0.156	0.729	0.05	0.652	0.573	0.136	0.878
TaMAPKK9	TaMEKK4	4565.A0A077RVQ4	4565.A0A3B6NRN9	0	0	0.169	0.703	0.05	0.652	0.573	0.136	0.88
TaMAPKK9	TaMEKK4-1	4565.A0A077RVQ4	4565.A0A3B6PNI6	0	0	0.168	0.705	0.05	0.652	0.573	0.136	0.88
TaMAPKK9	TaMEKK29	4565.A0A077RVQ4	4565.A0A3B6MSP6	0	0	0.159	0.738	0.05	0.652	0.614	0.136	0.89
TaMAPKK9	TaMEKK24	4565.A0A077RVQ4	4565.A0A3B6LLV5	0	0	0.159	0.738	0.05	0.652	0.614	0.136	0.89
TaMAPKK9	TaMEKK17	4565.A0A077RVQ4	4565.A0A3B6KFL8	0	0	0.159	0.739	0.05	0.652	0.614	0.136	0.89
TaMAPKKK1	TaMAPKKK16	4565.A0A3B5XZB3	4565.A0A3B6NTU1	0	0	0.058	0.964	0	0.231	0.43	0.043	0.552

TaMAPKKKK1	TaZIK1	4565.A0A3B5XZB3	4565.Q84XZ4	0	0	0.116	0.568	0	0.699	0	0.151	0.754
TaMAPKKKK1	TaZIK11	4565.A0A3B5XZB3	4565.A0A3B6LI77	0	0	0.117	0.568	0	0.699	0	0.151	0.754
TaMAPKKKK1	TaZIK8	4565.A0A3B5XZB3	4565.A0A3B6C5U6	0	0	0.126	0.616	0	0.699	0	0.151	0.757
TaMAPKKKK1	TaZIK5	4565.A0A3B5XZB3	4565.A0A3B6DA61	0	0	0.13	0.619	0	0.699	0	0.151	0.758
TaMAPKKKK1	TaZIK10	4565.A0A3B5XZB3	4565.A0A3B6NT46	0	0	0.134	0.57	0	0.699	0	0.151	0.759
TaMAPKKKK1	TaZIK2	4565.A0A3B5XZB3	4565.A0A3B6QG64	0	0	0.135	0.57	0	0.699	0	0.151	0.759
TaMAPKKKK1	TaZIK9	4565.A0A3B5XZB3	4565.A0A3B5ZP32	0	0	0.137	0.569	0	0.699	0	0.151	0.76
TaMAPKKKK1	TaZIK7	4565.A0A3B5XZB3	4565.A0A3B6C620	0	0	0.138	0.564	0	0.699	0	0.151	0.76
TaMAPKKKK1	TaZIK4	4565.A0A3B5XZB3	4565.A0A3B6PML2	0	0	0.136	0.572	0	0.699	0	0.151	0.76
TaMAPKKKK1	TaZIK3	4565.A0A3B5XZB3	4565.A0A3B6AUS6	0	0	0.139	0.564	0	0.699	0	0.151	0.761
TaMAPKKKK10	TaMEKK7	4565.A0A3B6KPR0	4565.A0A077RUI2	0	0	0.141	0.625	0.06	0.144	0.548	0	0.646
TaMAPKKKK10	TaRaf18	4565.A0A3B6KPR0	4565.A0A077RY41	0	0	0.11	0.61	0	0.122	0.493	0	0.569
TaMAPKKKK10	TaMEKK9	4565.A0A3B6KPR0	4565.A0A077S2G5	0	0	0.128	0.644	0.06	0.144	0.548	0	0.64
TaMAPKKKK10	TaRaf29	4565.A0A3B6KPR0	4565.A0A1D5UHD7	0	0	0.152	0.587	0	0.122	0.493	0	0.589
TaMAPKKKK10	TaRaf88	4565.A0A3B6KPR0	4565.A0A3B5Z5X1	0	0	0.114	0.616	0	0.122	0.493	0	0.571
TaMAPKKKK10	TaRaf30	4565.A0A3B6KPR0	4565.A0A3B6A1Z4	0	0	0.115	0.614	0	0.122	0.493	0	0.571
TaMAPKKKK10	TaMEKK1	4565.A0A3B6KPR0	4565.A0A3B6B3I4	0	0	0.121	0.617	0.06	0.144	0.548	0	0.637
TaMAPKKKK10	TaMEKK20	4565.A0A3B6KPR0	4565.A0A3B6B6T5	0	0	0.117	0.626	0.06	0.144	0.548	0	0.636

TaMAPKKKK10	TaRaf79	4565.A0A3B6KPR0	4565.A0A3B6B9C7	0	0	0.148	0.593	0	0.122	0.493	0	0.588
TaMAPKKKK10	TaMEKK18	4565.A0A3B6KPR0	4565.A0A3B6CEA6	0	0	0.117	0.627	0.06	0.144	0.548	0	0.636
TaMAPKKKK10	TaRaf59	4565.A0A3B6KPR0	4565.A0A3B6E9E2	0	0	0	0.638	0	0.122	0.493	0	0.535
TaMAPKKKK10	TaRaf60	4565.A0A3B6KPR0	4565.A0A3B6EHW0	0	0	0.142	0.572	0	0.122	0.493	0	0.584
TaMAPKKKK10	TaMEKK8	4565.A0A3B6KPR0	4565.A0A3B6FNU8	0	0	0.129	0.63	0.06	0.144	0.548	0	0.641
TaMAPKKKK10	TaRaf5	4565.A0A3B6KPR0	4565.A0A3B6GYQ1	0	0	0.146	0.576	0	0.122	0.493	0	0.586
TaMAPKKKK10	TaMEKK17	4565.A0A3B6KPR0	4565.A0A3B6KFL8	0	0	0.137	0.643	0.06	0.144	0.548	0	0.644
TaMAPKKKK10	TaRaf100	4565.A0A3B6KPR0	4565.A0A3B6ML06	0	0	0	0.58	0	0.122	0.493	0	0.535
TaMAPKKKK10	TaRaf52	4565.A0A3B6KPR0	4565.A0A3B6LRR0	0	0	0	0.594	0	0.122	0.493	0	0.535
TaMAPKKKK10	TaRaf1	4565.A0A3B6KPR0	4565.A0A3B6NP57	0	0	0.117	0.655	0	0.122	0.493	0	0.572
TaMAPKKKK10	TaRaf58	4565.A0A3B6KPR0	4565.A0A3B6N1Y2	0	0	0.118	0.602	0	0.122	0.493	0	0.573
TaMAPKKKK10	TaRaf46	4565.A0A3B6KPR0	4565.A0A3B6MW69	0	0	0.121	0.637	0	0.122	0.493	0	0.575
TaMAPKKKK10	TaRaf21	4565.A0A3B6KPR0	4565.A0A3B6TVC5	0	0	0.122	0.634	0	0.122	0.493	0	0.575
TaMAPKKKK10	TaRaf62	4565.A0A3B6KPR0	4565.A9RAA9	0	0	0.133	0.635	0	0.122	0.493	0	0.58
TaMAPKKKK10	TaRaf63	4565.A0A3B6KPR0	4565.A0A3B6RKW0	0	0	0.137	0.583	0	0.122	0.493	0	0.582
TaMAPKKKK10	TaRaf91	4565.A0A3B6KPR0	4565.A0A3B6PMI5	0	0	0.169	0.573	0	0.122	0.493	0	0.598
TaMAPKKKK10	TaMEKK4-1	4565.A0A3B6KPR0	4565.A0A3B6PNI6	0	0	0.114	0.617	0.06	0.144	0.548	0	0.635
TaMAPKKKK10	TaMEKK4	4565.A0A3B6KPR0	4565.A0A3B6NRN9	0	0	0.12	0.608	0.06	0.144	0.548	0	0.637

TaMAPKKKK10	TaMEKK29	4565.A0A3B6KPR0	4565.A0A3B6MSP6	0	0	0.137	0.643	0.06	0.144	0.548	0	0.644
TaMAPKKKK10	TaMEKK24	4565.A0A3B6KPR0	4565.A0A3B6LLV5	0	0	0.137	0.643	0.06	0.144	0.548	0	0.644
TaMAPKKKK11	TaZIK9	4565.A0A3B6KVE1	4565.A0A3B5ZP32	0	0	0.151	0.57	0	0.699	0	0.151	0.764
TaMAPKKKK11	TaZIK3	4565.A0A3B6KVE1	4565.A0A3B6AUS6	0	0	0.157	0.56	0	0.699	0	0.151	0.765
TaMAPKKKK11	TaZIK8	4565.A0A3B6KVE1	4565.A0A3B6C5U6	0	0	0.14	0.607	0	0.699	0	0.151	0.761
TaMAPKKKK11	TaZIK7	4565.A0A3B6KVE1	4565.A0A3B6C620	0	0	0.155	0.56	0	0.699	0	0.151	0.765
TaMAPKKKK11	TaZIK5	4565.A0A3B6KVE1	4565.A0A3B6DA61	0	0	0.143	0.61	0	0.699	0	0.151	0.762
TaMAPKKKK11	TaMAPKKKK16	4565.A0A3B6KVE1	4565.A0A3B6NTU1	0	0	0.06	0.96	0	0.231	0.43	0.043	0.553
TaMAPKKKK11	TaZIK11	4565.A0A3B6KVE1	4565.A0A3B6LI77	0	0	0.129	0.564	0	0.699	0	0.151	0.758
TaMAPKKKK11	TaZIK1	4565.A0A3B6KVE1	4565.Q84XZ4	0	0	0.13	0.563	0	0.699	0	0.151	0.758
TaMAPKKKK11	TaZIK2	4565.A0A3B6KVE1	4565.A0A3B6QG64	0	0	0.15	0.565	0	0.699	0	0.151	0.763
TaMAPKKKK11	TaZIK10	4565.A0A3B6KVE1	4565.A0A3B6NT46	0	0	0.15	0.564	0	0.699	0	0.151	0.763
TaMAPKKKK11	TaZIK4	4565.A0A3B6KVE1	4565.A0A3B6PML2	0	0	0.152	0.567	0	0.699	0	0.151	0.764
TaMAPKKKK12	TaMEKK7	4565.A0A3B6LTF3	4565.A0A077RUI2	0	0	0.14	0.625	0.06	0.144	0.548	0	0.645
TaMAPKKKK12	TaRaf18	4565.A0A3B6LTF3	4565.A0A077RY41	0	0	0.11	0.609	0	0.122	0.493	0	0.569
TaMAPKKKK12	TaMEKK9	4565.A0A3B6LTF3	4565.A0A077S2G5	0	0	0.127	0.645	0.06	0.144	0.548	0	0.64
TaMAPKKKK12	TaRaf29	4565.A0A3B6LTF3	4565.A0A1D5UHD7	0	0	0.151	0.589	0	0.122	0.493	0	0.589
TaMAPKKKK12	TaRaf88	4565.A0A3B6LTF3	4565.A0A3B5Z5X1	0	0	0.114	0.615	0	0.122	0.493	0	0.571

TaMAPKKKK12	TaRaf30	4565.A0A3B6LTF3	4565.A0A3B6A1Z4	0	0	0.115	0.613	0	0.122	0.493	0	0.571
TaMAPKKKK12	TaMEKK1	4565.A0A3B6LTF3	4565.A0A3B6B3I4	0	0	0.119	0.617	0.06	0.144	0.548	0	0.636
TaMAPKKKK12	TaMEKK20	4565.A0A3B6LTF3	4565.A0A3B6B6T5	0	0	0.118	0.62	0.06	0.144	0.548	0	0.636
TaMAPKKKK12	TaRaf79	4565.A0A3B6LTF3	4565.A0A3B6B9C7	0	0	0.147	0.595	0	0.122	0.493	0	0.587
TaMAPKKKK12	TaMEKK18	4565.A0A3B6LTF3	4565.A0A3B6CEA6	0	0	0.118	0.62	0.06	0.144	0.548	0	0.636
TaMAPKKKK12	TaRaf59	4565.A0A3B6LTF3	4565.A0A3B6E9E2	0	0	0	0.635	0	0.122	0.493	0	0.535
TaMAPKKKK12	TaRaf60	4565.A0A3B6LTF3	4565.A0A3B6EHW0	0	0	0.14	0.573	0	0.122	0.493	0	0.583
TaMAPKKKK12	TaMEKK8	4565.A0A3B6LTF3	4565.A0A3B6FNU8	0	0	0.128	0.629	0.06	0.144	0.548	0	0.64
TaMAPKKKK12	TaRaf5	4565.A0A3B6LTF3	4565.A0A3B6GYQ1	0	0	0.144	0.576	0	0.122	0.493	0	0.586
TaMAPKKKK12	TaMEKK17	4565.A0A3B6LTF3	4565.A0A3B6KFL8	0	0	0.136	0.644	0.06	0.144	0.548	0	0.643
TaMAPKKKK12	TaMEKK24	4565.A0A3B6LTF3	4565.A0A3B6LLV5	0	0	0.135	0.644	0.06	0.144	0.548	0	0.643
TaMAPKKKK12	TaRaf52	4565.A0A3B6LTF3	4565.A0A3B6LRR0	0	0	0	0.593	0	0.122	0.493	0	0.535
TaMAPKKKK12	TaRaf100	4565.A0A3B6LTF3	4565.A0A3B6ML06	0	0	0	0.578	0	0.122	0.493	0	0.535
TaMAPKKKK12	TaRaf1	4565.A0A3B6LTF3	4565.A0A3B6NP57	0	0	0.116	0.659	0	0.122	0.493	0	0.572
TaMAPKKKK12	TaRaf58	4565.A0A3B6LTF3	4565.A0A3B6N1Y2	0	0	0.118	0.603	0	0.122	0.493	0	0.573
TaMAPKKKK12	TaRaf21	4565.A0A3B6LTF3	4565.A0A3B6TVC5	0	0	0.121	0.637	0	0.122	0.493	0	0.574
TaMAPKKKK12	TaRaf46	4565.A0A3B6LTF3	4565.A0A3B6MW69	0	0	0.122	0.637	0	0.122	0.493	0	0.575
TaMAPKKKK12	TaRaf62	4565.A0A3B6LTF3	4565.A9RAA9	0	0	0.132	0.635	0	0.122	0.493	0	0.58

TaMAPKKKK12	TaRaf63	4565.A0A3B6LTF3	4565.A0A3B6RKW0	0	0	0.138	0.583	0	0.122	0.493	0	0.582
TaMAPKKKK12	TaRaf91	4565.A0A3B6LTF3	4565.A0A3B6PMI5	0	0	0.168	0.571	0	0.122	0.493	0	0.597
TaMAPKKKK12	TaMEKK4-1	4565.A0A3B6LTF3	4565.A0A3B6PNI6	0	0	0.113	0.617	0.06	0.144	0.548	0	0.634
TaMAPKKKK12	TaMEKK4	4565.A0A3B6LTF3	4565.A0A3B6NRN9	0	0	0.119	0.603	0.06	0.144	0.548	0	0.636
TaMAPKKKK12	TaMEKK29	4565.A0A3B6LTF3	4565.A0A3B6MSP6	0	0	0.136	0.644	0.06	0.144	0.548	0	0.643
TaMAPKKKK13	TaZIK9	4565.A0A3B6MRC5	4565.A0A3B5ZP32	0	0	0.109	0.572	0	0.699	0	0.151	0.752
TaMAPKKKK13	TaZIK3	4565.A0A3B6MRC5	4565.A0A3B6AUS6	0	0	0.118	0.571	0	0.699	0	0.151	0.754
TaMAPKKKK13	TaZIK8	4565.A0A3B6MRC5	4565.A0A3B6C5U6	0	0	0.111	0.596	0	0.699	0	0.151	0.753
TaMAPKKKK13	TaZIK7	4565.A0A3B6MRC5	4565.A0A3B6C620	0	0	0.117	0.571	0	0.699	0	0.151	0.754
TaMAPKKKK13	TaZIK5	4565.A0A3B6MRC5	4565.A0A3B6DA61	0	0	0.115	0.598	0	0.699	0	0.151	0.754
TaMAPKKKK13	TaZIK11	4565.A0A3B6MRC5	4565.A0A3B6LI77	0	0	0	0.565	0	0.699	0	0.151	0.733
TaMAPKKKK13	TaMAPKKKK16	4565.A0A3B6MRC5	4565.A0A3B6NTU1	0	0	0.079	0.916	0	0.231	0.43	0.043	0.562
TaMAPKKKK13	TaZIK1	4565.A0A3B6MRC5	4565.Q84XZ4	0	0	0	0.562	0	0.699	0	0.151	0.733
TaMAPKKKK13	TaZIK2	4565.A0A3B6MRC5	4565.A0A3B6QG64	0	0	0.109	0.57	0	0.699	0	0.151	0.752
TaMAPKKKK13	TaZIK4	4565.A0A3B6MRC5	4565.A0A3B6PML2	0	0	0.109	0.569	0	0.699	0	0.151	0.752
TaMAPKKKK13	TaZIK10	4565.A0A3B6MRC5	4565.A0A3B6NT46	0	0	0.109	0.569	0	0.699	0	0.151	0.752
TaMAPKKKK14	TaMEKK7	4565.A0A3B6MZG0	4565.A0A077RUI2	0	0	0.141	0.624	0.06	0.144	0.548	0	0.645
TaMAPKKKK14	TaRaf18	4565.A0A3B6MZG0	4565.A0A077RY41	0	0	0.11	0.609	0	0.122	0.493	0	0.569

TaMAPKKKK14	TaMEKK9	4565.A0A3B6MZG0	4565.A0A077S2G5	0	0	0.127	0.645	0.06	0.144	0.548	0	0.64
TaMAPKKKK14	TaRaf29	4565.A0A3B6MZG0	4565.A0A1D5UHD7	0	0	0.151	0.589	0	0.122	0.493	0	0.589
TaMAPKKKK14	TaRaf88	4565.A0A3B6MZG0	4565.A0A3B5Z5X1	0	0	0.114	0.615	0	0.122	0.493	0	0.571
TaMAPKKKK14	TaRaf30	4565.A0A3B6MZG0	4565.A0A3B6A1Z4	0	0	0.115	0.613	0	0.122	0.493	0	0.571
TaMAPKKKK14	TaMEKK1	4565.A0A3B6MZG0	4565.A0A3B6B3I4	0	0	0.119	0.617	0.06	0.144	0.548	0	0.637
TaMAPKKKK14	TaMEKK20	4565.A0A3B6MZG0	4565.A0A3B6B6T5	0	0	0.117	0.626	0.06	0.144	0.548	0	0.636
TaMAPKKKK14	TaRaf79	4565.A0A3B6MZG0	4565.A0A3B6B9C7	0	0	0.147	0.595	0	0.122	0.493	0	0.587
TaMAPKKKK14	TaMEKK18	4565.A0A3B6MZG0	4565.A0A3B6CEA6	0	0	0.117	0.627	0.06	0.144	0.548	0	0.636
TaMAPKKKK14	TaRaf59	4565.A0A3B6MZG0	4565.A0A3B6E9E2	0	0	0	0.635	0	0.122	0.493	0	0.535
TaMAPKKKK14	TaRaf60	4565.A0A3B6MZG0	4565.A0A3B6EHW0	0	0	0.139	0.574	0	0.122	0.493	0	0.583
TaMAPKKKK14	TaMEKK8	4565.A0A3B6MZG0	4565.A0A3B6FNU8	0	0	0.129	0.629	0.06	0.144	0.548	0	0.64
TaMAPKKKK14	TaRaf5	4565.A0A3B6MZG0	4565.A0A3B6GYQ1	0	0	0.144	0.576	0	0.122	0.493	0	0.585
TaMAPKKKK14	TaMEKK17	4565.A0A3B6MZG0	4565.A0A3B6KFL8	0	0	0.136	0.644	0.06	0.144	0.548	0	0.643
TaMAPKKKK14	TaMEKK24	4565.A0A3B6MZG0	4565.A0A3B6LLV5	0	0	0.135	0.644	0.06	0.144	0.548	0	0.643
TaMAPKKKK14	TaRaf52	4565.A0A3B6MZG0	4565.A0A3B6LRR0	0	0	0	0.593	0	0.122	0.493	0	0.535
TaMAPKKKK14	TaRaf100	4565.A0A3B6MZG0	4565.A0A3B6ML06	0	0	0	0.579	0	0.122	0.493	0	0.535
TaMAPKKKK14	TaMEKK29	4565.A0A3B6MZG0	4565.A0A3B6MSP6	0	0	0.136	0.644	0.06	0.144	0.548	0	0.643
TaMAPKKKK14	TaRaf46	4565.A0A3B6MZG0	4565.A0A3B6MW69	0	0	0.122	0.637	0	0.122	0.493	0	0.575

TaMAPKKKK14	TaRaf1	4565.A0A3B6MZG0	4565.A0A3B6NP57	0	0	0.116	0.659	0	0.122	0.493	0	0.572
TaMAPKKKK14	TaRaf58	4565.A0A3B6MZG0	4565.A0A3B6N1Y2	0	0	0.118	0.601	0	0.122	0.493	0	0.573
TaMAPKKKK14	TaRaf21	4565.A0A3B6MZG0	4565.A0A3B6TVC5	0	0	0.121	0.637	0	0.122	0.493	0	0.574
TaMAPKKKK14	TaRaf62	4565.A0A3B6MZG0	4565.A9RAA9	0	0	0.132	0.635	0	0.122	0.493	0	0.58
TaMAPKKKK14	TaRaf63	4565.A0A3B6MZG0	4565.A0A3B6RKW0	0	0	0.137	0.583	0	0.122	0.493	0	0.582
TaMAPKKKK14	TaRaf91	4565.A0A3B6MZG0	4565.A0A3B6PMI5	0	0	0.167	0.574	0	0.122	0.493	0	0.597
TaMAPKKKK14	TaMEKK4-1	4565.A0A3B6MZG0	4565.A0A3B6PNI6	0	0	0.113	0.617	0.06	0.144	0.548	0	0.634
TaMAPKKKK14	TaMEKK4	4565.A0A3B6MZG0	4565.A0A3B6NRN9	0	0	0.119	0.608	0.06	0.144	0.548	0	0.636
TaMAPKKKK15	TaZIK9	4565.A0A3B6NNC4	4565.A0A3B5ZP32	0	0	0.157	0.574	0	0.699	0	0.151	0.765
TaMAPKKKK15	TaZIK3	4565.A0A3B6NNC4	4565.A0A3B6AUS6	0	0	0.16	0.573	0	0.699	0	0.151	0.766
TaMAPKKKK15	TaZIK8	4565.A0A3B6NNC4	4565.A0A3B6C5U6	0	0	0.15	0.63	0	0.699	0	0.151	0.763
TaMAPKKKK15	TaZIK7	4565.A0A3B6NNC4	4565.A0A3B6C620	0	0	0.159	0.573	0	0.699	0	0.151	0.766
TaMAPKKKK15	TaZIK5	4565.A0A3B6NNC4	4565.A0A3B6DA61	0	0	0.155	0.634	0	0.699	0	0.151	0.765
TaMAPKKKK15	TaZIK11	4565.A0A3B6NNC4	4565.A0A3B6LI77	0	0	0.135	0.582	0	0.699	0	0.151	0.759
TaMAPKKKK15	TaMAPKKKK16	4565.A0A3B6NNC4	4565.A0A3B6NTU1	0	0	0.064	0.952	0	0.231	0.43	0.043	0.555
TaMAPKKKK15	TaZIK1	4565.A0A3B6NNC4	4565.Q84XZ4	0	0	0.135	0.581	0	0.699	0	0.151	0.759
TaMAPKKKK15	TaZIK10	4565.A0A3B6NNC4	4565.A0A3B6NT46	0	0	0.16	0.572	0	0.699	0	0.151	0.766
TaMAPKKKK15	TaZIK2	4565.A0A3B6NNC4	4565.A0A3B6QG64	0	0	0.16	0.573	0	0.699	0	0.151	0.766

TaMAPKKKK15	TaZIK4	4565.A0A3B6NNC4	4565.A0A3B6PML2	0	0	0.161	0.577	0	0.699	0	0.151	0.767
TaMAPKKKK16	TaMAPKKKK2	4565.A0A3B6NTU1	4565.A0A3B5YXW4	0	0	0.058	0.964	0	0.231	0.43	0.043	0.552
TaMAPKKKK16	TaMAPKKKK3	4565.A0A3B6NTU1	4565.A0A3B5ZTC4	0	0	0.059	0.963	0	0.231	0.43	0.043	0.552
TaMAPKKKK16	TaMEKK12	4565.A0A3B6NTU1	4565.A0A3B6HQC5	0	0	0.108	0.676	0	0.165	0.603	0.098	0.697
TaMAPKKKK16	TaMEKK3	4565.A0A3B6NTU1	4565.A0A3B6ISF1	0	0	0.109	0.671	0	0.165	0.603	0.098	0.698
TaMAPKKKK16	TaMAPKKKK7	4565.A0A3B6NTU1	4565.A0A3B6IX47	0	0	0.074	0.929	0	0.231	0.43	0.043	0.559
TaMAPKKKK16	TaMAPKKKK8	4565.A0A3B6NTU1	4565.A0A3B6J0T1	0	0	0.059	0.962	0	0.231	0.43	0.043	0.552
TaMAPKKKK16	TaMEKK10	4565.A0A3B6NTU1	4565.A0A3B6JM32	0	0	0.109	0.671	0	0.165	0.603	0.098	0.698
TaMAPKKKK16	TaMAPKKKK9	4565.A0A3B6NTU1	4565.A0A3B6KID5	0	0	0.079	0.916	0	0.231	0.43	0.043	0.562
TaMAPKKKK16	TaMEKK25	4565.A0A3B6NTU1	4565.A0A3B6LLP7	0	0	0.079	0.917	0	0.231	0.43	0.043	0.562
TaMAPKKKK16	TaMAPKKKK20	4565.A0A3B6NTU1	4565.A0A3B6QMW0	0	0	0.047	0.987	0	0.231	0.43	0.043	0.546
TaMAPKKKK16	TaMAPKKKK19	4565.A0A3B6NTU1	4565.A0A3B6PS74	0	0	0.047	0.987	0	0.231	0.43	0.043	0.546
TaMAPKKKK16	TaMAPKKKK17	4565.A0A3B6NTU1	4565.A0A3B6NVG6	0	0	0.048	0.985	0	0.231	0.43	0.043	0.547
TaMAPKKKK16	TaMAPKKKK23	4565.A0A3B6NTU1	4565.A0A3B6SHY6	0	0	0.055	0.97	0	0.231	0.43	0.043	0.55
TaMAPKKKK16	TaMAPKKKK24	4565.A0A3B6NTU1	4565.A0A3B6TDA8	0	0	0.055	0.97	0	0.231	0.43	0.043	0.55
TaMAPKKKK16	TaMAPKKKK22	4565.A0A3B6NTU1	4565.A0A3B6RD82	0	0	0.055	0.97	0	0.231	0.43	0.043	0.55
TaMAPKKKK16	TaMAPKKKK25	4565.A0A3B6NTU1	4565.A0A3B6UB28	0	0	0.059	0.963	0	0.231	0.43	0.043	0.552
TaMAPKKKK16	TaMAPKKKK18	4565.A0A3B6NTU1	4565.A0A3B6PL54	0	0	0.064	0.953	0	0.231	0.43	0.043	0.554

TaMAPKKKK16	TaMAPKKKK21	4565.A0A3B6NTU1	4565.A0A3B6QCF0	0	0	0.067	0.947	0	0.231	0.43	0.043	0.556
TaMAPKKKK17	TaZIK9	4565.A0A3B6NVG6	4565.A0A3B5ZP32	0	0	0.147	0.564	0	0.699	0	0.151	0.763
TaMAPKKKK17	TaZIK3	4565.A0A3B6NVG6	4565.A0A3B6AUS6	0	0	0.143	0.564	0	0.699	0	0.151	0.762
TaMAPKKKK17	TaZIK8	4565.A0A3B6NVG6	4565.A0A3B6C5U6	0	0	0.126	0.602	0	0.699	0	0.151	0.757
TaMAPKKKK17	TaZIK7	4565.A0A3B6NVG6	4565.A0A3B6C620	0	0	0.141	0.564	0	0.699	0	0.151	0.761
TaMAPKKKK17	TaZIK5	4565.A0A3B6NVG6	4565.A0A3B6DA61	0	0	0.13	0.605	0	0.699	0	0.151	0.758
TaMAPKKKK17	TaZIK11	4565.A0A3B6NVG6	4565.A0A3B6LI77	0	0	0.119	0.562	0	0.699	0	0.151	0.755
TaMAPKKKK17	TaZIK10	4565.A0A3B6NVG6	4565.A0A3B6NT46	0	0	0.141	0.564	0	0.699	0	0.151	0.761
TaMAPKKKK17	TaZIK1	4565.A0A3B6NVG6	4565.Q84XZ4	0	0	0.118	0.563	0	0.699	0	0.151	0.755
TaMAPKKKK17	TaZIK2	4565.A0A3B6NVG6	4565.A0A3B6QG64	0	0	0.141	0.565	0	0.699	0	0.151	0.761
TaMAPKKKK17	TaZIK4	4565.A0A3B6NVG6	4565.A0A3B6PML2	0	0	0.142	0.566	0	0.699	0	0.151	0.761
TaMAPKKKK18	TaZIK9	4565.A0A3B6PL54	4565.A0A3B5ZP32	0	0	0.156	0.574	0	0.699	0	0.151	0.765
TaMAPKKKK18	TaZIK3	4565.A0A3B6PL54	4565.A0A3B6AUS6	0	0	0.158	0.573	0	0.699	0	0.151	0.766
TaMAPKKKK18	TaZIK8	4565.A0A3B6PL54	4565.A0A3B6C5U6	0	0	0.149	0.63	0	0.699	0	0.151	0.763
TaMAPKKKK18	TaZIK7	4565.A0A3B6PL54	4565.A0A3B6C620	0	0	0.157	0.573	0	0.699	0	0.151	0.765
TaMAPKKKK18	TaZIK5	4565.A0A3B6PL54	4565.A0A3B6DA61	0	0	0.154	0.634	0	0.699	0	0.151	0.765
TaMAPKKKK18	TaZIK11	4565.A0A3B6PL54	4565.A0A3B6LI77	0	0	0.133	0.582	0	0.699	0	0.151	0.759
TaMAPKKKK18	TaZIK10	4565.A0A3B6PL54	4565.A0A3B6NT46	0	0	0.159	0.572	0	0.699	0	0.151	0.766

TaMAPKKKK18	TaZIK1	4565.A0A3B6PL54	4565.Q84XZ4	0	0	0.133	0.581	0	0.699	0	0.151	0.759
TaMAPKKKK18	TaZIK2	4565.A0A3B6PL54	4565.A0A3B6QG64	0	0	0.159	0.573	0	0.699	0	0.151	0.766
TaMAPKKKK18	TaZIK4	4565.A0A3B6PL54	4565.A0A3B6PML2	0	0	0.16	0.577	0	0.699	0	0.151	0.766
TaMAPKKKK19	TaZIK9	4565.A0A3B6PS74	4565.A0A3B5ZP32	0	0	0.142	0.573	0	0.699	0	0.151	0.761
TaMAPKKKK19	TaZIK3	4565.A0A3B6PS74	4565.A0A3B6AUS6	0	0	0.14	0.569	0	0.699	0	0.151	0.761
TaMAPKKKK19	TaZIK8	4565.A0A3B6PS74	4565.A0A3B6C5U6	0	0	0.126	0.629	0	0.699	0	0.151	0.757
TaMAPKKKK19	TaZIK7	4565.A0A3B6PS74	4565.A0A3B6C620	0	0	0.138	0.569	0	0.699	0	0.151	0.76
TaMAPKKKK19	TaZIK5	4565.A0A3B6PS74	4565.A0A3B6DA61	0	0	0.131	0.632	0	0.699	0	0.151	0.758
TaMAPKKKK19	TaZIK11	4565.A0A3B6PS74	4565.A0A3B6LI77	0	0	0.119	0.572	0	0.699	0	0.151	0.755
TaMAPKKKK19	TaZIK10	4565.A0A3B6PS74	4565.A0A3B6NT46	0	0	0.139	0.575	0	0.699	0	0.151	0.76
TaMAPKKKK19	TaZIK4	4565.A0A3B6PS74	4565.A0A3B6PML2	0	0	0.14	0.577	0	0.699	0	0.151	0.761
TaMAPKKKK19	TaZIK1	4565.A0A3B6PS74	4565.Q84XZ4	0	0	0.117	0.573	0	0.699	0	0.151	0.754
TaMAPKKKK19	TaZIK2	4565.A0A3B6PS74	4565.A0A3B6QG64	0	0	0.14	0.575	0	0.699	0	0.151	0.761
TaMAPKKKK2	TaZIK1	4565.A0A3B5YXW4	4565.Q84XZ4	0	0	0.116	0.568	0	0.699	0	0.151	0.754
TaMAPKKKK2	TaZIK11	4565.A0A3B5YXW4	4565.A0A3B6LI77	0	0	0.117	0.568	0	0.699	0	0.151	0.754
TaMAPKKKK2	TaZIK8	4565.A0A3B5YXW4	4565.A0A3B6C5U6	0	0	0.127	0.616	0	0.699	0	0.151	0.757
TaMAPKKKK2	TaZIK5	4565.A0A3B5YXW4	4565.A0A3B6DA61	0	0	0.131	0.618	0	0.699	0	0.151	0.758
TaMAPKKKK2	TaZIK2	4565.A0A3B5YXW4	4565.A0A3B6QG64	0	0	0.135	0.57	0	0.699	0	0.151	0.759

TaMAPKKKK2	TaZIK10	4565.A0A3B5YXW4	4565.A0A3B6NT46	0	0	0.134	0.57	0	0.699	0	0.151	0.759
TaMAPKKKK2	TaZIK7	4565.A0A3B5YXW4	4565.A0A3B6C620	0	0	0.138	0.564	0	0.699	0	0.151	0.76
TaMAPKKKK2	TaZIK9	4565.A0A3B5YXW4	4565.A0A3B5ZP32	0	0	0.137	0.569	0	0.699	0	0.151	0.76
TaMAPKKKK2	TaZIK4	4565.A0A3B5YXW4	4565.A0A3B6PML2	0	0	0.136	0.572	0	0.699	0	0.151	0.76
TaMAPKKKK2	TaZIK3	4565.A0A3B5YXW4	4565.A0A3B6AUS6	0	0	0.139	0.564	0	0.699	0	0.151	0.761
TaMAPKKKK20	TaZIK9	4565.A0A3B6QMW0	4565.A0A3B5ZP32	0	0	0.142	0.571	0	0.699	0	0.151	0.761
TaMAPKKKK20	TaZIK3	4565.A0A3B6QMW0	4565.A0A3B6AUS6	0	0	0.139	0.569	0	0.699	0	0.151	0.76
TaMAPKKKK20	TaZIK8	4565.A0A3B6QMW0	4565.A0A3B6C5U6	0	0	0.126	0.629	0	0.699	0	0.151	0.757
TaMAPKKKK20	TaZIK7	4565.A0A3B6QMW0	4565.A0A3B6C620	0	0	0.138	0.569	0	0.699	0	0.151	0.76
TaMAPKKKK20	TaZIK5	4565.A0A3B6QMW0	4565.A0A3B6DA61	0	0	0.131	0.632	0	0.699	0	0.151	0.758
TaMAPKKKK20	TaZIK11	4565.A0A3B6QMW0	4565.A0A3B6LI77	0	0	0.12	0.571	0	0.699	0	0.151	0.755
TaMAPKKKK20	TaZIK10	4565.A0A3B6QMW0	4565.A0A3B6NT46	0	0	0.139	0.575	0	0.699	0	0.151	0.76
TaMAPKKKK20	TaZIK4	4565.A0A3B6QMW0	4565.A0A3B6PML2	0	0	0.139	0.58	0	0.699	0	0.151	0.76
TaMAPKKKK20	TaZIK2	4565.A0A3B6QMW0	4565.A0A3B6QG64	0	0	0.14	0.575	0	0.699	0	0.151	0.761
TaMAPKKKK20	TaZIK1	4565.A0A3B6QMW0	4565.Q84XZ4	0	0	0.118	0.571	0	0.699	0	0.151	0.754
TaMAPKKKK21	TaZIK9	4565.A0A3B6QCF0	4565.A0A3B5ZP32	0	0	0.151	0.578	0	0.699	0	0.151	0.764
TaMAPKKKK21	TaZIK3	4565.A0A3B6QCF0	4565.A0A3B6AUS6	0	0	0.16	0.576	0	0.699	0	0.151	0.766
TaMAPKKKK21	TaZIK8	4565.A0A3B6QCF0	4565.A0A3B6C5U6	0	0	0.151	0.63	0	0.699	0	0.151	0.764

TaMAPKKKK21	TaZIK7	4565.A0A3B6QCF0	4565.A0A3B6C620	0	0	0.158	0.576	0	0.699	0	0.151	0.766
TaMAPKKKK21	TaZIK5	4565.A0A3B6QCF0	4565.A0A3B6DA61	0	0	0.156	0.633	0	0.699	0	0.151	0.765
TaMAPKKKK21	TaZIK11	4565.A0A3B6QCF0	4565.A0A3B6LI77	0	0	0.134	0.586	0	0.699	0	0.151	0.759
TaMAPKKKK21	TaZIK10	4565.A0A3B6QCF0	4565.A0A3B6NT46	0	0	0.16	0.575	0	0.699	0	0.151	0.766
TaMAPKKKK21	TaZIK4	4565.A0A3B6QCF0	4565.A0A3B6PML2	0	0	0.161	0.58	0	0.699	0	0.151	0.766
TaMAPKKKK21	TaZIK1	4565.A0A3B6QCF0	4565.Q84XZ4	0	0	0.133	0.586	0	0.699	0	0.151	0.759
TaMAPKKKK21	TaZIK2	4565.A0A3B6QCF0	4565.A0A3B6QG64	0	0	0.16	0.576	0	0.699	0	0.151	0.766
TaMAPKKKK22	TaZIK9	4565.A0A3B6RD82	4565.A0A3B5ZP32	0	0	0.136	0.572	0	0.699	0	0.151	0.76
TaMAPKKKK22	TaZIK3	4565.A0A3B6RD82	4565.A0A3B6AUS6	0	0	0.142	0.568	0	0.699	0	0.151	0.761
TaMAPKKKK22	TaZIK8	4565.A0A3B6RD82	4565.A0A3B6C5U6	0	0	0.13	0.619	0	0.699	0	0.151	0.758
TaMAPKKKK22	TaZIK7	4565.A0A3B6RD82	4565.A0A3B6C620	0	0	0.139	0.569	0	0.699	0	0.151	0.76
TaMAPKKKK22	TaZIK5	4565.A0A3B6RD82	4565.A0A3B6DA61	0	0	0.135	0.622	0	0.699	0	0.151	0.759
TaMAPKKKK22	TaZIK11	4565.A0A3B6RD82	4565.A0A3B6LI77	0	0	0.123	0.57	0	0.699	0	0.151	0.756
TaMAPKKKK22	TaZIK10	4565.A0A3B6RD82	4565.A0A3B6NT46	0	0	0.14	0.574	0	0.699	0	0.151	0.761
TaMAPKKKK22	TaZIK4	4565.A0A3B6RD82	4565.A0A3B6PML2	0	0	0.142	0.574	0	0.699	0	0.151	0.761
TaMAPKKKK22	TaZIK2	4565.A0A3B6RD82	4565.A0A3B6QG64	0	0	0.14	0.574	0	0.699	0	0.151	0.761
TaMAPKKKK22	TaZIK1	4565.A0A3B6RD82	4565.Q84XZ4	0	0	0.122	0.569	0	0.699	0	0.151	0.756
TaMAPKKKK23	TaZIK9	4565.A0A3B6SHY6	4565.A0A3B5ZP32	0	0	0.138	0.571	0	0.699	0	0.151	0.76

TaMAPKKKK23	TaZIK3	4565.A0A3B6SHY6	4565.A0A3B6AUS6	0	0	0.142	0.568	0	0.699	0	0.151	0.761
TaMAPKKKK23	TaZIK8	4565.A0A3B6SHY6	4565.A0A3B6C5U6	0	0	0.131	0.621	0	0.699	0	0.151	0.758
TaMAPKKKK23	TaZIK7	4565.A0A3B6SHY6	4565.A0A3B6C620	0	0	0.14	0.569	0	0.699	0	0.151	0.761
TaMAPKKKK23	TaZIK5	4565.A0A3B6SHY6	4565.A0A3B6DA61	0	0	0.135	0.623	0	0.699	0	0.151	0.759
TaMAPKKKK23	TaZIK11	4565.A0A3B6SHY6	4565.A0A3B6LI77	0	0	0.125	0.57	0	0.699	0	0.151	0.756
TaMAPKKKK23	TaZIK10	4565.A0A3B6SHY6	4565.A0A3B6NT46	0	0	0.141	0.574	0	0.699	0	0.151	0.761
TaMAPKKKK23	TaZIK4	4565.A0A3B6SHY6	4565.A0A3B6PML2	0	0	0.143	0.574	0	0.699	0	0.151	0.762
TaMAPKKKK23	TaZIK2	4565.A0A3B6SHY6	4565.A0A3B6QG64	0	0	0.141	0.574	0	0.699	0	0.151	0.761
TaMAPKKKK23	TaZIK1	4565.A0A3B6SHY6	4565.Q84XZ4	0	0	0.124	0.568	0	0.699	0	0.151	0.756
TaMAPKKKK24	TaZIK9	4565.A0A3B6TDA8	4565.A0A3B5ZP32	0	0	0.139	0.571	0	0.699	0	0.151	0.76
TaMAPKKKK24	TaZIK3	4565.A0A3B6TDA8	4565.A0A3B6AUS6	0	0	0.143	0.567	0	0.699	0	0.151	0.762
TaMAPKKKK24	TaZIK8	4565.A0A3B6TDA8	4565.A0A3B6C5U6	0	0	0.132	0.619	0	0.699	0	0.151	0.758
TaMAPKKKK24	TaZIK7	4565.A0A3B6TDA8	4565.A0A3B6C620	0	0	0.141	0.567	0	0.699	0	0.151	0.761
TaMAPKKKK24	TaZIK5	4565.A0A3B6TDA8	4565.A0A3B6DA61	0	0	0.137	0.622	0	0.699	0	0.151	0.76
TaMAPKKKK24	TaZIK11	4565.A0A3B6TDA8	4565.A0A3B6LI77	0	0	0.126	0.569	0	0.699	0	0.151	0.757
TaMAPKKKK24	TaZIK10	4565.A0A3B6TDA8	4565.A0A3B6NT46	0	0	0.142	0.573	0	0.699	0	0.151	0.761
TaMAPKKKK24	TaZIK4	4565.A0A3B6TDA8	4565.A0A3B6PML2	0	0	0.144	0.573	0	0.699	0	0.151	0.762
TaMAPKKKK24	TaZIK2	4565.A0A3B6TDA8	4565.A0A3B6QG64	0	0	0.142	0.574	0	0.699	0	0.151	0.761

TaMAPKKKK24	TaZIK1	4565.A0A3B6TDA8	4565.Q84XZ4	0	0	0.125	0.568	0	0.699	0	0.151	0.757
TaMAPKKKK25	TaZIK9	4565.A0A3B6UB28	4565.A0A3B5ZP32	0	0	0.145	0.577	0	0.699	0	0.151	0.762
TaMAPKKKK25	TaZIK3	4565.A0A3B6UB28	4565.A0A3B6AUS6	0	0	0.148	0.565	0	0.699	0	0.151	0.763
TaMAPKKKK25	TaZIK8	4565.A0A3B6UB28	4565.A0A3B6C5U6	0	0	0.131	0.615	0	0.699	0	0.151	0.758
TaMAPKKKK25	TaZIK7	4565.A0A3B6UB28	4565.A0A3B6C620	0	0	0.147	0.565	0	0.699	0	0.151	0.763
TaMAPKKKK25	TaZIK5	4565.A0A3B6UB28	4565.A0A3B6DA61	0	0	0.136	0.62	0	0.699	0	0.151	0.759
TaMAPKKKK25	TaZIK11	4565.A0A3B6UB28	4565.A0A3B6LI77	0	0	0.124	0.565	0	0.699	0	0.151	0.756
TaMAPKKKK25	TaZIK10	4565.A0A3B6UB28	4565.A0A3B6NT46	0	0	0.141	0.571	0	0.699	0	0.151	0.761
TaMAPKKKK25	TaZIK4	4565.A0A3B6UB28	4565.A0A3B6PML2	0	0	0.144	0.574	0	0.699	0	0.151	0.762
TaMAPKKKK25	TaZIK2	4565.A0A3B6UB28	4565.A0A3B6QG64	0	0	0.142	0.571	0	0.699	0	0.151	0.761
TaMAPKKKK25	TaZIK1	4565.A0A3B6UB28	4565.Q84XZ4	0	0	0.123	0.565	0	0.699	0	0.151	0.756
TaMAPKKKK3	TaZIK9	4565.A0A3B5ZTC4	4565.A0A3B5ZP32	0	0	0.138	0.569	0	0.699	0	0.151	0.76
TaMAPKKKK3	TaZIK1	4565.A0A3B5ZTC4	4565.Q84XZ4	0	0	0.116	0.568	0	0.699	0	0.151	0.754
TaMAPKKKK3	TaZIK11	4565.A0A3B5ZTC4	4565.A0A3B6LI77	0	0	0.117	0.568	0	0.699	0	0.151	0.754
TaMAPKKKK3	TaZIK8	4565.A0A3B5ZTC4	4565.A0A3B6C5U6	0	0	0.127	0.616	0	0.699	0	0.151	0.757
TaMAPKKKK3	TaZIK5	4565.A0A3B5ZTC4	4565.A0A3B6DA61	0	0	0.131	0.619	0	0.699	0	0.151	0.758
TaMAPKKKK3	TaZIK10	4565.A0A3B5ZTC4	4565.A0A3B6NT46	0	0	0.134	0.57	0	0.699	0	0.151	0.759
TaMAPKKKK3	TaZIK2	4565.A0A3B5ZTC4	4565.A0A3B6QG64	0	0	0.135	0.57	0	0.699	0	0.151	0.759

TaMAPKKKK3	TaZIK7	4565.A0A3B5ZTC4	4565.A0A3B6C620	0	0	0.138	0.564	0	0.699	0	0.151	0.76
TaMAPKKKK3	TaZIK4	4565.A0A3B5ZTC4	4565.A0A3B6PML2	0	0	0.136	0.572	0	0.699	0	0.151	0.76
TaMAPKKKK3	TaZIK3	4565.A0A3B5ZTC4	4565.A0A3B6AUS6	0	0	0.139	0.564	0	0.699	0	0.151	0.761
TaMAPKKKK4	TaMEKK7	4565.A0A3B6AY64	4565.A0A077RUI2	0	0	0.143	0.623	0	0.175	0.685	0.151	0.785
TaMAPKKKK4	TaMEKK9	4565.A0A3B6AY64	4565.A0A077S2G5	0	0	0.13	0.633	0	0.175	0.685	0.151	0.782
TaMAPKKKK4	TaRaf22	4565.A0A3B6AY64	4565.A0A3B6I1C9	0	0	0	0.562	0	0.384	0	0.097	0.419
TaMAPKKKK4	TaRaf81	4565.A0A3B6AY64	4565.A0A3B6D624	0	0	0	0.557	0	0.384	0	0.097	0.419
TaMAPKKKK4	TaRaf94	4565.A0A3B6AY64	4565.A0A3B6N1G5	0	0	0	0.562	0	0.384	0	0.097	0.419
TaMAPKKKK4	TaRaf89	4565.A0A3B6AY64	4565.A0A3B6PRF9	0	0	0	0.552	0	0.384	0	0.097	0.419
TaMAPKKKK4	TaRaf111	4565.A0A3B6AY64	4565.A0A3B6FHS8	0	0	0	0	0	0.148	0.509	0	0.563
TaMAPKKKK4	TaMEKK20	4565.A0A3B6AY64	4565.A0A3B6B6T5	0	0	0	0.664	0	0.175	0.685	0.151	0.76
TaMAPKKKK4	TaMEKK18	4565.A0A3B6AY64	4565.A0A3B6CEA6	0	0	0	0.664	0	0.175	0.685	0.151	0.76
TaMAPKKKK4	TaMEKK4	4565.A0A3B6AY64	4565.A0A3B6NRN9	0	0	0.12	0.669	0	0.175	0.685	0.151	0.779
TaMAPKKKK4	TaMEKK4-1	4565.A0A3B6AY64	4565.A0A3B6PNI6	0	0	0.119	0.671	0	0.175	0.685	0.151	0.779
TaMAPKKKK4	TaMEKK1	4565.A0A3B6AY64	4565.A0A3B6B3I4	0	0	0.123	0.671	0	0.175	0.685	0.151	0.78
TaMAPKKKK4	TaMEKK8	4565.A0A3B6AY64	4565.A0A3B6FNU8	0	0	0.136	0.62	0	0.175	0.685	0.151	0.783
TaMAPKKKK4	TaMEKK17	4565.A0A3B6AY64	4565.A0A3B6KFL8	0	0	0.145	0.655	0	0.175	0.685	0.151	0.786
TaMAPKKKK4	TaMEKK29	4565.A0A3B6AY64	4565.A0A3B6MSP6	0	0	0.146	0.654	0	0.175	0.685	0.151	0.786

TaMAPKKKK4	TaMEKK24	4565.A0A3B6AY64	4565.A0A3B6LLV5	0	0	0.145	0.655	0	0.175	0.685	0.151	0.786
TaMAPKKKK5	TaMEKK7	4565.A0A3B6C4T6	4565.A0A077RUI2	0	0	0.143	0.623	0	0.175	0.685	0.151	0.785
TaMAPKKKK5	TaMEKK9	4565.A0A3B6C4T6	4565.A0A077S2G5	0	0	0.13	0.632	0	0.175	0.685	0.151	0.782
TaMAPKKKK5	TaMEKK1	4565.A0A3B6C4T6	4565.A0A3B6B3I4	0	0	0.123	0.67	0	0.175	0.685	0.151	0.78
TaMAPKKKK5	TaMEKK20	4565.A0A3B6C4T6	4565.A0A3B6B6T5	0	0	0	0.664	0	0.175	0.685	0.151	0.76
TaMAPKKKK5	TaRaf89	4565.A0A3B6C4T6	4565.A0A3B6PRF9	0	0	0	0.551	0	0.384	0	0.097	0.419
TaMAPKKKK5	TaRaf94	4565.A0A3B6C4T6	4565.A0A3B6N1G5	0	0	0	0.562	0	0.384	0	0.097	0.419
TaMAPKKKK5	TaRaf81	4565.A0A3B6C4T6	4565.A0A3B6D624	0	0	0	0.557	0	0.384	0	0.097	0.419
TaMAPKKKK5	TaRaf22	4565.A0A3B6C4T6	4565.A0A3B6I1C9	0	0	0	0.562	0	0.384	0	0.097	0.419
TaMAPKKKK5	TaRaf111	4565.A0A3B6C4T6	4565.A0A3B6FHS8	0	0	0	0	0	0.148	0.509	0	0.563
TaMAPKKKK5	TaMEKK18	4565.A0A3B6C4T6	4565.A0A3B6CEA6	0	0	0	0.664	0	0.175	0.685	0.151	0.76
TaMAPKKKK5	TaMEKK4	4565.A0A3B6C4T6	4565.A0A3B6NRN9	0	0	0.12	0.669	0	0.175	0.685	0.151	0.779
TaMAPKKKK5	TaMEKK4-1	4565.A0A3B6C4T6	4565.A0A3B6PNI6	0	0	0.119	0.67	0	0.175	0.685	0.151	0.779
TaMAPKKKK5	TaMEKK8	4565.A0A3B6C4T6	4565.A0A3B6FNU8	0	0	0.136	0.62	0	0.175	0.685	0.151	0.783
TaMAPKKKK5	TaMEKK17	4565.A0A3B6C4T6	4565.A0A3B6KFL8	0	0	0.144	0.655	0	0.175	0.685	0.151	0.785
TaMAPKKKK5	TaMEKK29	4565.A0A3B6C4T6	4565.A0A3B6MSP6	0	0	0.145	0.654	0	0.175	0.685	0.151	0.786
TaMAPKKKK5	TaMEKK24	4565.A0A3B6C4T6	4565.A0A3B6LLV5	0	0	0.144	0.655	0	0.175	0.685	0.151	0.786
TaMAPKKKK6	TaMEKK7	4565.A0A3B6DEP0	4565.A0A077RUI2	0	0	0.144	0.624	0	0.175	0.685	0.151	0.785

TaMAPKKKK6	TaMEKK9	4565.A0A3B6DEP0	4565.A0A077S2G5	0	0	0.131	0.633	0	0.175	0.685	0.151	0.782
TaMAPKKKK6	TaMEKK1	4565.A0A3B6DEP0	4565.A0A3B6B3I4	0	0	0.124	0.671	0	0.175	0.685	0.151	0.78
TaMAPKKKK6	TaMEKK20	4565.A0A3B6DEP0	4565.A0A3B6B6T5	0	0	0	0.664	0	0.175	0.685	0.151	0.76
TaMAPKKKK6	TaMEKK18	4565.A0A3B6DEP0	4565.A0A3B6CEA6	0	0	0	0.665	0	0.175	0.685	0.151	0.76
TaMAPKKKK6	TaRaf81	4565.A0A3B6DEP0	4565.A0A3B6D624	0	0	0	0.557	0	0.384	0	0.097	0.419
TaMAPKKKK6	TaRaf22	4565.A0A3B6DEP0	4565.A0A3B6I1C9	0	0	0	0.562	0	0.384	0	0.097	0.419
TaMAPKKKK6	TaRaf89	4565.A0A3B6DEP0	4565.A0A3B6PRF9	0	0	0	0.551	0	0.384	0	0.097	0.419
TaMAPKKKK6	TaRaf94	4565.A0A3B6DEP0	4565.A0A3B6N1G5	0	0	0	0.562	0	0.384	0	0.097	0.419
TaMAPKKKK6	TaRaf111	4565.A0A3B6DEP0	4565.A0A3B6FHS8	0	0	0	0	0	0.148	0.509	0	0.563
TaMAPKKKK6	TaMEKK4-1	4565.A0A3B6DEP0	4565.A0A3B6PNI6	0	0	0.12	0.671	0	0.175	0.685	0.151	0.779
TaMAPKKKK6	TaMEKK4	4565.A0A3B6DEP0	4565.A0A3B6NRN9	0	0	0.12	0.669	0	0.175	0.685	0.151	0.78
TaMAPKKKK6	TaMEKK8	4565.A0A3B6DEP0	4565.A0A3B6FNU8	0	0	0.136	0.62	0	0.175	0.685	0.151	0.784
TaMAPKKKK6	TaMEKK17	4565.A0A3B6DEP0	4565.A0A3B6KFL8	0	0	0.145	0.654	0	0.175	0.685	0.151	0.786
TaMAPKKKK6	TaMEKK24	4565.A0A3B6DEP0	4565.A0A3B6LLV5	0	0	0.145	0.655	0	0.175	0.685	0.151	0.786
TaMAPKKKK6	TaMEKK29	4565.A0A3B6DEP0	4565.A0A3B6MSP6	0	0	0.146	0.654	0	0.175	0.685	0.151	0.786
TaMAPKKKK7	TaZIK9	4565.A0A3B6IX47	4565.A0A3B5ZP32	0	0	0.134	0.569	0	0.699	0	0.151	0.759
TaMAPKKKK7	TaZIK3	4565.A0A3B6IX47	4565.A0A3B6AUS6	0	0	0.131	0.558	0	0.699	0	0.151	0.758
TaMAPKKKK7	TaZIK8	4565.A0A3B6IX47	4565.A0A3B6C5U6	0	0	0.123	0.578	0	0.699	0	0.151	0.756

TaMAPKKKK7	TaZIK7	4565.A0A3B6IX47	4565.A0A3B6C620	0	0	0.129	0.558	0	0.699	0	0.151	0.758
TaMAPKKKK7	TaZIK5	4565.A0A3B6IX47	4565.A0A3B6DA61	0	0	0.126	0.578	0	0.699	0	0.151	0.757
TaMAPKKKK7	TaZIK11	4565.A0A3B6IX47	4565.A0A3B6LI77	0	0	0	0.556	0	0.699	0	0.151	0.733
TaMAPKKKK7	TaZIK1	4565.A0A3B6IX47	4565.Q84XZ4	0	0	0	0.556	0	0.699	0	0.151	0.733
TaMAPKKKK7	TaZIK10	4565.A0A3B6IX47	4565.A0A3B6NT46	0	0	0.124	0.564	0	0.699	0	0.151	0.756
TaMAPKKKK7	TaZIK2	4565.A0A3B6IX47	4565.A0A3B6QG64	0	0	0.124	0.564	0	0.699	0	0.151	0.756
TaMAPKKKK7	TaZIK4	4565.A0A3B6IX47	4565.A0A3B6PML2	0	0	0.127	0.565	0	0.699	0	0.151	0.757
TaMAPKKKK8	TaZIK9	4565.A0A3B6J0T1	4565.A0A3B5ZP32	0	0	0.145	0.578	0	0.699	0	0.151	0.762
TaMAPKKKK8	TaZIK3	4565.A0A3B6J0T1	4565.A0A3B6AUS6	0	0	0.149	0.565	0	0.699	0	0.151	0.763
TaMAPKKKK8	TaZIK8	4565.A0A3B6J0T1	4565.A0A3B6C5U6	0	0	0.133	0.613	0	0.699	0	0.151	0.759
TaMAPKKKK8	TaZIK7	4565.A0A3B6J0T1	4565.A0A3B6C620	0	0	0.148	0.565	0	0.699	0	0.151	0.763
TaMAPKKKK8	TaZIK5	4565.A0A3B6J0T1	4565.A0A3B6DA61	0	0	0.137	0.622	0	0.699	0	0.151	0.76
TaMAPKKKK8	TaZIK1	4565.A0A3B6J0T1	4565.Q84XZ4	0	0	0.124	0.564	0	0.699	0	0.151	0.756
TaMAPKKKK8	TaZIK11	4565.A0A3B6J0T1	4565.A0A3B6LI77	0	0	0.124	0.564	0	0.699	0	0.151	0.756
TaMAPKKKK8	TaZIK10	4565.A0A3B6J0T1	4565.A0A3B6NT46	0	0	0.142	0.571	0	0.699	0	0.151	0.761
TaMAPKKKK8	TaZIK2	4565.A0A3B6J0T1	4565.A0A3B6QG64	0	0	0.143	0.571	0	0.699	0	0.151	0.761
TaMAPKKKK8	TaZIK4	4565.A0A3B6J0T1	4565.A0A3B6PML2	0	0	0.144	0.574	0	0.699	0	0.151	0.762
TaMAPKKKK9	TaZIK9	4565.A0A3B6KID5	4565.A0A3B5ZP32	0	0	0.11	0.572	0	0.699	0	0.151	0.752

TaMAPKKKK9	TaZIK3	4565.A0A3B6KID5	4565.A0A3B6AUS6	0	0	0.119	0.572	0	0.699	0	0.151	0.755
TaMAPKKKK9	TaZIK8	4565.A0A3B6KID5	4565.A0A3B6C5U6	0	0	0.112	0.596	0	0.699	0	0.151	0.753
TaMAPKKKK9	TaZIK7	4565.A0A3B6KID5	4565.A0A3B6C620	0	0	0.118	0.572	0	0.699	0	0.151	0.755
TaMAPKKKK9	TaZIK5	4565.A0A3B6KID5	4565.A0A3B6DA61	0	0	0.116	0.599	0	0.699	0	0.151	0.754
TaMAPKKKK9	TaZIK1	4565.A0A3B6KID5	4565.Q84XZ4	0	0	0	0.562	0	0.699	0	0.151	0.733
TaMAPKKKK9	TaZIK11	4565.A0A3B6KID5	4565.A0A3B6LI77	0	0	0	0.565	0	0.699	0	0.151	0.733
TaMAPKKKK9	TaZIK10	4565.A0A3B6KID5	4565.A0A3B6NT46	0	0	0.111	0.57	0	0.699	0	0.151	0.753
TaMAPKKKK9	TaZIK2	4565.A0A3B6KID5	4565.A0A3B6QG64	0	0	0.111	0.571	0	0.699	0	0.151	0.753
TaMAPKKKK9	TaZIK4	4565.A0A3B6KID5	4565.A0A3B6PML2	0	0	0.111	0.57	0	0.699	0	0.151	0.753
TaMEKK1	TaMEKK17	4565.A0A3B6B3I4	4565.A0A3B6KFL8	0	0	0.158	0.757	0	0	0.59	0	0.64
TaMEKK1	TaMEKK29	4565.A0A3B6B3I4	4565.A0A3B6MSP6	0	0	0.158	0.756	0	0	0.59	0	0.64
TaMEKK1	TaMEKK24	4565.A0A3B6B3I4	4565.A0A3B6LLV5	0	0	0.158	0.757	0	0	0.59	0	0.64
TaMEKK1	TaMEKK4-1	4565.A0A3B6B3I4	4565.A0A3B6PNI6	0	0	0.052	0.977	0	0	0.927	0	0.927
TaMEKK1	TaMEKK4	4565.A0A3B6B3I4	4565.A0A3B6NRN9	0	0	0.054	0.973	0	0	0.927	0	0.928
TaMEKK10	TaRaf18	4565.A0A3B6JM32	4565.A0A077RY41	0	0	0.197	0.61	0	0.133	0.52	0	0.636
TaMEKK10	TaRaf29	4565.A0A3B6JM32	4565.A0A1D5UHD7	0	0	0.168	0.597	0	0.133	0.52	0	0.623
TaMEKK10	TaRaf88	4565.A0A3B6JM32	4565.A0A3B5Z5X1	0	0	0.196	0.62	0	0.133	0.52	0	0.636
TaMEKK10	TaRaf30	4565.A0A3B6JM32	4565.A0A3B6A1Z4	0	0	0.194	0.617	0	0.133	0.52	0	0.635

TaMEKK10	TaRaf79	4565.A0A3B6JM32	4565.A0A3B6B9C7	0	0	0.165	0.607	0	0.133	0.52	0	0.622
TaMEKK10	TaRaf59	4565.A0A3B6JM32	4565.A0A3B6E9E2	0	0	0.144	0.654	0	0.133	0.52	0	0.612
TaMEKK10	TaRaf60	4565.A0A3B6JM32	4565.A0A3B6EHW0	0	0	0.207	0.574	0	0.133	0.52	0	0.641
TaMEKK10	TaRaf5	4565.A0A3B6JM32	4565.A0A3B6GYQ1	0	0	0.214	0.575	0	0.133	0.52	0	0.644
TaMEKK10	TaRaf100	4565.A0A3B6JM32	4565.A0A3B6ML06	0	0	0	0.573	0	0.133	0.52	0	0.566
TaMEKK10	TaRaf91	4565.A0A3B6JM32	4565.A0A3B6PMI5	0	0	0.13	0.575	0	0.133	0.52	0	0.606
TaMEKK10	TaRaf1	4565.A0A3B6JM32	4565.A0A3B6NP57	0	0	0.141	0.652	0	0.133	0.52	0	0.611
TaMEKK10	TaRaf52	4565.A0A3B6JM32	4565.A0A3B6LRR0	0	0	0.144	0.608	0	0.133	0.52	0	0.612
TaMEKK10	TaRaf21	4565.A0A3B6JM32	4565.A0A3B6TVC5	0	0	0.145	0.633	0	0.133	0.52	0	0.613
TaMEKK10	TaRaf63	4565.A0A3B6JM32	4565.A0A3B6RKW0	0	0	0.147	0.599	0	0.133	0.52	0	0.614
TaMEKK10	TaRaf58	4565.A0A3B6JM32	4565.A0A3B6N1Y2	0	0	0.16	0.608	0	0.133	0.52	0	0.62
TaMEKK10	TaRaf46	4565.A0A3B6JM32	4565.A0A3B6MW69	0	0	0.188	0.623	0	0.133	0.52	0	0.632
TaMEKK10	TaRaf62	4565.A0A3B6JM32	4565.A9RAA9	0	0	0.19	0.632	0	0.133	0.52	0	0.633
TaMEKK11	TaRaf41	4565.A0A3B6N0D8	4565.A0A3B6KLD7	0	0	0.305	0	0	0.114	0.109	0	0.403
TaMEKK12	TaRaf18	4565.A0A3B6HQC5	4565.A0A077RY41	0	0	0.196	0.61	0	0.133	0.52	0	0.636
TaMEKK12	TaRaf29	4565.A0A3B6HQC5	4565.A0A1D5UHD7	0	0	0.169	0.595	0	0.133	0.52	0	0.624
TaMEKK12	TaRaf88	4565.A0A3B6HQC5	4565.A0A3B5Z5X1	0	0	0.193	0.625	0	0.133	0.52	0	0.634
TaMEKK12	TaRaf30	4565.A0A3B6HQC5	4565.A0A3B6A1Z4	0	0	0.193	0.618	0	0.133	0.52	0	0.635

TaMEKK12	TaRaf79	4565.A0A3B6HQC5	4565.A0A3B6B9C7	0	0	0.165	0.606	0	0.133	0.52	0	0.622
TaMEKK12	TaRaf59	4565.A0A3B6HQC5	4565.A0A3B6E9E2	0	0	0.144	0.655	0	0.133	0.52	0	0.612
TaMEKK12	TaRaf60	4565.A0A3B6HQC5	4565.A0A3B6EHW0	0	0	0.208	0.575	0	0.133	0.52	0	0.641
TaMEKK12	TaRaf5	4565.A0A3B6HQC5	4565.A0A3B6GYQ1	0	0	0.214	0.576	0	0.133	0.52	0	0.644
TaMEKK12	TaRaf100	4565.A0A3B6HQC5	4565.A0A3B6ML06	0	0	0	0.573	0	0.133	0.52	0	0.566
TaMEKK12	TaRaf91	4565.A0A3B6HQC5	4565.A0A3B6PMI5	0	0	0.128	0.573	0	0.133	0.52	0	0.605
TaMEKK12	TaRaf1	4565.A0A3B6HQC5	4565.A0A3B6NP57	0	0	0.139	0.654	0	0.133	0.52	0	0.61
TaMEKK12	TaRaf21	4565.A0A3B6HQC5	4565.A0A3B6TVC5	0	0	0.144	0.633	0	0.133	0.52	0	0.612
TaMEKK12	TaRaf52	4565.A0A3B6HQC5	4565.A0A3B6LRR0	0	0	0.146	0.607	0	0.133	0.52	0	0.613
TaMEKK12	TaRaf63	4565.A0A3B6HQC5	4565.A0A3B6RKW0	0	0	0.147	0.599	0	0.133	0.52	0	0.614
TaMEKK12	TaRaf58	4565.A0A3B6HQC5	4565.A0A3B6N1Y2	0	0	0.159	0.608	0	0.133	0.52	0	0.619
TaMEKK12	TaRaf46	4565.A0A3B6HQC5	4565.A0A3B6MW69	0	0	0.186	0.624	0	0.133	0.52	0	0.632
TaMEKK12	TaRaf62	4565.A0A3B6HQC5	4565.A9RAA9	0	0	0.189	0.632	0	0.133	0.52	0	0.633
TaMEKK15	TaRaf41	4565.A0A3B6KPK7	4565.A0A3B6KLD7	0	0	0.307	0	0	0.114	0.109	0	0.405
TaMEKK16	TaRaf41	4565.A0A3B6LW00	4565.A0A3B6KLD7	0	0	0.312	0	0	0.114	0.109	0	0.409
TaMEKK16	TaRaf7	4565.A0A3B6LW00	4565.A0A3B6TBH5	0	0	0.303	0	0	0.114	0.109	0	0.401
TaMEKK17	TaMEKK4-1	4565.A0A3B6KFL8	4565.A0A3B6PNI6	0	0	0.158	0.756	0	0	0.59	0	0.64
TaMEKK17	TaMEKK4	4565.A0A3B6KFL8	4565.A0A3B6NRN9	0	0	0.16	0.753	0	0	0.59	0	0.641

TaMEKK24	TaMEKK4-1	4565.A0A3B6LLV5	4565.A0A3B6PNI6	0	0	0.158	0.756	0	0	0.59	0	0.64
TaMEKK24	TaMEKK4	4565.A0A3B6LLV5	4565.A0A3B6NRN9	0	0	0.16	0.753	0	0	0.59	0	0.641
TaMEKK25	TaZIK9	4565.A0A3B6LLP7	4565.A0A3B5ZP32	0	0	0.112	0.572	0	0.699	0	0.151	0.753
TaMEKK25	TaZIK3	4565.A0A3B6LLP7	4565.A0A3B6AUS6	0	0	0.123	0.571	0	0.699	0	0.151	0.756
TaMEKK25	TaZIK8	4565.A0A3B6LLP7	4565.A0A3B6C5U6	0	0	0.115	0.598	0	0.699	0	0.151	0.754
TaMEKK25	TaZIK7	4565.A0A3B6LLP7	4565.A0A3B6C620	0	0	0.122	0.571	0	0.699	0	0.151	0.756
TaMEKK25	TaZIK5	4565.A0A3B6LLP7	4565.A0A3B6DA61	0	0	0.12	0.6	0	0.699	0	0.151	0.755
TaMEKK25	TaZIK11	4565.A0A3B6LLP7	4565.A0A3B6LI77	0	0	0	0.564	0	0.699	0	0.151	0.733
TaMEKK25	TaZIK1	4565.A0A3B6LLP7	4565.Q84XZ4	0	0	0	0.561	0	0.699	0	0.151	0.733
TaMEKK25	TaZIK4	4565.A0A3B6LLP7	4565.A0A3B6PML2	0	0	0.114	0.57	0	0.699	0	0.151	0.753
TaMEKK25	TaZIK2	4565.A0A3B6LLP7	4565.A0A3B6QG64	0	0	0.114	0.57	0	0.699	0	0.151	0.753
TaMEKK25	TaZIK10	4565.A0A3B6LLP7	4565.A0A3B6NT46	0	0	0.114	0.569	0	0.699	0	0.151	0.753
TaMEKK29	TaMEKK4-1	4565.A0A3B6MSP6	4565.A0A3B6PNI6	0	0	0.159	0.755	0	0	0.59	0	0.64
TaMEKK29	TaMEKK4	4565.A0A3B6MSP6	4565.A0A3B6NRN9	0	0	0.16	0.752	0	0	0.59	0	0.641
TaMEKK3	TaRaf18	4565.A0A3B6ISF1	4565.A0A077RY41	0	0	0.196	0.61	0	0.133	0.52	0	0.636
TaMEKK3	TaRaf29	4565.A0A3B6ISF1	4565.A0A1D5UHD7	0	0	0.17	0.596	0	0.133	0.52	0	0.624
TaMEKK3	TaRaf88	4565.A0A3B6ISF1	4565.A0A3B5Z5X1	0	0	0.194	0.62	0	0.133	0.52	0	0.635
TaMEKK3	TaRaf30	4565.A0A3B6ISF1	4565.A0A3B6A1Z4	0	0	0.193	0.617	0	0.133	0.52	0	0.635

TaMEKK3	TaRaf79	4565.A0A3B6ISF1	4565.A0A3B6B9C7	0	0	0.166	0.607	0	0.133	0.52	0	0.622
TaMEKK3	TaRaf59	4565.A0A3B6ISF1	4565.A0A3B6E9E2	0	0	0.144	0.654	0	0.133	0.52	0	0.612
TaMEKK3	TaRaf60	4565.A0A3B6ISF1	4565.A0A3B6EHW0	0	0	0.207	0.574	0	0.133	0.52	0	0.641
TaMEKK3	TaRaf5	4565.A0A3B6ISF1	4565.A0A3B6GYQ1	0	0	0.214	0.575	0	0.133	0.52	0	0.644
TaMEKK3	TaRaf100	4565.A0A3B6ISF1	4565.A0A3B6ML06	0	0	0	0.573	0	0.133	0.52	0	0.566
TaMEKK3	TaRaf91	4565.A0A3B6ISF1	4565.A0A3B6PMI5	0	0	0.127	0.575	0	0.133	0.52	0	0.605
TaMEKK3	TaRaf1	4565.A0A3B6ISF1	4565.A0A3B6NP57	0	0	0.14	0.652	0	0.133	0.52	0	0.611
TaMEKK3	TaRaf21	4565.A0A3B6ISF1	4565.A0A3B6TVC5	0	0	0.144	0.632	0	0.133	0.52	0	0.612
TaMEKK3	TaRaf52	4565.A0A3B6ISF1	4565.A0A3B6LRR0	0	0	0.146	0.607	0	0.133	0.52	0	0.613
TaMEKK3	TaRaf63	4565.A0A3B6ISF1	4565.A0A3B6RKW0	0	0	0.148	0.599	0	0.133	0.52	0	0.614
TaMEKK3	TaRaf58	4565.A0A3B6ISF1	4565.A0A3B6N1Y2	0	0	0.161	0.606	0	0.133	0.52	0	0.62
TaMEKK3	TaRaf46	4565.A0A3B6ISF1	4565.A0A3B6MW69	0	0	0.187	0.623	0	0.133	0.52	0	0.632
TaMEKK3	TaRaf62	4565.A0A3B6ISF1	4565.A9RAA9	0	0	0.19	0.632	0	0.133	0.52	0	0.633
TaMEKK5	TaRaf41	4565.A0A3B6AWC1	4565.A0A3B6KLD7	0	0	0.309	0	0	0.114	0.109	0	0.406
TaMEKK9	TaRaf14	4565.A0A077S2G5	4565.A0A3B6TYA5	0	0	0.289	0	0	0.139	0.137	0	0.425
TaRaf114	TaRaf51	4565.A0A3B6FV36	4565.A0A3B6JHE9	0	0	0.084	0.911	0	0	0.54	0	0.56

Table S5 The expression profile data of MAPK-MAP4Ks.

gene id	gene	6dhs2	6dhs1	1dhs2	1dhs1	6h2	6h1	1h2	1h1	6d2	6d1	1d2	1d1	c2	c1
**TraesCS6B02G296700.2**	**TaMAPK1**	4.23195	5.5986	2.93146	3.00281	5.36652	4.37479	2.30859	2.31239	5.12847	5.54706	8.79167	6.85185	5.99579	9.1542
**TraesCS4A02G336800.2**	**TaMAPK2**	0	2.26E-07	0	0.27777	0	0.12886	0.01781	0	2.06E-09	0	0	0	0	0
**TraesCS4A02G106400.1**	**TaMAPK3**	7.52281	6.79529	3.42336	4.91829	9.40216	9.57002	6.72631	4.25848	4.82013	4.49729	38.1351	29.3051	9.64112	12.2491
**TraesCS1D02G088000.2**	**TaMAPK4**	0	0	0	0	0	0	0	0	0	0	0.13285	0.1911	0	0
**TraesCS1D02G422800.1**	**TaMAPK5**	0.0481	0.05089	0	0	0	0.05709	0	0.03243	0.16597	0	0.26064	0.22346	0.24746	0.3424
**TraesCS7B02G009200.1**	**TaMAPK6**	7.21125	7.3137	1.58199	1.80781	8.30898	7.67016	3.06793	2.82839	4.9529	5.96049	5.77572	4.01002	4.51255	6.29247
**TraesCS7D02G342800.3**	**TaMAPK7**	4.78129	4.88311	1.94705	1.93348	5.02468	5.38939	1.6256	1.35235	3.68614	3.54684	3.37577	2.79714	2.71373	3.87964
**TraesCS3D02G225600.1**	**TaMAPK8**	4.44048	4.40988	1.41612	1.83542	10.9193	10.5782	2.10702	1.91478	1.94834	2.12059	2.47205	2.0767	2.18523	2.66536
**TraesCS6B02G146300.1**	**TaMAPK10**	12.4553	15.1263	5.52391	5.2575	15.6588	18.4475	6.98601	5.73359	7.32178	7.40639	11.2116	8.72001	6.98657	8.45537
**TraesCS1A02G086500.1**	**TaMAPK11**	1.58332	1.6488	1.01691	0.8972	2.1937	2.19314	1.36336	1.3095	2.45412	2.63541	3.22123	2.34123	1.89717	2.70034
**TraesCS7A02G422500.1**	**TaMAPK12**	0.52592	0.81962	1.46766	1.59519	0.78016	0.79401	3.54849	2.7636	11.4136	10.0916	16.2846	13.4388	6.91316	7.42971

**TraesCS6D02G245500.3**	**TaMAPK13**	7.59712	9.19514	1.88416	3.04018	6.11007	7.97333	2.14702	3.85856	4.4303	6.1805	8.03121	4.39587	5.23465	7.81215
**TraesCS1A02G184500.1**	**TaMAPK14**	6.18036	6.37175	3.25418	3.24831	8.01795	8.41098	5.21467	4.1883	4.3158	4.14187	9.98283	8.05199	6.19477	8.4653
**TraesCS3B02G270200.1**	**TaMAPK16**	2.93462	3.13547	1.77221	1.82908	3.33936	2.81853	3.48574	2.76808	6.91275	6.74878	9.44762	6.92497	9.0083	12.6339
**TraesCS6B02G127800.1**	**TaMAPK17**	0	0	0	0.01161	0	0.00885	0	0	0	0	0	0	0.00811	0
**TraesCS7D02G044100.1**	**TaMAPK18**	36.7621	37.6997	15.3365	17.6187	34.153	33.9526	12.8063	8.85031	34.3126	32.708	38.7491	31.8673	24.3932	31.5904
**TraesCS7A02G111300.1**	**TaMAPK19**	0.48408	0.6424	0.35618	0.17223	0.71562	0.70617	0.4959	0.24626	0.52304	0.46932	0.49554	0.39186	0.58151	0.79753
**TraesCS7D02G403700.1**	**TaMAPK20**	0	0	0.13939	0.46897	0.38733	0	0	0.61179	0.19132	0	0.32293	0	0	0.3691
**TraesCS7D02G414900.1**	**TaMAPK22**	0.1314	0.10205	0.01654	0.0328	0.10429	0.05069	0.03603	0.08189	0.97597	0.92162	0.27566	0.27698	0.07183	0.08988
**TraesCS3D02G221700.1**	**TaMAPK23**	0.81414	0.8702	0.58578	0.67828	1.1271	1.12849	1.27288	1.10799	0.9085	0.7681	1.23184	1.00786	0.98504	1.40208
**TraesCS3D02G242200.2**	**TaMAPK24**	5.08755	5.80447	2.66261	2.35555	5.0612	4.97478	3.59982	2.90991	7.53011	7.29655	9.68926	6.869	9.39714	13.1337
**TraesCS4D02G198600.1**	**TaMAPK25**	9.30833	9.7448	6.26549	7.6517	15.8908	16.6113	14.0556	10.2497	13.1424	11.4694	58.814	46.7737	13.816	19.2555
**TraesCS5D02G534000.2**	**TaMAPK26**	0.07056	0	0	0.20026	0	0	0	0.28926	0.15063	0.16221	0.73291	0.20936	0.24837	0.09393

**TraesCS1B02G104900.1**	**TaMAPK27**	3.21833	3.84264	1.95737	1.49807	3.99496	4.00491	3.17776	2.9718	5.65883	6.03874	9.2461	6.91724	6.05644	7.99628
**TraesCS1B02G192600.3**	**TaMAPK28**	19.2486	18.8315	8.40185	10.846	22.6783	24.749	14.3783	11.2447	9.57706	9.6599	24.4988	20.829	14.5758	20.1423
**TraesCS1B02G431400.2**	**TaMAPK29**	0	0	0	0	0	0	0	0	0	0	0	0	0	0
**TraesCS7A02G335300.2**	**TaMAPK30**	1.37221	2.07075	0.65552	0.67418	1.18988	0.47827	0.46454	0.81131	0.28059	0	0.42468	0.44881	0.077	0.54622
**TraesCS4A02G434800.1**	**TaMAPK31**	32.9484	34.1062	16.0239	17.8452	44.5389	43.5812	14.1792	11.2272	43.0727	41.3292	55.0736	40.2052	33.971	47.2982
**TraesCS1D02G410100.1**	**TaMAPK33**	0.95091	1.11258	4.14187	4.7573	3.26301	3.07644	4.68439	3.38876	0.80345	0.76813	3.65716	2.91019	3.91623	5.25675
**TraesCS1D02G428900.1**	**TaMAPK34**	1.89667	1.69602	1.50406	1.84487	2.57524	2.70178	1.58173	1.06395	1.50273	1.50103	1.8226	1.443	1.42618	1.70925
**TraesCS6A02G099600.1**	**TaMAPK35**	0.51874	0.53596	0.3101	0.23908	0.78886	0.75821	0.09613	0.14483	0.36408	0.37313	0.37118	0.15077	0.2648	0.35862
**TraesCS6A02G118100.1**	**TaMAPK36**	1.13388	0.45369	0.21184	0.88075	0.50791	0.58227	0.52206	0.18516	0	0.10854	0.14207	0.45025	0.07588	0.13511
**TraesCS6A02G269400.1**	**TaMAPK37**	3.52737	3.98609	2.11914	2.23084	2.65294	2.47348	3.02621	2.4121	3.30345	3.49093	6.56911	4.81278	4.3483	6.05093
**TraesCS3B02G256700.1**	**TaMAPK38**	4.6966	5.38759	2.32133	2.04594	6.49266	6.918	1.29142	1.6659	1.89907	1.97591	2.38167	1.71959	2.16352	3.31616
**TraesCS3A02G242100.1**	**TaMAPK39**	12.4671	13.8946	8.88259	9.24868	9.25483	8.69729	7.64156	6.71677	20.7972	21.3127	16.2988	11.7206	9.24374	13.7131

**TraesCS3A02G231700.1**	**TaMAPK40**	0.3752	0.33016	0.3027	0.2201	0.42919	0.52147	0.31193	0.29772	0.32683	0.39243	0.37615	0.40505	0.3795	0.68424
**TraesCS1A02G402400.2**	**TaMAPK41**	0.40289	0	0	0	0	0	0	0	0	0	0.11868	0	0	0
**TraesCS1A02G415300.1**	**TaMAPK42**	0.07339	0	0	0	0.03259	0.0922	0	0.02428	0.1432	0.20452	0.17696	0.25845	0.15821	0.17444
**TraesCS1A02G421000.1**	**TaMAPK43**	0.82996	0.77403	0.95563	1.11445	1.76808	1.58475	1.01659	0.94738	2.261	2.18709	2.27788	1.79634	1.4595	1.77985
**TraesCS6D02G082900.2**	**TaMAPK44**	0.16112	0.07454	0.02309	0	0.1212	0.12676	0.02092	0.03161	0.037	0.05628	0.18376	0.03995	0.10932	0.26929
**TraesCS6D02G108100.1**	**TaMAPK45**	14.804	14.7754	5.92981	6.45907	19.5433	21.8583	7.97544	5.94758	8.45869	7.37898	11.8397	10.0048	7.62172	8.83977
**TraesCS7A02G049000.1**	**TaMAPK46**	0.18617	0.46169	0	0	0.46561	0.22121	0	0	0.22833	0	0	0	0	0.13147
**TraesCS7A02G029700.1**	**TaMAPK47**	0.04989	0	0	0	0.0222	0.09747	0	0.02476	0.0198	0.04476	0	0.02119	0.0344	0
**TraesCS7A02G410700.2**	**TaMAPK49**	4.26898	4.2015	12.033	10.45	10.745	8.19399	14.576	7.96733	3.77313	4.13073	21.7066	18.0642	9.66043	9.25449
**TraesCS5B02G536500.1**	**TaMAPK50**	2.80573	2.792	2.17274	1.96763	2.60119	2.58541	2.89455	2.29606	2.86623	2.99652	5.04365	4.16383	3.92986	5.4127
**TraesCS7B02G246900.3**	**TaMAPK52**	4.23252	4.57851	1.40634	1.29474	5.20338	5.30493	0.98536	0.97205	4.9182	4.74551	3.72055	2.37989	2.83742	4.16063
**TraesCS7B02G309900.1**	**TaMAPK53**	2.85226	2.62992	9.29011	9.58135	4.24272	4.04917	8.7766	7.1969	5.81906	5.4976	42.0679	32.0539	9.18617	13.6186

**TraesCS7B02G322900.1**	**TaMAPK54**	0.87103	0.97932	0.90343	1.01354	0.91602	1.00237	2.29356	1.97854	17.2182	16.582	15.6355	11.9364	4.90468	5.09804
**TraesCS6D02G328800.1**	**TaMAPKK1**	0.26629	0.34945	0.22523	0.33555	0.6079	0.757	1.41682	0.79399	1.2526	1.02375	4.59049	3.55217	1.87216	1.96624
**TraesCS5B02G565100.3**	**TaMAPKK2**	0.84445	0.50026	0.3594	0.25633	0.57414	0.84319	0.12836	0.29566	0.20103	0.18114	0.39747	0.30431	0.27313	0.45475
**TraesCS5D02G130900.2**	**TaMAPKK3**	0.1215	0.22894	0	0.06634	0.42473	0.31952	0.18507	0	0.093	0	0.043	0.13559	0.04376	0.23045
**TraesCS5A02G122700.4**	**TaMAPKK4**	0	0.18777	0	0	0.05426	0.3629	0	0	0	0	0.24274	0	0	0
**TraesCS4B02G049000.1**	**TaMAPKK5**	0	0	0	0	0	0	0	0	0	0	0	0	0	0
**TraesCS4B02G048100.1**	**TaMAPKK6**	0	0	0	0	0	0	0	0	0	0	0	0	0	0
**TraesCS4B02G048600.1**	**TaMAPKK7**	0	0	0	0	0	0	0	0	0	0	0	0	0	0
**TraesCS4B02G048900.1**	**TaMAPKK8**	0	0	0	0	0.02453	0	0	0	0	0	0	0	0	0
**TraesCS3B02G066300.1**	**TaMAPKK9**	0	0	0	0	0	0	0	0	0	0	0	0	0	0
**TraesCS4D02G048800.1**	**TaMAPKK11**	0	0	0	0	0	0	0	0	0	0	0	0.02351	0	0.01796
**TraesCS4D02G048500.1**	**TaMAPKK12**	0	0	0	0	0	0	0	0	0	0	0	0	0	0

**TraesCS5D02G549600.1**	**TaMAPKK13**	1.4285	1.08623	0.43273	0.57456	1.15162	1.15724	0.39329	0.2215	1.12809	0.97402	0.91868	0.67468	0.6426	0.85863
**TraesCS4A02G265900.1**	**TaMAPKK14**	0	0	0	0	0	0	0	0	0	0	0	0.04644	0	0
**TraesCS4A02G266000.1**	**TaMAPKK15**	0	0.02358	0.02297	0	0.04079	0.05359	0	0	0	0	0.08348	0.03881	0.0317	0.07495
**TraesCS4A02G266100.1**	**TaMAPKK16**	0	0.01679	0	0	0.02916	0.01268	0.01759	0.01592	0.01275	0	0.03563	0.01373	0.03437	0.06468
**TraesCS4A02G266200.1**	**TaMAPKK17**	0	0	0	0	0	0	0	0	0	0	0	0	0	0
**TraesCS5B02G122600.1**	**TaMAPKK18**	0.22857	0.38865	0.5181	0.20987	0.25588	0.35272	0.26193	0.09163	0.51424	0.26547	0	0.15297	0.09215	0.18051
**TraesCS2A02G407600.1**	**TaMAPKKK1**	2.95166	3.1499	0.70939	0.57702	3.48565	3.31548	0.79137	0.84885	1.4631	1.26065	2.27143	1.25669	1.36714	1.97031
**TraesCS4D02G027600.1**	**TaMAPKKK2**	0.00846	0.03622	0.22378	0.26093	0.03094	0.04001	0.11175	0.13909	0.02661	0.06112	0.18549	0.26641	0.1254	0.1958
**TraesCS4B02G210600.2**	**TaMAPKKK3**	2.80564	3.22986	1.11361	1.39338	2.62476	2.77854	0.89878	1.26365	5.03704	5.54443	4.73014	3.42137	2.59243	3.55501
**TraesCS6A02G245000.3**	**TaMAPKKK4**	0.62307	0.59135	0.371	0.41442	0.99573	0.86181	0.80747	0.62668	0.93455	0.98732	1.19488	0.90388	0.97676	1.33874
**TraesCS6B02G279300.1**	**TaMAPKKK4-1**	1.48528	1.47287	0.76028	0.76786	1.64736	2.04093	0.85856	0.76733	1.12824	1.07014	1.348	1.11436	1.0329	1.56196
**TraesCS2A02G199700.1**	**TaMAPKKK5**	2.34899	2.94583	3.48032	3.38813	7.70029	6.9214	4.46107	3.62434	1.32672	1.04253	12.3163	9.48559	3.73646	4.85665

**TraesCS3B02G289500.1**	**TaMAPKKK7**	0.0148	0.03083	2.21757	2.90414	0.05356	0.11627	3.56013	2.56524	0.51389	0.43997	0.25063	0.13847	0.06329	0.03965
**TraesCS3B02G288100.1**	**TaMAPKKK8**	0	0	0.04787	0.0656	0.07124	0.04954	0.49815	0.12436	0.08717	0.0142	0.04641	0.08048	0.05603	0.07377
**TraesCS3B02G288300.1**	**TaMAPKKK9**	0	0	0	0.0257	0.02173	0	0.0267	0.03229	0	0.02189	0.03562	0.06217	0	0.03735
**TraesCS4D02G211300.2**	**TaMAPKKK10**	2.11936	2.26382	0.84054	0.13872	2.41683	2.72473	1.10759	0.88326	3.8288	3.01133	3.39022	2.62115	1.51695	2.62477
**TraesCS5D02G475900.1**	**TaMAPKKK11**	12.5603	13.2346	22.68	23.35	19.0274	18.6883	21.8191	18.3273	6.28282	6.55711	10.7954	9.12894	3.40582	5.27303
**TraesCS4A02G093800.2**	**TaMAPKKK12**	7.25974	6.98022	1.38128	1.38264	6.00668	6.4917	1.86018	1.80312	4.51145	4.71248	4.32523	3.33251	4.01453	5.64931
**TraesCS5A02G118200.1**	**TaMAPKKK14**	0	0	0	0	0	0	0	0	0	0	0	0	0	0
**TraesCS5A02G463100.2**	**TaMAPKKK15**	2.14226	2.21302	2.81747	3.42899	3.91184	2.7717	3.21233	2.48363	1.31363	1.24381	3.68881	3.00342	1.48382	2.04732
**TraesCS5B02G474500.1**	**TaMAPKKK16**	7.3675	8.13186	19.216	21.471	12.8103	12.6426	16.2963	12.9086	10.2903	10.4042	9.79586	7.6715	3.19681	4.18913
**TraesCS5A02G200800.1**	**TaMAPKKK17**	0.016	0.01814	0	0	0.03088	0	9.40E-11	3.90E-10	0	0.05912	0	0	0	0
**TraesCS2B02G526200.3**	**TaMAPKKK18**	0	0.34004	0.19468	0.09461	0.10172	0.19012	0	0	0.20953	0.29801	0.16943	0.34713	0.26679	0.08739
**TraesCS2A02G498000.3**	**TaMAPKKK20**	1.08719	1.00178	0.78158	0.89191	0.73398	0.93592	0.6472	0.98187	0.68028	0.62625	3.08488	2.43553	1.72868	2.2504

**TraesCS6A02G149900.1**	**TaMAPKKK21**	0.16657	0.04676	0	0.02292	0.08994	0	0.07235	0.08722	0.58202	0.52172	0.39441	0.32351	0.25506	0.28667
**TraesCS5A02G392500.1**	**TaMAPKKK22**	0.7601	0.58024	0.29207	0.30806	0.64475	0.66001	0.43531	0.3089	0.76629	0.69898	1.11569	1.01564	0.88683	0.93256
**TraesCS6D02G139200.1**	**TaMAPKKK23**	0.02584	0	0	0	0.01172	0	0	0.0127	0.02904	0.08819	0.10594	0.04777	0.04632	0.02176
**TraesCS5B02G199400.1**	**TaMAPKKK24**	0	0	0	0	0	0	0	0	0.00929	9.43E-11	0.03976	0.04302	0	0
**TraesCS5B02G196400.1**	**TaMAPKKK25**	1.22451	0.99063	0.25574	0.37532	1.36751	1.25358	0.15854	0.09436	0.58637	0.5304	0.98308	0.92545	0.49395	0.74495
**TraesCS2D02G093700.1**	**TaMAPKKK26**	0.20579	0.31203	0.02693	0.0371	0.11214	0.10852	0.02234	0.03526	0.07591	0.04805	0.05386	0.05976	0.07859	0.08197
**TraesCS2B02G110500.1**	**TaMAPKKK27**	0.2436	0.29044	0.14075	0.09415	0.43193	0.28081	0.15672	0.17304	0.19477	0.25345	0.23275	0.18613	0.19664	0.32167
**TraesCS2A02G095300.1**	**TaMAPKKK28**	0.07039	0.06707	0.02701	0.0424	0.05522	0.05018	0.09653	0.06647	0.12912	0.10616	0.0677	0.12448	0.14254	0.15332
**TraesCS5D02G206500.1**	**TaMAPKKK29**	0	0	0	0	0	0	0	0	0	0	0	0	0	0
**TraesCS5D02G145100.1**	**TaMAPKKK30**	85.6204	68.9509	31.2419	37.3132	88.8787	89.7952	35.8657	20.8951	35.8742	32.4089	21.924	17.2075	15.0639	20.7541
**TraesCS6D02G236400.1**	**TaMAPKKK31**	0.11676	0.06122	0.07636	0.01294	0.01672	0.06066	0.08136	0.09803	0.31347	0.28367	0.39927	0.27528	0.33714	0.4655
**TraesCS2A02G195900.2**	**TaMAPKKK32**	2.52344	2.52717	1.29054	1.43314	3.07368	2.74186	3.38007	2.28611	2.52392	2.40763	4.76928	4.16436	4.90255	5.94954

**TraesCS6B02G270400.1**	**TaMAPKKK33**	0.14262	0.22873	0.0512	0.06036	0.21473	0.16835	0.05457	0.00822	0.07381	0.0606	0.11178	0.05711	0.15631	0.13547
**TraesCS2D02G197600.1**	**TaMAPKKK34**	1.0691	0.55699	4.52867	7.0015	2.02996	2.00328	8.66455	5.78854	3.60931	3.23168	8.18385	7.30094	0.12836	0.20744
**TraesCS2B02G223600.1**	**TaMAPKKK36**	3.66219	3.74196	1.15269	1.19271	3.4584	3.36586	2.83179	2.17899	7.15687	6.34714	5.75296	4.04936	4.22243	6.01412
**TraesCS2B02G216800.1**	**TaMAPKKK37**	3.31017	2.04273	29.4994	42.3796	2.67358	2.59689	30.1557	14.7691	3.4181	3.39264	22.4231	19.0127	0.30991	0.36422
**TraesCS1D02G026200.2**	**TaMAPKKK38**	0.7568	0.11732	0	0	0.4242	0.17879	0.19019	0.32198	0.26463	0.31866	0.38902	0.11016	0.31533	0.08102
**TraesCS6A02G255100.2**	**TaMAPKKK39**	0.09268	0.01242	0.00982	0.00185	0.00304	0.03523	0.03653	0.04127	0.07949	0.01076	0.07685	0.0732	0.07912	0.15078
**TraesCS5B02G146100.1**	**TaMAPKKK40**	10.8564	9.25424	5.76834	6.78848	12.7382	11.6772	9.94851	6.10198	10.7125	10.3458	10.6998	8.09682	8.06928	10.3538
**TraesCS6A02G172600.1**	**TaMAPKKK41**	0.10754	0.1548	0.67831	0.47399	0.13243	0.13891	1.06503	0.2204	0.02136	0	0.05878	0.02855	0.03684	0
**TraesCSU02G203100.1**	**TaMAPKKK42**	0	0	0	0	0	0	0	0	0	0	0	0	0	0
**TraesCS3B02G110300.1**	**TaMAPKKK43**	0.23945	0.06976	0.03411	0	0	0.05946	0	0.03421	0.05152	0	0.1149	0.10655	0.08702	0.14615
**TraesCS2D02G003900.1**	**TaMAPKKK44**	0	0	0	0	0	0	0	0	0	0	0	0	0	0
**TraesCS3D02G273200.1**	**TaMAPKKK45**	2.43647	2.1997	3.34843	3.61467	5.50032	5.34547	9.57479	8.07539	1.38536	1.37893	2.21519	1.62491	0.73621	0.93203

TraesCS2D02G050700.1	TaMAPKKK46	0.01557	0.02451	0.09509	0.12792	0.05699	0.0982	0.17717	0.09911	0.08567	0.07032	0.42092	0.35779	0.37492	0.34503
TraesCS7D02G022200.1	TaMAPKKK47	0	0	0	0	0	0	0	0	0	0	0	0	0	0
TraesCS7D02G079100.1	TaMAPKKK48	0.23672	0.15849	0	0.06893	0.19647	0.11903	0.04342	0.02618	0.10239	0.16278	0.31778	0.15187	0.2116	0.25256
TraesCS7D02G099200.2	TaMAPKKK50	4.10313	3.70457	0.40353	0.43919	6.3839	6.80607	0.62369	0.67724	1.07243	0.97957	9.34686	5.99207	7.28494	10.906
TraesCS7D02G230200.1	TaMAPKKK51	0.4365	0.35632	0.12128	0.12193	0.76423	0.73428	0.11654	0.06944	0.52448	0.48268	0.28417	0.29488	0.12011	0.12142
TraesCS7D02G230500.1	TaMAPKKK52	0.46321	0.44993	0.07811	0.09627	0.41862	0.47283	0.1681	0.07572	0.07312	0.06952	0.28402	0.22322	0.26352	0.22714
TraesCS1B02G372400.1	TaMAPKKK53	0.87391	1.06556	0.39259	0.47703	3.52223	3.58072	0.58016	0.47799	0.50742	0.59013	0.4839	0.40199	0.49959	0.5098
TraesCS7D02G503600.1	TaMAPKKK54	0	0	0	0	0	0	0	0.00753	0	0	0.0114	0.0131	0.01123	0
TraesCS3A02G039100.1	TaMAPKKK56	0.13957	0.10169	0	0.02825	0.24328	0.38574	0.15415	0.1073	0.24977	0.30524	1.83748	1.33946	0.64881	0.69696
TraesCS3D02G040600.1	TaMAPKKK57	0	0	0	0	0	0	0	0	0	0	0	0	0	0
TraesCS3B02G259800.1	TaMAPKKK58	0.97491	0.92203	0.32781	0.53834	1.48314	1.73635	0.47654	0.27643	0.8264	0.66004	2.12968	1.37318	1.46892	2.07762
TraesCS2A02G216900.1	TaMAPKKK59	0	0	0.00986	0.00999	0.00885	0	0	0.00951	0.03052	0	0.03586	0.03301	0.00703	0.02632

**TraesCS2A02G217000.1**	**TaMAPKKK60**	0.01016	0	0.01032	0.02094	0	0	0.03308	0	0.06396	0.01833	1.70449	1.21878	0.26471	0.32358
**TraesCS7D02G384700.1**	**TaMAPKKK61**	1.41644	1.42899	0.13669	0.10516	3.8911	3.80627	0.16976	0.15123	1.79832	1.97933	0.85743	0.50621	0.61796	0.77611
**TraesCS4A02G313900.1**	**TaMAPKKK62**	0	0	0	0	0	0	0	0	0	0	0	0.00488	0	0
**TraesCS4A02G383000.1**	**TaMAPKKK63**	0.05147	0.08057	0	0	0.0326	0.04047	0.01399	0.01265	0.03289	0.05799	0.12831	0.01152	0.07639	0.06062
**TraesCS4A02G465900.2**	**TaMAPKKK64**	0	0	0	0	0	0	0	0	0	0	0	0	0	0
**TraesCS4A02G464700.1**	**TaMAPKKK64-1**	0.08889	0.1542	0	0	0.08881	0.13645	0	0.04953	0.03959	0.02238	0.20029	0.10597	0.10319	0.11404
**TraesCS4A02G465000.1**	**TaMAPKKK65**	0.03249	0.00852	0.00826	0	0.052	0.01928	0.03278	0.00796	0.00639	0	0.02406	0.01382	0.01184	0.00555
**TraesCS1D02G273800.2**	**TaMAPKKK66**	0.08006	0.12332	0.29939	0.22601	0.15022	0.12404	0.14104	0.07142	0.78504	0.74489	3.6652	2.88276	1.05919	1.18647
**TraesCS1D02G360600.1**	**TaMAPKKK67**	0.39304	0.35583	0.16472	0.2532	2.63055	2.27179	0.57619	0.36219	0.05451	0.08352	0.11986	0.11805	0.24184	0.32582
**TraesCS1D02G431400.1**	**TaMAPKKK68**	0	0.00993	0.06758	0.03922	0.29254	0.19502	0.25434	0.21835	0.09672	0.12091	0.67571	0.42215	0.77051	0.89233
**TraesCS2D02G588200.1**	**TaMAPKKK69**	0.24021	0	1.63628	1.40156	0.08948	0.11531	1.17113	0.78922	0	0	0.1432	0.03882	0.0397	0.027
**TraesCS1D02G423800.1**	**TaMAPKKK70**	1.91931	1.69617	1.27247	1.62078	3.16768	3.06723	1.8489	1.06356	3.53494	3.74194	1.92088	1.10623	1.64582	2.14677

**TraesCS6A02G004500.1**	**TaMAPKKK71**	0	0	0	0	0	0	0	0	0.03165	0	0	0.03909	0	0
**TraesCS3A02G003900.1**	**TaMAPKKK72**	0.64138	0.61881	0.27906	0.41986	0.9006	0.87449	0.33903	0.28551	0.66909	0.55071	0.90694	0.66643	0.56422	0.7327
**TraesCS3A02G039200.1**	**TaMAPKKK73**	0.07853	0.05864	0	0	0.03062	0.01766	0	0.01102	0.03534	0.06071	0.729	0.55355	0.03236	0.06824
**TraesCS3A02G039400.1**	**TaMAPKKK74**	0	0	0	0	0	0.01754	0	0	0	0	0	0	0	0
**TraesCS3A02G096500.1**	**TaMAPKKK75**	0.22117	0.28695	0.1341	0.11512	0.24979	0.3224	0.16529	0.24256	1.39391	1.26809	0.83838	0.62178	0.35578	0.35061
**TraesCS3A02G246100.1**	**TaMAPKKK76**	0	0	0.00932	0.01887	0.08208	0.02888	0.01988	0.00899	0	0	0.02154	0.00803	0.03994	0.01971
**TraesCS3D02G501400.1**	**TaMAPKKK78**	0	0	0	0.01764	0.00766	0.00661	0.01897	0.0588	0.02636	0.05779	0.20517	0.18702	0.13894	0.13742
**TraesCS3A02G493900.1**	**TaMAPKKK79**	0.01614	0	0	0.05029	0.03452	0.01579	0.10034	0.05544	0.02371	0	0.34897	0.23238	0.11641	0.14963
**TraesCS2A02G217600.1**	**TaMAPKKK80**	0.19688	0.23732	0.09424	0.18065	0.44567	0.57172	0.05594	0.0708	0.08488	0.10231	0.35804	0.40638	0.17262	0.22568
**TraesCS5A02G292500.1**	**TaMAPKKK81**	0	0	0	0	0	0	0	0	0	0	0	0	0	0
**TraesCSU02G072500.1**	**TaMAPKKK82**	0	0	0	0	0	0	0	0	0	0	0	0	0	0
**TraesCS5A02G351500.1**	**TaMAPKKK83**	0	0	0	0	0	0	0	0	0	0	0	0	0	0

**TraesCS5A02G351000.1**	**TaMAPKKK84**	0	0	0	0	0	0	0	0	0	0	0	0	0	0
**TraesCS5A02G352000.1**	**TaMAPKKK85**	0	0	0	0	0	0	0	0	0	0	0	0	0	0
**TraesCS5D02G386800.1**	**TaMAPKKK86**	8.62561	6.75547	4.05397	4.20793	12.3849	11.6175	6.49278	4.05719	4.43276	4.2128	4.77906	3.41961	2.42781	3.36972
**TraesCS1A02G422800.1**	**TaMAPKKK88**	0	0	0.30396	0	0.42522	0.318	0	0.82093	0.20925	0.21578	3.21276	2.18828	1.98226	2.2712
**TraesCS7A02G152100.1**	**TaMAPKKK90**	0	0	0	0	0	0	0	0.02457	0	0	0	0	0	0
**TraesCS4D02G089300.1**	**TaMAPKKK91**	0.41006	0.50335	0.16897	0.05582	0.4248	0.62209	0.05933	0.1606	1.1614	1.09704	8.27991	6.30753	1.62198	2.08146
**TraesCS5B02G337300.1**	**TaMAPKKK92**	7.00148	8.07734	4.9548	5.24568	9.72469	9.60211	10.578	8.43876	6.05662	5.59606	10.7583	8.00647	9.10318	12.7884
**TraesCS5D02G018800.1**	**TaMAPKKK93**	0.88644	0.69727	0.04175	0.03373	0.60791	0.54335	0.08007	0.08843	0.4323	0.3928	0.55294	0.40509	0.22125	0.35774
**TraesCS5B02G012000.1**	**TaMAPKKK94**	7.32708	5.98234	0.95357	1.08694	28.9352	22.9126	2.16728	1.26321	7.41194	7.34099	6.64124	5.44529	8.00655	9.60441
**TraesCS5B02G204900.1**	**TaMAPKKK95**	1.08188	0.74391	2.49955	3.07212	1.34324	1.27314	2.19199	1.65722	2.76657	2.74523	4.43549	3.74259	0.8813	1.49211
**TraesCS5B02G292000.2**	**TaMAPKKK96**	0.29092	0.23839	0.06082	0	0.09405	0.22501	0	0	0	0.05525	0.08621	0.25184	0.03644	0.21825
**TraesCS5B02G353800.1**	**TaMAPKKK97**	0	0	0	0	0	0	0	0	0	0	0	0	0	0

**TraesCS5D02G482000.1**	**TaMAPKKK98**	4.74231	4.56576	12.2959	14.2296	4.07181	4.70544	13.0377	11.08	6.80238	7.02045	23.774	19.7878	9.55695	13.0233
**TraesCS3A02G001500.1**	**TaMAPKKK99**	1.70786	1.81275	0.29146	0.2849	2.24816	2.43482	1.55215	0.86069	4.43424	4.41477	3.99988	3.17627	3.34339	4.71911
**TraesCS3A02G274000.1**	**TaMAPKKK100**	3.20608	3.06695	2.46266	2.47077	6.44812	5.87213	5.62298	4.39283	1.82817	2.1272	5.50488	4.68964	1.7347	2.20892
**TraesCS5D02G019400.1**	**TaMAPKKK101**	5.43302	4.99033	1.51429	1.65934	21.2365	17.0822	4.05096	2.24142	8.35315	8.18604	9.18916	7.7869	8.93939	9.89305
**TraesCS7D02G474700.1**	**TaMAPKKK102**	3.24916	3.25969	1.66059	1.58951	4.30951	3.67804	2.12014	1.8193	3.82723	3.73805	4.82737	4.01616	3.29634	3.74187
**TraesCS7A02G326700.1**	**TaMAPKKK103**	1.56968	1.75478	0.24194	0.30612	1.58481	1.51786	0.41615	0.37125	0.81184	0.81675	0.91464	0.96256	1.03668	1.42182
**TraesCS1D02G273600.1**	**TaMAPKKK104**	0	0	0	0	0	0.10441	0.01398	0	0.00603	0	0	0	0	0
**TraesCS1B02G283400.1**	**TaMAPKKK104-1**	0.0614	0.04466	0.05198	0.09493	0.13057	0.06197	0.16659	0.06024	0.58294	0.56324	3.81542	3.38243	1.07729	1.52166
**TraesCS3A02G045200.1**	**TaMAPKKK105**	2.37358	2.37077	1.16227	1.31166	2.38588	2.31901	2.94864	1.70425	2.43348	2.75599	5.64181	4.76838	1.46062	2.00966
**TraesCS4A02G456900.1**	**TaMAPKKK106**	0	0	0	0	0	0	0	0	0	0	0	0	0	0
**TraesCS7A02G032700.1**	**TaMAPKKK107**	0	0	0	0	0	0	0	0	0	0	0	0	0	0
**TraesCS3D02G023600.1**	**TaMAPKKK108**	0	0	0	0	0	0	0	0	0	0	0	0	0.00608	0.01136

**TraesCS2D02G219800.1**	**TaMAPKKK109**	0.4262	0.42039	0.3439	0.28562	0.83379	0.71119	0.74341	0.48211	1.65817	1.81043	3.78708	2.99825	2.88728	3.4216
**TraesCS4B02G289100.1**	**TaMAPKKK110**	0.05523	0.02893	0.05615	0.04736	0.01681	0.06526	0.06988	0.03609	0.07964	0.07478	0.62642	0.39413	0.35424	0.34392
**TraesCS3A02G315100.1**	**TaMAPKKK111**	0.41988	0.43497	0.18603	0.16826	0.8096	0.67212	0.2042	0.17256	0.44913	0.47843	1.48027	1.08762	0.85998	1.34854
**TraesCS5D02G359500.1**	**TaMAPKKK112**	0	0	0	0	0	0	0	0	0	0.02253	0	0	0	0
**TraesCS3D02G108500.1**	**TaMAPKKK113**	0.13904	0.11847	3.78331	6.01423	0.20773	0.38144	9.15155	5.49174	0	0.01885	0.318	0.35752	0.44983	0.44206
**TraesCS3A02G229800.1**	**TaMAPKKK115**	0	0	0	0	0	0	0	0	0	0	0	0	0	0
**TraesCS1B02G454000.2**	**TaMAPKKK116**	0	0.00341	0	0.00719	0.02776	0.02628	0.00356	0.01695	0.01792	0	7.56E-09	0.05956	4.94E-08	0
**TraesCS3D02G097000.1**	**TaMAPKKK117**	0.34534	0.3697	0.16404	0.18719	0.38838	0.46085	0.40519	0.45193	2.42467	2.76027	1.31021	0.95755	0.39036	0.49077
**TraesCS2A02G577000.1**	**TaMAPKKK119**	0.26218	0.41025	1.09746	1.0219	0.04099	0.09408	0.26348	0.28715	2.13145	2.14525	0.32285	0.28127	0.20477	0.23516
**TraesCS4A02G317600.1**	**TaMAPKKK120**	1.46858	1.32984	0.86638	0.94202	1.47341	1.35683	0.29493	0.13328	0.53474	0.56463	3.13182	2.37577	1.41587	1.97744
**TraesCS2D02G066900.1**	**TaMAPKKK121**	0.24894	0.32399	0.02399	0.04216	0.31758	0.36157	0.05094	0.05752	0.19547	0.23396	1.60469	1.18537	0.74686	0.83618
**TraesCS2A02G214000.1**	**TaMAPKKK122**	0.02248	0.01175	0.13688	0.24363	0.10225	0.05306	0.42712	0.29053	0.06195	0.05068	0.57257	0.61348	0.32417	0.58589

**TraesCS5D02G358700.1**	**TaMAPKKK123**	0	0	0	0	0	0	0	0	0	0	0	0	0	0
**TraesCS5D02G358200.1**	**TaMAPKKK123-1**	0	0	0	0	0	0	0	0	0	0	0	0	0	0
**TraesCS7D02G153800.1**	**TaMAPKKK125**	0.02678	0.02806	0.00908	0.00918	0.02446	0.00703	0.03871	0.02781	0	0.04834	0.01981	0	0.01297	0.00607
**TraesCSU02G011500.1**	**TaMAPKKK126**	0	0	0	0	0	0	0	0	0	0	0	0	0	0
**TraesCS6D02G339600.1**	**TaMAPKKK127**	0	0	0	0	0	0	0	0	0	0	0	0.00589	0	0
**TraesCS1B02G446500.1**	**TaMAPKKK128**	0.24121	0.20506	0.10544	0.12208	0.59533	0.53415	0.176	0.10472	0.15128	0.19865	0.20567	0.17757	0.15213	0.42048
**TraesCS6B02G320800.1**	**TaMAPKKK129**	0.08121	0.13175	0.01503	0.00758	0.07436	0.09314	0.008	0.02891	0.06382	0.04096	0.63995	0.4838	0.81853	1.03294
**TraesCS7A02G044600.1**	**TaMAPKKK130**	0	0	0	0	0	0	0	0	0	0	0	0	0	0
**TraesCS6B02G215100.2**	**TaMAPKKK131**	1.92534	2.32343	1.65133	1.86876	2.81617	2.6634	1.53695	1.89744	1.69118	1.7388	2.76982	2.38548	3.05617	4.24127
**TraesCS5B02G353600.1**	**TaMAPKKK132**	0	0	0	0	0	0	0	0	0	0	0	0	0	0
**TraesCS1D02G004300.1**	**TaMAPKKK133**	2.56131	2.54671	1.08298	1.31171	5.72206	5.55753	1.29972	1.18712	2.36427	2.15406	2.45614	2.09492	1.87532	2.20017
**TraesCS5D02G547500.1**	**TaMAPKKK134**	0	0	0	0	0	0.00774	0	0	0	0	0.00726	0	0	0

**TraesCS2D02G598800.1**	**TaMAPKKK135**	0	0	0	0	0	0	0	0.01096	0.01758	0	0.00824	0	0	0
**TraesCS2B02G241600.1**	**TaMAPKKK136**	0.06166	0.08603	0.07307	0.03178	0.23412	0.27506	0.24535	0.09074	0.12132	0.09272	0.675	0.54206	0.3643	0.57085
**TraesCS3D02G472000.1**	**TaMAPKKK137**	0.25739	0.32565	1.62195	1.47226	0.46179	0.35077	2.0938	1.62917	0.19108	0.1159	1.19322	0.87867	0.66079	0.78402
**TraesCS3A02G493500.1**	**TaMAPKKK139**	0	0	0	0.01708	0.03047	0	0.03534	0	0	0.01489	0.06024	0.01373	0.07312	0.03369
**TraesCS5D02G097900.1**	**TaMAPKKK140**	1.23504	1.66278	0.51463	0.55156	2.04946	2.0126	1.24707	1.02752	1.14462	1.29091	2.80952	2.45627	2.96012	4.07955
**TraesCS7D02G000800.1**	**TaMAPKKK141**	0	0	0	0	0	0	0	0	0	0	0	0	0	0
**TraesCS2A02G032200.1**	**TaMAPKKK142**	0	0	0	0	0	0	0	0	0	0	0	0	0	0
**TraesCS3D02G501100.1**	**TaMAPKKK144**	0	4.06E-05	6.28E-05	0	0.0511	3.25E-05	0	0.00011	0	0.13066	0	4.73E-05	0	0
**TraesCS2B02G242300.1**	**TaMAPKKK145**	0	0	0	0	0	0	0.03738	0	0	0	0.00847	0.00976	0	0
**TraesCS2B02G241400.1**	**TaMAPKKK146**	0.60248	0.73194	0.72949	0.90637	4.77495	5.51763	1.38907	1.32402	2.79314	2.86358	11.9779	10.2665	4.15872	5.69921
**TraesCS2A02G216600.1**	**TaMAPKKK147**	0.43675	0.18354	0.37694	0.16683	0.9913	0.94122	0	0.26345	1.26619	0.45762	0.45409	0.31642	0.06958	0
**TraesCS1A02G003900.1**	**TaMAPKKK148**	0	0	0	0	0	0	0	0	0	0	0	0	0	0

**TraesCS3B02G008600.2**	**TaMAPKKK150**	1.77208	1.62286	6.54584	6.0289	3.88328	2.90688	11.5323	7.90162	1.00099	0.8964	0.93935	0.51635	0.40509	0.9369
**TraesCS3B02G123800.1**	**TaMAPKKK151**	0.27004	0.24059	0.15002	0.19715	0.31872	0.24242	0.54368	0.54371	0.61723	0.539	4.18164	3.26446	1.46764	1.66453
**TraesCS3B02G259100.1**	**TaMAPKKK152**	0	0	0	0.00874	0	0	0.00922	0.01667	0	0	0	0	0	0
**TraesCS3B02G351800.1**	**TaMAPKKK153**	0	0	0	0	0.02815	0	0	0	0	0	0	0	0.08629	0.06162
**TraesCS3B02G478400.1**	**TaMAPKKK154**	0	0	0	0	0	0	0	0	0	0	0	0	0	0
**TraesCS6B02G217100.1**	**TaMAPKKK155**	0.69852	0.78108	0.0314	0	0.73707	0.99453	0.236	0.06549	0.22604	0.25597	1.21791	0.81562	1.14207	1.57291
**TraesCS1A02G181900.1**	**TaMAPKKKK1**	1.62504	2.11758	0.91637	1.0585	1.79878	1.83868	1.81083	1.3964	2.54547	2.43345	2.82126	2.01443	2.18498	2.79802
**TraesCS1B02G199100.2**	**TaMAPKKKK2**	0	0.13191	0.16029	0.1407	0.16915	0	0.48077	0.18765	0	0	0	0	0	0
**TraesCS1D02G185000.2**	**TaMAPKKKK3**	0	0	0.12277	0	0.03953	0.07642	0.0499	0	0.16435	0.03991	0.13696	0.04491	0	0.04216
**TraesCS2A02G233400.1**	**TaMAPKKKK4**	1.86152	2.11298	10.2224	9.82802	3.98018	4.1295	14.8158	15.318	1.50253	1.55684	2.25338	1.58938	1.53512	2.11029
**TraesCS2B02G249900.1**	**TaMAPKKKK5**	1.55142	2.10435	0.74058	0.80034	1.87058	1.87486	1.00206	1.08793	1.9831	1.56232	2.70628	1.97032	1.67978	2.38912
**TraesCS2D02G232200.1**	**TaMAPKKKK6**	0.90934	1.3684	0.51578	0.36557	0.99944	1.19529	0.78229	0.61027	1.19426	0.99193	1.6943	1.37059	1.23345	1.31221

**TraesCS4B02G395600.1**	**TaMAPKKKK7**	0.02491	0.01299	0.01261	0.01286	0	0.01958	0.02708	0	0	0	0.01852	0.01061	0.02738	0.03374
**TraesCS4B02G398400.3**	**TaMAPKKKK8**	10.8455	11.352	2.95191	2.90618	10.279	9.69322	4.07049	3.64961	6.40615	5.63819	5.92384	4.78263	4.38146	5.61746
**TraesCS5A02G187400.1**	**TaMAPKKKK9**	0.59195	0.31586	0.12411	0.19138	0.85462	0.76518	0.30106	0.1716	0.53685	0.54269	1.0049	0.90888	0.81647	1.06739
**TraesCS5A02G392500.1**	**TaMAPKKKK10**	0.7601	0.58024	0.29207	0.30806	0.64475	0.66001	0.43531	0.3089	0.76629	0.69898	1.11569	1.01564	0.88683	0.93256
**TraesCS5A02G556400.5**	**TaMAPKKKK11**	2.291	1.80467	0.89871	0.76402	1.95998	2.11002	0.32158	0.28028	0.59176	0.7218	0.78317	0.4944	0.47644	0.45092
**TraesCS5B02G397300.1**	**TaMAPKKKK12**	0.53629	0.53127	0.31022	0.27832	0.59363	0.57389	0.45787	0.32331	0.59828	0.66049	1.29169	0.8702	0.69796	1.07908
**TraesCS5D02G203600.1**	**TaMAPKKKK13**	0.98052	1.00271	0.63288	0.94447	1.70779	1.73185	0.56178	0.46077	1.03268	0.82323	2.13495	1.6657	1.14092	1.562
**TraesCS5D02G402300.1**	**TaMAPKKKK14**	0.56409	0.52604	0.27717	0.2854	0.67374	0.69601	0.39938	0.35014	0.87105	0.71165	1.28211	1.1839	0.88425	1.17366
**TraesCS6A02G149900.1**	**TaMAPKKKK15**	0.16657	0.04676	0	0.02292	0.08994	0	0.07235	0.08722	0.58202	0.52172	0.39441	0.32351	0.25506	0.28667
**TraesCS6A02G353400.1**	**TaMAPKKKK16**	0	0	0	0.00832	0	0	0.02865	0	0	0	0.01982	0	0	0.03586
**TraesCS6A02G353500.1**	**TaMAPKKKK17**	0	0	0	0.01247	0	0	0	0	0	0	0	0.01089	0	0
**TraesCS6B02G177800.1**	**TaMAPKKKK18**	0	0	0	0	0	0.11377	0.0511	0	0.19455	0.04878	0.18413	0.10482	0.09224	0.13239

**TraesCS6B02G386100.1**	**TaMAPKKKK19**	0	0	0	0	0	0	0	0.05847	0	0	6.96E-05	0	0.03412	0.03117
**TraesCS6D02G335800.1**	**TaMAPKKKK20**	0.11115	0.05378	0.19782	0.18328	0	0.16169	0.2688	0.28607	0.29463	0.26156	0.35259	0.25477	0.45652	0.46012
**TraesCS6D02G139200.1**	**TaMAPKKKK21**	0.02584	0	0	0	0.01172	0	0	0.0127	0.02904	0.08819	0.10594	0.04777	0.04632	0.02176
**TraesCS7A02G232300.1**	**TaMAPKKKK22**	2.87128	3.26008	2.06441	1.78999	3.90491	4.15381	1.86117	1.9852	3.45884	3.09586	4.34205	3.31826	3.09631	3.86221
**TraesCS7B02G130700.1**	**TaMAPKKKK23**	4.34625	4.42757	2.36408	2.30354	5.00861	4.8075	1.96832	2.05646	4.84156	4.50334	5.84287	4.61368	4.43647	5.80512
**TraesCS7D02G232400.1**	**TaMAPKKKK24**	1.13985	1.14621	0.7667	0.64502	1.92786	1.66159	0.73727	0.49652	1.32838	1.36351	2.50593	1.88067	1.61911	1.92075
**TraesCSU02G115300.1**	**TaMAPKKKK25**	2.58611	2.87015	0.6063	1.41354	2.65969	1.3235	1.38767	0.85941	0.7496	0.81622	2.01977	2.15227	0.50453	1.86912

Table S5 The expression profile data of MAPK-MAP4Ks.

Locus ID	gene name	grain_Z71_rep1	grain_Z71_rep2	grain_Z75_rep1	grain_Z75_rep2	grain_Z85_rep1	grain_Z85_rep2	leaf_Z10_rep1	leaf_Z10_rep2	leaf_Z23_rep1	leaf_Z23_rep2	leaf_Z71_rep1	leaf_Z71_rep2	root_Z10_rep1	root_Z10_rep2
**TraesCS6B02G296700.2**	**TaMAPK1**	24.4108	18.2798	0.61531	1.07984	13.9459	10.0219	11.142	13.5966	16.4973	20.7801	8.54232	6.02991	16.6236	20.5462
**TraesCS4A02G336800.2**	**TaMAPK2**	8.92267	6.83392	0.36245	0.23007	1.10328	1.14248	4.20125	1.85965	4.08424	5.03315	3.05025	2.11314	4.54081	5.05101
**TraesCS4A02G106400.1**	**TaMAPK3**	21.3555	20.3193	3.17351	3.12641	2.28869	2.50991	7.53268	10.4741	30.2178	28.3146	45.9295	42.2849	59.2289	55.2361
**TraesCS1D02G088000.2**	**TaMAPK4**	0	0	0	0	0	0.17562	0	0	0	0	0	0	0	0
**TraesCS1D02G422800.1**	**TaMAPK5**	0	0.23106	0.05498	0.01662	0	0	0.58104	0.60318	0.47781	0.51079	0	0.10952	1.77232	1.14527
**TraesCS7B02G009200.1**	**TaMAPK6**	20.5689	16.6073	2.57137	2.74313	9.82822	8.57782	11.1836	10.9977	10.9509	11.5053	8.76512	7.50133	15.2859	15.9999
**TraesCS7D02G342800.3**	**TaMAPK7**	8.60731	5.41097	1.30939	1.07319	8.87654	10.1143	3.16595	4.51884	2.74175	3.44316	2.98436	2.77692	6.19151	7.08251
**TraesCS3D02G225600.1**	**TaMAPK8**	10.7137	8.75814	0.62243	0.6643	7.44689	8.04799	4.23561	5.44132	4.54847	5.59256	1.48717	1.42698	8.32236	8.1398
**TraesCS6B02G146300.1**	**TaMAPK10**	18.4335	13.6394	3.20426	3.22607	6.94167	9.64951	9.28622	13.8863	9.22067	11.0656	16.7953	13.4871	17.4774	18.52
**TraesCS1A02G086500.1**	**TaMAPK11**	12.8121	10.2356	1.99606	1.84006	4.47383	4.69139	5.2771	5.49469	5.50953	6.62221	5.22618	4.85364	10.616	10.5671

**TraesCS7A02G422500.1**	**TaMAPK12**	29.6771	25.6298	2.96297	2.82358	5.19904	5.50976	5.12509	6.45095	27.4061	29.6161	32.7462	31.7244	26.1714	24.5724
**TraesCS6D02G245500.3**	**TaMAPK13**	33.5378	24.6461	1.34139	1.8994	20.6173	22.3282	6.53845	13.9472	14.7489	19.0317	5.5225	8.71895	15.7334	25.4527
**TraesCS1A02G184500.1**	**TaMAPK14**	4.2521	4.04878	0.66599	0.57822	2.13001	2.22837	4.49362	5.20652	5.9231	6.65699	5.70827	4.83869	9.40606	9.18744
**TraesCS3B02G270200.1**	**TaMAPK16**	11.1753	8.13023	0.58925	0.59799	0.71078	0.7682	5.73943	6.09576	8.31498	10.5884	3.61948	3.38482	13.3213	14.4698
**TraesCS6B02G127800.1**	**TaMAPK17**	0.60389	0.2953	1.083	1.06043	0.819	0.70023	0.51143	0.74152	0.41557	0.48546	0.21989	0.11025	0.68097	0.56471
**TraesCS7D02G044100.1**	**TaMAPK18**	33.2428	27.6054	8.33467	7.88306	28.099	37.5967	14.3107	23.5606	27.9787	29.0474	22.8587	22.2115	106.637	113.635
**TraesCS7A02G111300.1**	**TaMAPK19**	18.8547	14.9183	4.31642	3.67391	7.0426	7.34511	12.2515	13.6982	11.4215	12.7252	9.45014	9.10178	19.3921	19.1353
**TraesCS7D02G403700.1**	**TaMAPK20**	3.11329	3.96929	0.65968	0.63587	0.19671	0	0.70744	1.65627	4.20388	4.61771	2.19633	3.07653	3.91603	4.30549
**TraesCS7D02G414900.1**	**TaMAPK22**	1.24048	1.08416	0.22017	0.13985	0.24788	0.24669	0	0.03832	0.05419	0.06245	0.33598	0.2137	0.25221	0.16403
**TraesCS3D02G221700.1**	**TaMAPK23**	2.36118	1.93581	0.07175	0.09719	2.63888	2.77886	1.17186	1.24163	0.61368	0.57397	0.79886	0.77835	1.4628	1.55693
**TraesCS3D02G242200.2**	**TaMAPK24**	6.94279	5.85862	0.52798	0.55959	1.0201	1.15541	6.11051	9.97922	8.93664	10.1875	5.04361	5.25339	10.8371	13.1103
**TraesCS4D02G198600.1**	**TaMAPK25**	22.2729	21.3672	2.46341	2.03852	2.25425	3.46337	8.57298	10.584	47.3954	45.4849	60.7443	58.9115	60.0802	57.118

**TraesCS5D02G534000.2**	**TaMAPK26**	1.37636	0.82281	0	0	0.16345	0.55456	0.17258	0.67698	0.88757	0.18371	0.29226	0	0.80615	0.84788
**TraesCS1B02G104900.1**	**TaMAPK27**	8.01739	6.21115	1.65215	1.66301	5.21658	5.63829	4.87328	6.52552	5.67313	6.29126	6.52998	5.62767	11.2066	11.1558
**TraesCS1B02G192600.3**	**TaMAPK28**	8.11931	5.63032	1.41354	1.40239	3.54922	3.53752	9.21038	11.8714	13.3909	15.2318	14.9928	13.7273	21.284	20.1527
**TraesCS1B02G431400.2**	**TaMAPK29**	0	1.16448	7.96723	7.38679	1.92871	3.68807	0	1.02E-08	0	0	0	2.19E-06	0	0.00358
**TraesCS7A02G335300.2**	**TaMAPK30**	1.98227	1.50666	0.30223	0.94259	2.14211	2.09495	0.28659	1.86537	6.44E-06	1.65E-09	0	0.38293	2.09326	0.86134
**TraesCS4A02G434800.1**	**TaMAPK31**	37.1206	29.4098	6.64643	6.41026	24.5694	28.0097	19.8967	25.8702	34.7553	40.0466	24.0043	22.661	86.3673	90.2965
**TraesCS1D02G410100.1**	**TaMAPK33**	26.6062	22.7873	2.39683	2.382	10.1892	11.5303	12.6991	18.0488	25.1851	26.3399	0.91019	0.53648	22.0251	23.4647
**TraesCS1D02G428900.1**	**TaMAPK34**	11.4807	9.40497	1.21851	0.9519	1.52258	1.45662	5.06587	5.8041	5.28603	6.43792	7.50665	6.93921	15.4448	15.0399
**TraesCS6A02G099600.1**	**TaMAPK35**	0.63887	0.41254	2.41155	2.18999	1.3446	0.975	0.34516	0.33885	0.42215	0.31095	0.34916	0.50219	0.62353	0.74022
**TraesCS6A02G118100.1**	**TaMAPK36**	1.3753	0.44134	0.07446	0.06822	1.26551	0.17686	0.10484	0.79046	0.30391	0.20826	0	0	1.21496	0.34452
**TraesCS6A02G269400.1**	**TaMAPK37**	26.1507	20.5706	2.09788	1.55947	18.49	20.2267	10.6613	14.7988	17.6558	21.0793	10.3974	9.2032	26.4	26.9177
**TraesCS3B02G256700.1**	**TaMAPK38**	5.12743	3.50662	0.36871	0.33047	5.42377	4.4376	1.67841	2.60153	3.17613	3.78062	1.31615	0.9651	5.20825	5.56444

**TraesCS3A02G242100.1**	**TaMAPK39**	17.1361	10.8036	0.55149	0.49624	1.97364	2.16782	5.77238	11.5286	12.6882	13.4176	7.75854	7.13522	16.0331	16.6173
**TraesCS3A02G231700.1**	**TaMAPK40**	1.05532	0.82364	0.01741	0.04714	0.34248	0.38254	0.17706	0.13682	0.2653	0.22644	0.26333	0.30616	0.67361	0.49321
**TraesCS1A02G402400.2**	**TaMAPK41**	0.92585	1.47717	7.77735	7.34089	0.22812	0	0.28215	0.83411	0.99867	0.83869	0	0	0	0.5862
**TraesCS1A02G415300.1**	**TaMAPK42**	0	0.08273	0	0.06346	0	0	1.26867	1.33715	1.04253	0.44337	0.07567	0	2.31725	1.59044
**TraesCS1A02G421000.1**	**TaMAPK43**	20.5566	17.3681	1.04052	0.91519	1.71602	1.90335	6.33271	7.2999	6.79957	7.67153	5.38904	4.84162	16.0454	16.3349
**TraesCS6D02G082900.2**	**TaMAPK44**	0.03203	0	0.99812	1.19173	0.1726	0.07236	0.06296	0.02895	0.46789	0.2794	0.49238	0.87078	0.04015	0.07563
**TraesCS6D02G108100.1**	**TaMAPK45**	16.0884	11.6068	2.13492	2.12899	5.80279	8.09188	6.21092	7.90734	5.77277	7.8933	11.6375	10.1107	13.6191	12.0754
**TraesCS7A02G049000.1**	**TaMAPK46**	8.55907	7.28036	1.73392	0.97897	8.18916	5.25697	4.33672	2.35018	6.89166	7.19077	3.84691	4.31197	2.28244	8.88081
**TraesCS7A02G029700.1**	**TaMAPK47**	0.03121	0	0	0	0	0	0	0	0	0	0.03013	0.03798	0	0
**TraesCS7A02G410700.2**	**TaMAPK49**	21.5791	20.0382	3.51649	3.02103	2.67142	1.93742	3.27595	3.91124	13.5825	12.779	20.137	18.4074	19.4592	17.6976
**TraesCS5B02G536500.1**	**TaMAPK50**	24.9451	19.5727	1.02854	1.08844	1.93162	2.25525	5.37792	5.77288	6.32381	8.02527	5.37952	5.0492	10.1896	11.3577
**TraesCS7B02G246900.3**	**TaMAPK52**	5.87491	4.64332	0.9177	0.93156	7.51252	7.23931	2.74299	3.22119	2.25912	2.70103	1.50278	1.39424	4.93037	5.02366

**TraesCS7B02G309900.1**	**TaMAPK53**	88.1217	68.4065	18.249	15.744	5.31442	6.13227	16.2192	19.6957	79.5443	88.8381	99.6791	98.61	105.556	106.447
**TraesCS7B02G322900.1**	**TaMAPK54**	13.7707	12.4374	2.26827	2.29116	3.75945	4.16632	2.03287	2.13678	11.9547	14.0399	16.3686	16.38	16.786	18.0564
**TraesCS6D02G328800.1**	**TaMAPKK1**	6.91179	7.6271	0.5506	0.58448	2.12781	1.44832	3.99169	1.99874	6.04786	5.29396	2.60024	2.62553	7.53843	5.14681
**TraesCS5B02G565100.3**	**TaMAPKK2**	0.14474	0.08512	0.09182	0.10668	0.21938	0.66847	0	0.23442	0.10443	0.08455	0	0	0.15765	0.18389
**TraesCS5D02G130900.2**	**TaMAPKK3**	0.25736	0.25786	0	6.65E-08	0.73442	1.67051	0.17321	0.63027	0.5339	0.05	0	0.61817	0.61937	0.74701
**TraesCS5A02G122700.4**	**TaMAPKK4**	0	0	0	0	0.51749	0.90832	0	0	0.29952	0	0.3184	0	0	0
**TraesCS4B02G049000.1**	**TaMAPKK5**	0	0	0	0	0	0	0	0	0	0	0	0	0	0
**TraesCS4B02G048100.1**	**TaMAPKK6**	0	0	0	0	0	0	0	0	0	0	0	0	0	0
**TraesCS4B02G048600.1**	**TaMAPKK7**	0	0	0	0	0	0	0	0	0.05601	0	0	0	0	0
**TraesCS4B02G048900.1**	**TaMAPKK8**	0.13666	0.03856	0	0	0	0	0	0	0	0	0	0	0.06916	0.09856
**TraesCS3B02G066300.1**	**TaMAPKK9**	0	0	0	0	0	0	0	0	0	0	0	0	0	0
**TraesCS4D02G048800.1**	**TaMAPKK11**	0	0	0	0	0	0	0.03366	0	0	0	0	0	0.10374	0

**TraesCS 4D02G0 48500.1**	**TaMAP KK12**	0	0	0	0	0	0	0	0	0	0	0	0	0	0
**TraesCS 5D02G5 49600.1**	**TaMAP KK13**	4.29793	2.27704	0.64701	0.55724	2.25353	2.33976	1.18381	1.62496	2.14455	2.01219	1.69744	1.37434	2.66849	2.80538
**TraesCS 4A02G2 65900.1**	**TaMAP KK14**	0	0	0	0	0	0	0	0	0	0	0	0	0	0
**TraesCS 4A02G2 66000.1**	**TaMAP KK15**	0	0	0	0	0	0	0.02853	0	0	0	0	0	0.12204	0.13869
**TraesCS 4A02G2 66100.1**	**TaMAP KK16**	0	0	0	0	0	0	0	0	0	0	0	0	0.1434	0.0811
**TraesCS 4A02G2 66200.1**	**TaMAP KK17**	0	0	0	0	0	0	0	0	0	0	0	0	0	0
**TraesCS 5B02G1 22600.1**	**TaMAP KK18**	0.68029	0.65457	0.00246	0.01802	2.31229	3.10588	0.49587	0.82718	0.27317	0.3805	0.30593	0.33925	0.41242	0.46941
**TraesCS 2A02G4 07600.1**	**TaMAP KKK1**	2.35174	1.88039	0.33788	0.25596	2.15925	2.98777	1.10483	1.71051	1.65506	1.86873	1.48841	1.41758	3.10411	3.40259
**TraesCS 4D02G0 27600.1**	**TaMAP KKK2**	0.01153	0.07805	0.00608	0.01758	0	0	0.47405	0.27864	0.7237	0.78453	0.35406	0.35375	2.04002	1.27056
**TraesCS 4B02G2 10600.2**	**TaMAP KKK3**	5.69314	4.86494	1.15456	0.74299	9.00142	8.10682	2.24118	2.8522	3.69646	3.54357	4.696	4.71112	4.17251	4.23056
**TraesCS 6A02G2 45000.3**	**TaMAP KKK4**	4.10394	3.595	0.73318	0.58149	1.48813	1.47595	2.4135	2.33779	1.47386	2.0824	1.27535	1.26692	4.05505	3.95451
**TraesCS 6B02G2 79300.1**	**TaMAP KKK4-1**	4.50905	4.14894	1.0288	0.80005	3.14617	3.69181	1.62378	2.24084	1.35569	1.76678	0.98482	1.3221	2.8807	3.24199

**TraesCS2A02G199700.1**	**TaMAPKKK5**	75.602	60.1258	4.09574	2.88322	4.86827	4.42538	9.38417	10.5375	51.9077	63.1839	46.4819	43.5181	52.3298	55.3493
**TraesCS3B02G289500.1**	**TaMAPKKK7**	7.51801	8.50026	0.2857	0.26679	0	0.02019	2.91711	1.38952	5.51597	5.37164	3.10806	3.5224	6.55672	6.44169
**TraesCS3B02G288100.1**	**TaMAPKKK8**	6.88357	8.80792	0.45385	0.17531	0.01833	0	3.11613	1.84471	15.9909	16.0437	2.93322	3.10185	4.77258	2.78701
**TraesCS3B02G288300.1**	**TaMAPKKK9**	5.507	7.50469	0.21283	0.16671	0.1363	0.03189	2.15553	1.11933	10.5797	9.99206	1.02239	1.47239	2.81208	2.50142
**TraesCS4D02G211300.2**	**TaMAPKKK10**	3.71133	3.26215	0.5339	0.46183	5.36661	3.74851	0.75756	0.77318	2.37329	2.97444	3.61218	3.69995	1.78158	2.8203
**TraesCS5D02G475900.1**	**TaMAPKKK11**	14.2653	12.458	1.77172	1.81622	6.03408	6.50953	2.04141	2.8935	4.19042	4.81538	11.2977	11.0184	12.8364	13.1886
**TraesCS4A02G093800.2**	**TaMAPKKK12**	4.71409	4.05759	1.12309	1.15958	10.0147	10.8669	2.10535	2.7136	2.851	4.00701	5.38596	5.09952	4.82465	4.69392
**TraesCS5A02G118200.1**	**TaMAPKKK14**	0.57274	0.40958	0.00779	0	0	0	0	0	0.01236	0	0	0	0.01958	0.01724
**TraesCS5A02G463100.2**	**TaMAPKKK15**	4.02595	5.24187	0.63387	0.43493	1.44456	1.64272	0.94048	1.44922	2.18147	2.8053	2.8002	2.84668	3.37647	3.24116
**TraesCS5B02G474500.1**	**TaMAPKKK16**	14.3485	13.8914	1.6091	1.48336	3.60566	3.48611	2.91344	3.32762	6.93994	7.90799	17.1147	16.6678	14.2411	15.2137
**TraesCS5A02G200800.1**	**TaMAPKKK17**	4.78737	4.2042	0.42432	0.35805	6.74E-10	0.17073	3.42787	3.84077	1.53946	1.72752	0	0.04781	8.00209	8.57009
**TraesCS2B02G526200.3**	**TaMAPKKK18**	0.44461	0.20204	1.19E-10	0.32536	0.33791	0.28457	0.17921	0.97267	0.85071	0.86697	0.56525	1.23087	1.41885	1.13299

**TraesCS2A02G498000.3**	**TaMAPKKK20**	3.20797	1.79821	0.79956	1.07742	1.70347	2.172	1.4243	1.84587	3.07766	2.47634	4.61248	2.326	4.51206	4.47563
**TraesCS6A02G149900.1**	**TaMAPKKK21**	0.01491	0	0	0	0	0	0.20916	0.51871	0.08155	0.06032	0	0	0	0
**TraesCS5A02G392500.1**	**TaMAPKKK22**	2.8722	2.65297	0.9929	0.98402	0.69559	0.71462	2.0858	2.61465	1.79127	1.79001	1.37183	1.32328	3.34958	3.58375
**TraesCS6D02G139200.1**	**TaMAPKKK23**	0.01718	0.01939	0	0.00813	0	0.01777	0.29641	0.44582	0.20665	0.38949	0.11671	0.10457	0	0
**TraesCS5B02G199400.1**	**TaMAPKKK24**	3.28821	2.93767	0.24729	0.3374	0.25548	0.36205	2.38569	2.59669	1.26638	1.64795	0	0.03158	6.15245	6.02918
**TraesCS5B02G196400.1**	**TaMAPKKK25**	0.14441	0	0	0	0	0	0.12281	0.17517	0.42101	0.44867	0.37712	0.58057	0	0
**TraesCS2D02G093700.1**	**TaMAPKKK26**	3.74786	3.12338	0.18037	0.16359	0.43393	0.27281	1.72835	1.49211	1.29068	1.55037	0.16603	0.06784	3.41781	3.53014
**TraesCS2B02G110500.1**	**TaMAPKKK27**	4.07051	3.39519	0.1717	0.14141	3.2664	3.20587	1.21616	1.3566	1.1188	1.17164	0.30742	0.35167	3.54698	3.52324
**TraesCS2A02G095300.1**	**TaMAPKKK28**	3.18705	2.48468	0.1935	0.11797	0.98105	0.94226	1.14681	1.34419	1.02177	1.36532	0.12055	0.15312	3.01986	3.08257
**TraesCS5D02G206500.1**	**TaMAPKKK29**	4.58349	3.20572	0.261	0.28067	1.53E-10	1.42E-10	3.22232	3.71632	1.52816	1.8422	0	0	7.86587	7.56048
**TraesCS5D02G145100.1**	**TaMAPKKK30**	1.00668	1.20584	0.48835	0.44176	11.3585	13.6861	1.28374	1.60318	6.84991	6.03831	36.3768	35.6926	5.66349	4.94882
**TraesCS6D02G236400.1**	**TaMAPKKK31**	1.60911	1.64189	0.43379	0.4306	0.36475	0.26763	1.25351	1.21139	0.68951	0.8343	0.21331	0.23451	0.96546	1.02575

**TraesCS2A02G195900.2**	**TaMAPKKK32**	7.8317	6.80864	4.12605	3.69463	11.2165	15.1163	9.11636	12.367	6.10787	7.80143	2.98725	3.00183	19.6101	21.0264
**TraesCS6B02G270400.1**	**TaMAPKKK33**	3.10731	2.18732	0.09877	0.09955	0.12333	0.05129	1.64309	1.38671	0.81009	1.19715	1.12914	0.81779	0.29239	0.35434
**TraesCS2D02G197600.1**	**TaMAPKKK34**	3.53481	4.53489	2.17238	1.77715	0.41401	0.42059	0.63951	0.58887	0.31627	0.40665	0.32656	0.32781	9.58534	8.04465
**TraesCS2B02G223600.1**	**TaMAPKKK36**	8.9444	7.45825	3.40428	3.15649	7.36075	8.91951	6.81383	10.6064	5.30304	5.36642	3.79457	3.75189	12.2586	12.2802
**TraesCS2B02G216800.1**	**TaMAPKKK37**	3.31017	2.04273	29.4994	42.3796	2.67358	2.59689	30.1557	14.7691	3.4181	3.39264	22.4231	19.0127	0.30991	0.36422
**TraesCS1D02G026200.2**	**TaMAPKKK38**	0.96568	0.01192	0	0	0.39958	0.11429	0.14013	0.11098	0	0	0.19183	0	0.64058	0.63435
**TraesCS6A02G255100.2**	**TaMAPKKK39**	1.74022	1.47047	0.34115	0.23157	0.06505	0.11593	0.67173	0.98343	0.35974	0.54489	0.15737	0.08762	0.6592	0.732
**TraesCS5B02G146100.1**	**TaMAPKKK40**	0.75462	0.66387	0.17576	0.13766	1.09354	1.83625	1.26347	1.37777	2.52451	2.78511	11.3043	10.8743	6.1067	5.17908
**TraesCS6A02G172600.1**	**TaMAPKKK41**	0	0	0	0	0	0	0.06567	0	0.02761	0.15743	0	0.04055	0	0
**TraesCSU02G203100.1**	**TaMAPKKK42**	0	0	0	0	0	0	0	0	0	0	0	0.05147	0	0
**TraesCS3B02G110300.1**	**TaMAPKKK43**	0.08186	0.09237	0	0	0	0	0.12044	0	0	0.04047	7.41E-10	0	0.13767	0.03939
**TraesCS2D02G003900.1**	**TaMAPKKK44**	0	0	0	0	0.01367	0	0	0	0	0	0	0	0	0

**TraesCS3D02G273200.1**	**TaMAPKKK45**	3.14383	3.37244	1.0329	0.92159	4.83344	5.31382	0.40775	0.65886	1.196	0.97715	2.2432	1.79617	12.0578	12.5461
**TraesCS2D02G050700.1**	**TaMAPKKK46**	0	0	0	0	0	0	0	0	0.17784	0.19656	0.78669	0.77726	0	0
**TraesCS7D02G022200.1**	**TaMAPKKK47**	0.78357	0.67906	0.12384	0.0229	0.38933	0.28132	0.31148	0.35519	0.13834	0.13962	0.06901	0.02893	0.48343	0.57822
**TraesCS7D02G079100.1**	**TaMAPKKK48**	0.53595	0.59393	0.12849	0.08804	0.01564	0.07311	0.17666	0.27549	0.29801	0.15518	0.36529	0.3441	0.313	0.23178
**TraesCS7D02G099200.2**	**TaMAPKKK50**	0	0	0	0	0	0	0	0.01415	0.82171	0.81504	2.03274	1.68136	0	0
**TraesCS7D02G230200.1**	**TaMAPKKK51**	4.99257	4.499	0.05035	0.01692	0.04753	0.02727	2.44957	1.58453	1.57502	1.72997	0.45103	0.64943	1.28717	1.07893
**TraesCS7D02G230500.1**	**TaMAPKKK52**	0	0	0	0	0	0	0	0	0.08438	0.11885	0.29835	0.09868	0	0
**TraesCS1B02G372400.1**	**TaMAPKKK53**	0.26894	0.19428	0.05113	0.05135	0.01898	0	1.18205	1.07535	0.23366	0.15685	0.07323	0.10475	9.84023	8.63609
**TraesCS7D02G503600.1**	**TaMAPKKK54**	0	0	0.00556	0.00502	0	0	0	0	0.02638	0.02046	0.03071	0.03843	16.9281	16.5026
**TraesCS3A02G039100.1**	**TaMAPKKK56**	0.03613	0.02039	0	0	0	0	0	0	0.25834	0.24962	0.64009	0.67153	0.01535	0.11165
**TraesCS3D02G040600.1**	**TaMAPKKK57**	0	0	0	0	0	0	0	0	0	0	0	0	0.60381	0.64193
**TraesCS3B02G259800.1**	**TaMAPKKK58**	1.05051	1.20716	0.10371	0.11447	0.28619	0.23422	0.69445	0.61309	1.72022	1.3187	0.99455	0.66987	1.46116	1.58187

**TraesCS2A02G216900.1**	**TaMAPKKK59**	0	0	0	0	0	0	0	0	0.41626	0.54925	0.43356	0.39932	0	0
**TraesCS2A02G217000.1**	**TaMAPKKK60**	0	0	0	0	0	0	0	0	0.03433	0.0267	0.33303	0.43394	0	0
**TraesCS7D02G384700.1**	**TaMAPKKK61**	0.70369	0.94332	0	0.01672	0	0	0.71171	0.36209	0.23421	0.31093	0.26805	0.21046	0.03458	0.05086
**TraesCS4A02G313900.1**	**TaMAPKKK62**	0	0	0	0	0	0	0	0	0	0	0.00772	0	1.12459	1.02621
**TraesCS4A02G383000.1**	**TaMAPKKK63**	0.10272	0.03865	0.01796	0.02557	0.03031	0.03377	0	0.15649	0.03204	0.0668	0.08501	0.06698	0.04367	0.08212
**TraesCS4A02G465900.2**	**TaMAPKKK64**	0	0.17745	0.10262	0.15299	0.01461	0.00355	0	0	0	0.01569	0.00534	0.01629	0.02983	0
**TraesCS4A02G464700.1**	**TaMAPKKK64-1**	0.4808	0.21485	0.25821	0.27026	0	0.03413	0	0	0.05177	0.03068	0	0	0.07907	0.12533
**TraesCS4A02G465000.1**	**TaMAPKKK65**	0.03325	0	0.02926	0.01062	0.04891	0.01142	0.0222	0.03022	0.06964	0.09699	0.03233	0	4.1301	4.15439
**TraesCS1D02G273800.2**	**TaMAPKKK66**	1.70989	1.66046	0.06102	0.08237	0.47496	0.48955	1.17339	0.93309	5.90434	6.84849	8.12333	9.16127	1.11013	1.17692
**TraesCS1D02G360600.1**	**TaMAPKKK67**	0.04216	0.02379	0.02227	0.0151	0.0279	0.01086	0.24218	0.1724	0.02642	0.07171	0.13326	0.06414	2.1786	2.21447
**TraesCS1D02G431400.1**	**TaMAPKKK68**	0.11234	0.04227	0	0.01189	0	0	0.37055	0.32907	0.2996	0.23442	1.67548	1.30855	4.45429	4.4509
**TraesCS2D02G588200.1**	**TaMAPKKK69**	0	0	0	0	0.38018	0.11596	0	0	0	0	0.16726	0	0.2878	0.38783

**TraesCS1D02G423800.1**	**TaMAPKKK70**	2.43771	3.10006	1.13459	0.93365	8.39569	7.40727	1.76985	2.00039	1.74975	2.11401	3.31512	2.8613	2.63523	2.50199
**TraesCS6A02G004500.1**	**TaMAPKKK71**	0	0	0	0	3.64E-07	0	0	0	0	0	0	0	0.18794	0
**TraesCS3A02G003900.1**	**TaMAPKKK72**	8.58484	8.04143	0.69868	0.70771	1.70068	1.67427	2.16786	1.71954	1.59502	1.95965	0.90712	0.86184	3.30424	3.86852
**TraesCS3A02G039200.1**	**TaMAPKKK73**	0	0	0.00792	0	0	0	0	0	0.0628	0	0.36551	0.31154	0.21784	0.25994
**TraesCS3A02G039400.1**	**TaMAPKKK74**	0	0	0	0	0	0	0	0	0	0	0	0	0	0
**TraesCS3A02G096500.1**	**TaMAPKKK75**	3.03843	2.47025	0.18762	0.13337	0.09867	0.11315	0.27455	0.42809	0.43788	0.39695	0.70437	0.56117	1.03704	0.82018
**TraesCS3A02G246100.1**	**TaMAPKKK76**	0	0	0	0	0	0	0.05354	0.03392	0.01038	0.0121	0.08204	0.08102	0.05299	0.02476
**TraesCS3D02G501400.1**	**TaMAPKKK78**	0.50952	0.53312	0	0	0	0	0.06501	0	0.03816	0.03581	0.2639	0.31265	0.01948	0
**TraesCS3A02G493900.1**	**TaMAPKKK79**	5.18046	4.01847	0.04363	0.02465	0	0.01063	0.22467	0.1407	0.31726	0.28343	1.29589	1.089	0.23774	0.22901
**TraesCS2A02G217600.1**	**TaMAPKKK80**	0.01412	0.07846	0.01464	0.01322	0.02457	0	0.08886	0.03948	0.0116	0.08701	0.06752	0.15229	0.05919	0.06666
**TraesCS5A02G292500.1**	**TaMAPKKK81**	0	0	0	0	0	0	0	0	0	0	0	0	1.11244	1.11887
**TraesCSU02G072500.1**	**TaMAPKKK82**	0	0	0	0	0	0	0	0	0	0	0	0	0	0

**TraesCS5A02G351500.1**	**TaMAPKKK83**	0	0	0	0	0	0	0	0	0	0	0	0	0.02079	0.02356
**TraesCS5A02G351000.1**	**TaMAPKKK84**	0	0	0	0	0	0	0	0	0	0	0	0	0.25327	0.34378
**TraesCS5A02G352000.1**	**TaMAPKKK85**	0	0	0	0	0	0	0	0	0	0	0	0	0.03782	0.05861
**TraesCS5D02G386800.1**	**TaMAPKKK86**	3.98383	5.1795	0.24804	0.22154	10.8325	6.49831	2.3334	2.14553	3.00199	3.84607	6.37948	6.64424	4.32614	3.87485
**TraesCS1A02G422800.1**	**TaMAPKKK88**	0	0	0	0	0	0	0	0	0.54033	0.43465	1.35613	1.07731	0.93148	0.80886
**TraesCS7A02G152100.1**	**TaMAPKKK90**	0	0.06997	0	0	0	0	0	0	0	0	0	0.03773	0.1058	0.14898
**TraesCS4D02G089300.1**	**TaMAPKKK91**	0.3068	0.203	0	0.02525	0	0	0	0	1.00761	1.24733	15.4918	13.9645	1.44331	1.46103
**TraesCS5B02G337300.1**	**TaMAPKKK92**	9.72086	8.56627	1.3444	1.18644	50.1767	47.4539	17.1589	19.1392	11.9001	14.8359	17.451	18.5569	51.2447	58.5409
**TraesCS5D02G018800.1**	**TaMAPKKK93**	0.08956	0.06318	0.01182	0.01068	0.0494	0.04614	0.02236	0.02035	0.10286	0.03265	0.10885	0.1635	0.02863	0.08585
**TraesCS5B02G012000.1**	**TaMAPKKK94**	20.0577	23.9055	0.40397	0.37257	10.4063	10.5323	2.6272	1.64649	7.30104	6.46417	6.97869	7.37463	11.4601	10.264
**TraesCS5B02G204900.1**	**TaMAPKKK95**	10.9977	12.3229	1.20951	0.97431	0.41462	0.44303	1.99106	1.45318	7.35668	7.26881	13.0382	12.8533	2.95814	1.7996
**TraesCS5B02G292000.2**	**TaMAPKKK96**	0	0	0	0	0	0	0	0	0	0.10207	0	0.1345	0	0

**TraesCS5B02G353800.1**	**TaMAPKKK97**	0	0	0	0	0	0	0	0	0	0	0	0	0	0
**TraesCS5D02G482000.1**	**TaMAPKKK98**	21.5974	19.1707	1.63063	1.3914	5.93599	5.78704	5.84366	4.24742	14.2471	13.9253	21.215	20.3637	15.5599	13.8471
**TraesCS3A02G001500.1**	**TaMAPKKK99**	7.46099	7.3328	0.86174	0.52191	0.60157	0.79659	5.02424	6.78208	2.43452	2.97719	1.7239	1.67914	7.78754	6.53631
**TraesCS3A02G274000.1**	**TaMAPKKK100**	7.45335	6.46407	0.6481	0.6226	3.08862	3.3732	1.30397	1.34302	2.0575	1.76051	1.44866	1.4085	7.73655	8.13349
**TraesCS5D02G019400.1**	**TaMAPKKK101**	16.0054	17.4106	0.67718	0.57773	1.49106	1.4811	3.44181	2.31543	11.5566	10.7258	11.4425	11.5348	9.87933	8.40903
**TraesCS7D02G474700.1**	**TaMAPKKK102**	5.54887	5.50222	1.85514	1.58988	3.01266	2.99567	3.65657	3.89054	3.79904	4.06982	3.53496	3.8881	3.82787	4.67993
**TraesCS7A02G326700.1**	**TaMAPKKK103**	0.97122	0.83205	0.13053	0.10852	0.58354	0.62146	1.39831	1.36023	1.24802	1.58659	2.23294	1.88118	0.81183	0.78298
**TraesCS1D02G273600.1**	**TaMAPKKK104**	0	0	0.0065	0.00345	0	0	0.0077	0	0.08801	0	0	0	0	0
**TraesCS1B02G283400.1**	**TaMAPKKK104-1**	0.1933	0.12688	0.10903	0.04595	0.29282	0.32774	0.02753	0.05074	0.70862	0.86184	11.5287	11.6331	0.21955	0.29368
**TraesCS3A02G045200.1**	**TaMAPKKK105**	0.81043	0.79296	0.3367	0.35974	14.1226	14.2031	0.29201	0.18435	4.40762	4.4279	17.6246	19.2819	0.11486	0.09718
**TraesCS4A02G456900.1**	**TaMAPKKK106**	0	0	0	0	0	0	0	0	0	0	0	0	0	0
**TraesCS7A02G032700.1**	**TaMAPKKK107**	0	0	0	0	0	0	0	0	0	0	0	0	0	0

**TraesCS3D02G023600.1**	**TaMAPKKK108**	0	0	0	0	0	0	0	0	0	0	0.01106	0	0.01939	0
**TraesCS2D02G219800.1**	**TaMAPKKK109**	1.28862	1.05641	0.07258	0.06578	0.23871	0.23832	0.01495	0.0546	0.30047	0.32403	6.03829	6.24553	0.06387	0
**TraesCS4B02G289100.1**	**TaMAPKKK110**	0.07964	0.04229	0.01975	0.01189	0.11028	0.10303	0.03738	0.04541	0.45875	0.42526	1.56349	1.67192	0.03192	0
**TraesCS3A02G315100.1**	**TaMAPKKK111**	0.28545	0.26006	0.01778	0.01608	0.0666	0.01727	0.29394	0.28216	1.29163	1.48331	1.59346	1.46124	0.15769	0.24939
**TraesCS5D02G359500.1**	**TaMAPKKK112**	0.04511	0.10239	0.02392	0.01082	0.01001	0	0	0.03092	0.02841	0.02205	0.02205	0	0.12567	0.11957
**TraesCS3D02G108500.1**	**TaMAPKKK113**	0.20823	0.09401	0	0	0	0	0.02768	0.01262	0.06951	0.05407	0.09439	0.08448	0	0
**TraesCS3A02G229800.1**	**TaMAPKKK115**	0.02193	0.12374	0	0	0	0	0	0	0	0	0.02132	0	0.00935	0
**TraesCS1B02G454000.2**	**TaMAPKKK116**	0.004	0.00486	0	0	0	0	0.00424	0.00189	0	0	0.07432	0.02692	0	0.113
**TraesCS3D02G097000.1**	**TaMAPKKK117**	1.84902	1.196	0.23807	0.28747	0.07356	0.04218	0.22979	0.2063	0.41241	0.66668	1.11766	1.28857	0.32364	0.29404
**TraesCS2A02G577000.1**	**TaMAPKKK119**	0.08415	0.01726	0.14021	0.05294	0.78224	0.65264	0.11317	0.05192	0.34997	0.26647	0.43515	0.44534	6.2847	6.39633
**TraesCS4A02G317600.1**	**TaMAPKKK120**	0.03692	0	0	0	0	0	0.09826	0.10069	6.76168	7.47151	40.5375	38.0032	0	0
**TraesCS2D02G066900.1**	**TaMAPKKK121**	0.82117	0.64697	0.06198	0.07395	0.36445	0.30091	0.3544	0.30261	0.66359	0.6728	1.6118	1.27764	0.38968	0.31146

**TraesCS2A02G214000.1**	**TaMAPKKK122**	0.95653	0.80003	0.02379	0.03579	0	0	0.01503	0.01372	0.11322	0.08817	1.91071	2.00076	0.01283	0.01446
**TraesCS5D02G358700.1**	**TaMAPKKK123**	0	0	0	0	0	0	0	0	0	0	0	0	0.02049	0.04611
**TraesCS5D02G358200.1**	**TaMAPKKK123-1**	0	0	0	0	0	0	0	0	0	0	0	0	0.17993	0.29442
**TraesCS7D02G153800.1**	**TaMAPKKK125**	0.30317	0.38326	0.02351	0.01156	0.03212	0.0125	0	0	0	0.0118	0	0.01476	0.60926	0.84087
**TraesCSU02G011500.1**	**TaMAPKKK126**	0	0	0	0	0	0	0	0	0	0	0	0	0	0
**TraesCS6D02G339600.1**	**TaMAPKKK127**	0	0	0.05528	0.0318	0.05028	0.10761	0.22799	0.27627	0.14296	0.12923	0.39756	0.42794	3.50291	3.75212
**TraesCS1B02G446500.1**	**TaMAPKKK128**	2.99868	3.06142	0.57807	0.57957	21.2924	21.7879	1.02279	1.17629	0.87317	1.00298	0.80955	0.94914	1.84919	1.69197
**TraesCS6B02G320800.1**	**TaMAPKKK129**	0.15172	0.20551	0.01604	0.01933	0.04461	0	0	0	0.70997	0.89439	2.85923	2.70806	0	0
**TraesCS7A02G044600.1**	**TaMAPKKK130**	0	0	0	0	0	0	0	0	0	0	0	0	0	0
**TraesCS6B02G215100.2**	**TaMAPKKK131**	4.39641	3.40953	0.86764	0.99427	1.68233	1.35706	2.62723	2.61796	3.79121	4.88499	2.44438	2.48662	4.43897	5.32502
**TraesCS5B02G353600.1**	**TaMAPKKK132**	0	0	0	0	0	0	0	0	0	0	0	0	1.63638	1.67805
**TraesCS1D02G004300.1**	**TaMAPKKK133**	4.07468	3.67054	0.41576	0.49629	0.48041	0.63785	3.7692	3.74816	2.47779	2.56763	0.92998	0.79792	5.4117	4.66218

**TraesCS5D02G547500.1**	**TaMAPKKK134**	0	0	0	0	0	0	0	0	0.01109	0	0.03919	0.04873	0	0
**TraesCS2D02G598800.1**	**TaMAPKKK135**	0	0	0	0	0	0	0	0	0	0	0	0	0.01509	0.02874
**TraesCS2B02G241600.1**	**TaMAPKKK136**	0.33267	0.32855	0.2481	0.19109	0.83109	0.62946	0.41462	0.8445	0.13458	0.08098	0.02693	0.03374	2.2806	2.21401
**TraesCS3D02G472000.1**	**TaMAPKKK137**	0	0.01311	0	0	0	0	0.01159	0.01055	0.07942	0.18066	2.45868	2.28977	0	0
**TraesCS3A02G493500.1**	**TaMAPKKK139**	5.76298	3.67002	0.06132	0.06671	0	0	1.82375	2.436	1.18914	1.30284	1.49451	1.25761	0.01584	0
**TraesCS5D02G097900.1**	**TaMAPKKK140**	3.01934	2.7234	0.27135	0.28302	9.40875	8.67148	5.03646	5.86696	7.62267	9.19599	7.87631	7.03619	7.39227	7.97755
**TraesCS7D02G000800.1**	**TaMAPKKK141**	0	0	0.07441	0.10315	0.00827	0	0	0	0	0.02733	0	0	3.53755	3.74816
**TraesCS2A02G032200.1**	**TaMAPKKK142**	0	0	0	0	0	0	0	0	0	0	0	0	0	0
**TraesCS3D02G501100.1**	**TaMAPKKK144**	0.25657	0	0	5.20E-05	0	0	0	0	0.19208	0	0.12306	0	0	7.21E-06
**TraesCS2B02G242300.1**	**TaMAPKKK145**	0	0	0	0	0	0	0	0	0.02565	0.02997	0.02986	0.01872	0.01309	0
**TraesCS2B02G241400.1**	**TaMAPKKK146**	0.87989	1.2131	0	0.03045	0.10244	0.07953	0.20425	0.26754	1.0592	0.99967	2.07509	2.05562	0.897	1.07671
**TraesCS2A02G216600.1**	**TaMAPKKK147**	0.29877	0	0	0	2.70797	3.3302	0	0	0	0	0.12623	0	0	0.08719

**TraesCS1A02G003900.1**	**TaMAPKKK148**	0	0	0	0	0	0	0	0	0	0	0	0	0.08433	0.03203
**TraesCS3B02G008600.2**	**TaMAPKKK150**	10.6019	11.5836	1.22839	0.87764	2.68728	2.45564	2.75748	1.88679	2.1812	1.69276	0.97067	1.16586	6.79894	6.58454
**TraesCS3B02G123800.1**	**TaMAPKKK151**	0.43865	0.32209	0.06561	0.06666	0.05055	0	0.66365	0.65135	7.57625	8.04817	6.48776	6.77957	3.43963	3.64179
**TraesCS3B02G259100.1**	**TaMAPKKK152**	0.03475	0.02615	0	0	0	0	0	0.01053	0	0.01126	0	0	0	0
**TraesCS3B02G351800.1**	**TaMAPKKK153**	0.08216	0	0	0	0	0	0	0	0.12797	0.11458	0.26071	0.14122	0	0
**TraesCS3B02G478400.1**	**TaMAPKKK154**	0.03823	0.02868	0.02013	0.00607	0	0.01311	0.02544	0.02315	0.02119	0.11137	0	0.03096	1.03802	0.94909
**TraesCS6B02G217100.1**	**TaMAPKKK155**	0	0	0	0	0.03242	0	0	0.08851	0.42036	0.42493	1.36858	1.26551	0	0
**TraesCS1A02G181900.1**	**TaMAPKKKK1**	10.4092	7.22061	4.85381	4.4649	6.40321	7.74615	4.01949	6.35472	4.37574	4.99867	7.0492	6.12511	8.83577	10.0115
**TraesCS1B02G199100.2**	**TaMAPKKKK2**	0.09334	0.29541	0	0	0.14281	0.68513	0	0.1059	0	0	0.3334	0.10225	0	0.10189
**TraesCS1D02G185000.2**	**TaMAPKKKK3**	0.15467	0.35274	0.07568	0.04603	1.6627	1.41101	0.87096	0.08132	0.0682	0.12317	0.15549	0	0.30223	0.71389
**TraesCS2A02G233400.1**	**TaMAPKKKK4**	8.36674	6.93887	1.7164	1.55141	4.05354	4.78097	3.80637	3.96043	3.86049	4.4492	3.29856	3.11483	6.17328	6.68226
**TraesCS2B02G249900.1**	**TaMAPKKKK5**	8.07995	6.04508	1.28561	1.18312	3.73972	4.0003	3.85599	5.14908	4.30031	5.2378	3.65141	2.85223	7.11359	7.51037

**TraesCS2D02G232200.1**	**TaMAPKKKK6**	6.0911	3.37563	0.99381	0.93922	2.60534	1.78599	2.42687	2.54669	2.50905	2.31407	1.69735	1.42179	4.22524	4.41955
**TraesCS4B02G395600.1**	**TaMAPKKKK7**	0.04978	0.15046	0.00864	0.01562	0.10271	0.05137	0	0.03001	0.04247	0.04874	0.03235	0.02029	0.08555	0.01584
**TraesCS4B02G398400.3**	**TaMAPKKKK8**	2.7169	0.75444	1.23518	1.36935	5.43E-08	3.10E-08	1.38905	1.44588	1.7584	0.69352	1.56266	2.02216	2.20747	1.29356
**TraesCS5A02G187400.1**	**TaMAPKKKK9**	0	0	0	0	0	0	0.05617	0.22291	0.48118	0.61465	0.43085	0.70276	0	0
**TraesCS5A02G392500.1**	**TaMAPKKKK10**	2.8722	2.65297	0.9929	0.98402	0.69559	0.71462	2.0858	2.61465	1.79127	1.79001	1.37183	1.32328	3.34958	3.58375
**TraesCS5A02G556400.5**	**TaMAPKKKK11**	0	0.30568	0.10375	0	1.16944	1.44432	0	0	0.31141	0	0.41309	0	0	0.13892
**TraesCS5B02G397300.1**	**TaMAPKKKK12**	2.23882	1.69136	0.96295	0.86512	0.58147	0.39064	1.71095	1.96674	1.95909	1.75305	1.1706	1.08701	2.76776	2.98708
**TraesCS5D02G203600.1**	**TaMAPKKKK13**	0.31236	0.47698	0.00963	0	0	0	0.34459	0.30103	0.7226	1.16098	0.95533	0.7363	0	0
**TraesCS5D02G402300.1**	**TaMAPKKKK14**	2.55004	2.11723	0.84909	0.85037	0.50155	0.72753	1.91236	2.16896	1.88488	2.21538	1.37462	1.46595	3.14192	3.09779
**TraesCS6A02G149900.1**	**TaMAPKKKK15**	0.01491	0	0	0	0	0	0.20916	0.51871	0.08155	0.06032	0	0	0	0
**TraesCS6A02G353400.1**	**TaMAPKKKK16**	0	0.14356	0	0	0.02945	0	0	0	0	0	0	0	0.04284	0.09375
**TraesCS6A02G353500.1**	**TaMAPKKKK17**	0.17618	0.11985	0	0	0	0	0	0	0	0	0	0	0	0

**TraesCS 6B02G1 77800.1**	**TaMAP KKKK18**	0	0	0	0	0	0	0.12541	0.14695	0.03488	0	0.10696	0.11494	0	0
**TraesCS 6B02G3 86100.1**	**TaMAP KKKK19**	0	0	0	0	0	0	0	0	0	0	0	0	0	0
**TraesCS 6D02G3 35800.1**	**TaMAP KKKK20**	0.29582	0.4219	0.12283	0	0.83166	1.3741	0	0.14811	0.475	0.4473	0.42491	0.52418	0.1394	0.34591
**TraesCS 6D02G1 39200.1**	**TaMAP KKKK21**	0.01718	0.01939	0	0.00813	0	0.01777	0.29641	0.44582	0.20665	0.38949	0.11671	0.10457	0	0
**TraesCS 7A02G2 32300.1**	**TaMAP KKKK22**	11.9071	8.51867	1.65205	1.42042	8.39651	8.79259	4.19249	5.98501	7.48615	8.41833	9.41127	7.85262	8.7987	9.515
**TraesCS 7B02G1 30700.1**	**TaMAP KKKK23**	10.5672	8.08974	1.50889	1.4467	6.92037	7.55393	4.35357	5.9919	7.00833	7.86371	7.63091	7.02302	7.6167	8.23585
**TraesCS 7D02G2 32400.1**	**TaMAP KKKK24**	3.02309	1.93368	0.56678	0.31506	1.91074	1.84477	1.31711	0.83064	1.86956	4.10924	1.85262	2.11178	1.67245	1.45145
**TraesCS U02G11 5300.1**	**TaMAP KKKK25**	0.00032	0.00927	0.45046	0.22645	3.50E-05	0	0.42355	0.51336	2.41803	0.23834	0.72617	0.01878	1.46907	7.69E-06

Table S5 The expression profile data of MAPK-MAP4Ks.

root_Z13_rep1	root_Z13_rep2	root_Z39_rep1	root_Z39_rep2	spike_Z32_rep1	spike_Z32_rep2	spike_Z39_rep1	spike_Z39_rep2	spike_Z65_rep1	spike_Z65_rep2	stem_Z30_rep1	stem_Z30_rep2	stem_Z32_rep1	stem_Z32_rep2	stem_Z65_rep1	stem_Z65_rep2
14.0576	22.9723	22.1792	27.2761	37.1546	36.2331	42.7221	52.3714	48.9568	53.9285	30.7933	47.1066	32.0245	36.9101	39.7736	46.2367
10.1703	5.00269	3.95589	7.69367	6.46213	4.82619	11.8936	5.32985	10.7492	5.51821	1.51538	3.89698	8.18444	8.32315	5.46189	6.05371
32.5263	41.0413	44.6531	33.5171	8.85154	8.15926	11.6509	7.41388	3.20477	1.79714	17.4968	12.0993	54.6245	49.6663	5.19284	6.18819
0	0	0	0	0	0	0	0.12116	0	0	0	0.13367	0	0	0.14968	0
0.36499	2.13147	2.62574	0.49341	0.30519	0.69435	0.28664	0.98645	11.0262	11.7988	1.59177	0.87819	0.42364	0.64464	0	0
13.549	15.4127	13.2908	17.9273	22.8265	21.3338	25.7197	28.3975	15.0877	17.1789	28.8898	28.9146	18.2811	15.6186	14.3469	14.2561
2.30134	3.48672	3.36777	3.68395	6.98779	6.58382	5.50714	5.8255	5.95509	7.47205	4.86032	3.77625	6.8592	6.09792	4.60356	4.69741
7.31342	8.94774	8.25053	8.19043	9.93684	9.17727	18.836	16.2593	12.2242	12.0835	11.1487	10.7141	9.36791	8.14553	25.9531	26.5437
13.8199	9.29107	8.94326	20.1928	16.0299	10.7788	14.2919	16.5587	21.7805	25.0142	12.0621	9.93373	20.7812	16.6688	19.5257	23.9406
10.7639	13.3439	11.0751	15.901	15.7035	17.5045	24.3351	26.8325	13.2993	14.4258	19.9516	21.467	12.9838	12.3061	9.7203	10.381

24.0902	25.3378	23.9822	33.6276	19.0815	16.5207	18.4821	15.1146	19.5765	13.7705	18.6693	16.7534	40.7658	32.4535	7.45564	8.08425
12.5053	25.9503	21.626	30.0871	36.7938	37.2883	39.833	58.4175	69.0164	64.8055	35.0836	44.5384	31.1204	27.03	27.6593	38.0913
6.32855	6.6507	5.56969	9.57094	4.86748	4.21284	3.5248	4.84081	3.11702	3.01407	9.02475	9.94738	9.51446	8.40546	5.3066	5.69076
8.58182	18.1894	15.8747	11.6758	7.54856	7.94033	17.7033	21.9284	8.88704	10.8637	10.168	11.5424	5.31873	4.22497	7.29184	6.8308
0.79154	0.65082	0.74348	0.99345	1.37682	1.62356	1.12628	1.17349	0.67242	0.91249	1.28816	1.4493	0.97965	0.64055	0.47027	0.50734
52.9615	62.6989	60.8521	70.652	28.4164	22.6431	39.7819	38.0917	50.338	46.6747	24.5709	20.681	67.2601	59.846	53.8692	59.0489
14.2095	18.7772	15.2006	21.1137	21.0291	21.0541	23.0943	26.1332	16.1501	17.8644	28.8697	28.6705	17.5802	16.9757	16.1029	13.6409
5.57257	8.67627	5.61391	8.7792	2.13153	2.84777	2.10053	0.5288	2.63845	1.79479	3.56709	1.52024	11.8881	11.5776	1.63299	0.85939
0.08499	0.32372	0.4002	0.06363	0.33665	0.32471	0.54751	0.32585	21.9951	15.4058	0.2368	0.14587	0.18004	0.14365	0	0
0.88323	1.23589	1.15421	1.49841	2.28682	2.40537	2.7148	2.83971	5.66631	5.84598	1.27452	1.62092	1.87995	1.25784	1.79268	1.4753
8.30378	16.2075	12.1836	11.3179	7.4409	8.85821	14.8682	19.3539	7.69033	9.4422	14.6577	15.0993	7.24922	5.61451	7.36457	8.82504
33.7577	50.655	58.0777	36.453	4.64479	3.23831	3.86203	2.04963	3.11042	1.79633	13.0732	10.3215	76.7707	65.322	6.55273	6.14035

0.62472	1.28935	0.88232	0.90522	1.60758	0.64577	1.46513	1.77423	1.29635	1.0705	1.18448	0.29424	0.29884	0	0.86128	0.18669
16.3427	16.2049	13.6885	26.6282	14.8044	15.7607	20.8572	26.011	12.4557	13.2281	18.2147	19.2127	13.0774	11.366	9.03355	10.7712
15.7717	14.2223	13.5521	19.3605	12.4882	10.9612	9.09481	12.4313	6.00338	6.23063	22.1211	22.4987	20.675	20.1499	15.2287	15.6558
0	0	0	6.57E-10	0	0	0	0	1.31618	0	0	0	0.00025	0	0	0
0	0.6983	2.09E-08	0.003	0.37663	1.85623	1.17159	1.86785	0.80242	2.19985	1.64478	0.25706	0	0.24944	0.77836	0.06954
43.9209	51.8266	50.9193	51.6315	23.0208	22.6468	36.0987	32.4788	41.4214	39.4187	20.7803	18.2666	66.8496	60.9824	36.2715	39.6468
12.4311	22.862	21.6745	13.2093	55.1925	40.2436	17.1197	13.0938	4.93167	5.3688	11.427	8.53401	15.56	14.7932	3.92097	4.88575
8.43114	13.3243	13.3383	11.0112	5.69005	5.78832	14.5519	16.0229	6.26097	7.59664	6.50875	6.76517	4.73595	4.80104	13.3684	13.7364
1.03061	0.63088	0.54725	0.86061	1.10138	1.1398	0.88493	0.88434	0.81958	1.007	0.91735	0.79191	0.63164	0.66575	0.58678	0.61476
0.20511	0	0.10961	0	0.74438	1.37115	0.25011	0.44685	0.23327	0.2706	0.14058	0.6704	0.82133	0.3603	0.15497	0.32164
18.6271	30.7584	26.0151	27.6464	40.6612	44.9507	47.3841	46.3589	57.6588	51.1392	39.2523	40.9586	41.7219	43.2595	41.6816	38.9761
3.94253	6.16013	5.42609	6.61387	7.24097	7.1274	13.0353	12.2284	12.0754	10.9814	8.7584	9.75816	5.27966	4.19019	24.2173	23.6627

12.0377	16.7408	14.4721	18.697	10.2147	9.15399	18.4385	20.7458	10.8537	13.1278	17.2249	17.5212	10.5892	9.6068	6.36206	8.61252
0.47234	0.447	0.62279	0.43399	1.13109	1.45044	1.26493	1.12331	3.85323	4.48897	1.01241	1.04558	0.60668	0.57733	0.47505	0.39269
0	0.32518	0.5722	0.25817	0.83629	0.58499	0	0	0	0	0.25643	0.28932	0.47702	0.70699	0.33973	0
1.3427	3.04467	2.80768	0.45381	0.10526	0.23244	0.09454	0.34023	7.94791	7.84695	1.94921	0.77217	0.06982	0.17257	0.47282	0.22939
7.14051	15.2545	14.4761	8.33568	11.7254	11.2528	19.4263	18.599	8.47178	9.91083	7.00629	6.89793	9.34688	8.42388	12.0682	12.1826
0.2989	0.03409	0.09474	0.07997	0.16298	0.27117	0.22557	0.13325	0.18222	0.29854	0.16227	0	0.11832	0.03965	0.42697	0.63457
10.286	9.29099	7.47628	14.5168	14.8509	12.0121	14.2621	14.8244	15.3387	17.183	10.919	10.0554	13.2486	12.5764	13.1732	15.4645
12.7175	28.9865	24.5922	15.5731	5.35308	6.97664	12.8783	14.3081	14.4492	17.5289	3.50094	7.13594	15.8736	16.3121	8.51669	15.6452
0	0	0.03199	0.03482	0.08567	0.09414	0.51186	1.22328	2.26986	1.94162	0	0.0355	0.12888	0.03494	0	0.03287
8.04926	20.2037	27.9511	9.88855	19.5781	28.3442	21.1992	12.0302	6.10164	7.22337	9.38093	6.59862	23.8175	19.41	0.60106	0.66572
8.6319	8.68428	7.27279	10.3083	12.3209	11.5027	19.9157	18.7944	13.5337	13.8607	8.41928	8.78858	12.921	10.9464	8.46573	8.76201
2.12319	3.67889	3.24023	3.83571	4.98892	5.65274	4.84077	4.99677	7.13488	7.23675	3.52553	3.81175	6.91636	6.12625	4.70974	5.30154

20.4004	77.0491	71.9115	32.9191	88.6762	89.1804	63.3789	41.0361	32.5935	29.9804	32.5293	31.5968	141.457	121.727	2.52643	2.6277
18.0362	17.3841	15.7286	22.9914	10.9087	9.20789	13.1832	11.2079	17.0485	14.5834	10.6369	11.7654	18.0484	16.0681	2.38002	3.05635
8.48958	9.65638	14.3385	4.14694	4.36056	7.08405	3.26295	2.18852	4.31266	3.32475	5.4736	3.43794	9.42157	7.81568	4.63062	3.96596
0.18402	0.17936	0.29406	0	0.13181	0.81868	0.21507	0.8019	0.38049	0.09286	0	0.40484	0.11817	0.12271	0.14567	0
0.22986	0.30022	0.37552	0.46224	0.00636	0.3023	0.27799	0.72617	0.41759	0.48041	0.74285	1.27869	2.69E-08	0.54733	0.09651	0.58743
0	0.12905	0	0	0.27888	0	0.38966	0	0.39174	0	0	0	0	0.43215	0	0
0	0	0	0	0	0	0	0	0	0	0	0	0	0.03772	0	0
0	0	0	0	0	0	0	0	0	0	0	0	0	0	0	0
0	0.07316	0.03476	0	0.12367	0.13585	0.03694	0	0.01707	0	0.15169	0.11546	0.02788	0.03786	0	0
0	0.07397	0.07031	0	0	0	0.07467	0.03439	0.20701	0	0	0	0.01409	0	0	0
0	0	0	0	0	0	0	0	0	0	0	0	0	0	0	0
0	0	0.07031	0	0	0	0	0	0.0345	0.03134	0	0	0.01409	0	0	0

0	0	0	0	0	0	0	0	0	0	0	0	0	0	0	0
2.95681	2.34366	2.57277	4.74523	5.54771	6.09242	5.04609	7.48869	3.44445	4.29391	5.38401	6.29798	3.65125	3.58856	2.15878	2.70607
0	0	0	0	0	0	0	0	0.03413	0	0	0	0	0	0	0
0.0139	0	0.02951	0.029	0	0	0	0	0	0	0	0	0	0	0	0
0	0.06838	0.07485	0	0	0	0	0.02112	0.06409	0	0	0	0	0	0	0
0	0	0	0	0.03092	0	0	0.03402	0.01707	0	0	0	0	0	0	0
0.62858	0.43547	0.35553	0.66982	0.77783	1.03607	0.47734	1.03137	0.45488	0.69847	1.05403	0.96418	1.18991	0.45617	0.59094	0.41426
3.13187	3.36214	3.87593	4.00049	4.6001	6.22037	5.07724	6.43118	2.83339	3.51532	3.92868	3.99396	3.4476	2.95965	1.82681	2.41107
2.36026	2.3643	3.01112	1.97529	0.24298	0.21425	0.2264	0.06878	0.03501	0	0.98473	0.56757	0.21127	0.51254	0.0125	0
3.76406	4.41433	3.59965	3.98238	5.35388	5.40034	5.82423	7.436	5.87516	6.19959	4.28913	4.83012	4.89309	3.9698	3.43011	5.05833
0.97113	3.82586	3.03757	2.87109	5.33624	5.35962	5.98941	6.71458	2.30262	4.17244	3.81552	4.41101	3.48646	3.30705	3.03123	3.34222
1.53981	2.74265	2.05859	1.90496	4.02233	4.89647	5.3936	6.02601	2.90485	4.38319	3.16997	3.62127	3.7334	3.07846	2.26364	2.69655

25.761	72.0143	65.1546	34.4606	32.2067	42.0509	41.3399	32.5693	10.6267	9.5426	14.1444	17.7826	58.7771	55.6511	7.29324	7.40727
6.9106	20.3338	25.8455	4.59055	3.78532	3.73516	1.45802	1.32418	2.65331	1.20821	1.81794	1.57403	14.5884	17.0357	0.38078	0.59245
16.6304	27.8412	30.2591	9.24037	8.65584	17.3857	3.12582	1.52889	0.96187	0.63712	7.574	5.03184	20.7984	25.4847	0.04249	0.10478
16.6156	17.994	20.2664	7.99184	3.11151	7.66023	1.04327	0.54184	5.40941	3.03261	3.65296	2.99487	11.4647	17.6432	0.07447	0
1.25721	3.8241	3.48682	2.82243	3.85624	4.42628	4.60408	6.07459	3.17409	4.81855	2.34012	4.86171	1.45114	3.1523	2.21307	1.71798
12.8614	11.1338	9.1205	16.4105	4.94359	4.31963	6.52379	6.81954	13.3804	13.2177	3.75147	3.50718	10.4185	10.5798	4.44343	5.01251
4.41032	4.31942	4.22995	5.56601	6.38255	8.16576	6.88242	8.21442	6.66642	6.27513	4.69181	5.83209	2.33192	4.86906	4.9984	4.83535
0	0	0	0	0.01358	0	0.04377	0	51.8711	33.8269	0	0.01673	0	0	0	0.02993
9.57149	5.17012	5.57619	8.87404	3.05572	3.24475	5.51867	5.77902	6.48723	6.44677	3.02682	2.54086	1.98584	2.65123	2.25395	2.13337
11.8212	14.7163	13.0151	15.1069	8.50628	7.29474	12.3128	10.6701	13.9085	12.8759	6.60179	7.31553	33.7789	34.2633	9.18251	9.81397
2.05893	6.04571	5.24301	2.73077	7.56601	9.57444	2.90609	3.42954	1.52162	1.84917	9.14049	9.25645	2.02847	1.94698	3.45E-10	2.26E-10
0.72608	1.35051	0.6556	1.61926	0.6846	2.18286	0.78161	2.26024	0.65986	0	0.6048	1.4572	0.68081	1.04693	0.25185	0.93369

3.19644	3.60828	3.51254	3.49187	6.47548	6.38529	4.97641	9.01766	3.28399	4.4263	4.94017	6.7395	7.4551	6.58843	1.60229	2.2087
0	0	0	0	0.02066	0	0.45413	0	5.38208	4.09372	0	0.03368	0.01241	0	0.0846	0
2.34812	3.25082	3.65747	3.39388	4.62704	4.71786	4.41641	4.64122	3.39698	3.50035	5.38835	5.46683	4.14041	3.8707	1.58496	1.3341
0	0	0	0	0.03149	0.05201	0.21636	0	16.4279	17.4734	0	0.05827	0	0	0.15535	0.07817
1.80804	4.33642	4.1145	2.5301	5.68374	6.79071	2.38222	2.70672	1.0411	1.55474	7.53864	7.31929	1.65432	1.54419	2.00E-10	3.91E-10
0	0	0	0	0.0147	0.04858	0.14078	0.04799	0.21112	0.14487	0.07278	0.03626	0	0.07167	0.37707	0.25945
1.06987	3.09208	2.7638	1.2675	7.96871	10.7681	1.93102	2.68224	1.88161	2.54639	6.06971	6.93211	1.47946	1.56898	0.01016	0.07794
1.56216	3.00204	2.81571	1.73091	7.27284	10.2819	2.62211	4.16203	3.22177	3.70183	4.87263	7.0273	2.02045	2.02238	0.08834	0.09239
1.17104	2.38395	2.37317	1.54958	7.08032	10.1343	2.58807	3.6489	2.23401	2.36768	4.86669	5.70871	1.5818	1.8852	0.10595	0.04384
2.12404	5.96357	5.5652	3.51627	7.35895	8.49577	2.51522	3.24118	1.61691	1.70846	8.61926	7.66265	1.46376	2.05698	0	0
11.8056	8.69837	9.26185	10.5192	0.34277	0.26141	2.14975	1.60215	11.0379	6.67219	4.44007	3.15279	8.71236	8.36083	5.19022	4.68512
1.03718	1.11735	0.73065	0.891	2.75147	3.60935	3.35631	4.27141	10.6418	11.5808	1.06335	1.47566	2.04448	1.3295	1.43713	1.41368

9.03842	14.8326	13.8957	13.159	10.9909	10.2201	12.3911	13.0263	15.2704	17.7009	20.0636	16.6677	13.9805	13.3569	7.10789	8.5237
0.24159	0.22456	0.18067	0.40925	2.48485	2.52269	4.24894	5.57335	12.7671	11.8763	1.14946	1.94774	1.96628	1.90583	2.9788	2.99537
4.97501	5.69666	7.3981	3.49592	0.38807	0.3063	2.27294	1.61735	0.80036	0.88728	0.82798	0.4983	1.84934	1.5998	0.03301	0.07909
6.78963	8.13253	7.98239	7.05718	8.10653	8.2134	13.2711	14.0081	15.5569	15.75	10.5968	10.2471	9.27536	9.25077	6.52064	7.88371

0	0.16794	0	0.6928	0.40705	0.47861	0.46351	1.32828	0.76243	0.43752	0.54592	0.85594	0.37431	0	0.64304	0.3987
0.47461	0.47542	0.30956	0.79085	1.98631	2.03267	2.42468	3.40147	11.2949	11.762	0.9672	1.18741	1.14506	1.38016	1.16793	1.41617
9.05479	9.18674	11.3385	7.59386	0.54933	0.28804	1.88273	1.54494	4.20586	3.30333	3.84919	2.86478	5.53577	5.09708	4.78854	4.8696
0	0	0.11985	0	0	0	0.16827	0.13409	0.03785	0.068	0	0	0	0	0.10257	0
0	0	0	0.02136	0	0	0	0	0.046	0.00421	0	0.0101	0.059	0.05884	0.04341	0
0.06163	0.09006	0.02123	0.16845	0.0374	0	0.04467	0.0827	0	0	0	0	0	0.04589	0	0
0	0	0	0	0	0	0	0	0	0	0	0	0	0	0	0

7.91766	14.0224	15.3904	8.16332	0.43495	0.38024	0.92257	0.86464	2.58255	1.92961	1.23968	1.3642	4.68914	4.9063	0.67189	0.476
0.03071	0	0	0.12704	0	0	0	0	0	0	0	0	0	0	0	0
0.71715	1.2575	0.56361	1.41899	1.13441	1.57131	1.31789	1.98272	0.70436	1.08011	0.94925	1.18553	0.39121	0.51021	0.17874	0.43457
0.13601	0.21836	0.21248	0.37047	0.30378	0.72256	0.83501	0.80191	0.88226	0.8794	0.32538	0.50952	0.74285	0.96636	0.92699	0.91808
0.03139	0	0	0.10622	0.01426	0	0	0.06203	0.01575	0.04516	0	0	0	0.05211	1.55149	1.23384
1.67623	1.12481	1.03319	1.45034	0.77502	0.42809	11.3457	6.6058	1.76744	1.65876	2.32334	1.95818	1.1592	1.23335	2.13843	1.86732
0	0.02221	0.01025	0.02054	0	0	0	0	0	0.01847	0	0	0	0	0.0625	0
10.2473	6.93552	8.0153	8.67956	0	0	0.02362	0.03209	0.18856	0.18802	0.20559	0.21827	0.79803	0.5521	0.09963	0.20583
16.0012	22.6029	20.4156	24.5132	0.0193	0.0319	0.01155	0.05231	0.07461	0.03782	0.68967	0.5219	0.93869	0.88058	0.20577	0.18014
0.48684	0.02254	0.08201	0.73382	0	0	0	0	0.03656	0	0.01637	0.04086	0	0.02018	0	0
1.33619	0.51473	0.87032	1.70489	0.01327	0	0	0	0	0	0	0	0	0	0	0
1.25129	1.38547	1.33189	1.60417	3.76071	3.27008	2.45966	2.60373	2.30937	2.3164	1.37442	1.46777	2.16335	1.41499	1.54776	1.52239

0	0	0	0	0	0	0	0	0	0	0	0	0	0	0	0
0	0	0.00688	0	0.01257	0	0	0	0	0	0.01246	0	0	0	0	0
0.12375	0.04781	0.02947	0.06222	0.04373	0	0.07207	0.12235	0.12247	0.09585	0	0.07134	0.1172	0.09995	0.32325	0.39174
1.02602	0.76823	0.50192	1.52861	0	0	0.00872	0	0	0.00711	0	0	0	0.01769	0	0
0	0.03692	0.05175	0.01709	0.19803	0.07036	0.05925	0.18133	0.03745	0.22257	0.09313	0.03995	0.04707	0.01912	0.12485	0.19662
0.02429	0.02309	0.05224	0	0.0159	0	0	0.34721	0	0	0.00865	0.01309	0.02069	0.02898	0	0.04826
0.93027	0.37132	0.31992	0.72751	0.47028	0.28241	0.20474	0.53501	0.09591	0.19987	0.31075	0.21299	0.27332	0.21254	0.06797	0.06373
5.74583	3.85485	3.75607	5.57881	0.0508	0.0896	0.73031	0.62826	0.01186	0.05978	0.33303	0.42078	0.26829	0.18603	0.06844	0.08932
0.25458	1.47186	1.58073	0.06803	1.21645	0.64115	0.67997	0.4751	0.41561	0.2528	0.79691	0.76903	1.28715	1.34028	0	0.06383
4.84775	1.48164	2.19142	3.74052	0	0.01065	0.08101	0.08382	0.06695	0.02841	0.09593	0.11879	0.0615	0.07055	0	0
3.21657	2.8193	2.6501	3.71301	0	0.02522	0.0274	0.02484	0.10112	0.06741	0.4484	0.38771	0.21967	0.25569	0.99661	0.77803
1.30446	1.41753	0.81515	2.31438	0	0.08601	0.16387	0.11613	0	0	0	0	0.13672	0.24374	0	0

4.88622	1.44872	1.13084	4.71214	5.50254	6.23137	4.57106	4.02568	3.18629	3.02506	4.01208	4.00994	2.29825	3.25184	2.39008	1.94562
0	0	0	0	0	0	0.09329	0	0	0	0	0	0	0	0	0
2.17445	2.81668	2.85545	2.2114	6.21849	6.34386	7.56596	6.94989	5.04385	4.68066	6.08903	5.48135	5.58577	6.04635	1.71295	1.85551
0.05795	0	0.00764	0.1369	0	0	0	0	0	0	0.05467	0	0.06263	0.01681	0	0
0.31629	0	0.00751	0.55126	0	0	0	0	0	0	0.01357	0	0	0.03337	0	0
2.80631	0.72372	0.75593	3.54555	0.16499	0.08304	0.28676	0.6551	0.9609	0.6298	0.34863	0.33179	0.69621	0.43663	0.92314	0.81478
0.18643	0.11359	0.20559	0.10543	0	0	0.0273	0.01238	0.02519	0.01119	0.01131	0	0	0	0	0.02667
0.20101	0	0	0.11519	0.13955	0.1174	0.13305	0.27513	0.09649	0.10858	0.08144	0.03864	0.13906	0.19366	0.06284	0.02368
0.31851	0.12224	0.15472	0.53382	0.76873	0.56184	0.52034	0.95786	0.75065	0.73247	0.40444	0.46809	0.98942	0.79901	0.48091	0.38602
0.22734	0.17974	0.10763	0.16623	0.04174	0.07381	0.03518	0.02932	0.02878	0.09408	0.10531	0.01569	0.11572	0.0776	0.07868	0.11353
0.17883	1.38239	0.99448	0.56503	0.02841	0	0	0	0	0	0	0	0	0	0	0
0	0	0.00601	0	0	0	0	0	0	0	0	0	0	0	0	0

0	0	0.01275	0.12292	0	0	0	0	0	0	0	0	0	0	0	0
0.82308	1.10079	1.1853	0.97159	0	0	0	0	0	0.01069	0	0	0	0	0	0
0.06728	0.19103	0.12933	0.30904	0	0	0	0	0	0	0	0	0	0	0	0
7.58568	5.84894	4.66133	5.77646	3.32921	2.70994	4.7006	4.59474	7.53622	7.47281	3.00965	4.0138	7.89486	6.32173	7.47946	7.09181
3.40551	1.45087	1.86159	3.72457	0.0122	0	0	0	0	0.03598	0.33568	0.18033	0	0.41617	0	0.1209
0.22391	0.1006	0.03177	0.06788	0	0.03117	0.04025	0.03116	0	0.07368	0	0	0	0	0.03182	0.03165
0.7122	1.04784	0.81279	0.99961	0.48492	0.07484	0.10454	0	0.15001	0	0	0.07155	0	0.31868	0	0
12.5028	25.1366	22.0935	16.8522	9.60023	10.5102	12.778	14.8484	11.4318	13.5474	23.7327	27.6907	30.5449	29.0638	11.3815	12.1295
0.50064	0.19286	0.22395	0.83534	0.12314	0.09049	0.11061	0.10019	0.0907	0.17109	0.02037	0.10097	0.72767	0.49963	0.43774	0.65404
16.9325	7.35593	10.2391	9.83255	6.42432	2.58925	3.70593	2.10971	7.60795	6.39664	3.61231	2.57331	21.1678	23.2017	8.32024	7.59311
3.29816	2.43597	3.26452	2.74995	6.71605	6.51215	5.69941	2.13627	2.59074	1.82511	1.77641	1.39302	10.4826	10.8463	1.52255	1.7413
0	0	0	0.08086	0.20506	0.21458	0	0	0	0	0	0	0.08969	0	0	0

0	0.06163	0	0.05776	0	0	0	0	0	0	0	0	0	0	0	0
18.7227	15.2743	14.6759	19.2419	10.0584	8.18144	9.58147	6.94799	14.4299	14.9723	8.01666	8.3439	15.7612	14.8446	35.6986	36.5816
3.61558	6.56378	6.6421	4.26529	14.1513	12.0196	15.3303	14.1487	15.8381	17.7587	8.04795	7.11268	12.7142	11.0381	6.68	7.03446
6.68574	10.7407	10.4297	7.59839	1.80238	2.81999	1.96805	2.77302	3.63585	2.93502	2.85898	2.94327	4.73605	5.07851	2.06896	1.94424
15.5419	7.23823	10.4136	8.77315	5.74793	3.10997	3.21565	2.19556	5.93945	4.6605	3.74638	3.54179	23.5989	26.957	10.8635	10.3153
4.97426	5.87961	5.38825	5.9198	6.26783	5.35226	6.8996	7.2618	6.04208	7.14322	5.89972	6.21835	5.58736	5.69671	4.55575	4.98482
1.3057	1.28828	1.26906	2.25706	0.92138	1.07501	0.91614	1.09573	1.87661	1.98234	1.3288	1.13461	1.57501	1.2741	2.02143	1.92003
0.02669	0	0.03981	0.01102	0	0	0	0	0	0.01092	0	0	0	0	0	0
0.58761	0.28396	0.3185	0.16507	0.06327	0.04182	0.0303	0	0.16774	0.08431	0.10132	0.14026	0.44028	0.57589	0.01565	0.02963
0.81222	0.41868	0.15305	0.5729	0.57525	0.34072	0.56751	0.33975	1.60109	1.0707	0.26229	0.40076	1.2516	1.32545	0.41669	0.5197
0.01535	0	0.01605	0	0.04386	0.03104	0	0	0	0	0	0	0	0	0	0
0	0	0	0	0.05445	0.14086	0	0	0	0	0	0	0	0	0	0

0.27888	0.26938	0.30141	0.28287	0	0	0	0	0	0	0	0	0	0	0	0
0.74357	0.1876	0.20289	0.46386	0.05342	0.0606	0.03293	0.02991	3.50673	2.45614	0	0.01695	0.16232	0.21255	1.85909	1.74308
0.22821	0.04035	0.04379	0.16163	0	0.01261	0.01371	0.01243	0.1138	0.04496	0.07947	0.01409	0.72806	0.64109	0.15418	0.10072
0.08477	0.0271	0.09206	0.13345	0.51576	0.56873	0.32277	0.48353	0.41677	0.36895	0.43613	0.32143	0.48336	0.40682	0.14692	0.16883
0.15498	0.43955	0.45515	0.28118	0.19749	0.361	0.07469	0.11277	0.04575	0.13254	0.17537	0.13947	0.06614	0.06326	0.04643	0.02285
0.02671	0	0.0209	0.01383	0	0.01402	0.01523	0	0.01405	0	0	0.03134	0.03466	0.0155	0	0
0	0	0.00548	0	0	0	0	0	9.21155	4.9695	0	0	0	0	0	0.01104
0.778	0.25463	0	0.71035	0.00181	0	0	0	0.00197	0	0.00515	0.00223	0.02472	0.03289	0	0.009
2.30198	0.59858	0.32051	3.62036	0	0.02752	0.18529	0.24422	1.65138	1.08695	0.24759	0.40834	0.39671	0.52604	0.86907	0.96352
9.97288	14.2876	12.8571	13.8382	0.08333	0.22699	0.45463	0.25396	0.3024	0.25926	0.55498	0.58754	0.83361	0.46716	0.2415	0.24833
0	0	0	0	0	0	0	0	1.34595	0.79743	0	0	0.0205	0	1.96273	1.79897
0.46795	0.19639	0.26123	0.70187	1.08031	1.04973	1.09698	1.3385	1.68841	1.61185	0.94836	0.5764	0.83422	0.73066	0.87834	1.16503

0.07979	0.08126	0.01516	0.03007	0	0	0	0	0.19923	0.21991	0	0	0.01255	0	0.09307	0.03116
0.32379	0.18128	0.1264	0.40689	0	0	0	0	0	0	0	0	0	0	0	0
0.78324	1.19701	0.86228	1.27567	0	0	0	0	0	0	0	0	0	0	0	0
0.32596	0.68388	0.5829	0.31479	0.09084	0.147	0.05676	0.14479	0.34384	0.41901	0	0.02736	0.06061	0	0.07486	0.02445
0	0	0	0	0	0	0	0	0	0	0	0	0	0	0	0
1.90074	3.86617	4.06935	2.69209	0.05227	0.05763	0.11479	0.05665	0.0385	0.09384	0.15578	0.1927	0	0.0848	0.20539	0.10518
1.24906	1.08684	1.01021	0.95735	6.39113	8.08816	6.67698	4.40283	2.82264	3.63198	5.19185	4.97566	2.48523	2.65081	0.99586	0.98782
0	0	0	0	0.05564	0.08178	0.02221	0.07037	0.12966	0.19987	0.01842	0	0	0.05642	0	0
0	0	0	0.05296	0	0	0.02889	0	0	0	0	0	0	0	0	0
2.32854	3.7797	3.32796	3.66364	3.6371	4.12425	3.76079	4.89069	3.29742	3.9329	2.85394	2.9861	2.36787	2.22716	1.98965	2.27346
2.57705	4.76426	3.98977	2.00841	0	0	0	0	0	0	0	0	0	0	0	0
4.84481	4.40796	5.37058	4.4885	7.83923	10.0207	7.53088	8.56643	4.13738	4.69097	10.26	9.25812	4.34758	5.01292	3.60182	3.441

0	0	0	0	0	0	0	0	0	0	0	0	0	0	0	0
0.1946	0.09689	0.15058	0.22409	0	0	0	0	0	0	0.0136	0.01692	0	0	0	0
1.24303	0.89605	0.90031	2.38904	0.30488	0.33591	0.19772	0.05876	0.07017	0.19717	1.05492	1.32992	0.41898	0.49514	0.04278	0.05595
0	0	0	0.01155	0.02129	0.01173	0.01275	0.01155	0.02352	0.01044	0	0.01309	0	0	0.01195	0
0.03571	0	0	0	3.81353	2.44263	3.05141	4.52711	1.4205	1.32887	3.16433	2.45881	2.22464	2.80234	2.32288	1.9088
6.15919	9.79482	8.48547	8.23523	6.28734	7.56384	6.29469	7.68309	5.98011	6.23682	8.69235	9.16368	4.97315	5.09722	4.71732	4.93982
5.55581	6.71288	6.10325	8.10113	0.40415	0.19909	1.38022	1.92885	0.13301	0.21047	0.10251	0.10566	0.03129	0.06277	0	0
0	0.03854	0.03668	0.03588	0	0	0	0	0	0	0	0	0	0	0	0
4.59E-06	7.86E-06	2.20E-06	9.68E-06	0.09079	0.09249	0	0.1329	0	0	0.1708	0.27379	0.30777	0	0	0
0.04438	0	0	0.03067	0	0	0	0	0.01557	0	0.02791	0.01737	0.02558	0	0	0
3.90884	1.03327	0.70488	4.31554	0.11727	0.18092	0.1197	0.15988	0.32388	0.2419	0.27912	0.17323	0.82986	1.13072	0.27643	0.27905
0	0	0.07813	0.69858	0	0.38046	0	0	0.4849	0	0.12987	0	0	0	0	0

0	0.28844	0.18834	0.20127	0	0	0.03641	0	0	0	0	0	0.02749	0	0	0
3.35022	3.78545	4.10714	2.71196	6.81017	5.9982	8.8569	6.51771	5.2531	5.1292	6.06509	6.04223	14.0718	13.8262	2.98058	2.71753
1.83581	4.69551	3.46111	2.20116	1.36069	1.90506	0.81029	0.71213	3.78346	3.53421	2.06896	1.90214	2.89426	2.58033	0.16179	0.01544
0	0	0	0	0	0	0.01271	0.01152	6.60024	4.42111	0	0	0	0	0	0
0	0	0	0	0.10607	0.03882	0	0.03996	0.11708	0.14249	0	0	0	0	0	0
0.3601	0.91691	0.7436	0.93572	0.10475	0.14116	0.12557	0.22764	0.07747	0.06871	0.1156	0.20144	0.1691	0.21288	0.13125	0.11549
0	0	0.01963	0	0	0.1202	0.05286	0.39911	0.10989	0.03335	0.13009	0	0.08968	0	0.21932	0.20671
8.10155	10.8258	8.61844	15.0877	10.9288	11.4235	10.6347	14.19	9.71206	10.2822	13.9978	13.9439	11.7349	11.367	9.28293	9.76561
0.41422	0	0	0.25203	0.00015	0	0	0.37608	0.42538	0.0734	0.27266	0.59311	0	0.02963	0	0.08045
0.52654	0.53158	0.07405	0.42708	0.20145	0.24315	0.37829	0.52383	0.16755	0.38308	0.43102	0.55113	0.05194	0.34895	0.14687	0.13328
5.03013	5.71944	5.75152	6.42023	9.85414	11.2781	9.32168	10.937	7.51924	8.44769	8.6742	9.58565	8.05154	6.52744	4.78707	5.44439
4.5729	6.98754	6.25231	7.51812	9.2083	10.1418	9.53409	10.9157	6.9518	8.12441	9.92944	11.3859	7.5789	6.23448	5.67332	5.44836

3.64031	4.38747	3.68578	3.33974	5.40513	7.91945	6.27305	7.38399	5.7239	5.54927	5.3013	5.95327	3.79644	3.35627	3.40056	2.45649
0.03996	0.0715	0.00839	0.06611	0.06125	0.16987	0.05591	0.05029	0.08435	0.06007	0.03077	0.03758	0.0693	0	0.01699	0
5.78257	5.62327	3.478	6.22121	1.11551	2.81303	3.49411	6.31962	3.70734	3.83339	2.9602	4.82024	2.72E-09	0.77016	0	0.44602
0.02357	0.05283	0.00822	0.0163	0	0.01648	0.08957	0.02116	0.05239	0.13857	0	0	0	0	0.1808	0.41277
2.34812	3.25082	3.65747	3.39388	4.62704	4.71786	4.41641	4.64122	3.39698	3.50035	5.38835	5.46683	4.14041	3.8707	1.58496	1.3341
0	0.43422	0.3884	0.61243	0.1741	0.13491	0.22894	0	0.72327	0.35184	0	0.25768	0	0.14268	0	0.11322
2.17013	2.94378	2.47192	2.79715	3.93436	3.88434	4.72834	4.11772	3.09686	3.00879	4.93158	4.46393	3.79363	3.05064	1.65173	1.49235
0	0	0	0.01835	0	0	0.04029	0.04954	0.25714	0.15968	0.01665	0.04156	0.03053	0.08211	0.32844	0.35336
2.71663	3.39306	3.03929	3.78862	3.58047	5.16556	4.85308	4.88457	2.79473	3.00974	4.78346	4.62643	3.09671	3.48447	1.59686	1.5382
0	0	0	0	0.02066	0	0.45413	0	5.38208	4.09372	0	0.03368	0.01241	0	0.0846	0
0	0	0.03565	0	0.06144	0.19756	0.1255	0.07907	1.45694	1.97827	0.00436	0	0	0	0	0
0	0	0	0.01558	0	0.02333	0.07679	0.0267	1.34242	1.49212	0.02755	0	0	0	0	0

0	0	0	0	0	0	0.21008	0.31102	24.3923	28.7429	0	0	0.01309	0	0	0
0	0	0	0	0	0	0	0	2.3091	1.79455	0	0	0	0	0	0
0.50377	0.26322	0.22636	0.71697	1.18795	0.99844	0.7372	1.21373	1.59483	2.76197	0.655	0.2883	0.71766	0.26824	0.3237	0.27817
0	0	0	0	0.03149	0.05201	0.21636	0	16.4279	17.4734	0	0.05827	0	0	0.15535	0.07817
6.98154	8.46923	6.92043	10.5431	11.5312	11.3102	12.5518	13.5712	14.5213	14.7811	10.8609	12.223	9.70404	8.22371	7.45009	7.85811
6.46121	7.95549	6.66761	10.2028	10.3853	11.795	11.5382	13.4275	12.6455	13.0036	9.92934	10.235	8.00562	7.6103	5.59586	6.37301
1.40439	2.73531	2.07338	1.52232	2.7471	5.05326	4.09187	4.8219	3.91224	3.93711	2.36022	3.54483	1.79428	2.21543	1.17735	1.32474
1.31201	2.54379	1.99771	2.24793	0.35581	1.67E-09	0.63387	1.68097	0.00135	0.00408	1.34637	2.54599	2.36896	1.10E-10	2.129	1.64067

Table S6 The primers of MAPK-MAP4Ks and refrerence gene for the qRT-PCR analysis

Primer name	F-Primer sequence (5'-3')	R-Primer sequence (5'-3')
**TaMAPK3**	CGCTCCAACCAAGAACTCTC	GACCACGTACTCCGTCATCA
**TaMAPK6**	GCTTTATCGGAGGAGCACTG	CTGGTGCCCTGTACCATCTT
**TaMAPK14**	CGTTTGACAACCACATCGAC	GCGAATGATCTGGTGGAGAT
**TaMAP2K1**	CTCAACGACGGCAACTACAA	CGACTCGGAGTAGCAGATGG
**TaMAP2K11**	TACATGAGCCCCGAGAGATT	GAGCTCACCGAAGCAGATG
**TaMAP3K1**	CGCTTCTACTCCCCTGTCTG	GACCAAACTGCCCAAGGTTA
**TaMAP3K7**	CCTTTCGGTGACGATACCAT	GAGACGCACTGTGTTCTCCA
**TaMAP3K8**	CGAAACAGAGGACATGCTGA	CGGATTCCAATCCTTCTTCA
**TaRaf76**	TTCGGTGTCATGGTTCTTGA	TGAGAAGCTGGTGTTCATGC
**TaMAP4K3**	GAAAATCCGCAAGTGTTGGT	TGTTGCACGAGGTTCTCAAG
**TaMAP4K10**	GGCATCAATGAGCTCTCTCC	CCCAAAGTAGCGGACAACAT
**TaMAP4K24**	CACGTAAGAGGGGATTTCCA	GCGCGAAGAAGATTAGGATG
**TaRP15**	GCACACGTGCTTTGCAGATAAG	GCCCTCAAGCTCAACCATAACT
