# Supplementary figures and images for: Bioinformatic Identification and Expression Analyses of the MAPK–MAP4K Gene Family Reveal a Putative Functional MAP4K10-MAP3K7/8-MAP2K1/11-MAPK3/6 Cascade in Wheat (Triticum aestivum L.)
Source: Plants (Basel). 2024 Mar 24;13(7):941. doi: 10.3390/plants13070941 (PMC11013086; doi:10.3390/plants13070941)

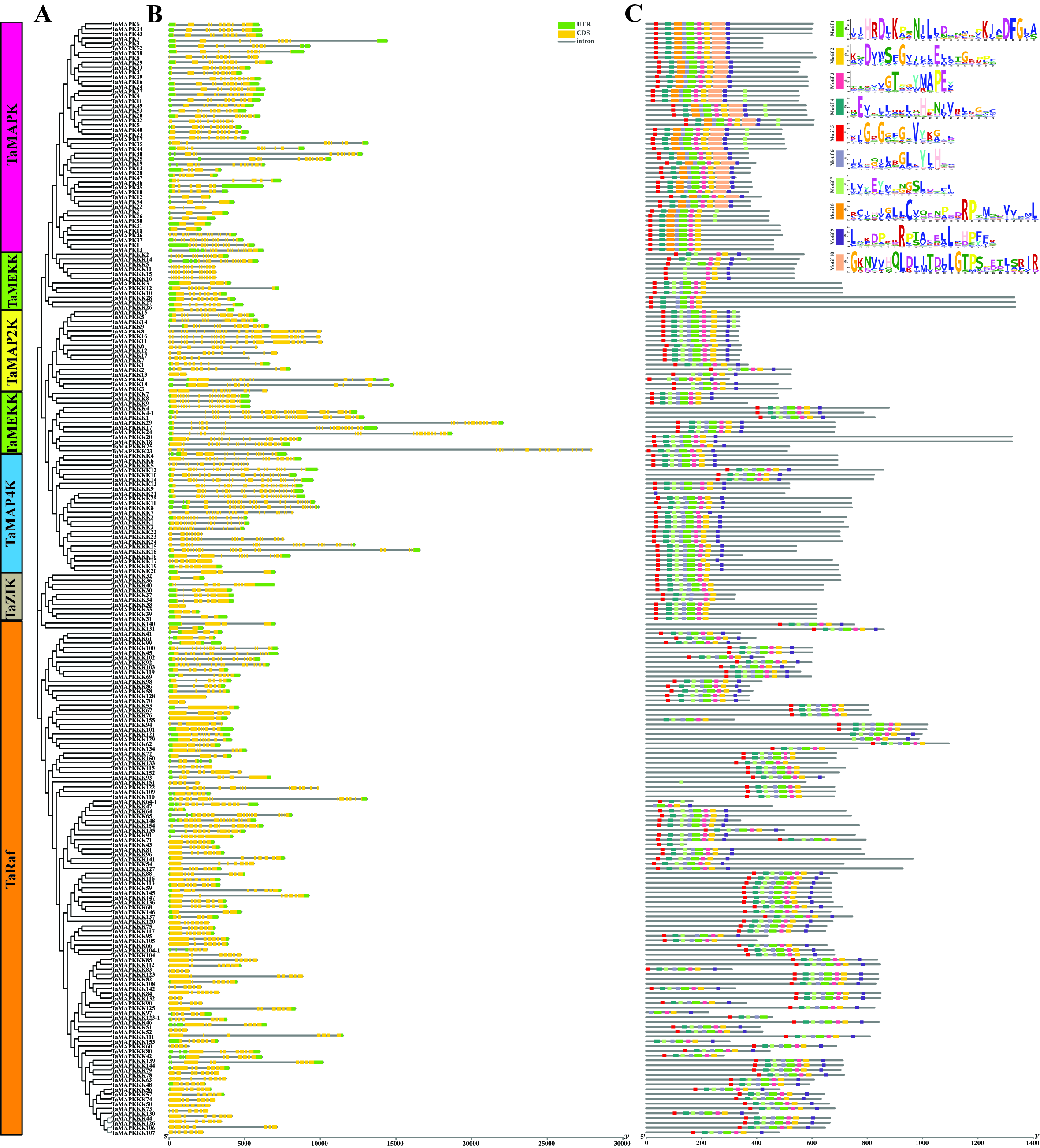

Supplement: Supplementary file 1 [file plants-13-00941-s001.zip › plants-2867660-supplementary/Supplementary Figure S1-S10 and Supplementary table S1-S7/Supplementary Figure S1-S10/Supplemental Figure S1.tif]

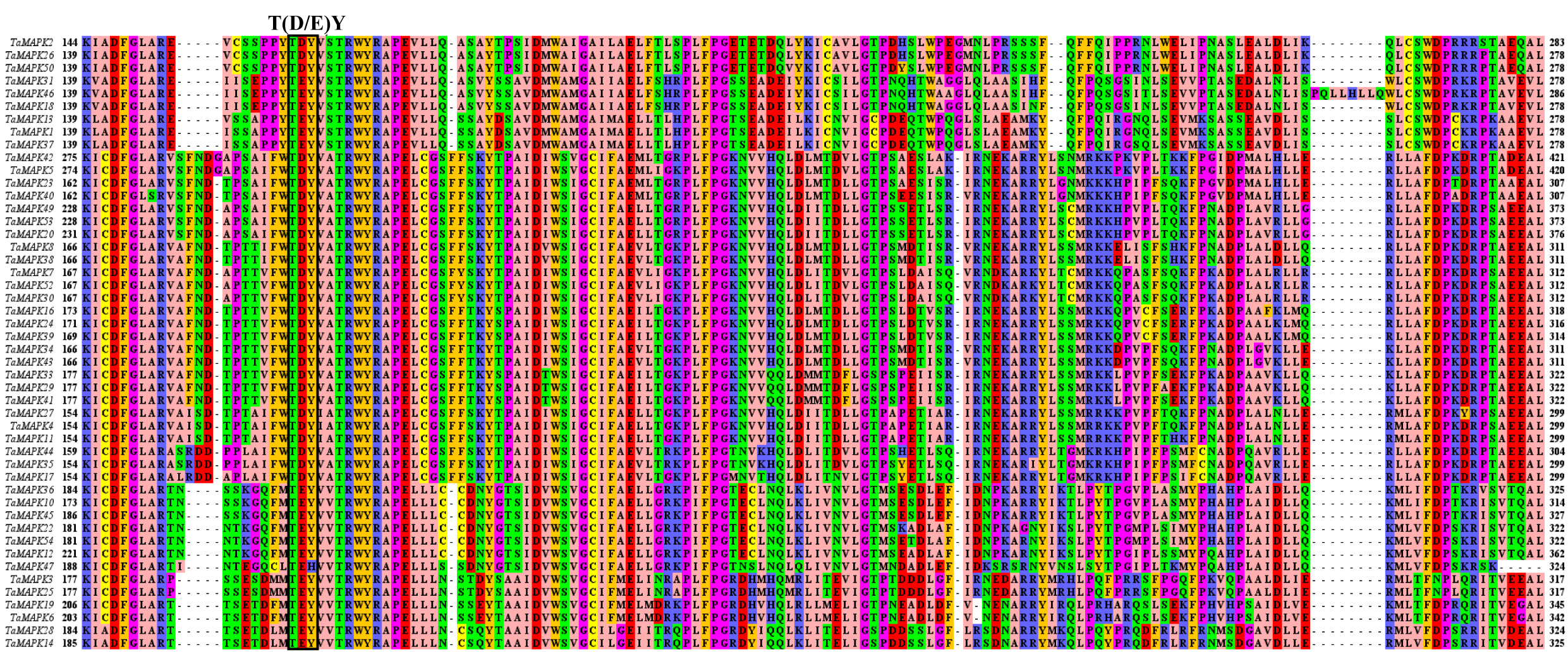

Supplement: Supplementary file 1 [file plants-13-00941-s001.zip › plants-2867660-supplementary/Supplementary Figure S1-S10 and Supplementary table S1-S7/Supplementary Figure S1-S10/Supplemental Figure S2.tif]

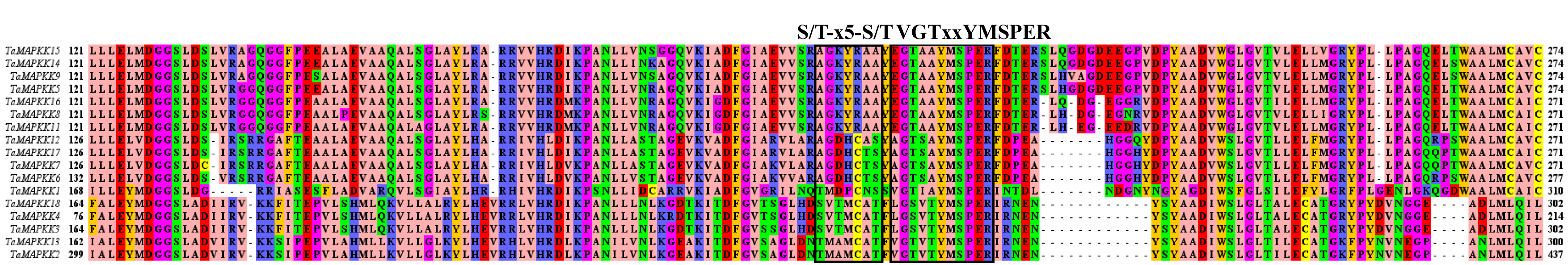

Supplement: Supplementary file 1 [file plants-13-00941-s001.zip › plants-2867660-supplementary/Supplementary Figure S1-S10 and Supplementary table S1-S7/Supplementary Figure S1-S10/Supplemental Figure S3.tif]

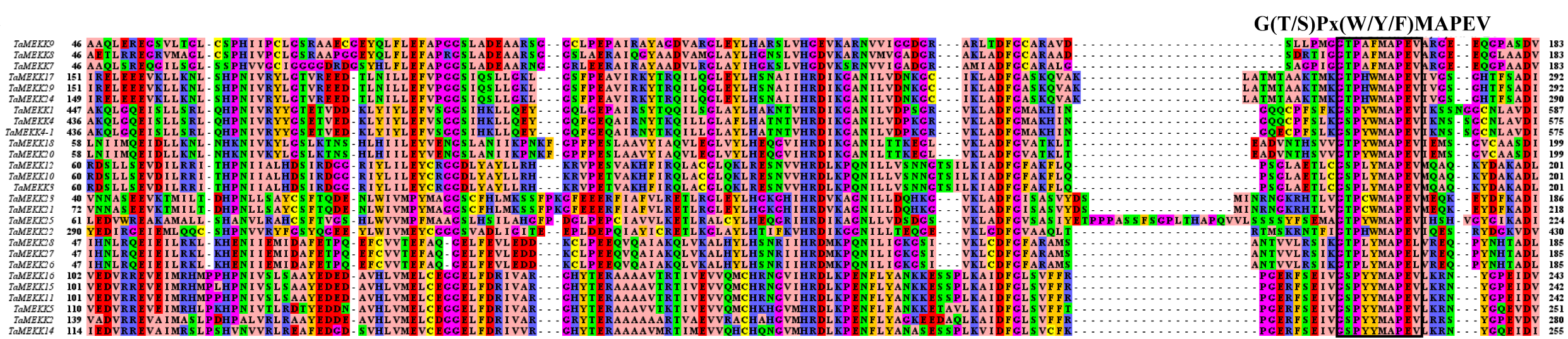

Supplement: Supplementary file 1 [file plants-13-00941-s001.zip › plants-2867660-supplementary/Supplementary Figure S1-S10 and Supplementary table S1-S7/Supplementary Figure S1-S10/Supplemental Figure S4.tif]

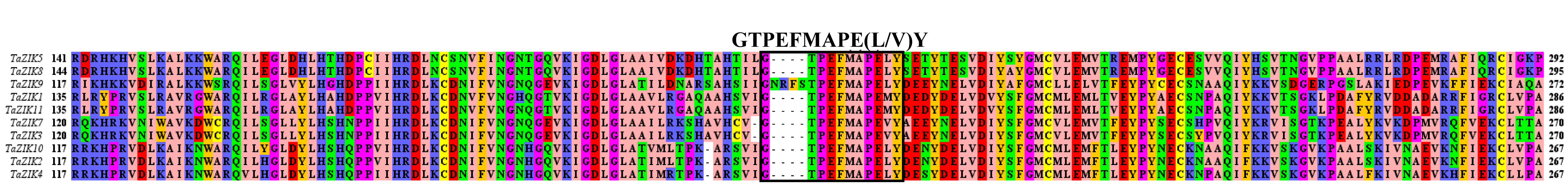

Supplement: Supplementary file 1 [file plants-13-00941-s001.zip › plants-2867660-supplementary/Supplementary Figure S1-S10 and Supplementary table S1-S7/Supplementary Figure S1-S10/Supplemental Figure S5.tif]

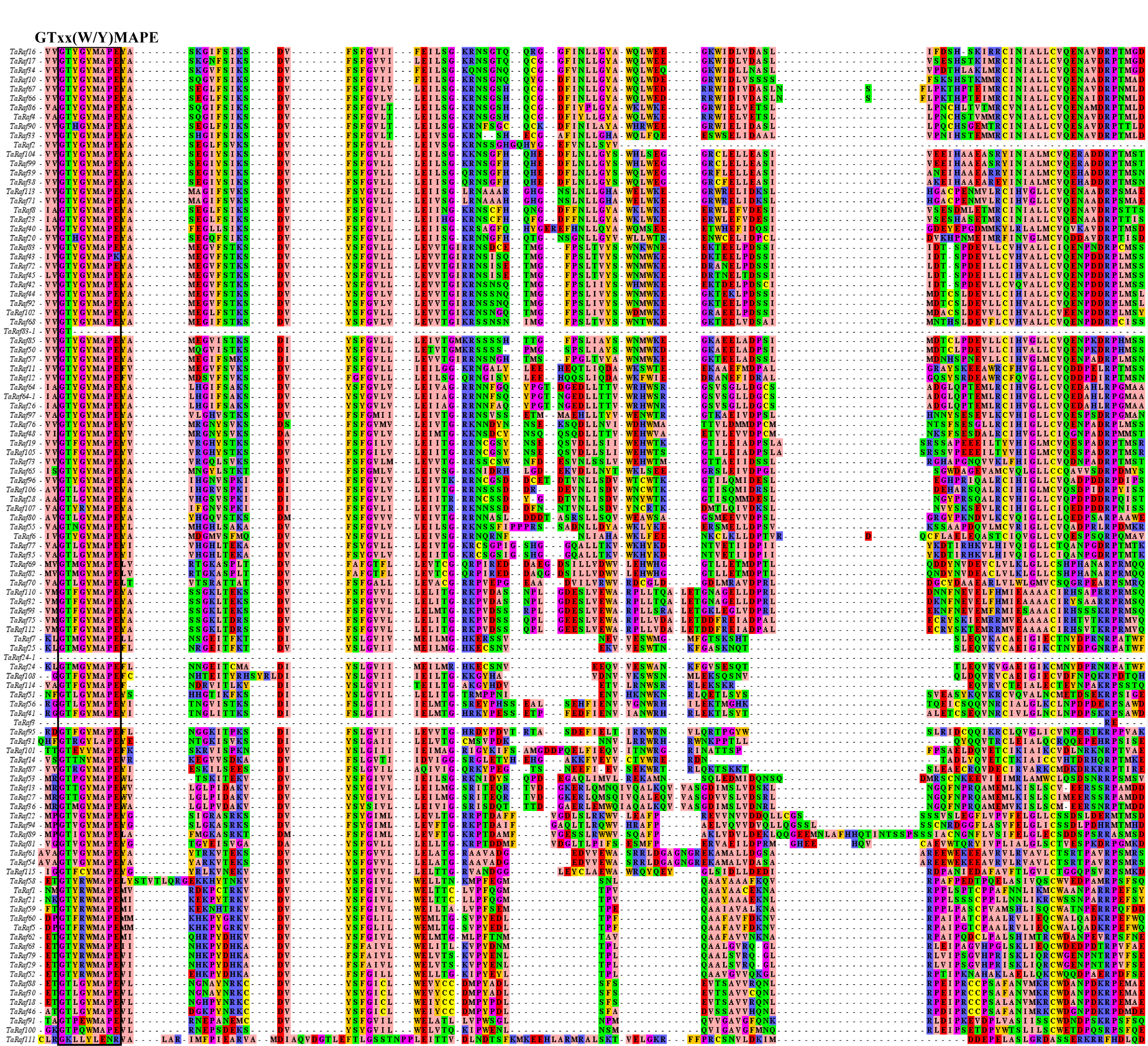

Supplement: Supplementary file 1 [file plants-13-00941-s001.zip › plants-2867660-supplementary/Supplementary Figure S1-S10 and Supplementary table S1-S7/Supplementary Figure S1-S10/Supplemental Figure S6.tif]

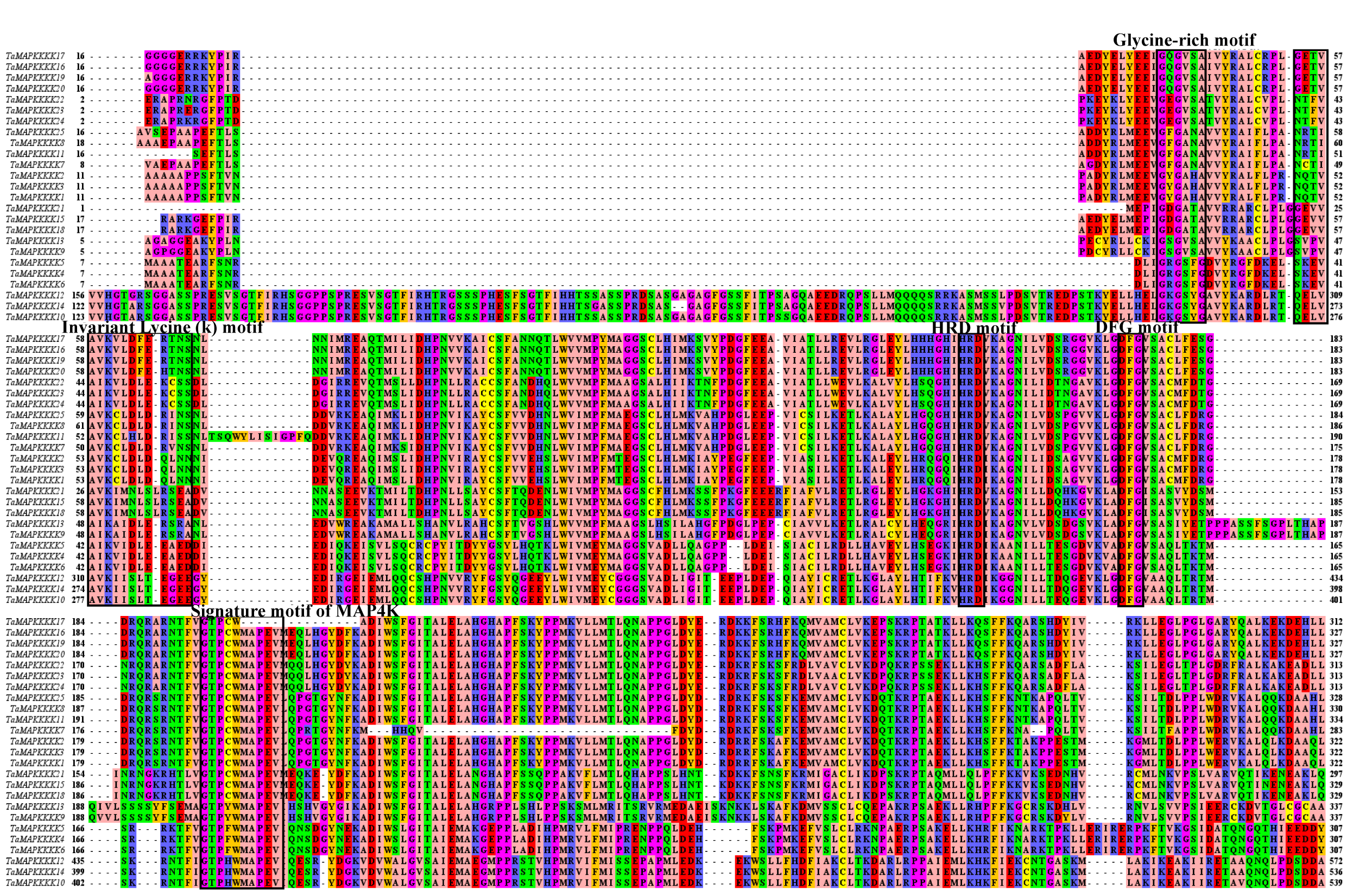

Supplement: Supplementary file 1 [file plants-13-00941-s001.zip › plants-2867660-supplementary/Supplementary Figure S1-S10 and Supplementary table S1-S7/Supplementary Figure S1-S10/Supplemental Figure S7.tif]

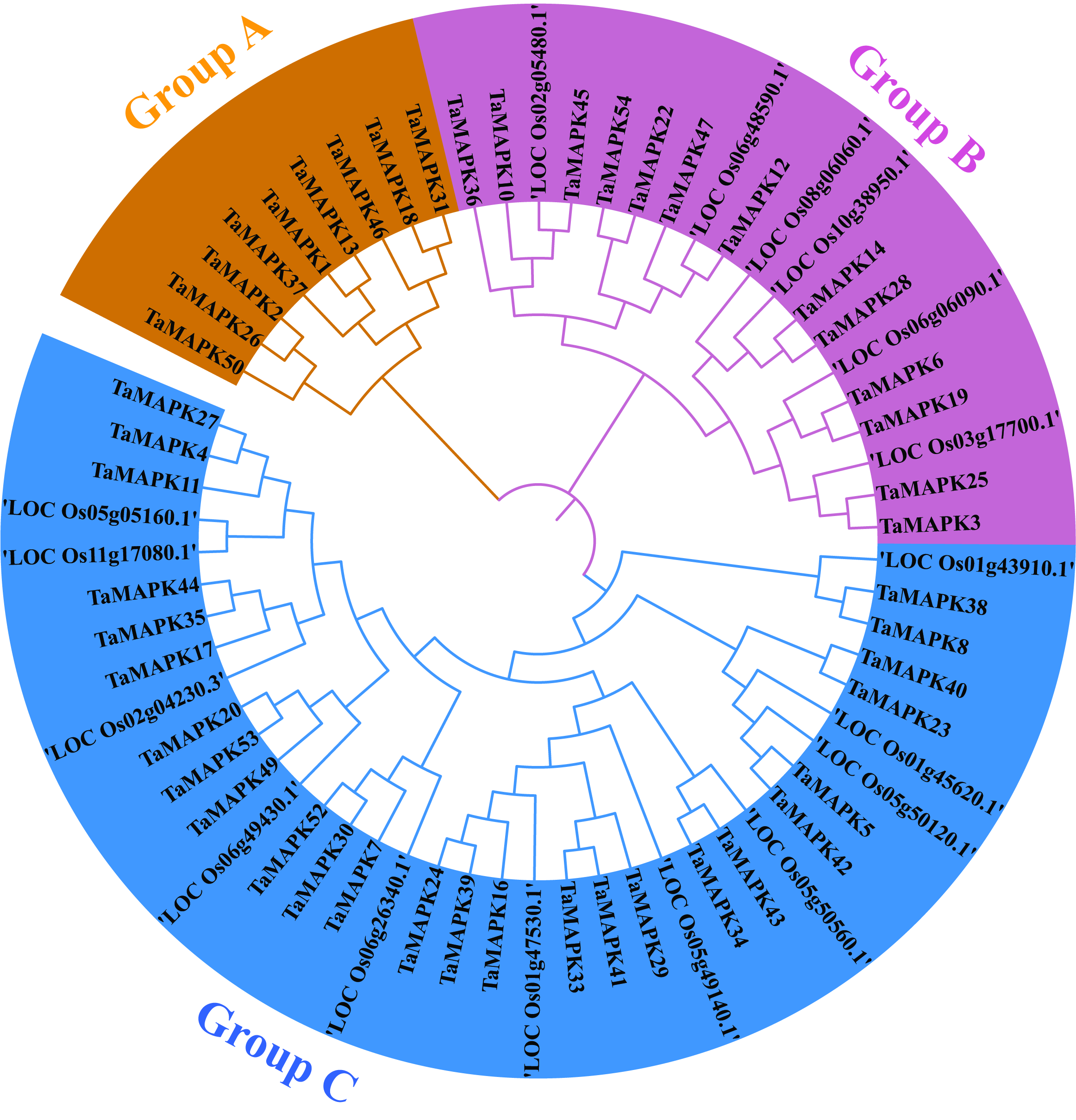

Supplement: Supplementary file 1 [file plants-13-00941-s001.zip › plants-2867660-supplementary/Supplementary Figure S1-S10 and Supplementary table S1-S7/Supplementary Figure S1-S10/Supplemental Figure S8.tif]

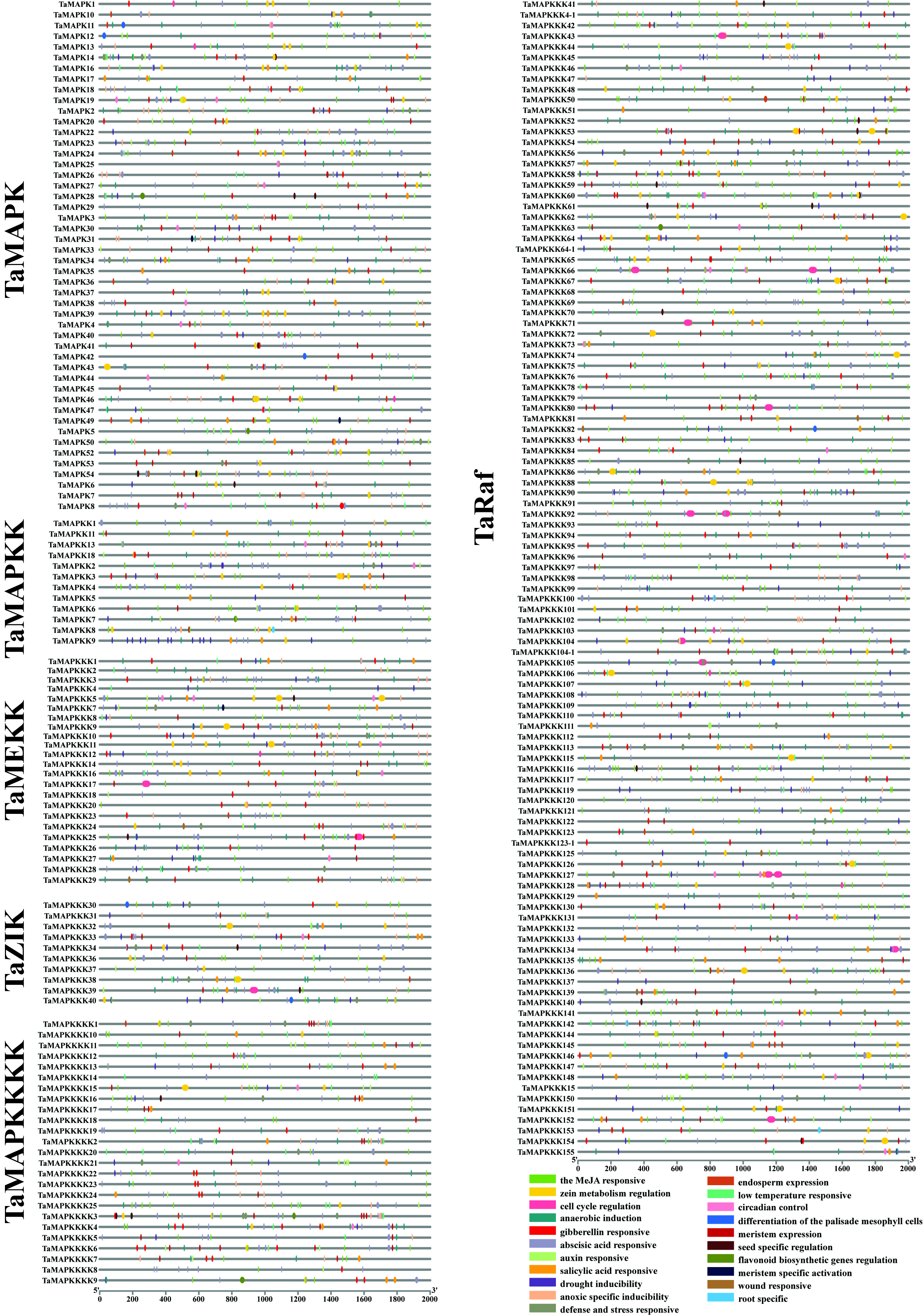

Supplement: Supplementary file 1 [file plants-13-00941-s001.zip › plants-2867660-supplementary/Supplementary Figure S1-S10 and Supplementary table S1-S7/Supplementary Figure S1-S10/Supplemental Figure S9.tif]

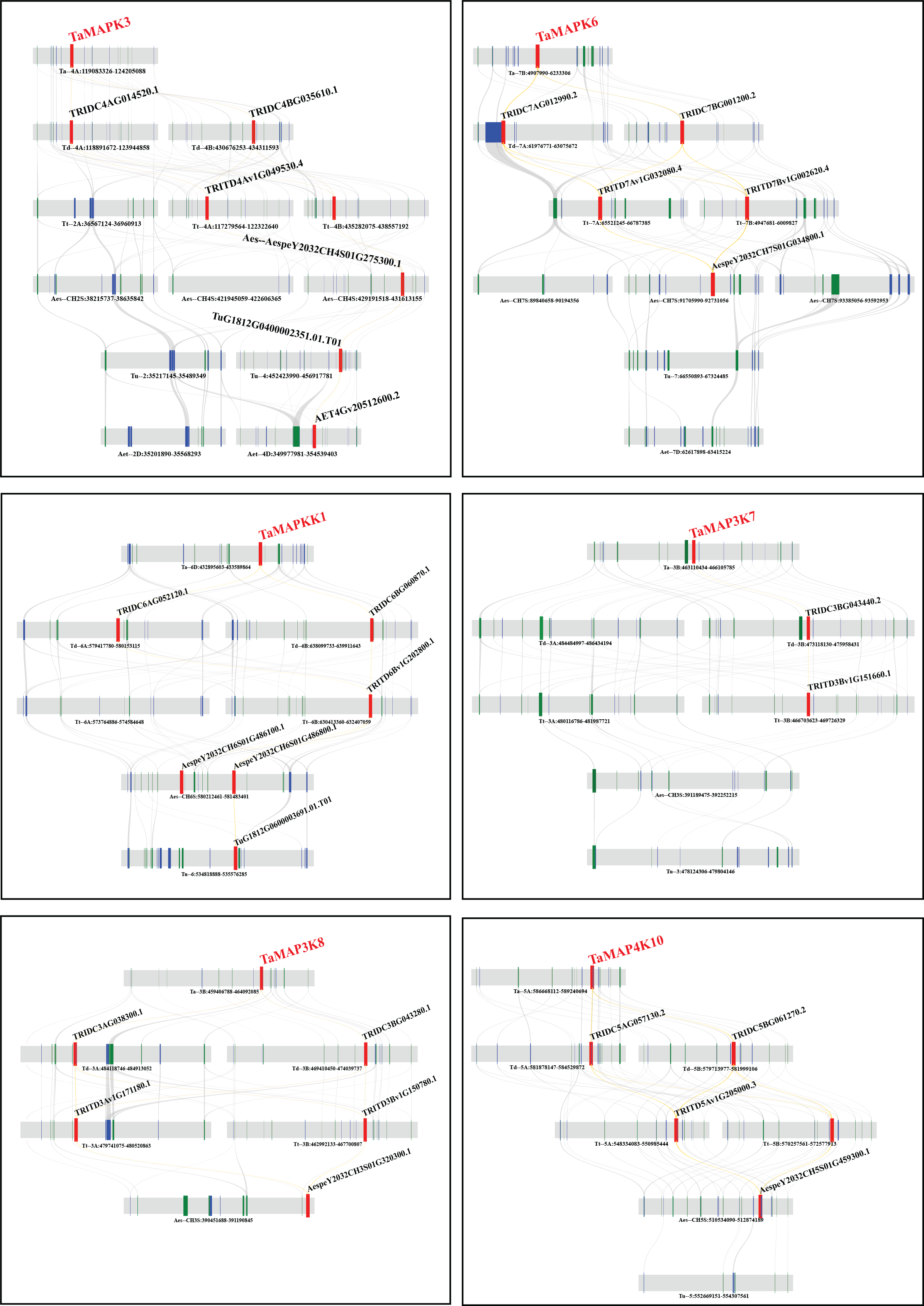

Supplement: Supplementary file 1 [file plants-13-00941-s001.zip › plants-2867660-supplementary/Supplementary Figure S1-S10 and Supplementary table S1-S7/Supplementary Figure S1-S10/Supplementary Figure S10.png]
